# Moving pictures of the human microbiome

**DOI:** 10.1186/gb-2011-12-5-r50

**Published:** 2011-05-30

**Authors:** J Gregory Caporaso, Christian L Lauber, Elizabeth K Costello, Donna Berg-Lyons, Antonio Gonzalez, Jesse Stombaugh, Dan Knights, Pawel Gajer, Jacques Ravel, Noah Fierer, Jeffrey I Gordon, Rob Knight

**Affiliations:** 1 Department of Chemistry and Biochemistry, University of Colorado, 215 UCB, Boulder, CO 80309, USA; 2 Cooperative Institute for Research in Environmental Sciences, University of Colorado, 216 UCB, Boulder, CO 80309, USA; 3 Department of Microbiology and Immunology, Stanford University School of Medicine, 299 Campus Drive, Stanford, CA 94305, USA; 4 Department of Computer Science, University of Colorado, 430 UCB, Boulder, CO 80309, USA; 5 Institute for Genome Sciences, University of Maryland School of Medicine, 801 W. Baltimore Street, Baltimore, MD 21201, USA; 6 Department of Ecology and Evolutionary Biology, University of Colorado, UCB 334, Boulder, CO 80309, USA; 7 Center for Genome Sciences and Systems Biology, Washington University School of Medicine, 4444 Forest Park Blvd, St Louis, MO 63108, USA; 8 Howard Hughes Medical Institute, University of Colorado, 215 UCB, Boulder, CO 80309, USA

## Abstract

**Background:**

Understanding the normal temporal variation in the human microbiome is critical to developing treatments for putative microbiome-related afflictions such as obesity, Crohn's disease, inflammatory bowel disease and malnutrition. Sequencing and computational technologies, however, have been a limiting factor in performing dense time series analysis of the human microbiome. Here, we present the largest human microbiota time series analysis to date, covering two individuals at four body sites over 396 timepoints.

**Results:**

We find that despite stable differences between body sites and individuals, there is pronounced variability in an individual's microbiota across months, weeks and even days. Additionally, only a small fraction of the total taxa found within a single body site appear to be present across all time points, suggesting that no core temporal microbiome exists at high abundance (although some microbes may be present but drop below the detection threshold). Many more taxa appear to be persistent but non-permanent community members.

**Conclusions:**

DNA sequencing and computational advances described here provide the ability to go beyond infrequent snapshots of our human-associated microbial ecology to high-resolution assessments of temporal variations over protracted periods, within and between body habitats and individuals. This capacity will allow us to define normal variation and pathologic states, and assess responses to therapeutic interventions.

## Background

As more attention is paid to viewing ourselves as a supraorganism, comprising interacting microbial and human cellular and genetic components, it is apparent that much more precise understanding is needed of what constitutes normal temporal variations in our microbial community structures and functions. Variation in the human microbiome within and between our various body habitats, lifecycle stages, and cultural settings is largely unexplored. High-resolution time series studies provide a foundation for discriminating between 'normal' perturbations and pathologic states, and between organisms that are simply passing through a body habitat or are entrenched residents of an ecosystem. Similarly, these types of studies are needed to understand the immigration and emigration patterns of microbes between our body sites, between cohabitating individuals, and between ourselves and the myriad of environments we contact on a daily basis [1-3].

The densest human microbiome time series reported to date studied the response of distal gut microbial communities to the antibiotic ciprofloxacin across three individuals, with sampling intervals varied from daily to weekly. Eighteen timepoints were collected per individual in an initial study [4], and between 52 and 56 timepoints were collected per subject in a follow-up study [5]. In both studies, rapid decreases in alpha diversity and a characteristic shift in community composition were observed in association with antibiotic therapy, followed by a rapid post-antibiotic increase in diversity as the gut community returned to a state similar (but not identical) to the pre-treatment state. Another dense human microbiome time series studied 60 fecal samples over the first 2.5 years of life for a single infant, and illustrated the successional pattern in the human gut microbiota as a rapidly changing community that develops over time into a community characteristic of that found in the adult human gut [2].

The availability of radically cheaper sequencing and analysis, described here, eases constraints on the number of timepoints and body sites that can be compared in a single study, and paves the way to a finer-grained understanding of how the human microbiota changes in different body habitats (including much better estimates of the relative variability of different body sites within a subject). Understanding this intrinsic variability will be crucial for performing power calculations to test whether antibiotics, probiotics, or other drugs actually affect the microbiome.

In this study, two healthy subjects, one male (M3) and one female (F4), one of whom (M3) participated in an earlier survey [6], were sampled daily at three body sites (gut (feces), mouth, and skin (left and right palms)), for 15 months (M3) and for 6 months (F4) using an institutional review board-approved protocol. Variable region 4 (V4) of 16S rRNA genes present in each community sample were amplified by PCR and subjected to multiplex sequencing on an Illumina Genome Analyzer IIx (GA-IIx; average read length after quality trimming, 123 ± 17 (standard deviation (SD)) nucleotides; 32,266 ± 19,723 (SD) reads per sample; *n *= 1,422 (M3 samples); *n *= 531 (F4 samples); average interval between sampling, 1.12 days). To control for differences across sequencing platforms and primer pairs, 331 of these samples had variable region 2 (V2) sequenced on 454 (average read length after quality filtering, 228 ± 11 (SD) nucleotides; 1,072 ± 375 (SD) reads per sample; *n *= 171 (M3 samples); *n *= 160 (F4 samples)). This study thus provides a key counterpoint to recent studies of one or a few subjects at tens of timepoints [2-5,7], or studies that examine hundreds of individuals but only at one or a few timepoints [6,8-12].

## Results and discussion

### Stable differences in microbial communities between body sites over time

**Figure 1:**
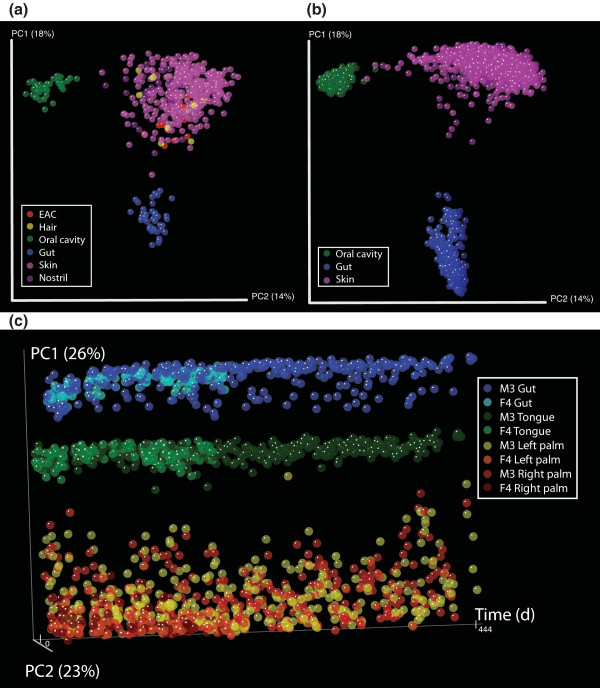
**Principal coordinates analysis of unweighted UniFrac distances between samples**. **(a) **Costello *et al*. [6] samples. **(b) **M3, F4 time series samples. **(c) **M3, F4 time series, PC1 versus time (days). Panels (a,b) and (c) show two independent principal coordinates analyses. To compare the Costello *et al*. 454 data (a) with the time series Illumina data (b), these data were generated in a single principal coordinates analysis of UniFrac distances at 500 sequences per sample. Panel (c) does not contain the 454 data, so makes use of the increased sampling depth possible on Illumina (evenly sampled to 5,000 sequences per sample for UniFrac calculations).

When the samples from Costello *et al*. [6] and the current study are compared directly, the samples cluster by body habitat, showing excellent concordance between the studies (Figure 1a in [6]; Figure 1b in the current study) despite differences in sequencing technology (454 and Illumina GA-IIx, respectively), mean read length (229 ± 16 (SD) nucleotides and 123 ± 17 (SD) nucleotides, respectively), and region of the 16S sequenced (V2 and V4, respectively). The UniFrac distances between the 331 time series samples that were sequenced on both Illumina and 454 were significantly correlated, as determined by Procrustes analysis of unweighted UniFrac principal coordinate matrices (M2 = 0.161; Monte Carlo *P *< 0.001; Additional file 1) and Pearson correlation of UniFrac distances for pairs of samples (r = 0.91; *P *< 0.001). As observed by Costello *et al*. [6], gut, oral, and skin bacterial communities were found to be compositionally distinct based on principal coordinates analysis of unweighted UniFrac distances between communities (UniFrac measures community similarity based on the degree to which they share branch length on a phylogenetic tree). The long-term time series shows for the first time that this body-site differentiation is highly stable over greater than one year (Figure 1c), but dynamic within sites over time (Additional files 2, 3, 4, 5, 6, 7, 8, 9, 10, 11, 12, 13, 14, 15).

### Minimal evidence for a temporal core microbiome between or within body sites

**Figure 2:**
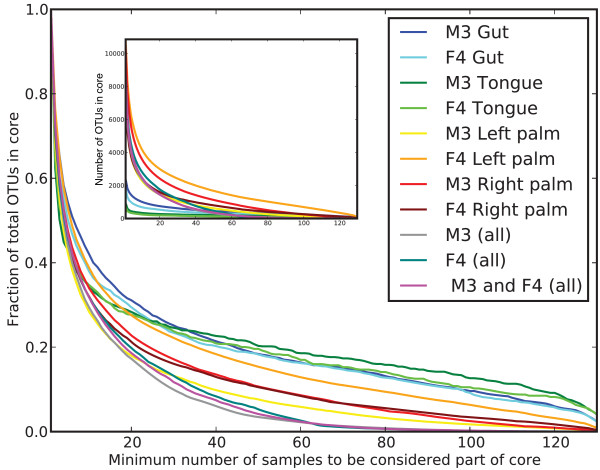
**Temporal core microbiome**. Fraction of species-level operational taxonomic units (OTUs) composing the core microbiota by number of samples in which an OTU must be present to be considered part of the core.

While overall compositional differences between body sites and individuals were relatively stable, our data also suggest a surprisingly small temporal 'core human microbiota' within an individual's body sites (Figure 2) when we define the 'core' as those species-level phylotypes in a given body habitat that were observed across all sampling events. These data suggest a minimal core microbiome across time, where the size of the core decreases as: mouth > gut > right palm ≈ left palm > across body sites within an individual > across body sites and individuals.

**Figure 3:**
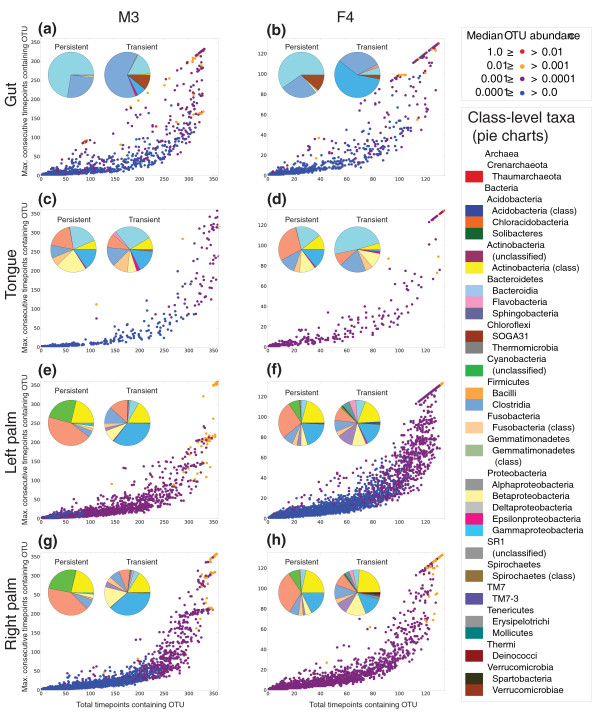
**Community membership**. Community membership summary for all OTUs in **(a) **M3 gut, **(b) **F4 gut, **(c) **M3 tongue, **(d) **F4 tongue, **(e) **M3 left palm, **(f) **F4 left palm, **(g) **M3 right palm, and **(h) **F4 right palm. Points are OTUs colored by their median relative abundance computed over all samples where they occur, and pie charts summarize the class-level taxa observed as persistent and transient OTUs.

At this depth of sequencing, many more OTUs are either persistent community members, which appear in a given body habitat and remain for an extended period of time but are not present consistently enough to be considered core members, or transient community members, which appear in a body habitat and disappear soon after (Figure 3). The taxa composing these persistent and transient categories are significantly different (for example, M3 gut: G_indep_, 84.78; *P *= 1.80 × 10^-14^). In M3 gut, both the persistent and transient communities are dominated by Clostridia, Bacteroidia, and to a lesser extent Erysipelotrichi. The persistent community is, however, also composed of Betaproteobacteria and Deltaproteobacteria, while the transient community is composed of Actinobacteria, Gammaproteobacteria, Epsilonproteobacteria, and Verrucomicrobiae. Taxonomic summaries of the persistent and transient groups for all body sites from both individuals are presented in Additional file 16.

We applied several techniques to control for the possibility of persistent groups being mistaken for transient groups if they occasionally fell below the detection threshold. First, in computing the maximum number of consecutive observations for an OTU, we counted a single zero-count for an OTU as not interrupting a run of consecutive observations, provided that both adjacent timepoints achieved non-zero counts for that OTU. Second, we re-sequenced 331 of the time series samples on the 454 platform, and recomputed the persistent and transient taxa summaries. We found that, for both individuals, the composition of the persistent gut and oral communities were not significantly different between 454 and Illumina despite a nearly 40-fold difference in sequencing depth. However, the persistent palm communities were significantly different in both cases. A possible confounding factor in this comparison is primer bias, as the V2 region was sequenced on 454. We therefore performed 1,000 jackknife iterations of this analysis by subsampling the Illumina data to 5,000 sequences per sample and recomputing the composition of the persistent group for each individual and body habitat. In this analysis, seven of the eight individual/body site pairs were never significantly different from the persistent community composition on the full Illumina data set. The one exception was the F4 gut community, which was significantly different in approximately half of the iterations. When re-sampled at a depth of 10,000 sequences per sample, the persistent F4 gut community never achieved significant difference from that determined on the full Illumina data set. The full results of these analyses are presented in Additional file 17. The designation of groups as 'persistent' was thus highly reproducible across sequencing platforms and amplicons, although it is still possible that sequencing error or very low abundance taxa occasionally falling below the detection threshold could result in underestimated size of the persistent group.

### Temporally dynamic microbial communities and correlations between body sites

Differences in UniFrac distances in the left and right palms in adjacent timepoints of both individuals were significantly correlated (Pearson correlation for M3, r = 0.69, *P *= 2.07 × 10^-46^; for F4, r = 0.64, *P *= 3.77 × 10^-16^), possibly due to equilibration of microbial communities across palms by physical contact. We did not see correlations between other body sites. While the magnitude and direction of change in phylogenetic dissimilarity between adjacent timepoints were correlated between the palm sites, the species-level microbial taxa present on each hand were not significantly correlated, confirming previous observations that, at a single timepoint, the left and right hand of a single individual may share relatively few OTUs [13].

For single body sites, within-subject distances were lower than between-subject distances, suggesting a stable pattern, consistent across body sites, in between-subject dissimilarities across time. For example, the between-subject fecal sample distances were significantly higher than the within M3 fecal sample distances (t = 15.52; *P *< 0.001; one-tailed, two sample *t*-test) and the within F4 fecal sample distances (t = 33.45; *P *≤ 0.001; one-tailed, two sample *t*-test).

The microbial community dynamics are especially apparent in principal coordinates analysis animations (Additional files 2, 3, 4, 5, 6, 7) where the sample types (subject, body site combinations) are represented as moving traces against a background of the Costello *et al*. data. The traces give an immediate picture of the variability within each body site, the relative distinctiveness of the sites and the subjects, and the relative speed of change in each site. Blooms of certain taxa contribute to the within site dissimilarities over time, as with the waning and waxing of the relative Proteobacteria abundance in the gut of both M3 and F4 (Additional files 8 and 9, Phylum panel (teal)). Similar patterns are apparent across all taxonomic levels and body sites (Additional files 8, 9, 10, 11, 12, 13, 14, 15). Such visualizations of microbial community dynamics will add another dimension to long-term studies of variable clinical states, such as inflammatory bowel disease or drug treatments, and changes in diet or lifestyle.

## Conclusions

Dense, deeply sequenced microbiome time series studies have been limited by the cost of sequencing, and the computational power necessary to analyze such studies has been expensive. Several technical advances made this study possible. First, using the Illumina sequencer and the protocol described in [14] reduced sequencing cost per sample ($11 USD) to below the cost associated with DNA extraction and PCR ($13). Second, cloud computing using Amazon Web Services (AWS) allowed us to perform the bioinformatics analysis for $200 using open-source and freely available software running on commodity services. This included the creation of a virtual cluster on AWS composed of 20 eight-core systems with 68 GB of RAM each (that is, 20 Amazon m2.4xlarge instances) to cluster approximately 69 million sequences into OTUs. This step alone would have required 20 days of computation on a desktop machine with 8 GB of RAM, but was performed on this virtual cluster in 3 hours for $120.

Figure 2 illustrates the sensitivity of conclusions about the core microbiome to the definition of the core microbiome: if an OTU is considered here to be a core member only if it is present in all 130 samples, as compared to 120 of the samples, the size of the core drops to approximately 5% of OTUs from about 10% of OTUs. This rapid change in slope beyond 120 samples on the x-axis does not occur when considering all M3 gut or oral samples rather than only 130 (data not shown), suggesting that this result likely stems from a few non-representative samples, rather than representing an interesting biological result. The definition of the core microbiome is thus a crucial consideration, because depth of sampling, PCR error, sequencing error, analysis techniques (such as OTU picking method and similarity threshold), among other factors, will affect whether an OTU will be observed in a given sample. OTU picking similarity threshold, in particular, will have a large affect on the size of the observed core microbiome. As this threshold is decreased, OTUs represent larger taxonomic groups, and the observed core will increase in size correspondingly. For example, there is clearly a temporal core at the phylum level in the fecal samples studied here composed of Bacteriodetes and Firmicutes (Additional files 8 and 9). For this reason our results do not necessarily contradict previous Denaturing Gradient Gel Electrophoresis (DGGE)-based work in this area [4,15-17], which is suggestive of a core gut microbiome but at a coarser phylogenetic resolution and more limited dynamic range. Sampling depth will also considerably affect the definition of the core, and although the dynamic range of this study is large compared to other studies of human body habitats, it is possible that microbes we define as 'non-core' still persist at very low abundance, perhaps in a manner analogous to a seed bank. Determining the role, if any, of these low-abundance microbes in responding to changes in diet, physiological status, and so on will be a fascinating challenge for future studies.

Taken together, our observations paint a picture of a human microbiome that is highly variable over time (Figures 2 and 3; Additional files 2, 3, 4, 5, 6, 7, 8, 9, 10, 11, 12, 13, 14, 15), but from which stable patterns of similarities and differences among body habitats and individuals emerge (Figure 1). This temporal variation may arise from extrinsic factors, such as exposure to different types of foods, medications, or physical environments (for example, due to travel), or from intrinsic factors, such as the adaptive immune system. Understanding the influence of these factors on an individual's microbiota is an additional challenge for future work.

Although some evidence was found for a high-abundance core microbiome at the 97% OTU level within body sites over time, particularly in the mouth and gut, this core appears to represent less than 10% of the total OTUs when defining the minimum number of samples to be considered part of the core as 90% of the samples. This observation does not, however, extend across body sites, suggesting that a body-wide core temporal microbiome that bridges the habitat types surveyed here does not exist.

The innovations in sequencing, cloud computing, and visualization applied here, together with advances in robust and inexpensive microfluidic sample preparation techniques, support the development and democratization of inexpensive, informative, and personalized microbiome-based phenotyping. Specifically, the ability to collect, process and analyze thousands of sequences using increasingly available sequencing technologies and using commercial computing infrastructure will make the ability to trace changes in the microbiome within each individual associated with drug administration, disease states, and environmental exposures routine. Because of the immense subject-to-subject variability in the microbiome, studies examining temporal variability, which give a view of dynamics beyond the static pictures previously available, have the potential to transform our understanding of what is 'normal' in the human body, and, perhaps, to develop predictive models for the effects of clinical interventions.

## Materials and methods

### Sample preparation and sequencing

Sample collection and DNA isolation were performed as described in Costello *et al*. [6]; and PCR, sequencing, and quality filtering of reads were performed as described in Caporaso *et al*. [14]. Samples were not collected on days 422 through 437.

To facilitate massively parallel sequencing (1,967 samples), barcodes were reused across six lanes in a single Illumina GAIIx, with 374, 372, 364, 271, 265, and 323 samples in lanes 1 through 6, respectively (differing from Caporaso *et al*. [14], where samples were pooled and run over seven lanes). Sixteen samples were ultimately excluded from the analysis as fourteen samples were identified as potentially mislabeled (discussed below), and the barcodes for two samples were not found in the sequencing output, likely indicating a problem with amplification for those two samples.

### Data analysis

To directly compare these M3/F4 time series samples with the samples presented in Costello *et al*. [6], which sequenced a different variable region (V2) using a different technology (454 FLX), a reference-based OTU picking protocol [18] was applied. After demultiplexing and quality filtering sequences, 97% OTUs were picked against the Greengenes database [19] (pre-filtered at 97% identity) using uclust [20]. Reads were assigned to OTUs based on their best match to a Greengenes sequence, and reads that did not match a Greengenes sequence at 97% or greater sequence identity were discarded. The Greengenes taxonomy associated with the best match in Greengenes was assigned to each OTU, and the Greengenes tree was used for phylogenetic diversity calculations. These steps and subsequent data analysis were performed using Quantitative Insights Into Microbial Ecology (QIIME) on AWS.

### Identifying mislabeled samples

To identify potentially mislabeled samples, we used the random forests classifier [21]. A 2,000-tree forest was trained on the OTU × Sample Abundance matrix after evenly sampling to 500 sequences per sample and removing OTUs present in less than 1% of samples. The posterior probability that a given sample came from each of the body habitats (gut, oral cavity, skin) was estimated using only those trees in the forest that did not contain that sample in their training sets, to avoid overfitting. The classifier considers samples to be mislabeled when their alleged environment labels have a low posterior probability (<60%). Fourteen such samples were identified, and these samples were removed from all analyses.

### Core microbiome calculation

The temporal core microbiome across body sites and individuals (Figure 2) was computed by varying the minimum number of samples in which an OTU must be observed to be considered part of the core microbiome, and then determining the number and fraction of total OTUs observed in each site (or combination of sites) that are part of the core. To facilitate direct comparison across sample types that contained different numbers of observations (for example, M3 (all) versus M3 gut), we randomly subsampled to exactly 130 observations per sample type, corresponding to the sample type for which we had the fewest observations.

### Community membership calculations

The number of consecutive timepoints containing an OTU (Figure 3) was calculated as the maximum number of consecutive timepoints where an OTU was observed, allowing a zero count at a single timepoint to be considered part of a continuous stretch of non-zero counts if both adjacent timepoints had a non-zero count. This controls for sampling error as, for example, a long contiguous stretch of non-zero counts for an OTU interrupted by a single zero count for that OTU would likely indicate a bad sample, rather than a biologically relevant fact about that OTU in relation to the community. Persistent taxa were defined as those observed in 20% or more of the timepoints, but with at least 90% of those observations being consecutive (that is, they appear and remain present). Transient taxa were defined as those observed in at least 60% of the samples, but with at most 75% of those observations being consecutive (that is, they appear and disappear from the community frequently).

### Animated microbial community dynamics

Animations were created in inVUE [22] based on the principal coordinate data presented in Figure 1a, b. inVUE files can be created in QIIME from the principal coordinate matrix and associated metadata file. After installing and opening inVUE, the user can run, pause, and stop the animations associated with different metadata categories.

### Data availability

All sequence data and sample metadata are publicly available under the 'Moving Pictures of the Human Microbiome' project [MG-RAST:4457768.3-4459735.3].

## Abbreviations

AWS: Amazon Web Services; DGGE: Denaturing Gradient Gel Electrophoresis; F4: female subject 4; GA-IIx: Genome Analyzer IIx M3: male subject 3; OTU: operational taxonomic unit; PCR: polymerase chain reaction; QIIME: Quantitative Insights Into Microbial Ecology; SD: standard deviation; V2: variable region 2; V4: variable region 4.

## Authors' contributions

JGC performed data analysis and wrote the manuscript; CLL assisted with study design, sample preparation and sequencing, and provided feedback on the manuscript; EKC assisted with study design and sample collection, and provided feedback on the manuscript; DB assisted with sample preparation and sequencing, and provided feedback on the manuscript; AG and JS assisted with data analysis; DK assisted with data analysis and provided feedback on the manuscript; PG and JR provided assistance with generation of InVUE animated plots; NF and JIG conceived of the study and provided feedback on the manuscript; and RK conceived of the study and wrote the manuscript. All authors have read and approved the manuscript.

## Supplementary Material

Additional file 1 **Comparison of beta diversity results for 331 samples sequenced on both 454 and Illumina**. Procrustes plot comparing principal coordinates of unweighted UniFrac distances. Lines connect paired samples sequences on 454 (white tip of line) and Illumina (red tip of line). The Illumina samples were evenly sampled to 5,000 sequences per sample and the 454 samples were evenly sampled to 500 sequences per sample. Click here for file

Additional file 2 **Animation tracing change in position in PC1 and PC2 with time for all body sites across both individuals**. The view presented in this video is directly comparable with Figure 1a. Background colors correspond to Figure 1a. The M3 time series is shown as a red trace (with left palm in orange), and the F4 time series is shown as a blue trace (with left palm in white). Click here for file

Additional file 3 **Animation tracing change in position in PC1 and PC2 with time for all body sites across M3**. The view presented in this video is directly comparable with Figure 1a. Background colors correspond to Figure 1a. The M3 time series is shown as a red trace (with left palm in orange). Click here for file

Additional file 4 **Animation tracing change in position in PC1 and PC2 with time for all body sites across F4**. The view presented in this video is directly comparable with Figure 1a. Background colors correspond to Figure 1a. The F4 time series is shown as a blue trace (with left palm in white). Click here for file

Additional file 5 **Animation tracing change in position in PC1, PC2 and PC3 with time for all body sites across both individuals**. Background colors correspond to Figure 1a. The M3 time series is shown as a red trace (with left palm in orange), and the F4 time series is shown as a blue trace (with left palm in white). Click here for file

Additional file 6 **Animation tracing change in position in PC1, PC2 and PC3 with time for all body sites across M3**. Background colors correspond to Figure 1a. The M3 time series is shown as a red trace (with left palm in orange). Click here for file

Additional file 7 **Animation tracing change in position in PC1, PC2 and PC3 with time for all body sites across F4**. Background colors correspond to Figure 1a. The F4 time series is shown as a blue trace (with left palm in white). Click here for file

Additional file 8 **Temporal variation in phylum, class, order, family, and genus abundances (M3 gut)**. The x-axis scale differs between M3 and F4 plots. Click here for file

Additional file 9 **Temporal variation in phylum, class, order, family, and genus abundances (F4 gut)**. The x-axis scale differs between M3 and F4 plots. Click here for file

Additional file 10 **Temporal variation in phylum, class, order, family, and genus abundances (M3 tongue)**. The x-axis scale differs between M3 and F4 plots. Click here for file

Additional file 11 **Temporal variation in phylum, class, order, family, and genus abundances (F4 tongue)**. The x-axis scale differs between M3 and F4 plots. Click here for file

Additional file 12 **Temporal variation in phylum, class, order, family, and genus abundances (M3 left palm)**. The x-axis scale differs between M3 and F4 plots. Click here for file

Additional file 13 **Temporal variation in phylum, class, order, family, and genus abundances (F4 left palm)**. The x-axis scale differs between M3 and F4 plots. Click here for file

Additional file 14 **Temporal variation in phylum, class, order, family, and genus abundances (M3 right palm)**. The x-axis scale differs between M3 and F4 plots. Click here for file

Additional file 15 **Temporal variation in phylum, class, order, family, and genus abundances (F4 right palm)**. The x-axis scale differs between M3 and F4 plots. Click here for file

Additional file 16 **Taxonomic summary of the persistent and transient OTUs for all individual, body site pairs**. Click here for file

Additional file 17 **Detailed results of persistent versus transient community compositions when compared on 454 time series and jackknife analysis**. Click here for file
