# Supplementary figures and images for: Moving pictures of the human microbiome
Source: Genome Biol. 2011 May 30;12(5):R50. doi: 10.1186/gb-2011-12-5-r50 (PMC3271711; doi:10.1186/gb-2011-12-5-r50)

PC1 (25%)

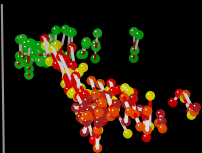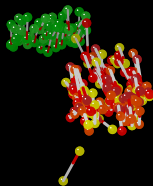

- M3 Gut
- F4 Gut
- M3 Tongue
- F4 Tongue
- M3 Left palm
- F4 Left palm
- M3 Right palm
- F4 Right palm

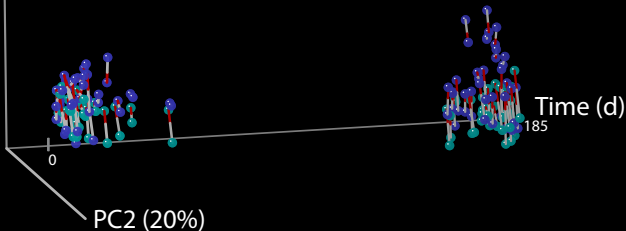

Supplement: Additional file 1 — Comparison of beta diversity results for 331 samples sequenced on both 454 and Illumina. Procrustes plot comparing principal coordinates of unweighted UniFrac distances. Lines connect paired samples sequences on 454 (white tip of line) and Illumina (red tip of line). The Illumina samples were evenly sampled to 5,000 sequences per sample and the 454 samples were evenly sampled to 500 sequences per sample. [file gb-2011-12-5-r50-S1.PDF]

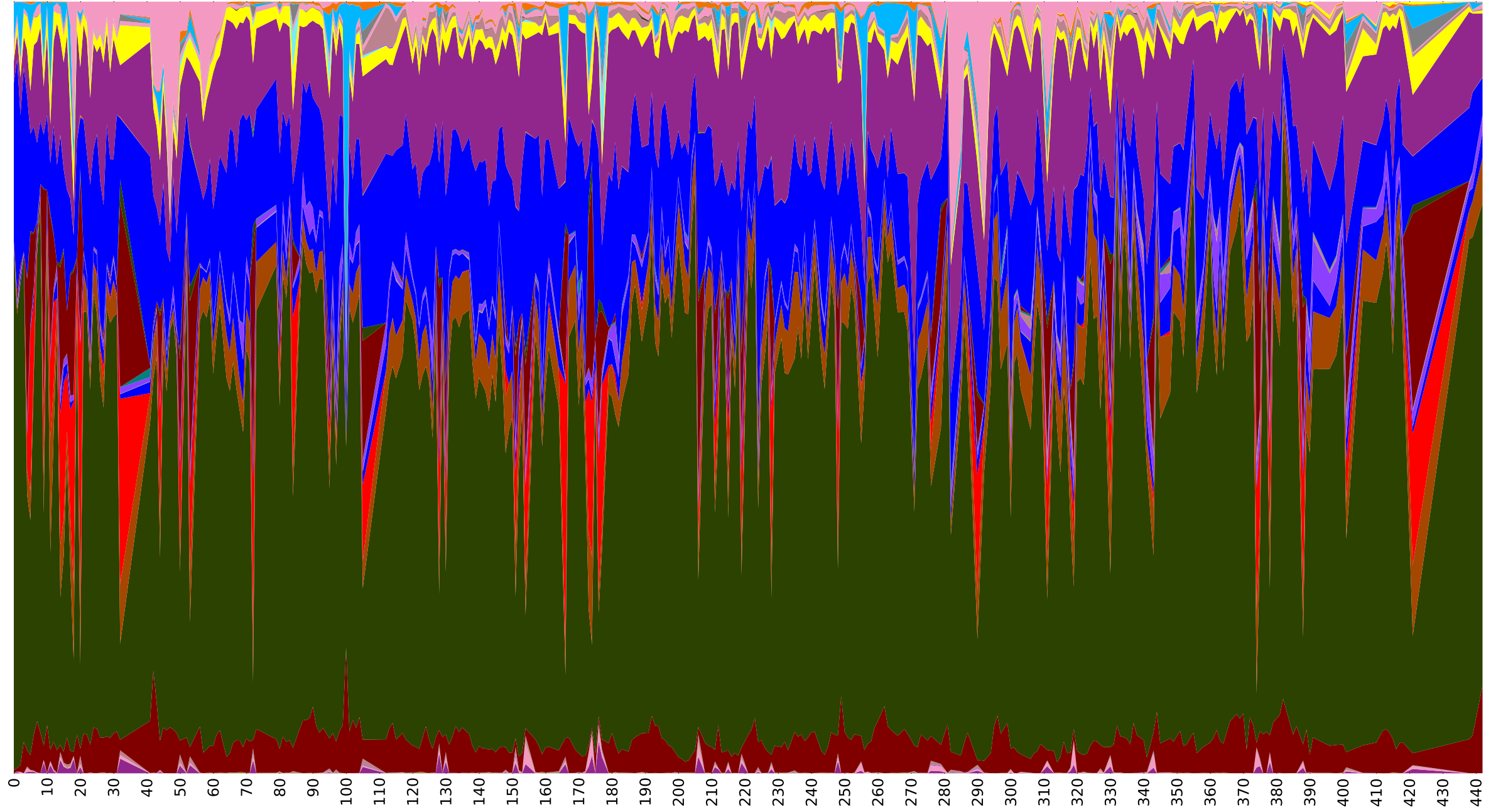

Supplement: Additional file 8 — Temporal variation in phylum, class, order, family, and genus abundances (M3 gut). The x-axis scale differs between M3 and F4 plots. [file gb-2011-12-5-r50-S8.ZIP › AdditionalFile8/charts/7Uke1YII7SaYuy5EOIkYP6J0m5Z94J.pdf]

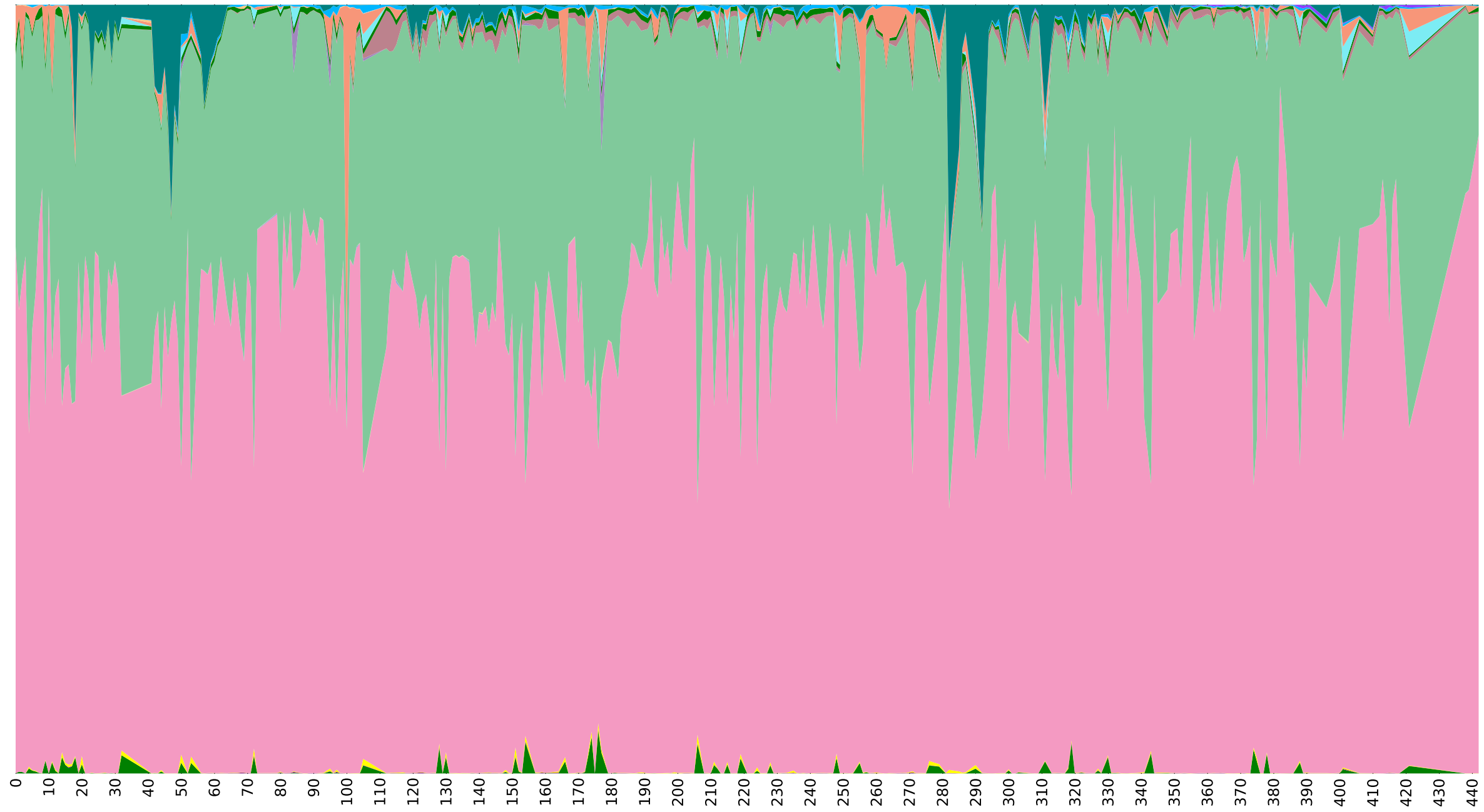

Supplement: Additional file 8 — Temporal variation in phylum, class, order, family, and genus abundances (M3 gut). The x-axis scale differs between M3 and F4 plots. [file gb-2011-12-5-r50-S8.ZIP › AdditionalFile8/charts/c9qAGYsrrBbTuLcmtZhzMzxym9hob0.pdf]

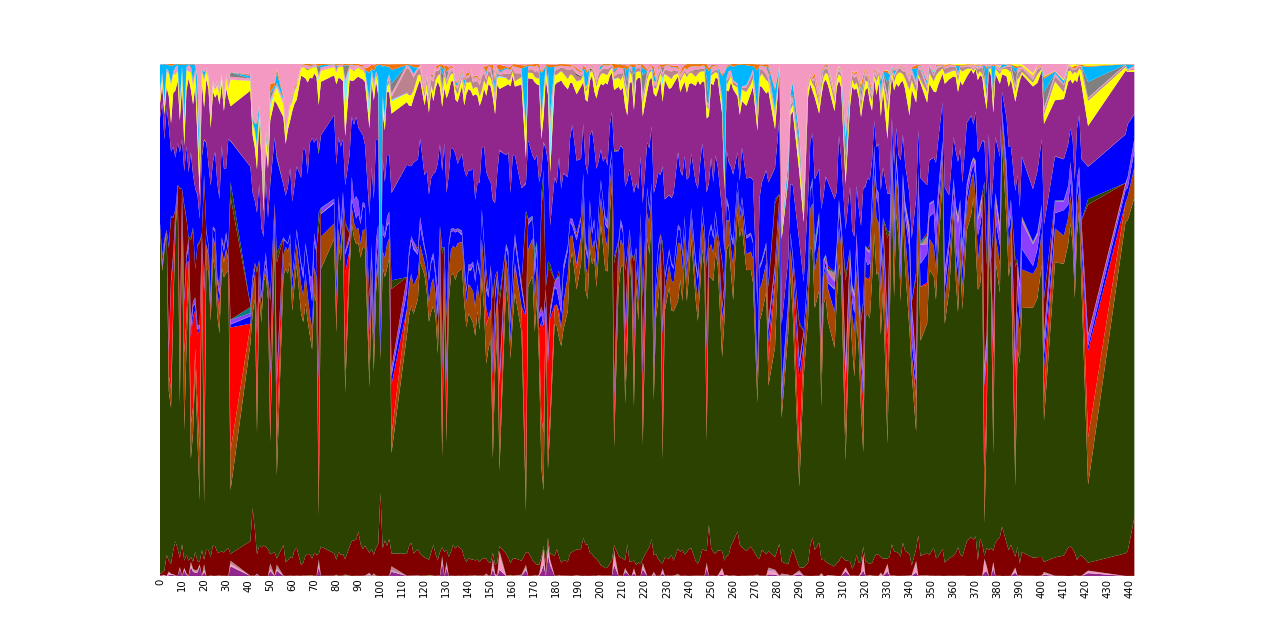

Supplement: Additional file 8 — Temporal variation in phylum, class, order, family, and genus abundances (M3 gut). The x-axis scale differs between M3 and F4 plots. [file gb-2011-12-5-r50-S8.ZIP › AdditionalFile8/charts/EX1Ug2hBLn0QT48pL9cSeTLtE2cYs1.png]

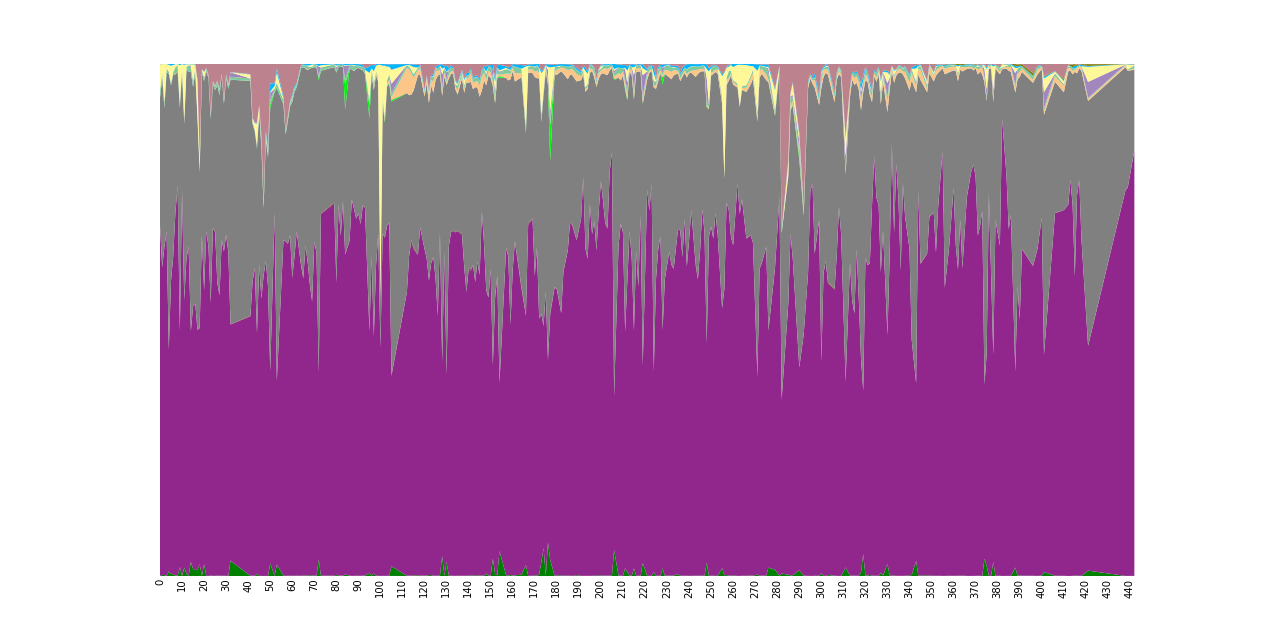

Supplement: Additional file 8 — Temporal variation in phylum, class, order, family, and genus abundances (M3 gut). The x-axis scale differs between M3 and F4 plots. [file gb-2011-12-5-r50-S8.ZIP › AdditionalFile8/charts/Ffo5hJJrOJuNRDqsbycjQhYIUQIqNb.png]

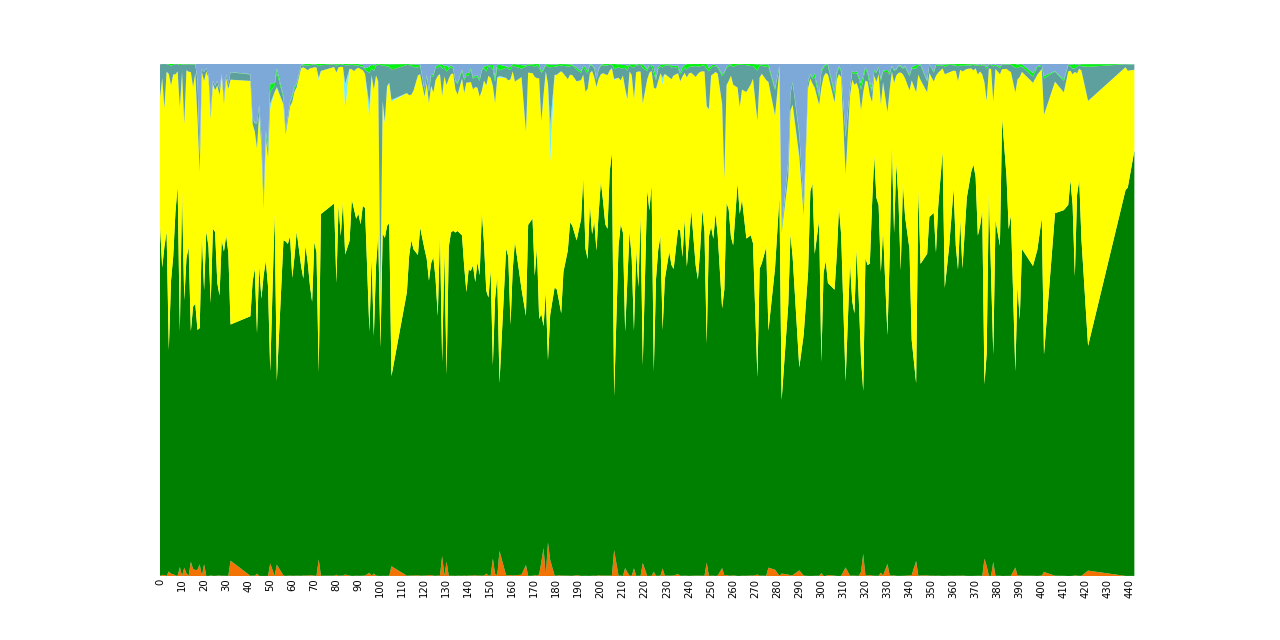

Supplement: Additional file 8 — Temporal variation in phylum, class, order, family, and genus abundances (M3 gut). The x-axis scale differs between M3 and F4 plots. [file gb-2011-12-5-r50-S8.ZIP › AdditionalFile8/charts/jsZYfyxzst5NP8DOOxLmtBl2hfGzmE.png]

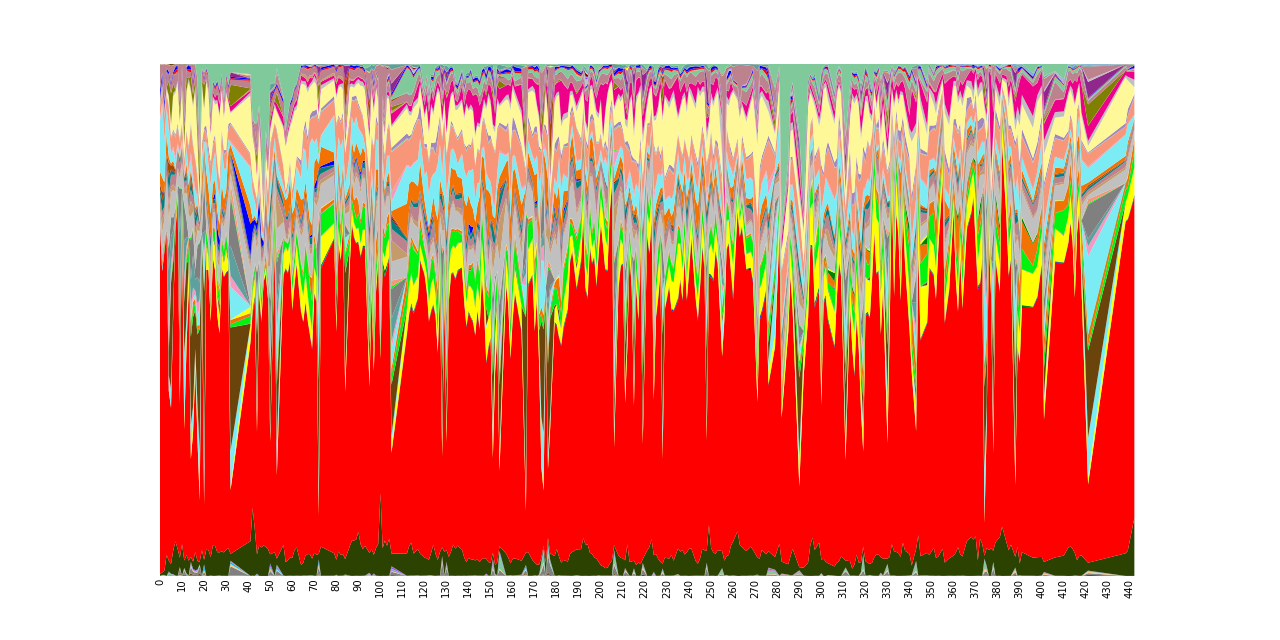

Supplement: Additional file 8 — Temporal variation in phylum, class, order, family, and genus abundances (M3 gut). The x-axis scale differs between M3 and F4 plots. [file gb-2011-12-5-r50-S8.ZIP › AdditionalFile8/charts/Sqcqcp4NdBDjuBsgzqgzqgWxZPioPa.png]

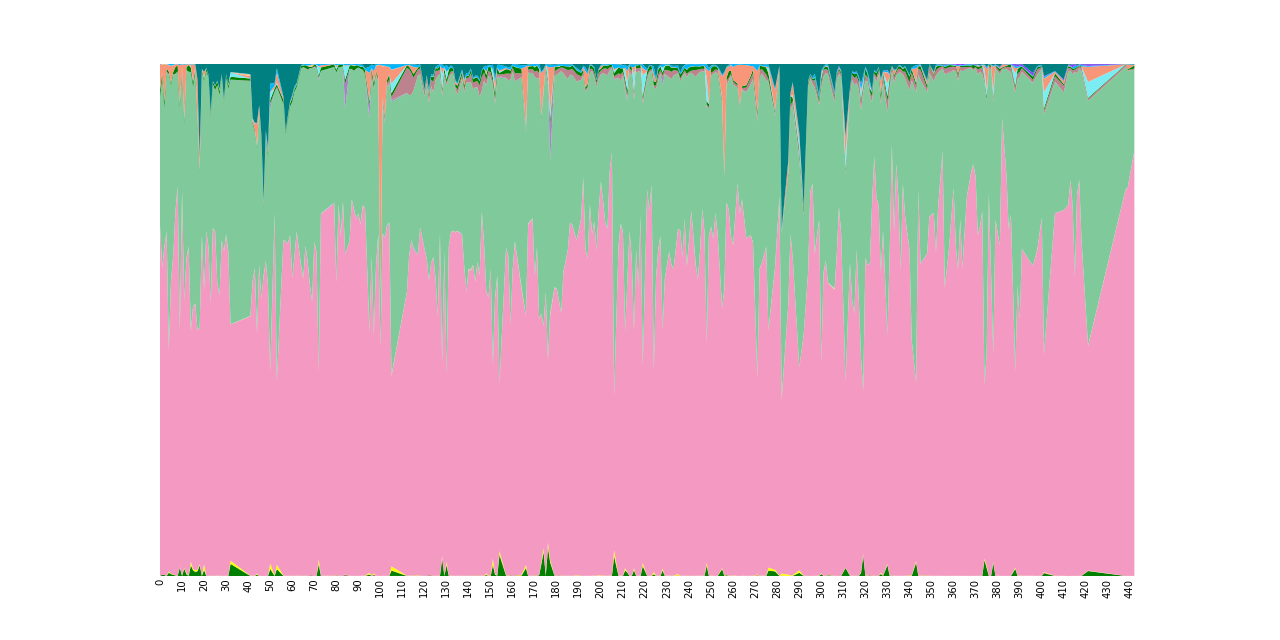

Supplement: Additional file 8 — Temporal variation in phylum, class, order, family, and genus abundances (M3 gut). The x-axis scale differs between M3 and F4 plots. [file gb-2011-12-5-r50-S8.ZIP › AdditionalFile8/charts/UEikss9E9sFp3Z2ZYKJZa71CjKGRjy.png]

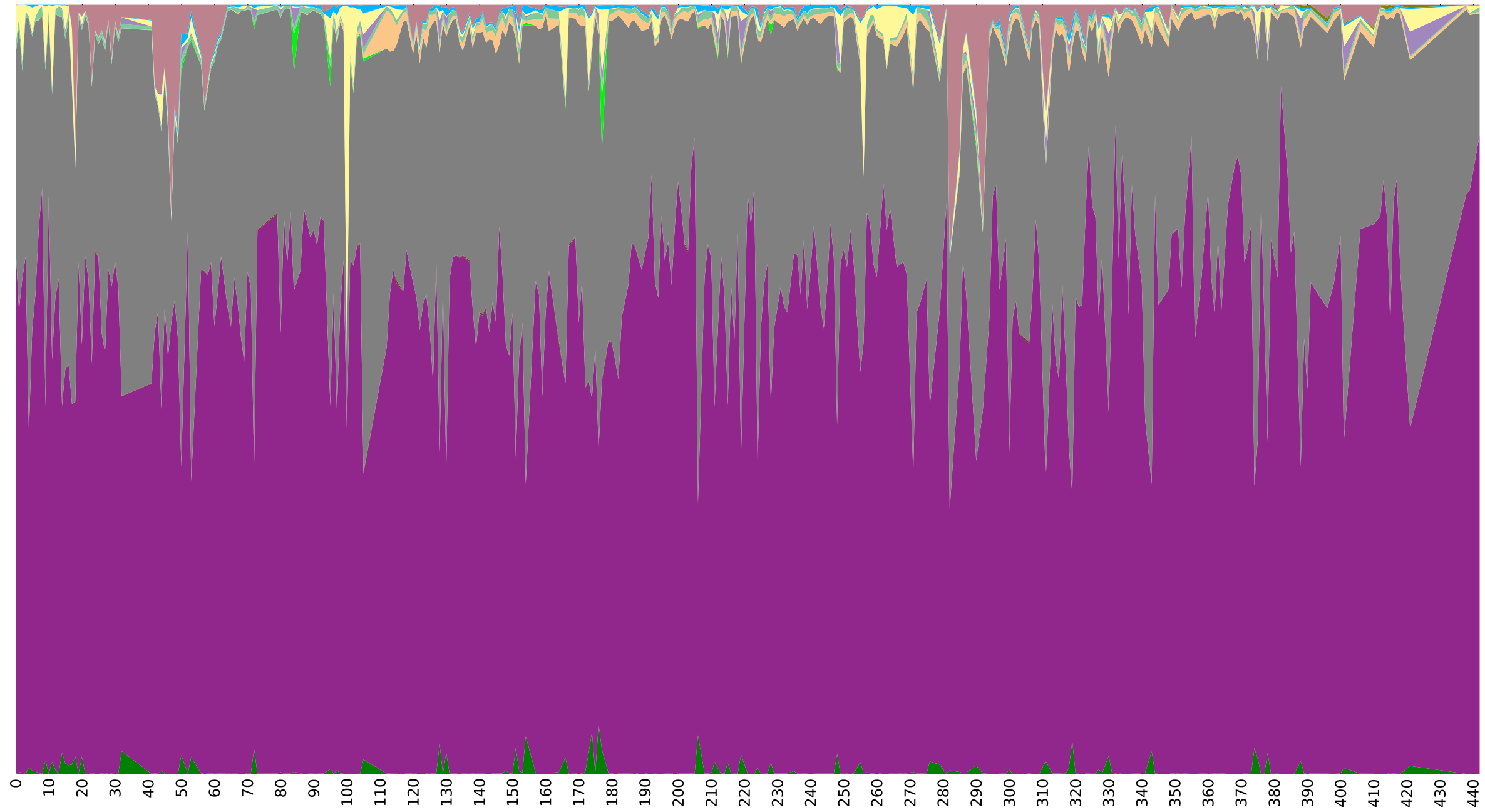

Supplement: Additional file 8 — Temporal variation in phylum, class, order, family, and genus abundances (M3 gut). The x-axis scale differs between M3 and F4 plots. [file gb-2011-12-5-r50-S8.ZIP › AdditionalFile8/charts/UkUuZ2Hs24eZW9ULoBOMuJ3XBydTzb.pdf]

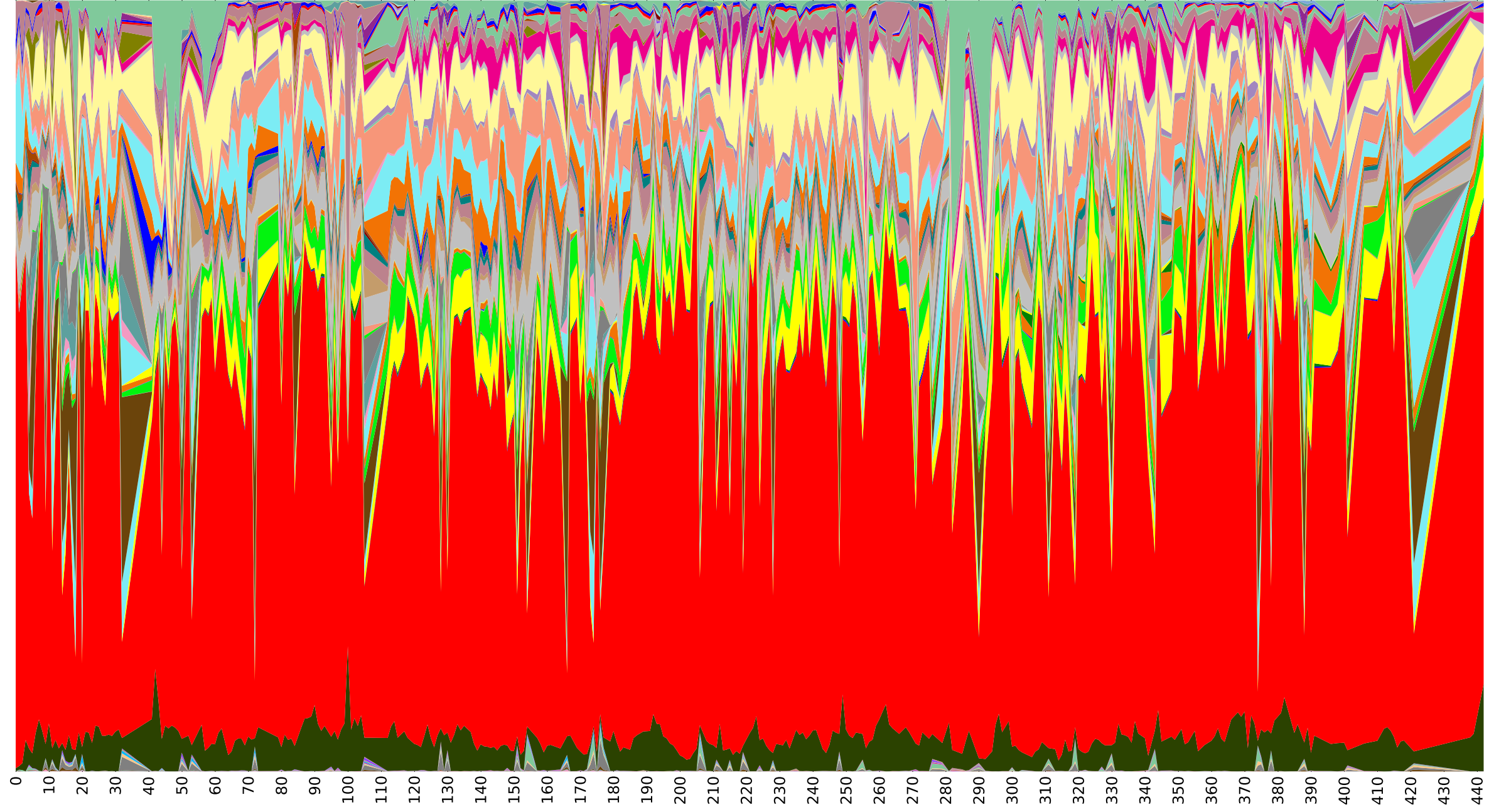

Supplement: Additional file 8 — Temporal variation in phylum, class, order, family, and genus abundances (M3 gut). The x-axis scale differs between M3 and F4 plots. [file gb-2011-12-5-r50-S8.ZIP › AdditionalFile8/charts/Zoti0UsGkzd9ND0EgFNDWXqZnonnUs.pdf]

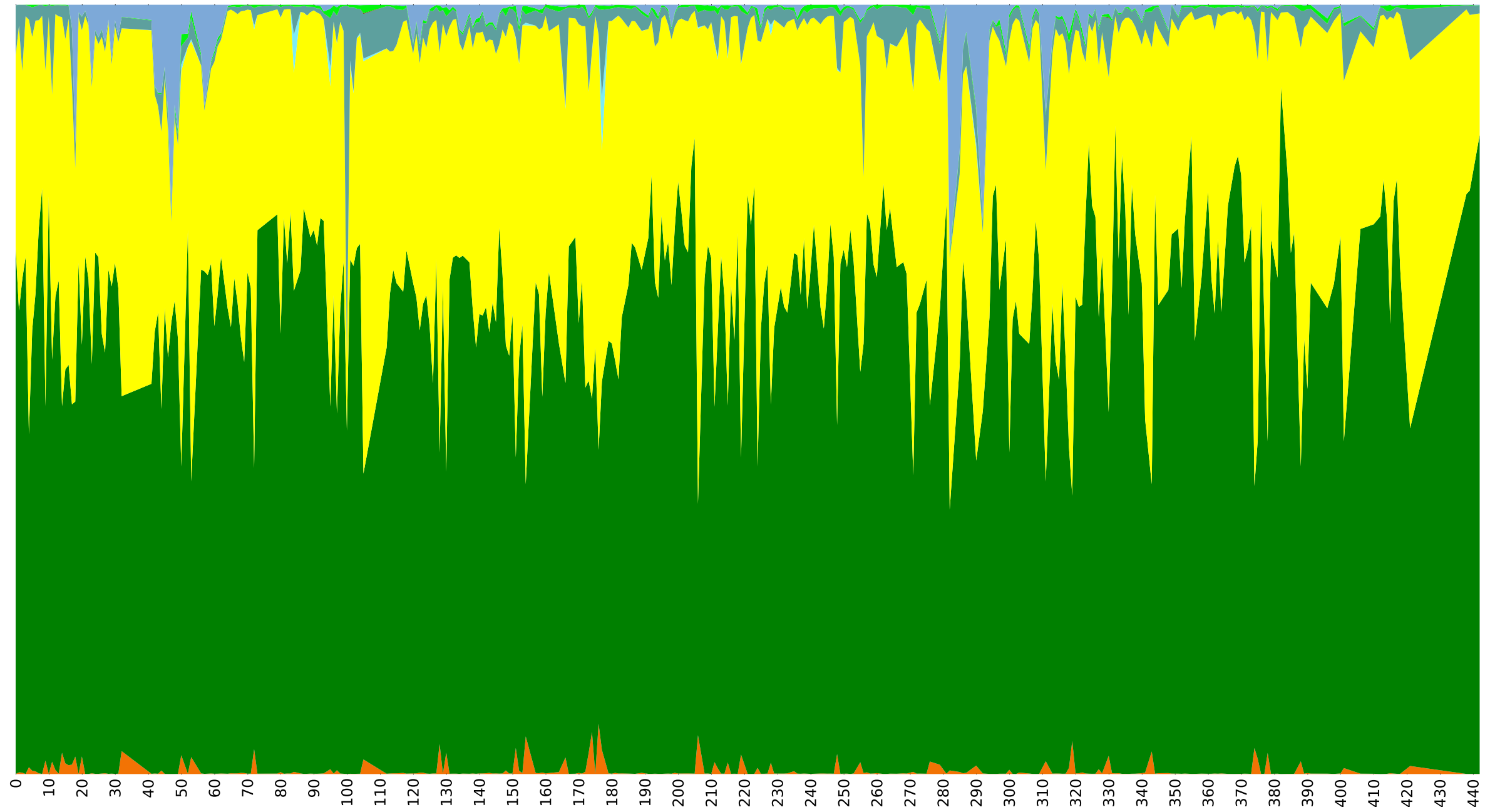

Supplement: Additional file 8 — Temporal variation in phylum, class, order, family, and genus abundances (M3 gut). The x-axis scale differs between M3 and F4 plots. [file gb-2011-12-5-r50-S8.ZIP › AdditionalFile8/charts/zOx2qsmaLyHfQOsNWYxgK53IyqRZjA.pdf]

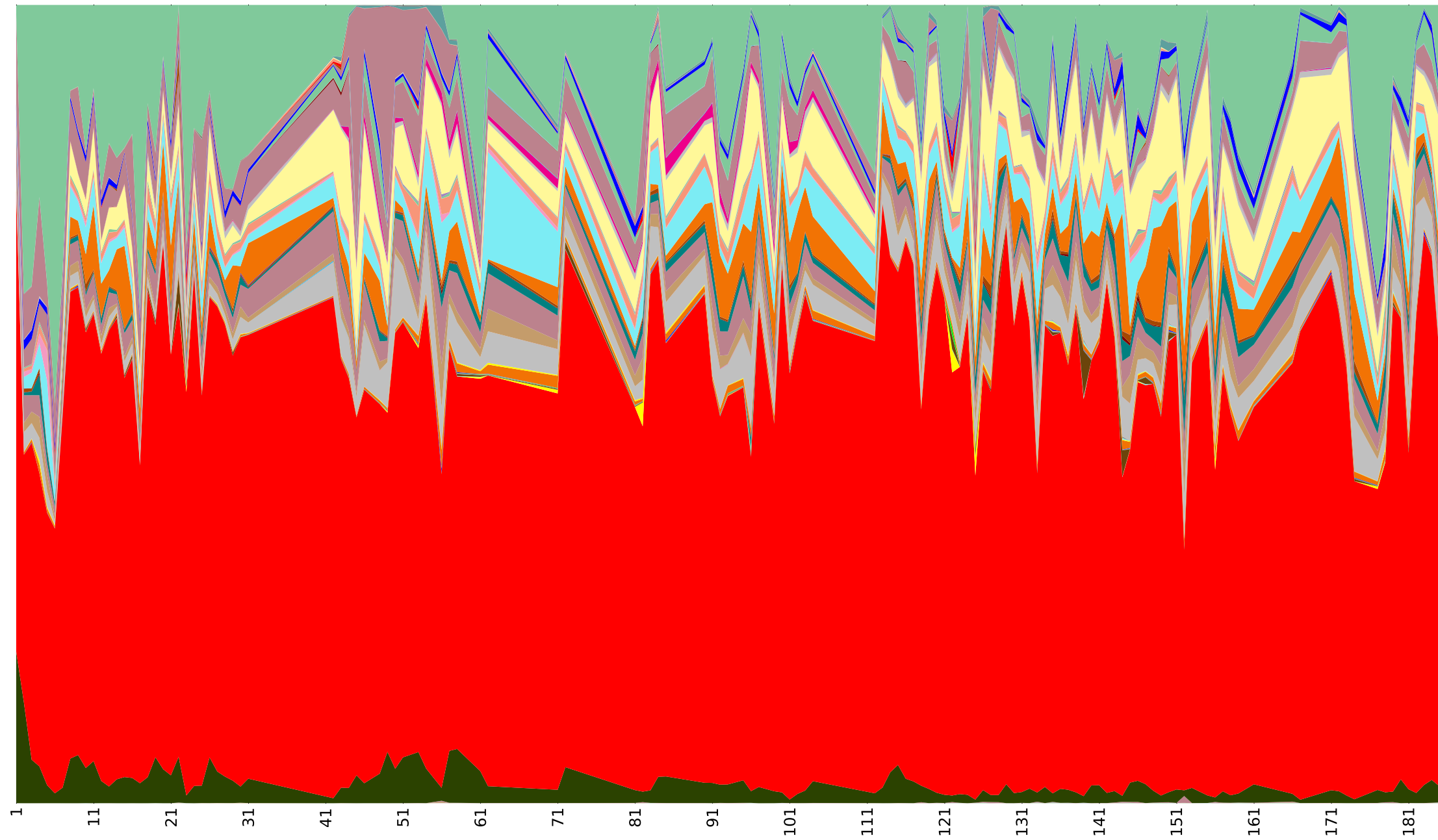

Supplement: Additional file 9 — Temporal variation in phylum, class, order, family, and genus abundances (F4 gut). The x-axis scale differs between M3 and F4 plots. [file gb-2011-12-5-r50-S9.ZIP › AdditionalFile9/charts/0CntbEZmLHFWinFNzr5DTh0C06C5z5.pdf]

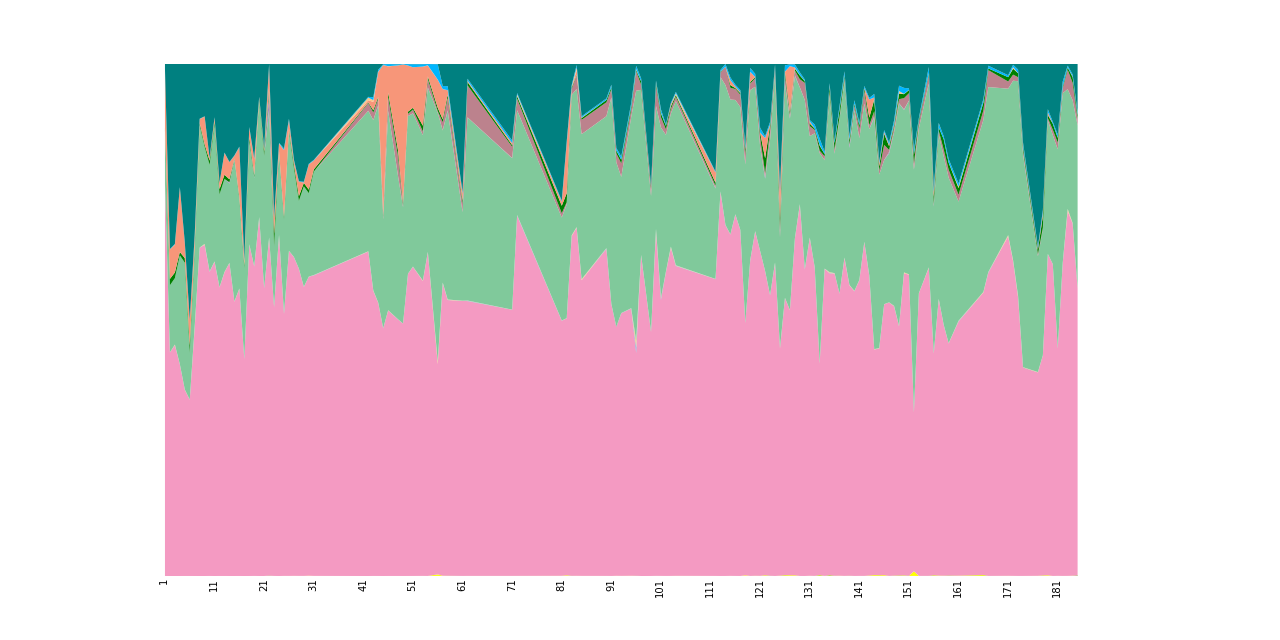

Supplement: Additional file 9 — Temporal variation in phylum, class, order, family, and genus abundances (F4 gut). The x-axis scale differs between M3 and F4 plots. [file gb-2011-12-5-r50-S9.ZIP › AdditionalFile9/charts/8R0WSmXApWzL5lsXjzj1GbezFFM6bm.png]

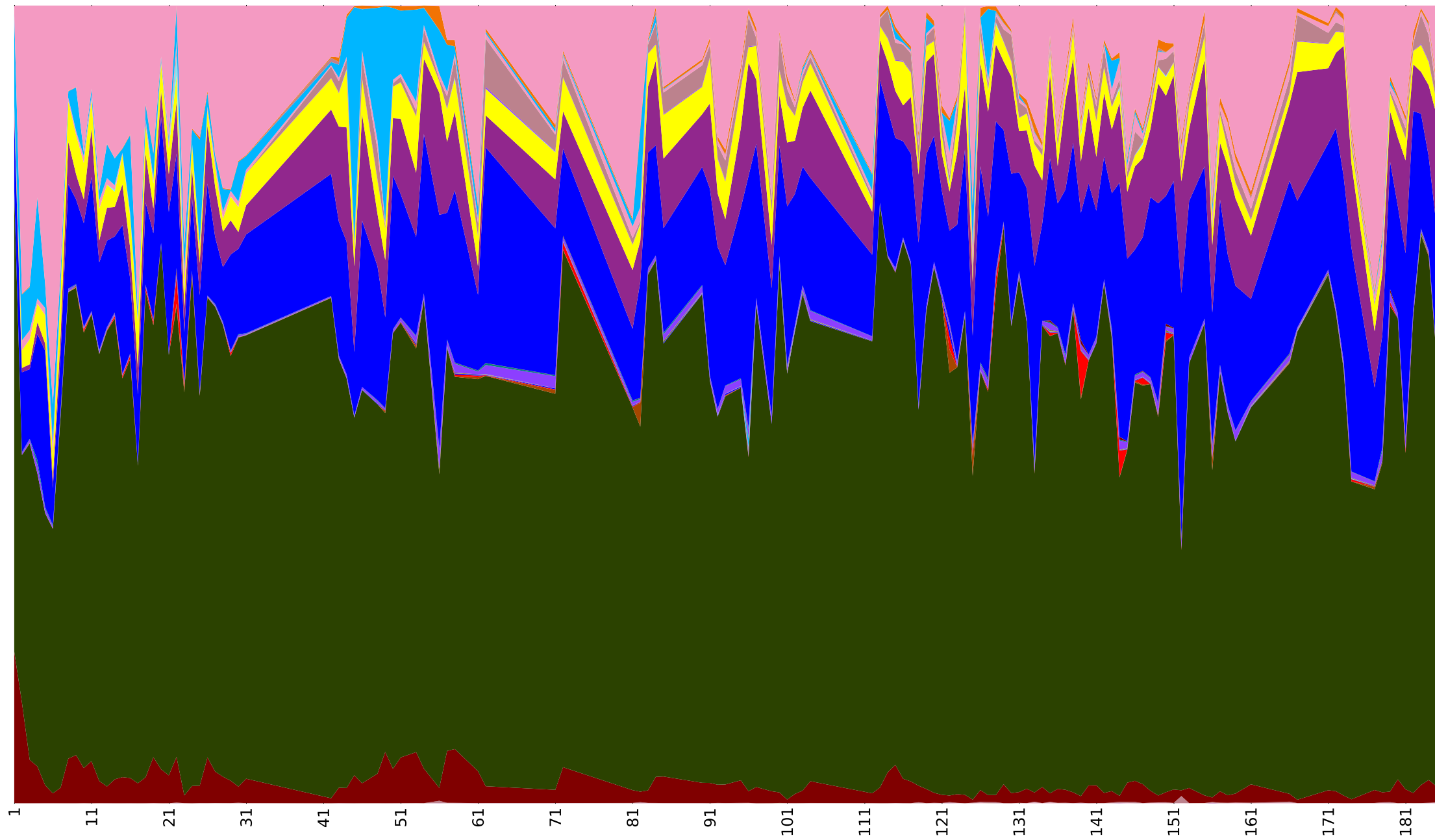

Supplement: Additional file 9 — Temporal variation in phylum, class, order, family, and genus abundances (F4 gut). The x-axis scale differs between M3 and F4 plots. [file gb-2011-12-5-r50-S9.ZIP › AdditionalFile9/charts/FnRk50Kni9ItWXg0pRDw7IqYa3b0ou.pdf]

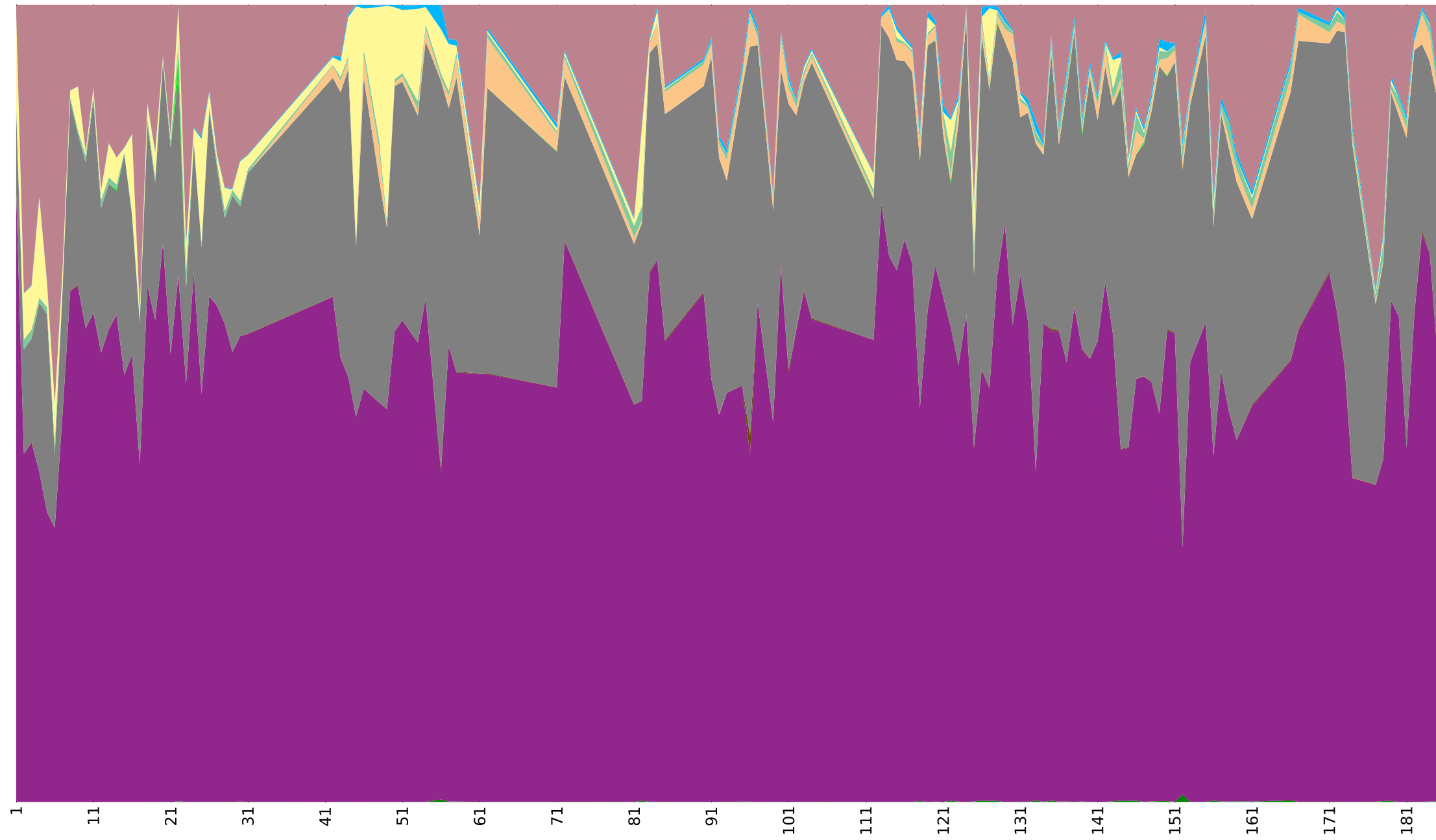

Supplement: Additional file 9 — Temporal variation in phylum, class, order, family, and genus abundances (F4 gut). The x-axis scale differs between M3 and F4 plots. [file gb-2011-12-5-r50-S9.ZIP › AdditionalFile9/charts/n7wHZKkCT7Llm5HXFQUlu8k9qpezxe.pdf]

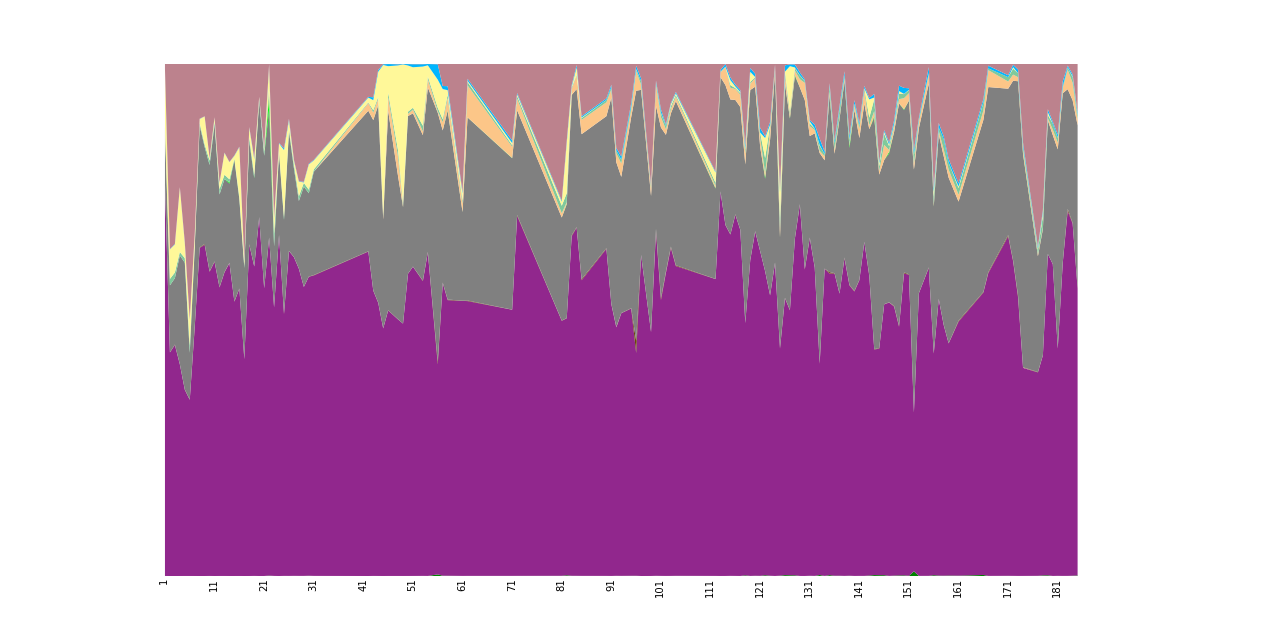

Supplement: Additional file 9 — Temporal variation in phylum, class, order, family, and genus abundances (F4 gut). The x-axis scale differs between M3 and F4 plots. [file gb-2011-12-5-r50-S9.ZIP › AdditionalFile9/charts/PHZt1WA8ZD0dzoJ8FjkQ9zjap7Zabu.png]

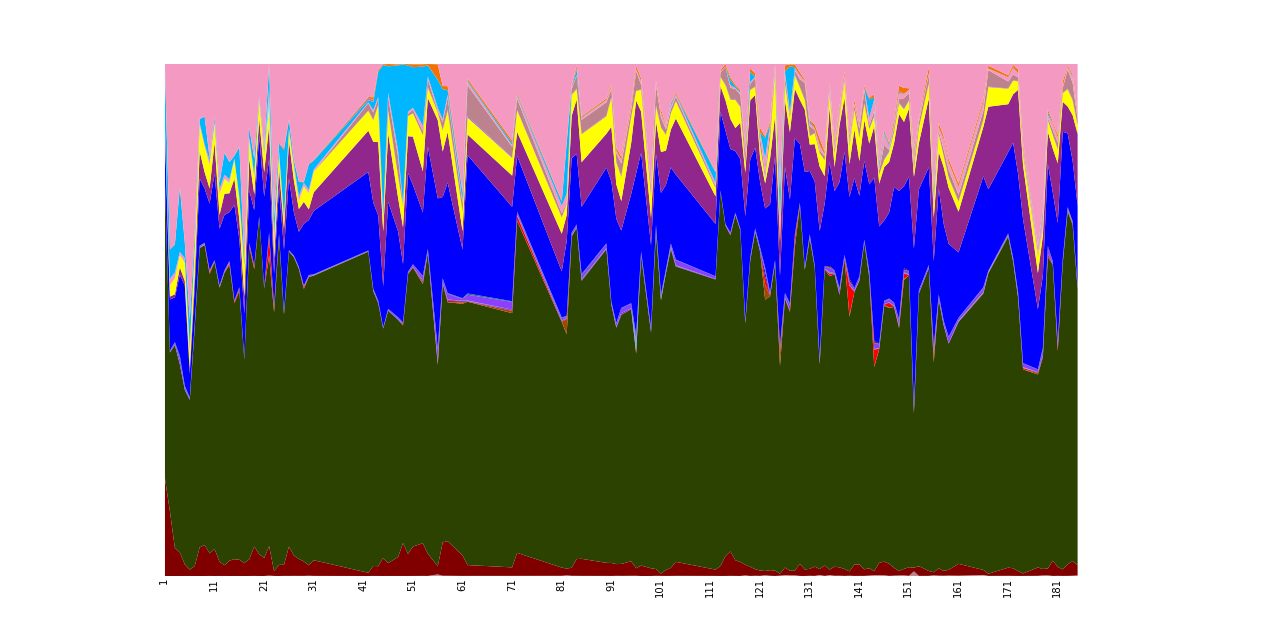

Supplement: Additional file 9 — Temporal variation in phylum, class, order, family, and genus abundances (F4 gut). The x-axis scale differs between M3 and F4 plots. [file gb-2011-12-5-r50-S9.ZIP › AdditionalFile9/charts/SDbn1Ry2rXRL4AoWrZmBrewcgZCZJc.png]

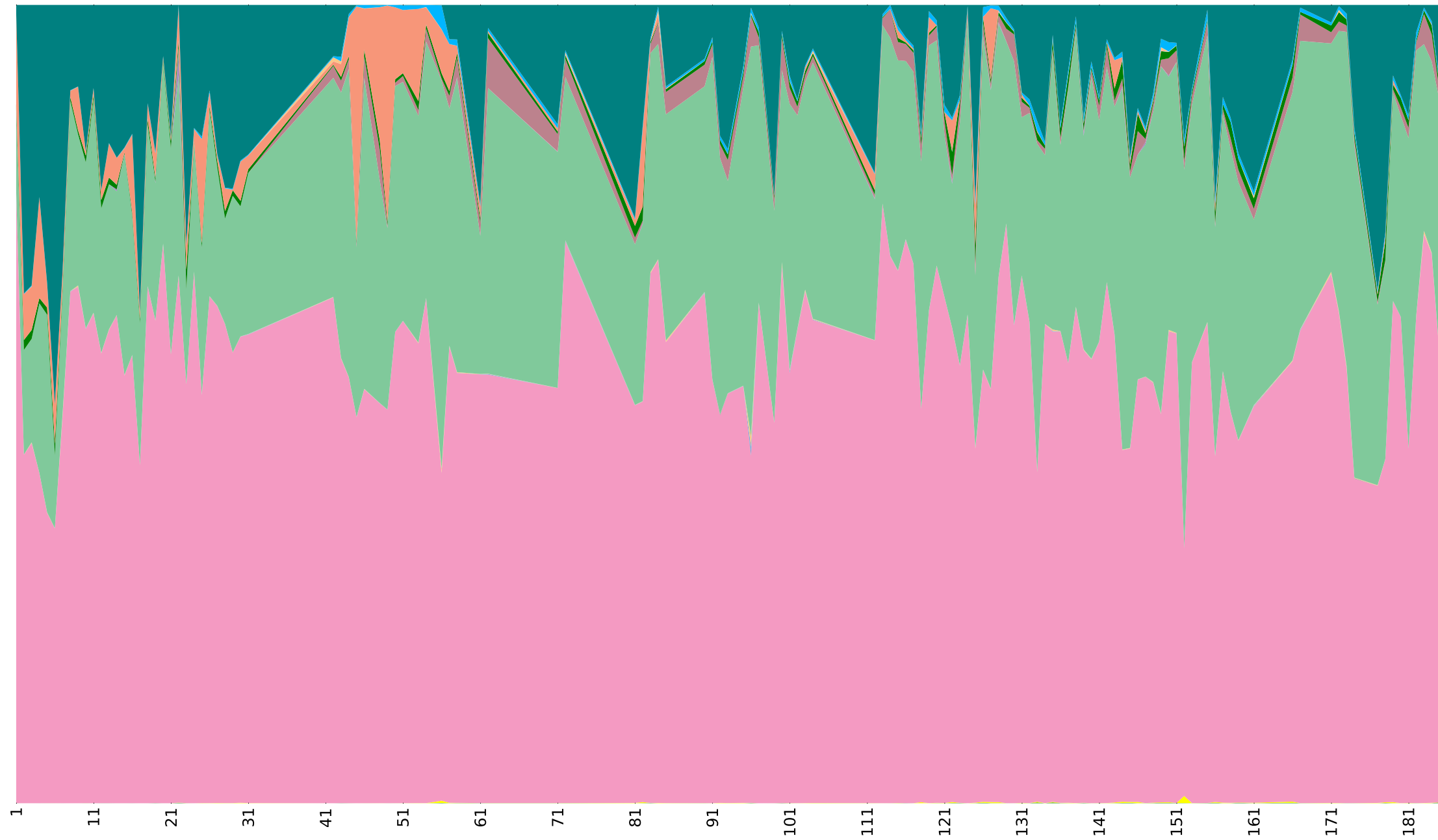

Supplement: Additional file 9 — Temporal variation in phylum, class, order, family, and genus abundances (F4 gut). The x-axis scale differs between M3 and F4 plots. [file gb-2011-12-5-r50-S9.ZIP › AdditionalFile9/charts/THliYUOOepkMNgHSjZYLqAG40i6E3D.pdf]

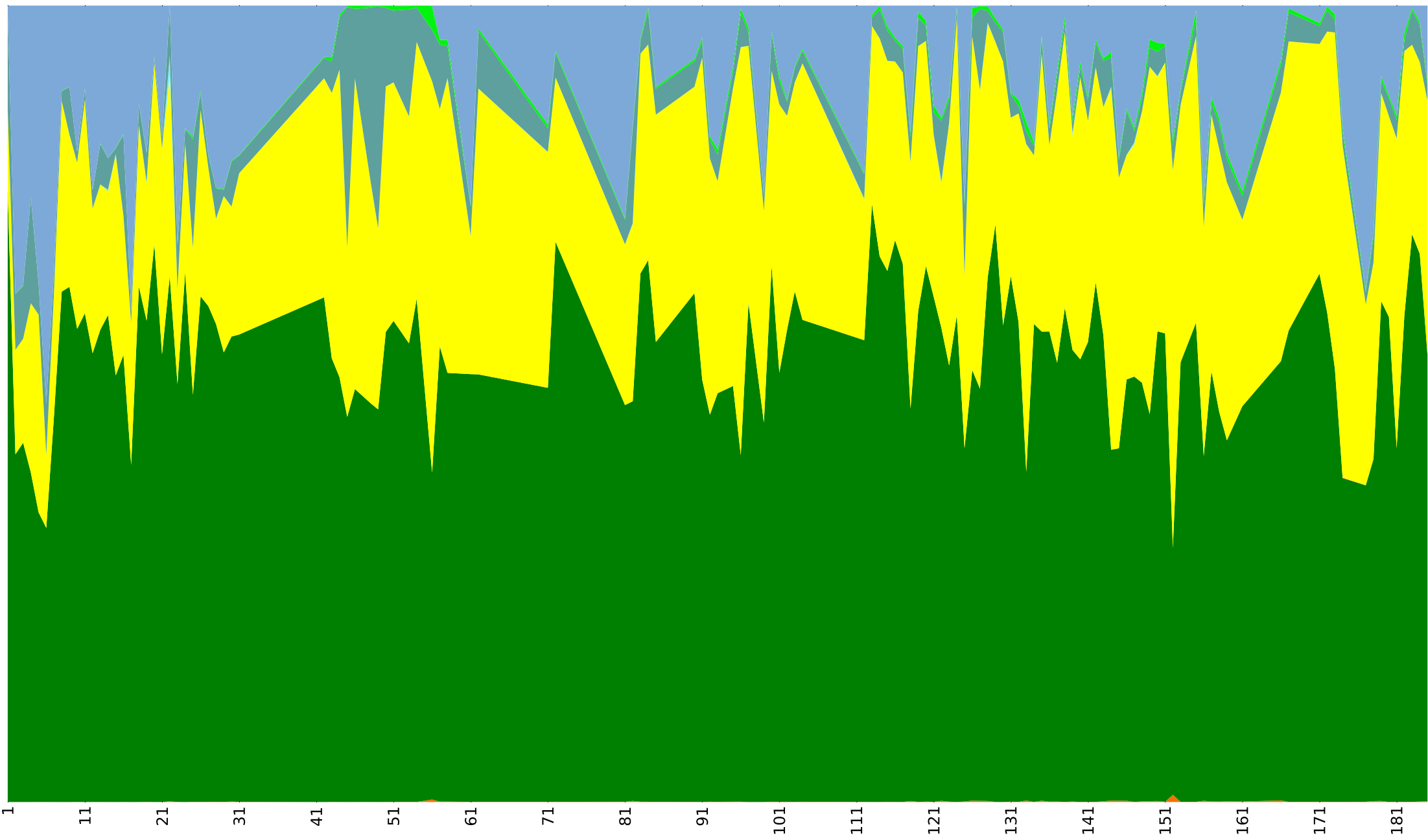

Supplement: Additional file 9 — Temporal variation in phylum, class, order, family, and genus abundances (F4 gut). The x-axis scale differs between M3 and F4 plots. [file gb-2011-12-5-r50-S9.ZIP › AdditionalFile9/charts/x9bBsWZRyuxDnj76wai5wtbF78yZjA.pdf]

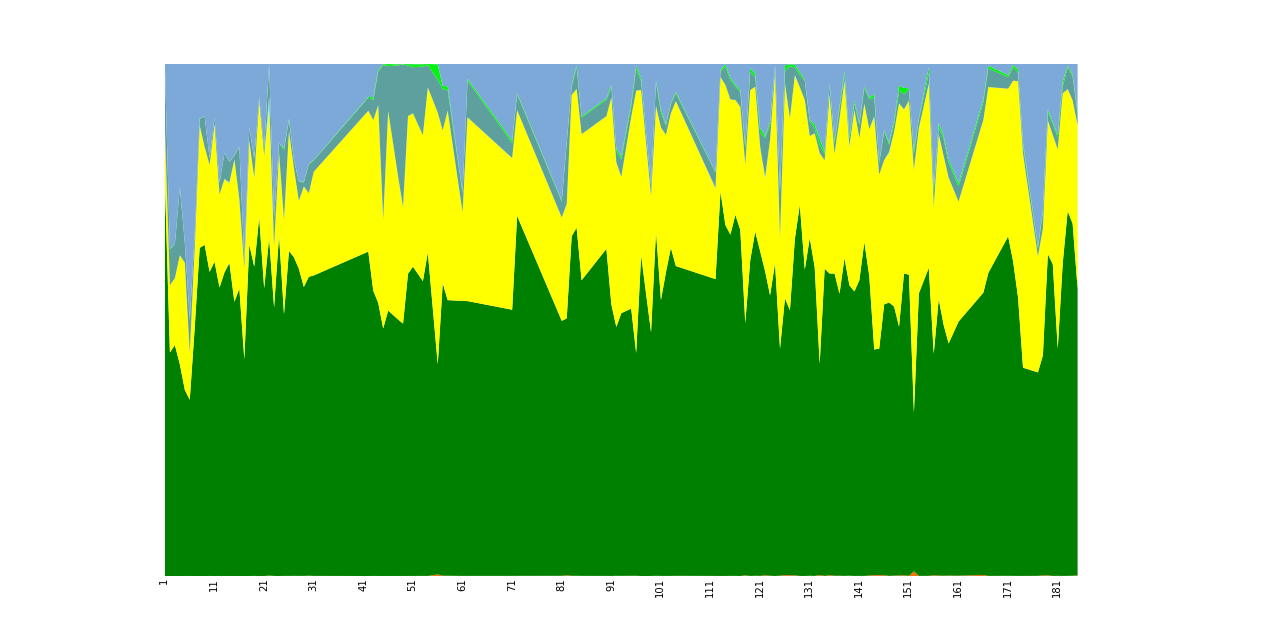

Supplement: Additional file 9 — Temporal variation in phylum, class, order, family, and genus abundances (F4 gut). The x-axis scale differs between M3 and F4 plots. [file gb-2011-12-5-r50-S9.ZIP › AdditionalFile9/charts/y1unFirMPXoefIdZq8HszSd1EI0oKU.png]

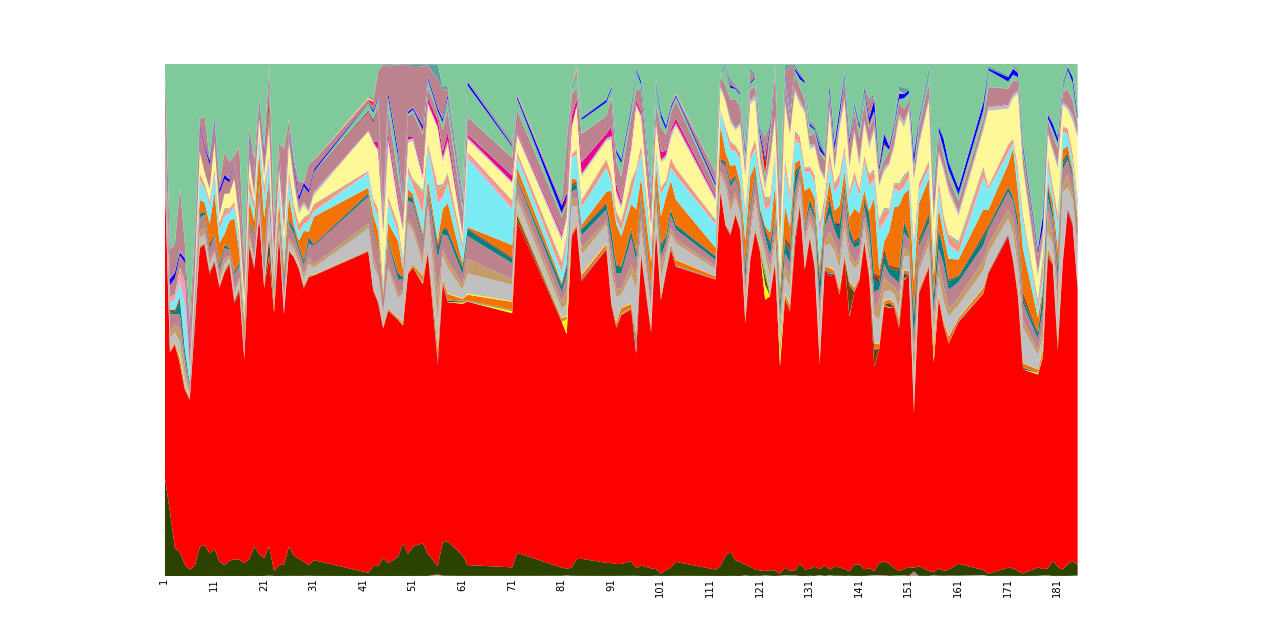

Supplement: Additional file 9 — Temporal variation in phylum, class, order, family, and genus abundances (F4 gut). The x-axis scale differs between M3 and F4 plots. [file gb-2011-12-5-r50-S9.ZIP › AdditionalFile9/charts/yT7MZHgpzbZx7M5jmDu1IqSgF8iXa8.png]

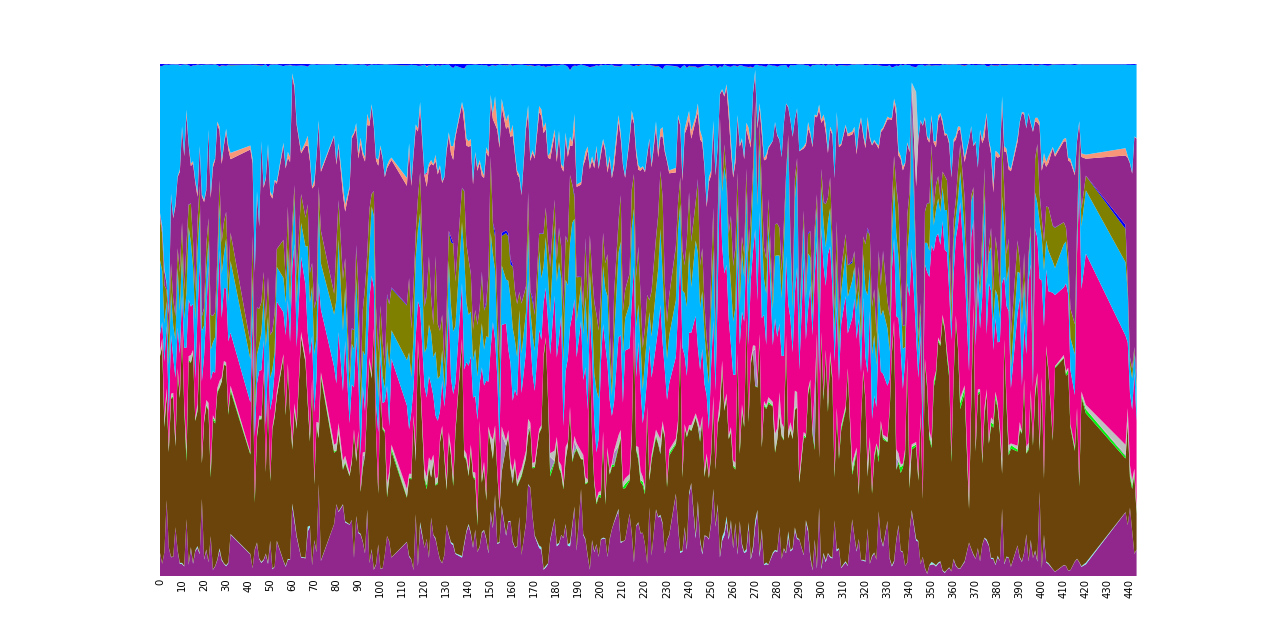

Supplement: Additional file 10 — Temporal variation in phylum, class, order, family, and genus abundances (M3 tongue). The x-axis scale differs between M3 and F4 plots. [file gb-2011-12-5-r50-S10.ZIP › AdditionalFile10/charts/7CTbajw2MLcCnALFzo7Q0b4ec6KbCh.png]

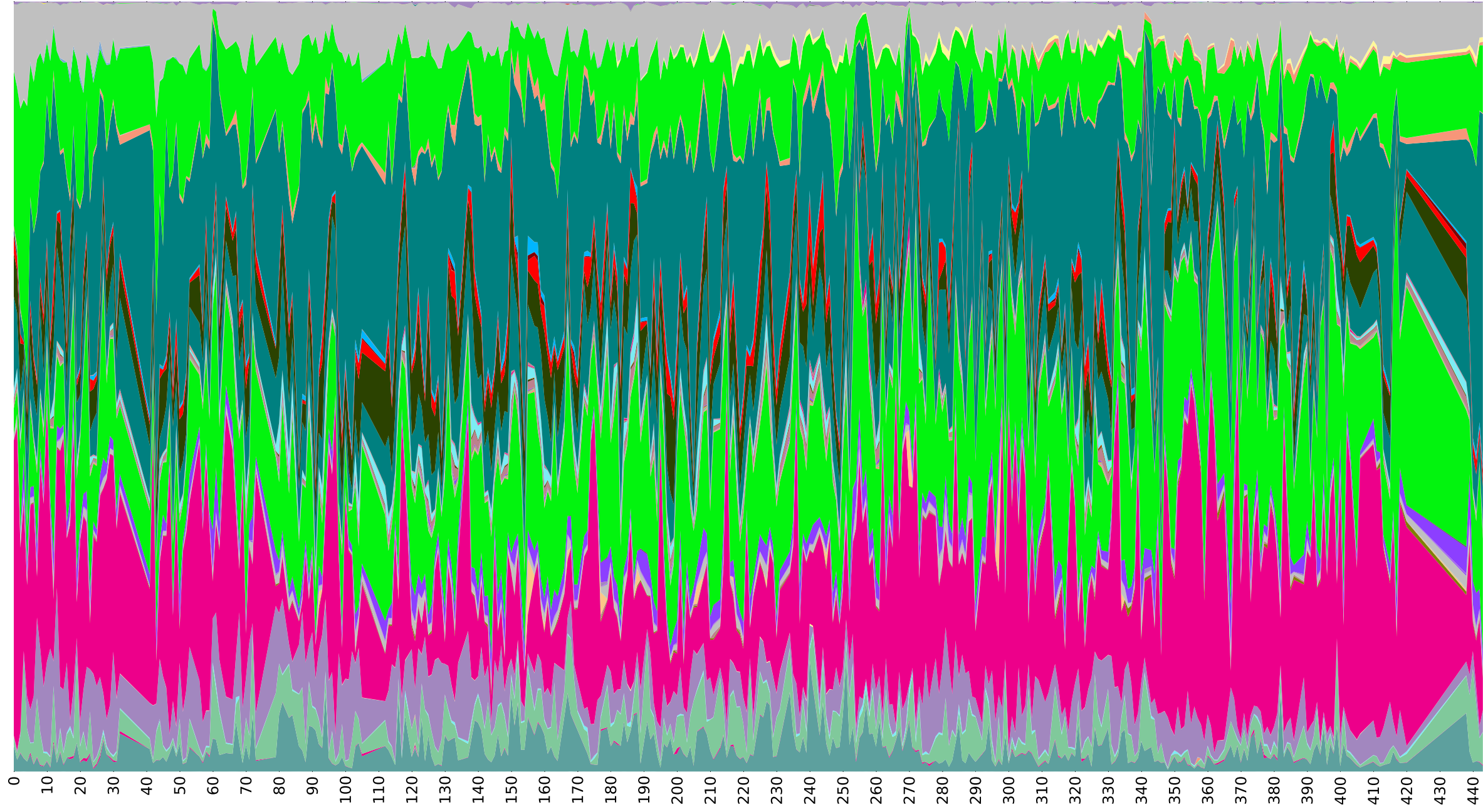

Supplement: Additional file 10 — Temporal variation in phylum, class, order, family, and genus abundances (M3 tongue). The x-axis scale differs between M3 and F4 plots. [file gb-2011-12-5-r50-S10.ZIP › AdditionalFile10/charts/9AhlZQWftKadaZ5eDwUnNrzPlTSGmN.pdf]

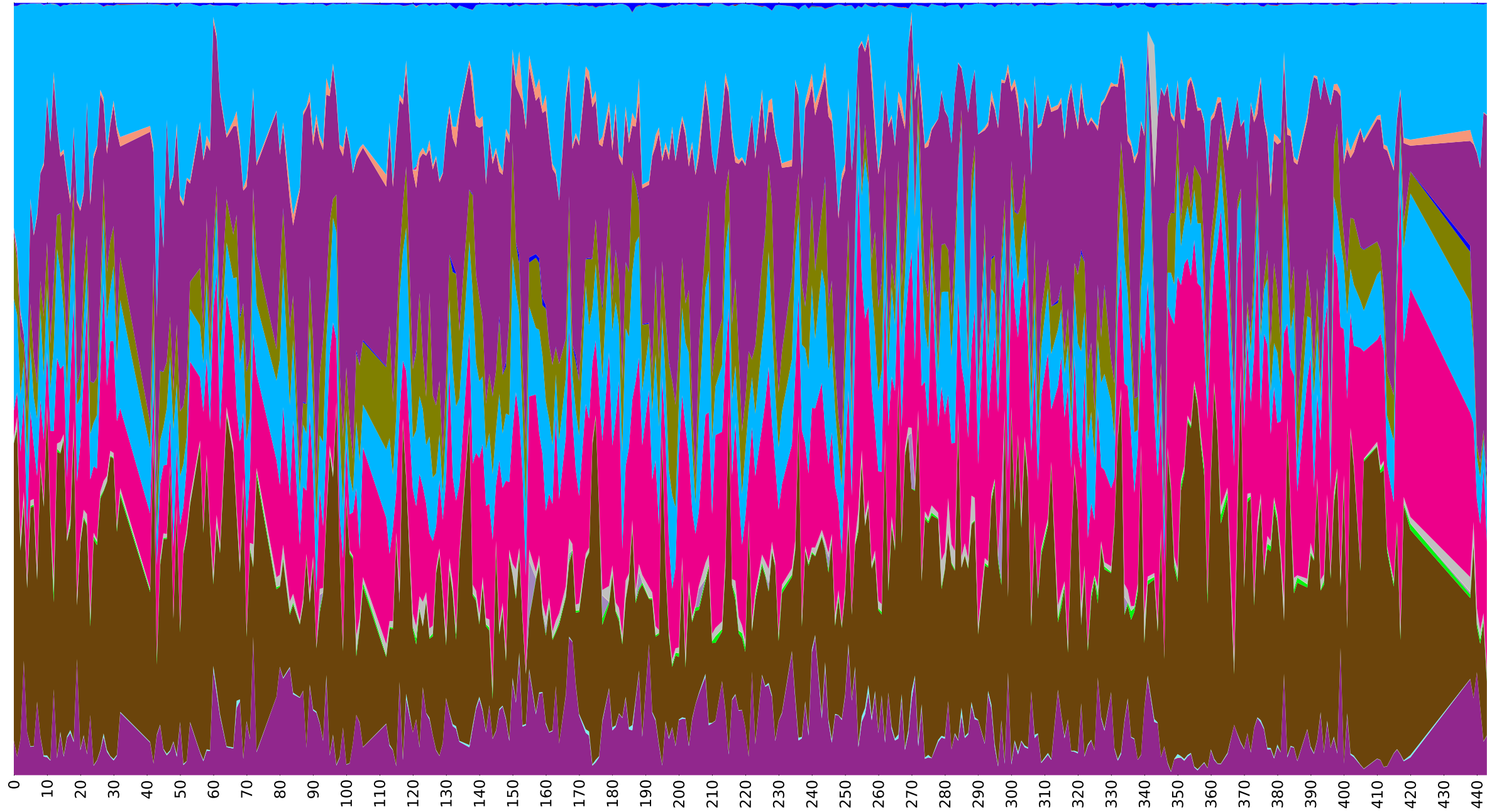

Supplement: Additional file 10 — Temporal variation in phylum, class, order, family, and genus abundances (M3 tongue). The x-axis scale differs between M3 and F4 plots. [file gb-2011-12-5-r50-S10.ZIP › AdditionalFile10/charts/BsrSJT0kSb6uJN63glbFe078J5oFLP.pdf]

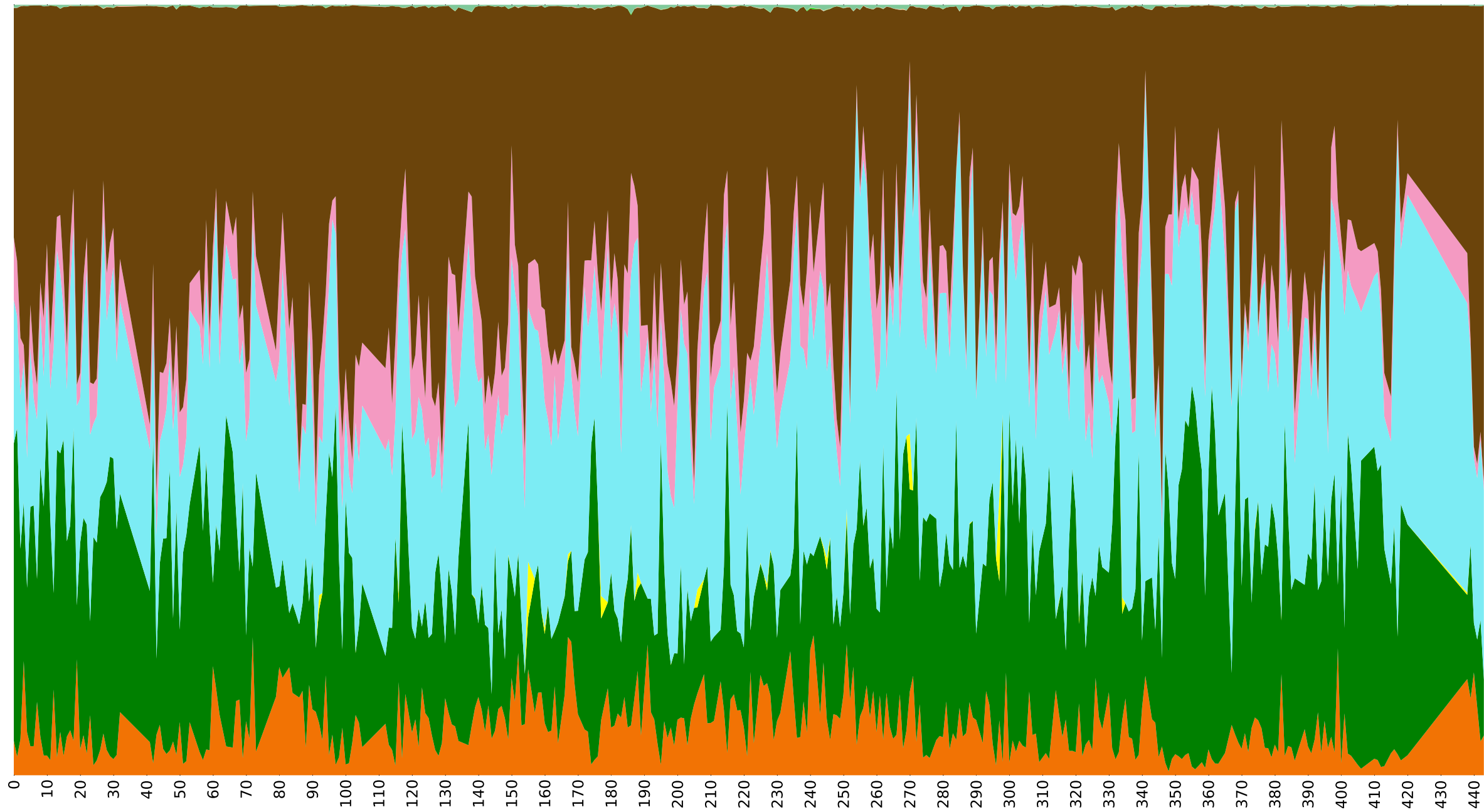

Supplement: Additional file 10 — Temporal variation in phylum, class, order, family, and genus abundances (M3 tongue). The x-axis scale differs between M3 and F4 plots. [file gb-2011-12-5-r50-S10.ZIP › AdditionalFile10/charts/iW3Too604lzisEczS3eqDkLSnFbRsC.pdf]

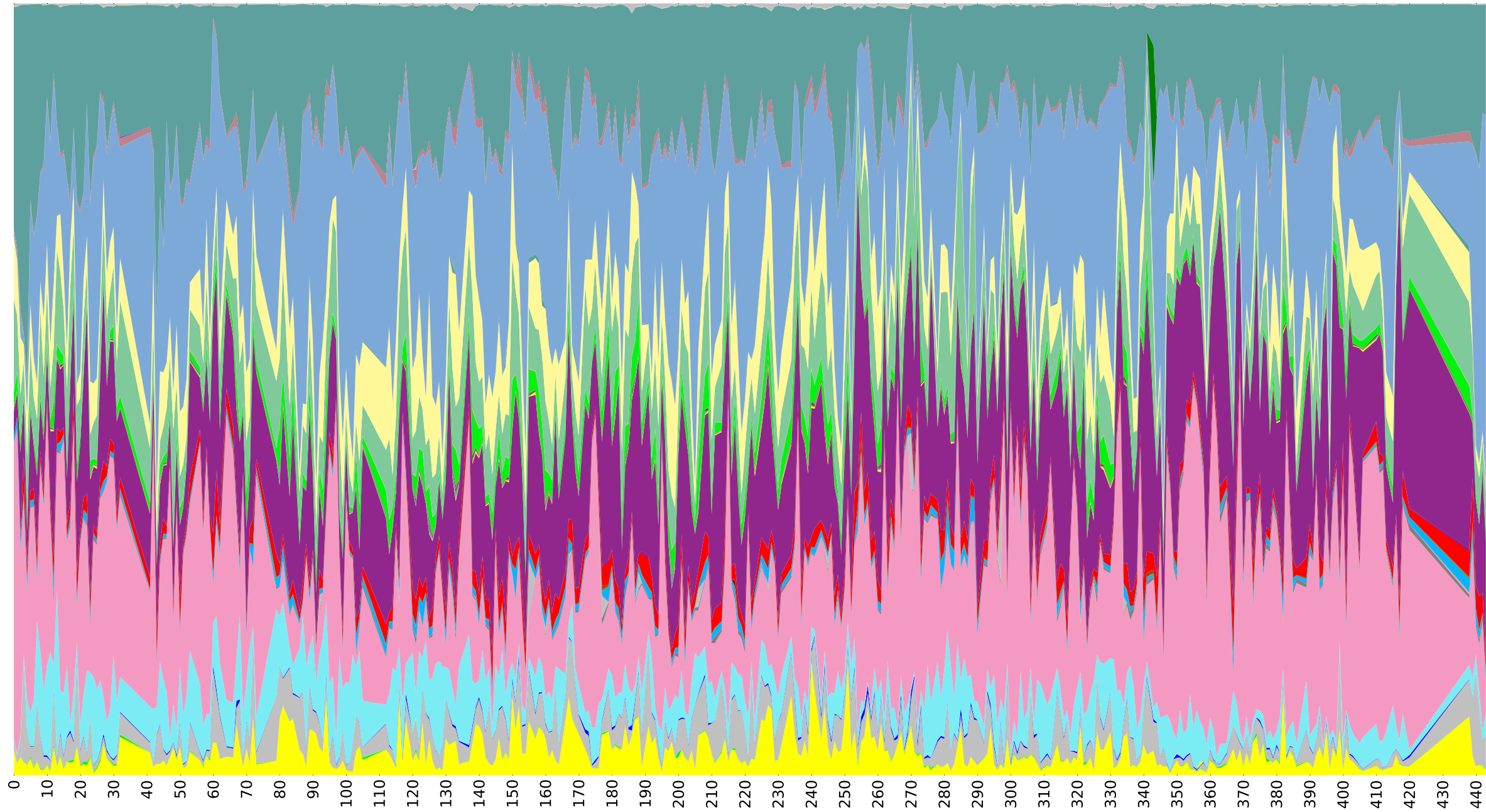

Supplement: Additional file 10 — Temporal variation in phylum, class, order, family, and genus abundances (M3 tongue). The x-axis scale differs between M3 and F4 plots. [file gb-2011-12-5-r50-S10.ZIP › AdditionalFile10/charts/kID4f4DzjNdi345pFXBF0fZaCBhaTe.pdf]

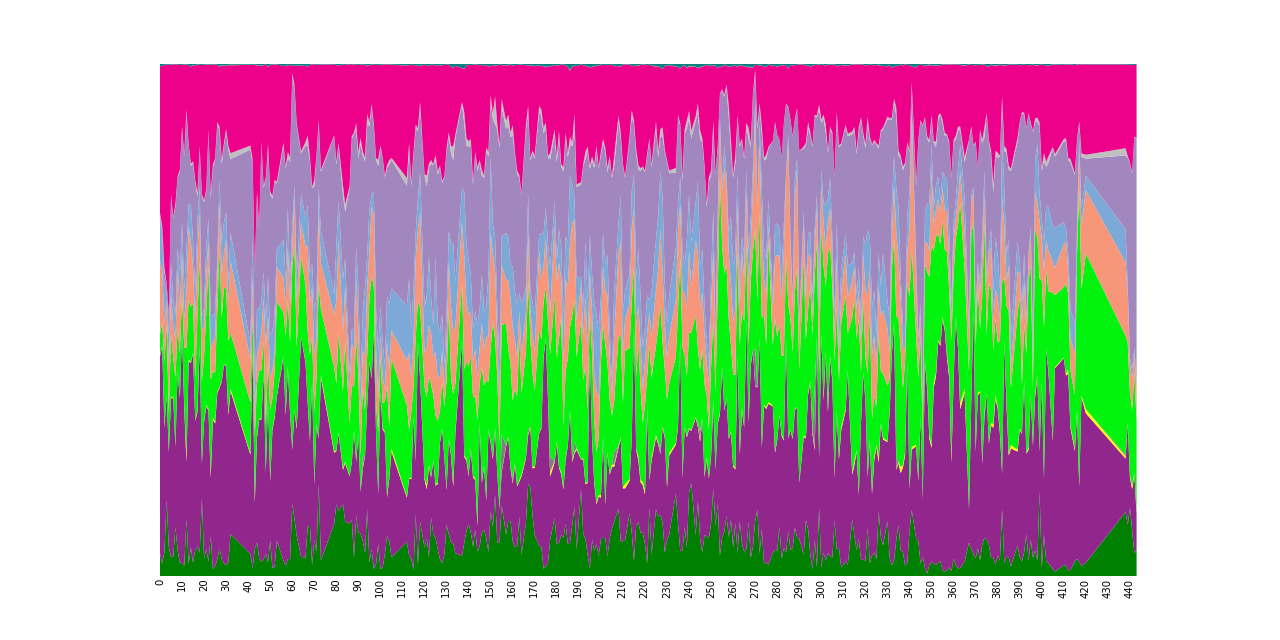

Supplement: Additional file 10 — Temporal variation in phylum, class, order, family, and genus abundances (M3 tongue). The x-axis scale differs between M3 and F4 plots. [file gb-2011-12-5-r50-S10.ZIP › AdditionalFile10/charts/lks4ZN7EWX0zoO6AZTXI3iw5pPdWK0.png]

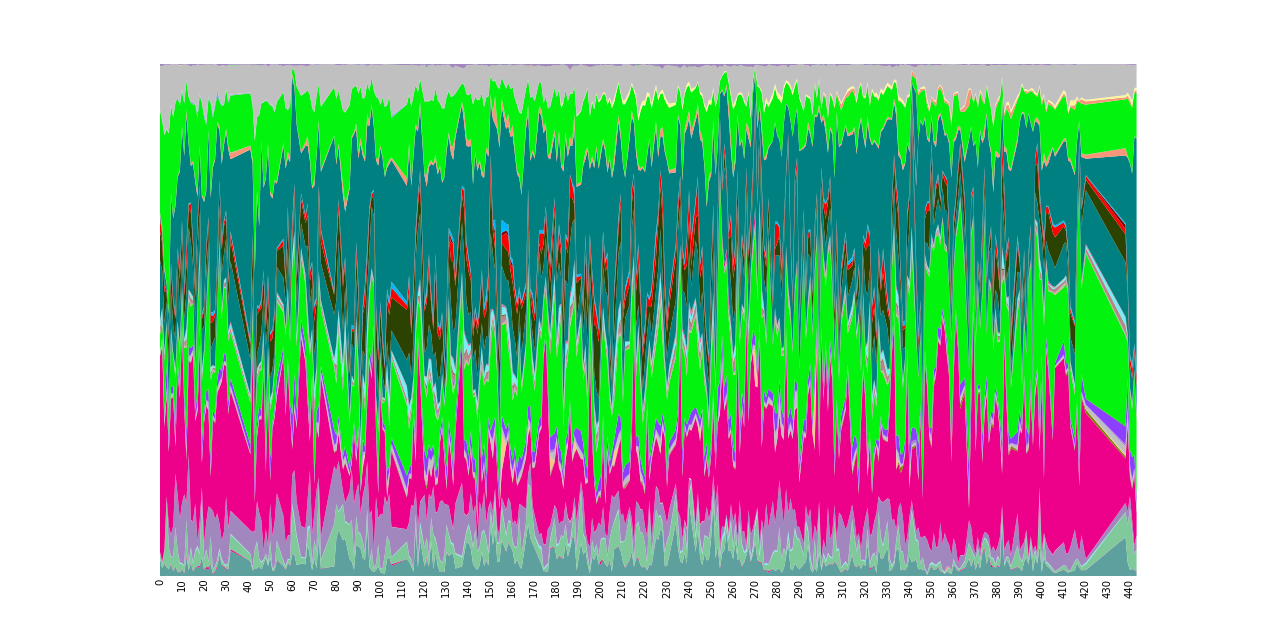

Supplement: Additional file 10 — Temporal variation in phylum, class, order, family, and genus abundances (M3 tongue). The x-axis scale differs between M3 and F4 plots. [file gb-2011-12-5-r50-S10.ZIP › AdditionalFile10/charts/r0WIwPPyAzzprdIhzYLWpZZRemXdg0.png]

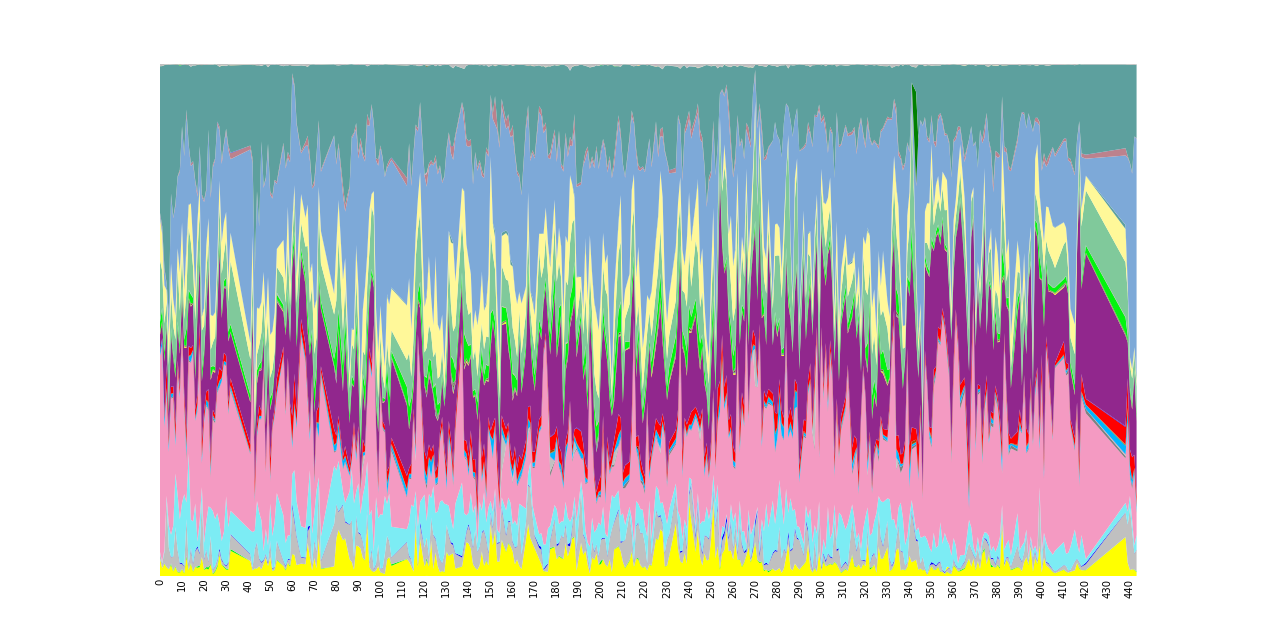

Supplement: Additional file 10 — Temporal variation in phylum, class, order, family, and genus abundances (M3 tongue). The x-axis scale differs between M3 and F4 plots. [file gb-2011-12-5-r50-S10.ZIP › AdditionalFile10/charts/SSSDusQWqRfnLsaBcsJEEssiBEZCDR.png]

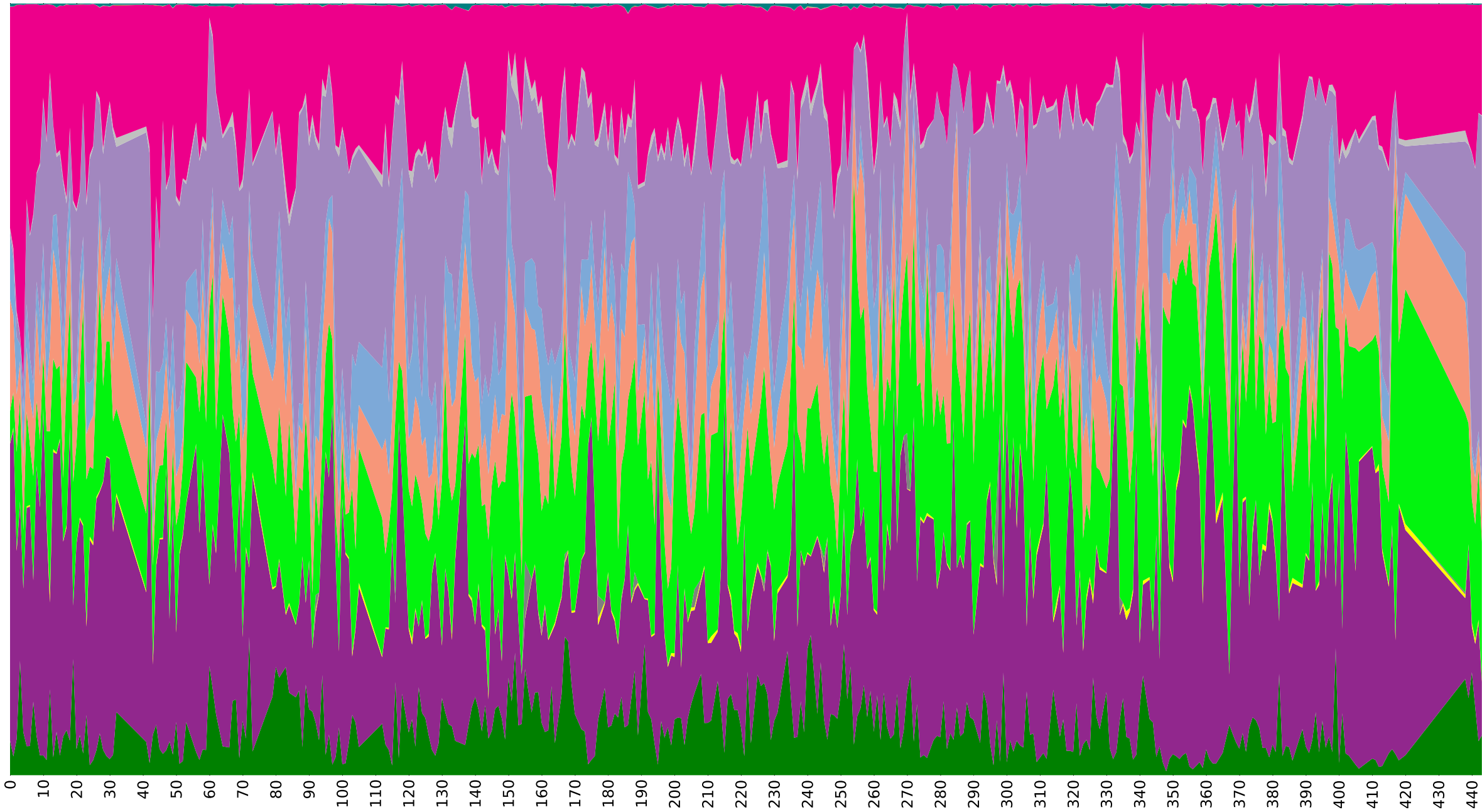

Supplement: Additional file 10 — Temporal variation in phylum, class, order, family, and genus abundances (M3 tongue). The x-axis scale differs between M3 and F4 plots. [file gb-2011-12-5-r50-S10.ZIP › AdditionalFile10/charts/zEyAPA0zib5WhaRqdHslP4JWzLMNzB.pdf]

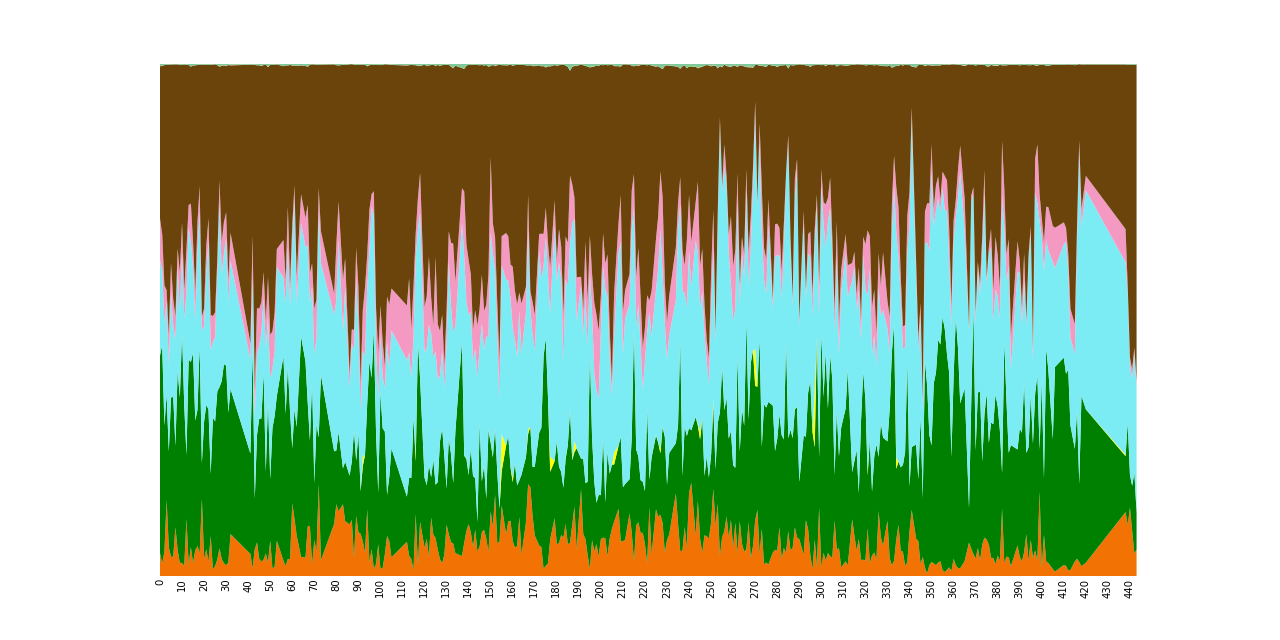

Supplement: Additional file 10 — Temporal variation in phylum, class, order, family, and genus abundances (M3 tongue). The x-axis scale differs between M3 and F4 plots. [file gb-2011-12-5-r50-S10.ZIP › AdditionalFile10/charts/ZffILFGCqKytuY01QyGF5SZwzQ626H.png]

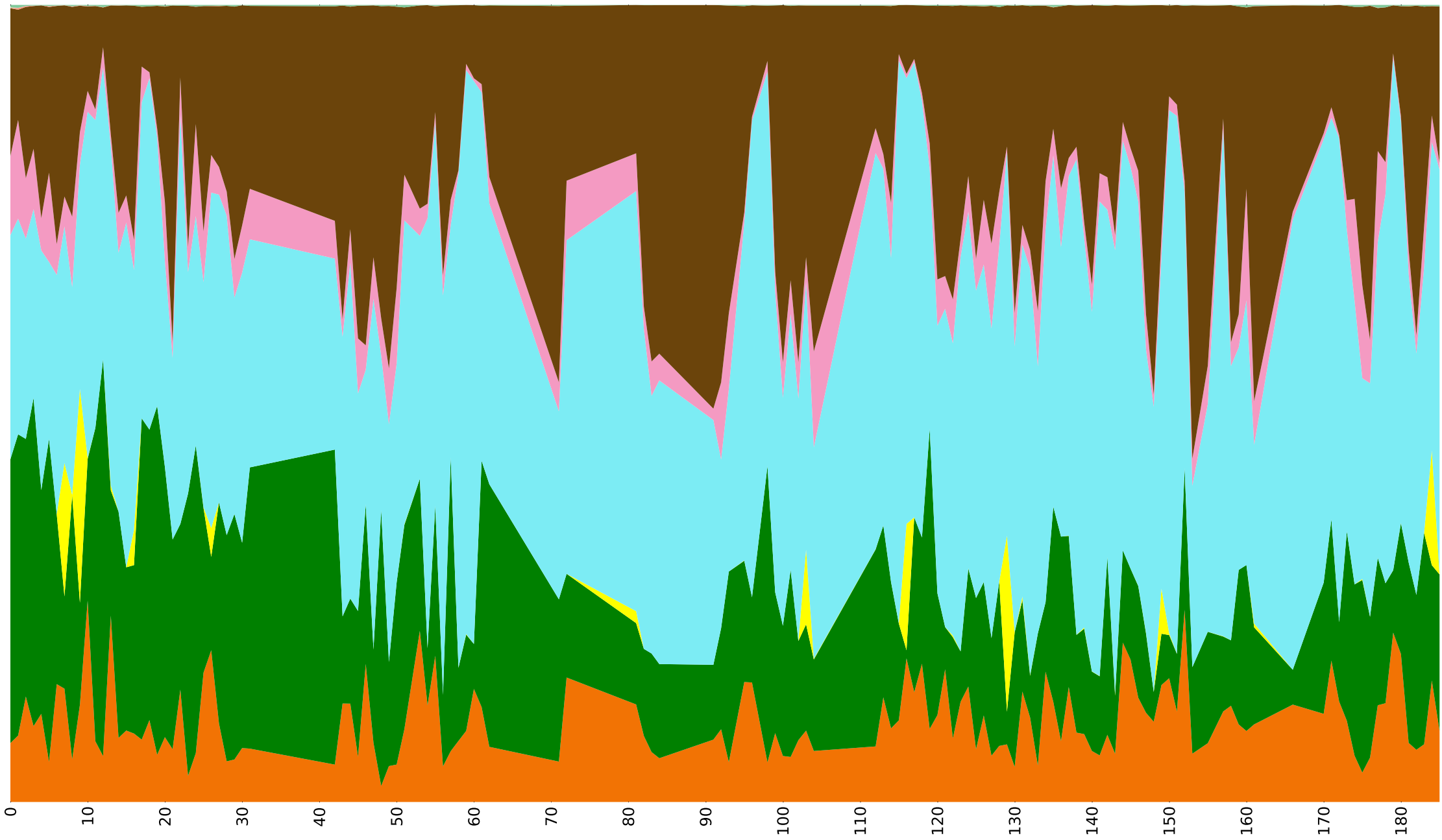

Supplement: Additional file 11 — Temporal variation in phylum, class, order, family, and genus abundances (F4 tongue). The x-axis scale differs between M3 and F4 plots. [file gb-2011-12-5-r50-S11.ZIP › AdditionalFile11/charts/7zPoprUfZe0mF6IJolUPT0Jlz4XagC.pdf]

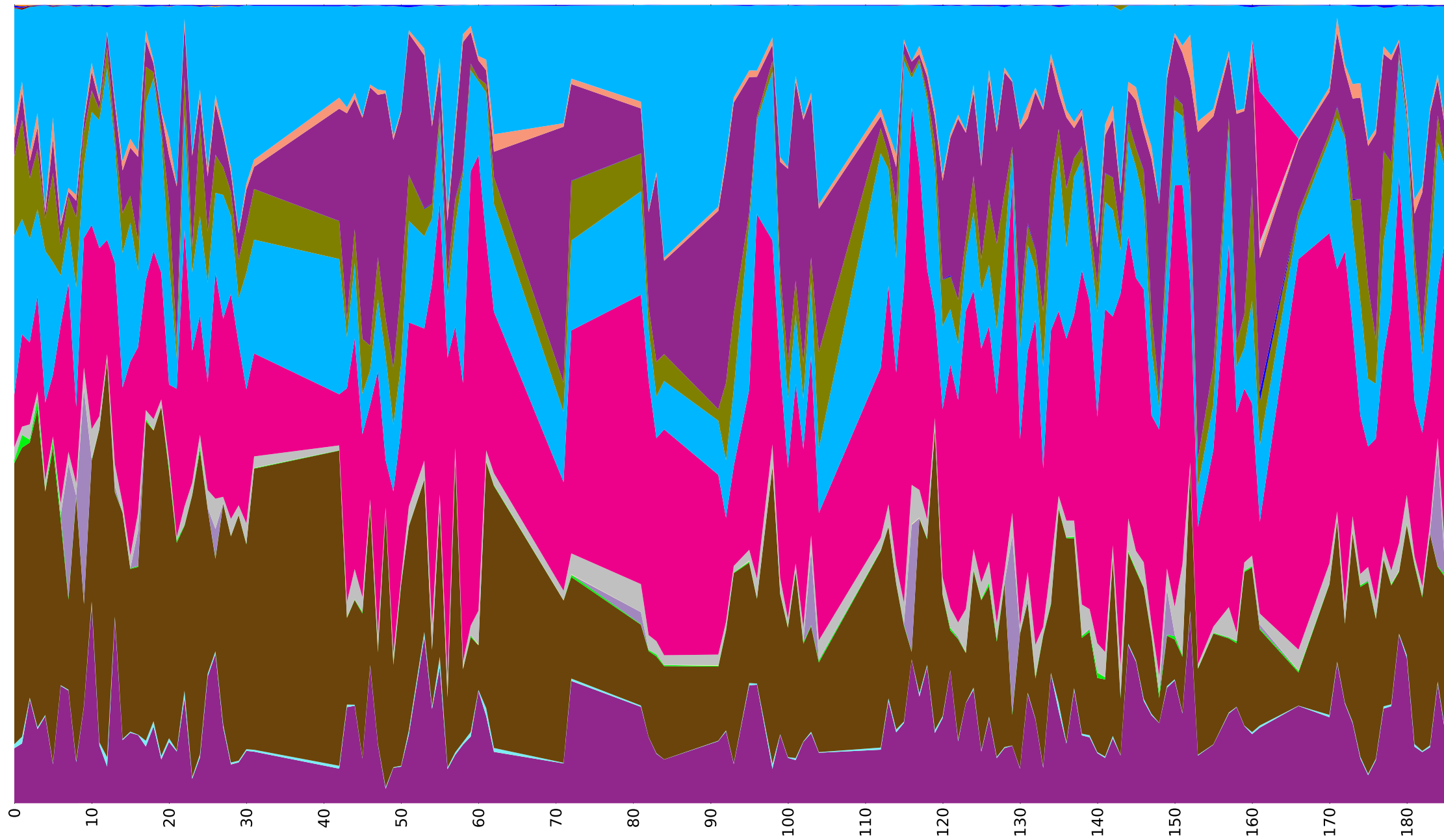

Supplement: Additional file 11 — Temporal variation in phylum, class, order, family, and genus abundances (F4 tongue). The x-axis scale differs between M3 and F4 plots. [file gb-2011-12-5-r50-S11.ZIP › AdditionalFile11/charts/EI5FMDibrYSZtlLIJfcQD4HyBDmNb7.pdf]

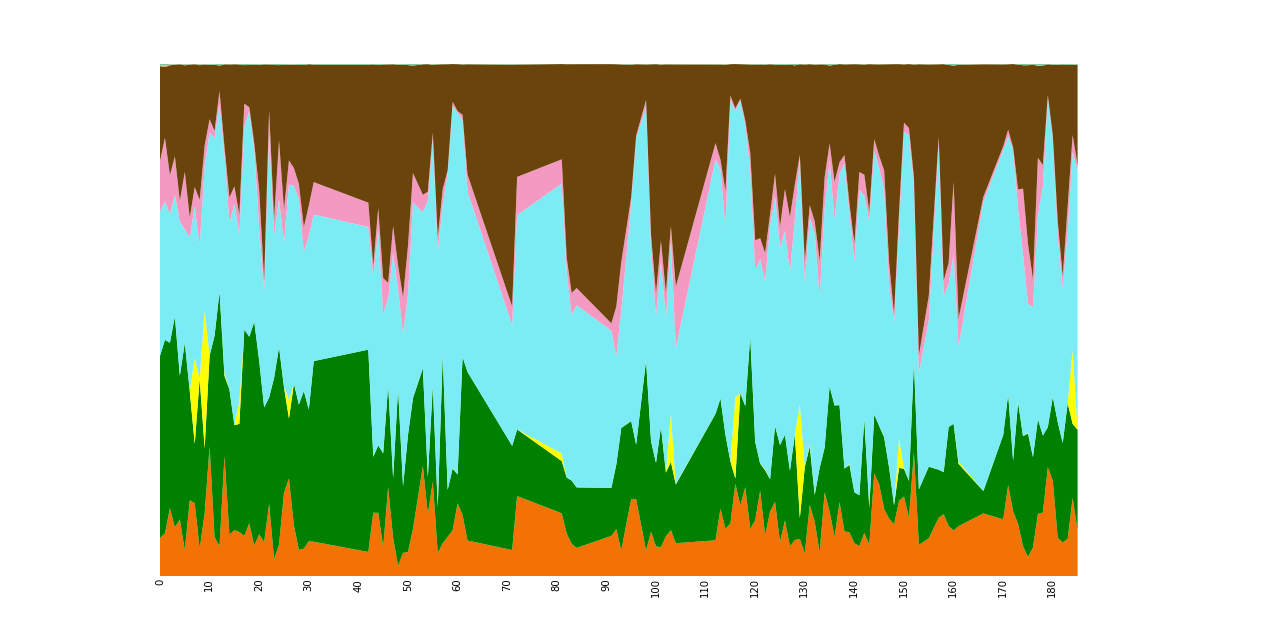

Supplement: Additional file 11 — Temporal variation in phylum, class, order, family, and genus abundances (F4 tongue). The x-axis scale differs between M3 and F4 plots. [file gb-2011-12-5-r50-S11.ZIP › AdditionalFile11/charts/FB9fzByI0aBpGclZXp34M4l6rCzK5Y.png]

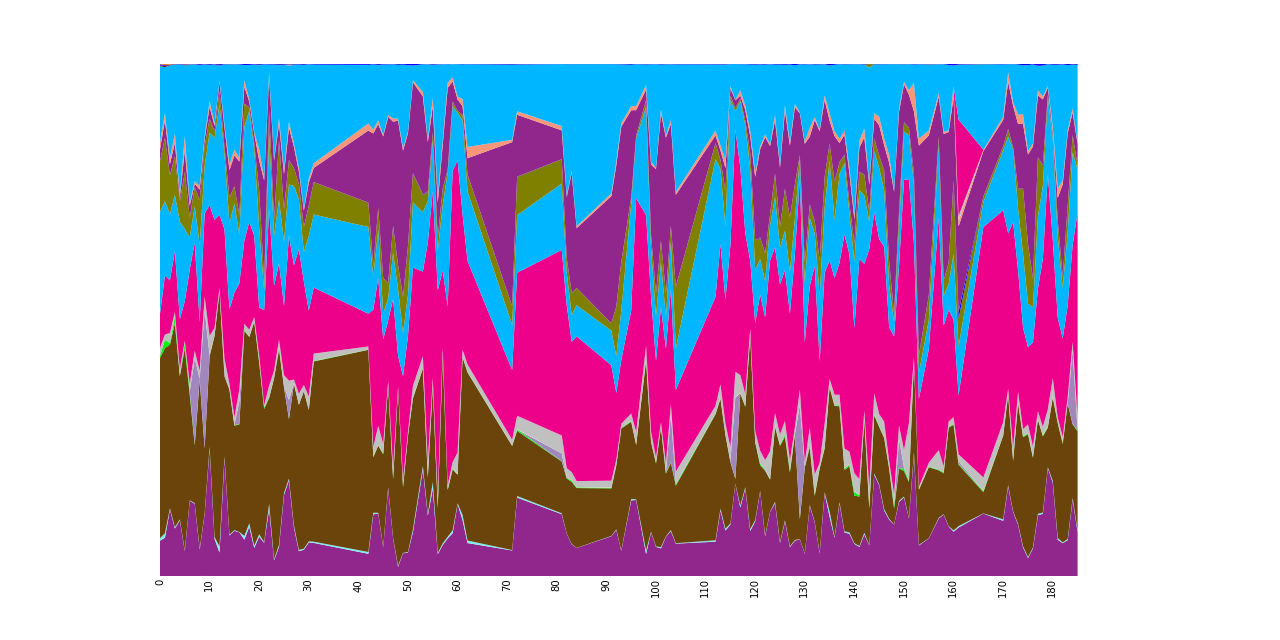

Supplement: Additional file 11 — Temporal variation in phylum, class, order, family, and genus abundances (F4 tongue). The x-axis scale differs between M3 and F4 plots. [file gb-2011-12-5-r50-S11.ZIP › AdditionalFile11/charts/fTsbxygbpEMYOfYgzqPynStCIUzrzq.png]

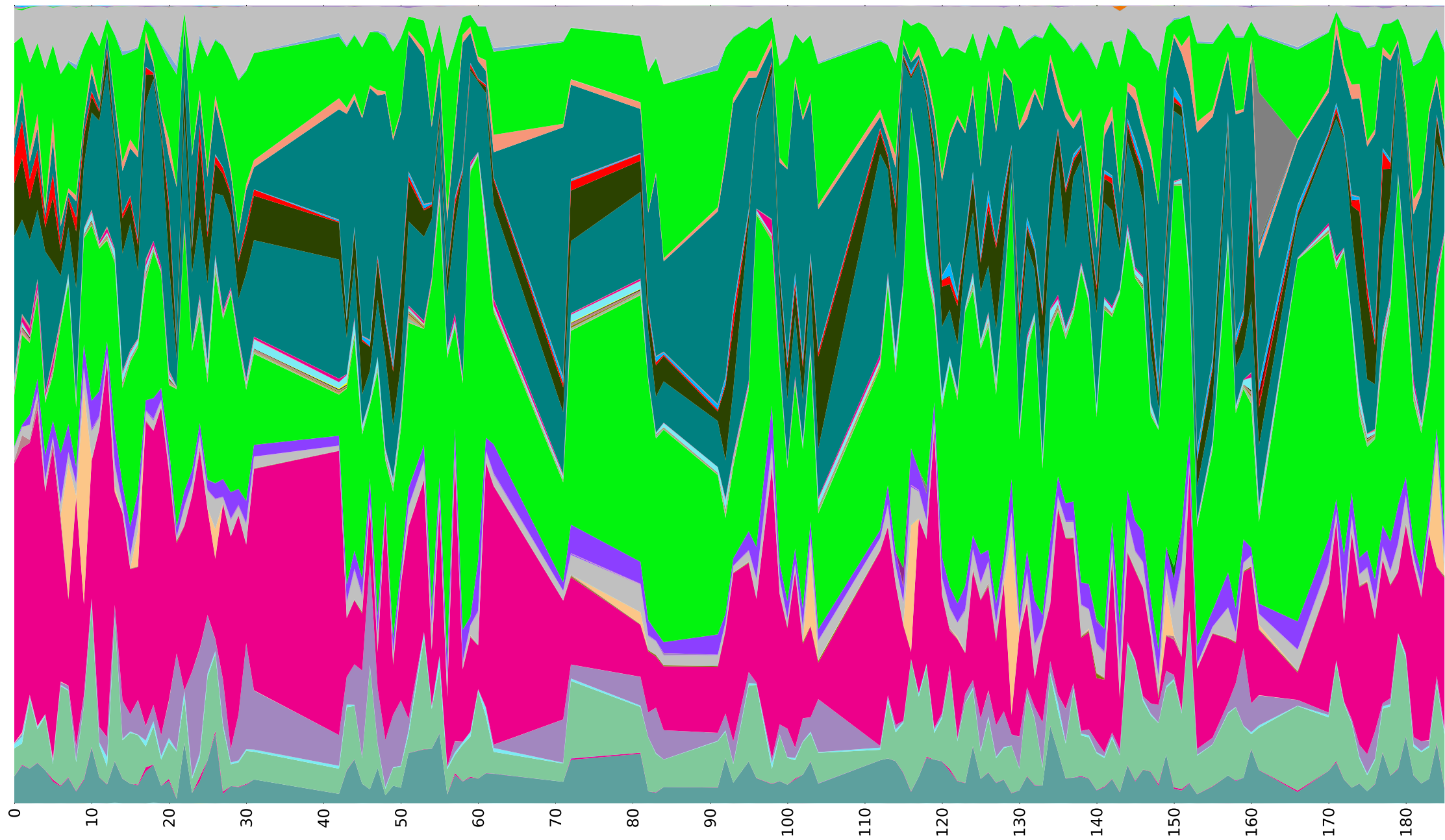

Supplement: Additional file 11 — Temporal variation in phylum, class, order, family, and genus abundances (F4 tongue). The x-axis scale differs between M3 and F4 plots. [file gb-2011-12-5-r50-S11.ZIP › AdditionalFile11/charts/ibiFNc8YM4mDx7cDA41EqPZildjzhj.pdf]

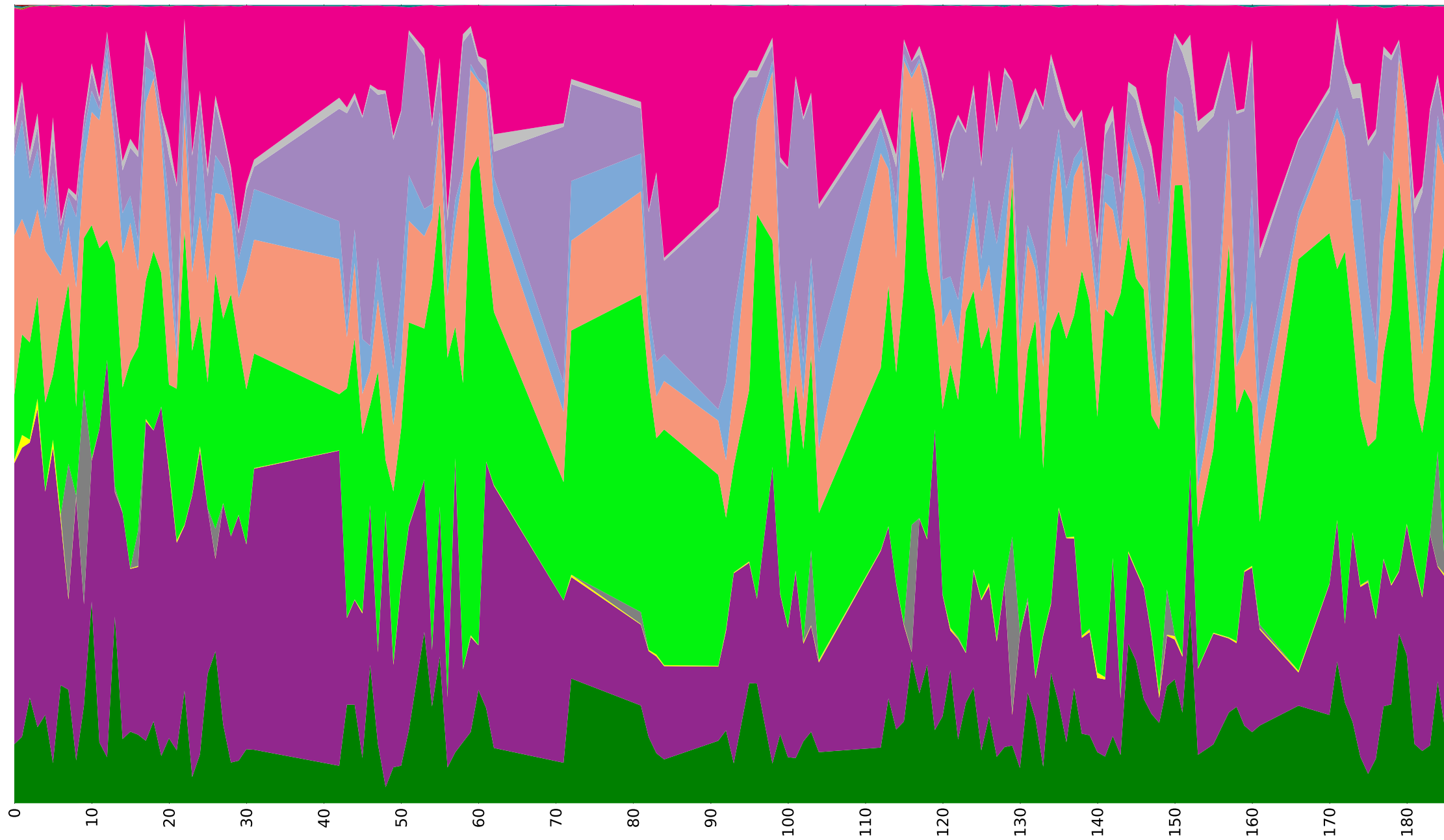

Supplement: Additional file 11 — Temporal variation in phylum, class, order, family, and genus abundances (F4 tongue). The x-axis scale differs between M3 and F4 plots. [file gb-2011-12-5-r50-S11.ZIP › AdditionalFile11/charts/JuIQnObWwZWr0GzNlLARtrP141Y9w9.pdf]

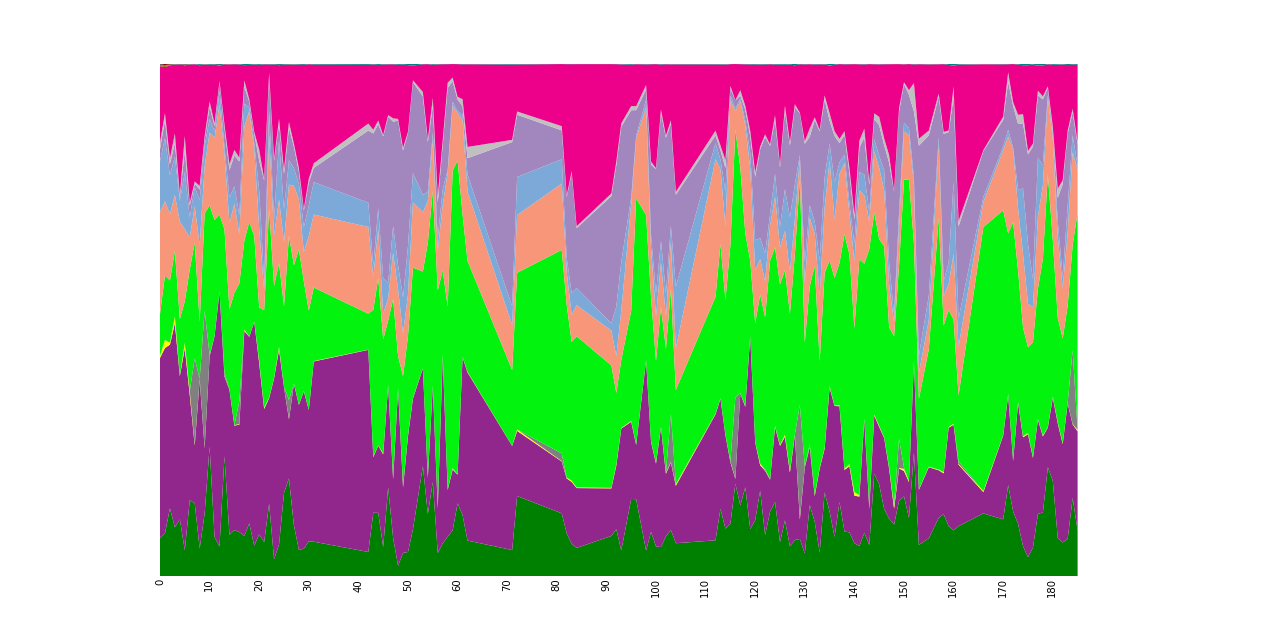

Supplement: Additional file 11 — Temporal variation in phylum, class, order, family, and genus abundances (F4 tongue). The x-axis scale differs between M3 and F4 plots. [file gb-2011-12-5-r50-S11.ZIP › AdditionalFile11/charts/kjFMnchF0EDBq9ZT4aCy20ORb7FdyL.png]

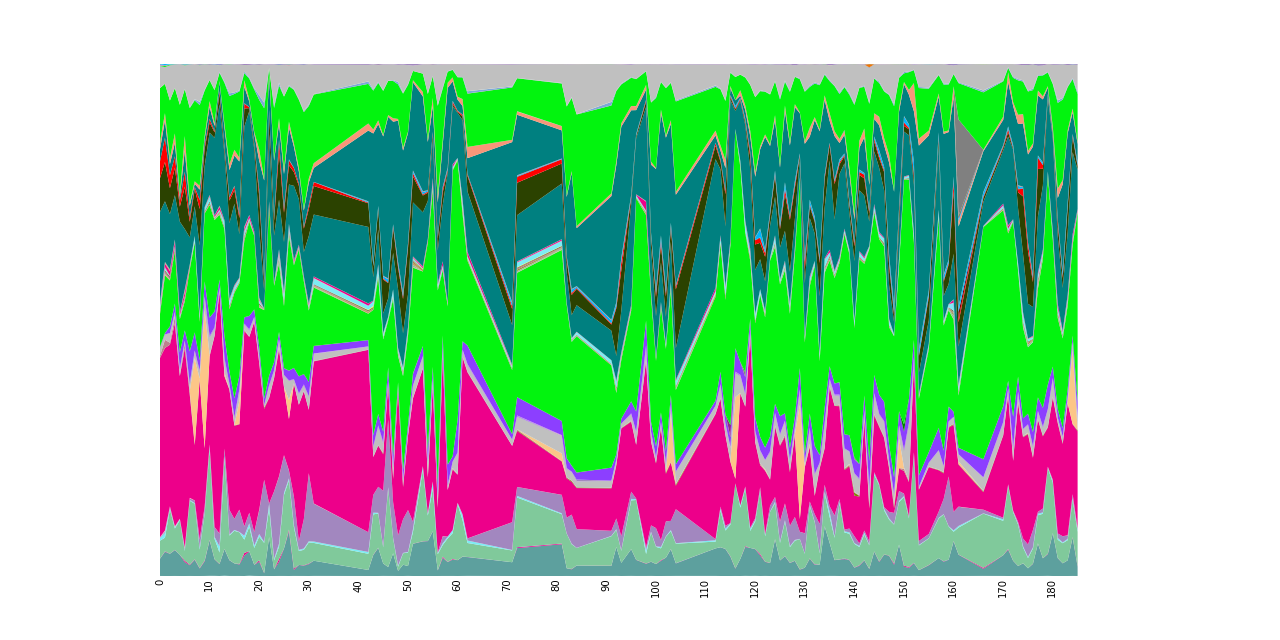

Supplement: Additional file 11 — Temporal variation in phylum, class, order, family, and genus abundances (F4 tongue). The x-axis scale differs between M3 and F4 plots. [file gb-2011-12-5-r50-S11.ZIP › AdditionalFile11/charts/ppHt6Jb6PoLnDmJTQSZSQikz8S05WR.png]

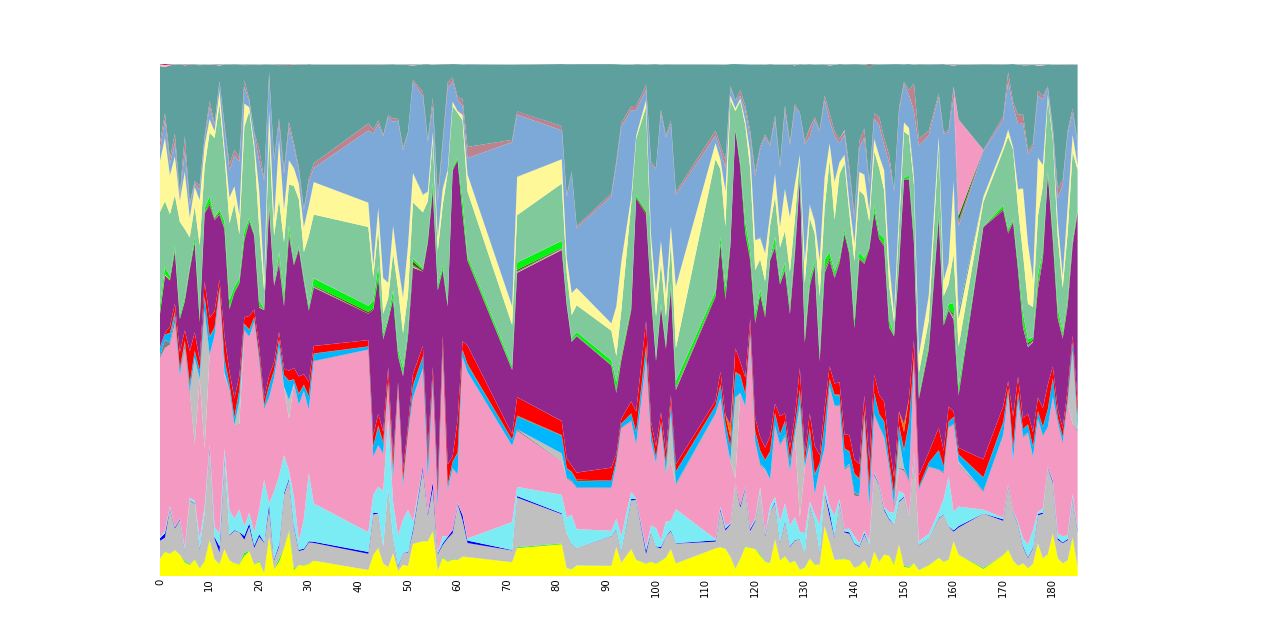

Supplement: Additional file 11 — Temporal variation in phylum, class, order, family, and genus abundances (F4 tongue). The x-axis scale differs between M3 and F4 plots. [file gb-2011-12-5-r50-S11.ZIP › AdditionalFile11/charts/SFzEguMay03YzLqbctzdkPBRQGn0gC.png]

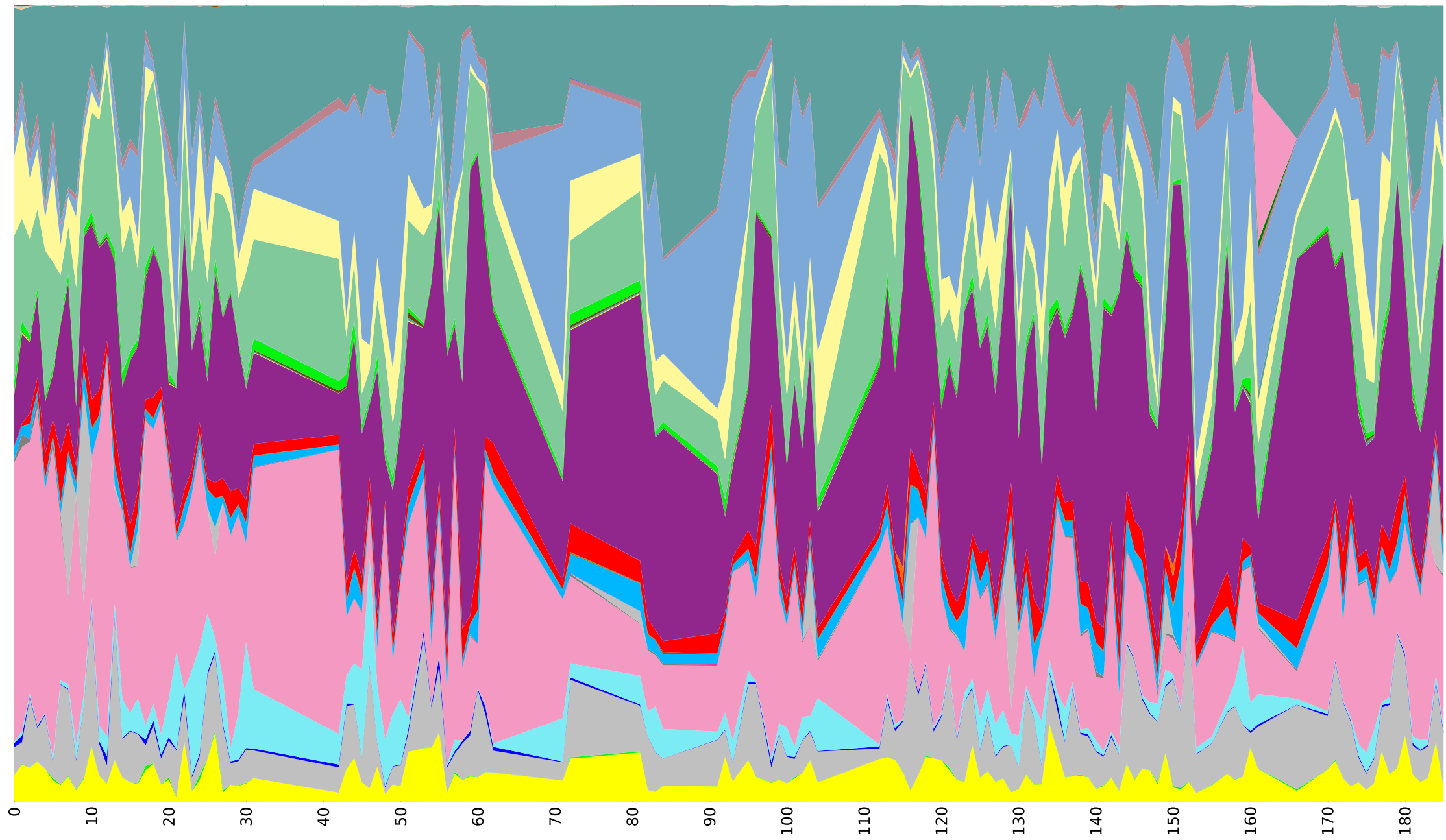

Supplement: Additional file 11 — Temporal variation in phylum, class, order, family, and genus abundances (F4 tongue). The x-axis scale differs between M3 and F4 plots. [file gb-2011-12-5-r50-S11.ZIP › AdditionalFile11/charts/zPAslt6uQiaqxdsDSbYSTbB0AMoRRG.pdf]

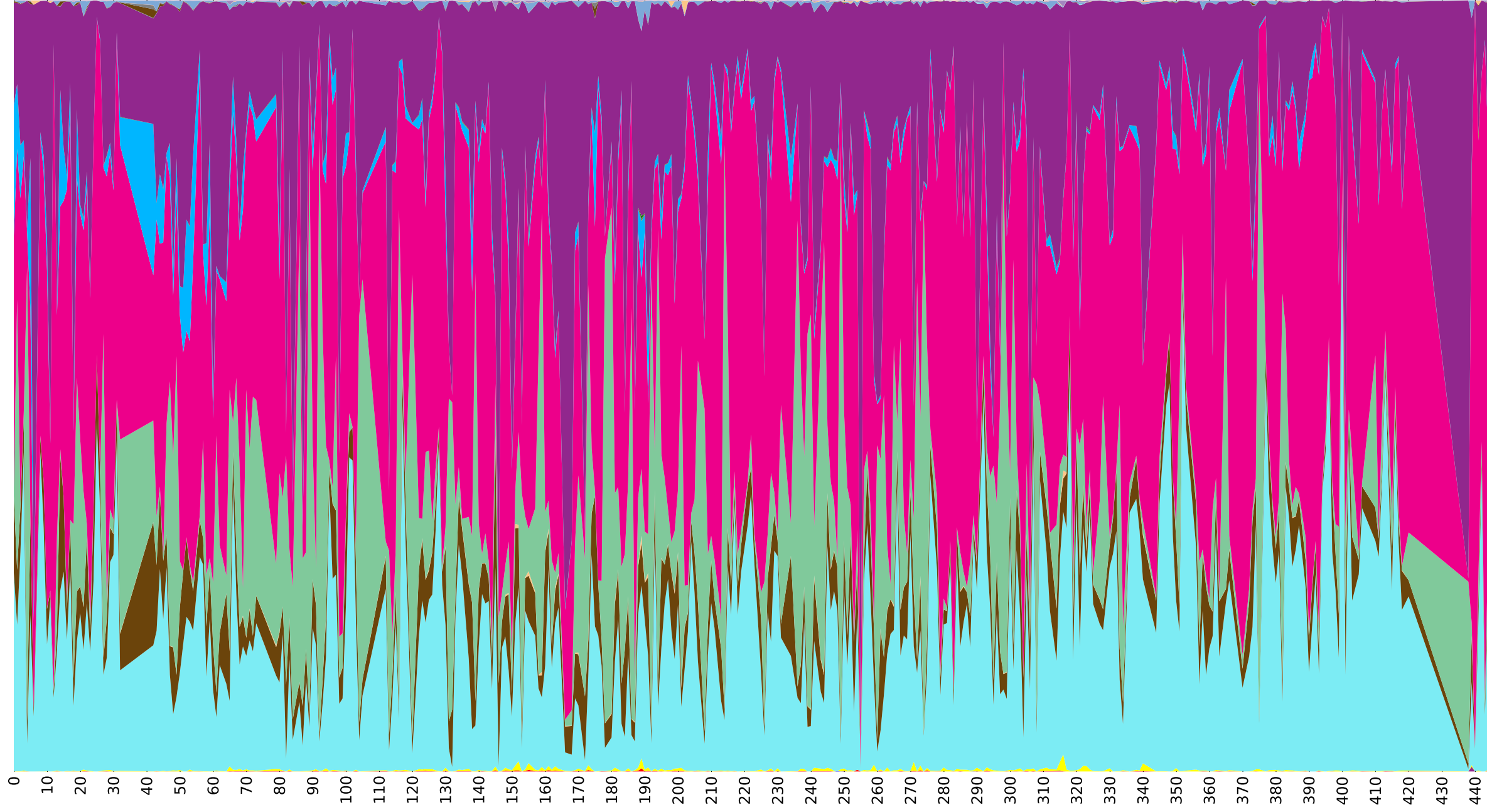

Supplement: Additional file 12 — Temporal variation in phylum, class, order, family, and genus abundances (M3 left palm). The x-axis scale differs between M3 and F4 plots. [file gb-2011-12-5-r50-S12.ZIP › AdditionalFile12/charts/3w4edUmTSPYcR3mReDWeb8ZZIKzdUJ.pdf]

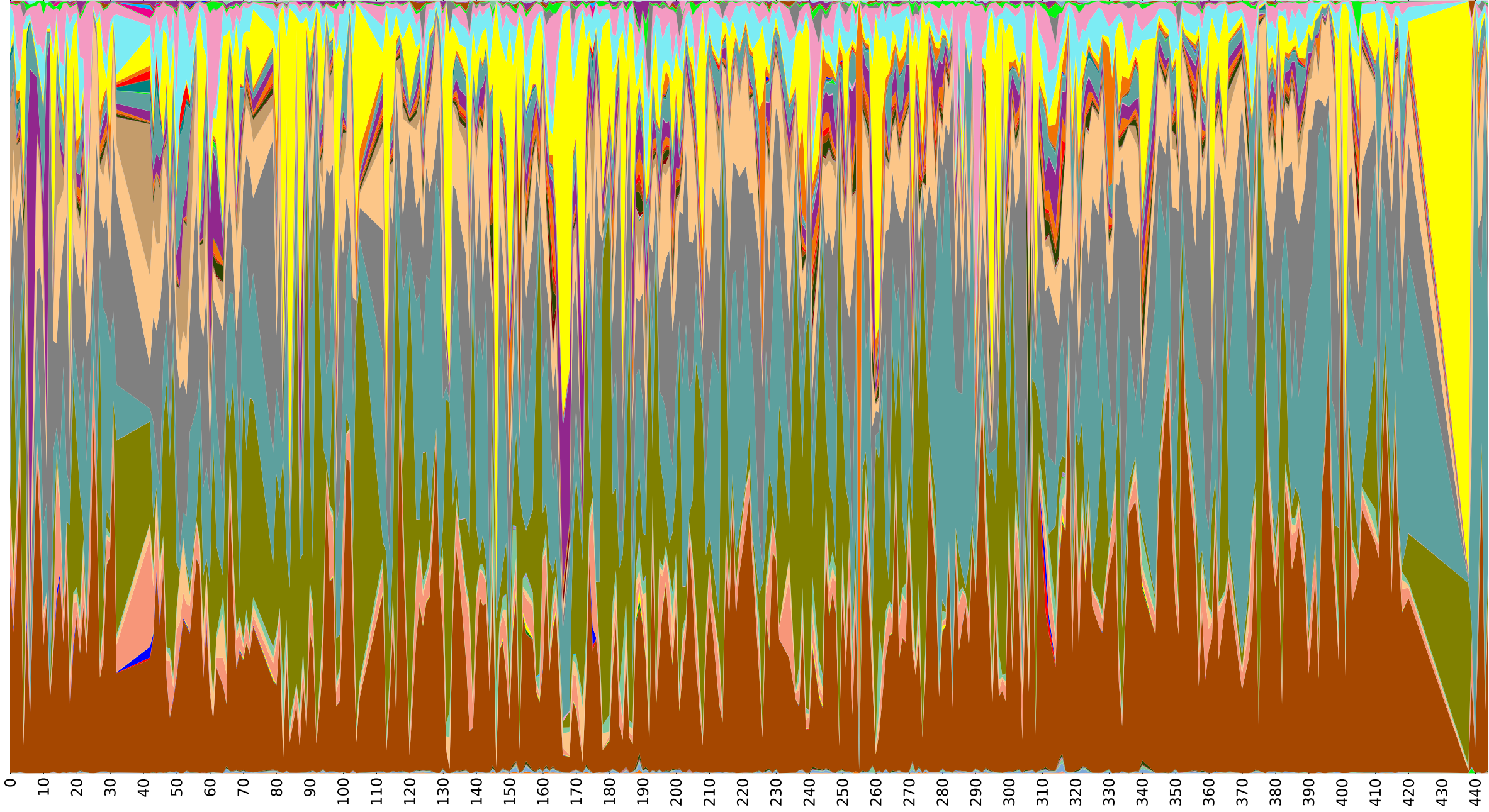

Supplement: Additional file 12 — Temporal variation in phylum, class, order, family, and genus abundances (M3 left palm). The x-axis scale differs between M3 and F4 plots. [file gb-2011-12-5-r50-S12.ZIP › AdditionalFile12/charts/4euUjnC7pNW4k63yTy5hmDxK6UJ0EZ.pdf]

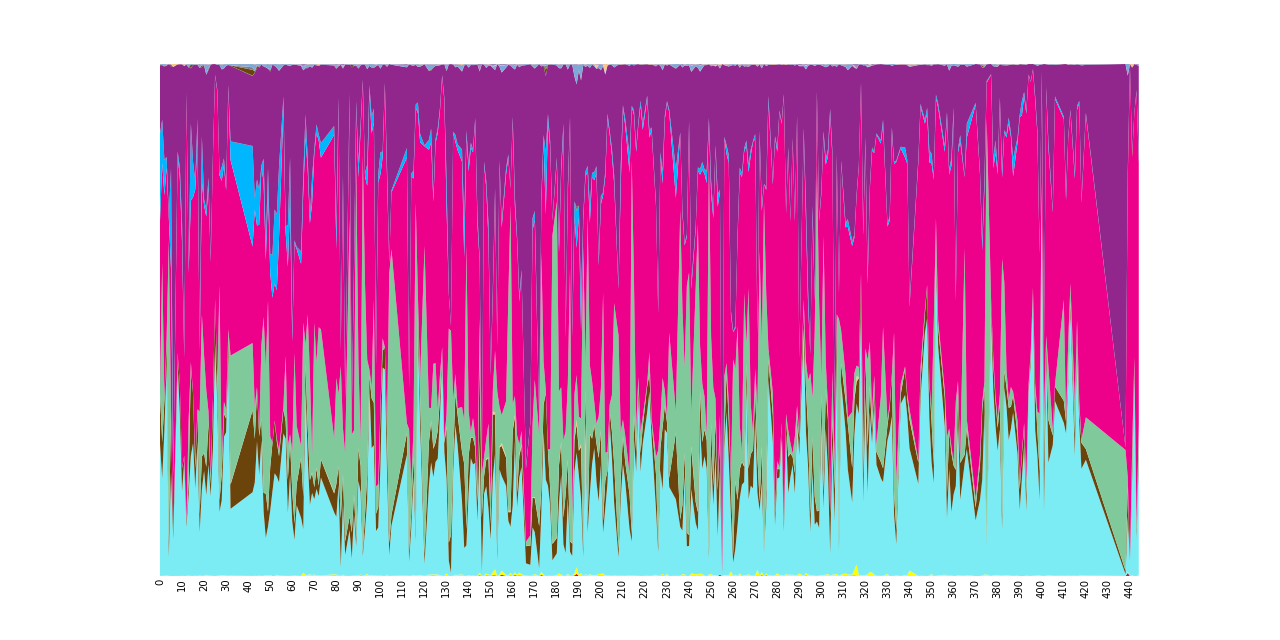

Supplement: Additional file 12 — Temporal variation in phylum, class, order, family, and genus abundances (M3 left palm). The x-axis scale differs between M3 and F4 plots. [file gb-2011-12-5-r50-S12.ZIP › AdditionalFile12/charts/BZaoA2WeZnZz70S5nSffAgepNt65DN.png]

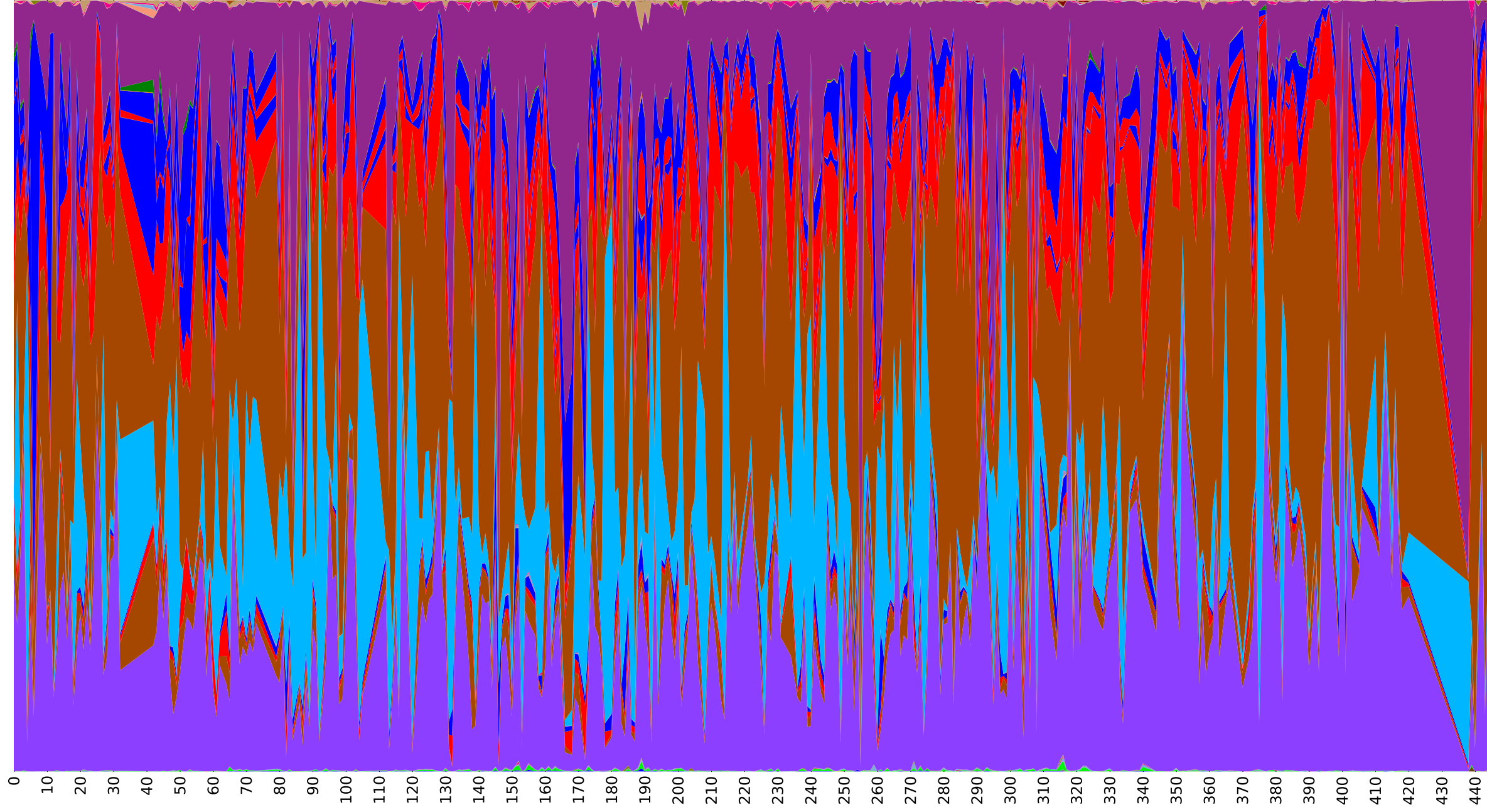

Supplement: Additional file 12 — Temporal variation in phylum, class, order, family, and genus abundances (M3 left palm). The x-axis scale differs between M3 and F4 plots. [file gb-2011-12-5-r50-S12.ZIP › AdditionalFile12/charts/N0sm7Su1Q7wAZDhIq4zTqadaICKI3O.pdf]

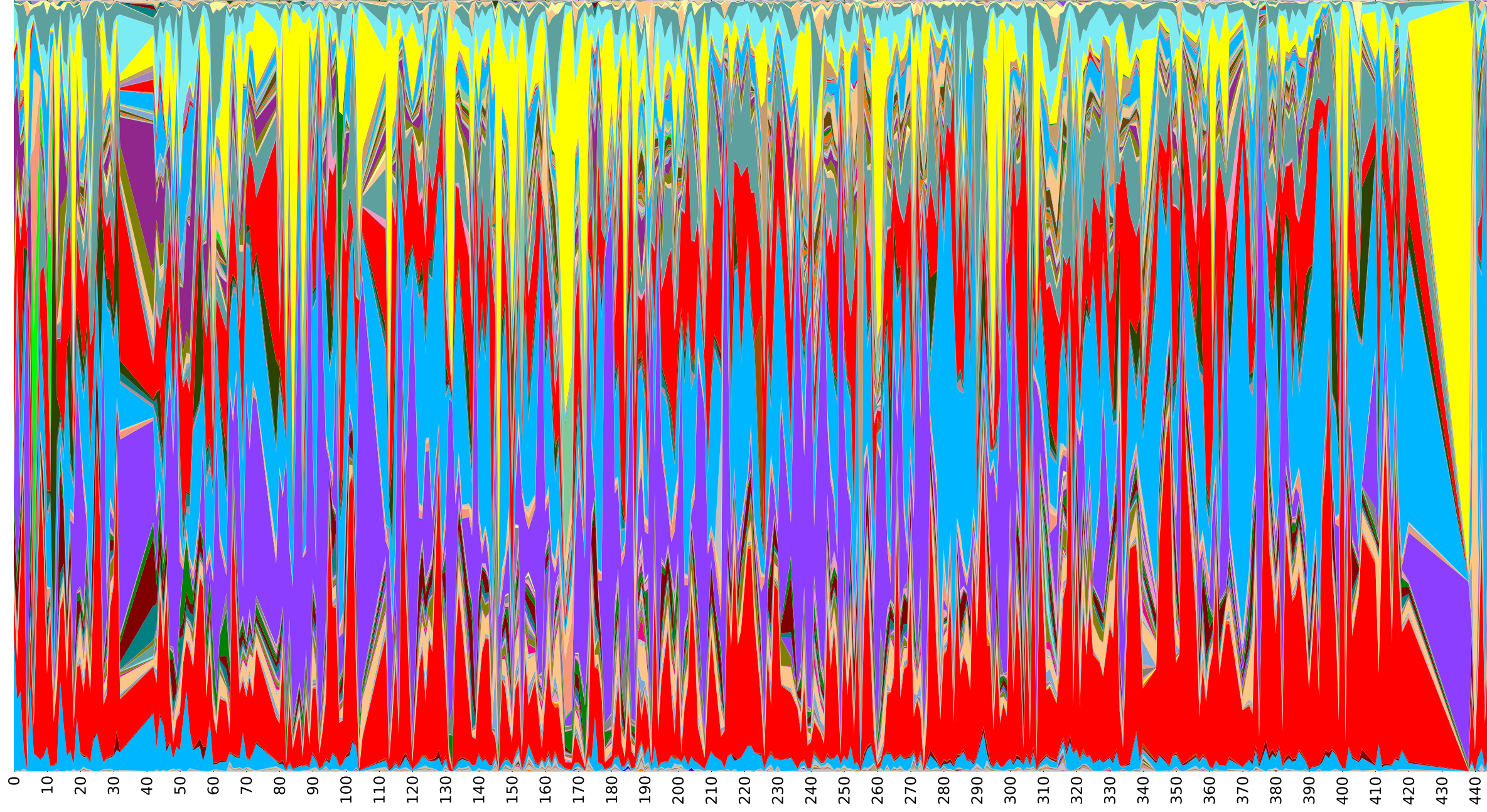

Supplement: Additional file 12 — Temporal variation in phylum, class, order, family, and genus abundances (M3 left palm). The x-axis scale differs between M3 and F4 plots. [file gb-2011-12-5-r50-S12.ZIP › AdditionalFile12/charts/PJTSJj8DtSRYMitSseIj9jMe5Ynu35.pdf]

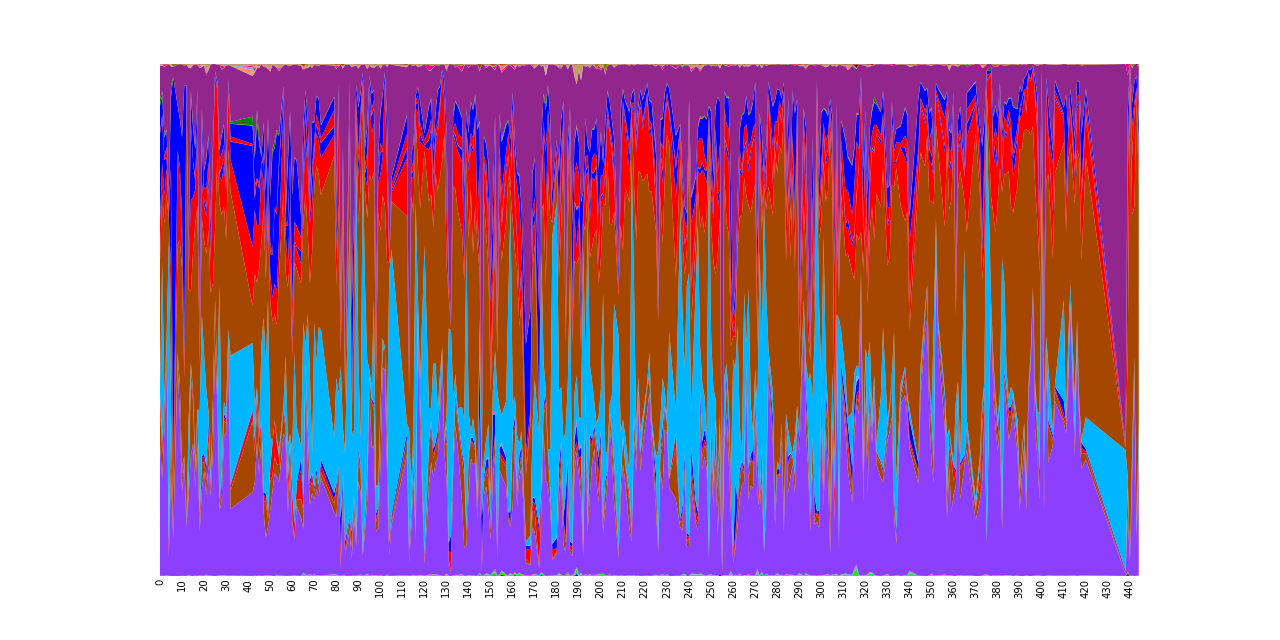

Supplement: Additional file 12 — Temporal variation in phylum, class, order, family, and genus abundances (M3 left palm). The x-axis scale differs between M3 and F4 plots. [file gb-2011-12-5-r50-S12.ZIP › AdditionalFile12/charts/RBYbJ0dHI0jnY8GAaYipSfYh9yz6ZU.png]

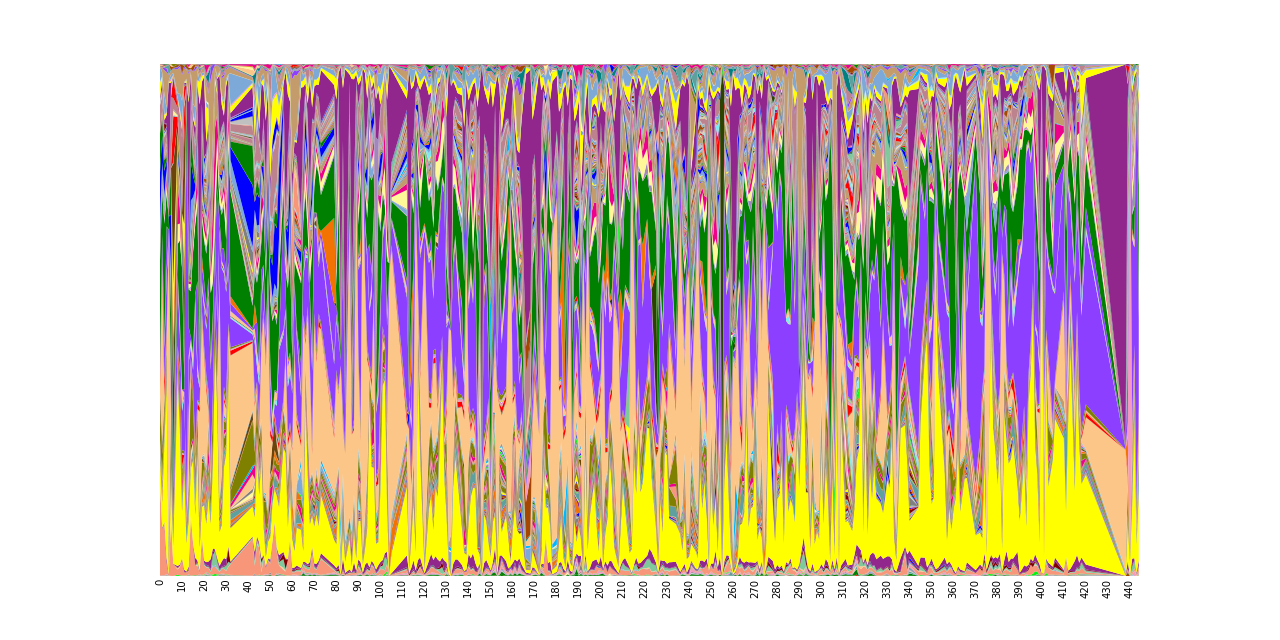

Supplement: Additional file 12 — Temporal variation in phylum, class, order, family, and genus abundances (M3 left palm). The x-axis scale differs between M3 and F4 plots. [file gb-2011-12-5-r50-S12.ZIP › AdditionalFile12/charts/sbW9PnWbpKRmPAiGMByLLn4RljykpD.png]

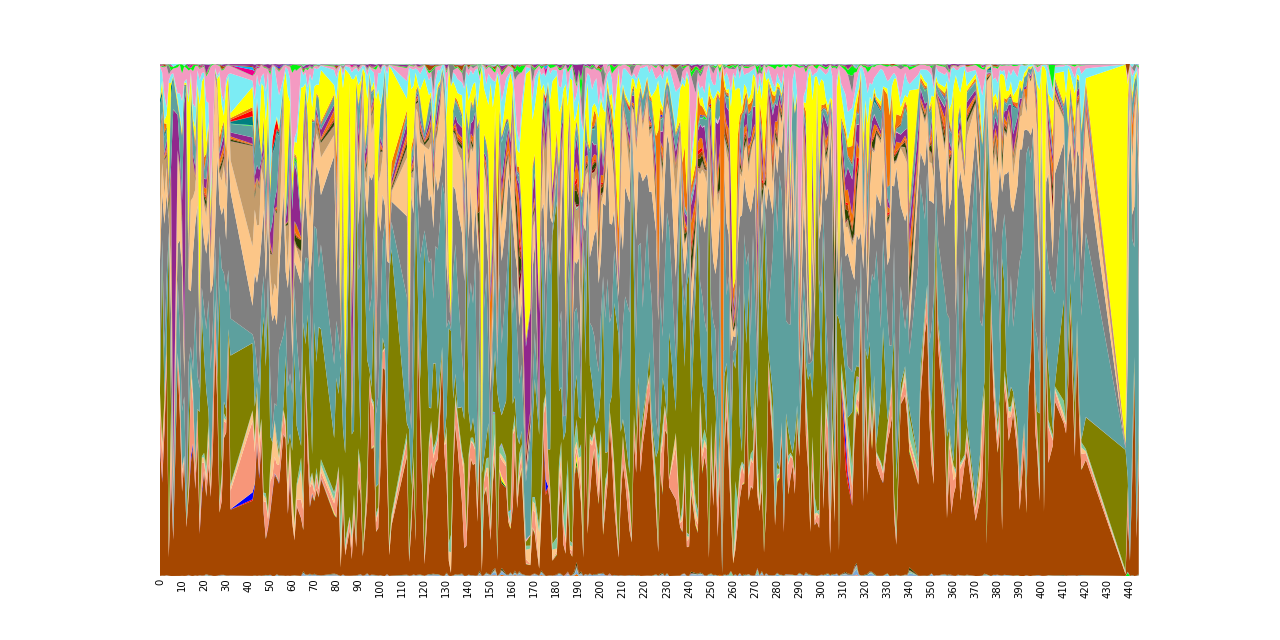

Supplement: Additional file 12 — Temporal variation in phylum, class, order, family, and genus abundances (M3 left palm). The x-axis scale differs between M3 and F4 plots. [file gb-2011-12-5-r50-S12.ZIP › AdditionalFile12/charts/UEM6q0byfJomR42T0MmQ6Apx1LMLI3.png]

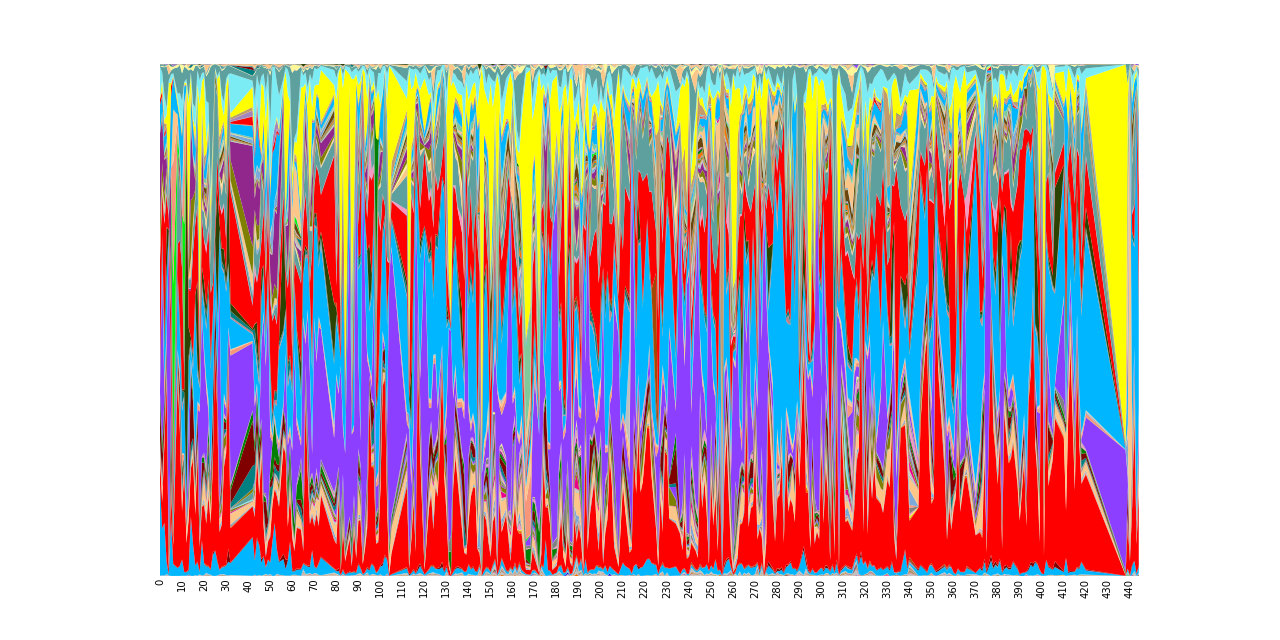

Supplement: Additional file 12 — Temporal variation in phylum, class, order, family, and genus abundances (M3 left palm). The x-axis scale differs between M3 and F4 plots. [file gb-2011-12-5-r50-S12.ZIP › AdditionalFile12/charts/USXDINjkWUm6UfcbeQiq1G6qELLYjf.png]

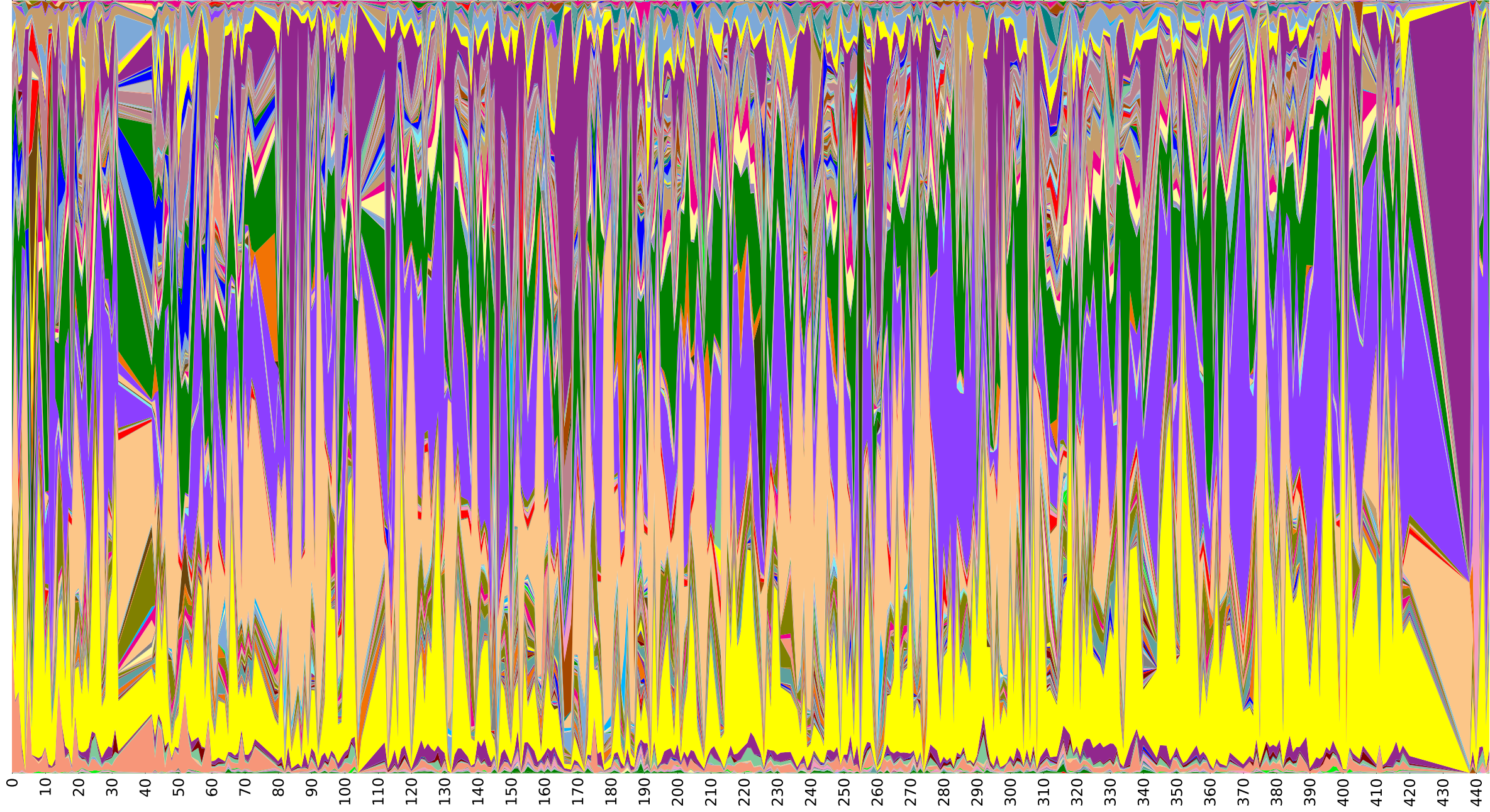

Supplement: Additional file 12 — Temporal variation in phylum, class, order, family, and genus abundances (M3 left palm). The x-axis scale differs between M3 and F4 plots. [file gb-2011-12-5-r50-S12.ZIP › AdditionalFile12/charts/xW1hs0oYBDF6ejJwPowJda9f0w0Tsz.pdf]

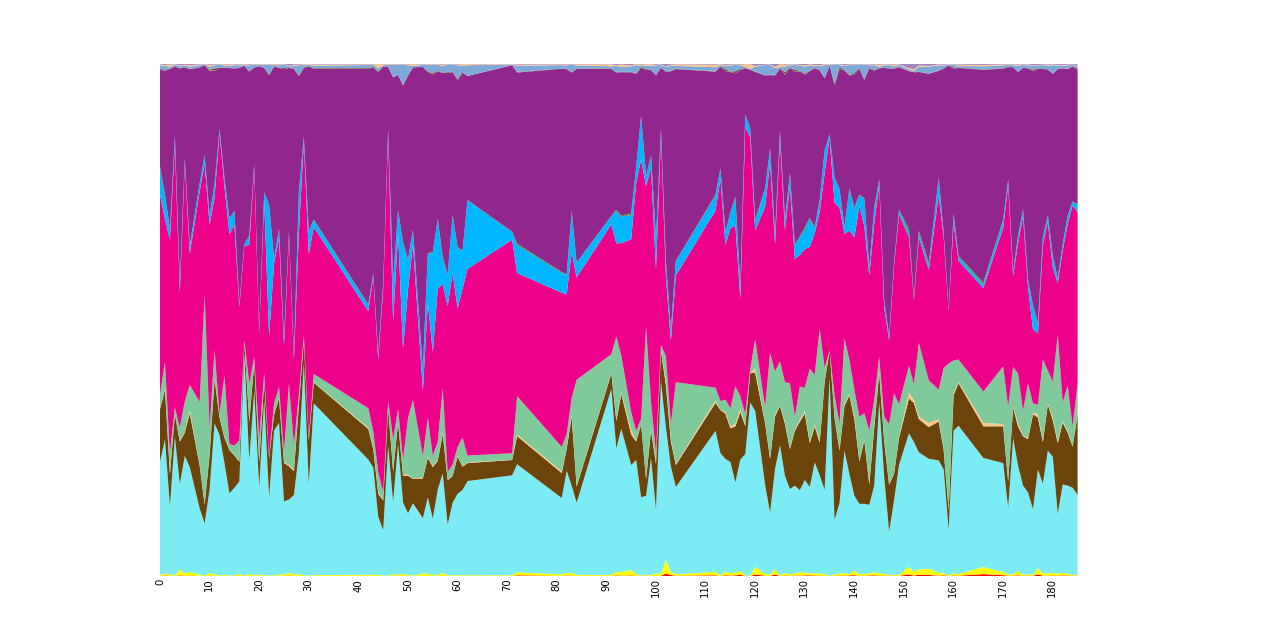

Supplement: Additional file 13 — Temporal variation in phylum, class, order, family, and genus abundances (F4 left palm). The x-axis scale differs between M3 and F4 plots. [file gb-2011-12-5-r50-S13.ZIP › AdditionalFile13/charts/0zzwJd1b5UabDq5ucfSj8Fot0428y3.png]

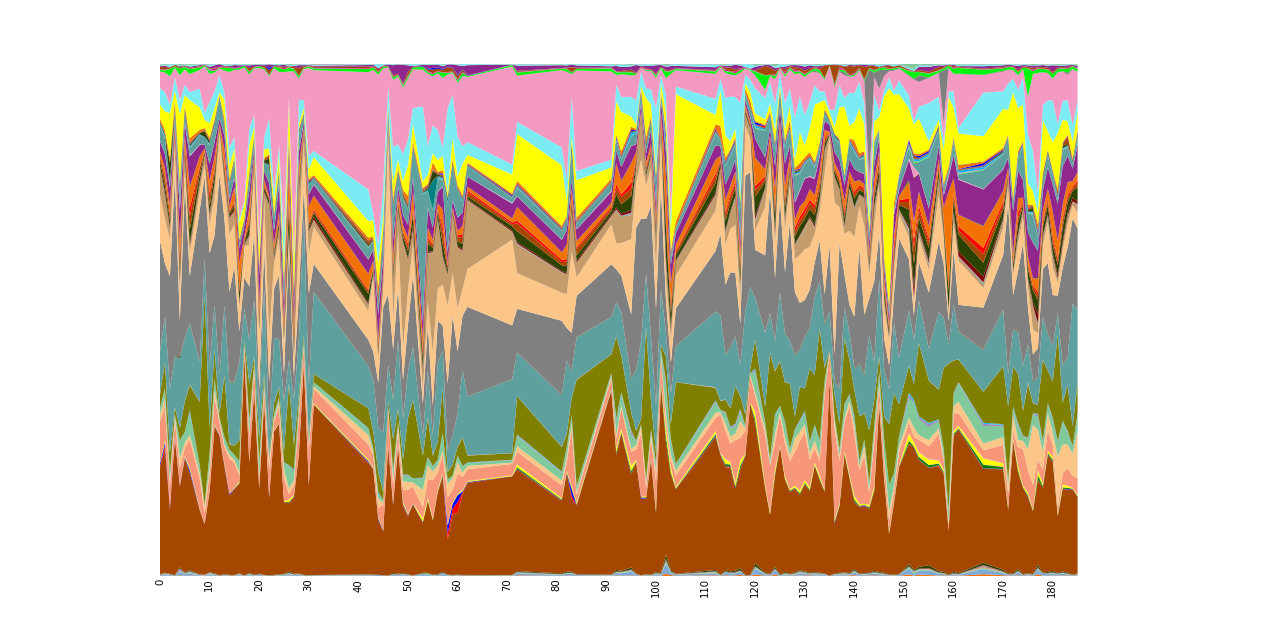

Supplement: Additional file 13 — Temporal variation in phylum, class, order, family, and genus abundances (F4 left palm). The x-axis scale differs between M3 and F4 plots. [file gb-2011-12-5-r50-S13.ZIP › AdditionalFile13/charts/3NKb2AJoqN2YcXxMEGY0kmrpN0CW0l.png]

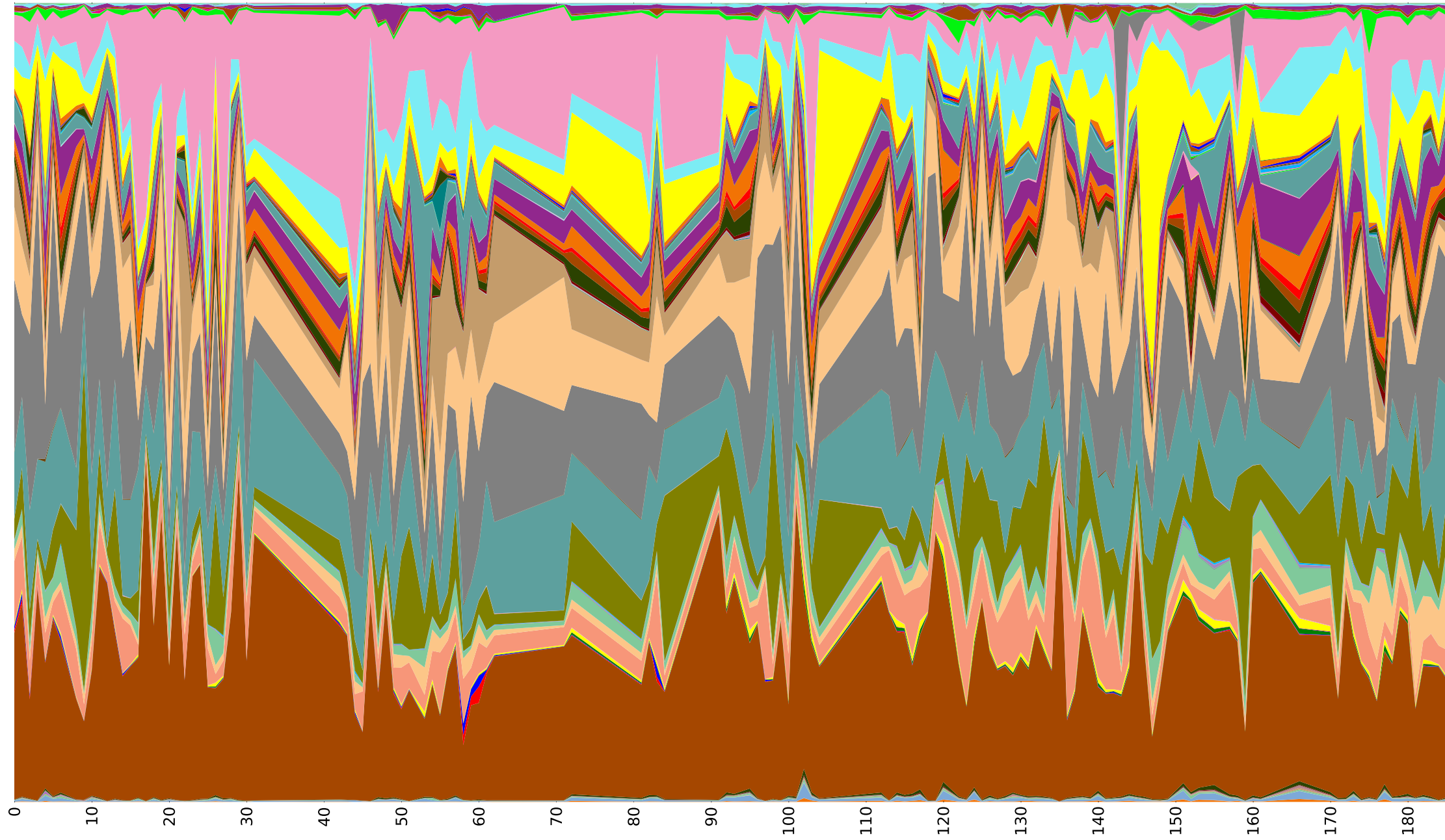

Supplement: Additional file 13 — Temporal variation in phylum, class, order, family, and genus abundances (F4 left palm). The x-axis scale differs between M3 and F4 plots. [file gb-2011-12-5-r50-S13.ZIP › AdditionalFile13/charts/47Z0XIqm9ZYrkdQUaDOFmxwOPnwpFn.pdf]

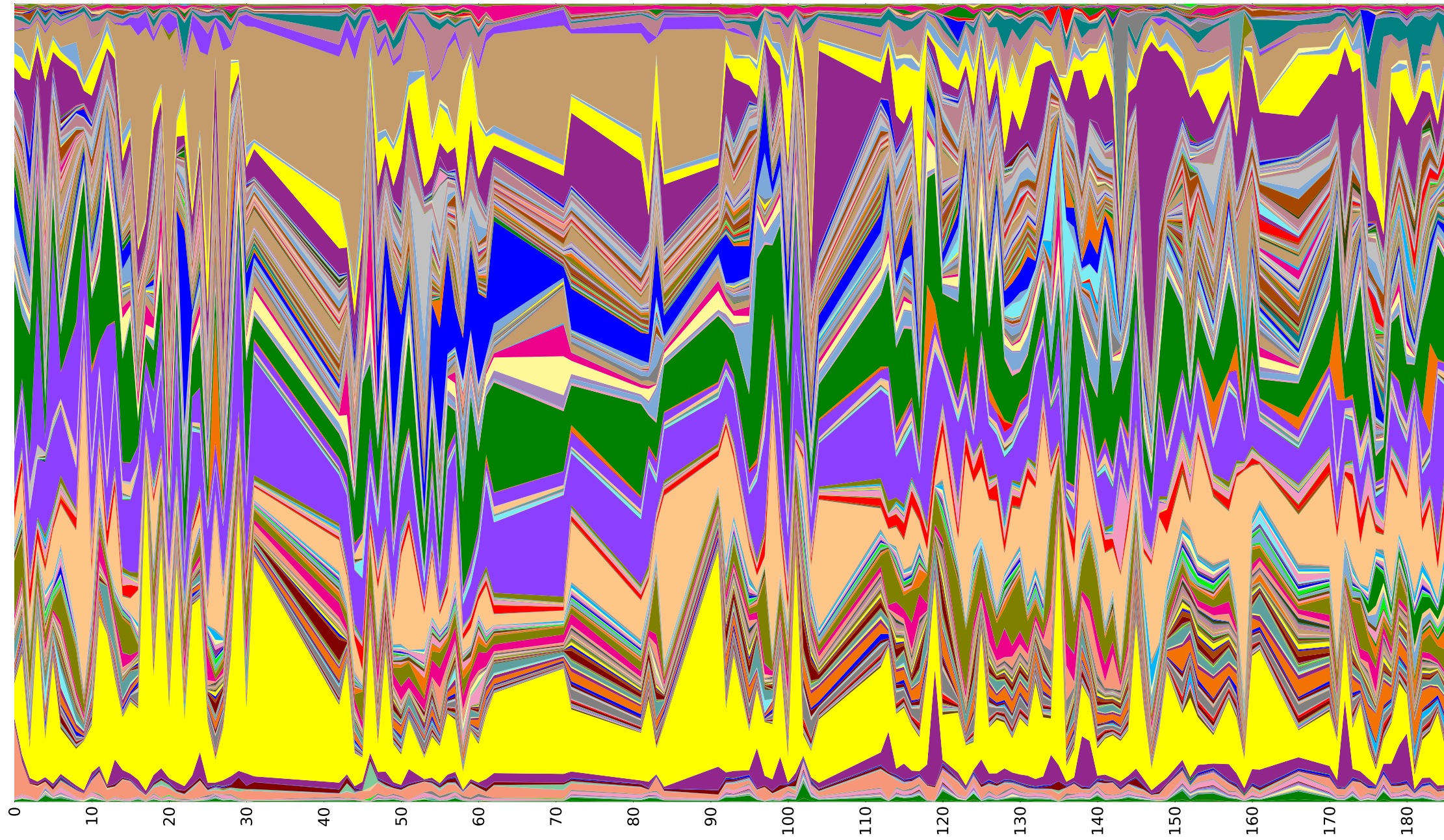

Supplement: Additional file 13 — Temporal variation in phylum, class, order, family, and genus abundances (F4 left palm). The x-axis scale differs between M3 and F4 plots. [file gb-2011-12-5-r50-S13.ZIP › AdditionalFile13/charts/Dnum9JeP03WeznGnxAfHw0cPiZlcY3.pdf]

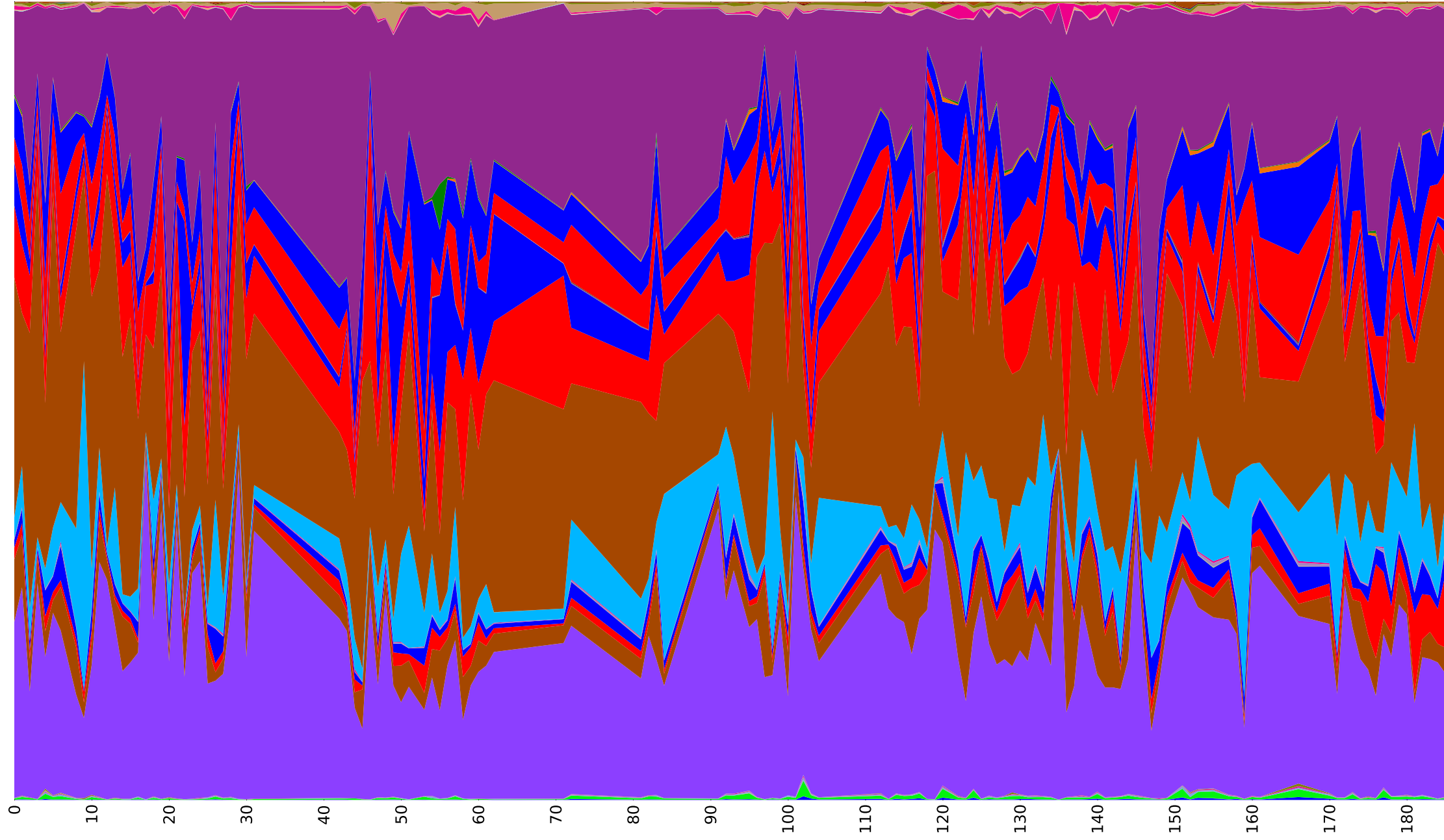

Supplement: Additional file 13 — Temporal variation in phylum, class, order, family, and genus abundances (F4 left palm). The x-axis scale differs between M3 and F4 plots. [file gb-2011-12-5-r50-S13.ZIP › AdditionalFile13/charts/E1M1FkZeBHo9y7ISZFo4ca8F8UYOOx.pdf]

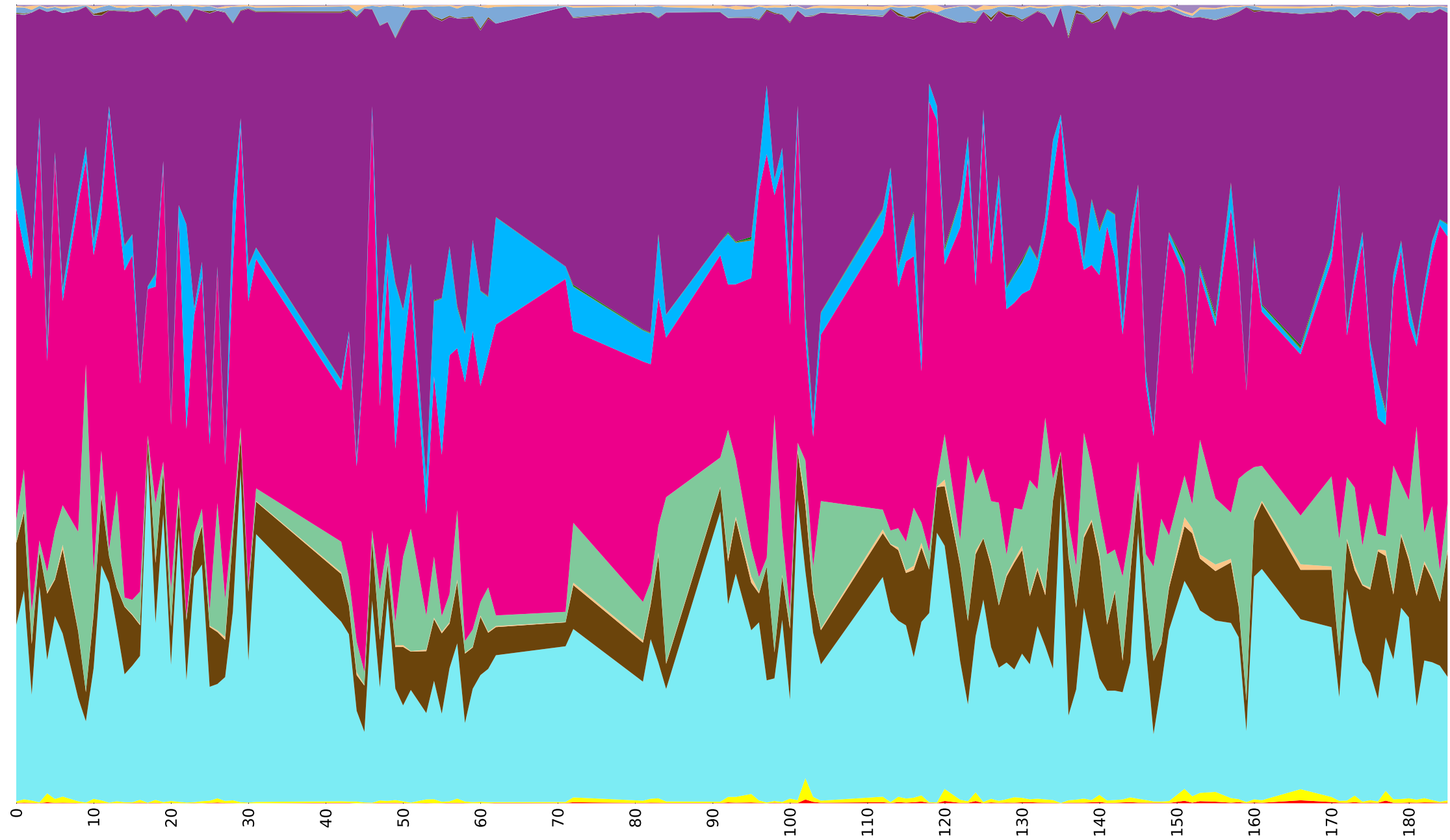

Supplement: Additional file 13 — Temporal variation in phylum, class, order, family, and genus abundances (F4 left palm). The x-axis scale differs between M3 and F4 plots. [file gb-2011-12-5-r50-S13.ZIP › AdditionalFile13/charts/hf67qBtwCbaFXt1B5NGS7ejtFfFRtH.pdf]

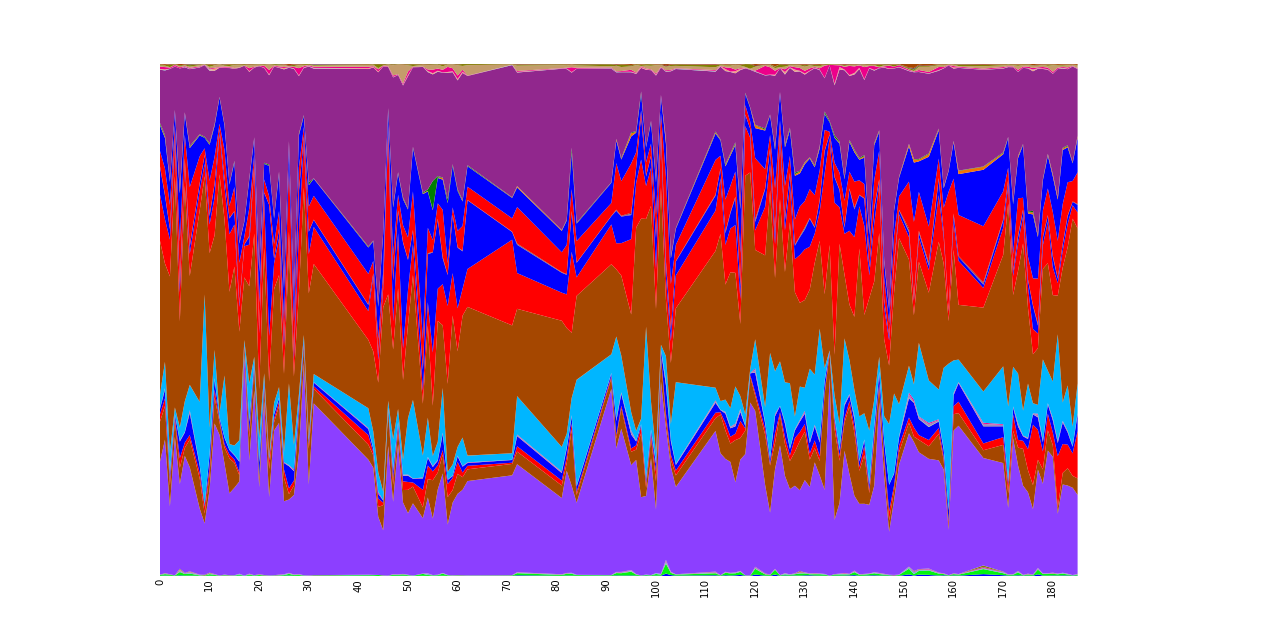

Supplement: Additional file 13 — Temporal variation in phylum, class, order, family, and genus abundances (F4 left palm). The x-axis scale differs between M3 and F4 plots. [file gb-2011-12-5-r50-S13.ZIP › AdditionalFile13/charts/hiZZ86luqjwBS2Lj9Z0KRMipr7KwGY.png]

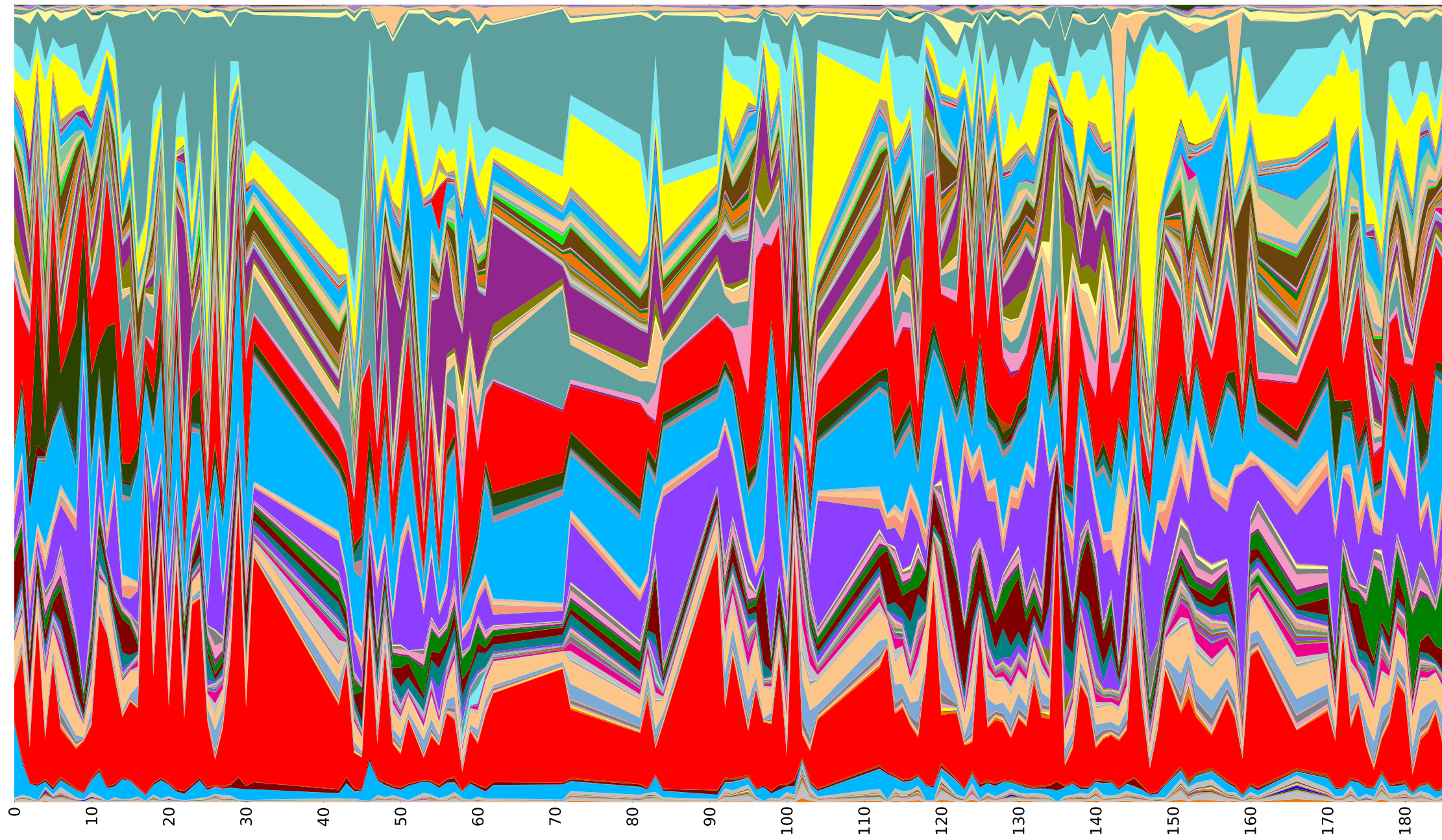

Supplement: Additional file 13 — Temporal variation in phylum, class, order, family, and genus abundances (F4 left palm). The x-axis scale differs between M3 and F4 plots. [file gb-2011-12-5-r50-S13.ZIP › AdditionalFile13/charts/pw7P0in0uSjP2paWnS6WhXH26Pqsqr.pdf]

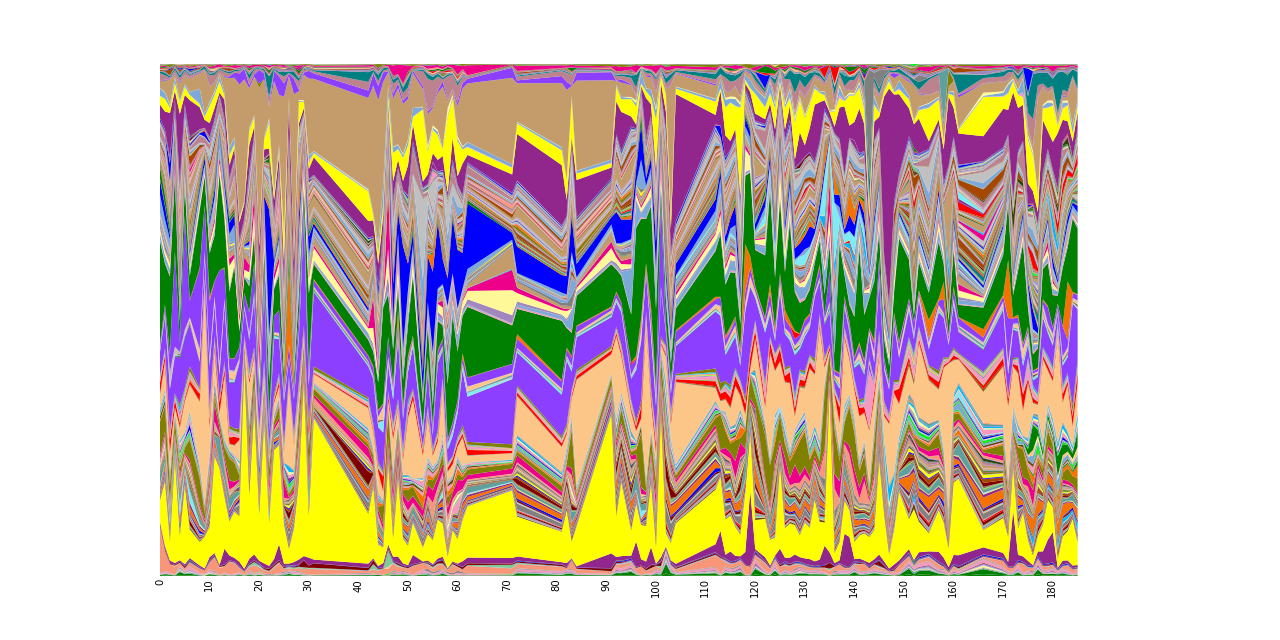

Supplement: Additional file 13 — Temporal variation in phylum, class, order, family, and genus abundances (F4 left palm). The x-axis scale differs between M3 and F4 plots. [file gb-2011-12-5-r50-S13.ZIP › AdditionalFile13/charts/YnanGE3InOEfx0E0OYH0N058BDS9ZS.png]

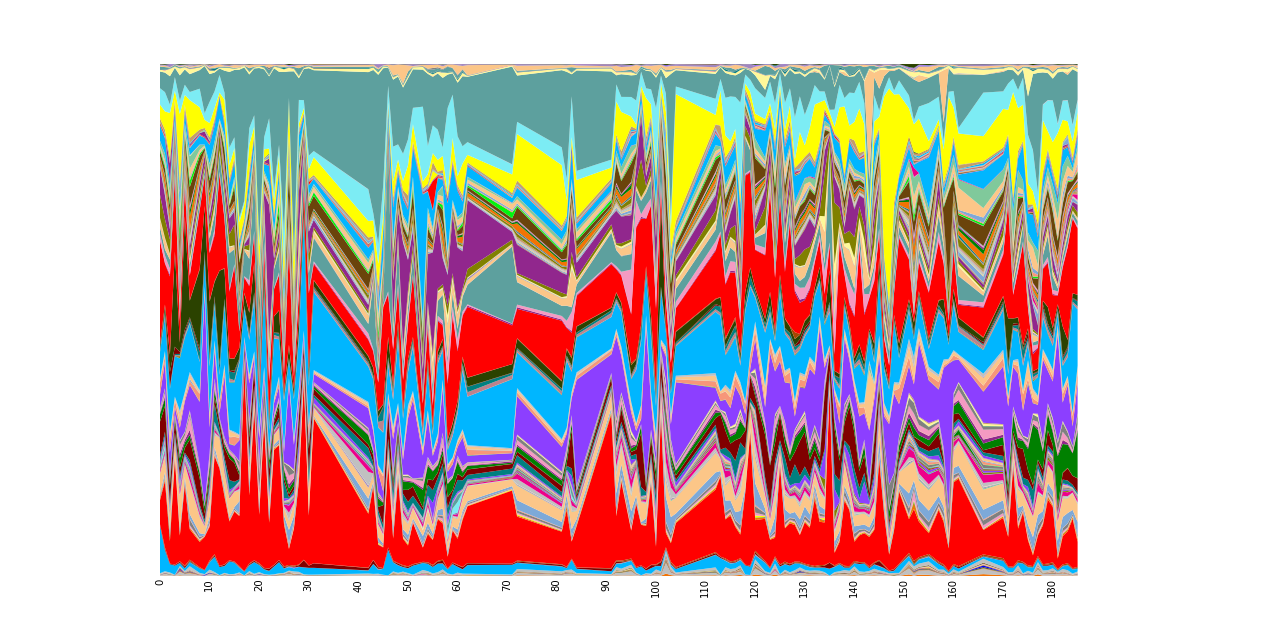

Supplement: Additional file 13 — Temporal variation in phylum, class, order, family, and genus abundances (F4 left palm). The x-axis scale differs between M3 and F4 plots. [file gb-2011-12-5-r50-S13.ZIP › AdditionalFile13/charts/YQz00ZeMutqBTxMZyr61PsSIczMSsC.png]

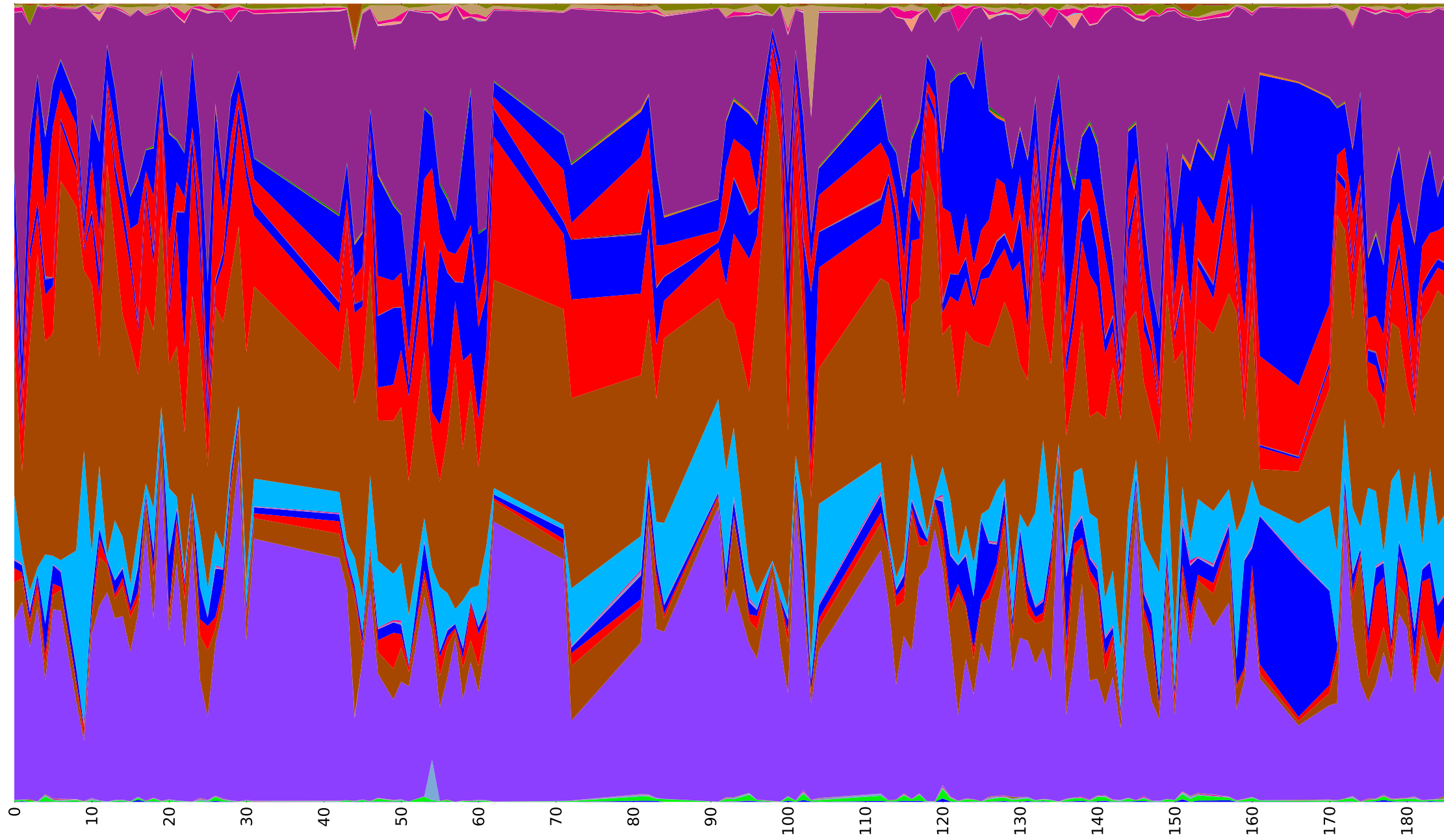

Supplement: Additional file 15 — Temporal variation in phylum, class, order, family, and genus abundances (F4 right palm). The x-axis scale differs between M3 and F4 plots. [file gb-2011-12-5-r50-S15.ZIP › AdditionalFile15/charts/0yhZgKc2C0fTcw5H4lfycyrQCHdDEw.pdf]

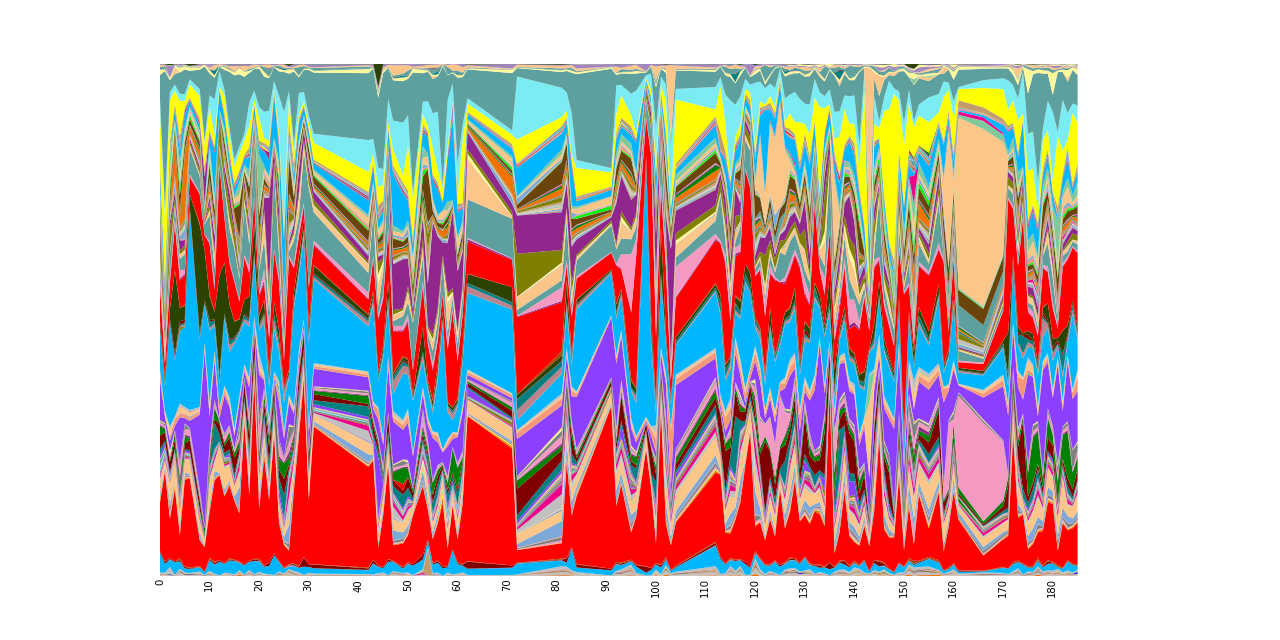

Supplement: Additional file 15 — Temporal variation in phylum, class, order, family, and genus abundances (F4 right palm). The x-axis scale differs between M3 and F4 plots. [file gb-2011-12-5-r50-S15.ZIP › AdditionalFile15/charts/27Xo3ZYZuSUWSax5EgIP9OsmRC4nSr.png]

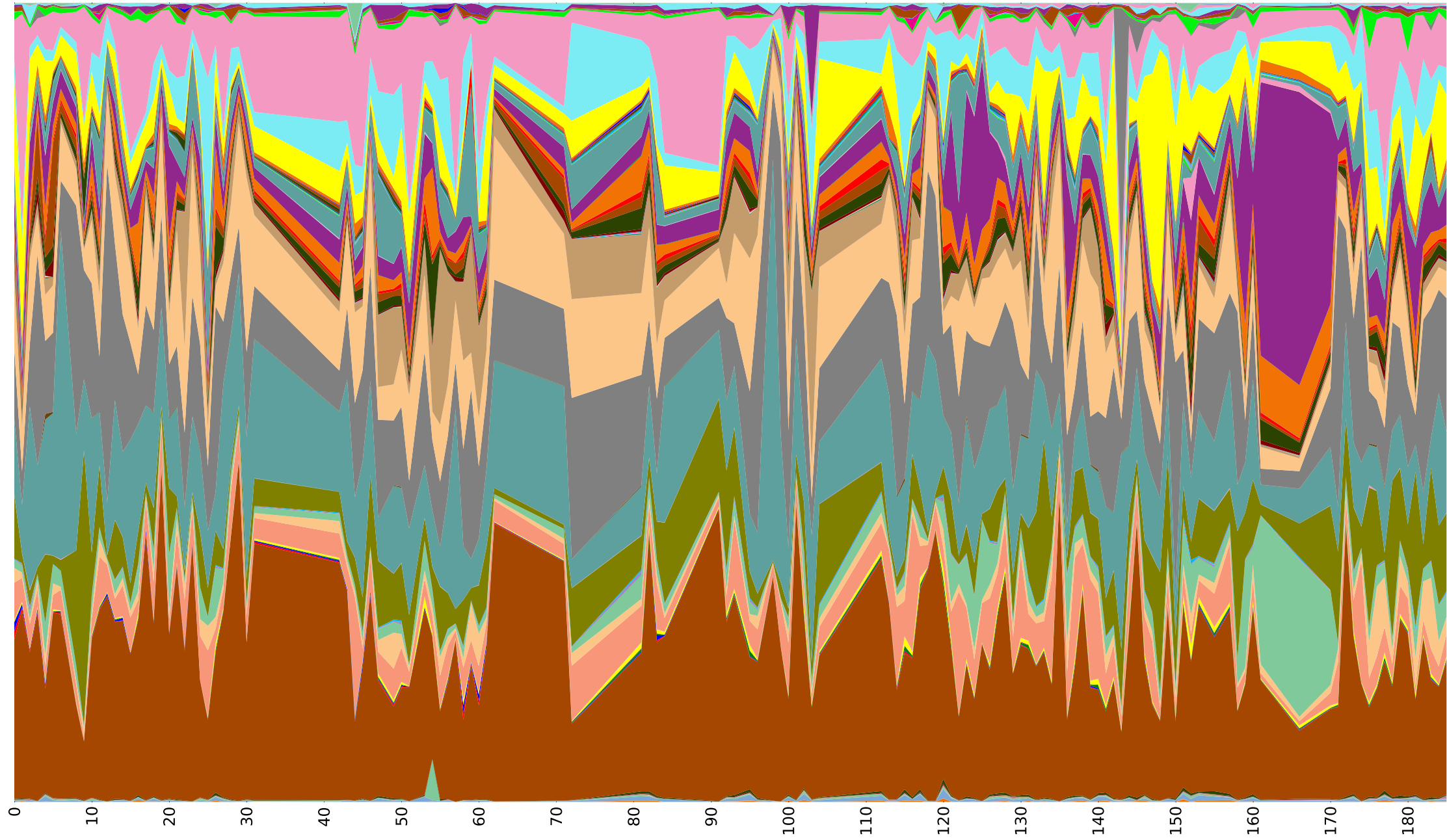

Supplement: Additional file 15 — Temporal variation in phylum, class, order, family, and genus abundances (F4 right palm). The x-axis scale differs between M3 and F4 plots. [file gb-2011-12-5-r50-S15.ZIP › AdditionalFile15/charts/39zYiy0bBZeFSJ4Z8qDW8hqMZh0LsM.pdf]

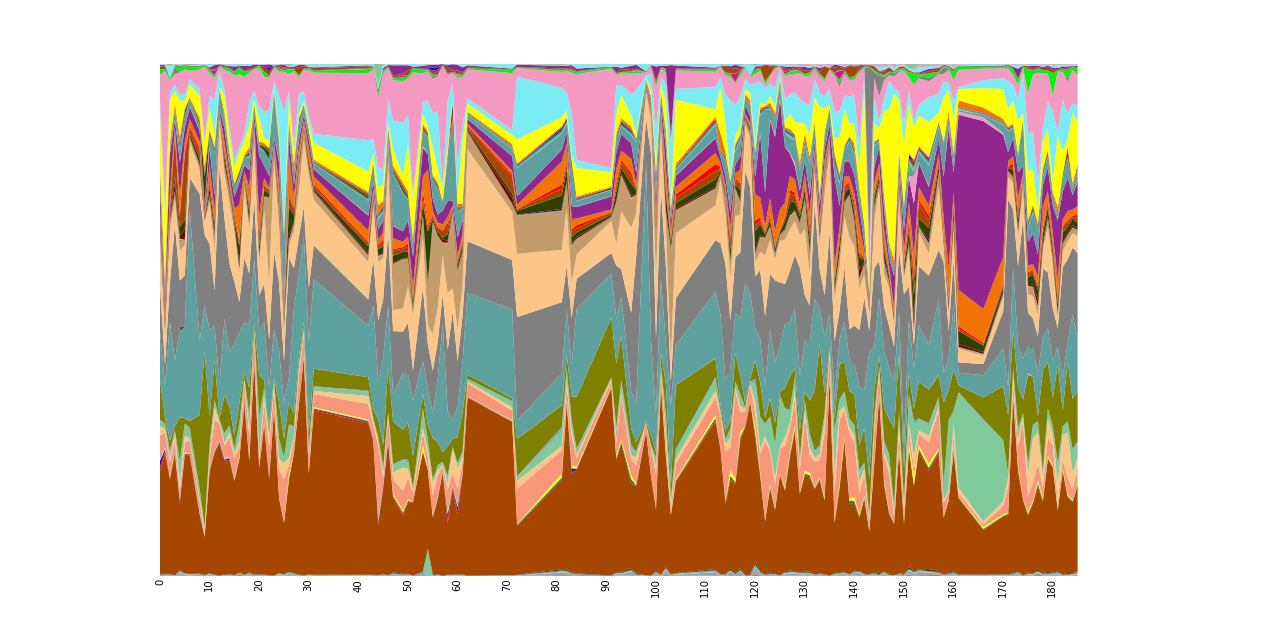

Supplement: Additional file 15 — Temporal variation in phylum, class, order, family, and genus abundances (F4 right palm). The x-axis scale differs between M3 and F4 plots. [file gb-2011-12-5-r50-S15.ZIP › AdditionalFile15/charts/4xmCwkgRufiZX7XeaZrcjAP8LPLIUU.png]

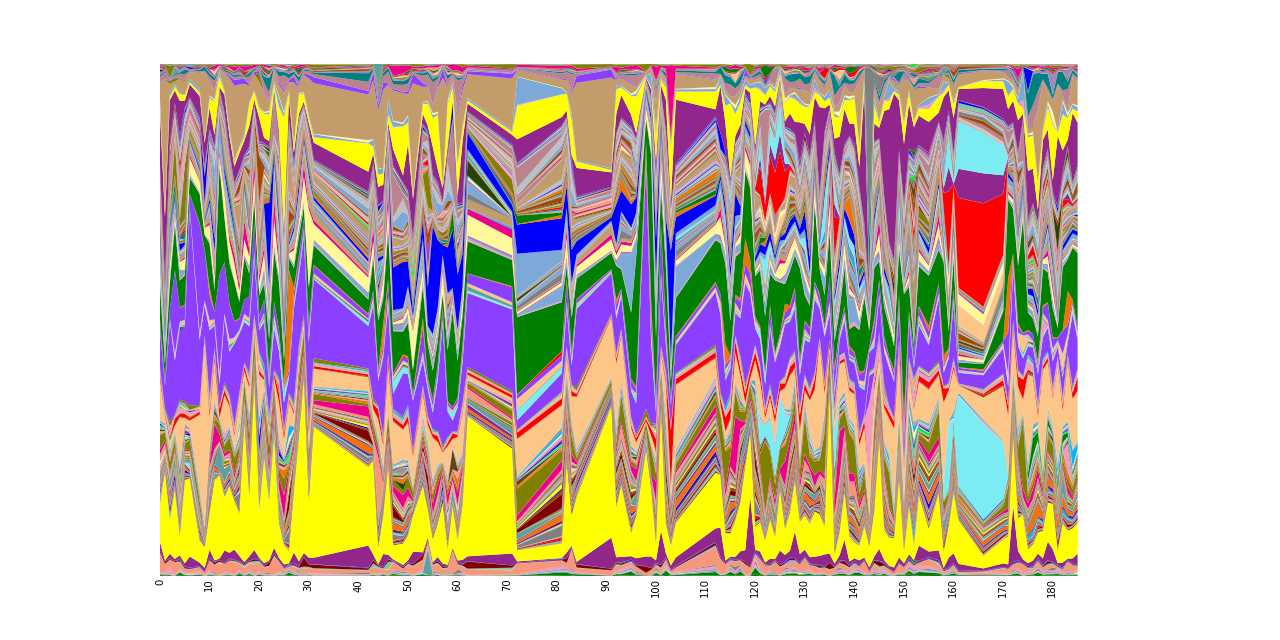

Supplement: Additional file 15 — Temporal variation in phylum, class, order, family, and genus abundances (F4 right palm). The x-axis scale differs between M3 and F4 plots. [file gb-2011-12-5-r50-S15.ZIP › AdditionalFile15/charts/a6PR3FH2eGtiRj0obUNePU20RTffSJ.png]

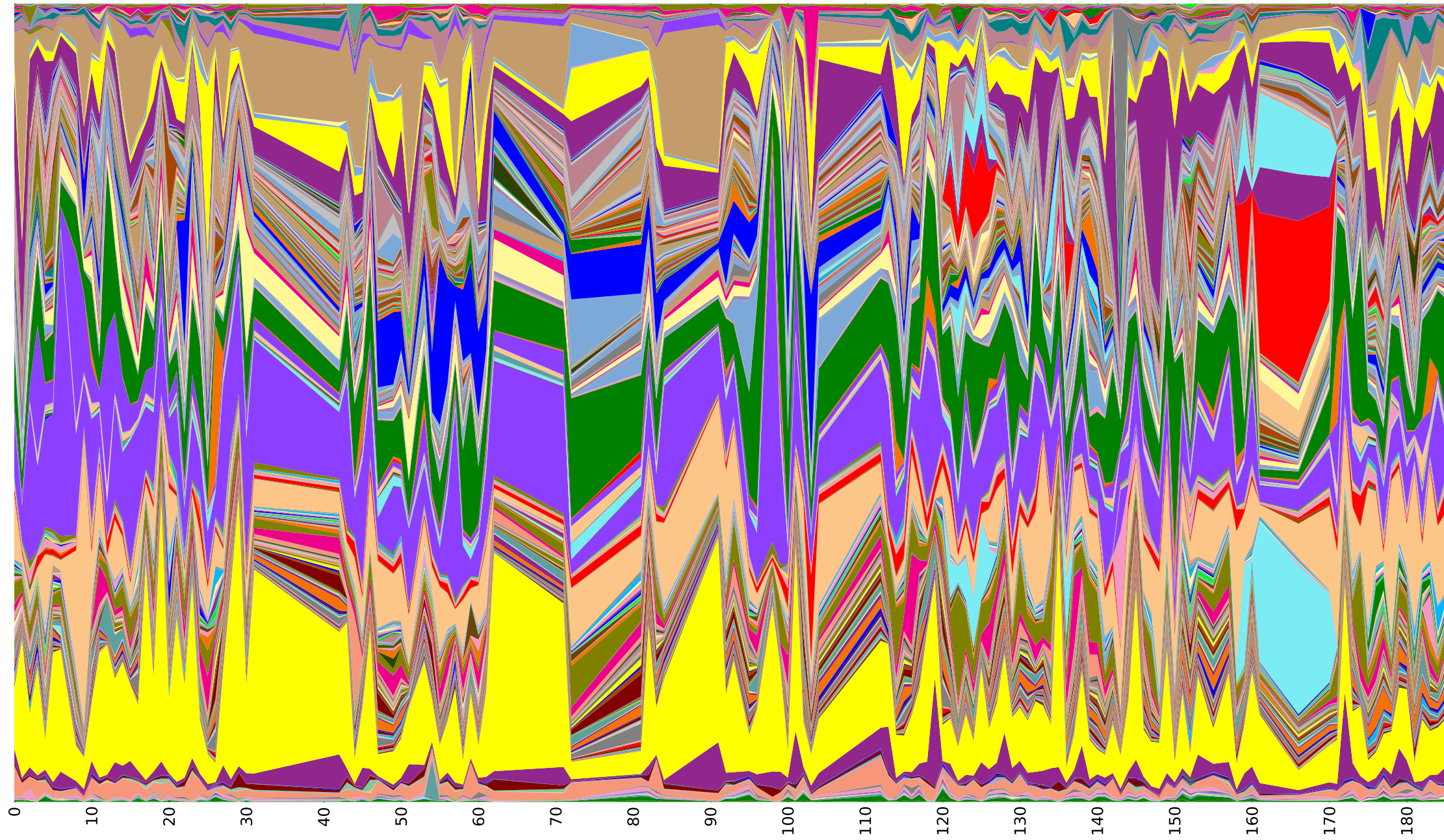

Supplement: Additional file 15 — Temporal variation in phylum, class, order, family, and genus abundances (F4 right palm). The x-axis scale differs between M3 and F4 plots. [file gb-2011-12-5-r50-S15.ZIP › AdditionalFile15/charts/NfwaX9goouXXz33TfPRtfZtoFHzelh.pdf]

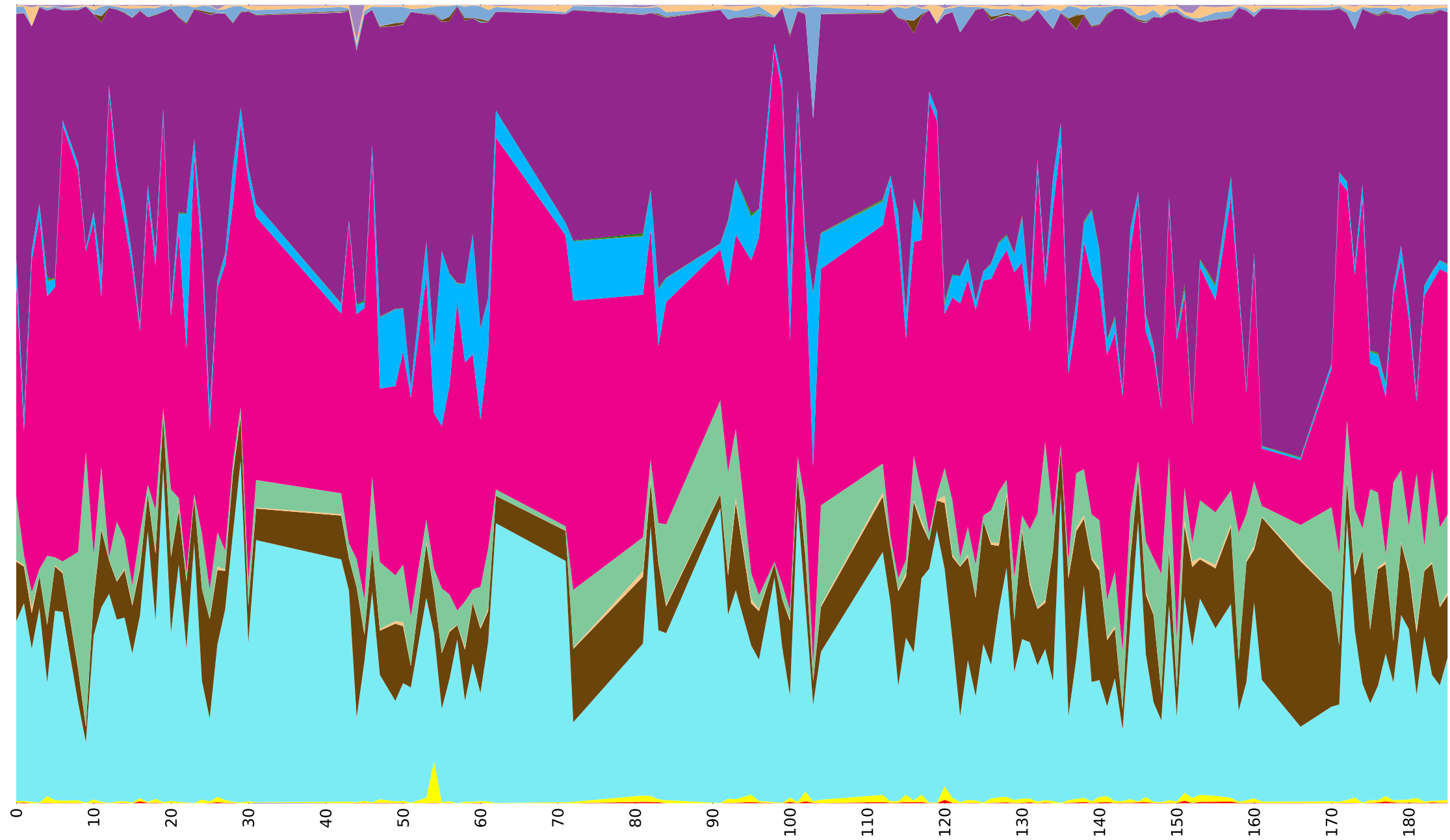

Supplement: Additional file 15 — Temporal variation in phylum, class, order, family, and genus abundances (F4 right palm). The x-axis scale differs between M3 and F4 plots. [file gb-2011-12-5-r50-S15.ZIP › AdditionalFile15/charts/OoJ5G7pfLGWuxxEZkSPJNTpOzb0Mz0.pdf]

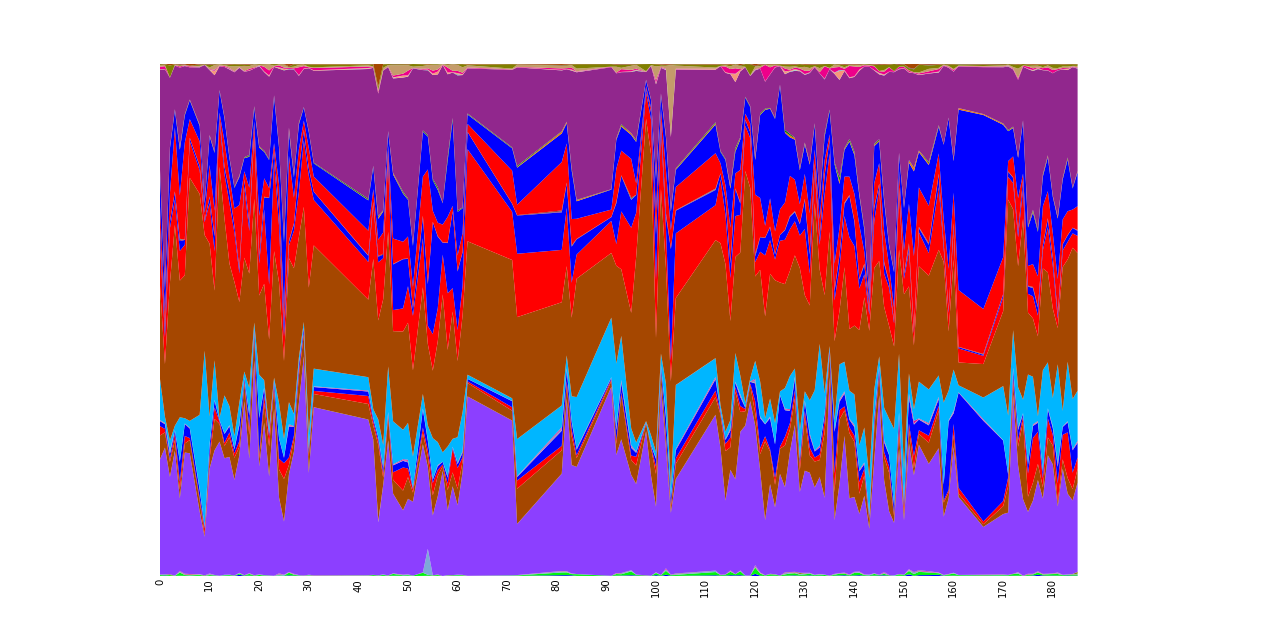

Supplement: Additional file 15 — Temporal variation in phylum, class, order, family, and genus abundances (F4 right palm). The x-axis scale differs between M3 and F4 plots. [file gb-2011-12-5-r50-S15.ZIP › AdditionalFile15/charts/p5T0HKQmOY2mZzZDaNzTk3BS3QaQuP.png]

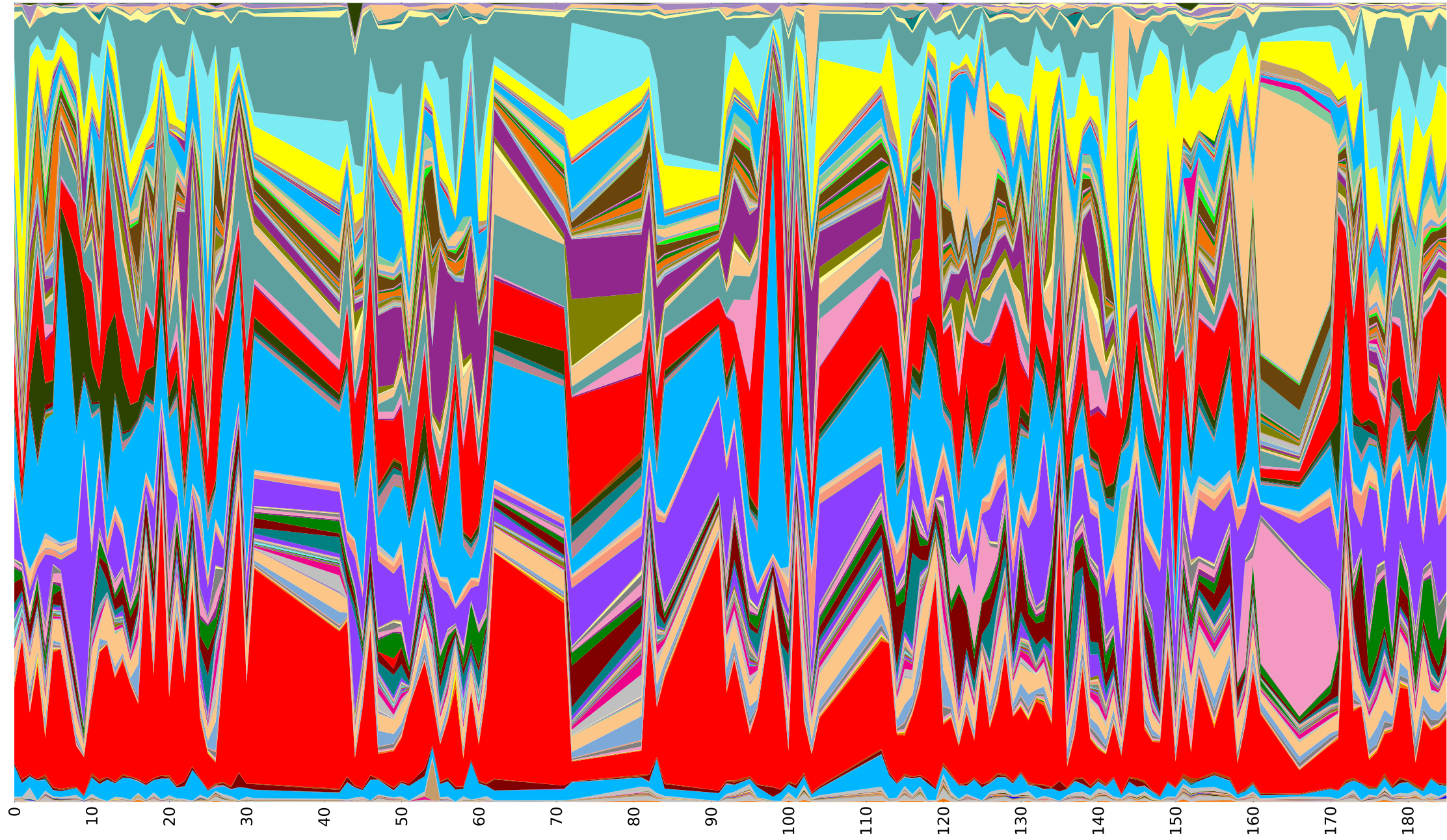

Supplement: Additional file 15 — Temporal variation in phylum, class, order, family, and genus abundances (F4 right palm). The x-axis scale differs between M3 and F4 plots. [file gb-2011-12-5-r50-S15.ZIP › AdditionalFile15/charts/s96gIDjFFZx4zFiRSWgzgpZYNJofyz.pdf]

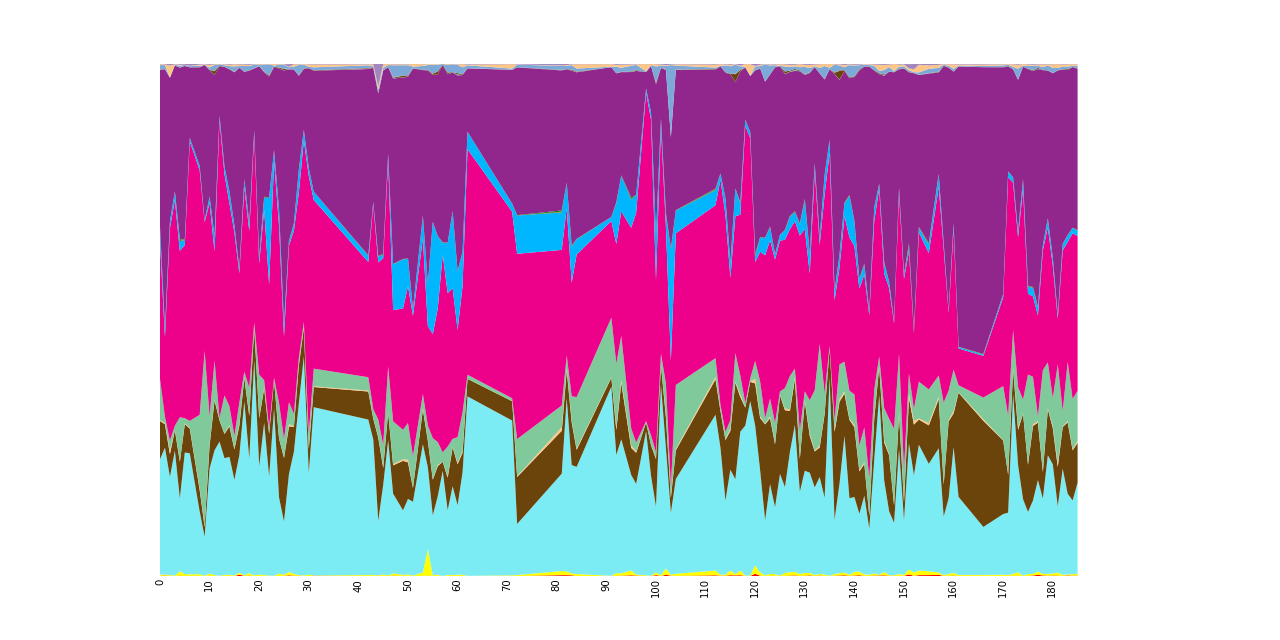

Supplement: Additional file 15 — Temporal variation in phylum, class, order, family, and genus abundances (F4 right palm). The x-axis scale differs between M3 and F4 plots. [file gb-2011-12-5-r50-S15.ZIP › AdditionalFile15/charts/Z0tB6pfgCp7Y0MzbrcOPzCxFKQYk8g.png]
