# Supplementary material for: Moving pictures of the human microbiome
Source: Genome Biol. 2011 May 30;12(5):R50. doi: 10.1186/gb-2011-12-5-r50 (PMC3271711; doi:10.1186/gb-2011-12-5-r50)
Supplement: Additional file 8 — Temporal variation in phylum, class, order, family, and genus abundances (M3 gut). The x-axis scale differs between M3 and F4 plots. [file gb-2011-12-5-r50-S8.ZIP › AdditionalFile8/charts/Ffo5hJJrOJuNRDqsbycjQhYIUQIqNb_legend.pdf]

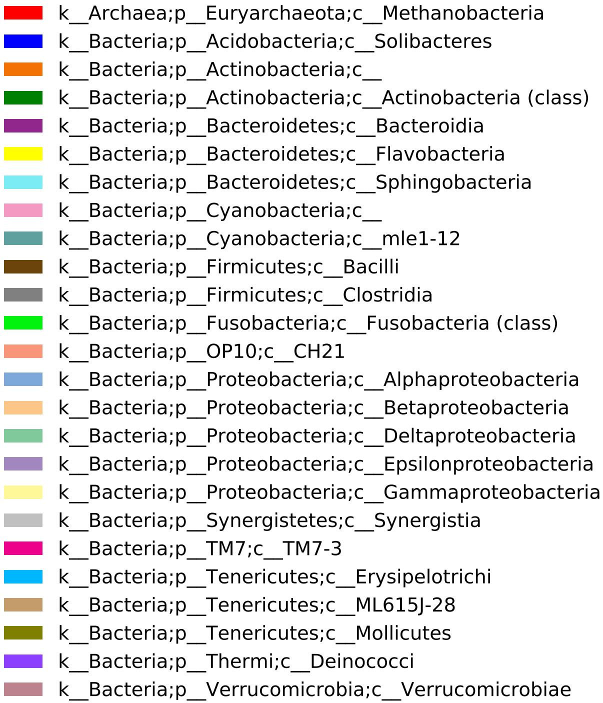
k__Archaea;p__Euryarchaeota;c__Methanobacteria
k__Bacteria;p__Acidobacteria;c__Solibacteres
k__Bacteria;p__Actinobacteria;c__
k__Bacteria;p__Actinobacteria;c__Actinobacteria (class)
k__Bacteria;p__Bacteroidetes;c__Bacteroidia
k__Bacteria;p__Bacteroidetes;c__Flavobacteria
k__Bacteria;p__Bacteroidetes;c__Sphingobacteria
k__Bacteria;p__Cyanobacteria;c__
k__Bacteria;p__Cyanobacteria;c__mle1-12
k__Bacteria;p__Firmicutes;c__Bacilli
k__Bacteria;p__Firmicutes;c__Clostridia
k__Bacteria;p__Fusobacteria;c__Fusobacteria (class)
k__Bacteria;p__OP10;c__CH21
k__Bacteria;p__Proteobacteria;c__Alphaproteobacteria
k__Bacteria;p__Proteobacteria;c__Betaproteobacteria
k__Bacteria;p__Proteobacteria;c__Deltaproteobacteria
k__Bacteria;p__Proteobacteria;c__Epsilonproteobacteria
k__Bacteria;p__Proteobacteria;c__Gammaproteobacteria
k__Bacteria;p__Synergistetes;c__Synergistia
k__Bacteria;p__TM7;c__TM7-3
k__Bacteria;p__Tenericutes;c__Erysipelotrichi
k__Bacteria;p__Tenericutes;c__ML615J-28
k__Bacteria;p__Tenericutes;c__Mollicutes
k__Bacteria;p__Thermi;c__Deinococci
k__Bacteria;p__Verrucomicrobia;c__Verrucomicrobiae
