# Supplementary material for: Moving pictures of the human microbiome
Source: Genome Biol. 2011 May 30;12(5):R50. doi: 10.1186/gb-2011-12-5-r50 (PMC3271711; doi:10.1186/gb-2011-12-5-r50)
Supplement: Additional file 8 — Temporal variation in phylum, class, order, family, and genus abundances (M3 gut). The x-axis scale differs between M3 and F4 plots. [file gb-2011-12-5-r50-S8.ZIP › AdditionalFile8/charts/jsZYfyxzst5NP8DOOxLmtBl2hfGzmE_legend.pdf]

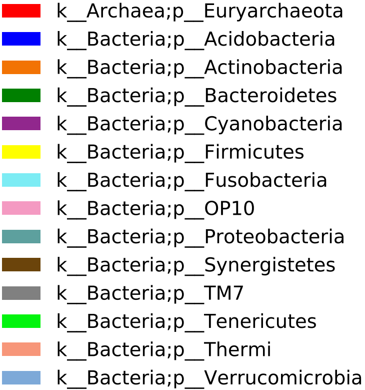
k__Archaea;p__Euryarchaeota
k__Bacteria;p__Acidobacteria
k__Bacteria;p__Actinobacteria
k__Bacteria;p__Bacteroidetes
k__Bacteria;p__Cyanobacteria
k__Bacteria;p__Firmicutes
k__Bacteria;p__Fusobacteria
k__Bacteria;p__OP10
k__Bacteria;p__Proteobacteria
k__Bacteria;p__Synergistetes
k__Bacteria;p__TM7
k__Bacteria;p__Tenericutes
k__Bacteria;p__Thermi
k__Bacteria;p__Verrucomicrobia
