# Supplementary material for: Moving pictures of the human microbiome
Source: Genome Biol. 2011 May 30;12(5):R50. doi: 10.1186/gb-2011-12-5-r50 (PMC3271711; doi:10.1186/gb-2011-12-5-r50)
Supplement: Additional file 8 — Temporal variation in phylum, class, order, family, and genus abundances (M3 gut). The x-axis scale differs between M3 and F4 plots. [file gb-2011-12-5-r50-S8.ZIP › AdditionalFile8/charts/UEikss9E9sFp3Z2ZYKJZa71CjKGRjy_legend.pdf]

k__Archaea;p__Euryarchaeota;c__Methanobacteria;o__Methanobacteriales
k__Bacteria;p__Acidobacteria;c__Solibacteres;o__Solibacterales
k__Bacteria;p__Actinobacteria;c__;o__
k__Bacteria;p__Actinobacteria;c__Actinobacteria (class);o__Actinomycetales
k__Bacteria;p__Actinobacteria;c__Actinobacteria (class);o__Bifidobacteriales
k__Bacteria;p__Actinobacteria;c__Actinobacteria (class);o__Coriobacteriales
k__Bacteria;p__Actinobacteria;c__Actinobacteria (class);o__Solirubrobacterales
k__Bacteria;p__Bacteroidetes;c__Bacteroidia;o__Bacteroidales
k__Bacteria;p__Bacteroidetes;c__Flavobacteria;o__Flavobacteriales
k__Bacteria;p__Bacteroidetes;c__Sphingobacteria;o__Sphingobacteriales
k__Bacteria;p__Cyanobacteria;c__;o__
k__Bacteria;p__Cyanobacteria;c__mle1-12;o__
k__Bacteria;p__Firmicutes;c__Bacilli;o__Bacillales
k__Bacteria;p__Firmicutes;c__Bacilli;o__Erysipelotrichales
k__Bacteria;p__Firmicutes;c__Bacilli;o__Lactobacillales
k__Bacteria;p__Firmicutes;c__Clostridia;o__Clostridiales
k__Bacteria;p__Fusobacteria;c__Fusobacteria (class);o__Fusobacteriales
k__Bacteria;p__OP10;c__CH21;o__
k__Bacteria;p__Proteobacteria;c__Alphaproteobacteria;o__Caulobacterales
k__Bacteria;p__Proteobacteria;c__Alphaproteobacteria;o__Rhizobiales
k__Bacteria;p__Proteobacteria;c__Alphaproteobacteria;o__Rhodobacterales
k__Bacteria;p__Proteobacteria;c__Alphaproteobacteria;o__Rhodospirillales
k__Bacteria;p__Proteobacteria;c__Alphaproteobacteria;o__Sphingomonadales
k__Bacteria;p__Proteobacteria;c__Betaproteobacteria;o__
k__Bacteria;p__Proteobacteria;c__Betaproteobacteria;o__Burkholderiales
k__Bacteria;p__Proteobacteria;c__Betaproteobacteria;o__Gallionellales
k__Bacteria;p__Proteobacteria;c__Betaproteobacteria;o__Hydrogenophilales
k__Bacteria;p__Proteobacteria;c__Betaproteobacteria;o__Methylophilales
k__Bacteria;p__Proteobacteria;c__Betaproteobacteria;o__Neisseriales
k__Bacteria;p__Proteobacteria;c__Betaproteobacteria;o__Nitrosomonadales
k__Bacteria;p__Proteobacteria;c__Betaproteobacteria;o__Rhodocyclales
k__Bacteria;p__Proteobacteria;c__Deltaproteobacteria;o__Bdellovibrionales
k__Bacteria;p__Proteobacteria;c__Deltaproteobacteria;o__Desulfovibrionales
k__Bacteria;p__Proteobacteria;c__Deltaproteobacteria;o__MIZ46
k__Bacteria;p__Proteobacteria;c__Deltaproteobacteria;o__Myxococcales
k__Bacteria;p__Proteobacteria;c__Epsilonproteobacteria;o__Campylobacterales
k__Bacteria;p__Proteobacteria;c__Gammaproteobacteria;o__
k__Bacteria;p__Proteobacteria;c__Gammaproteobacteria;o__Aeromonadales
k__Bacteria;p__Proteobacteria;c__Gammaproteobacteria;o__Alteromonadales
k__Bacteria;p__Proteobacteria;c__Gammaproteobacteria;o__Cardiobacteriales
k__Bacteria;p__Proteobacteria;c__Gammaproteobacteria;o__Chromatiales
k__Bacteria;p__Proteobacteria;c__Gammaproteobacteria;o__Enterobacteriales
k__Bacteria;p__Proteobacteria;c__Gammaproteobacteria;o__Oceanospirillales
k__Bacteria;p__Proteobacteria;c__Gammaproteobacteria;o__Pasteurellales
k__Bacteria;p__Proteobacteria;c__Gammaproteobacteria;o__Pseudomonadales
k__Bacteria;p__Proteobacteria;c__Gammaproteobacteria;o__Thiotrichales
k__Bacteria;p__Proteobacteria;c__Gammaproteobacteria;o__Xanthomonadales
k__Bacteria;p__Synergistetes;c__Synergistia;o__Synergistales
k__Bacteria;p__TM7;c__TM7-3;o__EW055
k__Bacteria;p__Tenericutes;c__Erysipelotrichi;o__Erysipelotrichales
k__Bacteria;p__Tenericutes;c__ML615J-28;o__
k__Bacteria;p__Tenericutes;c__Mollicutes;o__Mycoplasmatales
k__Bacteria;p__Tenericutes;c__Mollicutes;o__RF39
k__Bacteria;p__Thermi;c__Deinococci;o__Deinococcales
k__Bacteria;p__Verrucomicrobia;c__Verrucomicrobiae;o__Verrucomicrobiales
