# Supplementary material for: Moving pictures of the human microbiome
Source: Genome Biol. 2011 May 30;12(5):R50. doi: 10.1186/gb-2011-12-5-r50 (PMC3271711; doi:10.1186/gb-2011-12-5-r50)
Supplement: Additional file 11 — Temporal variation in phylum, class, order, family, and genus abundances (F4 tongue). The x-axis scale differs between M3 and F4 plots. [file gb-2011-12-5-r50-S11.ZIP › AdditionalFile11/charts/kjFMnchF0EDBq9ZT4aCy20ORb7FdyL_legend.pdf]

- k__Archaea;p__Crenarchaeota;c__Thaumarchaeota
- k__Bacteria;p__Acidobacteria;c__Chloracidobacteria
- k__Bacteria;p__Actinobacteria;c__
- k__Bacteria;p__Actinobacteria;c__Actinobacteria (class)
- k__Bacteria;p__Bacteroidetes;c__Bacteroidia
- k__Bacteria;p__Bacteroidetes;c__Flavobacteria
- k__Bacteria;p__Bacteroidetes;c__Sphingobacteria
- k__Bacteria;p__Chloroflexi;c__Bljii12
- k__Bacteria;p__Chloroflexi;c__SOGA31
- k__Bacteria;p__Chloroflexi;c__Thermomicrobia
- k__Bacteria;p__Cyanobacteria;c__
- k__Bacteria;p__Firmicutes;c__Bacilli
- k__Bacteria;p__Firmicutes;c__Clostridia
- k__Bacteria;p__Fusobacteria;c__Fusobacteria (class)
- k__Bacteria;p__Gemmatimonadetes;c__Gemmatimonadetes (class)
- k__Bacteria;p__Proteobacteria;c__Alphaproteobacteria
- k__Bacteria;p__Proteobacteria;c__Betaproteobacteria
- k__Bacteria;p__Proteobacteria;c__Deltaproteobacteria
- k__Bacteria;p__Proteobacteria;c__Epsilonproteobacteria
- k__Bacteria;p__Proteobacteria;c__Gammaproteobacteria
- k__Bacteria;p__SPAM;c__
- k__Bacteria;p__SR1;c__
- k__Bacteria;p__Spirochaetes;c__Spirochaetes (class)
- k__Bacteria;p__Synergistetes;c__Synergistia
- k__Bacteria;p__TM7;c__TM7-3
- k__Bacteria;p__Tenericutes;c__Erysipelotrichi
- k__Bacteria;p__Tenericutes;c__Mollicutes
- k__Bacteria;p__Thermi;c__Deinococci
- k__Bacteria;p__Verrucomicrobia;c__Spartobacteria
- k__Bacteria;p__Verrucomicrobia;c__Verrucomicrobiae
