# Supplementary material for: Moving pictures of the human microbiome
Source: Genome Biol. 2011 May 30;12(5):R50. doi: 10.1186/gb-2011-12-5-r50 (PMC3271711; doi:10.1186/gb-2011-12-5-r50)
Supplement: Additional file 11 — Temporal variation in phylum, class, order, family, and genus abundances (F4 tongue). The x-axis scale differs between M3 and F4 plots. [file gb-2011-12-5-r50-S11.ZIP › AdditionalFile11/charts/SFzEguMay03YzLqbctzdkPBRQGn0gC_legend.pdf]

k__Archaea;p__Crenarchaeota;c__Thaumarchaeota;o__Nitrososphaerales;f__Nitrososphaeraceae
k__Bacteria;p__Acidobacteria;c__Chloracidobacteria;o__;f__
k__Bacteria;p__Actinobacteria;c__;o__;f__
k__Bacteria;p__Actinobacteria;c__Actinobacteria (class);o__Acidimicrobiales;f__
k__Bacteria;p__Actinobacteria;c__Actinobacteria (class);o__Actinomycetales;f__
k__Bacteria;p__Actinobacteria;c__Actinobacteria (class);o__Actinomycetales;f__Actinomycetaceae
k__Bacteria;p__Actinobacteria;c__Actinobacteria (class);o__Actinomycetales;f__Actinosynnemataceae
k__Bacteria;p__Actinobacteria;c__Actinobacteria (class);o__Actinomycetales;f__Beutenbergiaceae
k__Bacteria;p__Actinobacteria;c__Actinobacteria (class);o__Actinomycetales;f__Bogoriellaceae
k__Bacteria;p__Actinobacteria;c__Actinobacteria (class);o__Actinomycetales;f__Brevibacteriaceae
k__Bacteria;p__Actinobacteria;c__Actinobacteria (class);o__Actinomycetales;f__Cellulomonadaceae
k__Bacteria;p__Actinobacteria;c__Actinobacteria (class);o__Actinomycetales;f__Corynebacteriaceae
k__Bacteria;p__Actinobacteria;c__Actinobacteria (class);o__Actinomycetales;f__Dermabacteraceae
k__Bacteria;p__Actinobacteria;c__Actinobacteria (class);o__Actinomycetales;f__Dermacoccaceae
k__Bacteria;p__Actinobacteria;c__Actinobacteria (class);o__Actinomycetales;f__Dermatophilaceae
k__Bacteria;p__Actinobacteria;c__Actinobacteria (class);o__Actinomycetales;f__Geodermatophilaceae
k__Bacteria;p__Actinobacteria;c__Actinobacteria (class);o__Actinomycetales;f__Intrasporangiaceae
k__Bacteria;p__Actinobacteria;c__Actinobacteria (class);o__Actinomycetales;f__Microbacteriaceae
k__Bacteria;p__Actinobacteria;c__Actinobacteria (class);o__Actinomycetales;f__Micrococcaceae
k__Bacteria;p__Actinobacteria;c__Actinobacteria (class);o__Actinomycetales;f__Mycobacteriaceae
k__Bacteria;p__Actinobacteria;c__Actinobacteria (class);o__Actinomycetales;f__Nakamurellaceae
k__Bacteria;p__Actinobacteria;c__Actinobacteria (class);o__Actinomycetales;f__Nocardiaceae
k__Bacteria;p__Actinobacteria;c__Actinobacteria (class);o__Actinomycetales;f__Nocardioidaceae
k__Bacteria;p__Actinobacteria;c__Actinobacteria (class);o__Actinomycetales;f__Nocardiopsaceae
k__Bacteria;p__Actinobacteria;c__Actinobacteria (class);o__Actinomycetales;f__Promicromonosporaceae
k__Bacteria;p__Actinobacteria;c__Actinobacteria (class);o__Actinomycetales;f__Propionibacteriaceae
k__Bacteria;p__Actinobacteria;c__Actinobacteria (class);o__Actinomycetales;f__Streptomycetaceae
k__Bacteria;p__Actinobacteria;c__Actinobacteria (class);o__Actinomycetales;f__Thermomonosporaceae
k__Bacteria;p__Actinobacteria;c__Actinobacteria (class);o__Actinomycetales;f__Tsukamurellaceae
k__Bacteria;p__Actinobacteria;c__Actinobacteria (class);o__Bifidobacteriales;f__Bifidobacteriaceae
k__Bacteria;p__Actinobacteria;c__Actinobacteria (class);o__Coriobacteriales;f__Coriobacteriaceae
k__Bacteria;p__Actinobacteria;c__Actinobacteria (class);o__MC47;f__
k__Bacteria;p__Actinobacteria;c__Actinobacteria (class);o__Solirubrobacterales;f__Solirubrobacteraceae
k__Bacteria;p__Bacteroidetes;c__Bacteroidia;o__Bacteroidales;f__
k__Bacteria;p__Bacteroidetes;c__Bacteroidia;o__Bacteroidales;f__Bacteroidaceae
k__Bacteria;p__Bacteroidetes;c__Bacteroidia;o__Bacteroidales;f__Porphyromonadaceae
k__Bacteria;p__Bacteroidetes;c__Bacteroidia;o__Bacteroidales;f__Prevotellaceae
k__Bacteria;p__Bacteroidetes;c__Bacteroidia;o__Bacteroidales;f__Rikenellaceae
k__Bacteria;p__Bacteroidetes;c__Flavobacteria;o__;f__
k__Bacteria;p__Bacteroidetes;c__Flavobacteria;o__Flavobacteriales;f__Flavobacteriaceae
k__Bacteria;p__Bacteroidetes;c__Sphingobacteria;o__Sphingobacteriales;f__
k__Bacteria;p__Bacteroidetes;c__Sphingobacteria;o__Sphingobacteriales;f__Cyclobacteriaceae
k__Bacteria;p__Bacteroidetes;c__Sphingobacteria;o__Sphingobacteriales;f__Flexibacteraceae
k__Bacteria;p__Bacteroidetes;c__Sphingobacteria;o__Sphingobacteriales;f__Sphingobacteriaceae
k__Bacteria;p__Chloroflexi;c__Bljii12;o__;f__
k__Bacteria;p__Chloroflexi;c__SOGA31;o__;f__
k__Bacteria;p__Chloroflexi;c__Thermomicrobia;o__HN1-15;f__
k__Bacteria;p__Cyanobacteria;c__;o__;f__
k__Bacteria;p__Firmicutes;c__Bacilli;o__;f__
k__Bacteria;p__Firmicutes;c__Bacilli;o__Bacillales;f__
k__Bacteria;p__Firmicutes;c__Bacilli;o__Bacillales;f__Alicyclobacillaceae
k__Bacteria;p__Firmicutes;c__Bacilli;o__Bacillales;f__Bacillaceae
k__Bacteria;p__Firmicutes;c__Bacilli;o__Bacillales;f__Listeriaceae
k__Bacteria;p__Firmicutes;c__Bacilli;o__Bacillales;f__Paenibacillaceae
k__Bacteria;p__Firmicutes;c__Bacilli;o__Bacillales;f__Planococcaceae
k__Bacteria;p__Firmicutes;c__Bacilli;o__Bacillales;f__Staphylococcaceae
k__Bacteria;p__Firmicutes;c__Bacilli;o__Lactobacillales;f__
k__Bacteria;p__Firmicutes;c__Bacilli;o__Lactobacillales;f__Aerococcaceae
k__Bacteria;p__Firmicutes;c__Bacilli;o__Lactobacillales;f__Carnobacteriaceae
k__Bacteria;p__Firmicutes;c__Bacilli;o__Lactobacillales;f__Enterococcaceae
k__Bacteria;p__Firmicutes;c__Bacilli;o__Lactobacillales;f__Lactobacillaceae
k__Bacteria;p__Firmicutes;c__Bacilli;o__Lactobacillales;f__Leuconostocaceae
k__Bacteria;p__Firmicutes;c__Bacilli;o__Lactobacillales;f__Streptococcaceae
k__Bacteria;p__Firmicutes;c__Clostridia;o__Clostridiales;f__
k__Bacteria;p__Firmicutes;c__Clostridia;o__Clostridiales;f__Catabacteriaceae
k__Bacteria;p__Firmicutes;c__Clostridia;o__Clostridiales;f__Clostridiaceae
k__Bacteria;p__Firmicutes;c__Clostridia;o__Clostridiales;f__Clostridiales Family XI. Incertae Sedis
k__Bacteria;p__Firmicutes;c__Clostridia;o__Clostridiales;f__Clostridiales Family XIII. Incertae Sedis
k__Bacteria;p__Firmicutes;c__Clostridia;o__Clostridiales;f__Eubacteriaceae
k__Bacteria;p__Firmicutes;c__Clostridia;o__Clostridiales;f__Lachnospiraceae
k__Bacteria;p__Firmicutes;c__Clostridia;o__Clostridiales;f__Peptococcaceae
k__Bacteria;p__Firmicutes;c__Clostridia;o__Clostridiales;f__Peptostreptococcaceae
k__Bacteria;p__Firmicutes;c__Clostridia;o__Clostridiales;f__Ruminococcaceae
k__Bacteria;p__Firmicutes;c__Clostridia;o__Clostridiales;f__Veillonellaceae
k__Bacteria;p__Firmicutes;c__Clostridia;o__MBA08;f__
k__Bacteria;p__Fusobacteria;c__Fusobacteria (class);o__Fusobacteriales;f__Fusobacteriaceae
k__Bacteria;p__Gemmatimonadetes;c__Gemmatimonadetes (class);o__Gemmatimonadales;f__
k__Bacteria;p__Proteobacteria;c__Alphaproteobacteria;o__Caulobacterales;f__Caulobacteraceae
k__Bacteria;p__Proteobacteria;c__Alphaproteobacteria;o__Rhizobiales;f__
k__Bacteria;p__Proteobacteria;c__Alphaproteobacteria;o__Rhizobiales;f__Bradyrhizobiaceae
k__Bacteria;p__Proteobacteria;c__Alphaproteobacteria;o__Rhizobiales;f__Brucellaceae
k__Bacteria;p__Proteobacteria;c__Alphaproteobacteria;o__Rhizobiales;f__Hyphomicrobiaceae
k__Bacteria;p__Proteobacteria;c__Alphaproteobacteria;o__Rhizobiales;f__Methylobacteriaceae
k__Bacteria;p__Proteobacteria;c__Alphaproteobacteria;o__Rhizobiales;f__Phyllobacteriaceae
k__Bacteria;p__Proteobacteria;c__Alphaproteobacteria;o__Rhizobiales;f__Rhizobiaceae
k__Bacteria;p__Proteobacteria;c__Alphaproteobacteria;o__Rhizobiales;f__Xanthobacteraceae
k__Bacteria;p__Proteobacteria;c__Alphaproteobacteria;o__Rhodobacterales;f__Rhodobacteraceae
k__Bacteria;p__Proteobacteria;c__Alphaproteobacteria;o__Rhodospirillales;f__Acetobacteraceae
k__Bacteria;p__Proteobacteria;c__Alphaproteobacteria;o__Rhodospirillales;f__Rhodospirillaceae
k__Bacteria;p__Proteobacteria;c__Alphaproteobacteria;o__Sphingomonadales;f__
k__Bacteria;p__Proteobacteria;c__Alphaproteobacteria;o__Sphingomonadales;f__Erythrobacteraceae
k__Bacteria;p__Proteobacteria;c__Alphaproteobacteria;o__Sphingomonadales;f__Sphingomonadaceae
k__Bacteria;p__Proteobacteria;c__Betaproteobacteria;o__;f__
k__Bacteria;p__Proteobacteria;c__Betaproteobacteria;o__Burkholderiales;f__
k__Bacteria;p__Proteobacteria;c__Betaproteobacteria;o__Burkholderiales;f__Alcaligenaceae
k__Bacteria;p__Proteobacteria;c__Betaproteobacteria;o__Burkholderiales;f__Burkholderiaceae
k__Bacteria;p__Proteobacteria;c__Betaproteobacteria;o__Burkholderiales;f__Comamonadaceae
k__Bacteria;p__Proteobacteria;c__Betaproteobacteria;o__Burkholderiales;f__Oxalobacteraceae
k__Bacteria;p__Proteobacteria;c__Betaproteobacteria;o__Hydrogenophilales;f__Hydrogenophilaceae
k__Bacteria;p__Proteobacteria;c__Betaproteobacteria;o__Methylophilales;f__Methylophilaceae
k__Bacteria;p__Proteobacteria;c__Betaproteobacteria;o__Neisseriales;f__Neisseriaceae
k__Bacteria;p__Proteobacteria;c__Betaproteobacteria;o__Rhodocyclales;f__
k__Bacteria;p__Proteobacteria;c__Betaproteobacteria;o__Rhodocyclales;f__Rhodocyclaceae
k__Bacteria;p__Proteobacteria;c__Deltaproteobacteria;o__CTD005-82B-02;f__
k__Bacteria;p__Proteobacteria;c__Deltaproteobacteria;o__Desulfobacterales;f__Desulfobulbaceae
k__Bacteria;p__Proteobacteria;c__Deltaproteobacteria;o__Desulfovibrionales;f__Desulfovibrionaceae
k__Bacteria;p__Proteobacteria;c__Deltaproteobacteria;o__MIZ46;f__
k__Bacteria;p__Proteobacteria;c__Deltaproteobacteria;o__Myxococcales;f__
k__Bacteria;p__Proteobacteria;c__Deltaproteobacteria;o__Myxococcales;f__Cystobacteraceae
k__Bacteria;p__Proteobacteria;c__Deltaproteobacteria;o__Myxococcales;f__Polyangiaceae
k__Bacteria;p__Proteobacteria;c__Deltaproteobacteria;o__Syntrophobacterales;f__Syntrophaceae
k__Bacteria;p__Proteobacteria;c__Epsilonproteobacteria;o__Campylobacterales;f__Campylobacteraceae
k__Bacteria;p__Proteobacteria;c__Gammaproteobacteria;o__;f__
k__Bacteria;p__Proteobacteria;c__Gammaproteobacteria;o__Aeromonadales;f__Aeromonadaceae
k__Bacteria;p__Proteobacteria;c__Gammaproteobacteria;o__Alteromonadales;f__Chromatiaceae
k__Bacteria;p__Proteobacteria;c__Gammaproteobacteria;o__Alteromonadales;f__Moritellaceae
k__Bacteria;p__Proteobacteria;c__Gammaproteobacteria;o__Alteromonadales;f__Psychromonadaceae
k__Bacteria;p__Proteobacteria;c__Gammaproteobacteria;o__Cardiobacteriales;f__Cardiobacteriaceae
k__Bacteria;p__Proteobacteria;c__Gammaproteobacteria;o__Chromatiales;f__
k__Bacteria;p__Proteobacteria;c__Gammaproteobacteria;o__Enterobacteriales;f__Enterobacteriaceae
k__Bacteria;p__Proteobacteria;c__Gammaproteobacteria;o__Oceanospirillales;f__
k__Bacteria;p__Proteobacteria;c__Gammaproteobacteria;o__Oceanospirillales;f__Alteromonadaceae
k__Bacteria;p__Proteobacteria;c__Gammaproteobacteria;o__Oceanospirillales;f__Halomonadaceae
k__Bacteria;p__Proteobacteria;c__Gammaproteobacteria;o__Oceanospirillales;f__Pseudomonadaceae
k__Bacteria;p__Proteobacteria;c__Gammaproteobacteria;o__Pasteurellales;f__Pasteurellaceae
k__Bacteria;p__Proteobacteria;c__Gammaproteobacteria;o__Pseudomonadales;f__Moraxellaceae
k__Bacteria;p__Proteobacteria;c__Gammaproteobacteria;o__Vibrionales;f__Vibrionaceae
k__Bacteria;p__Proteobacteria;c__Gammaproteobacteria;o__Xanthomonadales;f__Xanthomonadaceae
k__Bacteria;p__SPAM;c__;o__;f__
k__Bacteria;p__SR1;c__;o__;f__
k__Bacteria;p__Spirochaetes;c__Spirochaetes (class);o__Spirochaetales;f__Spirochaetaceae
k__Bacteria;p__Synergistetes;c__Synergistia;o__Synergistales;f__Dethiosulfovibrionaceae
k__Bacteria;p__TM7;c__TM7-3;o__CW040;f__
k__Bacteria;p__TM7;c__TM7-3;o__EW055;f__
k__Bacteria;p__Tenericutes;c__Erysipelotrichi;o__Erysipelotrichales;f__Erysipelotrichaceae
k__Bacteria;p__Tenericutes;c__Mollicutes;o__Mycoplasmatales;f__Mycoplasmataceae
k__Bacteria;p__Thermi;c__Deinococci;o__Deinococcales;f__Deinococcaceae
k__Bacteria;p__Thermi;c__Deinococci;o__Thermales;f__Thermaceae
k__Bacteria;p__Verrucomicrobia;c__Spartobacteria;o__;f__
k__Bacteria;p__Verrucomicrobia;c__Verrucomicrobiae;o__Verrucomicrobiales;f__Verrucomicrobiaceae
