# Supplementary material for: Moving pictures of the human microbiome
Source: Genome Biol. 2011 May 30;12(5):R50. doi: 10.1186/gb-2011-12-5-r50 (PMC3271711; doi:10.1186/gb-2011-12-5-r50)
Supplement: Additional file 12 — Temporal variation in phylum, class, order, family, and genus abundances (M3 left palm). The x-axis scale differs between M3 and F4 plots. [file gb-2011-12-5-r50-S12.ZIP › AdditionalFile12/charts/RBYbJ0dHI0jnY8GAaYipSfYh9yz6ZU_legend.pdf]

k__Archaea;p__Crenarchaeota;c__Sd-NA
k__Archaea;p__Crenarchaeota;c__Thaumarchaeota
k__Archaea;p__Euryarchaeota;c__Halobacteria
k__Archaea;p__Euryarchaeota;c__Methanobacteria
k__Archaea;p__Euryarchaeota;c__Methanomicrobia
k__Archaea;p__Euryarchaeota;c__Thermoplasmata
k__Bacteria;p__;c__
k__Bacteria;p__ABY1_OD1;c__
k__Bacteria;p__AD3;c__ABS-6
k__Bacteria;p__AD3;c__JG37-AG-4
k__Bacteria;p__Acidobacteria;c__
k__Bacteria;p__Acidobacteria;c__Acidobacteria (class)
k__Bacteria;p__Acidobacteria;c__Acidobacteria-5
k__Bacteria;p__Acidobacteria;c__Chloracidobacteria
k__Bacteria;p__Acidobacteria;c__Holophagae
k__Bacteria;p__Acidobacteria;c__MVS-40
k__Bacteria;p__Acidobacteria;c__OS-K
k__Bacteria;p__Acidobacteria;c__PAUC37f
k__Bacteria;p__Acidobacteria;c__RB25
k__Bacteria;p__Acidobacteria;c__Solibacteres
k__Bacteria;p__Acidobacteria;c__Sva0725
k__Bacteria;p__Acidobacteria;c__iii1-8
k__Bacteria;p__Actinobacteria;c__
k__Bacteria;p__Actinobacteria;c__Actinobacteria (class)
k__Bacteria;p__Aquificae;c__Aquificae (class)
k__Bacteria;p__BRC1;c__
k__Bacteria;p__BRC1;c__PRR-11
k__Bacteria;p__Bacteroidetes;c__
k__Bacteria;p__Bacteroidetes;c__Bacteroidia
k__Bacteria;p__Bacteroidetes;c__Flavobacteria
k__Bacteria;p__Bacteroidetes;c__Sphingobacteria
k__Bacteria;p__CCM11b;c__
k__Bacteria;p__Caldithrix_KSB1;c__Caldithrixae
k__Bacteria;p__Chlamydiae;c__Chlamydiae (class)
k__Bacteria;p__Chlorobi;c__
k__Bacteria;p__Chlorobi;c__BSV19
k__Bacteria;p__Chlorobi;c__OPB56
k__Bacteria;p__Chlorobi;c__SJA-28
k__Bacteria;p__Chlorobi;c__ZB1
k__Bacteria;p__Chloroflexi;c__
k__Bacteria;p__Chloroflexi;c__Anaerolineae
k__Bacteria;p__Chloroflexi;c__Bljii12
k__Bacteria;p__Chloroflexi;c__Chloroflexi (class)
k__Bacteria;p__Chloroflexi;c__Chloroflexi-4
k__Bacteria;p__Chloroflexi;c__Ktedonobacteria
k__Bacteria;p__Chloroflexi;c__SOGA31
k__Bacteria;p__Chloroflexi;c__TK17
k__Bacteria;p__Chloroflexi;c__Thermobacula
k__Bacteria;p__Chloroflexi;c__Thermomicrobia
k__Bacteria;p__Cyanobacteria;c__
k__Bacteria;p__Cyanobacteria;c__S15B-MN24
k__Bacteria;p__Cyanobacteria;c__SM1D11
k__Bacteria;p__Cyanobacteria;c__YS2
k__Bacteria;p__Cyanobacteria;c__mle1-12
k__Bacteria;p__Deferribacteres;c__Deferribacteres (class)
k__Bacteria;p__Elusimicrobia;c__Elusimicrobia (class)
k__Bacteria;p__Fibrobacteres;c__Fibrobacteres (class)
k__Bacteria;p__Firmicutes;c__Bacilli
k__Bacteria;p__Firmicutes;c__Clostridia
k__Bacteria;p__Fusobacteria;c__Fusobacteria (class)
k__Bacteria;p__GN02;c__
k__Bacteria;p__GN02;c__VC12-cl04
k__Bacteria;p__Gemmatimonadetes;c__Gemmatimonadetes (class)
k__Bacteria;p__Lentisphaerae;c__
k__Bacteria;p__Lentisphaerae;c__Lentisphaerae (class)
k__Bacteria;p__MVP-15;c__
k__Bacteria;p__NC10;c__
k__Bacteria;p__NKB19;c__
k__Bacteria;p__Nitrospirae;c__Nitrospira (class)
k__Bacteria;p__OP10;c__
k__Bacteria;p__OP10;c__5B-18
k__Bacteria;p__OP10;c__CH21
k__Bacteria;p__OP10;c__CL500-48
k__Bacteria;p__OP10;c__OS-L
k__Bacteria;p__OP10;c__S1a-1H
k__Bacteria;p__OP10;c__SJA-176
k__Bacteria;p__OP10;c__SJA-22
k__Bacteria;p__OP11;c__
k__Bacteria;p__OP3;c__
k__Bacteria;p__OP8;c__OP8
k__Bacteria;p__Planctomycetes;c__FFCH393
k__Bacteria;p__Planctomycetes;c__Kueneniae
k__Bacteria;p__Planctomycetes;c__PW285
k__Bacteria;p__Planctomycetes;c__Phycisphaerae
k__Bacteria;p__Planctomycetes;c__Planctomycea
k__Bacteria;p__Planctomycetes;c__agg27
k__Bacteria;p__Planctomycetes;c__vadinHA49
k__Bacteria;p__Proteobacteria;c__Alphaproteobacteria
k__Bacteria;p__Proteobacteria;c__Betaproteobacteria
k__Bacteria;p__Proteobacteria;c__Deltaproteobacteria
k__Bacteria;p__Proteobacteria;c__Epsilonproteobacteria
k__Bacteria;p__Proteobacteria;c__Gammaproteobacteria
k__Bacteria;p__SC3;c__
k__Bacteria;p__SC4;c__
k__Bacteria;p__SPAM;c__
k__Bacteria;p__SR1;c__
k__Bacteria;p__Spirochaetes;c__Brachyspirae
k__Bacteria;p__Spirochaetes;c__Leptospirae
k__Bacteria;p__Spirochaetes;c__SP_WWE1
k__Bacteria;p__Spirochaetes;c__Spirochaetes (class)
k__Bacteria;p__Synergistetes;c__Synergistia
k__Bacteria;p__TM6;c__
k__Bacteria;p__TM7;c__
k__Bacteria;p__TM7;c__TM7-1
k__Bacteria;p__TM7;c__TM7-3
k__Bacteria;p__Tenericutes;c__
k__Bacteria;p__Tenericutes;c__Erysipelotrichi
k__Bacteria;p__Tenericutes;c__ML615J-28
k__Bacteria;p__Tenericutes;c__Mollicutes
k__Bacteria;p__Thermi;c__Deinococci
k__Bacteria;p__Thermotogae;c__Thermotogae (class)
k__Bacteria;p__Verrucomicrobia;c__
k__Bacteria;p__Verrucomicrobia;c__Opitutae
k__Bacteria;p__Verrucomicrobia;c__Spartobacteria
k__Bacteria;p__Verrucomicrobia;c__TP21
k__Bacteria;p__Verrucomicrobia;c__Verrucomicrobiae
k__Bacteria;p__WPS-2;c__
k__Bacteria;p__WS3;c__PRR-12
k__Bacteria;p__ZB2;c__
