# Supplementary material for: Moving pictures of the human microbiome
Source: Genome Biol. 2011 May 30;12(5):R50. doi: 10.1186/gb-2011-12-5-r50 (PMC3271711; doi:10.1186/gb-2011-12-5-r50)
Supplement: Additional file 12 — Temporal variation in phylum, class, order, family, and genus abundances (M3 left palm). The x-axis scale differs between M3 and F4 plots. [file gb-2011-12-5-r50-S12.ZIP › AdditionalFile12/index.html]

 
 
 
 

 
 Taxa Summaries 
 
 
  
 
     
  Taxonomy Summary. Current Level: Phylum  
     View Figure (.pdf)    View Legend (.pdf)   
   
 
     
 
 

 
 
 
 
 
 
 
 
 
 
 
 
 
 
 
 
 
 
 
 
 
 
 
 
 
 
 
 
 
 
 
 
 
 
 
 
 
 
 
 
 
 
 
 
 
 
 
 
 
 
 
 
 
 
 
 
 
 
 
 
 
 
 
 
 
 
 
 
 
 
 
 
 
 
 
 
 
 
 
 
 
 
 
 
 
 
 
 
 
 
 
 
 
 
 
 
 
 
 
 
 
 
 
 
 
 
 
 
 
 
 
 
 
 
 
 
 
 
 
 
 
 
 
 
 
 
 
 
 
 
 
 
 
 
 
 
 
 
 
 
 
 
 
 
 
 
 
 
 
 
 
 
 
 
 
 
 
 
 
 
 
 
 
 
 
 
 
 
 
 
 
 
 
 
 
 
 
 
 
 
 
 
 
 
 
 
 
 
 
 
 
 
 
 
 
 
 
 
 
 
 
 
 
 
 
 
 
 
 
 
 
 
 
 
 
 
 
 
 
 
 
 
 
 
 
 
 
 
 
 
 
 
 
 
 
 
 
 
 
 
 
 
 
 
 
 
 
 
 
 
 
 
 
 
 
 
 
 
 
 
 
 
 
 
 
 
 
 
 
 
 
 
 
 
 
 
 
 
 
 
 
 
 
 
 
 
 
 
 
 
 
 
 
 
 
 
 
 
 
 
 
 
 
 
 
 
 
 
 
 
 
 
 
 
 
 
 
 
 
 
 
 
 
 
 
 
 
 
 
 
 
 
 
 
 
 
 
 
 
 
 
 
 
 
 
 
 
 
 
 
 
 
 
 
 
 
 
 
 
 
 
 
 
 
 
 
 
 
 
 
 
 
 
 
 
 
 
 
 
 
 
 
 
 
 
 
 
 
 
 
 
 
 
 
 
 
 
 
 
 
 
 
 
 
 
 
 
 
 
 
 
 
 
 
 
 
 
 
 
 
 
 
 
 
 
 
 
 
 
 
 
 
 
 
 
 
 
 
 
 
 
 
 
 
 
 
 
 
 
 
 
 
 
 
 
 
 
 
 
 
 
 
 
 
 
 
 
 
 
 
 
 
 
 
 
 
 
 
 
 
 
 
 
 
 
 
 
 
 
 
 
 
 
 
 
 
 
 
 
 
 
 
 
 
 
 
 
 
 
 
 
 
 
 
 
 
 
 
 
 
 
 
 
 
 
 
 
 
 
 
 
 
 
 
 
 
 
 
 
 
 
 
 
 
 
 
 
 
 
 
 
 
 
 
 
 
 
 
 
 
 
 
 
 
 
 
 
 
 
 
 
 
 
 
 
 
 
 
 
 
 
 
 
 
 
 
 
 
 
 
 
 
 
 
 
 
 
 
 
 
 
 
 
 
 
 
 
 
 
 
 
 
 
 
 
 
 
 
 
 
 
 
 
 
 
 
 
 
 
 
 
 
 
 
 
 
 
 
 
 
 
 
 
 
 
 
 
 
 
 
 
 
 
 
 
 
 
 
 
 
 
 
 
 
 
 
 
 
 
 
 
 
 
 
 
 
 
 
 
 
 
 
 
 
 
 
 
 
 
 
 
 
 
 
 
 
 
 
 
 
 
 
 
 
 
 
 
 
 
 
 
 
 
 
 
 
 
 
 
 
 
 
 
 
 
 
 
 
 
 
 
 
 
 
 
 
 
 
 
 
 
 
 
 
 
 
 
 
 
 
 
 
 
 
 
 
 
 
 
 
 
 
 
 
 
 
 
 
 
 
 
 
 
 
 
 
 
 
 
 
 
 
 
 
 
 
 
 
 
 
 
 
 
 
 
 
 
 
 
 
 
 
 
 
 
 
 
 
 
 
 
 
 
 
 
 
 
 
 
 
 
 
 
 
 
 
 
 
 
 
 
 
 
 
 
 
 
 
 
 
 
 
 
 
 
 
 
 
 
 
 
 
 
 
 
 
 
 
 
 
 
 
 
 
 
 
 
 
 
 
 
 
 
 
 
 
 
 
 
 
 
 
 
 
 
 
 
 
 
 
 
 
 
 
 
 
 
 
 
 
 
 
 
 
 
 
 
 
 
 
 
 
 
 
 
 
 
 
 
 
 
 
 
 
 
 
 
 
 
 
 
 
 
 
 
 
 
 
 
 
 
 
 
 
 
 
 
 
 
 
 
 
 
 
 
 
 
 
 
 
 
 
 
 
 
 
 
 
 
 
 
 
 
 
 
 
 
 
 
 
 
 
 
 
 
 
 
 
 
 
 
 
 
 
 
 
 
 
 
 
 
 
 
 
 
 
 
 
 
 
 
 
 
 
 
 
 
 
 
 
 
 
 
 
 
 
 
 
 
 
 
 
 
 
 
 
 
 
 
 
 
 
 
 
 
 
 
 
 
 
 
 
 
 
 
 
 
 
 
 
 
 
 
 
 
 
 
 
 
 
 
 
 
 
 
 
 
 
 
 
 
 
 
 
 
 
 
 
 
 
 
 
 
 
 
 
 
 
 
 
 
 
 
 
 
 
 
 
 
 
 
 
 
 
 
 
 
 
 
 
 
 
 
 
 
 
 
 
 
 
 
 
 
 
 
 
 
 
 
 
 
 
 
 
 
 
 
 
 
 
 
 
 
 
 
 
 
 
 
 
 
 
 
 
 
 
 
 
 
 
 
 
 
 
 
 
 
 
 
 
 
 
 
 
 
 
 
 
 
 
 
 
 
 
 
 
 
 
 
 
 
 
 
 
 
 
 
 
 
 
 
 
 
 
 
 
 
 
 
 
 
 
 
 
 
 
 
 
 
 
 
 
 
 
 
 
 
 
 
 
 
 
 
 
 
 
 
 
 
 
 
 
 
 
 
 
 
 
 
 
 
 
 
 
 
 
 
 
 
 
 
 
 
 
 
 
 
 
 
 
 
 
 
 
 
 
 
 
 
 
 
 
 
 
 
 
 
 
 
 
 
 
 
 
 
 
 
 
 
 
 
 
 
 
 
 
 
 
 
 
 
 
 
 
 
 
 
 
 
 
 
 
 
 
 
 
 
 
 
 
 
 
 
 
 
 
 
 
 
 
 
 
 
 
 
 
 
 
 
 
 
 
 
 
 
 
 
 
 
 
 
 
 
 
 
 
 
 
 
 
 
 
 
 
 
 
 
 
 
 
 
 
 
 
 
 
 
 
 
 
 
 
 
 
 
 
 
 
 
 
 
 
 
 
 
 
 
 
 
 
 
 
 
 
 
 
 
 
 
 
 
 
 
 
 
 
 
 
 
 
 
 
 
 
 
 
 
 
 
 
 
 
 
 
 
 
 
 
 
 
 
 
 
 
 
 
 
 
 
 
 
 
 
 
 
 
 
 
 
 
 
 
 
 
 
 
 
 
 
 
 
 
 
 
 
 
 
 
 
 
 
 
 
 
 
 
 
 
 
 
 
 
 
 
 
 
 
 
 
 
 
 
 
 
 
 
 
 
 
 
 
 
 
 
 
 
 
 
 
 
 
 
 
 
 
 
 
 
 
 
 
 
 
 
 
 
 
 
 
 
 
 
 
 
 
 
 
 
 
 
 
 
 
 
 
 
 
 
 
 
 
 
 
 
 
 
 
 
 
 
 
 
 
 
 
 
 
 
 
 
 
 
 
 
 
 
 
 
 
 
 
 
 
 
 
 
 
 
 
 
 
 
 
 
 
 
 
 
 
 
 
 
 
 
 
 
 
 
 
 
 
 
 
 
 
 
 
 
 
 
 
 
 
 
 
 
 
 
 
 
 
 
 
 
 
 
 
 
 
 
 
 
 
 
 
 
 
 
 
 
 
 
 
 
 
 
 
 
 
 
 
 
 
 
 
 
 
 
 
 
 
 
 
 
 
 
 
 
 
 
 
 
 
 
 
 
 
 
 
 
 
 
 
 
 
 
 
 
 
 
 
 
 
 
 
 
 
 
 
 
 
 
 
 
 
 
 
 
 
 
 
 
 
 
 
 
 
 
 
 
 
 
 
 
 
 
 
 
 
 
 
 
 
 
 
 
 
 
 
 
 
 
 
 
 
 
 
 
 
 
 
 
 
 
 
 
 
 
 
 
 
 
 
 
 
 
 
 
 
 
 
 
 
 
 
 
 
 
 
 
 
 
 
 
 
 
 
 
 
 
 
 
 
 
 
 
 
 
 
 
 
 
 
 
 
 
 
 
 
 
 
 
 
 
 
 
 
 
 
 
 
 
 
 
 
 
 
 
 
 
 
 
 
 
 
 
 
 
 
 
 
 
 
 
 
 
 
 
 
 
 
 
 
 
 
 
 
 
 
 
 
 
 
 
 
 
 
 
 
 
 
 
 
 
 
 
 
 
 
 
 
 
 
 
 
 
 
 
 
 
 
 
 
 
 
 
 
 
 
 
 
 
 
 
 
 
 
 
 
 
 
 
 
 
 
 
 
 
 
 
 
 
 
 
 
 
 
 
 
 
 
 
 
 
 
 
 
 
 
 
 
 
 
 
 
 
 
 
 
 
 
 
 
 
 
 
 
 
 
 
 
 
 
 
 
 
 
 
 
 
 
 
 
 
 
 
 
 
 
 
 
 
 
 
 
 
 
 
 
 
 
 
 
 
 
 
 
 
 
 
 
 
 
 
 
 
 
 
 
 
 
 
 
 
 
 
 
 
 
 
 
 
 
 
 
 
 
 
 
 
 
 
 
 
 
 
 
 
 
 
 
 
 
 
 
 
 
 
 
 
 
 
 
 
 
 
 
 
 
 
 
 
 
 
 
 
 
 
 
 
 
 
 
 
 
 
 
 
 
 
 
 
 
 
 
 
 
 
 
 
 
 
 
 
 
 
 
 
 
 
 
 
 
 
 
 
 
 
 
 
 
 
 
 
 
 
 
 
 
 
 
 
 
 
 
 
 
 
 
 
 
 
 
 
 
 
 
 
 
 
 
 
 
 
 
 
 
 
 
 
 
 
 
 
 
 
 
 
 
 
 
 
 
 
 
 
 
 
 
 
 
 
 
 
 
 
 
 
 
 
 
 
 
 
 
 
 
 
 
 
 
 
 
 
 
 
 
 
 
 
 
 
 
 
 
 
 
 
 
 
 
 
 
 
 
 
 
 
 
 
 
 
 
 
 
 
 
 
 
 
 
 
 
 
 
 
 
 
 
 
 
 
 
 
 
 
 
 
 
 
 
 
 
 
 
 
 
 
 
 
 
 
 
 
 
 
 
 
 
 
 
 
 
 
 
 
 
 
 
 
 
 
 
 
 
 
 
 
 
 
 
 
 
 
 
 
 
 
 
 
 
 
 
 
 
 
 
 
 
 
 
 
 
 
 
 
 
 
 
 
 
 
 
 
 
 
 
 
 
 
 
 
 
 
 
 
 
 
 
 
 
 
 
 
 
 
 
 
 
 
 
 
 
 
 
 
 
 
 
 
 
 
 
 
 
 
 
 
 
 
 
 
 
 
 
 
 
 
 
 
 
 
 
 
 
 
 
 
 
 
 
 
 
 
 
 
 
 
 
 
 
 
 
 
 
 
 
 
 
 
 
 
 
 
 
 
 
 
 
 
 
 
 
 
 
 
 
 
 
 
 
 
 
 
 
 
 
 
 
 
 
 
 
 
 
 
 
 
 
 
 
 
 
 
 
 
 
 
 
 
 
 
 
 
 
 
 
 
 
 
 
 
 
 
 
 
 
 
 
 
 
 
 
 
 
 
 
 
 
 
 
 
 
 
 
 
 
 
 
 
 
 
 
 
 
 
 
 
 
 
 
 
 
 
 
 
 
 
 
 
 
 
 
 
 
 
 
 
 
 
 
 
 
 
 
 
 
 
 
 
 
 
 
 
 
 
 
 
 
 
 
 
 
 
 
 
 
 
 
 
 
 
 
 
 
 
 
 
 
 
 
 
 
 
 
 
 
 
 
 
 
 
 
 
 
 
 
 
 
 
 
 
 
 
 
 
 
 
 
 
 
 
 
 
 
 
 
 
 
 
 
 
 
 
 
 
 
 
 
 
 
 
 
 
 
 
 
 
 
 
 
 
 
 
 
 
 
 
 
 
 
 
 
 
 
 
 
 
 
 
 
 
 
 
 
 
 
 
 
 
 
 
 
 
 
 
 
 
 
 
 
 
 
 
 
 
 
 
 
 
 
 
 
 
 
 
 
 
 
 
 
 
 
 
 
 
 
 
 
 
 
 
 
 
 
 
 
 
 
 
 
 
 
 
 
 
 
 
 
 
 
 
 
 
 
 
 
 
 
 
 
 
 
 
 
 
 
 
 
 
 
 
 
 
 
 
 
 
 
 
 
 
 
 
 
 
 
 
 
 
 
 
 
 
 
 
 
 
 
 
 
 
 
 
 
 
 
 
 
 
 
 
 
 
 
 
 
 
 
 
 
 
 
 
 
 
 
 
 
 
 
 
 
 
 
 
 
 
 
 
 
 
 
 
 
 
 
 
 
 
 
 
 
 
 
 
 
 
 
 
 
 
 
 
 
 
 
 
 
 
 
 
 
 
 
 
 
 
 
 
 
 
 
 
 
 
 
 
 
 
 
 
 
 
 
 
 
 
 
 
 
 
 
 
 
 
 
 
 
 
 
 
 
 
 
 
 
 
 
 
 
 
 
 
 
 
 
 
 
 
 
 
 
 
 
 
 
 
 
 
 
 
 
 
 
 
 
 
 
 
 
 
 
 
 
 
 
 
 
 
 
 
 
 
 
 
 
 
 
 
 
 
 
 
 
 
 
 
 
 
 
 
 
 
 
 
 
 
 
 
 
 
 
 
 
 
 
 
 
 
 
 
 
 
 
 
 
 
 
 
 
 
 
 
 
 
 
 
 
 
 
 
 
 
 
 
 
 
 
 
 
 
 
 
 
 
 
 
 
 
 
 
 
 
 
 
 
 
 
 
 
 
 
 
 
 
 
 
 
 
 
 
 
 
 
 
 
 
 
 
 
 
 
 
 
 
 
 
 
 
 
 
 
 
 
 
 
 
 
 
 
 
 
 
 
 
 
 
 
 
 
 
 
 
 
 
 
 
 
 
 
 
 
 
 
 
 
 
 
 
 
 
 
 
 
 
 
 
 
 
 
 
 
 
 
 
 
 
 
 
 
 
 
 
 
 
 
 
 
 
 
 
 
 
 
 
 
 
 
 
 
 
 
 
 
 
 
 
 
 
 
 
 
 
 
 
 
 
 
 
 
 
 
 
 
 
 
 
 
 
 
 
 
 
 
 
 
 
 
 
 
 
 
 
 
 
 
 
 
 
 
 
 
 
 
 
 
 
 
 
 
 
 
 
 
 
 
 
 
 
 
 
 
 
 
 
 
 
 
 
 
 
 
 
 
 
 
 
 
 
 
 
 
 
 
 
 
 
 
 
 
 
 
 
 
 
 
 
 
 
 
 
 
 
 
 
 
 
 
 
 
 
 
 
 
 
 
 
 
 
 
 
 
 
 
 
 
 
 
 
 
 
 
 
 
 
 
 
 
 
 
 
 
 
 
 
 
 
 
 
 
 
 
 
 
 
 
 
 
 
 
 
 
 
 
 
 
 
 
 
 
 
 
 
 
 
 
 
 
 
 
 
 
 
 
 
 
 
 
 
 
 
 
 
 
 
 
 
 
 
 
 
 
 
 
 
 
 
 
 
 
 
 
 
 
 
 
 
 
 
 
 
 
 
 
 
 
 
 
 
 
 
 
 
 
 
 
 
 
 
 
 
 
 
 
 
 
 
 
 
 
 
 
 
 
 
 
 
 
 
 
 
 
 
 
 
 
 
 
 
 
 
 
 
 
 
 
 
 
 
 
 
 
 
 
 
 
 
 
 
 
 
 
 
 
 
 
 
 
 
 
 
 
 
 
 
 
 
 
 
 
 
 
 
 
 
 
 
 
 
 
 
 
 
 
 
 
 
 
 
 
 
 
 
 
 
 
 
 
 
 
 
 
 
 
 
 
 
 
 
 
 
 
 
 
 
 
 
 
 
 
 
 
 
 
 
 
 
 
 
 
 
 
 
 
 
 
 
 
 
 
 
 
 
 
 
 
 
 
 
 
 
 
 
 
 
 
 
 
 
 
 
 
 
 
 
 
 
 
 
 
 
 
 
 
 
 
 
 
 
 
 
 
 
 
 
 
 
 
 
 
 
 
 
 
 
 
 
 
 
 
 
 
 
 
 
 
 
 
 
 
 
 
 
 
 
 
 
 
 
 
 
 
 
 
 
 
 
 
 
 
 
 
 
 
 
 
 
 
 
 
 
 
 
 
 
 
 
 
 
 
 
 
 
 
 
 
 
 
 
 
 
 
 
 
 
 
 
 
 
 
 
 
 
 
 
 
 
 
 
 
 
 
 
 
 
 
 
 
 
 
 
 
 
 
 
 
 
 
 
 
 
 
 
 
 
 
 
 
 
 
 
 
 
 
 
 
 
 
 
 
 
 
 
 
 
 
 
 
 
 
 
 
 
 
 
 
 
 
 
 
 
 
 
 
 
 
 
 
 
 
 
 
 
 
 
 
 
 
 
 
 
 
 
 
 
 
 
 
 
 
 
 
 
 
 
 
 
 
 
 
 
 
 
 
 
 
 
 
 
 
 
 
 
 
 
 
 
 
 
 
 
 
 
 
 
 
 
 
 
 
 
 
 
 
 
 
 
 
 
 
 
 
 
 
 
 
 
 
 
 
 
 
 
 
 
 
 
 
 
 
 
 
 
 
 
 
 
 
 
 
 
 
 
 
 
 
 
 
 
 
 
 
 
 
 
 
 
 
 
 
 
 
 
 
 
 
 
 
 
 
 
 
 
 
 
 
 
 
 
 
 
 
 
 
 
 
 
 
 
 
 
 
 
 
 
 
 
 
 
 
 
 
 
 
 
 
 
 
 
 
 
 
 
 
 
 
 
 
 
 
 
 
 
 
 
 
 
 
 
 
 
 
 
 
 
 
 
 
 
 
 
 
 
 
 
 
 
 
 
 
 
 
 
 
 
 
 
 
 
 
 
 
 
 
 
 
 
 
 
 
 
 
 
 
 
 
 
 
 
 
 
 
 
 
 
 
 
 
 
 
 
 
 
 
 
 
 
 
 
 
 
 
 
 
 
 
 
 
 
 
 
 
 
 
 
 
 
 
 
 
 
 
 
 
 
 
 
 
 
 
 
 
 
 
 
 
 
 
 
 
 
 
 
 
 
 
 
 
 
 
 
 
 
 
 
 
 
 
 
 
 
 
 
 
 
 
 
 
 
 
 
 
 
 
 
 
 
 
 
 
 
 
 
 
 
 
 
 
 
 
 
 
 
 
 
 
 
 
 
 
 
 
 
 
 
 
 
 
 
 
 
 
 
 
 
 
 
 
 
 
 
 
 
 
 
 
 
 
 
 
 
 
 
 
 
 
 
 
 
 
 
 
 
 
 
 
 
 
 
 
 
 
 
 
 
 
 
 
 
 
 
 
 
 
 
 
 
 
 
 
 
 
 
 
 
 
 
 
 
 
 
 
 
 
 
 
 
 
 
 
 
 
 
 
 
 
 
 
 
 
 
 
 
 
 
 
 
 
 
 
 
 
 
 
 
 
 
 
 
 
 
 
 
 
 
 
 
 
 
 
 
 
 
 
 
 
 
 
 
 
 
 
 
 
 
 
 
 
 
 
 
 
 
 
 
 
 
 
 
 
 
 
 
 
 
 
 
 
 
 
 
 
 
 
 
 
 
 
 
 
 
 
 
 
 
 
 
 
 
 
 
 
 
 
 
 
 
 
 
 
 
 
 
 
 
 
 
 
 
 
 
 
 
 
 
 
 
 
 
 
 
 
 
 
 
 
 
 
 
 
 
 
 
 
 
 
 
 
 
 
 
 
 
 
 
 
 
 
 
 
 
 
 
 
 
 
 
 
 
 
 
 
 
 
 
 
 
 
 
 
 
 
 
 
 
 
 
 
 
 
 
 
 
 
 
 
 
 
 
 
 
 
 
 
 
 
 
 
 
 
 
 
 
 
 
 
 
 
 
 
 
 
 
 
 
 
 
 
 
 
 
 
 
 
 
 
 
 
 
 
 
 
 
 
 
 
 
 
 
 
 
 
 
 
 
 
 
 
 
 
 
 
 
 
 
 
 
 
 
 
 
 
 
 
 
 
 
 
 
 
 
 
 
 
 
 
 
 
 
 
 
 
 
 
 
 
 
 
 
 
 
 
 
 
 
 
 
 
 
 
 
 
 
 
 
 
 
 
 
 
 
 
 
 
 
 
 
 
 
 
 
 
 
 
 
 
 
 
 
 
 
 
 
 
 
 
 
 
 
 
 
 
 
 
 
 
 
 
 
 
 
 
 
 
 
 
 
 
 
 
 
 
 
 
 
 
 
 
 
 
 
 
 
 
 
 
 
 
 
 
 
 
 
 
 
 
 
 
 
 
 
 
 
 
 
 
 
 
 
 
 
 
 
 
 
 
 
 
 
 
 
 
 
 
 
 
 
 
 
 
 
 
 
 
 
 
 
 
 
 
 
 
 
 
 
 
 
 
 
 
 
 
 
 
 
 
 
 
 
 
 
 
 
 
 
 
 
 
 
 
 
 
 
 
 
 
 
 
 
 
 
 
 
 
 
 
 
 
 
 
 
 
 
 
 
 
 
 
 
 
 
 
 
 
 
 
 
 
 
 
 
 
 
 
 
 
 
 
 
 
 
 
 
 
 
 
 
 
 
 
 
 
 
 
 
 
 
 
 
 
 
 
 
 
 
 
 
 
 
 
 
 
 
 
 
 
 
 
 
 
 
 
 
 
 
 
 
 
 
 
 
 
 
 
 
 
 
 
 
 
 
 
 
 
 
 
 
 
 
 
 
 
 
 
 
 
 
 
 
 
 
 
 
 
 
 
 
 
 
 
 
 
 
 
 
 
 
 
 
 
 
 
 
 
 
 
 
 
 
 
 
 
 
 
 
 
 
 
 
 
 
 
 
 
 
 
 
 
 
 
 
 
 
 
 
 
 
 
 
 
 
 
 
 
 
 
 
 
 
 
 
 
 
 
 
 
 
 
 
 
 
 
 
 
 
 
 
 
 
 
 
 
 
 
 
 
 
 
 
 
 
 
 
 
 
 
 
 
 
 
 
 
 
 
 
 
 
 
 
 
 
 
 
 
 
 
 
 
 
 
 
 
 
 
 
 
 
 
 
 
 
 
 
 
 
 
 
 
 
 
 
 
 
 
 
 
 
 
 
 
 
 
 
 
 
 
 
 
 
 
 
 
 
 
 
 
 
 
 
 
 
 
 
 
 
 
 
 
 
 
 
 
 
 
 
 
 
 
 
 
 
 
 
 
 
 
 
 
 
 
 
 
 
 
 
 
 
 
 
 
 
 
 
 
 
 
 
 
 
 
 
 
 
 
 
 
 
 
 
 
 
 
 
 
 
 
 
 
 
 
 
 
 
 
 
 
 
 
 
 
 
 
 
 
 
 
 
 
 
 
 
 
 
 
 
 
 
 
 
 
 
 
 
 
 
 
 
 
 
 
 
 
 
 
 
 
 
 
 
 
 
 
 
 
 
 
 
 
 
 
 
 
 
 
 
 
 
 
 
 
 
 
 
 
 
 
 
 
 
 
 
 
 
 
 
 
 
 
 
 
 
 
 
 
 
 
 
 
 
 
 
 
 
 
 
 
 
 
 
 
 
 
 
 
 
 
 
 
 
 
 
 
 
 
 
 
 
 
 
 
 
 
 
 
 
 
 
 
 
 
 
 
 
 
 
 
 
 
 
 
 
 
 
 
 
 
 
 
 
 
 
 
 
 
 
 
 
 
 
 
 
 
 
 
 
 
 
 
 
 
 
 
 
 
 
 
 
 
 
 
 
 
 
 
 
 
 
 
 
 
 
 
 
 
 
 
 
 
 
 
 
 
 
 
 
 
 
 
 
 
 
 
 
 
 
 
 
 
 
 
 
 
 
 
 
 
 
 
 
 
 
 
 
 
 
 
 
 
 
 
 
 
 
 
 
 
 
 
 
 
 
 
 
 
 
 
 
 
 
 
 
 
 
 
 
 
 
 
 
 
 
 
 
 
 
 
 
 
 
 
 
 
 
 
 
 
 
 
 
 
 
 
 
 
 
 
 
 
 
 
 
 
 
 
 
 
 
 
 
 
 
 
 
 
 
 
 
 
 
 
 
 
 
 
 
 
 
 
 
 
 
 
 
 
 
 
 
 
 
 
 
 
 
 
 
 
 
 
 
 
 
 
 
 
 
 
 
 

 

    View Table (.txt)         Total  0  1  2  3  4  5  6  8  9  10  11  12  13  14  15  16  17  18  19  20  21  22  23  24  25  26  27  28  29  30  31  32  42  43  44  45  46  47  48  49  50  51  52  53  54  56  57  58  59  60  61  62  64  65  66  67  68  69  70  71  72  73  79  80  81  82  83  84  86  87  88  89  90  91  92  93  94  95  96  97  98  99  100  101  102  103  104  105  112  113  114  115  116  117  118  120  122  123  124  125  126  127  128  129  130  131  132  133  134  135  137  138  139  140  141  142  143  144  145  146  147  148  149  150  151  152  153  154  155  157  158  159  160  161  162  163  164  166  168  169  170  172  173  174  175  176  177  178  180  181  182  183  184  185  186  187  188  189  190  191  192  193  194  195  196  197  198  199  200  201  202  203  204  205  206  208  209  210  211  213  214  215  216  217  218  219  221  222  223  225  226  227  228  229  230  231  234  236  237  238  239  240  241  243  244  245  246  247  248  249  250  251  252  253  254  255  256  257  258  259  260  261  262  263  264  265  266  267  268  269  270  271  272  273  274  275  276  278  279  280  281  282  283  284  285  286  287  288  289  290  292  293  294  295  296  297  298  299  300  301  302  303  304  305  306  307  308  309  311  312  314  315  316  317  318  319  320  321  322  323  324  325  327  328  330  331  332  333  334  336  338  339  340  344  345  347  348  349  350  351  352  353  356  357  358  359  360  361  362  363  365  366  370  372  373  374  375  377  378  379  380  381  382  383  384  385  386  387  389  390  391  392  393  394  395  396  397  398  399  400  401  402  403  405  406  410  411  412  413  415  416  417  418  420  438  439  440  441  442  443  444    Legend  Taxonomy  count  %  %  %  %  %  %  %  %  %  %  %  %  %  %  %  %  %  %  %  %  %  %  %  %  %  %  %  %  %  %  %  %  %  %  %  %  %  %  %  %  %  %  %  %  %  %  %  %  %  %  %  %  %  %  %  %  %  %  %  %  %  %  %  %  %  %  %  %  %  %  %  %  %  %  %  %  %  %  %  %  %  %  %  %  %  %  %  %  %  %  %  %  %  %  %  %  %  %  %  %  %  %  %  %  %  %  %  %  %  %  %  %  %  %  %  %  %  %  %  %  %  %  %  %  %  %  %  %  %  %  %  %  %  %  %  %  %  %  %  %  %  %  %  %  %  %  %  %  %  %  %  %  %  %  %  %  %  %  %  %  %  %  %  %  %  %  %  %  %  %  %  %  %  %  %  %  %  %  %  %  %  %  %  %  %  %  %  %  %  %  %  %  %  %  %  %  %  %  %  %  %  %  %  %  %  %  %  %  %  %  %  %  %  %  %  %  %  %  %  %  %  %  %  %  %  %  %  %  %  %  %  %  %  %  %  %  %  %  %  %  %  %  %  %  %  %  %  %  %  %  %  %  %  %  %  %  %  %  %  %  %  %  %  %  %  %  %  %  %  %  %  %  %  %  %  %  %  %  %  %  %  %  %  %  %  %  %  %  %  %  %  %  %  %  %  %  %  %  %  %  %  %  %  %  %  %  %  %  %  %  %  %  %  %  %  %  %  %  %  %  %  %  %  %  %  %  %  %  %  %  %  %  %  %  %  %  %  %  %  %  %  %  %  %  %  %  %  %  %  %  %  %  %  %  %  %  %  %  %  %  %  %        k__Archaea; p__Crenarchaeota    1250    0.0%    0.0%    0.0%    0.0%    0.0%    0.0%    0.0%    0.0%    0.0%    0.0%    0.0%    0.0%    0.0%    0.0%    0.0%    0.0%    0.0%    0.0%    0.0%    0.0%    0.0%    0.0%    0.0%    0.0%    0.0%    0.0%    0.0%    0.0%    0.0%    0.0%    0.0%    0.0%    0.0%    0.0%    0.0%    0.0%    0.0%    0.0%    0.0%    0.0%    0.0%    0.0%    0.0%    0.0%    0.0%    0.0%    0.0%    0.0%    0.0%    0.0%    0.0%    0.0%    0.0%    0.0%    0.0%    0.0%    0.0%    0.0%    0.0%    0.0%    0.0%    0.0%    0.0%    0.1%    0.1%    0.0%    0.0%    0.0%    0.0%    0.0%    0.0%    0.0%    0.0%    0.0%    0.1%    0.0%    0.0%    0.1%    0.0%    0.0%    0.0%    0.0%    0.0%    0.0%    0.0%    0.0%    0.0%    0.0%    0.0%    0.0%    0.0%    0.0%    0.0%    0.0%    0.0%    0.0%    0.0%    0.0%    0.1%    0.1%    0.0%    0.0%    0.0%    0.0%    0.0%    0.1%    0.0%    0.0%    0.0%    0.0%    0.0%    0.1%    0.0%    0.0%    0.0%    0.0%    0.0%    0.0%    0.0%    0.1%    0.0%    0.0%    0.1%    0.1%    0.0%    0.1%    0.1%    0.0%    0.1%    0.2%    0.0%    0.0%    0.1%    0.0%    0.2%    0.1%    0.1%    0.1%    0.0%    0.0%    0.1%    0.0%    0.0%    0.2%    0.1%    0.0%    0.0%    0.0%    0.0%    0.0%    0.1%    0.0%    0.0%    0.0%    0.0%    0.0%    0.1%    0.1%    0.4%    0.0%    0.1%    0.0%    0.1%    0.0%    0.0%    0.0%    0.0%    0.0%    0.1%    0.1%    0.1%    0.0%    0.0%    0.0%    0.0%    0.0%    0.0%    0.0%    0.0%    0.0%    0.0%    0.0%    0.0%    0.0%    0.0%    0.0%    0.0%    0.0%    0.0%    0.0%    0.0%    0.0%    0.0%    0.1%    0.0%    0.0%    0.0%    0.0%    0.0%    0.1%    0.0%    0.0%    0.0%    0.0%    0.0%    0.0%    0.1%    0.0%    0.0%    0.0%    0.0%    0.0%    0.0%    0.0%    0.0%    0.2%    0.0%    0.0%    0.0%    0.0%    0.0%    0.0%    0.1%    0.0%    0.0%    0.0%    0.0%    0.0%    0.0%    0.0%    0.0%    0.0%    0.1%    0.0%    0.1%    0.0%    0.1%    0.0%    0.0%    0.0%    0.0%    0.0%    0.0%    0.0%    0.0%    0.0%    0.0%    0.0%    0.0%    0.0%    0.0%    0.0%    0.1%    0.0%    0.0%    0.0%    0.0%    0.0%    0.0%    0.0%    0.0%    0.0%    0.0%    0.0%    0.0%    0.0%    0.0%    0.0%    0.0%    0.1%    0.0%    0.0%    0.1%    0.0%    0.0%    0.0%    0.0%    0.0%    0.0%    0.0%    0.0%    0.0%    0.0%    0.0%    0.0%    0.0%    0.0%    0.0%    0.0%    0.0%    0.0%    0.0%    0.0%    0.0%    0.0%    0.0%    0.0%    0.0%    0.0%    0.0%    0.0%    0.0%    0.0%    0.0%    0.0%    0.0%    0.0%    0.0%    0.0%    0.0%    0.0%    0.0%    0.0%    0.0%    0.0%    0.0%    0.0%    0.0%    0.0%    0.0%    0.0%    0.0%    0.0%    0.0%    0.0%    0.0%    0.0%    0.0%    0.0%    0.0%    0.0%    0.0%    0.0%    0.0%    0.0%    0.0%    0.0%    0.0%    0.0%    0.0%    0.0%    0.0%    0.0%    0.0%    0.0%    0.0%    0.0%    0.0%    0.0%    0.0%    0.0%    0.0%    0.0%    0.0%    0.0%    0.0%    0.0%    0.0%    0.0%    0.0%    0.0%    0.0%        k__Archaea; p__Euryarchaeota      29    0.0%    0.0%    0.0%    0.0%    0.0%    0.0%    0.0%    0.0%    0.0%    0.0%    0.0%    0.0%    0.0%    0.0%    0.0%    0.0%    0.0%    0.0%    0.0%    0.0%    0.0%    0.0%    0.0%    0.0%    0.0%    0.0%    0.0%    0.0%    0.0%    0.0%    0.0%    0.0%    0.0%    0.0%    0.0%    0.0%    0.0%    0.0%    0.0%    0.0%    0.0%    0.0%    0.0%    0.0%    0.0%    0.0%    0.0%    0.0%    0.0%    0.0%    0.0%    0.0%    0.0%    0.0%    0.0%    0.0%    0.0%    0.0%    0.0%    0.0%    0.0%    0.0%    0.0%    0.0%    0.0%    0.0%    0.0%    0.0%    0.0%    0.0%    0.0%    0.0%    0.0%    0.0%    0.0%    0.0%    0.0%    0.0%    0.0%    0.0%    0.0%    0.0%    0.0%    0.0%    0.0%    0.0%    0.0%    0.0%    0.0%    0.0%    0.0%    0.0%    0.0%    0.0%    0.0%    0.0%    0.0%    0.0%    0.0%    0.0%    0.0%    0.0%    0.0%    0.0%    0.0%    0.0%    0.0%    0.0%    0.0%    0.0%    0.0%    0.0%    0.0%    0.0%    0.0%    0.0%    0.0%    0.0%    0.0%    0.0%    0.0%    0.0%    0.0%    0.0%    0.0%    0.0%    0.0%    0.0%    0.0%    0.0%    0.0%    0.0%    0.0%    0.0%    0.0%    0.0%    0.0%    0.0%    0.0%    0.0%    0.0%    0.0%    0.0%    0.0%    0.0%    0.0%    0.0%    0.0%    0.0%    0.0%    0.0%    0.0%    0.0%    0.0%    0.0%    0.0%    0.0%    0.0%    0.0%    0.0%    0.0%    0.0%    0.0%    0.0%    0.0%    0.0%    0.0%    0.0%    0.0%    0.0%    0.0%    0.0%    0.0%    0.0%    0.0%    0.0%    0.0%    0.0%    0.0%    0.0%    0.0%    0.0%    0.0%    0.0%    0.0%    0.0%    0.0%    0.0%    0.0%    0.0%    0.0%    0.0%    0.0%    0.0%    0.0%    0.0%    0.0%    0.0%    0.0%    0.0%    0.0%    0.0%    0.0%    0.0%    0.0%    0.0%    0.0%    0.0%    0.0%    0.0%    0.0%    0.0%    0.0%    0.0%    0.0%    0.0%    0.0%    0.0%    0.0%    0.0%    0.0%    0.0%    0.0%    0.0%    0.0%    0.0%    0.0%    0.0%    0.0%    0.0%    0.0%    0.0%    0.0%    0.0%    0.0%    0.0%    0.0%    0.0%    0.0%    0.0%    0.0%    0.0%    0.0%    0.0%    0.0%    0.0%    0.0%    0.0%    0.0%    0.0%    0.0%    0.0%    0.0%    0.0%    0.0%    0.0%    0.0%    0.0%    0.0%    0.0%    0.0%    0.0%    0.0%    0.0%    0.0%    0.0%    0.0%    0.0%    0.0%    0.0%    0.0%    0.0%    0.0%    0.0%    0.0%    0.0%    0.0%    0.0%    0.0%    0.0%    0.0%    0.0%    0.0%    0.0%    0.0%    0.0%    0.0%    0.0%    0.0%    0.0%    0.0%    0.0%    0.0%    0.0%    0.0%    0.0%    0.0%    0.0%    0.0%    0.0%    0.0%    0.0%    0.0%    0.0%    0.0%    0.0%    0.0%    0.0%    0.0%    0.0%    0.0%    0.0%    0.0%    0.0%    0.0%    0.0%    0.0%    0.0%    0.0%    0.0%    0.0%    0.0%    0.0%    0.0%    0.0%    0.0%    0.0%    0.0%    0.0%    0.0%    0.0%    0.0%    0.0%    0.0%    0.0%    0.0%    0.0%    0.0%    0.0%    0.0%    0.0%    0.0%    0.0%    0.0%    0.0%    0.0%    0.0%    0.0%    0.0%    0.0%    0.0%    0.0%    0.0%    0.0%    0.0%    0.0%    0.0%    0.0%    0.0%    0.0%    0.0%    0.0%        k__Bacteria; p__       3    0.0%    0.0%    0.0%    0.0%    0.0%    0.0%    0.0%    0.0%    0.0%    0.0%    0.0%    0.0%    0.0%    0.0%    0.0%    0.0%    0.0%    0.0%    0.0%    0.0%    0.0%    0.0%    0.0%    0.0%    0.0%    0.0%    0.0%    0.0%    0.0%    0.0%    0.0%    0.0%    0.0%    0.0%    0.0%    0.0%    0.0%    0.0%    0.0%    0.0%    0.0%    0.0%    0.0%    0.0%    0.0%    0.0%    0.0%    0.0%    0.0%    0.0%    0.0%    0.0%    0.0%    0.0%    0.0%    0.0%    0.0%    0.0%    0.0%    0.0%    0.0%    0.0%    0.0%    0.0%    0.0%    0.0%    0.0%    0.0%    0.0%    0.0%    0.0%    0.0%    0.0%    0.0%    0.0%    0.0%    0.0%    0.0%    0.0%    0.0%    0.0%    0.0%    0.0%    0.0%    0.0%    0.0%    0.0%    0.0%    0.0%    0.0%    0.0%    0.0%    0.0%    0.0%    0.0%    0.0%    0.0%    0.0%    0.0%    0.0%    0.0%    0.0%    0.0%    0.0%    0.0%    0.0%    0.0%    0.0%    0.0%    0.0%    0.0%    0.0%    0.0%    0.0%    0.0%    0.0%    0.0%    0.0%    0.0%    0.0%    0.0%    0.0%    0.0%    0.0%    0.0%    0.0%    0.0%    0.0%    0.0%    0.0%    0.0%    0.0%    0.0%    0.0%    0.0%    0.0%    0.0%    0.0%    0.0%    0.0%    0.0%    0.0%    0.0%    0.0%    0.0%    0.0%    0.0%    0.0%    0.0%    0.0%    0.0%    0.0%    0.0%    0.0%    0.0%    0.0%    0.0%    0.0%    0.0%    0.0%    0.0%    0.0%    0.0%    0.0%    0.0%    0.0%    0.0%    0.0%    0.0%    0.0%    0.0%    0.0%    0.0%    0.0%    0.0%    0.0%    0.0%    0.0%    0.0%    0.0%    0.0%    0.0%    0.0%    0.0%    0.0%    0.0%    0.0%    0.0%    0.0%    0.0%    0.0%    0.0%    0.0%    0.0%    0.0%    0.0%    0.0%    0.0%    0.0%    0.0%    0.0%    0.0%    0.0%    0.0%    0.0%    0.0%    0.0%    0.0%    0.0%    0.0%    0.0%    0.0%    0.0%    0.0%    0.0%    0.0%    0.0%    0.0%    0.0%    0.0%    0.0%    0.0%    0.0%    0.0%    0.0%    0.0%    0.0%    0.0%    0.0%    0.0%    0.0%    0.0%    0.0%    0.0%    0.0%    0.0%    0.0%    0.0%    0.0%    0.0%    0.0%    0.0%    0.0%    0.0%    0.0%    0.0%    0.0%    0.0%    0.0%    0.0%    0.0%    0.0%    0.0%    0.0%    0.0%    0.0%    0.0%    0.0%    0.0%    0.0%    0.0%    0.0%    0.0%    0.0%    0.0%    0.0%    0.0%    0.0%    0.0%    0.0%    0.0%    0.0%    0.0%    0.0%    0.0%    0.0%    0.0%    0.0%    0.0%    0.0%    0.0%    0.0%    0.0%    0.0%    0.0%    0.0%    0.0%    0.0%    0.0%    0.0%    0.0%    0.0%    0.0%    0.0%    0.0%    0.0%    0.0%    0.0%    0.0%    0.0%    0.0%    0.0%    0.0%    0.0%    0.0%    0.0%    0.0%    0.0%    0.0%    0.0%    0.0%    0.0%    0.0%    0.0%    0.0%    0.0%    0.0%    0.0%    0.0%    0.0%    0.0%    0.0%    0.0%    0.0%    0.0%    0.0%    0.0%    0.0%    0.0%    0.0%    0.0%    0.0%    0.0%    0.0%    0.0%    0.0%    0.0%    0.0%    0.0%    0.0%    0.0%    0.0%    0.0%    0.0%    0.0%    0.0%    0.0%    0.0%    0.0%    0.0%    0.0%    0.0%    0.0%    0.0%    0.0%    0.0%    0.0%    0.0%    0.0%    0.0%    0.0%    0.0%        k__Bacteria; p__ABY1_OD1       0    0.0%    0.0%    0.0%    0.0%    0.0%    0.0%    0.0%    0.0%    0.0%    0.0%    0.0%    0.0%    0.0%    0.0%    0.0%    0.0%    0.0%    0.0%    0.0%    0.0%    0.0%    0.0%    0.0%    0.0%    0.0%    0.0%    0.0%    0.0%    0.0%    0.0%    0.0%    0.0%    0.0%    0.0%    0.0%    0.0%    0.0%    0.0%    0.0%    0.0%    0.0%    0.0%    0.0%    0.0%    0.0%    0.0%    0.0%    0.0%    0.0%    0.0%    0.0%    0.0%    0.0%    0.0%    0.0%    0.0%    0.0%    0.0%    0.0%    0.0%    0.0%    0.0%    0.0%    0.0%    0.0%    0.0%    0.0%    0.0%    0.0%    0.0%    0.0%    0.0%    0.0%    0.0%    0.0%    0.0%    0.0%    0.0%    0.0%    0.0%    0.0%    0.0%    0.0%    0.0%    0.0%    0.0%    0.0%    0.0%    0.0%    0.0%    0.0%    0.0%    0.0%    0.0%    0.0%    0.0%    0.0%    0.0%    0.0%    0.0%    0.0%    0.0%    0.0%    0.0%    0.0%    0.0%    0.0%    0.0%    0.0%    0.0%    0.0%    0.0%    0.0%    0.0%    0.0%    0.0%    0.0%    0.0%    0.0%    0.0%    0.0%    0.0%    0.0%    0.0%    0.0%    0.0%    0.0%    0.0%    0.0%    0.0%    0.0%    0.0%    0.0%    0.0%    0.0%    0.0%    0.0%    0.0%    0.0%    0.0%    0.0%    0.0%    0.0%    0.0%    0.0%    0.0%    0.0%    0.0%    0.0%    0.0%    0.0%    0.0%    0.0%    0.0%    0.0%    0.0%    0.0%    0.0%    0.0%    0.0%    0.0%    0.0%    0.0%    0.0%    0.0%    0.0%    0.0%    0.0%    0.0%    0.0%    0.0%    0.0%    0.0%    0.0%    0.0%    0.0%    0.0%    0.0%    0.0%    0.0%    0.0%    0.0%    0.0%    0.0%    0.0%    0.0%    0.0%    0.0%    0.0%    0.0%    0.0%    0.0%    0.0%    0.0%    0.0%    0.0%    0.0%    0.0%    0.0%    0.0%    0.0%    0.0%    0.0%    0.0%    0.0%    0.0%    0.0%    0.0%    0.0%    0.0%    0.0%    0.0%    0.0%    0.0%    0.0%    0.0%    0.0%    0.0%    0.0%    0.0%    0.0%    0.0%    0.0%    0.0%    0.0%    0.0%    0.0%    0.0%    0.0%    0.0%    0.0%    0.0%    0.0%    0.0%    0.0%    0.0%    0.0%    0.0%    0.0%    0.0%    0.0%    0.0%    0.0%    0.0%    0.0%    0.0%    0.0%    0.0%    0.0%    0.0%    0.0%    0.0%    0.0%    0.0%    0.0%    0.0%    0.0%    0.0%    0.0%    0.0%    0.0%    0.0%    0.0%    0.0%    0.0%    0.0%    0.0%    0.0%    0.0%    0.0%    0.0%    0.0%    0.0%    0.0%    0.0%    0.0%    0.0%    0.0%    0.0%    0.0%    0.0%    0.0%    0.0%    0.0%    0.0%    0.0%    0.0%    0.0%    0.0%    0.0%    0.0%    0.0%    0.0%    0.0%    0.0%    0.0%    0.0%    0.0%    0.0%    0.0%    0.0%    0.0%    0.0%    0.0%    0.0%    0.0%    0.0%    0.0%    0.0%    0.0%    0.0%    0.0%    0.0%    0.0%    0.0%    0.0%    0.0%    0.0%    0.0%    0.0%    0.0%    0.0%    0.0%    0.0%    0.0%    0.0%    0.0%    0.0%    0.0%    0.0%    0.0%    0.0%    0.0%    0.0%    0.0%    0.0%    0.0%    0.0%    0.0%    0.0%    0.0%    0.0%    0.0%    0.0%    0.0%    0.0%    0.0%    0.0%    0.0%    0.0%    0.0%    0.0%    0.0%    0.0%    0.0%    0.0%    0.0%    0.0%    0.0%    0.0%    0.0%    0.0%        k__Bacteria; p__AD3       4    0.0%    0.0%    0.0%    0.0%    0.0%    0.0%    0.0%    0.0%    0.0%    0.0%    0.0%    0.0%    0.0%    0.0%    0.0%    0.0%    0.0%    0.0%    0.0%    0.0%    0.0%    0.0%    0.0%    0.0%    0.0%    0.0%    0.0%    0.0%    0.0%    0.0%    0.0%    0.0%    0.0%    0.0%    0.0%    0.0%    0.0%    0.0%    0.0%    0.0%    0.0%    0.0%    0.0%    0.0%    0.0%    0.0%    0.0%    0.0%    0.0%    0.0%    0.0%    0.0%    0.0%    0.0%    0.0%    0.0%    0.0%    0.0%    0.0%    0.0%    0.0%    0.0%    0.0%    0.0%    0.0%    0.0%    0.0%    0.0%    0.0%    0.0%    0.0%    0.0%    0.0%    0.0%    0.0%    0.0%    0.0%    0.0%    0.0%    0.0%    0.0%    0.0%    0.0%    0.0%    0.0%    0.0%    0.0%    0.0%    0.0%    0.0%    0.0%    0.0%    0.0%    0.0%    0.0%    0.0%    0.0%    0.0%    0.0%    0.0%    0.0%    0.0%    0.0%    0.0%    0.0%    0.0%    0.0%    0.0%    0.0%    0.0%    0.0%    0.0%    0.0%    0.0%    0.0%    0.0%    0.0%    0.0%    0.0%    0.0%    0.0%    0.0%    0.0%    0.0%    0.0%    0.0%    0.0%    0.0%    0.0%    0.0%    0.0%    0.0%    0.0%    0.0%    0.0%    0.0%    0.0%    0.0%    0.0%    0.0%    0.0%    0.0%    0.0%    0.0%    0.0%    0.0%    0.0%    0.0%    0.0%    0.0%    0.0%    0.0%    0.0%    0.0%    0.0%    0.0%    0.0%    0.0%    0.0%    0.0%    0.0%    0.0%    0.0%    0.0%    0.0%    0.0%    0.0%    0.0%    0.0%    0.0%    0.0%    0.0%    0.0%    0.0%    0.0%    0.0%    0.0%    0.0%    0.0%    0.0%    0.0%    0.0%    0.0%    0.0%    0.0%    0.0%    0.0%    0.0%    0.0%    0.0%    0.0%    0.0%    0.0%    0.0%    0.0%    0.0%    0.0%    0.0%    0.0%    0.0%    0.0%    0.0%    0.0%    0.0%    0.0%    0.0%    0.0%    0.0%    0.0%    0.0%    0.0%    0.0%    0.0%    0.0%    0.0%    0.0%    0.0%    0.0%    0.0%    0.0%    0.0%    0.0%    0.0%    0.0%    0.0%    0.0%    0.0%    0.0%    0.0%    0.0%    0.0%    0.0%    0.0%    0.0%    0.0%    0.0%    0.0%    0.0%    0.0%    0.0%    0.0%    0.0%    0.0%    0.0%    0.0%    0.0%    0.0%    0.0%    0.0%    0.0%    0.0%    0.0%    0.0%    0.0%    0.0%    0.0%    0.0%    0.0%    0.0%    0.0%    0.0%    0.0%    0.0%    0.0%    0.0%    0.0%    0.0%    0.0%    0.0%    0.0%    0.0%    0.0%    0.0%    0.0%    0.0%    0.0%    0.0%    0.0%    0.0%    0.0%    0.0%    0.0%    0.0%    0.0%    0.0%    0.0%    0.0%    0.0%    0.0%    0.0%    0.0%    0.0%    0.0%    0.0%    0.0%    0.0%    0.0%    0.0%    0.0%    0.0%    0.0%    0.0%    0.0%    0.0%    0.0%    0.0%    0.0%    0.0%    0.0%    0.0%    0.0%    0.0%    0.0%    0.0%    0.0%    0.0%    0.0%    0.0%    0.0%    0.0%    0.0%    0.0%    0.0%    0.0%    0.0%    0.0%    0.0%    0.0%    0.0%    0.0%    0.0%    0.0%    0.0%    0.0%    0.0%    0.0%    0.0%    0.0%    0.0%    0.0%    0.0%    0.0%    0.0%    0.0%    0.0%    0.0%    0.0%    0.0%    0.0%    0.0%    0.0%    0.0%    0.0%    0.0%    0.0%    0.0%    0.6%    0.0%    0.0%    0.0%    0.0%    0.0%        k__Bacteria; p__Acidobacteria    8063    0.1%    0.0%    0.0%    0.0%    0.1%    0.0%    0.0%    0.0%    0.0%    0.0%    0.0%    0.0%    0.0%    0.1%    0.0%    0.0%    0.1%    0.0%    0.1%    0.0%    0.1%    0.2%    0.1%    0.1%    0.0%    0.0%    0.0%    0.1%    0.1%    0.2%    0.1%    0.1%    0.1%    0.0%    0.0%    0.0%    0.1%    0.0%    0.1%    0.1%    0.1%    0.2%    0.0%    0.0%    0.2%    0.1%    0.0%    0.0%    0.0%    0.0%    0.1%    0.1%    0.1%    0.0%    0.6%    0.2%    0.2%    0.3%    0.1%    0.2%    0.1%    0.1%    0.1%    0.3%    0.2%    0.1%    0.0%    0.2%    0.0%    0.0%    0.0%    0.1%    0.1%    0.1%    0.2%    0.0%    0.1%    0.4%    0.1%    0.2%    0.1%    0.1%    0.1%    0.1%    0.0%    0.0%    0.2%    0.0%    0.0%    0.1%    0.0%    0.1%    0.2%    0.1%    0.1%    0.2%    0.0%    0.3%    0.2%    0.2%    0.2%    0.3%    0.1%    0.0%    0.0%    0.4%    0.0%    0.0%    0.1%    0.2%    0.2%    0.3%    0.0%    0.0%    0.2%    0.1%    0.1%    0.0%    0.1%    0.5%    0.0%    0.1%    0.4%    0.2%    0.1%    0.6%    1.2%    0.0%    0.2%    0.9%    0.2%    0.1%    0.5%    0.1%    0.5%    0.2%    0.6%    0.3%    0.1%    0.0%    0.1%    0.3%    0.0%    0.6%    0.3%    0.0%    0.0%    0.1%    0.0%    0.1%    0.4%    0.3%    0.0%    0.0%    0.4%    0.0%    0.1%    0.4%    1.3%    0.2%    0.5%    0.0%    0.3%    0.1%    0.3%    0.1%    0.2%    0.2%    0.3%    0.4%    0.4%    0.1%    0.1%    0.1%    0.0%    0.1%    0.0%    0.1%    0.1%    0.0%    0.1%    0.1%    0.1%    0.0%    0.1%    0.0%    0.0%    0.1%    0.0%    0.0%    0.3%    0.0%    0.1%    0.3%    0.0%    0.4%    0.0%    0.0%    0.0%    0.3%    0.3%    0.0%    0.0%    0.4%    0.3%    0.3%    0.4%    0.3%    0.1%    0.0%    0.3%    0.4%    0.1%    0.2%    0.1%    0.0%    0.0%    0.2%    0.1%    0.2%    0.9%    0.0%    0.2%    0.0%    0.5%    0.0%    0.2%    0.2%    0.3%    0.1%    0.0%    0.1%    1.2%    0.0%    0.6%    0.0%    0.3%    0.1%    0.1%    0.1%    0.5%    0.2%    0.2%    0.0%    0.3%    0.2%    0.2%    0.2%    0.1%    0.1%    0.5%    0.0%    0.4%    0.3%    0.2%    0.1%    0.1%    0.0%    0.1%    0.2%    0.1%    0.2%    0.4%    0.1%    0.2%    0.3%    0.4%    0.0%    0.3%    0.4%    0.2%    0.2%    1.1%    2.2%    0.3%    0.1%    0.1%    0.2%    0.3%    0.7%    0.7%    0.3%    0.1%    0.0%    0.0%    0.4%    0.0%    0.0%    0.0%    0.1%    0.0%    0.2%    0.1%    1.0%    0.0%    0.0%    0.0%    0.0%    0.1%    0.4%    0.0%    0.0%    0.1%    0.3%    0.1%    0.1%    0.1%    0.0%    0.0%    0.2%    0.1%    0.0%    0.2%    0.0%    0.0%    0.1%    0.3%    0.3%    0.0%    0.2%    0.2%    0.2%    0.1%    0.1%    0.2%    0.1%    0.1%    0.1%    0.0%    0.1%    0.0%    0.0%    0.0%    0.1%    0.0%    0.0%    0.1%    0.1%    0.1%    0.1%    0.0%    0.0%    0.1%    0.1%    0.1%    0.1%    0.1%    0.0%    0.1%    0.1%    0.1%    0.0%    0.1%    0.1%    0.1%    0.0%    0.2%    0.0%    0.1%    0.0%    0.0%    0.2%        k__Bacteria; p__Actinobacteria   1067161   18.7%   25.7%   19.0%   28.4%   41.3%    3.6%   20.3%    7.1%   41.4%   31.5%   16.4%   22.4%    9.6%   15.7%   23.4%   25.9%   17.0%   26.2%    8.4%   16.8%   20.5%   15.5%   21.7%   15.5%   24.7%   45.5%   33.6%   12.4%   14.6%   27.0%   28.0%   42.9%   13.0%   16.3%   18.2%   26.2%   19.7%   26.4%   12.5%    7.3%    9.6%   12.6%   16.5%   20.1%   19.2%   18.2%   27.8%   26.8%   12.3%   19.8%   10.5%    6.9%   13.7%   11.3%    8.5%   37.1%   22.8%   13.5%   16.1%   14.7%   16.9%   15.4%   19.1%   12.1%   11.3%   17.6%    1.6%   14.3%    4.1%    9.0%    3.3%   11.9%    5.7%   18.4%   16.1%    3.9%    8.7%   14.5%   36.8%   24.8%   25.4%    8.7%    9.4%   21.3%   40.7%   40.3%   19.7%    4.0%    9.8%   23.5%    2.7%    9.4%   19.1%    6.8%   47.2%   29.2%    2.3%   17.4%   21.9%   19.0%   22.0%   22.6%   31.0%   42.8%   24.1%   18.5%    3.0%    0.6%   20.3%   29.2%   25.2%   14.2%    5.5%    5.9%   16.0%   22.9%   21.7%   22.1%   10.6%   25.8%    0.8%   15.9%   17.0%   12.8%    6.9%   18.5%   22.6%    3.0%   20.6%   18.4%   17.4%   10.6%    9.0%   13.6%   25.6%   13.2%   18.6%   20.9%    2.4%    2.1%    9.3%    8.2%    1.4%   11.7%   27.8%   18.8%   17.6%   13.6%    3.0%    4.3%   13.7%   19.5%    6.2%    4.4%   14.8%    4.7%    3.7%   19.9%   22.4%   19.4%   14.5%    3.6%   30.3%    8.4%   15.7%   21.7%   24.7%   18.2%   14.2%   21.3%    7.9%   13.1%   18.1%   29.7%   23.0%   14.2%    3.6%   14.9%   21.8%   18.5%    9.0%    6.6%   26.6%   20.3%   34.9%   20.3%   25.8%   31.9%   36.0%   27.0%   12.7%    4.6%   20.5%   16.4%   28.6%   27.5%   17.4%   14.9%    9.5%    8.5%   19.1%    5.8%    5.9%   16.4%    9.9%    8.6%   28.7%   20.6%   23.3%   20.9%    3.3%   23.1%   13.6%   23.4%    7.7%   20.4%    0.4%   24.6%   31.8%   22.4%   10.8%    2.5%    5.0%    9.9%   14.7%   17.8%   18.2%   37.3%   14.7%   17.5%   17.1%   30.8%   15.0%   12.7%   17.5%    4.6%   12.8%   38.3%   26.0%    9.8%   18.5%   19.0%   27.2%    8.6%   27.5%   16.0%   19.1%   21.5%   16.0%   30.1%   23.1%   48.6%   27.7%   21.0%    8.5%   20.4%    9.9%   10.6%    9.3%   30.2%   13.1%   28.4%   20.3%    4.4%   25.2%   10.4%   41.7%    5.0%   37.5%   27.8%   20.6%   14.1%   26.3%   31.5%   31.3%   52.8%   14.4%   34.9%   15.9%   31.2%   25.2%   33.6%   21.7%   19.1%   18.3%   26.1%   28.2%   31.2%   11.9%    6.0%   33.6%   35.1%   31.0%   23.9%   18.0%   34.7%   47.4%   50.3%   30.6%   21.8%   18.1%   61.4%   44.6%   28.9%   11.2%   20.8%   12.4%   15.9%   17.5%   31.0%   14.7%   21.9%   24.6%   10.8%   14.9%   18.7%   31.7%    6.0%   51.2%   36.1%   29.1%   24.2%   28.5%    9.0%   36.7%   32.8%   26.4%   28.7%   31.9%   23.7%   12.9%   19.1%   25.5%   12.6%   35.8%   41.5%   55.3%   28.4%   23.4%   14.7%   75.2%   12.5%   42.8%   22.0%   25.5%   34.1%   29.8%   27.8%   37.5%   51.7%   23.3%   45.4%   29.6%   20.8%   22.6%    0.3%   10.8%    3.0%   19.1%   39.3%    7.4%   22.6%        k__Bacteria; p__Aquificae      13    0.0%    0.0%    0.0%    0.0%    0.0%    0.0%    0.0%    0.0%    0.0%    0.0%    0.0%    0.0%    0.0%    0.0%    0.0%    0.0%    0.0%    0.0%    0.0%    0.0%    0.0%    0.0%    0.0%    0.0%    0.0%    0.0%    0.0%    0.0%    0.0%    0.0%    0.0%    0.0%    0.0%    0.0%    0.0%    0.0%    0.0%    0.0%    0.0%    0.0%    0.0%    0.0%    0.0%    0.0%    0.0%    0.0%    0.0%    0.0%    0.0%    0.0%    0.0%    0.0%    0.0%    0.0%    0.0%    0.0%    0.0%    0.0%    0.0%    0.0%    0.0%    0.0%    0.0%    0.0%    0.0%    0.0%    0.0%    0.0%    0.0%    0.0%    0.0%    0.0%    0.0%    0.0%    0.0%    0.0%    0.0%    0.0%    0.0%    0.0%    0.0%    0.0%    0.0%    0.0%    0.0%    0.0%    0.0%    0.0%    0.0%    0.0%    0.0%    0.0%    0.0%    0.0%    0.0%    0.0%    0.0%    0.0%    0.0%    0.0%    0.0%    0.0%    0.0%    0.0%    0.0%    0.0%    0.0%    0.0%    0.0%    0.0%    0.0%    0.0%    0.0%    0.0%    0.0%    0.0%    0.0%    0.0%    0.0%    0.0%    0.0%    0.0%    0.0%    0.0%    0.0%    0.0%    0.0%    0.0%    0.0%    0.0%    0.0%    0.0%    0.0%    0.0%    0.0%    0.0%    0.0%    0.0%    0.0%    0.0%    0.0%    0.0%    0.0%    0.0%    0.0%    0.0%    0.0%    0.0%    0.0%    0.0%    0.0%    0.0%    0.0%    0.0%    0.0%    0.0%    0.0%    0.0%    0.0%    0.0%    0.0%    0.0%    0.0%    0.0%    0.0%    0.0%    0.0%    0.0%    0.0%    0.0%    0.0%    0.0%    0.0%    0.0%    0.0%    0.0%    0.0%    0.0%    0.0%    0.0%    0.0%    0.0%    0.0%    0.0%    0.0%    0.0%    0.0%    0.0%    0.0%    0.0%    0.0%    0.0%    0.0%    0.0%    0.0%    0.0%    0.0%    0.0%    0.0%    0.0%    0.0%    0.0%    0.0%    0.0%    0.0%    0.0%    0.0%    0.0%    0.0%    0.0%    0.0%    0.0%    0.0%    0.0%    0.0%    0.0%    0.0%    0.0%    0.0%    0.0%    0.0%    0.0%    0.0%    0.0%    0.0%    0.0%    0.0%    0.0%    0.0%    0.0%    0.0%    0.0%    0.0%    0.0%    0.0%    0.0%    0.0%    0.0%    0.0%    0.0%    0.0%    0.0%    0.0%    0.0%    0.0%    0.0%    0.0%    0.0%    0.0%    0.0%    0.0%    0.0%    0.0%    0.0%    0.0%    0.0%    0.0%    0.0%    0.0%    0.0%    0.0%    0.0%    0.0%    0.0%    0.0%    0.0%    0.0%    0.0%    0.0%    0.0%    0.0%    0.0%    0.0%    0.0%    0.0%    0.0%    0.0%    0.0%    0.0%    0.0%    0.0%    0.0%    0.0%    0.0%    0.0%    0.0%    0.0%    0.0%    0.0%    0.0%    0.0%    0.0%    0.0%    0.0%    0.0%    0.0%    0.0%    0.0%    0.0%    0.0%    0.0%    0.0%    0.0%    0.0%    0.0%    0.0%    0.0%    0.0%    0.0%    0.0%    0.0%    0.0%    0.0%    0.0%    0.0%    0.0%    0.0%    0.0%    0.0%    0.0%    0.0%    0.0%    0.0%    0.0%    0.0%    0.0%    0.0%    0.0%    0.0%    0.0%    0.0%    0.0%    0.0%    0.0%    0.0%    0.0%    0.0%    0.0%    0.0%    0.0%    0.0%    0.0%    0.0%    0.0%    0.0%    0.0%    0.0%    0.0%    0.0%    0.0%    0.0%    0.0%    0.0%    0.0%    0.0%    0.0%    0.0%    0.0%    0.0%    0.0%    0.0%    0.0%        k__Bacteria; p__BRC1      59    0.0%    0.0%    0.0%    0.0%    0.0%    0.0%    0.0%    0.0%    0.0%    0.0%    0.0%    0.0%    0.0%    0.0%    0.0%    0.0%    0.0%    0.0%    0.0%    0.0%    0.0%    0.0%    0.0%    0.0%    0.0%    0.0%    0.0%    0.0%    0.0%    0.0%    0.0%    0.0%    0.0%    0.0%    0.0%    0.0%    0.0%    0.0%    0.0%    0.0%    0.0%    0.0%    0.0%    0.0%    0.0%    0.0%    0.0%    0.0%    0.0%    0.0%    0.0%    0.0%    0.0%    0.0%    0.0%    0.0%    0.0%    0.0%    0.0%    0.0%    0.0%    0.0%    0.0%    0.0%    0.0%    0.0%    0.0%    0.0%    0.0%    0.0%    0.0%    0.0%    0.0%    0.0%    0.0%    0.0%    0.0%    0.0%    0.0%    0.0%    0.0%    0.0%    0.0%    0.0%    0.0%    0.0%    0.0%    0.0%    0.0%    0.0%    0.0%    0.0%    0.0%    0.0%    0.0%    0.0%    0.0%    0.0%    0.0%    0.0%    0.0%    0.0%    0.0%    0.0%    0.0%    0.0%    0.0%    0.0%    0.0%    0.0%    0.0%    0.0%    0.0%    0.0%    0.0%    0.0%    0.0%    0.0%    0.0%    0.0%    0.0%    0.0%    0.0%    0.0%    0.0%    0.0%    0.0%    0.0%    0.0%    0.0%    0.0%    0.0%    0.0%    0.0%    0.0%    0.0%    0.0%    0.0%    0.0%    0.0%    0.0%    0.0%    0.0%    0.0%    0.0%    0.0%    0.0%    0.0%    0.0%    0.0%    0.0%    0.0%    0.0%    0.0%    0.0%    0.0%    0.0%    0.0%    0.0%    0.0%    0.0%    0.0%    0.0%    0.0%    0.0%    0.0%    0.0%    0.0%    0.0%    0.0%    0.0%    0.0%    0.0%    0.0%    0.0%    0.0%    0.0%    0.0%    0.0%    0.0%    0.0%    0.0%    0.0%    0.0%    0.0%    0.0%    0.0%    0.0%    0.0%    0.0%    0.0%    0.0%    0.0%    0.0%    0.0%    0.0%    0.0%    0.0%    0.0%    0.0%    0.0%    0.0%    0.0%    0.0%    0.0%    0.0%    0.0%    0.0%    0.0%    0.0%    0.0%    0.0%    0.0%    0.0%    0.0%    0.0%    0.0%    0.0%    0.0%    0.0%    0.0%    0.0%    0.0%    0.0%    0.0%    0.0%    0.0%    0.0%    0.0%    0.0%    0.0%    0.0%    0.0%    0.0%    0.0%    0.0%    0.0%    0.0%    0.0%    0.0%    0.0%    0.0%    0.0%    0.0%    0.0%    0.0%    0.0%    0.0%    0.0%    0.0%    0.0%    0.0%    0.0%    0.0%    0.0%    0.0%    0.0%    0.0%    0.0%    0.0%    0.0%    0.0%    0.0%    0.0%    0.0%    0.0%    0.0%    0.0%    0.0%    0.0%    0.0%    0.0%    0.0%    0.0%    0.0%    0.0%    0.0%    0.0%    0.0%    0.0%    0.0%    0.0%    0.0%    0.0%    0.0%    0.0%    0.0%    0.0%    0.0%    0.0%    0.0%    0.0%    0.0%    0.0%    0.0%    0.0%    0.0%    0.0%    0.0%    0.0%    0.0%    0.0%    0.0%    0.0%    0.0%    0.0%    0.0%    0.0%    0.0%    0.0%    0.0%    0.0%    0.0%    0.0%    0.0%    0.0%    0.0%    0.0%    0.0%    0.0%    0.0%    0.0%    0.0%    0.0%    0.0%    0.0%    0.0%    0.0%    0.0%    0.0%    0.0%    0.0%    0.0%    0.0%    0.0%    0.0%    0.0%    0.0%    0.0%    0.0%    0.0%    0.0%    0.0%    0.0%    0.0%    0.0%    0.0%    0.0%    0.0%    0.0%    0.0%    0.0%    0.0%    0.0%    0.0%    0.0%    0.0%    0.0%    0.0%    0.0%    0.0%    0.0%        k__Bacteria; p__Bacteroidetes   315489    5.5%   10.0%    7.1%    5.8%    4.4%    2.9%   12.3%    1.0%    1.8%    6.0%    4.6%    1.0%    0.8%    2.7%   17.4%    9.7%    3.9%    6.1%    3.6%    6.5%    3.5%    5.5%    8.4%    2.4%    9.0%    8.5%    4.8%    2.2%    5.0%    4.5%    2.7%    4.7%    4.7%   16.0%    8.5%   10.0%    7.5%    4.3%    3.7%    8.6%    2.8%    8.5%    9.4%   10.2%    6.8%    5.1%    4.7%    3.4%    6.5%    6.3%    5.9%    3.2%    4.1%   11.7%    6.4%    4.1%    6.0%    4.9%    3.9%    2.3%    4.0%    2.0%    3.6%    3.7%    6.4%    3.6%    9.6%    5.3%    2.7%    2.6%    5.1%    3.8%    1.6%    6.3%    5.2%    0.8%    3.1%    4.9%    2.3%    9.7%    8.3%    3.4%    4.0%   10.8%    3.3%    4.3%    7.4%    2.3%    2.1%    4.2%    2.0%    3.0%    3.9%    1.2%    3.9%    5.3%    2.0%    8.5%    7.9%    5.5%    3.8%    5.4%    3.6%    1.5%    1.7%   10.1%    3.5%    7.4%    2.8%    6.1%    6.0%    9.7%   16.4%    3.1%    7.3%    3.9%    5.2%    3.0%    3.7%   16.6%   10.6%    3.4%    5.2%    9.8%    3.0%   12.3%    7.7%    1.6%    4.2%    5.9%    5.5%    1.7%    2.9%   14.7%    4.8%    2.8%    4.2%    4.2%    3.4%    3.7%    5.8%    6.6%    8.3%    4.5%    5.4%   16.9%    6.6%    3.4%    3.2%    3.0%    7.4%    6.3%    5.2%   11.3%    7.1%    2.1%    2.4%    6.2%    5.6%    4.8%   10.2%    1.6%    5.7%    2.0%   11.6%    5.0%    4.5%    7.5%    8.5%    6.4%    2.4%    5.3%    2.4%    2.6%    5.2%    7.6%    1.6%    3.9%    5.5%    3.8%    6.8%    1.4%    4.7%    3.9%    2.0%    3.6%    3.4%    5.1%    3.3%    3.0%    6.9%    1.2%    4.3%   12.3%    4.6%    2.0%    1.8%   12.9%    1.5%    5.0%    4.5%    2.7%    2.1%    8.1%    4.1%    3.6%    6.6%    5.2%    5.2%    5.6%    1.0%    6.5%    3.8%    6.8%    9.1%    6.0%    0.1%    2.6%    7.4%    7.6%    6.8%    1.6%   13.0%    4.9%    5.6%    4.5%    3.0%    3.9%    5.9%    6.2%    7.5%    5.3%    6.9%    1.7%    6.3%    0.2%    4.0%    3.8%    5.2%    2.3%    2.0%    1.5%    2.1%    1.9%    3.3%    7.1%    4.6%    1.5%    4.0%    1.1%    3.3%    3.6%    8.8%    7.0%    3.2%    6.7%    5.5%    2.0%    3.4%    5.0%    3.2%    7.7%    9.9%    7.7%    5.1%    6.2%    6.7%    1.8%    3.6%    6.0%    9.0%    6.8%    6.9%    4.3%    7.1%    5.8%    5.2%    8.1%    9.1%    5.0%    2.6%    3.1%    2.5%    3.2%    2.7%    2.9%    4.6%    7.9%    4.1%    2.8%    1.8%    5.1%    3.5%    5.7%    4.1%    3.2%    3.9%    6.2%    7.1%    7.9%    3.4%    2.3%    3.9%    5.6%    4.9%    7.4%   11.5%    5.6%    3.2%    4.4%    7.2%    1.7%    4.0%    2.5%    7.1%   13.7%    4.7%    0.3%    2.8%    5.4%    5.0%    4.4%    5.0%    2.8%    3.1%    3.9%    6.3%    4.3%    3.3%    3.5%    4.0%    4.8%    3.5%    3.1%    0.8%    1.4%    0.7%    7.9%    4.8%    5.6%    1.1%    9.0%    3.4%    8.3%    1.9%    2.9%    4.3%    2.3%    1.2%    1.7%    4.2%    2.7%    2.8%    5.1%    2.1%    0.7%    5.8%    0.1%    2.9%    1.5%    1.4%    2.2%        k__Bacteria; p__CCM11b       7    0.0%    0.0%    0.0%    0.0%    0.0%    0.0%    0.0%    0.0%    0.0%    0.0%    0.0%    0.0%    0.0%    0.0%    0.0%    0.0%    0.0%    0.0%    0.0%    0.0%    0.0%    0.0%    0.0%    0.0%    0.0%    0.0%    0.0%    0.0%    0.0%    0.0%    0.0%    0.0%    0.0%    0.0%    0.0%    0.0%    0.0%    0.0%    0.0%    0.0%    0.0%    0.0%    0.0%    0.0%    0.0%    0.0%    0.0%    0.0%    0.0%    0.0%    0.0%    0.0%    0.0%    0.0%    0.0%    0.0%    0.0%    0.0%    0.0%    0.0%    0.0%    0.0%    0.0%    0.0%    0.0%    0.0%    0.0%    0.0%    0.0%    0.0%    0.0%    0.0%    0.0%    0.0%    0.0%    0.0%    0.0%    0.0%    0.0%    0.0%    0.0%    0.0%    0.0%    0.0%    0.0%    0.0%    0.0%    0.0%    0.0%    0.0%    0.0%    0.0%    0.0%    0.0%    0.0%    0.0%    0.0%    0.0%    0.0%    0.0%    0.0%    0.0%    0.0%    0.0%    0.0%    0.0%    0.0%    0.0%    0.0%    0.0%    0.0%    0.0%    0.0%    0.0%    0.0%    0.0%    0.0%    0.0%    0.0%    0.0%    0.0%    0.0%    0.0%    0.0%    0.0%    0.0%    0.0%    0.0%    0.0%    0.0%    0.0%    0.0%    0.0%    0.0%    0.0%    0.0%    0.0%    0.0%    0.0%    0.0%    0.0%    0.0%    0.0%    0.0%    0.0%    0.0%    0.0%    0.0%    0.0%    0.0%    0.0%    0.0%    0.0%    0.0%    0.0%    0.0%    0.0%    0.0%    0.0%    0.0%    0.0%    0.0%    0.0%    0.0%    0.0%    0.0%    0.0%    0.0%    0.0%    0.0%    0.0%    0.0%    0.0%    0.0%    0.0%    0.0%    0.0%    0.0%    0.0%    0.0%    0.0%    0.0%    0.0%    0.0%    0.0%    0.0%    0.0%    0.0%    0.0%    0.0%    0.0%    0.0%    0.0%    0.0%    0.0%    0.0%    0.0%    0.0%    0.0%    0.0%    0.0%    0.0%    0.0%    0.0%    0.0%    0.0%    0.0%    0.0%    0.0%    0.0%    0.0%    0.0%    0.0%    0.0%    0.0%    0.0%    0.0%    0.0%    0.0%    0.0%    0.0%    0.0%    0.0%    0.0%    0.0%    0.0%    0.0%    0.0%    0.0%    0.0%    0.0%    0.0%    0.0%    0.0%    0.0%    0.0%    0.0%    0.0%    0.0%    0.0%    0.0%    0.0%    0.0%    0.0%    0.0%    0.0%    0.0%    0.0%    0.0%    0.0%    0.0%    0.0%    0.0%    0.0%    0.0%    0.0%    0.0%    0.0%    0.0%    0.0%    0.0%    0.0%    0.0%    0.0%    0.0%    0.0%    0.0%    0.0%    0.0%    0.0%    0.0%    0.0%    0.0%    0.0%    0.0%    0.0%    0.0%    0.0%    0.0%    0.0%    0.0%    0.0%    0.0%    0.0%    0.0%    0.0%    0.0%    0.0%    0.0%    0.0%    0.0%    0.0%    0.0%    0.0%    0.0%    0.0%    0.0%    0.0%    0.0%    0.0%    0.0%    0.0%    0.0%    0.0%    0.0%    0.0%    0.0%    0.0%    0.0%    0.0%    0.0%    0.0%    0.0%    0.0%    0.0%    0.0%    0.0%    0.0%    0.0%    0.0%    0.0%    0.0%    0.0%    0.0%    0.0%    0.0%    0.0%    0.0%    0.0%    0.0%    0.0%    0.0%    0.0%    0.0%    0.0%    0.0%    0.0%    0.0%    0.0%    0.0%    0.0%    0.0%    0.0%    0.0%    0.0%    0.0%    0.0%    0.0%    0.0%    0.0%    0.0%    0.0%    0.0%    0.0%    0.0%    0.0%    0.0%    0.0%    0.0%    0.0%    0.0%    0.0%        k__Bacteria; p__Caldithrix_KSB1       1    0.0%    0.0%    0.0%    0.0%    0.0%    0.0%    0.0%    0.0%    0.0%    0.0%    0.0%    0.0%    0.0%    0.0%    0.0%    0.0%    0.0%    0.0%    0.0%    0.0%    0.0%    0.0%    0.0%    0.0%    0.0%    0.0%    0.0%    0.0%    0.0%    0.0%    0.0%    0.0%    0.0%    0.0%    0.0%    0.0%    0.0%    0.0%    0.0%    0.0%    0.0%    0.0%    0.0%    0.0%    0.0%    0.0%    0.0%    0.0%    0.0%    0.0%    0.0%    0.0%    0.0%    0.0%    0.0%    0.0%    0.0%    0.0%    0.0%    0.0%    0.0%    0.0%    0.0%    0.0%    0.0%    0.0%    0.0%    0.0%    0.0%    0.0%    0.0%    0.0%    0.0%    0.0%    0.0%    0.0%    0.0%    0.0%    0.0%    0.0%    0.0%    0.0%    0.0%    0.0%    0.0%    0.0%    0.0%    0.0%    0.0%    0.0%    0.0%    0.0%    0.0%    0.0%    0.0%    0.0%    0.0%    0.0%    0.0%    0.0%    0.0%    0.0%    0.0%    0.0%    0.0%    0.0%    0.0%    0.0%    0.0%    0.0%    0.0%    0.0%    0.0%    0.0%    0.0%    0.0%    0.0%    0.0%    0.0%    0.0%    0.0%    0.0%    0.0%    0.0%    0.0%    0.0%    0.0%    0.0%    0.0%    0.0%    0.0%    0.0%    0.0%    0.0%    0.0%    0.0%    0.0%    0.0%    0.0%    0.0%    0.0%    0.0%    0.0%    0.0%    0.0%    0.0%    0.0%    0.0%    0.0%    0.0%    0.0%    0.0%    0.0%    0.0%    0.0%    0.0%    0.0%    0.0%    0.0%    0.0%    0.0%    0.0%    0.0%    0.0%    0.0%    0.0%    0.0%    0.0%    0.0%    0.0%    0.0%    0.0%    0.0%    0.0%    0.0%    0.0%    0.0%    0.0%    0.0%    0.0%    0.0%    0.0%    0.0%    0.0%    0.0%    0.0%    0.0%    0.0%    0.0%    0.0%    0.0%    0.0%    0.0%    0.0%    0.0%    0.0%    0.0%    0.0%    0.0%    0.0%    0.0%    0.0%    0.0%    0.0%    0.0%    0.0%    0.0%    0.0%    0.0%    0.0%    0.0%    0.0%    0.0%    0.0%    0.0%    0.0%    0.0%    0.0%    0.0%    0.0%    0.0%    0.0%    0.0%    0.0%    0.0%    0.0%    0.0%    0.0%    0.0%    0.0%    0.0%    0.0%    0.0%    0.0%    0.0%    0.0%    0.0%    0.0%    0.0%    0.0%    0.0%    0.0%    0.0%    0.0%    0.0%    0.0%    0.0%    0.0%    0.0%    0.0%    0.0%    0.0%    0.0%    0.0%    0.0%    0.0%    0.0%    0.0%    0.0%    0.0%    0.0%    0.0%    0.0%    0.0%    0.0%    0.0%    0.0%    0.0%    0.0%    0.0%    0.0%    0.0%    0.0%    0.0%    0.0%    0.0%    0.0%    0.0%    0.0%    0.0%    0.0%    0.0%    0.0%    0.0%    0.0%    0.0%    0.0%    0.0%    0.0%    0.0%    0.0%    0.0%    0.0%    0.0%    0.0%    0.0%    0.0%    0.0%    0.0%    0.0%    0.0%    0.0%    0.0%    0.0%    0.0%    0.0%    0.0%    0.0%    0.0%    0.0%    0.0%    0.0%    0.0%    0.0%    0.0%    0.0%    0.0%    0.0%    0.0%    0.0%    0.0%    0.0%    0.0%    0.0%    0.0%    0.0%    0.0%    0.0%    0.0%    0.0%    0.0%    0.0%    0.0%    0.0%    0.0%    0.0%    0.0%    0.0%    0.0%    0.0%    0.0%    0.0%    0.0%    0.0%    0.0%    0.0%    0.0%    0.0%    0.0%    0.0%    0.0%    0.0%    0.0%    0.0%    0.0%    0.0%    0.0%    0.0%    0.0%    0.0%    0.0%    0.0%        k__Bacteria; p__Chlamydiae      13    0.0%    0.0%    0.0%    0.0%    0.0%    0.0%    0.0%    0.0%    0.0%    0.0%    0.0%    0.0%    0.0%    0.0%    0.0%    0.0%    0.0%    0.0%    0.0%    0.0%    0.0%    0.0%    0.0%    0.0%    0.0%    0.0%    0.0%    0.0%    0.0%    0.0%    0.0%    0.0%    0.0%    0.0%    0.0%    0.0%    0.0%    0.0%    0.0%    0.0%    0.0%    0.0%    0.0%    0.0%    0.0%    0.0%    0.0%    0.0%    0.0%    0.0%    0.0%    0.0%    0.0%    0.0%    0.0%    0.0%    0.0%    0.0%    0.0%    0.0%    0.0%    0.0%    0.0%    0.0%    0.0%    0.0%    0.0%    0.0%    0.0%    0.0%    0.0%    0.0%    0.0%    0.0%    0.0%    0.0%    0.0%    0.0%    0.0%    0.0%    0.0%    0.0%    0.0%    0.0%    0.0%    0.0%    0.0%    0.0%    0.0%    0.0%    0.0%    0.0%    0.0%    0.0%    0.0%    0.0%    0.0%    0.0%    0.0%    0.0%    0.0%    0.0%    0.0%    0.0%    0.0%    0.0%    0.0%    0.0%    0.0%    0.0%    0.0%    0.0%    0.0%    0.0%    0.0%    0.0%    0.0%    0.0%    0.0%    0.0%    0.0%    0.0%    0.0%    0.0%    0.0%    0.0%    0.0%    0.0%    0.0%    0.0%    0.0%    0.0%    0.0%    0.0%    0.0%    0.0%    0.0%    0.0%    0.0%    0.0%    0.0%    0.0%    0.0%    0.0%    0.0%    0.0%    0.0%    0.0%    0.0%    0.0%    0.0%    0.0%    0.0%    0.0%    0.0%    0.0%    0.0%    0.0%    0.0%    0.0%    0.0%    0.0%    0.0%    0.0%    0.0%    0.0%    0.0%    0.0%    0.0%    0.0%    0.0%    0.0%    0.0%    0.0%    0.0%    0.0%    0.0%    0.0%    0.0%    0.0%    0.0%    0.0%    0.0%    0.0%    0.0%    0.0%    0.0%    0.0%    0.0%    0.0%    0.0%    0.0%    0.0%    0.0%    0.0%    0.0%    0.0%    0.0%    0.0%    0.0%    0.0%    0.0%    0.0%    0.0%    0.0%    0.0%    0.0%    0.0%    0.0%    0.0%    0.0%    0.0%    0.0%    0.0%    0.0%    0.0%    0.0%    0.0%    0.0%    0.0%    0.0%    0.0%    0.0%    0.0%    0.0%    0.0%    0.0%    0.0%    0.0%    0.0%    0.0%    0.0%    0.0%    0.0%    0.0%    0.0%    0.0%    0.0%    0.0%    0.0%    0.0%    0.0%    0.0%    0.0%    0.0%    0.0%    0.0%    0.0%    0.0%    0.0%    0.0%    0.0%    0.0%    0.0%    0.0%    0.0%    0.0%    0.0%    0.0%    0.0%    0.0%    0.0%    0.0%    0.0%    0.0%    0.0%    0.0%    0.0%    0.0%    0.0%    0.0%    0.0%    0.0%    0.0%    0.0%    0.0%    0.0%    0.0%    0.0%    0.0%    0.0%    0.0%    0.0%    0.0%    0.0%    0.0%    0.0%    0.0%    0.0%    0.0%    0.0%    0.0%    0.0%    0.0%    0.0%    0.0%    0.0%    0.0%    0.0%    0.0%    0.0%    0.0%    0.0%    0.0%    0.0%    0.0%    0.0%    0.0%    0.0%    0.0%    0.0%    0.0%    0.0%    0.0%    0.0%    0.0%    0.0%    0.0%    0.0%    0.0%    0.0%    0.0%    0.0%    0.0%    0.0%    0.0%    0.0%    0.0%    0.0%    0.0%    0.0%    0.0%    0.0%    0.0%    0.0%    0.0%    0.0%    0.0%    0.0%    0.0%    0.0%    0.0%    0.0%    0.0%    0.0%    0.0%    0.0%    0.0%    0.0%    0.0%    0.0%    0.0%    0.0%    0.0%    0.0%    0.0%    0.0%    0.0%    0.0%    0.0%    0.0%    0.0%        k__Bacteria; p__Chlorobi      17    0.0%    0.0%    0.0%    0.0%    0.0%    0.0%    0.0%    0.0%    0.0%    0.0%    0.0%    0.0%    0.0%    0.0%    0.0%    0.0%    0.0%    0.0%    0.0%    0.0%    0.0%    0.0%    0.0%    0.0%    0.0%    0.0%    0.0%    0.0%    0.0%    0.0%    0.0%    0.0%    0.0%    0.0%    0.0%    0.0%    0.0%    0.0%    0.0%    0.0%    0.0%    0.0%    0.0%    0.0%    0.0%    0.0%    0.0%    0.0%    0.0%    0.0%    0.0%    0.0%    0.0%    0.0%    0.0%    0.0%    0.0%    0.0%    0.0%    0.0%    0.0%    0.0%    0.0%    0.0%    0.0%    0.0%    0.0%    0.0%    0.0%    0.0%    0.0%    0.0%    0.0%    0.0%    0.0%    0.0%    0.0%    0.0%    0.0%    0.0%    0.0%    0.0%    0.0%    0.0%    0.0%    0.0%    0.0%    0.0%    0.0%    0.0%    0.0%    0.0%    0.0%    0.0%    0.0%    0.0%    0.0%    0.0%    0.0%    0.0%    0.0%    0.0%    0.0%    0.0%    0.0%    0.0%    0.0%    0.0%    0.0%    0.0%    0.0%    0.0%    0.0%    0.0%    0.0%    0.0%    0.0%    0.0%    0.0%    0.0%    0.0%    0.0%    0.0%    0.0%    0.0%    0.0%    0.0%    0.0%    0.0%    0.0%    0.0%    0.0%    0.0%    0.0%    0.0%    0.0%    0.0%    0.0%    0.0%    0.0%    0.0%    0.0%    0.0%    0.0%    0.0%    0.0%    0.0%    0.0%    0.0%    0.0%    0.0%    0.0%    0.0%    0.0%    0.0%    0.0%    0.0%    0.0%    0.0%    0.0%    0.0%    0.0%    0.0%    0.0%    0.0%    0.0%    0.0%    0.0%    0.0%    0.0%    0.0%    0.0%    0.0%    0.0%    0.0%    0.0%    0.0%    0.0%    0.0%    0.0%    0.0%    0.0%    0.0%    0.0%    0.0%    0.0%    0.0%    0.0%    0.0%    0.0%    0.0%    0.0%    0.0%    0.0%    0.0%    0.0%    0.0%    0.0%    0.0%    0.0%    0.0%    0.0%    0.0%    0.0%    0.0%    0.0%    0.0%    0.0%    0.0%    0.0%    0.0%    0.0%    0.0%    0.0%    0.0%    0.0%    0.0%    0.0%    0.0%    0.0%    0.0%    0.0%    0.0%    0.0%    0.0%    0.0%    0.0%    0.0%    0.0%    0.0%    0.0%    0.0%    0.0%    0.0%    0.0%    0.0%    0.0%    0.0%    0.0%    0.0%    0.0%    0.0%    0.0%    0.0%    0.0%    0.0%    0.0%    0.0%    0.0%    0.0%    0.0%    0.0%    0.0%    0.0%    0.0%    0.0%    0.0%    0.0%    0.0%    0.0%    0.0%    0.0%    0.0%    0.0%    0.0%    0.0%    0.0%    0.0%    0.0%    0.0%    0.0%    0.0%    0.0%    0.0%    0.0%    0.0%    0.0%    0.0%    0.0%    0.0%    0.0%    0.0%    0.0%    0.0%    0.0%    0.0%    0.0%    0.0%    0.0%    0.0%    0.0%    0.0%    0.0%    0.0%    0.0%    0.0%    0.0%    0.0%    0.0%    0.0%    0.0%    0.0%    0.0%    0.0%    0.0%    0.0%    0.0%    0.0%    0.0%    0.0%    0.0%    0.0%    0.0%    0.0%    0.0%    0.0%    0.0%    0.0%    0.0%    0.0%    0.0%    0.0%    0.0%    0.0%    0.0%    0.0%    0.0%    0.0%    0.0%    0.0%    0.0%    0.0%    0.0%    0.0%    0.0%    0.0%    0.0%    0.0%    0.0%    0.0%    0.0%    0.0%    0.0%    0.0%    0.0%    0.0%    0.0%    0.0%    0.0%    0.0%    0.0%    0.0%    0.0%    0.0%    0.0%    0.0%    0.0%    0.0%    0.0%    0.0%    0.0%    0.0%        k__Bacteria; p__Chloroflexi    5397    0.1%    0.0%    0.1%    0.0%    0.1%    0.0%    0.0%    0.0%    0.0%    0.0%    0.0%    0.0%    0.0%    0.1%    0.0%    0.0%    0.0%    0.0%    0.1%    0.0%    0.0%    0.1%    0.1%    0.0%    0.0%    0.0%    0.0%    0.1%    0.1%    0.1%    0.1%    0.0%    0.1%    0.0%    0.1%    0.0%    0.0%    0.0%    0.1%    0.1%    0.1%    0.1%    0.0%    0.0%    0.1%    0.0%    0.0%    0.0%    0.0%    0.0%    0.0%    0.0%    0.1%    0.0%    0.2%    0.1%    0.0%    0.1%    0.1%    0.1%    0.2%    0.1%    0.0%    0.2%    0.2%    0.0%    0.0%    0.0%    0.0%    0.0%    0.0%    0.1%    0.1%    0.1%    0.1%    0.0%    0.1%    0.2%    0.1%    0.1%    0.1%    0.0%    0.0%    0.1%    0.0%    0.0%    0.1%    0.0%    0.0%    0.1%    0.0%    0.1%    0.1%    0.1%    0.2%    0.3%    0.0%    0.1%    0.2%    0.1%    0.2%    0.1%    0.1%    0.0%    0.0%    0.2%    0.0%    0.0%    0.0%    0.1%    0.2%    0.2%    0.0%    0.0%    0.2%    0.1%    0.1%    0.1%    0.1%    0.5%    0.0%    0.1%    0.2%    0.2%    0.1%    0.6%    0.4%    0.0%    0.1%    0.6%    0.2%    0.1%    0.4%    0.1%    0.5%    0.1%    0.4%    0.2%    0.1%    0.0%    0.1%    0.2%    0.0%    0.5%    0.2%    0.0%    0.0%    0.1%    0.0%    0.1%    0.4%    0.2%    0.0%    0.0%    0.4%    0.0%    0.1%    0.3%    0.9%    0.2%    0.5%    0.1%    0.1%    0.1%    0.1%    0.0%    0.1%    0.1%    0.4%    0.3%    0.4%    0.1%    0.0%    0.0%    0.1%    0.2%    0.0%    0.2%    0.1%    0.0%    0.1%    0.0%    0.2%    0.0%    0.1%    0.0%    0.0%    0.0%    0.1%    0.0%    0.1%    0.0%    0.1%    0.2%    0.0%    0.1%    0.0%    0.0%    0.0%    0.1%    0.2%    0.0%    0.1%    0.4%    0.2%    0.3%    0.3%    0.3%    0.1%    0.1%    0.0%    0.0%    0.2%    0.1%    0.0%    0.0%    0.0%    0.1%    0.0%    0.1%    0.1%    0.0%    0.2%    0.0%    0.2%    0.0%    0.3%    0.2%    0.2%    0.1%    0.0%    0.1%    0.4%    0.1%    0.8%    0.0%    0.3%    0.1%    0.1%    0.1%    0.0%    0.1%    0.1%    0.0%    0.0%    0.1%    0.1%    0.0%    0.0%    0.1%    0.1%    0.1%    0.3%    0.2%    0.0%    0.0%    0.0%    0.1%    0.1%    0.0%    0.0%    0.1%    0.2%    0.0%    0.2%    0.1%    0.1%    0.0%    0.0%    0.3%    0.1%    0.2%    0.5%    1.0%    0.2%    0.0%    0.1%    0.1%    0.3%    0.3%    0.6%    0.1%    0.0%    0.1%    0.0%    0.1%    0.0%    0.1%    0.1%    0.0%    0.0%    0.1%    0.1%    0.3%    0.0%    0.0%    0.1%    0.1%    0.1%    0.1%    0.1%    0.0%    0.0%    0.2%    0.0%    0.1%    0.1%    0.0%    0.1%    0.1%    0.1%    0.0%    0.1%    0.1%    0.0%    0.1%    0.1%    0.1%    0.0%    0.2%    0.2%    0.1%    0.0%    0.1%    0.2%    0.1%    0.1%    0.1%    0.1%    0.2%    0.1%    0.0%    0.0%    0.0%    0.0%    0.0%    0.0%    0.1%    0.1%    0.1%    0.0%    0.0%    0.0%    0.1%    0.0%    0.0%    0.0%    0.0%    0.1%    0.0%    0.0%    0.1%    0.0%    0.1%    0.0%    0.0%    0.2%    0.0%    0.3%    0.0%    0.1%    0.1%        k__Bacteria; p__Cyanobacteria   745640   13.1%    0.9%   35.0%    0.6%    0.7%   58.9%    0.3%    0.0%    0.3%    0.1%    0.1%    0.1%    0.3%   12.1%    0.9%    0.1%    1.5%    0.2%   19.8%   27.7%   19.0%   14.9%    1.8%    2.0%    0.6%    0.1%    0.3%   41.9%    2.3%    2.3%    1.8%    0.5%   25.1%   13.1%    6.2%    0.7%    0.9%   14.4%   34.3%   24.8%   41.3%    5.9%    0.2%    0.1%    0.5%    1.2%    0.6%   12.8%    6.7%    1.6%    8.0%   33.3%   11.4%    2.3%   33.7%    3.8%   22.0%   20.5%    3.5%   32.0%   20.6%   30.9%   25.4%   10.6%   20.5%   14.1%   29.7%    8.9%   17.2%   58.2%   19.1%   12.5%   73.4%   17.2%    4.7%   77.5%   44.9%   22.2%    1.1%    2.1%   20.0%    5.3%    4.4%    0.9%    2.4%    0.1%    1.9%   52.6%   51.9%    1.9%   23.9%    5.2%    5.6%   64.7%    3.1%    3.5%   60.2%    6.5%    2.5%   16.5%   15.3%    4.1%    4.4%    0.4%    0.8%    2.4%   41.8%   39.9%   11.5%    3.8%    1.1%    8.6%    8.6%   55.6%    8.5%    1.2%    3.8%    2.1%    2.8%    6.7%    8.6%    3.6%    3.8%    6.6%    5.3%    4.3%   12.0%   31.4%    7.9%    5.5%   10.8%   41.6%   59.6%    5.1%    3.6%   11.1%    1.9%    2.6%    0.8%    2.0%   14.1%   23.0%   17.8%   45.6%    8.3%    0.7%    0.5%    7.5%   60.2%   65.6%   13.9%   10.6%   15.1%   12.4%   14.9%   53.2%    5.7%    8.6%    8.7%    6.5%    5.0%   59.0%    4.9%   59.7%   13.4%   11.2%    4.4%    3.7%    7.8%    8.1%   44.1%    5.3%    3.6%    1.1%    7.0%   31.1%   41.8%    9.0%    3.2%    5.2%    4.0%   73.2%   26.3%    4.7%    1.9%    4.2%    1.9%    0.4%    4.4%    0.7%    3.3%   18.6%    5.6%    9.4%    2.9%    0.9%   28.4%    4.2%   60.4%   38.1%   16.8%   48.3%   51.2%    6.4%   41.7%   55.9%    8.7%   11.1%    6.4%    1.1%   76.7%   16.8%   19.0%    4.1%    0.7%    4.8%    0.0%   11.6%    2.2%    3.5%    0.9%   38.1%   21.9%   34.0%    8.4%    1.9%   33.6%    3.8%   35.0%    9.4%    3.7%    4.2%    9.3%   48.8%   20.5%   68.1%   37.7%    2.1%    4.3%    0.7%    1.4%    0.4%    0.4%    0.3%    0.6%    5.0%    1.8%    0.7%    7.1%    1.8%    0.5%    1.6%    4.6%    9.9%   27.8%    7.6%   32.1%   67.5%   40.3%    3.3%   50.0%    5.4%    2.3%    0.5%    9.2%    5.0%    2.3%   43.3%    6.4%    4.7%    1.0%   10.7%    4.7%    2.1%    1.9%    0.4%    5.8%    1.1%   16.6%    8.5%    2.9%    2.1%    1.4%   12.8%   27.6%    1.0%    0.7%    0.3%   31.4%    9.8%    2.1%    0.4%    0.7%    1.6%    0.5%    2.8%    0.7%    0.2%    0.3%    1.8%   27.7%    6.0%    1.4%    1.2%   11.0%    0.5%    1.2%    0.8%   13.3%    2.3%    1.4%   40.4%    1.7%    1.7%    1.7%    1.4%    1.2%   82.7%    0.0%    1.0%    0.7%    1.6%    1.9%   50.0%   17.2%    3.3%    1.6%    3.8%    0.7%    2.7%    1.3%    0.4%    0.9%    2.9%    0.7%    1.0%    0.2%    1.1%    3.7%   11.2%    0.3%    9.6%    0.7%   10.4%    1.0%    0.4%   19.8%    0.3%    7.0%    3.6%    6.2%    1.7%    1.5%    0.5%    6.2%   23.6%    1.7%    0.1%    2.5%    2.0%    1.0%    4.5%        k__Bacteria; p__Deferribacteres      68    0.0%    0.0%    0.0%    0.0%    0.0%    0.0%    0.0%    0.0%    0.0%    0.0%    0.0%    0.0%    0.0%    0.0%    0.0%    0.0%    0.0%    0.0%    0.0%    0.0%    0.0%    0.0%    0.0%    0.0%    0.0%    0.0%    0.0%    0.0%    0.0%    0.0%    0.0%    0.0%    0.0%    0.0%    0.0%    0.0%    0.0%    0.0%    0.0%    0.0%    0.0%    0.0%    0.0%    0.0%    0.0%    0.0%    0.0%    0.0%    0.0%    0.0%    0.0%    0.0%    0.0%    0.0%    0.1%    0.0%    0.0%    0.0%    0.0%    0.0%    0.0%    0.0%    0.0%    0.0%    0.0%    0.0%    0.0%    0.0%    0.0%    0.0%    0.0%    0.0%    0.0%    0.0%    0.0%    0.0%    0.0%    0.0%    0.0%    0.0%    0.0%    0.0%    0.0%    0.0%    0.0%    0.0%    0.0%    0.0%    0.0%    0.0%    0.0%    0.0%    0.0%    0.0%    0.0%    0.0%    0.0%    0.0%    0.0%    0.0%    0.0%    0.0%    0.0%    0.0%    0.0%    0.0%    0.0%    0.0%    0.0%    0.0%    0.0%    0.0%    0.0%    0.0%    0.0%    0.0%    0.0%    0.0%    0.0%    0.0%    0.0%    0.0%    0.0%    0.0%    0.0%    0.0%    0.0%    0.0%    0.0%    0.0%    0.0%    0.0%    0.0%    0.0%    0.0%    0.0%    0.0%    0.0%    0.0%    0.0%    0.0%    0.0%    0.0%    0.0%    0.0%    0.0%    0.0%    0.0%    0.0%    0.0%    0.0%    0.0%    0.0%    0.0%    0.0%    0.0%    0.0%    0.0%    0.0%    0.0%    0.0%    0.0%    0.0%    0.0%    0.0%    0.0%    0.0%    0.0%    0.0%    0.0%    0.0%    0.0%    0.0%    0.0%    0.0%    0.0%    0.0%    0.0%    0.0%    0.0%    0.0%    0.0%    0.0%    0.0%    0.0%    0.0%    0.0%    0.0%    0.0%    0.0%    0.0%    0.0%    0.0%    0.0%    0.0%    0.0%    0.0%    0.0%    0.0%    0.0%    0.0%    0.0%    0.0%    0.0%    0.0%    0.0%    0.0%    0.0%    0.0%    0.0%    0.0%    0.0%    0.0%    0.0%    0.0%    0.0%    0.0%    0.0%    0.0%    0.0%    0.0%    0.0%    0.0%    0.0%    0.0%    0.0%    0.0%    0.0%    0.0%    0.0%    0.0%    0.0%    0.0%    0.0%    0.0%    0.0%    0.0%    0.0%    0.0%    0.0%    0.0%    0.0%    0.0%    0.0%    0.0%    0.0%    0.0%    0.0%    0.0%    0.0%    0.0%    0.0%    0.0%    0.0%    0.0%    0.0%    0.0%    0.0%    0.0%    0.0%    0.0%    0.0%    0.0%    0.0%    0.0%    0.0%    0.0%    0.0%    0.0%    0.0%    0.0%    0.0%    0.0%    0.0%    0.0%    0.0%    0.0%    0.0%    0.0%    0.0%    0.0%    0.0%    0.0%    0.0%    0.0%    0.0%    0.0%    0.0%    0.0%    0.0%    0.0%    0.0%    0.0%    0.0%    0.0%    0.0%    0.0%    0.0%    0.0%    0.0%    0.0%    0.0%    0.0%    0.0%    0.0%    0.0%    0.0%    0.0%    0.0%    0.0%    0.0%    0.0%    0.0%    0.0%    0.0%    0.0%    0.0%    0.0%    0.0%    0.0%    0.0%    0.0%    0.0%    0.0%    0.0%    0.0%    0.0%    0.0%    0.0%    0.0%    0.0%    0.0%    0.0%    0.0%    0.0%    0.0%    0.0%    0.0%    0.0%    0.0%    0.0%    0.0%    0.0%    0.0%    0.0%    0.0%    0.0%    0.0%    0.0%    0.0%    0.0%    0.0%    0.0%    0.0%    0.0%    0.0%    0.0%    0.0%    0.0%    0.0%    0.0%    0.0%        k__Bacteria; p__Elusimicrobia      12    0.0%    0.0%    0.0%    0.0%    0.0%    0.0%    0.0%    0.0%    0.0%    0.0%    0.0%    0.0%    0.0%    0.0%    0.0%    0.0%    0.0%    0.0%    0.0%    0.0%    0.0%    0.0%    0.0%    0.0%    0.0%    0.0%    0.0%    0.0%    0.0%    0.0%    0.0%    0.0%    0.0%    0.0%    0.0%    0.0%    0.0%    0.0%    0.0%    0.0%    0.0%    0.0%    0.0%    0.0%    0.0%    0.0%    0.0%    0.0%    0.0%    0.0%    0.0%    0.0%    0.0%    0.0%    0.0%    0.0%    0.0%    0.0%    0.0%    0.0%    0.0%    0.0%    0.0%    0.0%    0.0%    0.0%    0.0%    0.0%    0.0%    0.0%    0.0%    0.0%    0.0%    0.0%    0.0%    0.0%    0.0%    0.0%    0.0%    0.0%    0.0%    0.0%    0.0%    0.0%    0.0%    0.0%    0.0%    0.0%    0.0%    0.0%    0.0%    0.0%    0.0%    0.0%    0.0%    0.0%    0.0%    0.0%    0.0%    0.0%    0.0%    0.0%    0.0%    0.0%    0.0%    0.0%    0.0%    0.0%    0.0%    0.0%    0.0%    0.0%    0.0%    0.0%    0.0%    0.0%    0.0%    0.0%    0.0%    0.0%    0.0%    0.0%    0.0%    0.0%    0.0%    0.0%    0.0%    0.0%    0.0%    0.0%    0.0%    0.0%    0.0%    0.0%    0.0%    0.0%    0.0%    0.0%    0.0%    0.0%    0.0%    0.0%    0.0%    0.0%    0.0%    0.0%    0.0%    0.0%    0.0%    0.0%    0.0%    0.0%    0.0%    0.0%    0.0%    0.0%    0.0%    0.0%    0.0%    0.0%    0.0%    0.0%    0.0%    0.0%    0.0%    0.0%    0.0%    0.0%    0.0%    0.0%    0.0%    0.0%    0.0%    0.0%    0.0%    0.0%    0.0%    0.0%    0.0%    0.0%    0.0%    0.0%    0.0%    0.0%    0.0%    0.0%    0.0%    0.0%    0.0%    0.0%    0.0%    0.0%    0.0%    0.0%    0.0%    0.0%    0.0%    0.0%    0.0%    0.0%    0.0%    0.0%    0.0%    0.0%    0.0%    0.0%    0.0%    0.0%    0.0%    0.0%    0.0%    0.0%    0.0%    0.0%    0.0%    0.0%    0.0%    0.0%    0.0%    0.0%    0.0%    0.0%    0.0%    0.0%    0.0%    0.0%    0.0%    0.0%    0.0%    0.0%    0.0%    0.0%    0.0%    0.0%    0.0%    0.0%    0.0%    0.0%    0.0%    0.0%    0.0%    0.0%    0.0%    0.0%    0.0%    0.0%    0.0%    0.0%    0.0%    0.0%    0.0%    0.0%    0.0%    0.0%    0.0%    0.0%    0.0%    0.0%    0.0%    0.0%    0.0%    0.0%    0.0%    0.0%    0.0%    0.0%    0.0%    0.0%    0.0%    0.0%    0.0%    0.0%    0.0%    0.0%    0.0%    0.0%    0.0%    0.0%    0.0%    0.0%    0.0%    0.0%    0.0%    0.0%    0.0%    0.0%    0.0%    0.0%    0.0%    0.0%    0.0%    0.0%    0.0%    0.0%    0.0%    0.0%    0.0%    0.0%    0.0%    0.0%    0.0%    0.0%    0.0%    0.0%    0.0%    0.0%    0.0%    0.0%    0.0%    0.0%    0.0%    0.0%    0.0%    0.0%    0.0%    0.0%    0.0%    0.0%    0.0%    0.0%    0.0%    0.0%    0.0%    0.0%    0.0%    0.0%    0.0%    0.0%    0.0%    0.0%    0.0%    0.0%    0.0%    0.0%    0.0%    0.0%    0.0%    0.0%    0.0%    0.0%    0.0%    0.0%    0.0%    0.0%    0.0%    0.0%    0.0%    0.0%    0.0%    0.0%    0.0%    0.0%    0.0%    0.0%    0.0%    0.0%    0.0%    0.0%    0.0%    0.0%    0.0%    0.0%        k__Bacteria; p__Fibrobacteres       1    0.0%    0.0%    0.0%    0.0%    0.0%    0.0%    0.0%    0.0%    0.0%    0.0%    0.0%    0.0%    0.0%    0.0%    0.0%    0.0%    0.0%    0.0%    0.0%    0.0%    0.0%    0.0%    0.0%    0.0%    0.0%    0.0%    0.0%    0.0%    0.0%    0.0%    0.0%    0.0%    0.0%    0.0%    0.0%    0.0%    0.0%    0.0%    0.0%    0.0%    0.0%    0.0%    0.0%    0.0%    0.0%    0.0%    0.0%    0.0%    0.0%    0.0%    0.0%    0.0%    0.0%    0.0%    0.0%    0.0%    0.0%    0.0%    0.0%    0.0%    0.0%    0.0%    0.0%    0.0%    0.0%    0.0%    0.0%    0.0%    0.0%    0.0%    0.0%    0.0%    0.0%    0.0%    0.0%    0.0%    0.0%    0.0%    0.0%    0.0%    0.0%    0.0%    0.0%    0.0%    0.0%    0.0%    0.0%    0.0%    0.0%    0.0%    0.0%    0.0%    0.0%    0.0%    0.0%    0.0%    0.0%    0.0%    0.0%    0.0%    0.0%    0.0%    0.0%    0.0%    0.0%    0.0%    0.0%    0.0%    0.0%    0.0%    0.0%    0.0%    0.0%    0.0%    0.0%    0.0%    0.0%    0.0%    0.0%    0.0%    0.0%    0.0%    0.0%    0.0%    0.0%    0.0%    0.0%    0.0%    0.0%    0.0%    0.0%    0.0%    0.0%    0.0%    0.0%    0.0%    0.0%    0.0%    0.0%    0.0%    0.0%    0.0%    0.0%    0.0%    0.0%    0.0%    0.0%    0.0%    0.0%    0.0%    0.0%    0.0%    0.0%    0.0%    0.0%    0.0%    0.0%    0.0%    0.0%    0.0%    0.0%    0.0%    0.0%    0.0%    0.0%    0.0%    0.0%    0.0%    0.0%    0.0%    0.0%    0.0%    0.0%    0.0%    0.0%    0.0%    0.0%    0.0%    0.0%    0.0%    0.0%    0.0%    0.0%    0.0%    0.0%    0.0%    0.0%    0.0%    0.0%    0.0%    0.0%    0.0%    0.0%    0.0%    0.0%    0.0%    0.0%    0.0%    0.0%    0.0%    0.0%    0.0%    0.0%    0.0%    0.0%    0.0%    0.0%    0.0%    0.0%    0.0%    0.0%    0.0%    0.0%    0.0%    0.0%    0.0%    0.0%    0.0%    0.0%    0.0%    0.0%    0.0%    0.0%    0.0%    0.0%    0.0%    0.0%    0.0%    0.0%    0.0%    0.0%    0.0%    0.0%    0.0%    0.0%    0.0%    0.0%    0.0%    0.0%    0.0%    0.0%    0.0%    0.0%    0.0%    0.0%    0.0%    0.0%    0.0%    0.0%    0.0%    0.0%    0.0%    0.0%    0.0%    0.0%    0.0%    0.0%    0.0%    0.0%    0.0%    0.0%    0.0%    0.0%    0.0%    0.0%    0.0%    0.0%    0.0%    0.0%    0.0%    0.0%    0.0%    0.0%    0.0%    0.0%    0.0%    0.0%    0.0%    0.0%    0.0%    0.0%    0.0%    0.0%    0.0%    0.0%    0.0%    0.0%    0.0%    0.0%    0.0%    0.0%    0.0%    0.0%    0.0%    0.0%    0.0%    0.0%    0.0%    0.0%    0.0%    0.0%    0.0%    0.0%    0.0%    0.0%    0.0%    0.0%    0.0%    0.0%    0.0%    0.0%    0.0%    0.0%    0.0%    0.0%    0.0%    0.0%    0.0%    0.0%    0.0%    0.0%    0.0%    0.0%    0.0%    0.0%    0.0%    0.0%    0.0%    0.0%    0.0%    0.0%    0.0%    0.0%    0.0%    0.0%    0.0%    0.0%    0.0%    0.0%    0.0%    0.0%    0.0%    0.0%    0.0%    0.0%    0.0%    0.0%    0.0%    0.0%    0.0%    0.0%    0.0%    0.0%    0.0%    0.0%    0.0%    0.0%    0.0%    0.0%    0.0%    0.0%    0.0%        k__Bacteria; p__Firmicutes   2057011   36.0%   32.7%   19.5%   39.4%   33.2%    4.6%   27.1%   11.3%   38.7%   40.1%   43.8%   19.1%   83.4%   28.5%   31.5%   38.3%   52.9%   53.1%   12.0%   25.9%   29.3%   33.9%   43.9%   41.5%   47.6%   43.7%   55.9%   21.6%   54.9%   46.1%   42.7%   47.3%   38.2%   18.9%   38.5%   31.5%   40.4%   34.6%   26.4%   20.8%   21.4%   32.0%   28.2%   26.8%   28.9%   41.5%   58.0%   24.1%   34.9%   44.7%   21.3%   21.4%   34.5%   35.6%   25.6%   40.3%   28.4%   29.4%   49.1%   33.0%   44.7%   36.4%   33.5%   59.2%   25.1%   56.9%    2.9%   47.5%   10.8%   23.4%    4.2%   30.7%   10.9%   35.7%   60.8%   14.4%   21.0%   34.0%   54.1%   49.5%   34.5%   23.7%   58.9%   45.2%   35.4%   51.4%   48.3%    2.1%   11.1%   51.9%    7.8%   60.2%   48.7%   18.5%   35.7%   46.2%   19.4%   50.4%   51.9%   31.7%   42.9%   54.0%   51.5%   52.9%   66.2%   40.1%    6.8%    0.9%   51.8%   45.3%   50.4%   47.9%   31.6%   21.4%   46.9%   55.4%   51.9%   61.8%   51.5%   11.1%    1.6%   57.2%   49.2%   35.9%   23.7%   18.9%   30.2%    8.8%   47.3%   36.6%   44.3%   27.6%    3.2%   55.3%   37.0%   38.1%   27.8%   30.9%   14.2%   21.9%   38.1%   31.0%    3.3%   10.0%   43.0%   34.2%   64.0%   59.0%    2.9%    7.9%   27.0%   42.2%   60.3%   18.3%   36.9%   29.2%   34.7%   34.6%   24.8%   39.0%   17.0%    5.3%   36.6%    8.6%   27.8%   39.8%   43.4%   48.8%   29.5%   36.0%   19.0%   54.0%   65.2%   52.8%   46.9%   23.5%    9.0%   48.0%   60.8%   60.6%   58.8%   10.3%   32.2%   53.9%   49.3%   63.8%   56.0%   55.8%   41.9%   56.1%   49.0%   26.7%   51.2%   37.9%   53.0%   61.4%   43.1%   41.6%   15.0%   18.6%   23.7%    9.5%   28.9%   24.3%   11.5%   10.4%   33.5%   41.8%   43.5%   49.1%    7.4%   27.9%   33.1%   48.9%   54.6%   43.5%    2.5%   46.5%   40.8%   46.9%   31.4%    5.3%    7.9%   17.3%   49.3%   53.7%   27.6%   38.6%   22.7%   48.9%   56.4%   45.0%   14.6%   22.7%   25.4%    3.5%   20.2%   48.1%   40.7%   72.6%   60.4%   69.4%   58.3%   82.9%   39.1%   54.6%   43.3%   61.3%   42.0%   56.4%   15.0%   32.9%   28.2%   14.6%    3.7%   36.1%    6.2%   14.2%   16.1%   35.9%   19.8%   38.5%   48.8%   77.7%   42.0%   10.8%   30.3%    4.9%   32.7%   28.8%   37.3%   32.4%   26.5%   33.0%   39.1%   37.0%   41.0%   40.1%   14.8%   29.8%   51.1%   43.5%   60.5%   49.3%   39.5%   37.8%   35.8%   47.4%   32.8%   62.0%   46.0%   40.7%   45.0%   20.0%   58.2%   50.9%   36.2%   33.5%   42.6%   48.6%   28.1%   23.4%   41.5%   46.6%   62.7%   49.6%   54.8%   67.8%   19.8%   44.7%   61.4%   13.8%   55.1%   76.9%   54.5%   29.6%   38.1%    6.9%   43.8%   36.9%   47.2%   47.9%   57.3%   15.8%   29.2%   45.4%   53.8%   48.9%   42.0%   54.4%   71.4%   65.7%   63.8%   71.6%   60.6%   52.5%   42.6%   55.5%   54.4%   31.3%   21.3%   13.6%   48.0%   42.8%   42.5%   55.7%   35.3%   42.9%   39.7%   33.6%   43.7%   41.2%   58.2%   46.3%   59.1%    0.4%   58.3%   96.6%   57.0%   47.8%   84.7%   51.1%        k__Bacteria; p__Fusobacteria   139776    2.4%   17.1%    8.4%    6.9%    2.1%    0.3%   19.6%    1.7%    0.6%    2.1%    4.0%    0.1%    0.2%    0.4%   15.1%    6.9%    1.7%    3.6%    0.5%    8.8%    1.1%    1.4%    1.8%    0.4%    0.2%    0.1%    0.3%    0.2%    2.6%    1.4%    0.6%    0.3%    3.6%   19.6%    2.1%    9.0%    6.9%    0.4%    4.5%    3.3%    4.2%    3.7%    8.4%   14.4%   14.9%   13.9%    2.4%    1.2%    8.1%    9.3%    1.7%    0.6%    0.4%    2.4%    2.9%    4.5%    2.2%    1.3%    5.1%    0.7%    1.8%    1.2%    2.8%    1.7%    3.9%    1.0%    0.1%    1.8%    0.6%    0.9%    0.6%    1.6%    0.9%    2.8%    1.0%    0.3%    0.7%    4.1%    1.4%    3.3%    2.8%    0.7%    1.5%    4.3%    1.0%    0.2%    2.0%    0.0%    0.4%    1.8%    0.0%    0.6%    1.4%    0.7%    2.0%    1.6%    0.2%    1.9%    2.6%    1.2%    0.7%    0.9%    0.5%    0.2%    0.4%    5.4%    0.1%    0.0%    0.3%    1.3%    1.0%    2.2%    6.8%    0.8%    1.5%    1.0%    0.6%    0.4%    1.8%    0.5%    0.0%    0.6%    1.1%    3.0%    0.5%    0.8%    0.9%    0.0%    0.6%    0.8%    1.1%    0.5%    0.1%    0.7%    1.6%    0.6%    1.1%    0.5%    0.4%    0.0%    2.3%    1.8%    0.1%    0.9%    1.1%   12.2%    1.5%    1.1%    0.1%    0.6%    2.2%    1.0%    1.5%    0.4%    1.6%    0.2%    0.5%    2.9%    7.3%    2.1%    6.8%    0.7%    0.9%    1.1%    3.2%    0.8%    1.6%    1.4%    0.7%    1.6%    0.5%    1.0%    0.9%    0.6%    1.3%    1.4%    0.3%    4.0%    0.6%    1.2%    3.5%    0.2%    0.9%    0.8%    0.4%    0.9%    1.2%    0.4%    1.4%    0.6%    0.4%    0.2%    0.8%    2.3%    0.7%    0.2%    0.5%    5.7%    0.1%    0.6%    0.3%    0.4%    0.7%    1.0%    0.5%    0.6%    1.0%    1.5%    0.8%    2.3%    0.6%    2.4%    0.9%    0.4%    2.5%    0.5%    0.0%    0.2%    1.5%    1.5%    0.8%    0.2%    0.4%    0.7%    0.9%    0.8%    0.5%    0.9%    1.3%    1.1%    0.2%    0.9%    0.2%    0.7%    0.8%    0.0%    0.5%    1.2%    0.5%    0.2%    0.9%    0.2%    0.3%    0.3%    0.3%    0.6%    0.3%    0.2%    0.6%    0.0%    0.4%    0.5%    2.7%    2.8%    0.2%    1.4%    0.6%    0.1%    0.4%    0.5%    0.6%    1.0%    1.4%    0.9%    1.0%    0.2%    0.8%    0.0%    0.4%    0.7%    1.4%    0.8%    0.3%    0.3%    0.2%    0.2%    1.0%    1.0%    1.6%    0.4%    0.4%    0.2%    0.2%    0.7%    0.9%    0.5%    0.9%    0.8%    0.5%    0.2%    0.3%    2.0%    0.7%    0.4%    0.6%    0.6%    0.6%    1.1%    2.3%    1.7%    0.6%    0.7%    0.7%    1.0%    0.7%    0.9%    1.5%    1.3%    0.6%    0.6%    1.3%    0.1%    2.6%    0.4%    0.5%    1.8%    1.8%    0.2%    0.2%    1.7%    2.6%    1.1%    0.6%    1.5%    0.4%    0.6%    1.1%    0.8%    3.5%    0.9%    0.5%    2.8%    0.8%    0.5%    0.1%    0.1%    0.1%    0.6%    0.6%    0.7%    0.5%    0.1%    0.5%    1.1%    0.4%    0.5%    0.4%    1.1%    0.2%    0.3%    0.9%    0.7%    0.4%    0.5%    0.3%    0.0%    0.8%    0.0%    0.1%    0.0%    0.3%    0.8%        k__Bacteria; p__GN02       1    0.0%    0.0%    0.0%    0.0%    0.0%    0.0%    0.0%    0.0%    0.0%    0.0%    0.0%    0.0%    0.0%    0.0%    0.0%    0.0%    0.0%    0.0%    0.0%    0.0%    0.0%    0.0%    0.0%    0.0%    0.0%    0.0%    0.0%    0.0%    0.0%    0.0%    0.0%    0.0%    0.0%    0.0%    0.0%    0.0%    0.0%    0.0%    0.0%    0.0%    0.0%    0.0%    0.0%    0.0%    0.0%    0.0%    0.0%    0.0%    0.0%    0.0%    0.0%    0.0%    0.0%    0.0%    0.0%    0.0%    0.0%    0.0%    0.0%    0.0%    0.0%    0.0%    0.0%    0.0%    0.0%    0.0%    0.0%    0.0%    0.0%    0.0%    0.0%    0.0%    0.0%    0.0%    0.0%    0.0%    0.0%    0.0%    0.0%    0.0%    0.0%    0.0%    0.0%    0.0%    0.0%    0.0%    0.0%    0.0%    0.0%    0.0%    0.0%    0.0%    0.0%    0.0%    0.0%    0.0%    0.0%    0.0%    0.0%    0.0%    0.0%    0.0%    0.0%    0.0%    0.0%    0.0%    0.0%    0.0%    0.0%    0.0%    0.0%    0.0%    0.0%    0.0%    0.0%    0.0%    0.0%    0.0%    0.0%    0.0%    0.0%    0.0%    0.0%    0.0%    0.0%    0.0%    0.0%    0.0%    0.0%    0.0%    0.0%    0.0%    0.0%    0.0%    0.0%    0.0%    0.0%    0.0%    0.0%    0.0%    0.0%    0.0%    0.0%    0.0%    0.0%    0.0%    0.0%    0.0%    0.0%    0.0%    0.0%    0.0%    0.0%    0.0%    0.0%    0.0%    0.0%    0.0%    0.0%    0.0%    0.0%    0.0%    0.0%    0.0%    0.0%    0.0%    0.0%    0.0%    0.0%    0.0%    0.0%    0.0%    0.0%    0.0%    0.0%    0.0%    0.0%    0.0%    0.0%    0.0%    0.0%    0.0%    0.0%    0.0%    0.0%    0.0%    0.0%    0.0%    0.0%    0.0%    0.0%    0.0%    0.0%    0.0%    0.0%    0.0%    0.0%    0.0%    0.0%    0.0%    0.0%    0.0%    0.0%    0.0%    0.0%    0.0%    0.0%    0.0%    0.0%    0.0%    0.0%    0.0%    0.0%    0.0%    0.0%    0.0%    0.0%    0.0%    0.0%    0.0%    0.0%    0.0%    0.0%    0.0%    0.0%    0.0%    0.0%    0.0%    0.0%    0.0%    0.0%    0.0%    0.0%    0.0%    0.0%    0.0%    0.0%    0.0%    0.0%    0.0%    0.0%    0.0%    0.0%    0.0%    0.0%    0.0%    0.0%    0.0%    0.0%    0.0%    0.0%    0.0%    0.0%    0.0%    0.0%    0.0%    0.0%    0.0%    0.0%    0.0%    0.0%    0.0%    0.0%    0.0%    0.0%    0.0%    0.0%    0.0%    0.0%    0.0%    0.0%    0.0%    0.0%    0.0%    0.0%    0.0%    0.0%    0.0%    0.0%    0.0%    0.0%    0.0%    0.0%    0.0%    0.0%    0.0%    0.0%    0.0%    0.0%    0.0%    0.0%    0.0%    0.0%    0.0%    0.0%    0.0%    0.0%    0.0%    0.0%    0.0%    0.0%    0.0%    0.0%    0.0%    0.0%    0.0%    0.0%    0.0%    0.0%    0.0%    0.0%    0.0%    0.0%    0.0%    0.0%    0.0%    0.0%    0.0%    0.0%    0.0%    0.0%    0.0%    0.0%    0.0%    0.0%    0.0%    0.0%    0.0%    0.0%    0.0%    0.0%    0.0%    0.0%    0.0%    0.0%    0.0%    0.0%    0.0%    0.0%    0.0%    0.0%    0.0%    0.0%    0.0%    0.0%    0.0%    0.0%    0.0%    0.0%    0.0%    0.0%    0.0%    0.0%    0.0%    0.0%    0.0%    0.0%    0.0%    0.0%    0.0%    0.0%    0.0%        k__Bacteria; p__Gemmatimonadetes    1336    0.0%    0.0%    0.0%    0.0%    0.0%    0.0%    0.0%    0.0%    0.0%    0.0%    0.0%    0.0%    0.0%    0.0%    0.0%    0.0%    0.0%    0.0%    0.0%    0.0%    0.0%    0.0%    0.0%    0.0%    0.0%    0.0%    0.0%    0.0%    0.1%    0.0%    0.0%    0.0%    0.0%    0.0%    0.0%    0.0%    0.0%    0.0%    0.0%    0.0%    0.0%    0.0%    0.0%    0.0%    0.0%    0.0%    0.0%    0.0%    0.0%    0.0%    0.0%    0.0%    0.0%    0.0%    0.0%    0.0%    0.0%    0.0%    0.0%    0.0%    0.0%    0.0%    0.0%    0.0%    0.0%    0.0%    0.0%    0.0%    0.0%    0.0%    0.0%    0.0%    0.0%    0.0%    0.1%    0.0%    0.1%    0.0%    0.0%    0.0%    0.0%    0.0%    0.0%    0.0%    0.0%    0.0%    0.0%    0.0%    0.0%    0.0%    0.0%    0.0%    0.0%    0.0%    0.0%    0.0%    0.0%    0.0%    0.0%    0.0%    0.0%    0.0%    0.0%    0.0%    0.0%    0.1%    0.0%    0.0%    0.0%    0.0%    0.1%    0.0%    0.0%    0.0%    0.0%    0.0%    0.0%    0.0%    0.0%    0.1%    0.0%    0.0%    0.1%    0.1%    0.0%    0.1%    0.3%    0.0%    0.1%    0.1%    0.1%    0.0%    0.1%    0.0%    0.1%    0.0%    0.1%    0.0%    0.0%    0.0%    0.0%    0.0%    0.0%    0.1%    0.1%    0.0%    0.0%    0.0%    0.0%    0.0%    0.1%    0.0%    0.0%    0.0%    0.1%    0.0%    0.0%    0.0%    0.2%    0.1%    0.2%    0.0%    0.0%    0.0%    0.0%    0.1%    0.0%    0.0%    0.1%    0.0%    0.0%    0.0%    0.0%    0.0%    0.0%    0.1%    0.0%    0.0%    0.0%    0.0%    0.0%    0.0%    0.0%    0.0%    0.0%    0.0%    0.0%    0.0%    0.0%    0.0%    0.1%    0.0%    0.0%    0.1%    0.0%    0.0%    0.0%    0.0%    0.0%    0.0%    0.1%    0.0%    0.0%    0.1%    0.0%    0.0%    0.0%    0.0%    0.0%    0.1%    0.0%    0.0%    0.0%    0.0%    0.0%    0.0%    0.0%    0.0%    0.2%    0.1%    0.0%    0.0%    0.0%    0.0%    0.1%    0.0%    0.0%    0.1%    0.0%    0.0%    0.0%    0.0%    0.1%    0.0%    0.1%    0.0%    0.0%    0.0%    0.1%    0.0%    0.0%    0.0%    0.0%    0.0%    0.0%    0.0%    0.0%    0.0%    0.0%    0.1%    0.0%    0.0%    0.2%    0.1%    0.0%    0.0%    0.0%    0.0%    0.1%    0.0%    0.0%    0.1%    0.0%    0.0%    0.0%    0.0%    0.1%    0.0%    0.0%    0.1%    0.0%    0.0%    0.1%    0.1%    0.0%    0.0%    0.0%    0.1%    0.1%    0.1%    0.1%    0.0%    0.0%    0.0%    0.0%    0.0%    0.0%    0.0%    0.0%    0.0%    0.0%    0.0%    0.0%    0.1%    0.0%    0.0%    0.0%    0.0%    0.0%    0.0%    0.0%    0.0%    0.0%    0.1%    0.0%    0.1%    0.0%    0.0%    0.0%    0.0%    0.0%    0.0%    0.0%    0.0%    0.0%    0.0%    0.0%    0.0%    0.0%    0.0%    0.0%    0.0%    0.0%    0.0%    0.0%    0.0%    0.0%    0.0%    0.0%    0.0%    0.0%    0.0%    0.0%    0.0%    0.0%    0.0%    0.0%    0.0%    0.0%    0.0%    0.0%    0.0%    0.0%    0.0%    0.0%    0.0%    0.0%    0.0%    0.0%    0.0%    0.0%    0.0%    0.0%    0.0%    0.0%    0.0%    0.4%    0.0%    0.6%    0.0%    0.0%    0.1%        k__Bacteria; p__Lentisphaerae       1    0.0%    0.0%    0.0%    0.0%    0.0%    0.0%    0.0%    0.0%    0.0%    0.0%    0.0%    0.0%    0.0%    0.0%    0.0%    0.0%    0.0%    0.0%    0.0%    0.0%    0.0%    0.0%    0.0%    0.0%    0.0%    0.0%    0.0%    0.0%    0.0%    0.0%    0.0%    0.0%    0.0%    0.0%    0.0%    0.0%    0.0%    0.0%    0.0%    0.0%    0.0%    0.0%    0.0%    0.0%    0.0%    0.0%    0.0%    0.0%    0.0%    0.0%    0.0%    0.0%    0.0%    0.0%    0.0%    0.0%    0.0%    0.0%    0.0%    0.0%    0.0%    0.0%    0.0%    0.0%    0.0%    0.0%    0.0%    0.0%    0.0%    0.0%    0.0%    0.0%    0.0%    0.0%    0.0%    0.0%    0.0%    0.0%    0.0%    0.0%    0.0%    0.0%    0.0%    0.0%    0.0%    0.0%    0.0%    0.0%    0.0%    0.0%    0.0%    0.0%    0.0%    0.0%    0.0%    0.0%    0.0%    0.0%    0.0%    0.0%    0.0%    0.0%    0.0%    0.0%    0.0%    0.0%    0.0%    0.0%    0.0%    0.0%    0.0%    0.0%    0.0%    0.0%    0.0%    0.0%    0.0%    0.0%    0.0%    0.0%    0.0%    0.0%    0.0%    0.0%    0.0%    0.0%    0.0%    0.0%    0.0%    0.0%    0.0%    0.0%    0.0%    0.0%    0.0%    0.0%    0.0%    0.0%    0.0%    0.0%    0.0%    0.0%    0.0%    0.0%    0.0%    0.0%    0.0%    0.0%    0.0%    0.0%    0.0%    0.0%    0.0%    0.0%    0.0%    0.0%    0.0%    0.0%    0.0%    0.0%    0.0%    0.0%    0.0%    0.0%    0.0%    0.0%    0.0%    0.0%    0.0%    0.0%    0.0%    0.0%    0.0%    0.0%    0.0%    0.0%    0.0%    0.0%    0.0%    0.0%    0.0%    0.0%    0.0%    0.0%    0.0%    0.0%    0.0%    0.0%    0.0%    0.0%    0.0%    0.0%    0.0%    0.0%    0.0%    0.0%    0.0%    0.0%    0.0%    0.0%    0.0%    0.0%    0.0%    0.0%    0.0%    0.0%    0.0%    0.0%    0.0%    0.0%    0.0%    0.0%    0.0%    0.0%    0.0%    0.0%    0.0%    0.0%    0.0%    0.0%    0.0%    0.0%    0.0%    0.0%    0.0%    0.0%    0.0%    0.0%    0.0%    0.0%    0.0%    0.0%    0.0%    0.0%    0.0%    0.0%    0.0%    0.0%    0.0%    0.0%    0.0%    0.0%    0.0%    0.0%    0.0%    0.0%    0.0%    0.0%    0.0%    0.0%    0.0%    0.0%    0.0%    0.0%    0.0%    0.0%    0.0%    0.0%    0.0%    0.0%    0.0%    0.0%    0.0%    0.0%    0.0%    0.0%    0.0%    0.0%    0.0%    0.0%    0.0%    0.0%    0.0%    0.0%    0.0%    0.0%    0.0%    0.0%    0.0%    0.0%    0.0%    0.0%    0.0%    0.0%    0.0%    0.0%    0.0%    0.0%    0.0%    0.0%    0.0%    0.0%    0.0%    0.0%    0.0%    0.0%    0.0%    0.0%    0.0%    0.0%    0.0%    0.0%    0.0%    0.0%    0.0%    0.0%    0.0%    0.0%    0.0%    0.0%    0.0%    0.0%    0.0%    0.0%    0.0%    0.0%    0.0%    0.0%    0.0%    0.0%    0.0%    0.0%    0.0%    0.0%    0.0%    0.0%    0.0%    0.0%    0.0%    0.0%    0.0%    0.0%    0.0%    0.0%    0.0%    0.0%    0.0%    0.0%    0.0%    0.0%    0.0%    0.0%    0.0%    0.0%    0.0%    0.0%    0.0%    0.0%    0.0%    0.0%    0.0%    0.0%    0.0%    0.0%    0.0%    0.0%    0.0%    0.0%    0.0%    0.0%    0.0%    0.0%        k__Bacteria; p__MVP-15       3    0.0%    0.0%    0.0%    0.0%    0.0%    0.0%    0.0%    0.0%    0.0%    0.0%    0.0%    0.0%    0.0%    0.0%    0.0%    0.0%    0.0%    0.0%    0.0%    0.0%    0.0%    0.0%    0.0%    0.0%    0.0%    0.0%    0.0%    0.0%    0.0%    0.0%    0.0%    0.0%    0.0%    0.0%    0.0%    0.0%    0.0%    0.0%    0.0%    0.0%    0.0%    0.0%    0.0%    0.0%    0.0%    0.0%    0.0%    0.0%    0.0%    0.0%    0.0%    0.0%    0.0%    0.0%    0.0%    0.0%    0.0%    0.0%    0.0%    0.0%    0.0%    0.0%    0.0%    0.0%    0.0%    0.0%    0.0%    0.0%    0.0%    0.0%    0.0%    0.0%    0.0%    0.0%    0.0%    0.0%    0.0%    0.0%    0.0%    0.0%    0.0%    0.0%    0.0%    0.0%    0.0%    0.0%    0.0%    0.0%    0.0%    0.0%    0.0%    0.0%    0.0%    0.0%    0.0%    0.0%    0.0%    0.0%    0.0%    0.0%    0.0%    0.0%    0.0%    0.0%    0.0%    0.0%    0.0%    0.0%    0.0%    0.0%    0.0%    0.0%    0.0%    0.0%    0.0%    0.0%    0.0%    0.0%    0.0%    0.0%    0.0%    0.0%    0.0%    0.0%    0.0%    0.0%    0.0%    0.0%    0.0%    0.0%    0.0%    0.0%    0.0%    0.0%    0.0%    0.0%    0.0%    0.0%    0.0%    0.0%    0.0%    0.0%    0.0%    0.0%    0.0%    0.0%    0.0%    0.0%    0.0%    0.0%    0.0%    0.0%    0.0%    0.0%    0.0%    0.0%    0.0%    0.0%    0.0%    0.0%    0.0%    0.0%    0.0%    0.0%    0.0%    0.0%    0.0%    0.0%    0.0%    0.0%    0.0%    0.0%    0.0%    0.0%    0.0%    0.0%    0.0%    0.0%    0.0%    0.0%    0.0%    0.0%    0.0%    0.0%    0.0%    0.0%    0.0%    0.0%    0.0%    0.0%    0.0%    0.0%    0.0%    0.0%    0.0%    0.0%    0.0%    0.0%    0.0%    0.0%    0.0%    0.0%    0.0%    0.0%    0.0%    0.0%    0.0%    0.0%    0.0%    0.0%    0.0%    0.0%    0.0%    0.0%    0.0%    0.0%    0.0%    0.0%    0.0%    0.0%    0.0%    0.0%    0.0%    0.0%    0.0%    0.0%    0.0%    0.0%    0.0%    0.0%    0.0%    0.0%    0.0%    0.0%    0.0%    0.0%    0.0%    0.0%    0.0%    0.0%    0.0%    0.0%    0.0%    0.0%    0.0%    0.0%    0.0%    0.0%    0.0%    0.0%    0.0%    0.0%    0.0%    0.0%    0.0%    0.0%    0.0%    0.0%    0.0%    0.0%    0.0%    0.0%    0.0%    0.0%    0.0%    0.0%    0.0%    0.0%    0.0%    0.0%    0.0%    0.0%    0.0%    0.0%    0.0%    0.0%    0.0%    0.0%    0.0%    0.0%    0.0%    0.0%    0.0%    0.0%    0.0%    0.0%    0.0%    0.0%    0.0%    0.0%    0.0%    0.0%    0.0%    0.0%    0.0%    0.0%    0.0%    0.0%    0.0%    0.0%    0.0%    0.0%    0.0%    0.0%    0.0%    0.0%    0.0%    0.0%    0.0%    0.0%    0.0%    0.0%    0.0%    0.0%    0.0%    0.0%    0.0%    0.0%    0.0%    0.0%    0.0%    0.0%    0.0%    0.0%    0.0%    0.0%    0.0%    0.0%    0.0%    0.0%    0.0%    0.0%    0.0%    0.0%    0.0%    0.0%    0.0%    0.0%    0.0%    0.0%    0.0%    0.0%    0.0%    0.0%    0.0%    0.0%    0.0%    0.0%    0.0%    0.0%    0.0%    0.0%    0.0%    0.0%    0.0%    0.0%    0.0%    0.0%    0.0%    0.0%    0.0%    0.0%        k__Bacteria; p__NC10      10    0.0%    0.0%    0.0%    0.0%    0.0%    0.0%    0.0%    0.0%    0.0%    0.0%    0.0%    0.0%    0.0%    0.0%    0.0%    0.0%    0.0%    0.0%    0.0%    0.0%    0.0%    0.0%    0.0%    0.0%    0.0%    0.0%    0.0%    0.0%    0.0%    0.0%    0.0%    0.0%    0.0%    0.0%    0.0%    0.0%    0.0%    0.0%    0.0%    0.0%    0.0%    0.0%    0.0%    0.0%    0.0%    0.0%    0.0%    0.0%    0.0%    0.0%    0.0%    0.0%    0.0%    0.0%    0.0%    0.0%    0.0%    0.0%    0.0%    0.0%    0.0%    0.0%    0.0%    0.0%    0.0%    0.0%    0.0%    0.0%    0.0%    0.0%    0.0%    0.0%    0.0%    0.0%    0.0%    0.0%    0.0%    0.0%    0.0%    0.0%    0.0%    0.0%    0.0%    0.0%    0.0%    0.0%    0.0%    0.0%    0.0%    0.0%    0.0%    0.0%    0.0%    0.0%    0.0%    0.0%    0.0%    0.0%    0.0%    0.0%    0.0%    0.0%    0.0%    0.0%    0.0%    0.0%    0.0%    0.0%    0.0%    0.0%    0.0%    0.0%    0.0%    0.0%    0.0%    0.0%    0.0%    0.0%    0.0%    0.0%    0.0%    0.0%    0.0%    0.0%    0.0%    0.0%    0.0%    0.0%    0.0%    0.0%    0.0%    0.0%    0.0%    0.0%    0.0%    0.0%    0.0%    0.0%    0.0%    0.0%    0.0%    0.0%    0.0%    0.0%    0.0%    0.0%    0.0%    0.0%    0.0%    0.0%    0.0%    0.0%    0.0%    0.0%    0.0%    0.0%    0.0%    0.0%    0.0%    0.0%    0.0%    0.0%    0.0%    0.0%    0.0%    0.0%    0.0%    0.0%    0.0%    0.0%    0.0%    0.0%    0.0%    0.0%    0.0%    0.0%    0.0%    0.0%    0.0%    0.0%    0.0%    0.0%    0.0%    0.0%    0.0%    0.0%    0.0%    0.0%    0.0%    0.0%    0.0%    0.0%    0.0%    0.0%    0.0%    0.0%    0.0%    0.0%    0.0%    0.0%    0.0%    0.0%    0.0%    0.0%    0.0%    0.0%    0.0%    0.0%    0.0%    0.0%    0.0%    0.0%    0.0%    0.0%    0.0%    0.0%    0.0%    0.0%    0.0%    0.0%    0.0%    0.0%    0.0%    0.0%    0.0%    0.0%    0.0%    0.0%    0.0%    0.0%    0.0%    0.0%    0.0%    0.0%    0.0%    0.0%    0.0%    0.0%    0.0%    0.0%    0.0%    0.0%    0.0%    0.0%    0.0%    0.0%    0.0%    0.0%    0.0%    0.0%    0.0%    0.0%    0.0%    0.0%    0.0%    0.0%    0.0%    0.0%    0.0%    0.0%    0.0%    0.0%    0.0%    0.0%    0.0%    0.0%    0.0%    0.0%    0.0%    0.0%    0.0%    0.0%    0.0%    0.0%    0.0%    0.0%    0.0%    0.0%    0.0%    0.0%    0.0%    0.0%    0.0%    0.0%    0.0%    0.0%    0.0%    0.0%    0.0%    0.0%    0.0%    0.0%    0.0%    0.0%    0.0%    0.0%    0.0%    0.0%    0.0%    0.0%    0.0%    0.0%    0.0%    0.0%    0.0%    0.0%    0.0%    0.0%    0.0%    0.0%    0.0%    0.0%    0.0%    0.0%    0.0%    0.0%    0.0%    0.0%    0.0%    0.0%    0.0%    0.0%    0.0%    0.0%    0.0%    0.0%    0.0%    0.0%    0.0%    0.0%    0.0%    0.0%    0.0%    0.0%    0.0%    0.0%    0.0%    0.0%    0.0%    0.0%    0.0%    0.0%    0.0%    0.0%    0.0%    0.0%    0.0%    0.0%    0.0%    0.0%    0.0%    0.0%    0.0%    0.0%    0.0%    0.0%    0.0%    0.0%    0.0%    0.0%    0.0%    0.0%        k__Bacteria; p__NKB19       2    0.0%    0.0%    0.0%    0.0%    0.0%    0.0%    0.0%    0.0%    0.0%    0.0%    0.0%    0.0%    0.0%    0.0%    0.0%    0.0%    0.0%    0.0%    0.0%    0.0%    0.0%    0.0%    0.0%    0.0%    0.0%    0.0%    0.0%    0.0%    0.0%    0.0%    0.0%    0.0%    0.0%    0.0%    0.0%    0.0%    0.0%    0.0%    0.0%    0.0%    0.0%    0.0%    0.0%    0.0%    0.0%    0.0%    0.0%    0.0%    0.0%    0.0%    0.0%    0.0%    0.0%    0.0%    0.0%    0.0%    0.0%    0.0%    0.0%    0.0%    0.0%    0.0%    0.0%    0.0%    0.0%    0.0%    0.0%    0.0%    0.0%    0.0%    0.0%    0.0%    0.0%    0.0%    0.0%    0.0%    0.0%    0.0%    0.0%    0.0%    0.0%    0.0%    0.0%    0.0%    0.0%    0.0%    0.0%    0.0%    0.0%    0.0%    0.0%    0.0%    0.0%    0.0%    0.0%    0.0%    0.0%    0.0%    0.0%    0.0%    0.0%    0.0%    0.0%    0.0%    0.0%    0.0%    0.0%    0.0%    0.0%    0.0%    0.0%    0.0%    0.0%    0.0%    0.0%    0.0%    0.0%    0.0%    0.0%    0.0%    0.0%    0.0%    0.0%    0.0%    0.0%    0.0%    0.0%    0.0%    0.0%    0.0%    0.0%    0.0%    0.0%    0.0%    0.0%    0.0%    0.0%    0.0%    0.0%    0.0%    0.0%    0.0%    0.0%    0.0%    0.0%    0.0%    0.0%    0.0%    0.0%    0.0%    0.0%    0.0%    0.0%    0.0%    0.0%    0.0%    0.0%    0.0%    0.0%    0.0%    0.0%    0.0%    0.0%    0.0%    0.0%    0.0%    0.0%    0.0%    0.0%    0.0%    0.0%    0.0%    0.0%    0.0%    0.0%    0.0%    0.0%    0.0%    0.0%    0.0%    0.0%    0.0%    0.0%    0.0%    0.0%    0.0%    0.0%    0.0%    0.0%    0.0%    0.0%    0.0%    0.0%    0.0%    0.0%    0.0%    0.0%    0.0%    0.0%    0.0%    0.0%    0.0%    0.0%    0.0%    0.0%    0.0%    0.0%    0.0%    0.0%    0.0%    0.0%    0.0%    0.0%    0.0%    0.0%    0.0%    0.0%    0.0%    0.0%    0.0%    0.0%    0.0%    0.0%    0.0%    0.0%    0.0%    0.0%    0.0%    0.0%    0.0%    0.0%    0.0%    0.0%    0.0%    0.0%    0.0%    0.0%    0.0%    0.0%    0.0%    0.0%    0.0%    0.0%    0.0%    0.0%    0.0%    0.0%    0.0%    0.0%    0.0%    0.0%    0.0%    0.0%    0.0%    0.0%    0.0%    0.0%    0.0%    0.0%    0.0%    0.0%    0.0%    0.0%    0.0%    0.0%    0.0%    0.0%    0.0%    0.0%    0.0%    0.0%    0.0%    0.0%    0.0%    0.0%    0.0%    0.0%    0.0%    0.0%    0.0%    0.0%    0.0%    0.0%    0.0%    0.0%    0.0%    0.0%    0.0%    0.0%    0.0%    0.0%    0.0%    0.0%    0.0%    0.0%    0.0%    0.0%    0.0%    0.0%    0.0%    0.0%    0.0%    0.0%    0.0%    0.0%    0.0%    0.0%    0.0%    0.0%    0.0%    0.0%    0.0%    0.0%    0.0%    0.0%    0.0%    0.0%    0.0%    0.0%    0.0%    0.0%    0.0%    0.0%    0.0%    0.0%    0.0%    0.0%    0.0%    0.0%    0.0%    0.0%    0.0%    0.0%    0.0%    0.0%    0.0%    0.0%    0.0%    0.0%    0.0%    0.0%    0.0%    0.0%    0.0%    0.0%    0.0%    0.0%    0.0%    0.0%    0.0%    0.0%    0.0%    0.0%    0.0%    0.0%    0.0%    0.0%    0.0%    0.0%    0.0%    0.0%    0.0%        k__Bacteria; p__Nitrospirae     155    0.0%    0.0%    0.0%    0.0%    0.0%    0.0%    0.0%    0.0%    0.0%    0.0%    0.0%    0.0%    0.0%    0.0%    0.0%    0.0%    0.0%    0.0%    0.0%    0.0%    0.0%    0.0%    0.0%    0.0%    0.0%    0.0%    0.0%    0.0%    0.0%    0.0%    0.0%    0.0%    0.0%    0.0%    0.0%    0.0%    0.0%    0.0%    0.0%    0.0%    0.0%    0.0%    0.0%    0.0%    0.0%    0.0%    0.0%    0.0%    0.0%    0.0%    0.0%    0.0%    0.0%    0.0%    0.0%    0.0%    0.0%    0.0%    0.0%    0.0%    0.0%    0.0%    0.0%    0.0%    0.0%    0.0%    0.0%    0.0%    0.0%    0.0%    0.0%    0.0%    0.0%    0.0%    0.0%    0.0%    0.0%    0.0%    0.0%    0.0%    0.0%    0.0%    0.0%    0.0%    0.0%    0.0%    0.0%    0.0%    0.0%    0.0%    0.0%    0.0%    0.0%    0.0%    0.0%    0.0%    0.0%    0.0%    0.0%    0.0%    0.0%    0.0%    0.0%    0.0%    0.0%    0.0%    0.0%    0.0%    0.0%    0.0%    0.0%    0.0%    0.0%    0.0%    0.0%    0.0%    0.0%    0.0%    0.0%    0.0%    0.0%    0.0%    0.0%    0.0%    0.0%    0.0%    0.0%    0.0%    0.0%    0.0%    0.0%    0.0%    0.0%    0.0%    0.0%    0.0%    0.0%    0.0%    0.0%    0.0%    0.0%    0.0%    0.0%    0.0%    0.0%    0.0%    0.0%    0.0%    0.0%    0.0%    0.0%    0.0%    0.0%    0.0%    0.0%    0.0%    0.0%    0.0%    0.0%    0.0%    0.0%    0.0%    0.0%    0.0%    0.0%    0.0%    0.0%    0.0%    0.0%    0.0%    0.0%    0.0%    0.0%    0.0%    0.0%    0.0%    0.0%    0.0%    0.0%    0.0%    0.0%    0.0%    0.0%    0.0%    0.0%    0.0%    0.0%    0.0%    0.0%    0.0%    0.0%    0.0%    0.0%    0.0%    0.0%    0.0%    0.0%    0.0%    0.0%    0.0%    0.0%    0.0%    0.0%    0.0%    0.0%    0.0%    0.0%    0.0%    0.0%    0.0%    0.0%    0.0%    0.0%    0.0%    0.0%    0.0%    0.0%    0.0%    0.0%    0.0%    0.0%    0.0%    0.0%    0.0%    0.0%    0.0%    0.0%    0.0%    0.0%    0.0%    0.0%    0.0%    0.0%    0.0%    0.0%    0.0%    0.0%    0.0%    0.0%    0.0%    0.0%    0.0%    0.0%    0.0%    0.0%    0.0%    0.0%    0.0%    0.0%    0.0%    0.0%    0.0%    0.0%    0.0%    0.0%    0.0%    0.0%    0.0%    0.0%    0.0%    0.0%    0.0%    0.0%    0.0%    0.0%    0.0%    0.0%    0.0%    0.0%    0.0%    0.0%    0.0%    0.0%    0.0%    0.0%    0.0%    0.0%    0.0%    0.0%    0.0%    0.0%    0.0%    0.0%    0.0%    0.0%    0.0%    0.0%    0.0%    0.0%    0.0%    0.0%    0.0%    0.0%    0.0%    0.0%    0.0%    0.0%    0.0%    0.0%    0.0%    0.0%    0.0%    0.0%    0.0%    0.0%    0.0%    0.0%    0.0%    0.0%    0.0%    0.0%    0.0%    0.0%    0.0%    0.0%    0.0%    0.0%    0.0%    0.0%    0.0%    0.0%    0.0%    0.0%    0.0%    0.0%    0.0%    0.0%    0.0%    0.0%    0.0%    0.0%    0.0%    0.0%    0.0%    0.0%    0.0%    0.0%    0.0%    0.0%    0.0%    0.0%    0.0%    0.0%    0.0%    0.0%    0.0%    0.0%    0.0%    0.0%    0.0%    0.0%    0.0%    0.0%    0.0%    0.0%    0.0%    0.0%    0.0%    0.0%    0.0%    0.0%    0.0%        k__Bacteria; p__OP10     140    0.0%    0.0%    0.0%    0.0%    0.0%    0.0%    0.0%    0.0%    0.0%    0.0%    0.0%    0.0%    0.0%    0.0%    0.0%    0.0%    0.0%    0.0%    0.0%    0.0%    0.0%    0.0%    0.0%    0.0%    0.0%    0.0%    0.0%    0.0%    0.0%    0.0%    0.0%    0.0%    0.0%    0.0%    0.0%    0.0%    0.0%    0.0%    0.0%    0.0%    0.0%    0.0%    0.0%    0.0%    0.0%    0.0%    0.0%    0.0%    0.0%    0.0%    0.0%    0.0%    0.0%    0.0%    0.0%    0.0%    0.0%    0.0%    0.0%    0.0%    0.0%    0.0%    0.0%    0.0%    0.0%    0.0%    0.0%    0.0%    0.0%    0.0%    0.0%    0.0%    0.0%    0.0%    0.0%    0.0%    0.0%    0.0%    0.0%    0.0%    0.0%    0.0%    0.0%    0.0%    0.0%    0.0%    0.0%    0.0%    0.0%    0.0%    0.0%    0.0%    0.0%    0.0%    0.0%    0.0%    0.0%    0.0%    0.0%    0.0%    0.0%    0.0%    0.0%    0.0%    0.0%    0.0%    0.0%    0.0%    0.0%    0.0%    0.0%    0.0%    0.0%    0.0%    0.0%    0.0%    0.0%    0.0%    0.0%    0.0%    0.0%    0.0%    0.0%    0.0%    0.0%    0.0%    0.0%    0.0%    0.0%    0.0%    0.0%    0.0%    0.0%    0.0%    0.0%    0.0%    0.0%    0.0%    0.0%    0.0%    0.0%    0.0%    0.0%    0.0%    0.0%    0.0%    0.0%    0.0%    0.0%    0.0%    0.0%    0.0%    0.0%    0.0%    0.0%    0.0%    0.0%    0.0%    0.0%    0.0%    0.0%    0.0%    0.0%    0.0%    0.0%    0.0%    0.0%    0.0%    0.0%    0.0%    0.0%    0.0%    0.0%    0.0%    0.0%    0.0%    0.0%    0.0%    0.0%    0.0%    0.0%    0.0%    0.0%    0.0%    0.0%    0.0%    0.0%    0.0%    0.0%    0.0%    0.0%    0.0%    0.0%    0.0%    0.0%    0.0%    0.0%    0.0%    0.0%    0.0%    0.0%    0.0%    0.0%    0.0%    0.0%    0.0%    0.0%    0.0%    0.0%    0.0%    0.0%    0.0%    0.0%    0.0%    0.0%    0.0%    0.0%    0.0%    0.0%    0.0%    0.0%    0.0%    0.0%    0.0%    0.0%    0.0%    0.0%    0.0%    0.0%    0.0%    0.0%    0.0%    0.0%    0.0%    0.0%    0.0%    0.0%    0.0%    0.0%    0.0%    0.0%    0.0%    0.0%    0.0%    0.0%    0.0%    0.0%    0.0%    0.0%    0.0%    0.0%    0.0%    0.0%    0.0%    0.0%    0.0%    0.0%    0.0%    0.0%    0.0%    0.0%    0.0%    0.0%    0.0%    0.0%    0.0%    0.0%    0.0%    0.0%    0.0%    0.0%    0.1%    0.0%    0.0%    0.0%    0.0%    0.0%    0.0%    0.0%    0.0%    0.0%    0.0%    0.0%    0.0%    0.0%    0.0%    0.0%    0.0%    0.0%    0.0%    0.0%    0.0%    0.0%    0.0%    0.0%    0.0%    0.0%    0.0%    0.0%    0.0%    0.0%    0.0%    0.0%    0.0%    0.0%    0.0%    0.0%    0.0%    0.0%    0.0%    0.0%    0.0%    0.0%    0.0%    0.0%    0.0%    0.0%    0.0%    0.0%    0.0%    0.0%    0.0%    0.0%    0.0%    0.0%    0.0%    0.0%    0.0%    0.0%    0.0%    0.0%    0.0%    0.0%    0.0%    0.0%    0.0%    0.0%    0.0%    0.0%    0.0%    0.0%    0.0%    0.0%    0.0%    0.0%    0.0%    0.0%    0.0%    0.0%    0.0%    0.0%    0.0%    0.0%    0.0%    0.0%    0.0%    0.0%    0.0%    0.0%    0.0%    0.0%    0.0%        k__Bacteria; p__OP11       0    0.0%    0.0%    0.0%    0.0%    0.0%    0.0%    0.0%    0.0%    0.0%    0.0%    0.0%    0.0%    0.0%    0.0%    0.0%    0.0%    0.0%    0.0%    0.0%    0.0%    0.0%    0.0%    0.0%    0.0%    0.0%    0.0%    0.0%    0.0%    0.0%    0.0%    0.0%    0.0%    0.0%    0.0%    0.0%    0.0%    0.0%    0.0%    0.0%    0.0%    0.0%    0.0%    0.0%    0.0%    0.0%    0.0%    0.0%    0.0%    0.0%    0.0%    0.0%    0.0%    0.0%    0.0%    0.0%    0.0%    0.0%    0.0%    0.0%    0.0%    0.0%    0.0%    0.0%    0.0%    0.0%    0.0%    0.0%    0.0%    0.0%    0.0%    0.0%    0.0%    0.0%    0.0%    0.0%    0.0%    0.0%    0.0%    0.0%    0.0%    0.0%    0.0%    0.0%    0.0%    0.0%    0.0%    0.0%    0.0%    0.0%    0.0%    0.0%    0.0%    0.0%    0.0%    0.0%    0.0%    0.0%    0.0%    0.0%    0.0%    0.0%    0.0%    0.0%    0.0%    0.0%    0.0%    0.0%    0.0%    0.0%    0.0%    0.0%    0.0%    0.0%    0.0%    0.0%    0.0%    0.0%    0.0%    0.0%    0.0%    0.0%    0.0%    0.0%    0.0%    0.0%    0.0%    0.0%    0.0%    0.0%    0.0%    0.0%    0.0%    0.0%    0.0%    0.0%    0.0%    0.0%    0.0%    0.0%    0.0%    0.0%    0.0%    0.0%    0.0%    0.0%    0.0%    0.0%    0.0%    0.0%    0.0%    0.0%    0.0%    0.0%    0.0%    0.0%    0.0%    0.0%    0.0%    0.0%    0.0%    0.0%    0.0%    0.0%    0.0%    0.0%    0.0%    0.0%    0.0%    0.0%    0.0%    0.0%    0.0%    0.0%    0.0%    0.0%    0.0%    0.0%    0.0%    0.0%    0.0%    0.0%    0.0%    0.0%    0.0%    0.0%    0.0%    0.0%    0.0%    0.0%    0.0%    0.0%    0.0%    0.0%    0.0%    0.0%    0.0%    0.0%    0.0%    0.0%    0.0%    0.0%    0.0%    0.0%    0.0%    0.0%    0.0%    0.0%    0.0%    0.0%    0.0%    0.0%    0.0%    0.0%    0.0%    0.0%    0.0%    0.0%    0.0%    0.0%    0.0%    0.0%    0.0%    0.0%    0.0%    0.0%    0.0%    0.0%    0.0%    0.0%    0.0%    0.0%    0.0%    0.0%    0.0%    0.0%    0.0%    0.0%    0.0%    0.0%    0.0%    0.0%    0.0%    0.0%    0.0%    0.0%    0.0%    0.0%    0.0%    0.0%    0.0%    0.0%    0.0%    0.0%    0.0%    0.0%    0.0%    0.0%    0.0%    0.0%    0.0%    0.0%    0.0%    0.0%    0.0%    0.0%    0.0%    0.0%    0.0%    0.0%    0.0%    0.0%    0.0%    0.0%    0.0%    0.0%    0.0%    0.0%    0.0%    0.0%    0.0%    0.0%    0.0%    0.0%    0.0%    0.0%    0.0%    0.0%    0.0%    0.0%    0.0%    0.0%    0.0%    0.0%    0.0%    0.0%    0.0%    0.0%    0.0%    0.0%    0.0%    0.0%    0.0%    0.0%    0.0%    0.0%    0.0%    0.0%    0.0%    0.0%    0.0%    0.0%    0.0%    0.0%    0.0%    0.0%    0.0%    0.0%    0.0%    0.0%    0.0%    0.0%    0.0%    0.0%    0.0%    0.0%    0.0%    0.0%    0.0%    0.0%    0.0%    0.0%    0.0%    0.0%    0.0%    0.0%    0.0%    0.0%    0.0%    0.0%    0.0%    0.0%    0.0%    0.0%    0.0%    0.0%    0.0%    0.0%    0.0%    0.0%    0.0%    0.0%    0.0%    0.0%    0.0%    0.0%    0.0%    0.0%    0.0%    0.0%    0.0%    0.0%    0.0%        k__Bacteria; p__OP3       1    0.0%    0.0%    0.0%    0.0%    0.0%    0.0%    0.0%    0.0%    0.0%    0.0%    0.0%    0.0%    0.0%    0.0%    0.0%    0.0%    0.0%    0.0%    0.0%    0.0%    0.0%    0.0%    0.0%    0.0%    0.0%    0.0%    0.0%    0.0%    0.0%    0.0%    0.0%    0.0%    0.0%    0.0%    0.0%    0.0%    0.0%    0.0%    0.0%    0.0%    0.0%    0.0%    0.0%    0.0%    0.0%    0.0%    0.0%    0.0%    0.0%    0.0%    0.0%    0.0%    0.0%    0.0%    0.0%    0.0%    0.0%    0.0%    0.0%    0.0%    0.0%    0.0%    0.0%    0.0%    0.0%    0.0%    0.0%    0.0%    0.0%    0.0%    0.0%    0.0%    0.0%    0.0%    0.0%    0.0%    0.0%    0.0%    0.0%    0.0%    0.0%    0.0%    0.0%    0.0%    0.0%    0.0%    0.0%    0.0%    0.0%    0.0%    0.0%    0.0%    0.0%    0.0%    0.0%    0.0%    0.0%    0.0%    0.0%    0.0%    0.0%    0.0%    0.0%    0.0%    0.0%    0.0%    0.0%    0.0%    0.0%    0.0%    0.0%    0.0%    0.0%    0.0%    0.0%    0.0%    0.0%    0.0%    0.0%    0.0%    0.0%    0.0%    0.0%    0.0%    0.0%    0.0%    0.0%    0.0%    0.0%    0.0%    0.0%    0.0%    0.0%    0.0%    0.0%    0.0%    0.0%    0.0%    0.0%    0.0%    0.0%    0.0%    0.0%    0.0%    0.0%    0.0%    0.0%    0.0%    0.0%    0.0%    0.0%    0.0%    0.0%    0.0%    0.0%    0.0%    0.0%    0.0%    0.0%    0.0%    0.0%    0.0%    0.0%    0.0%    0.0%    0.0%    0.0%    0.0%    0.0%    0.0%    0.0%    0.0%    0.0%    0.0%    0.0%    0.0%    0.0%    0.0%    0.0%    0.0%    0.0%    0.0%    0.0%    0.0%    0.0%    0.0%    0.0%    0.0%    0.0%    0.0%    0.0%    0.0%    0.0%    0.0%    0.0%    0.0%    0.0%    0.0%    0.0%    0.0%    0.0%    0.0%    0.0%    0.0%    0.0%    0.0%    0.0%    0.0%    0.0%    0.0%    0.0%    0.0%    0.0%    0.0%    0.0%    0.0%    0.0%    0.0%    0.0%    0.0%    0.0%    0.0%    0.0%    0.0%    0.0%    0.0%    0.0%    0.0%    0.0%    0.0%    0.0%    0.0%    0.0%    0.0%    0.0%    0.0%    0.0%    0.0%    0.0%    0.0%    0.0%    0.0%    0.0%    0.0%    0.0%    0.0%    0.0%    0.0%    0.0%    0.0%    0.0%    0.0%    0.0%    0.0%    0.0%    0.0%    0.0%    0.0%    0.0%    0.0%    0.0%    0.0%    0.0%    0.0%    0.0%    0.0%    0.0%    0.0%    0.0%    0.0%    0.0%    0.0%    0.0%    0.0%    0.0%    0.0%    0.0%    0.0%    0.0%    0.0%    0.0%    0.0%    0.0%    0.0%    0.0%    0.0%    0.0%    0.0%    0.0%    0.0%    0.0%    0.0%    0.0%    0.0%    0.0%    0.0%    0.0%    0.0%    0.0%    0.0%    0.0%    0.0%    0.0%    0.0%    0.0%    0.0%    0.0%    0.0%    0.0%    0.0%    0.0%    0.0%    0.0%    0.0%    0.0%    0.0%    0.0%    0.0%    0.0%    0.0%    0.0%    0.0%    0.0%    0.0%    0.0%    0.0%    0.0%    0.0%    0.0%    0.0%    0.0%    0.0%    0.0%    0.0%    0.0%    0.0%    0.0%    0.0%    0.0%    0.0%    0.0%    0.0%    0.0%    0.0%    0.0%    0.0%    0.0%    0.0%    0.0%    0.0%    0.0%    0.0%    0.0%    0.0%    0.0%    0.0%    0.0%    0.0%    0.0%    0.0%    0.0%    0.0%        k__Bacteria; p__OP8       0    0.0%    0.0%    0.0%    0.0%    0.0%    0.0%    0.0%    0.0%    0.0%    0.0%    0.0%    0.0%    0.0%    0.0%    0.0%    0.0%    0.0%    0.0%    0.0%    0.0%    0.0%    0.0%    0.0%    0.0%    0.0%    0.0%    0.0%    0.0%    0.0%    0.0%    0.0%    0.0%    0.0%    0.0%    0.0%    0.0%    0.0%    0.0%    0.0%    0.0%    0.0%    0.0%    0.0%    0.0%    0.0%    0.0%    0.0%    0.0%    0.0%    0.0%    0.0%    0.0%    0.0%    0.0%    0.0%    0.0%    0.0%    0.0%    0.0%    0.0%    0.0%    0.0%    0.0%    0.0%    0.0%    0.0%    0.0%    0.0%    0.0%    0.0%    0.0%    0.0%    0.0%    0.0%    0.0%    0.0%    0.0%    0.0%    0.0%    0.0%    0.0%    0.0%    0.0%    0.0%    0.0%    0.0%    0.0%    0.0%    0.0%    0.0%    0.0%    0.0%    0.0%    0.0%    0.0%    0.0%    0.0%    0.0%    0.0%    0.0%    0.0%    0.0%    0.0%    0.0%    0.0%    0.0%    0.0%    0.0%    0.0%    0.0%    0.0%    0.0%    0.0%    0.0%    0.0%    0.0%    0.0%    0.0%    0.0%    0.0%    0.0%    0.0%    0.0%    0.0%    0.0%    0.0%    0.0%    0.0%    0.0%    0.0%    0.0%    0.0%    0.0%    0.0%    0.0%    0.0%    0.0%    0.0%    0.0%    0.0%    0.0%    0.0%    0.0%    0.0%    0.0%    0.0%    0.0%    0.0%    0.0%    0.0%    0.0%    0.0%    0.0%    0.0%    0.0%    0.0%    0.0%    0.0%    0.0%    0.0%    0.0%    0.0%    0.0%    0.0%    0.0%    0.0%    0.0%    0.0%    0.0%    0.0%    0.0%    0.0%    0.0%    0.0%    0.0%    0.0%    0.0%    0.0%    0.0%    0.0%    0.0%    0.0%    0.0%    0.0%    0.0%    0.0%    0.0%    0.0%    0.0%    0.0%    0.0%    0.0%    0.0%    0.0%    0.0%    0.0%    0.0%    0.0%    0.0%    0.0%    0.0%    0.0%    0.0%    0.0%    0.0%    0.0%    0.0%    0.0%    0.0%    0.0%    0.0%    0.0%    0.0%    0.0%    0.0%    0.0%    0.0%    0.0%    0.0%    0.0%    0.0%    0.0%    0.0%    0.0%    0.0%    0.0%    0.0%    0.0%    0.0%    0.0%    0.0%    0.0%    0.0%    0.0%    0.0%    0.0%    0.0%    0.0%    0.0%    0.0%    0.0%    0.0%    0.0%    0.0%    0.0%    0.0%    0.0%    0.0%    0.0%    0.0%    0.0%    0.0%    0.0%    0.0%    0.0%    0.0%    0.0%    0.0%    0.0%    0.0%    0.0%    0.0%    0.0%    0.0%    0.0%    0.0%    0.0%    0.0%    0.0%    0.0%    0.0%    0.0%    0.0%    0.0%    0.0%    0.0%    0.0%    0.0%    0.0%    0.0%    0.0%    0.0%    0.0%    0.0%    0.0%    0.0%    0.0%    0.0%    0.0%    0.0%    0.0%    0.0%    0.0%    0.0%    0.0%    0.0%    0.0%    0.0%    0.0%    0.0%    0.0%    0.0%    0.0%    0.0%    0.0%    0.0%    0.0%    0.0%    0.0%    0.0%    0.0%    0.0%    0.0%    0.0%    0.0%    0.0%    0.0%    0.0%    0.0%    0.0%    0.0%    0.0%    0.0%    0.0%    0.0%    0.0%    0.0%    0.0%    0.0%    0.0%    0.0%    0.0%    0.0%    0.0%    0.0%    0.0%    0.0%    0.0%    0.0%    0.0%    0.0%    0.0%    0.0%    0.0%    0.0%    0.0%    0.0%    0.0%    0.0%    0.0%    0.0%    0.0%    0.0%    0.0%    0.0%    0.0%    0.0%    0.0%    0.0%    0.0%    0.0%    0.0%        k__Bacteria; p__Planctomycetes    1731    0.0%    0.0%    0.0%    0.0%    0.0%    0.0%    0.0%    0.0%    0.0%    0.0%    0.0%    0.0%    0.0%    0.0%    0.0%    0.0%    0.0%    0.0%    0.0%    0.0%    0.0%    0.1%    0.0%    0.0%    0.0%    0.0%    0.0%    0.0%    0.0%    0.0%    0.0%    0.0%    0.0%    0.0%    0.0%    0.0%    0.0%    0.0%    0.0%    0.0%    0.0%    0.0%    0.0%    0.0%    0.0%    0.0%    0.0%    0.0%    0.0%    0.0%    0.0%    0.0%    0.0%    0.0%    0.2%    0.1%    0.1%    0.0%    0.0%    0.1%    0.0%    0.0%    0.0%    0.1%    0.1%    0.0%    0.0%    0.0%    0.0%    0.0%    0.0%    0.0%    0.0%    0.0%    0.0%    0.0%    0.0%    0.1%    0.0%    0.0%    0.0%    0.0%    0.0%    0.0%    0.0%    0.0%    0.0%    0.0%    0.0%    0.0%    0.0%    0.0%    0.0%    0.0%    0.0%    0.0%    0.0%    0.1%    0.1%    0.0%    0.1%    0.0%    0.0%    0.0%    0.0%    0.0%    0.0%    0.0%    0.0%    0.1%    0.0%    0.1%    0.0%    0.0%    0.0%    0.0%    0.0%    0.0%    0.0%    0.1%    0.0%    0.0%    0.1%    0.0%    0.0%    0.3%    0.2%    0.0%    0.0%    0.2%    0.0%    0.0%    0.1%    0.0%    0.1%    0.0%    0.1%    0.0%    0.0%    0.0%    0.0%    0.1%    0.0%    0.2%    0.0%    0.0%    0.0%    0.0%    0.0%    0.1%    0.1%    0.0%    0.0%    0.0%    0.1%    0.0%    0.0%    0.1%    0.3%    0.0%    0.1%    0.0%    0.0%    0.0%    0.1%    0.0%    0.0%    0.0%    0.1%    0.0%    0.1%    0.0%    0.0%    0.0%    0.0%    0.0%    0.0%    0.0%    0.0%    0.0%    0.0%    0.0%    0.0%    0.0%    0.0%    0.0%    0.0%    0.0%    0.0%    0.0%    0.1%    0.0%    0.0%    0.1%    0.0%    0.0%    0.0%    0.0%    0.0%    0.0%    0.0%    0.0%    0.0%    0.1%    0.0%    0.0%    0.0%    0.1%    0.1%    0.0%    0.0%    0.1%    0.0%    0.1%    0.0%    0.0%    0.0%    0.0%    0.1%    0.0%    0.0%    0.0%    0.1%    0.0%    0.1%    0.0%    0.0%    0.0%    0.1%    0.0%    0.0%    0.0%    0.1%    0.1%    0.2%    0.0%    0.1%    0.0%    0.0%    0.0%    0.0%    0.0%    0.1%    0.1%    0.0%    0.1%    0.1%    0.0%    0.0%    0.0%    0.0%    0.1%    0.0%    0.0%    0.0%    0.0%    0.0%    0.0%    0.0%    0.0%    0.0%    0.0%    0.0%    0.0%    0.0%    0.1%    0.0%    0.0%    0.1%    0.1%    0.0%    0.1%    0.2%    0.1%    0.0%    0.0%    0.0%    0.0%    0.2%    0.1%    0.1%    0.1%    0.0%    0.0%    0.0%    0.1%    0.0%    0.0%    0.0%    0.0%    0.0%    0.0%    0.0%    0.2%    0.0%    0.0%    0.1%    0.0%    0.0%    0.1%    0.0%    0.0%    0.0%    0.1%    0.0%    0.1%    0.0%    0.0%    0.0%    0.1%    0.0%    0.0%    0.1%    0.0%    0.0%    0.0%    0.1%    0.1%    0.0%    0.0%    0.0%    0.1%    0.0%    0.0%    0.1%    0.0%    0.0%    0.0%    0.0%    0.0%    0.0%    0.0%    0.0%    0.0%    0.0%    0.0%    0.0%    0.0%    0.0%    0.0%    0.0%    0.0%    0.0%    0.0%    0.1%    0.0%    0.0%    0.0%    0.0%    0.0%    0.0%    0.0%    0.0%    0.0%    0.0%    0.0%    0.0%    0.0%    0.0%    0.0%    0.0%    0.1%        k__Bacteria; p__Proteobacteria   1341255   23.5%   13.2%   10.5%   18.2%   17.8%   29.6%   20.0%   78.2%   17.0%   20.1%   30.9%   57.0%    5.6%   39.7%   10.8%   18.9%   22.7%   10.5%   54.6%   13.5%   26.2%   26.1%   21.0%   38.1%   17.9%    2.1%    4.9%   21.2%   19.0%   17.6%   23.7%    3.9%   14.7%   13.7%   24.6%   21.7%   23.7%   19.7%   17.9%   34.2%   19.5%   35.6%   37.1%   28.0%   28.5%   18.7%    6.1%   31.6%   31.2%   17.9%   52.3%   34.3%   35.5%   36.2%   20.5%    9.0%   17.5%   29.4%   21.3%   16.4%   11.5%   13.3%   15.3%   11.8%   31.3%    6.1%   55.8%   20.9%   64.5%    5.7%   66.8%   38.8%    7.0%   18.4%   11.5%    3.0%   21.1%   18.6%    3.8%    9.5%    7.9%   57.7%   21.4%   16.3%   17.0%    3.6%   19.7%   38.8%   24.5%   15.8%   63.4%   21.1%   20.5%    7.7%    6.9%   13.1%   15.8%   13.5%   11.4%   24.9%   14.4%   12.1%    8.4%    2.0%    6.6%   21.3%   44.7%   51.2%   13.1%   13.1%   15.2%   15.2%   30.6%   12.9%   18.7%   15.2%   16.5%   10.4%   29.4%   36.4%   78.4%   18.9%   22.1%   30.8%   59.9%   42.7%   23.1%   55.1%   18.3%   29.1%   19.6%   17.5%   23.8%    9.5%   24.9%   33.7%   44.5%   39.9%   78.4%   70.0%   29.6%   27.8%   69.1%   25.2%   13.6%   14.9%    9.6%   15.0%   30.4%   18.0%   33.7%   19.0%   11.5%   53.0%   22.6%   10.3%   52.6%   24.4%   24.0%   26.4%   42.0%   29.5%   20.4%   19.5%   26.9%   21.2%   20.6%   19.0%   37.7%   24.6%   24.7%   18.9%    9.4%   12.9%   16.2%   21.0%   43.6%   19.7%    8.0%   10.6%   17.4%    8.2%    8.7%   16.3%   11.2%    7.0%   11.6%    6.1%   12.7%   12.3%   26.7%   48.4%   16.9%   19.6%   10.0%    7.0%    8.8%   19.5%   13.3%   28.0%   34.7%   33.0%   10.9%   41.2%   31.2%   19.5%   19.0%   18.3%   20.3%   20.6%   10.4%   22.3%   28.9%   15.4%   25.2%   23.7%   96.9%   14.0%   15.4%   17.2%   47.9%   52.0%   50.8%   33.1%   19.5%   21.1%   16.0%   14.5%   19.1%   16.4%   14.9%   13.6%   51.7%   13.0%   26.8%   23.5%   23.6%    6.1%   22.8%   13.9%   16.1%    9.0%   11.1%    5.8%   28.7%   16.1%   30.2%   14.4%   29.8%   10.1%   56.5%   12.1%   25.9%   43.9%   56.3%   27.5%   45.1%    5.2%   30.0%   24.6%   12.7%   17.9%   16.2%    8.7%   16.5%   66.5%   17.2%   44.8%   18.6%   30.5%   29.1%   34.2%   32.6%   24.4%   19.7%    3.6%   32.1%   14.1%   40.4%   23.4%   16.0%   16.7%   13.5%   14.8%   10.8%   31.1%   29.6%   12.0%   18.9%   18.7%   16.1%   16.2%   18.5%   46.1%   18.4%    7.6%   10.9%    8.3%   16.6%   17.1%   21.8%    5.9%    7.7%   15.7%    9.4%   19.2%   18.0%    8.3%   45.2%   16.1%   13.7%   21.8%   11.1%    7.6%   21.2%   33.9%   21.4%    3.3%    2.0%   18.1%   14.4%   19.8%    6.5%   20.4%   12.7%   13.5%   10.2%   13.3%   18.3%   14.2%    9.9%    7.1%    5.4%    9.1%    2.0%    3.4%    1.0%    6.1%   12.6%   36.2%    1.5%   55.1%    4.5%   15.0%   28.4%    6.1%   10.3%   25.5%   14.1%    8.8%   21.3%    8.1%    7.0%   26.3%    9.4%   75.0%   18.9%    0.3%   16.7%    9.5%    4.9%   18.0%        k__Bacteria; p__SC3       3    0.0%    0.0%    0.0%    0.0%    0.0%    0.0%    0.0%    0.0%    0.0%    0.0%    0.0%    0.0%    0.0%    0.0%    0.0%    0.0%    0.0%    0.0%    0.0%    0.0%    0.0%    0.0%    0.0%    0.0%    0.0%    0.0%    0.0%    0.0%    0.0%    0.0%    0.0%    0.0%    0.0%    0.0%    0.0%    0.0%    0.0%    0.0%    0.0%    0.0%    0.0%    0.0%    0.0%    0.0%    0.0%    0.0%    0.0%    0.0%    0.0%    0.0%    0.0%    0.0%    0.0%    0.0%    0.0%    0.0%    0.0%    0.0%    0.0%    0.0%    0.0%    0.0%    0.0%    0.0%    0.0%    0.0%    0.0%    0.0%    0.0%    0.0%    0.0%    0.0%    0.0%    0.0%    0.0%    0.0%    0.0%    0.0%    0.0%    0.0%    0.0%    0.0%    0.0%    0.0%    0.0%    0.0%    0.0%    0.0%    0.0%    0.0%    0.0%    0.0%    0.0%    0.0%    0.0%    0.0%    0.0%    0.0%    0.0%    0.0%    0.0%    0.0%    0.0%    0.0%    0.0%    0.0%    0.0%    0.0%    0.0%    0.0%    0.0%    0.0%    0.0%    0.0%    0.0%    0.0%    0.0%    0.0%    0.0%    0.0%    0.0%    0.0%    0.0%    0.0%    0.0%    0.0%    0.0%    0.0%    0.0%    0.0%    0.0%    0.0%    0.0%    0.0%    0.0%    0.0%    0.0%    0.0%    0.0%    0.0%    0.0%    0.0%    0.0%    0.0%    0.0%    0.0%    0.0%    0.0%    0.0%    0.0%    0.0%    0.0%    0.0%    0.0%    0.0%    0.0%    0.0%    0.0%    0.0%    0.0%    0.0%    0.0%    0.0%    0.0%    0.0%    0.0%    0.0%    0.0%    0.0%    0.0%    0.0%    0.0%    0.0%    0.0%    0.0%    0.0%    0.0%    0.0%    0.0%    0.0%    0.0%    0.0%    0.0%    0.0%    0.0%    0.0%    0.0%    0.0%    0.0%    0.0%    0.0%    0.0%    0.0%    0.0%    0.0%    0.0%    0.0%    0.0%    0.0%    0.0%    0.0%    0.0%    0.0%    0.0%    0.0%    0.0%    0.0%    0.0%    0.0%    0.0%    0.0%    0.0%    0.0%    0.0%    0.0%    0.0%    0.0%    0.0%    0.0%    0.0%    0.0%    0.0%    0.0%    0.0%    0.0%    0.0%    0.0%    0.0%    0.0%    0.0%    0.0%    0.0%    0.0%    0.0%    0.0%    0.0%    0.0%    0.0%    0.0%    0.0%    0.0%    0.0%    0.0%    0.0%    0.0%    0.0%    0.0%    0.0%    0.0%    0.0%    0.0%    0.0%    0.0%    0.0%    0.0%    0.0%    0.0%    0.0%    0.0%    0.0%    0.0%    0.0%    0.0%    0.0%    0.0%    0.0%    0.0%    0.0%    0.0%    0.0%    0.0%    0.0%    0.0%    0.0%    0.0%    0.0%    0.0%    0.0%    0.0%    0.0%    0.0%    0.0%    0.0%    0.0%    0.0%    0.0%    0.0%    0.0%    0.0%    0.0%    0.0%    0.0%    0.0%    0.0%    0.0%    0.0%    0.0%    0.0%    0.0%    0.0%    0.0%    0.0%    0.0%    0.0%    0.0%    0.0%    0.0%    0.0%    0.0%    0.0%    0.0%    0.0%    0.0%    0.0%    0.0%    0.0%    0.0%    0.0%    0.0%    0.0%    0.0%    0.0%    0.0%    0.0%    0.0%    0.0%    0.0%    0.0%    0.0%    0.0%    0.0%    0.0%    0.0%    0.0%    0.0%    0.0%    0.0%    0.0%    0.0%    0.0%    0.0%    0.0%    0.0%    0.0%    0.0%    0.0%    0.0%    0.0%    0.0%    0.0%    0.0%    0.0%    0.0%    0.0%    0.0%    0.0%    0.0%    0.0%    0.0%    0.0%    0.0%    0.0%        k__Bacteria; p__SC4     271    0.0%    0.0%    0.0%    0.0%    0.0%    0.0%    0.0%    0.0%    0.0%    0.0%    0.0%    0.0%    0.0%    0.0%    0.0%    0.0%    0.0%    0.0%    0.0%    0.0%    0.0%    0.0%    0.0%    0.0%    0.0%    0.0%    0.0%    0.0%    0.0%    0.0%    0.0%    0.0%    0.0%    0.0%    0.0%    0.0%    0.0%    0.0%    0.0%    0.0%    0.0%    0.0%    0.0%    0.0%    0.0%    0.0%    0.0%    0.0%    0.0%    0.0%    0.0%    0.0%    0.0%    0.0%    0.0%    0.0%    0.0%    0.0%    0.0%    0.0%    0.0%    0.0%    0.0%    0.0%    0.0%    0.0%    0.0%    0.0%    0.0%    0.0%    0.0%    0.0%    0.0%    0.0%    0.0%    0.0%    0.0%    0.0%    0.0%    0.0%    0.0%    0.0%    0.0%    0.0%    0.0%    0.0%    0.0%    0.0%    0.0%    0.0%    0.0%    0.0%    0.0%    0.0%    0.0%    0.0%    0.0%    0.0%    0.0%    0.0%    0.0%    0.0%    0.0%    0.0%    0.0%    0.0%    0.0%    0.0%    0.0%    0.0%    0.0%    0.0%    0.0%    0.0%    0.0%    0.0%    0.0%    0.0%    0.0%    0.0%    0.0%    0.0%    0.0%    0.0%    0.0%    0.0%    0.0%    0.0%    0.0%    0.0%    0.0%    0.0%    0.0%    0.0%    0.0%    0.0%    0.0%    0.0%    0.0%    0.0%    0.0%    0.0%    0.0%    0.0%    0.0%    0.0%    0.0%    0.0%    0.0%    0.0%    0.1%    0.0%    0.0%    0.0%    0.0%    0.0%    0.0%    0.0%    0.0%    0.0%    0.0%    0.0%    0.0%    0.0%    0.0%    0.0%    0.0%    0.0%    0.0%    0.0%    0.0%    0.0%    0.0%    0.0%    0.0%    0.0%    0.0%    0.0%    0.0%    0.0%    0.0%    0.0%    0.0%    0.0%    0.0%    0.0%    0.0%    0.0%    0.0%    0.0%    0.0%    0.0%    0.0%    0.0%    0.0%    0.0%    0.0%    0.0%    0.0%    0.0%    0.0%    0.0%    0.0%    0.0%    0.0%    0.0%    0.0%    0.0%    0.0%    0.0%    0.0%    0.0%    0.0%    0.0%    0.0%    0.0%    0.0%    0.0%    0.0%    0.0%    0.0%    0.0%    0.0%    0.0%    0.1%    0.0%    0.0%    0.0%    0.0%    0.0%    0.0%    0.0%    0.0%    0.0%    0.0%    0.0%    0.0%    0.0%    0.0%    0.0%    0.0%    0.0%    0.0%    0.0%    0.0%    0.0%    0.0%    0.0%    0.0%    0.0%    0.1%    0.0%    0.0%    0.0%    0.0%    0.0%    0.0%    0.0%    0.0%    0.0%    0.0%    0.0%    0.0%    0.0%    0.0%    0.0%    0.0%    0.0%    0.0%    0.0%    0.0%    0.0%    0.0%    0.0%    0.0%    0.0%    0.0%    0.0%    0.1%    0.0%    0.0%    0.0%    0.0%    0.0%    0.0%    0.0%    0.0%    0.0%    0.0%    0.0%    0.0%    0.0%    0.0%    0.1%    0.0%    0.0%    0.0%    0.0%    0.0%    0.0%    0.0%    0.0%    0.0%    0.0%    0.0%    0.0%    0.0%    0.0%    0.0%    0.0%    0.0%    0.0%    0.0%    0.0%    0.0%    0.0%    0.0%    0.0%    0.0%    0.0%    0.0%    0.0%    0.0%    0.0%    0.0%    0.1%    0.0%    0.0%    0.0%    0.0%    0.0%    0.0%    0.0%    0.0%    0.0%    0.0%    0.0%    0.0%    0.0%    0.0%    0.0%    0.0%    0.0%    0.0%    0.0%    0.0%    0.0%    0.0%    0.0%    0.0%    0.0%    0.0%    0.0%    0.0%    0.0%    0.0%    0.0%    0.0%    0.0%    0.0%    0.0%    0.0%        k__Bacteria; p__SPAM      73    0.0%    0.0%    0.0%    0.0%    0.0%    0.0%    0.0%    0.0%    0.0%    0.0%    0.0%    0.0%    0.0%    0.0%    0.0%    0.0%    0.0%    0.0%    0.0%    0.0%    0.0%    0.0%    0.0%    0.0%    0.0%    0.0%    0.0%    0.0%    0.0%    0.0%    0.0%    0.0%    0.0%    0.0%    0.0%    0.0%    0.0%    0.0%    0.0%    0.0%    0.0%    0.0%    0.0%    0.0%    0.0%    0.0%    0.0%    0.0%    0.0%    0.0%    0.0%    0.0%    0.0%    0.0%    0.0%    0.0%    0.0%    0.0%    0.0%    0.0%    0.0%    0.0%    0.0%    0.0%    0.0%    0.0%    0.0%    0.0%    0.0%    0.0%    0.0%    0.0%    0.0%    0.0%    0.0%    0.0%    0.0%    0.0%    0.0%    0.0%    0.0%    0.0%    0.0%    0.0%    0.0%    0.0%    0.0%    0.0%    0.0%    0.0%    0.0%    0.0%    0.0%    0.0%    0.0%    0.0%    0.0%    0.0%    0.0%    0.0%    0.0%    0.0%    0.0%    0.0%    0.0%    0.0%    0.0%    0.0%    0.0%    0.0%    0.0%    0.0%    0.0%    0.0%    0.0%    0.0%    0.0%    0.0%    0.0%    0.0%    0.0%    0.0%    0.0%    0.0%    0.0%    0.0%    0.0%    0.0%    0.0%    0.0%    0.0%    0.0%    0.0%    0.0%    0.0%    0.0%    0.0%    0.0%    0.0%    0.0%    0.0%    0.0%    0.0%    0.0%    0.0%    0.0%    0.0%    0.0%    0.0%    0.0%    0.0%    0.0%    0.0%    0.0%    0.0%    0.0%    0.0%    0.0%    0.0%    0.0%    0.0%    0.0%    0.0%    0.0%    0.0%    0.0%    0.0%    0.0%    0.0%    0.0%    0.0%    0.0%    0.0%    0.0%    0.0%    0.0%    0.0%    0.0%    0.0%    0.0%    0.0%    0.0%    0.0%    0.0%    0.0%    0.0%    0.0%    0.0%    0.0%    0.0%    0.0%    0.0%    0.0%    0.0%    0.0%    0.0%    0.0%    0.0%    0.0%    0.0%    0.0%    0.0%    0.0%    0.0%    0.0%    0.0%    0.0%    0.0%    0.0%    0.0%    0.0%    0.0%    0.0%    0.0%    0.0%    0.0%    0.0%    0.0%    0.0%    0.0%    0.0%    0.0%    0.0%    0.0%    0.0%    0.0%    0.0%    0.0%    0.0%    0.0%    0.0%    0.0%    0.0%    0.0%    0.0%    0.0%    0.0%    0.0%    0.0%    0.0%    0.0%    0.0%    0.0%    0.0%    0.0%    0.0%    0.0%    0.0%    0.0%    0.0%    0.0%    0.0%    0.0%    0.0%    0.0%    0.0%    0.0%    0.0%    0.0%    0.0%    0.0%    0.0%    0.0%    0.0%    0.0%    0.0%    0.0%    0.0%    0.0%    0.0%    0.0%    0.0%    0.0%    0.0%    0.0%    0.0%    0.0%    0.0%    0.0%    0.0%    0.0%    0.0%    0.0%    0.0%    0.0%    0.0%    0.0%    0.0%    0.0%    0.0%    0.0%    0.0%    0.1%    0.0%    0.0%    0.0%    0.0%    0.0%    0.0%    0.0%    0.0%    0.0%    0.0%    0.0%    0.0%    0.0%    0.0%    0.0%    0.0%    0.0%    0.0%    0.0%    0.0%    0.0%    0.0%    0.0%    0.0%    0.0%    0.0%    0.0%    0.0%    0.0%    0.0%    0.0%    0.0%    0.0%    0.0%    0.0%    0.0%    0.0%    0.0%    0.0%    0.0%    0.0%    0.0%    0.0%    0.0%    0.0%    0.0%    0.0%    0.0%    0.0%    0.0%    0.0%    0.0%    0.0%    0.0%    0.0%    0.0%    0.0%    0.0%    0.0%    0.0%    0.0%    0.0%    0.0%    0.0%    0.0%    0.0%    0.0%    0.0%    0.0%        k__Bacteria; p__SR1     438    0.0%    0.0%    0.0%    0.0%    0.0%    0.0%    0.0%    0.0%    0.0%    0.0%    0.0%    0.0%    0.0%    0.0%    0.1%    0.0%    0.0%    0.0%    0.0%    0.0%    0.0%    0.0%    0.1%    0.0%    0.0%    0.0%    0.0%    0.0%    0.0%    0.0%    0.0%    0.0%    0.0%    0.1%    0.0%    0.0%    0.0%    0.0%    0.0%    0.0%    0.0%    0.0%    0.0%    0.0%    0.0%    0.0%    0.0%    0.0%    0.0%    0.0%    0.0%    0.0%    0.0%    0.0%    0.0%    0.0%    0.0%    0.0%    0.0%    0.0%    0.0%    0.0%    0.0%    0.0%    0.0%    0.0%    0.0%    0.0%    0.0%    0.0%    0.0%    0.0%    0.0%    0.0%    0.0%    0.0%    0.0%    0.1%    0.0%    0.0%    0.1%    0.0%    0.0%    0.0%    0.0%    0.0%    0.0%    0.0%    0.0%    0.0%    0.0%    0.0%    0.0%    0.0%    0.0%    0.0%    0.0%    0.0%    0.0%    0.0%    0.0%    0.0%    0.0%    0.0%    0.0%    0.0%    0.0%    0.0%    0.0%    0.0%    0.0%    0.0%    0.1%    0.0%    0.0%    0.0%    0.0%    0.0%    0.0%    0.0%    0.0%    0.0%    0.0%    0.1%    0.0%    0.0%    0.0%    0.0%    0.0%    0.0%    0.0%    0.0%    0.0%    0.0%    0.0%    0.0%    0.0%    0.0%    0.0%    0.0%    0.0%    0.0%    0.0%    0.0%    0.0%    0.1%    0.0%    0.0%    0.0%    0.0%    0.0%    0.0%    0.0%    0.0%    0.0%    0.0%    0.0%    0.0%    0.0%    0.0%    0.1%    0.0%    0.0%    0.0%    0.0%    0.0%    0.0%    0.0%    0.0%    0.0%    0.0%    0.0%    0.0%    0.0%    0.0%    0.0%    0.0%    0.0%    0.0%    0.0%    0.0%    0.0%    0.0%    0.0%    0.0%    0.0%    0.0%    0.0%    0.0%    0.0%    0.0%    0.0%    0.0%    0.0%    0.0%    0.0%    0.0%    0.0%    0.0%    0.0%    0.0%    0.0%    0.0%    0.0%    0.0%    0.0%    0.0%    0.0%    0.0%    0.0%    0.0%    0.0%    0.0%    0.0%    0.1%    0.0%    0.0%    0.0%    0.0%    0.0%    0.0%    0.0%    0.0%    0.0%    0.0%    0.0%    0.0%    0.0%    0.0%    0.0%    0.0%    0.0%    0.0%    0.0%    0.0%    0.0%    0.0%    0.0%    0.0%    0.0%    0.0%    0.0%    0.0%    0.0%    0.0%    0.0%    0.0%    0.0%    0.0%    0.0%    0.0%    0.0%    0.0%    0.0%    0.0%    0.0%    0.0%    0.0%    0.0%    0.0%    0.0%    0.0%    0.0%    0.0%    0.0%    0.0%    0.0%    0.0%    0.0%    0.0%    0.0%    0.0%    0.0%    0.0%    0.0%    0.0%    0.0%    0.0%    0.0%    0.0%    0.0%    0.0%    0.0%    0.0%    0.0%    0.0%    0.0%    0.0%    0.0%    0.0%    0.0%    0.0%    0.0%    0.0%    0.0%    0.0%    0.0%    0.0%    0.0%    0.0%    0.0%    0.0%    0.0%    0.0%    0.0%    0.0%    0.0%    0.0%    0.0%    0.0%    0.0%    0.0%    0.0%    0.0%    0.0%    0.1%    0.0%    0.0%    0.0%    0.0%    0.0%    0.0%    0.0%    0.0%    0.0%    0.0%    0.0%    0.0%    0.0%    0.0%    0.0%    0.0%    0.0%    0.0%    0.0%    0.0%    0.0%    0.0%    0.0%    0.0%    0.0%    0.0%    0.0%    0.0%    0.0%    0.0%    0.0%    0.0%    0.0%    0.0%    0.0%    0.0%    0.0%    0.0%    0.0%    0.0%    0.0%    0.0%    0.0%    0.0%    0.0%    0.0%        k__Bacteria; p__Spirochaetes    3657    0.1%    0.2%    0.1%    0.1%    0.0%    0.0%    0.3%    0.0%    0.0%    0.0%    0.0%    0.0%    0.0%    0.0%    0.6%    0.1%    0.0%    0.0%    0.0%    0.3%    0.0%    0.0%    0.2%    0.0%    0.0%    0.0%    0.0%    0.0%    0.1%    0.0%    0.0%    0.0%    0.1%    1.1%    0.2%    0.4%    0.2%    0.0%    0.1%    0.0%    0.0%    0.3%    0.1%    0.1%    0.1%    0.1%    0.0%    0.0%    0.0%    0.1%    0.0%    0.0%    0.0%    0.0%    0.0%    0.0%    0.1%    0.0%    0.0%    0.0%    0.0%    0.0%    0.0%    0.0%    0.1%    0.0%    0.0%    0.1%    0.0%    0.0%    0.5%    0.1%    0.0%    0.0%    0.0%    0.0%    0.0%    0.0%    0.0%    0.0%    0.1%    0.0%    0.0%    0.0%    0.0%    0.0%    0.0%    0.0%    0.0%    0.0%    0.0%    0.0%    0.0%    0.0%    0.0%    0.0%    0.0%    0.0%    0.2%    0.0%    0.0%    0.0%    0.0%    0.0%    0.0%    0.1%    0.0%    0.0%    0.0%    0.1%    0.0%    0.1%    0.1%    0.0%    0.1%    0.0%    0.0%    0.0%    0.0%    0.1%    0.0%    0.0%    0.1%    0.1%    0.0%    0.0%    0.0%    0.0%    0.0%    0.0%    0.0%    0.0%    0.0%    0.0%    0.0%    0.0%    0.0%    0.0%    0.0%    0.0%    0.1%    0.0%    0.0%    0.0%    0.0%    1.2%    0.0%    0.0%    0.0%    0.0%    0.0%    0.0%    0.0%    0.0%    0.0%    0.0%    0.0%    0.1%    0.0%    0.0%    0.1%    0.0%    0.0%    0.0%    0.0%    0.0%    0.0%    0.0%    0.0%    0.0%    0.0%    0.0%    0.0%    0.0%    0.0%    0.0%    0.0%    0.0%    0.0%    0.0%    0.0%    0.0%    0.0%    0.0%    0.0%    0.1%    0.0%    0.0%    0.1%    0.0%    0.1%    0.0%    0.0%    0.0%    0.0%    0.0%    0.0%    0.1%    0.0%    0.0%    0.0%    0.0%    0.0%    0.0%    0.0%    0.0%    0.0%    0.0%    0.1%    0.0%    0.0%    0.1%    0.0%    0.0%    0.0%    0.0%    0.0%    0.0%    0.0%    0.0%    0.0%    0.0%    0.0%    0.0%    0.1%    0.0%    0.0%    0.0%    0.0%    0.0%    0.0%    0.0%    0.0%    0.0%    0.0%    0.0%    0.0%    0.0%    0.0%    0.0%    0.0%    0.0%    0.0%    0.0%    0.0%    0.0%    0.0%    0.0%    0.0%    0.0%    0.0%    0.0%    0.0%    0.0%    0.0%    0.0%    0.0%    0.0%    0.0%    0.0%    0.0%    0.0%    0.0%    0.0%    0.0%    0.0%    0.0%    0.0%    0.0%    0.0%    0.0%    0.0%    0.0%    0.0%    0.0%    0.0%    0.0%    0.1%    0.0%    0.0%    0.0%    0.0%    0.0%    0.0%    0.0%    0.0%    0.0%    0.0%    0.0%    0.0%    0.0%    0.0%    0.0%    0.0%    0.0%    0.0%    0.0%    0.0%    0.1%    0.1%    0.1%    0.0%    0.0%    0.0%    0.0%    0.0%    0.1%    0.0%    0.0%    0.0%    0.0%    0.0%    0.0%    0.0%    0.0%    0.3%    0.0%    0.0%    0.0%    0.1%    0.0%    0.1%    0.0%    0.1%    0.0%    0.0%    0.0%    0.0%    0.0%    0.0%    0.0%    0.0%    0.0%    0.0%    0.0%    0.0%    0.0%    0.0%    0.0%    0.0%    0.0%    0.0%    0.0%    0.0%    0.0%    0.0%    0.0%    0.0%    0.0%    0.0%    0.0%    0.0%    0.0%    0.0%    0.0%    0.0%    0.0%    0.0%    0.1%    0.0%    0.0%    0.0%        k__Bacteria; p__Synergistetes    1177    0.0%    0.0%    0.0%    0.0%    0.0%    0.0%    0.1%    0.0%    0.0%    0.0%    0.0%    0.0%    0.0%    0.0%    0.1%    0.0%    0.0%    0.0%    0.0%    0.0%    0.0%    0.0%    0.0%    0.0%    0.0%    0.0%    0.0%    0.0%    0.0%    0.0%    0.0%    0.1%    0.0%    0.5%    0.0%    0.2%    0.0%    0.0%    0.0%    0.0%    0.0%    0.0%    0.0%    0.0%    0.0%    0.0%    0.1%    0.0%    0.0%    0.0%    0.0%    0.0%    0.0%    0.0%    0.0%    0.0%    0.0%    0.0%    0.0%    0.0%    0.0%    0.0%    0.0%    0.0%    0.0%    0.0%    0.0%    0.0%    0.0%    0.0%    0.1%    0.0%    0.0%    0.0%    0.0%    0.0%    0.0%    0.0%    0.0%    0.0%    0.0%    0.0%    0.0%    0.0%    0.0%    0.0%    0.0%    0.0%    0.0%    0.0%    0.0%    0.0%    0.0%    0.0%    0.0%    0.0%    0.0%    0.0%    0.0%    0.0%    0.0%    0.0%    0.0%    0.0%    0.0%    0.0%    0.0%    0.0%    0.0%    0.0%    0.0%    0.0%    0.0%    0.0%    0.0%    0.0%    0.0%    0.0%    0.0%    0.0%    0.0%    0.0%    0.0%    0.0%    0.0%    0.0%    0.0%    0.0%    0.0%    0.0%    0.0%    0.0%    0.0%    0.0%    0.0%    0.0%    0.0%    0.0%    0.0%    0.0%    0.0%    0.0%    0.0%    0.0%    0.0%    0.7%    0.0%    0.0%    0.0%    0.0%    0.0%    0.0%    0.0%    0.0%    0.0%    0.0%    0.0%    0.0%    0.0%    0.0%    0.0%    0.0%    0.0%    0.0%    0.0%    0.0%    0.0%    0.0%    0.0%    0.0%    0.0%    0.0%    0.0%    0.0%    0.0%    0.0%    0.0%    0.0%    0.0%    0.0%    0.0%    0.0%    0.0%    0.0%    0.0%    0.0%    0.0%    0.0%    0.0%    0.0%    0.0%    0.0%    0.0%    0.0%    0.0%    0.0%    0.0%    0.0%    0.0%    0.0%    0.0%    0.0%    0.0%    0.0%    0.0%    0.0%    0.0%    0.0%    0.0%    0.0%    0.0%    0.1%    0.0%    0.0%    0.0%    0.0%    0.0%    0.0%    0.0%    0.0%    0.0%    0.0%    0.0%    0.0%    0.1%    0.0%    0.0%    0.0%    0.0%    0.0%    0.0%    0.0%    0.0%    0.0%    0.0%    0.0%    0.0%    0.0%    0.0%    0.0%    0.0%    0.0%    0.0%    0.0%    0.0%    0.1%    0.0%    0.0%    0.0%    0.0%    0.0%    0.0%    0.0%    0.0%    0.0%    0.0%    0.0%    0.0%    0.0%    0.0%    0.0%    0.0%    0.0%    0.0%    0.0%    0.0%    0.0%    0.0%    0.0%    0.0%    0.0%    0.0%    0.0%    0.0%    0.0%    0.0%    0.0%    0.0%    0.0%    0.0%    0.0%    0.0%    0.0%    0.0%    0.0%    0.0%    0.0%    0.0%    0.0%    0.0%    0.0%    0.0%    0.0%    0.0%    0.0%    0.0%    0.0%    0.0%    0.0%    0.1%    0.0%    0.0%    0.0%    0.0%    0.0%    0.0%    0.0%    0.0%    0.0%    0.0%    0.0%    0.0%    0.0%    0.0%    0.0%    0.0%    0.0%    0.0%    0.0%    0.0%    0.0%    0.0%    0.0%    0.0%    0.0%    0.0%    0.0%    0.0%    0.0%    0.0%    0.0%    0.1%    0.0%    0.0%    0.0%    0.0%    0.0%    0.1%    0.0%    0.0%    0.0%    0.0%    0.0%    0.0%    0.0%    0.0%    0.0%    0.0%    0.0%    0.0%    0.0%    0.0%    0.0%    0.0%    0.0%    0.0%    0.0%    0.0%    0.0%    0.0%    0.0%    0.0%        k__Bacteria; p__TM6       2    0.0%    0.0%    0.0%    0.0%    0.0%    0.0%    0.0%    0.0%    0.0%    0.0%    0.0%    0.0%    0.0%    0.0%    0.0%    0.0%    0.0%    0.0%    0.0%    0.0%    0.0%    0.0%    0.0%    0.0%    0.0%    0.0%    0.0%    0.0%    0.0%    0.0%    0.0%    0.0%    0.0%    0.0%    0.0%    0.0%    0.0%    0.0%    0.0%    0.0%    0.0%    0.0%    0.0%    0.0%    0.0%    0.0%    0.0%    0.0%    0.0%    0.0%    0.0%    0.0%    0.0%    0.0%    0.0%    0.0%    0.0%    0.0%    0.0%    0.0%    0.0%    0.0%    0.0%    0.0%    0.0%    0.0%    0.0%    0.0%    0.0%    0.0%    0.0%    0.0%    0.0%    0.0%    0.0%    0.0%    0.0%    0.0%    0.0%    0.0%    0.0%    0.0%    0.0%    0.0%    0.0%    0.0%    0.0%    0.0%    0.0%    0.0%    0.0%    0.0%    0.0%    0.0%    0.0%    0.0%    0.0%    0.0%    0.0%    0.0%    0.0%    0.0%    0.0%    0.0%    0.0%    0.0%    0.0%    0.0%    0.0%    0.0%    0.0%    0.0%    0.0%    0.0%    0.0%    0.0%    0.0%    0.0%    0.0%    0.0%    0.0%    0.0%    0.0%    0.0%    0.0%    0.0%    0.0%    0.0%    0.0%    0.0%    0.0%    0.0%    0.0%    0.0%    0.0%    0.0%    0.0%    0.0%    0.0%    0.0%    0.0%    0.0%    0.0%    0.0%    0.0%    0.0%    0.0%    0.0%    0.0%    0.0%    0.0%    0.0%    0.0%    0.0%    0.0%    0.0%    0.0%    0.0%    0.0%    0.0%    0.0%    0.0%    0.0%    0.0%    0.0%    0.0%    0.0%    0.0%    0.0%    0.0%    0.0%    0.0%    0.0%    0.0%    0.0%    0.0%    0.0%    0.0%    0.0%    0.0%    0.0%    0.0%    0.0%    0.0%    0.0%    0.0%    0.0%    0.0%    0.0%    0.0%    0.0%    0.0%    0.0%    0.0%    0.0%    0.0%    0.0%    0.0%    0.0%    0.0%    0.0%    0.0%    0.0%    0.0%    0.0%    0.0%    0.0%    0.0%    0.0%    0.0%    0.0%    0.0%    0.0%    0.0%    0.0%    0.0%    0.0%    0.0%    0.0%    0.0%    0.0%    0.0%    0.0%    0.0%    0.0%    0.0%    0.0%    0.0%    0.0%    0.0%    0.0%    0.0%    0.0%    0.0%    0.0%    0.0%    0.0%    0.0%    0.0%    0.0%    0.0%    0.0%    0.0%    0.0%    0.0%    0.0%    0.0%    0.0%    0.0%    0.0%    0.0%    0.0%    0.0%    0.0%    0.0%    0.0%    0.0%    0.0%    0.0%    0.0%    0.0%    0.0%    0.0%    0.0%    0.0%    0.0%    0.0%    0.0%    0.0%    0.0%    0.0%    0.0%    0.0%    0.0%    0.0%    0.0%    0.0%    0.0%    0.0%    0.0%    0.0%    0.0%    0.0%    0.0%    0.0%    0.0%    0.0%    0.0%    0.0%    0.0%    0.0%    0.0%    0.0%    0.0%    0.0%    0.0%    0.0%    0.0%    0.0%    0.0%    0.0%    0.0%    0.0%    0.0%    0.0%    0.0%    0.0%    0.0%    0.0%    0.0%    0.0%    0.0%    0.0%    0.0%    0.0%    0.0%    0.0%    0.0%    0.0%    0.0%    0.0%    0.0%    0.0%    0.0%    0.0%    0.0%    0.0%    0.0%    0.0%    0.0%    0.0%    0.0%    0.0%    0.0%    0.0%    0.0%    0.0%    0.0%    0.0%    0.0%    0.0%    0.0%    0.0%    0.0%    0.0%    0.0%    0.0%    0.0%    0.0%    0.0%    0.0%    0.0%    0.0%    0.0%    0.0%    0.0%    0.0%    0.0%    0.0%    0.0%    0.0%    0.0%        k__Bacteria; p__TM7     227    0.0%    0.0%    0.0%    0.0%    0.0%    0.0%    0.0%    0.0%    0.0%    0.0%    0.0%    0.0%    0.0%    0.0%    0.0%    0.0%    0.0%    0.0%    0.0%    0.0%    0.0%    0.0%    0.0%    0.0%    0.0%    0.0%    0.0%    0.0%    0.0%    0.0%    0.0%    0.0%    0.0%    0.0%    0.0%    0.0%    0.0%    0.0%    0.0%    0.0%    0.0%    0.0%    0.0%    0.0%    0.0%    0.0%    0.0%    0.0%    0.0%    0.0%    0.0%    0.0%    0.0%    0.0%    0.0%    0.0%    0.0%    0.0%    0.0%    0.0%    0.0%    0.0%    0.0%    0.0%    0.0%    0.0%    0.0%    0.0%    0.0%    0.0%    0.0%    0.0%    0.0%    0.0%    0.0%    0.0%    0.0%    0.0%    0.0%    0.0%    0.0%    0.0%    0.0%    0.0%    0.0%    0.0%    0.0%    0.0%    0.0%    0.0%    0.0%    0.0%    0.0%    0.0%    0.0%    0.0%    0.0%    0.0%    0.0%    0.0%    0.0%    0.0%    0.0%    0.0%    0.0%    0.0%    0.0%    0.0%    0.0%    0.0%    0.0%    0.0%    0.0%    0.0%    0.0%    0.0%    0.0%    0.0%    0.0%    0.0%    0.0%    0.0%    0.0%    0.0%    0.0%    0.0%    0.0%    0.0%    0.0%    0.0%    0.0%    0.0%    0.0%    0.0%    0.0%    0.0%    0.0%    0.0%    0.0%    0.0%    0.0%    0.0%    0.0%    0.0%    0.0%    0.1%    0.0%    0.0%    0.0%    0.0%    0.0%    0.0%    0.0%    0.0%    0.0%    0.0%    0.0%    0.0%    0.0%    0.0%    0.0%    0.0%    0.0%    0.0%    0.0%    0.0%    0.0%    0.0%    0.0%    0.0%    0.0%    0.0%    0.0%    0.0%    0.0%    0.0%    0.0%    0.0%    0.0%    0.0%    0.0%    0.0%    0.0%    0.0%    0.0%    0.0%    0.0%    0.0%    0.0%    0.0%    0.0%    0.0%    0.0%    0.0%    0.0%    0.0%    0.0%    0.0%    0.0%    0.0%    0.0%    0.0%    0.0%    0.0%    0.0%    0.0%    0.0%    0.0%    0.0%    0.0%    0.0%    0.0%    0.0%    0.0%    0.0%    0.1%    0.0%    0.0%    0.0%    0.0%    0.0%    0.0%    0.0%    0.0%    0.0%    0.0%    0.0%    0.0%    0.0%    0.0%    0.0%    0.0%    0.0%    0.0%    0.0%    0.0%    0.0%    0.0%    0.0%    0.0%    0.0%    0.0%    0.0%    0.0%    0.0%    0.0%    0.0%    0.0%    0.0%    0.0%    0.0%    0.0%    0.0%    0.0%    0.0%    0.0%    0.0%    0.0%    0.0%    0.0%    0.0%    0.0%    0.0%    0.0%    0.0%    0.0%    0.0%    0.0%    0.0%    0.0%    0.0%    0.0%    0.0%    0.0%    0.0%    0.0%    0.0%    0.0%    0.0%    0.0%    0.0%    0.0%    0.0%    0.0%    0.0%    0.0%    0.0%    0.0%    0.0%    0.0%    0.0%    0.0%    0.0%    0.0%    0.0%    0.0%    0.0%    0.0%    0.0%    0.0%    0.0%    0.0%    0.0%    0.0%    0.0%    0.0%    0.0%    0.0%    0.0%    0.0%    0.0%    0.0%    0.0%    0.0%    0.0%    0.0%    0.0%    0.0%    0.0%    0.0%    0.0%    0.0%    0.0%    0.0%    0.0%    0.0%    0.0%    0.0%    0.0%    0.0%    0.0%    0.0%    0.0%    0.0%    0.0%    0.0%    0.0%    0.0%    0.0%    0.0%    0.0%    0.0%    0.0%    0.0%    0.0%    0.0%    0.0%    0.0%    0.0%    0.0%    0.0%    0.0%    0.0%    0.0%    0.0%    0.0%    0.0%    0.0%    0.0%    0.0%    0.0%    0.0%        k__Bacteria; p__Tenericutes   16163    0.3%    0.1%    0.1%    0.4%    0.1%    0.0%    0.0%    0.0%    0.0%    0.0%    0.0%    0.0%    0.1%    0.6%    0.1%    0.1%    0.1%    0.1%    0.5%    0.2%    0.1%    1.9%    0.7%    0.0%    0.0%    0.0%    0.1%    0.1%    0.9%    0.8%    0.3%    0.1%    0.2%    0.6%    1.3%    0.2%    0.5%    0.1%    0.3%    0.6%    0.9%    1.0%    0.1%    0.3%    0.6%    1.2%    0.2%    0.1%    0.2%    0.3%    0.1%    0.1%    0.1%    0.2%    0.6%    0.5%    0.5%    0.3%    0.6%    0.1%    0.1%    0.2%    0.1%    0.1%    0.6%    0.2%    0.0%    0.3%    0.1%    0.1%    0.0%    0.2%    0.2%    0.7%    0.2%    0.1%    0.1%    0.8%    0.3%    0.6%    0.6%    0.2%    0.2%    0.8%    0.1%    0.0%    0.3%    0.0%    0.1%    0.4%    0.0%    0.1%    0.2%    0.1%    0.5%    0.3%    0.0%    1.2%    1.0%    0.6%    0.2%    0.3%    0.1%    0.0%    0.0%    1.1%    0.0%    0.0%    0.1%    0.4%    0.3%    1.2%    0.1%    0.0%    0.6%    0.1%    0.1%    0.1%    0.0%    0.1%    0.0%    0.1%    0.5%    0.1%    0.1%    0.2%    0.7%    0.0%    0.3%    1.2%    0.5%    0.1%    0.0%    0.6%    0.7%    0.1%    0.4%    0.2%    0.2%    0.0%    0.3%    0.8%    0.0%    0.2%    0.2%    0.3%    0.1%    0.0%    0.1%    0.2%    0.6%    0.7%    0.1%    0.1%    0.8%    0.1%    0.1%    2.2%    3.7%    1.1%    2.9%    0.2%    0.4%    0.3%    0.6%    0.1%    0.4%    0.1%    0.6%    1.1%    0.3%    0.2%    0.1%    0.1%    0.2%    0.6%    0.1%    0.1%    0.0%    0.0%    0.3%    0.0%    0.3%    0.0%    0.0%    0.1%    0.0%    0.0%    0.0%    0.1%    0.1%    0.1%    0.2%    1.0%    0.0%    0.1%    0.0%    0.8%    0.1%    0.4%    0.2%    0.3%    0.2%    1.1%    0.3%    0.6%    1.1%    0.6%    0.1%    0.1%    0.0%    0.1%    0.2%    0.1%    0.1%    0.5%    0.0%    0.0%    0.0%    0.4%    0.2%    0.1%    0.2%    0.0%    0.4%    0.1%    0.3%    0.3%    0.4%    0.1%    0.0%    0.0%    0.2%    0.0%    0.5%    0.0%    0.2%    0.1%    0.0%    0.0%    0.0%    0.0%    0.1%    0.0%    0.0%    0.0%    0.1%    0.1%    0.2%    0.0%    0.1%    0.3%    0.7%    0.2%    0.1%    0.1%    0.3%    0.1%    0.1%    0.1%    0.4%    0.5%    0.4%    0.0%    0.4%    0.2%    0.5%    0.0%    0.2%    0.3%    1.0%    0.3%    0.2%    0.1%    0.1%    0.0%    0.2%    0.1%    0.3%    0.3%    0.0%    0.0%    0.0%    0.0%    0.1%    0.1%    0.1%    0.3%    0.2%    0.0%    0.0%    0.0%    0.1%    0.2%    0.2%    0.1%    0.0%    0.1%    0.1%    0.2%    0.0%    0.1%    0.0%    0.2%    0.1%    1.1%    0.3%    0.2%    0.3%    0.5%    0.1%    0.1%    0.5%    0.0%    0.1%    0.1%    0.4%    0.0%    0.0%    0.3%    0.5%    0.4%    0.0%    0.0%    0.2%    0.1%    0.1%    0.1%    0.0%    0.1%    0.0%    0.0%    0.1%    0.0%    0.0%    0.0%    0.0%    0.1%    0.2%    0.1%    0.1%    0.0%    0.1%    0.1%    0.1%    0.1%    0.0%    0.0%    0.1%    0.0%    0.1%    0.0%    0.1%    0.1%    0.1%    0.0%    2.3%    0.0%    0.0%    0.0%    0.0%    0.0%        k__Bacteria; p__Thermi    1925    0.0%    0.0%    0.0%    0.0%    0.0%    0.0%    0.0%    0.5%    0.1%    0.0%    0.0%    0.3%    0.0%    0.0%    0.0%    0.0%    0.0%    0.0%    0.0%    0.0%    0.0%    0.0%    0.1%    0.0%    0.0%    0.0%    0.0%    0.0%    0.1%    0.0%    0.0%    0.0%    0.0%    0.0%    0.0%    0.0%    0.0%    0.0%    0.0%    0.0%    0.0%    0.0%    0.0%    0.0%    0.0%    0.0%    0.0%    0.0%    0.0%    0.0%    0.0%    0.0%    0.0%    0.0%    0.0%    0.1%    0.1%    0.0%    0.0%    0.0%    0.0%    0.2%    0.0%    0.0%    0.0%    0.1%    0.0%    0.1%    0.0%    0.0%    0.0%    0.0%    0.0%    0.0%    0.0%    0.0%    0.0%    0.0%    0.0%    0.0%    0.0%    0.1%    0.0%    0.0%    0.0%    0.0%    0.0%    0.0%    0.0%    0.0%    0.0%    0.0%    0.0%    0.0%    0.0%    0.1%    0.0%    0.0%    0.0%    0.0%    0.0%    0.0%    0.1%    0.0%    0.0%    0.1%    0.0%    0.0%    0.0%    0.1%    0.1%    0.2%    0.0%    0.0%    0.0%    0.0%    0.0%    0.0%    0.0%    0.0%    0.0%    0.0%    0.1%    0.1%    0.0%    0.1%    0.1%    0.0%    0.0%    0.1%    0.0%    0.0%    0.1%    0.0%    0.1%    0.0%    0.1%    0.0%    0.0%    0.0%    0.0%    0.0%    0.0%    0.1%    0.0%    0.0%    0.0%    0.0%    0.0%    0.0%    0.1%    0.0%    0.0%    0.0%    0.1%    0.0%    0.0%    0.0%    0.0%    0.1%    0.0%    0.0%    0.1%    0.0%    0.0%    0.0%    0.0%    0.7%    0.0%    0.0%    0.1%    1.8%    0.2%    0.0%    0.0%    0.0%    0.0%    0.0%    0.0%    0.0%    0.0%    0.0%    0.0%    0.0%    0.0%    0.0%    0.0%    0.0%    0.1%    0.0%    0.1%    0.0%    0.0%    0.0%    0.1%    0.1%    0.0%    0.0%    0.0%    0.0%    0.1%    0.0%    0.0%    0.1%    0.0%    0.0%    0.0%    0.1%    0.1%    0.0%    0.0%    0.1%    0.1%    0.3%    0.0%    0.2%    0.0%    0.1%    0.2%    0.0%    0.0%    0.0%    0.0%    0.0%    0.1%    0.0%    0.1%    0.0%    0.1%    0.0%    0.0%    0.0%    0.1%    0.0%    0.1%    0.0%    0.1%    0.0%    0.1%    0.1%    0.0%    0.0%    0.1%    0.0%    0.1%    0.0%    0.0%    0.0%    0.0%    0.0%    0.0%    0.0%    0.2%    0.0%    0.0%    0.0%    0.0%    0.0%    0.0%    0.0%    0.0%    0.1%    0.0%    0.0%    0.0%    0.0%    0.0%    0.0%    0.0%    0.0%    0.0%    0.0%    0.1%    0.0%    0.0%    0.0%    0.0%    0.0%    0.0%    0.0%    0.0%    0.0%    0.0%    0.0%    0.0%    0.0%    0.0%    0.0%    0.0%    0.0%    0.1%    0.0%    0.0%    0.2%    0.0%    0.1%    0.0%    0.0%    0.0%    0.1%    0.0%    0.0%    0.0%    0.0%    0.0%    0.0%    0.0%    0.0%    0.0%    0.0%    0.0%    0.0%    0.0%    0.0%    0.0%    0.0%    0.1%    0.0%    0.0%    0.0%    0.0%    0.1%    0.0%    0.0%    0.0%    0.1%    0.0%    0.0%    0.0%    0.1%    0.0%    0.0%    0.0%    0.1%    0.0%    0.0%    0.0%    0.0%    0.0%    0.0%    0.0%    0.0%    0.0%    0.0%    0.0%    0.0%    0.0%    0.0%    0.0%    0.0%    0.0%    0.0%    0.0%    0.0%    0.0%    0.0%    0.0%    0.0%    0.6%    0.0%    0.0%    0.1%        k__Bacteria; p__Thermotogae       3    0.0%    0.0%    0.0%    0.0%    0.0%    0.0%    0.0%    0.0%    0.0%    0.0%    0.0%    0.0%    0.0%    0.0%    0.0%    0.0%    0.0%    0.0%    0.0%    0.0%    0.0%    0.0%    0.0%    0.0%    0.0%    0.0%    0.0%    0.0%    0.0%    0.0%    0.0%    0.0%    0.0%    0.0%    0.0%    0.0%    0.0%    0.0%    0.0%    0.0%    0.0%    0.0%    0.0%    0.0%    0.0%    0.0%    0.0%    0.0%    0.0%    0.0%    0.0%    0.0%    0.0%    0.0%    0.0%    0.0%    0.0%    0.0%    0.0%    0.0%    0.0%    0.0%    0.0%    0.0%    0.0%    0.0%    0.0%    0.0%    0.0%    0.0%    0.0%    0.0%    0.0%    0.0%    0.0%    0.0%    0.0%    0.0%    0.0%    0.0%    0.0%    0.0%    0.0%    0.0%    0.0%    0.0%    0.0%    0.0%    0.0%    0.0%    0.0%    0.0%    0.0%    0.0%    0.0%    0.0%    0.0%    0.0%    0.0%    0.0%    0.0%    0.0%    0.0%    0.0%    0.0%    0.0%    0.0%    0.0%    0.0%    0.0%    0.0%    0.0%    0.0%    0.0%    0.0%    0.0%    0.0%    0.0%    0.0%    0.0%    0.0%    0.0%    0.0%    0.0%    0.0%    0.0%    0.0%    0.0%    0.0%    0.0%    0.0%    0.0%    0.0%    0.0%    0.0%    0.0%    0.0%    0.0%    0.0%    0.0%    0.0%    0.0%    0.0%    0.0%    0.0%    0.0%    0.0%    0.0%    0.0%    0.0%    0.0%    0.0%    0.0%    0.0%    0.0%    0.0%    0.0%    0.0%    0.0%    0.0%    0.0%    0.0%    0.0%    0.0%    0.0%    0.0%    0.0%    0.0%    0.0%    0.0%    0.0%    0.0%    0.0%    0.0%    0.0%    0.0%    0.0%    0.0%    0.0%    0.0%    0.0%    0.0%    0.0%    0.0%    0.0%    0.0%    0.0%    0.0%    0.0%    0.0%    0.0%    0.0%    0.0%    0.0%    0.0%    0.0%    0.0%    0.0%    0.0%    0.0%    0.0%    0.0%    0.0%    0.0%    0.0%    0.0%    0.0%    0.0%    0.0%    0.0%    0.0%    0.0%    0.0%    0.0%    0.0%    0.0%    0.0%    0.0%    0.0%    0.0%    0.0%    0.0%    0.0%    0.0%    0.0%    0.0%    0.0%    0.0%    0.0%    0.0%    0.0%    0.0%    0.0%    0.0%    0.0%    0.0%    0.0%    0.0%    0.0%    0.0%    0.0%    0.0%    0.0%    0.0%    0.0%    0.0%    0.0%    0.0%    0.0%    0.0%    0.0%    0.0%    0.0%    0.0%    0.0%    0.0%    0.0%    0.0%    0.0%    0.0%    0.0%    0.0%    0.0%    0.0%    0.0%    0.0%    0.0%    0.0%    0.0%    0.0%    0.0%    0.0%    0.0%    0.0%    0.0%    0.0%    0.0%    0.0%    0.0%    0.0%    0.0%    0.0%    0.0%    0.0%    0.0%    0.0%    0.0%    0.0%    0.0%    0.0%    0.0%    0.0%    0.0%    0.0%    0.0%    0.0%    0.0%    0.0%    0.0%    0.0%    0.0%    0.0%    0.0%    0.0%    0.0%    0.0%    0.0%    0.0%    0.0%    0.0%    0.0%    0.0%    0.0%    0.0%    0.0%    0.0%    0.0%    0.0%    0.0%    0.0%    0.0%    0.0%    0.0%    0.0%    0.0%    0.0%    0.0%    0.0%    0.0%    0.0%    0.0%    0.0%    0.0%    0.0%    0.0%    0.0%    0.0%    0.0%    0.0%    0.0%    0.0%    0.0%    0.0%    0.0%    0.0%    0.0%    0.0%    0.0%    0.0%    0.0%    0.0%    0.0%    0.0%    0.0%    0.0%    0.0%    0.0%    0.0%    0.0%    0.0%    0.0%    0.0%        k__Bacteria; p__Verrucomicrobia    3586    0.1%    0.0%    0.0%    0.0%    0.1%    0.0%    0.0%    0.0%    0.0%    0.0%    0.0%    0.1%    0.0%    0.1%    0.0%    0.0%    0.0%    0.0%    0.2%    0.0%    0.1%    0.1%    0.1%    0.1%    0.0%    0.0%    0.0%    0.1%    0.1%    0.0%    0.0%    0.0%    0.0%    0.0%    0.1%    0.0%    0.0%    0.0%    0.1%    0.0%    0.0%    0.1%    0.0%    0.0%    0.0%    0.0%    0.0%    0.0%    0.1%    0.0%    0.0%    0.0%    0.1%    0.1%    0.6%    0.2%    0.1%    0.0%    0.1%    0.1%    0.1%    0.0%    0.0%    0.1%    0.2%    0.1%    0.1%    0.4%    0.0%    0.0%    0.0%    0.0%    0.0%    0.1%    0.1%    0.0%    0.0%    0.1%    0.0%    0.0%    0.1%    0.1%    0.0%    0.0%    0.0%    0.1%    0.1%    0.0%    0.0%    0.2%    0.0%    0.1%    0.0%    0.0%    0.0%    0.0%    0.0%    0.1%    0.1%    0.1%    0.1%    0.0%    0.1%    0.1%    0.1%    0.1%    0.0%    0.0%    0.0%    0.1%    0.1%    0.0%    0.0%    0.0%    0.1%    0.0%    0.0%    0.1%    0.0%    1.2%    0.0%    0.0%    0.1%    0.0%    0.2%    0.4%    0.3%    0.0%    0.1%    0.3%    0.2%    0.0%    0.2%    0.1%    0.2%    0.0%    0.1%    0.1%    0.0%    0.0%    0.0%    0.0%    0.0%    0.1%    0.1%    0.0%    0.0%    0.0%    0.0%    0.0%    0.1%    0.1%    0.0%    0.0%    0.1%    0.0%    0.0%    0.1%    0.1%    0.0%    0.1%    0.0%    0.1%    0.0%    0.0%    0.0%    0.0%    0.1%    0.1%    0.1%    0.1%    0.0%    0.0%    0.0%    0.0%    0.0%    0.0%    0.0%    0.0%    0.0%    0.0%    0.0%    0.0%    0.0%    0.0%    0.0%    0.0%    0.0%    0.0%    0.0%    0.1%    0.0%    0.1%    0.1%    0.0%    0.1%    0.1%    0.0%    0.0%    0.2%    0.0%    0.0%    0.0%    0.2%    0.1%    0.0%    0.3%    0.1%    0.1%    0.0%    0.1%    0.1%    0.1%    0.1%    0.0%    0.0%    0.0%    0.1%    0.1%    0.1%    0.0%    0.0%    0.1%    0.0%    0.0%    0.0%    0.1%    0.1%    0.0%    0.0%    0.1%    0.0%    0.1%    0.0%    0.1%    0.0%    0.1%    0.0%    0.0%    0.0%    0.1%    0.0%    0.0%    0.0%    0.0%    0.0%    0.1%    0.0%    0.1%    0.1%    0.3%    0.1%    0.2%    0.1%    0.1%    0.0%    0.1%    0.1%    0.0%    0.1%    0.0%    0.0%    0.0%    0.0%    0.0%    0.1%    0.0%    0.0%    0.0%    0.1%    0.1%    0.1%    0.4%    0.8%    0.1%    0.0%    0.1%    0.0%    0.2%    0.1%    0.2%    0.1%    0.1%    0.0%    0.0%    0.0%    0.0%    0.0%    0.0%    0.0%    0.0%    0.0%    0.0%    0.1%    0.0%    0.0%    0.0%    0.0%    0.0%    0.0%    0.0%    0.0%    0.0%    0.1%    0.0%    0.2%    0.0%    0.0%    0.0%    0.0%    0.0%    0.0%    0.0%    0.0%    0.0%    0.2%    0.0%    0.0%    0.0%    0.1%    0.0%    0.1%    0.0%    0.1%    0.1%    0.0%    0.0%    0.0%    0.1%    0.0%    0.0%    0.0%    0.0%    0.0%    0.0%    0.0%    0.0%    0.0%    0.0%    0.0%    0.0%    0.0%    0.0%    0.0%    0.0%    0.0%    0.0%    0.0%    0.0%    0.0%    0.0%    0.1%    0.0%    0.0%    0.1%    0.0%    0.0%    0.0%    0.0%    0.0%    0.2%    0.1%        k__Bacteria; p__WPS-2      33    0.0%    0.0%    0.0%    0.0%    0.0%    0.0%    0.0%    0.0%    0.0%    0.0%    0.0%    0.0%    0.0%    0.0%    0.0%    0.0%    0.0%    0.0%    0.0%    0.0%    0.0%    0.0%    0.0%    0.0%    0.0%    0.0%    0.0%    0.0%    0.0%    0.0%    0.0%    0.0%    0.0%    0.0%    0.0%    0.0%    0.0%    0.0%    0.0%    0.0%    0.0%    0.0%    0.0%    0.0%    0.0%    0.0%    0.0%    0.0%    0.0%    0.0%    0.0%    0.0%    0.0%    0.0%    0.0%    0.0%    0.0%    0.0%    0.0%    0.0%    0.0%    0.0%    0.0%    0.0%    0.0%    0.0%    0.0%    0.0%    0.0%    0.0%    0.0%    0.0%    0.0%    0.0%    0.0%    0.0%    0.0%    0.0%    0.0%    0.0%    0.0%    0.0%    0.0%    0.0%    0.0%    0.0%    0.0%    0.0%    0.0%    0.0%    0.0%    0.0%    0.0%    0.0%    0.0%    0.0%    0.0%    0.0%    0.0%    0.0%    0.0%    0.0%    0.0%    0.0%    0.0%    0.0%    0.0%    0.0%    0.0%    0.0%    0.0%    0.0%    0.0%    0.0%    0.0%    0.0%    0.0%    0.0%    0.0%    0.0%    0.0%    0.0%    0.0%    0.0%    0.0%    0.0%    0.0%    0.0%    0.0%    0.0%    0.0%    0.0%    0.0%    0.0%    0.0%    0.0%    0.0%    0.0%    0.0%    0.0%    0.0%    0.0%    0.0%    0.0%    0.0%    0.0%    0.0%    0.0%    0.0%    0.0%    0.0%    0.0%    0.0%    0.0%    0.0%    0.0%    0.0%    0.0%    0.0%    0.0%    0.0%    0.0%    0.0%    0.0%    0.0%    0.0%    0.0%    0.0%    0.0%    0.0%    0.0%    0.0%    0.0%    0.0%    0.0%    0.0%    0.0%    0.0%    0.0%    0.0%    0.0%    0.0%    0.0%    0.0%    0.0%    0.0%    0.0%    0.0%    0.0%    0.0%    0.0%    0.0%    0.0%    0.0%    0.0%    0.0%    0.0%    0.0%    0.0%    0.0%    0.0%    0.0%    0.0%    0.0%    0.0%    0.0%    0.0%    0.0%    0.0%    0.0%    0.0%    0.0%    0.0%    0.0%    0.0%    0.0%    0.0%    0.0%    0.0%    0.0%    0.0%    0.0%    0.0%    0.0%    0.0%    0.0%    0.0%    0.0%    0.0%    0.0%    0.0%    0.0%    0.0%    0.0%    0.0%    0.0%    0.0%    0.0%    0.0%    0.0%    0.0%    0.0%    0.0%    0.0%    0.0%    0.0%    0.0%    0.0%    0.0%    0.0%    0.0%    0.0%    0.0%    0.0%    0.0%    0.0%    0.0%    0.0%    0.0%    0.0%    0.0%    0.0%    0.0%    0.0%    0.0%    0.0%    0.0%    0.0%    0.0%    0.0%    0.0%    0.0%    0.0%    0.0%    0.0%    0.0%    0.0%    0.0%    0.0%    0.1%    0.0%    0.0%    0.0%    0.0%    0.0%    0.0%    0.0%    0.0%    0.0%    0.0%    0.0%    0.0%    0.0%    0.0%    0.0%    0.0%    0.0%    0.0%    0.0%    0.0%    0.0%    0.0%    0.0%    0.0%    0.0%    0.0%    0.0%    0.0%    0.0%    0.0%    0.0%    0.0%    0.0%    0.0%    0.0%    0.0%    0.0%    0.0%    0.0%    0.0%    0.0%    0.0%    0.0%    0.0%    0.0%    0.0%    0.0%    0.0%    0.0%    0.0%    0.0%    0.0%    0.0%    0.0%    0.0%    0.0%    0.0%    0.0%    0.0%    0.0%    0.0%    0.0%    0.0%    0.0%    0.0%    0.0%    0.0%    0.0%    0.0%    0.0%    0.0%    0.0%    0.0%    0.0%    0.0%    0.0%    0.0%    0.0%    0.0%    0.0%    0.0%    0.0%        k__Bacteria; p__WS3       8    0.0%    0.0%    0.0%    0.0%    0.0%    0.0%    0.0%    0.0%    0.0%    0.0%    0.0%    0.0%    0.0%    0.0%    0.0%    0.0%    0.0%    0.0%    0.0%    0.0%    0.0%    0.0%    0.0%    0.0%    0.0%    0.0%    0.0%    0.0%    0.0%    0.0%    0.0%    0.0%    0.0%    0.0%    0.0%    0.0%    0.0%    0.0%    0.0%    0.0%    0.0%    0.0%    0.0%    0.0%    0.0%    0.0%    0.0%    0.0%    0.0%    0.0%    0.0%    0.0%    0.0%    0.0%    0.0%    0.0%    0.0%    0.0%    0.0%    0.0%    0.0%    0.0%    0.0%    0.0%    0.0%    0.0%    0.0%    0.0%    0.0%    0.0%    0.0%    0.0%    0.0%    0.0%    0.0%    0.0%    0.0%    0.0%    0.0%    0.0%    0.0%    0.0%    0.0%    0.0%    0.0%    0.0%    0.0%    0.0%    0.0%    0.0%    0.0%    0.0%    0.0%    0.0%    0.0%    0.0%    0.0%    0.0%    0.0%    0.0%    0.0%    0.0%    0.0%    0.0%    0.0%    0.0%    0.0%    0.0%    0.0%    0.0%    0.0%    0.0%    0.0%    0.0%    0.0%    0.0%    0.0%    0.0%    0.0%    0.0%    0.0%    0.0%    0.0%    0.0%    0.0%    0.0%    0.0%    0.0%    0.0%    0.0%    0.0%    0.0%    0.0%    0.0%    0.0%    0.0%    0.0%    0.0%    0.0%    0.0%    0.0%    0.0%    0.0%    0.0%    0.0%    0.0%    0.0%    0.0%    0.0%    0.0%    0.0%    0.0%    0.0%    0.0%    0.0%    0.0%    0.0%    0.0%    0.0%    0.0%    0.0%    0.0%    0.0%    0.0%    0.0%    0.0%    0.0%    0.0%    0.0%    0.0%    0.0%    0.0%    0.0%    0.0%    0.0%    0.0%    0.0%    0.0%    0.0%    0.0%    0.0%    0.0%    0.0%    0.0%    0.0%    0.0%    0.0%    0.0%    0.0%    0.0%    0.0%    0.0%    0.0%    0.0%    0.0%    0.0%    0.0%    0.0%    0.0%    0.0%    0.0%    0.0%    0.0%    0.0%    0.0%    0.0%    0.0%    0.0%    0.0%    0.0%    0.0%    0.0%    0.0%    0.0%    0.0%    0.0%    0.0%    0.0%    0.0%    0.0%    0.0%    0.0%    0.0%    0.0%    0.0%    0.0%    0.0%    0.0%    0.0%    0.0%    0.0%    0.0%    0.0%    0.0%    0.0%    0.0%    0.0%    0.0%    0.0%    0.0%    0.0%    0.0%    0.0%    0.0%    0.0%    0.0%    0.0%    0.0%    0.0%    0.0%    0.0%    0.0%    0.0%    0.0%    0.0%    0.0%    0.0%    0.0%    0.0%    0.0%    0.0%    0.0%    0.0%    0.0%    0.0%    0.0%    0.0%    0.0%    0.0%    0.0%    0.0%    0.0%    0.0%    0.0%    0.0%    0.0%    0.0%    0.0%    0.0%    0.0%    0.0%    0.0%    0.0%    0.0%    0.0%    0.0%    0.0%    0.0%    0.0%    0.0%    0.0%    0.0%    0.0%    0.0%    0.0%    0.0%    0.0%    0.0%    0.0%    0.0%    0.0%    0.0%    0.0%    0.0%    0.0%    0.0%    0.0%    0.0%    0.0%    0.0%    0.0%    0.0%    0.0%    0.0%    0.0%    0.0%    0.0%    0.0%    0.0%    0.0%    0.0%    0.0%    0.0%    0.0%    0.0%    0.0%    0.0%    0.0%    0.0%    0.0%    0.0%    0.0%    0.0%    0.0%    0.0%    0.0%    0.0%    0.0%    0.0%    0.0%    0.0%    0.0%    0.0%    0.0%    0.0%    0.0%    0.0%    0.0%    0.0%    0.0%    0.0%    0.0%    0.0%    0.0%    0.0%    0.0%    0.0%    0.0%    0.0%    0.0%    0.0%    0.0%        k__Bacteria; p__ZB2       2    0.0%    0.0%    0.0%    0.0%    0.0%    0.0%    0.0%    0.0%    0.0%    0.0%    0.0%    0.0%    0.0%    0.0%    0.0%    0.0%    0.0%    0.0%    0.0%    0.0%    0.0%    0.0%    0.0%    0.0%    0.0%    0.0%    0.0%    0.0%    0.0%    0.0%    0.0%    0.0%    0.0%    0.0%    0.0%    0.0%    0.0%    0.0%    0.0%    0.0%    0.0%    0.0%    0.0%    0.0%    0.0%    0.0%    0.0%    0.0%    0.0%    0.0%    0.0%    0.0%    0.0%    0.0%    0.0%    0.0%    0.0%    0.0%    0.0%    0.0%    0.0%    0.0%    0.0%    0.0%    0.0%    0.0%    0.0%    0.0%    0.0%    0.0%    0.0%    0.0%    0.0%    0.0%    0.0%    0.0%    0.0%    0.0%    0.0%    0.0%    0.0%    0.0%    0.0%    0.0%    0.0%    0.0%    0.0%    0.0%    0.0%    0.0%    0.0%    0.0%    0.0%    0.0%    0.0%    0.0%    0.0%    0.0%    0.0%    0.0%    0.0%    0.0%    0.0%    0.0%    0.0%    0.0%    0.0%    0.0%    0.0%    0.0%    0.0%    0.0%    0.0%    0.0%    0.0%    0.0%    0.0%    0.0%    0.0%    0.0%    0.0%    0.0%    0.0%    0.0%    0.0%    0.0%    0.0%    0.0%    0.0%    0.0%    0.0%    0.0%    0.0%    0.0%    0.0%    0.0%    0.0%    0.0%    0.0%    0.0%    0.0%    0.0%    0.0%    0.0%    0.0%    0.0%    0.0%    0.0%    0.0%    0.0%    0.0%    0.0%    0.0%    0.0%    0.0%    0.0%    0.0%    0.0%    0.0%    0.0%    0.0%    0.0%    0.0%    0.0%    0.0%    0.0%    0.0%    0.0%    0.0%    0.0%    0.0%    0.0%    0.0%    0.0%    0.0%    0.0%    0.0%    0.0%    0.0%    0.0%    0.0%    0.0%    0.0%    0.0%    0.0%    0.0%    0.0%    0.0%    0.0%    0.0%    0.0%    0.0%    0.0%    0.0%    0.0%    0.0%    0.0%    0.0%    0.0%    0.0%    0.0%    0.0%    0.0%    0.0%    0.0%    0.0%    0.0%    0.0%    0.0%    0.0%    0.0%    0.0%    0.0%    0.0%    0.0%    0.0%    0.0%    0.0%    0.0%    0.0%    0.0%    0.0%    0.0%    0.0%    0.0%    0.0%    0.0%    0.0%    0.0%    0.0%    0.0%    0.0%    0.0%    0.0%    0.0%    0.0%    0.0%    0.0%    0.0%    0.0%    0.0%    0.0%    0.0%    0.0%    0.0%    0.0%    0.0%    0.0%    0.0%    0.0%    0.0%    0.0%    0.0%    0.0%    0.0%    0.0%    0.0%    0.0%    0.0%    0.0%    0.0%    0.0%    0.0%    0.0%    0.0%    0.0%    0.0%    0.0%    0.0%    0.0%    0.0%    0.0%    0.0%    0.0%    0.0%    0.0%    0.0%    0.0%    0.0%    0.0%    0.0%    0.0%    0.0%    0.0%    0.0%    0.0%    0.0%    0.0%    0.0%    0.0%    0.0%    0.0%    0.0%    0.0%    0.0%    0.0%    0.0%    0.0%    0.0%    0.0%    0.0%    0.0%    0.0%    0.0%    0.0%    0.0%    0.0%    0.0%    0.0%    0.0%    0.0%    0.0%    0.0%    0.0%    0.0%    0.0%    0.0%    0.0%    0.0%    0.0%    0.0%    0.0%    0.0%    0.0%    0.0%    0.0%    0.0%    0.0%    0.0%    0.0%    0.0%    0.0%    0.0%    0.0%    0.0%    0.0%    0.0%    0.0%    0.0%    0.0%    0.0%    0.0%    0.0%    0.0%    0.0%    0.0%    0.0%    0.0%    0.0%    0.0%    0.0%    0.0%    0.0%    0.0%    0.0%    0.0%    0.0%    0.0%    0.0%    0.0%    0.0%    0.0%    
     
  Taxonomy Summary. Current Level: Class  
     View Figure (.pdf)    View Legend (.pdf)   
  
[truncated: 9,584,449 more chars]
