# Supplementary material for: Moving pictures of the human microbiome
Source: Genome Biol. 2011 May 30;12(5):R50. doi: 10.1186/gb-2011-12-5-r50 (PMC3271711; doi:10.1186/gb-2011-12-5-r50)
Supplement: Additional file 13 — Temporal variation in phylum, class, order, family, and genus abundances (F4 left palm). The x-axis scale differs between M3 and F4 plots. [file gb-2011-12-5-r50-S13.ZIP › AdditionalFile13/charts/3NKb2AJoqN2YcXxMEGY0kmrpN0CW0l_legend.pdf]

k__Archaea;p__Crenarchaeota;c__Sd-NA;o__NRP-J
k__Archaea;p__Crenarchaeota;c__Thaumarchaeota;o__Cenarchaeales
k__Archaea;p__Crenarchaeota;c__Thaumarchaeota;o__Nitrososphaerales
k__Archaea;p__Euryarchaeota;c__Halobacteria;o__Halobacteriales
k__Archaea;p__Euryarchaeota;c__Methanobacteria;o__Methanobacteriales
k__Archaea;p__Euryarchaeota;c__Methanomicrobia;o__Methanomicrobiales
k__Archaea;p__Euryarchaeota;c__Methanomicrobia;o__Methanosarcinales
k__Archaea;p__Euryarchaeota;c__Thermoplasmata;o__E2
k__Bacteria;p__;c__;o__
k__Bacteria;p__ABY1_OD1;c__;o__
k__Bacteria;p__AD3;c__ABS-6;o__
k__Bacteria;p__AD3;c__JG37-AG-4;o__
k__Bacteria;p__Acidobacteria;c__;o__
k__Bacteria;p__Acidobacteria;c__Acidobacteria (class);o__Acidobacteriales
k__Bacteria;p__Acidobacteria;c__Acidobacteria-5;o__
k__Bacteria;p__Acidobacteria;c__Chloracidobacteria;o__
k__Bacteria;p__Acidobacteria;c__Holophagae;o__Holophagales
k__Bacteria;p__Acidobacteria;c__MVS-40;o__
k__Bacteria;p__Acidobacteria;c__OS-K;o__
k__Bacteria;p__Acidobacteria;c__PAUC37f;o__
k__Bacteria;p__Acidobacteria;c__RB25;o__
k__Bacteria;p__Acidobacteria;c__Solibacteres;o__Solibacterales
k__Bacteria;p__Acidobacteria;c__Sva0725;o__
k__Bacteria;p__Acidobacteria;c__iii1-8;o__
k__Bacteria;p__Actinobacteria;c__;o__
k__Bacteria;p__Actinobacteria;c__Actinobacteria (class);o__
k__Bacteria;p__Actinobacteria;c__Actinobacteria (class);o__0319-7L14
k__Bacteria;p__Actinobacteria;c__Actinobacteria (class);o__Acidimicrobiales
k__Bacteria;p__Actinobacteria;c__Actinobacteria (class);o__Actinomycetales
k__Bacteria;p__Actinobacteria;c__Actinobacteria (class);o__Bifidobacteriales
k__Bacteria;p__Actinobacteria;c__Actinobacteria (class);o__Coriobacteriales
k__Bacteria;p__Actinobacteria;c__Actinobacteria (class);o__Euzebiales
k__Bacteria;p__Actinobacteria;c__Actinobacteria (class);o__MC47
k__Bacteria;p__Actinobacteria;c__Actinobacteria (class);o__Rubrobacterales
k__Bacteria;p__Actinobacteria;c__Actinobacteria (class);o__Solirubrobacterales
k__Bacteria;p__Actinobacteria;c__Actinobacteria (class);o__WCHB1-81
k__Bacteria;p__Actinobacteria;c__Actinobacteria (class);o__koll13
k__Bacteria;p__Aquificae;c__Aquificae (class);o__Aquificales
k__Bacteria;p__BRC1;c__;o__
k__Bacteria;p__BRC1;c__PRR-11;o__
k__Bacteria;p__Bacteroidetes;c__;o__
k__Bacteria;p__Bacteroidetes;c__Bacteroidia;o__Bacteroidales
k__Bacteria;p__Bacteroidetes;c__Flavobacteria;o__
k__Bacteria;p__Bacteroidetes;c__Flavobacteria;o__Flavobacteriales
k__Bacteria;p__Bacteroidetes;c__Sphingobacteria;o__Sphingobacteriales
k__Bacteria;p__CCM11b;c__;o__
k__Bacteria;p__Caldithrix_KSB1;c__Caldithrixae;o__Caldithrixales
k__Bacteria;p__Chlamydiae;c__Chlamydiae (class);o__Chlamydiales
k__Bacteria;p__Chlorobi;c__;o__
k__Bacteria;p__Chlorobi;c__BSV19;o__
k__Bacteria;p__Chlorobi;c__OPB56;o__
k__Bacteria;p__Chlorobi;c__SJA-28;o__
k__Bacteria;p__Chlorobi;c__ZB1;o__
k__Bacteria;p__Chloroflexi;c__;o__
k__Bacteria;p__Chloroflexi;c__Anaerolineae;o__
k__Bacteria;p__Chloroflexi;c__Anaerolineae;o__A31
k__Bacteria;p__Chloroflexi;c__Anaerolineae;o__A4b
k__Bacteria;p__Chloroflexi;c__Anaerolineae;o__Anaerolineales
k__Bacteria;p__Chloroflexi;c__Anaerolineae;o__CFB-26
k__Bacteria;p__Chloroflexi;c__Anaerolineae;o__Caldilineales
k__Bacteria;p__Chloroflexi;c__Anaerolineae;o__DRC31
k__Bacteria;p__Chloroflexi;c__Anaerolineae;o__GCA004
k__Bacteria;p__Chloroflexi;c__Anaerolineae;o__H39
k__Bacteria;p__Chloroflexi;c__Anaerolineae;o__S0208
k__Bacteria;p__Chloroflexi;c__Anaerolineae;o__SB-34
k__Bacteria;p__Chloroflexi;c__Anaerolineae;o__SJA-101
k__Bacteria;p__Chloroflexi;c__Anaerolineae;o__SJA-15
k__Bacteria;p__Chloroflexi;c__Anaerolineae;o__WCHB1-50
k__Bacteria;p__Chloroflexi;c__Anaerolineae;o__envOPS12
k__Bacteria;p__Chloroflexi;c__Bljii12;o__
k__Bacteria;p__Chloroflexi;c__Chloroflexi (class);o__Herpetosiphonales
k__Bacteria;p__Chloroflexi;c__Chloroflexi (class);o__Roseiflexales
k__Bacteria;p__Chloroflexi;c__Chloroflexi-4;o__
k__Bacteria;p__Chloroflexi;c__Ktedonobacteria;o__
k__Bacteria;p__Chloroflexi;c__SOGA31;o__
k__Bacteria;p__Chloroflexi;c__TK17;o__
k__Bacteria;p__Chloroflexi;c__Thermobacula;o__Thermobaculales
k__Bacteria;p__Chloroflexi;c__Thermomicrobia;o__
k__Bacteria;p__Chloroflexi;c__Thermomicrobia;o__HN1-15
k__Bacteria;p__Chloroflexi;c__Thermomicrobia;o__Thermomicrobiales
k__Bacteria;p__Cyanobacteria;c__;o__
k__Bacteria;p__Cyanobacteria;c__;o__Chroococcales
k__Bacteria;p__Cyanobacteria;c__;o__Nostocales
k__Bacteria;p__Cyanobacteria;c__;o__Oscillatoriales
k__Bacteria;p__Cyanobacteria;c__;o__Pleurocapsales
k__Bacteria;p__Cyanobacteria;c__;o__Stigonematales
k__Bacteria;p__Cyanobacteria;c__S15B-MN24;o__
k__Bacteria;p__Cyanobacteria;c__SM1D11;o__
k__Bacteria;p__Cyanobacteria;c__YS2;o__
k__Bacteria;p__Cyanobacteria;c__mle1-12;o__
k__Bacteria;p__Deferribacteres;c__Deferribacteres (class);o__Deferribacterales
k__Bacteria;p__Elusimicrobia;c__Elusimicrobia (class);o__
k__Bacteria;p__Elusimicrobia;c__Elusimicrobia (class);o__Elusimicrobiales
k__Bacteria;p__Fibrobacteres;c__Fibrobacteres (class);o__Fibrobacterales
k__Bacteria;p__Firmicutes;c__Bacilli;o__
k__Bacteria;p__Firmicutes;c__Bacilli;o__Bacillales
k__Bacteria;p__Firmicutes;c__Bacilli;o__Erysipelotrichales
k__Bacteria;p__Firmicutes;c__Bacilli;o__Lactobacillales
k__Bacteria;p__Firmicutes;c__Clostridia;o__
k__Bacteria;p__Firmicutes;c__Clostridia;o__1B07
k__Bacteria;p__Firmicutes;c__Clostridia;o__BSA2B-08
k__Bacteria;p__Firmicutes;c__Clostridia;o__Clostridiales
k__Bacteria;p__Firmicutes;c__Clostridia;o__Desulfitobacterales
k__Bacteria;p__Firmicutes;c__Clostridia;o__Halanaerobiales
k__Bacteria;p__Firmicutes;c__Clostridia;o__MBA08
k__Bacteria;p__Firmicutes;c__Clostridia;o__Natranaerobiales
k__Bacteria;p__Firmicutes;c__Clostridia;o__OPB54
k__Bacteria;p__Firmicutes;c__Clostridia;o__Thermoanaerobacterales
k__Bacteria;p__Fusobacteria;c__Fusobacteria (class);o__Fusobacteriales
k__Bacteria;p__GN02;c__;o__
k__Bacteria;p__GN02;c__VC12-cl04;o__
k__Bacteria;p__Gemmatimonadetes;c__Gemmatimonadetes (class);o__
k__Bacteria;p__Gemmatimonadetes;c__Gemmatimonadetes (class);o__Gemmatimonadales
k__Bacteria;p__Lentisphaerae;c__;o__
k__Bacteria;p__Lentisphaerae;c__Lentisphaerae (class);o__Victivallales
k__Bacteria;p__MVP-15;c__;o__
k__Bacteria;p__NC10;c__;o__
k__Bacteria;p__NKB19;c__;o__
k__Bacteria;p__Nitrospirae;c__Nitrospira (class);o__Nitrospirales
k__Bacteria;p__OP10;c__5B-18;o__
k__Bacteria;p__OP10;c__;o__
k__Bacteria;p__OP10;c__CH21;o__
k__Bacteria;p__OP10;c__CL500-48;o__
k__Bacteria;p__OP10;c__OS-L;o__
k__Bacteria;p__OP10;c__S1a-1H;o__
k__Bacteria;p__OP10;c__SJA-176;o__
k__Bacteria;p__OP10;c__SJA-22;o__
k__Bacteria;p__OP11;c__;o__
k__Bacteria;p__OP3;c__;o__
k__Bacteria;p__OP8;c__OP8;o__
k__Bacteria;p__Planctomycetes;c__FFCH393;o__
k__Bacteria;p__Planctomycetes;c__Kueneniae;o__Kueneniales
k__Bacteria;p__Planctomycetes;c__PW285;o__
k__Bacteria;p__Planctomycetes;c__Phycisphaerae;o__
k__Bacteria;p__Planctomycetes;c__Phycisphaerae;o__Phycisphaerales
k__Bacteria;p__Planctomycetes;c__Planctomycea;o__Gemmatales
k__Bacteria;p__Planctomycetes;c__Planctomycea;o__Pirellulales
k__Bacteria;p__Planctomycetes;c__Planctomycea;o__Planctomycetales
k__Bacteria;p__Planctomycetes;c__agg27;o__CL500-15
k__Bacteria;p__Planctomycetes;c__agg27;o__OM190
k__Bacteria;p__Planctomycetes;c__vadinHA49;o__
k__Bacteria;p__Proteobacteria;c__Alphaproteobacteria;o__
k__Bacteria;p__Proteobacteria;c__Alphaproteobacteria;o__Caulobacterales
k__Bacteria;p__Proteobacteria;c__Alphaproteobacteria;o__Rhizobiales
k__Bacteria;p__Proteobacteria;c__Alphaproteobacteria;o__Rhodobacterales
k__Bacteria;p__Proteobacteria;c__Alphaproteobacteria;o__Rhodospirillales
k__Bacteria;p__Proteobacteria;c__Alphaproteobacteria;o__Rickettsiales
k__Bacteria;p__Proteobacteria;c__Alphaproteobacteria;o__Sphingomonadales
k__Bacteria;p__Proteobacteria;c__Betaproteobacteria;o__
k__Bacteria;p__Proteobacteria;c__Betaproteobacteria;o__Burkholderiales
k__Bacteria;p__Proteobacteria;c__Betaproteobacteria;o__Gallionellales
k__Bacteria;p__Proteobacteria;c__Betaproteobacteria;o__Hydrogenophilales
k__Bacteria;p__Proteobacteria;c__Betaproteobacteria;o__Methylophilales
k__Bacteria;p__Proteobacteria;c__Betaproteobacteria;o__Neisseriales
k__Bacteria;p__Proteobacteria;c__Betaproteobacteria;o__Nitrosomonadales
k__Bacteria;p__Proteobacteria;c__Betaproteobacteria;o__Procabacteriales
k__Bacteria;p__Proteobacteria;c__Betaproteobacteria;o__Rhodocyclales
k__Bacteria;p__Proteobacteria;c__Deltaproteobacteria;o__
k__Bacteria;p__Proteobacteria;c__Deltaproteobacteria;o__Bdellovibrionales
k__Bacteria;p__Proteobacteria;c__Deltaproteobacteria;o__CTD005-82B-02
k__Bacteria;p__Proteobacteria;c__Deltaproteobacteria;o__Desulfobacterales
k__Bacteria;p__Proteobacteria;c__Deltaproteobacteria;o__Desulfovibrionales
k__Bacteria;p__Proteobacteria;c__Deltaproteobacteria;o__Desulfuromonadales
k__Bacteria;p__Proteobacteria;c__Deltaproteobacteria;o__Entotheonellales
k__Bacteria;p__Proteobacteria;c__Deltaproteobacteria;o__MIZ46
k__Bacteria;p__Proteobacteria;c__Deltaproteobacteria;o__Myxococcales
k__Bacteria;p__Proteobacteria;c__Deltaproteobacteria;o__NB1-j
k__Bacteria;p__Proteobacteria;c__Deltaproteobacteria;o__NKB15
k__Bacteria;p__Proteobacteria;c__Deltaproteobacteria;o__Syntrophobacterales
k__Bacteria;p__Proteobacteria;c__Epsilonproteobacteria;o__
k__Bacteria;p__Proteobacteria;c__Epsilonproteobacteria;o__Campylobacterales
k__Bacteria;p__Proteobacteria;c__Gammaproteobacteria;o__
k__Bacteria;p__Proteobacteria;c__Gammaproteobacteria;o__Aeromonadales
k__Bacteria;p__Proteobacteria;c__Gammaproteobacteria;o__Alteromonadales
k__Bacteria;p__Proteobacteria;c__Gammaproteobacteria;o__Cardiobacteriales
k__Bacteria;p__Proteobacteria;c__Gammaproteobacteria;o__Chromatiales
k__Bacteria;p__Proteobacteria;c__Gammaproteobacteria;o__Enterobacteriales
k__Bacteria;p__Proteobacteria;c__Gammaproteobacteria;o__Legionellales
k__Bacteria;p__Proteobacteria;c__Gammaproteobacteria;o__Methylococcales
k__Bacteria;p__Proteobacteria;c__Gammaproteobacteria;o__Oceanospirillales
k__Bacteria;p__Proteobacteria;c__Gammaproteobacteria;o__Pasteurellales
k__Bacteria;p__Proteobacteria;c__Gammaproteobacteria;o__Pseudomonadales
k__Bacteria;p__Proteobacteria;c__Gammaproteobacteria;o__Salinisphaerales
k__Bacteria;p__Proteobacteria;c__Gammaproteobacteria;o__Thiotrichales
k__Bacteria;p__Proteobacteria;c__Gammaproteobacteria;o__Vibrionales
k__Bacteria;p__Proteobacteria;c__Gammaproteobacteria;o__Xanthomonadales
k__Bacteria;p__SC3;c__;o__
k__Bacteria;p__SC4;c__;o__
k__Bacteria;p__SPAM;c__;o__
k__Bacteria;p__SR1;c__;o__
k__Bacteria;p__Spirochaetes;c__Brachyspirae;o__Brachyspirales
k__Bacteria;p__Spirochaetes;c__Leptospirae;o__Leptospirales
k__Bacteria;p__Spirochaetes;c__SP_WWE1;o__
k__Bacteria;p__Spirochaetes;c__Spirochaetes (class);o__Spirochaetales
k__Bacteria;p__Synergistetes;c__Synergistia;o__Synergistales
k__Bacteria;p__TM6;c__;o__
k__Bacteria;p__TM7;c__;o__
k__Bacteria;p__TM7;c__TM7-1;o__
k__Bacteria;p__TM7;c__TM7-3;o__CW040
k__Bacteria;p__TM7;c__TM7-3;o__EW055
k__Bacteria;p__TM7;c__TM7-3;o__I025
k__Bacteria;p__Tenericutes;c__;o__
k__Bacteria;p__Tenericutes;c__Erysipelotrichi;o__Erysipelotrichales
k__Bacteria;p__Tenericutes;c__ML615J-28;o__
k__Bacteria;p__Tenericutes;c__Mollicutes;o__Acholeplasmatales
k__Bacteria;p__Tenericutes;c__Mollicutes;o__Anaeroplasmatales
k__Bacteria;p__Tenericutes;c__Mollicutes;o__Entomoplasmatales
k__Bacteria;p__Tenericutes;c__Mollicutes;o__Mycoplasmatales
k__Bacteria;p__Tenericutes;c__Mollicutes;o__RF39
k__Bacteria;p__Thermi;c__Deinococci;o__Deinococcales
k__Bacteria;p__Thermi;c__Deinococci;o__Thermales
k__Bacteria;p__Thermotogae;c__Thermotogae (class);o__Thermotogales
k__Bacteria;p__Verrucomicrobia;c__;o__
k__Bacteria;p__Verrucomicrobia;c__;o__Methylacidiphilales
k__Bacteria;p__Verrucomicrobia;c__Opitutae;o__
k__Bacteria;p__Verrucomicrobia;c__Opitutae;o__Puniceicoccales
k__Bacteria;p__Verrucomicrobia;c__Spartobacteria;o__
k__Bacteria;p__Verrucomicrobia;c__TP21;o__
k__Bacteria;p__Verrucomicrobia;c__Verrucomicrobiae;o__Verrucomicrobiales
k__Bacteria;p__WPS-2;c__;o__
k__Bacteria;p__WS3;c__PRR-12;o__
k__Bacteria;p__WS3;c__PRR-12;o__LCP-67
k__Bacteria;p__WS3;c__PRR-12;o__PRR-10
k__Bacteria;p__ZB2;c__;o__
