# Supplementary material for: Moving pictures of the human microbiome
Source: Genome Biol. 2011 May 30;12(5):R50. doi: 10.1186/gb-2011-12-5-r50 (PMC3271711; doi:10.1186/gb-2011-12-5-r50)
Supplement: Additional file 13 — Temporal variation in phylum, class, order, family, and genus abundances (F4 left palm). The x-axis scale differs between M3 and F4 plots. [file gb-2011-12-5-r50-S13.ZIP › AdditionalFile13/index.html]

 
 
 
 

 
 Taxa Summaries 
 
 
  
 
     
  Taxonomy Summary. Current Level: Phylum  
     View Figure (.pdf)    View Legend (.pdf)   
   
 
     
 
 

 
 
 
 
 
 
 
 
 
 
 
 
 
 
 
 
 
 
 
 
 
 
 
 
 
 
 
 
 
 
 
 
 
 
 
 
 
 
 
 
 
 
 
 
 
 
 
 
 
 
 
 
 
 
 
 
 
 
 
 
 
 
 
 
 
 
 
 
 
 
 
 
 
 
 
 
 
 
 
 
 
 
 
 
 
 
 
 
 
 
 
 
 
 
 
 
 
 
 
 
 
 
 
 
 
 
 
 
 
 
 
 
 
 
 
 
 
 
 
 
 
 
 
 
 
 
 
 
 
 
 
 
 
 
 
 
 
 
 
 
 
 
 
 
 
 
 
 
 
 
 
 
 
 
 
 
 
 
 
 
 
 
 
 
 
 
 
 
 
 
 
 
 
 
 
 
 
 
 
 
 
 
 
 
 
 
 
 
 
 
 
 
 
 
 
 
 
 
 
 
 
 
 
 
 
 
 
 
 
 
 
 
 
 
 
 
 
 
 
 
 
 
 
 
 
 
 
 
 
 
 
 
 
 
 
 
 
 
 
 
 
 
 
 
 
 
 
 
 
 
 
 
 
 
 
 
 
 
 
 
 
 
 
 
 
 
 
 
 
 
 
 
 
 
 
 
 
 
 
 
 
 
 
 
 
 
 
 
 
 
 
 
 
 
 
 
 
 
 
 
 
 
 
 
 
 
 
 
 
 
 
 
 
 
 
 
 
 
 
 
 
 
 
 
 
 
 
 
 
 
 
 
 
 
 
 
 
 
 
 
 
 
 
 
 
 
 
 
 
 
 
 
 
 
 
 
 
 
 
 
 
 
 
 
 
 
 
 
 
 
 
 
 
 
 
 
 
 
 
 
 
 
 
 
 
 
 
 
 
 
 
 
 
 
 
 
 
 
 
 
 
 
 
 
 
 
 
 
 
 
 
 
 
 
 
 
 
 
 
 
 
 
 
 
 
 
 
 
 
 
 
 
 
 
 
 
 
 
 
 
 
 
 
 
 
 
 
 
 
 
 
 
 
 
 
 
 
 
 
 
 
 
 
 
 
 
 
 
 
 
 
 
 
 
 
 
 
 
 
 
 
 
 
 
 
 
 
 
 
 
 
 
 
 
 
 
 
 
 
 
 
 
 
 
 
 
 
 
 
 
 
 
 
 
 
 
 
 
 
 
 
 
 
 
 
 
 
 
 
 
 
 
 
 
 
 
 
 
 
 
 
 
 
 
 
 
 
 
 
 
 
 
 
 
 
 
 
 
 
 
 
 
 
 
 
 
 
 
 
 
 
 
 
 
 
 
 
 
 
 
 
 
 
 
 
 
 
 
 
 
 
 
 
 
 
 
 
 
 
 
 
 
 
 
 
 
 
 
 
 
 
 
 
 
 
 
 
 
 
 
 
 
 
 
 
 
 
 
 
 
 
 
 
 
 
 
 
 
 
 
 
 
 
 
 
 
 
 
 
 
 
 
 
 
 
 
 
 
 
 
 
 
 
 
 
 
 
 
 
 
 
 
 
 
 
 
 
 
 
 
 
 
 
 
 
 
 
 
 
 
 
 
 
 
 
 
 
 
 
 
 
 
 
 
 
 
 
 
 
 
 
 
 
 
 
 
 
 
 
 
 
 
 
 
 
 
 
 
 
 
 
 
 
 
 
 
 
 
 
 
 
 
 
 
 
 
 
 
 
 
 
 
 
 
 
 
 
 
 
 
 
 
 
 
 
 
 
 
 
 
 
 
 
 
 
 
 
 
 
 
 
 
 
 
 
 
 
 
 
 
 
 
 
 
 
 
 
 
 
 
 
 
 
 
 
 
 
 
 
 
 
 
 
 
 
 
 
 
 
 
 
 
 
 
 
 
 
 
 
 
 
 
 
 
 
 
 
 
 
 
 
 
 
 
 
 
 
 
 
 
 
 
 
 
 
 
 
 
 
 
 
 
 
 
 
 
 
 
 
 
 
 
 
 
 
 
 
 
 
 
 
 
 
 
 
 
 
 
 
 
 
 
 
 
 
 
 
 
 
 
 
 
 
 
 
 
 
 
 
 
 
 
 
 
 
 
 
 
 
 
 
 
 
 
 
 
 
 
 
 
 
 
 
 
 
 
 
 
 
 
 
 
 
 
 
 
 
 
 
 
 
 
 
 
 
 
 
 
 
 
 
 
 
 
 
 
 
 
 
 
 
 
 
 
 
 
 
 
 
 
 
 
 
 
 
 
 
 
 
 
 
 
 
 
 
 
 
 
 
 
 
 
 
 
 
 
 
 
 
 
 
 
 
 
 
 
 
 
 
 
 
 
 
 
 
 
 
 
 
 
 
 
 
 
 
 
 
 
 
 
 
 
 
 
 
 
 
 
 
 
 
 
 
 
 
 
 
 
 
 
 
 
 
 
 
 
 
 
 
 
 
 
 
 
 
 
 
 
 
 
 
 
 
 
 
 
 
 
 
 
 
 
 
 
 
 
 
 
 
 
 
 
 
 
 
 
 
 
 
 
 
 
 
 
 
 
 
 
 
 
 
 
 
 
 
 
 
 
 
 
 
 
 
 
 
 
 
 
 
 
 
 
 
 
 
 
 
 
 
 
 
 
 
 
 
 
 
 
 
 
 
 
 
 
 
 
 
 
 
 
 
 
 
 
 
 
 
 
 
 
 
 
 
 
 
 
 
 
 
 
 
 
 
 
 
 
 
 
 
 
 
 
 
 
 
 
 
 
 
 
 
 
 
 
 
 
 
 
 
 
 
 
 
 
 
 
 
 
 
 
 
 
 
 
 
 
 
 
 
 
 
 
 
 
 
 
 
 
 
 
 
 
 
 
 
 
 
 
 
 
 
 
 
 
 
 
 
 
 
 
 
 
 
 
 
 
 
 
 
 
 
 
 
 
 
 
 
 
 
 
 
 
 
 
 
 
 
 
 
 
 
 
 
 
 
 
 
 
 
 
 
 
 
 
 
 
 
 
 
 
 
 
 
 
 
 
 
 
 
 
 
 
 
 
 
 
 
 
 
 
 
 
 
 
 
 
 
 
 
 
 
 
 
 
 
 
 
 
 
 
 
 
 
 
 
 
 
 
 
 
 
 
 
 
 
 
 
 
 
 
 
 
 
 
 
 
 
 
 
 
 
 
 
 
 
 
 
 
 
 
 
 
 
 
 
 
 
 
 
 
 
 
 
 
 
 
 
 
 
 
 
 
 
 
 
 
 
 
 
 
 
 
 
 
 
 
 
 
 
 
 
 
 
 
 
 
 
 
 
 
 
 
 
 
 
 
 
 
 
 
 
 
 
 
 
 
 
 
 
 
 
 
 
 
 
 
 
 
 
 
 
 
 
 
 
 
 
 
 
 
 
 
 
 
 
 
 
 
 
 
 
 
 
 
 
 
 
 
 
 
 
 
 
 
 
 
 
 
 
 
 
 
 
 
 
 
 
 
 
 
 
 
 
 
 
 
 
 
 
 
 
 
 
 
 
 
 
 
 
 
 
 
 
 
 
 
 
 
 
 
 
 
 
 
 
 
 
 
 
 
 
 
 
 
 
 
 
 
 
 
 
 
 
 
 
 
 
 
 
 
 
 
 
 
 
 
 
 
 
 
 
 
 
 
 
 
 
 
 
 
 
 
 
 
 
 
 
 
 
 
 
 
 
 
 
 
 
 
 
 
 
 
 
 
 
 
 
 
 
 
 
 
 
 
 
 
 
 
 
 
 
 
 
 
 
 
 
 
 
 
 
 
 
 
 
 
 
 
 
 
 
 
 
 
 
 
 
 
 
 
 
 
 
 
 
 
 
 
 
 
 
 
 
 
 
 
 
 
 
 
 
 
 
 
 
 
 
 
 
 
 
 
 
 
 
 
 
 
 
 
 
 
 
 
 
 
 
 
 
 
 
 
 
 
 
 
 
 
 
 
 
 
 
 
 
 
 
 
 
 
 
 
 
 
 
 
 
 
 
 
 
 
 
 
 
 
 
 
 
 
 
 
 
 
 
 
 
 
 
 
 
 
 
 
 
 
 
 
 
 
 
 
 
 
 
 
 
 
 
 
 
 
 
 
 
 
 
 
 
 
 
 
 
 
 
 
 
 
 
 
 
 
 
 
 
 
 
 
 
 
 
 
 
 
 
 
 
 
 
 
 
 
 
 
 
 
 
 
 
 
 
 
 
 
 
 
 
 
 
 
 
 
 
 
 
 
 
 
 
 
 
 
 
 
 
 
 
 
 
 
 
 
 
 
 
 
 
 
 
 
 
 
 
 
 
 
 
 
 
 
 
 
 
 
 
 
 
 
 
 
 
 
 
 
 
 
 
 
 
 
 
 
 
 
 
 
 
 
 
 
 
 
 
 
 
 
 
 
 
 
 
 
 
 
 
 
 
 
 
 
 
 
 
 
 
 
 
 
 
 
 
 
 
 
 
 
 
 
 
 
 
 
 
 
 
 
 
 
 
 
 
 
 
 
 
 
 
 
 
 
 
 
 
 
 
 
 
 
 
 
 
 
 
 
 
 
 
 
 
 
 
 
 
 
 
 
 
 
 
 
 
 
 
 
 
 
 
 
 
 
 
 
 
 
 
 
 
 
 
 
 
 
 
 
 
 
 
 
 
 
 
 
 
 
 
 
 
 
 
 
 
 
 
 
 
 
 
 
 
 
 
 
 
 
 
 
 
 
 
 
 
 
 
 
 
 
 
 
 
 
 
 
 
 
 
 
 
 
 
 
 
 
 
 
 
 
 
 
 
 
 
 
 
 
 
 
 
 
 
 
 
 
 
 
 
 
 
 
 
 
 
 
 
 
 
 
 
 
 
 
 
 
 
 
 
 
 
 
 
 
 
 
 
 
 
 
 
 
 
 
 
 
 
 
 
 
 
 
 
 
 
 
 
 
 
 
 
 
 
 
 
 
 
 
 
 
 
 
 
 
 
 
 
 
 
 
 
 
 
 
 
 
 
 
 
 
 
 
 
 
 
 
 
 
 
 
 
 
 
 
 
 
 
 
 
 
 
 
 
 
 
 
 
 
 
 
 
 
 
 
 
 
 
 
 
 
 
 
 
 
 
 
 
 
 
 
 
 
 
 
 
 
 
 
 
 
 
 
 
 
 
 
 
 
 
 
 
 
 
 
 
 
 
 
 
 
 
 
 
 
 
 
 
 
 
 
 
 
 
 
 
 
 
 
 
 
 
 
 
 
 
 
 
 
 
 
 
 
 
 
 
 
 
 
 
 
 
 
 
 
 
 
 
 
 
 
 
 
 
 
 
 
 
 
 
 
 
 
 
 
 
 
 
 
 
 
 
 
 
 
 
 
 
 
 
 
 
 
 
 
 
 
 
 
 
 
 
 
 
 
 
 
 
 
 
 
 
 
 
 
 
 
 
 
 
 
 
 
 
 
 
 
 
 
 
 
 
 
 
 
 
 
 
 
 
 
 
 
 
 
 
 
 
 
 
 
 
 
 
 
 
 
 
 
 
 
 
 
 
 
 
 
 
 
 
 
 
 
 
 
 
 
 
 
 
 
 
 
 
 
 
 
 
 
 
 
 
 
 
 
 
 
 
 
 
 
 
 
 
 
 
 
 
 
 
 
 
 
 
 
 
 
 
 
 
 
 
 
 
 
 
 
 
 
 
 
 
 
 
 
 
 
 
 
 
 
 
 
 
 
 
 
 
 
 
 
 
 
 
 
 
 
 
 
 
 
 
 
 
 
 
 
 
 
 
 
 
 
 
 
 
 
 
 
 
 
 
 
 
 
 
 
 
 
 
 
 
 
 
 
 
 
 
 
 
 
 
 
 
 
 
 
 
 
 
 
 
 
 
 
 
 
 
 
 
 
 
 
 
 
 
 
 
 
 
 
 
 
 
 
 
 
 
 
 
 
 
 
 
 
 
 
 
 
 
 
 
 
 
 
 
 
 
 
 
 
 
 
 
 
 
 
 
 
 
 
 
 
 
 
 
 
 
 
 
 
 
 
 
 
 
 
 
 
 
 
 
 
 
 
 
 
 
 
 
 
 
 
 
 
 
 
 
 
 
 
 
 
 
 
 
 
 
 
 
 
 
 
 
 
 
 
 
 
 
 
 
 
 
 
 
 
 
 
 
 
 
 
 
 
 
 
 
 
 
 
 
 
 
 
 
 
 
 
 
 
 
 
 
 
 
 
 
 
 
 
 
 
 
 
 
 
 
 
 
 
 
 
 
 
 
 
 
 
 
 
 
 
 
 
 
 
 
 
 
 
 
 
 
 
 
 
 
 
 
 
 
 
 
 
 
 
 
 
 
 
 
 
 
 
 
 
 
 
 
 
 
 
 
 
 
 
 
 
 
 
 
 
 
 
 
 
 
 
 
 
 
 
 
 
 
 
 
 
 
 
 
 
 
 
 
 
 
 
 

 

    View Table (.txt)         Total  0  1  2  3  4  5  6  8  9  10  11  12  13  14  15  16  17  18  19  20  21  22  23  24  25  26  27  28  29  30  31  42  43  44  45  46  47  48  49  50  51  53  54  55  56  57  58  59  60  61  62  71  72  81  82  83  84  91  92  93  95  96  97  98  99  100  101  102  103  104  112  113  114  115  116  117  118  119  120  122  123  124  125  126  127  128  129  130  131  132  133  134  135  136  137  138  139  140  141  142  143  144  145  146  147  148  149  151  152  153  155  157  158  159  160  161  166  170  171  172  173  174  175  176  177  178  179  180  181  182  183  184  185    Legend  Taxonomy  count  %  %  %  %  %  %  %  %  %  %  %  %  %  %  %  %  %  %  %  %  %  %  %  %  %  %  %  %  %  %  %  %  %  %  %  %  %  %  %  %  %  %  %  %  %  %  %  %  %  %  %  %  %  %  %  %  %  %  %  %  %  %  %  %  %  %  %  %  %  %  %  %  %  %  %  %  %  %  %  %  %  %  %  %  %  %  %  %  %  %  %  %  %  %  %  %  %  %  %  %  %  %  %  %  %  %  %  %  %  %  %  %  %  %  %  %  %  %  %  %  %  %  %  %  %  %  %  %  %  %  %  %  %  %        k__Archaea; p__Crenarchaeota    3113    0.1%    0.0%    0.1%    0.0%    0.0%    0.1%    0.0%    0.1%    0.0%    0.0%    0.1%    0.0%    0.0%    0.0%    0.0%    0.0%    0.1%    0.0%    0.1%    0.0%    0.0%    0.0%    0.0%    0.0%    0.0%    0.0%    0.1%    0.0%    0.0%    0.0%    0.0%    0.0%    0.0%    0.0%    0.0%    0.0%    0.0%    0.0%    0.1%    0.1%    0.0%    0.0%    0.0%    0.0%    0.0%    0.0%    0.0%    0.0%    0.0%    0.0%    0.0%    0.0%    0.0%    0.1%    0.0%    0.1%    0.1%    0.0%    0.0%    0.1%    0.1%    0.1%    0.1%    0.0%    0.1%    0.0%    0.1%    0.0%    0.5%    0.2%    0.1%    0.1%    0.0%    0.1%    0.1%    0.1%    0.2%    0.0%    0.0%    0.3%    0.1%    0.0%    0.3%    0.0%    0.1%    0.1%    0.1%    0.1%    0.1%    0.1%    0.1%    0.1%    0.1%    0.0%    0.1%    0.1%    0.1%    0.1%    0.2%    0.0%    0.1%    0.1%    0.1%    0.1%    0.1%    0.1%    0.1%    0.0%    0.3%    0.1%    0.3%    0.2%    0.1%    0.1%    0.0%    0.1%    0.1%    0.4%    0.2%    0.1%    0.1%    0.1%    0.0%    0.1%    0.1%    0.3%    0.0%    0.0%    0.1%    0.1%    0.1%    0.1%    0.1%    0.1%        k__Archaea; p__Euryarchaeota      40    0.0%    0.0%    0.0%    0.0%    0.0%    0.0%    0.0%    0.0%    0.0%    0.0%    0.0%    0.0%    0.0%    0.0%    0.0%    0.0%    0.0%    0.0%    0.0%    0.0%    0.0%    0.0%    0.0%    0.0%    0.0%    0.0%    0.0%    0.0%    0.0%    0.0%    0.0%    0.0%    0.0%    0.0%    0.0%    0.0%    0.0%    0.0%    0.0%    0.0%    0.0%    0.0%    0.0%    0.0%    0.0%    0.0%    0.0%    0.0%    0.0%    0.0%    0.0%    0.0%    0.0%    0.0%    0.0%    0.0%    0.0%    0.0%    0.0%    0.0%    0.0%    0.0%    0.0%    0.0%    0.0%    0.0%    0.0%    0.0%    0.0%    0.0%    0.0%    0.0%    0.0%    0.0%    0.0%    0.0%    0.0%    0.0%    0.0%    0.0%    0.0%    0.0%    0.0%    0.0%    0.0%    0.0%    0.0%    0.0%    0.0%    0.0%    0.0%    0.0%    0.0%    0.0%    0.0%    0.0%    0.0%    0.0%    0.0%    0.0%    0.0%    0.0%    0.0%    0.0%    0.0%    0.0%    0.0%    0.0%    0.0%    0.0%    0.0%    0.0%    0.0%    0.0%    0.0%    0.0%    0.0%    0.0%    0.0%    0.0%    0.0%    0.0%    0.0%    0.0%    0.0%    0.0%    0.0%    0.0%    0.0%    0.0%    0.0%    0.0%    0.0%    0.0%        k__Bacteria; p__       3    0.0%    0.0%    0.0%    0.0%    0.0%    0.0%    0.0%    0.0%    0.0%    0.0%    0.0%    0.0%    0.0%    0.0%    0.0%    0.0%    0.0%    0.0%    0.0%    0.0%    0.0%    0.0%    0.0%    0.0%    0.0%    0.0%    0.0%    0.0%    0.0%    0.0%    0.0%    0.0%    0.0%    0.0%    0.0%    0.0%    0.0%    0.0%    0.0%    0.0%    0.0%    0.0%    0.0%    0.0%    0.0%    0.0%    0.0%    0.0%    0.0%    0.0%    0.0%    0.0%    0.0%    0.0%    0.0%    0.0%    0.0%    0.0%    0.0%    0.0%    0.0%    0.0%    0.0%    0.0%    0.0%    0.0%    0.0%    0.0%    0.0%    0.0%    0.0%    0.0%    0.0%    0.0%    0.0%    0.0%    0.0%    0.0%    0.0%    0.0%    0.0%    0.0%    0.0%    0.0%    0.0%    0.0%    0.0%    0.0%    0.0%    0.0%    0.0%    0.0%    0.0%    0.0%    0.0%    0.0%    0.0%    0.0%    0.0%    0.0%    0.0%    0.0%    0.0%    0.0%    0.0%    0.0%    0.0%    0.0%    0.0%    0.0%    0.0%    0.0%    0.0%    0.0%    0.0%    0.0%    0.0%    0.0%    0.0%    0.0%    0.0%    0.0%    0.0%    0.0%    0.0%    0.0%    0.0%    0.0%    0.0%    0.0%    0.0%    0.0%    0.0%    0.0%        k__Bacteria; p__ABY1_OD1       1    0.0%    0.0%    0.0%    0.0%    0.0%    0.0%    0.0%    0.0%    0.0%    0.0%    0.0%    0.0%    0.0%    0.0%    0.0%    0.0%    0.0%    0.0%    0.0%    0.0%    0.0%    0.0%    0.0%    0.0%    0.0%    0.0%    0.0%    0.0%    0.0%    0.0%    0.0%    0.0%    0.0%    0.0%    0.0%    0.0%    0.0%    0.0%    0.0%    0.0%    0.0%    0.0%    0.0%    0.0%    0.0%    0.0%    0.0%    0.0%    0.0%    0.0%    0.0%    0.0%    0.0%    0.0%    0.0%    0.0%    0.0%    0.0%    0.0%    0.0%    0.0%    0.0%    0.0%    0.0%    0.0%    0.0%    0.0%    0.0%    0.0%    0.0%    0.0%    0.0%    0.0%    0.0%    0.0%    0.0%    0.0%    0.0%    0.0%    0.0%    0.0%    0.0%    0.0%    0.0%    0.0%    0.0%    0.0%    0.0%    0.0%    0.0%    0.0%    0.0%    0.0%    0.0%    0.0%    0.0%    0.0%    0.0%    0.0%    0.0%    0.0%    0.0%    0.0%    0.0%    0.0%    0.0%    0.0%    0.0%    0.0%    0.0%    0.0%    0.0%    0.0%    0.0%    0.0%    0.0%    0.0%    0.0%    0.0%    0.0%    0.0%    0.0%    0.0%    0.0%    0.0%    0.0%    0.0%    0.0%    0.0%    0.0%    0.0%    0.0%    0.0%    0.0%        k__Bacteria; p__AD3       0    0.0%    0.0%    0.0%    0.0%    0.0%    0.0%    0.0%    0.0%    0.0%    0.0%    0.0%    0.0%    0.0%    0.0%    0.0%    0.0%    0.0%    0.0%    0.0%    0.0%    0.0%    0.0%    0.0%    0.0%    0.0%    0.0%    0.0%    0.0%    0.0%    0.0%    0.0%    0.0%    0.0%    0.0%    0.0%    0.0%    0.0%    0.0%    0.0%    0.0%    0.0%    0.0%    0.0%    0.0%    0.0%    0.0%    0.0%    0.0%    0.0%    0.0%    0.0%    0.0%    0.0%    0.0%    0.0%    0.0%    0.0%    0.0%    0.0%    0.0%    0.0%    0.0%    0.0%    0.0%    0.0%    0.0%    0.0%    0.0%    0.0%    0.0%    0.0%    0.0%    0.0%    0.0%    0.0%    0.0%    0.0%    0.0%    0.0%    0.0%    0.0%    0.0%    0.0%    0.0%    0.0%    0.0%    0.0%    0.0%    0.0%    0.0%    0.0%    0.0%    0.0%    0.0%    0.0%    0.0%    0.0%    0.0%    0.0%    0.0%    0.0%    0.0%    0.0%    0.0%    0.0%    0.0%    0.0%    0.0%    0.0%    0.0%    0.0%    0.0%    0.0%    0.0%    0.0%    0.0%    0.0%    0.0%    0.0%    0.0%    0.0%    0.0%    0.0%    0.0%    0.0%    0.0%    0.0%    0.0%    0.0%    0.0%    0.0%    0.0%    0.0%    0.0%        k__Bacteria; p__Acidobacteria   17278    0.4%    0.2%    0.4%    0.3%    0.1%    1.1%    0.5%    0.8%    0.2%    0.1%    0.5%    0.3%    0.1%    0.2%    0.1%    0.1%    0.4%    0.0%    0.4%    0.1%    0.3%    0.2%    0.1%    0.1%    0.2%    0.3%    0.5%    0.3%    0.3%    0.1%    0.1%    0.2%    0.2%    0.2%    0.2%    0.1%    0.1%    0.3%    0.2%    0.3%    0.2%    0.1%    0.5%    0.5%    0.2%    0.2%    0.5%    0.2%    0.1%    0.2%    0.1%    0.1%    0.1%    0.6%    0.3%    0.5%    0.6%    0.2%    0.2%    0.7%    0.7%    1.1%    0.3%    0.1%    0.2%    0.1%    0.5%    0.4%    2.7%    0.6%    0.3%    0.7%    0.1%    0.7%    0.5%    0.6%    0.8%    0.1%    0.1%    1.5%    0.4%    0.2%    1.1%    0.1%    0.4%    0.3%    0.4%    0.7%    0.6%    0.4%    0.5%    0.4%    0.3%    0.0%    0.3%    0.4%    0.5%    0.3%    0.9%    0.3%    0.4%    0.4%    0.6%    0.5%    0.3%    0.2%    0.1%    0.2%    1.5%    0.8%    1.0%    1.2%    0.6%    0.4%    0.1%    0.4%    0.3%    1.4%    0.6%    0.2%    0.3%    0.8%    0.2%    0.3%    0.2%    1.2%    0.4%    0.5%    0.5%    0.4%    0.5%    0.3%    0.1%    0.3%        k__Bacteria; p__Actinobacteria   870896   20.2%   22.1%   26.1%   13.3%   27.0%   16.7%   22.9%   20.4%   12.9%   10.2%   16.4%   29.4%   27.5%   21.8%   16.0%   17.1%   18.0%   43.1%   22.1%   36.1%   17.1%   34.1%   15.3%   28.3%   29.6%   14.3%   14.3%   15.5%   23.6%   41.5%   17.8%   33.5%   22.5%   20.9%   11.4%    8.8%   25.3%   14.2%   25.4%   14.0%   12.0%   14.1%   10.8%   14.8%   11.0%   16.7%   19.4%    9.8%   14.2%   15.9%   16.7%   18.4%   19.5%   21.1%   14.9%   20.0%   17.0%   14.1%   36.3%   24.1%   28.0%   20.5%   22.3%   15.3%   15.4%   22.8%   12.4%   37.6%   25.9%   20.5%   17.1%   27.5%   23.8%   22.1%   21.7%   17.6%   21.7%   23.7%   33.8%   30.5%   17.4%   12.1%   19.5%   25.3%   19.0%   16.7%   17.1%   16.0%   18.1%   16.9%   21.6%   19.2%   16.4%   38.6%   10.6%   13.8%   23.9%   19.5%   14.6%   13.7%   13.7%   13.4%   16.9%   33.4%   19.0%    8.4%   14.6%   21.4%   26.1%   25.3%   22.9%   21.4%   21.9%   20.3%    8.9%   27.9%   29.0%   21.3%   21.3%   13.0%   26.5%   20.6%   17.5%   16.0%   12.8%   19.2%   17.5%   24.0%   22.7%   11.7%   17.2%   17.3%   17.1%   15.5%        k__Bacteria; p__Aquificae      18    0.0%    0.0%    0.0%    0.0%    0.0%    0.0%    0.0%    0.0%    0.0%    0.0%    0.0%    0.0%    0.0%    0.0%    0.0%    0.0%    0.0%    0.0%    0.0%    0.0%    0.0%    0.0%    0.0%    0.0%    0.0%    0.0%    0.0%    0.0%    0.0%    0.0%    0.0%    0.0%    0.0%    0.0%    0.0%    0.0%    0.0%    0.0%    0.0%    0.0%    0.0%    0.0%    0.0%    0.0%    0.0%    0.0%    0.0%    0.0%    0.0%    0.0%    0.0%    0.0%    0.0%    0.0%    0.0%    0.0%    0.0%    0.0%    0.0%    0.0%    0.0%    0.0%    0.0%    0.0%    0.0%    0.0%    0.0%    0.0%    0.0%    0.0%    0.0%    0.0%    0.0%    0.0%    0.0%    0.0%    0.0%    0.0%    0.0%    0.0%    0.0%    0.0%    0.0%    0.0%    0.0%    0.0%    0.0%    0.0%    0.0%    0.0%    0.0%    0.0%    0.0%    0.0%    0.0%    0.0%    0.0%    0.0%    0.0%    0.0%    0.0%    0.0%    0.0%    0.0%    0.0%    0.0%    0.0%    0.0%    0.0%    0.0%    0.0%    0.0%    0.0%    0.0%    0.0%    0.0%    0.0%    0.0%    0.0%    0.0%    0.0%    0.0%    0.0%    0.0%    0.0%    0.0%    0.0%    0.0%    0.0%    0.0%    0.0%    0.0%    0.0%    0.0%        k__Bacteria; p__BRC1     136    0.0%    0.0%    0.0%    0.0%    0.0%    0.0%    0.0%    0.1%    0.0%    0.0%    0.0%    0.0%    0.0%    0.0%    0.0%    0.0%    0.0%    0.0%    0.0%    0.0%    0.0%    0.0%    0.0%    0.0%    0.0%    0.0%    0.0%    0.0%    0.0%    0.0%    0.0%    0.0%    0.0%    0.0%    0.0%    0.0%    0.0%    0.0%    0.0%    0.0%    0.0%    0.0%    0.0%    0.0%    0.0%    0.0%    0.0%    0.0%    0.0%    0.0%    0.0%    0.0%    0.0%    0.0%    0.0%    0.0%    0.0%    0.0%    0.0%    0.0%    0.0%    0.0%    0.0%    0.0%    0.0%    0.0%    0.0%    0.0%    0.0%    0.0%    0.0%    0.0%    0.0%    0.0%    0.0%    0.0%    0.0%    0.0%    0.0%    0.0%    0.0%    0.0%    0.0%    0.0%    0.0%    0.0%    0.0%    0.0%    0.0%    0.0%    0.0%    0.0%    0.0%    0.0%    0.0%    0.0%    0.0%    0.0%    0.0%    0.0%    0.0%    0.0%    0.0%    0.0%    0.0%    0.0%    0.0%    0.0%    0.0%    0.0%    0.0%    0.0%    0.0%    0.0%    0.0%    0.0%    0.0%    0.0%    0.0%    0.0%    0.0%    0.0%    0.0%    0.0%    0.0%    0.0%    0.0%    0.0%    0.0%    0.0%    0.0%    0.0%    0.0%    0.0%        k__Bacteria; p__Bacteroidetes   328577    7.6%   10.3%    9.8%    6.3%    4.4%    8.3%    4.6%   10.5%    8.4%    3.6%    6.7%    8.6%    3.4%    5.0%    8.5%    6.3%    3.8%    2.4%    7.5%    5.5%    4.8%    3.8%    7.5%    3.1%    4.8%    7.5%    6.5%    4.7%    9.3%    4.3%    8.6%    4.1%    6.0%    3.6%    4.5%    5.8%    7.5%    6.0%    4.2%    5.2%    7.3%    4.8%    7.8%    7.7%   10.1%    5.6%    7.7%    8.7%    5.2%    7.3%    4.5%    3.5%    3.0%    5.5%    4.9%    4.3%   13.5%    3.2%    3.0%    5.3%    6.8%    6.0%    3.6%   14.2%    3.3%    5.5%    8.9%    6.0%    8.3%    5.0%    4.3%    5.5%    8.4%    8.8%    6.6%   10.9%    9.3%    5.4%    5.7%    7.5%   12.0%   10.4%   10.3%    7.7%   10.2%    7.8%   10.8%   13.4%   13.0%    8.5%    7.0%    6.4%   21.1%    5.3%   19.9%   10.3%    8.8%   15.4%   15.0%    8.3%   12.4%    3.9%   11.3%    5.7%    7.0%    9.1%    5.2%    5.3%    6.8%    7.6%    6.5%    6.2%    7.6%    3.9%    3.8%    6.9%    8.3%    6.2%    7.2%    5.1%    6.0%    7.6%    9.6%   10.4%   18.5%   10.2%    8.1%    9.0%    7.1%   13.8%   12.1%   10.4%    8.0%   15.7%        k__Bacteria; p__CCM11b      20    0.0%    0.0%    0.0%    0.0%    0.0%    0.0%    0.0%    0.0%    0.0%    0.0%    0.0%    0.0%    0.0%    0.0%    0.0%    0.0%    0.0%    0.0%    0.0%    0.0%    0.0%    0.0%    0.0%    0.0%    0.0%    0.0%    0.0%    0.0%    0.0%    0.0%    0.0%    0.0%    0.0%    0.0%    0.0%    0.0%    0.0%    0.0%    0.0%    0.0%    0.0%    0.0%    0.0%    0.0%    0.0%    0.0%    0.0%    0.0%    0.0%    0.0%    0.0%    0.0%    0.0%    0.0%    0.0%    0.0%    0.0%    0.0%    0.0%    0.0%    0.0%    0.0%    0.0%    0.0%    0.0%    0.0%    0.0%    0.0%    0.0%    0.0%    0.0%    0.0%    0.0%    0.0%    0.0%    0.0%    0.0%    0.0%    0.0%    0.0%    0.0%    0.0%    0.0%    0.0%    0.0%    0.0%    0.0%    0.0%    0.0%    0.0%    0.0%    0.0%    0.0%    0.0%    0.0%    0.0%    0.0%    0.0%    0.0%    0.0%    0.0%    0.0%    0.0%    0.0%    0.0%    0.0%    0.0%    0.0%    0.0%    0.0%    0.0%    0.0%    0.0%    0.0%    0.0%    0.0%    0.0%    0.0%    0.0%    0.0%    0.0%    0.0%    0.0%    0.0%    0.0%    0.0%    0.0%    0.0%    0.0%    0.0%    0.0%    0.0%    0.0%    0.0%        k__Bacteria; p__Caldithrix_KSB1       0    0.0%    0.0%    0.0%    0.0%    0.0%    0.0%    0.0%    0.0%    0.0%    0.0%    0.0%    0.0%    0.0%    0.0%    0.0%    0.0%    0.0%    0.0%    0.0%    0.0%    0.0%    0.0%    0.0%    0.0%    0.0%    0.0%    0.0%    0.0%    0.0%    0.0%    0.0%    0.0%    0.0%    0.0%    0.0%    0.0%    0.0%    0.0%    0.0%    0.0%    0.0%    0.0%    0.0%    0.0%    0.0%    0.0%    0.0%    0.0%    0.0%    0.0%    0.0%    0.0%    0.0%    0.0%    0.0%    0.0%    0.0%    0.0%    0.0%    0.0%    0.0%    0.0%    0.0%    0.0%    0.0%    0.0%    0.0%    0.0%    0.0%    0.0%    0.0%    0.0%    0.0%    0.0%    0.0%    0.0%    0.0%    0.0%    0.0%    0.0%    0.0%    0.0%    0.0%    0.0%    0.0%    0.0%    0.0%    0.0%    0.0%    0.0%    0.0%    0.0%    0.0%    0.0%    0.0%    0.0%    0.0%    0.0%    0.0%    0.0%    0.0%    0.0%    0.0%    0.0%    0.0%    0.0%    0.0%    0.0%    0.0%    0.0%    0.0%    0.0%    0.0%    0.0%    0.0%    0.0%    0.0%    0.0%    0.0%    0.0%    0.0%    0.0%    0.0%    0.0%    0.0%    0.0%    0.0%    0.0%    0.0%    0.0%    0.0%    0.0%    0.0%    0.0%        k__Bacteria; p__Chlamydiae      17    0.0%    0.0%    0.0%    0.0%    0.0%    0.0%    0.0%    0.0%    0.0%    0.0%    0.0%    0.0%    0.0%    0.0%    0.0%    0.0%    0.0%    0.0%    0.0%    0.0%    0.0%    0.0%    0.0%    0.0%    0.0%    0.0%    0.0%    0.0%    0.0%    0.0%    0.0%    0.0%    0.0%    0.0%    0.0%    0.0%    0.0%    0.0%    0.0%    0.0%    0.0%    0.0%    0.0%    0.0%    0.0%    0.0%    0.0%    0.0%    0.0%    0.0%    0.0%    0.0%    0.0%    0.0%    0.0%    0.0%    0.0%    0.0%    0.0%    0.0%    0.0%    0.0%    0.0%    0.0%    0.0%    0.0%    0.0%    0.0%    0.0%    0.0%    0.0%    0.0%    0.0%    0.0%    0.0%    0.0%    0.0%    0.0%    0.0%    0.0%    0.0%    0.0%    0.0%    0.0%    0.0%    0.0%    0.0%    0.0%    0.0%    0.0%    0.0%    0.0%    0.0%    0.0%    0.0%    0.0%    0.0%    0.0%    0.0%    0.0%    0.0%    0.0%    0.0%    0.0%    0.0%    0.0%    0.0%    0.0%    0.0%    0.0%    0.0%    0.0%    0.0%    0.0%    0.0%    0.0%    0.0%    0.0%    0.0%    0.0%    0.0%    0.0%    0.0%    0.0%    0.0%    0.0%    0.0%    0.0%    0.0%    0.0%    0.0%    0.0%    0.0%    0.0%        k__Bacteria; p__Chlorobi      33    0.0%    0.0%    0.0%    0.0%    0.0%    0.0%    0.0%    0.0%    0.0%    0.0%    0.0%    0.0%    0.0%    0.0%    0.0%    0.0%    0.0%    0.0%    0.0%    0.0%    0.0%    0.0%    0.0%    0.0%    0.0%    0.0%    0.0%    0.0%    0.0%    0.0%    0.0%    0.0%    0.0%    0.0%    0.0%    0.0%    0.0%    0.0%    0.0%    0.0%    0.0%    0.0%    0.0%    0.0%    0.0%    0.0%    0.0%    0.0%    0.0%    0.0%    0.0%    0.0%    0.0%    0.0%    0.0%    0.0%    0.0%    0.0%    0.0%    0.0%    0.0%    0.0%    0.0%    0.0%    0.0%    0.0%    0.0%    0.0%    0.0%    0.0%    0.0%    0.0%    0.0%    0.0%    0.0%    0.0%    0.0%    0.0%    0.0%    0.0%    0.0%    0.0%    0.0%    0.0%    0.0%    0.0%    0.0%    0.0%    0.0%    0.0%    0.0%    0.0%    0.0%    0.0%    0.0%    0.0%    0.0%    0.0%    0.0%    0.0%    0.0%    0.0%    0.0%    0.0%    0.0%    0.0%    0.0%    0.0%    0.0%    0.0%    0.0%    0.0%    0.0%    0.0%    0.0%    0.0%    0.0%    0.0%    0.0%    0.0%    0.0%    0.0%    0.0%    0.0%    0.0%    0.0%    0.0%    0.0%    0.0%    0.0%    0.0%    0.0%    0.0%    0.0%        k__Bacteria; p__Chloroflexi   11521    0.3%    0.2%    0.3%    0.3%    0.1%    0.2%    0.1%    0.6%    0.2%    0.1%    0.3%    0.2%    0.1%    0.1%    0.1%    0.1%    0.1%    0.0%    0.3%    0.1%    0.2%    0.1%    0.1%    0.1%    0.1%    0.2%    0.2%    0.4%    0.2%    0.0%    0.1%    0.1%    0.2%    0.2%    0.4%    0.1%    0.0%    0.3%    0.2%    0.2%    0.2%    0.1%    0.2%    0.2%    0.2%    0.2%    0.3%    0.1%    0.1%    0.2%    0.1%    0.1%    0.0%    0.3%    0.3%    0.2%    0.3%    0.1%    0.1%    0.4%    0.3%    0.8%    0.2%    0.0%    0.1%    0.2%    0.4%    0.2%    0.9%    0.4%    0.2%    0.5%    0.1%    0.3%    0.3%    0.5%    0.6%    0.1%    0.0%    0.8%    0.2%    0.1%    0.4%    0.0%    0.4%    0.1%    0.3%    0.3%    0.6%    0.3%    0.3%    0.5%    0.2%    0.0%    0.3%    0.3%    0.3%    0.3%    0.6%    0.2%    0.2%    0.3%    0.5%    0.3%    0.2%    0.1%    0.1%    0.3%    1.1%    0.6%    0.5%    0.8%    0.5%    0.3%    0.1%    0.4%    0.2%    0.7%    0.4%    0.2%    0.2%    0.6%    0.1%    0.3%    0.2%    0.7%    0.3%    0.2%    0.5%    0.4%    0.3%    0.3%    0.2%    0.3%        k__Bacteria; p__Cyanobacteria   278089    6.5%    2.6%    5.2%    3.8%    1.2%    2.6%    6.0%    4.9%   12.3%   41.0%    5.0%    5.5%    0.8%   12.0%    1.1%    1.8%    4.1%    0.5%    7.2%    1.0%    4.5%    1.4%    0.4%    2.1%    2.0%    2.0%   15.9%    4.8%    1.8%    1.1%    0.6%    1.6%    3.8%    3.3%    3.8%    1.7%    1.3%    5.9%    2.5%    2.9%   11.0%   15.3%    4.3%    7.7%    2.0%    3.5%    8.7%    1.5%    2.2%    1.7%    5.6%    1.3%    1.3%    7.5%    4.8%    2.6%    3.3%   20.7%    3.6%   16.1%    7.2%    3.6%    1.9%    1.1%   29.7%    5.4%    1.8%    0.9%    4.8%    3.0%   16.0%    2.5%    1.7%    2.4%    3.6%    7.3%    2.5%    2.2%    0.8%    5.7%    2.9%   20.6%    8.4%    8.7%    7.7%   12.8%    2.5%    6.5%    4.5%   14.2%    9.8%   21.8%    2.5%    0.1%    4.1%    5.0%   12.8%    6.6%    5.1%    8.6%    5.1%   10.2%    5.1%    2.9%    4.6%   11.8%   15.5%    6.3%    5.2%    3.0%   14.4%    8.3%    5.8%   15.7%   28.5%    6.4%    4.4%    6.1%   11.3%   14.5%    7.7%    9.7%    4.9%   10.6%    2.0%    1.7%   15.9%    6.3%    7.1%   20.9%    3.6%    8.9%    3.8%    5.7%        k__Bacteria; p__Deferribacteres     109    0.0%    0.0%    0.0%    0.0%    0.0%    0.0%    0.0%    0.0%    0.0%    0.0%    0.0%    0.0%    0.0%    0.0%    0.0%    0.0%    0.0%    0.0%    0.0%    0.0%    0.0%    0.0%    0.0%    0.0%    0.0%    0.0%    0.0%    0.0%    0.0%    0.0%    0.0%    0.0%    0.0%    0.0%    0.0%    0.0%    0.0%    0.0%    0.0%    0.0%    0.0%    0.0%    0.0%    0.0%    0.0%    0.0%    0.0%    0.0%    0.0%    0.0%    0.0%    0.0%    0.0%    0.0%    0.0%    0.0%    0.0%    0.0%    0.0%    0.0%    0.0%    0.0%    0.0%    0.0%    0.0%    0.0%    0.0%    0.0%    0.0%    0.0%    0.0%    0.0%    0.0%    0.0%    0.0%    0.0%    0.0%    0.0%    0.0%    0.0%    0.0%    0.0%    0.0%    0.0%    0.0%    0.0%    0.0%    0.0%    0.0%    0.0%    0.0%    0.0%    0.0%    0.0%    0.0%    0.0%    0.0%    0.0%    0.0%    0.0%    0.0%    0.0%    0.0%    0.0%    0.0%    0.0%    0.0%    0.0%    0.0%    0.0%    0.0%    0.0%    0.0%    0.0%    0.0%    0.0%    0.0%    0.0%    0.0%    0.0%    0.0%    0.0%    0.0%    0.0%    0.0%    0.0%    0.0%    0.0%    0.0%    0.0%    0.0%    0.0%    0.0%    0.0%        k__Bacteria; p__Elusimicrobia      18    0.0%    0.0%    0.0%    0.0%    0.0%    0.0%    0.0%    0.0%    0.0%    0.0%    0.0%    0.0%    0.0%    0.0%    0.0%    0.0%    0.0%    0.0%    0.0%    0.0%    0.0%    0.0%    0.0%    0.0%    0.0%    0.0%    0.0%    0.0%    0.0%    0.0%    0.0%    0.0%    0.0%    0.0%    0.0%    0.0%    0.0%    0.0%    0.0%    0.0%    0.0%    0.0%    0.0%    0.0%    0.0%    0.0%    0.0%    0.0%    0.0%    0.0%    0.0%    0.0%    0.0%    0.0%    0.0%    0.0%    0.0%    0.0%    0.0%    0.0%    0.0%    0.0%    0.0%    0.0%    0.0%    0.0%    0.0%    0.0%    0.0%    0.0%    0.0%    0.0%    0.0%    0.0%    0.0%    0.0%    0.0%    0.0%    0.0%    0.0%    0.0%    0.0%    0.0%    0.0%    0.0%    0.0%    0.0%    0.0%    0.0%    0.0%    0.0%    0.0%    0.0%    0.0%    0.0%    0.0%    0.0%    0.0%    0.0%    0.0%    0.0%    0.0%    0.0%    0.0%    0.0%    0.0%    0.0%    0.0%    0.0%    0.0%    0.0%    0.0%    0.0%    0.0%    0.0%    0.0%    0.0%    0.0%    0.0%    0.0%    0.0%    0.0%    0.0%    0.0%    0.0%    0.0%    0.0%    0.0%    0.0%    0.0%    0.0%    0.0%    0.0%    0.0%        k__Bacteria; p__Fibrobacteres      14    0.0%    0.0%    0.0%    0.0%    0.0%    0.0%    0.0%    0.0%    0.0%    0.0%    0.0%    0.0%    0.0%    0.0%    0.0%    0.0%    0.0%    0.0%    0.0%    0.0%    0.0%    0.0%    0.0%    0.0%    0.0%    0.0%    0.0%    0.0%    0.0%    0.0%    0.0%    0.0%    0.0%    0.0%    0.0%    0.0%    0.0%    0.0%    0.0%    0.0%    0.0%    0.0%    0.0%    0.0%    0.0%    0.0%    0.0%    0.0%    0.0%    0.0%    0.0%    0.0%    0.0%    0.0%    0.0%    0.0%    0.0%    0.0%    0.0%    0.0%    0.0%    0.0%    0.0%    0.0%    0.0%    0.0%    0.0%    0.0%    0.0%    0.0%    0.0%    0.0%    0.0%    0.0%    0.0%    0.0%    0.0%    0.0%    0.0%    0.0%    0.0%    0.0%    0.0%    0.0%    0.0%    0.0%    0.0%    0.0%    0.0%    0.0%    0.0%    0.0%    0.0%    0.0%    0.0%    0.0%    0.0%    0.0%    0.0%    0.0%    0.0%    0.0%    0.0%    0.0%    0.0%    0.0%    0.0%    0.0%    0.0%    0.0%    0.0%    0.0%    0.0%    0.0%    0.0%    0.0%    0.0%    0.0%    0.0%    0.0%    0.0%    0.0%    0.0%    0.0%    0.0%    0.0%    0.0%    0.0%    0.0%    0.0%    0.0%    0.0%    0.0%    0.0%        k__Bacteria; p__Firmicutes   1308379   30.4%   39.0%   27.9%   41.6%   51.5%   26.3%   46.7%   25.6%   41.1%   25.5%   39.7%   29.7%   54.8%   36.9%   41.0%   43.2%   26.0%   18.3%   27.1%   37.0%   20.5%   34.0%   23.5%   27.3%   29.0%   20.7%   29.0%   16.7%   31.7%   37.8%   35.7%   28.7%   19.0%   30.3%   21.9%   39.7%   52.2%   23.0%   34.3%   21.7%   25.0%   30.4%   12.7%   22.6%   20.2%   29.9%   20.3%   32.4%   37.4%   27.1%   29.1%   36.4%   41.7%   24.0%   30.1%   27.2%   28.5%   20.0%   25.4%   18.2%   22.0%   33.8%   48.5%   50.6%   27.5%   45.7%   35.8%   40.9%   15.5%   16.2%   20.8%   34.6%   43.5%   30.2%   35.1%   31.5%   19.0%   56.3%   45.1%   21.2%   39.1%   37.1%   24.8%   43.3%   29.7%   38.5%   30.7%   25.7%   27.0%   23.8%   27.5%   22.8%   38.0%   41.5%   37.6%   42.3%   20.4%   25.2%   29.8%   41.0%   36.6%   30.3%   34.3%   33.6%   21.2%   16.3%   24.9%   37.1%   25.1%   16.3%   20.6%   21.5%   38.0%   25.5%   10.2%   26.9%   19.3%   20.2%   27.0%   43.2%   17.8%   25.5%   37.9%   18.9%   14.5%   13.9%   22.3%   29.1%   22.3%   10.0%   29.7%   31.5%   43.2%   33.4%        k__Bacteria; p__Fusobacteria   138028    3.2%    5.4%    4.6%    2.0%    1.4%    1.6%    0.6%    1.4%    1.5%    1.7%    1.6%    2.6%    0.8%    1.7%    3.1%    2.6%    0.6%    0.3%    1.5%    0.6%    0.4%    1.4%   25.6%    0.9%    1.9%    1.4%    0.4%    0.8%    7.9%    0.9%    4.4%    1.4%    1.2%    0.6%    0.9%    1.1%    0.9%    8.0%    4.4%   20.7%    5.8%    2.7%    4.0%    9.4%   19.6%   13.6%    5.0%    6.0%   11.4%   11.9%    7.3%   13.4%    1.5%    5.5%    3.9%    3.8%    7.8%    2.8%    1.6%    6.3%    5.2%    4.7%    2.9%    8.6%    1.9%    2.4%    6.1%    1.2%    2.4%    2.0%    2.7%    2.9%    1.9%    2.3%    3.0%    5.3%    2.5%    2.2%    1.8%    1.7%    3.4%    2.7%    1.7%    1.6%    2.0%    2.4%    2.6%    3.5%    3.9%    5.5%    1.1%    2.1%    4.3%    0.7%    5.0%    3.3%    1.2%    8.1%    5.4%    2.1%    5.3%    1.4%    2.9%    0.9%    1.5%    0.8%    0.7%    0.8%    0.9%    0.2%    0.9%    0.9%    3.2%    0.8%    0.2%    1.5%    0.8%    0.7%    1.4%    1.0%    0.8%    1.2%    1.3%    1.3%    4.6%    1.5%    1.5%    1.2%    2.1%    0.8%    1.2%    1.5%    0.7%    1.5%        k__Bacteria; p__GN02       0    0.0%    0.0%    0.0%    0.0%    0.0%    0.0%    0.0%    0.0%    0.0%    0.0%    0.0%    0.0%    0.0%    0.0%    0.0%    0.0%    0.0%    0.0%    0.0%    0.0%    0.0%    0.0%    0.0%    0.0%    0.0%    0.0%    0.0%    0.0%    0.0%    0.0%    0.0%    0.0%    0.0%    0.0%    0.0%    0.0%    0.0%    0.0%    0.0%    0.0%    0.0%    0.0%    0.0%    0.0%    0.0%    0.0%    0.0%    0.0%    0.0%    0.0%    0.0%    0.0%    0.0%    0.0%    0.0%    0.0%    0.0%    0.0%    0.0%    0.0%    0.0%    0.0%    0.0%    0.0%    0.0%    0.0%    0.0%    0.0%    0.0%    0.0%    0.0%    0.0%    0.0%    0.0%    0.0%    0.0%    0.0%    0.0%    0.0%    0.0%    0.0%    0.0%    0.0%    0.0%    0.0%    0.0%    0.0%    0.0%    0.0%    0.0%    0.0%    0.0%    0.0%    0.0%    0.0%    0.0%    0.0%    0.0%    0.0%    0.0%    0.0%    0.0%    0.0%    0.0%    0.0%    0.0%    0.0%    0.0%    0.0%    0.0%    0.0%    0.0%    0.0%    0.0%    0.0%    0.0%    0.0%    0.0%    0.0%    0.0%    0.0%    0.0%    0.0%    0.0%    0.0%    0.0%    0.0%    0.0%    0.0%    0.0%    0.0%    0.0%    0.0%    0.0%        k__Bacteria; p__Gemmatimonadetes    2480    0.1%    0.1%    0.0%    0.0%    0.0%    0.1%    0.0%    0.2%    0.0%    0.0%    0.1%    0.0%    0.0%    0.0%    0.0%    0.0%    0.0%    0.0%    0.1%    0.0%    0.1%    0.0%    0.0%    0.0%    0.0%    0.1%    0.0%    0.1%    0.1%    0.0%    0.0%    0.0%    0.0%    0.0%    0.0%    0.0%    0.0%    0.0%    0.0%    0.1%    0.0%    0.0%    0.1%    0.1%    0.0%    0.0%    0.1%    0.0%    0.0%    0.0%    0.1%    0.0%    0.0%    0.1%    0.1%    0.1%    0.1%    0.0%    0.0%    0.1%    0.1%    0.2%    0.0%    0.0%    0.0%    0.0%    0.0%    0.1%    0.2%    0.1%    0.1%    0.1%    0.0%    0.1%    0.1%    0.0%    0.1%    0.0%    0.0%    0.2%    0.1%    0.0%    0.1%    0.0%    0.1%    0.1%    0.1%    0.1%    0.1%    0.1%    0.1%    0.1%    0.1%    0.0%    0.0%    0.0%    0.1%    0.1%    0.2%    0.1%    0.0%    0.0%    0.1%    0.1%    0.1%    0.0%    0.0%    0.0%    0.2%    0.1%    0.2%    0.2%    0.1%    0.1%    0.0%    0.1%    0.0%    0.2%    0.1%    0.0%    0.1%    0.1%    0.0%    0.1%    0.1%    0.1%    0.1%    0.1%    0.1%    0.1%    0.1%    0.1%    0.0%    0.0%        k__Bacteria; p__Lentisphaerae       0    0.0%    0.0%    0.0%    0.0%    0.0%    0.0%    0.0%    0.0%    0.0%    0.0%    0.0%    0.0%    0.0%    0.0%    0.0%    0.0%    0.0%    0.0%    0.0%    0.0%    0.0%    0.0%    0.0%    0.0%    0.0%    0.0%    0.0%    0.0%    0.0%    0.0%    0.0%    0.0%    0.0%    0.0%    0.0%    0.0%    0.0%    0.0%    0.0%    0.0%    0.0%    0.0%    0.0%    0.0%    0.0%    0.0%    0.0%    0.0%    0.0%    0.0%    0.0%    0.0%    0.0%    0.0%    0.0%    0.0%    0.0%    0.0%    0.0%    0.0%    0.0%    0.0%    0.0%    0.0%    0.0%    0.0%    0.0%    0.0%    0.0%    0.0%    0.0%    0.0%    0.0%    0.0%    0.0%    0.0%    0.0%    0.0%    0.0%    0.0%    0.0%    0.0%    0.0%    0.0%    0.0%    0.0%    0.0%    0.0%    0.0%    0.0%    0.0%    0.0%    0.0%    0.0%    0.0%    0.0%    0.0%    0.0%    0.0%    0.0%    0.0%    0.0%    0.0%    0.0%    0.0%    0.0%    0.0%    0.0%    0.0%    0.0%    0.0%    0.0%    0.0%    0.0%    0.0%    0.0%    0.0%    0.0%    0.0%    0.0%    0.0%    0.0%    0.0%    0.0%    0.0%    0.0%    0.0%    0.0%    0.0%    0.0%    0.0%    0.0%    0.0%    0.0%        k__Bacteria; p__MVP-15       3    0.0%    0.0%    0.0%    0.0%    0.0%    0.0%    0.0%    0.0%    0.0%    0.0%    0.0%    0.0%    0.0%    0.0%    0.0%    0.0%    0.0%    0.0%    0.0%    0.0%    0.0%    0.0%    0.0%    0.0%    0.0%    0.0%    0.0%    0.0%    0.0%    0.0%    0.0%    0.0%    0.0%    0.0%    0.0%    0.0%    0.0%    0.0%    0.0%    0.0%    0.0%    0.0%    0.0%    0.0%    0.0%    0.0%    0.0%    0.0%    0.0%    0.0%    0.0%    0.0%    0.0%    0.0%    0.0%    0.0%    0.0%    0.0%    0.0%    0.0%    0.0%    0.0%    0.0%    0.0%    0.0%    0.0%    0.0%    0.0%    0.0%    0.0%    0.0%    0.0%    0.0%    0.0%    0.0%    0.0%    0.0%    0.0%    0.0%    0.0%    0.0%    0.0%    0.0%    0.0%    0.0%    0.0%    0.0%    0.0%    0.0%    0.0%    0.0%    0.0%    0.0%    0.0%    0.0%    0.0%    0.0%    0.0%    0.0%    0.0%    0.0%    0.0%    0.0%    0.0%    0.0%    0.0%    0.0%    0.0%    0.0%    0.0%    0.0%    0.0%    0.0%    0.0%    0.0%    0.0%    0.0%    0.0%    0.0%    0.0%    0.0%    0.0%    0.0%    0.0%    0.0%    0.0%    0.0%    0.0%    0.0%    0.0%    0.0%    0.0%    0.0%    0.0%        k__Bacteria; p__NC10       1    0.0%    0.0%    0.0%    0.0%    0.0%    0.0%    0.0%    0.0%    0.0%    0.0%    0.0%    0.0%    0.0%    0.0%    0.0%    0.0%    0.0%    0.0%    0.0%    0.0%    0.0%    0.0%    0.0%    0.0%    0.0%    0.0%    0.0%    0.0%    0.0%    0.0%    0.0%    0.0%    0.0%    0.0%    0.0%    0.0%    0.0%    0.0%    0.0%    0.0%    0.0%    0.0%    0.0%    0.0%    0.0%    0.0%    0.0%    0.0%    0.0%    0.0%    0.0%    0.0%    0.0%    0.0%    0.0%    0.0%    0.0%    0.0%    0.0%    0.0%    0.0%    0.0%    0.0%    0.0%    0.0%    0.0%    0.0%    0.0%    0.0%    0.0%    0.0%    0.0%    0.0%    0.0%    0.0%    0.0%    0.0%    0.0%    0.0%    0.0%    0.0%    0.0%    0.0%    0.0%    0.0%    0.0%    0.0%    0.0%    0.0%    0.0%    0.0%    0.0%    0.0%    0.0%    0.0%    0.0%    0.0%    0.0%    0.0%    0.0%    0.0%    0.0%    0.0%    0.0%    0.0%    0.0%    0.0%    0.0%    0.0%    0.0%    0.0%    0.0%    0.0%    0.0%    0.0%    0.0%    0.0%    0.0%    0.0%    0.0%    0.0%    0.0%    0.0%    0.0%    0.0%    0.0%    0.0%    0.0%    0.0%    0.0%    0.0%    0.0%    0.0%    0.0%        k__Bacteria; p__NKB19       8    0.0%    0.0%    0.0%    0.0%    0.0%    0.0%    0.0%    0.0%    0.0%    0.0%    0.0%    0.0%    0.0%    0.0%    0.0%    0.0%    0.0%    0.0%    0.0%    0.0%    0.0%    0.0%    0.0%    0.0%    0.0%    0.0%    0.0%    0.0%    0.0%    0.0%    0.0%    0.0%    0.0%    0.0%    0.0%    0.0%    0.0%    0.0%    0.0%    0.0%    0.0%    0.0%    0.0%    0.0%    0.0%    0.0%    0.0%    0.0%    0.0%    0.0%    0.0%    0.0%    0.0%    0.0%    0.0%    0.0%    0.0%    0.0%    0.0%    0.0%    0.0%    0.0%    0.0%    0.0%    0.0%    0.0%    0.0%    0.0%    0.0%    0.0%    0.0%    0.0%    0.0%    0.0%    0.0%    0.0%    0.0%    0.0%    0.0%    0.0%    0.0%    0.0%    0.0%    0.0%    0.0%    0.0%    0.0%    0.0%    0.0%    0.0%    0.0%    0.0%    0.0%    0.0%    0.0%    0.0%    0.0%    0.0%    0.0%    0.0%    0.0%    0.0%    0.0%    0.0%    0.0%    0.0%    0.0%    0.0%    0.0%    0.0%    0.0%    0.0%    0.0%    0.0%    0.0%    0.0%    0.0%    0.0%    0.0%    0.0%    0.0%    0.0%    0.0%    0.0%    0.0%    0.0%    0.0%    0.0%    0.0%    0.0%    0.0%    0.0%    0.0%    0.0%        k__Bacteria; p__Nitrospirae     492    0.0%    0.0%    0.0%    0.0%    0.0%    0.1%    0.0%    0.0%    0.0%    0.0%    0.0%    0.0%    0.0%    0.0%    0.0%    0.0%    0.0%    0.0%    0.0%    0.0%    0.0%    0.0%    0.0%    0.0%    0.0%    0.0%    0.0%    0.0%    0.0%    0.0%    0.0%    0.0%    0.0%    0.0%    0.0%    0.0%    0.0%    0.0%    0.0%    0.0%    0.0%    0.0%    0.0%    0.0%    0.0%    0.0%    0.0%    0.0%    0.0%    0.0%    0.0%    0.0%    0.0%    0.0%    0.0%    0.0%    0.0%    0.0%    0.0%    0.0%    0.0%    0.0%    0.0%    0.0%    0.0%    0.0%    0.0%    0.0%    0.1%    0.0%    0.0%    0.0%    0.0%    0.0%    0.0%    0.0%    0.0%    0.0%    0.0%    0.0%    0.0%    0.0%    0.0%    0.0%    0.0%    0.0%    0.0%    0.0%    0.0%    0.0%    0.0%    0.0%    0.0%    0.0%    0.0%    0.0%    0.0%    0.0%    0.0%    0.0%    0.0%    0.0%    0.0%    0.0%    0.0%    0.0%    0.0%    0.0%    0.1%    0.0%    0.0%    0.1%    0.0%    0.0%    0.0%    0.0%    0.0%    0.0%    0.0%    0.0%    0.0%    0.0%    0.0%    0.0%    0.0%    0.0%    0.0%    0.0%    0.0%    0.0%    0.0%    0.0%    0.0%    0.0%        k__Bacteria; p__OP10     266    0.0%    0.0%    0.0%    0.0%    0.0%    0.0%    0.0%    0.0%    0.0%    0.0%    0.0%    0.0%    0.0%    0.0%    0.0%    0.0%    0.0%    0.0%    0.0%    0.0%    0.0%    0.0%    0.0%    0.0%    0.0%    0.0%    0.0%    0.0%    0.0%    0.0%    0.0%    0.0%    0.0%    0.0%    0.0%    0.0%    0.0%    0.0%    0.0%    0.0%    0.0%    0.0%    0.0%    0.0%    0.0%    0.0%    0.0%    0.0%    0.0%    0.0%    0.0%    0.0%    0.0%    0.0%    0.0%    0.0%    0.0%    0.0%    0.0%    0.0%    0.0%    0.0%    0.0%    0.0%    0.0%    0.0%    0.0%    0.0%    0.0%    0.0%    0.0%    0.0%    0.0%    0.0%    0.0%    0.0%    0.0%    0.0%    0.0%    0.0%    0.0%    0.0%    0.0%    0.0%    0.0%    0.0%    0.0%    0.0%    0.0%    0.0%    0.0%    0.0%    0.0%    0.0%    0.0%    0.0%    0.0%    0.0%    0.0%    0.0%    0.0%    0.0%    0.0%    0.0%    0.0%    0.0%    0.0%    0.0%    0.0%    0.0%    0.0%    0.0%    0.0%    0.0%    0.0%    0.0%    0.0%    0.0%    0.0%    0.0%    0.0%    0.0%    0.0%    0.0%    0.0%    0.0%    0.0%    0.0%    0.0%    0.0%    0.0%    0.0%    0.0%    0.0%        k__Bacteria; p__OP11       0    0.0%    0.0%    0.0%    0.0%    0.0%    0.0%    0.0%    0.0%    0.0%    0.0%    0.0%    0.0%    0.0%    0.0%    0.0%    0.0%    0.0%    0.0%    0.0%    0.0%    0.0%    0.0%    0.0%    0.0%    0.0%    0.0%    0.0%    0.0%    0.0%    0.0%    0.0%    0.0%    0.0%    0.0%    0.0%    0.0%    0.0%    0.0%    0.0%    0.0%    0.0%    0.0%    0.0%    0.0%    0.0%    0.0%    0.0%    0.0%    0.0%    0.0%    0.0%    0.0%    0.0%    0.0%    0.0%    0.0%    0.0%    0.0%    0.0%    0.0%    0.0%    0.0%    0.0%    0.0%    0.0%    0.0%    0.0%    0.0%    0.0%    0.0%    0.0%    0.0%    0.0%    0.0%    0.0%    0.0%    0.0%    0.0%    0.0%    0.0%    0.0%    0.0%    0.0%    0.0%    0.0%    0.0%    0.0%    0.0%    0.0%    0.0%    0.0%    0.0%    0.0%    0.0%    0.0%    0.0%    0.0%    0.0%    0.0%    0.0%    0.0%    0.0%    0.0%    0.0%    0.0%    0.0%    0.0%    0.0%    0.0%    0.0%    0.0%    0.0%    0.0%    0.0%    0.0%    0.0%    0.0%    0.0%    0.0%    0.0%    0.0%    0.0%    0.0%    0.0%    0.0%    0.0%    0.0%    0.0%    0.0%    0.0%    0.0%    0.0%    0.0%    0.0%        k__Bacteria; p__OP3       0    0.0%    0.0%    0.0%    0.0%    0.0%    0.0%    0.0%    0.0%    0.0%    0.0%    0.0%    0.0%    0.0%    0.0%    0.0%    0.0%    0.0%    0.0%    0.0%    0.0%    0.0%    0.0%    0.0%    0.0%    0.0%    0.0%    0.0%    0.0%    0.0%    0.0%    0.0%    0.0%    0.0%    0.0%    0.0%    0.0%    0.0%    0.0%    0.0%    0.0%    0.0%    0.0%    0.0%    0.0%    0.0%    0.0%    0.0%    0.0%    0.0%    0.0%    0.0%    0.0%    0.0%    0.0%    0.0%    0.0%    0.0%    0.0%    0.0%    0.0%    0.0%    0.0%    0.0%    0.0%    0.0%    0.0%    0.0%    0.0%    0.0%    0.0%    0.0%    0.0%    0.0%    0.0%    0.0%    0.0%    0.0%    0.0%    0.0%    0.0%    0.0%    0.0%    0.0%    0.0%    0.0%    0.0%    0.0%    0.0%    0.0%    0.0%    0.0%    0.0%    0.0%    0.0%    0.0%    0.0%    0.0%    0.0%    0.0%    0.0%    0.0%    0.0%    0.0%    0.0%    0.0%    0.0%    0.0%    0.0%    0.0%    0.0%    0.0%    0.0%    0.0%    0.0%    0.0%    0.0%    0.0%    0.0%    0.0%    0.0%    0.0%    0.0%    0.0%    0.0%    0.0%    0.0%    0.0%    0.0%    0.0%    0.0%    0.0%    0.0%    0.0%    0.0%        k__Bacteria; p__OP8       0    0.0%    0.0%    0.0%    0.0%    0.0%    0.0%    0.0%    0.0%    0.0%    0.0%    0.0%    0.0%    0.0%    0.0%    0.0%    0.0%    0.0%    0.0%    0.0%    0.0%    0.0%    0.0%    0.0%    0.0%    0.0%    0.0%    0.0%    0.0%    0.0%    0.0%    0.0%    0.0%    0.0%    0.0%    0.0%    0.0%    0.0%    0.0%    0.0%    0.0%    0.0%    0.0%    0.0%    0.0%    0.0%    0.0%    0.0%    0.0%    0.0%    0.0%    0.0%    0.0%    0.0%    0.0%    0.0%    0.0%    0.0%    0.0%    0.0%    0.0%    0.0%    0.0%    0.0%    0.0%    0.0%    0.0%    0.0%    0.0%    0.0%    0.0%    0.0%    0.0%    0.0%    0.0%    0.0%    0.0%    0.0%    0.0%    0.0%    0.0%    0.0%    0.0%    0.0%    0.0%    0.0%    0.0%    0.0%    0.0%    0.0%    0.0%    0.0%    0.0%    0.0%    0.0%    0.0%    0.0%    0.0%    0.0%    0.0%    0.0%    0.0%    0.0%    0.0%    0.0%    0.0%    0.0%    0.0%    0.0%    0.0%    0.0%    0.0%    0.0%    0.0%    0.0%    0.0%    0.0%    0.0%    0.0%    0.0%    0.0%    0.0%    0.0%    0.0%    0.0%    0.0%    0.0%    0.0%    0.0%    0.0%    0.0%    0.0%    0.0%    0.0%    0.0%        k__Bacteria; p__Planctomycetes    3629    0.1%    0.1%    0.1%    0.1%    0.0%    0.2%    0.1%    0.1%    0.0%    0.0%    0.1%    0.1%    0.0%    0.0%    0.0%    0.0%    0.0%    0.0%    0.1%    0.0%    0.1%    0.0%    0.0%    0.0%    0.0%    0.0%    0.1%    0.0%    0.1%    0.0%    0.1%    0.0%    0.1%    0.0%    0.1%    0.0%    0.0%    0.1%    0.0%    0.1%    0.1%    0.0%    0.1%    0.1%    0.0%    0.0%    0.2%    0.0%    0.0%    0.0%    0.1%    0.0%    0.0%    0.2%    0.1%    0.0%    0.1%    0.0%    0.0%    0.1%    0.1%    0.2%    0.1%    0.0%    0.1%    0.0%    0.1%    0.1%    0.5%    0.2%    0.0%    0.1%    0.0%    0.2%    0.1%    0.2%    0.2%    0.0%    0.0%    0.3%    0.1%    0.0%    0.2%    0.0%    0.1%    0.1%    0.1%    0.1%    0.3%    0.0%    0.1%    0.1%    0.0%    0.0%    0.1%    0.1%    0.1%    0.1%    0.2%    0.1%    0.1%    0.1%    0.2%    0.1%    0.1%    0.0%    0.0%    0.0%    0.5%    0.3%    0.2%    0.3%    0.1%    0.1%    0.0%    0.2%    0.2%    0.2%    0.1%    0.0%    0.1%    0.2%    0.0%    0.1%    0.0%    0.2%    0.1%    0.1%    0.1%    0.1%    0.1%    0.1%    0.0%    0.1%        k__Bacteria; p__Proteobacteria   1290123   30.0%   18.9%   24.3%   31.2%   13.6%   41.7%   17.8%   34.5%   22.6%   17.5%   28.2%   22.1%   12.0%   21.5%   29.3%   27.8%   46.1%   34.9%   32.0%   18.9%   51.6%   24.3%   25.3%   37.5%   31.4%   52.5%   32.1%   55.8%   22.5%   13.4%   32.2%   29.5%   46.0%   40.2%   55.2%   42.2%   12.1%   39.6%   26.4%   30.6%   35.8%   31.8%   59.1%   35.4%   34.7%   28.7%   36.0%   39.4%   27.8%   32.6%   34.7%   24.2%   32.5%   33.4%   39.7%   40.1%   27.0%   37.8%   28.7%   26.8%   28.0%   27.5%   18.3%    9.3%   20.7%   16.7%   31.7%   11.9%   36.9%   50.4%   37.5%   24.0%   19.8%   31.4%   27.4%   24.1%   41.9%    9.0%   11.5%   28.8%   22.1%   14.3%   30.8%   12.2%   28.3%   20.5%   33.8%   32.1%   29.8%   28.7%   31.0%   25.4%   14.1%   13.4%   17.9%   23.4%   30.5%   22.0%   26.0%   24.6%   23.1%   38.9%   26.7%   21.7%   45.2%   52.2%   37.8%   27.8%   30.7%   43.9%   31.0%   36.9%   20.9%   32.0%   47.8%   28.3%   36.7%   41.5%   29.5%   22.0%   39.8%   31.9%   27.7%   41.2%   45.7%   49.8%   32.8%   28.4%   35.5%   40.8%   34.2%   28.6%   26.4%   26.4%        k__Bacteria; p__SC3       5    0.0%    0.0%    0.0%    0.0%    0.0%    0.0%    0.0%    0.0%    0.0%    0.0%    0.0%    0.0%    0.0%    0.0%    0.0%    0.0%    0.0%    0.0%    0.0%    0.0%    0.0%    0.0%    0.0%    0.0%    0.0%    0.0%    0.0%    0.0%    0.0%    0.0%    0.0%    0.0%    0.0%    0.0%    0.0%    0.0%    0.0%    0.0%    0.0%    0.0%    0.0%    0.0%    0.0%    0.0%    0.0%    0.0%    0.0%    0.0%    0.0%    0.0%    0.0%    0.0%    0.0%    0.0%    0.0%    0.0%    0.0%    0.0%    0.0%    0.0%    0.0%    0.0%    0.0%    0.0%    0.0%    0.0%    0.0%    0.0%    0.0%    0.0%    0.0%    0.0%    0.0%    0.0%    0.0%    0.0%    0.0%    0.0%    0.0%    0.0%    0.0%    0.0%    0.0%    0.0%    0.0%    0.0%    0.0%    0.0%    0.0%    0.0%    0.0%    0.0%    0.0%    0.0%    0.0%    0.0%    0.0%    0.0%    0.0%    0.0%    0.0%    0.0%    0.0%    0.0%    0.0%    0.0%    0.0%    0.0%    0.0%    0.0%    0.0%    0.0%    0.0%    0.0%    0.0%    0.0%    0.0%    0.0%    0.0%    0.0%    0.0%    0.0%    0.0%    0.0%    0.0%    0.0%    0.0%    0.0%    0.0%    0.0%    0.0%    0.0%    0.0%    0.0%        k__Bacteria; p__SC4     624    0.0%    0.0%    0.0%    0.0%    0.0%    0.0%    0.0%    0.1%    0.0%    0.0%    0.0%    0.0%    0.0%    0.0%    0.0%    0.0%    0.0%    0.0%    0.0%    0.0%    0.0%    0.0%    0.0%    0.0%    0.0%    0.0%    0.0%    0.0%    0.0%    0.0%    0.0%    0.0%    0.0%    0.0%    0.0%    0.0%    0.0%    0.0%    0.0%    0.0%    0.0%    0.0%    0.0%    0.0%    0.0%    0.0%    0.0%    0.0%    0.0%    0.0%    0.0%    0.0%    0.1%    0.0%    0.0%    0.0%    0.0%    0.0%    0.0%    0.0%    0.0%    0.0%    0.0%    0.0%    0.0%    0.0%    0.0%    0.0%    0.0%    0.0%    0.0%    0.1%    0.0%    0.0%    0.0%    0.0%    0.0%    0.0%    0.0%    0.0%    0.0%    0.0%    0.0%    0.0%    0.0%    0.0%    0.0%    0.0%    0.0%    0.0%    0.0%    0.1%    0.0%    0.0%    0.0%    0.0%    0.0%    0.0%    0.0%    0.0%    0.0%    0.0%    0.0%    0.0%    0.0%    0.0%    0.0%    0.0%    0.0%    0.0%    0.0%    0.0%    0.0%    0.0%    0.0%    0.0%    0.0%    0.0%    0.0%    0.0%    0.0%    0.0%    0.0%    0.0%    0.0%    0.0%    0.0%    0.0%    0.0%    0.0%    0.0%    0.0%    0.0%    0.0%        k__Bacteria; p__SPAM     139    0.0%    0.0%    0.0%    0.0%    0.0%    0.0%    0.0%    0.0%    0.0%    0.0%    0.0%    0.0%    0.0%    0.0%    0.0%    0.0%    0.0%    0.0%    0.0%    0.0%    0.0%    0.0%    0.0%    0.0%    0.0%    0.0%    0.0%    0.0%    0.0%    0.0%    0.0%    0.0%    0.0%    0.0%    0.0%    0.0%    0.0%    0.0%    0.0%    0.0%    0.0%    0.0%    0.0%    0.0%    0.0%    0.0%    0.0%    0.0%    0.0%    0.0%    0.0%    0.0%    0.0%    0.0%    0.0%    0.0%    0.0%    0.0%    0.0%    0.0%    0.0%    0.0%    0.0%    0.0%    0.0%    0.0%    0.0%    0.0%    0.0%    0.0%    0.0%    0.0%    0.0%    0.0%    0.0%    0.0%    0.0%    0.0%    0.0%    0.0%    0.0%    0.0%    0.0%    0.0%    0.0%    0.0%    0.0%    0.0%    0.0%    0.0%    0.0%    0.0%    0.0%    0.0%    0.0%    0.0%    0.0%    0.0%    0.0%    0.0%    0.0%    0.0%    0.0%    0.0%    0.0%    0.0%    0.0%    0.0%    0.0%    0.0%    0.0%    0.0%    0.0%    0.0%    0.0%    0.0%    0.0%    0.0%    0.0%    0.0%    0.0%    0.0%    0.0%    0.0%    0.0%    0.0%    0.0%    0.0%    0.0%    0.0%    0.0%    0.0%    0.0%    0.0%        k__Bacteria; p__SR1     586    0.0%    0.0%    0.0%    0.0%    0.0%    0.0%    0.0%    0.0%    0.0%    0.0%    0.0%    0.0%    0.0%    0.0%    0.0%    0.0%    0.0%    0.0%    0.0%    0.0%    0.0%    0.0%    0.0%    0.0%    0.0%    0.0%    0.0%    0.0%    0.0%    0.0%    0.0%    0.0%    0.0%    0.0%    0.0%    0.0%    0.0%    0.0%    0.0%    0.0%    0.0%    0.0%    0.0%    0.0%    0.0%    0.0%    0.0%    0.0%    0.0%    0.0%    0.0%    0.0%    0.0%    0.0%    0.0%    0.1%    0.0%    0.0%    0.1%    0.0%    0.0%    0.0%    0.0%    0.0%    0.0%    0.0%    0.0%    0.0%    0.0%    0.0%    0.0%    0.0%    0.0%    0.0%    0.0%    0.0%    0.0%    0.0%    0.0%    0.0%    0.0%    0.0%    0.0%    0.0%    0.1%    0.0%    0.0%    0.0%    0.0%    0.0%    0.0%    0.0%    0.0%    0.0%    0.0%    0.0%    0.0%    0.0%    0.0%    0.0%    0.0%    0.0%    0.0%    0.0%    0.0%    0.0%    0.0%    0.0%    0.0%    0.0%    0.0%    0.0%    0.0%    0.0%    0.0%    0.0%    0.0%    0.0%    0.0%    0.0%    0.0%    0.0%    0.0%    0.0%    0.0%    0.0%    0.0%    0.0%    0.0%    0.0%    0.0%    0.0%    0.0%    0.0%        k__Bacteria; p__Spirochaetes    3445    0.1%    0.1%    0.1%    0.0%    0.0%    0.0%    0.0%    0.0%    0.0%    0.0%    0.1%    0.4%    0.1%    0.0%    0.0%    0.1%    0.0%    0.0%    0.1%    0.0%    0.0%    0.1%    0.1%    0.0%    0.0%    0.2%    0.0%    0.1%    0.1%    0.1%    0.1%    0.1%    0.1%    0.1%    0.1%    0.0%    0.0%    0.0%    0.0%    0.1%    0.2%    0.0%    0.0%    0.1%    0.2%    0.1%    0.0%    0.1%    0.1%    0.2%    0.2%    0.0%    0.0%    0.1%    0.0%    0.0%    0.1%    0.0%    0.0%    0.0%    0.0%    0.1%    0.1%    0.2%    0.1%    0.0%    0.1%    0.0%    0.0%    0.1%    0.0%    0.1%    0.1%    0.3%    0.0%    0.4%    0.2%    0.0%    0.0%    0.0%    0.0%    0.1%    0.2%    0.0%    0.4%    0.0%    0.3%    0.1%    0.2%    0.1%    0.1%    0.0%    0.0%    0.0%    0.2%    0.4%    0.1%    0.1%    0.2%    0.2%    0.1%    0.1%    0.1%    0.0%    0.1%    0.1%    0.0%    0.1%    0.0%    0.0%    0.0%    0.0%    0.1%    0.0%    0.0%    0.2%    0.0%    0.0%    0.1%    0.0%    0.0%    0.0%    0.0%    0.1%    0.2%    0.1%    0.1%    0.1%    0.0%    0.0%    0.1%    0.0%    0.0%    0.0%        k__Bacteria; p__Synergistetes     385    0.0%    0.0%    0.0%    0.0%    0.0%    0.0%    0.0%    0.0%    0.0%    0.0%    0.1%    0.0%    0.0%    0.0%    0.0%    0.0%    0.0%    0.0%    0.0%    0.0%    0.0%    0.0%    0.0%    0.0%    0.0%    0.0%    0.0%    0.0%    0.0%    0.0%    0.0%    0.1%    0.0%    0.0%    0.0%    0.0%    0.0%    0.0%    0.0%    0.0%    0.0%    0.0%    0.0%    0.0%    0.0%    0.0%    0.0%    0.0%    0.0%    0.0%    0.0%    0.0%    0.0%    0.0%    0.0%    0.0%    0.0%    0.0%    0.0%    0.0%    0.0%    0.0%    0.0%    0.0%    0.0%    0.0%    0.0%    0.0%    0.0%    0.0%    0.0%    0.0%    0.0%    0.0%    0.0%    0.0%    0.0%    0.0%    0.0%    0.0%    0.0%    0.0%    0.0%    0.0%    0.0%    0.0%    0.1%    0.0%    0.0%    0.0%    0.0%    0.0%    0.0%    0.0%    0.0%    0.1%    0.0%    0.0%    0.0%    0.0%    0.0%    0.0%    0.0%    0.0%    0.0%    0.0%    0.0%    0.0%    0.0%    0.0%    0.0%    0.0%    0.0%    0.0%    0.0%    0.0%    0.0%    0.0%    0.0%    0.0%    0.0%    0.0%    0.0%    0.0%    0.1%    0.0%    0.0%    0.0%    0.0%    0.0%    0.0%    0.0%    0.0%    0.0%        k__Bacteria; p__TM6       5    0.0%    0.0%    0.0%    0.0%    0.0%    0.0%    0.0%    0.0%    0.0%    0.0%    0.0%    0.0%    0.0%    0.0%    0.0%    0.0%    0.0%    0.0%    0.0%    0.0%    0.0%    0.0%    0.0%    0.0%    0.0%    0.0%    0.0%    0.0%    0.0%    0.0%    0.0%    0.0%    0.0%    0.0%    0.0%    0.0%    0.0%    0.0%    0.0%    0.0%    0.0%    0.0%    0.0%    0.0%    0.0%    0.0%    0.0%    0.0%    0.0%    0.0%    0.0%    0.0%    0.0%    0.0%    0.0%    0.0%    0.0%    0.0%    0.0%    0.0%    0.0%    0.0%    0.0%    0.0%    0.0%    0.0%    0.0%    0.0%    0.0%    0.0%    0.0%    0.0%    0.0%    0.0%    0.0%    0.0%    0.0%    0.0%    0.0%    0.0%    0.0%    0.0%    0.0%    0.0%    0.0%    0.0%    0.0%    0.0%    0.0%    0.0%    0.0%    0.0%    0.0%    0.0%    0.0%    0.0%    0.0%    0.0%    0.0%    0.0%    0.0%    0.0%    0.0%    0.0%    0.0%    0.0%    0.0%    0.0%    0.0%    0.0%    0.0%    0.0%    0.0%    0.0%    0.0%    0.0%    0.0%    0.0%    0.0%    0.0%    0.0%    0.0%    0.0%    0.0%    0.0%    0.0%    0.0%    0.0%    0.0%    0.0%    0.0%    0.0%    0.0%    0.0%        k__Bacteria; p__TM7     128    0.0%    0.0%    0.0%    0.0%    0.0%    0.0%    0.0%    0.0%    0.0%    0.0%    0.0%    0.0%    0.0%    0.0%    0.0%    0.0%    0.0%    0.0%    0.0%    0.0%    0.0%    0.0%    0.0%    0.0%    0.0%    0.0%    0.0%    0.0%    0.0%    0.0%    0.0%    0.0%    0.0%    0.0%    0.0%    0.0%    0.0%    0.0%    0.0%    0.0%    0.0%    0.0%    0.0%    0.0%    0.0%    0.0%    0.0%    0.0%    0.0%    0.0%    0.0%    0.0%    0.0%    0.0%    0.0%    0.0%    0.0%    0.0%    0.0%    0.0%    0.0%    0.0%    0.0%    0.0%    0.0%    0.0%    0.0%    0.0%    0.0%    0.0%    0.0%    0.0%    0.0%    0.0%    0.0%    0.0%    0.0%    0.0%    0.0%    0.0%    0.0%    0.0%    0.0%    0.0%    0.0%    0.0%    0.0%    0.0%    0.0%    0.0%    0.0%    0.0%    0.0%    0.0%    0.0%    0.0%    0.0%    0.0%    0.0%    0.0%    0.0%    0.0%    0.0%    0.0%    0.0%    0.0%    0.0%    0.0%    0.0%    0.0%    0.0%    0.0%    0.0%    0.0%    0.0%    0.0%    0.0%    0.0%    0.0%    0.0%    0.0%    0.0%    0.0%    0.0%    0.0%    0.0%    0.0%    0.0%    0.0%    0.0%    0.0%    0.0%    0.0%    0.0%        k__Bacteria; p__Tenericutes   35536    0.8%    0.7%    0.7%    0.3%    0.3%    0.4%    0.3%    0.4%    0.4%    0.1%    0.5%    0.6%    0.6%    0.4%    0.3%    0.6%    0.4%    0.1%    1.1%    0.5%    0.3%    0.5%    2.0%    0.3%    0.4%    0.6%    0.2%    0.4%    2.0%    0.4%    0.2%    0.4%    0.6%    0.4%    0.7%    0.2%    0.4%    2.3%    2.0%    3.9%    1.9%    0.2%    0.4%    1.2%    1.6%    1.2%    1.2%    1.5%    1.3%    2.7%    1.1%    2.1%    0.0%    1.2%    0.6%    0.6%    1.5%    0.6%    0.4%    1.1%    0.9%    1.0%    1.6%    0.2%    0.7%    0.8%    1.8%    0.5%    0.9%    0.9%    0.5%    0.7%    0.2%    0.7%    1.1%    1.2%    0.6%    0.2%    0.1%    0.9%    2.0%    1.9%    1.3%    0.2%    1.0%    0.4%    0.9%    1.1%    1.3%    1.1%    0.3%    0.7%    2.5%    0.1%    3.8%    0.4%    0.7%    2.0%    1.5%    0.6%    2.8%    0.5%    0.9%    0.3%    0.4%    0.7%    0.4%    0.4%    0.3%    0.2%    1.0%    1.3%    0.9%    0.5%    0.1%    0.5%    0.3%    0.6%    0.5%    0.4%    0.4%    1.1%    0.5%    0.5%    0.7%    0.5%    0.7%    0.7%    1.6%    0.7%    0.4%    0.7%    0.2%    0.6%        k__Bacteria; p__Thermi    7764    0.2%    0.2%    0.1%    0.5%    0.1%    0.2%    0.1%    0.3%    0.1%    0.0%    0.4%    0.2%    0.0%    0.2%    0.3%    0.1%    0.1%    0.2%    0.1%    0.1%    0.1%    0.1%    0.0%    0.2%    0.2%    0.1%    0.1%    0.2%    0.1%    0.2%    0.1%    0.2%    0.1%    0.1%    0.8%    0.2%    0.1%    0.2%    0.1%    0.1%    0.2%    0.4%    0.1%    0.1%    0.1%    0.1%    0.3%    0.1%    0.0%    0.2%    0.4%    0.2%    0.1%    0.2%    0.1%    0.2%    0.0%    0.2%    0.4%    0.1%    0.5%    0.2%    0.2%    0.3%    0.3%    0.3%    0.1%    0.1%    0.1%    0.2%    0.3%    0.4%    0.2%    0.1%    0.2%    0.1%    0.1%    0.6%    0.9%    0.1%    0.1%    0.1%    0.3%    0.6%    0.4%    0.1%    0.1%    0.1%    0.3%    0.2%    0.2%    0.2%    0.1%    0.1%    0.0%    0.1%    0.2%    0.1%    0.1%    0.1%    0.1%    0.2%    0.1%    0.4%    0.2%    0.1%    0.4%    0.1%    0.2%    0.3%    0.3%    0.2%    0.2%    0.2%    0.1%    0.1%    0.3%    0.2%    0.2%    0.1%    0.1%    0.2%    0.2%    0.2%    0.1%    0.1%    0.1%    0.2%    0.1%    0.1%    0.1%    0.1%    0.1%    0.1%        k__Bacteria; p__Thermotogae      13    0.0%    0.0%    0.0%    0.0%    0.0%    0.0%    0.0%    0.0%    0.0%    0.0%    0.0%    0.0%    0.0%    0.0%    0.0%    0.0%    0.0%    0.0%    0.0%    0.0%    0.0%    0.0%    0.0%    0.0%    0.0%    0.0%    0.0%    0.0%    0.0%    0.0%    0.0%    0.0%    0.0%    0.0%    0.0%    0.0%    0.0%    0.0%    0.0%    0.0%    0.0%    0.0%    0.0%    0.0%    0.0%    0.0%    0.0%    0.0%    0.0%    0.0%    0.0%    0.0%    0.0%    0.0%    0.0%    0.0%    0.0%    0.0%    0.0%    0.0%    0.0%    0.0%    0.0%    0.0%    0.0%    0.0%    0.0%    0.0%    0.0%    0.0%    0.0%    0.0%    0.0%    0.0%    0.0%    0.0%    0.0%    0.0%    0.0%    0.0%    0.0%    0.0%    0.0%    0.0%    0.0%    0.0%    0.0%    0.0%    0.0%    0.0%    0.0%    0.0%    0.0%    0.0%    0.0%    0.0%    0.0%    0.0%    0.0%    0.0%    0.0%    0.0%    0.0%    0.0%    0.0%    0.0%    0.0%    0.0%    0.0%    0.0%    0.0%    0.0%    0.0%    0.0%    0.0%    0.0%    0.0%    0.0%    0.0%    0.0%    0.0%    0.0%    0.0%    0.0%    0.0%    0.0%    0.0%    0.0%    0.0%    0.0%    0.0%    0.0%    0.0%    0.0%        k__Bacteria; p__Verrucomicrobia    5302    0.1%    0.1%    0.2%    0.1%    0.0%    0.3%    0.2%    0.2%    0.1%    0.0%    0.2%    0.1%    0.0%    0.1%    0.1%    0.1%    0.1%    0.0%    0.2%    0.0%    0.1%    0.0%    0.0%    0.0%    0.1%    0.1%    0.3%    0.1%    0.1%    0.0%    0.0%    0.1%    0.1%    0.0%    0.0%    0.0%    0.0%    0.1%    0.0%    0.1%    0.1%    0.0%    0.1%    0.1%    0.1%    0.0%    0.1%    0.0%    0.0%    0.1%    0.0%    0.0%    0.1%    0.1%    0.1%    0.1%    0.1%    0.1%    0.1%    0.2%    0.1%    0.3%    0.0%    0.1%    0.0%    0.1%    0.1%    0.1%    0.5%    0.1%    0.1%    0.2%    0.0%    0.2%    0.1%    0.1%    0.2%    0.0%    0.0%    0.4%    0.1%    0.1%    0.5%    0.0%    0.1%    0.1%    0.1%    0.2%    0.2%    0.1%    0.2%    0.2%    0.1%    0.0%    0.1%    0.1%    0.2%    0.1%    0.1%    0.1%    0.1%    0.1%    0.1%    0.1%    0.1%    0.1%    0.1%    0.1%    0.8%    1.1%    0.3%    0.4%    0.1%    0.1%    0.0%    0.1%    0.1%    0.2%    0.1%    0.1%    0.1%    0.1%    0.1%    0.1%    0.3%    0.2%    0.1%    0.1%    0.1%    0.2%    0.2%    0.1%    0.1%    0.2%        k__Bacteria; p__WPS-2      44    0.0%    0.0%    0.0%    0.0%    0.0%    0.0%    0.0%    0.0%    0.0%    0.0%    0.0%    0.0%    0.0%    0.0%    0.0%    0.0%    0.0%    0.0%    0.0%    0.0%    0.0%    0.0%    0.0%    0.0%    0.0%    0.0%    0.0%    0.0%    0.0%    0.0%    0.0%    0.0%    0.0%    0.0%    0.0%    0.0%    0.0%    0.0%    0.0%    0.0%    0.0%    0.0%    0.0%    0.0%    0.0%    0.0%    0.0%    0.0%    0.0%    0.0%    0.0%    0.0%    0.0%    0.0%    0.0%    0.0%    0.0%    0.0%    0.0%    0.0%    0.0%    0.0%    0.0%    0.0%    0.0%    0.0%    0.0%    0.0%    0.0%    0.0%    0.0%    0.0%    0.0%    0.0%    0.0%    0.0%    0.0%    0.0%    0.0%    0.0%    0.0%    0.0%    0.0%    0.0%    0.0%    0.0%    0.0%    0.0%    0.0%    0.0%    0.0%    0.0%    0.0%    0.0%    0.0%    0.0%    0.0%    0.0%    0.0%    0.0%    0.0%    0.0%    0.0%    0.0%    0.0%    0.0%    0.0%    0.0%    0.0%    0.0%    0.0%    0.0%    0.0%    0.0%    0.0%    0.0%    0.0%    0.0%    0.0%    0.0%    0.0%    0.0%    0.0%    0.0%    0.0%    0.0%    0.0%    0.0%    0.0%    0.0%    0.0%    0.0%    0.0%    0.0%        k__Bacteria; p__WS3      31    0.0%    0.0%    0.0%    0.0%    0.0%    0.0%    0.0%    0.0%    0.0%    0.0%    0.0%    0.0%    0.0%    0.0%    0.0%    0.0%    0.0%    0.0%    0.0%    0.0%    0.0%    0.0%    0.0%    0.0%    0.0%    0.0%    0.0%    0.0%    0.0%    0.0%    0.0%    0.0%    0.0%    0.0%    0.0%    0.0%    0.0%    0.0%    0.0%    0.0%    0.0%    0.0%    0.0%    0.0%    0.0%    0.0%    0.0%    0.0%    0.0%    0.0%    0.0%    0.0%    0.0%    0.0%    0.0%    0.0%    0.0%    0.0%    0.0%    0.0%    0.0%    0.0%    0.0%    0.0%    0.0%    0.0%    0.0%    0.0%    0.0%    0.0%    0.0%    0.0%    0.0%    0.0%    0.0%    0.0%    0.0%    0.0%    0.0%    0.0%    0.0%    0.0%    0.0%    0.0%    0.0%    0.0%    0.0%    0.0%    0.0%    0.0%    0.0%    0.0%    0.0%    0.0%    0.0%    0.0%    0.0%    0.0%    0.0%    0.0%    0.0%    0.0%    0.0%    0.0%    0.0%    0.0%    0.0%    0.0%    0.0%    0.0%    0.0%    0.0%    0.0%    0.0%    0.0%    0.0%    0.0%    0.0%    0.0%    0.0%    0.0%    0.0%    0.0%    0.0%    0.0%    0.0%    0.0%    0.0%    0.0%    0.0%    0.0%    0.0%    0.0%    0.0%        k__Bacteria; p__ZB2       1    0.0%    0.0%    0.0%    0.0%    0.0%    0.0%    0.0%    0.0%    0.0%    0.0%    0.0%    0.0%    0.0%    0.0%    0.0%    0.0%    0.0%    0.0%    0.0%    0.0%    0.0%    0.0%    0.0%    0.0%    0.0%    0.0%    0.0%    0.0%    0.0%    0.0%    0.0%    0.0%    0.0%    0.0%    0.0%    0.0%    0.0%    0.0%    0.0%    0.0%    0.0%    0.0%    0.0%    0.0%    0.0%    0.0%    0.0%    0.0%    0.0%    0.0%    0.0%    0.0%    0.0%    0.0%    0.0%    0.0%    0.0%    0.0%    0.0%    0.0%    0.0%    0.0%    0.0%    0.0%    0.0%    0.0%    0.0%    0.0%    0.0%    0.0%    0.0%    0.0%    0.0%    0.0%    0.0%    0.0%    0.0%    0.0%    0.0%    0.0%    0.0%    0.0%    0.0%    0.0%    0.0%    0.0%    0.0%    0.0%    0.0%    0.0%    0.0%    0.0%    0.0%    0.0%    0.0%    0.0%    0.0%    0.0%    0.0%    0.0%    0.0%    0.0%    0.0%    0.0%    0.0%    0.0%    0.0%    0.0%    0.0%    0.0%    0.0%    0.0%    0.0%    0.0%    0.0%    0.0%    0.0%    0.0%    0.0%    0.0%    0.0%    0.0%    0.0%    0.0%    0.0%    0.0%    0.0%    0.0%    0.0%    0.0%    0.0%    0.0%    0.0%    0.0%    
     
  Taxonomy Summary. Current Level: Class  
     View Figure (.pdf)    View Legend (.pdf)   
   
 
     
 
 

 
 
 
 
 
 
 
 
 
 
 
 
 
 
 
 
 
 
 
 
 
 
 
 
 
 
 
 
 
 
 
 
 
 
 
 
 
 
 
 
 
 
 
 
 
 
 
 
 
 
 
 
 
 
 
 
 
 
 
 
 
 
 
 
 
 
 
 
 
 
 
 
 
 
 
 
 
 
 
 
 
 
 
 
 
 
 
 
 
 
 
 
 
 
 
 
 
 
 
 
 
 
 
 
 
 
 
 
 
 
 
 
 
 
 
 
 
 
 
 
 
 
 
 
 
 
 
 
 
 
 
 
 
 
 
 
 
 
 
 
 
 
 
 
 
 
 
 
 
 
 
 
 
 
 
 
 
 
 
 
 
 
 
 
 
 
 
 
 
 
 
 
 
 
 
 
 
 
 
 
 
 
 
 
 
 
 
 
 
 
 
 
 
 
 
 
 
 
 
 
 
 
 
 
 
 
 
 
 
 
 
 
 
 
 
 
 
 
 
 
 
 
 
 
 
 
 
 
 
 
 
 
 
 
 
 
 
 
 
 
 
 
 
 
 
 
 
 
 
 
 
 
 
 
 
 
 
 
 
 
 
 
 
 
 
 
 
 
 
 
 
 
 
 
 
 
 
 
 
 
 
 
 
 
 
 
 
 
 
 
 
 
 
 
 
 
 
 
 
 
 
 
 
 
 
 
 
 
 
 
 
 
 
 
 
 
 
 
 
 
 
 
 
 
 
 
 
 
 
 
 
 
 
 
 
 
 
 
 
 
 
 
 
 
 
 
 
 
 
 
 
 
 
 
 
 
 
 
 
 
 
 
 
 
 
 
 
 
 
 
 
 
 
 
 
 
 
 
 
 
 
 
 
 
 
 
 
 
 
 
 
 
 
 
 
 
 
 
 
 
 
 
 
 
 
 
 
 
 
 
 
 
 
 
 
 
 
 
 
 
 
 
 
 
 
 
 
 
 
 
 
 
 
 
 
 
 
 
 
 
 
 
 
 
 
 
 
 
 
 
 
 
 
 
 
 
 
 
 
 
 
 
 
 
 
 
 
 
 
 
 
 
 
 
 
 
 
 
 
 
 
 
 
 
 
 
 
 
 
 
 
 
 
 
 
 
 
 
 
 
 
 
 
 
 
 
 
 
 
 
 
 
 
 
 
 
 
 
 
 
 
 
 
 
 
 
 
 
 
 
 
 
 
 
 
 
 
 
 
 
 
 
 
 
 
 
 
 
 
 
 
 
 
 
 
 
 
 
 
 
 
 
 
 
 
 
 
 
 
 
 
 
 
 
 
 
 
 
 
 
 
 
 
 
 
 
 
 
 
 
 
 
 
 
 
 
 
 
 
 
 
 
 
 
 
 
 
 
 
 
 
 
 
 
 
 
 
 
 
 
 
 
 
 
 
 
 
 
 
 
 
 
 
 
 
 
 
 
 
 
 
 
 
 
 
 
 
 
 
 
 
 
 
 
 
 
 
 
 
 
 
 
 
 
 
 
 
 
 
 
 
 
 
 
 
 
 
 
 
 
 
 
 
 
 
 
 
 
 
 
 
 
 
 
 
 
 
 
 
 
 
 
 
 
 
 
 
 
 
 
 
 
 
 
 
 
 
 
 
 
 
 
 
 
 
 
 
 
 
 
 
 
 
 
 
 
 
 
 
 
 
 
 
 
 
 
 
 
 
 
 
 
 
 
 
 
 
 
 
 
 
 
 
 
 
 
 
 
 
 
 
 
 
 
 
 
 
 
 
 
 
 
 
 
 
 
 
 
 
 
 
 
 
 
 
 
 
 
 
 
 
 
 
 
 
 
 
 
 
 
 
 
 
 
 
 
 
 
 
 
 
 
 
 
 
 
 
 
 
 
 
 
 
 
 
 
 
 
 
 
 
 
 
 
 
 
 
 
 
 
 
 
 
 
 
 
 
 
 
 
 
 
 
 
 
 
 
 
 
 
 
 
 
 
 
 
 
 
 
 
 
 
 
 
 
 
 
 
 
 
 
 
 
 
 
 
 
 
 
 
 
 
 
 
 
 
 
 
 
 
 
 
 
 
 
 
 
 
 
 
 
 
 
 
 
 
 
 
 
 
 
 
 
 
 
 
 
 
 
 
 
 
 
 
 
 
 
 
 
 
 
 
 
 
 
 
 
 
 
 
 
 
 
 
 
 
 
 
 
 
 
 
 
 
 
 
 
 
 
 
 
 
 
 
 
 
 
 
 
 
 
 
 
 
 
 
 
 
 
 
 
 
 
 
 
 
 
 
 
 
 
 
 
 
 
 
 
 
 
 
 
 
 
 
 
 
 
 
 
 
 
 
 
 
 
 
 
 
 
 
 
 
 
 
 
 
 
 
 
 
 
 
 
 
 
 
 
 
 
 
 
 
 
 
 
 
 
 
 
 
 
 
 
 
 
 
 
 
 
 
 
 
 
 
 
 
 
 
 
 
 
 
 
 
 
 
 
 
 
 
 
 
 
 
 
 
 
 
 
 
 
 
 
 
 
 
 
 
 
 
 
 
 
 
 
 
 
 
 
 
 
 
 
 
 
 
 
 
 
 
 
 
 
 
 
 
 
 
 
 
 
 
 
 
 
 
 
 
 
 
 
 
 
 
 
 
 
 
 
 
 
 
 
 
 
 
 
 
 
 
 
 
 
 
 
 
 
 
 
 
 
 
 
 
 
 
 
 
 
 
 
 
 
 
 
 
 
 
 
 
 
 
 
 
 
 
 
 
 
 
 
 
 
 
 
 
 
 
 
 
 
 
 
 
 
 
 
 
 
 
 
 
 
 
 
 
 
 
 
 
 
 
 
 
 
 
 
 
 
 
 
 
 
 
 
 
 
 
 
 
 
 
 
 
 
 
 
 
 
 
 
 
 
 
 
 
 
 
 
 
 
 
 
 
 
 
 
 
 
 
 
 
 
 
 
 
 
 
 
 
 
 
 
 
 
 
 
 
 
 
 
 
 
 
 
 
 
 
 
 
 
 
 
 
 
 
 
 
 
 
 
 
 
 
 
 
 
 
 
 
 
 
 
 
 
 
 
 
 
 
 
 
 
 
 
 
 
 
 
 
 
 
 
 
 
 
 
 
 
 
 
 
 
 
 
 
 
 
 
 
 
 
 
 
 
 
 
 
 
 
 
 
 
 
 
 
 
 
 
 
 
 
 
 
 
 
 
 
 
 
 
 
 
 
 
 
 
 
 
 
 
 
 
 
 
 
 
 
 
 
 
 
 
 
 
 
 
 
 
 
 
 
 
 
 
 
 
 
 
 
 
 
 
 
 
 
 
 
 
 
 
 
 
 
 
 
 
 
 
 
 
 
 
 
 
 
 
 
 
 
 
 
 
 
 
 
 
 
 
 
 
 
 
 
 
 
 
 
 
 
 
 
 
 
 
 
 
 
 
 
 
 
 
 
 
 
 
 
 
 
 
 
 
 
 
 
 
 
 
 
 
 
 
 
 
 
 
 
 
 
 
 
 
 
 
 
 
 
 
 
 
 
 
 
 
 
 
 
 
 
 
 
 
 
 
 
 
 
 
 
 
 
 
 
 
 
 
 
 
 
 
 
 
 
 
 
 
 
 
 
 
 
 
 
 
 
 
 
 
 
 
 
 
 
 
 
 
 
 
 
 
 
 
 
 
 
 
 
 
 
 
 
 
 
 
 
 
 
 
 
 
 
 
 
 
 
 
 
 
 
 
 
 
 
 
 
 
 
 
 
 
 
 
 
 
 
 
 
 
 
 
 
 
 
 
 
 
 
 
 
 
 
 
 
 
 
 
 
 
 
 
 
 
 
 
 
 
 
 
 
 
 
 
 
 
 
 
 
 
 
 
 
 
 
 
 
 
 
 
 
 
 
 
 
 
 
 
 
 
 
 
 
 
 
 
 
 
 
 
 
 
 
 
 
 
 
 
 
 
 
 
 
 
 
 
 
 
 
 
 
 
 
 
 
 
 
 
 
 
 
 
 
 
 
 
 
 
 
 
 
 
 
 
 
 
 
 
 
 
 
 
 
 
 
 
 
 
 
 
 
 
 
 
 
 
 
 
 
 
 
 
 
 
 
 
 
 
 
 
 
 
 
 
 
 
 
 
 
 
 
 
 
 
 
 
 
 
 
 
 
 
 
 
 
 
 
 
 
 
 
 
 
 
 
 
 
 
 
 
 
 
 
 
 
 
 
 
 
 
 
 
 
 
 
 
 
 
 
 
 
 
 
 
 
 
 
 
 
 
 
 
 
 
 
 
 
 
 
 
 
 
 
 
 
 
 
 
 
 
 
 
 
 
 
 
 
 
 
 
 
 
 
 
 
 
 
 
 
 
 
 
 
 
 
 
 
 
 
 
 
 
 
 
 
 
 
 
 
 
 
 
 
 
 
 
 
 
 
 
 
 
 
 
 
 
 
 
 
 
 
 
 
 
 
 
 
 
 
 
 
 
 
 
 
 
 
 
 
 
 
 
 
 
 
 
 
 
 
 
 
 
 
 
 
 
 
 
 
 
 
 
 
 
 
 
 
 
 
 
 
 
 
 
 
 
 
 
 
 
 
 
 
 
 
 
 
 
 
 
 
 
 
 
 
 
 
 
 
 
 
 
 
 
 
 
 
 
 
 
 
 
 
 
 
 
 
 
 
 
 
 
 
 
 
 
 
 
 
 
 
 
 
 
 
 
 
 
 
 
 
 
 
 
 
 
 
 
 
 
 
 
 
 
 
 
 
 
 
 
 
 
 
 
 
 
 
 
 
 
 
 
 
 
 
 
 
 
 
 
 
 
 
 
 
 
 
 
 
 
 
 
 
 
 
 
 
 
 
 
 
 
 
 
 
 
 
 
 
 
 
 
 
 
 
 
 
 
 
 
 
 
 
 
 
 
 
 
 
 
 
 
 
 
 
 
 
 
 
 
 
 
 
 
 
 
 
 
 
 
 
 
 
 
 
 
 
 
 
 
 
 
 
 
 
 
 
 
 
 
 
 
 
 
 
 
 
 
 
 
 
 
 
 
 
 
 
 
 
 
 
 
 
 
 
 
 
 
 
 
 
 
 
 
 
 
 
 
 
 
 
 
 
 
 
 
 
 
 
 
 
 
 
 
 
 
 
 
 
 
 
 
 
 
 
 
 
 
 
 
 
 
 
 
 
 
 
 
 
 
 
 
 
 
 
 
 
 
 
 
 
 
 
 
 
 
 
 
 
 
 
 
 
 
 
 
 
 
 
 
 
 
 
 
 
 
 
 
 
 
 
 
 
 
 
 
 
 
 
 
 
 
 
 
 
 
 
 
 
 
 
 
 
 
 
 
 
 
 
 
 
 
 
 
 
 
 
 
 
 
 
 
 
 
 
 
 
 
 
 
 
 
 
 
 
 
 
 
 
 
 
 
 
 
 
 
 
 
 
 
 
 
 
 
 
 
 
 
 
 
 
 
 
 
 
 
 
 
 
 
 
 
 
 
 
 
 
 
 
 
 
 
 
 
 
 
 
 
 
 
 
 
 
 
 
 
 
 
 
 
 
 
 
 
 
 
 
 
 
 
 
 
 
 
 
 
 
 
 
 
 
 
 
 
 
 
 
 
 
 
 
 
 
 
 
 
 
 
 
 
 
 
 
 
 
 
 
 
 
 
 
 
 
 
 
 
 
 
 
 
 
 
 
 
 
 
 
 
 
 
 
 
 
 
 
 
 
 
 
 
 
 
 
 
 
 
 
 
 
 
 
 
 
 
 
 
 
 
 
 
 
 
 
 
 
 
 
 
 
 
 
 
 
 
 
 
 
 
 
 
 
 
 
 
 
 
 
 
 
 
 
 
 
 
 
 
 
 
 
 
 
 
 
 
 
 
 
 
 
 
 
 
 
 
 
 
 
 
 
 
 
 
 
 
 
 
 
 
 
 
 
 
 
 
 
 
 
 
 
 
 
 
 
 
 
 
 
 
 
 
 
 
 
 
 
 
 
 
 
 
 
 
 
 
 
 
 
 
 
 
 
 
 
 
 
 
 
 
 
 
 
 
 
 
 
 
 
 
 
 
 
 
 
 
 
 
 
 
 
 
 
 
 
 
 
 
 
 
 
 
 
 
 
 
 
 
 
 
 
 
 
 
 
 
 
 
 
 
 
 
 
 
 
 
 
 
 
 
 
 
 
 
 
 
 
 
 
 
 
 
 
 
 
 
 
 
 
 
 
 
 
 
 
 
 
 
 
 
 
 
 
 
 
 
 
 
 
 
 
 
 
 
 
 
 
 
 
 
 
 
 
 
 
 
 
 
 
 
 
 
 
 
 
 
 
 
 
 
 
 
 
 
 
 
 
 
 
 
 
 
 
 
 
 
 
 
 
 
 
 
 
 
 
 
 
 
 
 
 
 
 
 
 
 
 
 
 
 
 
 
 
 
 
 
 
 
 
 
 
 
 
 
 
 
 
 
 
 
 
 
 
 
 
 
 
 
 
 
 
 
 
 
 
 
 
 
 
 
 
 
 
 
 
 
 
 
 
 
 
 
 
 
 
 
 
 
 
 
 
 
 
 
 
 
 
 
 
 
 
 
 
 
 
 
 
 
 
 
 
 
 
 
 
 
 
 
 
 
 
 
 
 
 
 
 
 
 
 
 
 
 
 
 
 
 
 
 
 
 
 
 
 
 
 
 
 
 
 
 
 
 
 
 
 
 
 
 
 
 
 
 
 
 
 
 
 
 
 
 
 
 
 
 
 
 
 
 
 
 
 
 
 
 
 
 
 
 
 
 
 
 
 
 
 
 
 
 
 
 
 
 
 
 
 
 
 
 
 
 
 
 
 
 
 
 
 
 
 
 
 
 
 
 
 
 
 
 
 
 
 
 
 
 
 
 
 
 
 
 
 
 
 
 
 
 
 
 
 
 
 
 
 
 
 
 
 
 
 
 
 
 
 
 
 
 
 
 
 
 
 
 
 
 
 
 
 
 
 
 
 
 
 
 
 
 
 
 
 
 
 
 
 
 
 
 
 
 
 
 
 
 
 
 
 
 
 
 
 
 
 
 
 
 
 
 
 
 
 
 
 
 
 
 
 
 
 
 
 
 
 
 
 
 
 
 
 
 
 
 
 
 
 
 
 
 
 
 
 
 
 
 
 
 
 
 
 
 
 
 
 
 
 
 
 
 
 
 
 
 
 
 
 
 
 
 
 
 
 
 
 
 
 
 
 
 
 
 
 
 
 
 
 
 
 
 
 
 
 
 
 
 
 
 
 
 
 
 
 
 
 
 
 
 
 
 
 
 
 
 
 
 
 
 
 
 
 
 
 
 
 
 
 
 
 
 
 
 
 
 
 
 
 
 
 
 
 
 
 
 
 
 
 
 
 
 
 
 
 
 
 
 
 
 
 
 
 
 
 
 
 
 
 
 
 
 
 
 
 
 
 
 
 
 
 
 
 
 
 
 
 
 
 
 
 
 
 
 
 
 
 
 
 
 
 
 
 
 
 
 
 
 
 
 
 
 
 
 
 
 
 
 
 
 
 
 
 
 
 
 
 
 
 
 
 
 
 
 
 
 
 
 
 
 
 
 
 
 
 
 
 
 
 
 
 
 
 
 
 
 
 
 
 
 
 
 
 
 
 
 
 
 
 
 
 
 
 
 
 
 
 
 
 
 
 
 
 
 
 
 
 
 
 
 
 
 
 
 
 
 
 
 
 
 
 
 
 
 
 
 
 
 
 
 
 
 
 
 
 
 
 
 
 
 
 
 
 
 
 
 
 
 
 
 
 
 
 
 
 
 
 
 
 
 
 
 
 
 
 
 
 
 
 
 
 
 
 
 
 
 
 
 
 
 
 
 
 
 
 
 
 
 
 
 
 
 
 
 
 
 
 
 
 
 
 
 
 
 
 
 
 
 
 
 
 
 
 
 
 
 
 
 
 
 
 
 
 
 
 
 
 
 
 
 
 
 
 
 
 
 
 
 
 
 
 
 
 
 
 
 
 
 
 
 
 
 
 
 
 
 
 
 
 
 
 
 
 
 
 
 
 
 
 
 
 
 
 
 
 
 
 
 
 
 
 
 
 
 
 
 
 
 
 
 
 
 
 
 
 
 
 
 
 
 
 
 
 
 
 
 
 
 
 
 
 
 
 
 
 
 
 
 
 
 
 
 
 
 
 
 
 
 
 
 
 
 
 
 
 
 
 
 
 
 
 
 
 
 
 
 
 
 
 
 
 
 
 
 
 
 
 
 
 
 
 
 
 
 
 
 
 
 
 
 
 
 
 
 
 
 
 
 
 
 
 
 
 
 
 
 
 
 
 
 
 
 
 
 
 
 
 
 
 
 
 
 
 
 
 
 
 
 
 
 
 
 
 
 
 
 
 
 
 
 
 
 
 
 
 
 
 
 
 
 
 
 
 
 
 
 
 
 
 
 
 
 
 
 
 
 
 
 
 
 
 
 
 
 
 
 
 
 
 
 
 
 
 
 
 
 
 
 
 
 
 
 
 
 
 
 
 
 
 
 
 
 
 
 
 
 
 
 
 
 
 
 
 
 
 
 
 
 
 
 
 
 
 
 
 
 
 
 
 
 
 
 
 
 
 
 
 
 
 
 
 
 
 
 
 
 
 
 
 
 
 
 
 
 
 
 
 
 
 
 
 
 
 
 
 
 
 
 
 
 
 
 
 
 
 
 
 
 
 
 
 
 
 
 
 
 
 
 
 
 
 
 
 
 
 
 
 
 
 
 
 
 
 
 
 
 
 
 
 
 
 
 
 
 
 
 
 
 
 
 
 
 
 
 
 
 
 
 
 
 
 
 
 
 
 
 
 
 
 
 
 
 
 
 
 
 
 
 
 
 
 
 
 
 
 
 
 
 
 
 
 
 
 
 
 
 
 
 
 
 
 
 
 
 
 
 
 
 
 
 
 
 
 
 
 
 
 
 
 
 
 
 
 
 
 
 
 
 
 
 
 
 
 
 
 
 
 
 
 
 
 
 
 
 
 
 
 
 
 
 
 
 
 
 
 
 
 
 
 
 
 
 
 
 
 
 
 
 
 
 
 
 
 
 
 
 
 
 
 
 
 
 
 
 
 
 
 
 
 
 
 
 
 
 
 
 
 
 
 
 
 
 
 
 
 
 
 
 
 
 
 
 
 
 
 
 
 
 
 
 
 
 
 
 
 
 
 
 
 
 
 
 
 
 
 
 
 
 
 
 
 
 
 
 
 
 
 
 
 
 
 
 
 
 
 
 
 
 
 
 
 
 
 
 
 
 
 
 
 
 
 
 
 
 
 
 
 
 
 
 
 
 
 
 
 
 
 
 
 
 
 
 
 
 
 
 
 
 
 
 
 
 
 
 
 
 
 
 
 
 
 
 
 
 
 
 
 
 
 
 
 
 
 
 
 
 
 
 
 
 
 
 
 
 
 
 
 
 
 
 
 
 
 
 
 
 
 
 
 
 
 
 
 
 
 
 
 
 
 
 
 
 
 
 
 
 
 
 
 
 
 
 
 
 
 
 
 
 
 
 
 
 
 
 
 
 
 
 
 
 
 
 
 
 
 
 
 
 
 
 
 
 
 
 
 
 
 
 
 
 
 
 
 
 
 
 
 
 
 
 
 
 
 
 
 
 
 
 
 
 
 
 
 
 
 
 
 
 
 
 
 
 
 
 
 
 
 
 
 
 
 
 
 
 
 
 
 
 
 
 
 
 
 
 
 
 
 
 
 
 
 
 
 
 
 
 
 
 
 
 
 
 
 
 
 
 
 
 
 
 
 
 
 
 
 
 
 
 
 
 
 
 
 
 
 
 
 
 
 
 
 
 
 
 
 
 
 
 
 
 
 
 
 
 
 
 
 
 
 
 
 
 
 
 
 
 
 
 
 
 
 
 
 
 
 
 
 
 
 
 
 
 
 
 
 
 
 
 
 
 
 
 
 
 
 
 
 
 
 
 
 
 
 
 
 
 
 
 
 
 
 
 
 
 
 
 
 
 
 
 
 
 
 
 
 
 
 
 
 
 
 
 
 
 
 
 
 
 
 
 
 
 
 
 
 
 
 
 
 
 
 
 
 
 
 
 
 
 
 
 
 
 
 
 
 
 
 
 
 
 
 
 
 
 
 
 
 
 
 
 
 
 
 
 
 
 
 
 
 
 
 
 
 
 
 
 
 
 
 
 
 
 
 
 
 
 
 
 
 
 
 
 
 
 
 
 
 
 
 
 
 
 
 
 
 
 
 
 
 
 
 
 
 
 
 
 
 
 
 
 
 
 
 
 
 
 
 
 
 
 
 
 
 
 
 
 
 
 
 
 
 
 
 
 
 
 
 
 
 
 
 
 
 
 
 
 
 
 
 
 
 
 
 
 
 
 
 
 
 
 
 
 
 
 
 
 
 
 
 
 
 
 
 
 
 
 
 
 
 
 
 
 
 
 
 
 
 
 
 
 
 
 
 
 
 
 
 
 
 
 
 
 
 
 
 
 
 
 
 
 
 
 
 
 
 
 
 
 
 
 
 
 
 
 
 
 
 
 
 
 
 
 
 
 
 
 
 
 
 
 
 
 
 
 
 
 
 
 
 
 
 
 
 
 
 
 
 
 
 
 
 
 
 
 
 
 
 
 
 
 
 
 
 
 
 
 
 
 
 
 
 
 
 
 
 
 
 
 
 
 
 
 
 
 
 
 
 
 
 
 
 
 
 
 
 
 
 
 
 
 
 
 
 
 
 
 
 
 
 
 
 
 
 
 
 
 
 
 
 
 
 
 
 
 
 
 
 
 
 
 
 
 
 
 
 
 
 
 
 
 
 
 
 
 
 
 
 
 
 
 
 
 
 
 
 
 
 
 
 
 
 
 
 
 
 
 
 
 
 
 
 
 
 
 
 
 
 
 
 
 
 
 
 
 
 
 
 
 
 
 
 
 
 
 
 
 
 
 
 
 
 
 
 
 
 
 
 
 
 
 
 
 
 
 
 
 
 
 
 
 
 
 
 
 
 
 
 
 
 
 
 
 
 
 
 
 
 
 
 
 
 
 
 
 
 
 
 
 
 
 
 
 
 
 
 
 
 
 
 
 
 
 
 
 
 
 
 
 
 
 
 
 
 
 
 
 
 
 
 
 
 
 
 
 
 
 
 
 
 
 
 
 
 
 
 
 
 
 
 
 
 
 
 
 
 
 
 
 
 
 
 
 
 
 
 
 
 
 
 
 
 
 
 
 
 
 
 
 
 
 
 
 
 
 
 
 
 
 
 
 
 
 
 
 
 
 
 
 
 
 
 
 
 
 
 
 
 
 
 
 
 
 
 
 
 
 
 
 
 
 
 
 
 
 
 
 
 
 
 
 
 
 
 
 
 
 
 
 
 
 
 
 
 
 
 
 
 
 
 
 
 
 
 
 
 
 
 
 
 
 
 
 
 
 
 
 
 
 
 
 
 
 
 
 
 
 
 
 
 
 
 
 
 
 
 
 
 
 
 
 
 
 
 
 
 
 
 
 
 
 
 
 
 
 
 
 
 
 
 
 
 
 
 
 
 
 
 
 
 
 
 
 
 
 
 
 
 
 
 
 
 
 
 
 
 
 
 
 
 
 
 
 
 
 
 
 
 
 
 
 
 
 
 
 
 
 
 
 
 
 
 
 
 
 
 
 
 
 
 
 
 
 
 
 
 
 
 
 
 
 
 
 
 
 
 
 
 
 
 
 
 
 
 
 
 
 
 
 
 
 
 
 
 
 
 
 
 
 
 
 
 
 
 
 
 
 
 
 
 
 
 
 
 
 
 
 
 
 
 
 
 
 
 
 
 
 
 
 
 
 
 
 
 
 
 
 
 
 
 
 
 
 
 
 
 
 
 
 
 
 
 
 
 
 
 
 
 
 
 
 
 
 
 
 
 
 
 
 
 
 
 
 
 
 
 
 
 
 
 
 
 
 
 
 
 
 
 
 
 
 
 
 
 
 
 
 
 
 
 
 
 
 
 
 
 
 
 
 
 
 
 
 
 
 
 
 
 
 
 
 
 
 
 
 
 
 
 
 
 
 
 
 
 
 
 
 
 
 
 
 
 
 
 
 
 
 
 
 
 
 
 
 
 
 
 
 
 
 
 
 
 
 
 
 
 
 
 
 
 
 
 
 
 
 
 
 
 
 
 
 
 
 
 
 
 
 
 
 
 
 
 
 
 
 
 
 
 
 
 
 
 
 
 
 
 
 
 
 
 
 
 
 
 
 
 
 
 
 
 
 
 
 
 
 
 
 
 
 
 
 
 
 
 
 
 
 
 
 
 
 
 
 
 
 
 
 
 
 
 
 
 
 
 
 
 
 
 
 
 
 
 
 
 
 
 
 
 
 
 
 
 
 
 
 
 
 
 
 
 
 
 
 
 
 
 
 
 
 
 
 
 
 
 
 
 
 
 
 
 
 
 
 
 
 
 
 
 
 
 
 
 
 
 
 
 
 
 
 
 
 
 
 
 
 
 
 
 
 
 
 
 
 
 
 
 
 
 
 
 
 
 
 
 
 
 
 
 
 
 
 
 
 
 
 
 

 

    View Table (.txt)         Total  0  1  2  3  4  5  6  8  9  10  11  12  13  14  15  16  17  18  19  20  21  22  23  24  25  26  27  28  29  30  31  42  43  44  45  46  47  48  49  50  51  53  54  55  56  57  58  59  60  61  62  71  72  81  82  83  84  91  92  93  95  96  97  98  99  100  101  102  103  104  112  113  114  115  116  117  118  119  120  122  123  124  125  126  127  128  129  130  131  132  133  134  135  136  137  138  139  140  141  142  143  144  145  146  147  148  149  151  152  153  155  157  158  159  160  161  166  170  171  172  173  174  175  176  177  178  179  180  181  182  183  184  185    Legend  Taxonomy  count  %  %  %  %  %  %  %  %  %  %  %  %  %  %  %  %  %  %  %  %  %  %  %  %  %  %  %  %  %  %  %  %  %  %  %  %  %  %  %  %  %  %  %  %  %  %  %  %  %  %  %  %  %  %  %  %  %  %  %  %  %  %  %  %  %  %  %  %  %  %  %  %  %  %  %  %  %  %  %  %  %  %  %  %  %  %  %  %  %  %  %  %  %  %  %  %  %  %  %  %  %  %  %  %  %  %  %  %  %  %  %  %  %  %  %  %  %  %  %  %  %  %  %  %  %  %  %  %  %  %  %  %  %  %        k__Archaea;p__Crenarchaeota; c__Sd-NA       0    0.0%    0.0%    0.0%    0.0%    0.0%    0.0%    0.0%    0.0%    0.0%    0.0%    0.0%    0.0%    0.0%    0.0%    0.0%    0.0%    0.0%    0.0%    0.0%    0.0%    0.0%    0.0%    0.0%    0.0%    0.0%    0.0%    0.0%    0.0%    0.0%    0.0%    0.0%    0.0%    0.0%    0.0%    0.0%    0.0%    0.0%    0.0%    0.0%    0.0%    0.0%    0.0%    0.0%    0.0%    0.0%    0.0%    0.0%    0.0%    0.0%    0.0%    0.0%    0.0%    0.0%    0.0%    0.0%    0.0%    0.0%    0.0%    0.0%    0.0%    0.0%    0.0%    0.0%    0.0%    0.0%    0.0%    0.0%    0.0%    0.0%    0.0%    0.0%    0.0%    0.0%    0.0%    0.0%    0.0%    0.0%    0.0%    0.0%    0.0%    0.0%    0.0%    0.0%    0.0%    0.0%    0.0%    0.0%    0.0%    0.0%    0.0%    0.0%    0.0%    0.0%    0.0%    0.0%    0.0%    0.0%    0.0%    0.0%    0.0%    0.0%    0.0%    0.0%    0.0%    0.0%    0.0%    0.0%    0.0%    0.0%    0.0%    0.0%    0.0%    0.0%    0.0%    0.0%    0.0%    0.0%    0.0%    0.0%    0.0%    0.0%    0.0%    0.0%    0.0%    0.0%    0.0%    0.0%    0.0%    0.0%    0.0%    0.0%    0.0%    0.0%    0.0%        k__Archaea;p__Crenarchaeota; c__Thaumarchaeota    3113    0.1%    0.0%    0.1%    0.0%    0.0%    0.1%    0.0%    0.1%    0.0%    0.0%    0.1%    0.0%    0.0%    0.0%    0.0%    0.0%    0.1%    0.0%    0.1%    0.0%    0.0%    0.0%    0.0%    0.0%    0.0%    0.0%    0.1%    0.0%    0.0%    0.0%    0.0%    0.0%    0.0%    0.0%    0.0%    0.0%    0.0%    0.0%    0.1%    0.1%    0.0%    0.0%    0.0%    0.0%    0.0%    0.0%    0.0%    0.0%    0.0%    0.0%    0.0%    0.0%    0.0%    0.1%    0.0%    0.1%    0.1%    0.0%    0.0%    0.1%    0.1%    0.1%    0.1%    0.0%    0.1%    0.0%    0.1%    0.0%    0.5%    0.2%    0.1%    0.1%    0.0%    0.1%    0.1%    0.1%    0.2%    0.0%    0.0%    0.3%    0.1%    0.0%    0.3%    0.0%    0.1%    0.1%    0.1%    0.1%    0.1%    0.1%    0.1%    0.1%    0.1%    0.0%    0.1%    0.1%    0.1%    0.1%    0.2%    0.0%    0.1%    0.1%    0.1%    0.1%    0.1%    0.1%    0.1%    0.0%    0.3%    0.1%    0.3%    0.2%    0.1%    0.1%    0.0%    0.1%    0.1%    0.4%    0.2%    0.1%    0.1%    0.1%    0.0%    0.1%    0.1%    0.3%    0.0%    0.0%    0.1%    0.1%    0.1%    0.1%    0.1%    0.1%        k__Archaea;p__Euryarchaeota; c__Halobacteria       5    0.0%    0.0%    0.0%    0.0%    0.0%    0.0%    0.0%    0.0%    0.0%    0.0%    0.0%    0.0%    0.0%    0.0%    0.0%    0.0%    0.0%    0.0%    0.0%    0.0%    0.0%    0.0%    0.0%    0.0%    0.0%    0.0%    0.0%    0.0%    0.0%    0.0%    0.0%    0.0%    0.0%    0.0%    0.0%    0.0%    0.0%    0.0%    0.0%    0.0%    0.0%    0.0%    0.0%    0.0%    0.0%    0.0%    0.0%    0.0%    0.0%    0.0%    0.0%    0.0%    0.0%    0.0%    0.0%    0.0%    0.0%    0.0%    0.0%    0.0%    0.0%    0.0%    0.0%    0.0%    0.0%    0.0%    0.0%    0.0%    0.0%    0.0%    0.0%    0.0%    0.0%    0.0%    0.0%    0.0%    0.0%    0.0%    0.0%    0.0%    0.0%    0.0%    0.0%    0.0%    0.0%    0.0%    0.0%    0.0%    0.0%    0.0%    0.0%    0.0%    0.0%    0.0%    0.0%    0.0%    0.0%    0.0%    0.0%    0.0%    0.0%    0.0%    0.0%    0.0%    0.0%    0.0%    0.0%    0.0%    0.0%    0.0%    0.0%    0.0%    0.0%    0.0%    0.0%    0.0%    0.0%    0.0%    0.0%    0.0%    0.0%    0.0%    0.0%    0.0%    0.0%    0.0%    0.0%    0.0%    0.0%    0.0%    0.0%    0.0%    0.0%    0.0%        k__Archaea;p__Euryarchaeota; c__Methanobacteria      26    0.0%    0.0%    0.0%    0.0%    0.0%    0.0%    0.0%    0.0%    0.0%    0.0%    0.0%    0.0%    0.0%    0.0%    0.0%    0.0%    0.0%    0.0%    0.0%    0.0%    0.0%    0.0%    0.0%    0.0%    0.0%    0.0%    0.0%    0.0%    0.0%    0.0%    0.0%    0.0%    0.0%    0.0%    0.0%    0.0%    0.0%    0.0%    0.0%    0.0%    0.0%    0.0%    0.0%    0.0%    0.0%    0.0%    0.0%    0.0%    0.0%    0.0%    0.0%    0.0%    0.0%    0.0%    0.0%    0.0%    0.0%    0.0%    0.0%    0.0%    0.0%    0.0%    0.0%    0.0%    0.0%    0.0%    0.0%    0.0%    0.0%    0.0%    0.0%    0.0%    0.0%    0.0%    0.0%    0.0%    0.0%    0.0%    0.0%    0.0%    0.0%    0.0%    0.0%    0.0%    0.0%    0.0%    0.0%    0.0%    0.0%    0.0%    0.0%    0.0%    0.0%    0.0%    0.0%    0.0%    0.0%    0.0%    0.0%    0.0%    0.0%    0.0%    0.0%    0.0%    0.0%    0.0%    0.0%    0.0%    0.0%    0.0%    0.0%    0.0%    0.0%    0.0%    0.0%    0.0%    0.0%    0.0%    0.0%    0.0%    0.0%    0.0%    0.0%    0.0%    0.0%    0.0%    0.0%    0.0%    0.0%    0.0%    0.0%    0.0%    0.0%    0.0%        k__Archaea;p__Euryarchaeota; c__Methanomicrobia       6    0.0%    0.0%    0.0%    0.0%    0.0%    0.0%    0.0%    0.0%    0.0%    0.0%    0.0%    0.0%    0.0%    0.0%    0.0%    0.0%    0.0%    0.0%    0.0%    0.0%    0.0%    0.0%    0.0%    0.0%    0.0%    0.0%    0.0%    0.0%    0.0%    0.0%    0.0%    0.0%    0.0%    0.0%    0.0%    0.0%    0.0%    0.0%    0.0%    0.0%    0.0%    0.0%    0.0%    0.0%    0.0%    0.0%    0.0%    0.0%    0.0%    0.0%    0.0%    0.0%    0.0%    0.0%    0.0%    0.0%    0.0%    0.0%    0.0%    0.0%    0.0%    0.0%    0.0%    0.0%    0.0%    0.0%    0.0%    0.0%    0.0%    0.0%    0.0%    0.0%    0.0%    0.0%    0.0%    0.0%    0.0%    0.0%    0.0%    0.0%    0.0%    0.0%    0.0%    0.0%    0.0%    0.0%    0.0%    0.0%    0.0%    0.0%    0.0%    0.0%    0.0%    0.0%    0.0%    0.0%    0.0%    0.0%    0.0%    0.0%    0.0%    0.0%    0.0%    0.0%    0.0%    0.0%    0.0%    0.0%    0.0%    0.0%    0.0%    0.0%    0.0%    0.0%    0.0%    0.0%    0.0%    0.0%    0.0%    0.0%    0.0%    0.0%    0.0%    0.0%    0.0%    0.0%    0.0%    0.0%    0.0%    0.0%    0.0%    0.0%    0.0%    0.0%        k__Archaea;p__Euryarchaeota; c__Thermoplasmata       3    0.0%    0.0%    0.0%    0.0%    0.0%    0.0%    0.0%    0.0%    0.0%    0.0%    0.0%    0.0%    0.0%    0.0%    0.0%    0.0%    0.0%    0.0%    0.0%    0.0%    0.0%    0.0%    0.0%    0.0%    0.0%    0.0%    0.0%    0.0%    0.0%    0.0%    0.0%    0.0%    0.0%    0.0%    0.0%    0.0%    0.0%    0.0%    0.0%    0.0%    0.0%    0.0%    0.0%    0.0%    0.0%    0.0%    0.0%    0.0%    0.0%    0.0%    0.0%    0.0%    0.0%    0.0%    0.0%    0.0%    0.0%    0.0%    0.0%    0.0%    0.0%    0.0%    0.0%    0.0%    0.0%    0.0%    0.0%    0.0%    0.0%    0.0%    0.0%    0.0%    0.0%    0.0%    0.0%    0.0%    0.0%    0.0%    0.0%    0.0%    0.0%    0.0%    0.0%    0.0%    0.0%    0.0%    0.0%    0.0%    0.0%    0.0%    0.0%    0.0%    0.0%    0.0%    0.0%    0.0%    0.0%    0.0%    0.0%    0.0%    0.0%    0.0%    0.0%    0.0%    0.0%    0.0%    0.0%    0.0%    0.0%    0.0%    0.0%    0.0%    0.0%    0.0%    0.0%    0.0%    0.0%    0.0%    0.0%    0.0%    0.0%    0.0%    0.0%    0.0%    0.0%    0.0%    0.0%    0.0%    0.0%    0.0%    0.0%    0.0%    0.0%    0.0%        k__Bacteria;p__; c__       3    0.0%    0.0%    0.0%    0.0%    0.0%    0.0%    0.0%    0.0%    0.0%    0.0%    0.0%    0.0%    0.0%    0.0%    0.0%    0.0%    0.0%    0.0%    0.0%    0.0%    0.0%    0.0%    0.0%    0.0%    0.0%    0.0%    0.0%    0.0%    0.0%    0.0%    0.0%    0.0%    0.0%    0.0%    0.0%    0.0%    0.0%    0.0%    0.0%    0.0%    0.0%    0.0%    0.0%    0.0%    0.0%    0.0%    0.0%    0.0%    0.0%    0.0%    0.0%    0.0%    0.0%    0.0%    0.0%    0.0%    0.0%    0.0%    0.0%    0.0%    0.0%    0.0%    0.0%    0.0%    0.0%    0.0%    0.0%    0.0%    0.0%    0.0%    0.0%    0.0%    0.0%    0.0%    0.0%    0.0%    0.0%    0.0%    0.0%    0.0%    0.0%    0.0%    0.0%    0.0%    0.0%    0.0%    0.0%    0.0%    0.0%    0.0%    0.0%    0.0%    0.0%    0.0%    0.0%    0.0%    0.0%    0.0%    0.0%    0.0%    0.0%    0.0%    0.0%    0.0%    0.0%    0.0%    0.0%    0.0%    0.0%    0.0%    0.0%    0.0%    0.0%    0.0%    0.0%    0.0%    0.0%    0.0%    0.0%    0.0%    0.0%    0.0%    0.0%    0.0%    0.0%    0.0%    0.0%    0.0%    0.0%    0.0%    0.0%    0.0%    0.0%    0.0%        k__Bacteria;p__ABY1_OD1; c__       1    0.0%    0.0%    0.0%    0.0%    0.0%    0.0%    0.0%    0.0%    0.0%    0.0%    0.0%    0.0%    0.0%    0.0%    0.0%    0.0%    0.0%    0.0%    0.0%    0.0%    0.0%    0.0%    0.0%    0.0%    0.0%    0.0%    0.0%    0.0%    0.0%    0.0%    0.0%    0.0%    0.0%    0.0%    0.0%    0.0%    0.0%    0.0%    0.0%    0.0%    0.0%    0.0%    0.0%    0.0%    0.0%    0.0%    0.0%    0.0%    0.0%    0.0%    0.0%    0.0%    0.0%    0.0%    0.0%    0.0%    0.0%    0.0%    0.0%    0.0%    0.0%    0.0%    0.0%    0.0%    0.0%    0.0%    0.0%    0.0%    0.0%    0.0%    0.0%    0.0%    0.0%    0.0%    0.0%    0.0%    0.0%    0.0%    0.0%    0.0%    0.0%    0.0%    0.0%    0.0%    0.0%    0.0%    0.0%    0.0%    0.0%    0.0%    0.0%    0.0%    0.0%    0.0%    0.0%    0.0%    0.0%    0.0%    0.0%    0.0%    0.0%    0.0%    0.0%    0.0%    0.0%    0.0%    0.0%    0.0%    0.0%    0.0%    0.0%    0.0%    0.0%    0.0%    0.0%    0.0%    0.0%    0.0%    0.0%    0.0%    0.0%    0.0%    0.0%    0.0%    0.0%    0.0%    0.0%    0.0%    0.0%    0.0%    0.0%    0.0%    0.0%    0.0%        k__Bacteria;p__AD3; c__ABS-6       0    0.0%    0.0%    0.0%    0.0%    0.0%    0.0%    0.0%    0.0%    0.0%    0.0%    0.0%    0.0%    0.0%    0.0%    0.0%    0.0%    0.0%    0.0%    0.0%    0.0%    0.0%    0.0%    0.0%    0.0%    0.0%    0.0%    0.0%    0.0%    0.0%    0.0%    0.0%    0.0%    0.0%    0.0%    0.0%    0.0%    0.0%    0.0%    0.0%    0.0%    0.0%    0.0%    0.0%    0.0%    0.0%    0.0%    0.0%    0.0%    0.0%    0.0%    0.0%    0.0%    0.0%    0.0%    0.0%    0.0%    0.0%    0.0%    0.0%    0.0%    0.0%    0.0%    0.0%    0.0%    0.0%    0.0%    0.0%    0.0%    0.0%    0.0%    0.0%    0.0%    0.0%    0.0%    0.0%    0.0%    0.0%    0.0%    0.0%    0.0%    0.0%    0.0%    0.0%    0.0%    0.0%    0.0%    0.0%    0.0%    0.0%    0.0%    0.0%    0.0%    0.0%    0.0%    0.0%    0.0%    0.0%    0.0%    0.0%    0.0%    0.0%    0.0%    0.0%    0.0%    0.0%    0.0%    0.0%    0.0%    0.0%    0.0%    0.0%    0.0%    0.0%    0.0%    0.0%    0.0%    0.0%    0.0%    0.0%    0.0%    0.0%    0.0%    0.0%    0.0%    0.0%    0.0%    0.0%    0.0%    0.0%    0.0%    0.0%    0.0%    0.0%    0.0%        k__Bacteria;p__AD3; c__JG37-AG-4       0    0.0%    0.0%    0.0%    0.0%    0.0%    0.0%    0.0%    0.0%    0.0%    0.0%    0.0%    0.0%    0.0%    0.0%    0.0%    0.0%    0.0%    0.0%    0.0%    0.0%    0.0%    0.0%    0.0%    0.0%    0.0%    0.0%    0.0%    0.0%    0.0%    0.0%    0.0%    0.0%    0.0%    0.0%    0.0%    0.0%    0.0%    0.0%    0.0%    0.0%    0.0%    0.0%    0.0%    0.0%    0.0%    0.0%    0.0%    0.0%    0.0%    0.0%    0.0%    0.0%    0.0%    0.0%    0.0%    0.0%    0.0%    0.0%    0.0%    0.0%    0.0%    0.0%    0.0%    0.0%    0.0%    0.0%    0.0%    0.0%    0.0%    0.0%    0.0%    0.0%    0.0%    0.0%    0.0%    0.0%    0.0%    0.0%    0.0%    0.0%    0.0%    0.0%    0.0%    0.0%    0.0%    0.0%    0.0%    0.0%    0.0%    0.0%    0.0%    0.0%    0.0%    0.0%    0.0%    0.0%    0.0%    0.0%    0.0%    0.0%    0.0%    0.0%    0.0%    0.0%    0.0%    0.0%    0.0%    0.0%    0.0%    0.0%    0.0%    0.0%    0.0%    0.0%    0.0%    0.0%    0.0%    0.0%    0.0%    0.0%    0.0%    0.0%    0.0%    0.0%    0.0%    0.0%    0.0%    0.0%    0.0%    0.0%    0.0%    0.0%    0.0%    0.0%        k__Bacteria;p__Acidobacteria; c__     190    0.0%    0.0%    0.0%    0.0%    0.0%    0.0%    0.0%    0.0%    0.0%    0.0%    0.0%    0.0%    0.0%    0.0%    0.0%    0.0%    0.0%    0.0%    0.0%    0.0%    0.0%    0.0%    0.0%    0.0%    0.0%    0.0%    0.0%    0.0%    0.0%    0.0%    0.0%    0.0%    0.0%    0.0%    0.0%    0.0%    0.0%    0.0%    0.0%    0.0%    0.0%    0.0%    0.0%    0.0%    0.0%    0.0%    0.0%    0.0%    0.0%    0.0%    0.0%    0.0%    0.0%    0.0%    0.0%    0.0%    0.0%    0.0%    0.0%    0.0%    0.0%    0.0%    0.0%    0.0%    0.0%    0.0%    0.0%    0.0%    0.0%    0.0%    0.0%    0.0%    0.0%    0.0%    0.0%    0.0%    0.0%    0.0%    0.0%    0.0%    0.0%    0.0%    0.0%    0.0%    0.0%    0.0%    0.0%    0.0%    0.0%    0.0%    0.0%    0.0%    0.0%    0.0%    0.0%    0.0%    0.0%    0.0%    0.0%    0.0%    0.0%    0.0%    0.0%    0.0%    0.0%    0.0%    0.0%    0.0%    0.0%    0.0%    0.0%    0.0%    0.0%    0.0%    0.0%    0.0%    0.0%    0.0%    0.0%    0.0%    0.0%    0.0%    0.0%    0.0%    0.0%    0.0%    0.0%    0.0%    0.0%    0.0%    0.0%    0.0%    0.0%    0.0%        k__Bacteria;p__Acidobacteria; c__Acidobacteria (class)   11314    0.3%    0.2%    0.3%    0.2%    0.1%    0.7%    0.3%    0.4%    0.1%    0.1%    0.3%    0.2%    0.0%    0.1%    0.1%    0.0%    0.2%    0.0%    0.2%    0.1%    0.2%    0.1%    0.1%    0.1%    0.1%    0.1%    0.3%    0.1%    0.2%    0.1%    0.0%    0.1%    0.1%    0.1%    0.2%    0.1%    0.0%    0.2%    0.2%    0.2%    0.1%    0.0%    0.4%    0.2%    0.1%    0.1%    0.4%    0.1%    0.1%    0.1%    0.1%    0.0%    0.1%    0.4%    0.2%    0.4%    0.4%    0.2%    0.1%    0.5%    0.5%    0.7%    0.2%    0.1%    0.1%    0.1%    0.3%    0.2%    1.9%    0.4%    0.2%    0.4%    0.1%    0.5%    0.4%    0.4%    0.6%    0.1%    0.0%    1.1%    0.3%    0.1%    0.9%    0.1%    0.2%    0.2%    0.3%    0.4%    0.4%    0.2%    0.3%    0.3%    0.2%    0.0%    0.2%    0.2%    0.2%    0.2%    0.6%    0.2%    0.2%    0.3%    0.4%    0.3%    0.2%    0.1%    0.0%    0.2%    1.1%    0.3%    0.7%    0.8%    0.4%    0.2%    0.1%    0.3%    0.2%    1.0%    0.4%    0.2%    0.2%    0.6%    0.1%    0.2%    0.1%    0.9%    0.3%    0.3%    0.3%    0.3%    0.4%    0.2%    0.1%    0.2%        k__Bacteria;p__Acidobacteria; c__Acidobacteria-5     158    0.0%    0.0%    0.0%    0.0%    0.0%    0.0%    0.0%    0.0%    0.0%    0.0%    0.0%    0.0%    0.0%    0.0%    0.0%    0.0%    0.0%    0.0%    0.0%    0.0%    0.0%    0.0%    0.0%    0.0%    0.0%    0.0%    0.0%    0.0%    0.0%    0.0%    0.0%    0.0%    0.0%    0.0%    0.0%    0.0%    0.0%    0.0%    0.0%    0.0%    0.0%    0.0%    0.0%    0.0%    0.0%    0.0%    0.0%    0.0%    0.0%    0.0%    0.0%    0.0%    0.0%    0.0%    0.0%    0.0%    0.0%    0.0%    0.0%    0.0%    0.0%    0.0%    0.0%    0.0%    0.0%    0.0%    0.0%    0.0%    0.0%    0.0%    0.0%    0.0%    0.0%    0.0%    0.0%    0.0%    0.0%    0.0%    0.0%    0.0%    0.0%    0.0%    0.0%    0.0%    0.0%    0.0%    0.0%    0.0%    0.0%    0.0%    0.0%    0.0%    0.0%    0.0%    0.0%    0.0%    0.0%    0.0%    0.0%    0.0%    0.0%    0.0%    0.0%    0.0%    0.0%    0.0%    0.0%    0.0%    0.0%    0.0%    0.0%    0.0%    0.0%    0.0%    0.0%    0.0%    0.0%    0.0%    0.0%    0.0%    0.0%    0.0%    0.0%    0.0%    0.0%    0.0%    0.0%    0.0%    0.0%    0.0%    0.0%    0.0%    0.0%    0.0%        k__Bacteria;p__Acidobacteria; c__Chloracidobacteria    3483    0.1%    0.0%    0.1%    0.0%    0.0%    0.2%    0.1%    0.2%    0.1%    0.0%    0.1%    0.1%    0.0%    0.0%    0.0%    0.0%    0.1%    0.0%    0.1%    0.0%    0.0%    0.0%    0.0%    0.0%    0.1%    0.1%    0.1%    0.1%    0.1%    0.0%    0.0%    0.0%    0.1%    0.0%    0.0%    0.0%    0.0%    0.1%    0.0%    0.1%    0.1%    0.0%    0.0%    0.3%    0.0%    0.0%    0.1%    0.1%    0.0%    0.0%    0.0%    0.0%    0.0%    0.1%    0.1%    0.1%    0.2%    0.0%    0.0%    0.1%    0.1%    0.2%    0.0%    0.0%    0.0%    0.0%    0.1%    0.1%    0.4%    0.1%    0.1%    0.1%    0.0%    0.1%    0.0%    0.1%    0.1%    0.0%    0.0%    0.2%    0.1%    0.0%    0.1%    0.0%    0.2%    0.1%    0.1%    0.1%    0.1%    0.1%    0.1%    0.1%    0.1%    0.0%    0.0%    0.1%    0.2%    0.0%    0.2%    0.1%    0.1%    0.1%    0.1%    0.1%    0.1%    0.0%    0.0%    0.0%    0.3%    0.4%    0.2%    0.2%    0.1%    0.1%    0.0%    0.0%    0.1%    0.2%    0.2%    0.0%    0.1%    0.2%    0.0%    0.1%    0.0%    0.1%    0.0%    0.1%    0.1%    0.1%    0.1%    0.1%    0.0%    0.1%        k__Bacteria;p__Acidobacteria; c__Holophagae       2    0.0%    0.0%    0.0%    0.0%    0.0%    0.0%    0.0%    0.0%    0.0%    0.0%    0.0%    0.0%    0.0%    0.0%    0.0%    0.0%    0.0%    0.0%    0.0%    0.0%    0.0%    0.0%    0.0%    0.0%    0.0%    0.0%    0.0%    0.0%    0.0%    0.0%    0.0%    0.0%    0.0%    0.0%    0.0%    0.0%    0.0%    0.0%    0.0%    0.0%    0.0%    0.0%    0.0%    0.0%    0.0%    0.0%    0.0%    0.0%    0.0%    0.0%    0.0%    0.0%    0.0%    0.0%    0.0%    0.0%    0.0%    0.0%    0.0%    0.0%    0.0%    0.0%    0.0%    0.0%    0.0%    0.0%    0.0%    0.0%    0.0%    0.0%    0.0%    0.0%    0.0%    0.0%    0.0%    0.0%    0.0%    0.0%    0.0%    0.0%    0.0%    0.0%    0.0%    0.0%    0.0%    0.0%    0.0%    0.0%    0.0%    0.0%    0.0%    0.0%    0.0%    0.0%    0.0%    0.0%    0.0%    0.0%    0.0%    0.0%    0.0%    0.0%    0.0%    0.0%    0.0%    0.0%    0.0%    0.0%    0.0%    0.0%    0.0%    0.0%    0.0%    0.0%    0.0%    0.0%    0.0%    0.0%    0.0%    0.0%    0.0%    0.0%    0.0%    0.0%    0.0%    0.0%    0.0%    0.0%    0.0%    0.0%    0.0%    0.0%    0.0%    0.0%        k__Bacteria;p__Acidobacteria; c__MVS-40      18    0.0%    0.0%    0.0%    0.0%    0.0%    0.0%    0.0%    0.0%    0.0%    0.0%    0.0%    0.0%    0.0%    0.0%    0.0%    0.0%    0.0%    0.0%    0.0%    0.0%    0.0%    0.0%    0.0%    0.0%    0.0%    0.0%    0.0%    0.0%    0.0%    0.0%    0.0%    0.0%    0.0%    0.0%    0.0%    0.0%    0.0%    0.0%    0.0%    0.0%    0.0%    0.0%    0.0%    0.0%    0.0%    0.0%    0.0%    0.0%    0.0%    0.0%    0.0%    0.0%    0.0%    0.0%    0.0%    0.0%    0.0%    0.0%    0.0%    0.0%    0.0%    0.0%    0.0%    0.0%    0.0%    0.0%    0.0%    0.0%    0.0%    0.0%    0.0%    0.0%    0.0%    0.0%    0.0%    0.0%    0.0%    0.0%    0.0%    0.0%    0.0%    0.0%    0.0%    0.0%    0.0%    0.0%    0.0%    0.0%    0.0%    0.0%    0.0%    0.0%    0.0%    0.0%    0.0%    0.0%    0.0%    0.0%    0.0%    0.0%    0.0%    0.0%    0.0%    0.0%    0.0%    0.0%    0.0%    0.0%    0.0%    0.0%    0.0%    0.0%    0.0%    0.0%    0.0%    0.0%    0.0%    0.0%    0.0%    0.0%    0.0%    0.0%    0.0%    0.0%    0.0%    0.0%    0.0%    0.0%    0.0%    0.0%    0.0%    0.0%    0.0%    0.0%        k__Bacteria;p__Acidobacteria; c__OS-K       0    0.0%    0.0%    0.0%    0.0%    0.0%    0.0%    0.0%    0.0%    0.0%    0.0%    0.0%    0.0%    0.0%    0.0%    0.0%    0.0%    0.0%    0.0%    0.0%    0.0%    0.0%    0.0%    0.0%    0.0%    0.0%    0.0%    0.0%    0.0%    0.0%    0.0%    0.0%    0.0%    0.0%    0.0%    0.0%    0.0%    0.0%    0.0%    0.0%    0.0%    0.0%    0.0%    0.0%    0.0%    0.0%    0.0%    0.0%    0.0%    0.0%    0.0%    0.0%    0.0%    0.0%    0.0%    0.0%    0.0%    0.0%    0.0%    0.0%    0.0%    0.0%    0.0%    0.0%    0.0%    0.0%    0.0%    0.0%    0.0%    0.0%    0.0%    0.0%    0.0%    0.0%    0.0%    0.0%    0.0%    0.0%    0.0%    0.0%    0.0%    0.0%    0.0%    0.0%    0.0%    0.0%    0.0%    0.0%    0.0%    0.0%    0.0%    0.0%    0.0%    0.0%    0.0%    0.0%    0.0%    0.0%    0.0%    0.0%    0.0%    0.0%    0.0%    0.0%    0.0%    0.0%    0.0%    0.0%    0.0%    0.0%    0.0%    0.0%    0.0%    0.0%    0.0%    0.0%    0.0%    0.0%    0.0%    0.0%    0.0%    0.0%    0.0%    0.0%    0.0%    0.0%    0.0%    0.0%    0.0%    0.0%    0.0%    0.0%    0.0%    0.0%    0.0%        k__Bacteria;p__Acidobacteria; c__PAUC37f      30    0.0%    0.0%    0.0%    0.0%    0.0%    0.0%    0.0%    0.0%    0.0%    0.0%    0.0%    0.0%    0.0%    0.0%    0.0%    0.0%    0.0%    0.0%    0.0%    0.0%    0.0%    0.0%    0.0%    0.0%    0.0%    0.0%    0.0%    0.0%    0.0%    0.0%    0.0%    0.0%    0.0%    0.0%    0.0%    0.0%    0.0%    0.0%    0.0%    0.0%    0.0%    0.0%    0.0%    0.0%    0.0%    0.0%    0.0%    0.0%    0.0%    0.0%    0.0%    0.0%    0.0%    0.0%    0.0%    0.0%    0.0%    0.0%    0.0%    0.0%    0.0%    0.0%    0.0%    0.0%    0.0%    0.0%    0.0%    0.0%    0.0%    0.0%    0.0%    0.0%    0.0%    0.0%    0.0%    0.0%    0.0%    0.0%    0.0%    0.0%    0.0%    0.0%    0.0%    0.0%    0.0%    0.0%    0.0%    0.0%    0.0%    0.0%    0.0%    0.0%    0.0%    0.0%    0.0%    0.0%    0.0%    0.0%    0.0%    0.0%    0.0%    0.0%    0.0%    0.0%    0.0%    0.0%    0.0%    0.0%    0.0%    0.0%    0.0%    0.0%    0.0%    0.0%    0.0%    0.0%    0.0%    0.0%    0.0%    0.0%    0.0%    0.0%    0.0%    0.0%    0.0%    0.0%    0.0%    0.0%    0.0%    0.0%    0.0%    0.0%    0.0%    0.0%        k__Bacteria;p__Acidobacteria; c__RB25      18    0.0%    0.0%    0.0%    0.0%    0.0%    0.0%    0.0%    0.0%    0.0%    0.0%    0.0%    0.0%    0.0%    0.0%    0.0%    0.0%    0.0%    0.0%    0.0%    0.0%    0.0%    0.0%    0.0%    0.0%    0.0%    0.0%    0.0%    0.0%    0.0%    0.0%    0.0%    0.0%    0.0%    0.0%    0.0%    0.0%    0.0%    0.0%    0.0%    0.0%    0.0%    0.0%    0.0%    0.0%    0.0%    0.0%    0.0%    0.0%    0.0%    0.0%    0.0%    0.0%    0.0%    0.0%    0.0%    0.0%    0.0%    0.0%    0.0%    0.0%    0.0%    0.0%    0.0%    0.0%    0.0%    0.0%    0.0%    0.0%    0.0%    0.0%    0.0%    0.0%    0.0%    0.0%    0.0%    0.0%    0.0%    0.0%    0.0%    0.0%    0.0%    0.0%    0.0%    0.0%    0.0%    0.0%    0.0%    0.0%    0.0%    0.0%    0.0%    0.0%    0.0%    0.0%    0.0%    0.0%    0.0%    0.0%    0.0%    0.0%    0.0%    0.0%    0.0%    0.0%    0.0%    0.0%    0.0%    0.0%    0.0%    0.0%    0.0%    0.0%    0.0%    0.0%    0.0%    0.0%    0.0%    0.0%    0.0%    0.0%    0.0%    0.0%    0.0%    0.0%    0.0%    0.0%    0.0%    0.0%    0.0%    0.0%    0.0%    0.0%    0.0%    0.0%        k__Bacteria;p__Acidobacteria; c__Solibacteres    1506    0.0%    0.0%    0.0%    0.1%    0.0%    0.1%    0.0%    0.1%    0.0%    0.0%    0.0%    0.1%    0.0%    0.0%    0.0%    0.0%    0.0%    0.0%    0.0%    0.0%    0.0%    0.0%    0.0%    0.0%    0.1%    0.0%    0.0%    0.0%    0.0%    0.0%    0.0%    0.0%    0.0%    0.0%    0.0%    0.0%    0.0%    0.0%    0.0%    0.0%    0.0%    0.0%    0.0%    0.0%    0.0%    0.0%    0.0%    0.0%    0.0%    0.0%    0.0%    0.0%    0.0%    0.0%    0.0%    0.0%    0.0%    0.0%    0.0%    0.0%    0.1%    0.1%    0.0%    0.0%    0.0%    0.0%    0.0%    0.0%    0.2%    0.0%    0.0%    0.1%    0.0%    0.0%    0.0%    0.0%    0.0%    0.0%    0.0%    0.1%    0.1%    0.0%    0.0%    0.0%    0.0%    0.0%    0.0%    0.1%    0.0%    0.0%    0.1%    0.0%    0.0%    0.0%    0.0%    0.0%    0.1%    0.0%    0.1%    0.0%    0.0%    0.0%    0.0%    0.0%    0.0%    0.0%    0.1%    0.0%    0.0%    0.1%    0.1%    0.1%    0.0%    0.1%    0.0%    0.1%    0.0%    0.2%    0.1%    0.0%    0.0%    0.1%    0.0%    0.0%    0.0%    0.1%    0.0%    0.0%    0.1%    0.0%    0.0%    0.0%    0.0%    0.0%        k__Bacteria;p__Acidobacteria; c__Sva0725     261    0.0%    0.0%    0.0%    0.0%    0.0%    0.0%    0.0%    0.0%    0.0%    0.0%    0.0%    0.0%    0.0%    0.0%    0.0%    0.0%    0.0%    0.0%    0.0%    0.0%    0.0%    0.0%    0.0%    0.0%    0.0%    0.0%    0.0%    0.0%    0.0%    0.0%    0.0%    0.0%    0.0%    0.0%    0.0%    0.0%    0.0%    0.0%    0.0%    0.0%    0.0%    0.0%    0.0%    0.0%    0.0%    0.0%    0.0%    0.0%    0.0%    0.0%    0.0%    0.0%    0.0%    0.0%    0.0%    0.0%    0.0%    0.0%    0.0%    0.0%    0.0%    0.0%    0.0%    0.0%    0.0%    0.0%    0.0%    0.0%    0.0%    0.0%    0.0%    0.0%    0.0%    0.0%    0.0%    0.0%    0.0%    0.0%    0.0%    0.0%    0.0%    0.0%    0.0%    0.0%    0.0%    0.0%    0.0%    0.0%    0.0%    0.0%    0.0%    0.0%    0.0%    0.0%    0.0%    0.0%    0.0%    0.0%    0.0%    0.0%    0.0%    0.0%    0.0%    0.0%    0.0%    0.0%    0.0%    0.0%    0.0%    0.0%    0.0%    0.0%    0.0%    0.0%    0.0%    0.0%    0.0%    0.0%    0.0%    0.0%    0.0%    0.0%    0.0%    0.0%    0.0%    0.0%    0.0%    0.0%    0.0%    0.0%    0.0%    0.0%    0.0%    0.0%        k__Bacteria;p__Acidobacteria; c__iii1-8     298    0.0%    0.0%    0.0%    0.0%    0.0%    0.1%    0.0%    0.0%    0.0%    0.0%    0.0%    0.0%    0.0%    0.0%    0.0%    0.0%    0.0%    0.0%    0.0%    0.0%    0.0%    0.0%    0.0%    0.0%    0.0%    0.0%    0.0%    0.0%    0.0%    0.0%    0.0%    0.0%    0.0%    0.0%    0.0%    0.0%    0.0%    0.0%    0.0%    0.0%    0.0%    0.0%    0.0%    0.0%    0.0%    0.0%    0.0%    0.0%    0.0%    0.0%    0.0%    0.0%    0.0%    0.0%    0.0%    0.0%    0.0%    0.0%    0.0%    0.0%    0.0%    0.0%    0.0%    0.0%    0.0%    0.0%    0.0%    0.0%    0.0%    0.0%    0.0%    0.0%    0.0%    0.0%    0.0%    0.0%    0.0%    0.0%    0.0%    0.0%    0.0%    0.0%    0.0%    0.0%    0.0%    0.0%    0.0%    0.0%    0.0%    0.0%    0.0%    0.0%    0.0%    0.0%    0.0%    0.0%    0.0%    0.0%    0.0%    0.0%    0.0%    0.0%    0.0%    0.0%    0.0%    0.0%    0.0%    0.0%    0.0%    0.0%    0.0%    0.0%    0.0%    0.0%    0.0%    0.0%    0.0%    0.0%    0.0%    0.0%    0.0%    0.0%    0.0%    0.0%    0.0%    0.0%    0.0%    0.0%    0.0%    0.0%    0.0%    0.0%    0.0%    0.0%        k__Bacteria;p__Actinobacteria; c__    1146    0.0%    0.0%    0.1%    0.0%    0.0%    0.2%    0.0%    0.1%    0.0%    0.1%    0.1%    0.0%    0.0%    0.0%    0.0%    0.0%    0.0%    0.0%    0.0%    0.0%    0.1%    0.0%    0.0%    0.0%    0.0%    0.0%    0.0%    0.0%    0.0%    0.0%    0.0%    0.0%    0.0%    0.0%    0.0%    0.0%    0.0%    0.0%    0.0%    0.0%    0.0%    0.0%    0.0%    0.0%    0.0%    0.0%    0.0%    0.0%    0.0%    0.0%    0.0%    0.0%    0.0%    0.0%    0.0%    0.0%    0.0%    0.0%    0.0%    0.0%    0.0%    0.0%    0.0%    0.0%    0.0%    0.0%    0.0%    0.0%    0.0%    0.0%    0.0%    0.0%    0.0%    0.0%    0.0%    0.0%    0.0%    0.0%    0.0%    0.0%    0.1%    0.0%    0.0%    0.0%    0.0%    0.0%    0.0%    0.2%    0.0%    0.0%    0.0%    0.0%    0.0%    0.0%    0.0%    0.0%    0.0%    0.0%    0.0%    0.0%    0.0%    0.0%    0.0%    0.0%    0.0%    0.0%    0.0%    0.0%    0.0%    0.1%    0.1%    0.0%    0.0%    0.0%    0.0%    0.0%    0.0%    0.3%    0.1%    0.0%    0.0%    0.0%    0.0%    0.1%    0.0%    0.0%    0.0%    0.0%    0.0%    0.0%    0.0%    0.0%    0.0%    0.0%        k__Bacteria;p__Actinobacteria; c__Actinobacteria (class)   869750   20.2%   22.1%   26.1%   13.3%   27.0%   16.5%   22.9%   20.3%   12.9%   10.0%   16.3%   29.4%   27.5%   21.7%   16.0%   17.1%   18.0%   43.1%   22.1%   36.1%   17.0%   34.1%   15.3%   28.2%   29.6%   14.3%   14.3%   15.5%   23.6%   41.5%   17.8%   33.5%   22.5%   20.9%   11.4%    8.8%   25.3%   14.1%   25.4%   13.9%   12.0%   14.1%   10.8%   14.8%   11.0%   16.7%   19.4%    9.8%   14.2%   15.9%   16.7%   18.4%   19.5%   21.1%   14.9%   20.0%   17.0%   14.1%   36.3%   24.1%   28.0%   20.5%   22.3%   15.3%   15.3%   22.8%   12.4%   37.6%   25.9%   20.5%   17.1%   27.5%   23.8%   22.0%   21.7%   17.6%   21.7%   23.7%   33.8%   30.4%   17.3%   12.1%   19.5%   25.3%   19.0%   16.7%   17.1%   15.8%   18.0%   16.9%   21.6%   19.2%   16.4%   38.6%   10.6%   13.8%   23.9%   19.5%   14.6%   13.7%   13.7%   13.4%   16.9%   33.3%   19.0%    8.4%   14.6%   21.4%   26.0%   25.2%   22.8%   21.4%   21.9%   20.3%    8.9%   27.9%   29.0%   21.0%   21.2%   13.0%   26.5%   20.6%   17.4%   15.9%   12.8%   19.2%   17.5%   23.9%   22.6%   11.7%   17.2%   17.2%   17.0%   15.4%        k__Bacteria;p__Aquificae; c__Aquificae (class)      18    0.0%    0.0%    0.0%    0.0%    0.0%    0.0%    0.0%    0.0%    0.0%    0.0%    0.0%    0.0%    0.0%    0.0%    0.0%    0.0%    0.0%    0.0%    0.0%    0.0%    0.0%    0.0%    0.0%    0.0%    0.0%    0.0%    0.0%    0.0%    0.0%    0.0%    0.0%    0.0%    0.0%    0.0%    0.0%    0.0%    0.0%    0.0%    0.0%    0.0%    0.0%    0.0%    0.0%    0.0%    0.0%    0.0%    0.0%    0.0%    0.0%    0.0%    0.0%    0.0%    0.0%    0.0%    0.0%    0.0%    0.0%    0.0%    0.0%    0.0%    0.0%    0.0%    0.0%    0.0%    0.0%    0.0%    0.0%    0.0%    0.0%    0.0%    0.0%    0.0%    0.0%    0.0%    0.0%    0.0%    0.0%    0.0%    0.0%    0.0%    0.0%    0.0%    0.0%    0.0%    0.0%    0.0%    0.0%    0.0%    0.0%    0.0%    0.0%    0.0%    0.0%    0.0%    0.0%    0.0%    0.0%    0.0%    0.0%    0.0%    0.0%    0.0%    0.0%    0.0%    0.0%    0.0%    0.0%    0.0%    0.0%    0.0%    0.0%    0.0%    0.0%    0.0%    0.0%    0.0%    0.0%    0.0%    0.0%    0.0%    0.0%    0.0%    0.0%    0.0%    0.0%    0.0%    0.0%    0.0%    0.0%    0.0%    0.0%    0.0%    0.0%    0.0%        k__Bacteria;p__BRC1; c__       0    0.0%    0.0%    0.0%    0.0%    0.0%    0.0%    0.0%    0.0%    0.0%    0.0%    0.0%    0.0%    0.0%    0.0%    0.0%    0.0%    0.0%    0.0%    0.0%    0.0%    0.0%    0.0%    0.0%    0.0%    0.0%    0.0%    0.0%    0.0%    0.0%    0.0%    0.0%    0.0%    0.0%    0.0%    0.0%    0.0%    0.0%    0.0%    0.0%    0.0%    0.0%    0.0%    0.0%    0.0%    0.0%    0.0%    0.0%    0.0%    0.0%    0.0%    0.0%    0.0%    0.0%    0.0%    0.0%    0.0%    0.0%    0.0%    0.0%    0.0%    0.0%    0.0%    0.0%    0.0%    0.0%    0.0%    0.0%    0.0%    0.0%    0.0%    0.0%    0.0%    0.0%    0.0%    0.0%    0.0%    0.0%    0.0%    0.0%    0.0%    0.0%    0.0%    0.0%    0.0%    0.0%    0.0%    0.0%    0.0%    0.0%    0.0%    0.0%    0.0%    0.0%    0.0%    0.0%    0.0%    0.0%    0.0%    0.0%    0.0%    0.0%    0.0%    0.0%    0.0%    0.0%    0.0%    0.0%    0.0%    0.0%    0.0%    0.0%    0.0%    0.0%    0.0%    0.0%    0.0%    0.0%    0.0%    0.0%    0.0%    0.0%    0.0%    0.0%    0.0%    0.0%    0.0%    0.0%    0.0%    0.0%    0.0%    0.0%    0.0%    0.0%    0.0%        k__Bacteria;p__BRC1; c__PRR-11     136    0.0%    0.0%    0.0%    0.0%    0.0%    0.0%    0.0%    0.1%    0.0%    0.0%    0.0%    0.0%    0.0%    0.0%    0.0%    0.0%    0.0%    0.0%    0.0%    0.0%    0.0%    0.0%    0.0%    0.0%    0.0%    0.0%    0.0%    0.0%    0.0%    0.0%    0.0%    0.0%    0.0%    0.0%    0.0%    0.0%    0.0%    0.0%    0.0%    0.0%    0.0%    0.0%    0.0%    0.0%    0.0%    0.0%    0.0%    0.0%    0.0%    0.0%    0.0%    0.0%    0.0%    0.0%    0.0%    0.0%    0.0%    0.0%    0.0%    0.0%    0.0%    0.0%    0.0%    0.0%    0.0%    0.0%    0.0%    0.0%    0.0%    0.0%    0.0%    0.0%    0.0%    0.0%    0.0%    0.0%    0.0%    0.0%    0.0%    0.0%    0.0%    0.0%    0.0%    0.0%    0.0%    0.0%    0.0%    0.0%    0.0%    0.0%    0.0%    0.0%    0.0%    0.0%    0.0%    0.0%    0.0%    0.0%    0.0%    0.0%    0.0%    0.0%    0.0%    0.0%    0.0%    0.0%    0.0%    0.0%    0.0%    0.0%    0.0%    0.0%    0.0%    0.0%    0.0%    0.0%    0.0%    0.0%    0.0%    0.0%    0.0%    0.0%    0.0%    0.0%    0.0%    0.0%    0.0%    0.0%    0.0%    0.0%    0.0%    0.0%    0.0%    0.0%        k__Bacteria;p__Bacteroidetes; c__      19    0.0%    0.0%    0.0%    0.0%    0.0%    0.0%    0.0%    0.0%    0.0%    0.0%    0.0%    0.0%    0.0%    0.0%    0.0%    0.0%    0.0%    0.0%    0.0%    0.0%    0.0%    0.0%    0.0%    0.0%    0.0%    0.0%    0.0%    0.0%    0.0%    0.0%    0.0%    0.0%    0.0%    0.0%    0.0%    0.0%    0.0%    0.0%    0.0%    0.0%    0.0%    0.0%    0.0%    0.0%    0.0%    0.0%    0.0%    0.0%    0.0%    0.0%    0.0%    0.0%    0.0%    0.0%    0.0%    0.0%    0.0%    0.0%    0.0%    0.0%    0.0%    0.0%    0.0%    0.0%    0.0%    0.0%    0.0%    0.0%    0.0%    0.0%    0.0%    0.0%    0.0%    0.0%    0.0%    0.0%    0.0%    0.0%    0.0%    0.0%    0.0%    0.0%    0.0%    0.0%    0.0%    0.0%    0.0%    0.0%    0.0%    0.0%    0.0%    0.0%    0.0%    0.0%    0.0%    0.0%    0.0%    0.0%    0.0%    0.0%    0.0%    0.0%    0.0%    0.0%    0.0%    0.0%    0.0%    0.0%    0.0%    0.0%    0.0%    0.0%    0.0%    0.0%    0.0%    0.0%    0.0%    0.0%    0.0%    0.0%    0.0%    0.0%    0.0%    0.0%    0.0%    0.0%    0.0%    0.0%    0.0%    0.0%    0.0%    0.0%    0.0%    0.0%        k__Bacteria;p__Bacteroidetes; c__Bacteroidia   191534    4.4%    7.8%    6.6%    4.1%    3.1%    3.2%    1.6%    5.4%    5.9%    1.9%    3.0%    5.2%    2.5%    3.1%    6.8%    4.9%    1.2%    1.7%    4.5%    4.4%    1.5%    2.4%    5.5%    1.6%    3.3%    4.6%    1.1%    1.9%    7.0%    3.2%    7.0%    2.8%    2.9%    2.4%    1.9%    4.9%    6.9%    3.3%    2.6%    2.4%    4.6%    3.3%    2.1%    3.6%    7.5%    3.3%    3.7%    5.4%    2.4%    3.9%    2.5%    2.3%    2.2%    2.5%    2.4%    1.5%   10.9%    1.5%    2.1%    1.9%    4.0%    2.6%    2.0%   12.6%    1.6%    4.0%    5.5%    4.7%    1.5%    1.4%    2.3%    2.5%    7.6%    5.5%    3.6%    8.2%    4.2%    4.6%    5.0%    2.9%    9.8%    9.2%    5.1%    5.9%    6.2%    5.5%    6.6%    9.6%    9.4%    5.3%    2.8%    2.8%   18.7%    4.8%   17.8%    8.2%    5.5%   13.2%   12.3%    6.4%    9.5%    2.3%    8.5%    3.1%    3.2%    2.2%    1.9%    2.5%    2.1%    1.0%    2.4%    2.4%    5.2%    1.9%    0.9%    3.2%    2.5%    1.5%    3.5%    2.9%    1.5%    3.5%    7.2%    4.3%    4.7%    1.9%    2.8%    4.0%    2.3%    1.2%    2.3%    3.4%    2.3%    3.2%        k__Bacteria;p__Bacteroidetes; c__Flavobacteria   66100    1.5%    1.4%    1.5%    1.0%    0.7%    1.9%    1.0%    0.9%    1.1%    1.3%    1.3%    1.5%    0.4%    0.6%    0.7%    0.6%    0.6%    0.4%    1.1%    0.4%    0.4%    0.6%    1.6%    0.6%    0.8%    1.6%    1.1%    1.0%    0.9%    0.4%    0.9%    0.5%    1.9%    0.6%    1.6%    0.5%    0.3%    1.2%    0.9%    1.7%    1.5%    0.8%    3.4%    2.7%    1.7%    1.4%    0.9%    2.5%    2.4%    2.3%    0.9%    0.7%    0.2%    1.0%    1.0%    1.2%    1.2%    0.6%    0.4%    0.6%    0.5%    0.6%    0.8%    1.3%    0.9%    0.7%    1.9%    0.3%    1.3%    1.0%    0.9%    1.1%    0.5%    1.5%    1.4%    1.2%    3.3%    0.4%    0.3%    0.7%    1.1%    0.3%    1.9%    0.5%    1.4%    1.1%    2.7%    1.7%    1.5%    1.2%    1.5%    0.9%    0.6%    0.3%    1.4%    1.0%    0.8%    1.1%    1.2%    0.8%    1.8%    0.6%    1.2%    0.6%    1.1%    1.8%    1.0%    1.2%    1.0%    1.8%    0.9%    1.2%    1.1%    0.6%    0.7%    1.6%    2.2%    1.3%    1.5%    1.6%    3.2%    1.5%    1.6%    4.2%   11.8%    5.8%    3.5%    3.2%    2.5%   10.0%    5.6%    4.6%    4.4%    9.2%        k__Bacteria;p__Bacteroidetes; c__Sphingobacteria   70924    1.6%    1.0%    1.7%    1.2%    0.5%    3.2%    2.0%    4.3%    1.4%    0.4%    2.4%    2.0%    0.4%    1.3%    0.9%    0.8%    2.1%    0.3%    1.9%    0.6%    2.8%    0.9%    0.4%    0.9%    0.7%    1.3%    4.3%    1.8%    1.5%    0.7%    0.8%    0.7%    1.2%    0.6%    0.9%    0.4%    0.3%    1.4%    0.7%    1.1%    1.2%    0.7%    2.2%    1.5%    0.9%    0.9%    3.1%    0.8%    0.4%    1.1%    1.1%    0.5%    0.5%    2.0%    1.5%    1.6%    1.4%    1.1%    0.5%    2.8%    2.3%    2.7%    0.8%    0.4%    0.8%    0.7%    1.4%    0.9%    5.5%    2.6%    1.1%    1.9%    0.4%    1.9%    1.6%    1.4%    1.8%    0.5%    0.4%    3.9%    1.1%    0.9%    3.3%    1.2%    2.6%    1.2%    1.5%    2.0%    2.0%    2.0%    2.7%    2.7%    1.8%    0.2%    0.8%    1.1%    2.5%    1.1%    1.5%    1.1%    1.0%    1.0%    1.6%    1.9%    2.7%    5.2%    2.3%    1.6%    3.7%    4.8%    3.1%    2.5%    1.3%    1.4%    2.2%    2.2%    3.6%    3.3%    2.1%    0.7%    1.4%    2.6%    0.9%    1.9%    2.0%    2.4%    1.8%    1.8%    2.4%    2.6%    4.2%    2.4%    1.3%    3.3%        k__Bacteria;p__CCM11b; c__      20    0.0%    0.0%    0.0%    0.0%    0.0%    0.0%    0.0%    0.0%    0.0%    0.0%    0.0%    0.0%    0.0%    0.0%    0.0%    0.0%    0.0%    0.0%    0.0%    0.0%    0.0%    0.0%    0.0%    0.0%    0.0%    0.0%    0.0%    0.0%    0.0%    0.0%    0.0%    0.0%    0.0%    0.0%    0.0%    0.0%    0.0%    0.0%    0.0%    0.0%    0.0%    0.0%    0.0%    0.0%    0.0%    0.0%    0.0%    0.0%    0.0%    0.0%    0.0%    0.0%    0.0%    0.0%    0.0%    0.0%    0.0%    0.0%    0.0%    0.0%    0.0%    0.0%    0.0%    0.0%    0.0%    0.0%    0.0%    0.0%    0.0%    0.0%    0.0%    0.0%    0.0%    0.0%    0.0%    0.0%    0.0%    0.0%    0.0%    0.0%    0.0%    0.0%    0.0%    0.0%    0.0%    0.0%    0.0%    0.0%    0.0%    0.0%    0.0%    0.0%    0.0%    0.0%    0.0%    0.0%    0.0%    0.0%    0.0%    0.0%    0.0%    0.0%    0.0%    0.0%    0.0%    0.0%    0.0%    0.0%    0.0%    0.0%    0.0%    0.0%    0.0%    0.0%    0.0%    0.0%    0.0%    0.0%    0.0%    0.0%    0.0%    0.0%    0.0%    0.0%    0.0%    0.0%    0.0%    0.0%    0.0%    0.0%    0.0%    0.0%    0.0%    0.0%        k__Bacteria;p__Caldithrix_KSB1; c__Caldithrixae       0    0.0%    0.0%    0.0%    0.0%    0.0%    0.0%    0.0%    0.0%    0.0%    0.0%    0.0%    0.0%    0.0%    0.0%    0.0%    0.0%    0.0%    0.0%    0.0%    0.0%    0.0%    0.0%    0.0%    0.0%    0.0%    0.0%    0.0%    0.0%    0.0%    0.0%    0.0%    0.0%    0.0%    0.0%    0.0%    0.0%    0.0%    0.0%    0.0%    0.0%    0.0%    0.0%    0.0%    0.0%    0.0%    0.0%    0.0%    0.0%    0.0%    0.0%    0.0%    0.0%    0.0%    0.0%    0.0%    0.0%    0.0%    0.0%    0.0%    0.0%    0.0%    0.0%    0.0%    0.0%    0.0%    0.0%    0.0%    0.0%    0.0%    0.0%    0.0%    0.0%    0.0%    0.0%    0.0%    0.0%    0.0%    0.0%    0.0%    0.0%    0.0%    0.0%    0.0%    0.0%    0.0%    0.0%    0.0%    0.0%    0.0%    0.0%    0.0%    0.0%    0.0%    0.0%    0.0%    0.0%    0.0%    0.0%    0.0%    0.0%    0.0%    0.0%    0.0%    0.0%    0.0%    0.0%    0.0%    0.0%    0.0%    0.0%    0.0%    0.0%    0.0%    0.0%    0.0%    0.0%    0.0%    0.0%    0.0%    0.0%    0.0%    0.0%    0.0%    0.0%    0.0%    0.0%    0.0%    0.0%    0.0%    0.0%    0.0%    0.0%    0.0%    0.0%        k__Bacteria;p__Chlamydiae; c__Chlamydiae (class)      17    0.0%    0.0%    0.0%    0.0%    0.0%    0.0%    0.0%    0.0%    0.0%    0.0%    0.0%    0.0%    0.0%    0.0%    0.0%    0.0%    0.0%    0.0%    0.0%    0.0%    0.0%    0.0%    0.0%    0.0%    0.0%    0.0%    0.0%    0.0%    0.0%    0.0%    0.0%    0.0%    0.0%    0.0%    0.0%    0.0%    0.0%    0.0%    0.0%    0.0%    0.0%    0.0%    0.0%    0.0%    0.0%    0.0%    0.0%    0.0%    0.0%    0.0%    0.0%    0.0%    0.0%    0.0%    0.0%    0.0%    0.0%    0.0%    0.0%    0.0%    0.0%    0.0%    0.0%    0.0%    0.0%    0.0%    0.0%    0.0%    0.0%    0.0%    0.0%    0.0%    0.0%    0.0%    0.0%    0.0%    0.0%    0.0%    0.0%    0.0%    0.0%    0.0%    0.0%    0.0%    0.0%    0.0%    0.0%    0.0%    0.0%    0.0%    0.0%    0.0%    0.0%    0.0%    0.0%    0.0%    0.0%    0.0%    0.0%    0.0%    0.0%    0.0%    0.0%    0.0%    0.0%    0.0%    0.0%    0.0%    0.0%    0.0%    0.0%    0.0%    0.0%    0.0%    0.0%    0.0%    0.0%    0.0%    0.0%    0.0%    0.0%    0.0%    0.0%    0.0%    0.0%    0.0%    0.0%    0.0%    0.0%    0.0%    0.0%    0.0%    0.0%    0.0%        k__Bacteria;p__Chlorobi; c__       0    0.0%    0.0%    0.0%    0.0%    0.0%    0.0%    0.0%    0.0%    0.0%    0.0%    0.0%    0.0%    0.0%    0.0%    0.0%    0.0%    0.0%    0.0%    0.0%    0.0%    0.0%    0.0%    0.0%    0.0%    0.0%    0.0%    0.0%    0.0%    0.0%    0.0%    0.0%    0.0%    0.0%    0.0%    0.0%    0.0%    0.0%    0.0%    0.0%    0.0%    0.0%    0.0%    0.0%    0.0%    0.0%    0.0%    0.0%    0.0%    0.0%    0.0%    0.0%    0.0%    0.0%    0.0%    0.0%    0.0%    0.0%    0.0%    0.0%    0.0%    0.0%    0.0%    0.0%    0.0%    0.0%    0.0%    0.0%    0.0%    0.0%    0.0%    0.0%    0.0%    0.0%    0.0%    0.0%    0.0%    0.0%    0.0%    0.0%    0.0%    0.0%    0.0%    0.0%    0.0%    0.0%    0.0%    0.0%    0.0%    0.0%    0.0%    0.0%    0.0%    0.0%    0.0%    0.0%    0.0%    0.0%    0.0%    0.0%    0.0%    0.0%    0.0%    0.0%    0.0%    0.0%    0.0%    0.0%    0.0%    0.0%    0.0%    0.0%    0.0%    0.0%    0.0%    0.0%    0.0%    0.0%    0.0%    0.0%    0.0%    0.0%    0.0%    0.0%    0.0%    0.0%    0.0%    0.0%    0.0%    0.0%    0.0%    0.0%    0.0%    0.0%    0.0%        k__Bacteria;p__Chlorobi; c__BSV19       1    0.0%    0.0%    0.0%    0.0%    0.0%    0.0%    0.0%    0.0%    0.0%    0.0%    0.0%    0.0%    0.0%    0.0%    0.0%    0.0%    0.0%    0.0%    0.0%    0.0%    0.0%    0.0%    0.0%    0.0%    0.0%    0.0%    0.0%    0.0%    0.0%    0.0%    0.0%    0.0%    0.0%    0.0%    0.0%    0.0%    0.0%    0.0%    0.0%    0.0%    0.0%    0.0%    0.0%    0.0%    0.0%    0.0%    0.0%    0.0%    0.0%    0.0%    0.0%    0.0%    0.0%    0.0%    0.0%    0.0%    0.0%    0.0%    0.0%    0.0%    0.0%    0.0%    0.0%    0.0%    0.0%    0.0%    0.0%    0.0%    0.0%    0.0%    0.0%    0.0%    0.0%    0.0%    0.0%    0.0%    0.0%    0.0%    0.0%    0.0%    0.0%    0.0%    0.0%    0.0%    0.0%    0.0%    0.0%    0.0%    0.0%    0.0%    0.0%    0.0%    0.0%    0.0%    0.0%    0.0%    0.0%    0.0%    0.0%    0.0%    0.0%    0.0%    0.0%    0.0%    0.0%    0.0%    0.0%    0.0%    0.0%    0.0%    0.0%    0.0%    0.0%    0.0%    0.0%    0.0%    0.0%    0.0%    0.0%    0.0%    0.0%    0.0%    0.0%    0.0%    0.0%    0.0%    0.0%    0.0%    0.0%    0.0%    0.0%    0.0%    0.0%    0.0%        k__Bacteria;p__Chlorobi; c__OPB56       8    0.0%    0.0%    0.0%    0.0%    0.0%    0.0%    0.0%    0.0%    0.0%    0.0%    0.0%    0.0%    0.0%    0.0%    0.0%    0.0%    0.0%    0.0%    0.0%    0.0%    0.0%    0.0%    0.0%    0.0%    0.0%    0.0%    0.0%    0.0%    0.0%    0.0%    0.0%    0.0%    0.0%    0.0%    0.0%    0.0%    0.0%    0.0%    0.0%    0.0%    0.0%    0.0%    0.0%    0.0%    0.0%    0.0%    0.0%    0.0%    0.0%    0.0%    0.0%    0.0%    0.0%    0.0%    0.0%    0.0%    0.0%    0.0%    0.0%    0.0%    0.0%    0.0%    0.0%    0.0%    0.0%    0.0%    0.0%    0.0%    0.0%    0.0%    0.0%    0.0%    0.0%    0.0%    0.0%    0.0%    0.0%    0.0%    0.0%    0.0%    0.0%    0.0%    0.0%    0.0%    0.0%    0.0%    0.0%    0.0%    0.0%    0.0%    0.0%    0.0%    0.0%    0.0%    0.0%    0.0%    0.0%    0.0%    0.0%    0.0%    0.0%    0.0%    0.0%    0.0%    0.0%    0.0%    0.0%    0.0%    0.0%    0.0%    0.0%    0.0%    0.0%    0.0%    0.0%    0.0%    0.0%    0.0%    0.0%    0.0%    0.0%    0.0%    0.0%    0.0%    0.0%    0.0%    0.0%    0.0%    0.0%    0.0%    0.0%    0.0%    0.0%    0.0%        k__Bacteria;p__Chlorobi; c__SJA-28      22    0.0%    0.0%    0.0%    0.0%    0.0%    0.0%    0.0%    0.0%    0.0%    0.0%    0.0%    0.0%    0.0%    0.0%    0.0%    0.0%    0.0%    0.0%    0.0%    0.0%    0.0%    0.0%    0.0%    0.0%    0.0%    0.0%    0.0%    0.0%    0.0%    0.0%    0.0%    0.0%    0.0%    0.0%    0.0%    0.0%    0.0%    0.0%    0.0%    0.0%    0.0%    0.0%    0.0%    0.0%    0.0%    0.0%    0.0%    0.0%    0.0%    0.0%    0.0%    0.0%    0.0%    0.0%    0.0%    0.0%    0.0%    0.0%    0.0%    0.0%    0.0%    0.0%    0.0%    0.0%    0.0%    0.0%    0.0%    0.0%    0.0%    0.0%    0.0%    0.0%    0.0%    0.0%    0.0%    0.0%    0.0%    0.0%    0.0%    0.0%    0.0%    0.0%    0.0%    0.0%    0.0%    0.0%    0.0%    0.0%    0.0%    0.0%    0.0%    0.0%    0.0%    0.0%    0.0%    0.0%    0.0%    0.0%    0.0%    0.0%    0.0%    0.0%    0.0%    0.0%    0.0%    0.0%    0.0%    0.0%    0.0%    0.0%    0.0%    0.0%    0.0%    0.0%    0.0%    0.0%    0.0%    0.0%    0.0%    0.0%    0.0%    0.0%    0.0%    0.0%    0.0%    0.0%    0.0%    0.0%    0.0%    0.0%    0.0%    0.0%    0.0%    0.0%        k__Bacteria;p__Chlorobi; c__ZB1       2    0.0%    0.0%    0.0%    0.0%    0.0%    0.0%    0.0%    0.0%    0.0%    0.0%    0.0%    0.0%    0.0%    0.0%    0.0%    0.0%    0.0%    0.0%    0.0%    0.0%    0.0%    0.0%    0.0%    0.0%    0.0%    0.0%    0.0%    0.0%    0.0%    0.0%    0.0%    0.0%    0.0%    0.0%    0.0%    0.0%    0.0%    0.0%    0.0%    0.0%    0.0%    0.0%    0.0%    0.0%    0.0%    0.0%    0.0%    0.0%    0.0%    0.0%    0.0%    0.0%    0.0%    0.0%    0.0%    0.0%    0.0%    0.0%    0.0%    0.0%    0.0%    0.0%    0.0%    0.0%    0.0%    0.0%    0.0%    0.0%    0.0%    0.0%    0.0%    0.0%    0.0%    0.0%    0.0%    0.0%    0.0%    0.0%    0.0%    0.0%    0.0%    0.0%    0.0%    0.0%    0.0%    0.0%    0.0%    0.0%    0.0%    0.0%    0.0%    0.0%    0.0%    0.0%    0.0%    0.0%    0.0%    0.0%    0.0%    0.0%    0.0%    0.0%    0.0%    0.0%    0.0%    0.0%    0.0%    0.0%    0.0%    0.0%    0.0%    0.0%    0.0%    0.0%    0.0%    0.0%    0.0%    0.0%    0.0%    0.0%    0.0%    0.0%    0.0%    0.0%    0.0%    0.0%    0.0%    0.0%    0.0%    0.0%    0.0%    0.0%    0.0%    0.0%        k__Bacteria;p__Chloroflexi; c__       0    0.0%    0.0%    0.0%    0.0%    0.0%    0.0%    0.0%    0.0%    0.0%    0.0%    0.0%    0.0%    0.0%    0.0%    0.0%    0.0%    0.0%    0.0%    0.0%    0.0%    0.0%    0.0%    0.0%    0.0%    0.0%    0.0%    0.0%    0.0%    0.0%    0.0%    0.0%    0.0%    0.0%    0.0%    0.0%    0.0%    0.0%    0.0%    0.0%    0.0%    0.0%    0.0%    0.0%    0.0%    0.0%    0.0%    0.0%    0.0%    0.0%    0.0%    0.0%    0.0%    0.0%    0.0%    0.0%    0.0%    0.0%    0.0%    0.0%    0.0%    0.0%    0.0%    0.0%    0.0%    0.0%    0.0%    0.0%    0.0%    0.0%    0.0%    0.0%    0.0%    0.0%    0.0%    0.0%    0.0%    0.0%    0.0%    0.0%    0.0%    0.0%    0.0%    0.0%    0.0%    0.0%    0.0%    0.0%    0.0%    0.0%    0.0%    0.0%    0.0%    0.0%    0.0%    0.0%    0.0%    0.0%    0.0%    0.0%    0.0%    0.0%    0.0%    0.0%    0.0%    0.0%    0.0%    0.0%    0.0%    0.0%    0.0%    0.0%    0.0%    0.0%    0.0%    0.0%    0.0%    0.0%    0.0%    0.0%    0.0%    0.0%    0.0%    0.0%    0.0%    0.0%    0.0%    0.0%    0.0%    0.0%    0.0%    0.0%    0.0%    0.0%    0.0%        k__Bacteria;p__Chloroflexi; c__Anaerolineae     443    0.0%    0.0%    0.0%    0.0%    0.0%    0.0%    0.0%    0.1%    0.0%    0.0%    0.0%    0.0%    0.0%    0.0%    0.0%    0.0%    0.0%    0.0%    0.0%    0.0%    0.0%    0.0%    0.0%    0.0%    0.0%    0.0%    0.0%    0.0%    0.0%    0.0%    0.0%    0.0%    0.0%    0.0%    0.0%    0.0%    0.0%    0.0%    0.0%    0.0%    0.0%    0.0%    0.0%    0.0%    0.0%    0.0%    0.0%    0.0%    0.0%    0.0%    0.0%    0.0%    0.0%    0.0%    0.0%    0.0%    0.0%    0.0%    0.0%    0.0%    0.0%    0.0%    0.0%    0.0%    0.0%    0.0%    0.0%    0.0%    0.0%    0.0%    0.0%    0.0%    0.0%    0.0%    0.0%    0.0%    0.0%    0.0%    0.0%    0.0%    0.0%    0.0%    0.0%    0.0%    0.0%    0.0%    0.0%    0.0%    0.0%    0.0%    0.0%    0.1%    0.0%    0.0%    0.0%    0.0%    0.0%    0.0%    0.0%    0.0%    0.0%    0.0%    0.0%    0.0%    0.0%    0.0%    0.0%    0.0%    0.1%    0.0%    0.0%    0.0%    0.0%    0.0%    0.0%    0.0%    0.0%    0.0%    0.0%    0.0%    0.0%    0.0%    0.0%    0.0%    0.0%    0.0%    0.0%    0.0%    0.0%    0.0%    0.0%    0.0%    0.0%    0.0%        k__Bacteria;p__Chloroflexi; c__Bljii12     468    0.0%    0.0%    0.0%    0.1%    0.0%    0.0%    0.0%    0.0%    0.0%    0.0%    0.0%    0.0%    0.0%    0.0%    0.0%    0.0%    0.0%    0.0%    0.0%    0.0%    0.0%    0.0%    0.0%    0.0%    0.0%    0.0%    0.0%    0.0%    0.0%    0.0%    0.0%    0.0%    0.0%    0.0%    0.0%    0.0%    0.0%    0.0%    0.0%    0.0%    0.0%    0.0%    0.0%    0.0%    0.0%    0.0%    0.0%    0.0%    0.0%    0.0%    0.0%    0.0%    0.0%    0.0%    0.0%    0.0%    0.0%    0.0%    0.0%    0.0%    0.0%    0.0%    0.0%    0.0%    0.0%    0.0%    0.0%    0.0%    0.1%    0.0%    0.0%    0.0%    0.0%    0.0%    0.0%    0.0%    0.0%    0.0%    0.0%    0.0%    0.0%    0.0%    0.0%    0.0%    0.0%    0.0%    0.0%    0.0%    0.0%    0.0%    0.0%    0.0%    0.0%    0.0%    0.0%    0.0%    0.0%    0.0%    0.0%    0.0%    0.0%    0.0%    0.0%    0.0%    0.0%    0.0%    0.0%    0.0%    0.0%    0.0%    0.0%    0.0%    0.0%    0.0%    0.0%    0.0%    0.0%    0.1%    0.0%    0.0%    0.0%    0.0%    0.0%    0.0%    0.0%    0.1%    0.0%    0.0%    0.0%    0.0%    0.0%    0.0%    0.0%    0.0%        k__Bacteria;p__Chloroflexi; c__Chloroflexi (class)      29    0.0%    0.0%    0.0%    0.0%    0.0%    0.0%    0.0%    0.0%    0.0%    0.0%    0.0%    0.0%    0.0%    0.0%    0.0%    0.0%    0.0%    0.0%    0.0%    0.0%    0.0%    0.0%    0.0%    0.0%    0.0%    0.0%    0.0%    0.0%    0.0%    0.0%    0.0%    0.0%    0.0%    0.0%    0.0%    0.0%    0.0%    0.0%    0.0%    0.0%    0.0%    0.0%    0.0%    0.0%    0.0%    0.0%    0.0%    0.0%    0.0%    0.0%    0.0%    0.0%    0.0%    0.0%    0.0%    0.0%    0.0%    0.0%    0.0%    0.0%    0.0%    0.0%    0.0%    0.0%    0.0%    0.0%    0.0%    0.0%    0.0%    0.0%    0.0%    0.0%    0.0%    0.0%    0.0%    0.0%    0.0%    0.0%    0.0%    0.0%    0.0%    0.0%    0.0%    0.0%    0.0%    0.0%    0.0%    0.0%    0.0%    0.0%    0.0%    0.0%    0.0%    0.0%    0.0%    0.0%    0.0%    0.0%    0.0%    0.0%    0.0%    0.0%    0.0%    0.0%    0.0%    0.0%    0.0%    0.0%    0.0%    0.0%    0.0%    0.0%    0.0%    0.0%    0.0%    0.0%    0.0%    0.0%    0.0%    0.0%    0.0%    0.0%    0.0%    0.0%    0.0%    0.0%    0.0%    0.0%    0.0%    0.0%    0.0%    0.0%    0.0%    0.0%        k__Bacteria;p__Chloroflexi; c__Chloroflexi-4       1    0.0%    0.0%    0.0%    0.0%    0.0%    0.0%    0.0%    0.0%    0.0%    0.0%    0.0%    0.0%    0.0%    0.0%    0.0%    0.0%    0.0%    0.0%    0.0%    0.0%    0.0%    0.0%    0.0%    0.0%    0.0%    0.0%    0.0%    0.0%    0.0%    0.0%    0.0%    0.0%    0.0%    0.0%    0.0%    0.0%    0.0%    0.0%    0.0%    0.0%    0.0%    0.0%    0.0%    0.0%    0.0%    0.0%    0.0%    0.0%    0.0%    0.0%    0.0%    0.0%    0.0%    0.0%    0.0%    0.0%    0.0%    0.0%    0.0%    0.0%    0.0%    0.0%    0.0%    0.0%    0.0%    0.0%    0.0%    0.0%    0.0%    0.0%    0.0%    0.0%    0.0%    0.0%    0.0%    0.0%    0.0%    0.0%    0.0%    0.0%    0.0%    0.0%    0.0%    0.0%    0.0%    0.0%    0.0%    0.0%    0.0%    0.0%    0.0%    0.0%    0.0%    0.0%    0.0%    0.0%    0.0%    0.0%    0.0%    0.0%    0.0%    0.0%    0.0%    0.0%    0.0%    0.0%    0.0%    0.0%    0.0%    0.0%    0.0%    0.0%    0.0%    0.0%    0.0%    0.0%    0.0%    0.0%    0.0%    0.0%    0.0%    0.0%    0.0%    0.0%    0.0%    0.0%    0.0%    0.0%    0.0%    0.0%    0.0%    0.0%    0.0%    0.0%        k__Bacteria;p__Chloroflexi; c__Ktedonobacteria       3    0.0%    0.0%    0.0%    0.0%    0.0%    0.0%    0.0%    0.0%    0.0%    0.0%    0.0%    0.0%    0.0%    0.0%    0.0%    0.0%    0.0%    0.0%    0.0%    0.0%    0.0%    0.0%    0.0%    0.0%    0.0%    0.0%    0.0%    0.0%    0.0%    0.0%    0.0%    0.0%    0.0%    0.0%    0.0%    0.0%    0.0%    0.0%    0.0%    0.0%    0.0%    0.0%    0.0%    0.0%    0.0%    0.0%    0.0%    0.0%    0.0%    0.0%    0.0%    0.0%    0.0%    0.0%    0.0%    0.0%    0.0%    0.0%    0.0%    0.0%    0.0%    0.0%    0.0%    0.0%    0.0%    0.0%    0.0%    0.0%    0.0%    0.0%    0.0%    0.0%    0.0%    0.0%    0.0%    0.0%    0.0%    0.0%    0.0%    0.0%    0.0%    0.0%    0.0%    0.0%    0.0%    0.0%    0.0%    0.0%    0.0%    0.0%    0.0%    0.0%    0.0%    0.0%    0.0%    0.0%    0.0%    0.0%    0.0%    0.0%    0.0%    0.0%    0.0%    0.0%    0.0%    0.0%    0.0%    0.0%    0.0%    0.0%    0.0%    0.0%    0.0%    0.0%    0.0%    0.0%    0.0%    0.0%    0.0%    0.0%    0.0%    0.0%    0.0%    0.0%    0.0%    0.0%    0.0%    0.0%    0.0%    0.0%    0.0%    0.0%    0.0%    0.0%        k__Bacteria;p__Chloroflexi; c__SOGA31    5915    0.1%    0.1%    0.1%    0.1%    0.0%    0.1%    0.1%    0.2%    0.1%    0.0%    0.2%    0.1%    0.0%    0.1%    0.0%    0.0%    0.1%    0.0%    0.1%    0.0%    0.1%    0.0%    0.0%    0.1%    0.1%    0.0%    0.1%    0.1%    0.0%    0.0%    0.0%    0.0%    0.1%    0.1%    0.1%    0.0%    0.0%    0.1%    0.1%    0.1%    0.1%    0.0%    0.1%    0.1%    0.1%    0.1%    0.1%    0.0%    0.0%    0.1%    0.1%    0.1%    0.0%    0.2%    0.1%    0.1%    0.2%    0.1%    0.1%    0.2%    0.1%    0.5%    0.1%    0.0%    0.1%    0.1%    0.3%    0.1%    0.6%    0.2%    0.1%    0.2%    0.0%    0.2%    0.2%    0.4%    0.4%    0.0%    0.0%    0.5%    0.1%    0.1%    0.2%    0.0%    0.2%    0.1%    0.1%    0.2%    0.3%    0.1%    0.1%    0.2%    0.1%    0.0%    0.1%    0.1%    0.1%    0.1%    0.3%    0.1%    0.1%    0.2%    0.2%    0.1%    0.1%    0.1%    0.1%    0.1%    0.7%    0.1%    0.3%    0.5%    0.3%    0.1%    0.1%    0.2%    0.1%    0.4%    0.3%    0.1%    0.1%    0.4%    0.1%    0.2%    0.1%    0.5%    0.2%    0.1%    0.3%    0.1%    0.2%    0.2%    0.1%    0.1%        k__Bacteria;p__Chloroflexi; c__TK17     238    0.0%    0.0%    0.0%    0.0%    0.0%    0.0%    0.0%    0.0%    0.0%    0.0%    0.0%    0.0%    0.0%    0.0%    0.0%    0.0%    0.0%    0.0%    0.0%    0.0%    0.0%    0.0%    0.0%    0.0%    0.0%    0.0%    0.0%    0.0%    0.0%    0.0%    0.0%    0.0%    0.0%    0.0%    0.0%    0.0%    0.0%    0.0%    0.0%    0.0%    0.0%    0.0%    0.0%    0.0%    0.0%    0.0%    0.0%    0.0%    0.0%    0.0%    0.0%    0.0%    0.0%    0.0%    0.0%    0.0%    0.0%    0.0%    0.0%    0.0%    0.0%    0.0%    0.0%    0.0%    0.0%    0.0%    0.0%    0.0%    0.0%    0.0%    0.0%    0.0%    0.0%    0.0%    0.0%    0.0%    0.0%    0.0%    0.0%    0.0%    0.0%    0.0%    0.0%    0.0%    0.0%    0.0%    0.0%    0.0%    0.0%    0.0%    0.0%    0.0%    0.0%    0.0%    0.0%    0.0%    0.0%    0.0%    0.0%    0.0%    0.0%    0.0%    0.0%    0.0%    0.0%    0.0%    0.0%    0.0%    0.0%    0.0%    0.0%    0.0%    0.0%    0.0%    0.0%    0.0%    0.0%    0.0%    0.0%    0.0%    0.0%    0.0%    0.0%    0.0%    0.0%    0.0%    0.0%    0.0%    0.0%    0.0%    0.0%    0.0%    0.0%    0.0%        k__Bacteria;p__Chloroflexi; c__Thermobacula       0    0.0%    0.0%    0.0%    0.0%    0.0%    0.0%    0.0%    0.0%    0.0%    0.0%    0.0%    0.0%    0.0%    0.0%    0.0%    0.0%    0.0%    0.0%    0.0%    0.0%    0.0%    0.0%    0.0%    0.0%    0.0%    0.0%    0.0%    0.0%    0.0%    0.0%    0.0%    0.0%    0.0%    0.0%    0.0%    0.0%    0.0%    0.0%    0.0%    0.0%    0.0%    0.0%    0.0%    0.0%    0.0%    0.0%    0.0%    0.0%    0.0%    0.0%    0.0%    0.0%    0.0%    0.0%    0.0%    0.0%    0.0%    0.0%    0.0%    0.0%    0.0%    0.0%    0.0%    0.0%    0.0%    0.0%    0.0%    0.0%    0.0%    0.0%    0.0%    0.0%    0.0%    0.0%    0.0%    0.0%    0.0%    0.0%    0.0%    0.0%    0.0%    0.0%    0.0%    0.0%    0.0%    0.0%    0.0%    0.0%    0.0%    0.0%    0.0%    0.0%    0.0%    0.0%    0.0%    0.0%    0.0%    0.0%    0.0%    0.0%    0.0%    0.0%    0.0%    0.0%    0.0%    0.0%    0.0%    0.0%    0.0%    0.0%    0.0%    0.0%    0.0%    0.0%    0.0%    0.0%    0.0%    0.0%    0.0%    0.0%    0.0%    0.0%    0.0%    0.0%    0.0%    0.0%    0.0%    0.0%    0.0%    0.0%    0.0%    0.0%    0.0%    0.0%        k__Bacteria;p__Chloroflexi; c__Thermomicrobia    4424    0.1%    0.1%    0.1%    0.2%    0.0%    0.1%    0.0%    0.3%    0.1%    0.0%    0.1%    0.1%    0.0%    0.0%    0.0%    0.1%    0.0%    0.0%    0.2%    0.1%    0.1%    0.0%    0.0%    0.1%    0.0%    0.1%    0.1%    0.2%    0.2%    0.0%    0.1%    0.1%    0.1%    0.1%    0.3%    0.0%    0.0%    0.2%    0.1%    0.1%    0.1%    0.0%    0.1%    0.1%    0.1%    0.1%    0.2%    0.1%    0.0%    0.1%    0.1%    0.1%    0.0%    0.1%    0.1%    0.1%    0.1%    0.1%    0.1%    0.2%    0.1%    0.2%    0.0%    0.0%    0.0%    0.1%    0.1%    0.1%    0.1%    0.1%    0.1%    0.2%    0.0%    0.1%    0.1%    0.1%    0.1%    0.0%    0.0%    0.2%    0.1%    0.0%    0.2%    0.0%    0.2%    0.0%    0.1%    0.1%    0.2%    0.1%    0.2%    0.2%    0.1%    0.0%    0.1%    0.1%    0.2%    0.1%    0.2%    0.1%    0.1%    0.1%    0.2%    0.2%    0.1%    0.0%    0.0%    0.1%    0.3%    0.4%    0.1%    0.2%    0.1%    0.1%    0.0%    0.1%    0.1%    0.2%    0.1%    0.1%    0.1%    0.2%    0.1%    0.1%    0.1%    0.2%    0.1%    0.1%    0.2%    0.2%    0.1%    0.1%    0.1%    0.2%        k__Bacteria;p__Cyanobacteria; c__   277838    6.5%    2.6%    5.2%    3.8%    1.2%    2.5%    6.0%    4.9%   12.3%   41.0%    5.0%    5.5%    0.8%   12.0%    1.1%    1.8%    4.1%    0.5%    7.2%    1.0%    4.5%    1.4%    0.4%    2.1%    2.0%    1.9%   15.9%    4.8%    1.8%    1.1%    0.6%    1.6%    3.8%    3.3%    3.8%    1.7%    1.3%    5.9%    2.5%    2.9%   11.0%   15.3%    4.3%    7.7%    2.0%    3.5%    8.6%    1.5%    2.2%    1.7%    5.5%    1.3%    1.3%    7.5%    4.8%    2.6%    3.3%   20.7%    3.6%   16.1%    7.2%    3.6%    1.9%    1.0%   29.7%    5.4%    1.8%    0.9%    4.8%    3.0%   16.0%    2.5%    1.7%    2.4%    3.6%    7.3%    2.5%    2.2%    0.8%    5.7%    2.9%   20.6%    8.3%    8.7%    7.7%   12.8%    2.5%    6.5%    4.5%   14.2%    9.8%   21.8%    2.5%    0.1%    4.1%    5.0%   12.8%    6.6%    5.1%    8.6%    5.1%   10.2%    5.1%    2.9%    4.6%   11.8%   15.5%    6.3%    5.2%    3.0%   14.4%    8.3%    5.8%   15.7%   28.5%    6.4%    4.4%    6.1%   11.3%   14.5%    7.7%    9.7%    4.9%   10.6%    2.0%    1.7%   15.9%    6.3%    7.1%   20.9%    3.6%    8.9%    3.8%    5.7%        k__Bacteria;p__Cyanobacteria; c__S15B-MN24      66    0.0%    0.0%    0.0%    0.0%    0.0%    0.0%    0.0%    0.0%    0.0%    0.0%    0.0%    0.0%    0.0%    0.0%    0.0%    0.0%    0.0%    0.0%    0.0%    0.0%    0.0%    0.0%    0.0%    0.0%    0.0%    0.0%    0.0%    0.0%    0.0%    0.0%    0.0%    0.0%    0.0%    0.0%    0.0%    0.0%    0.0%    0.0%    0.0%    0.0%    0.0%    0.0%    0.0%    0.0%    0.0%    0.0%    0.0%    0.0%    0.0%    0.0%    0.0%    0.0%    0.0%    0.0%    0.0%    0.0%    0.0%    0.0%    0.0%    0.0%    0.0%    0.0%    0.0%    0.0%    0.0%    0.0%    0.0%    0.0%    0.0%    0.0%    0.0%    0.0%    0.0%    0.0%    0.0%    0.0%    0.0%    0.0%    0.0%    0.0%    0.0%    0.0%    0.0%    0.0%    0.0%    0.0%    0.0%    0.0%    0.0%    0.0%    0.0%    0.0%    0.0%    0.0%    0.0%    0.0%    0.0%    0.0%    0.0%    0.0%    0.0%    0.0%    0.0%    0.0%    0.0%    0.0%    0.0%    0.0%    0.0%    0.0%    0.0%    0.0%    0.0%    0.0%    0.0%    0.0%    0.0%    0.0%    0.0%    0.0%    0.0%    0.0%    0.0%    0.0%    0.0%    0.0%    0.0%    0.0%    0.0%    0.0%    0.0%    0.0%    0.0%    0.0%        k__Bacteria;p__Cyanobacteria; c__SM1D11       0    0.0%    0.0%    0.0%    0.0%    0.0%    0.0%    0.0%    0.0%    0.0%    0.0%    0.0%    0.0%    0.0%    0.0%    0.0%    0.0%    0.0%    0.0%    0.0%    0.0%    0.0%    0.0%    0.0%    0.0%    0.0%    0.0%    0.0%    0.0%    0.0%    0.0%    0.0%    0.0%    0.0%    0.0%    0.0%    0.0%    0.0%    0.0%    0.0%    0.0%    0.0%    0.0%    0.0%    0.0%    0.0%    0.0%    0.0%    0.0%    0.0%    0.0%    0.0%    0.0%    0.0%    0.0%    0.0%    0.0%    0.0%    0.0%    0.0%    0.0%    0.0%    0.0%    0.0%    0.0%    0.0%    0.0%    0.0%    0.0%    0.0%    0.0%    0.0%    0.0%    0.0%    0.0%    0.0%    0.0%    0.0%    0.0%    0.0%    0.0%    0.0%    0.0%    0.0%    0.0%    0.0%    0.0%    0.0%    0.0%    0.0%    0.0%    0.0%    0.0%    0.0%    0.0%    0.0%    0.0%    0.0%    0.0%    0.0%    0.0%    0.0%    0.0%    0.0%    0.0%    0.0%    0.0%    0.0%    0.0%    0.0%    0.0%    0.0%    0.0%    0.0%    0.0%    0.0%    0.0%    0.0%    0.0%    0.0%    0.0%    0.0%    0.0%    0.0%    0.0%    0.0%    0.0%    0.0%    0.0%    0.0%    0.0%    0.0%    0.0%    0.0%    0.0%        k__Bacteria;p__Cyanobacteria; c__YS2      21    0.0%    0.0%    0.0%    0.0%    0.0%    0.0%    0.0%    0.0%    0.0%    0.0%    0.0%    0.0%    0.0%    0.0%    0.0%    0.0%    0.0%    0.0%    0.0%    0.0%    0.0%    0.0%    0.0%    0.0%    0.0%    0.0%    0.0%    0.0%    0.0%    0.0%    0.0%    0.0%    0.0%    0.0%    0.0%    0.0%    0.0%    0.0%    0.0%    0.0%    0.0%    0.0%    0.0%    0.0%    0.0%    0.0%    0.0%    0.0%    0.0%    0.0%    0.0%    0.0%    0.0%    0.0%    0.0%    0.0%    0.0%    0.0%    0.0%    0.0%    0.0%    0.0%    0.0%    0.0%    0.0%    0.0%    0.0%    0.0%    0.0%    0.0%    0.0%    0.0%    0.0%    0.0%    0.0%    0.0%    0.0%    0.0%    0.0%    0.0%    0.0%    0.0%    0.0%    0.0%    0.0%    0.0%    0.0%    0.0%    0.0%    0.0%    0.0%    0.0%    0.0%    0.0%    0.0%    0.0%    0.0%    0.0%    0.0%    0.0%    0.0%    0.0%    0.0%    0.0%    0.0%    0.0%    0.0%    0.0%    0.0%    0.0%    0.0%    0.0%    0.0%    0.0%    0.0%    0.0%    0.0%    0.0%    0.0%    0.0%    0.0%    0.0%    0.0%    0.0%    0.0%    0.0%    0.0%    0.0%    0.0%    0.0%    0.0%    0.0%    0.0%    0.0%        k__Bacteria;p__Cyanobacteria; c__mle1-12     164    0.0%    0.0%    0.0%    0.0%    0.0%    0.0%    0.0%    0.0%    0.0%    0.0%    0.0%    0.0%    0.0%    0.0%    0.0%    0.0%    0.0%    0.0%    0.0%    0.0%    0.0%    0.0%    0.0%    0.0%    0.0%    0.0%    0.0%    0.0%    0.0%    0.0%    0.0%    0.0%    0.0%    0.0%    0.0%    0.0%    0.0%    0.0%    0.0%    0.0%    0.0%    0.0%    0.0%    0.0%    0.0%    0.0%    0.1%    0.0%    0.0%    0.0%    0.0%    0.0%    0.0%    0.0%    0.0%    0.0%    0.0%    0.0%    0.0%    0.0%    0.0%    0.0%    0.0%    0.1%    0.0%    0.0%    0.0%    0.0%    0.0%    0.0%    0.0%    0.0%    0.0%    0.0%    0.0%    0.0%    0.0%    0.0%    0.0%    0.0%    0.0%    0.0%    0.0%    0.0%    0.0%    0.0%    0.0%    0.0%    0.0%    0.0%    0.0%    0.0%    0.0%    0.0%    0.0%    0.0%    0.0%    0.0%    0.0%    0.0%    0.0%    0.0%    0.0%    0.0%    0.0%    0.0%    0.0%    0.0%    0.0%    0.0%    0.0%    0.0%    0.0%    0.0%    0.0%    0.0%    0.0%    0.0%    0.0%    0.0%    0.0%    0.0%    0.0%    0.0%    0.0%    0.0%    0.0%    0.0%    0.0%    0.0%    0.0%    0.0%    0.0%    0.0%        k__Bacteria;p__Deferribacteres; c__Deferribacteres (class)     109    0.0%    0.0%    0.0%    0.0%    0.0%    0.0%    0.0%    0.0%    0.0%    0.0%    0.0%    0.0%    0.0%    0.0%    0.0%    0.0%    0.0%    0.0%    0.0%    0.0%    0.0%    0.0%    0.0%    0.0%    0.0%    0.0%    0.0%    0.0%    0.0%    0.0%    0.0%    0.0%    0.0%    0.0%    0.0%    0.0%    0.0%    0.0%    0.0%    0.0%    0.0%    0.0%    0.0%    0.0%    0.0%    0.0%    0.0%    0.0%    0.0%    0.0%    0.0%    0.0%    0.0%    0.0%    0.0%    0.0%    0.0%    0.0%    0.0%    0.0%    0.0%    0.0%    0.0%    0.0%    0.0%    0.0%    0.0%    0.0%    0.0%    0.0%    0.0%    0.0%    0.0%    0.0%    0.0%    0.0%    0.0%    0.0%    0.0%    0.0%    0.0%    0.0%    0.0%    0.0%    0.0%    0.0%    0.0%    0.0%    0.0%    0.0%    0.0%    0.0%    0.0%    0.0%    0.0%    0.0%    0.0%    0.0%    0.0%    0.0%    0.0%    0.0%    0.0%    0.0%    0.0%    0.0%    0.0%    0.0%    0.0%    0.0%    0.0%    0.0%    0.0%    0.0%    0.0%    0.0%    0.0%    0.0%    0.0%    0.0%    0.0%    0.0%    0.0%    0.0%    0.0%    0.0%    0.0%    0.0%    0.0%    0.0%    0.0%    0.0%    0.0%    0.0%        k__Bacteria;p__Elusimicrobia; c__Elusimicrobia (class)      18    0.0%    0.0%    0.0%    0.0%    0.0%    0.0%    0.0%    0.0%    0.0%    0.0%    0.0%    0.0%    0.0%    0.0%    0.0%    0.0%    0.0%    0.0%    0.0%    0.0%    0.0%    0.0%    0.0%    0.0%    0.0%    0.0%    0.0%    0.0%    0.0%    0.0%    0.0%    0.0%    0.0%    0.0%    0.0%    0.0%    0.0%    0.0%    0.0%    0.0%    0.0%    0.0%    0.0%    0.0%    0.0%    0.0%    0.0%    0.0%    0.0%    0.0%    0.0%    0.0%    0.0%    0.0%    0.0%    0.0%    0.0%    0.0%    0.0%    0.0%    0.0%    0.0%    0.0%    0.0%    0.0%    0.0%    0.0%    0.0%    0.0%    0.0%    0.0%    0.0%    0.0%    0.0%    0.0%    0.0%    0.0%    0.0%    0.0%    0.0%    0.0%    0.0%    0.0%    0.0%    0.0%    0.0%    0.0%    0.0%    0.0%    0.0%    0.0%    0.0%    0.0%    0.0%    0.0%    0.0%    0.0%    0.0%    0.0%    0.0%    0.0%    0.0%    0.0%    0.0%    0.0%    0.0%    0.0%    0.0%    0.0%    0.0%    0.0%    0.0%    0.0%    0.0%    0.0%    0.0%    0.0%    0.0%    0.0%    0.0%    0.0%    0.0%    0.0%    0.0%    0.0%    0.0%    0.0%    0.0%    0.0%    0.0%    0.0%    0.0%    0.0%    0.0%        k__Bacteria;p__Fibrobacteres; c__Fibrobacteres (class)      14    0.0%    0.0%    0.0%    0.0%    0.0%    0.0%    0.0%    0.0%    0.0%    0.0%    0.0%    0.0%    0.0%    0.0%    0.0%    0.0%    0.0%    0.0%    0.0%    0.0%    0.0%    0.0%    0.0%    0.0%    0.0%    0.0%    0.0%    0.0%    0.0%    0.0%    0.0%    0.0%    0.0%    0.0%    0.0%    0.0%    0.0%    0.0%    0.0%    0.0%    0.0%    0.0%    0.0%    0.0%    0.0%    0.0%    0.0%    0.0%    0.0%    0.0%    0.0%    0.0%    0.0%    0.0%    0.0%    0.0%    0.0%    0.0%    0.0%    0.0%    0.0%    0.0%    0.0%    0.0%    0.0%    0.0%    0.0%    0.0%    0.0%    0.0%    0.0%    0.0%    0.0%    0.0%    0.0%    0.0%    0.0%    0.0%    0.0%    0.0%    0.0%    0.0%    0.0%    0.0%    0.0%    0.0%    0.0%    0.0%    0.0%    0.0%    0.0%    0.0%    0.0%    0.0%    0.0%    0.0%    0.0%    0.0%    0.0%    0.0%    0.0%    0.0%    0.0%    0.0%    0.0%    0.0%    0.0%    0.0%    0.0%    0.0%    0.0%    0.0%    0.0%    0.0%    0.0%    0.0%    0.0%    0.0%    0.0%    0.0%    0.0%    0.0%    0.0%    0.0%    0.0%    0.0%    0.0%    0.0%    0.0%    0.0%    0.0%    0.0%    0.0%    0.0%        k__Bacteria;p__Firmicutes; c__Bacilli   971753   22.6%   30.0%   19.2%   34.4%   45.2%   20.7%   42.1%   21.3%   37.4%   23.1%   34.1%   22.4%   46.7%   30.5%   29.7%   34.9%   21.2%   12.2%   19.0%   24.1%    9.6%   24.6%   14.5%   22.3%   21.9%   15.3%   26.6%   13.3%   19.8%   28.8%   28.0%   21.5%   13.4%   15.6%   17.5%   36.0%   20.8%   17.5%   23.6%   15.5%   18.3%   24.4%   10.1%   18.0%    9.8%   23.6%   12.2%   17.2%   29.1%   18.7%   23.8%   29.0%   25.0%   17.1%   24.6%   20.7%   12.7%   16.4%   17.6%   13.1%   15.6%   19.1%   39.7%   39.1%   21.0%   38.3%   28.1%   30.8%   11.1%   11.9%   14.4%   26.7%   32.6%   22.5%   26.5%   22.2%   14.1%   46.6%   38.3%   17.4%   29.6%   31.7%   18.2%   31.7%   21.5%   31.5%   24.3%   16.4%   17.1%   15.5%   21.9%   17.2%   14.4%   20.6%    7.9%   35.1%   12.5%   10.8%   14.3%   32.8%   19.2%   25.9%   23.1%   24.2%   15.1%   11.4%   19.7%   32.4%   20.7%   13.5%   15.8%   17.1%   28.9%   20.3%    8.3%   21.6%   10.7%   16.4%   21.8%   39.6%   14.0%   20.1%   32.7%   14.7%    9.5%   11.1%   17.7%   21.0%   16.9%    7.6%   26.3%   27.0%   40.6%   30.3%        k__Bacteria;p__Firmicutes; c__Clostridia   336626    7.8%    9.0%    8.7%    7.2%    6.3%    5.7%    4.6%    4.3%    3.7%    2.4%    5.6%    7.4%    8.1%    6.3%   11.2%    8.2%    4.8%    6.1%    8.1%   12.9%   10.9%    9.4%    8.9%    5.0%    7.1%    5.5%    2.5%    3.4%   11.9%    9.0%    7.6%    7.2%    5.6%   14.7%    4.4%    3.6%   31.4%    5.5%   10.7%    6.1%    6.8%    6.0%    2.6%    4.5%   10.4%    6.3%    8.0%   15.2%    8.3%    8.4%    5.3%    7.4%   16.7%    7.0%    5.5%    6.6%   15.8%    3.6%    7.8%    5.1%    6.3%   14.7%    8.7%   11.5%    6.4%    7.4%    7.7%   10.1%    4.4%    4.3%    6.4%    7.9%   10.9%    7.8%    8.5%    9.3%    4.9%    9.7%    6.7%    3.8%    9.5%    5.4%    6.6%   11.6%    8.1%    7.1%    6.5%    9.3%    9.9%    8.4%    5.7%    5.7%   23.6%   20.9%   29.7%    7.1%    8.0%   14.4%   15.6%    8.2%   17.4%    4.4%   11.3%    9.5%    6.2%    4.9%    5.2%    4.7%    4.4%    2.8%    4.8%    4.4%    9.1%    5.1%    1.9%    5.3%    8.6%    3.8%    5.3%    3.6%    3.7%    5.4%    5.2%    4.1%    4.9%    2.9%    4.6%    8.1%    5.4%    2.5%    3.3%    4.5%    2.6%    3.1%        k__Bacteria;p__Fusobacteria; c__Fusobacteria (class)   138028    3.2%    5.4%    4.6%    2.0%    1.4%    1.6%    0.6%    1.4%    1.5%    1.7%    1.6%    2.6%    0.8%    1.7%    3.1%    2.6%    0.6%    0.3%    1.5%    0.6%    0.4%    1.4%   25.6%    0.9%    1.9%    1.4%    0.4%    0.8%    7.9%    0.9%    4.4%    1.4%    1.2%    0.6%    0.9%    1.1%    0.9%    8.0%    4.4%   20.7%    5.8%    2.7%    4.0%    9.4%   19.6%   13.6%    5.0%    6.0%   11.4%   11.9%    7.3%   13.4%    1.5%    5.5%    3.9%    3.8%    7.8%    2.8%    1.6%    6.3%    5.2%    4.7%    2.9%    8.6%    1.9%    2.4%    6.1%    1.2%    2.4%    2.0%    2.7%    2.9%    1.9%    2.3%    3.0%    5.3%    2.5%    2.2%    1.8%    1.7%    3.4%    2.7%    1.7%    1.6%    2.0%    2.4%    2.6%    3.5%    3.9%    5.5%    1.1%    2.1%    4.3%    0.7%    5.0%    3.3%    1.2%    8.1%    5.4%    2.1%    5.3%    1.4%    2.9%    0.9%    1.5%    0.8%    0.7%    0.8%    0.9%    0.2%    0.9%    0.9%    3.2%    0.8%    0.2%    1.5%    0.8%    0.7%    1.4%    1.0%    0.8%    1.2%    1.3%    1.3%    4.6%    1.5%    1.5%    1.2%    2.1%    0.8%    1.2%    1.5%    0.7%    1.5%        k__Bacteria;p__GN02; c__       0    0.0%    0.0%    0.0%    0.0%    0.0%    0.0%    0.0%    0.0%    0.0%    0.0%    0.0%    0.0%    0.0%    0.0%    0.0%    0.0%    0.0%    0.0%    0.0%    0.0%    0.0%    0.0%    0.0%    0.0%    0.0%    0.0%    0.0%    0.0%    0.0%    0.0%    0.0%    0.0%    0.0%    0.0%    0.0%    0.0%    0.0%    0.0%    0.0%    0.0%    0.0%    0.0%    0.0%    0.0%    0.0%    0.0%    0.0%    0.0%    0.0%    0.0%    0.0%    0.0%    0.0%    0.0%    0.0%    0.0%    0.0%    0.0%    0.0%    0.0%    0.0%    0.0%    0.0%    0.0%    0.0%    0.0%    0.0%    0.0%    0.0%    0.0%    0.0%    0.0%    0.0%    0.0%    0.0%    0.0%    0.0%    0.0%    0.0%    0.0%    0.0%    0.0%    0.0%    0.0%    0.0%    0.0%    0.0%    0.0%    0.0%    0.0%    0.0%    0.0%    0.0%    0.0%    0.0%    0.0%    0.0%    0.0%    0.0%    0.0%    0.0%    0.0%    0.0%    0.0%    0.0%    0.0%    0.0%    0.0%    0.0%    0.0%    0.0%    0.0%    0.0%    0.0%    0.0%    0.0%    0.0%    0.0%    0.0%    0.0%    0.0%    0.0%    0.0%    0.0%    0.0%    0.0%    0.0%    0.0%    0.0%    0.0%    0.0%    0.0%    0.0%    0.0%        k__Bacteria;p__GN02; c__VC12-cl04       0    0.0%    0.0%    0.0%    0.0%    0.0%    0.0%    0.0%    0.0%    0.0%    0.0%    0.0%    0.0%    0.0%    0.0%    0.0%    0.0%    0.0%    0.0%    0.0%    0.0%    0.0%    0.0%    0.0%    0.0%    0.0%    0.0%    0.0%    0.0%    0.0%    0.0%    0.0%    0.0%    0.0%    0.0%    0.0%    0.0%    0.0%    0.0%    0.0%    0.0%    0.0%    0.0%    0.0%    0.0%    0.0%    0.0%    0.0%    0.0%    0.0%    0.0%    0.0%    0.0%    0.0%    0.0%    0.0%    0.0%    0.0%    0.0%    0.0%    0.0%    0.0%    0.0%    0.0%    0.0%    0.0%    0.0%    0.0%    0.0%    0.0%    0.0%    0.0%    0.0%    0.0%    0.0%    0.0%    0.0%    0.0%    0.0%    0.0%    0.0%    0.0%    0.0%    0.0%    0.0%    0.0%    0.0%    0.0%    0.0%    0.0%    0.0%    0.0%    0.0%    0.0%    0.0%    0.0%    0.0%    0.0%    0.0%    0.0%    0.0%    0.0%    0.0%    0.0%    0.0%    0.0%    0.0%    0.0%    0.0%    0.0%    0.0%    0.0%    0.0%    0.0%    0.0%    0.0%    0.0%    0.0%    0.0%    0.0%    0.0%    0.0%    0.0%    0.0%    0.0%    0.0%    0.0%    0.0%    0.0%    0.0%    0.0%    0.0%    0.0%    0.0%    0.0%        k__Bacteria;p__Gemmatimonadetes; c__Gemmatimonadetes (class)    2480    0.1%    0.1%    0.0%    0.0%    0.0%    0.1%    0.0%    0.2%    0.0%    0.0%    0.1%    0.0%    0.0%    0.0%    0.0%    0.0%    0.0%    0.0%    0.1%    0.0%    0.1%    0.0%    0.0%    0.0%    0.0%    0.1%    0.0%    0.1%    0.1%    0.0%    0.0%    0.0%    0.0%    0.0%    0.0%    0.0%    0.0%    0.0%    0.0%    0.1%    0.0%    0.0%    0.1%    0.1%    0.0%    0.0%    0.1%    0.0%    0.0%    0.0%    0.1%    0.0%    0.0%    0.1%    0.1%    0.1%    0.1%    0.0%    0.0%    0.1%    0.1%    0.2%    0.0%    0.0%    0.0%    0.0%    0.0%    0.1%    0.2%    0.1%    0.1%    0.1%    0.0%    0.1%    0.1%    0.0%    0.1%    0.0%    0.0%    0.2%    0.1%    0.0%    0.1%    0.0%    0.1%    0.1%    0.1%    0.1%    0.1%    0.1%    0.1%    0.1%    0.1%    0.0%    0.0%    0.0%    0.1%    0.1%    0.2%    0.1%    0.0%    0.0%    0.1%    0.1%    0.1%    0.0%    0.0%    0.0%    0.2%    0.1%    0.2%    0.2%    0.1%    0.1%    0.0%    0.1%    0.0%    0.2%    0.1%    0.0%    0.1%    0.1%    0.0%    0.1%    0.1%    0.1%    0.1%    0.1%    0.1%    0.1%    0.1%    0.1%    0.0%    0.0%        k__Bacteria;p__Lentisphaerae; c__       0    0.0%    0.0%    0.0%    0.0%    0.0%    0.0%    0.0%    0.0%    0.0%    0.0%    0.0%    0.0%    0.0%    0.0%    0.0%    0.0%    0.0%    0.0%    0.0%    0.0%    0.0%    0.0%    0.0%    0.0%    0.0%    0.0%    0.0%    0.0%    0.0%    0.0%    0.0%    0.0%    0.0%    0.0%    0.0%    0.0%    0.0%    0.0%    0.0%    0.0%    0.0%    0.0%    0.0%    0.0%    0.0%    0.0%    0.0%    0.0%    0.0%    0.0%    0.0%    0.0%    0.0%    0.0%    0.0%    0.0%    0.0%    0.0%    0.0%    0.0%    0.0%    0.0%    0.0%    0.0%    0.0%    0.0%    0.0%    0.0%    0.0%    0.0%    0.0%    0.0%    0.0%    0.0%    0.0%    0.0%    0.0%    0.0%    0.0%    0.0%    0.0%    0.0%    0.0%    0.0%    0.0%    0.0%    0.0%    0.0%    0.0%    0.0%    0.0%    0.0%    0.0%    0.0%    0.0%    0.0%    0.0%    0.0%    0.0%    0.0%    0.0%    0.0%    0.0%    0.0%    0.0%    0.0%    0.0%    0.0%    0.0%    0.0%    0.0%    0.0%    0.0%    0.0%    0.0%    0.0%    0.0%    0.0%    0.0%    0.0%    0.0%    0.0%    0.0%    0.0%    0.0%    0.0%    0.0%    0.0%    0.0%    0.0%    0.0%    0.0%    0.0%    0.0%        k__Bacteria;p__Lentisphaerae; c__Lentisphaerae (class)       0    0.0%    0.0%    0.0%    0.0%    0.0%    0.0%    0.0%    0.0%    0.0%    0.0%    0.0%    0.0%    0.0%    0.0%    0.0%    0.0%    0.0%    0.0%    0.0%    0.0%    0.0%    0.0%    0.0%    0.0%    0.0%    0.0%    0.0%    0.0%    0.0%    0.0%    0.0%    0.0%    0.0%    0.0%    0.0%    0.0%    0.0%    0.0%    0.0%    0.0%    0.0%    0.0%    0.0%    0.0%    0.0%    0.0%    0.0%    0.0%    0.0%    0.0%    0.0%    0.0%    0.0%    0.0%    0.0%    0.0%    0.0%    0.0%    0.0%    0.0%    0.0%    0.0%    0.0%    0.0%    0.0%    0.0%    0.0%    0.0%    0.0%    0.0%    0.0%    0.0%    0.0%    0.0%    0.0%    0.0%    0.0%    0.0%    0.0%    0.0%    0.0%    0.0%    0.0%    0.0%    0.0%    0.0%    0.0%    0.0%    0.0%    0.0%    0.0%    0.0%    0.0%    0.0%    0.0%    0.0%    0.0%    0.0%    0.0%    0.0%    0.0%    0.0%    0.0%    0.0%    0.0%    0.0%    0.0%    0.0%    0.0%    0.0%    0.0%    0.0%    0.0%    0.0%    0.0%    0.0%    0.0%    0.0%    0.0%    0.0%    0.0%    0.0%    0.0%    0.0%    0.0%    0.0%    0.0%    0.0%    0.0%    0.0%    0.0%    0.0%    0.0%    0.0%        k__Bacteria;p__MVP-15; c__       3    0.0%    0.0%    0.0%    0.0%    0.0%    0.0%    0.0%    0.0%    0.0%    0.0%    0.0%    0.0%    0.0%    0.0%    0.0%    0.0%    0.0%    0.0%    0.0%    0.0%    0.0%    0.0%    0.0%    0.0%    0.0%    0.0%    0.0%    0.0%    0.0%    0.0%    0.0%    0.0%    0.0%    0.0%    0.0%    0.0%    0.0%    0.0%    0.0%    0.0%    0.0%    0.0%    0.0%    0.0%    0.0%    0.0%    0.0%    0.0%    0.0%    0.0%    0.0%    0.0%    0.0%    0.0%    0.0%    0.0%    0.0%    0.0%    0.0%    0.0%    0.0%    0.0%    0.0%    0.0%    0.0%    0.0%    0.0%    0.0%    0.0%    0.0%    0.0%    0.0%    0.0%    0.0%    0.0%    0.0%    0.0%    0.0%    0.0%    0.0%    0.0%    0.0%    0.0%    0.0%    0.0%    0.0%    0.0%    0.0%    0.0%    0.0%    0.0%    0.0%    0.0%    0.0%    0.0%    0.0%    0.0%    0.0%    0.0%    0.0%    0.0%    0.0%    0.0%    0.0%    0.0%    0.0%    0.0%    0.0%    0.0%    0.0%    0.0%    0.0%    0.0%    0.0%    0.0%    0.0%    0.0%    0.0%    0.0%    0.0%    0.0%    0.0%    0.0%    0.0%    0.0%    0.0%    0.0%    0.0%    0.0%    0.0%    0.0%    0.0%    0.0%    0.0%        k__Bacteria;p__NC10; c__       1    0.0%    0.0%    0.0%    0.0%    0.0%    0.0%    0.0%    0.0%    0.0%    0.0%    0.0%    0.0%    0.0%    0.0%    0.0%    0.0%    0.0%    0.0%    0.0%    0.0%    0.0%    0.0%    0.0%    0.0%    0.0%    0.0%    0.0%    0.0%    0.0%    0.0%    0.0%    0.0%    0.0%    0.0%    0.0%    0.0%    0.0%    0.0%    0.0%    0.0%    0.0%    0.0%    0.0%    0.0%    0.0%    0.0%    0.0%    0.0%    0.0%    0.0%    0.0%    0.0%    0.0%    0.0%    0.0%    0.0%    0.0%    0.0%    0.0%    0.0%    0.0%    0.0%    0.0%    0.0%    0.0%    0.0%    0.0%    0.0%    0.0%    0.0%    0.0%    0.0%    0.0%    0.0%    0.0%    0.0%    0.0%    0.0%    0.0%    0.0%    0.0%    0.0%    0.0%    0.0%    0.0%    0.0%    0.0%    0.0%    0.0%    0.0%    0.0%    0.0%    0.0%    0.0%    0.0%    0.0%    0.0%    0.0%    0.0%    0.0%    0.0%    0.0%    0.0%    0.0%    0.0%    0.0%    0.0%    0.0%    0.0%    0.0%    0.0%    0.0%    0.0%    0.0%    0.0%    0.0%    0.0%    0.0%    0.0%    0.0%    0.0%    0.0%    0.0%    0.0%    0.0%    0.0%    0.0%    0.0%    0.0%    0.0%    0.0%    0.0%    0.0%    0.0%        k__Bacteria;p__NKB19; c__       8    0.0%    0.0%    0.0%    0.0%    0.0%    0.0%    0.0%    0.0%    0.0%    0.0%    0.0%    0.0%    0.0%    0.0%    0.0%    0.0%    0.0%    0.0%    0.0%    0.0%    0.0%    0.0%    0.0%    0.0%    0.0%    0.0%    0.0%    0.0%    0.0%    0.0%    0.0%    0.0%    0.0%    0.0%    0.0%    0.0%    0.0%    0.0%    0.0%    0.0%    0.0%    0.0%    0.0%    0.0%    0.0%    0.0%    0.0%    0.0%    0.0%    0.0%    0.0%    0.0%    0.0%    0.0%    0.0%    0.0%    0.0%    0.0%    0.0%    0.0%    0.0%    0.0%    0.0%    0.0%    0.0%    0.0%    0.0%    0.0%    0.0%    0.0%    0.0%    0.0%    0.0%    0.0%    0.0%    0.0%    0.0%    0.0%    0.0%    0.0%    0.0%    0.0%    0.0%    0.0%    0.0%    0.0%    0.0%    0.0%    0.0%    0.0%    0.0%    0.0%    0.0%    0.0%    0.0%    0.0%    0.0%    0.0%    0.0%    0.0%    0.0%    0.0%    0.0%    0.0%    0.0%    0.0%    0.0%    0.0%    0.0%    0.0%    0.0%    0.0%    0.0%    0.0%    0.0%    0.0%    0.0%    0.0%    0.0%    0.0%    0.0%    0.0%    0.0%    0.0%    0.0%    0.0%    0.0%    0.0%    0.0%    0.0%    0.0%    0.0%    0.0%    0.0%        k__Bacteria;p__Nitrospirae; c__Nitrospira (class)     492    0.0%    0.0%    0.0%    0.0%    0.0%    0.1%    0.0%    0.0%    0.0%    0.0%    0.0%    0.0%    0.0%    0.0%    0.0%    0.0%    0.0%    0.0%    0.0%    0.0%    0.0%    0.0%    0.0%    0.0%    0.0%    0.0%    0.0%    0.0%    0.0%    0.0%    0.0%    0.0%    0.0%    0.0%    0.0%    0.0%    0.0%    0.0%    0.0%    0.0%    0.0%    0.0%    0.0%    0.0%    0.0%    0.0%    0.0%    0.0%    0.0%    0.0%    0.0%    0.0%    0.0%    0.0%    0.0%    0.0%    0.0%    0.0%    0.0%    0.0%    0.0%    0.0%    0.0%    0.0%    0.0%    0.0%    0.0%    0.0%    0.1%    0.0%    0.0%    0.0%    0.0%    0.0%    0.0%    0.0%    0.0%    0.0%    0.0%    0.0%    0.0%    0.0%    0.0%    0.0%    0.0%    0.0%    0.0%    0.0%    0.0%    0.0%    0.0%    0.0%    0.0%    0.0%    0.0%    0.0%    0.0%    0.0%    0.0%    0.0%    0.0%    0.0%    0.0%    0.0%    0.0%    0.0%    0.0%    0.0%    0.1%    0.0%    0.0%    0.1%    0.0%    0.0%    0.0%    0.0%    0.0%    0.0%    0.0%    0.0%    0.0%    0.0%    0.0%    0.0%    0.0%    0.0%    0.0%    0.0%    0.0%    0.0%    0.0%    0.0%    0.0%    0.0%        k__Bacteria;p__OP10; c__       0    0.0%    0.0%    0.0%    0.0%    0.0%    0.0%    0.0%    0.0%    0.0%    0.0%    0.0%    0.0%    0.0%    0.0%    0.0%    0.0%    0.0%    0.0%    0.0%    0.0%    0.0%    0.0%    0.0%    0.0%    0.0%    0.0%    0.0%    0.0%    0.0%    0.0%    0.0%    0.0%    0.0%    0.0%    0.0%    0.0%    0.0%    0.0%    0.0%    0.0%    0.0%    0.0%    0.0%    0.0%    0.0%    0.0%    0.0%    0.0%    0.0%    0.0%    0.0%    0.0%    0.0%    0.0%    0.0%    0.0%    0.0%    0.0%    0.0%    0.0%    0.0%    0.0%    0.0%    0.0%    0.0%    0.0%    0.0%    0.0%    0.0%    0.0%    0.0%    0.0%    0.0%    0.0%    0.0%    0.0%    0.0%    0.0%    0.0%    0.0%    0.0%    0.0%    0.0%    0.0%    0.0%    0.0%    0.0%    0.0%    0.0%    0.0%    0.0%    0.0%    0.0%    0.0%    0.0%    0.0%    0.0%    0.0%    0.0%    0.0%    0.0%    0.0%    0.0%    0.0%    0.0%    0.0%    0.0%    0.0%    0.0%    0.0%    0.0%    0.0%    0.0%    0.0%    0.0%    0.0%    0.0%    0.0%    0.0%    0.0%    0.0%    0.0%    0.0%    0.0%    0.0%    0.0%    0.0%    0.0%    0.0%    0.0%    0.0%    0.0%    0.0%    0.0%        k__Bacteria;p__OP10; c__5B-18      30    0.0%    0.0%    0.0%    0.0%    0.0%    0.0%    0.0%    0.0%    0.0%    0.0%    0.0%    0.0%    0.0%    0.0%    0.0%    0.0%    0.0%    0.0%    0.0%    0.0%    0.0%    0.0%    0.0%    0.0%    0.0%    0.0%    0.0%    0.0%    0.0%    0.0%    0.0%    0.0%    0.0%    0.0%    0.0%    0.0%    0.0%    0.0%    0.0%    0.0%    0.0%    0.0%    0.0%    0.0%    0.0%    0.0%    0.0%    0.0%    0.0%    0.0%    0.0%    0.0%    0.0%    0.0%    0.0%    0.0%    0.0%    0.0%    0.0%    0.0%    0.0%    0.0%    0.0%    0.0%    0.0%    0.0%    0.0%    0.0%    0.0%    0.0%    0.0%    0.0%    0.0%    0.0%    0.0%    0.0%    0.0%    0.0%    0.0%    0.0%    0.0%    0.0%    0.0%    0.0%    0.0%    0.0%    0.0%    0.0%    0.0%    0.0%    0.0%    0.0%    0.0%    0.0%    0.0%    0.0%    0.0%    0.0%    0.0%    0.0%    0.0%    0.0%    0.0%    0.0%    0.0%    0.0%    0.0%    0.0%    0.0%    0.0%    0.0%    0.0%    0.0%    0.0%    0.0%    0.0%    0.0%    0.0%    0.0%    0.0%    0.0%    0.0%    0.0%    0.0%    0.0%    0.0%    0.0%    0.0%    0.0%    0.0%    0.0%    0.0%    0.0%    0.0%        k__Bacteria;p__OP10; c__CH21      67    0.0%    0.0%    0.0%    0.0%    0.0%    0.0%    0.0%    0.0%    0.0%    0.0%    0.0%    0.0%    0.0%    0.0%    0.0%    0.0%    0.0%    0.0%    0.0%    0.0%    0.0%    0.0%    0.0%    0.0%    0.0%    0.0%    0.0%    0.0%    0.0%    0.0%    0.0%    0.0%    0.0%    0.0%    0.0%    0.0%    0.0%    0.0%    0.0%    0.0%    0.0%    0.0%    0.0%    0.0%    0.0%    0.0%    0.0%    0.0%    0.0%    0.0%    0.0%    0.0%    0.0%    0.0%    0.0%    0.0%    0.0%    0.0%    0.0%    0.0%    0.0%    0.0%    0.0%    0.0%    0.0%    0.0%    0.0%    0.0%    0.0%    0.0%    0.0%    0.0%    0.0%    0.0%    0.0%    0.0%    0.0%    0.0%    0.0%    0.0%    0.0%    0.0%    0.0%    0.0%    0.0%    0.0%    0.0%    0.0%    0.0%    0.0%    0.0%    0.0%    0.0%    0.0%    0.0%    0.0%    0.0%    0.0%    0.0%    0.0%    0.0%    0.0%    0.0%    0.0%    0.0%    0.0%    0.0%    0.0%    0.0%    0.0%    0.0%    0.0%    0.0%    0.0%    0.0%    0.0%    0.0%    0.0%    0.0%    0.0%    0.0%    0.0%    0.0%    0.0%    0.0%    0.0%    0.0%    0.0%    0.0%    0.0%    0.0%    0.0%    0.0%    0.0%        k__Bacteria;p__OP10; c__CL500-48     157    0.0%    0.0%    0.0%    0.0%    0.0%    0.0%    0.0%    0.0%    0.0%    0.0%    0.0%    0.0%    0.0%    0.0%    0.0%    0.0%    0.0%    0.0%    0.0%    0.0%    0.0%    0.0%    0.0%    0.0%    0.0%    0.0%    0.0%    0.0%    0.0%    0.0%    0.0%    0.0%    0.0%    0.0%    0.0%    0.0%    0.0%    0.0%    0.0%    0.0%    0.0%    0.0%    0.0%    0.0%    0.0%    0.0%    0.0%    0.0%    0.0%    0.0%    0.0%    0.0%    0.0%    0.0%    0.0%    0.0%    0.0%    0.0%    0.0%    0.0%    0.0%    0.0%    0.0%    0.0%    0.0%    0.0%    0.0%    0.0%    0.0%    0.0%    0.0%    0.0%    0.0%    0.0%    0.0%    0.0%    0.0%    0.0%    0.0%    0.0%    0.0%    0.0%    0.0%    0.0%    0.0%    0.0%    0.0%    0.0%    0.0%    0.0%    0.0%    0.0%    0.0%    0.0%    0.0%    0.0%    0.0%    0.0%    0.0%    0.0%    0.0%    0.0%    0.0%    0.0%    0.0%    0.0%    0.0%    0.0%    0.0%    0.0%    0.0%    0.0%    0.0%    0.0%    0.0%    0.0%    0.0%    0.0%    0.0%    0.0%    0.0%    0.0%    0.0%    0.0%    0.0%    0.0%    0.0%    0.0%    0.0%    0.0%    0.0%    0.0%    0.0%    0.0%        k__Bacteria;p__OP10; c__OS-L       1    0.0%    0.0%    0.0%    0.0%    0.0%    0.0%    0.0%    0.0%    0.0%    0.0%    0.0%    0.0%    0.0%    0.0%    0.0%    0.0%    0.0%    0.0%    0.0%    0.0%    0.0%    0.0%    0.0%    0.0%    0.0%    0.0%    0.0%    0.0%    0.0%    0.0%    0.0%    0.0%    0.0%    0.0%    0.0%    0.0%    0.0%    0.0%    0.0%    0.0%    0.0%    0.0%    0.0%    0.0%    0.0%    0.0%    0.0%    0.0%    0.0%    0.0%    0.0%    0.0%    0.0%    0.0%    0.0%    0.0%    0.0%    0.0%    0.0%    0.0%    0.0%    0.0%    0.0%    0.0%    0.0%    0.0%    0.0%    0.0%    0.0%    0.0%    0.0%    0.0%    0.0%    0.0%    0.0%    0.0%    0.0%    0.0%    0.0%    0.0%    0.0%    0.0%    0.0%    0.0%    0.0%    0.0%    0.0%    0.0%    0.0%    0.0%    0.0%    0.0%    0.0%    0.0%    0.0%    0.0%    0.0%    0.0%    0.0%    0.0%    0.0%    0.0%    0.0%    0.0%    0.0%    0.0%    0.0%    0.0%    0.0%    0.0%    0.0%    0.0%    0.0%    0.0%    0.0%    0.0%    0.0%    0.0%    0.0%    0.0%    0.0%    0.0%    0.0%    0.0%    0.0%    0.0%    0.0%    0.0%    0.0%    0.0%    0.0%    0.0%    0.0%    0.0%        k__Bacteria;p__OP10; c__S1a-1H      11    0.0%    0.0%    0.0%    0.0%    0.0%    0.0%    0.0%    0.0%    0.0%    0.0%    0.0%    0.0%    0.0%    0.0%    0.0%    0.0%    0.0%    0.0%    0.0%    0.0%    0.0%    0.0%    0.0%    0.0%    0.0%    0.0%    0.0%    0.0%    0.0%    0.0%    0.0%    0.0%    0.0%    0.0%    0.0%    0.0%    0.0%    0.0%    0.0%    0.0%    0.0%    0.0%    0.0%    0.0%    0.0%    0.0%    0.0%    0.0%    0.0%    0.0%    0.0%    0.0%    0.0%    0.0%    0.0%    0.0%    0.0%    0.0%    0.0%    0.0%    0.0%    0.0%    0.0%    0.0%    0.0%    0.0%    0.0%    0.0%    0.0%    0.0%    0.0%    0.0%    0.0%    0.0%    0.0%    0.0%    0.0%    0.0%    0.0%    0.0%    0.0%    0.0%    0.0%    0.0%    0.0%    0.0%    0.0%    0.0%    0.0%    0.0%    0.0%    0.0%    0.0%    0.0%    0.0%    0.0%    0.0%    0.0%    0.0%    0.0%    0.0%    0.0%    0.0%    0.0%    0.0%    0.0%    0.0%    0.0%    0.0%    0.0%    0.0%    0.0%    0.0%    0.0%    0.0%    0.0%    0.0%    0.0%    0.0%    0.0%    0.0%    0.0%    0.0%    0.0%    0.0%    0.0%    0.0%    0.0%    0.0%    0.0%    0.0%    0.0%    0.0%    0.0%        k__Bacteria;p__OP10; c__SJA-176       0    0.0%    0.0%    0.0%    0.0%    0.0%    0.0%    0.0%    0.0%    0.0%    0.0%    0.0%    0.0%    0.0%    0.0%    0.0%    0.0%    0.0%    0.0%    0.0%    0.0%    0.0%    0.0%    0.0%    0.0%    0.0%    0.0%    0.0%    0.0%    0.0%    0.0%    0.0%    0.0%    0.0%    0.0%    0.0%    0.0%    0.0%    0.0%    0.0%    0.0%    0.0%    0.0%    0.0%    0.0%    0.0%    0.0%    0.0%    0.0%    0.0%    0.0%    0.0%    0.0%    0.0%    0.0%    0.0%    0.0%    0.0%    0.0%    0.0%    0.0%    0.0%    0.0%    0.0%    0.0%    0.0%    0.0%    0.0%    0.0%    0.0%    0.0%    0.0%    0.0%    0.0%    0.0%    0.0%    0.0%    0.0%    0.0%    0.0%    0.0%    0.0%    0.0%    0.0%    0.0%    0.0%    0.0%    0.0%    0.0%    0.0%    0.0%    0.0%    0.0%    0.0%    0.0%    0.0%    0.0%    0.0%    0.0%    0.0%    0.0%    0.0%    0.0%    0.0%    0.0%    0.0%    0.0%    0.0%    0.0%    0.0%    0.0%    0.0%    0.0%    0.0%    0.0%    0.0%    0.0%    0.0%    0.0%    0.0%    0.0%    0.0%    0.0%    0.0%    0.0%    0.0%    0.0%    0.0%    0.0%    0.0%    0.0%    0.0%    0.0%    0.0%    0.0%        k__Bacteria;p__OP10; c__SJA-22       0    0.0%    0.0%    0.0%    0.0%    0.0%    0.0%    0.0%    0.0%    0.0%    0.0%    0.0%    0.0%    0.0%    0.0%    0.0%    0.0%    0.0%    0.0%    0.0%    0.0%    0.0%    0.0%    0.0%    0.0%    0.0%    0.0%    0.0%    0.0%    0.0%    0.0%    0.0%    0.0%    0.0%    0.0%    0.0%    0.0%    0.0%    0.0%    0.0%    0.0%    0.0%    0.0%    0.0%    0.0%    0.0%    0.0%    0.0%    0.0%    0.0%    0.0%    0.0%    0.0%    0.0%    0.0%    0.0%    0.0%    0.0%    0.0%    0.0%    0.0%    0.0%    0.0%    0.0%    0.0%    0.0%    0.0%    0.0%    0.0%    0.0%    0.0%    0.0%    0.0%    0.0%    0.0%    0.0%    0.0%    0.0%    0.0%    0.0%    0.0%    0.0%    0.0%    0.0%    0.0%    0.0%    0.0%    0.0%    0.0%    0.0%    0.0%    0.0%    0.0%    0.0%    0.0%    0.0%    0.0%    0.0%    0.0%    0.0%    0.0%    0.0%    0.0%    0.0%    0.0%    0.0%    0.0%    0.0%    0.0%    0.0%    0.0%    0.0%    0.0%    0.0%    0.0%    0.0%    0.0%    0.0%    0.0%    0.0%    0.0%    0.0%    0.0%    0.0%    0.0%    0.0%    0.0%    0.0%    0.0%    0.0%    0.0%    0.0%    0.0%    0.0%    0.0%        k__Bacteria;p__OP11; c__       0    0.0%    0.0%    0.0%    0.0%    0.0%    0.0%    0.0%    0.0%    0.0%    0.0%    0.0%    0.0%    0.0%    0.0%    0.0%    0.0%    0.0%    0.0%    0.0%    0.0%    0.0%    0.0%    0.0%    0.0%    0.0%    0.0%    0.0%    0.0%    0.0%    0.0%    0.0%    0.0%    0.0%    0.0%    0.0%    0.0%    0.0%    0.0%    0.0%    0.0%    0.0%    0.0%    0.0%    0.0%    0.0%    0.0%    0.0%    0.0%    0.0%    0.0%    0.0%    0.0%    0.0%    0.0%    0.0%    0.0%    0.0%    0.0%    0.0%    0.0%    0.0%    0.0%    0.0%    0.0%    0.0%    0.0%    0.0%    0.0%    0.0%    0.0%    0.0%    0.0%    0.0%    0.0%    0.0%    0.0%    0.0%    0.0%    0.0%    0.0%    0.0%    0.0%    0.0%    0.0%    0.0%    0.0%    0.0%    0.0%    0.0%    0.0%    0.0%    0.0%    0.0%    0.0%    0.0%    0.0%    0.0%    0.0%    0.0%    0.0%    0.0%    0.0%    0.0%    0.0%    0.0%    0.0%    0.0%    0.0%    0.0%    0.0%    0.0%    0.0%    0.0%    0.0%    0.0%    0.0%    0.0%    0.0%    0.0%    0.0%    0.0%    0.0%    0.0%    0.0%    0.0%    0.0%    0.0%    0.0%    0.0%    0.0%    0.0%    0.0%    0.0%    0.0%        k__Bacteria;p__OP3; c__       0    0.0%    0.0%    0.0%    0.0%    0.0%    0.0%    0.0%    0.0%    0.0%    0.0%    0.0%    0.0%    0.0%    0.0%    0.0%    0.0%    0.0%    0.0%    0.0%    0.0%    0.0%    0.0%    0.0%    0.0%    0.0%    0.0%    0.0%    0.0%    0.0%    0.0%    0.0%    0.0%    0.0%    0.0%    0.0%    0.0%    0.0%    0.0%    0.0%    0.0%    0.0%    0.0%    0.0%    0.0%    0.0%    0.0%    0.0%    0.0%    0.0%    0.0%    0.0%    0.0%    0.0%    0.0%    0.0%    0.0%    0.0%    0.0%    0.0%    0.0%    0.0%    0.0%    0.0%    0.0%    0.0%    0.0%    0.0%    0.0%    0.0%    0.0%    0.0%    0.0%    0.0%    0.0%    0.0%    0.0%    0.0%    0.0%    0.0%    0.0%    0.0%    0.0%    0.0%    0.0%    0.0%    0.0%    0.0%    0.0%    0.0%    0.0%    0.0%    0.0%    0.0%    0.0%    0.0%    0.0%    0.0%    0.0%    0.0%    0.0%    0.0%    0.0%    0.0%    0.0%    0.0%    0.0%    0.0%    0.0%    0.0%    0.0%    0.0%    0.0%    0.0%    0.0%    0.0%    0.0%    0.0%    0.0%    0.0%    0.0%    0.0%    0.0%    0.0%    0.0%    0.0%    0.0%    0.0%    0.0%    0.0%    0.0%    0.0%    0.0%    0.0%    0.0%        k__Bacteria;p__OP8; c__OP8       0    0.0%    0.0%    0.0%    0.0%    0.0%    0.0%    0.0%    0.0%    0.0%    0.0%    0.0%    0.0%    0.0%    0.0%    0.0%    0.0%    0.0%    0.0%    0.0%    0.0%    0.0%    0.0%    0.0%    0.0%    0.0%    0.0%    0.0%    0.0%    0.0%    0.0%    0.0%    0.0%    0.0%    0.0%    0.0%    0.0%    0.0%    0.0%    0.0%    0.0%    0.0%    0.0%    0.0%    0.0%    0.0%    0.0%    0.0%    0.0%    0.0%    0.0%    0.0%    0.0%    0.0%    0.0%    0.0%    0.0%    0.0%    0.0%    0.0%    0.0%    0.0%    0.0%    0.0%    0.0%    0.0%    0.0%    0.0%    0.0%    0.0%    0.0%    0.0%    0.0%    0.0%    0.0%    0.0%    0.0%    0.0%    0.0%    0.0%    0.0%    0.0%    0.0%    0.0%    0.0%    0.0%    0.0%    0.0%    0.0%    0.0%    0.0%    0.0%    0.0%    0.0%    0.0%    0.0%    0.0%    0.0%    0.0%    0.0%    0.0%    0.0%    0.0%    0.0%    0.0%    0.0%    0.0%    0.0%    0.0%    0.0%    0.0%    0.0%    0.0%    0.0%    0.0%    0.0%    0.0%    0.0%    0.0%    0.0%    0.0%    0.0%    0.0%    0.0%    0.0%    0.0%    0.0%    0.0%    0.0%    0.0%    0.0%    0.0%    0.0%    0.0%    0.0%        k__Bacteria;p__Planctomycetes; c__FFCH393      97    0.0%    0.0%    0.0%    0.0%    0.0%    0.0%    0.0%    0.0%    0.0%    0.0%    0.0%    0.0%    0.0%    0.0%    0.0%    0.0%    0.0%    0.0%    0.0%    0.0%    0.0%    0.0%    0.0%    0.0%    0.0%    0.0%    0.0%    0.0%    0.0%    0.0%    0.0%    0.0%    0.0%    0.0%    0.0%    0.0%    0.0%    0.0%    0.0%    0.0%    0.0%    0.0%    0.0%    0.0%    0.0%    0.0%    0.0%    0.0%    0.0%    0.0%    0.0%    0.0%    0.0%    0.0%    0.0%    0.0%    0.0%    0.0%    0.0%    0.0%    0.0%    0.0%    0.0%    0.0%    0.0%    0.0%    0.0%    0.0%    0.0%    0.0%    0.0%    0.0%    0.0%    0.0%    0.0%    0.0%    0.0%    0.0%    0.0%    0.0%    0.0%    0.0%    0.0%    0.0%    0.0%    0.0%    0.0%    0.0%    0.0%    0.0%    0.0%    0.0%    0.0%    0.0%    0.0%    0.0%    0.0%    0.0%    0.0%    0.0%    0.0%    0.0%    0.0%    0.0%    0.0%    0.0%    0.0%    0.0%    0.0%    0.0%    0.0%    0.0%    0.0%    0.0%    0.0%    0.0%    0.0%    0.0%    0.0%    0.0%    0.0%    0.0%    0.0%    0.0%    0.0%    0.0%    0.0%    0.0%    0.0%    0.0%    0.0%    0.0%    0.0%    0.0%        k__Bacteria;p__Planctomycetes; c__Kueneniae       0    0.0%    0.0%    0.0%    0.0%    0.0%    0.0%    0.0%    0.0%    0.0%    0.0%    0.0%    0.0%    0.0%    0.0%    0.0%    0.0%    0.0%    0.0%    0.0%    0.0%    0.0%    0.0%    0.0%    0.0%    0.0%    0.0%    0.0%    0.0%    0.0%    0.0%    0.0%    0.0%    0.0%    0.0%    0.0%    0.0%    0.0%    0.0%    0.0%    0.0%    0.0%    0.0%    0.0%    0.0%    0.0%    0.0%    0.0%    0.0%    0.0%    0.0%    0.0%    0.0%    0.0%    0.0%    0.0%    0.0%    0.0%    0.0%    0.0%    0.0%    0.0%    0.0%    0.0%    0.0%    0.0%    0.0%    0.0%    0.0%    0.0%    0.0%    0.0%    0.0%    0.0%    0.0%    0.0%    0.0%    0.0%    0.0%    0.0%    0.0%    0.0%    0.0%    0.0%    0.0%    0.0%    0.0%    0.0%    0.0%    0.0%    0.0%    0.0%    0.0%    0.0%    0.0%    0.0%    0.0%    0.0%    0.0%    0.0%    0.0%    0.0%    0.0%    0.0%    0.0%    0.0%    0.0%    0.0%    0.0%    0.0%    0.0%    0.0%    0.0%    0.0%    0.0%    0.0%    0.0%    0.0%    0.0%    0.0%    0.0%    0.0%    0.0%    0.0%    0.0%    0.0%    0.0%    0.0%    0.0%    0.0%    0.0%    0.0%    0.0%    0.0%    0.0%        k__Bacteria;p__Planctomycetes; c__PW285       6    0.0%    0.0%    0.0%    0.0%    0.0%    0.0%    0.0%    0.0%    0.0%    0.0%    0.0%    0.0%    0.0%    0.0%    0.0%    0.0%    0.0%    0.0%    0.0%    0.0%    0.0%    0.0%    0.0%    0.0%    0.0%    0.0%    0.0%    0.0%    0.0%    0.0%    0.0%    0.0%    0.0%    0.0%    0.0%    0.0%    0.0%    0.0%    0.0%    0.0%    0.0%    0.0%    0.0%    0.0%    0.0%    0.0%    0.0%    0.0%    0.0%    0.0%    0.0%    0.0%    0.0%    0.0%    0.0%    0.0%    0.0%    0.0%    0.0%    0.0%    0.0%    0.0%    0.0%    0.0%    0.0%    0.0%    0.0%    0.0%    0.0%    0.0%    0.0%    0.0%    0.0%    0.0%    0.0%    0.0%    0.0%    0.0%    0.0%    0.0%    0.0%    0.0%    0.0%    0.0%    0.0%    0.0%    0.0%    0.0%    0.0%    0.0%    0.0%    0.0%    0.0%    0.0%    0.0%    0.0%    0.0%    0.0%    0.0%    0.0%    0.0%    0.0%    0.0%    0.0%    0.0%    0.0%    0.0%    0.0%    0.0%    0.0%    0.0%    0.0%    0.0%    0.0%    0.0%    0.0%    0.0%    0.0%    0.0%    0.0%    0.0%    0.0%    0.0%    0.0%    0.0%    0.0%    0.0%    0.0%    0.0%    0.0%    0.0%    0.0%    0.0%    0.0%        k__Bacteria;p__Planctomycetes; c__Phycisphaerae    1371    0.0%    0.0%    0.0%    0.0%    0.0%    0.1%    0.0%    0.0%    0.0%    0.0%    0.1%    0.0%    0.0%    0.0%    0.0%    0.0%    0.0%    0.0%    0.1%    0.0%    0.0%    0.0%    0.0%    0.0%    0.0%    0.0%    0.0%    0.0%    0.0%    0.0%    0.0%    0.0%    0.0%    0.0%    0.0%    0.0%    0.0%    0.0%    0.0%    0.0%    0.0%    0.0%    0.0%    0.0%    0.0%    0.0%    0.0%    0.0%    0.0%    0.0%    0.0%    0.0%    0.0%    0.1%    0.0%    0.0%    0.0%    0.0%    0.0%    0.0%    0.0%    0.1%    0.0%    0.0%    0.0%    0.0%    0.0%    0.0%    0.2%    0.1%    0.0%    0.0%    0.0%    0.1%    0.0%    0.0%    0.0%    0.0%    0.0%    0.1%    0.0%    0.0%    0.1%    0.0%    0.0%    0.0%    0.0%    0.1%    0.2%    0.0%    0.1%    0.0%    0.0%    0.0%    0.0%    0.0%    0.0%    0.0%    0.1%    0.0%    0.0%    0.0%    0.1%    0.0%    0.0%    0.0%    0.0%    0.0%    0.1%    0.1%    0.0%    0.1%    0.0%    0.0%    0.0%    0.1%    0.0%    0.1%    0.1%    0.0%    0.0%    0.1%    0.0%    0.0%    0.0%    0.1%    0.0%    0.0%    0.0%    0.0%    0.1%    0.0%    0.0%    0.0%        k__Bacteria;p__Planctomycetes; c__Planctomycea    2099    0.0%    0.0%    0.0%    0.1%    0.0%    0.1%    0.1%    0.0%    0.0%    0.0%    0.1%    0.0%    0.0%    0.0%    0.0%    0.0%    0.0%    0.0%    0.0%    0.0%    0.0%    0.0%    0.0%    0.0%    0.0%    0.0%    0.1%    0.0%    0.0%    0.0%    0.0%    0.0%    0.0%    0.0%    0.1%    0.0%    0.0%    0.0%    0.0%    0.0%    0.0%    0.0%    0.1%    0.0%    0.0%    0.0%    0.1%    0.0%    0.0%    0.0%    0.0%    0.0%    0.0%    0.1%    0.0%    0.0%    0.1%    0.0%    0.0%    0.1%    0.0%    0.2%    0.1%    0.0%    0.0%    0.0%    0.0%    0.0%    0.3%    0.1%    0.0%    0.1%    0.0%    0.1%    0.1%    0.1%    0.1%    0.0%    0.0%    0.1%    0.0%    0.0%    0.2%    0.0%    0.1%    0.0%    0.1%    0.1%    0.1%    0.0%    0.0%    0.1%    0.0%    0.0%    0.0%    0.1%    0.1%    0.0%    0.1%    0.1%    0.0%    0.0%    0.1%    0.0%    0.0%    0.0%    0.0%    0.0%    0.4%    0.2%    0.1%    0.2%    0.1%    0.0%    0.0%    0.1%    0.1%    0.2%    0.0%    0.0%    0.0%    0.1%    0.0%    0.0%    0.0%    0.1%    0.1%    0.0%    0.1%    0.1%    0.0%    0.0%    0.0%    0.1%        k__Bacteria;p__Planctomycetes; c__agg27      26    0.0%    0.0%    0.0%    0.0%    0.0%    0.0%    0.0%    0.0%    0.0%    0.0%    0.0%    0.0%    0.0%    0.0%    0.0%    0.0%    0.0%    0.0%    0.0%    0.0%    0.0%    0.0%    0.0%    0.0%    0.0%    0.0%    0.0%    0.0%    0.0%    0.0%    0.0%    0.0%    0.0%    0.0%    0.0%    0.0%    0.0%    0.0%    0.0%    0.0%    0.0%    0.0%    0.0%    0.0%    0.0%    0.0%    0.0%    0.0%    0.0%    0.0%    0.0%    0.0%    0.0%    0.0%    0.0%    0.0%    0.0%    0.0%    0.0%    0.0%    0.0%    0.0%    0.0%    0.0%    0.0%    0.0%    0.0%    0.0%    0.0%    0.0%    0.0%    0.0%    0.0%    0.0%    0.0%    0.0%    0.0%    0.0%    0.0%    0.0%    0.0%    0.0%    0.0%    0.0%    0.0%    0.0%    0.0%    0.0%    0.0%    0.0%    0.0%    0.0%    0.0%    0.0%    0.0%    0.0%    0.0%    0.0%    0.0%    0.0%    0.0%    0.0%    0.0%    0.0%    0.0%    0.0%    0.0%    0.0%    0.0%    0.0%    0.0%    0.0%    0.0%    0.0%    0.0%    0.0%    0.0%    0.0%    0.0%    0.0%    0.0%    0.0%    0.0%    0.0%    0.0%    0.0%    0.0%    0.0%    0.0%    0.0%    0.0%    0.0%    0.0%    0.0%        k__Bacteria;p__Planctomycetes; c__vadinHA49      30    0.0%    0.0%    0.0%    0.0%    0.0%    0.0%    0.0%    0.0%    0.0%    0.0%    0.0%    0.0%    0.0%    0.0%    0.0%    0.0%    0.0%    0.0%    0.0%    0.0%    0.0%    0.0%    0.0%    0.0%    0.0%    0.0%    0.0%    0.0%    0.0%    0.0%    0.0%    0.0%    0.0%    0.0%    0.0%    0.0%    0.0%    0.0%    0.0%    0.0%    0.0%    0.0%    0.0%    0.0%    0.0%    0.0%    0.0%    0.0%    0.0%    0.0%    0.0%    0.0%    0.0%    0.0%    0.0%    0.0%    0.0%    0.0%    0.0%    0.0%    0.0%    0.0%    0.0%    0.0%    0.0%    0.0%    0.0%    0.0%    0.0%    0.0%    0.0%    0.0%    0.0%    0.0%    0.0%    0.0%    0.0%    0.0%    0.0%    0.0%    0.0%    0.0%    0.0%    0.0%    0.0%    0.0%    0.0%    0.0%    0.0%    0.0%    0.0%    0.0%    0.0%    0.0%    0.0%    0.0%    0.0%    0.0%    0.0%    0.0%    0.0%    0.0%    0.0%    0.0%    0.0%    0.0%    0.0%    0.0%    0.0%    0.0%    0.0%    0.0%    0.0%    0.0%    0.0%    0.0%    0.0%    0.0%    0.0%    0.0%    0.0%    0.0%    0.0%    0.0%    0.0%    0.0%    0.0%    0.0%    0.0%    0.0%    0.0%    0.0%    0.0%    0.0%        k__Bacteria;p__Proteobacteria; c__Alphaproteobacteria   214287    5.0%    3.0%    5.1%    4.7%    2.1%    8.0%    4.6%   11.4%    5.3%    1.3%    6.7%    5.3%    1.0%    5.5%    2.3%    4.7%    8.0%    1.5%    5.4%    1.9%    5.4%    2.5%    1.7%    2.9%    3.5%    3.6%   13.1%    6.4%    4.6%    1.9%    4.9%    4.6%    6.0%    2.4%    3.1%    2.2%    1.1%    4.8%    3.1%    3.3%    4.3%    7.5%    5.9%    5.0%    2.3%    3.0%    9.2%    2.2%    1.4%    3.2%    4.8%    2.6%    2.6%    7.1%    3.9%    5.7%    4.3%    4.2%    2.5%    9.1%    6.8%    9.6%    2.9%    1.0%    2.6%    2.4%    4.4%    3.1%   16.5%    8.8%    3.2%    6.8%    2.4%    6.3%    5.2%    4.9%    6.2%    1.8%    1.4%   11.9%    3.7%    2.6%    8.9%    2.0%    6.5%    3.3%    4.9%    5.8%    5.1%    5.6%    6.2%    5.8%    4.0%    0.5%    2.8%    3.3%    5.0%    3.3%    5.1%    2.8%    2.7%    3.0%    5.3%    5.6%    4.1%    2.5%    5.3%    2.6%    9.2%   14.7%    7.6%    7.1%    3.8%    4.9%   23.1%    6.8%    8.0%   11.0%    5.5%    2.3%    3.3%    7.2%    2.3%    4.5%    5.2%    8.8%    5.1%    5.2%    8.5%    7.3%    7.8%    6.5%    4.0%    6.2%        k__Bacteria;p__Proteobacteria; c__Betaproteobacteria   249831    5.8%    5.0%    6.0%    4.5%    2.8%    9.4%    4.1%    7.6%    4.1%    2.0%    7.1%    6.0%    5.1%    4.8%    4.2%    4.8%    3.7%    2.9%    5.6%    3.1%    6.0%    3.0%    5.9%    5.0%    7.4%    6.1%    4.3%    4.7%    6.3%    2.1%    4.2%    3.3%    5.2%    3.8%    6.2%    7.4%    2.6%    9.4%    4.2%    5.1%    5.4%    8.5%   28.3%    7.1%    5.8%    5.0%    5.9%   11.7%    8.1%    7.8%    4.8%    4.0%    4.0%    3.8%    4.2%    4.8%    7.0%    3.4%    3.9%    4.4%    4.2%    5.3%    3.8%    3.2%    2.9%    4.1%    6.4%    3.3%    6.5%    5.0%    3.1%    5.2%    3.0%    6.5%    5.9%    5.2%    8.6%    2.2%    2.6%    5.9%    7.6%    3.8%    7.5%    5.4%    7.6%    5.1%    8.6%    6.4%    7.2%    6.0%    5.5%    4.3%    3.1%    1.9%    4.9%    5.7%    4.2%    5.8%    5.7%    4.0%    5.2%    3.3%    6.8%    3.7%    3.2%    2.6%    4.7%    3.6%    6.9%   11.6%    6.0%   13.6%    5.5%    3.8%    4.1%    7.1%    8.0%   11.0%    7.3%    5.8%    9.8%    7.9%   10.3%    8.4%   12.5%    8.1%    5.9%    6.4%    7.0%    8.1%   10.6%    6.8%    3.5%    6.7%        k__Bacteria;p__Proteobacteria; c__Deltaproteobacteria    7934    0.2%    0.2%    0.3%    0.1%    0.0%    0.7%    0.2%    0.2%    0.1%    0.0%    0.1%    0.2%    0.0%    0.1%    0.1%    0.1%    0.1%    0.0%    0.1%    0.1%    0.1%    0.1%    0.0%    0.1%    0.1%    0.1%    0.2%    0.1%    0.2%    0.1%    0.1%    0.1%    0.1%    0.1%    0.0%    0.0%    0.0%    0.2%    0.1%    0.2%    0.1%    0.0%    0.1%    0.1%    0.1%    0.1%    0.4%    0.1%    0.0%    0.1%    0.1%    0.0%    0.1%    0.2%    0.1%    0.1%    0.2%    0.1%    0.1%    0.3%    0.2%    0.6%    0.1%    0.1%    0.0%    0.1%    0.2%    0.1%    0.8%    0.3%    0.1%    0.2%    0.1%    0.2%    0.3%    0.2%    0.3%    0.1%    0.0%    0.6%    0.2%    0.1%    0.3%    0.0%    0.3%    0.2%    0.3%    0.4%    0.3%    0.2%    0.2%    0.3%    0.1%    0.0%    0.1%    0.2%    0.2%    0.2%    0.3%    0.2%    0.3%    0.2%    0.2%    0.2%    0.3%    0.1%    0.1%    0.1%    0.4%    0.6%    0.3%    0.5%    0.2%    0.2%    0.1%    0.2%    0.6%    0.6%    0.2%    0.1%    0.1%    0.3%    0.1%    0.2%    0.4%    0.3%    0.1%    0.3%    0.3%    0.4%    0.3%    0.2%    0.1%    0.3%        k__Bacteria;p__Proteobacteria; c__Epsilonproteobacteria    8350    0.2%    0.3%    0.3%    0.1%    0.1%    0.1%    0.1%    0.2%    0.1%    0.2%    0.2%    0.1%    0.1%    0.1%    0.1%    0.2%    0.1%    0.1%    0.1%    0.1%    0.1%    0.2%    0.2%    0.1%    0.2%    0.2%    0.1%    0.1%    0.1%    0.1%    0.5%    0.0%    0.1%    0.1%    0.2%    0.0%    0.4%    0.1%    0.2%    0.2%    0.1%    0.2%    0.2%    0.3%    5.6%    0.3%    0.1%    0.7%    0.3%    0.2%    0.2%    0.1%    0.1%    0.0%    0.1%    0.1%    0.9%    0.1%    0.1%    0.1%    0.1%    0.2%    0.1%    0.4%    0.1%    0.2%    0.2%    0.1%    0.1%    0.1%    0.1%    0.1%    0.1%    0.2%    0.1%    0.3%    0.2%    0.2%    0.1%    0.1%    0.2%    0.1%    0.2%    0.1%    0.2%    0.3%    0.3%    0.1%    0.1%    0.1%    0.2%    0.1%    0.4%    0.2%    0.4%    0.2%    0.2%    0.2%    0.2%    0.2%    0.2%    0.1%    0.2%    0.1%    0.1%    0.1%    0.0%    0.1%    0.1%    0.1%    0.1%    0.1%    0.1%    0.1%    0.0%    0.1%    0.1%    0.1%    0.1%    0.1%    0.2%    0.1%    0.1%    0.2%    0.3%    0.1%    0.1%    0.1%    0.1%    0.1%    0.1%    0.2%    0.1%    0.1%        k__Bacteria;p__Proteobacteria; c__Gammaproteobacteria   809721   18.8%   10.4%   12.6%   21.9%    8.5%   23.7%    8.8%   15.0%   13.0%   14.0%   14.1%   10.4%    5.7%   11.0%   22.5%   18.0%   34.1%   30.4%   20.8%   13.6%   40.1%   18.5%   17.4%   29.5%   20.3%   42.6%   14.4%   44.5%   11.2%    9.2%   22.6%   21.4%   34.6%   33.8%   45.6%   32.5%    8.0%   25.1%   19.0%   21.9%   25.8%   15.5%   24.5%   22.9%   20.8%   20.4%   20.5%   24.7%   18.0%   21.3%   24.8%   17.4%   25.8%   22.2%   31.5%   29.4%   14.7%   30.1%   22.2%   13.0%   16.8%   11.7%   11.2%    4.6%   15.0%    9.9%   20.4%    5.3%   13.1%   36.1%   31.1%   11.7%   14.2%   18.2%   15.9%   13.5%   26.7%    4.8%    7.4%   10.3%   10.4%    7.7%   14.0%    4.7%   13.7%   11.7%   19.7%   19.4%   17.1%   16.8%   18.9%   14.9%    6.5%   10.8%    9.7%   14.0%   20.9%   12.5%   14.7%   17.4%   14.6%   32.2%   14.2%   12.1%   37.5%   46.9%   27.6%   21.4%   14.2%   17.0%   17.0%   15.6%   11.3%   23.1%   20.5%   14.1%   20.1%   18.9%   16.4%   13.6%   26.5%   16.4%   14.9%   27.9%   27.3%   32.4%   21.5%   16.4%   19.6%   25.0%   15.5%   14.9%   18.7%   13.0%        k__Bacteria;p__SC3; c__       5    0.0%    0.0%    0.0%    0.0%    0.0%    0.0%    0.0%    0.0%    0.0%    0.0%    0.0%    0.0%    0.0%    0.0%    0.0%    0.0%    0.0%    0.0%    0.0%    0.0%    0.0%    0.0%    0.0%    0.0%    0.0%    0.0%    0.0%    0.0%    0.0%    0.0%    0.0%    0.0%    0.0%    0.0%    0.0%    0.0%    0.0%    0.0%    0.0%    0.0%    0.0%    0.0%    0.0%    0.0%    0.0%    0.0%    0.0%    0.0%    0.0%    0.0%    0.0%    0.0%    0.0%    0.0%    0.0%    0.0%    0.0%    0.0%    0.0%    0.0%    0.0%    0.0%    0.0%    0.0%    0.0%    0.0%    0.0%    0.0%    0.0%    0.0%    0.0%    0.0%    0.0%    0.0%    0.0%    0.0%    0.0%    0.0%    0.0%    0.0%    0.0%    0.0%    0.0%    0.0%    0.0%    0.0%    0.0%    0.0%    0.0%    0.0%    0.0%    0.0%    0.0%    0.0%    0.0%    0.0%    0.0%    0.0%    0.0%    0.0%    0.0%    0.0%    0.0%    0.0%    0.0%    0.0%    0.0%    0.0%    0.0%    0.0%    0.0%    0.0%    0.0%    0.0%    0.0%    0.0%    0.0%    0.0%    0.0%    0.0%    0.0%    0.0%    0.0%    0.0%    0.0%    0.0%    0.0%    0.0%    0.0%    0.0%    0.0%    0.0%    0.0%    0.0%        k__Bacteria;p__SC4; c__     624    0.0%    0.0%    0.0%    0.0%    0.0%    0.0%    0.0%    0.1%    0.0%    0.0%    0.0%    0.0%    0.0%    0.0%    0.0%    0.0%    0.0%    0.0%    0.0%    0.0%    0.0%    0.0%    0.0%    0.0%    0.0%    0.0%    0.0%    0.0%    0.0%    0.0%    0.0%    0.0%    0.0%    0.0%    0.0%    0.0%    0.0%    0.0%    0.0%    0.0%    0.0%    0.0%    0.0%    0.0%    0.0%    0.0%    0.0%    0.0%    0.0%    0.0%    0.0%    0.0%    0.1%    0.0%    0.0%    0.0%    0.0%    0.0%    0.0%    0.0%    0.0%    0.0%    0.0%    0.0%    0.0%    0.0%    0.0%    0.0%    0.0%    0.0%    0.0%    0.1%    0.0%    0.0%    0.0%    0.0%    0.0%    0.0%    0.0%    0.0%    0.0%    0.0%    0.0%    0.0%    0.0%    0.0%    0.0%    0.0%    0.0%    0.0%    0.0%    0.1%    0.0%    0.0%    0.0%    0.0%    0.0%    0.0%    0.0%    0.0%    0.0%    0.0%    0.0%    0.0%    0.0%    0.0%    0.0%    0.0%    0.0%    0.0%    0.0%    0.0%    0.0%    0.0%    0.0%    0.0%    0.0%    0.0%    0.0%    0.0%    0.0%    0.0%    0.0%    0.0%    0.0%    0.0%    0.0%    0.0%    0.0%    0.0%    0.0%    0.0%    0.0%    0.0%        k__Bacteria;p__SPAM; c__     139    0.0%    0.0%    0.0%    0.0%    0.0%    0.0%    0.0%    0.0%    0.0%    0.0%    0.0%    0.0%    0.0%    0.0%    0.0%    0.0%    0.0%    0.0%    0.0%    0.0%    0.0%    0.0%    0.0%    0.0%    0.0%    0.0%    0.0%    0.0%    0.0%    0.0%    0.0%    0.0%    0.0%    0.0%    0.0%    0.0%    0.0%    0.0%    0.0%    0.0%    0.0%    0.0%    0.0%    0.0%    0.0%    0.0%    0.0%    0.0%    0.0%    0.0%    0.0%    0.0%    0.0%    0.0%    0.0%    0.0%    0.0%    0.0%    0.0%    0.0%    0.0%    0.0%    0.0%    0.0%    0.0%    0.0%    0.0%    0.0%    0.0%    0.0%    0.0%    0.0%    0.0%    0.0%    0.0%    0.0%    0.0%    0.0%    0.0%    0.0%    0.0%    0.0%    0.0%    0.0%    0.0%    0.0%    0.0%    0.0%    0.0%    0.0%    0.0%    0.0%    0.0%    0.0%    0.0%    0.0%    0.0%    0.0%    0.0%    0.0%    0.0%    0.0%    0.0%    0.0%    0.0%    0.0%    0.0%    0.0%    0.0%    0.0%    0.0%    0.0%    0.0%    0.0%    0.0%    0.0%    0.0%    0.0%    0.0%    0.0%    0.0%    0.0%    0.0%    0.0%    0.0%    0.0%    0.0%    0.0%    0.0%    0.0%    0.0%    0.0%    0.0%    0.0%        k__Bacteria;p__SR1; c__     586    0.0%    0.0%    0.0%    0.0%    0.0%    0.0%    0.0%    0.0%    0.0%    0.0%    0.0%    0.0%    0.0%    0.0%    0.0%    0.0%    0.0%    0.0%    0.0%    0.0%    0.0%    0.0%    0.0%    0.0%    0.0%    0.0%    0.0%    0.0%    0.0%    0.0%    0.0%    0.0%    0.0%    0.0%    0.0%    0.0%    0.0%    0.0%    0.0%    0.0%    0.0%    0.0%    0.0%    0.0%    0.0%    0.0%    0.0%    0.0%    0.0%    0.0%    0.0%    0.0%    0.0%    0.0%    0.0%    0.1%    0.0%    0.0%    0.1%    0.0%    0.0%    0.0%    0.0%    0.0%    0.0%    0.0%    0.0%    0.0%    0.0%    0.0%    0.0%    0.0%    0.0%    0.0%    0.0%    0.0%    0.0%    0.0%    0.0%    0.0%    0.0%    0.0%    0.0%    0.0%    0.1%    0.0%    0.0%    0.0%    0.0%    0.0%    0.0%    0.0%    0.0%    0.0%    0.0%    0.0%    0.0%    0.0%    0.0%    0.0%    0.0%    0.0%    0.0%    0.0%    0.0%    0.0%    0.0%    0.0%    0.0%    0.0%    0.0%    0.0%    0.0%    0.0%    0.0%    0.0%    0.0%    0.0%    0.0%    0.0%    0.0%    0.0%    0.0%    0.0%    0.0%    0.0%    0.0%    0.0%    0.0%    0.0%    0.0%    0.0%    0.0%    0.0%        k__Bacteria;p__Spirochaetes; c__Brachyspirae       0    0.0%    0.0%    0.0%    0.0%    0.0%    0.0%    0.0%    0.0%    0.0%    0.0%    0.0%    0.0%    0.0%    0.0%    0.0%    0.0%    0.0%    0.0%    0.0%    0.0%    0.0%    0.0%    0.0%    0.0%    0.0%    0.0%    0.0%    0.0%    0.0%    0.0%    0.0%    0.0%    0.0%    0.0%    0.0%    0.0%    0.0%    0.0%    0.0%    0.0%    0.0%    0.0%    0.0%    0.0%    0.0%    0.0%    0.0%    0.0%    0.0%    0.0%    0.0%    0.0%    0.0%    0.0%    0.0%    0.0%    0.0%    0.0%    0.0%    0.0%    0.0%    0.0%    0.0%    0.0%    0.0%    0.0%    0.0%    0.0%    0.0%    0.0%    0.0%    0.0%    0.0%    0.0%    0.0%    0.0%    0.0%    0.0%    0.0%    0.0%    0.0%    0.0%    0.0%    0.0%    0.0%    0.0%    0.0%    0.0%    0.0%    0.0%    0.0%    0.0%    0.0%    0.0%    0.0%    0.0%    0.0%    0.0%    0.0%    0.0%    0.0%    0.0%    0.0%    0.0%    0.0%    0.0%    0.0%    0.0%    0.0%    0.0%    0.0%    0.0%    0.0%    0.0%    0.0%    0.0%    0.0%    0.0%    0.0%    0.0%    0.0%    0.0%    0.0%    0.0%    0.0%    0.0%    0.0%    0.0%    0.0%    0.0%    0.0%    0.0%    0.0%    0.0%        k__Bacteria;p__Spirochaetes; c__Leptospirae       1    0.0%    0.0%    0.0%    0.0%    0.0%    0.0%    0.0%    0.0%    0.0%    0.0%    0.0%    0.0%    0.0%    0.0%    0.0%    0.0%    0.0%    0.0%    0.0%    0.0%    0.0%    0.0%    0.0%    0.0%    0.0%    0.0%    0.0%    0.0%    0.0%    0.0%    0.0%    0.0%    0.0%    0.0%    0.0%    0.0%    0.0%    0.0%    0.0%    0.0%    0.0%    0.0%    0.0%    0.0%    0.0%    0.0%    0.0%    0.0%    0.0%    0.0%    0.0%    0.0%    0.0%    0.0%    0.0%    0.0%    0.0%    0.0%    0.0%    0.0%    0.0%    0.0%    0.0%    0.0%    0.0%    0.0%    0.0%    0.0%    0.0%    0.0%    0.0%    0.0%    0.0%    0.0%    0.0%    0.0%    0.0%    0.0%    0.0%    0.0%    0.0%    0.0%    0.0%    0.0%    0.0%    0.0%    0.0%    0.0%    0.0%    0.0%    0.0%    0.0%    0.0%    0.0%    0.0%    0.0%    0.0%    0.0%    0.0%    0.0%    0.0%    0.0%    0.0%    0.0%    0.0%    0.0%    0.0%    0.0%    0.0%    0.0%    0.0%    0.0%    0.0%    0.0%    0.0%    0.0%    0.0%    0.0%    0.0%    0.0%    0.0%    0.0%    0.0%    0.0%    0.0%    0.0%    0.0%    0.0%    0.0%    0.0%    0.0%    0.0%    0.0%    0.0%        k__Bacteria;p__Spirochaetes; c__SP_WWE1       0    0.0%    0.0%    0.0%    0.0%    0.0%    0.0%    0.0%    0.0%    0.0%    0.0%    0.0%    0.0%    0.0%    0.0%    0.0%    0.0%    0.0%    0.0%    0.0%    0.0%    0.0%    0.0%    0.0%    0.0%    0.0%    0.0%    0.0%    0.0%    0.0%    0.0%    0.0%    0.0%    0.0%    0.0%    0.0%    0.0%    0.0%    0.0%    0.0%    0.0%    0.0%    0.0%    0.0%    0.0%    0.0%    0.0%    0.0%    0.0%    0.0%    0.0%    0.0%    0.0%    0.0%    0.0%    0.0%    0.0%    0.0%    0.0%    0.0%    0.0%    0.0%    0.0%    0.0%    0.0%    0.0%    0.0%    0.0%    0.0%    0.0%    0.0%    0.0%    0.0%    0.0%    0.0%    0.0%    0.0%    0.0%    0.0%    0.0%    0.0%    0.0%    0.0%    0.0%    0.0%    0.0%    0.0%    0.0%    0.0%    0.0%    0.0%    0.0%    0.0%    0.0%    0.0%    0.0%    0.0%    0.0%    0.0%    0.0%    0.0%    0.0%    0.0%    0.0%    0.0%    0.0%    0.0%    0.0%    0.0%    0.0%    0.0%    0.0%    0.0%    0.0%    0.0%    0.0%    0.0%    0.0%    0.0%    0.0%    0.0%    0.0%    0.0%    0.0%    0.0%    0.0%    0.0%    0.0%    0.0%    0.0%    0.0%    0.0%    0.0%    0.0%    0.0%        k__Bacteria;p__Spirochaetes; c__Spirochaetes (class)    3444    0.1%    0.1%    0.1%    0.0%    0.0%    0.0%    0.0%    0.0%    0.0%    0.0%    0.1%    0.4%    0.1%    0.0%    0.0%    0.1%    0.0%    0.0%    0.1%    0.0%    0.0%    0.1%    0.1%    0.0%    0.0%    0.2%    0.0%    0.1%    0.1%    0.1%    0.1%    0.1%    0.1%    0.1%    0.1%    0.0%    0.0%    0.0%    0.0%    0.1%    0.2%    0.0%    0.0%    0.1%    0.2%    0.1%    0.0%    0.1%    0.1%    0.2%    0.2%    0.0%    0.0%    0.1%    0.0%    0.0%    0.1%    0.0%    0.0%    0.0%    0.0%    0.1%    0.1%    0.2%    0.1%    0.0%    0.1%    0.0%    0.0%    0.1%    0.0%    0.1%    0.1%    0.3%    0.0%    0.4%    0.2%    0.0%    0.0%    0.0%    0.0%    0.1%    0.2%    0.0%    0.4%    0.0%    0.3%    0.1%    0.2%    0.1%    0.1%    0.0%    0.0%    0.0%    0.2%    0.4%    0.1%    0.1%    0.2%    0.2%    0.1%    0.1%    0.1%    0.0%    0.1%    0.1%    0.0%    0.1%    0.0%    0.0%    0.0%    0.0%    0.1%    0.0%    0.0%    0.2%    0.0%    0.0%    0.1%    0.0%    0.0%    0.0%    0.0%    0.1%    0.2%    0.1%    0.1%    0.1%    0.0%    0.0%    0.1%    0.0%    0.0%    0.0%        k__Bacteria;p__Synergistetes; c__Synergistia     385    0.0%    0.0%    0.0%    0.0%    0.0%    0.0%    0.0%    0.0%    0.0%    0.0%    0.1%    0.0%    0.0%    0.0%    0.0%    0.0%    0.0%    0.0%    0.0%    0.0%    0.0%    0.0%    0.0%    0.0%    0.0%    0.0%    0.0%    0.0%    0.0%    0.0%    0.0%    0.1%    0.0%    0.0%    0.0%    0.0%    0.0%    0.0%    0.0%    0.0%    0.0%    0.0%    0.0%    0.0%    0.0%    0.0%    0.0%    0.0%    0.0%    0.0%    0.0%    0.0%    0.0%    0.0%    0.0%    0.0%    0.0%    0.0%    0.0%    0.0%    0.0%    0.0%    0.0%    0.0%    0.0%    0.0%    0.0%    0.0%    0.0%    0.0%    0.0%    0.0%    0.0%    0.0%    0.0%    0.0%    0.0%    0.0%    0.0%    0.0%    0.0%    0.0%    0.0%    0.0%    0.0%    0.0%    0.1%    0.0%    0.0%    0.0%    0.0%    0.0%    0.0%    0.0%    0.0%    0.1%    0.0%    0.0%    0.0%    0.0%    0.0%    0.0%    0.0%    0.0%    0.0%    0.0%    0.0%    0.0%    0.0%    0.0%    0.0%    0.0%    0.0%    0.0%    0.0%    0.0%    0.0%    0.0%    0.0%    0.0%    0.0%    0.0%    0.0%    0.0%    0.1%    0.0%    0.0%    0.0%    0.0%    0.0%    0.0%    0.0%    0.0%    0.0%        k__Bacteria;p__TM6; c__       5    0.0%    0.0%    0.0%    0.0%    0.0%    0.0%    0.0%    0.0%    0.0%    0.0%    0.0%    0.0%    0.0%    0.0%    0.0%    0.0%    0.0%    0.0%    0.0%    0.0%    0.0%    0.0%    0.0%    0.0%    0.0%    0.0%    0.0%    0.0%    0.0%    0.0%    0.0%    0.0%    0.0%    0.0%    0.0%    0.0%    0.0%    0.0%    0.0%    0.0%    0.0%    0.0%    0.0%    0.0%    0.0%    0.0%    0.0%    0.0%    0.0%    0.0%    0.0%    0.0%    0.0%    0.0%    0.0%    0.0%    0.0%    0.0%    0.0%    0.0%    0.0%    0.0%    0.0%    0.0%    0.0%    0.0%    0.0%    0.0%    0.0%    0.0%    0.0%    0.0%    0.0%    0.0%    0.0%    0.0%    0.0%    0.0%    0.0%    0.0%    0.0%    0.0%    0.0%    0.0%    0.0%    0.0%    0.0%    0.0%    0.0%    0.0%    0.0%    0.0%    0.0%    0.0%    0.0%    0.0%    0.0%    0.0%    0.0%    0.0%    0.0%    0.0%    0.0%    0.0%    0.0%    0.0%    0.0%    0.0%    0.0%    0.0%    0.0%    0.0%    0.0%    0.0%    0.0%    0.0%    0.0%    0.0%    0.0%    0.0%    0.0%    0.0%    0.0%    0.0%    0.0%    0.0%    0.0%    0.0%    0.0%    0.0%    0.0%    0.0%    0.0%    0.0%        k__Bacteria;p__TM7; c__       0    0.0%    0.0%    0.0%    0.0%    0.0%    0.0%    0.0%    0.0%    0.0%    0.0%    0.0%    0.0%    0.0%    0.0%    0.0%    0.0%    0.0%    0.0%    0.0%    0.0%    0.0%    0.0%    0.0%    0.0%    0.0%    0.0%    0.0%    0.0%    0.0%    0.0%    0.0%    0.0%    0.0%    0.0%    0.0%    0.0%    0.0%    0.0%    0.0%    0.0%    0.0%    0.0%    0.0%    0.0%    0.0%    0.0%    0.0%    0.0%    0.0%    0.0%    0.0%    0.0%    0.0%    0.0%    0.0%    0.0%    0.0%    0.0%    0.0%    0.0%    0.0%    0.0%    0.0%    0.0%    0.0%    0.0%    0.0%    0.0%    0.0%    0.0%    0.0%    0.0%    0.0%    0.0%    0.0%    0.0%    0.0%    0.0%    0.0%    0.0%    0.0%    0.0%    0.0%    0.0%    0.0%    0.0%    0.0%    0.0%    0.0%    0.0%    0.0%    0.0%    0.0%    0.0%    0.0%    0.0%    0.0%    0.0%    0.0%    0.0%    0.0%    0.0%    0.0%    0.0%    0.0%    0.0%    0.0%    0.0%    0.0%    0.0%    0.0%    0.0%    0.0%    0.0%    0.0%    0.0%    0.0%    0.0%    0.0%    0.0%    0.0%    0.0%    0.0%    0.0%    0.0%    0.0%    0.0%    0.0%    0.0%    0.0%    0.0%    0.0%    0.0%    0.0%        k__Bacteria;p__TM7; c__TM7-1       3    0.0%    0.0%    0.0%    0.0%    0.0%    0.0%    0.0%    0.0%    0.0%    0.0%    0.0%    0.0%    0.0%    0.0%    0.0%    0.0%    0.0%    0.0%    0.0%    0.0%    0.0%    0.0%    0.0%    0.0%    0.0%    0.0%    0.0%    0.0%    0.0%    0.0%    0.0%    0.0%    0.0%    0.0%    0.0%    0.0%    0.0%    0.0%    0.0%    0.0%    0.0%    0.0%    0.0%    0.0%    0.0%    0.0%    0.0%    0.0%    0.0%    0.0%    0.0%    0.0%    0.0%    0.0%    0.0%    0.0%    0.0%    0.0%    0.0%    0.0%    0.0%    0.0%    0.0%    0.0%    0.0%    0.0%    0.0%    0.0%    0.0%    0.0%    0.0%    0.0%    0.0%    0.0%    0.0%    0.0%    0.0%    0.0%    0.0%    0.0%    0.0%    0.0%    0.0%    0.0%    0.0%    0.0%    0.0%    0.0%    0.0%    0.0%    0.0%    0.0%    0.0%    0.0%    0.0%    0.0%    0.0%    0.0%    0.0%    0.0%    0.0%    0.0%    0.0%    0.0%    0.0%    0.0%    0.0%    0.0%    0.0%    0.0%    0.0%    0.0%    0.0%    0.0%    0.0%    0.0%    0.0%    0.0%    0.0%    0.0%    0.0%    0.0%    0.0%    0.0%    0.0%    0.0%    0.0%    0.0%    0.0%    0.0%    0.0%    0.0%    0.0%    0.0%        k__Bacteria;p__TM7; c__TM7-3     125    0.0%    0.0%    0.0%    0.0%    0.0%    0.0%    0.0%    0.0%    0.0%    0.0%    0.0%    0.0%    0.0%    0.0%    0.0%    0.0%    0.0%    0.0%    0.0%    0.0%    0.0%    0.0%    0.0%    0.0%    0.0%    0.0%    0.0%    0.0%    0.0%    0.0%    0.0%    0.0%    0.0%    0.0%    0.0%    0.0%    0.0%    0.0%    0.0%    0.0%    0.0%    0.0%    0.0%    0.0%    0.0%    0.0%    0.0%    0.0%    0.0%    0.0%    0.0%    0.0%    0.0%    0.0%    0.0%    0.0%    0.0%    0.0%    0.0%    0.0%    0.0%    0.0%    0.0%    0.0%    0.0%    0.0%    0.0%    0.0%    0.0%    0.0%    0.0%    0.0%    0.0%    0.0%    0.0%    0.0%    0.0%    0.0%    0.0%    0.0%    0.0%    0.0%    0.0%    0.0%    0.0%    0.0%    0.0%    0.0%    0.0%    0.0%    0.0%    0.0%    0.0%    0.0%    0.0%    0.0%    0.0%    0.0%    0.0%    0.0%    0.0%    0.0%    0.0%    0.0%    0.0%    0.0%    0.0%    0.0%    0.0%    0.0%    0.0%    0.0%    0.0%    0.0%    0.0%    0.0%    0.0%    0.0%    0.0%    0.0%    0.0%    0.0%    0.0%    0.0%    0.0%    0.0%    0.0%    0.0%    0.0%    0.0%    0.0%    0.0%    0.0%    0.0%        k__Bacteria;p__Tenericutes; c__       2    0.0%    0.0%    0.0%    0.0%    0.0%    0.0%    0.0%    0.0%    0.0%    0.0%    0.0%    0.0%    0.0%    0.0%    0.0%    0.0%    0.0%    0.0%    0.0%    0.0%    0.0%    0.0%    0.0%    0.0%    0.0%    0.0%    0.0%    0.0%    0.0%    0.0%    0.0%    0.0%    0.0%    0.0%    0.0%    0.0%    0.0%    0.0%    0.0%    0.0%    0.0%    0.0%    0.0%    0.0%    0.0%    0.0%    0.0%    0.0%    0.0%    0.0%    0.0%    0.0%    0.0%    0.0%    0.0%    0.0%    0.0%    0.0%    0.0%    0.0%    0.0%    0.0%    0.0%    0.0%    0.0%    0.0%    0.0%    0.0%    0.0%    0.0%    0.0%    0.0%    0.0%    0.0%    0.0%    0.0%    0.0%    0.0%    0.0%    0.0%    0.0%    0.0%    0.0%    0.0%    0.0%    0.0%    0.0%    0.0%    0.0%    0.0%    0.0%    0.0%    0.0%    0.0%    0.0%    0.0%    0.0%    0.0%    0.0%    0.0%    0.0%    0.0%    0.0%    0.0%    0.0%    0.0%    0.0%    0.0%    0.0%    0.0%    0.0%    0.0%    0.0%    0.0%    0.0%    0.0%    0.0%    0.0%    0.0%    0.0%    0.0%    0.0%    0.0%    0.0%    0.0%    0.0%    0.0%    0.0%    0.0%    0.0%    0.0%    0.0%    0.0%    0.0%        k__Bacteria;p__Tenericutes; c__Erysipelotrichi   16012    0.4%    0.5%    0.5%    0.2%    0.3%    0.2%    0.1%    0.2%    0.1%    0.0%    0.2%    0.1%    0.1%    0.2%    0.1%    0.1%    0.0%    0.0%    0.6%    0.1%    0.0%    0.4%    0.9%    0.1%    0.2%    0.2%    0.1%    0.3%    1.4%    0.2%    0.1%    0.2%    0.3%    0.1%    0.4%    0.1%    0.1%    0.3%    0.1%    0.3%    0.7%    0.1%    0.0%    0.3%    0.7%    0.4%    0.5%    1.0%    0.6%    0.7%    0.4%    0.1%    0.0%    0.2%    0.1%    0.2%    0.9%    0.2%    0.1%    0.1%    0.2%    0.4%    0.1%    0.1%    0.1%    0.1%    0.2%    0.1%    0.2%    0.2%    0.1%    0.2%    0.0%    0.3%    0.8%    0.3%    0.1%    0.1%    0.1%    0.1%    1.8%    1.4%    0.8%    0.1%    0.5%    0.2%    0.5%    0.6%    0.5%    0.5%    0.1%    0.2%    2.4%    0.1%    3.7%    0.3%    0.5%    1.8%    1.3%    0.5%    2.4%    0.4%    0.8%    0.1%    0.2%    0.5%    0.3%    0.3%    0.2%    0.1%    0.3%    0.3%    0.6%    0.2%    0.1%    0.3%    0.2%    0.2%    0.2%    0.1%    0.1%    0.3%    0.2%    0.3%    0.3%    0.2%    0.3%    0.3%    0.3%    0.2%    0.2%    0.3%    0.1%    0.1%        k__Bacteria;p__Tenericutes; c__ML615J-28       0    0.0%    0.0%    0.0%    0.0%    0.0%    0.0%    0.0%    0.0%    0.0%    0.0%    0.0%    0.0%    0.0%    0.0%    0.0%    0.0%    0.0%    0.0%    0.0%    0.0%    0.0%    0.0%    0.0%    0.0%    0.0%    0.0%    0.0%    0.0%    0.0%    0.0%    0.0%    0.0%    0.0%    0.0%    0.0%    0.0%    0.0%    0.0%    0.0%    0.0%    0.0%    0.0%    0.0%    0.0%    0.0%    0.0%    0.0%    0.0%    0.0%    0.0%    0.0%    0.0%    0.0%    0.0%    0.0%    0.0%    0.0%    0.0%    0.0%    0.0%    0.0%    0.0%    0.0%    0.0%    0.0%    0.0%    0.0%    0.0%    0.0%    0.0%    0.0%    0.0%    0.0%    0.0%    0.0%    0.0%    0.0%    0.0%    0.0%    0.0%    0.0%    0.0%    0.0%    0.0%    0.0%    0.0%    0.0%    0.0%    0.0%    0.0%    0.0%    0.0%    0.0%    0.0%    0.0%    0.0%    0.0%    0.0%    0.0%    0.0%    0.0%    0.0%    0.0%    0.0%    0.0%    0.0%    0.0%    0.0%    0.0%    0.0%    0.0%    0.0%    0.0%    0.0%    0.0%    0.0%    0.0%    0.0%    0.0%    0.0%    0.0%    0.0%    0.0%    0.0%    0.0%    0.0%    0.0%    0.0%    0.0%    0.0%    0.0%    0.0%    0.0%    0.0%        k__Bacteria;p__Tenericutes; c__Mollicutes   19522    0.5%    0.2%    0.3%    0.1%    0.1%    0.2%    0.2%    0.2%    0.3%    0.1%    0.3%    0.5%    0.5%    0.2%    0.2%    0.6%    0.4%    0.1%    0.5%    0.4%    0.3%    0.2%    1.1%    0.2%    0.2%    0.4%    0.1%    0.2%    0.6%    0.2%    0.1%    0.2%    0.3%    0.3%    0.2%    0.1%    0.3%    2.0%    1.9%    3.7%    1.2%    0.2%    0.3%    0.9%    0.9%    0.8%    0.7%    0.5%    0.7%    2.0%    0.8%    2.0%    0.0%    1.0%    0.6%    0.4%    0.5%    0.5%    0.2%    1.0%    0.8%    0.7%    1.5%    0.1%    0.6%    0.7%    1.6%    0.4%    0.7%    0.7%    0.5%    0.6%    0.1%    0.4%    0.4%    0.9%    0.5%    0.1%    0.1%    0.8%    0.2%    0.4%    0.5%    0.2%    0.5%    0.2%    0.4%    0.5%    0.8%    0.6%    0.2%    0.4%    0.1%    0.0%    0.1%    0.1%    0.2%    0.2%    0.2%    0.1%    0.5%    0.2%    0.2%    0.1%    0.2%    0.1%    0.0%    0.0%    0.1%    0.1%    0.6%    1.0%    0.3%    0.3%    0.1%    0.3%    0.1%    0.5%    0.3%    0.3%    0.2%    0.8%    0.3%    0.3%    0.4%    0.3%    0.4%    0.4%    1.3%    0.4%    0.3%    0.4%    0.2%    0.5%        k__Bacteria;p__Thermi; c__Deinococci    7764    0.2%    0.2%    0.1%    0.5%    0.1%    0.2%    0.1%    0.3%    0.1%    0.0%    0.4%    0.2%    0.0%    0.2%    0.3%    0.1%    0.1%    0.2%    0.1%    0.1%    0.1%    0.1%    0.0%    0.2%    0.2%    0.1%    0.1%    0.2%    0.1%    0.2%    0.1%    0.2%    0.1%    0.1%    0.8%    0.2%    0.1%    0.2%    0.1%    0.1%    0.2%    0.4%    0.1%    0.1%    0.1%    0.1%    0.3%    0.1%    0.0%    0.2%    0.4%    0.2%    0.1%    0.2%    0.1%    0.2%    0.0%    0.2%    0.4%    0.1%    0.5%    0.2%    0.2%    0.3%    0.3%    0.3%    0.1%    0.1%    0.1%    0.2%    0.3%    0.4%    0.2%    0.1%    0.2%    0.1%    0.1%    0.6%    0.9%    0.1%    0.1%    0.1%    0.3%    0.6%    0.4%    0.1%    0.1%    0.1%    0.3%    0.2%    0.2%    0.2%    0.1%    0.1%    0.0%    0.1%    0.2%    0.1%    0.1%    0.1%    0.1%    0.2%    0.1%    0.4%    0.2%    0.1%    0.4%    0.1%    0.2%    0.3%    0.3%    0.2%    0.2%    0.2%    0.1%    0.1%    0.3%    0.2%    0.2%    0.1%    0.1%    0.2%    0.2%    0.2%    0.1%    0.1%    0.1%    0.2%    0.1%    0.1%    0.1%    0.1%    0.1%    0.1%        k__Bacteria;p__Thermotogae; c__Thermotogae (class)      13    0.0%    0.0%    0.0%    0.0%    0.0%    0.0%    0.0%    0.0%    0.0%    0.0%    0.0%    0.0%    0.0%    0.0%    0.0%    0.0%    0.0%    0.0%    0.0%    0.0%    0.0%    0.0%    0.0%    0.0%    0.0%    0.0%    0.0%    0.0%    0.0%    0.0%    0.0%    0.0%    0.0%    0.0%    0.0%    0.0%    0.0%    0.0%    0.0%    0.0%    0.0%    0.0%    0.0%    0.0%    0.0%    0.0%    0.0%    0.0%    0.0%    0.0%    0.0%    0.0%    0.0%    0.0%    0.0%    0.0%    0.0%    0.0%    0.0%    0.0%    0.0%    0.0%    0.0%    0.0%    0.0%    0.0%    0.0%    0.0%    0.0%    0.0%    0.0%    0.0%    0.0%    0.0%    0.0%    0.0%    0.0%    0.0%    0.0%    0.0%    0.0%    0.0%    0.0%    0.0%    0.0%    0.0%    0.0%    0.0%    0.0%    0.0%    0.0%    0.0%    0.0%    0.0%    0.0%    0.0%    0.0%    0.0%    0.0%    0.0%    0.0%    0.0%    0.0%    0.0%    0.0%    0.0%    0.0%    0.0%    0.0%    0.0%    0.0%    0.0%    0.0%    0.0%    0.0%    0.0%    0.0%    0.0%    0.0%    0.0%    0.0%    0.0%    0.0%    0.0%    0.0%    0.0%    0.0%    0.0%    0.0%    0.0%    0.0%    0.0%    0.0%    0.0%        k__Bacteria;p__Verrucomicrobia; c__      41    0.0%    0.0%    0.0%    0.0%    0.0%    0.0%    0.0%    0.0%    0.0%    0.0%    0.0%    0.0%    0.0%    0.0%    0.0%    0.0%    0.0%    0.0%    0.0%    0.0%    0.0%    0.0%    0.0%    0.0%    0.0%    0.0%    0.0%    0.0%    0.0%    0.0%    0.0%    0.0%    0.0%    0.0%    0.0%    0.0%    0.0%    0.0%    0.0%    0.0%    0.0%    0.0%    0.0%    0.0%    0.0%    0.0%    0.0%    0.0%    0.0%    0.0%    0.0%    0.0%    0.0%    0.0%    0.0%    0.0%    0.0%    0.0%    0.0%    0.0%    0.0%    0.0%    0.0%    0.0%    0.0%    0.0%    0.0%    0.0%    0.0%    0.0%    0.0%    0.0%    0.0%    0.0%    0.0%    0.0%    0.0%    0.0%    0.0%    0.0%    0.0%    0.0%    0.0%    0.0%    0.0%    0.0%    0.0%    0.0%    0.0%    0.0%    0.0%    0.0%    0.0%    0.0%    0.0%    0.0%    0.0%    0.0%    0.0%    0.0%    0.0%    0.0%    0.0%    0.0%    0.0%    0.0%    0.0%    0.0%    0.0%    0.0%    0.0%    0.0%    0.0%    0.0%    0.0%    0.0%    0.0%    0.0%    0.0%    0.0%    0.0%    0.0%    0.0%    0.0%    0.0%    0.0%    0.0%    0.0%    0.0%    0.0%    0.0%    0.0%    0.0%    0.0%        k__Bacteria;p__Verrucomicrobia; c__Opitutae     288    0.0%    0.0%    0.0%    0.0%    0.0%    0.0%    0.0%    0.1%    0.0%    0.0%    0.0%    0.0%    0.0%    0.0%    0.0%    0.0%    0.0%    0.0%    0.0%    0.0%    0.0%    0.0%    0.0%    0.0%    0.0%    0.0%    0.0%    0.0%    0.0%    0.0%    0.0%    0.0%    0.0%    0.0%    0.0%    0.0%    0.0%    0.0%    0.0%    0.0%    0.0%    0.0%    0.0%    0.0%    0.0%    0.0%    0.0%    0.0%    0.0%    0.0%    0.0%    0.0%    0.0%    0.0%    0.0%    0.0%    0.0%    0.0%    0.0%    0.0%    0.0%    0.0%    0.0%    0.0%    0.0%    0.0%    0.0%    0.0%    0.0%    0.0%    0.0%    0.0%    0.0%    0.0%    0.0%    0.0%    0.0%    0.0%    0.0%    0.0%    0.0%    0.0%    0.0%    0.0%    0.0%    0.0%    0.0%    0.0%    0.0%    0.0%    0.0%    0.0%    0.0%    0.0%    0.0%    0.0%    0.0%    0.0%    0.0%    0.0%    0.0%    0.0%    0.0%    0.0%    0.0%    0.0%    0.0%    0.0%    0.0%    0.2%    0.0%    0.0%    0.0%    0.0%    0.0%    0.0%    0.0%    0.0%    0.0%    0.0%    0.0%    0.0%    0.0%    0.0%    0.0%    0.0%    0.0%    0.0%    0.0%    0.0%    0.0%    0.0%    0.0%    0.0%        k__Bacteria;p__Verrucomicrobia; c__Spartobacteria    1514    0.0%    0.0%    0.0%    0.1%    0.0%    0.0%    0.0%    0.0%    0.0%    0.0%    0.0%    0.0%    0.0%    0.0%    0.0%    0.0%    0.0%    0.0%    0.0%    0.0%    0.0%    0.0%    0.0%    0.0%    0.0%    0.0%    0.1%    0.1%    0.1%    0.0%    0.0%    0.0%    0.0%    0.0%    0.0%    0.0%    0.0%    0.0%    0.0%    0.0%    0.0%    0.0%    0.0%    0.0%    0.0%    0.0%    0.0%    0.0%    0.0%    0.0%    0.0%    0.0%    0.0%    0.1%    0.0%    0.0%    0.0%    0.1%    0.0%    0.1%    0.0%    0.1%    0.0%    0.0%    0.0%    0.0%    0.0%    0.0%    0.2%    0.1%    0.0%    0.1%    0.0%    0.1%    0.1%    0.0%    0.1%    0.0%    0.0%    0.1%    0.1%    0.0%    0.0%    0.0%    0.1%    0.0%    0.0%    0.1%    0.1%    0.0%    0.1%    0.0%    0.0%    0.0%    0.0%    0.0%    0.1%    0.0%    0.0%    0.0%    0.0%    0.0%    0.0%    0.0%    0.0%    0.0%    0.0%    0.0%    0.1%    0.1%    0.1%    0.1%    0.0%    0.1%    0.0%    0.0%    0.0%    0.1%    0.0%    0.0%    0.0%    0.0%    0.0%    0.0%    0.0%    0.1%    0.0%    0.0%    0.0%    0.0%    0.0%    0.0%    0.0%    0.0%        k__Bacteria;p__Verrucomicrobia; c__TP21       0    0.0%    0.0%    0.0%    0.0%    0.0%    0.0%    0.0%    0.0%    0.0%    0.0%    0.0%    0.0%    0.0%    0.0%    0.0%    0.0%    0.0%    0.0%    0.0%    0.0%    0.0%    0.0%    0.0%    0.0%    0.0%    0.0%    0.0%    0.0%    0.0%    0.0%    0.0%    0.0%    0.0%    0.0%    0.0%    0.0%    0.0%    0.0%    0.0%    0.0%    0.0%    0.0%    0.0%    0.0%    0.0%    0.0%    0.0%    0.0%    0.0%    0.0%    0.0%    0.0%    0.0%    0.0%    0.0%    0.0%    0.0%    0.0%    0.0%    0.0%    0.0%    0.0%    0.0%    0.0%    0.0%    0.0%    0.0%    0.0%    0.0%    0.0%    0.0%    0.0%    0.0%    0.0%    0.0%    0.0%    0.0%    0.0%    0.0%    0.0%    0.0%    0.0%    0.0%    0.0%    0.0%    0.0%    0.0%    0.0%    0.0%    0.0%    0.0%    0.0%    0.0%    0.0%    0.0%    0.0%    0.0%    0.0%    0.0%    0.0%    0.0%    0.0%    0.0%    0.0%    0.0%    0.0%    0.0%    0.0%    0.0%    0.0%    0.0%    0.0%    0.0%    0.0%    0.0%    0.0%    0.0%    0.0%    0.0%    0.0%    0.0%    0.0%    0.0%    0.0%    0.0%    0.0%    0.0%    0.0%    0.0%    0.0%    0.0%    0.0%    0.0%    0.0%        k__Bacteria;p__Verrucomicrobia; c__Verrucomicrobiae    3459    0.1%    0.1%    0.2%    0.0%    0.0%    0.2%    0.1%    0.1%    0.1%    0.0%    0.1%    0.1%    0.0%    0.1%    0.1%    0.0%    0.1%    0.0%    0.1%    0.0%    0.0%    0.0%    0.0%    0.0%    0.1%    0.0%    0.2%    0.1%    0.1%    0.0%    0.0%    0.1%    0.1%    0.0%    0.0%    0.0%    0.0%    0.1%    0.0%    0.1%    0.0%    0.0%    0.1%    0.0%    0.0%    0.0%    0.1%    0.0%    0.0%    0.0%    0.0%    0.0%    0.1%    0.0%    0.1%    0.1%    0.1%    0.0%    0.0%    0.1%    0.1%    0.2%    0.0%    0.1%    0.0%    0.0%    0.1%    0.0%    0.3%    0.1%    0.0%    0.1%    0.0%    0.1%    0.1%    0.0%    0.1%    0.0%    0.0%    0.2%    0.0%    0.0%    0.5%    0.0%    0.0%    0.0%    0.1%    0.1%    0.1%    0.0%    0.1%    0.2%    0.0%    0.0%    0.0%    0.0%    0.1%    0.0%    0.1%    0.0%    0.0%    0.0%    0.1%    0.1%    0.0%    0.0%    0.1%    0.0%    0.7%    0.8%    0.2%    0.3%    0.1%    0.1%    0.0%    0.0%    0.1%    0.1%    0.1%    0.0%    0.1%    0.1%    0.0%    0.1%    0.2%    0.1%    0.0%    0.1%    0.1%    0.2%    0.1%    0.1%    0.1%    0.2%        k__Bacteria;p__WPS-2; c__      44    0.0%    0.0%    0.0%    0.0%    0.0%    0.0%    0.0%    0.0%    0.0%    0.0%    0.0%    0.0%    0.0%    0.0%    0.0%    0.0%    0.0%    0.0%    0.0%    0.0%    0.0%    0.0%    0.0%    0.0%    0.0%    0.0%    0.0%    0.0%    0.0%    0.0%    0.0%    0.0%    0.0%    0.0%    0.0%    0.0%    0.0%    0.0%    0.0%    0.0%    0.0%    0.0%    0.0%    0.0%    0.0%    0.0%    0.0%    0.0%    0.0%    0.0%    0.0%    0.0%    0.0%    0.0%    0.0%    0.0%    0.0%    0.0%    0.0%    0.0%    0.0%    0.0%    0.0%    0.0%    0.0%    0.0%    0.0%    0.0%    0.0%    0.0%    0.0%    0.0%    0.0%    0.0%    0.0%    0.0%    0.0%    0.0%    0.0%    0.0%    0.0%    0.0%    0.0%    0.0%    0.0%    0.0%    0.0%    0.0%    0.0%    0.0%    0.0%    0.0%    0.0%    0.0%    0.0%    0.0%    0.0%    0.0%    0.0%    0.0%    0.0%    0.0%    0.0%    0.0%    0.0%    0.0%    0.0%    0.0%    0.0%    0.0%    0.0%    0.0%    0.0%    0.0%    0.0%    0.0%    0.0%    0.0%    0.0%    0.0%    0.0%    0.0%    0.0%    0.0%    0.0%    0.0%    0.0%    0.0%    0.0%    0.0%    0.0%    0.0%    0.0%    0.0%        k__Bacteria;p__WS3; c__PRR-12      31    0.0%    0.0%    0.0%    0.0%    0.0%    0.0%    0.0%    0.0%    0.0%    0.0%    0.0%    0.0%    0.0%    0.0%    0.0%    0.0%    0.0%    0.0%    0.0%    0.0%    0.0%    0.0%    0.0%    0.0%    0.0%    0.0%    0.0%    0.0%    0.0%    0.0%    0.0%    0.0%    0.0%    0.0%    0.0%    0.0%    0.0%    0.0%    0.0%    0.0%    0.0%    0.0%    0.0%    0.0%    0.0%    0.0%    0.0%    0.0%    0.0%    0.0%    0.0%    0.0%    0.0%    0.0%    0.0%    0.0%    0.0%    0.0%    0.0%    0.0%    0.0%    0.0%    0.0%    0.0%    0.0%    0.0%    0.0%    0.0%    0.0%    0.0%    0.0%    0.0%    0.0%    0.0%    0.0%    0.0%    0.0%    0.0%    0.0%    0.0%    0.0%    0.0%    0.0%    0.0%    0.0%    0.0%    0.0%    0.0%    0.0%    0.0%    0.0%    0.0%    0.0%    0.0%    0.0%    0.0%    0.0%    0.0%    0.0%    0.0%    0.0%    0.0%    0.0%    0.0%    0.0%    0.0%    0.0%    0.0%    0.0%    0.0%    0.0%    0.0%    0.0%    0.0%    0.0%    0.0%    0.0%    0.0%    0.0%    0.0%    0.0%    0.0%    0.0%    0.0%    0.0%    0.0%    0.0%    0.0%    0.0%    0.0%    0.0%    0.0%    0.0%    0.0%        k__Bacteria;p__ZB2; c__       1    0.0%    0.0%    0.0%    0.0%    0.0%    0.0%    0.0%    0.0%    0.0%    0.0%    0.0%    0.0%    0.0%    0.0%    0.0%    0.0%    0.0%    0.0%    0.0%    0.0%    0.0%    0.0%    0.0%    0.0%    0.0%    0.0%    0.0%    0.0%    0.0%    0.0%    0.0%    0.0%    0.0%    0.0%    0.0%    0.0%    0.0%    0.0%    0.0%    0.0%    0.0%    0.0%    0.0%    0.0%    0.0%    0.0%    0.0%    0.0%    0.0%    0.0%    0.0%    0.0%    0.0%    0.0%    0.0%    0.0%    0.0%    0.0%    0.0%    0.0%    0.0%    0.0%    0.0%    0.0%    0.0%    0.0%    0.0%    0.0%    0.0%    0.0%    0.0%    0.0%    0.0%    0.0%    0.0%    0.0%    0.0%    0.0%    0.0%    0.0%    0.0%    0.0%    0.0%    0.0%    0.0%    0.0%    0.0%    0.0%    0.0%    0.0%    0.0%    0.0%    0.0%    0.0%    0.0%    0.0%    0.0%    0.0%    0.0%    0.0%    0.0%    0.0%    0.0%    0.0%    0.0%    0.0%    0.0%    0.0%    0.0%    0.0%    0.0%    0.0%    0.0%    0.0%    0.0%    0.0%    0.0%    0.0%    0.0%    0.0%    0.0%    0.0%    0.0%    0.0%    0.0%    0.0%    0.0%    0.0%    0.0%    0.0%    0.0%    0.0%    0.0%    0.0%    
     
  Taxonomy Summary. Current Level: Order  
     View Figure (.pdf)    View Legend (.pdf)   
  
[truncated: 3,542,588 more chars]
