# Supplementary material for: Moving pictures of the human microbiome
Source: Genome Biol. 2011 May 30;12(5):R50. doi: 10.1186/gb-2011-12-5-r50 (PMC3271711; doi:10.1186/gb-2011-12-5-r50)
Supplement: Additional file 15 — Temporal variation in phylum, class, order, family, and genus abundances (F4 right palm). The x-axis scale differs between M3 and F4 plots. [file gb-2011-12-5-r50-S15.ZIP › AdditionalFile15/index.html]

 
 
 
 

 
 Taxa Summaries 
 
 
  
 
     
  Taxonomy Summary. Current Level: Phylum  
     View Figure (.pdf)    View Legend (.pdf)   
   
 
     
 
 

 
 
 
 
 
 
 
 
 
 
 
 
 
 
 
 
 
 
 
 
 
 
 
 
 
 
 
 
 
 
 
 
 
 
 
 
 
 
 
 
 
 
 
 
 
 
 
 
 
 
 
 
 
 
 
 
 
 
 
 
 
 
 
 
 
 
 
 
 
 
 
 
 
 
 
 
 
 
 
 
 
 
 
 
 
 
 
 
 
 
 
 
 
 
 
 
 
 
 
 
 
 
 
 
 
 
 
 
 
 
 
 
 
 
 
 
 
 
 
 
 
 
 
 
 
 
 
 
 
 
 
 
 
 
 
 
 
 
 
 
 
 
 
 
 
 
 
 
 
 
 
 
 
 
 
 
 
 
 
 
 
 
 
 
 
 
 
 
 
 
 
 
 
 
 
 
 
 
 
 
 
 
 
 
 
 
 
 
 
 
 
 
 
 
 
 
 
 
 
 
 
 
 
 
 
 
 
 
 
 
 
 
 
 
 
 
 
 
 
 
 
 
 
 
 
 
 
 
 
 
 
 
 
 
 
 
 
 
 
 
 
 
 
 
 
 
 
 
 
 
 
 
 
 
 
 
 
 
 
 
 
 
 
 
 
 
 
 
 
 
 
 
 
 
 
 
 
 
 
 
 
 
 
 
 
 
 
 
 
 
 
 
 
 
 
 
 
 
 
 
 
 
 
 
 
 
 
 
 
 
 
 
 
 
 
 
 
 
 
 
 
 
 
 
 
 
 
 
 
 
 
 
 
 
 
 
 
 
 
 
 
 
 
 
 
 
 
 
 
 
 
 
 
 
 
 
 
 
 
 
 
 
 
 
 
 
 
 
 
 
 
 
 
 
 
 
 
 
 
 
 
 
 
 
 
 
 
 
 
 
 
 
 
 
 
 
 
 
 
 
 
 
 
 
 
 
 
 
 
 
 
 
 
 
 
 
 
 
 
 
 
 
 
 
 
 
 
 
 
 
 
 
 
 
 
 
 
 
 
 
 
 
 
 
 
 
 
 
 
 
 
 
 
 
 
 
 
 
 
 
 
 
 
 
 
 
 
 
 
 
 
 
 
 
 
 
 
 
 
 
 
 
 
 
 
 
 
 
 
 
 
 
 
 
 
 
 
 
 
 
 
 
 
 
 
 
 
 
 
 
 
 
 
 
 
 
 
 
 
 
 
 
 
 
 
 
 
 
 
 
 
 
 
 
 
 
 
 
 
 
 
 
 
 
 
 
 
 
 
 
 
 
 
 
 
 
 
 
 
 
 
 
 
 
 
 
 
 
 
 
 
 
 
 
 
 
 
 
 
 
 
 
 
 
 
 
 
 
 
 
 
 
 
 
 
 
 
 
 
 
 
 
 
 
 
 
 
 
 
 
 
 
 
 
 
 
 
 
 
 
 
 
 
 
 
 
 
 
 
 
 
 
 
 
 
 
 
 
 
 
 
 
 
 
 
 
 
 
 
 
 
 
 
 
 
 
 
 
 
 
 
 
 
 
 
 
 
 
 
 
 
 
 
 
 
 
 
 
 
 
 
 
 
 
 
 
 
 
 
 
 
 
 
 
 
 
 
 
 
 
 
 
 
 
 
 
 
 
 
 
 
 
 
 
 
 
 
 
 
 
 
 
 
 
 
 
 
 
 
 
 
 
 
 
 
 
 
 
 
 
 
 
 
 
 
 
 
 
 
 
 
 
 
 
 
 
 
 
 
 
 
 
 
 
 
 
 
 
 
 
 
 
 
 
 
 
 
 
 
 
 
 
 
 
 
 
 
 
 
 
 
 
 
 
 
 
 
 
 
 
 
 
 
 
 
 
 
 
 
 
 
 
 
 
 
 
 
 
 
 
 
 
 
 
 
 
 
 
 
 
 
 
 
 
 
 
 
 
 
 
 
 
 
 
 
 
 
 
 
 
 
 
 
 
 
 
 
 
 
 
 
 
 
 
 
 
 
 
 
 
 
 
 
 
 
 
 
 
 
 
 
 
 
 
 
 
 
 
 
 
 
 
 
 
 
 
 
 
 
 
 
 
 
 
 
 
 
 
 
 
 
 
 
 
 
 
 
 
 
 
 
 
 
 
 
 
 
 
 
 
 
 
 
 
 
 
 
 
 
 
 
 
 
 
 
 
 
 
 
 
 
 
 
 
 
 
 
 
 
 
 
 
 
 
 
 
 
 
 
 
 
 
 
 
 
 
 
 
 
 
 
 
 
 
 
 
 
 
 
 
 
 
 
 
 
 
 
 
 
 
 
 
 
 
 
 
 
 
 
 
 
 
 
 
 
 
 
 
 
 
 
 
 
 
 
 
 
 
 
 
 
 
 
 
 
 
 
 
 
 
 
 
 
 
 
 
 
 
 
 
 
 
 
 
 
 
 
 
 
 
 
 
 
 
 
 
 
 
 
 
 
 
 
 
 
 
 
 
 
 
 
 
 
 
 
 
 
 
 
 
 
 
 
 
 
 
 
 
 
 
 
 
 
 
 
 
 
 
 
 
 
 
 
 
 
 
 
 
 
 
 
 
 
 
 
 
 
 
 
 
 
 
 
 
 
 
 
 
 
 
 
 
 
 
 
 
 
 
 
 
 
 
 
 
 
 
 
 
 
 
 
 
 
 
 
 
 
 
 
 
 
 
 
 
 
 
 
 
 
 
 
 
 
 
 
 
 
 
 
 
 
 
 
 
 
 
 
 
 
 
 
 
 
 
 
 
 
 
 
 
 
 
 
 
 
 
 
 
 
 
 
 
 
 
 
 
 
 
 
 
 
 
 
 
 
 
 
 
 
 
 
 
 
 
 
 
 
 
 
 
 
 
 
 
 
 
 
 
 
 
 
 
 
 
 
 
 
 
 
 
 
 
 
 
 
 
 
 
 
 
 
 
 
 
 
 
 
 
 
 
 
 
 
 
 
 
 
 
 
 
 
 
 
 
 
 
 
 
 
 
 
 
 
 
 
 
 
 
 
 
 
 
 
 
 
 
 
 
 
 
 
 
 
 
 
 
 
 
 
 
 
 
 
 
 
 
 
 
 
 
 
 
 
 
 
 
 
 
 
 
 
 
 
 
 
 
 
 
 
 
 
 
 
 
 
 
 
 
 
 
 
 
 
 
 
 
 
 
 
 
 
 
 
 
 
 
 
 
 
 
 
 
 
 
 
 
 
 
 
 
 
 
 
 
 
 
 
 
 
 
 
 
 
 
 
 
 
 
 
 
 
 
 
 
 
 
 
 
 
 
 
 
 
 
 
 
 
 
 
 
 
 
 
 
 
 
 
 
 
 
 
 
 
 
 
 
 
 
 
 
 
 
 
 
 
 
 
 
 
 
 
 
 
 
 
 
 
 
 
 
 
 
 
 
 
 
 
 
 
 
 
 
 
 
 
 
 
 
 
 
 
 
 
 
 
 
 
 
 
 
 
 
 
 
 
 
 
 
 
 
 
 
 
 
 
 
 
 
 
 
 
 
 
 
 
 
 
 
 
 
 
 
 
 
 
 
 
 
 
 
 
 
 
 
 
 
 
 
 
 
 
 
 
 
 
 
 
 
 
 
 
 
 
 
 
 
 
 
 
 
 
 
 
 
 
 
 
 
 
 
 
 
 
 
 
 
 
 
 
 
 
 
 
 
 
 
 
 
 
 
 
 
 
 
 
 
 
 
 
 
 
 
 
 
 
 
 
 
 
 
 
 
 
 
 
 
 
 
 
 
 
 
 
 
 
 
 
 
 
 
 
 
 
 
 
 
 
 
 
 
 
 
 
 
 
 
 
 
 
 
 
 
 
 
 
 
 
 
 
 
 
 
 
 
 
 
 
 
 
 
 
 
 
 
 
 
 
 
 
 
 
 
 
 
 
 
 
 
 
 
 
 
 
 
 
 
 
 
 
 
 
 
 
 
 
 
 
 
 
 
 
 
 
 
 
 
 
 
 
 
 
 
 
 
 
 
 
 
 
 
 
 
 
 
 
 
 
 
 
 
 
 
 
 
 
 
 
 
 
 
 
 
 
 
 
 
 
 
 
 
 
 
 
 
 
 
 
 
 
 
 
 
 
 
 
 
 
 
 
 
 
 
 
 
 
 
 
 
 
 
 
 
 
 
 
 
 
 
 
 
 
 
 
 
 
 
 
 
 
 
 
 
 
 
 
 
 
 
 
 
 
 
 
 
 
 
 
 
 
 
 
 
 
 
 
 
 
 
 
 
 
 
 
 
 
 
 
 
 
 
 
 
 
 
 
 
 
 
 
 
 
 
 
 
 
 
 
 
 
 
 
 
 
 
 
 
 
 
 
 
 
 
 
 
 
 
 
 
 
 
 
 
 
 
 
 
 
 
 
 
 
 
 
 
 
 
 
 
 
 
 
 
 
 
 
 
 
 
 
 
 
 
 
 
 
 
 
 
 
 
 
 
 
 
 
 
 
 
 
 
 
 
 
 
 
 
 
 
 
 
 
 
 
 
 
 
 
 
 
 
 
 
 
 
 
 
 
 
 
 
 
 
 
 
 
 
 
 
 
 
 
 
 
 
 
 
 
 
 
 
 
 
 
 
 
 
 
 
 
 
 
 
 
 
 
 
 
 
 
 
 
 
 
 
 
 
 
 
 
 
 
 
 
 
 
 
 
 
 
 
 
 
 
 
 
 
 
 
 
 
 
 
 
 
 
 
 
 
 
 
 
 
 
 
 
 
 
 
 
 
 
 
 
 
 
 
 
 
 
 
 
 
 
 
 
 
 
 
 
 
 
 
 
 
 
 
 
 
 
 
 
 
 
 
 
 
 
 
 
 
 
 
 
 
 
 
 
 
 
 
 
 
 
 
 
 
 
 
 
 
 
 
 
 
 
 
 
 
 
 
 
 
 
 
 
 
 
 
 
 
 
 
 
 
 
 
 
 
 
 
 
 
 
 
 
 
 
 
 
 
 
 
 
 
 
 
 
 
 
 
 
 
 
 
 
 
 
 
 
 
 
 
 
 
 
 
 
 
 
 
 
 
 
 
 
 
 
 
 
 
 
 
 
 
 
 
 
 
 
 
 
 
 
 
 
 
 
 
 
 
 
 
 
 
 
 
 
 
 
 
 
 
 
 
 
 
 
 
 
 
 
 
 
 
 
 
 
 
 
 
 
 
 
 
 
 
 
 
 
 
 
 
 
 

 

    View Table (.txt)         Total  0  1  2  3  4  5  6  8  9  10  11  12  13  14  15  16  17  18  19  20  21  22  23  24  25  26  27  28  29  30  31  42  43  44  45  46  47  49  50  51  53  54  55  56  57  58  59  60  61  62  71  72  81  82  83  84  91  92  93  95  96  98  99  100  101  102  103  104  112  113  114  115  116  117  118  119  120  121  122  123  124  125  126  127  128  129  130  131  132  133  134  135  136  137  138  139  140  141  142  143  144  145  146  147  148  149  150  151  152  153  155  157  158  159  160  161  166  170  171  172  173  174  175  176  177  178  179  180  181  182  183  184  185    Legend  Taxonomy  count  %  %  %  %  %  %  %  %  %  %  %  %  %  %  %  %  %  %  %  %  %  %  %  %  %  %  %  %  %  %  %  %  %  %  %  %  %  %  %  %  %  %  %  %  %  %  %  %  %  %  %  %  %  %  %  %  %  %  %  %  %  %  %  %  %  %  %  %  %  %  %  %  %  %  %  %  %  %  %  %  %  %  %  %  %  %  %  %  %  %  %  %  %  %  %  %  %  %  %  %  %  %  %  %  %  %  %  %  %  %  %  %  %  %  %  %  %  %  %  %  %  %  %  %  %  %  %  %  %  %  %  %  %  %        k__Archaea; p__Crenarchaeota     558    0.1%    0.0%    0.1%    0.0%    0.0%    0.1%    0.0%    0.0%    0.0%    0.0%    0.0%    0.0%    0.0%    0.0%    0.0%    0.0%    0.3%    0.0%    0.1%    0.0%    0.0%    0.0%    0.0%    0.0%    0.0%    0.0%    0.1%    0.1%    0.0%    0.0%    0.0%    0.0%    0.0%    0.0%    0.0%    0.0%    0.0%    0.1%    0.0%    0.0%    0.0%    0.0%    0.1%    0.0%    0.0%    0.0%    0.0%    0.0%    0.0%    0.0%    0.0%    0.0%    0.0%    0.2%    0.2%    0.1%    0.0%    0.0%    0.0%    0.1%    0.1%    0.1%    0.0%    0.0%    0.2%    0.0%    0.2%    0.1%    0.0%    0.1%    0.1%    0.1%    0.2%    0.1%    0.1%    0.0%    0.0%    0.4%    0.1%    0.0%    0.0%    0.0%    0.0%    0.0%    0.1%    0.1%    0.0%    0.0%    0.1%    0.0%    0.1%    0.0%    0.0%    0.1%    0.1%    0.1%    0.0%    0.1%    0.2%    0.0%    0.1%    0.1%    0.1%    0.1%    0.0%    0.0%    0.0%    0.0%    0.3%    0.0%    0.2%    0.2%    0.2%    0.0%    0.0%    0.1%    0.0%    0.0%    0.0%    0.0%    0.0%    0.0%    0.0%    0.0%    0.1%    0.2%    0.1%    0.0%    0.1%    0.1%    0.0%    0.1%    0.1%    0.1%        k__Archaea; p__Euryarchaeota      10    0.0%    0.0%    0.0%    0.0%    0.0%    0.0%    0.0%    0.0%    0.0%    0.0%    0.0%    0.0%    0.0%    0.0%    0.0%    0.0%    0.0%    0.0%    0.0%    0.0%    0.0%    0.0%    0.0%    0.0%    0.0%    0.0%    0.0%    0.0%    0.0%    0.0%    0.0%    0.1%    0.0%    0.0%    0.0%    0.0%    0.0%    0.0%    0.0%    0.0%    0.0%    0.0%    0.0%    0.0%    0.0%    0.0%    0.0%    0.0%    0.0%    0.0%    0.0%    0.0%    0.0%    0.0%    0.0%    0.0%    0.0%    0.0%    0.0%    0.0%    0.0%    0.0%    0.0%    0.0%    0.0%    0.0%    0.0%    0.0%    0.0%    0.0%    0.0%    0.0%    0.0%    0.0%    0.0%    0.0%    0.0%    0.0%    0.0%    0.0%    0.0%    0.0%    0.0%    0.0%    0.0%    0.0%    0.0%    0.0%    0.0%    0.0%    0.0%    0.0%    0.0%    0.0%    0.0%    0.0%    0.0%    0.0%    0.0%    0.0%    0.0%    0.0%    0.0%    0.0%    0.0%    0.0%    0.0%    0.0%    0.0%    0.0%    0.0%    0.0%    0.0%    0.0%    0.0%    0.0%    0.0%    0.0%    0.0%    0.0%    0.0%    0.0%    0.0%    0.0%    0.0%    0.0%    0.0%    0.0%    0.0%    0.0%    0.0%    0.0%    0.0%    0.0%        k__Bacteria; p__       0    0.0%    0.0%    0.0%    0.0%    0.0%    0.0%    0.0%    0.0%    0.0%    0.0%    0.0%    0.0%    0.0%    0.0%    0.0%    0.0%    0.0%    0.0%    0.0%    0.0%    0.0%    0.0%    0.0%    0.0%    0.0%    0.0%    0.0%    0.0%    0.0%    0.0%    0.0%    0.0%    0.0%    0.0%    0.0%    0.0%    0.0%    0.0%    0.0%    0.0%    0.0%    0.0%    0.0%    0.0%    0.0%    0.0%    0.0%    0.0%    0.0%    0.0%    0.0%    0.0%    0.0%    0.0%    0.0%    0.0%    0.0%    0.0%    0.0%    0.0%    0.0%    0.0%    0.0%    0.0%    0.0%    0.0%    0.0%    0.0%    0.0%    0.0%    0.0%    0.0%    0.0%    0.0%    0.0%    0.0%    0.0%    0.0%    0.0%    0.0%    0.0%    0.0%    0.0%    0.0%    0.0%    0.0%    0.0%    0.0%    0.0%    0.0%    0.0%    0.0%    0.0%    0.0%    0.0%    0.0%    0.0%    0.0%    0.0%    0.0%    0.0%    0.0%    0.0%    0.0%    0.0%    0.0%    0.0%    0.0%    0.0%    0.0%    0.0%    0.0%    0.0%    0.0%    0.0%    0.0%    0.0%    0.0%    0.0%    0.0%    0.0%    0.0%    0.0%    0.0%    0.0%    0.0%    0.0%    0.0%    0.0%    0.0%    0.0%    0.0%    0.0%    0.0%        k__Bacteria; p__ABY1_OD1       0    0.0%    0.0%    0.0%    0.0%    0.0%    0.0%    0.0%    0.0%    0.0%    0.0%    0.0%    0.0%    0.0%    0.0%    0.0%    0.0%    0.0%    0.0%    0.0%    0.0%    0.0%    0.0%    0.0%    0.0%    0.0%    0.0%    0.0%    0.0%    0.0%    0.0%    0.0%    0.0%    0.0%    0.0%    0.0%    0.0%    0.0%    0.0%    0.0%    0.0%    0.0%    0.0%    0.0%    0.0%    0.0%    0.0%    0.0%    0.0%    0.0%    0.0%    0.0%    0.0%    0.0%    0.0%    0.0%    0.0%    0.0%    0.0%    0.0%    0.0%    0.0%    0.0%    0.0%    0.0%    0.0%    0.0%    0.0%    0.0%    0.0%    0.0%    0.0%    0.0%    0.0%    0.0%    0.0%    0.0%    0.0%    0.0%    0.0%    0.0%    0.0%    0.0%    0.0%    0.0%    0.0%    0.0%    0.0%    0.0%    0.0%    0.0%    0.0%    0.0%    0.0%    0.0%    0.0%    0.0%    0.0%    0.0%    0.0%    0.0%    0.0%    0.0%    0.0%    0.0%    0.0%    0.0%    0.0%    0.0%    0.0%    0.0%    0.0%    0.0%    0.0%    0.0%    0.0%    0.0%    0.0%    0.0%    0.0%    0.0%    0.0%    0.0%    0.0%    0.0%    0.0%    0.0%    0.0%    0.0%    0.0%    0.0%    0.0%    0.0%    0.0%    0.0%        k__Bacteria; p__AD3       0    0.0%    0.0%    0.0%    0.0%    0.0%    0.0%    0.0%    0.0%    0.0%    0.0%    0.0%    0.0%    0.0%    0.0%    0.0%    0.0%    0.0%    0.0%    0.0%    0.0%    0.0%    0.0%    0.0%    0.0%    0.0%    0.0%    0.0%    0.0%    0.0%    0.0%    0.0%    0.0%    0.0%    0.0%    0.0%    0.0%    0.0%    0.0%    0.0%    0.0%    0.0%    0.0%    0.0%    0.0%    0.0%    0.0%    0.0%    0.0%    0.0%    0.0%    0.0%    0.0%    0.0%    0.0%    0.0%    0.0%    0.0%    0.0%    0.0%    0.0%    0.0%    0.0%    0.0%    0.0%    0.0%    0.0%    0.0%    0.0%    0.0%    0.0%    0.0%    0.0%    0.0%    0.0%    0.0%    0.0%    0.0%    0.0%    0.0%    0.0%    0.0%    0.0%    0.0%    0.0%    0.0%    0.0%    0.0%    0.0%    0.0%    0.0%    0.0%    0.0%    0.0%    0.0%    0.0%    0.0%    0.0%    0.0%    0.0%    0.0%    0.0%    0.0%    0.0%    0.0%    0.0%    0.0%    0.0%    0.0%    0.0%    0.0%    0.0%    0.0%    0.0%    0.0%    0.0%    0.0%    0.0%    0.0%    0.0%    0.0%    0.0%    0.0%    0.0%    0.0%    0.0%    0.0%    0.0%    0.0%    0.0%    0.0%    0.0%    0.0%    0.0%    0.0%        k__Bacteria; p__Acidobacteria    3672    0.4%    0.3%    0.2%    0.2%    0.1%    0.8%    0.4%    0.3%    0.3%    0.1%    0.4%    0.2%    0.0%    0.2%    0.2%    0.1%    0.4%    0.1%    0.5%    0.1%    0.3%    0.2%    0.1%    0.0%    0.5%    0.2%    0.7%    0.4%    0.1%    0.1%    0.3%    0.1%    0.2%    0.2%    0.1%    0.3%    0.1%    0.4%    0.2%    0.3%    0.1%    0.7%    5.2%    0.1%    0.2%    0.0%    0.2%    0.2%    0.2%    0.2%    0.0%    0.1%    0.2%    0.8%    0.7%    0.4%    0.4%    0.0%    0.5%    0.5%    0.9%    0.2%    0.1%    0.0%    0.5%    0.2%    1.2%    0.2%    0.4%    0.9%    0.2%    0.2%    0.9%    0.2%    0.9%    0.1%    0.1%    1.7%    0.6%    0.1%    0.4%    0.3%    0.1%    0.6%    0.7%    0.7%    0.4%    0.5%    0.5%    0.2%    0.3%    0.3%    0.0%    0.3%    0.5%    0.5%    0.2%    0.7%    0.7%    0.3%    0.2%    0.5%    0.1%    0.6%    0.2%    0.1%    0.3%    0.2%    1.0%    0.6%    0.9%    0.8%    0.5%    0.1%    0.3%    0.5%    0.2%    0.2%    0.2%    0.2%    0.4%    0.7%    0.1%    0.3%    0.3%    0.7%    0.3%    0.4%    0.4%    0.6%    0.2%    0.2%    0.3%    0.4%        k__Bacteria; p__Actinobacteria   203850   20.9%   22.5%   24.8%   19.2%   24.4%   14.3%   23.7%   23.6%   12.2%    7.6%   20.5%   24.4%   26.2%   22.8%   23.0%   18.7%   22.9%   34.0%   22.4%   41.9%   21.1%   29.7%   19.3%   32.6%   14.9%   10.4%   19.1%   23.9%   33.6%   42.8%   19.9%   32.8%   30.4%   26.5%   10.4%   17.5%   26.4%   15.6%   12.6%   14.7%   14.4%   25.0%   16.0%   11.7%   15.3%   20.5%   12.7%   17.3%   13.6%   20.0%   35.0%   30.2%   10.0%   19.0%   33.8%   21.1%   20.9%   36.9%   23.1%   26.2%   18.7%   17.7%   28.2%   19.5%   12.8%   37.2%   23.5%   12.1%   18.5%   30.4%   24.8%   14.4%   19.7%   18.5%   27.1%   29.3%   34.1%   27.3%   19.9%   10.8%   17.5%   13.1%   19.9%   16.7%   23.5%   28.7%   16.0%   20.0%   19.6%   17.1%   18.9%   15.0%   38.7%   10.5%   17.5%   26.7%   14.9%   14.6%   11.3%   15.4%    9.0%   22.1%   35.1%   17.9%   12.4%   10.2%   24.4%   10.6%   24.6%   19.0%   24.6%   21.0%   24.3%   11.4%   14.8%   24.6%   15.2%    9.4%   11.8%   12.2%   35.1%   20.9%   14.9%   12.1%   14.3%   17.9%   14.7%   23.2%   21.3%   12.9%   20.7%   15.8%   14.4%   17.8%        k__Bacteria; p__Aquificae      19    0.0%    0.0%    0.0%    0.0%    0.0%    0.0%    0.0%    0.0%    0.0%    0.0%    0.0%    0.0%    0.0%    0.0%    0.0%    0.0%    0.0%    0.0%    0.0%    0.0%    0.0%    0.0%    0.0%    0.0%    0.0%    0.0%    0.0%    0.0%    0.0%    0.0%    0.0%    0.0%    0.0%    0.0%    0.3%    0.0%    0.0%    0.0%    0.0%    0.0%    0.0%    0.0%    0.0%    0.0%    0.0%    0.0%    0.0%    0.0%    0.0%    0.0%    0.0%    0.0%    0.0%    0.0%    0.0%    0.0%    0.0%    0.0%    0.0%    0.0%    0.0%    0.0%    0.0%    0.0%    0.0%    0.0%    0.0%    0.0%    0.0%    0.0%    0.0%    0.0%    0.0%    0.0%    0.0%    0.0%    0.0%    0.0%    0.0%    0.0%    0.0%    0.0%    0.0%    0.0%    0.0%    0.0%    0.0%    0.0%    0.0%    0.0%    0.0%    0.0%    0.0%    0.0%    0.0%    0.0%    0.0%    0.0%    0.0%    0.0%    0.0%    0.0%    0.0%    0.0%    0.0%    0.0%    0.0%    0.0%    0.0%    0.0%    0.0%    0.0%    0.0%    0.0%    0.0%    0.0%    0.0%    0.0%    0.0%    0.0%    0.0%    0.0%    0.0%    0.0%    0.0%    0.0%    0.0%    0.0%    0.0%    0.0%    0.0%    0.0%    0.0%    0.0%        k__Bacteria; p__BRC1      40    0.0%    0.0%    0.0%    0.0%    0.0%    0.0%    0.0%    0.0%    0.0%    0.0%    0.0%    0.0%    0.0%    0.0%    0.0%    0.0%    0.0%    0.0%    0.0%    0.0%    0.0%    0.0%    0.0%    0.0%    0.0%    0.0%    0.0%    0.0%    0.0%    0.0%    0.0%    0.0%    0.0%    0.0%    0.0%    0.0%    0.0%    0.0%    0.0%    0.0%    0.0%    0.0%    0.0%    0.0%    0.0%    0.0%    0.0%    0.0%    0.0%    0.0%    0.0%    0.0%    0.0%    0.0%    0.0%    0.0%    0.0%    0.0%    0.0%    0.0%    0.0%    0.0%    0.0%    0.0%    0.0%    0.0%    0.0%    0.0%    0.0%    0.0%    0.0%    0.0%    0.0%    0.0%    0.0%    0.0%    0.0%    0.0%    0.0%    0.0%    0.0%    0.0%    0.0%    0.0%    0.0%    0.0%    0.0%    0.0%    0.0%    0.0%    0.0%    0.0%    0.0%    0.0%    0.0%    0.0%    0.0%    0.0%    0.0%    0.0%    0.0%    0.0%    0.0%    0.0%    0.0%    0.0%    0.0%    0.0%    0.1%    0.0%    0.0%    0.0%    0.0%    0.0%    0.0%    0.0%    0.0%    0.0%    0.0%    0.0%    0.0%    0.0%    0.0%    0.0%    0.0%    0.0%    0.0%    0.0%    0.0%    0.0%    0.0%    0.0%    0.0%    0.0%        k__Bacteria; p__Bacteroidetes   75938    7.8%    7.5%    4.6%    4.4%    4.0%    7.2%    5.5%    4.8%    4.2%    1.9%    4.6%    9.7%    4.2%    4.7%    6.0%    5.9%    5.8%    4.5%    8.4%    6.4%    9.5%    6.7%    8.3%    5.5%   11.5%   12.4%    9.3%    4.8%    7.4%    5.5%    4.7%    3.9%    5.5%    4.1%   16.3%    3.3%    5.5%    5.5%    9.7%    7.1%    2.7%    6.8%    5.1%    7.0%    5.9%    1.8%    6.4%    7.6%    8.0%    3.8%    3.4%    3.8%    9.2%    8.4%    5.4%    8.4%    3.4%    1.8%    4.9%   11.0%    5.3%    6.1%    1.7%    6.0%    9.3%    3.8%    6.0%    2.9%    5.6%    6.9%    7.2%   11.8%    7.6%   18.9%    6.8%    3.5%    3.8%    8.3%   10.3%   18.7%   12.9%   12.3%   15.3%   15.0%    8.0%    9.0%    6.4%   13.5%    7.2%    7.0%    5.8%   16.4%    5.6%   17.2%   16.0%    8.3%   15.3%   13.6%    8.3%    6.2%    2.5%    8.1%    5.5%    7.4%   10.9%    3.4%    6.8%    3.5%    8.9%    9.9%    4.9%    7.5%    9.6%    6.3%   15.1%    6.6%   20.3%   20.7%   14.4%    7.4%    4.8%    6.9%   16.7%    9.2%   14.6%   11.2%    5.2%    9.0%    7.1%    7.7%    8.2%   13.9%    9.8%    7.8%        k__Bacteria; p__CCM11b       5    0.0%    0.0%    0.0%    0.0%    0.0%    0.0%    0.0%    0.0%    0.0%    0.0%    0.0%    0.0%    0.0%    0.0%    0.0%    0.0%    0.0%    0.0%    0.0%    0.0%    0.0%    0.0%    0.0%    0.0%    0.0%    0.0%    0.0%    0.0%    0.0%    0.0%    0.0%    0.0%    0.0%    0.0%    0.0%    0.0%    0.0%    0.0%    0.0%    0.0%    0.0%    0.0%    0.0%    0.0%    0.0%    0.0%    0.0%    0.0%    0.0%    0.0%    0.0%    0.0%    0.0%    0.0%    0.0%    0.0%    0.0%    0.0%    0.0%    0.0%    0.0%    0.0%    0.0%    0.0%    0.0%    0.0%    0.0%    0.0%    0.0%    0.0%    0.0%    0.0%    0.0%    0.0%    0.0%    0.0%    0.0%    0.0%    0.0%    0.0%    0.0%    0.0%    0.0%    0.0%    0.0%    0.0%    0.0%    0.0%    0.0%    0.0%    0.0%    0.0%    0.0%    0.0%    0.0%    0.0%    0.0%    0.0%    0.0%    0.0%    0.0%    0.0%    0.0%    0.0%    0.0%    0.0%    0.0%    0.0%    0.0%    0.0%    0.0%    0.0%    0.0%    0.0%    0.0%    0.0%    0.0%    0.0%    0.0%    0.0%    0.0%    0.0%    0.0%    0.0%    0.0%    0.0%    0.0%    0.0%    0.0%    0.0%    0.0%    0.0%    0.0%    0.0%        k__Bacteria; p__Caldithrix_KSB1       0    0.0%    0.0%    0.0%    0.0%    0.0%    0.0%    0.0%    0.0%    0.0%    0.0%    0.0%    0.0%    0.0%    0.0%    0.0%    0.0%    0.0%    0.0%    0.0%    0.0%    0.0%    0.0%    0.0%    0.0%    0.0%    0.0%    0.0%    0.0%    0.0%    0.0%    0.0%    0.0%    0.0%    0.0%    0.0%    0.0%    0.0%    0.0%    0.0%    0.0%    0.0%    0.0%    0.0%    0.0%    0.0%    0.0%    0.0%    0.0%    0.0%    0.0%    0.0%    0.0%    0.0%    0.0%    0.0%    0.0%    0.0%    0.0%    0.0%    0.0%    0.0%    0.0%    0.0%    0.0%    0.0%    0.0%    0.0%    0.0%    0.0%    0.0%    0.0%    0.0%    0.0%    0.0%    0.0%    0.0%    0.0%    0.0%    0.0%    0.0%    0.0%    0.0%    0.0%    0.0%    0.0%    0.0%    0.0%    0.0%    0.0%    0.0%    0.0%    0.0%    0.0%    0.0%    0.0%    0.0%    0.0%    0.0%    0.0%    0.0%    0.0%    0.0%    0.0%    0.0%    0.0%    0.0%    0.0%    0.0%    0.0%    0.0%    0.0%    0.0%    0.0%    0.0%    0.0%    0.0%    0.0%    0.0%    0.0%    0.0%    0.0%    0.0%    0.0%    0.0%    0.0%    0.0%    0.0%    0.0%    0.0%    0.0%    0.0%    0.0%    0.0%    0.0%        k__Bacteria; p__Chlamydiae       1    0.0%    0.0%    0.0%    0.0%    0.0%    0.0%    0.0%    0.0%    0.0%    0.0%    0.0%    0.0%    0.0%    0.0%    0.0%    0.0%    0.0%    0.0%    0.0%    0.0%    0.0%    0.0%    0.0%    0.0%    0.0%    0.0%    0.0%    0.0%    0.0%    0.0%    0.0%    0.0%    0.0%    0.0%    0.0%    0.0%    0.0%    0.0%    0.0%    0.0%    0.0%    0.0%    0.0%    0.0%    0.0%    0.0%    0.0%    0.0%    0.0%    0.0%    0.0%    0.0%    0.0%    0.0%    0.0%    0.0%    0.0%    0.0%    0.0%    0.0%    0.0%    0.0%    0.0%    0.0%    0.0%    0.0%    0.0%    0.0%    0.0%    0.0%    0.0%    0.0%    0.0%    0.0%    0.0%    0.0%    0.0%    0.0%    0.0%    0.0%    0.0%    0.0%    0.0%    0.0%    0.0%    0.0%    0.0%    0.0%    0.0%    0.0%    0.0%    0.0%    0.0%    0.0%    0.0%    0.0%    0.0%    0.0%    0.0%    0.0%    0.0%    0.0%    0.0%    0.0%    0.0%    0.0%    0.0%    0.0%    0.0%    0.0%    0.0%    0.0%    0.0%    0.0%    0.0%    0.0%    0.0%    0.0%    0.0%    0.0%    0.0%    0.0%    0.0%    0.0%    0.0%    0.0%    0.0%    0.0%    0.0%    0.0%    0.0%    0.0%    0.0%    0.0%        k__Bacteria; p__Chlorobi       9    0.0%    0.0%    0.0%    0.0%    0.0%    0.0%    0.0%    0.0%    0.0%    0.0%    0.0%    0.0%    0.0%    0.0%    0.0%    0.0%    0.0%    0.0%    0.0%    0.0%    0.0%    0.0%    0.0%    0.0%    0.0%    0.0%    0.0%    0.0%    0.0%    0.0%    0.0%    0.0%    0.0%    0.0%    0.0%    0.0%    0.0%    0.0%    0.0%    0.0%    0.0%    0.0%    0.0%    0.0%    0.0%    0.0%    0.0%    0.0%    0.0%    0.0%    0.0%    0.0%    0.0%    0.0%    0.0%    0.0%    0.0%    0.0%    0.0%    0.0%    0.0%    0.0%    0.0%    0.0%    0.0%    0.0%    0.0%    0.0%    0.0%    0.0%    0.0%    0.0%    0.0%    0.0%    0.0%    0.0%    0.0%    0.0%    0.0%    0.0%    0.0%    0.0%    0.0%    0.0%    0.0%    0.0%    0.0%    0.0%    0.0%    0.0%    0.0%    0.0%    0.0%    0.0%    0.0%    0.0%    0.0%    0.0%    0.0%    0.0%    0.0%    0.0%    0.0%    0.0%    0.0%    0.0%    0.0%    0.0%    0.0%    0.0%    0.0%    0.0%    0.0%    0.0%    0.0%    0.0%    0.0%    0.0%    0.0%    0.0%    0.0%    0.0%    0.0%    0.0%    0.0%    0.0%    0.0%    0.0%    0.0%    0.0%    0.0%    0.0%    0.0%    0.0%        k__Bacteria; p__Chloroflexi    2400    0.2%    0.2%    0.2%    0.7%    0.0%    0.1%    0.1%    0.2%    0.2%    0.1%    0.3%    0.1%    0.1%    0.1%    0.2%    0.2%    0.4%    0.1%    0.3%    0.1%    0.2%    0.2%    0.1%    0.1%    0.0%    0.1%    0.4%    0.2%    0.3%    0.2%    0.1%    0.1%    0.2%    0.1%    0.1%    0.3%    0.1%    0.2%    0.3%    0.5%    0.0%    0.2%    0.2%    0.1%    0.2%    0.0%    0.3%    0.1%    0.2%    0.3%    0.0%    0.0%    0.1%    0.7%    0.4%    0.3%    0.2%    0.1%    0.6%    0.6%    0.5%    0.3%    0.0%    0.0%    0.2%    0.1%    0.6%    0.1%    0.3%    0.5%    0.1%    0.2%    0.5%    0.2%    0.5%    0.0%    0.1%    0.9%    0.3%    0.1%    0.3%    0.1%    0.1%    0.3%    0.4%    0.5%    0.1%    0.3%    0.5%    0.2%    0.4%    0.2%    0.0%    0.4%    0.5%    0.4%    0.2%    0.4%    0.3%    0.3%    0.1%    0.4%    0.2%    0.4%    0.2%    0.1%    0.3%    0.2%    0.8%    0.7%    0.2%    0.4%    0.4%    0.1%    0.2%    0.5%    0.1%    0.3%    0.1%    0.1%    0.4%    0.4%    0.1%    0.2%    0.3%    0.3%    0.2%    0.1%    0.4%    0.7%    0.1%    0.2%    0.2%    0.4%        k__Bacteria; p__Cyanobacteria   52912    5.4%    8.0%    1.2%    2.0%    0.8%    8.5%    1.0%    1.3%   14.5%   34.3%    5.3%    7.7%    0.4%    7.4%    3.7%    2.4%    4.1%    1.2%    5.2%    1.0%    8.2%    1.4%    1.1%    0.5%    6.9%    3.6%    4.3%    2.4%    1.2%    1.0%    2.9%    3.4%    2.6%    1.7%    3.4%    4.2%    8.9%    8.4%    5.8%    7.2%    6.2%    2.8%    2.8%    8.0%    4.4%    1.8%    5.8%    1.5%    5.0%    7.6%    0.8%    0.5%    7.3%    4.2%    2.5%    4.7%   10.1%   11.6%   12.4%    8.5%    3.3%    1.7%    0.2%    1.9%    1.1%    2.2%    6.0%    2.1%   12.5%    3.7%    1.0%    1.5%    1.6%    5.6%    3.6%    0.9%    0.6%    3.5%    6.8%    1.1%    3.5%    4.1%    0.7%    4.0%    6.5%    1.6%    5.5%    1.7%    6.5%   11.8%   19.9%    3.8%    0.5%    1.9%    6.8%    5.7%    5.4%    5.9%    4.8%    6.8%    7.2%    5.2%    1.9%    6.3%    7.0%   14.9%   11.5%    6.1%    3.9%    2.3%    7.2%    6.6%    4.2%   16.0%    5.8%    8.1%    1.4%    4.2%   10.5%   11.4%    7.3%    7.9%    2.6%   17.4%    9.3%    0.9%   19.7%    9.0%    5.5%   19.3%    3.0%   11.6%    9.8%    9.8%        k__Bacteria; p__Deferribacteres      28    0.0%    0.0%    0.0%    0.0%    0.0%    0.0%    0.0%    0.0%    0.0%    0.0%    0.0%    0.0%    0.0%    0.0%    0.0%    0.0%    0.0%    0.0%    0.0%    0.0%    0.0%    0.0%    0.0%    0.0%    0.0%    0.0%    0.0%    0.0%    0.0%    0.0%    0.0%    0.0%    0.0%    0.0%    0.0%    0.0%    0.0%    0.0%    0.0%    0.0%    0.0%    0.0%    0.0%    0.0%    0.0%    0.0%    0.0%    0.0%    0.0%    0.0%    0.0%    0.0%    0.0%    0.0%    0.0%    0.0%    0.0%    0.0%    0.0%    0.1%    0.0%    0.0%    0.0%    0.0%    0.0%    0.0%    0.0%    0.0%    0.0%    0.0%    0.0%    0.0%    0.0%    0.0%    0.0%    0.0%    0.0%    0.0%    0.0%    0.0%    0.0%    0.0%    0.0%    0.0%    0.0%    0.0%    0.0%    0.0%    0.0%    0.0%    0.0%    0.0%    0.0%    0.0%    0.0%    0.0%    0.0%    0.0%    0.0%    0.0%    0.0%    0.0%    0.0%    0.0%    0.0%    0.0%    0.0%    0.0%    0.0%    0.0%    0.0%    0.0%    0.0%    0.0%    0.0%    0.0%    0.0%    0.0%    0.0%    0.0%    0.0%    0.0%    0.0%    0.0%    0.0%    0.0%    0.0%    0.0%    0.0%    0.0%    0.0%    0.0%    0.0%    0.0%        k__Bacteria; p__Elusimicrobia       1    0.0%    0.0%    0.0%    0.0%    0.0%    0.0%    0.0%    0.0%    0.0%    0.0%    0.0%    0.0%    0.0%    0.0%    0.0%    0.0%    0.0%    0.0%    0.0%    0.0%    0.0%    0.0%    0.0%    0.0%    0.0%    0.0%    0.0%    0.0%    0.0%    0.0%    0.0%    0.0%    0.0%    0.0%    0.0%    0.0%    0.0%    0.0%    0.0%    0.0%    0.0%    0.0%    0.0%    0.0%    0.0%    0.0%    0.0%    0.0%    0.0%    0.0%    0.0%    0.0%    0.0%    0.0%    0.0%    0.0%    0.0%    0.0%    0.0%    0.0%    0.0%    0.0%    0.0%    0.0%    0.0%    0.0%    0.0%    0.0%    0.0%    0.0%    0.0%    0.0%    0.0%    0.0%    0.0%    0.0%    0.0%    0.0%    0.0%    0.0%    0.0%    0.0%    0.0%    0.0%    0.0%    0.0%    0.0%    0.0%    0.0%    0.0%    0.0%    0.0%    0.0%    0.0%    0.0%    0.0%    0.0%    0.0%    0.0%    0.0%    0.0%    0.0%    0.0%    0.0%    0.0%    0.0%    0.0%    0.0%    0.0%    0.0%    0.0%    0.0%    0.0%    0.0%    0.0%    0.0%    0.0%    0.0%    0.0%    0.0%    0.0%    0.0%    0.0%    0.0%    0.0%    0.0%    0.0%    0.0%    0.0%    0.0%    0.0%    0.0%    0.0%    0.0%        k__Bacteria; p__Fibrobacteres       0    0.0%    0.0%    0.0%    0.0%    0.0%    0.0%    0.0%    0.0%    0.0%    0.0%    0.0%    0.0%    0.0%    0.0%    0.0%    0.0%    0.0%    0.0%    0.0%    0.0%    0.0%    0.0%    0.0%    0.0%    0.0%    0.0%    0.0%    0.0%    0.0%    0.0%    0.0%    0.0%    0.0%    0.0%    0.0%    0.0%    0.0%    0.0%    0.0%    0.0%    0.0%    0.0%    0.0%    0.0%    0.0%    0.0%    0.0%    0.0%    0.0%    0.0%    0.0%    0.0%    0.0%    0.0%    0.0%    0.0%    0.0%    0.0%    0.0%    0.0%    0.0%    0.0%    0.0%    0.0%    0.0%    0.0%    0.0%    0.0%    0.0%    0.0%    0.0%    0.0%    0.0%    0.0%    0.0%    0.0%    0.0%    0.0%    0.0%    0.0%    0.0%    0.0%    0.0%    0.0%    0.0%    0.0%    0.0%    0.0%    0.0%    0.0%    0.0%    0.0%    0.0%    0.0%    0.0%    0.0%    0.0%    0.0%    0.0%    0.0%    0.0%    0.0%    0.0%    0.0%    0.0%    0.0%    0.0%    0.0%    0.0%    0.0%    0.0%    0.0%    0.0%    0.0%    0.0%    0.0%    0.0%    0.0%    0.0%    0.0%    0.0%    0.0%    0.0%    0.0%    0.0%    0.0%    0.0%    0.0%    0.0%    0.0%    0.0%    0.0%    0.0%    0.0%        k__Bacteria; p__Firmicutes   305895   31.4%   26.9%   15.5%   41.5%   44.2%   32.5%   33.8%   54.6%   47.7%   25.1%   41.7%   21.3%   58.2%   43.2%   39.6%   39.7%   25.2%   36.0%   30.6%   36.6%   21.7%   33.3%   28.0%   42.5%   34.6%   20.0%   30.6%   35.8%   32.3%   35.4%   50.3%   32.9%   22.5%   40.1%   30.7%   36.5%   40.3%   21.7%   23.7%   26.7%   27.4%   30.4%   19.4%   20.3%   26.1%   38.6%   29.9%   29.5%   20.9%   24.7%   44.1%   36.4%   36.2%   30.4%   28.8%   22.2%   27.8%   18.9%   23.3%   24.3%   39.2%   44.8%   64.2%   61.6%   33.7%   44.2%   30.6%   24.6%   29.6%   29.9%   44.1%   42.3%   27.7%   26.8%   31.4%   54.1%   46.9%   19.3%   25.4%   31.8%   31.0%   31.9%   29.4%   29.0%   28.7%   28.8%   38.1%   31.7%   24.8%   42.9%   19.4%   38.8%   37.7%   23.4%   18.9%   28.4%   29.9%   29.0%   30.6%   29.9%   31.9%   32.9%   32.8%   26.2%   25.5%   20.6%   31.4%   37.2%   23.9%   14.9%   29.2%   26.6%   36.8%   30.1%   15.2%   27.3%    7.2%    8.1%   17.3%   46.6%   28.7%   29.3%   41.3%   15.8%   15.7%   19.7%   23.4%   26.4%   25.8%    8.9%   31.3%   23.4%   32.3%   30.2%        k__Bacteria; p__Fusobacteria   29286    3.0%    2.7%    1.6%    1.3%    1.6%    1.9%    1.0%    0.7%    0.8%    0.3%    1.1%    2.9%    0.8%    1.4%    2.2%    1.3%    0.7%    1.4%    1.4%    0.9%    1.1%    2.3%   16.9%    2.0%    3.2%    2.4%    0.7%    1.3%    4.3%    2.0%    1.5%    1.6%    1.2%    0.4%    1.1%    0.7%    1.2%    8.9%    9.6%    5.3%    1.4%    4.2%    8.1%   21.9%   14.0%    2.3%    9.8%   15.0%   11.4%    6.6%    3.4%    1.5%    7.5%    7.3%    4.8%    7.1%    3.0%    0.8%    8.0%    6.9%    5.5%    3.5%    0.7%    1.4%    8.8%    1.7%    2.2%   21.8%    4.4%    3.0%    1.1%    3.2%    2.8%    5.3%    2.2%    1.2%    1.2%    1.1%    2.8%    3.4%    2.5%    1.0%    1.1%    1.8%    2.4%    1.8%    2.2%    5.6%    3.7%    1.2%    1.3%    3.8%    2.6%    1.8%    2.4%    2.4%    8.1%    4.7%    1.8%    1.8%    0.7%    2.5%    0.9%    2.0%    1.1%    0.4%    1.0%    0.8%    0.7%    0.2%    0.8%    1.7%    2.4%    1.0%    0.3%    1.1%    0.3%    0.2%    0.6%    1.0%    1.0%    1.0%    1.7%    1.4%    1.6%    1.5%    0.9%    1.6%    1.5%    0.6%    1.2%    1.3%    1.1%    1.0%        k__Bacteria; p__GN02       0    0.0%    0.0%    0.0%    0.0%    0.0%    0.0%    0.0%    0.0%    0.0%    0.0%    0.0%    0.0%    0.0%    0.0%    0.0%    0.0%    0.0%    0.0%    0.0%    0.0%    0.0%    0.0%    0.0%    0.0%    0.0%    0.0%    0.0%    0.0%    0.0%    0.0%    0.0%    0.0%    0.0%    0.0%    0.0%    0.0%    0.0%    0.0%    0.0%    0.0%    0.0%    0.0%    0.0%    0.0%    0.0%    0.0%    0.0%    0.0%    0.0%    0.0%    0.0%    0.0%    0.0%    0.0%    0.0%    0.0%    0.0%    0.0%    0.0%    0.0%    0.0%    0.0%    0.0%    0.0%    0.0%    0.0%    0.0%    0.0%    0.0%    0.0%    0.0%    0.0%    0.0%    0.0%    0.0%    0.0%    0.0%    0.0%    0.0%    0.0%    0.0%    0.0%    0.0%    0.0%    0.0%    0.0%    0.0%    0.0%    0.0%    0.0%    0.0%    0.0%    0.0%    0.0%    0.0%    0.0%    0.0%    0.0%    0.0%    0.0%    0.0%    0.0%    0.0%    0.0%    0.0%    0.0%    0.0%    0.0%    0.0%    0.0%    0.0%    0.0%    0.0%    0.0%    0.0%    0.0%    0.0%    0.0%    0.0%    0.0%    0.0%    0.0%    0.0%    0.0%    0.0%    0.0%    0.0%    0.0%    0.0%    0.0%    0.0%    0.0%    0.0%    0.0%        k__Bacteria; p__Gemmatimonadetes     510    0.1%    0.1%    0.0%    0.1%    0.1%    0.2%    0.1%    0.0%    0.0%    0.0%    0.1%    0.0%    0.0%    0.0%    0.0%    0.0%    0.1%    0.0%    0.1%    0.0%    0.0%    0.1%    0.0%    0.0%    0.1%    0.0%    0.1%    0.1%    0.0%    0.0%    0.0%    0.0%    0.0%    0.0%    0.0%    0.0%    0.0%    0.1%    0.0%    0.0%    0.0%    0.0%    0.0%    0.0%    0.0%    0.1%    0.0%    0.0%    0.0%    0.0%    0.0%    0.0%    0.0%    0.1%    0.0%    0.1%    0.0%    0.0%    0.1%    0.0%    0.1%    0.0%    0.0%    0.0%    0.1%    0.0%    0.2%    0.0%    0.0%    0.1%    0.0%    0.0%    0.1%    0.0%    0.1%    0.0%    0.0%    0.2%    0.0%    0.0%    0.1%    0.1%    0.0%    0.1%    0.1%    0.0%    0.1%    0.1%    0.1%    0.1%    0.0%    0.0%    0.0%    0.1%    0.1%    0.1%    0.1%    0.1%    0.1%    0.1%    0.0%    0.1%    0.0%    0.2%    0.1%    0.0%    0.0%    0.1%    0.2%    0.1%    0.1%    0.1%    0.1%    0.0%    0.0%    0.2%    0.0%    0.0%    0.0%    0.0%    0.0%    0.0%    0.0%    0.1%    0.1%    0.1%    0.0%    0.1%    0.1%    0.1%    0.0%    0.0%    0.0%    0.1%        k__Bacteria; p__Lentisphaerae       0    0.0%    0.0%    0.0%    0.0%    0.0%    0.0%    0.0%    0.0%    0.0%    0.0%    0.0%    0.0%    0.0%    0.0%    0.0%    0.0%    0.0%    0.0%    0.0%    0.0%    0.0%    0.0%    0.0%    0.0%    0.0%    0.0%    0.0%    0.0%    0.0%    0.0%    0.0%    0.0%    0.0%    0.0%    0.0%    0.0%    0.0%    0.0%    0.0%    0.0%    0.0%    0.0%    0.0%    0.0%    0.0%    0.0%    0.0%    0.0%    0.0%    0.0%    0.0%    0.0%    0.0%    0.0%    0.0%    0.0%    0.0%    0.0%    0.0%    0.0%    0.0%    0.0%    0.0%    0.0%    0.0%    0.0%    0.0%    0.0%    0.0%    0.0%    0.0%    0.0%    0.0%    0.0%    0.0%    0.0%    0.0%    0.0%    0.0%    0.0%    0.0%    0.0%    0.0%    0.0%    0.0%    0.0%    0.0%    0.0%    0.0%    0.0%    0.0%    0.0%    0.0%    0.0%    0.0%    0.0%    0.0%    0.0%    0.0%    0.0%    0.0%    0.0%    0.0%    0.0%    0.0%    0.0%    0.0%    0.0%    0.0%    0.0%    0.0%    0.0%    0.0%    0.0%    0.0%    0.0%    0.0%    0.0%    0.0%    0.0%    0.0%    0.0%    0.0%    0.0%    0.0%    0.0%    0.0%    0.0%    0.0%    0.0%    0.0%    0.0%    0.0%    0.0%        k__Bacteria; p__MVP-15       0    0.0%    0.0%    0.0%    0.0%    0.0%    0.0%    0.0%    0.0%    0.0%    0.0%    0.0%    0.0%    0.0%    0.0%    0.0%    0.0%    0.0%    0.0%    0.0%    0.0%    0.0%    0.0%    0.0%    0.0%    0.0%    0.0%    0.0%    0.0%    0.0%    0.0%    0.0%    0.0%    0.0%    0.0%    0.0%    0.0%    0.0%    0.0%    0.0%    0.0%    0.0%    0.0%    0.0%    0.0%    0.0%    0.0%    0.0%    0.0%    0.0%    0.0%    0.0%    0.0%    0.0%    0.0%    0.0%    0.0%    0.0%    0.0%    0.0%    0.0%    0.0%    0.0%    0.0%    0.0%    0.0%    0.0%    0.0%    0.0%    0.0%    0.0%    0.0%    0.0%    0.0%    0.0%    0.0%    0.0%    0.0%    0.0%    0.0%    0.0%    0.0%    0.0%    0.0%    0.0%    0.0%    0.0%    0.0%    0.0%    0.0%    0.0%    0.0%    0.0%    0.0%    0.0%    0.0%    0.0%    0.0%    0.0%    0.0%    0.0%    0.0%    0.0%    0.0%    0.0%    0.0%    0.0%    0.0%    0.0%    0.0%    0.0%    0.0%    0.0%    0.0%    0.0%    0.0%    0.0%    0.0%    0.0%    0.0%    0.0%    0.0%    0.0%    0.0%    0.0%    0.0%    0.0%    0.0%    0.0%    0.0%    0.0%    0.0%    0.0%    0.0%    0.0%        k__Bacteria; p__NC10       0    0.0%    0.0%    0.0%    0.0%    0.0%    0.0%    0.0%    0.0%    0.0%    0.0%    0.0%    0.0%    0.0%    0.0%    0.0%    0.0%    0.0%    0.0%    0.0%    0.0%    0.0%    0.0%    0.0%    0.0%    0.0%    0.0%    0.0%    0.0%    0.0%    0.0%    0.0%    0.0%    0.0%    0.0%    0.0%    0.0%    0.0%    0.0%    0.0%    0.0%    0.0%    0.0%    0.0%    0.0%    0.0%    0.0%    0.0%    0.0%    0.0%    0.0%    0.0%    0.0%    0.0%    0.0%    0.0%    0.0%    0.0%    0.0%    0.0%    0.0%    0.0%    0.0%    0.0%    0.0%    0.0%    0.0%    0.0%    0.0%    0.0%    0.0%    0.0%    0.0%    0.0%    0.0%    0.0%    0.0%    0.0%    0.0%    0.0%    0.0%    0.0%    0.0%    0.0%    0.0%    0.0%    0.0%    0.0%    0.0%    0.0%    0.0%    0.0%    0.0%    0.0%    0.0%    0.0%    0.0%    0.0%    0.0%    0.0%    0.0%    0.0%    0.0%    0.0%    0.0%    0.0%    0.0%    0.0%    0.0%    0.0%    0.0%    0.0%    0.0%    0.0%    0.0%    0.0%    0.0%    0.0%    0.0%    0.0%    0.0%    0.0%    0.0%    0.0%    0.0%    0.0%    0.0%    0.0%    0.0%    0.0%    0.0%    0.0%    0.0%    0.0%    0.0%        k__Bacteria; p__NKB19       1    0.0%    0.0%    0.0%    0.0%    0.0%    0.0%    0.0%    0.0%    0.0%    0.0%    0.0%    0.0%    0.0%    0.0%    0.0%    0.0%    0.0%    0.0%    0.0%    0.0%    0.0%    0.0%    0.0%    0.0%    0.0%    0.0%    0.0%    0.0%    0.0%    0.0%    0.0%    0.0%    0.0%    0.0%    0.0%    0.0%    0.0%    0.0%    0.0%    0.0%    0.0%    0.0%    0.0%    0.0%    0.0%    0.0%    0.0%    0.0%    0.0%    0.0%    0.0%    0.0%    0.0%    0.0%    0.0%    0.0%    0.0%    0.0%    0.0%    0.0%    0.0%    0.0%    0.0%    0.0%    0.0%    0.0%    0.0%    0.0%    0.0%    0.0%    0.0%    0.0%    0.0%    0.0%    0.0%    0.0%    0.0%    0.0%    0.0%    0.0%    0.0%    0.0%    0.0%    0.0%    0.0%    0.0%    0.0%    0.0%    0.0%    0.0%    0.0%    0.0%    0.0%    0.0%    0.0%    0.0%    0.0%    0.0%    0.0%    0.0%    0.0%    0.0%    0.0%    0.0%    0.0%    0.0%    0.0%    0.0%    0.0%    0.0%    0.0%    0.0%    0.0%    0.0%    0.0%    0.0%    0.0%    0.0%    0.0%    0.0%    0.0%    0.0%    0.0%    0.0%    0.0%    0.0%    0.0%    0.0%    0.0%    0.0%    0.0%    0.0%    0.0%    0.0%        k__Bacteria; p__Nitrospirae      99    0.0%    0.0%    0.0%    0.0%    0.0%    0.1%    0.0%    0.0%    0.0%    0.0%    0.0%    0.0%    0.0%    0.0%    0.0%    0.0%    0.0%    0.0%    0.0%    0.0%    0.0%    0.0%    0.0%    0.0%    0.0%    0.0%    0.0%    0.0%    0.0%    0.0%    0.0%    0.0%    0.0%    0.0%    0.0%    0.0%    0.0%    0.0%    0.0%    0.0%    0.0%    0.0%    0.0%    0.0%    0.0%    0.0%    0.0%    0.0%    0.0%    0.0%    0.0%    0.0%    0.0%    0.0%    0.0%    0.0%    0.0%    0.0%    0.0%    0.0%    0.1%    0.0%    0.0%    0.0%    0.0%    0.0%    0.0%    0.0%    0.0%    0.1%    0.0%    0.0%    0.0%    0.0%    0.0%    0.0%    0.0%    0.0%    0.0%    0.0%    0.0%    0.0%    0.0%    0.0%    0.0%    0.0%    0.0%    0.0%    0.0%    0.0%    0.0%    0.0%    0.0%    0.0%    0.0%    0.0%    0.0%    0.0%    0.1%    0.0%    0.0%    0.0%    0.0%    0.0%    0.0%    0.0%    0.0%    0.0%    0.1%    0.0%    0.0%    0.0%    0.0%    0.0%    0.0%    0.0%    0.0%    0.0%    0.0%    0.0%    0.0%    0.0%    0.0%    0.0%    0.0%    0.0%    0.0%    0.0%    0.0%    0.0%    0.0%    0.0%    0.0%    0.0%        k__Bacteria; p__OP10      55    0.0%    0.1%    0.0%    0.0%    0.0%    0.0%    0.0%    0.0%    0.0%    0.0%    0.0%    0.0%    0.0%    0.0%    0.0%    0.0%    0.0%    0.0%    0.0%    0.0%    0.0%    0.0%    0.0%    0.0%    0.0%    0.0%    0.0%    0.0%    0.0%    0.0%    0.0%    0.0%    0.0%    0.0%    0.0%    0.0%    0.0%    0.0%    0.0%    0.0%    0.0%    0.1%    0.0%    0.0%    0.0%    0.0%    0.0%    0.0%    0.0%    0.0%    0.0%    0.0%    0.0%    0.0%    0.0%    0.0%    0.0%    0.0%    0.0%    0.0%    0.0%    0.0%    0.0%    0.0%    0.0%    0.0%    0.0%    0.0%    0.0%    0.0%    0.0%    0.0%    0.0%    0.0%    0.0%    0.0%    0.0%    0.0%    0.0%    0.0%    0.0%    0.0%    0.0%    0.0%    0.0%    0.0%    0.0%    0.0%    0.0%    0.0%    0.0%    0.0%    0.0%    0.0%    0.0%    0.0%    0.0%    0.0%    0.0%    0.0%    0.0%    0.0%    0.0%    0.0%    0.0%    0.0%    0.0%    0.0%    0.0%    0.0%    0.0%    0.0%    0.0%    0.0%    0.0%    0.0%    0.0%    0.0%    0.0%    0.0%    0.0%    0.0%    0.0%    0.0%    0.0%    0.0%    0.0%    0.0%    0.0%    0.0%    0.0%    0.0%    0.0%    0.0%        k__Bacteria; p__OP11       0    0.0%    0.0%    0.0%    0.0%    0.0%    0.0%    0.0%    0.0%    0.0%    0.0%    0.0%    0.0%    0.0%    0.0%    0.0%    0.0%    0.0%    0.0%    0.0%    0.0%    0.0%    0.0%    0.0%    0.0%    0.0%    0.0%    0.0%    0.0%    0.0%    0.0%    0.0%    0.0%    0.0%    0.0%    0.0%    0.0%    0.0%    0.0%    0.0%    0.0%    0.0%    0.0%    0.0%    0.0%    0.0%    0.0%    0.0%    0.0%    0.0%    0.0%    0.0%    0.0%    0.0%    0.0%    0.0%    0.0%    0.0%    0.0%    0.0%    0.0%    0.0%    0.0%    0.0%    0.0%    0.0%    0.0%    0.0%    0.0%    0.0%    0.0%    0.0%    0.0%    0.0%    0.0%    0.0%    0.0%    0.0%    0.0%    0.0%    0.0%    0.0%    0.0%    0.0%    0.0%    0.0%    0.0%    0.0%    0.0%    0.0%    0.0%    0.0%    0.0%    0.0%    0.0%    0.0%    0.0%    0.0%    0.0%    0.0%    0.0%    0.0%    0.0%    0.0%    0.0%    0.0%    0.0%    0.0%    0.0%    0.0%    0.0%    0.0%    0.0%    0.0%    0.0%    0.0%    0.0%    0.0%    0.0%    0.0%    0.0%    0.0%    0.0%    0.0%    0.0%    0.0%    0.0%    0.0%    0.0%    0.0%    0.0%    0.0%    0.0%    0.0%    0.0%        k__Bacteria; p__OP3       1    0.0%    0.0%    0.0%    0.0%    0.0%    0.0%    0.0%    0.0%    0.0%    0.0%    0.0%    0.0%    0.0%    0.0%    0.0%    0.0%    0.0%    0.0%    0.0%    0.0%    0.0%    0.0%    0.0%    0.0%    0.0%    0.0%    0.0%    0.0%    0.0%    0.0%    0.0%    0.0%    0.0%    0.0%    0.0%    0.0%    0.0%    0.0%    0.0%    0.0%    0.0%    0.0%    0.0%    0.0%    0.0%    0.0%    0.0%    0.0%    0.0%    0.0%    0.0%    0.0%    0.0%    0.0%    0.0%    0.0%    0.0%    0.0%    0.0%    0.0%    0.0%    0.0%    0.0%    0.0%    0.0%    0.0%    0.0%    0.0%    0.0%    0.0%    0.0%    0.0%    0.0%    0.0%    0.0%    0.0%    0.0%    0.0%    0.0%    0.0%    0.0%    0.0%    0.0%    0.0%    0.0%    0.0%    0.0%    0.0%    0.0%    0.0%    0.0%    0.0%    0.0%    0.0%    0.0%    0.0%    0.0%    0.0%    0.0%    0.0%    0.0%    0.0%    0.0%    0.0%    0.0%    0.0%    0.0%    0.0%    0.0%    0.0%    0.0%    0.0%    0.0%    0.0%    0.0%    0.0%    0.0%    0.0%    0.0%    0.0%    0.0%    0.0%    0.0%    0.0%    0.0%    0.0%    0.0%    0.0%    0.0%    0.0%    0.0%    0.0%    0.0%    0.0%        k__Bacteria; p__OP8       0    0.0%    0.0%    0.0%    0.0%    0.0%    0.0%    0.0%    0.0%    0.0%    0.0%    0.0%    0.0%    0.0%    0.0%    0.0%    0.0%    0.0%    0.0%    0.0%    0.0%    0.0%    0.0%    0.0%    0.0%    0.0%    0.0%    0.0%    0.0%    0.0%    0.0%    0.0%    0.0%    0.0%    0.0%    0.0%    0.0%    0.0%    0.0%    0.0%    0.0%    0.0%    0.0%    0.0%    0.0%    0.0%    0.0%    0.0%    0.0%    0.0%    0.0%    0.0%    0.0%    0.0%    0.0%    0.0%    0.0%    0.0%    0.0%    0.0%    0.0%    0.0%    0.0%    0.0%    0.0%    0.0%    0.0%    0.0%    0.0%    0.0%    0.0%    0.0%    0.0%    0.0%    0.0%    0.0%    0.0%    0.0%    0.0%    0.0%    0.0%    0.0%    0.0%    0.0%    0.0%    0.0%    0.0%    0.0%    0.0%    0.0%    0.0%    0.0%    0.0%    0.0%    0.0%    0.0%    0.0%    0.0%    0.0%    0.0%    0.0%    0.0%    0.0%    0.0%    0.0%    0.0%    0.0%    0.0%    0.0%    0.0%    0.0%    0.0%    0.0%    0.0%    0.0%    0.0%    0.0%    0.0%    0.0%    0.0%    0.0%    0.0%    0.0%    0.0%    0.0%    0.0%    0.0%    0.0%    0.0%    0.0%    0.0%    0.0%    0.0%    0.0%    0.0%        k__Bacteria; p__Planctomycetes     710    0.1%    0.1%    0.1%    0.0%    0.0%    0.1%    0.0%    0.0%    0.1%    0.0%    0.1%    0.1%    0.0%    0.0%    0.1%    0.0%    0.1%    0.1%    0.1%    0.0%    0.1%    0.1%    0.0%    0.0%    0.0%    0.0%    0.2%    0.1%    0.1%    0.1%    0.0%    0.0%    0.0%    0.0%    0.1%    0.2%    0.0%    0.0%    0.0%    0.1%    0.0%    0.1%    0.0%    0.0%    0.1%    0.0%    0.0%    0.0%    0.1%    0.0%    0.0%    0.0%    0.1%    0.2%    0.1%    0.1%    0.0%    0.0%    0.1%    0.1%    0.3%    0.0%    0.0%    0.0%    0.1%    0.0%    0.4%    0.1%    0.0%    0.1%    0.0%    0.1%    0.2%    0.1%    0.2%    0.0%    0.0%    0.3%    0.1%    0.0%    0.0%    0.0%    0.0%    0.1%    0.1%    0.1%    0.0%    0.2%    0.1%    0.1%    0.2%    0.0%    0.0%    0.0%    0.1%    0.1%    0.1%    0.1%    0.1%    0.2%    0.0%    0.1%    0.0%    0.1%    0.1%    0.1%    0.1%    0.0%    0.5%    0.3%    0.1%    0.2%    0.1%    0.0%    0.1%    0.1%    0.0%    0.1%    0.0%    0.0%    0.0%    0.1%    0.0%    0.1%    0.1%    0.1%    0.1%    0.0%    0.1%    0.1%    0.0%    0.1%    0.0%    0.1%        k__Bacteria; p__Proteobacteria   284434   29.2%   30.7%   50.6%   28.1%   24.6%   33.4%   33.9%   13.8%   19.2%   30.3%   24.7%   31.4%    9.7%   19.4%   23.8%   29.9%   39.2%   21.0%   29.7%   12.1%   37.2%   24.3%   23.6%   16.2%   27.4%   49.5%   33.5%   30.1%   18.2%   11.9%   19.5%   23.6%   36.4%   26.2%   31.7%   35.8%   16.9%   36.2%   35.4%   35.5%   47.0%   28.4%   41.7%   28.7%   31.6%   34.6%   32.9%   27.1%   38.2%   34.5%   12.4%   26.1%   28.8%   27.4%   22.1%   34.2%   32.5%   29.2%   25.2%   20.1%   24.5%   24.2%    3.2%    9.0%   29.2%    9.9%   27.9%   21.7%   27.4%   23.2%   20.9%   24.5%   36.6%   20.8%   25.6%   10.2%   10.9%   35.9%   32.9%   30.5%   29.7%   36.5%   32.9%   30.4%   28.1%   27.4%   29.7%   24.2%   35.2%   18.8%   31.9%   18.6%   13.8%   42.4%   34.0%   25.7%   22.8%   28.1%   40.5%   38.5%   47.8%   26.7%   21.4%   36.4%   41.0%   48.6%   23.1%   40.1%   33.3%   50.0%   29.7%   33.1%   19.8%   34.6%   47.4%   29.6%   54.7%   56.0%   44.3%   20.5%   21.1%   29.6%   22.0%   42.3%   42.2%   46.4%   34.1%   28.8%   36.0%   47.6%   34.0%   32.2%   31.4%   31.5%        k__Bacteria; p__SC3       0    0.0%    0.0%    0.0%    0.0%    0.0%    0.0%    0.0%    0.0%    0.0%    0.0%    0.0%    0.0%    0.0%    0.0%    0.0%    0.0%    0.0%    0.0%    0.0%    0.0%    0.0%    0.0%    0.0%    0.0%    0.0%    0.0%    0.0%    0.0%    0.0%    0.0%    0.0%    0.0%    0.0%    0.0%    0.0%    0.0%    0.0%    0.0%    0.0%    0.0%    0.0%    0.0%    0.0%    0.0%    0.0%    0.0%    0.0%    0.0%    0.0%    0.0%    0.0%    0.0%    0.0%    0.0%    0.0%    0.0%    0.0%    0.0%    0.0%    0.0%    0.0%    0.0%    0.0%    0.0%    0.0%    0.0%    0.0%    0.0%    0.0%    0.0%    0.0%    0.0%    0.0%    0.0%    0.0%    0.0%    0.0%    0.0%    0.0%    0.0%    0.0%    0.0%    0.0%    0.0%    0.0%    0.0%    0.0%    0.0%    0.0%    0.0%    0.0%    0.0%    0.0%    0.0%    0.0%    0.0%    0.0%    0.0%    0.0%    0.0%    0.0%    0.0%    0.0%    0.0%    0.0%    0.0%    0.0%    0.0%    0.0%    0.0%    0.0%    0.0%    0.0%    0.0%    0.0%    0.0%    0.0%    0.0%    0.0%    0.0%    0.0%    0.0%    0.0%    0.0%    0.0%    0.0%    0.0%    0.0%    0.0%    0.0%    0.0%    0.0%    0.0%    0.0%        k__Bacteria; p__SC4      98    0.0%    0.0%    0.0%    0.0%    0.0%    0.0%    0.0%    0.0%    0.0%    0.0%    0.0%    0.0%    0.0%    0.0%    0.0%    0.0%    0.0%    0.0%    0.0%    0.0%    0.1%    0.0%    0.0%    0.0%    0.0%    0.0%    0.1%    0.0%    0.0%    0.0%    0.0%    0.0%    0.0%    0.0%    0.0%    0.0%    0.0%    0.0%    0.0%    0.0%    0.0%    0.0%    0.0%    0.0%    0.0%    0.0%    0.0%    0.0%    0.0%    0.0%    0.0%    0.0%    0.0%    0.0%    0.0%    0.0%    0.0%    0.0%    0.0%    0.0%    0.0%    0.0%    0.0%    0.0%    0.0%    0.0%    0.1%    0.0%    0.0%    0.0%    0.0%    0.0%    0.0%    0.0%    0.0%    0.0%    0.0%    0.1%    0.0%    0.0%    0.0%    0.0%    0.0%    0.0%    0.0%    0.1%    0.0%    0.0%    0.0%    0.0%    0.0%    0.0%    0.0%    0.0%    0.0%    0.0%    0.0%    0.0%    0.0%    0.0%    0.0%    0.0%    0.0%    0.1%    0.0%    0.0%    0.0%    0.0%    0.0%    0.0%    0.0%    0.0%    0.0%    0.0%    0.0%    0.0%    0.0%    0.0%    0.0%    0.0%    0.0%    0.0%    0.0%    0.0%    0.0%    0.0%    0.0%    0.0%    0.0%    0.0%    0.0%    0.0%    0.0%    0.0%        k__Bacteria; p__SPAM      26    0.0%    0.0%    0.0%    0.0%    0.0%    0.0%    0.0%    0.0%    0.0%    0.0%    0.0%    0.0%    0.0%    0.0%    0.0%    0.0%    0.0%    0.0%    0.0%    0.0%    0.0%    0.0%    0.0%    0.0%    0.0%    0.0%    0.0%    0.0%    0.0%    0.0%    0.0%    0.0%    0.0%    0.0%    0.0%    0.0%    0.0%    0.0%    0.0%    0.0%    0.0%    0.0%    0.0%    0.0%    0.0%    0.0%    0.0%    0.0%    0.0%    0.0%    0.0%    0.0%    0.0%    0.0%    0.0%    0.0%    0.0%    0.0%    0.0%    0.0%    0.0%    0.0%    0.0%    0.0%    0.0%    0.0%    0.0%    0.0%    0.0%    0.0%    0.0%    0.0%    0.0%    0.0%    0.0%    0.0%    0.0%    0.0%    0.0%    0.0%    0.0%    0.0%    0.0%    0.0%    0.0%    0.0%    0.0%    0.0%    0.0%    0.0%    0.0%    0.0%    0.0%    0.0%    0.0%    0.0%    0.0%    0.0%    0.0%    0.0%    0.0%    0.0%    0.0%    0.0%    0.0%    0.0%    0.0%    0.0%    0.0%    0.0%    0.0%    0.0%    0.0%    0.0%    0.0%    0.0%    0.0%    0.0%    0.0%    0.0%    0.0%    0.0%    0.0%    0.0%    0.0%    0.0%    0.0%    0.0%    0.0%    0.0%    0.0%    0.0%    0.0%    0.0%        k__Bacteria; p__SR1     139    0.0%    0.0%    0.0%    0.0%    0.0%    0.0%    0.0%    0.0%    0.0%    0.0%    0.0%    0.0%    0.0%    0.0%    0.0%    0.0%    0.0%    0.0%    0.0%    0.0%    0.0%    0.0%    0.0%    0.0%    0.0%    0.1%    0.0%    0.0%    0.0%    0.0%    0.0%    0.0%    0.0%    0.0%    0.0%    0.0%    0.0%    0.0%    0.0%    0.0%    0.0%    0.0%    0.1%    0.0%    0.0%    0.0%    0.0%    0.0%    0.2%    0.0%    0.0%    0.0%    0.0%    0.0%    0.0%    0.0%    0.0%    0.0%    0.0%    0.0%    0.0%    0.1%    0.0%    0.0%    0.0%    0.0%    0.0%    0.0%    0.0%    0.0%    0.0%    0.0%    0.0%    0.0%    0.0%    0.0%    0.0%    0.0%    0.0%    0.0%    0.0%    0.0%    0.0%    0.0%    0.0%    0.0%    0.0%    0.1%    0.0%    0.0%    0.0%    0.1%    0.0%    0.0%    0.0%    0.0%    0.0%    0.0%    0.0%    0.0%    0.0%    0.0%    0.0%    0.0%    0.0%    0.0%    0.0%    0.0%    0.0%    0.0%    0.0%    0.0%    0.0%    0.0%    0.0%    0.0%    0.0%    0.0%    0.0%    0.1%    0.0%    0.0%    0.0%    0.0%    0.1%    0.1%    0.0%    0.0%    0.0%    0.0%    0.0%    0.0%    0.0%    0.0%        k__Bacteria; p__Spirochaetes     997    0.1%    0.0%    0.0%    0.0%    0.0%    0.0%    0.0%    0.0%    0.0%    0.0%    0.0%    0.8%    0.1%    0.0%    0.1%    0.0%    0.1%    0.0%    0.1%    0.0%    0.0%    0.1%    0.2%    0.0%    0.2%    0.2%    0.0%    0.0%    0.1%    0.1%    0.1%    0.1%    0.1%    0.1%    0.1%    0.0%    0.0%    0.1%    0.3%    0.2%    0.0%    0.1%    0.1%    0.2%    0.6%    0.1%    0.3%    0.1%    0.2%    0.1%    0.0%    0.0%    0.0%    0.1%    0.0%    0.2%    0.1%    0.0%    0.0%    0.0%    0.1%    0.1%    0.0%    0.0%    0.2%    0.0%    0.1%    0.0%    0.0%    0.1%    0.0%    0.1%    0.1%    1.5%    0.2%    0.0%    0.0%    0.0%    0.0%    0.0%    0.0%    0.1%    0.0%    0.5%    0.2%    0.2%    0.1%    0.1%    0.1%    0.0%    0.0%    0.0%    0.0%    0.2%    1.7%    0.2%    0.1%    0.1%    0.2%    0.0%    0.0%    0.1%    0.2%    0.1%    0.0%    0.0%    0.1%    0.0%    0.1%    0.0%    0.1%    0.0%    0.1%    0.0%    0.0%    0.0%    0.0%    0.0%    0.0%    0.1%    0.1%    0.0%    0.0%    0.1%    0.1%    0.2%    0.0%    0.0%    0.0%    0.0%    0.0%    0.2%    0.0%    0.0%        k__Bacteria; p__Synergistetes     111    0.0%    0.0%    0.0%    0.0%    0.0%    0.0%    0.0%    0.0%    0.0%    0.0%    0.0%    0.1%    0.0%    0.0%    0.1%    0.0%    0.0%    0.0%    0.0%    0.0%    0.0%    0.0%    0.0%    0.0%    0.1%    0.0%    0.0%    0.0%    0.0%    0.0%    0.0%    0.0%    0.0%    0.0%    0.0%    0.0%    0.0%    0.0%    0.0%    0.0%    0.0%    0.0%    0.0%    0.0%    0.0%    0.0%    0.0%    0.0%    0.0%    0.0%    0.0%    0.0%    0.0%    0.0%    0.0%    0.0%    0.0%    0.0%    0.0%    0.0%    0.0%    0.0%    0.0%    0.0%    0.0%    0.0%    0.0%    0.0%    0.0%    0.0%    0.0%    0.0%    0.0%    0.1%    0.0%    0.0%    0.0%    0.0%    0.0%    0.0%    0.0%    0.0%    0.0%    0.0%    0.0%    0.0%    0.0%    0.0%    0.0%    0.0%    0.0%    0.0%    0.0%    0.1%    0.2%    0.0%    0.0%    0.0%    0.0%    0.0%    0.0%    0.0%    0.1%    0.0%    0.0%    0.0%    0.0%    0.0%    0.0%    0.0%    0.0%    0.0%    0.0%    0.0%    0.0%    0.0%    0.0%    0.0%    0.0%    0.0%    0.0%    0.0%    0.0%    0.0%    0.0%    0.0%    0.0%    0.0%    0.0%    0.0%    0.0%    0.0%    0.0%    0.0%        k__Bacteria; p__TM6       0    0.0%    0.0%    0.0%    0.0%    0.0%    0.0%    0.0%    0.0%    0.0%    0.0%    0.0%    0.0%    0.0%    0.0%    0.0%    0.0%    0.0%    0.0%    0.0%    0.0%    0.0%    0.0%    0.0%    0.0%    0.0%    0.0%    0.0%    0.0%    0.0%    0.0%    0.0%    0.0%    0.0%    0.0%    0.0%    0.0%    0.0%    0.0%    0.0%    0.0%    0.0%    0.0%    0.0%    0.0%    0.0%    0.0%    0.0%    0.0%    0.0%    0.0%    0.0%    0.0%    0.0%    0.0%    0.0%    0.0%    0.0%    0.0%    0.0%    0.0%    0.0%    0.0%    0.0%    0.0%    0.0%    0.0%    0.0%    0.0%    0.0%    0.0%    0.0%    0.0%    0.0%    0.0%    0.0%    0.0%    0.0%    0.0%    0.0%    0.0%    0.0%    0.0%    0.0%    0.0%    0.0%    0.0%    0.0%    0.0%    0.0%    0.0%    0.0%    0.0%    0.0%    0.0%    0.0%    0.0%    0.0%    0.0%    0.0%    0.0%    0.0%    0.0%    0.0%    0.0%    0.0%    0.0%    0.0%    0.0%    0.0%    0.0%    0.0%    0.0%    0.0%    0.0%    0.0%    0.0%    0.0%    0.0%    0.0%    0.0%    0.0%    0.0%    0.0%    0.0%    0.0%    0.0%    0.0%    0.0%    0.0%    0.0%    0.0%    0.0%    0.0%    0.0%        k__Bacteria; p__TM7      31    0.0%    0.0%    0.0%    0.0%    0.0%    0.0%    0.0%    0.0%    0.0%    0.0%    0.0%    0.0%    0.0%    0.0%    0.0%    0.0%    0.0%    0.0%    0.0%    0.0%    0.0%    0.0%    0.0%    0.0%    0.0%    0.0%    0.0%    0.0%    0.0%    0.0%    0.0%    0.0%    0.0%    0.0%    0.0%    0.0%    0.0%    0.0%    0.0%    0.0%    0.0%    0.0%    0.0%    0.0%    0.0%    0.0%    0.0%    0.0%    0.0%    0.0%    0.0%    0.0%    0.0%    0.0%    0.0%    0.0%    0.0%    0.0%    0.0%    0.0%    0.0%    0.0%    0.0%    0.0%    0.0%    0.0%    0.0%    0.0%    0.0%    0.0%    0.0%    0.0%    0.0%    0.0%    0.0%    0.0%    0.0%    0.0%    0.0%    0.0%    0.0%    0.0%    0.0%    0.0%    0.0%    0.0%    0.0%    0.0%    0.0%    0.0%    0.0%    0.0%    0.0%    0.0%    0.0%    0.0%    0.0%    0.0%    0.0%    0.0%    0.0%    0.0%    0.0%    0.0%    0.0%    0.0%    0.0%    0.0%    0.0%    0.0%    0.0%    0.0%    0.0%    0.0%    0.0%    0.0%    0.0%    0.0%    0.0%    0.0%    0.0%    0.0%    0.0%    0.0%    0.0%    0.0%    0.0%    0.0%    0.0%    0.0%    0.0%    0.0%    0.0%    0.0%        k__Bacteria; p__Tenericutes    7994    0.8%    0.8%    0.8%    0.0%    0.1%    0.4%    0.2%    0.2%    0.2%    0.0%    0.4%    1.1%    0.3%    0.3%    0.5%    1.1%    0.2%    1.1%    0.8%    0.8%    0.1%    1.3%    2.2%    0.2%    0.6%    0.8%    0.3%    0.5%    2.0%    0.7%    0.2%    0.6%    0.5%    0.2%    0.6%    0.5%    0.4%    2.4%    2.0%    2.0%    0.3%    0.7%    0.8%    1.7%    1.3%    0.1%    1.4%    1.6%    1.6%    1.3%    0.6%    0.2%    0.5%    0.8%    0.6%    0.9%    0.6%    0.1%    1.3%    0.8%    1.1%    1.0%    0.2%    0.2%    3.2%    0.4%    0.6%   14.1%    0.7%    0.3%    0.1%    1.3%    1.4%    1.8%    0.7%    0.1%    0.1%    0.6%    0.5%    3.3%    1.7%    0.4%    0.1%    0.9%    0.8%    0.5%    0.6%    1.4%    0.8%    0.1%    1.0%    2.5%    0.2%    1.5%    0.9%    0.5%    2.4%    2.1%    0.8%    0.4%    0.3%    0.8%    0.4%    0.9%    0.9%    0.1%    0.4%    0.4%    0.3%    0.2%    0.4%    0.9%    0.7%    0.2%    0.3%    0.5%    0.2%    0.2%    0.3%    0.2%    0.4%    2.2%    0.2%    0.5%    0.8%    0.4%    0.5%    1.0%    1.0%    0.4%    0.5%    0.5%    0.2%    0.4%        k__Bacteria; p__Thermi    3441    0.4%    0.2%    0.1%    2.4%    0.1%    0.2%    0.0%    0.1%    0.2%    0.1%    0.5%    0.1%    0.0%    0.2%    0.3%    0.4%    0.3%    0.3%    0.3%    0.1%    0.1%    0.1%    0.0%    0.3%    0.1%    0.1%    0.1%    0.5%    0.1%    0.1%    0.5%    0.6%    0.2%    0.4%    0.6%    0.7%    0.2%    0.2%    0.2%    0.2%    0.5%    0.3%    0.1%    0.1%    0.1%    0.0%    0.2%    0.0%    0.1%    0.6%    0.1%    0.9%    0.1%    0.1%    0.1%    0.1%    0.8%    0.5%    0.4%    0.6%    0.3%    0.1%    1.4%    0.2%    0.4%    0.2%    0.1%    0.1%    0.2%    0.6%    0.3%    0.2%    0.3%    0.1%    0.1%    0.5%    2.2%    0.1%    0.3%    0.1%    0.2%    0.1%    0.3%    0.4%    0.4%    0.3%    0.6%    0.4%    0.6%    0.3%    0.7%    0.4%    0.8%    0.1%    0.2%    0.5%    0.1%    0.1%    0.1%    0.1%    0.1%    0.2%    1.2%    1.0%    0.5%    1.4%    0.4%    0.4%    0.4%    0.6%    1.5%    0.7%    0.6%    0.1%    0.3%    0.9%    0.2%    0.3%    0.2%    0.1%    0.5%    0.8%    0.3%    0.3%    0.2%    0.2%    0.5%    0.2%    0.7%    0.7%    0.4%    0.4%    0.3%    0.3%        k__Bacteria; p__Thermotogae       4    0.0%    0.0%    0.0%    0.0%    0.0%    0.0%    0.0%    0.0%    0.0%    0.0%    0.0%    0.0%    0.0%    0.0%    0.0%    0.0%    0.0%    0.0%    0.0%    0.0%    0.0%    0.0%    0.0%    0.0%    0.0%    0.0%    0.0%    0.0%    0.0%    0.0%    0.0%    0.0%    0.0%    0.0%    0.0%    0.0%    0.0%    0.0%    0.0%    0.0%    0.0%    0.0%    0.0%    0.0%    0.0%    0.0%    0.0%    0.0%    0.0%    0.0%    0.0%    0.0%    0.0%    0.0%    0.0%    0.0%    0.0%    0.0%    0.0%    0.0%    0.0%    0.0%    0.0%    0.0%    0.0%    0.0%    0.0%    0.0%    0.0%    0.0%    0.0%    0.0%    0.0%    0.0%    0.0%    0.0%    0.0%    0.0%    0.0%    0.0%    0.0%    0.0%    0.0%    0.0%    0.0%    0.0%    0.0%    0.0%    0.0%    0.0%    0.0%    0.0%    0.0%    0.0%    0.0%    0.0%    0.0%    0.0%    0.0%    0.0%    0.0%    0.0%    0.0%    0.0%    0.0%    0.0%    0.0%    0.0%    0.0%    0.0%    0.0%    0.0%    0.0%    0.0%    0.0%    0.0%    0.0%    0.0%    0.0%    0.0%    0.0%    0.0%    0.0%    0.0%    0.0%    0.0%    0.0%    0.0%    0.0%    0.0%    0.0%    0.0%    0.0%    0.0%        k__Bacteria; p__Verrucomicrobia    1291    0.1%    0.0%    0.1%    0.3%    0.1%    0.1%    0.2%    0.4%    0.1%    0.0%    0.2%    0.1%    0.0%    0.0%    0.1%    0.0%    0.1%    0.1%    0.2%    0.0%    0.1%    0.0%    0.0%    0.0%    0.0%    0.0%    0.5%    0.1%    0.1%    0.0%    0.1%    0.1%    0.1%    0.1%    4.4%    0.1%    0.0%    0.1%    0.1%    0.1%    0.0%    0.1%    0.0%    0.0%    0.0%    0.0%    0.1%    0.0%    0.1%    0.0%    0.1%    0.0%    0.1%    0.3%    0.2%    0.1%    0.0%    0.0%    0.2%    0.1%    0.1%    0.0%    0.0%    0.0%    0.1%    0.1%    0.2%    0.1%    0.1%    0.1%    0.0%    0.1%    0.2%    0.0%    0.3%    0.0%    0.0%    0.4%    0.1%    0.1%    0.1%    0.1%    0.0%    0.1%    0.2%    0.2%    0.0%    0.2%    0.2%    0.1%    0.1%    0.0%    0.0%    0.1%    0.1%    0.2%    0.1%    0.1%    0.1%    0.0%    0.0%    0.1%    0.1%    0.2%    0.1%    0.0%    0.1%    0.1%    0.8%    1.0%    0.2%    0.3%    0.2%    0.0%    0.1%    0.1%    0.1%    0.1%    0.1%    0.0%    0.1%    0.2%    0.0%    0.1%    0.1%    0.1%    0.1%    0.1%    0.1%    0.0%    0.1%    0.1%    0.0%    0.1%        k__Bacteria; p__WPS-2       9    0.0%    0.0%    0.0%    0.0%    0.0%    0.0%    0.0%    0.0%    0.0%    0.0%    0.0%    0.0%    0.0%    0.0%    0.0%    0.0%    0.0%    0.0%    0.0%    0.0%    0.0%    0.0%    0.0%    0.0%    0.0%    0.0%    0.0%    0.0%    0.0%    0.0%    0.0%    0.0%    0.0%    0.0%    0.0%    0.0%    0.0%    0.0%    0.0%    0.0%    0.0%    0.0%    0.0%    0.0%    0.0%    0.0%    0.0%    0.0%    0.0%    0.0%    0.0%    0.0%    0.0%    0.0%    0.0%    0.0%    0.0%    0.0%    0.0%    0.0%    0.0%    0.0%    0.0%    0.0%    0.0%    0.0%    0.0%    0.0%    0.0%    0.0%    0.0%    0.0%    0.0%    0.0%    0.0%    0.0%    0.0%    0.0%    0.0%    0.0%    0.0%    0.0%    0.0%    0.0%    0.0%    0.0%    0.0%    0.0%    0.0%    0.0%    0.0%    0.0%    0.0%    0.0%    0.0%    0.0%    0.0%    0.0%    0.0%    0.0%    0.0%    0.0%    0.0%    0.0%    0.0%    0.0%    0.0%    0.0%    0.0%    0.0%    0.0%    0.0%    0.0%    0.0%    0.0%    0.0%    0.0%    0.0%    0.0%    0.0%    0.0%    0.0%    0.0%    0.0%    0.0%    0.0%    0.0%    0.0%    0.0%    0.0%    0.0%    0.0%    0.0%    0.0%        k__Bacteria; p__WS3       0    0.0%    0.0%    0.0%    0.0%    0.0%    0.0%    0.0%    0.0%    0.0%    0.0%    0.0%    0.0%    0.0%    0.0%    0.0%    0.0%    0.0%    0.0%    0.0%    0.0%    0.0%    0.0%    0.0%    0.0%    0.0%    0.0%    0.0%    0.0%    0.0%    0.0%    0.0%    0.0%    0.0%    0.0%    0.0%    0.0%    0.0%    0.0%    0.0%    0.0%    0.0%    0.0%    0.0%    0.0%    0.0%    0.0%    0.0%    0.0%    0.0%    0.0%    0.0%    0.0%    0.0%    0.0%    0.0%    0.0%    0.0%    0.0%    0.0%    0.0%    0.0%    0.0%    0.0%    0.0%    0.0%    0.0%    0.0%    0.0%    0.0%    0.0%    0.0%    0.0%    0.0%    0.0%    0.0%    0.0%    0.0%    0.0%    0.0%    0.0%    0.0%    0.0%    0.0%    0.0%    0.0%    0.0%    0.0%    0.0%    0.0%    0.0%    0.0%    0.0%    0.0%    0.0%    0.0%    0.0%    0.0%    0.0%    0.0%    0.0%    0.0%    0.0%    0.0%    0.0%    0.0%    0.0%    0.0%    0.0%    0.0%    0.0%    0.0%    0.0%    0.0%    0.0%    0.0%    0.0%    0.0%    0.0%    0.0%    0.0%    0.0%    0.0%    0.0%    0.0%    0.0%    0.0%    0.0%    0.0%    0.0%    0.0%    0.0%    0.0%    0.0%    0.0%        k__Bacteria; p__ZB2       0    0.0%    0.0%    0.0%    0.0%    0.0%    0.0%    0.0%    0.0%    0.0%    0.0%    0.0%    0.0%    0.0%    0.0%    0.0%    0.0%    0.0%    0.0%    0.0%    0.0%    0.0%    0.0%    0.0%    0.0%    0.0%    0.0%    0.0%    0.0%    0.0%    0.0%    0.0%    0.0%    0.0%    0.0%    0.0%    0.0%    0.0%    0.0%    0.0%    0.0%    0.0%    0.0%    0.0%    0.0%    0.0%    0.0%    0.0%    0.0%    0.0%    0.0%    0.0%    0.0%    0.0%    0.0%    0.0%    0.0%    0.0%    0.0%    0.0%    0.0%    0.0%    0.0%    0.0%    0.0%    0.0%    0.0%    0.0%    0.0%    0.0%    0.0%    0.0%    0.0%    0.0%    0.0%    0.0%    0.0%    0.0%    0.0%    0.0%    0.0%    0.0%    0.0%    0.0%    0.0%    0.0%    0.0%    0.0%    0.0%    0.0%    0.0%    0.0%    0.0%    0.0%    0.0%    0.0%    0.0%    0.0%    0.0%    0.0%    0.0%    0.0%    0.0%    0.0%    0.0%    0.0%    0.0%    0.0%    0.0%    0.0%    0.0%    0.0%    0.0%    0.0%    0.0%    0.0%    0.0%    0.0%    0.0%    0.0%    0.0%    0.0%    0.0%    0.0%    0.0%    0.0%    0.0%    0.0%    0.0%    0.0%    0.0%    0.0%    0.0%    0.0%    0.0%    
     
  Taxonomy Summary. Current Level: Class  
     View Figure (.pdf)    View Legend (.pdf)   
   
 
     
 
 

 
 
 
 
 
 
 
 
 
 
 
 
 
 
 
 
 
 
 
 
 
 
 
 
 
 
 
 
 
 
 
 
 
 
 
 
 
 
 
 
 
 
 
 
 
 
 
 
 
 
 
 
 
 
 
 
 
 
 
 
 
 
 
 
 
 
 
 
 
 
 
 
 
 
 
 
 
 
 
 
 
 
 
 
 
 
 
 
 
 
 
 
 
 
 
 
 
 
 
 
 
 
 
 
 
 
 
 
 
 
 
 
 
 
 
 
 
 
 
 
 
 
 
 
 
 
 
 
 
 
 
 
 
 
 
 
 
 
 
 
 
 
 
 
 
 
 
 
 
 
 
 
 
 
 
 
 
 
 
 
 
 
 
 
 
 
 
 
 
 
 
 
 
 
 
 
 
 
 
 
 
 
 
 
 
 
 
 
 
 
 
 
 
 
 
 
 
 
 
 
 
 
 
 
 
 
 
 
 
 
 
 
 
 
 
 
 
 
 
 
 
 
 
 
 
 
 
 
 
 
 
 
 
 
 
 
 
 
 
 
 
 
 
 
 
 
 
 
 
 
 
 
 
 
 
 
 
 
 
 
 
 
 
 
 
 
 
 
 
 
 
 
 
 
 
 
 
 
 
 
 
 
 
 
 
 
 
 
 
 
 
 
 
 
 
 
 
 
 
 
 
 
 
 
 
 
 
 
 
 
 
 
 
 
 
 
 
 
 
 
 
 
 
 
 
 
 
 
 
 
 
 
 
 
 
 
 
 
 
 
 
 
 
 
 
 
 
 
 
 
 
 
 
 
 
 
 
 
 
 
 
 
 
 
 
 
 
 
 
 
 
 
 
 
 
 
 
 
 
 
 
 
 
 
 
 
 
 
 
 
 
 
 
 
 
 
 
 
 
 
 
 
 
 
 
 
 
 
 
 
 
 
 
 
 
 
 
 
 
 
 
 
 
 
 
 
 
 
 
 
 
 
 
 
 
 
 
 
 
 
 
 
 
 
 
 
 
 
 
 
 
 
 
 
 
 
 
 
 
 
 
 
 
 
 
 
 
 
 
 
 
 
 
 
 
 
 
 
 
 
 
 
 
 
 
 
 
 
 
 
 
 
 
 
 
 
 
 
 
 
 
 
 
 
 
 
 
 
 
 
 
 
 
 
 
 
 
 
 
 
 
 
 
 
 
 
 
 
 
 
 
 
 
 
 
 
 
 
 
 
 
 
 
 
 
 
 
 
 
 
 
 
 
 
 
 
 
 
 
 
 
 
 
 
 
 
 
 
 
 
 
 
 
 
 
 
 
 
 
 
 
 
 
 
 
 
 
 
 
 
 
 
 
 
 
 
 
 
 
 
 
 
 
 
 
 
 
 
 
 
 
 
 
 
 
 
 
 
 
 
 
 
 
 
 
 
 
 
 
 
 
 
 
 
 
 
 
 
 
 
 
 
 
 
 
 
 
 
 
 
 
 
 
 
 
 
 
 
 
 
 
 
 
 
 
 
 
 
 
 
 
 
 
 
 
 
 
 
 
 
 
 
 
 
 
 
 
 
 
 
 
 
 
 
 
 
 
 
 
 
 
 
 
 
 
 
 
 
 
 
 
 
 
 
 
 
 
 
 
 
 
 
 
 
 
 
 
 
 
 
 
 
 
 
 
 
 
 
 
 
 
 
 
 
 
 
 
 
 
 
 
 
 
 
 
 
 
 
 
 
 
 
 
 
 
 
 
 
 
 
 
 
 
 
 
 
 
 
 
 
 
 
 
 
 
 
 
 
 
 
 
 
 
 
 
 
 
 
 
 
 
 
 
 
 
 
 
 
 
 
 
 
 
 
 
 
 
 
 
 
 
 
 
 
 
 
 
 
 
 
 
 
 
 
 
 
 
 
 
 
 
 
 
 
 
 
 
 
 
 
 
 
 
 
 
 
 
 
 
 
 
 
 
 
 
 
 
 
 
 
 
 
 
 
 
 
 
 
 
 
 
 
 
 
 
 
 
 
 
 
 
 
 
 
 
 
 
 
 
 
 
 
 
 
 
 
 
 
 
 
 
 
 
 
 
 
 
 
 
 
 
 
 
 
 
 
 
 
 
 
 
 
 
 
 
 
 
 
 
 
 
 
 
 
 
 
 
 
 
 
 
 
 
 
 
 
 
 
 
 
 
 
 
 
 
 
 
 
 
 
 
 
 
 
 
 
 
 
 
 
 
 
 
 
 
 
 
 
 
 
 
 
 
 
 
 
 
 
 
 
 
 
 
 
 
 
 
 
 
 
 
 
 
 
 
 
 
 
 
 
 
 
 
 
 
 
 
 
 
 
 
 
 
 
 
 
 
 
 
 
 
 
 
 
 
 
 
 
 
 
 
 
 
 
 
 
 
 
 
 
 
 
 
 
 
 
 
 
 
 
 
 
 
 
 
 
 
 
 
 
 
 
 
 
 
 
 
 
 
 
 
 
 
 
 
 
 
 
 
 
 
 
 
 
 
 
 
 
 
 
 
 
 
 
 
 
 
 
 
 
 
 
 
 
 
 
 
 
 
 
 
 
 
 
 
 
 
 
 
 
 
 
 
 
 
 
 
 
 
 
 
 
 
 
 
 
 
 
 
 
 
 
 
 
 
 
 
 
 
 
 
 
 
 
 
 
 
 
 
 
 
 
 
 
 
 
 
 
 
 
 
 
 
 
 
 
 
 
 
 
 
 
 
 
 
 
 
 
 
 
 
 
 
 
 
 
 
 
 
 
 
 
 
 
 
 
 
 
 
 
 
 
 
 
 
 
 
 
 
 
 
 
 
 
 
 
 
 
 
 
 
 
 
 
 
 
 
 
 
 
 
 
 
 
 
 
 
 
 
 
 
 
 
 
 
 
 
 
 
 
 
 
 
 
 
 
 
 
 
 
 
 
 
 
 
 
 
 
 
 
 
 
 
 
 
 
 
 
 
 
 
 
 
 
 
 
 
 
 
 
 
 
 
 
 
 
 
 
 
 
 
 
 
 
 
 
 
 
 
 
 
 
 
 
 
 
 
 
 
 
 
 
 
 
 
 
 
 
 
 
 
 
 
 
 
 
 
 
 
 
 
 
 
 
 
 
 
 
 
 
 
 
 
 
 
 
 
 
 
 
 
 
 
 
 
 
 
 
 
 
 
 
 
 
 
 
 
 
 
 
 
 
 
 
 
 
 
 
 
 
 
 
 
 
 
 
 
 
 
 
 
 
 
 
 
 
 
 
 
 
 
 
 
 
 
 
 
 
 
 
 
 
 
 
 
 
 
 
 
 
 
 
 
 
 
 
 
 
 
 
 
 
 
 
 
 
 
 
 
 
 
 
 
 
 
 
 
 
 
 
 
 
 
 
 
 
 
 
 
 
 
 
 
 
 
 
 
 
 
 
 
 
 
 
 
 
 
 
 
 
 
 
 
 
 
 
 
 
 
 
 
 
 
 
 
 
 
 
 
 
 
 
 
 
 
 
 
 
 
 
 
 
 
 
 
 
 
 
 
 
 
 
 
 
 
 
 
 
 
 
 
 
 
 
 
 
 
 
 
 
 
 
 
 
 
 
 
 
 
 
 
 
 
 
 
 
 
 
 
 
 
 
 
 
 
 
 
 
 
 
 
 
 
 
 
 
 
 
 
 
 
 
 
 
 
 
 
 
 
 
 
 
 
 
 
 
 
 
 
 
 
 
 
 
 
 
 
 
 
 
 
 
 
 
 
 
 
 
 
 
 
 
 
 
 
 
 
 
 
 
 
 
 
 
 
 
 
 
 
 
 
 
 
 
 
 
 
 
 
 
 
 
 
 
 
 
 
 
 
 
 
 
 
 
 
 
 
 
 
 
 
 
 
 
 
 
 
 
 
 
 
 
 
 
 
 
 
 
 
 
 
 
 
 
 
 
 
 
 
 
 
 
 
 
 
 
 
 
 
 
 
 
 
 
 
 
 
 
 
 
 
 
 
 
 
 
 
 
 
 
 
 
 
 
 
 
 
 
 
 
 
 
 
 
 
 
 
 
 
 
 
 
 
 
 
 
 
 
 
 
 
 
 
 
 
 
 
 
 
 
 
 
 
 
 
 
 
 
 
 
 
 
 
 
 
 
 
 
 
 
 
 
 
 
 
 
 
 
 
 
 
 
 
 
 
 
 
 
 
 
 
 
 
 
 
 
 
 
 
 
 
 
 
 
 
 
 
 
 
 
 
 
 
 
 
 
 
 
 
 
 
 
 
 
 
 
 
 
 
 
 
 
 
 
 
 
 
 
 
 
 
 
 
 
 
 
 
 
 
 
 
 
 
 
 
 
 
 
 
 
 
 
 
 
 
 
 
 
 
 
 
 
 
 
 
 
 
 
 
 
 
 
 
 
 
 
 
 
 
 
 
 
 
 
 
 
 
 
 
 
 
 
 
 
 
 
 
 
 
 
 
 
 
 
 
 
 
 
 
 
 
 
 
 
 
 
 
 
 
 
 
 
 
 
 
 
 
 
 
 
 
 
 
 
 
 
 
 
 
 
 
 
 
 
 
 
 
 
 
 
 
 
 
 
 
 
 
 
 
 
 
 
 
 
 
 
 
 
 
 
 
 
 
 
 
 
 
 
 
 
 
 
 
 
 
 
 
 
 
 
 
 
 
 
 
 
 
 
 
 
 
 
 
 
 
 
 
 
 
 
 
 
 
 
 
 
 
 
 
 
 
 
 
 
 
 
 
 
 
 
 
 
 
 
 
 
 
 
 
 
 
 
 
 
 
 
 
 
 
 
 
 
 
 
 
 
 
 
 
 
 
 
 
 
 
 
 
 
 
 
 
 
 
 
 
 
 
 
 
 
 
 
 
 
 
 
 
 
 
 
 
 
 
 
 
 
 
 
 
 
 
 
 
 
 
 
 
 
 
 
 
 
 
 
 
 
 
 
 
 
 
 
 
 
 
 
 
 
 
 
 
 
 
 
 
 
 
 
 
 
 
 
 
 
 
 
 
 
 
 
 
 
 
 
 
 
 
 
 
 
 
 
 
 
 
 
 
 
 
 
 
 
 
 
 
 
 
 
 
 
 
 
 
 
 
 
 
 
 
 
 
 
 
 
 
 
 
 
 
 
 
 
 
 
 
 
 
 
 
 
 
 
 
 
 
 
 
 
 
 
 
 
 
 
 
 
 
 
 
 
 
 
 
 
 
 
 
 
 
 
 
 
 
 
 
 
 
 
 
 
 
 
 
 
 
 
 
 
 
 
 
 
 
 
 
 
 
 
 
 
 
 
 
 
 
 
 
 
 
 
 
 
 
 
 
 
 
 
 
 
 
 
 
 
 
 
 
 
 
 
 
 
 
 
 
 
 
 
 
 
 
 
 
 
 
 
 
 
 
 
 
 
 
 
 
 
 
 
 
 
 
 
 
 
 
 
 
 
 
 
 
 
 
 
 
 
 
 
 
 
 
 
 
 
 
 
 
 
 
 
 
 
 
 
 
 
 
 
 
 
 
 
 
 
 
 
 
 
 
 
 
 
 
 
 
 
 
 
 
 
 
 
 
 
 
 
 
 
 
 
 
 
 
 
 
 
 
 
 
 
 
 
 
 
 
 
 
 
 
 
 
 
 
 
 
 
 
 
 
 
 
 
 
 
 
 
 
 
 
 
 
 
 
 
 
 
 
 
 
 
 
 
 
 
 
 
 
 
 
 
 
 
 
 
 
 
 
 
 
 
 
 
 
 
 
 
 
 
 
 
 
 
 
 
 
 
 
 
 
 
 
 
 
 
 
 
 
 
 
 
 
 
 
 
 
 
 
 
 
 
 
 
 
 
 
 
 
 
 
 
 
 
 
 
 
 
 
 
 
 
 
 
 
 
 
 
 
 
 
 
 
 
 
 
 
 
 
 
 
 
 
 
 
 
 
 
 
 
 
 
 
 
 
 
 
 
 
 
 
 
 
 
 
 
 
 
 
 
 
 
 
 
 
 
 
 
 
 
 
 
 
 
 
 
 
 
 
 
 
 
 
 
 
 
 
 
 
 
 
 
 
 
 
 
 
 
 
 
 
 
 
 
 
 
 
 
 
 
 
 
 
 
 
 
 
 
 
 
 
 
 
 
 
 
 
 
 
 
 
 
 
 
 
 
 
 
 
 
 
 
 
 
 
 
 
 
 
 
 
 
 
 
 
 
 
 
 
 
 
 
 
 
 
 
 
 
 
 
 
 
 
 
 
 
 
 
 
 
 
 
 
 
 
 
 
 
 
 
 
 
 
 
 
 
 
 
 
 
 
 
 
 
 
 
 
 
 
 
 
 
 
 
 
 
 
 
 
 
 
 
 
 
 
 
 
 
 
 
 
 
 
 
 
 
 
 
 
 
 
 
 
 
 
 
 
 
 
 
 
 
 
 
 
 
 
 
 
 
 
 
 
 
 
 
 
 
 
 
 
 
 
 
 
 
 
 
 
 
 
 
 
 
 
 
 
 
 
 
 
 
 
 
 
 
 
 
 
 
 
 
 
 
 
 
 
 
 
 
 
 
 
 
 
 
 
 
 
 
 
 
 
 
 
 
 
 
 
 
 
 
 
 
 
 
 
 
 
 
 
 
 
 
 
 
 
 
 
 
 
 
 
 
 
 
 
 
 
 
 
 
 
 
 
 
 
 
 
 
 
 
 
 
 
 
 
 
 
 
 
 
 
 
 
 
 
 
 
 
 
 
 
 
 
 
 
 
 
 
 
 
 
 
 
 
 
 
 
 
 
 
 
 
 
 
 
 
 
 
 
 
 
 
 
 
 
 
 
 
 
 
 
 
 
 
 
 
 
 
 
 
 
 
 
 
 
 
 
 
 
 
 
 
 
 
 
 
 
 
 
 
 
 
 
 
 
 
 
 
 
 
 
 
 
 
 
 
 
 
 
 
 
 
 
 
 
 
 
 
 
 
 
 
 
 
 
 
 
 
 
 
 
 
 
 
 
 
 
 
 
 
 
 
 
 
 
 
 
 
 
 
 
 
 
 
 
 
 
 
 
 
 
 
 
 
 
 
 
 
 
 
 
 
 
 
 
 
 
 
 
 
 
 
 
 
 
 
 
 
 
 
 
 
 
 
 
 
 
 
 
 
 
 
 
 
 
 
 
 
 
 
 
 
 
 
 
 
 
 
 
 
 
 
 
 
 
 
 
 
 
 
 
 
 
 
 
 
 
 
 
 
 
 
 
 
 
 
 
 
 
 
 
 
 
 
 
 
 
 
 
 
 
 
 
 
 
 
 
 
 
 
 
 
 
 
 
 
 
 
 
 
 
 
 
 
 
 
 
 
 
 
 
 
 
 
 
 
 
 
 
 
 
 
 
 
 
 
 
 
 
 
 
 
 
 
 
 
 
 
 
 
 
 
 
 
 
 
 
 
 
 
 
 
 
 
 
 
 
 
 
 
 
 
 
 
 
 
 
 
 
 
 
 
 
 
 
 
 
 
 
 
 
 
 
 
 
 
 
 
 
 
 
 
 
 
 
 
 
 
 
 
 
 
 
 
 
 
 
 
 
 
 
 
 
 
 
 
 
 
 
 
 
 
 
 
 
 
 
 
 
 
 
 
 
 
 
 
 
 
 
 
 
 
 
 
 
 
 
 
 
 
 
 
 
 
 
 
 
 
 
 
 
 
 
 
 
 
 
 
 
 
 
 
 
 
 
 
 
 
 
 
 
 
 
 
 
 
 
 
 
 
 
 
 
 
 
 
 
 
 
 
 
 
 
 
 
 
 
 
 
 
 
 
 
 
 
 
 
 
 
 
 
 
 
 
 
 
 
 
 
 
 
 
 
 
 
 
 
 
 
 
 
 
 
 
 
 
 
 
 
 
 
 
 
 
 
 
 
 
 
 
 
 
 
 
 
 
 
 
 
 
 
 
 
 
 
 
 
 
 
 
 
 
 
 
 
 
 
 
 
 
 
 
 
 
 
 
 
 
 
 
 
 
 
 
 
 
 
 
 
 
 
 
 
 
 
 
 
 
 
 
 
 
 
 
 
 
 
 
 
 
 
 
 
 
 
 
 
 
 
 
 
 
 
 
 
 
 
 
 
 
 
 
 
 
 
 
 
 
 
 
 
 
 
 
 
 
 
 
 
 
 
 
 
 
 
 
 
 
 
 
 
 
 
 
 
 
 
 
 
 
 
 
 
 
 
 
 
 
 
 
 
 
 
 
 
 
 
 
 
 
 
 
 
 
 
 
 
 
 
 
 
 
 
 
 
 
 
 
 
 
 
 
 
 
 
 
 
 
 
 
 
 
 
 
 
 
 
 
 
 
 
 
 
 
 
 
 
 
 
 
 
 
 
 
 
 
 
 
 
 
 
 
 
 
 
 
 
 
 
 
 
 
 
 
 
 
 
 
 
 
 
 
 
 
 
 
 
 
 
 
 
 
 
 
 
 
 
 
 
 
 
 
 
 
 
 
 
 
 
 
 
 
 
 
 
 
 
 
 
 
 
 
 
 
 
 
 
 
 
 
 
 
 
 
 
 
 
 
 
 
 
 
 
 
 
 
 
 
 
 
 
 
 
 
 
 
 
 
 
 
 
 
 
 
 
 
 
 
 
 
 
 
 
 
 
 
 
 
 
 
 
 
 
 
 
 
 
 
 
 
 
 
 
 
 
 
 
 
 
 
 
 
 
 
 
 
 
 
 
 
 
 
 
 
 
 
 
 
 
 
 
 
 
 
 
 
 
 
 
 
 
 
 
 
 
 
 
 
 
 
 
 
 
 
 
 
 
 
 
 
 
 
 
 
 
 
 
 
 
 
 
 
 
 
 
 
 
 
 
 
 
 
 
 
 
 
 
 
 
 
 
 
 
 
 
 
 
 
 
 
 
 
 
 
 
 
 
 
 
 
 
 
 
 
 
 
 
 
 
 
 
 
 
 
 
 
 
 
 
 
 
 
 
 
 
 
 
 
 
 
 
 
 
 
 
 
 
 
 
 
 
 
 
 
 
 
 
 
 
 
 
 
 
 
 
 
 
 
 
 
 
 
 
 
 
 
 
 
 
 
 
 
 
 
 
 
 
 
 
 
 
 
 
 
 
 
 
 
 
 
 
 
 
 
 
 
 
 
 
 
 
 
 
 
 
 
 
 
 
 
 
 
 
 
 
 
 
 
 
 
 
 
 
 
 
 
 
 
 
 
 
 
 
 
 
 
 
 
 
 
 
 
 
 
 
 
 
 
 
 
 
 
 
 
 
 
 
 
 
 
 
 
 
 
 
 
 
 
 
 
 
 
 
 
 
 
 
 
 
 
 
 
 
 
 
 
 
 
 
 
 
 
 
 
 
 
 
 
 
 
 
 
 
 
 
 
 
 
 
 
 
 
 
 
 
 
 
 
 
 
 
 
 
 
 
 
 
 
 
 
 
 
 
 
 
 
 
 
 
 
 
 
 
 
 
 
 
 
 
 
 
 
 
 
 
 
 
 
 
 
 
 
 
 
 
 
 
 
 
 
 
 
 
 
 
 
 
 
 
 
 
 
 
 
 
 
 
 
 
 
 
 
 
 
 
 
 
 
 
 
 
 
 
 
 
 
 
 
 
 
 
 
 
 
 
 
 
 
 
 
 
 
 
 
 
 
 
 
 
 
 
 
 
 
 
 
 

 

    View Table (.txt)         Total  0  1  2  3  4  5  6  8  9  10  11  12  13  14  15  16  17  18  19  20  21  22  23  24  25  26  27  28  29  30  31  42  43  44  45  46  47  49  50  51  53  54  55  56  57  58  59  60  61  62  71  72  81  82  83  84  91  92  93  95  96  98  99  100  101  102  103  104  112  113  114  115  116  117  118  119  120  121  122  123  124  125  126  127  128  129  130  131  132  133  134  135  136  137  138  139  140  141  142  143  144  145  146  147  148  149  150  151  152  153  155  157  158  159  160  161  166  170  171  172  173  174  175  176  177  178  179  180  181  182  183  184  185    Legend  Taxonomy  count  %  %  %  %  %  %  %  %  %  %  %  %  %  %  %  %  %  %  %  %  %  %  %  %  %  %  %  %  %  %  %  %  %  %  %  %  %  %  %  %  %  %  %  %  %  %  %  %  %  %  %  %  %  %  %  %  %  %  %  %  %  %  %  %  %  %  %  %  %  %  %  %  %  %  %  %  %  %  %  %  %  %  %  %  %  %  %  %  %  %  %  %  %  %  %  %  %  %  %  %  %  %  %  %  %  %  %  %  %  %  %  %  %  %  %  %  %  %  %  %  %  %  %  %  %  %  %  %  %  %  %  %  %  %        k__Archaea;p__Crenarchaeota; c__Sd-NA       0    0.0%    0.0%    0.0%    0.0%    0.0%    0.0%    0.0%    0.0%    0.0%    0.0%    0.0%    0.0%    0.0%    0.0%    0.0%    0.0%    0.0%    0.0%    0.0%    0.0%    0.0%    0.0%    0.0%    0.0%    0.0%    0.0%    0.0%    0.0%    0.0%    0.0%    0.0%    0.0%    0.0%    0.0%    0.0%    0.0%    0.0%    0.0%    0.0%    0.0%    0.0%    0.0%    0.0%    0.0%    0.0%    0.0%    0.0%    0.0%    0.0%    0.0%    0.0%    0.0%    0.0%    0.0%    0.0%    0.0%    0.0%    0.0%    0.0%    0.0%    0.0%    0.0%    0.0%    0.0%    0.0%    0.0%    0.0%    0.0%    0.0%    0.0%    0.0%    0.0%    0.0%    0.0%    0.0%    0.0%    0.0%    0.0%    0.0%    0.0%    0.0%    0.0%    0.0%    0.0%    0.0%    0.0%    0.0%    0.0%    0.0%    0.0%    0.0%    0.0%    0.0%    0.0%    0.0%    0.0%    0.0%    0.0%    0.0%    0.0%    0.0%    0.0%    0.0%    0.0%    0.0%    0.0%    0.0%    0.0%    0.0%    0.0%    0.0%    0.0%    0.0%    0.0%    0.0%    0.0%    0.0%    0.0%    0.0%    0.0%    0.0%    0.0%    0.0%    0.0%    0.0%    0.0%    0.0%    0.0%    0.0%    0.0%    0.0%    0.0%    0.0%    0.0%        k__Archaea;p__Crenarchaeota; c__Thaumarchaeota     558    0.1%    0.0%    0.1%    0.0%    0.0%    0.1%    0.0%    0.0%    0.0%    0.0%    0.0%    0.0%    0.0%    0.0%    0.0%    0.0%    0.3%    0.0%    0.1%    0.0%    0.0%    0.0%    0.0%    0.0%    0.0%    0.0%    0.1%    0.1%    0.0%    0.0%    0.0%    0.0%    0.0%    0.0%    0.0%    0.0%    0.0%    0.1%    0.0%    0.0%    0.0%    0.0%    0.1%    0.0%    0.0%    0.0%    0.0%    0.0%    0.0%    0.0%    0.0%    0.0%    0.0%    0.2%    0.2%    0.1%    0.0%    0.0%    0.0%    0.1%    0.1%    0.1%    0.0%    0.0%    0.2%    0.0%    0.2%    0.1%    0.0%    0.1%    0.1%    0.1%    0.2%    0.1%    0.1%    0.0%    0.0%    0.4%    0.1%    0.0%    0.0%    0.0%    0.0%    0.0%    0.1%    0.1%    0.0%    0.0%    0.1%    0.0%    0.1%    0.0%    0.0%    0.1%    0.1%    0.1%    0.0%    0.1%    0.2%    0.0%    0.1%    0.1%    0.1%    0.1%    0.0%    0.0%    0.0%    0.0%    0.3%    0.0%    0.2%    0.2%    0.2%    0.0%    0.0%    0.1%    0.0%    0.0%    0.0%    0.0%    0.0%    0.0%    0.0%    0.0%    0.1%    0.2%    0.1%    0.0%    0.1%    0.1%    0.0%    0.1%    0.1%    0.1%        k__Archaea;p__Euryarchaeota; c__Halobacteria       1    0.0%    0.0%    0.0%    0.0%    0.0%    0.0%    0.0%    0.0%    0.0%    0.0%    0.0%    0.0%    0.0%    0.0%    0.0%    0.0%    0.0%    0.0%    0.0%    0.0%    0.0%    0.0%    0.0%    0.0%    0.0%    0.0%    0.0%    0.0%    0.0%    0.0%    0.0%    0.0%    0.0%    0.0%    0.0%    0.0%    0.0%    0.0%    0.0%    0.0%    0.0%    0.0%    0.0%    0.0%    0.0%    0.0%    0.0%    0.0%    0.0%    0.0%    0.0%    0.0%    0.0%    0.0%    0.0%    0.0%    0.0%    0.0%    0.0%    0.0%    0.0%    0.0%    0.0%    0.0%    0.0%    0.0%    0.0%    0.0%    0.0%    0.0%    0.0%    0.0%    0.0%    0.0%    0.0%    0.0%    0.0%    0.0%    0.0%    0.0%    0.0%    0.0%    0.0%    0.0%    0.0%    0.0%    0.0%    0.0%    0.0%    0.0%    0.0%    0.0%    0.0%    0.0%    0.0%    0.0%    0.0%    0.0%    0.0%    0.0%    0.0%    0.0%    0.0%    0.0%    0.0%    0.0%    0.0%    0.0%    0.0%    0.0%    0.0%    0.0%    0.0%    0.0%    0.0%    0.0%    0.0%    0.0%    0.0%    0.0%    0.0%    0.0%    0.0%    0.0%    0.0%    0.0%    0.0%    0.0%    0.0%    0.0%    0.0%    0.0%    0.0%    0.0%        k__Archaea;p__Euryarchaeota; c__Methanobacteria       4    0.0%    0.0%    0.0%    0.0%    0.0%    0.0%    0.0%    0.0%    0.0%    0.0%    0.0%    0.0%    0.0%    0.0%    0.0%    0.0%    0.0%    0.0%    0.0%    0.0%    0.0%    0.0%    0.0%    0.0%    0.0%    0.0%    0.0%    0.0%    0.0%    0.0%    0.0%    0.0%    0.0%    0.0%    0.0%    0.0%    0.0%    0.0%    0.0%    0.0%    0.0%    0.0%    0.0%    0.0%    0.0%    0.0%    0.0%    0.0%    0.0%    0.0%    0.0%    0.0%    0.0%    0.0%    0.0%    0.0%    0.0%    0.0%    0.0%    0.0%    0.0%    0.0%    0.0%    0.0%    0.0%    0.0%    0.0%    0.0%    0.0%    0.0%    0.0%    0.0%    0.0%    0.0%    0.0%    0.0%    0.0%    0.0%    0.0%    0.0%    0.0%    0.0%    0.0%    0.0%    0.0%    0.0%    0.0%    0.0%    0.0%    0.0%    0.0%    0.0%    0.0%    0.0%    0.0%    0.0%    0.0%    0.0%    0.0%    0.0%    0.0%    0.0%    0.0%    0.0%    0.0%    0.0%    0.0%    0.0%    0.0%    0.0%    0.0%    0.0%    0.0%    0.0%    0.0%    0.0%    0.0%    0.0%    0.0%    0.0%    0.0%    0.0%    0.0%    0.0%    0.0%    0.0%    0.0%    0.0%    0.0%    0.0%    0.0%    0.0%    0.0%    0.0%        k__Archaea;p__Euryarchaeota; c__Methanomicrobia       5    0.0%    0.0%    0.0%    0.0%    0.0%    0.0%    0.0%    0.0%    0.0%    0.0%    0.0%    0.0%    0.0%    0.0%    0.0%    0.0%    0.0%    0.0%    0.0%    0.0%    0.0%    0.0%    0.0%    0.0%    0.0%    0.0%    0.0%    0.0%    0.0%    0.0%    0.0%    0.1%    0.0%    0.0%    0.0%    0.0%    0.0%    0.0%    0.0%    0.0%    0.0%    0.0%    0.0%    0.0%    0.0%    0.0%    0.0%    0.0%    0.0%    0.0%    0.0%    0.0%    0.0%    0.0%    0.0%    0.0%    0.0%    0.0%    0.0%    0.0%    0.0%    0.0%    0.0%    0.0%    0.0%    0.0%    0.0%    0.0%    0.0%    0.0%    0.0%    0.0%    0.0%    0.0%    0.0%    0.0%    0.0%    0.0%    0.0%    0.0%    0.0%    0.0%    0.0%    0.0%    0.0%    0.0%    0.0%    0.0%    0.0%    0.0%    0.0%    0.0%    0.0%    0.0%    0.0%    0.0%    0.0%    0.0%    0.0%    0.0%    0.0%    0.0%    0.0%    0.0%    0.0%    0.0%    0.0%    0.0%    0.0%    0.0%    0.0%    0.0%    0.0%    0.0%    0.0%    0.0%    0.0%    0.0%    0.0%    0.0%    0.0%    0.0%    0.0%    0.0%    0.0%    0.0%    0.0%    0.0%    0.0%    0.0%    0.0%    0.0%    0.0%    0.0%        k__Archaea;p__Euryarchaeota; c__Thermoplasmata       0    0.0%    0.0%    0.0%    0.0%    0.0%    0.0%    0.0%    0.0%    0.0%    0.0%    0.0%    0.0%    0.0%    0.0%    0.0%    0.0%    0.0%    0.0%    0.0%    0.0%    0.0%    0.0%    0.0%    0.0%    0.0%    0.0%    0.0%    0.0%    0.0%    0.0%    0.0%    0.0%    0.0%    0.0%    0.0%    0.0%    0.0%    0.0%    0.0%    0.0%    0.0%    0.0%    0.0%    0.0%    0.0%    0.0%    0.0%    0.0%    0.0%    0.0%    0.0%    0.0%    0.0%    0.0%    0.0%    0.0%    0.0%    0.0%    0.0%    0.0%    0.0%    0.0%    0.0%    0.0%    0.0%    0.0%    0.0%    0.0%    0.0%    0.0%    0.0%    0.0%    0.0%    0.0%    0.0%    0.0%    0.0%    0.0%    0.0%    0.0%    0.0%    0.0%    0.0%    0.0%    0.0%    0.0%    0.0%    0.0%    0.0%    0.0%    0.0%    0.0%    0.0%    0.0%    0.0%    0.0%    0.0%    0.0%    0.0%    0.0%    0.0%    0.0%    0.0%    0.0%    0.0%    0.0%    0.0%    0.0%    0.0%    0.0%    0.0%    0.0%    0.0%    0.0%    0.0%    0.0%    0.0%    0.0%    0.0%    0.0%    0.0%    0.0%    0.0%    0.0%    0.0%    0.0%    0.0%    0.0%    0.0%    0.0%    0.0%    0.0%    0.0%    0.0%        k__Bacteria;p__; c__       0    0.0%    0.0%    0.0%    0.0%    0.0%    0.0%    0.0%    0.0%    0.0%    0.0%    0.0%    0.0%    0.0%    0.0%    0.0%    0.0%    0.0%    0.0%    0.0%    0.0%    0.0%    0.0%    0.0%    0.0%    0.0%    0.0%    0.0%    0.0%    0.0%    0.0%    0.0%    0.0%    0.0%    0.0%    0.0%    0.0%    0.0%    0.0%    0.0%    0.0%    0.0%    0.0%    0.0%    0.0%    0.0%    0.0%    0.0%    0.0%    0.0%    0.0%    0.0%    0.0%    0.0%    0.0%    0.0%    0.0%    0.0%    0.0%    0.0%    0.0%    0.0%    0.0%    0.0%    0.0%    0.0%    0.0%    0.0%    0.0%    0.0%    0.0%    0.0%    0.0%    0.0%    0.0%    0.0%    0.0%    0.0%    0.0%    0.0%    0.0%    0.0%    0.0%    0.0%    0.0%    0.0%    0.0%    0.0%    0.0%    0.0%    0.0%    0.0%    0.0%    0.0%    0.0%    0.0%    0.0%    0.0%    0.0%    0.0%    0.0%    0.0%    0.0%    0.0%    0.0%    0.0%    0.0%    0.0%    0.0%    0.0%    0.0%    0.0%    0.0%    0.0%    0.0%    0.0%    0.0%    0.0%    0.0%    0.0%    0.0%    0.0%    0.0%    0.0%    0.0%    0.0%    0.0%    0.0%    0.0%    0.0%    0.0%    0.0%    0.0%    0.0%    0.0%        k__Bacteria;p__ABY1_OD1; c__       0    0.0%    0.0%    0.0%    0.0%    0.0%    0.0%    0.0%    0.0%    0.0%    0.0%    0.0%    0.0%    0.0%    0.0%    0.0%    0.0%    0.0%    0.0%    0.0%    0.0%    0.0%    0.0%    0.0%    0.0%    0.0%    0.0%    0.0%    0.0%    0.0%    0.0%    0.0%    0.0%    0.0%    0.0%    0.0%    0.0%    0.0%    0.0%    0.0%    0.0%    0.0%    0.0%    0.0%    0.0%    0.0%    0.0%    0.0%    0.0%    0.0%    0.0%    0.0%    0.0%    0.0%    0.0%    0.0%    0.0%    0.0%    0.0%    0.0%    0.0%    0.0%    0.0%    0.0%    0.0%    0.0%    0.0%    0.0%    0.0%    0.0%    0.0%    0.0%    0.0%    0.0%    0.0%    0.0%    0.0%    0.0%    0.0%    0.0%    0.0%    0.0%    0.0%    0.0%    0.0%    0.0%    0.0%    0.0%    0.0%    0.0%    0.0%    0.0%    0.0%    0.0%    0.0%    0.0%    0.0%    0.0%    0.0%    0.0%    0.0%    0.0%    0.0%    0.0%    0.0%    0.0%    0.0%    0.0%    0.0%    0.0%    0.0%    0.0%    0.0%    0.0%    0.0%    0.0%    0.0%    0.0%    0.0%    0.0%    0.0%    0.0%    0.0%    0.0%    0.0%    0.0%    0.0%    0.0%    0.0%    0.0%    0.0%    0.0%    0.0%    0.0%    0.0%        k__Bacteria;p__AD3; c__ABS-6       0    0.0%    0.0%    0.0%    0.0%    0.0%    0.0%    0.0%    0.0%    0.0%    0.0%    0.0%    0.0%    0.0%    0.0%    0.0%    0.0%    0.0%    0.0%    0.0%    0.0%    0.0%    0.0%    0.0%    0.0%    0.0%    0.0%    0.0%    0.0%    0.0%    0.0%    0.0%    0.0%    0.0%    0.0%    0.0%    0.0%    0.0%    0.0%    0.0%    0.0%    0.0%    0.0%    0.0%    0.0%    0.0%    0.0%    0.0%    0.0%    0.0%    0.0%    0.0%    0.0%    0.0%    0.0%    0.0%    0.0%    0.0%    0.0%    0.0%    0.0%    0.0%    0.0%    0.0%    0.0%    0.0%    0.0%    0.0%    0.0%    0.0%    0.0%    0.0%    0.0%    0.0%    0.0%    0.0%    0.0%    0.0%    0.0%    0.0%    0.0%    0.0%    0.0%    0.0%    0.0%    0.0%    0.0%    0.0%    0.0%    0.0%    0.0%    0.0%    0.0%    0.0%    0.0%    0.0%    0.0%    0.0%    0.0%    0.0%    0.0%    0.0%    0.0%    0.0%    0.0%    0.0%    0.0%    0.0%    0.0%    0.0%    0.0%    0.0%    0.0%    0.0%    0.0%    0.0%    0.0%    0.0%    0.0%    0.0%    0.0%    0.0%    0.0%    0.0%    0.0%    0.0%    0.0%    0.0%    0.0%    0.0%    0.0%    0.0%    0.0%    0.0%    0.0%        k__Bacteria;p__AD3; c__JG37-AG-4       0    0.0%    0.0%    0.0%    0.0%    0.0%    0.0%    0.0%    0.0%    0.0%    0.0%    0.0%    0.0%    0.0%    0.0%    0.0%    0.0%    0.0%    0.0%    0.0%    0.0%    0.0%    0.0%    0.0%    0.0%    0.0%    0.0%    0.0%    0.0%    0.0%    0.0%    0.0%    0.0%    0.0%    0.0%    0.0%    0.0%    0.0%    0.0%    0.0%    0.0%    0.0%    0.0%    0.0%    0.0%    0.0%    0.0%    0.0%    0.0%    0.0%    0.0%    0.0%    0.0%    0.0%    0.0%    0.0%    0.0%    0.0%    0.0%    0.0%    0.0%    0.0%    0.0%    0.0%    0.0%    0.0%    0.0%    0.0%    0.0%    0.0%    0.0%    0.0%    0.0%    0.0%    0.0%    0.0%    0.0%    0.0%    0.0%    0.0%    0.0%    0.0%    0.0%    0.0%    0.0%    0.0%    0.0%    0.0%    0.0%    0.0%    0.0%    0.0%    0.0%    0.0%    0.0%    0.0%    0.0%    0.0%    0.0%    0.0%    0.0%    0.0%    0.0%    0.0%    0.0%    0.0%    0.0%    0.0%    0.0%    0.0%    0.0%    0.0%    0.0%    0.0%    0.0%    0.0%    0.0%    0.0%    0.0%    0.0%    0.0%    0.0%    0.0%    0.0%    0.0%    0.0%    0.0%    0.0%    0.0%    0.0%    0.0%    0.0%    0.0%    0.0%    0.0%        k__Bacteria;p__Acidobacteria; c__      40    0.0%    0.0%    0.0%    0.0%    0.0%    0.0%    0.0%    0.0%    0.0%    0.0%    0.0%    0.0%    0.0%    0.0%    0.0%    0.0%    0.0%    0.0%    0.0%    0.0%    0.0%    0.0%    0.0%    0.0%    0.0%    0.0%    0.0%    0.0%    0.0%    0.0%    0.0%    0.0%    0.0%    0.0%    0.0%    0.0%    0.0%    0.0%    0.0%    0.0%    0.0%    0.0%    0.0%    0.0%    0.0%    0.0%    0.0%    0.0%    0.0%    0.0%    0.0%    0.0%    0.0%    0.0%    0.0%    0.0%    0.0%    0.0%    0.0%    0.0%    0.0%    0.0%    0.0%    0.0%    0.0%    0.0%    0.0%    0.0%    0.0%    0.0%    0.0%    0.0%    0.0%    0.0%    0.0%    0.0%    0.0%    0.0%    0.0%    0.0%    0.0%    0.0%    0.0%    0.0%    0.0%    0.0%    0.0%    0.0%    0.0%    0.0%    0.0%    0.0%    0.0%    0.0%    0.0%    0.0%    0.0%    0.0%    0.0%    0.0%    0.0%    0.0%    0.0%    0.0%    0.0%    0.0%    0.0%    0.0%    0.1%    0.0%    0.0%    0.0%    0.0%    0.0%    0.0%    0.0%    0.0%    0.0%    0.0%    0.0%    0.0%    0.0%    0.0%    0.0%    0.0%    0.0%    0.0%    0.0%    0.0%    0.0%    0.0%    0.0%    0.0%    0.0%        k__Bacteria;p__Acidobacteria; c__Acidobacteria (class)    2129    0.2%    0.2%    0.1%    0.2%    0.0%    0.6%    0.2%    0.1%    0.2%    0.0%    0.2%    0.1%    0.0%    0.1%    0.1%    0.0%    0.2%    0.1%    0.3%    0.1%    0.2%    0.1%    0.1%    0.0%    0.1%    0.0%    0.5%    0.2%    0.1%    0.0%    0.1%    0.0%    0.1%    0.2%    0.0%    0.2%    0.0%    0.4%    0.1%    0.1%    0.1%    0.6%    0.2%    0.1%    0.2%    0.0%    0.1%    0.1%    0.1%    0.1%    0.0%    0.1%    0.1%    0.5%    0.5%    0.2%    0.3%    0.0%    0.3%    0.3%    0.7%    0.1%    0.1%    0.0%    0.4%    0.1%    0.8%    0.1%    0.2%    0.6%    0.1%    0.2%    0.7%    0.2%    0.8%    0.0%    0.0%    1.2%    0.4%    0.1%    0.3%    0.2%    0.1%    0.3%    0.4%    0.4%    0.2%    0.3%    0.3%    0.1%    0.2%    0.2%    0.0%    0.2%    0.3%    0.3%    0.1%    0.5%    0.5%    0.2%    0.1%    0.3%    0.1%    0.3%    0.1%    0.0%    0.3%    0.2%    0.6%    0.3%    0.6%    0.5%    0.3%    0.1%    0.2%    0.4%    0.1%    0.1%    0.1%    0.1%    0.3%    0.4%    0.0%    0.2%    0.2%    0.5%    0.2%    0.3%    0.3%    0.3%    0.1%    0.1%    0.2%    0.2%        k__Bacteria;p__Acidobacteria; c__Acidobacteria-5      37    0.0%    0.0%    0.0%    0.0%    0.0%    0.0%    0.0%    0.0%    0.0%    0.0%    0.0%    0.0%    0.0%    0.0%    0.0%    0.0%    0.0%    0.0%    0.0%    0.0%    0.0%    0.0%    0.0%    0.0%    0.0%    0.0%    0.0%    0.0%    0.0%    0.0%    0.0%    0.0%    0.0%    0.0%    0.0%    0.0%    0.0%    0.0%    0.0%    0.0%    0.0%    0.0%    0.0%    0.0%    0.0%    0.0%    0.0%    0.0%    0.0%    0.0%    0.0%    0.0%    0.0%    0.0%    0.0%    0.0%    0.0%    0.0%    0.0%    0.0%    0.0%    0.0%    0.0%    0.0%    0.0%    0.0%    0.0%    0.0%    0.0%    0.0%    0.0%    0.0%    0.0%    0.0%    0.0%    0.0%    0.0%    0.0%    0.0%    0.0%    0.0%    0.0%    0.0%    0.0%    0.0%    0.0%    0.0%    0.0%    0.0%    0.0%    0.0%    0.0%    0.0%    0.0%    0.0%    0.0%    0.0%    0.0%    0.0%    0.0%    0.0%    0.0%    0.0%    0.0%    0.0%    0.0%    0.0%    0.0%    0.0%    0.0%    0.0%    0.0%    0.0%    0.0%    0.0%    0.0%    0.0%    0.0%    0.0%    0.0%    0.0%    0.0%    0.0%    0.0%    0.0%    0.0%    0.0%    0.0%    0.0%    0.0%    0.0%    0.0%    0.0%    0.0%        k__Bacteria;p__Acidobacteria; c__Chloracidobacteria    1070    0.1%    0.0%    0.0%    0.0%    0.1%    0.2%    0.1%    0.1%    0.1%    0.0%    0.2%    0.0%    0.0%    0.1%    0.1%    0.0%    0.1%    0.0%    0.1%    0.0%    0.1%    0.0%    0.0%    0.0%    0.2%    0.1%    0.1%    0.1%    0.0%    0.0%    0.1%    0.0%    0.1%    0.0%    0.0%    0.1%    0.0%    0.0%    0.1%    0.1%    0.0%    0.0%    5.0%    0.0%    0.0%    0.0%    0.1%    0.0%    0.1%    0.0%    0.0%    0.0%    0.0%    0.2%    0.1%    0.1%    0.0%    0.0%    0.1%    0.2%    0.1%    0.0%    0.0%    0.0%    0.1%    0.0%    0.3%    0.0%    0.1%    0.3%    0.0%    0.0%    0.1%    0.0%    0.1%    0.0%    0.0%    0.2%    0.1%    0.0%    0.0%    0.0%    0.0%    0.2%    0.2%    0.1%    0.1%    0.2%    0.1%    0.1%    0.1%    0.1%    0.0%    0.1%    0.1%    0.1%    0.1%    0.1%    0.1%    0.1%    0.1%    0.1%    0.0%    0.2%    0.0%    0.1%    0.0%    0.0%    0.2%    0.3%    0.2%    0.1%    0.1%    0.0%    0.0%    0.1%    0.0%    0.0%    0.1%    0.0%    0.0%    0.2%    0.0%    0.0%    0.1%    0.1%    0.0%    0.0%    0.0%    0.2%    0.0%    0.0%    0.1%    0.0%        k__Bacteria;p__Acidobacteria; c__Holophagae       2    0.0%    0.0%    0.0%    0.0%    0.0%    0.0%    0.0%    0.0%    0.0%    0.0%    0.0%    0.0%    0.0%    0.0%    0.0%    0.0%    0.0%    0.0%    0.0%    0.0%    0.0%    0.0%    0.0%    0.0%    0.0%    0.0%    0.0%    0.0%    0.0%    0.0%    0.0%    0.0%    0.0%    0.0%    0.0%    0.0%    0.0%    0.0%    0.0%    0.0%    0.0%    0.0%    0.0%    0.0%    0.0%    0.0%    0.0%    0.0%    0.0%    0.0%    0.0%    0.0%    0.0%    0.0%    0.0%    0.0%    0.0%    0.0%    0.0%    0.0%    0.0%    0.0%    0.0%    0.0%    0.0%    0.0%    0.0%    0.0%    0.0%    0.0%    0.0%    0.0%    0.0%    0.0%    0.0%    0.0%    0.0%    0.0%    0.0%    0.0%    0.0%    0.0%    0.0%    0.0%    0.0%    0.0%    0.0%    0.0%    0.0%    0.0%    0.0%    0.0%    0.0%    0.0%    0.0%    0.0%    0.0%    0.0%    0.0%    0.0%    0.0%    0.0%    0.0%    0.0%    0.0%    0.0%    0.0%    0.0%    0.0%    0.0%    0.0%    0.0%    0.0%    0.0%    0.0%    0.0%    0.0%    0.0%    0.0%    0.0%    0.0%    0.0%    0.0%    0.0%    0.0%    0.0%    0.0%    0.0%    0.0%    0.0%    0.0%    0.0%    0.0%    0.0%        k__Bacteria;p__Acidobacteria; c__MVS-40       8    0.0%    0.0%    0.0%    0.0%    0.0%    0.0%    0.0%    0.0%    0.0%    0.0%    0.0%    0.0%    0.0%    0.0%    0.0%    0.0%    0.0%    0.0%    0.0%    0.0%    0.0%    0.0%    0.0%    0.0%    0.0%    0.0%    0.0%    0.0%    0.0%    0.0%    0.0%    0.0%    0.0%    0.0%    0.0%    0.0%    0.0%    0.0%    0.0%    0.0%    0.0%    0.0%    0.0%    0.0%    0.0%    0.0%    0.0%    0.0%    0.0%    0.0%    0.0%    0.0%    0.0%    0.0%    0.0%    0.0%    0.0%    0.0%    0.0%    0.0%    0.0%    0.0%    0.0%    0.0%    0.0%    0.0%    0.0%    0.0%    0.0%    0.0%    0.0%    0.0%    0.0%    0.0%    0.0%    0.0%    0.0%    0.0%    0.0%    0.0%    0.0%    0.0%    0.0%    0.0%    0.0%    0.0%    0.0%    0.0%    0.0%    0.0%    0.0%    0.0%    0.0%    0.0%    0.0%    0.0%    0.0%    0.0%    0.0%    0.0%    0.0%    0.0%    0.0%    0.0%    0.0%    0.0%    0.0%    0.0%    0.0%    0.0%    0.0%    0.0%    0.0%    0.0%    0.0%    0.0%    0.0%    0.0%    0.0%    0.0%    0.0%    0.0%    0.0%    0.0%    0.0%    0.0%    0.0%    0.0%    0.0%    0.0%    0.0%    0.0%    0.0%    0.0%        k__Bacteria;p__Acidobacteria; c__OS-K       0    0.0%    0.0%    0.0%    0.0%    0.0%    0.0%    0.0%    0.0%    0.0%    0.0%    0.0%    0.0%    0.0%    0.0%    0.0%    0.0%    0.0%    0.0%    0.0%    0.0%    0.0%    0.0%    0.0%    0.0%    0.0%    0.0%    0.0%    0.0%    0.0%    0.0%    0.0%    0.0%    0.0%    0.0%    0.0%    0.0%    0.0%    0.0%    0.0%    0.0%    0.0%    0.0%    0.0%    0.0%    0.0%    0.0%    0.0%    0.0%    0.0%    0.0%    0.0%    0.0%    0.0%    0.0%    0.0%    0.0%    0.0%    0.0%    0.0%    0.0%    0.0%    0.0%    0.0%    0.0%    0.0%    0.0%    0.0%    0.0%    0.0%    0.0%    0.0%    0.0%    0.0%    0.0%    0.0%    0.0%    0.0%    0.0%    0.0%    0.0%    0.0%    0.0%    0.0%    0.0%    0.0%    0.0%    0.0%    0.0%    0.0%    0.0%    0.0%    0.0%    0.0%    0.0%    0.0%    0.0%    0.0%    0.0%    0.0%    0.0%    0.0%    0.0%    0.0%    0.0%    0.0%    0.0%    0.0%    0.0%    0.0%    0.0%    0.0%    0.0%    0.0%    0.0%    0.0%    0.0%    0.0%    0.0%    0.0%    0.0%    0.0%    0.0%    0.0%    0.0%    0.0%    0.0%    0.0%    0.0%    0.0%    0.0%    0.0%    0.0%    0.0%    0.0%        k__Bacteria;p__Acidobacteria; c__PAUC37f       4    0.0%    0.0%    0.0%    0.0%    0.0%    0.0%    0.0%    0.0%    0.0%    0.0%    0.0%    0.0%    0.0%    0.0%    0.0%    0.0%    0.0%    0.0%    0.0%    0.0%    0.0%    0.0%    0.0%    0.0%    0.0%    0.0%    0.0%    0.0%    0.0%    0.0%    0.0%    0.0%    0.0%    0.0%    0.0%    0.0%    0.0%    0.0%    0.0%    0.0%    0.0%    0.0%    0.0%    0.0%    0.0%    0.0%    0.0%    0.0%    0.0%    0.0%    0.0%    0.0%    0.0%    0.0%    0.0%    0.0%    0.0%    0.0%    0.0%    0.0%    0.0%    0.0%    0.0%    0.0%    0.0%    0.0%    0.0%    0.0%    0.0%    0.0%    0.0%    0.0%    0.0%    0.0%    0.0%    0.0%    0.0%    0.0%    0.0%    0.0%    0.0%    0.0%    0.0%    0.0%    0.0%    0.0%    0.0%    0.0%    0.0%    0.0%    0.0%    0.0%    0.0%    0.0%    0.0%    0.0%    0.0%    0.0%    0.0%    0.0%    0.0%    0.0%    0.0%    0.0%    0.0%    0.0%    0.0%    0.0%    0.0%    0.0%    0.0%    0.0%    0.0%    0.0%    0.0%    0.0%    0.0%    0.0%    0.0%    0.0%    0.0%    0.0%    0.0%    0.0%    0.0%    0.0%    0.0%    0.0%    0.0%    0.0%    0.0%    0.0%    0.0%    0.0%        k__Bacteria;p__Acidobacteria; c__RB25       5    0.0%    0.0%    0.0%    0.0%    0.0%    0.0%    0.0%    0.0%    0.0%    0.0%    0.0%    0.0%    0.0%    0.0%    0.0%    0.0%    0.0%    0.0%    0.0%    0.0%    0.0%    0.0%    0.0%    0.0%    0.0%    0.0%    0.0%    0.0%    0.0%    0.0%    0.0%    0.0%    0.0%    0.0%    0.0%    0.0%    0.0%    0.0%    0.0%    0.0%    0.0%    0.0%    0.0%    0.0%    0.0%    0.0%    0.0%    0.0%    0.0%    0.0%    0.0%    0.0%    0.0%    0.0%    0.0%    0.0%    0.0%    0.0%    0.0%    0.0%    0.0%    0.0%    0.0%    0.0%    0.0%    0.0%    0.0%    0.0%    0.0%    0.0%    0.0%    0.0%    0.0%    0.0%    0.0%    0.0%    0.0%    0.0%    0.0%    0.0%    0.0%    0.0%    0.0%    0.0%    0.0%    0.0%    0.0%    0.0%    0.0%    0.0%    0.0%    0.0%    0.0%    0.0%    0.0%    0.0%    0.0%    0.0%    0.0%    0.0%    0.0%    0.0%    0.0%    0.0%    0.0%    0.0%    0.0%    0.0%    0.0%    0.0%    0.0%    0.0%    0.0%    0.0%    0.0%    0.0%    0.0%    0.0%    0.0%    0.0%    0.0%    0.0%    0.0%    0.0%    0.0%    0.0%    0.0%    0.0%    0.0%    0.0%    0.0%    0.0%    0.0%    0.0%        k__Bacteria;p__Acidobacteria; c__Solibacteres     287    0.0%    0.0%    0.0%    0.0%    0.0%    0.0%    0.1%    0.1%    0.0%    0.0%    0.1%    0.0%    0.0%    0.0%    0.0%    0.0%    0.0%    0.0%    0.0%    0.0%    0.0%    0.0%    0.0%    0.0%    0.1%    0.0%    0.1%    0.1%    0.0%    0.0%    0.0%    0.0%    0.0%    0.0%    0.0%    0.0%    0.0%    0.0%    0.0%    0.0%    0.0%    0.0%    0.0%    0.0%    0.0%    0.0%    0.0%    0.0%    0.0%    0.0%    0.0%    0.0%    0.0%    0.0%    0.0%    0.0%    0.0%    0.0%    0.1%    0.0%    0.0%    0.0%    0.0%    0.0%    0.0%    0.0%    0.2%    0.0%    0.1%    0.0%    0.1%    0.0%    0.1%    0.0%    0.0%    0.0%    0.0%    0.2%    0.1%    0.0%    0.0%    0.0%    0.0%    0.1%    0.1%    0.1%    0.0%    0.1%    0.0%    0.0%    0.0%    0.0%    0.0%    0.0%    0.0%    0.1%    0.0%    0.1%    0.1%    0.1%    0.0%    0.0%    0.0%    0.1%    0.0%    0.0%    0.0%    0.0%    0.1%    0.0%    0.1%    0.1%    0.0%    0.0%    0.0%    0.0%    0.0%    0.0%    0.0%    0.0%    0.0%    0.0%    0.0%    0.1%    0.0%    0.1%    0.0%    0.0%    0.0%    0.0%    0.0%    0.0%    0.0%    0.0%        k__Bacteria;p__Acidobacteria; c__Sva0725      42    0.0%    0.0%    0.0%    0.0%    0.0%    0.0%    0.0%    0.0%    0.0%    0.0%    0.0%    0.0%    0.0%    0.0%    0.0%    0.0%    0.0%    0.0%    0.0%    0.0%    0.0%    0.0%    0.0%    0.0%    0.0%    0.0%    0.0%    0.0%    0.0%    0.0%    0.0%    0.0%    0.0%    0.0%    0.0%    0.0%    0.0%    0.0%    0.0%    0.0%    0.0%    0.0%    0.0%    0.0%    0.0%    0.0%    0.0%    0.0%    0.0%    0.0%    0.0%    0.0%    0.0%    0.0%    0.0%    0.0%    0.0%    0.0%    0.0%    0.0%    0.0%    0.0%    0.0%    0.0%    0.0%    0.0%    0.0%    0.0%    0.0%    0.0%    0.0%    0.0%    0.0%    0.0%    0.0%    0.0%    0.0%    0.0%    0.0%    0.0%    0.0%    0.0%    0.0%    0.0%    0.0%    0.0%    0.0%    0.0%    0.0%    0.0%    0.0%    0.0%    0.0%    0.0%    0.0%    0.0%    0.0%    0.0%    0.0%    0.0%    0.0%    0.0%    0.0%    0.0%    0.0%    0.0%    0.0%    0.0%    0.0%    0.0%    0.0%    0.0%    0.0%    0.0%    0.0%    0.0%    0.0%    0.0%    0.0%    0.0%    0.0%    0.0%    0.0%    0.0%    0.0%    0.0%    0.0%    0.0%    0.0%    0.0%    0.0%    0.0%    0.0%    0.0%        k__Bacteria;p__Acidobacteria; c__iii1-8      48    0.0%    0.0%    0.0%    0.0%    0.0%    0.0%    0.0%    0.0%    0.0%    0.0%    0.0%    0.0%    0.0%    0.0%    0.0%    0.0%    0.0%    0.0%    0.0%    0.0%    0.0%    0.0%    0.0%    0.0%    0.0%    0.0%    0.0%    0.0%    0.0%    0.0%    0.0%    0.0%    0.0%    0.0%    0.0%    0.0%    0.0%    0.0%    0.0%    0.0%    0.0%    0.0%    0.0%    0.0%    0.0%    0.0%    0.0%    0.0%    0.0%    0.0%    0.0%    0.0%    0.0%    0.0%    0.0%    0.0%    0.0%    0.0%    0.0%    0.0%    0.0%    0.0%    0.0%    0.0%    0.0%    0.0%    0.0%    0.0%    0.0%    0.0%    0.0%    0.0%    0.0%    0.0%    0.0%    0.0%    0.0%    0.0%    0.0%    0.0%    0.0%    0.0%    0.0%    0.0%    0.0%    0.0%    0.0%    0.0%    0.0%    0.0%    0.0%    0.0%    0.0%    0.0%    0.0%    0.0%    0.0%    0.0%    0.0%    0.0%    0.0%    0.0%    0.0%    0.0%    0.0%    0.0%    0.0%    0.0%    0.0%    0.0%    0.0%    0.0%    0.0%    0.0%    0.0%    0.0%    0.0%    0.0%    0.0%    0.0%    0.0%    0.0%    0.0%    0.0%    0.0%    0.0%    0.0%    0.0%    0.0%    0.1%    0.0%    0.0%    0.0%    0.0%        k__Bacteria;p__Actinobacteria; c__     205    0.0%    0.0%    0.0%    0.1%    0.0%    0.1%    0.1%    0.1%    0.0%    0.1%    0.0%    0.0%    0.0%    0.0%    0.0%    0.0%    0.0%    0.0%    0.0%    0.0%    0.0%    0.0%    0.0%    0.0%    0.1%    0.0%    0.0%    0.0%    0.0%    0.0%    0.0%    0.0%    0.0%    0.0%    0.0%    0.0%    0.0%    0.0%    0.0%    0.0%    0.0%    0.0%    0.0%    0.0%    0.1%    0.0%    0.0%    0.0%    0.0%    0.0%    0.0%    0.0%    0.0%    0.0%    0.0%    0.0%    0.0%    0.0%    0.0%    0.1%    0.0%    0.0%    0.0%    0.0%    0.0%    0.0%    0.0%    0.0%    0.1%    0.0%    0.0%    0.0%    0.0%    0.0%    0.0%    0.0%    0.0%    0.0%    0.0%    0.0%    0.0%    0.0%    0.0%    0.1%    0.0%    0.0%    0.2%    0.0%    0.0%    0.0%    0.0%    0.0%    0.0%    0.0%    0.0%    0.0%    0.0%    0.0%    0.0%    0.0%    0.0%    0.0%    0.0%    0.0%    0.0%    0.0%    0.0%    0.0%    0.0%    0.1%    0.1%    0.0%    0.0%    0.0%    0.0%    0.0%    0.0%    0.0%    0.0%    0.0%    0.0%    0.0%    0.0%    0.0%    0.0%    0.0%    0.0%    0.0%    0.0%    0.0%    0.0%    0.0%    0.1%    0.4%        k__Bacteria;p__Actinobacteria; c__Actinobacteria (class)   203645   20.9%   22.5%   24.8%   19.0%   24.4%   14.2%   23.7%   23.6%   12.2%    7.5%   20.5%   24.3%   26.2%   22.8%   23.0%   18.7%   22.9%   34.0%   22.4%   41.9%   21.1%   29.7%   19.3%   32.6%   14.8%   10.4%   19.1%   23.9%   33.6%   42.8%   19.9%   32.8%   30.4%   26.4%   10.3%   17.5%   26.4%   15.6%   12.6%   14.7%   14.4%   25.0%   16.0%   11.7%   15.3%   20.5%   12.7%   17.3%   13.6%   20.0%   35.0%   30.2%   10.0%   19.0%   33.8%   21.1%   20.9%   36.9%   23.1%   26.1%   18.6%   17.7%   28.2%   19.5%   12.8%   37.2%   23.5%   12.1%   18.5%   30.4%   24.8%   14.4%   19.7%   18.5%   27.1%   29.3%   34.1%   27.2%   19.9%   10.8%   17.5%   13.1%   19.9%   16.7%   23.5%   28.7%   15.8%   19.9%   19.6%   17.1%   18.9%   15.0%   38.7%   10.5%   17.5%   26.7%   14.9%   14.6%   11.3%   15.4%    9.0%   22.1%   35.1%   17.9%   12.4%   10.2%   24.4%   10.6%   24.5%   18.9%   24.5%   21.0%   24.3%   11.4%   14.8%   24.5%   15.2%    9.3%   11.8%   12.1%   35.1%   20.9%   14.9%   12.1%   14.3%   17.8%   14.7%   23.1%   21.3%   12.9%   20.7%   15.8%   14.4%   17.4%        k__Bacteria;p__Aquificae; c__Aquificae (class)      19    0.0%    0.0%    0.0%    0.0%    0.0%    0.0%    0.0%    0.0%    0.0%    0.0%    0.0%    0.0%    0.0%    0.0%    0.0%    0.0%    0.0%    0.0%    0.0%    0.0%    0.0%    0.0%    0.0%    0.0%    0.0%    0.0%    0.0%    0.0%    0.0%    0.0%    0.0%    0.0%    0.0%    0.0%    0.3%    0.0%    0.0%    0.0%    0.0%    0.0%    0.0%    0.0%    0.0%    0.0%    0.0%    0.0%    0.0%    0.0%    0.0%    0.0%    0.0%    0.0%    0.0%    0.0%    0.0%    0.0%    0.0%    0.0%    0.0%    0.0%    0.0%    0.0%    0.0%    0.0%    0.0%    0.0%    0.0%    0.0%    0.0%    0.0%    0.0%    0.0%    0.0%    0.0%    0.0%    0.0%    0.0%    0.0%    0.0%    0.0%    0.0%    0.0%    0.0%    0.0%    0.0%    0.0%    0.0%    0.0%    0.0%    0.0%    0.0%    0.0%    0.0%    0.0%    0.0%    0.0%    0.0%    0.0%    0.0%    0.0%    0.0%    0.0%    0.0%    0.0%    0.0%    0.0%    0.0%    0.0%    0.0%    0.0%    0.0%    0.0%    0.0%    0.0%    0.0%    0.0%    0.0%    0.0%    0.0%    0.0%    0.0%    0.0%    0.0%    0.0%    0.0%    0.0%    0.0%    0.0%    0.0%    0.0%    0.0%    0.0%    0.0%    0.0%        k__Bacteria;p__BRC1; c__       1    0.0%    0.0%    0.0%    0.0%    0.0%    0.0%    0.0%    0.0%    0.0%    0.0%    0.0%    0.0%    0.0%    0.0%    0.0%    0.0%    0.0%    0.0%    0.0%    0.0%    0.0%    0.0%    0.0%    0.0%    0.0%    0.0%    0.0%    0.0%    0.0%    0.0%    0.0%    0.0%    0.0%    0.0%    0.0%    0.0%    0.0%    0.0%    0.0%    0.0%    0.0%    0.0%    0.0%    0.0%    0.0%    0.0%    0.0%    0.0%    0.0%    0.0%    0.0%    0.0%    0.0%    0.0%    0.0%    0.0%    0.0%    0.0%    0.0%    0.0%    0.0%    0.0%    0.0%    0.0%    0.0%    0.0%    0.0%    0.0%    0.0%    0.0%    0.0%    0.0%    0.0%    0.0%    0.0%    0.0%    0.0%    0.0%    0.0%    0.0%    0.0%    0.0%    0.0%    0.0%    0.0%    0.0%    0.0%    0.0%    0.0%    0.0%    0.0%    0.0%    0.0%    0.0%    0.0%    0.0%    0.0%    0.0%    0.0%    0.0%    0.0%    0.0%    0.0%    0.0%    0.0%    0.0%    0.0%    0.0%    0.0%    0.0%    0.0%    0.0%    0.0%    0.0%    0.0%    0.0%    0.0%    0.0%    0.0%    0.0%    0.0%    0.0%    0.0%    0.0%    0.0%    0.0%    0.0%    0.0%    0.0%    0.0%    0.0%    0.0%    0.0%    0.0%        k__Bacteria;p__BRC1; c__PRR-11      39    0.0%    0.0%    0.0%    0.0%    0.0%    0.0%    0.0%    0.0%    0.0%    0.0%    0.0%    0.0%    0.0%    0.0%    0.0%    0.0%    0.0%    0.0%    0.0%    0.0%    0.0%    0.0%    0.0%    0.0%    0.0%    0.0%    0.0%    0.0%    0.0%    0.0%    0.0%    0.0%    0.0%    0.0%    0.0%    0.0%    0.0%    0.0%    0.0%    0.0%    0.0%    0.0%    0.0%    0.0%    0.0%    0.0%    0.0%    0.0%    0.0%    0.0%    0.0%    0.0%    0.0%    0.0%    0.0%    0.0%    0.0%    0.0%    0.0%    0.0%    0.0%    0.0%    0.0%    0.0%    0.0%    0.0%    0.0%    0.0%    0.0%    0.0%    0.0%    0.0%    0.0%    0.0%    0.0%    0.0%    0.0%    0.0%    0.0%    0.0%    0.0%    0.0%    0.0%    0.0%    0.0%    0.0%    0.0%    0.0%    0.0%    0.0%    0.0%    0.0%    0.0%    0.0%    0.0%    0.0%    0.0%    0.0%    0.0%    0.0%    0.0%    0.0%    0.0%    0.0%    0.0%    0.0%    0.0%    0.0%    0.1%    0.0%    0.0%    0.0%    0.0%    0.0%    0.0%    0.0%    0.0%    0.0%    0.0%    0.0%    0.0%    0.0%    0.0%    0.0%    0.0%    0.0%    0.0%    0.0%    0.0%    0.0%    0.0%    0.0%    0.0%    0.0%        k__Bacteria;p__Bacteroidetes; c__       4    0.0%    0.0%    0.0%    0.0%    0.0%    0.0%    0.0%    0.0%    0.0%    0.0%    0.0%    0.0%    0.0%    0.0%    0.0%    0.0%    0.0%    0.0%    0.0%    0.0%    0.0%    0.0%    0.0%    0.0%    0.0%    0.0%    0.0%    0.0%    0.0%    0.0%    0.0%    0.0%    0.0%    0.0%    0.0%    0.0%    0.0%    0.0%    0.0%    0.0%    0.0%    0.0%    0.0%    0.0%    0.0%    0.0%    0.0%    0.0%    0.0%    0.0%    0.0%    0.0%    0.0%    0.0%    0.0%    0.0%    0.0%    0.0%    0.0%    0.0%    0.0%    0.0%    0.0%    0.0%    0.0%    0.0%    0.0%    0.0%    0.0%    0.0%    0.0%    0.0%    0.0%    0.0%    0.0%    0.0%    0.0%    0.0%    0.0%    0.0%    0.0%    0.0%    0.0%    0.0%    0.0%    0.0%    0.0%    0.0%    0.0%    0.0%    0.0%    0.0%    0.0%    0.0%    0.0%    0.0%    0.0%    0.0%    0.0%    0.0%    0.0%    0.0%    0.0%    0.0%    0.0%    0.0%    0.0%    0.0%    0.0%    0.0%    0.0%    0.0%    0.0%    0.0%    0.0%    0.0%    0.0%    0.0%    0.0%    0.0%    0.0%    0.0%    0.0%    0.0%    0.0%    0.0%    0.0%    0.0%    0.0%    0.0%    0.0%    0.0%    0.0%    0.0%        k__Bacteria;p__Bacteroidetes; c__Bacteroidia   40427    4.1%    4.6%    3.0%    2.9%    2.1%    1.7%    1.8%    2.6%    2.1%    0.5%    1.9%    6.1%    3.5%    2.4%    3.8%    4.3%    1.7%    3.6%    4.6%    5.3%    1.5%    3.9%    5.9%    4.1%    5.5%    8.3%    1.8%    2.3%    5.3%    4.3%    3.2%    2.6%    3.0%    2.7%   14.9%    1.4%    4.6%    2.7%    3.9%    4.2%    1.8%    2.2%    2.0%    3.9%    4.0%    0.9%    4.0%    2.7%    3.2%    2.2%    2.6%    1.9%    6.9%    4.6%    2.5%    5.6%    1.7%    1.1%    2.1%    8.6%    2.9%    3.6%    1.5%    5.4%    6.4%    2.9%    1.9%    1.1%    3.0%    3.4%    6.5%    9.7%    4.4%   17.1%    3.9%    2.8%    3.0%    2.5%    3.2%   15.4%    6.4%    4.4%    4.9%    8.1%    4.4%    5.2%    4.2%   10.4%    3.4%    5.1%    3.3%   12.7%    4.6%    5.4%   12.5%    5.0%   12.7%   10.8%    3.9%    4.0%    1.5%    6.0%    4.0%    4.5%    7.5%    1.3%    3.4%    1.5%    2.8%    1.3%    2.1%    4.1%    7.4%    2.7%    1.1%    2.5%    0.7%    0.5%    1.5%    5.2%    2.2%    3.6%   15.2%    3.0%    3.5%    3.2%    2.2%    3.5%    3.7%    0.9%    1.8%    3.1%    2.2%    2.7%        k__Bacteria;p__Bacteroidetes; c__Flavobacteria   13919    1.4%    1.8%    0.7%    0.6%    1.3%    1.5%    1.4%    0.4%    0.6%    1.1%    0.9%    2.2%    0.5%    0.6%    1.0%    0.9%    1.1%    0.6%    1.4%    0.6%    0.9%    1.6%    1.9%    0.7%    2.1%    3.1%    1.5%    0.8%    1.0%    0.5%    0.6%    0.6%    1.6%    0.8%    0.8%    0.9%    0.6%    1.5%    4.5%    1.7%    0.7%    1.1%    1.3%    2.4%    1.1%    0.3%    1.2%    4.6%    4.1%    0.8%    0.3%    0.7%    1.6%    0.9%    0.8%    1.3%    0.6%    0.4%    0.7%    0.6%    0.9%    1.7%    0.1%    0.3%    1.4%    0.2%    0.8%    0.5%    1.1%    1.3%    0.3%    1.4%    1.9%    0.9%    1.2%    0.4%    0.4%    2.3%    1.1%    1.0%    0.4%    1.3%    0.4%    1.7%    1.2%    1.5%    1.4%    1.3%    1.5%    0.8%    1.1%    2.4%    0.6%    3.7%    1.8%    0.8%    1.2%    1.2%    2.3%    1.2%    0.5%    0.9%    0.4%    1.0%    1.2%    0.5%    1.9%    1.1%    1.0%    2.4%    0.7%    1.3%    0.9%    0.4%    0.5%    2.1%    1.1%    0.5%    1.0%    1.7%    1.2%    1.3%    1.0%    4.5%    8.7%    6.3%    1.8%    3.7%    2.1%    3.7%    4.8%    8.9%    3.2%    2.2%        k__Bacteria;p__Bacteroidetes; c__Sphingobacteria   21588    2.2%    1.0%    0.9%    1.0%    0.6%    3.9%    2.3%    1.9%    1.6%    0.3%    1.8%    1.4%    0.2%    1.7%    1.2%    0.8%    3.1%    0.4%    2.3%    0.5%    7.1%    1.2%    0.6%    0.7%    4.0%    1.0%    6.1%    1.7%    1.1%    0.8%    0.8%    0.8%    0.9%    0.6%    0.6%    1.1%    0.3%    1.4%    1.3%    1.2%    0.2%    3.5%    1.9%    0.6%    0.8%    0.5%    1.2%    0.3%    0.8%    0.9%    0.5%    1.2%    0.7%    2.9%    2.1%    1.5%    1.1%    0.3%    2.0%    1.8%    1.5%    0.8%    0.1%    0.3%    1.5%    0.8%    3.3%    1.3%    1.6%    2.2%    0.4%    0.8%    1.3%    0.9%    1.8%    0.3%    0.3%    3.5%    5.9%    2.3%    6.1%    6.6%   10.0%    5.2%    2.4%    2.3%    0.9%    1.8%    2.3%    1.2%    1.4%    1.3%    0.3%    8.1%    1.7%    2.5%    1.5%    1.6%    2.1%    0.9%    0.6%    1.2%    1.1%    1.9%    2.3%    1.5%    1.5%    0.9%    5.0%    6.1%    2.1%    2.0%    1.3%    3.1%   13.4%    2.1%   18.6%   19.7%   11.8%    0.5%    1.4%    2.0%    0.6%    1.7%    2.3%    1.8%    1.2%    1.8%    1.3%    3.2%    1.5%    2.0%    4.4%    2.9%        k__Bacteria;p__CCM11b; c__       5    0.0%    0.0%    0.0%    0.0%    0.0%    0.0%    0.0%    0.0%    0.0%    0.0%    0.0%    0.0%    0.0%    0.0%    0.0%    0.0%    0.0%    0.0%    0.0%    0.0%    0.0%    0.0%    0.0%    0.0%    0.0%    0.0%    0.0%    0.0%    0.0%    0.0%    0.0%    0.0%    0.0%    0.0%    0.0%    0.0%    0.0%    0.0%    0.0%    0.0%    0.0%    0.0%    0.0%    0.0%    0.0%    0.0%    0.0%    0.0%    0.0%    0.0%    0.0%    0.0%    0.0%    0.0%    0.0%    0.0%    0.0%    0.0%    0.0%    0.0%    0.0%    0.0%    0.0%    0.0%    0.0%    0.0%    0.0%    0.0%    0.0%    0.0%    0.0%    0.0%    0.0%    0.0%    0.0%    0.0%    0.0%    0.0%    0.0%    0.0%    0.0%    0.0%    0.0%    0.0%    0.0%    0.0%    0.0%    0.0%    0.0%    0.0%    0.0%    0.0%    0.0%    0.0%    0.0%    0.0%    0.0%    0.0%    0.0%    0.0%    0.0%    0.0%    0.0%    0.0%    0.0%    0.0%    0.0%    0.0%    0.0%    0.0%    0.0%    0.0%    0.0%    0.0%    0.0%    0.0%    0.0%    0.0%    0.0%    0.0%    0.0%    0.0%    0.0%    0.0%    0.0%    0.0%    0.0%    0.0%    0.0%    0.0%    0.0%    0.0%    0.0%    0.0%        k__Bacteria;p__Caldithrix_KSB1; c__Caldithrixae       0    0.0%    0.0%    0.0%    0.0%    0.0%    0.0%    0.0%    0.0%    0.0%    0.0%    0.0%    0.0%    0.0%    0.0%    0.0%    0.0%    0.0%    0.0%    0.0%    0.0%    0.0%    0.0%    0.0%    0.0%    0.0%    0.0%    0.0%    0.0%    0.0%    0.0%    0.0%    0.0%    0.0%    0.0%    0.0%    0.0%    0.0%    0.0%    0.0%    0.0%    0.0%    0.0%    0.0%    0.0%    0.0%    0.0%    0.0%    0.0%    0.0%    0.0%    0.0%    0.0%    0.0%    0.0%    0.0%    0.0%    0.0%    0.0%    0.0%    0.0%    0.0%    0.0%    0.0%    0.0%    0.0%    0.0%    0.0%    0.0%    0.0%    0.0%    0.0%    0.0%    0.0%    0.0%    0.0%    0.0%    0.0%    0.0%    0.0%    0.0%    0.0%    0.0%    0.0%    0.0%    0.0%    0.0%    0.0%    0.0%    0.0%    0.0%    0.0%    0.0%    0.0%    0.0%    0.0%    0.0%    0.0%    0.0%    0.0%    0.0%    0.0%    0.0%    0.0%    0.0%    0.0%    0.0%    0.0%    0.0%    0.0%    0.0%    0.0%    0.0%    0.0%    0.0%    0.0%    0.0%    0.0%    0.0%    0.0%    0.0%    0.0%    0.0%    0.0%    0.0%    0.0%    0.0%    0.0%    0.0%    0.0%    0.0%    0.0%    0.0%    0.0%    0.0%        k__Bacteria;p__Chlamydiae; c__Chlamydiae (class)       1    0.0%    0.0%    0.0%    0.0%    0.0%    0.0%    0.0%    0.0%    0.0%    0.0%    0.0%    0.0%    0.0%    0.0%    0.0%    0.0%    0.0%    0.0%    0.0%    0.0%    0.0%    0.0%    0.0%    0.0%    0.0%    0.0%    0.0%    0.0%    0.0%    0.0%    0.0%    0.0%    0.0%    0.0%    0.0%    0.0%    0.0%    0.0%    0.0%    0.0%    0.0%    0.0%    0.0%    0.0%    0.0%    0.0%    0.0%    0.0%    0.0%    0.0%    0.0%    0.0%    0.0%    0.0%    0.0%    0.0%    0.0%    0.0%    0.0%    0.0%    0.0%    0.0%    0.0%    0.0%    0.0%    0.0%    0.0%    0.0%    0.0%    0.0%    0.0%    0.0%    0.0%    0.0%    0.0%    0.0%    0.0%    0.0%    0.0%    0.0%    0.0%    0.0%    0.0%    0.0%    0.0%    0.0%    0.0%    0.0%    0.0%    0.0%    0.0%    0.0%    0.0%    0.0%    0.0%    0.0%    0.0%    0.0%    0.0%    0.0%    0.0%    0.0%    0.0%    0.0%    0.0%    0.0%    0.0%    0.0%    0.0%    0.0%    0.0%    0.0%    0.0%    0.0%    0.0%    0.0%    0.0%    0.0%    0.0%    0.0%    0.0%    0.0%    0.0%    0.0%    0.0%    0.0%    0.0%    0.0%    0.0%    0.0%    0.0%    0.0%    0.0%    0.0%        k__Bacteria;p__Chlorobi; c__       0    0.0%    0.0%    0.0%    0.0%    0.0%    0.0%    0.0%    0.0%    0.0%    0.0%    0.0%    0.0%    0.0%    0.0%    0.0%    0.0%    0.0%    0.0%    0.0%    0.0%    0.0%    0.0%    0.0%    0.0%    0.0%    0.0%    0.0%    0.0%    0.0%    0.0%    0.0%    0.0%    0.0%    0.0%    0.0%    0.0%    0.0%    0.0%    0.0%    0.0%    0.0%    0.0%    0.0%    0.0%    0.0%    0.0%    0.0%    0.0%    0.0%    0.0%    0.0%    0.0%    0.0%    0.0%    0.0%    0.0%    0.0%    0.0%    0.0%    0.0%    0.0%    0.0%    0.0%    0.0%    0.0%    0.0%    0.0%    0.0%    0.0%    0.0%    0.0%    0.0%    0.0%    0.0%    0.0%    0.0%    0.0%    0.0%    0.0%    0.0%    0.0%    0.0%    0.0%    0.0%    0.0%    0.0%    0.0%    0.0%    0.0%    0.0%    0.0%    0.0%    0.0%    0.0%    0.0%    0.0%    0.0%    0.0%    0.0%    0.0%    0.0%    0.0%    0.0%    0.0%    0.0%    0.0%    0.0%    0.0%    0.0%    0.0%    0.0%    0.0%    0.0%    0.0%    0.0%    0.0%    0.0%    0.0%    0.0%    0.0%    0.0%    0.0%    0.0%    0.0%    0.0%    0.0%    0.0%    0.0%    0.0%    0.0%    0.0%    0.0%    0.0%    0.0%        k__Bacteria;p__Chlorobi; c__BSV19       0    0.0%    0.0%    0.0%    0.0%    0.0%    0.0%    0.0%    0.0%    0.0%    0.0%    0.0%    0.0%    0.0%    0.0%    0.0%    0.0%    0.0%    0.0%    0.0%    0.0%    0.0%    0.0%    0.0%    0.0%    0.0%    0.0%    0.0%    0.0%    0.0%    0.0%    0.0%    0.0%    0.0%    0.0%    0.0%    0.0%    0.0%    0.0%    0.0%    0.0%    0.0%    0.0%    0.0%    0.0%    0.0%    0.0%    0.0%    0.0%    0.0%    0.0%    0.0%    0.0%    0.0%    0.0%    0.0%    0.0%    0.0%    0.0%    0.0%    0.0%    0.0%    0.0%    0.0%    0.0%    0.0%    0.0%    0.0%    0.0%    0.0%    0.0%    0.0%    0.0%    0.0%    0.0%    0.0%    0.0%    0.0%    0.0%    0.0%    0.0%    0.0%    0.0%    0.0%    0.0%    0.0%    0.0%    0.0%    0.0%    0.0%    0.0%    0.0%    0.0%    0.0%    0.0%    0.0%    0.0%    0.0%    0.0%    0.0%    0.0%    0.0%    0.0%    0.0%    0.0%    0.0%    0.0%    0.0%    0.0%    0.0%    0.0%    0.0%    0.0%    0.0%    0.0%    0.0%    0.0%    0.0%    0.0%    0.0%    0.0%    0.0%    0.0%    0.0%    0.0%    0.0%    0.0%    0.0%    0.0%    0.0%    0.0%    0.0%    0.0%    0.0%    0.0%        k__Bacteria;p__Chlorobi; c__OPB56       1    0.0%    0.0%    0.0%    0.0%    0.0%    0.0%    0.0%    0.0%    0.0%    0.0%    0.0%    0.0%    0.0%    0.0%    0.0%    0.0%    0.0%    0.0%    0.0%    0.0%    0.0%    0.0%    0.0%    0.0%    0.0%    0.0%    0.0%    0.0%    0.0%    0.0%    0.0%    0.0%    0.0%    0.0%    0.0%    0.0%    0.0%    0.0%    0.0%    0.0%    0.0%    0.0%    0.0%    0.0%    0.0%    0.0%    0.0%    0.0%    0.0%    0.0%    0.0%    0.0%    0.0%    0.0%    0.0%    0.0%    0.0%    0.0%    0.0%    0.0%    0.0%    0.0%    0.0%    0.0%    0.0%    0.0%    0.0%    0.0%    0.0%    0.0%    0.0%    0.0%    0.0%    0.0%    0.0%    0.0%    0.0%    0.0%    0.0%    0.0%    0.0%    0.0%    0.0%    0.0%    0.0%    0.0%    0.0%    0.0%    0.0%    0.0%    0.0%    0.0%    0.0%    0.0%    0.0%    0.0%    0.0%    0.0%    0.0%    0.0%    0.0%    0.0%    0.0%    0.0%    0.0%    0.0%    0.0%    0.0%    0.0%    0.0%    0.0%    0.0%    0.0%    0.0%    0.0%    0.0%    0.0%    0.0%    0.0%    0.0%    0.0%    0.0%    0.0%    0.0%    0.0%    0.0%    0.0%    0.0%    0.0%    0.0%    0.0%    0.0%    0.0%    0.0%        k__Bacteria;p__Chlorobi; c__SJA-28       7    0.0%    0.0%    0.0%    0.0%    0.0%    0.0%    0.0%    0.0%    0.0%    0.0%    0.0%    0.0%    0.0%    0.0%    0.0%    0.0%    0.0%    0.0%    0.0%    0.0%    0.0%    0.0%    0.0%    0.0%    0.0%    0.0%    0.0%    0.0%    0.0%    0.0%    0.0%    0.0%    0.0%    0.0%    0.0%    0.0%    0.0%    0.0%    0.0%    0.0%    0.0%    0.0%    0.0%    0.0%    0.0%    0.0%    0.0%    0.0%    0.0%    0.0%    0.0%    0.0%    0.0%    0.0%    0.0%    0.0%    0.0%    0.0%    0.0%    0.0%    0.0%    0.0%    0.0%    0.0%    0.0%    0.0%    0.0%    0.0%    0.0%    0.0%    0.0%    0.0%    0.0%    0.0%    0.0%    0.0%    0.0%    0.0%    0.0%    0.0%    0.0%    0.0%    0.0%    0.0%    0.0%    0.0%    0.0%    0.0%    0.0%    0.0%    0.0%    0.0%    0.0%    0.0%    0.0%    0.0%    0.0%    0.0%    0.0%    0.0%    0.0%    0.0%    0.0%    0.0%    0.0%    0.0%    0.0%    0.0%    0.0%    0.0%    0.0%    0.0%    0.0%    0.0%    0.0%    0.0%    0.0%    0.0%    0.0%    0.0%    0.0%    0.0%    0.0%    0.0%    0.0%    0.0%    0.0%    0.0%    0.0%    0.0%    0.0%    0.0%    0.0%    0.0%        k__Bacteria;p__Chlorobi; c__ZB1       1    0.0%    0.0%    0.0%    0.0%    0.0%    0.0%    0.0%    0.0%    0.0%    0.0%    0.0%    0.0%    0.0%    0.0%    0.0%    0.0%    0.0%    0.0%    0.0%    0.0%    0.0%    0.0%    0.0%    0.0%    0.0%    0.0%    0.0%    0.0%    0.0%    0.0%    0.0%    0.0%    0.0%    0.0%    0.0%    0.0%    0.0%    0.0%    0.0%    0.0%    0.0%    0.0%    0.0%    0.0%    0.0%    0.0%    0.0%    0.0%    0.0%    0.0%    0.0%    0.0%    0.0%    0.0%    0.0%    0.0%    0.0%    0.0%    0.0%    0.0%    0.0%    0.0%    0.0%    0.0%    0.0%    0.0%    0.0%    0.0%    0.0%    0.0%    0.0%    0.0%    0.0%    0.0%    0.0%    0.0%    0.0%    0.0%    0.0%    0.0%    0.0%    0.0%    0.0%    0.0%    0.0%    0.0%    0.0%    0.0%    0.0%    0.0%    0.0%    0.0%    0.0%    0.0%    0.0%    0.0%    0.0%    0.0%    0.0%    0.0%    0.0%    0.0%    0.0%    0.0%    0.0%    0.0%    0.0%    0.0%    0.0%    0.0%    0.0%    0.0%    0.0%    0.0%    0.0%    0.0%    0.0%    0.0%    0.0%    0.0%    0.0%    0.0%    0.0%    0.0%    0.0%    0.0%    0.0%    0.0%    0.0%    0.0%    0.0%    0.0%    0.0%    0.0%        k__Bacteria;p__Chloroflexi; c__       0    0.0%    0.0%    0.0%    0.0%    0.0%    0.0%    0.0%    0.0%    0.0%    0.0%    0.0%    0.0%    0.0%    0.0%    0.0%    0.0%    0.0%    0.0%    0.0%    0.0%    0.0%    0.0%    0.0%    0.0%    0.0%    0.0%    0.0%    0.0%    0.0%    0.0%    0.0%    0.0%    0.0%    0.0%    0.0%    0.0%    0.0%    0.0%    0.0%    0.0%    0.0%    0.0%    0.0%    0.0%    0.0%    0.0%    0.0%    0.0%    0.0%    0.0%    0.0%    0.0%    0.0%    0.0%    0.0%    0.0%    0.0%    0.0%    0.0%    0.0%    0.0%    0.0%    0.0%    0.0%    0.0%    0.0%    0.0%    0.0%    0.0%    0.0%    0.0%    0.0%    0.0%    0.0%    0.0%    0.0%    0.0%    0.0%    0.0%    0.0%    0.0%    0.0%    0.0%    0.0%    0.0%    0.0%    0.0%    0.0%    0.0%    0.0%    0.0%    0.0%    0.0%    0.0%    0.0%    0.0%    0.0%    0.0%    0.0%    0.0%    0.0%    0.0%    0.0%    0.0%    0.0%    0.0%    0.0%    0.0%    0.0%    0.0%    0.0%    0.0%    0.0%    0.0%    0.0%    0.0%    0.0%    0.0%    0.0%    0.0%    0.0%    0.0%    0.0%    0.0%    0.0%    0.0%    0.0%    0.0%    0.0%    0.0%    0.0%    0.0%    0.0%    0.0%        k__Bacteria;p__Chloroflexi; c__Anaerolineae      63    0.0%    0.0%    0.0%    0.0%    0.0%    0.0%    0.0%    0.0%    0.0%    0.0%    0.0%    0.0%    0.0%    0.0%    0.0%    0.0%    0.0%    0.0%    0.0%    0.0%    0.0%    0.0%    0.0%    0.0%    0.0%    0.0%    0.0%    0.0%    0.0%    0.0%    0.0%    0.0%    0.0%    0.0%    0.0%    0.0%    0.0%    0.0%    0.0%    0.0%    0.0%    0.0%    0.0%    0.0%    0.0%    0.0%    0.0%    0.0%    0.0%    0.0%    0.0%    0.0%    0.0%    0.0%    0.1%    0.0%    0.0%    0.0%    0.0%    0.0%    0.0%    0.0%    0.0%    0.0%    0.0%    0.0%    0.0%    0.0%    0.0%    0.0%    0.0%    0.0%    0.0%    0.0%    0.0%    0.0%    0.0%    0.0%    0.0%    0.0%    0.0%    0.0%    0.0%    0.0%    0.0%    0.0%    0.0%    0.0%    0.0%    0.0%    0.0%    0.0%    0.0%    0.0%    0.0%    0.0%    0.0%    0.0%    0.0%    0.0%    0.0%    0.0%    0.0%    0.0%    0.0%    0.0%    0.0%    0.0%    0.1%    0.0%    0.0%    0.0%    0.0%    0.0%    0.0%    0.0%    0.0%    0.0%    0.0%    0.0%    0.0%    0.0%    0.0%    0.0%    0.0%    0.0%    0.0%    0.0%    0.0%    0.0%    0.0%    0.0%    0.0%    0.0%        k__Bacteria;p__Chloroflexi; c__Bljii12      81    0.0%    0.0%    0.0%    0.0%    0.0%    0.0%    0.0%    0.0%    0.0%    0.0%    0.0%    0.0%    0.0%    0.0%    0.0%    0.0%    0.0%    0.0%    0.0%    0.0%    0.0%    0.0%    0.0%    0.0%    0.0%    0.0%    0.0%    0.0%    0.0%    0.0%    0.0%    0.0%    0.0%    0.0%    0.0%    0.0%    0.0%    0.0%    0.0%    0.0%    0.0%    0.0%    0.0%    0.0%    0.0%    0.0%    0.0%    0.0%    0.0%    0.0%    0.0%    0.0%    0.0%    0.0%    0.0%    0.0%    0.0%    0.0%    0.1%    0.0%    0.0%    0.0%    0.0%    0.0%    0.0%    0.0%    0.0%    0.0%    0.0%    0.0%    0.0%    0.0%    0.0%    0.0%    0.0%    0.0%    0.0%    0.0%    0.0%    0.0%    0.0%    0.0%    0.0%    0.0%    0.0%    0.0%    0.0%    0.0%    0.0%    0.0%    0.0%    0.0%    0.0%    0.0%    0.0%    0.0%    0.0%    0.0%    0.0%    0.0%    0.0%    0.0%    0.0%    0.0%    0.0%    0.0%    0.0%    0.0%    0.0%    0.0%    0.0%    0.0%    0.0%    0.0%    0.0%    0.0%    0.0%    0.0%    0.0%    0.0%    0.0%    0.0%    0.0%    0.0%    0.0%    0.0%    0.0%    0.0%    0.0%    0.0%    0.0%    0.0%    0.0%    0.0%        k__Bacteria;p__Chloroflexi; c__Chloroflexi (class)       3    0.0%    0.0%    0.0%    0.0%    0.0%    0.0%    0.0%    0.0%    0.0%    0.0%    0.0%    0.0%    0.0%    0.0%    0.0%    0.0%    0.0%    0.0%    0.0%    0.0%    0.0%    0.0%    0.0%    0.0%    0.0%    0.0%    0.0%    0.0%    0.0%    0.0%    0.0%    0.0%    0.0%    0.0%    0.0%    0.0%    0.0%    0.0%    0.0%    0.0%    0.0%    0.0%    0.0%    0.0%    0.0%    0.0%    0.0%    0.0%    0.0%    0.0%    0.0%    0.0%    0.0%    0.0%    0.0%    0.0%    0.0%    0.0%    0.0%    0.0%    0.0%    0.0%    0.0%    0.0%    0.0%    0.0%    0.0%    0.0%    0.0%    0.0%    0.0%    0.0%    0.0%    0.0%    0.0%    0.0%    0.0%    0.0%    0.0%    0.0%    0.0%    0.0%    0.0%    0.0%    0.0%    0.0%    0.0%    0.0%    0.0%    0.0%    0.0%    0.0%    0.0%    0.0%    0.0%    0.0%    0.0%    0.0%    0.0%    0.0%    0.0%    0.0%    0.0%    0.0%    0.0%    0.0%    0.0%    0.0%    0.0%    0.0%    0.0%    0.0%    0.0%    0.0%    0.0%    0.0%    0.0%    0.0%    0.0%    0.0%    0.0%    0.0%    0.0%    0.0%    0.0%    0.0%    0.0%    0.0%    0.0%    0.0%    0.0%    0.0%    0.0%    0.0%        k__Bacteria;p__Chloroflexi; c__Chloroflexi-4       0    0.0%    0.0%    0.0%    0.0%    0.0%    0.0%    0.0%    0.0%    0.0%    0.0%    0.0%    0.0%    0.0%    0.0%    0.0%    0.0%    0.0%    0.0%    0.0%    0.0%    0.0%    0.0%    0.0%    0.0%    0.0%    0.0%    0.0%    0.0%    0.0%    0.0%    0.0%    0.0%    0.0%    0.0%    0.0%    0.0%    0.0%    0.0%    0.0%    0.0%    0.0%    0.0%    0.0%    0.0%    0.0%    0.0%    0.0%    0.0%    0.0%    0.0%    0.0%    0.0%    0.0%    0.0%    0.0%    0.0%    0.0%    0.0%    0.0%    0.0%    0.0%    0.0%    0.0%    0.0%    0.0%    0.0%    0.0%    0.0%    0.0%    0.0%    0.0%    0.0%    0.0%    0.0%    0.0%    0.0%    0.0%    0.0%    0.0%    0.0%    0.0%    0.0%    0.0%    0.0%    0.0%    0.0%    0.0%    0.0%    0.0%    0.0%    0.0%    0.0%    0.0%    0.0%    0.0%    0.0%    0.0%    0.0%    0.0%    0.0%    0.0%    0.0%    0.0%    0.0%    0.0%    0.0%    0.0%    0.0%    0.0%    0.0%    0.0%    0.0%    0.0%    0.0%    0.0%    0.0%    0.0%    0.0%    0.0%    0.0%    0.0%    0.0%    0.0%    0.0%    0.0%    0.0%    0.0%    0.0%    0.0%    0.0%    0.0%    0.0%    0.0%    0.0%        k__Bacteria;p__Chloroflexi; c__Ktedonobacteria       2    0.0%    0.0%    0.0%    0.0%    0.0%    0.0%    0.0%    0.0%    0.0%    0.0%    0.0%    0.0%    0.0%    0.0%    0.0%    0.0%    0.0%    0.0%    0.0%    0.0%    0.0%    0.0%    0.0%    0.0%    0.0%    0.0%    0.0%    0.0%    0.0%    0.0%    0.0%    0.0%    0.0%    0.0%    0.0%    0.0%    0.0%    0.0%    0.0%    0.0%    0.0%    0.0%    0.0%    0.0%    0.0%    0.0%    0.0%    0.0%    0.0%    0.0%    0.0%    0.0%    0.0%    0.0%    0.0%    0.0%    0.0%    0.0%    0.0%    0.0%    0.0%    0.0%    0.0%    0.0%    0.0%    0.0%    0.0%    0.0%    0.0%    0.0%    0.0%    0.0%    0.0%    0.0%    0.0%    0.0%    0.0%    0.0%    0.0%    0.0%    0.0%    0.0%    0.0%    0.0%    0.0%    0.0%    0.0%    0.0%    0.0%    0.0%    0.0%    0.0%    0.0%    0.0%    0.0%    0.0%    0.0%    0.0%    0.0%    0.0%    0.0%    0.0%    0.0%    0.0%    0.0%    0.0%    0.0%    0.0%    0.0%    0.0%    0.0%    0.0%    0.0%    0.0%    0.0%    0.0%    0.0%    0.0%    0.0%    0.0%    0.0%    0.0%    0.0%    0.0%    0.0%    0.0%    0.0%    0.0%    0.0%    0.0%    0.0%    0.0%    0.0%    0.0%        k__Bacteria;p__Chloroflexi; c__SOGA31    1195    0.1%    0.1%    0.2%    0.7%    0.0%    0.1%    0.1%    0.0%    0.1%    0.0%    0.2%    0.1%    0.1%    0.1%    0.1%    0.0%    0.3%    0.0%    0.1%    0.0%    0.1%    0.0%    0.0%    0.0%    0.0%    0.0%    0.3%    0.1%    0.1%    0.0%    0.0%    0.1%    0.1%    0.0%    0.0%    0.1%    0.0%    0.1%    0.1%    0.3%    0.0%    0.1%    0.1%    0.0%    0.1%    0.0%    0.2%    0.1%    0.1%    0.1%    0.0%    0.0%    0.1%    0.5%    0.1%    0.1%    0.1%    0.0%    0.2%    0.3%    0.3%    0.2%    0.0%    0.0%    0.1%    0.0%    0.4%    0.1%    0.1%    0.2%    0.0%    0.1%    0.3%    0.1%    0.4%    0.0%    0.0%    0.6%    0.2%    0.1%    0.2%    0.1%    0.0%    0.2%    0.2%    0.3%    0.0%    0.2%    0.1%    0.0%    0.1%    0.1%    0.0%    0.2%    0.2%    0.2%    0.1%    0.2%    0.1%    0.1%    0.1%    0.2%    0.2%    0.2%    0.1%    0.1%    0.1%    0.2%    0.4%    0.1%    0.1%    0.2%    0.2%    0.0%    0.1%    0.3%    0.0%    0.1%    0.1%    0.1%    0.2%    0.3%    0.0%    0.1%    0.1%    0.2%    0.1%    0.1%    0.3%    0.3%    0.1%    0.1%    0.1%    0.2%        k__Bacteria;p__Chloroflexi; c__TK17      36    0.0%    0.0%    0.0%    0.0%    0.0%    0.0%    0.0%    0.0%    0.0%    0.0%    0.0%    0.0%    0.0%    0.0%    0.0%    0.0%    0.0%    0.0%    0.0%    0.0%    0.0%    0.0%    0.0%    0.0%    0.0%    0.0%    0.0%    0.0%    0.0%    0.0%    0.0%    0.0%    0.0%    0.0%    0.0%    0.0%    0.0%    0.0%    0.0%    0.0%    0.0%    0.0%    0.0%    0.0%    0.0%    0.0%    0.0%    0.0%    0.0%    0.0%    0.0%    0.0%    0.0%    0.0%    0.0%    0.0%    0.0%    0.0%    0.0%    0.0%    0.0%    0.0%    0.0%    0.0%    0.0%    0.0%    0.0%    0.0%    0.0%    0.0%    0.0%    0.0%    0.0%    0.0%    0.0%    0.0%    0.0%    0.0%    0.0%    0.0%    0.0%    0.0%    0.0%    0.0%    0.0%    0.0%    0.0%    0.0%    0.0%    0.0%    0.0%    0.0%    0.0%    0.0%    0.0%    0.0%    0.0%    0.0%    0.0%    0.0%    0.0%    0.0%    0.0%    0.0%    0.0%    0.0%    0.0%    0.0%    0.0%    0.0%    0.0%    0.0%    0.0%    0.0%    0.0%    0.0%    0.0%    0.0%    0.0%    0.0%    0.0%    0.0%    0.0%    0.0%    0.0%    0.0%    0.0%    0.0%    0.0%    0.0%    0.0%    0.0%    0.0%    0.0%        k__Bacteria;p__Chloroflexi; c__Thermobacula       1    0.0%    0.0%    0.0%    0.0%    0.0%    0.0%    0.0%    0.0%    0.0%    0.0%    0.0%    0.0%    0.0%    0.0%    0.0%    0.0%    0.0%    0.0%    0.0%    0.0%    0.0%    0.0%    0.0%    0.0%    0.0%    0.0%    0.0%    0.0%    0.0%    0.0%    0.0%    0.0%    0.0%    0.0%    0.0%    0.0%    0.0%    0.0%    0.0%    0.0%    0.0%    0.0%    0.0%    0.0%    0.0%    0.0%    0.0%    0.0%    0.0%    0.0%    0.0%    0.0%    0.0%    0.0%    0.0%    0.0%    0.0%    0.0%    0.0%    0.0%    0.0%    0.0%    0.0%    0.0%    0.0%    0.0%    0.0%    0.0%    0.0%    0.0%    0.0%    0.0%    0.0%    0.0%    0.0%    0.0%    0.0%    0.0%    0.0%    0.0%    0.0%    0.0%    0.0%    0.0%    0.0%    0.0%    0.0%    0.0%    0.0%    0.0%    0.0%    0.0%    0.0%    0.0%    0.0%    0.0%    0.0%    0.0%    0.0%    0.0%    0.0%    0.0%    0.0%    0.0%    0.0%    0.0%    0.0%    0.0%    0.0%    0.0%    0.0%    0.0%    0.0%    0.0%    0.0%    0.0%    0.0%    0.0%    0.0%    0.0%    0.0%    0.0%    0.0%    0.0%    0.0%    0.0%    0.0%    0.0%    0.0%    0.0%    0.0%    0.0%    0.0%    0.0%        k__Bacteria;p__Chloroflexi; c__Thermomicrobia    1019    0.1%    0.1%    0.1%    0.0%    0.0%    0.1%    0.0%    0.1%    0.1%    0.1%    0.1%    0.1%    0.0%    0.0%    0.1%    0.1%    0.1%    0.1%    0.2%    0.1%    0.2%    0.2%    0.1%    0.1%    0.0%    0.0%    0.1%    0.1%    0.2%    0.1%    0.1%    0.0%    0.1%    0.1%    0.1%    0.1%    0.1%    0.1%    0.2%    0.2%    0.0%    0.1%    0.1%    0.1%    0.1%    0.0%    0.2%    0.0%    0.1%    0.2%    0.0%    0.0%    0.0%    0.2%    0.3%    0.2%    0.1%    0.0%    0.3%    0.2%    0.2%    0.0%    0.0%    0.0%    0.1%    0.0%    0.1%    0.0%    0.2%    0.2%    0.1%    0.1%    0.1%    0.1%    0.1%    0.0%    0.0%    0.2%    0.1%    0.0%    0.1%    0.0%    0.0%    0.1%    0.1%    0.2%    0.1%    0.1%    0.3%    0.1%    0.2%    0.0%    0.0%    0.1%    0.2%    0.1%    0.1%    0.2%    0.2%    0.2%    0.0%    0.2%    0.0%    0.2%    0.0%    0.0%    0.2%    0.1%    0.4%    0.5%    0.1%    0.2%    0.2%    0.0%    0.1%    0.1%    0.1%    0.2%    0.0%    0.1%    0.2%    0.1%    0.0%    0.0%    0.1%    0.1%    0.1%    0.1%    0.0%    0.4%    0.0%    0.1%    0.1%    0.2%        k__Bacteria;p__Cyanobacteria; c__   52882    5.4%    8.0%    1.2%    2.0%    0.8%    8.5%    1.0%    1.3%   14.5%   34.3%    5.3%    7.7%    0.4%    7.4%    3.7%    2.4%    4.1%    1.2%    5.2%    1.0%    8.2%    1.4%    1.1%    0.5%    6.9%    3.6%    4.3%    2.4%    1.2%    1.0%    2.9%    3.4%    2.6%    1.7%    3.4%    4.2%    8.9%    8.4%    5.7%    7.2%    6.1%    2.8%    2.8%    8.0%    4.4%    1.8%    5.8%    1.5%    5.0%    7.6%    0.8%    0.5%    7.3%    4.2%    2.5%    4.7%   10.1%   11.6%   12.4%    8.5%    3.3%    1.7%    0.2%    1.9%    1.1%    2.2%    6.0%    2.1%   12.5%    3.7%    1.0%    1.5%    1.6%    5.6%    3.6%    0.9%    0.6%    3.5%    6.8%    1.1%    3.5%    4.1%    0.7%    4.0%    6.5%    1.6%    5.5%    1.7%    6.5%   11.8%   19.9%    3.8%    0.5%    1.9%    6.8%    5.7%    5.4%    5.9%    4.8%    6.8%    7.2%    5.2%    1.9%    6.3%    7.0%   14.9%   11.5%    6.1%    3.9%    2.3%    7.2%    6.6%    4.2%   16.0%    5.8%    8.1%    1.4%    4.2%   10.5%   11.4%    7.3%    7.9%    2.6%   17.4%    9.3%    0.9%   19.7%    9.0%    5.5%   19.3%    3.0%   11.6%    9.8%    9.8%        k__Bacteria;p__Cyanobacteria; c__S15B-MN24      17    0.0%    0.0%    0.0%    0.0%    0.0%    0.0%    0.0%    0.0%    0.0%    0.0%    0.0%    0.0%    0.0%    0.0%    0.0%    0.0%    0.0%    0.0%    0.0%    0.0%    0.0%    0.0%    0.0%    0.0%    0.0%    0.0%    0.0%    0.0%    0.0%    0.0%    0.0%    0.0%    0.0%    0.0%    0.0%    0.0%    0.0%    0.0%    0.0%    0.0%    0.0%    0.0%    0.0%    0.0%    0.0%    0.0%    0.0%    0.0%    0.0%    0.0%    0.0%    0.0%    0.0%    0.0%    0.0%    0.0%    0.0%    0.0%    0.0%    0.0%    0.0%    0.0%    0.0%    0.0%    0.0%    0.0%    0.0%    0.0%    0.0%    0.0%    0.0%    0.0%    0.0%    0.0%    0.0%    0.0%    0.0%    0.0%    0.0%    0.0%    0.0%    0.0%    0.0%    0.0%    0.0%    0.0%    0.0%    0.0%    0.0%    0.0%    0.0%    0.0%    0.0%    0.0%    0.0%    0.0%    0.0%    0.0%    0.0%    0.0%    0.0%    0.0%    0.0%    0.0%    0.0%    0.0%    0.0%    0.0%    0.0%    0.0%    0.0%    0.0%    0.0%    0.0%    0.0%    0.0%    0.0%    0.0%    0.0%    0.0%    0.0%    0.0%    0.0%    0.0%    0.0%    0.0%    0.0%    0.0%    0.0%    0.0%    0.0%    0.0%    0.0%    0.0%        k__Bacteria;p__Cyanobacteria; c__SM1D11       0    0.0%    0.0%    0.0%    0.0%    0.0%    0.0%    0.0%    0.0%    0.0%    0.0%    0.0%    0.0%    0.0%    0.0%    0.0%    0.0%    0.0%    0.0%    0.0%    0.0%    0.0%    0.0%    0.0%    0.0%    0.0%    0.0%    0.0%    0.0%    0.0%    0.0%    0.0%    0.0%    0.0%    0.0%    0.0%    0.0%    0.0%    0.0%    0.0%    0.0%    0.0%    0.0%    0.0%    0.0%    0.0%    0.0%    0.0%    0.0%    0.0%    0.0%    0.0%    0.0%    0.0%    0.0%    0.0%    0.0%    0.0%    0.0%    0.0%    0.0%    0.0%    0.0%    0.0%    0.0%    0.0%    0.0%    0.0%    0.0%    0.0%    0.0%    0.0%    0.0%    0.0%    0.0%    0.0%    0.0%    0.0%    0.0%    0.0%    0.0%    0.0%    0.0%    0.0%    0.0%    0.0%    0.0%    0.0%    0.0%    0.0%    0.0%    0.0%    0.0%    0.0%    0.0%    0.0%    0.0%    0.0%    0.0%    0.0%    0.0%    0.0%    0.0%    0.0%    0.0%    0.0%    0.0%    0.0%    0.0%    0.0%    0.0%    0.0%    0.0%    0.0%    0.0%    0.0%    0.0%    0.0%    0.0%    0.0%    0.0%    0.0%    0.0%    0.0%    0.0%    0.0%    0.0%    0.0%    0.0%    0.0%    0.0%    0.0%    0.0%    0.0%    0.0%        k__Bacteria;p__Cyanobacteria; c__YS2      10    0.0%    0.0%    0.0%    0.0%    0.0%    0.0%    0.0%    0.0%    0.0%    0.0%    0.0%    0.0%    0.0%    0.0%    0.0%    0.0%    0.0%    0.0%    0.0%    0.0%    0.0%    0.0%    0.0%    0.0%    0.0%    0.0%    0.0%    0.0%    0.0%    0.0%    0.0%    0.0%    0.0%    0.0%    0.0%    0.0%    0.0%    0.0%    0.0%    0.0%    0.0%    0.0%    0.0%    0.0%    0.0%    0.0%    0.0%    0.0%    0.0%    0.0%    0.0%    0.0%    0.0%    0.0%    0.0%    0.0%    0.0%    0.0%    0.0%    0.0%    0.0%    0.0%    0.0%    0.0%    0.0%    0.0%    0.0%    0.0%    0.0%    0.0%    0.0%    0.0%    0.0%    0.0%    0.0%    0.0%    0.0%    0.0%    0.0%    0.0%    0.0%    0.0%    0.0%    0.0%    0.0%    0.0%    0.0%    0.0%    0.0%    0.0%    0.0%    0.0%    0.0%    0.0%    0.0%    0.0%    0.0%    0.0%    0.0%    0.0%    0.0%    0.0%    0.0%    0.0%    0.0%    0.0%    0.0%    0.0%    0.0%    0.0%    0.0%    0.0%    0.0%    0.0%    0.0%    0.0%    0.0%    0.0%    0.0%    0.0%    0.0%    0.0%    0.0%    0.0%    0.0%    0.0%    0.0%    0.0%    0.0%    0.0%    0.0%    0.0%    0.0%    0.0%        k__Bacteria;p__Cyanobacteria; c__mle1-12       3    0.0%    0.0%    0.0%    0.0%    0.0%    0.0%    0.0%    0.0%    0.0%    0.0%    0.0%    0.0%    0.0%    0.0%    0.0%    0.0%    0.0%    0.0%    0.0%    0.0%    0.0%    0.0%    0.0%    0.0%    0.0%    0.0%    0.0%    0.0%    0.0%    0.0%    0.0%    0.0%    0.0%    0.0%    0.0%    0.0%    0.0%    0.0%    0.0%    0.0%    0.0%    0.0%    0.0%    0.0%    0.0%    0.0%    0.0%    0.0%    0.0%    0.0%    0.0%    0.0%    0.0%    0.0%    0.0%    0.0%    0.0%    0.0%    0.0%    0.0%    0.0%    0.0%    0.0%    0.0%    0.0%    0.0%    0.0%    0.0%    0.0%    0.0%    0.0%    0.0%    0.0%    0.0%    0.0%    0.0%    0.0%    0.0%    0.0%    0.0%    0.0%    0.0%    0.0%    0.0%    0.0%    0.0%    0.0%    0.0%    0.0%    0.0%    0.0%    0.0%    0.0%    0.0%    0.0%    0.0%    0.0%    0.0%    0.0%    0.0%    0.0%    0.0%    0.0%    0.0%    0.0%    0.0%    0.0%    0.0%    0.0%    0.0%    0.0%    0.0%    0.0%    0.0%    0.0%    0.0%    0.0%    0.0%    0.0%    0.0%    0.0%    0.0%    0.0%    0.0%    0.0%    0.0%    0.0%    0.0%    0.0%    0.0%    0.0%    0.0%    0.0%    0.0%        k__Bacteria;p__Deferribacteres; c__Deferribacteres (class)      28    0.0%    0.0%    0.0%    0.0%    0.0%    0.0%    0.0%    0.0%    0.0%    0.0%    0.0%    0.0%    0.0%    0.0%    0.0%    0.0%    0.0%    0.0%    0.0%    0.0%    0.0%    0.0%    0.0%    0.0%    0.0%    0.0%    0.0%    0.0%    0.0%    0.0%    0.0%    0.0%    0.0%    0.0%    0.0%    0.0%    0.0%    0.0%    0.0%    0.0%    0.0%    0.0%    0.0%    0.0%    0.0%    0.0%    0.0%    0.0%    0.0%    0.0%    0.0%    0.0%    0.0%    0.0%    0.0%    0.0%    0.0%    0.0%    0.0%    0.1%    0.0%    0.0%    0.0%    0.0%    0.0%    0.0%    0.0%    0.0%    0.0%    0.0%    0.0%    0.0%    0.0%    0.0%    0.0%    0.0%    0.0%    0.0%    0.0%    0.0%    0.0%    0.0%    0.0%    0.0%    0.0%    0.0%    0.0%    0.0%    0.0%    0.0%    0.0%    0.0%    0.0%    0.0%    0.0%    0.0%    0.0%    0.0%    0.0%    0.0%    0.0%    0.0%    0.0%    0.0%    0.0%    0.0%    0.0%    0.0%    0.0%    0.0%    0.0%    0.0%    0.0%    0.0%    0.0%    0.0%    0.0%    0.0%    0.0%    0.0%    0.0%    0.0%    0.0%    0.0%    0.0%    0.0%    0.0%    0.0%    0.0%    0.0%    0.0%    0.0%    0.0%    0.0%        k__Bacteria;p__Elusimicrobia; c__Elusimicrobia (class)       1    0.0%    0.0%    0.0%    0.0%    0.0%    0.0%    0.0%    0.0%    0.0%    0.0%    0.0%    0.0%    0.0%    0.0%    0.0%    0.0%    0.0%    0.0%    0.0%    0.0%    0.0%    0.0%    0.0%    0.0%    0.0%    0.0%    0.0%    0.0%    0.0%    0.0%    0.0%    0.0%    0.0%    0.0%    0.0%    0.0%    0.0%    0.0%    0.0%    0.0%    0.0%    0.0%    0.0%    0.0%    0.0%    0.0%    0.0%    0.0%    0.0%    0.0%    0.0%    0.0%    0.0%    0.0%    0.0%    0.0%    0.0%    0.0%    0.0%    0.0%    0.0%    0.0%    0.0%    0.0%    0.0%    0.0%    0.0%    0.0%    0.0%    0.0%    0.0%    0.0%    0.0%    0.0%    0.0%    0.0%    0.0%    0.0%    0.0%    0.0%    0.0%    0.0%    0.0%    0.0%    0.0%    0.0%    0.0%    0.0%    0.0%    0.0%    0.0%    0.0%    0.0%    0.0%    0.0%    0.0%    0.0%    0.0%    0.0%    0.0%    0.0%    0.0%    0.0%    0.0%    0.0%    0.0%    0.0%    0.0%    0.0%    0.0%    0.0%    0.0%    0.0%    0.0%    0.0%    0.0%    0.0%    0.0%    0.0%    0.0%    0.0%    0.0%    0.0%    0.0%    0.0%    0.0%    0.0%    0.0%    0.0%    0.0%    0.0%    0.0%    0.0%    0.0%        k__Bacteria;p__Fibrobacteres; c__Fibrobacteres (class)       0    0.0%    0.0%    0.0%    0.0%    0.0%    0.0%    0.0%    0.0%    0.0%    0.0%    0.0%    0.0%    0.0%    0.0%    0.0%    0.0%    0.0%    0.0%    0.0%    0.0%    0.0%    0.0%    0.0%    0.0%    0.0%    0.0%    0.0%    0.0%    0.0%    0.0%    0.0%    0.0%    0.0%    0.0%    0.0%    0.0%    0.0%    0.0%    0.0%    0.0%    0.0%    0.0%    0.0%    0.0%    0.0%    0.0%    0.0%    0.0%    0.0%    0.0%    0.0%    0.0%    0.0%    0.0%    0.0%    0.0%    0.0%    0.0%    0.0%    0.0%    0.0%    0.0%    0.0%    0.0%    0.0%    0.0%    0.0%    0.0%    0.0%    0.0%    0.0%    0.0%    0.0%    0.0%    0.0%    0.0%    0.0%    0.0%    0.0%    0.0%    0.0%    0.0%    0.0%    0.0%    0.0%    0.0%    0.0%    0.0%    0.0%    0.0%    0.0%    0.0%    0.0%    0.0%    0.0%    0.0%    0.0%    0.0%    0.0%    0.0%    0.0%    0.0%    0.0%    0.0%    0.0%    0.0%    0.0%    0.0%    0.0%    0.0%    0.0%    0.0%    0.0%    0.0%    0.0%    0.0%    0.0%    0.0%    0.0%    0.0%    0.0%    0.0%    0.0%    0.0%    0.0%    0.0%    0.0%    0.0%    0.0%    0.0%    0.0%    0.0%    0.0%    0.0%        k__Bacteria;p__Firmicutes; c__Bacilli   227613   23.4%   18.3%   10.3%   30.8%   39.2%   26.7%   27.9%   47.5%   43.1%   22.6%   33.7%   13.6%   49.1%   34.6%   27.8%   30.1%   19.6%   22.2%   22.3%   24.2%   15.5%   18.8%   17.2%   24.7%   23.5%   15.2%   28.1%   28.4%   23.5%   22.5%   28.5%   24.1%   15.2%   29.4%   19.3%   28.5%   26.3%   17.6%   19.2%   19.6%   16.8%   20.8%   15.9%   13.2%   19.7%   30.9%   18.9%   24.9%   14.9%   18.9%   26.1%   27.0%   23.8%   20.2%   17.4%   15.3%   23.1%   12.6%   19.1%   13.0%   22.6%   36.3%   59.0%   54.9%   22.3%   32.1%   22.7%   20.5%   17.0%   23.0%   31.6%   32.7%   19.3%   18.8%   24.0%   45.3%   37.1%   16.5%   21.8%   19.9%   24.4%   27.8%   21.3%   20.3%   20.5%   22.1%   31.7%   18.8%   18.5%   36.5%   14.5%   19.2%   22.3%   15.4%   10.5%   18.5%   12.0%   13.4%   22.5%   25.4%   28.8%   23.8%   18.6%   19.7%   18.1%   16.1%   20.4%   34.2%   17.0%   12.4%   22.5%   22.2%   24.5%   27.4%   11.6%   21.5%    4.5%    6.5%   14.6%   42.2%   23.6%   23.6%   35.3%   12.1%   11.4%   15.6%   19.8%   17.6%   17.5%    7.1%   28.1%   20.0%   29.5%   26.9%        k__Bacteria;p__Firmicutes; c__Clostridia   78282    8.0%    8.6%    5.3%   10.7%    4.9%    5.8%    5.9%    7.1%    4.6%    2.5%    7.9%    7.7%    9.1%    8.6%   11.8%    9.6%    5.6%   13.8%    8.3%   12.4%    6.2%   14.5%   10.7%   17.7%   11.1%    4.8%    2.5%    7.3%    8.8%   13.0%   21.8%    8.9%    7.3%   10.7%   11.4%    8.0%   14.0%    4.1%    4.5%    7.1%   10.6%    9.6%    3.6%    7.1%    6.5%    7.8%   11.1%    4.6%    6.1%    5.8%   18.0%    9.4%   12.4%   10.2%   11.4%    6.8%    4.7%    6.3%    4.2%   11.3%   16.6%    8.6%    5.2%    6.7%   11.4%   12.1%    7.9%    4.1%   12.6%    6.8%   12.4%    9.6%    8.4%    8.0%    7.4%    8.8%    9.7%    2.8%    3.6%   11.9%    6.6%    4.1%    8.2%    8.7%    8.1%    6.7%    6.4%   12.9%    6.2%    6.4%    4.8%   19.7%   15.3%    8.0%    8.5%   10.0%   17.9%   15.5%    8.1%    4.5%    3.1%    9.1%   14.2%    6.5%    7.4%    4.5%   11.0%    3.0%    6.9%    2.4%    6.7%    4.4%   12.2%    2.6%    3.6%    5.8%    2.7%    1.6%    2.7%    4.4%    5.1%    5.6%    6.0%    3.7%    4.3%    4.1%    3.6%    8.8%    8.4%    1.8%    3.2%    3.4%    2.8%    3.4%        k__Bacteria;p__Fusobacteria; c__Fusobacteria (class)   29286    3.0%    2.7%    1.6%    1.3%    1.6%    1.9%    1.0%    0.7%    0.8%    0.3%    1.1%    2.9%    0.8%    1.4%    2.2%    1.3%    0.7%    1.4%    1.4%    0.9%    1.1%    2.3%   16.9%    2.0%    3.2%    2.4%    0.7%    1.3%    4.3%    2.0%    1.5%    1.6%    1.2%    0.4%    1.1%    0.7%    1.2%    8.9%    9.6%    5.3%    1.4%    4.2%    8.1%   21.9%   14.0%    2.3%    9.8%   15.0%   11.4%    6.6%    3.4%    1.5%    7.5%    7.3%    4.8%    7.1%    3.0%    0.8%    8.0%    6.9%    5.5%    3.5%    0.7%    1.4%    8.8%    1.7%    2.2%   21.8%    4.4%    3.0%    1.1%    3.2%    2.8%    5.3%    2.2%    1.2%    1.2%    1.1%    2.8%    3.4%    2.5%    1.0%    1.1%    1.8%    2.4%    1.8%    2.2%    5.6%    3.7%    1.2%    1.3%    3.8%    2.6%    1.8%    2.4%    2.4%    8.1%    4.7%    1.8%    1.8%    0.7%    2.5%    0.9%    2.0%    1.1%    0.4%    1.0%    0.8%    0.7%    0.2%    0.8%    1.7%    2.4%    1.0%    0.3%    1.1%    0.3%    0.2%    0.6%    1.0%    1.0%    1.0%    1.7%    1.4%    1.6%    1.5%    0.9%    1.6%    1.5%    0.6%    1.2%    1.3%    1.1%    1.0%        k__Bacteria;p__GN02; c__       0    0.0%    0.0%    0.0%    0.0%    0.0%    0.0%    0.0%    0.0%    0.0%    0.0%    0.0%    0.0%    0.0%    0.0%    0.0%    0.0%    0.0%    0.0%    0.0%    0.0%    0.0%    0.0%    0.0%    0.0%    0.0%    0.0%    0.0%    0.0%    0.0%    0.0%    0.0%    0.0%    0.0%    0.0%    0.0%    0.0%    0.0%    0.0%    0.0%    0.0%    0.0%    0.0%    0.0%    0.0%    0.0%    0.0%    0.0%    0.0%    0.0%    0.0%    0.0%    0.0%    0.0%    0.0%    0.0%    0.0%    0.0%    0.0%    0.0%    0.0%    0.0%    0.0%    0.0%    0.0%    0.0%    0.0%    0.0%    0.0%    0.0%    0.0%    0.0%    0.0%    0.0%    0.0%    0.0%    0.0%    0.0%    0.0%    0.0%    0.0%    0.0%    0.0%    0.0%    0.0%    0.0%    0.0%    0.0%    0.0%    0.0%    0.0%    0.0%    0.0%    0.0%    0.0%    0.0%    0.0%    0.0%    0.0%    0.0%    0.0%    0.0%    0.0%    0.0%    0.0%    0.0%    0.0%    0.0%    0.0%    0.0%    0.0%    0.0%    0.0%    0.0%    0.0%    0.0%    0.0%    0.0%    0.0%    0.0%    0.0%    0.0%    0.0%    0.0%    0.0%    0.0%    0.0%    0.0%    0.0%    0.0%    0.0%    0.0%    0.0%    0.0%    0.0%        k__Bacteria;p__GN02; c__VC12-cl04       0    0.0%    0.0%    0.0%    0.0%    0.0%    0.0%    0.0%    0.0%    0.0%    0.0%    0.0%    0.0%    0.0%    0.0%    0.0%    0.0%    0.0%    0.0%    0.0%    0.0%    0.0%    0.0%    0.0%    0.0%    0.0%    0.0%    0.0%    0.0%    0.0%    0.0%    0.0%    0.0%    0.0%    0.0%    0.0%    0.0%    0.0%    0.0%    0.0%    0.0%    0.0%    0.0%    0.0%    0.0%    0.0%    0.0%    0.0%    0.0%    0.0%    0.0%    0.0%    0.0%    0.0%    0.0%    0.0%    0.0%    0.0%    0.0%    0.0%    0.0%    0.0%    0.0%    0.0%    0.0%    0.0%    0.0%    0.0%    0.0%    0.0%    0.0%    0.0%    0.0%    0.0%    0.0%    0.0%    0.0%    0.0%    0.0%    0.0%    0.0%    0.0%    0.0%    0.0%    0.0%    0.0%    0.0%    0.0%    0.0%    0.0%    0.0%    0.0%    0.0%    0.0%    0.0%    0.0%    0.0%    0.0%    0.0%    0.0%    0.0%    0.0%    0.0%    0.0%    0.0%    0.0%    0.0%    0.0%    0.0%    0.0%    0.0%    0.0%    0.0%    0.0%    0.0%    0.0%    0.0%    0.0%    0.0%    0.0%    0.0%    0.0%    0.0%    0.0%    0.0%    0.0%    0.0%    0.0%    0.0%    0.0%    0.0%    0.0%    0.0%    0.0%    0.0%        k__Bacteria;p__Gemmatimonadetes; c__Gemmatimonadetes (class)     510    0.1%    0.1%    0.0%    0.1%    0.1%    0.2%    0.1%    0.0%    0.0%    0.0%    0.1%    0.0%    0.0%    0.0%    0.0%    0.0%    0.1%    0.0%    0.1%    0.0%    0.0%    0.1%    0.0%    0.0%    0.1%    0.0%    0.1%    0.1%    0.0%    0.0%    0.0%    0.0%    0.0%    0.0%    0.0%    0.0%    0.0%    0.1%    0.0%    0.0%    0.0%    0.0%    0.0%    0.0%    0.0%    0.1%    0.0%    0.0%    0.0%    0.0%    0.0%    0.0%    0.0%    0.1%    0.0%    0.1%    0.0%    0.0%    0.1%    0.0%    0.1%    0.0%    0.0%    0.0%    0.1%    0.0%    0.2%    0.0%    0.0%    0.1%    0.0%    0.0%    0.1%    0.0%    0.1%    0.0%    0.0%    0.2%    0.0%    0.0%    0.1%    0.1%    0.0%    0.1%    0.1%    0.0%    0.1%    0.1%    0.1%    0.1%    0.0%    0.0%    0.0%    0.1%    0.1%    0.1%    0.1%    0.1%    0.1%    0.1%    0.0%    0.1%    0.0%    0.2%    0.1%    0.0%    0.0%    0.1%    0.2%    0.1%    0.1%    0.1%    0.1%    0.0%    0.0%    0.2%    0.0%    0.0%    0.0%    0.0%    0.0%    0.0%    0.0%    0.1%    0.1%    0.1%    0.0%    0.1%    0.1%    0.1%    0.0%    0.0%    0.0%    0.1%        k__Bacteria;p__Lentisphaerae; c__       0    0.0%    0.0%    0.0%    0.0%    0.0%    0.0%    0.0%    0.0%    0.0%    0.0%    0.0%    0.0%    0.0%    0.0%    0.0%    0.0%    0.0%    0.0%    0.0%    0.0%    0.0%    0.0%    0.0%    0.0%    0.0%    0.0%    0.0%    0.0%    0.0%    0.0%    0.0%    0.0%    0.0%    0.0%    0.0%    0.0%    0.0%    0.0%    0.0%    0.0%    0.0%    0.0%    0.0%    0.0%    0.0%    0.0%    0.0%    0.0%    0.0%    0.0%    0.0%    0.0%    0.0%    0.0%    0.0%    0.0%    0.0%    0.0%    0.0%    0.0%    0.0%    0.0%    0.0%    0.0%    0.0%    0.0%    0.0%    0.0%    0.0%    0.0%    0.0%    0.0%    0.0%    0.0%    0.0%    0.0%    0.0%    0.0%    0.0%    0.0%    0.0%    0.0%    0.0%    0.0%    0.0%    0.0%    0.0%    0.0%    0.0%    0.0%    0.0%    0.0%    0.0%    0.0%    0.0%    0.0%    0.0%    0.0%    0.0%    0.0%    0.0%    0.0%    0.0%    0.0%    0.0%    0.0%    0.0%    0.0%    0.0%    0.0%    0.0%    0.0%    0.0%    0.0%    0.0%    0.0%    0.0%    0.0%    0.0%    0.0%    0.0%    0.0%    0.0%    0.0%    0.0%    0.0%    0.0%    0.0%    0.0%    0.0%    0.0%    0.0%    0.0%    0.0%        k__Bacteria;p__Lentisphaerae; c__Lentisphaerae (class)       0    0.0%    0.0%    0.0%    0.0%    0.0%    0.0%    0.0%    0.0%    0.0%    0.0%    0.0%    0.0%    0.0%    0.0%    0.0%    0.0%    0.0%    0.0%    0.0%    0.0%    0.0%    0.0%    0.0%    0.0%    0.0%    0.0%    0.0%    0.0%    0.0%    0.0%    0.0%    0.0%    0.0%    0.0%    0.0%    0.0%    0.0%    0.0%    0.0%    0.0%    0.0%    0.0%    0.0%    0.0%    0.0%    0.0%    0.0%    0.0%    0.0%    0.0%    0.0%    0.0%    0.0%    0.0%    0.0%    0.0%    0.0%    0.0%    0.0%    0.0%    0.0%    0.0%    0.0%    0.0%    0.0%    0.0%    0.0%    0.0%    0.0%    0.0%    0.0%    0.0%    0.0%    0.0%    0.0%    0.0%    0.0%    0.0%    0.0%    0.0%    0.0%    0.0%    0.0%    0.0%    0.0%    0.0%    0.0%    0.0%    0.0%    0.0%    0.0%    0.0%    0.0%    0.0%    0.0%    0.0%    0.0%    0.0%    0.0%    0.0%    0.0%    0.0%    0.0%    0.0%    0.0%    0.0%    0.0%    0.0%    0.0%    0.0%    0.0%    0.0%    0.0%    0.0%    0.0%    0.0%    0.0%    0.0%    0.0%    0.0%    0.0%    0.0%    0.0%    0.0%    0.0%    0.0%    0.0%    0.0%    0.0%    0.0%    0.0%    0.0%    0.0%    0.0%        k__Bacteria;p__MVP-15; c__       0    0.0%    0.0%    0.0%    0.0%    0.0%    0.0%    0.0%    0.0%    0.0%    0.0%    0.0%    0.0%    0.0%    0.0%    0.0%    0.0%    0.0%    0.0%    0.0%    0.0%    0.0%    0.0%    0.0%    0.0%    0.0%    0.0%    0.0%    0.0%    0.0%    0.0%    0.0%    0.0%    0.0%    0.0%    0.0%    0.0%    0.0%    0.0%    0.0%    0.0%    0.0%    0.0%    0.0%    0.0%    0.0%    0.0%    0.0%    0.0%    0.0%    0.0%    0.0%    0.0%    0.0%    0.0%    0.0%    0.0%    0.0%    0.0%    0.0%    0.0%    0.0%    0.0%    0.0%    0.0%    0.0%    0.0%    0.0%    0.0%    0.0%    0.0%    0.0%    0.0%    0.0%    0.0%    0.0%    0.0%    0.0%    0.0%    0.0%    0.0%    0.0%    0.0%    0.0%    0.0%    0.0%    0.0%    0.0%    0.0%    0.0%    0.0%    0.0%    0.0%    0.0%    0.0%    0.0%    0.0%    0.0%    0.0%    0.0%    0.0%    0.0%    0.0%    0.0%    0.0%    0.0%    0.0%    0.0%    0.0%    0.0%    0.0%    0.0%    0.0%    0.0%    0.0%    0.0%    0.0%    0.0%    0.0%    0.0%    0.0%    0.0%    0.0%    0.0%    0.0%    0.0%    0.0%    0.0%    0.0%    0.0%    0.0%    0.0%    0.0%    0.0%    0.0%        k__Bacteria;p__NC10; c__       0    0.0%    0.0%    0.0%    0.0%    0.0%    0.0%    0.0%    0.0%    0.0%    0.0%    0.0%    0.0%    0.0%    0.0%    0.0%    0.0%    0.0%    0.0%    0.0%    0.0%    0.0%    0.0%    0.0%    0.0%    0.0%    0.0%    0.0%    0.0%    0.0%    0.0%    0.0%    0.0%    0.0%    0.0%    0.0%    0.0%    0.0%    0.0%    0.0%    0.0%    0.0%    0.0%    0.0%    0.0%    0.0%    0.0%    0.0%    0.0%    0.0%    0.0%    0.0%    0.0%    0.0%    0.0%    0.0%    0.0%    0.0%    0.0%    0.0%    0.0%    0.0%    0.0%    0.0%    0.0%    0.0%    0.0%    0.0%    0.0%    0.0%    0.0%    0.0%    0.0%    0.0%    0.0%    0.0%    0.0%    0.0%    0.0%    0.0%    0.0%    0.0%    0.0%    0.0%    0.0%    0.0%    0.0%    0.0%    0.0%    0.0%    0.0%    0.0%    0.0%    0.0%    0.0%    0.0%    0.0%    0.0%    0.0%    0.0%    0.0%    0.0%    0.0%    0.0%    0.0%    0.0%    0.0%    0.0%    0.0%    0.0%    0.0%    0.0%    0.0%    0.0%    0.0%    0.0%    0.0%    0.0%    0.0%    0.0%    0.0%    0.0%    0.0%    0.0%    0.0%    0.0%    0.0%    0.0%    0.0%    0.0%    0.0%    0.0%    0.0%    0.0%    0.0%        k__Bacteria;p__NKB19; c__       1    0.0%    0.0%    0.0%    0.0%    0.0%    0.0%    0.0%    0.0%    0.0%    0.0%    0.0%    0.0%    0.0%    0.0%    0.0%    0.0%    0.0%    0.0%    0.0%    0.0%    0.0%    0.0%    0.0%    0.0%    0.0%    0.0%    0.0%    0.0%    0.0%    0.0%    0.0%    0.0%    0.0%    0.0%    0.0%    0.0%    0.0%    0.0%    0.0%    0.0%    0.0%    0.0%    0.0%    0.0%    0.0%    0.0%    0.0%    0.0%    0.0%    0.0%    0.0%    0.0%    0.0%    0.0%    0.0%    0.0%    0.0%    0.0%    0.0%    0.0%    0.0%    0.0%    0.0%    0.0%    0.0%    0.0%    0.0%    0.0%    0.0%    0.0%    0.0%    0.0%    0.0%    0.0%    0.0%    0.0%    0.0%    0.0%    0.0%    0.0%    0.0%    0.0%    0.0%    0.0%    0.0%    0.0%    0.0%    0.0%    0.0%    0.0%    0.0%    0.0%    0.0%    0.0%    0.0%    0.0%    0.0%    0.0%    0.0%    0.0%    0.0%    0.0%    0.0%    0.0%    0.0%    0.0%    0.0%    0.0%    0.0%    0.0%    0.0%    0.0%    0.0%    0.0%    0.0%    0.0%    0.0%    0.0%    0.0%    0.0%    0.0%    0.0%    0.0%    0.0%    0.0%    0.0%    0.0%    0.0%    0.0%    0.0%    0.0%    0.0%    0.0%    0.0%        k__Bacteria;p__Nitrospirae; c__Nitrospira (class)      99    0.0%    0.0%    0.0%    0.0%    0.0%    0.1%    0.0%    0.0%    0.0%    0.0%    0.0%    0.0%    0.0%    0.0%    0.0%    0.0%    0.0%    0.0%    0.0%    0.0%    0.0%    0.0%    0.0%    0.0%    0.0%    0.0%    0.0%    0.0%    0.0%    0.0%    0.0%    0.0%    0.0%    0.0%    0.0%    0.0%    0.0%    0.0%    0.0%    0.0%    0.0%    0.0%    0.0%    0.0%    0.0%    0.0%    0.0%    0.0%    0.0%    0.0%    0.0%    0.0%    0.0%    0.0%    0.0%    0.0%    0.0%    0.0%    0.0%    0.0%    0.1%    0.0%    0.0%    0.0%    0.0%    0.0%    0.0%    0.0%    0.0%    0.1%    0.0%    0.0%    0.0%    0.0%    0.0%    0.0%    0.0%    0.0%    0.0%    0.0%    0.0%    0.0%    0.0%    0.0%    0.0%    0.0%    0.0%    0.0%    0.0%    0.0%    0.0%    0.0%    0.0%    0.0%    0.0%    0.0%    0.0%    0.0%    0.1%    0.0%    0.0%    0.0%    0.0%    0.0%    0.0%    0.0%    0.0%    0.0%    0.1%    0.0%    0.0%    0.0%    0.0%    0.0%    0.0%    0.0%    0.0%    0.0%    0.0%    0.0%    0.0%    0.0%    0.0%    0.0%    0.0%    0.0%    0.0%    0.0%    0.0%    0.0%    0.0%    0.0%    0.0%    0.0%        k__Bacteria;p__OP10; c__       0    0.0%    0.0%    0.0%    0.0%    0.0%    0.0%    0.0%    0.0%    0.0%    0.0%    0.0%    0.0%    0.0%    0.0%    0.0%    0.0%    0.0%    0.0%    0.0%    0.0%    0.0%    0.0%    0.0%    0.0%    0.0%    0.0%    0.0%    0.0%    0.0%    0.0%    0.0%    0.0%    0.0%    0.0%    0.0%    0.0%    0.0%    0.0%    0.0%    0.0%    0.0%    0.0%    0.0%    0.0%    0.0%    0.0%    0.0%    0.0%    0.0%    0.0%    0.0%    0.0%    0.0%    0.0%    0.0%    0.0%    0.0%    0.0%    0.0%    0.0%    0.0%    0.0%    0.0%    0.0%    0.0%    0.0%    0.0%    0.0%    0.0%    0.0%    0.0%    0.0%    0.0%    0.0%    0.0%    0.0%    0.0%    0.0%    0.0%    0.0%    0.0%    0.0%    0.0%    0.0%    0.0%    0.0%    0.0%    0.0%    0.0%    0.0%    0.0%    0.0%    0.0%    0.0%    0.0%    0.0%    0.0%    0.0%    0.0%    0.0%    0.0%    0.0%    0.0%    0.0%    0.0%    0.0%    0.0%    0.0%    0.0%    0.0%    0.0%    0.0%    0.0%    0.0%    0.0%    0.0%    0.0%    0.0%    0.0%    0.0%    0.0%    0.0%    0.0%    0.0%    0.0%    0.0%    0.0%    0.0%    0.0%    0.0%    0.0%    0.0%    0.0%    0.0%        k__Bacteria;p__OP10; c__5B-18       8    0.0%    0.1%    0.0%    0.0%    0.0%    0.0%    0.0%    0.0%    0.0%    0.0%    0.0%    0.0%    0.0%    0.0%    0.0%    0.0%    0.0%    0.0%    0.0%    0.0%    0.0%    0.0%    0.0%    0.0%    0.0%    0.0%    0.0%    0.0%    0.0%    0.0%    0.0%    0.0%    0.0%    0.0%    0.0%    0.0%    0.0%    0.0%    0.0%    0.0%    0.0%    0.0%    0.0%    0.0%    0.0%    0.0%    0.0%    0.0%    0.0%    0.0%    0.0%    0.0%    0.0%    0.0%    0.0%    0.0%    0.0%    0.0%    0.0%    0.0%    0.0%    0.0%    0.0%    0.0%    0.0%    0.0%    0.0%    0.0%    0.0%    0.0%    0.0%    0.0%    0.0%    0.0%    0.0%    0.0%    0.0%    0.0%    0.0%    0.0%    0.0%    0.0%    0.0%    0.0%    0.0%    0.0%    0.0%    0.0%    0.0%    0.0%    0.0%    0.0%    0.0%    0.0%    0.0%    0.0%    0.0%    0.0%    0.0%    0.0%    0.0%    0.0%    0.0%    0.0%    0.0%    0.0%    0.0%    0.0%    0.0%    0.0%    0.0%    0.0%    0.0%    0.0%    0.0%    0.0%    0.0%    0.0%    0.0%    0.0%    0.0%    0.0%    0.0%    0.0%    0.0%    0.0%    0.0%    0.0%    0.0%    0.0%    0.0%    0.0%    0.0%    0.0%        k__Bacteria;p__OP10; c__CH21       2    0.0%    0.0%    0.0%    0.0%    0.0%    0.0%    0.0%    0.0%    0.0%    0.0%    0.0%    0.0%    0.0%    0.0%    0.0%    0.0%    0.0%    0.0%    0.0%    0.0%    0.0%    0.0%    0.0%    0.0%    0.0%    0.0%    0.0%    0.0%    0.0%    0.0%    0.0%    0.0%    0.0%    0.0%    0.0%    0.0%    0.0%    0.0%    0.0%    0.0%    0.0%    0.0%    0.0%    0.0%    0.0%    0.0%    0.0%    0.0%    0.0%    0.0%    0.0%    0.0%    0.0%    0.0%    0.0%    0.0%    0.0%    0.0%    0.0%    0.0%    0.0%    0.0%    0.0%    0.0%    0.0%    0.0%    0.0%    0.0%    0.0%    0.0%    0.0%    0.0%    0.0%    0.0%    0.0%    0.0%    0.0%    0.0%    0.0%    0.0%    0.0%    0.0%    0.0%    0.0%    0.0%    0.0%    0.0%    0.0%    0.0%    0.0%    0.0%    0.0%    0.0%    0.0%    0.0%    0.0%    0.0%    0.0%    0.0%    0.0%    0.0%    0.0%    0.0%    0.0%    0.0%    0.0%    0.0%    0.0%    0.0%    0.0%    0.0%    0.0%    0.0%    0.0%    0.0%    0.0%    0.0%    0.0%    0.0%    0.0%    0.0%    0.0%    0.0%    0.0%    0.0%    0.0%    0.0%    0.0%    0.0%    0.0%    0.0%    0.0%    0.0%    0.0%        k__Bacteria;p__OP10; c__CL500-48      36    0.0%    0.0%    0.0%    0.0%    0.0%    0.0%    0.0%    0.0%    0.0%    0.0%    0.0%    0.0%    0.0%    0.0%    0.0%    0.0%    0.0%    0.0%    0.0%    0.0%    0.0%    0.0%    0.0%    0.0%    0.0%    0.0%    0.0%    0.0%    0.0%    0.0%    0.0%    0.0%    0.0%    0.0%    0.0%    0.0%    0.0%    0.0%    0.0%    0.0%    0.0%    0.1%    0.0%    0.0%    0.0%    0.0%    0.0%    0.0%    0.0%    0.0%    0.0%    0.0%    0.0%    0.0%    0.0%    0.0%    0.0%    0.0%    0.0%    0.0%    0.0%    0.0%    0.0%    0.0%    0.0%    0.0%    0.0%    0.0%    0.0%    0.0%    0.0%    0.0%    0.0%    0.0%    0.0%    0.0%    0.0%    0.0%    0.0%    0.0%    0.0%    0.0%    0.0%    0.0%    0.0%    0.0%    0.0%    0.0%    0.0%    0.0%    0.0%    0.0%    0.0%    0.0%    0.0%    0.0%    0.0%    0.0%    0.0%    0.0%    0.0%    0.0%    0.0%    0.0%    0.0%    0.0%    0.0%    0.0%    0.0%    0.0%    0.0%    0.0%    0.0%    0.0%    0.0%    0.0%    0.0%    0.0%    0.0%    0.0%    0.0%    0.0%    0.0%    0.0%    0.0%    0.0%    0.0%    0.0%    0.0%    0.0%    0.0%    0.0%    0.0%    0.0%        k__Bacteria;p__OP10; c__OS-L       7    0.0%    0.0%    0.0%    0.0%    0.0%    0.0%    0.0%    0.0%    0.0%    0.0%    0.0%    0.0%    0.0%    0.0%    0.0%    0.0%    0.0%    0.0%    0.0%    0.0%    0.0%    0.0%    0.0%    0.0%    0.0%    0.0%    0.0%    0.0%    0.0%    0.0%    0.0%    0.0%    0.0%    0.0%    0.0%    0.0%    0.0%    0.0%    0.0%    0.0%    0.0%    0.0%    0.0%    0.0%    0.0%    0.0%    0.0%    0.0%    0.0%    0.0%    0.0%    0.0%    0.0%    0.0%    0.0%    0.0%    0.0%    0.0%    0.0%    0.0%    0.0%    0.0%    0.0%    0.0%    0.0%    0.0%    0.0%    0.0%    0.0%    0.0%    0.0%    0.0%    0.0%    0.0%    0.0%    0.0%    0.0%    0.0%    0.0%    0.0%    0.0%    0.0%    0.0%    0.0%    0.0%    0.0%    0.0%    0.0%    0.0%    0.0%    0.0%    0.0%    0.0%    0.0%    0.0%    0.0%    0.0%    0.0%    0.0%    0.0%    0.0%    0.0%    0.0%    0.0%    0.0%    0.0%    0.0%    0.0%    0.0%    0.0%    0.0%    0.0%    0.0%    0.0%    0.0%    0.0%    0.0%    0.0%    0.0%    0.0%    0.0%    0.0%    0.0%    0.0%    0.0%    0.0%    0.0%    0.0%    0.0%    0.0%    0.0%    0.0%    0.0%    0.0%        k__Bacteria;p__OP10; c__S1a-1H       2    0.0%    0.0%    0.0%    0.0%    0.0%    0.0%    0.0%    0.0%    0.0%    0.0%    0.0%    0.0%    0.0%    0.0%    0.0%    0.0%    0.0%    0.0%    0.0%    0.0%    0.0%    0.0%    0.0%    0.0%    0.0%    0.0%    0.0%    0.0%    0.0%    0.0%    0.0%    0.0%    0.0%    0.0%    0.0%    0.0%    0.0%    0.0%    0.0%    0.0%    0.0%    0.0%    0.0%    0.0%    0.0%    0.0%    0.0%    0.0%    0.0%    0.0%    0.0%    0.0%    0.0%    0.0%    0.0%    0.0%    0.0%    0.0%    0.0%    0.0%    0.0%    0.0%    0.0%    0.0%    0.0%    0.0%    0.0%    0.0%    0.0%    0.0%    0.0%    0.0%    0.0%    0.0%    0.0%    0.0%    0.0%    0.0%    0.0%    0.0%    0.0%    0.0%    0.0%    0.0%    0.0%    0.0%    0.0%    0.0%    0.0%    0.0%    0.0%    0.0%    0.0%    0.0%    0.0%    0.0%    0.0%    0.0%    0.0%    0.0%    0.0%    0.0%    0.0%    0.0%    0.0%    0.0%    0.0%    0.0%    0.0%    0.0%    0.0%    0.0%    0.0%    0.0%    0.0%    0.0%    0.0%    0.0%    0.0%    0.0%    0.0%    0.0%    0.0%    0.0%    0.0%    0.0%    0.0%    0.0%    0.0%    0.0%    0.0%    0.0%    0.0%    0.0%        k__Bacteria;p__OP10; c__SJA-176       0    0.0%    0.0%    0.0%    0.0%    0.0%    0.0%    0.0%    0.0%    0.0%    0.0%    0.0%    0.0%    0.0%    0.0%    0.0%    0.0%    0.0%    0.0%    0.0%    0.0%    0.0%    0.0%    0.0%    0.0%    0.0%    0.0%    0.0%    0.0%    0.0%    0.0%    0.0%    0.0%    0.0%    0.0%    0.0%    0.0%    0.0%    0.0%    0.0%    0.0%    0.0%    0.0%    0.0%    0.0%    0.0%    0.0%    0.0%    0.0%    0.0%    0.0%    0.0%    0.0%    0.0%    0.0%    0.0%    0.0%    0.0%    0.0%    0.0%    0.0%    0.0%    0.0%    0.0%    0.0%    0.0%    0.0%    0.0%    0.0%    0.0%    0.0%    0.0%    0.0%    0.0%    0.0%    0.0%    0.0%    0.0%    0.0%    0.0%    0.0%    0.0%    0.0%    0.0%    0.0%    0.0%    0.0%    0.0%    0.0%    0.0%    0.0%    0.0%    0.0%    0.0%    0.0%    0.0%    0.0%    0.0%    0.0%    0.0%    0.0%    0.0%    0.0%    0.0%    0.0%    0.0%    0.0%    0.0%    0.0%    0.0%    0.0%    0.0%    0.0%    0.0%    0.0%    0.0%    0.0%    0.0%    0.0%    0.0%    0.0%    0.0%    0.0%    0.0%    0.0%    0.0%    0.0%    0.0%    0.0%    0.0%    0.0%    0.0%    0.0%    0.0%    0.0%        k__Bacteria;p__OP10; c__SJA-22       0    0.0%    0.0%    0.0%    0.0%    0.0%    0.0%    0.0%    0.0%    0.0%    0.0%    0.0%    0.0%    0.0%    0.0%    0.0%    0.0%    0.0%    0.0%    0.0%    0.0%    0.0%    0.0%    0.0%    0.0%    0.0%    0.0%    0.0%    0.0%    0.0%    0.0%    0.0%    0.0%    0.0%    0.0%    0.0%    0.0%    0.0%    0.0%    0.0%    0.0%    0.0%    0.0%    0.0%    0.0%    0.0%    0.0%    0.0%    0.0%    0.0%    0.0%    0.0%    0.0%    0.0%    0.0%    0.0%    0.0%    0.0%    0.0%    0.0%    0.0%    0.0%    0.0%    0.0%    0.0%    0.0%    0.0%    0.0%    0.0%    0.0%    0.0%    0.0%    0.0%    0.0%    0.0%    0.0%    0.0%    0.0%    0.0%    0.0%    0.0%    0.0%    0.0%    0.0%    0.0%    0.0%    0.0%    0.0%    0.0%    0.0%    0.0%    0.0%    0.0%    0.0%    0.0%    0.0%    0.0%    0.0%    0.0%    0.0%    0.0%    0.0%    0.0%    0.0%    0.0%    0.0%    0.0%    0.0%    0.0%    0.0%    0.0%    0.0%    0.0%    0.0%    0.0%    0.0%    0.0%    0.0%    0.0%    0.0%    0.0%    0.0%    0.0%    0.0%    0.0%    0.0%    0.0%    0.0%    0.0%    0.0%    0.0%    0.0%    0.0%    0.0%    0.0%        k__Bacteria;p__OP11; c__       0    0.0%    0.0%    0.0%    0.0%    0.0%    0.0%    0.0%    0.0%    0.0%    0.0%    0.0%    0.0%    0.0%    0.0%    0.0%    0.0%    0.0%    0.0%    0.0%    0.0%    0.0%    0.0%    0.0%    0.0%    0.0%    0.0%    0.0%    0.0%    0.0%    0.0%    0.0%    0.0%    0.0%    0.0%    0.0%    0.0%    0.0%    0.0%    0.0%    0.0%    0.0%    0.0%    0.0%    0.0%    0.0%    0.0%    0.0%    0.0%    0.0%    0.0%    0.0%    0.0%    0.0%    0.0%    0.0%    0.0%    0.0%    0.0%    0.0%    0.0%    0.0%    0.0%    0.0%    0.0%    0.0%    0.0%    0.0%    0.0%    0.0%    0.0%    0.0%    0.0%    0.0%    0.0%    0.0%    0.0%    0.0%    0.0%    0.0%    0.0%    0.0%    0.0%    0.0%    0.0%    0.0%    0.0%    0.0%    0.0%    0.0%    0.0%    0.0%    0.0%    0.0%    0.0%    0.0%    0.0%    0.0%    0.0%    0.0%    0.0%    0.0%    0.0%    0.0%    0.0%    0.0%    0.0%    0.0%    0.0%    0.0%    0.0%    0.0%    0.0%    0.0%    0.0%    0.0%    0.0%    0.0%    0.0%    0.0%    0.0%    0.0%    0.0%    0.0%    0.0%    0.0%    0.0%    0.0%    0.0%    0.0%    0.0%    0.0%    0.0%    0.0%    0.0%        k__Bacteria;p__OP3; c__       1    0.0%    0.0%    0.0%    0.0%    0.0%    0.0%    0.0%    0.0%    0.0%    0.0%    0.0%    0.0%    0.0%    0.0%    0.0%    0.0%    0.0%    0.0%    0.0%    0.0%    0.0%    0.0%    0.0%    0.0%    0.0%    0.0%    0.0%    0.0%    0.0%    0.0%    0.0%    0.0%    0.0%    0.0%    0.0%    0.0%    0.0%    0.0%    0.0%    0.0%    0.0%    0.0%    0.0%    0.0%    0.0%    0.0%    0.0%    0.0%    0.0%    0.0%    0.0%    0.0%    0.0%    0.0%    0.0%    0.0%    0.0%    0.0%    0.0%    0.0%    0.0%    0.0%    0.0%    0.0%    0.0%    0.0%    0.0%    0.0%    0.0%    0.0%    0.0%    0.0%    0.0%    0.0%    0.0%    0.0%    0.0%    0.0%    0.0%    0.0%    0.0%    0.0%    0.0%    0.0%    0.0%    0.0%    0.0%    0.0%    0.0%    0.0%    0.0%    0.0%    0.0%    0.0%    0.0%    0.0%    0.0%    0.0%    0.0%    0.0%    0.0%    0.0%    0.0%    0.0%    0.0%    0.0%    0.0%    0.0%    0.0%    0.0%    0.0%    0.0%    0.0%    0.0%    0.0%    0.0%    0.0%    0.0%    0.0%    0.0%    0.0%    0.0%    0.0%    0.0%    0.0%    0.0%    0.0%    0.0%    0.0%    0.0%    0.0%    0.0%    0.0%    0.0%        k__Bacteria;p__OP8; c__OP8       0    0.0%    0.0%    0.0%    0.0%    0.0%    0.0%    0.0%    0.0%    0.0%    0.0%    0.0%    0.0%    0.0%    0.0%    0.0%    0.0%    0.0%    0.0%    0.0%    0.0%    0.0%    0.0%    0.0%    0.0%    0.0%    0.0%    0.0%    0.0%    0.0%    0.0%    0.0%    0.0%    0.0%    0.0%    0.0%    0.0%    0.0%    0.0%    0.0%    0.0%    0.0%    0.0%    0.0%    0.0%    0.0%    0.0%    0.0%    0.0%    0.0%    0.0%    0.0%    0.0%    0.0%    0.0%    0.0%    0.0%    0.0%    0.0%    0.0%    0.0%    0.0%    0.0%    0.0%    0.0%    0.0%    0.0%    0.0%    0.0%    0.0%    0.0%    0.0%    0.0%    0.0%    0.0%    0.0%    0.0%    0.0%    0.0%    0.0%    0.0%    0.0%    0.0%    0.0%    0.0%    0.0%    0.0%    0.0%    0.0%    0.0%    0.0%    0.0%    0.0%    0.0%    0.0%    0.0%    0.0%    0.0%    0.0%    0.0%    0.0%    0.0%    0.0%    0.0%    0.0%    0.0%    0.0%    0.0%    0.0%    0.0%    0.0%    0.0%    0.0%    0.0%    0.0%    0.0%    0.0%    0.0%    0.0%    0.0%    0.0%    0.0%    0.0%    0.0%    0.0%    0.0%    0.0%    0.0%    0.0%    0.0%    0.0%    0.0%    0.0%    0.0%    0.0%        k__Bacteria;p__Planctomycetes; c__FFCH393      15    0.0%    0.0%    0.0%    0.0%    0.0%    0.0%    0.0%    0.0%    0.0%    0.0%    0.0%    0.0%    0.0%    0.0%    0.0%    0.0%    0.0%    0.0%    0.0%    0.0%    0.0%    0.0%    0.0%    0.0%    0.0%    0.0%    0.0%    0.0%    0.0%    0.0%    0.0%    0.0%    0.0%    0.0%    0.0%    0.0%    0.0%    0.0%    0.0%    0.0%    0.0%    0.0%    0.0%    0.0%    0.0%    0.0%    0.0%    0.0%    0.0%    0.0%    0.0%    0.0%    0.0%    0.0%    0.0%    0.0%    0.0%    0.0%    0.0%    0.0%    0.0%    0.0%    0.0%    0.0%    0.0%    0.0%    0.0%    0.0%    0.0%    0.0%    0.0%    0.0%    0.0%    0.0%    0.0%    0.0%    0.0%    0.0%    0.0%    0.0%    0.0%    0.0%    0.0%    0.0%    0.0%    0.0%    0.0%    0.0%    0.0%    0.0%    0.0%    0.0%    0.0%    0.0%    0.0%    0.0%    0.0%    0.0%    0.0%    0.0%    0.0%    0.0%    0.0%    0.0%    0.0%    0.0%    0.0%    0.0%    0.0%    0.0%    0.0%    0.0%    0.0%    0.0%    0.0%    0.0%    0.0%    0.0%    0.0%    0.0%    0.0%    0.0%    0.0%    0.0%    0.0%    0.0%    0.0%    0.0%    0.0%    0.0%    0.0%    0.0%    0.0%    0.0%        k__Bacteria;p__Planctomycetes; c__Kueneniae       0    0.0%    0.0%    0.0%    0.0%    0.0%    0.0%    0.0%    0.0%    0.0%    0.0%    0.0%    0.0%    0.0%    0.0%    0.0%    0.0%    0.0%    0.0%    0.0%    0.0%    0.0%    0.0%    0.0%    0.0%    0.0%    0.0%    0.0%    0.0%    0.0%    0.0%    0.0%    0.0%    0.0%    0.0%    0.0%    0.0%    0.0%    0.0%    0.0%    0.0%    0.0%    0.0%    0.0%    0.0%    0.0%    0.0%    0.0%    0.0%    0.0%    0.0%    0.0%    0.0%    0.0%    0.0%    0.0%    0.0%    0.0%    0.0%    0.0%    0.0%    0.0%    0.0%    0.0%    0.0%    0.0%    0.0%    0.0%    0.0%    0.0%    0.0%    0.0%    0.0%    0.0%    0.0%    0.0%    0.0%    0.0%    0.0%    0.0%    0.0%    0.0%    0.0%    0.0%    0.0%    0.0%    0.0%    0.0%    0.0%    0.0%    0.0%    0.0%    0.0%    0.0%    0.0%    0.0%    0.0%    0.0%    0.0%    0.0%    0.0%    0.0%    0.0%    0.0%    0.0%    0.0%    0.0%    0.0%    0.0%    0.0%    0.0%    0.0%    0.0%    0.0%    0.0%    0.0%    0.0%    0.0%    0.0%    0.0%    0.0%    0.0%    0.0%    0.0%    0.0%    0.0%    0.0%    0.0%    0.0%    0.0%    0.0%    0.0%    0.0%    0.0%    0.0%        k__Bacteria;p__Planctomycetes; c__PW285       0    0.0%    0.0%    0.0%    0.0%    0.0%    0.0%    0.0%    0.0%    0.0%    0.0%    0.0%    0.0%    0.0%    0.0%    0.0%    0.0%    0.0%    0.0%    0.0%    0.0%    0.0%    0.0%    0.0%    0.0%    0.0%    0.0%    0.0%    0.0%    0.0%    0.0%    0.0%    0.0%    0.0%    0.0%    0.0%    0.0%    0.0%    0.0%    0.0%    0.0%    0.0%    0.0%    0.0%    0.0%    0.0%    0.0%    0.0%    0.0%    0.0%    0.0%    0.0%    0.0%    0.0%    0.0%    0.0%    0.0%    0.0%    0.0%    0.0%    0.0%    0.0%    0.0%    0.0%    0.0%    0.0%    0.0%    0.0%    0.0%    0.0%    0.0%    0.0%    0.0%    0.0%    0.0%    0.0%    0.0%    0.0%    0.0%    0.0%    0.0%    0.0%    0.0%    0.0%    0.0%    0.0%    0.0%    0.0%    0.0%    0.0%    0.0%    0.0%    0.0%    0.0%    0.0%    0.0%    0.0%    0.0%    0.0%    0.0%    0.0%    0.0%    0.0%    0.0%    0.0%    0.0%    0.0%    0.0%    0.0%    0.0%    0.0%    0.0%    0.0%    0.0%    0.0%    0.0%    0.0%    0.0%    0.0%    0.0%    0.0%    0.0%    0.0%    0.0%    0.0%    0.0%    0.0%    0.0%    0.0%    0.0%    0.0%    0.0%    0.0%    0.0%    0.0%        k__Bacteria;p__Planctomycetes; c__Phycisphaerae     263    0.0%    0.1%    0.0%    0.0%    0.0%    0.1%    0.0%    0.0%    0.1%    0.0%    0.0%    0.0%    0.0%    0.0%    0.0%    0.0%    0.0%    0.0%    0.1%    0.0%    0.0%    0.0%    0.0%    0.0%    0.0%    0.0%    0.1%    0.0%    0.0%    0.0%    0.0%    0.0%    0.0%    0.0%    0.0%    0.0%    0.0%    0.0%    0.0%    0.0%    0.0%    0.1%    0.0%    0.0%    0.0%    0.0%    0.0%    0.0%    0.0%    0.0%    0.0%    0.0%    0.0%    0.1%    0.0%    0.1%    0.0%    0.0%    0.1%    0.0%    0.1%    0.0%    0.0%    0.0%    0.1%    0.0%    0.2%    0.0%    0.0%    0.1%    0.0%    0.0%    0.1%    0.0%    0.1%    0.0%    0.0%    0.1%    0.0%    0.0%    0.0%    0.0%    0.0%    0.0%    0.1%    0.1%    0.0%    0.0%    0.1%    0.0%    0.0%    0.0%    0.0%    0.0%    0.1%    0.1%    0.0%    0.0%    0.1%    0.1%    0.0%    0.1%    0.0%    0.0%    0.0%    0.0%    0.0%    0.0%    0.1%    0.0%    0.0%    0.0%    0.1%    0.0%    0.0%    0.0%    0.0%    0.0%    0.0%    0.0%    0.0%    0.1%    0.0%    0.0%    0.1%    0.0%    0.0%    0.0%    0.0%    0.1%    0.0%    0.0%    0.0%    0.0%        k__Bacteria;p__Planctomycetes; c__Planctomycea     421    0.0%    0.1%    0.1%    0.0%    0.0%    0.0%    0.0%    0.0%    0.0%    0.0%    0.0%    0.1%    0.0%    0.0%    0.1%    0.0%    0.1%    0.0%    0.0%    0.0%    0.1%    0.1%    0.0%    0.0%    0.0%    0.0%    0.1%    0.1%    0.1%    0.0%    0.0%    0.0%    0.0%    0.0%    0.1%    0.2%    0.0%    0.0%    0.0%    0.0%    0.0%    0.0%    0.0%    0.0%    0.0%    0.0%    0.0%    0.0%    0.1%    0.0%    0.0%    0.0%    0.1%    0.1%    0.1%    0.1%    0.0%    0.0%    0.0%    0.1%    0.2%    0.0%    0.0%    0.0%    0.0%    0.0%    0.2%    0.0%    0.0%    0.0%    0.0%    0.0%    0.1%    0.0%    0.1%    0.0%    0.0%    0.2%    0.1%    0.0%    0.0%    0.0%    0.0%    0.0%    0.1%    0.0%    0.0%    0.1%    0.0%    0.0%    0.1%    0.0%    0.0%    0.0%    0.1%    0.0%    0.0%    0.1%    0.0%    0.1%    0.0%    0.1%    0.0%    0.1%    0.1%    0.1%    0.0%    0.0%    0.3%    0.3%    0.0%    0.1%    0.1%    0.0%    0.0%    0.0%    0.0%    0.0%    0.0%    0.0%    0.0%    0.0%    0.0%    0.0%    0.1%    0.0%    0.1%    0.0%    0.1%    0.0%    0.0%    0.0%    0.0%    0.0%        k__Bacteria;p__Planctomycetes; c__agg27       7    0.0%    0.0%    0.0%    0.0%    0.0%    0.0%    0.0%    0.0%    0.0%    0.0%    0.0%    0.0%    0.0%    0.0%    0.0%    0.0%    0.0%    0.0%    0.0%    0.0%    0.0%    0.0%    0.0%    0.0%    0.0%    0.0%    0.0%    0.0%    0.0%    0.0%    0.0%    0.0%    0.0%    0.0%    0.0%    0.0%    0.0%    0.0%    0.0%    0.0%    0.0%    0.0%    0.0%    0.0%    0.0%    0.0%    0.0%    0.0%    0.0%    0.0%    0.0%    0.0%    0.0%    0.0%    0.0%    0.0%    0.0%    0.0%    0.0%    0.0%    0.0%    0.0%    0.0%    0.0%    0.0%    0.0%    0.0%    0.0%    0.0%    0.0%    0.0%    0.0%    0.0%    0.0%    0.0%    0.0%    0.0%    0.0%    0.0%    0.0%    0.0%    0.0%    0.0%    0.0%    0.0%    0.0%    0.0%    0.0%    0.0%    0.0%    0.0%    0.0%    0.0%    0.0%    0.0%    0.0%    0.0%    0.0%    0.0%    0.0%    0.0%    0.0%    0.0%    0.0%    0.0%    0.0%    0.0%    0.0%    0.0%    0.0%    0.0%    0.0%    0.0%    0.0%    0.0%    0.0%    0.0%    0.0%    0.0%    0.0%    0.0%    0.0%    0.0%    0.0%    0.0%    0.0%    0.0%    0.0%    0.0%    0.0%    0.0%    0.0%    0.0%    0.0%        k__Bacteria;p__Planctomycetes; c__vadinHA49       4    0.0%    0.0%    0.0%    0.0%    0.0%    0.0%    0.0%    0.0%    0.0%    0.0%    0.0%    0.0%    0.0%    0.0%    0.0%    0.0%    0.0%    0.0%    0.0%    0.0%    0.0%    0.0%    0.0%    0.0%    0.0%    0.0%    0.0%    0.0%    0.0%    0.0%    0.0%    0.0%    0.0%    0.0%    0.0%    0.0%    0.0%    0.0%    0.0%    0.0%    0.0%    0.0%    0.0%    0.0%    0.0%    0.0%    0.0%    0.0%    0.0%    0.0%    0.0%    0.0%    0.0%    0.0%    0.0%    0.0%    0.0%    0.0%    0.0%    0.0%    0.0%    0.0%    0.0%    0.0%    0.0%    0.0%    0.0%    0.0%    0.0%    0.0%    0.0%    0.0%    0.0%    0.0%    0.0%    0.0%    0.0%    0.0%    0.0%    0.0%    0.0%    0.0%    0.0%    0.0%    0.0%    0.0%    0.0%    0.0%    0.0%    0.0%    0.0%    0.0%    0.0%    0.0%    0.0%    0.0%    0.0%    0.0%    0.0%    0.0%    0.0%    0.0%    0.0%    0.0%    0.0%    0.0%    0.0%    0.0%    0.0%    0.0%    0.0%    0.0%    0.0%    0.0%    0.0%    0.0%    0.0%    0.0%    0.0%    0.0%    0.0%    0.0%    0.0%    0.0%    0.0%    0.0%    0.0%    0.0%    0.0%    0.0%    0.0%    0.0%    0.0%    0.0%        k__Bacteria;p__Proteobacteria; c__Alphaproteobacteria   47946    4.9%    3.9%    3.0%    5.4%   12.5%    8.5%   18.9%    3.6%    4.7%    1.5%    6.2%    4.9%    0.6%    4.9%    3.0%    3.3%   12.4%    1.5%    6.9%    1.8%    8.0%    3.6%    1.9%    1.3%    4.8%    2.5%   16.5%    5.0%    3.8%    1.8%    3.9%    3.0%    4.8%    2.5%    3.2%    4.1%    1.5%    5.0%    3.4%    4.3%    4.3%    7.6%   22.3%    2.2%    2.6%    3.5%    5.0%    1.0%    3.0%    3.7%    1.5%    6.4%    2.0%    9.5%    7.6%    5.2%    3.9%    1.4%    6.5%    4.8%    7.6%    2.3%    0.5%    0.8%    4.4%    2.0%    8.5%    3.6%    4.5%    6.9%    2.0%    2.5%    6.6%    2.9%    6.3%    1.2%    1.7%   11.6%    7.6%    2.3%    5.5%    4.5%    5.0%    5.3%    7.8%    6.1%    3.8%    4.9%    7.8%    2.7%    3.5%    2.8%    1.1%    5.3%    5.1%    5.1%    3.6%    5.3%   10.4%    2.3%    1.5%    4.7%    4.0%    6.1%    3.1%    2.6%    3.2%    4.6%    7.7%   14.3%    7.7%    7.3%    4.0%    4.7%    8.1%    6.0%   11.1%    8.8%    7.2%    2.0%    4.1%    6.5%    1.1%    3.9%    4.5%    5.9%    7.5%    5.3%    4.7%    6.9%    4.8%    4.6%    3.5%    4.6%        k__Bacteria;p__Proteobacteria; c__Betaproteobacteria   73421    7.5%    6.8%    4.3%    8.9%    3.4%    8.9%    5.3%    3.7%    3.1%    1.8%    5.6%   11.3%    4.3%    4.5%    3.5%    4.0%    5.5%    2.6%    6.0%    2.7%   13.3%    5.2%    5.5%    9.1%    7.2%   13.4%    4.9%    4.0%    4.9%    2.6%    3.1%    2.6%    5.9%    4.3%    4.1%    4.1%    2.7%   12.5%    9.4%    7.2%    8.0%    8.8%    6.4%    5.9%    6.4%    4.2%   11.0%   16.7%    8.5%    4.9%    1.8%    4.4%    7.2%    5.6%    4.0%    9.5%    3.4%    3.9%    5.6%    4.7%    4.7%    8.1%    1.2%    1.6%    6.3%    2.7%    6.4%    3.6%    3.4%    5.6%    2.2%    5.0%    7.6%    4.3%    6.0%    3.1%    3.2%    6.8%   16.2%   22.6%   17.5%   22.0%   24.2%   13.6%    7.5%    7.8%    6.6%    5.7%    9.5%    4.8%    3.8%    4.7%    4.6%   19.4%    8.6%    5.2%    6.8%    7.6%    5.7%    4.4%    1.6%    7.2%    4.3%    5.8%    3.8%    7.0%    3.4%    8.3%    8.1%   16.6%    6.9%    8.0%    5.2%   14.5%   29.4%    6.2%   35.2%   37.9%   25.8%    5.9%    5.5%    7.9%   10.2%    7.5%   10.1%    8.7%    5.8%    6.6%    7.1%   11.8%    7.8%   10.1%    4.1%    6.7%        k__Bacteria;p__Proteobacteria; c__Deltaproteobacteria    1519    0.2%    0.1%    0.1%    0.2%    0.0%    0.3%    0.1%    0.1%    0.1%    0.0%    0.2%    0.1%    0.0%    0.1%    0.1%    0.0%    0.2%    0.0%    0.1%    0.1%    0.2%    0.1%    0.0%    0.0%    0.2%    0.1%    0.6%    0.1%    0.2%    0.0%    0.0%    0.0%    0.1%    0.0%    0.3%    0.2%    0.1%    0.2%    0.1%    0.1%    0.0%    0.1%    0.1%    0.0%    0.2%    0.0%    0.1%    0.1%    0.0%    0.1%    0.1%    0.1%    0.1%    0.3%    0.2%    0.1%    0.2%    0.0%    0.2%    0.3%    0.3%    0.2%    0.0%    0.0%    0.2%    0.1%    0.3%    0.2%    0.2%    0.2%    0.0%    0.0%    0.4%    0.2%    0.3%    0.1%    0.0%    0.5%    0.1%    0.1%    0.1%    0.1%    0.0%    0.2%    0.4%    0.3%    0.2%    0.3%    0.3%    0.1%    0.2%    0.2%    0.0%    0.2%    0.5%    0.2%    0.2%    0.3%    0.2%    0.2%    0.1%    0.3%    0.2%    0.3%    0.1%    0.1%    0.2%    0.1%    0.4%    0.7%    0.3%    0.3%    0.2%    0.0%    0.1%    0.3%    0.3%    0.2%    0.2%    0.1%    0.2%    0.3%    0.0%    0.2%    0.3%    0.1%    0.2%    0.3%    0.1%    0.3%    0.2%    0.2%    0.0%    0.2%        k__Bacteria;p__Proteobacteria; c__Epsilonproteobacteria    1447    0.1%    0.3%    0.2%    0.0%    0.1%    0.1%    0.1%    0.1%    0.1%    0.0%    0.1%    0.1%    0.1%    0.1%    0.1%    0.1%    0.1%    0.1%    0.2%    0.2%    0.1%    0.2%    0.1%    0.2%    0.1%    0.3%    0.1%    0.1%    0.1%    0.1%    0.1%    0.1%    0.2%    0.2%    0.2%    0.1%    0.1%    0.1%    0.2%    0.2%    0.1%    0.2%    0.2%    0.2%    0.4%    0.0%    0.6%    0.4%    0.2%    0.1%    0.1%    0.1%    0.2%    0.1%    0.0%    0.3%    0.1%    0.0%    0.1%    0.2%    0.1%    0.2%    0.0%    0.1%    0.2%    0.1%    0.0%    0.1%    0.1%    0.2%    0.1%    0.2%    0.1%    0.4%    0.1%    0.1%    0.2%    0.1%    0.2%    0.1%    0.0%    0.1%    0.1%    0.4%    0.3%    0.1%    0.2%    0.1%    0.1%    0.2%    0.1%    0.3%    0.2%    0.2%    0.7%    0.1%    0.3%    0.4%    0.2%    0.3%    0.0%    0.2%    0.2%    0.1%    0.2%    0.1%    0.1%    0.1%    0.1%    0.0%    0.0%    0.3%    0.1%    0.0%    0.0%    0.1%    0.0%    0.0%    0.1%    0.1%    0.1%    0.0%    0.2%    0.1%    0.2%    0.2%    0.2%    0.1%    0.1%    0.0%    0.1%    0.2%    0.1%    0.0%        k__Bacteria;p__Proteobacteria; c__Gammaproteobacteria   160101   16.4%   19.5%   43.0%   13.6%    8.5%   15.5%    9.4%    6.3%   11.2%   27.1%   12.7%   15.0%    4.7%    9.8%   17.2%   22.4%   21.1%   16.7%   16.5%    7.4%   15.6%   15.3%   16.1%    5.6%   15.1%   33.1%   11.4%   20.8%    9.2%    7.4%   12.3%   18.0%   25.4%   19.1%   24.0%   27.3%   12.5%   18.5%   22.3%   23.8%   34.5%   11.8%   12.7%   20.3%   22.0%   26.9%   16.2%    8.9%   26.4%   25.9%    8.8%   15.2%   19.2%   11.9%   10.2%   19.1%   24.9%   23.8%   12.7%   10.2%   12.0%   13.5%    1.5%    6.5%   18.2%    5.0%   12.8%   14.2%   19.2%   10.3%   16.6%   16.8%   21.9%   12.9%   12.8%    5.7%    5.9%   17.0%    8.7%    5.3%    6.5%    9.8%    3.6%   10.9%   12.1%   13.1%   19.0%   13.2%   17.6%   11.0%   24.4%   10.6%    7.9%   17.2%   19.1%   15.1%   11.9%   14.6%   24.1%   31.4%   44.5%   14.3%   12.7%   24.1%   33.8%   38.9%   16.3%   27.1%   17.1%   18.4%   14.7%   17.3%   10.3%   15.3%    9.7%   17.1%    8.1%    9.2%   11.0%   12.4%   11.2%   15.0%   10.5%   30.6%   27.1%   31.4%   20.4%   16.5%   23.9%   28.6%   21.2%   17.1%   23.6%   20.0%        k__Bacteria;p__SC3; c__       0    0.0%    0.0%    0.0%    0.0%    0.0%    0.0%    0.0%    0.0%    0.0%    0.0%    0.0%    0.0%    0.0%    0.0%    0.0%    0.0%    0.0%    0.0%    0.0%    0.0%    0.0%    0.0%    0.0%    0.0%    0.0%    0.0%    0.0%    0.0%    0.0%    0.0%    0.0%    0.0%    0.0%    0.0%    0.0%    0.0%    0.0%    0.0%    0.0%    0.0%    0.0%    0.0%    0.0%    0.0%    0.0%    0.0%    0.0%    0.0%    0.0%    0.0%    0.0%    0.0%    0.0%    0.0%    0.0%    0.0%    0.0%    0.0%    0.0%    0.0%    0.0%    0.0%    0.0%    0.0%    0.0%    0.0%    0.0%    0.0%    0.0%    0.0%    0.0%    0.0%    0.0%    0.0%    0.0%    0.0%    0.0%    0.0%    0.0%    0.0%    0.0%    0.0%    0.0%    0.0%    0.0%    0.0%    0.0%    0.0%    0.0%    0.0%    0.0%    0.0%    0.0%    0.0%    0.0%    0.0%    0.0%    0.0%    0.0%    0.0%    0.0%    0.0%    0.0%    0.0%    0.0%    0.0%    0.0%    0.0%    0.0%    0.0%    0.0%    0.0%    0.0%    0.0%    0.0%    0.0%    0.0%    0.0%    0.0%    0.0%    0.0%    0.0%    0.0%    0.0%    0.0%    0.0%    0.0%    0.0%    0.0%    0.0%    0.0%    0.0%    0.0%    0.0%        k__Bacteria;p__SC4; c__      98    0.0%    0.0%    0.0%    0.0%    0.0%    0.0%    0.0%    0.0%    0.0%    0.0%    0.0%    0.0%    0.0%    0.0%    0.0%    0.0%    0.0%    0.0%    0.0%    0.0%    0.1%    0.0%    0.0%    0.0%    0.0%    0.0%    0.1%    0.0%    0.0%    0.0%    0.0%    0.0%    0.0%    0.0%    0.0%    0.0%    0.0%    0.0%    0.0%    0.0%    0.0%    0.0%    0.0%    0.0%    0.0%    0.0%    0.0%    0.0%    0.0%    0.0%    0.0%    0.0%    0.0%    0.0%    0.0%    0.0%    0.0%    0.0%    0.0%    0.0%    0.0%    0.0%    0.0%    0.0%    0.0%    0.0%    0.1%    0.0%    0.0%    0.0%    0.0%    0.0%    0.0%    0.0%    0.0%    0.0%    0.0%    0.1%    0.0%    0.0%    0.0%    0.0%    0.0%    0.0%    0.0%    0.1%    0.0%    0.0%    0.0%    0.0%    0.0%    0.0%    0.0%    0.0%    0.0%    0.0%    0.0%    0.0%    0.0%    0.0%    0.0%    0.0%    0.0%    0.1%    0.0%    0.0%    0.0%    0.0%    0.0%    0.0%    0.0%    0.0%    0.0%    0.0%    0.0%    0.0%    0.0%    0.0%    0.0%    0.0%    0.0%    0.0%    0.0%    0.0%    0.0%    0.0%    0.0%    0.0%    0.0%    0.0%    0.0%    0.0%    0.0%    0.0%        k__Bacteria;p__SPAM; c__      26    0.0%    0.0%    0.0%    0.0%    0.0%    0.0%    0.0%    0.0%    0.0%    0.0%    0.0%    0.0%    0.0%    0.0%    0.0%    0.0%    0.0%    0.0%    0.0%    0.0%    0.0%    0.0%    0.0%    0.0%    0.0%    0.0%    0.0%    0.0%    0.0%    0.0%    0.0%    0.0%    0.0%    0.0%    0.0%    0.0%    0.0%    0.0%    0.0%    0.0%    0.0%    0.0%    0.0%    0.0%    0.0%    0.0%    0.0%    0.0%    0.0%    0.0%    0.0%    0.0%    0.0%    0.0%    0.0%    0.0%    0.0%    0.0%    0.0%    0.0%    0.0%    0.0%    0.0%    0.0%    0.0%    0.0%    0.0%    0.0%    0.0%    0.0%    0.0%    0.0%    0.0%    0.0%    0.0%    0.0%    0.0%    0.0%    0.0%    0.0%    0.0%    0.0%    0.0%    0.0%    0.0%    0.0%    0.0%    0.0%    0.0%    0.0%    0.0%    0.0%    0.0%    0.0%    0.0%    0.0%    0.0%    0.0%    0.0%    0.0%    0.0%    0.0%    0.0%    0.0%    0.0%    0.0%    0.0%    0.0%    0.0%    0.0%    0.0%    0.0%    0.0%    0.0%    0.0%    0.0%    0.0%    0.0%    0.0%    0.0%    0.0%    0.0%    0.0%    0.0%    0.0%    0.0%    0.0%    0.0%    0.0%    0.0%    0.0%    0.0%    0.0%    0.0%        k__Bacteria;p__SR1; c__     139    0.0%    0.0%    0.0%    0.0%    0.0%    0.0%    0.0%    0.0%    0.0%    0.0%    0.0%    0.0%    0.0%    0.0%    0.0%    0.0%    0.0%    0.0%    0.0%    0.0%    0.0%    0.0%    0.0%    0.0%    0.0%    0.1%    0.0%    0.0%    0.0%    0.0%    0.0%    0.0%    0.0%    0.0%    0.0%    0.0%    0.0%    0.0%    0.0%    0.0%    0.0%    0.0%    0.1%    0.0%    0.0%    0.0%    0.0%    0.0%    0.2%    0.0%    0.0%    0.0%    0.0%    0.0%    0.0%    0.0%    0.0%    0.0%    0.0%    0.0%    0.0%    0.1%    0.0%    0.0%    0.0%    0.0%    0.0%    0.0%    0.0%    0.0%    0.0%    0.0%    0.0%    0.0%    0.0%    0.0%    0.0%    0.0%    0.0%    0.0%    0.0%    0.0%    0.0%    0.0%    0.0%    0.0%    0.0%    0.1%    0.0%    0.0%    0.0%    0.1%    0.0%    0.0%    0.0%    0.0%    0.0%    0.0%    0.0%    0.0%    0.0%    0.0%    0.0%    0.0%    0.0%    0.0%    0.0%    0.0%    0.0%    0.0%    0.0%    0.0%    0.0%    0.0%    0.0%    0.0%    0.0%    0.0%    0.0%    0.1%    0.0%    0.0%    0.0%    0.0%    0.1%    0.1%    0.0%    0.0%    0.0%    0.0%    0.0%    0.0%    0.0%    0.0%        k__Bacteria;p__Spirochaetes; c__Brachyspirae       0    0.0%    0.0%    0.0%    0.0%    0.0%    0.0%    0.0%    0.0%    0.0%    0.0%    0.0%    0.0%    0.0%    0.0%    0.0%    0.0%    0.0%    0.0%    0.0%    0.0%    0.0%    0.0%    0.0%    0.0%    0.0%    0.0%    0.0%    0.0%    0.0%    0.0%    0.0%    0.0%    0.0%    0.0%    0.0%    0.0%    0.0%    0.0%    0.0%    0.0%    0.0%    0.0%    0.0%    0.0%    0.0%    0.0%    0.0%    0.0%    0.0%    0.0%    0.0%    0.0%    0.0%    0.0%    0.0%    0.0%    0.0%    0.0%    0.0%    0.0%    0.0%    0.0%    0.0%    0.0%    0.0%    0.0%    0.0%    0.0%    0.0%    0.0%    0.0%    0.0%    0.0%    0.0%    0.0%    0.0%    0.0%    0.0%    0.0%    0.0%    0.0%    0.0%    0.0%    0.0%    0.0%    0.0%    0.0%    0.0%    0.0%    0.0%    0.0%    0.0%    0.0%    0.0%    0.0%    0.0%    0.0%    0.0%    0.0%    0.0%    0.0%    0.0%    0.0%    0.0%    0.0%    0.0%    0.0%    0.0%    0.0%    0.0%    0.0%    0.0%    0.0%    0.0%    0.0%    0.0%    0.0%    0.0%    0.0%    0.0%    0.0%    0.0%    0.0%    0.0%    0.0%    0.0%    0.0%    0.0%    0.0%    0.0%    0.0%    0.0%    0.0%    0.0%        k__Bacteria;p__Spirochaetes; c__Leptospirae       0    0.0%    0.0%    0.0%    0.0%    0.0%    0.0%    0.0%    0.0%    0.0%    0.0%    0.0%    0.0%    0.0%    0.0%    0.0%    0.0%    0.0%    0.0%    0.0%    0.0%    0.0%    0.0%    0.0%    0.0%    0.0%    0.0%    0.0%    0.0%    0.0%    0.0%    0.0%    0.0%    0.0%    0.0%    0.0%    0.0%    0.0%    0.0%    0.0%    0.0%    0.0%    0.0%    0.0%    0.0%    0.0%    0.0%    0.0%    0.0%    0.0%    0.0%    0.0%    0.0%    0.0%    0.0%    0.0%    0.0%    0.0%    0.0%    0.0%    0.0%    0.0%    0.0%    0.0%    0.0%    0.0%    0.0%    0.0%    0.0%    0.0%    0.0%    0.0%    0.0%    0.0%    0.0%    0.0%    0.0%    0.0%    0.0%    0.0%    0.0%    0.0%    0.0%    0.0%    0.0%    0.0%    0.0%    0.0%    0.0%    0.0%    0.0%    0.0%    0.0%    0.0%    0.0%    0.0%    0.0%    0.0%    0.0%    0.0%    0.0%    0.0%    0.0%    0.0%    0.0%    0.0%    0.0%    0.0%    0.0%    0.0%    0.0%    0.0%    0.0%    0.0%    0.0%    0.0%    0.0%    0.0%    0.0%    0.0%    0.0%    0.0%    0.0%    0.0%    0.0%    0.0%    0.0%    0.0%    0.0%    0.0%    0.0%    0.0%    0.0%    0.0%    0.0%        k__Bacteria;p__Spirochaetes; c__SP_WWE1       0    0.0%    0.0%    0.0%    0.0%    0.0%    0.0%    0.0%    0.0%    0.0%    0.0%    0.0%    0.0%    0.0%    0.0%    0.0%    0.0%    0.0%    0.0%    0.0%    0.0%    0.0%    0.0%    0.0%    0.0%    0.0%    0.0%    0.0%    0.0%    0.0%    0.0%    0.0%    0.0%    0.0%    0.0%    0.0%    0.0%    0.0%    0.0%    0.0%    0.0%    0.0%    0.0%    0.0%    0.0%    0.0%    0.0%    0.0%    0.0%    0.0%    0.0%    0.0%    0.0%    0.0%    0.0%    0.0%    0.0%    0.0%    0.0%    0.0%    0.0%    0.0%    0.0%    0.0%    0.0%    0.0%    0.0%    0.0%    0.0%    0.0%    0.0%    0.0%    0.0%    0.0%    0.0%    0.0%    0.0%    0.0%    0.0%    0.0%    0.0%    0.0%    0.0%    0.0%    0.0%    0.0%    0.0%    0.0%    0.0%    0.0%    0.0%    0.0%    0.0%    0.0%    0.0%    0.0%    0.0%    0.0%    0.0%    0.0%    0.0%    0.0%    0.0%    0.0%    0.0%    0.0%    0.0%    0.0%    0.0%    0.0%    0.0%    0.0%    0.0%    0.0%    0.0%    0.0%    0.0%    0.0%    0.0%    0.0%    0.0%    0.0%    0.0%    0.0%    0.0%    0.0%    0.0%    0.0%    0.0%    0.0%    0.0%    0.0%    0.0%    0.0%    0.0%        k__Bacteria;p__Spirochaetes; c__Spirochaetes (class)     997    0.1%    0.0%    0.0%    0.0%    0.0%    0.0%    0.0%    0.0%    0.0%    0.0%    0.0%    0.8%    0.1%    0.0%    0.1%    0.0%    0.1%    0.0%    0.1%    0.0%    0.0%    0.1%    0.2%    0.0%    0.2%    0.2%    0.0%    0.0%    0.1%    0.1%    0.1%    0.1%    0.1%    0.1%    0.1%    0.0%    0.0%    0.1%    0.3%    0.2%    0.0%    0.1%    0.1%    0.2%    0.6%    0.1%    0.3%    0.1%    0.2%    0.1%    0.0%    0.0%    0.0%    0.1%    0.0%    0.2%    0.1%    0.0%    0.0%    0.0%    0.1%    0.1%    0.0%    0.0%    0.2%    0.0%    0.1%    0.0%    0.0%    0.1%    0.0%    0.1%    0.1%    1.5%    0.2%    0.0%    0.0%    0.0%    0.0%    0.0%    0.0%    0.1%    0.0%    0.5%    0.2%    0.2%    0.1%    0.1%    0.1%    0.0%    0.0%    0.0%    0.0%    0.2%    1.7%    0.2%    0.1%    0.1%    0.2%    0.0%    0.0%    0.1%    0.2%    0.1%    0.0%    0.0%    0.1%    0.0%    0.1%    0.0%    0.1%    0.0%    0.1%    0.0%    0.0%    0.0%    0.0%    0.0%    0.0%    0.1%    0.1%    0.0%    0.0%    0.1%    0.1%    0.2%    0.0%    0.0%    0.0%    0.0%    0.0%    0.2%    0.0%    0.0%        k__Bacteria;p__Synergistetes; c__Synergistia     111    0.0%    0.0%    0.0%    0.0%    0.0%    0.0%    0.0%    0.0%    0.0%    0.0%    0.0%    0.1%    0.0%    0.0%    0.1%    0.0%    0.0%    0.0%    0.0%    0.0%    0.0%    0.0%    0.0%    0.0%    0.1%    0.0%    0.0%    0.0%    0.0%    0.0%    0.0%    0.0%    0.0%    0.0%    0.0%    0.0%    0.0%    0.0%    0.0%    0.0%    0.0%    0.0%    0.0%    0.0%    0.0%    0.0%    0.0%    0.0%    0.0%    0.0%    0.0%    0.0%    0.0%    0.0%    0.0%    0.0%    0.0%    0.0%    0.0%    0.0%    0.0%    0.0%    0.0%    0.0%    0.0%    0.0%    0.0%    0.0%    0.0%    0.0%    0.0%    0.0%    0.0%    0.1%    0.0%    0.0%    0.0%    0.0%    0.0%    0.0%    0.0%    0.0%    0.0%    0.0%    0.0%    0.0%    0.0%    0.0%    0.0%    0.0%    0.0%    0.0%    0.0%    0.1%    0.2%    0.0%    0.0%    0.0%    0.0%    0.0%    0.0%    0.0%    0.1%    0.0%    0.0%    0.0%    0.0%    0.0%    0.0%    0.0%    0.0%    0.0%    0.0%    0.0%    0.0%    0.0%    0.0%    0.0%    0.0%    0.0%    0.0%    0.0%    0.0%    0.0%    0.0%    0.0%    0.0%    0.0%    0.0%    0.0%    0.0%    0.0%    0.0%    0.0%        k__Bacteria;p__TM6; c__       0    0.0%    0.0%    0.0%    0.0%    0.0%    0.0%    0.0%    0.0%    0.0%    0.0%    0.0%    0.0%    0.0%    0.0%    0.0%    0.0%    0.0%    0.0%    0.0%    0.0%    0.0%    0.0%    0.0%    0.0%    0.0%    0.0%    0.0%    0.0%    0.0%    0.0%    0.0%    0.0%    0.0%    0.0%    0.0%    0.0%    0.0%    0.0%    0.0%    0.0%    0.0%    0.0%    0.0%    0.0%    0.0%    0.0%    0.0%    0.0%    0.0%    0.0%    0.0%    0.0%    0.0%    0.0%    0.0%    0.0%    0.0%    0.0%    0.0%    0.0%    0.0%    0.0%    0.0%    0.0%    0.0%    0.0%    0.0%    0.0%    0.0%    0.0%    0.0%    0.0%    0.0%    0.0%    0.0%    0.0%    0.0%    0.0%    0.0%    0.0%    0.0%    0.0%    0.0%    0.0%    0.0%    0.0%    0.0%    0.0%    0.0%    0.0%    0.0%    0.0%    0.0%    0.0%    0.0%    0.0%    0.0%    0.0%    0.0%    0.0%    0.0%    0.0%    0.0%    0.0%    0.0%    0.0%    0.0%    0.0%    0.0%    0.0%    0.0%    0.0%    0.0%    0.0%    0.0%    0.0%    0.0%    0.0%    0.0%    0.0%    0.0%    0.0%    0.0%    0.0%    0.0%    0.0%    0.0%    0.0%    0.0%    0.0%    0.0%    0.0%    0.0%    0.0%        k__Bacteria;p__TM7; c__       0    0.0%    0.0%    0.0%    0.0%    0.0%    0.0%    0.0%    0.0%    0.0%    0.0%    0.0%    0.0%    0.0%    0.0%    0.0%    0.0%    0.0%    0.0%    0.0%    0.0%    0.0%    0.0%    0.0%    0.0%    0.0%    0.0%    0.0%    0.0%    0.0%    0.0%    0.0%    0.0%    0.0%    0.0%    0.0%    0.0%    0.0%    0.0%    0.0%    0.0%    0.0%    0.0%    0.0%    0.0%    0.0%    0.0%    0.0%    0.0%    0.0%    0.0%    0.0%    0.0%    0.0%    0.0%    0.0%    0.0%    0.0%    0.0%    0.0%    0.0%    0.0%    0.0%    0.0%    0.0%    0.0%    0.0%    0.0%    0.0%    0.0%    0.0%    0.0%    0.0%    0.0%    0.0%    0.0%    0.0%    0.0%    0.0%    0.0%    0.0%    0.0%    0.0%    0.0%    0.0%    0.0%    0.0%    0.0%    0.0%    0.0%    0.0%    0.0%    0.0%    0.0%    0.0%    0.0%    0.0%    0.0%    0.0%    0.0%    0.0%    0.0%    0.0%    0.0%    0.0%    0.0%    0.0%    0.0%    0.0%    0.0%    0.0%    0.0%    0.0%    0.0%    0.0%    0.0%    0.0%    0.0%    0.0%    0.0%    0.0%    0.0%    0.0%    0.0%    0.0%    0.0%    0.0%    0.0%    0.0%    0.0%    0.0%    0.0%    0.0%    0.0%    0.0%        k__Bacteria;p__TM7; c__TM7-1       1    0.0%    0.0%    0.0%    0.0%    0.0%    0.0%    0.0%    0.0%    0.0%    0.0%    0.0%    0.0%    0.0%    0.0%    0.0%    0.0%    0.0%    0.0%    0.0%    0.0%    0.0%    0.0%    0.0%    0.0%    0.0%    0.0%    0.0%    0.0%    0.0%    0.0%    0.0%    0.0%    0.0%    0.0%    0.0%    0.0%    0.0%    0.0%    0.0%    0.0%    0.0%    0.0%    0.0%    0.0%    0.0%    0.0%    0.0%    0.0%    0.0%    0.0%    0.0%    0.0%    0.0%    0.0%    0.0%    0.0%    0.0%    0.0%    0.0%    0.0%    0.0%    0.0%    0.0%    0.0%    0.0%    0.0%    0.0%    0.0%    0.0%    0.0%    0.0%    0.0%    0.0%    0.0%    0.0%    0.0%    0.0%    0.0%    0.0%    0.0%    0.0%    0.0%    0.0%    0.0%    0.0%    0.0%    0.0%    0.0%    0.0%    0.0%    0.0%    0.0%    0.0%    0.0%    0.0%    0.0%    0.0%    0.0%    0.0%    0.0%    0.0%    0.0%    0.0%    0.0%    0.0%    0.0%    0.0%    0.0%    0.0%    0.0%    0.0%    0.0%    0.0%    0.0%    0.0%    0.0%    0.0%    0.0%    0.0%    0.0%    0.0%    0.0%    0.0%    0.0%    0.0%    0.0%    0.0%    0.0%    0.0%    0.0%    0.0%    0.0%    0.0%    0.0%        k__Bacteria;p__TM7; c__TM7-3      30    0.0%    0.0%    0.0%    0.0%    0.0%    0.0%    0.0%    0.0%    0.0%    0.0%    0.0%    0.0%    0.0%    0.0%    0.0%    0.0%    0.0%    0.0%    0.0%    0.0%    0.0%    0.0%    0.0%    0.0%    0.0%    0.0%    0.0%    0.0%    0.0%    0.0%    0.0%    0.0%    0.0%    0.0%    0.0%    0.0%    0.0%    0.0%    0.0%    0.0%    0.0%    0.0%    0.0%    0.0%    0.0%    0.0%    0.0%    0.0%    0.0%    0.0%    0.0%    0.0%    0.0%    0.0%    0.0%    0.0%    0.0%    0.0%    0.0%    0.0%    0.0%    0.0%    0.0%    0.0%    0.0%    0.0%    0.0%    0.0%    0.0%    0.0%    0.0%    0.0%    0.0%    0.0%    0.0%    0.0%    0.0%    0.0%    0.0%    0.0%    0.0%    0.0%    0.0%    0.0%    0.0%    0.0%    0.0%    0.0%    0.0%    0.0%    0.0%    0.0%    0.0%    0.0%    0.0%    0.0%    0.0%    0.0%    0.0%    0.0%    0.0%    0.0%    0.0%    0.0%    0.0%    0.0%    0.0%    0.0%    0.0%    0.0%    0.0%    0.0%    0.0%    0.0%    0.0%    0.0%    0.0%    0.0%    0.0%    0.0%    0.0%    0.0%    0.0%    0.0%    0.0%    0.0%    0.0%    0.0%    0.0%    0.0%    0.0%    0.0%    0.0%    0.0%        k__Bacteria;p__Tenericutes; c__       1    0.0%    0.0%    0.0%    0.0%    0.0%    0.0%    0.0%    0.0%    0.0%    0.0%    0.0%    0.0%    0.0%    0.0%    0.0%    0.0%    0.0%    0.0%    0.0%    0.0%    0.0%    0.0%    0.0%    0.0%    0.0%    0.0%    0.0%    0.0%    0.0%    0.0%    0.0%    0.0%    0.0%    0.0%    0.0%    0.0%    0.0%    0.0%    0.0%    0.0%    0.0%    0.0%    0.0%    0.0%    0.0%    0.0%    0.0%    0.0%    0.0%    0.0%    0.0%    0.0%    0.0%    0.0%    0.0%    0.0%    0.0%    0.0%    0.0%    0.0%    0.0%    0.0%    0.0%    0.0%    0.0%    0.0%    0.0%    0.0%    0.0%    0.0%    0.0%    0.0%    0.0%    0.0%    0.0%    0.0%    0.0%    0.0%    0.0%    0.0%    0.0%    0.0%    0.0%    0.0%    0.0%    0.0%    0.0%    0.0%    0.0%    0.0%    0.0%    0.0%    0.0%    0.0%    0.0%    0.0%    0.0%    0.0%    0.0%    0.0%    0.0%    0.0%    0.0%    0.0%    0.0%    0.0%    0.0%    0.0%    0.0%    0.0%    0.0%    0.0%    0.0%    0.0%    0.0%    0.0%    0.0%    0.0%    0.0%    0.0%    0.0%    0.0%    0.0%    0.0%    0.0%    0.0%    0.0%    0.0%    0.0%    0.0%    0.0%    0.0%    0.0%    0.0%        k__Bacteria;p__Tenericutes; c__Erysipelotrichi    3409    0.3%    0.5%    0.5%    0.0%    0.0%    0.2%    0.1%    0.2%    0.1%    0.0%    0.1%    0.2%    0.0%    0.2%    0.1%    0.1%    0.0%    0.1%    0.3%    0.2%    0.0%    0.9%    1.0%    0.1%    0.4%    0.2%    0.2%    0.2%    1.6%    0.5%    0.1%    0.4%    0.3%    0.1%    0.3%    0.1%    0.1%    0.4%    0.4%    0.9%    0.0%    0.2%    0.2%    0.6%    0.4%    0.0%    1.0%    0.3%    0.4%    0.3%    0.1%    0.1%    0.1%    0.3%    0.2%    0.3%    0.1%    0.1%    0.1%    0.5%    0.3%    0.2%    0.0%    0.0%    0.5%    0.1%    0.1%    0.1%    0.1%    0.1%    0.0%    1.1%    0.8%    1.1%    0.2%    0.0%    0.0%    0.1%    0.1%    3.2%    1.3%    0.3%    0.1%    0.5%    0.2%    0.3%    0.4%    0.6%    0.3%    0.1%    0.2%    2.4%    0.1%    1.0%    0.6%    0.2%    2.2%    1.9%    0.5%    0.2%    0.2%    0.7%    0.3%    0.6%    0.8%    0.1%    0.3%    0.2%    0.2%    0.1%    0.2%    0.4%    0.5%    0.1%    0.1%    0.3%    0.1%    0.1%    0.1%    0.1%    0.1%    0.3%    0.2%    0.4%    0.3%    0.1%    0.3%    0.6%    0.6%    0.2%    0.2%    0.1%    0.1%    0.1%        k__Bacteria;p__Tenericutes; c__ML615J-28       0    0.0%    0.0%    0.0%    0.0%    0.0%    0.0%    0.0%    0.0%    0.0%    0.0%    0.0%    0.0%    0.0%    0.0%    0.0%    0.0%    0.0%    0.0%    0.0%    0.0%    0.0%    0.0%    0.0%    0.0%    0.0%    0.0%    0.0%    0.0%    0.0%    0.0%    0.0%    0.0%    0.0%    0.0%    0.0%    0.0%    0.0%    0.0%    0.0%    0.0%    0.0%    0.0%    0.0%    0.0%    0.0%    0.0%    0.0%    0.0%    0.0%    0.0%    0.0%    0.0%    0.0%    0.0%    0.0%    0.0%    0.0%    0.0%    0.0%    0.0%    0.0%    0.0%    0.0%    0.0%    0.0%    0.0%    0.0%    0.0%    0.0%    0.0%    0.0%    0.0%    0.0%    0.0%    0.0%    0.0%    0.0%    0.0%    0.0%    0.0%    0.0%    0.0%    0.0%    0.0%    0.0%    0.0%    0.0%    0.0%    0.0%    0.0%    0.0%    0.0%    0.0%    0.0%    0.0%    0.0%    0.0%    0.0%    0.0%    0.0%    0.0%    0.0%    0.0%    0.0%    0.0%    0.0%    0.0%    0.0%    0.0%    0.0%    0.0%    0.0%    0.0%    0.0%    0.0%    0.0%    0.0%    0.0%    0.0%    0.0%    0.0%    0.0%    0.0%    0.0%    0.0%    0.0%    0.0%    0.0%    0.0%    0.0%    0.0%    0.0%    0.0%    0.0%        k__Bacteria;p__Tenericutes; c__Mollicutes    4584    0.5%    0.3%    0.2%    0.0%    0.1%    0.1%    0.1%    0.1%    0.2%    0.0%    0.3%    0.9%    0.3%    0.2%    0.3%    1.0%    0.2%    1.0%    0.5%    0.6%    0.1%    0.4%    1.2%    0.1%    0.2%    0.6%    0.1%    0.3%    0.4%    0.3%    0.1%    0.1%    0.2%    0.1%    0.3%    0.5%    0.3%    2.0%    1.6%    1.1%    0.2%    0.6%    0.6%    1.2%    0.9%    0.1%    0.5%    1.3%    1.2%    1.1%    0.4%    0.2%    0.4%    0.5%    0.5%    0.5%    0.5%    0.0%    1.2%    0.3%    0.8%    0.8%    0.1%    0.1%    2.7%    0.3%    0.5%   13.9%    0.6%    0.2%    0.1%    0.2%    0.5%    0.7%    0.5%    0.1%    0.1%    0.6%    0.3%    0.1%    0.4%    0.0%    0.0%    0.4%    0.6%    0.2%    0.3%    0.8%    0.5%    0.0%    0.8%    0.1%    0.1%    0.5%    0.3%    0.3%    0.2%    0.2%    0.3%    0.1%    0.1%    0.1%    0.1%    0.3%    0.1%    0.0%    0.1%    0.1%    0.1%    0.1%    0.2%    0.5%    0.2%    0.1%    0.2%    0.2%    0.1%    0.1%    0.2%    0.2%    0.3%    1.9%    0.0%    0.1%    0.5%    0.3%    0.2%    0.4%    0.4%    0.2%    0.3%    0.3%    0.1%    0.3%        k__Bacteria;p__Thermi; c__Deinococci    3441    0.4%    0.2%    0.1%    2.4%    0.1%    0.2%    0.0%    0.1%    0.2%    0.1%    0.5%    0.1%    0.0%    0.2%    0.3%    0.4%    0.3%    0.3%    0.3%    0.1%    0.1%    0.1%    0.0%    0.3%    0.1%    0.1%    0.1%    0.5%    0.1%    0.1%    0.5%    0.6%    0.2%    0.4%    0.6%    0.7%    0.2%    0.2%    0.2%    0.2%    0.5%    0.3%    0.1%    0.1%    0.1%    0.0%    0.2%    0.0%    0.1%    0.6%    0.1%    0.9%    0.1%    0.1%    0.1%    0.1%    0.8%    0.5%    0.4%    0.6%    0.3%    0.1%    1.4%    0.2%    0.4%    0.2%    0.1%    0.1%    0.2%    0.6%    0.3%    0.2%    0.3%    0.1%    0.1%    0.5%    2.2%    0.1%    0.3%    0.1%    0.2%    0.1%    0.3%    0.4%    0.4%    0.3%    0.6%    0.4%    0.6%    0.3%    0.7%    0.4%    0.8%    0.1%    0.2%    0.5%    0.1%    0.1%    0.1%    0.1%    0.1%    0.2%    1.2%    1.0%    0.5%    1.4%    0.4%    0.4%    0.4%    0.6%    1.5%    0.7%    0.6%    0.1%    0.3%    0.9%    0.2%    0.3%    0.2%    0.1%    0.5%    0.8%    0.3%    0.3%    0.2%    0.2%    0.5%    0.2%    0.7%    0.7%    0.4%    0.4%    0.3%    0.3%        k__Bacteria;p__Thermotogae; c__Thermotogae (class)       4    0.0%    0.0%    0.0%    0.0%    0.0%    0.0%    0.0%    0.0%    0.0%    0.0%    0.0%    0.0%    0.0%    0.0%    0.0%    0.0%    0.0%    0.0%    0.0%    0.0%    0.0%    0.0%    0.0%    0.0%    0.0%    0.0%    0.0%    0.0%    0.0%    0.0%    0.0%    0.0%    0.0%    0.0%    0.0%    0.0%    0.0%    0.0%    0.0%    0.0%    0.0%    0.0%    0.0%    0.0%    0.0%    0.0%    0.0%    0.0%    0.0%    0.0%    0.0%    0.0%    0.0%    0.0%    0.0%    0.0%    0.0%    0.0%    0.0%    0.0%    0.0%    0.0%    0.0%    0.0%    0.0%    0.0%    0.0%    0.0%    0.0%    0.0%    0.0%    0.0%    0.0%    0.0%    0.0%    0.0%    0.0%    0.0%    0.0%    0.0%    0.0%    0.0%    0.0%    0.0%    0.0%    0.0%    0.0%    0.0%    0.0%    0.0%    0.0%    0.0%    0.0%    0.0%    0.0%    0.0%    0.0%    0.0%    0.0%    0.0%    0.0%    0.0%    0.0%    0.0%    0.0%    0.0%    0.0%    0.0%    0.0%    0.0%    0.0%    0.0%    0.0%    0.0%    0.0%    0.0%    0.0%    0.0%    0.0%    0.0%    0.0%    0.0%    0.0%    0.0%    0.0%    0.0%    0.0%    0.0%    0.0%    0.0%    0.0%    0.0%    0.0%    0.0%        k__Bacteria;p__Verrucomicrobia; c__       7    0.0%    0.0%    0.0%    0.0%    0.0%    0.0%    0.0%    0.0%    0.0%    0.0%    0.0%    0.0%    0.0%    0.0%    0.0%    0.0%    0.0%    0.0%    0.0%    0.0%    0.0%    0.0%    0.0%    0.0%    0.0%    0.0%    0.0%    0.0%    0.0%    0.0%    0.0%    0.0%    0.0%    0.0%    0.0%    0.0%    0.0%    0.0%    0.0%    0.0%    0.0%    0.0%    0.0%    0.0%    0.0%    0.0%    0.0%    0.0%    0.0%    0.0%    0.0%    0.0%    0.0%    0.0%    0.0%    0.0%    0.0%    0.0%    0.0%    0.0%    0.0%    0.0%    0.0%    0.0%    0.0%    0.0%    0.0%    0.0%    0.0%    0.0%    0.0%    0.0%    0.0%    0.0%    0.0%    0.0%    0.0%    0.0%    0.0%    0.0%    0.0%    0.0%    0.0%    0.0%    0.0%    0.0%    0.0%    0.0%    0.0%    0.0%    0.0%    0.0%    0.0%    0.0%    0.0%    0.0%    0.0%    0.0%    0.0%    0.0%    0.0%    0.0%    0.0%    0.0%    0.0%    0.0%    0.0%    0.0%    0.0%    0.0%    0.0%    0.0%    0.0%    0.0%    0.0%    0.0%    0.0%    0.0%    0.0%    0.0%    0.0%    0.0%    0.0%    0.0%    0.0%    0.0%    0.0%    0.0%    0.0%    0.0%    0.0%    0.0%    0.0%    0.0%        k__Bacteria;p__Verrucomicrobia; c__Opitutae      40    0.0%    0.0%    0.0%    0.0%    0.0%    0.0%    0.0%    0.0%    0.0%    0.0%    0.0%    0.0%    0.0%    0.0%    0.0%    0.0%    0.0%    0.0%    0.0%    0.0%    0.0%    0.0%    0.0%    0.0%    0.0%    0.0%    0.0%    0.0%    0.0%    0.0%    0.0%    0.0%    0.0%    0.0%    0.0%    0.0%    0.0%    0.0%    0.0%    0.0%    0.0%    0.0%    0.0%    0.0%    0.0%    0.0%    0.0%    0.0%    0.0%    0.0%    0.0%    0.0%    0.0%    0.0%    0.0%    0.0%    0.0%    0.0%    0.0%    0.0%    0.0%    0.0%    0.0%    0.0%    0.0%    0.0%    0.0%    0.0%    0.0%    0.0%    0.0%    0.0%    0.0%    0.0%    0.0%    0.0%    0.0%    0.0%    0.0%    0.0%    0.0%    0.0%    0.0%    0.0%    0.0%    0.0%    0.0%    0.0%    0.0%    0.0%    0.0%    0.0%    0.0%    0.0%    0.0%    0.0%    0.0%    0.0%    0.0%    0.0%    0.0%    0.0%    0.0%    0.0%    0.0%    0.0%    0.0%    0.0%    0.1%    0.1%    0.0%    0.0%    0.0%    0.0%    0.0%    0.0%    0.0%    0.0%    0.0%    0.0%    0.0%    0.0%    0.0%    0.0%    0.0%    0.0%    0.0%    0.0%    0.0%    0.0%    0.0%    0.0%    0.0%    0.0%        k__Bacteria;p__Verrucomicrobia; c__Spartobacteria     269    0.0%    0.0%    0.0%    0.0%    0.0%    0.1%    0.0%    0.0%    0.1%    0.0%    0.1%    0.0%    0.0%    0.0%    0.1%    0.0%    0.0%    0.1%    0.1%    0.0%    0.0%    0.0%    0.0%    0.0%    0.0%    0.0%    0.1%    0.0%    0.0%    0.0%    0.0%    0.0%    0.0%    0.1%    0.0%    0.0%    0.0%    0.0%    0.0%    0.0%    0.0%    0.0%    0.0%    0.0%    0.0%    0.0%    0.0%    0.0%    0.0%    0.0%    0.0%    0.0%    0.0%    0.1%    0.1%    0.0%    0.0%    0.0%    0.1%    0.1%    0.0%    0.0%    0.0%    0.0%    0.0%    0.0%    0.1%    0.1%    0.0%    0.0%    0.0%    0.0%    0.0%    0.0%    0.1%    0.0%    0.0%    0.1%    0.1%    0.0%    0.0%    0.0%    0.0%    0.0%    0.0%    0.1%    0.0%    0.0%    0.1%    0.1%    0.0%    0.0%    0.0%    0.0%    0.0%    0.1%    0.1%    0.1%    0.1%    0.0%    0.0%    0.0%    0.1%    0.0%    0.0%    0.0%    0.1%    0.0%    0.1%    0.1%    0.0%    0.1%    0.0%    0.0%    0.0%    0.0%    0.0%    0.0%    0.1%    0.0%    0.0%    0.0%    0.0%    0.0%    0.0%    0.0%    0.0%    0.0%    0.0%    0.0%    0.0%    0.0%    0.0%    0.0%        k__Bacteria;p__Verrucomicrobia; c__TP21       0    0.0%    0.0%    0.0%    0.0%    0.0%    0.0%    0.0%    0.0%    0.0%    0.0%    0.0%    0.0%    0.0%    0.0%    0.0%    0.0%    0.0%    0.0%    0.0%    0.0%    0.0%    0.0%    0.0%    0.0%    0.0%    0.0%    0.0%    0.0%    0.0%    0.0%    0.0%    0.0%    0.0%    0.0%    0.0%    0.0%    0.0%    0.0%    0.0%    0.0%    0.0%    0.0%    0.0%    0.0%    0.0%    0.0%    0.0%    0.0%    0.0%    0.0%    0.0%    0.0%    0.0%    0.0%    0.0%    0.0%    0.0%    0.0%    0.0%    0.0%    0.0%    0.0%    0.0%    0.0%    0.0%    0.0%    0.0%    0.0%    0.0%    0.0%    0.0%    0.0%    0.0%    0.0%    0.0%    0.0%    0.0%    0.0%    0.0%    0.0%    0.0%    0.0%    0.0%    0.0%    0.0%    0.0%    0.0%    0.0%    0.0%    0.0%    0.0%    0.0%    0.0%    0.0%    0.0%    0.0%    0.0%    0.0%    0.0%    0.0%    0.0%    0.0%    0.0%    0.0%    0.0%    0.0%    0.0%    0.0%    0.0%    0.0%    0.0%    0.0%    0.0%    0.0%    0.0%    0.0%    0.0%    0.0%    0.0%    0.0%    0.0%    0.0%    0.0%    0.0%    0.0%    0.0%    0.0%    0.0%    0.0%    0.0%    0.0%    0.0%    0.0%    0.0%        k__Bacteria;p__Verrucomicrobia; c__Verrucomicrobiae     975    0.1%    0.0%    0.1%    0.3%    0.1%    0.0%    0.1%    0.4%    0.0%    0.0%    0.0%    0.0%    0.0%    0.0%    0.1%    0.0%    0.1%    0.1%    0.1%    0.0%    0.0%    0.0%    0.0%    0.0%    0.0%    0.0%    0.4%    0.0%    0.0%    0.0%    0.1%    0.1%    0.1%    0.0%    4.4%    0.1%    0.0%    0.0%    0.0%    0.0%    0.0%    0.1%    0.0%    0.0%    0.0%    0.0%    0.0%    0.0%    0.0%    0.0%    0.1%    0.0%    0.0%    0.2%    0.1%    0.0%    0.0%    0.0%    0.1%    0.0%    0.1%    0.0%    0.0%    0.0%    0.1%    0.1%    0.1%    0.1%    0.1%    0.1%    0.0%    0.1%    0.2%    0.0%    0.2%    0.0%    0.0%    0.3%    0.0%    0.0%    0.0%    0.1%    0.0%    0.1%    0.1%    0.1%    0.0%    0.1%    0.1%    0.1%    0.0%    0.0%    0.0%    0.1%    0.1%    0.1%    0.0%    0.1%    0.1%    0.0%    0.0%    0.1%    0.0%    0.1%    0.1%    0.0%    0.0%    0.0%    0.7%    0.9%    0.1%    0.1%    0.1%    0.0%    0.0%    0.1%    0.1%    0.1%    0.0%    0.0%    0.0%    0.1%    0.0%    0.1%    0.1%    0.1%    0.1%    0.1%    0.0%    0.0%    0.1%    0.0%    0.0%    0.1%        k__Bacteria;p__WPS-2; c__       9    0.0%    0.0%    0.0%    0.0%    0.0%    0.0%    0.0%    0.0%    0.0%    0.0%    0.0%    0.0%    0.0%    0.0%    0.0%    0.0%    0.0%    0.0%    0.0%    0.0%    0.0%    0.0%    0.0%    0.0%    0.0%    0.0%    0.0%    0.0%    0.0%    0.0%    0.0%    0.0%    0.0%    0.0%    0.0%    0.0%    0.0%    0.0%    0.0%    0.0%    0.0%    0.0%    0.0%    0.0%    0.0%    0.0%    0.0%    0.0%    0.0%    0.0%    0.0%    0.0%    0.0%    0.0%    0.0%    0.0%    0.0%    0.0%    0.0%    0.0%    0.0%    0.0%    0.0%    0.0%    0.0%    0.0%    0.0%    0.0%    0.0%    0.0%    0.0%    0.0%    0.0%    0.0%    0.0%    0.0%    0.0%    0.0%    0.0%    0.0%    0.0%    0.0%    0.0%    0.0%    0.0%    0.0%    0.0%    0.0%    0.0%    0.0%    0.0%    0.0%    0.0%    0.0%    0.0%    0.0%    0.0%    0.0%    0.0%    0.0%    0.0%    0.0%    0.0%    0.0%    0.0%    0.0%    0.0%    0.0%    0.0%    0.0%    0.0%    0.0%    0.0%    0.0%    0.0%    0.0%    0.0%    0.0%    0.0%    0.0%    0.0%    0.0%    0.0%    0.0%    0.0%    0.0%    0.0%    0.0%    0.0%    0.0%    0.0%    0.0%    0.0%    0.0%        k__Bacteria;p__WS3; c__PRR-12       0    0.0%    0.0%    0.0%    0.0%    0.0%    0.0%    0.0%    0.0%    0.0%    0.0%    0.0%    0.0%    0.0%    0.0%    0.0%    0.0%    0.0%    0.0%    0.0%    0.0%    0.0%    0.0%    0.0%    0.0%    0.0%    0.0%    0.0%    0.0%    0.0%    0.0%    0.0%    0.0%    0.0%    0.0%    0.0%    0.0%    0.0%    0.0%    0.0%    0.0%    0.0%    0.0%    0.0%    0.0%    0.0%    0.0%    0.0%    0.0%    0.0%    0.0%    0.0%    0.0%    0.0%    0.0%    0.0%    0.0%    0.0%    0.0%    0.0%    0.0%    0.0%    0.0%    0.0%    0.0%    0.0%    0.0%    0.0%    0.0%    0.0%    0.0%    0.0%    0.0%    0.0%    0.0%    0.0%    0.0%    0.0%    0.0%    0.0%    0.0%    0.0%    0.0%    0.0%    0.0%    0.0%    0.0%    0.0%    0.0%    0.0%    0.0%    0.0%    0.0%    0.0%    0.0%    0.0%    0.0%    0.0%    0.0%    0.0%    0.0%    0.0%    0.0%    0.0%    0.0%    0.0%    0.0%    0.0%    0.0%    0.0%    0.0%    0.0%    0.0%    0.0%    0.0%    0.0%    0.0%    0.0%    0.0%    0.0%    0.0%    0.0%    0.0%    0.0%    0.0%    0.0%    0.0%    0.0%    0.0%    0.0%    0.0%    0.0%    0.0%    0.0%    0.0%        k__Bacteria;p__ZB2; c__       0    0.0%    0.0%    0.0%    0.0%    0.0%    0.0%    0.0%    0.0%    0.0%    0.0%    0.0%    0.0%    0.0%    0.0%    0.0%    0.0%    0.0%    0.0%    0.0%    0.0%    0.0%    0.0%    0.0%    0.0%    0.0%    0.0%    0.0%    0.0%    0.0%    0.0%    0.0%    0.0%    0.0%    0.0%    0.0%    0.0%    0.0%    0.0%    0.0%    0.0%    0.0%    0.0%    0.0%    0.0%    0.0%    0.0%    0.0%    0.0%    0.0%    0.0%    0.0%    0.0%    0.0%    0.0%    0.0%    0.0%    0.0%    0.0%    0.0%    0.0%    0.0%    0.0%    0.0%    0.0%    0.0%    0.0%    0.0%    0.0%    0.0%    0.0%    0.0%    0.0%    0.0%    0.0%    0.0%    0.0%    0.0%    0.0%    0.0%    0.0%    0.0%    0.0%    0.0%    0.0%    0.0%    0.0%    0.0%    0.0%    0.0%    0.0%    0.0%    0.0%    0.0%    0.0%    0.0%    0.0%    0.0%    0.0%    0.0%    0.0%    0.0%    0.0%    0.0%    0.0%    0.0%    0.0%    0.0%    0.0%    0.0%    0.0%    0.0%    0.0%    0.0%    0.0%    0.0%    0.0%    0.0%    0.0%    0.0%    0.0%    0.0%    0.0%    0.0%    0.0%    0.0%    0.0%    0.0%    0.0%    0.0%    0.0%    0.0%    0.0%    0.0%    0.0%    
     
  Taxonomy Summary. Current Level: Order  
     View Figure (.pdf)    View Legend (.pdf)   
  
[truncated: 3,493,821 more chars]
