# Supplementary material for: Moving pictures of the human microbiome
Source: Genome Biol. 2011 May 30;12(5):R50. doi: 10.1186/gb-2011-12-5-r50 (PMC3271711; doi:10.1186/gb-2011-12-5-r50)
Supplement: Additional file 11 — Temporal variation in phylum, class, order, family, and genus abundances (F4 tongue). The x-axis scale differs between M3 and F4 plots. [file ppHt6Jb6PoLnDmJTQSZSQikz8S05WR_legend.pdf › AdditionalFile11/charts/ppHt6Jb6PoLnDmJTQSZSQikz8S05WR_legend.pdf]

k__Archaea;p__Crenarchaeota;c__Thaumarchaeota;o__Nitrososphaerales;f__Nitrososphaeraceae;g__Candidatus Nitrososphaera
k__Bacteria;p__Acidobacteria;c__Chloracidobacteria;o__;f__;g__
k__Bacteria;p__Actinobacteria;c__;o__;f__;g__
k__Bacteria;p__Actinobacteria;c__Actinobacteria (class);o__Acidimicrobiales;f__;g__
k__Bacteria;p__Actinobacteria;c__Actinobacteria (class);o__Actinomycetales;f__;g__
k__Bacteria;p__Actinobacteria;c__Actinobacteria (class);o__Actinomycetales;f__;g__Tropheryma
k__Bacteria;p__Actinobacteria;c__Actinobacteria (class);o__Actinomycetales;f__Actinomycetaceae;g__
k__Bacteria;p__Actinobacteria;c__Actinobacteria (class);o__Actinomycetales;f__Actinomycetaceae;g__Actinobaculum
k__Bacteria;p__Actinobacteria;c__Actinobacteria (class);o__Actinomycetales;f__Actinomycetaceae;g__Actinomyces
k__Bacteria;p__Actinobacteria;c__Actinobacteria (class);o__Actinomycetales;f__Actinomycetaceae;g__Arcanobacterium
k__Bacteria;p__Actinobacteria;c__Actinobacteria (class);o__Actinomycetales;f__Actinomycetaceae;g__Mobiluncus
k__Bacteria;p__Actinobacteria;c__Actinobacteria (class);o__Actinomycetales;f__Actinomycetaceae;g__Varibaculum
k__Bacteria;p__Actinobacteria;c__Actinobacteria (class);o__Actinomycetales;f__Actinosynnemataceae;g__Saccharothrix
k__Bacteria;p__Actinobacteria;c__Actinobacteria (class);o__Actinomycetales;f__Beutenbergiaceae;g__
k__Bacteria;p__Actinobacteria;c__Actinobacteria (class);o__Actinomycetales;f__Bogoriellaceae;g__Georgenia
k__Bacteria;p__Actinobacteria;c__Actinobacteria (class);o__Actinomycetales;f__Brevibacteriaceae;g__Brevibacterium
k__Bacteria;p__Actinobacteria;c__Actinobacteria (class);o__Actinomycetales;f__Cellulomonadaceae;g__Actinotalea
k__Bacteria;p__Actinobacteria;c__Actinobacteria (class);o__Actinomycetales;f__Cellulomonadaceae;g__Cellulomonas
k__Bacteria;p__Actinobacteria;c__Actinobacteria (class);o__Actinomycetales;f__Corynebacteriaceae;g__
k__Bacteria;p__Actinobacteria;c__Actinobacteria (class);o__Actinomycetales;f__Corynebacteriaceae;g__Corynebacterium
k__Bacteria;p__Actinobacteria;c__Actinobacteria (class);o__Actinomycetales;f__Dermabacteraceae;g__
k__Bacteria;p__Actinobacteria;c__Actinobacteria (class);o__Actinomycetales;f__Dermabacteraceae;g__Brachybacterium
k__Bacteria;p__Actinobacteria;c__Actinobacteria (class);o__Actinomycetales;f__Dermabacteraceae;g__Dermabacter
k__Bacteria;p__Actinobacteria;c__Actinobacteria (class);o__Actinomycetales;f__Dermacoccaceae;g__Dermacoccus
k__Bacteria;p__Actinobacteria;c__Actinobacteria (class);o__Actinomycetales;f__Dermatophilaceae;g__
k__Bacteria;p__Actinobacteria;c__Actinobacteria (class);o__Actinomycetales;f__Dermatophilaceae;g__Dermatophilus
k__Bacteria;p__Actinobacteria;c__Actinobacteria (class);o__Actinomycetales;f__Geodermatophilaceae;g__
k__Bacteria;p__Actinobacteria;c__Actinobacteria (class);o__Actinomycetales;f__Geodermatophilaceae;g__Blastococcus
k__Bacteria;p__Actinobacteria;c__Actinobacteria (class);o__Actinomycetales;f__Geodermatophilaceae;g__Geodermatophilus
k__Bacteria;p__Actinobacteria;c__Actinobacteria (class);o__Actinomycetales;f__Geodermatophilaceae;g__Modestobacter
k__Bacteria;p__Actinobacteria;c__Actinobacteria (class);o__Actinomycetales;f__Intrasporangiaceae;g__
k__Bacteria;p__Actinobacteria;c__Actinobacteria (class);o__Actinomycetales;f__Intrasporangiaceae;g__Knoellia
k__Bacteria;p__Actinobacteria;c__Actinobacteria (class);o__Actinomycetales;f__Intrasporangiaceae;g__Phycicoccus
k__Bacteria;p__Actinobacteria;c__Actinobacteria (class);o__Actinomycetales;f__Intrasporangiaceae;g__Serinicoccus
k__Bacteria;p__Actinobacteria;c__Actinobacteria (class);o__Actinomycetales;f__Intrasporangiaceae;g__Terracoccus
k__Bacteria;p__Actinobacteria;c__Actinobacteria (class);o__Actinomycetales;f__Microbacteriaceae;g__
k__Bacteria;p__Actinobacteria;c__Actinobacteria (class);o__Actinomycetales;f__Microbacteriaceae;g__Curtobacterium
k__Bacteria;p__Actinobacteria;c__Actinobacteria (class);o__Actinomycetales;f__Microbacteriaceae;g__Microbacterium
k__Bacteria;p__Actinobacteria;c__Actinobacteria (class);o__Actinomycetales;f__Micrococcaceae;g__
k__Bacteria;p__Actinobacteria;c__Actinobacteria (class);o__Actinomycetales;f__Micrococcaceae;g__Arthrobacter
k__Bacteria;p__Actinobacteria;c__Actinobacteria (class);o__Actinomycetales;f__Micrococcaceae;g__Citricoccus
k__Bacteria;p__Actinobacteria;c__Actinobacteria (class);o__Actinomycetales;f__Micrococcaceae;g__Kocuria
k__Bacteria;p__Actinobacteria;c__Actinobacteria (class);o__Actinomycetales;f__Micrococcaceae;g__Nesterenkonia
k__Bacteria;p__Actinobacteria;c__Actinobacteria (class);o__Actinomycetales;f__Micrococcaceae;g__Renibacterium
k__Bacteria;p__Actinobacteria;c__Actinobacteria (class);o__Actinomycetales;f__Micrococcaceae;g__Rothia
k__Bacteria;p__Actinobacteria;c__Actinobacteria (class);o__Actinomycetales;f__Micrococcaceae;g__Zhihengliuella
k__Bacteria;p__Actinobacteria;c__Actinobacteria (class);o__Actinomycetales;f__Mycobacteriaceae;g__Mycobacterium
k__Bacteria;p__Actinobacteria;c__Actinobacteria (class);o__Actinomycetales;f__Nakamurellaceae;g__
k__Bacteria;p__Actinobacteria;c__Actinobacteria (class);o__Actinomycetales;f__Nocardiaceae;g__Rhodococcus
k__Bacteria;p__Actinobacteria;c__Actinobacteria (class);o__Actinomycetales;f__Nocardioidaceae;g__
k__Bacteria;p__Actinobacteria;c__Actinobacteria (class);o__Actinomycetales;f__Nocardioidaceae;g__Aeromicrobium
k__Bacteria;p__Actinobacteria;c__Actinobacteria (class);o__Actinomycetales;f__Nocardioidaceae;g__Nocardioides
k__Bacteria;p__Actinobacteria;c__Actinobacteria (class);o__Actinomycetales;f__Nocardiopsaceae;g__
k__Bacteria;p__Actinobacteria;c__Actinobacteria (class);o__Actinomycetales;f__Nocardiopsaceae;g__Nocardiopsis
k__Bacteria;p__Actinobacteria;c__Actinobacteria (class);o__Actinomycetales;f__Promicromonosporaceae;g__
k__Bacteria;p__Actinobacteria;c__Actinobacteria (class);o__Actinomycetales;f__Propionibacteriaceae;g__Propionibacterium
k__Bacteria;p__Actinobacteria;c__Actinobacteria (class);o__Actinomycetales;f__Streptomycetaceae;g__Streptomyces
k__Bacteria;p__Actinobacteria;c__Actinobacteria (class);o__Actinomycetales;f__Thermomonosporaceae;g__Actinomadura
k__Bacteria;p__Actinobacteria;c__Actinobacteria (class);o__Actinomycetales;f__Thermomonosporaceae;g__Thermomonospora
k__Bacteria;p__Actinobacteria;c__Actinobacteria (class);o__Actinomycetales;f__Tsukamurellaceae;g__Tsukamurella
k__Bacteria;p__Actinobacteria;c__Actinobacteria (class);o__Bifidobacteriales;f__Bifidobacteriaceae;g__Alloscardovia
k__Bacteria;p__Actinobacteria;c__Actinobacteria (class);o__Bifidobacteriales;f__Bifidobacteriaceae;g__Bifidobacterium
k__Bacteria;p__Actinobacteria;c__Actinobacteria (class);o__Bifidobacteriales;f__Bifidobacteriaceae;g__Scardovia
k__Bacteria;p__Actinobacteria;c__Actinobacteria (class);o__Coriobacteriales;f__Coriobacteriaceae;g__
k__Bacteria;p__Actinobacteria;c__Actinobacteria (class);o__Coriobacteriales;f__Coriobacteriaceae;g__Atopobium
k__Bacteria;p__Actinobacteria;c__Actinobacteria (class);o__Coriobacteriales;f__Coriobacteriaceae;g__Olsenella
k__Bacteria;p__Actinobacteria;c__Actinobacteria (class);o__Coriobacteriales;f__Coriobacteriaceae;g__Slackia
k__Bacteria;p__Actinobacteria;c__Actinobacteria (class);o__MC47;f__;g__
k__Bacteria;p__Actinobacteria;c__Actinobacteria (class);o__Solirubrobacterales;f__Solirubrobacteraceae;g__
k__Bacteria;p__Bacteroidetes;c__Bacteroidia;o__Bacteroidales;f__;g__
k__Bacteria;p__Bacteroidetes;c__Bacteroidia;o__Bacteroidales;f__Bacteroidaceae;g__Bacteroides
k__Bacteria;p__Bacteroidetes;c__Bacteroidia;o__Bacteroidales;f__Porphyromonadaceae;g__
k__Bacteria;p__Bacteroidetes;c__Bacteroidia;o__Bacteroidales;f__Porphyromonadaceae;g__Paludibacter
k__Bacteria;p__Bacteroidetes;c__Bacteroidia;o__Bacteroidales;f__Porphyromonadaceae;g__Parabacteroides
k__Bacteria;p__Bacteroidetes;c__Bacteroidia;o__Bacteroidales;f__Porphyromonadaceae;g__Porphyromonas
k__Bacteria;p__Bacteroidetes;c__Bacteroidia;o__Bacteroidales;f__Porphyromonadaceae;g__Tannerella
k__Bacteria;p__Bacteroidetes;c__Bacteroidia;o__Bacteroidales;f__Prevotellaceae;g__
k__Bacteria;p__Bacteroidetes;c__Bacteroidia;o__Bacteroidales;f__Prevotellaceae;g__Prevotella
k__Bacteria;p__Bacteroidetes;c__Bacteroidia;o__Bacteroidales;f__Rikenellaceae;g__Alistipes
k__Bacteria;p__Bacteroidetes;c__Flavobacteria;o__;f__;g__
k__Bacteria;p__Bacteroidetes;c__Flavobacteria;o__Flavobacteriales;f__Flavobacteriaceae;g__
k__Bacteria;p__Bacteroidetes;c__Flavobacteria;o__Flavobacteriales;f__Flavobacteriaceae;g__Bergeyella
k__Bacteria;p__Bacteroidetes;c__Flavobacteria;o__Flavobacteriales;f__Flavobacteriaceae;g__Capnocytophaga
k__Bacteria;p__Bacteroidetes;c__Flavobacteria;o__Flavobacteriales;f__Flavobacteriaceae;g__Chryseobacterium
k__Bacteria;p__Bacteroidetes;c__Flavobacteria;o__Flavobacteriales;f__Flavobacteriaceae;g__Flavobacterium
k__Bacteria;p__Bacteroidetes;c__Flavobacteria;o__Flavobacteriales;f__Flavobacteriaceae;g__Kaistella
k__Bacteria;p__Bacteroidetes;c__Flavobacteria;o__Flavobacteriales;f__Flavobacteriaceae;g__Wautersiella
k__Bacteria;p__Bacteroidetes;c__Sphingobacteria;o__Sphingobacteriales;f__;g__
k__Bacteria;p__Bacteroidetes;c__Sphingobacteria;o__Sphingobacteriales;f__;g__Segetibacter
k__Bacteria;p__Bacteroidetes;c__Sphingobacteria;o__Sphingobacteriales;f__Cyclobacteriaceae;g__Cyclobacterium
k__Bacteria;p__Bacteroidetes;c__Sphingobacteria;o__Sphingobacteriales;f__Flexibacteraceae;g__
k__Bacteria;p__Bacteroidetes;c__Sphingobacteria;o__Sphingobacteriales;f__Flexibacteraceae;g__Dyadobacter
k__Bacteria;p__Bacteroidetes;c__Sphingobacteria;o__Sphingobacteriales;f__Flexibacteraceae;g__Flectobacillus
k__Bacteria;p__Bacteroidetes;c__Sphingobacteria;o__Sphingobacteriales;f__Flexibacteraceae;g__Hymenobacter
k__Bacteria;p__Bacteroidetes;c__Sphingobacteria;o__Sphingobacteriales;f__Flexibacteraceae;g__Pontibacter
k__Bacteria;p__Bacteroidetes;c__Sphingobacteria;o__Sphingobacteriales;f__Sphingobacteriaceae;g__
k__Bacteria;p__Bacteroidetes;c__Sphingobacteria;o__Sphingobacteriales;f__Sphingobacteriaceae;g__Pedobacter
k__Bacteria;p__Bacteroidetes;c__Sphingobacteria;o__Sphingobacteriales;f__Sphingobacteriaceae;g__Sphingobacterium
k__Bacteria;p__Chloroflexi;c__Bljii12;o__;f__;g__
k__Bacteria;p__Chloroflexi;c__SOGA31;o__;f__;g__
k__Bacteria;p__Chloroflexi;c__Thermomicrobia;o__HN1-15;f__;g__
k__Bacteria;p__Cyanobacteria;c__;o__;f__;g__
k__Bacteria;p__Firmicutes;c__Bacilli;o__;f__;g__
k__Bacteria;p__Firmicutes;c__Bacilli;o__Bacillales;f__;g__
k__Bacteria;p__Firmicutes;c__Bacilli;o__Bacillales;f__;g__Exiguobacterium
k__Bacteria;p__Firmicutes;c__Bacilli;o__Bacillales;f__;g__Gemella
k__Bacteria;p__Firmicutes;c__Bacilli;o__Bacillales;f__Alicyclobacillaceae;g__Alicyclobacillus
k__Bacteria;p__Firmicutes;c__Bacilli;o__Bacillales;f__Bacillaceae;g__
k__Bacteria;p__Firmicutes;c__Bacilli;o__Bacillales;f__Bacillaceae;g__Anaerobacillus
k__Bacteria;p__Firmicutes;c__Bacilli;o__Bacillales;f__Bacillaceae;g__Anoxybacillus
k__Bacteria;p__Firmicutes;c__Bacilli;o__Bacillales;f__Bacillaceae;g__Bacillus
k__Bacteria;p__Firmicutes;c__Bacilli;o__Bacillales;f__Bacillaceae;g__Geobacillus
k__Bacteria;p__Firmicutes;c__Bacilli;o__Bacillales;f__Bacillaceae;g__Natronobacillus
k__Bacteria;p__Firmicutes;c__Bacilli;o__Bacillales;f__Bacillaceae;g__Pontibacillus
k__Bacteria;p__Firmicutes;c__Bacilli;o__Bacillales;f__Bacillaceae;g__Virgibacillus
k__Bacteria;p__Firmicutes;c__Bacilli;o__Bacillales;f__Listeriaceae;g__
k__Bacteria;p__Firmicutes;c__Bacilli;o__Bacillales;f__Listeriaceae;g__Brochothrix
k__Bacteria;p__Firmicutes;c__Bacilli;o__Bacillales;f__Paenibacillaceae;g__Ammoniphilus
k__Bacteria;p__Firmicutes;c__Bacilli;o__Bacillales;f__Paenibacillaceae;g__Paenibacillus
k__Bacteria;p__Firmicutes;c__Bacilli;o__Bacillales;f__Planococcaceae;g__
k__Bacteria;p__Firmicutes;c__Bacilli;o__Bacillales;f__Planococcaceae;g__Kurthia
k__Bacteria;p__Firmicutes;c__Bacilli;o__Bacillales;f__Planococcaceae;g__Lysinibacillus
k__Bacteria;p__Firmicutes;c__Bacilli;o__Bacillales;f__Planococcaceae;g__Marinibacillus
k__Bacteria;p__Firmicutes;c__Bacilli;o__Bacillales;f__Planococcaceae;g__Solibacillus
k__Bacteria;p__Firmicutes;c__Bacilli;o__Bacillales;f__Planococcaceae;g__Sporosarcina
k__Bacteria;p__Firmicutes;c__Bacilli;o__Bacillales;f__Planococcaceae;g__Ureibacillus
k__Bacteria;p__Firmicutes;c__Bacilli;o__Bacillales;f__Staphylococcaceae;g__
k__Bacteria;p__Firmicutes;c__Bacilli;o__Bacillales;f__Staphylococcaceae;g__Jeotgalicoccus
k__Bacteria;p__Firmicutes;c__Bacilli;o__Bacillales;f__Staphylococcaceae;g__Macrococcus
k__Bacteria;p__Firmicutes;c__Bacilli;o__Bacillales;f__Staphylococcaceae;g__Salinicoccus
k__Bacteria;p__Firmicutes;c__Bacilli;o__Bacillales;f__Staphylococcaceae;g__Staphylococcus
k__Bacteria;p__Firmicutes;c__Bacilli;o__Lactobacillales;f__;g__
k__Bacteria;p__Firmicutes;c__Bacilli;o__Lactobacillales;f__Aerococcaceae;g__Abiotrophia
k__Bacteria;p__Firmicutes;c__Bacilli;o__Lactobacillales;f__Aerococcaceae;g__Aerococcus
k__Bacteria;p__Firmicutes;c__Bacilli;o__Lactobacillales;f__Aerococcaceae;g__Facklamia
k__Bacteria;p__Firmicutes;c__Bacilli;o__Lactobacillales;f__Carnobacteriaceae;g__
k__Bacteria;p__Firmicutes;c__Bacilli;o__Lactobacillales;f__Carnobacteriaceae;g__Alloiococcus
k__Bacteria;p__Firmicutes;c__Bacilli;o__Lactobacillales;f__Carnobacteriaceae;g__Carnobacterium
k__Bacteria;p__Firmicutes;c__Bacilli;o__Lactobacillales;f__Carnobacteriaceae;g__Desemzia
k__Bacteria;p__Firmicutes;c__Bacilli;o__Lactobacillales;f__Carnobacteriaceae;g__Granulicatella
k__Bacteria;p__Firmicutes;c__Bacilli;o__Lactobacillales;f__Carnobacteriaceae;g__Marinilactibacillus
k__Bacteria;p__Firmicutes;c__Bacilli;o__Lactobacillales;f__Carnobacteriaceae;g__Trichococcus
k__Bacteria;p__Firmicutes;c__Bacilli;o__Lactobacillales;f__Enterococcaceae;g__
k__Bacteria;p__Firmicutes;c__Bacilli;o__Lactobacillales;f__Enterococcaceae;g__Enterococcus
k__Bacteria;p__Firmicutes;c__Bacilli;o__Lactobacillales;f__Enterococcaceae;g__Melissococcus
k__Bacteria;p__Firmicutes;c__Bacilli;o__Lactobacillales;f__Enterococcaceae;g__Tetragenococcus
k__Bacteria;p__Firmicutes;c__Bacilli;o__Lactobacillales;f__Enterococcaceae;g__Vagococcus
k__Bacteria;p__Firmicutes;c__Bacilli;o__Lactobacillales;f__Lactobacillaceae;g__
k__Bacteria;p__Firmicutes;c__Bacilli;o__Lactobacillales;f__Lactobacillaceae;g__Lactobacillus
k__Bacteria;p__Firmicutes;c__Bacilli;o__Lactobacillales;f__Lactobacillaceae;g__Pediococcus
k__Bacteria;p__Firmicutes;c__Bacilli;o__Lactobacillales;f__Leuconostocaceae;g__
k__Bacteria;p__Firmicutes;c__Bacilli;o__Lactobacillales;f__Leuconostocaceae;g__Fructobacillus
k__Bacteria;p__Firmicutes;c__Bacilli;o__Lactobacillales;f__Leuconostocaceae;g__Leuconostoc
k__Bacteria;p__Firmicutes;c__Bacilli;o__Lactobacillales;f__Leuconostocaceae;g__Weissella
k__Bacteria;p__Firmicutes;c__Bacilli;o__Lactobacillales;f__Streptococcaceae;g__
k__Bacteria;p__Firmicutes;c__Bacilli;o__Lactobacillales;f__Streptococcaceae;g__Lactococcus
k__Bacteria;p__Firmicutes;c__Bacilli;o__Lactobacillales;f__Streptococcaceae;g__Streptococcus
k__Bacteria;p__Firmicutes;c__Clostridia;o__Clostridiales;f__;g__
k__Bacteria;p__Firmicutes;c__Clostridia;o__Clostridiales;f__Catabacteriaceae;g__
k__Bacteria;p__Firmicutes;c__Clostridia;o__Clostridiales;f__Clostridiaceae;g__Clostridium
k__Bacteria;p__Firmicutes;c__Clostridia;o__Clostridiales;f__Clostridiales Family XI. Incertae Sedis;g__
k__Bacteria;p__Firmicutes;c__Clostridia;o__Clostridiales;f__Clostridiales Family XI. Incertae Sedis;g__Anaerococcus
k__Bacteria;p__Firmicutes;c__Clostridia;o__Clostridiales;f__Clostridiales Family XI. Incertae Sedis;g__Finegoldia
k__Bacteria;p__Firmicutes;c__Clostridia;o__Clostridiales;f__Clostridiales Family XI. Incertae Sedis;g__Helcococcus
k__Bacteria;p__Firmicutes;c__Clostridia;o__Clostridiales;f__Clostridiales Family XI. Incertae Sedis;g__Peptoniphilus
k__Bacteria;p__Firmicutes;c__Clostridia;o__Clostridiales;f__Clostridiales Family XIII. Incertae Sedis;g__
k__Bacteria;p__Firmicutes;c__Clostridia;o__Clostridiales;f__Clostridiales Family XIII. Incertae Sedis;g__Eubacterium
k__Bacteria;p__Firmicutes;c__Clostridia;o__Clostridiales;f__Clostridiales Family XIII. Incertae Sedis;g__Mogibacterium
k__Bacteria;p__Firmicutes;c__Clostridia;o__Clostridiales;f__Eubacteriaceae;g__Eubacterium
k__Bacteria;p__Firmicutes;c__Clostridia;o__Clostridiales;f__Lachnospiraceae;g__
k__Bacteria;p__Firmicutes;c__Clostridia;o__Clostridiales;f__Lachnospiraceae;g__Blautia
k__Bacteria;p__Firmicutes;c__Clostridia;o__Clostridiales;f__Lachnospiraceae;g__Butyrivibrio
k__Bacteria;p__Firmicutes;c__Clostridia;o__Clostridiales;f__Lachnospiraceae;g__Catonella
k__Bacteria;p__Firmicutes;c__Clostridia;o__Clostridiales;f__Lachnospiraceae;g__Clostridium
k__Bacteria;p__Firmicutes;c__Clostridia;o__Clostridiales;f__Lachnospiraceae;g__Coprococcus
k__Bacteria;p__Firmicutes;c__Clostridia;o__Clostridiales;f__Lachnospiraceae;g__Eubacterium
k__Bacteria;p__Firmicutes;c__Clostridia;o__Clostridiales;f__Lachnospiraceae;g__Johnsonella
k__Bacteria;p__Firmicutes;c__Clostridia;o__Clostridiales;f__Lachnospiraceae;g__Lachnobacterium
k__Bacteria;p__Firmicutes;c__Clostridia;o__Clostridiales;f__Lachnospiraceae;g__Lachnospira
k__Bacteria;p__Firmicutes;c__Clostridia;o__Clostridiales;f__Lachnospiraceae;g__Moryella
k__Bacteria;p__Firmicutes;c__Clostridia;o__Clostridiales;f__Lachnospiraceae;g__Oribacterium
k__Bacteria;p__Firmicutes;c__Clostridia;o__Clostridiales;f__Lachnospiraceae;g__Roseburia
k__Bacteria;p__Firmicutes;c__Clostridia;o__Clostridiales;f__Lachnospiraceae;g__Ruminococcus
k__Bacteria;p__Firmicutes;c__Clostridia;o__Clostridiales;f__Lachnospiraceae;g__Shuttleworthia
k__Bacteria;p__Firmicutes;c__Clostridia;o__Clostridiales;f__Peptococcaceae;g__Peptococcus
k__Bacteria;p__Firmicutes;c__Clostridia;o__Clostridiales;f__Peptostreptococcaceae;g__Filifactor
k__Bacteria;p__Firmicutes;c__Clostridia;o__Clostridiales;f__Peptostreptococcaceae;g__Peptostreptococcus
k__Bacteria;p__Firmicutes;c__Clostridia;o__Clostridiales;f__Ruminococcaceae;g__
k__Bacteria;p__Firmicutes;c__Clostridia;o__Clostridiales;f__Ruminococcaceae;g__Bacteroides
k__Bacteria;p__Firmicutes;c__Clostridia;o__Clostridiales;f__Ruminococcaceae;g__Eubacterium
k__Bacteria;p__Firmicutes;c__Clostridia;o__Clostridiales;f__Ruminococcaceae;g__Faecalibacterium
k__Bacteria;p__Firmicutes;c__Clostridia;o__Clostridiales;f__Ruminococcaceae;g__Oscillospira
k__Bacteria;p__Firmicutes;c__Clostridia;o__Clostridiales;f__Ruminococcaceae;g__Ruminococcus
k__Bacteria;p__Firmicutes;c__Clostridia;o__Clostridiales;f__Veillonellaceae;g__
k__Bacteria;p__Firmicutes;c__Clostridia;o__Clostridiales;f__Veillonellaceae;g__Anaeroglobus
k__Bacteria;p__Firmicutes;c__Clostridia;o__Clostridiales;f__Veillonellaceae;g__Dialister
k__Bacteria;p__Firmicutes;c__Clostridia;o__Clostridiales;f__Veillonellaceae;g__Megamonas
k__Bacteria;p__Firmicutes;c__Clostridia;o__Clostridiales;f__Veillonellaceae;g__Phascolarctobacterium
k__Bacteria;p__Firmicutes;c__Clostridia;o__Clostridiales;f__Veillonellaceae;g__Selenomonas
k__Bacteria;p__Firmicutes;c__Clostridia;o__Clostridiales;f__Veillonellaceae;g__Veillonella
k__Bacteria;p__Firmicutes;c__Clostridia;o__MBA08;f__;g__
k__Bacteria;p__Fusobacteria;c__Fusobacteria (class);o__Fusobacteriales;f__Fusobacteriaceae;g__Fusobacterium
k__Bacteria;p__Fusobacteria;c__Fusobacteria (class);o__Fusobacteriales;f__Fusobacteriaceae;g__J2-29
k__Bacteria;p__Fusobacteria;c__Fusobacteria (class);o__Fusobacteriales;f__Fusobacteriaceae;g__Leptotrichia
k__Bacteria;p__Gemmatimonadetes;c__Gemmatimonadetes (class);o__Gemmatimonadales;f__;g__
k__Bacteria;p__Proteobacteria;c__Alphaproteobacteria;o__Caulobacterales;f__Caulobacteraceae;g__
k__Bacteria;p__Proteobacteria;c__Alphaproteobacteria;o__Caulobacterales;f__Caulobacteraceae;g__Brevundimonas
k__Bacteria;p__Proteobacteria;c__Alphaproteobacteria;o__Caulobacterales;f__Caulobacteraceae;g__Caulobacter
k__Bacteria;p__Proteobacteria;c__Alphaproteobacteria;o__Caulobacterales;f__Caulobacteraceae;g__Phenylobacterium
k__Bacteria;p__Proteobacteria;c__Alphaproteobacteria;o__Rhizobiales;f__;g__
k__Bacteria;p__Proteobacteria;c__Alphaproteobacteria;o__Rhizobiales;f__Bradyrhizobiaceae;g__
k__Bacteria;p__Proteobacteria;c__Alphaproteobacteria;o__Rhizobiales;f__Bradyrhizobiaceae;g__Afipia
k__Bacteria;p__Proteobacteria;c__Alphaproteobacteria;o__Rhizobiales;f__Bradyrhizobiaceae;g__Balneimonas
k__Bacteria;p__Proteobacteria;c__Alphaproteobacteria;o__Rhizobiales;f__Bradyrhizobiaceae;g__Bosea
k__Bacteria;p__Proteobacteria;c__Alphaproteobacteria;o__Rhizobiales;f__Bradyrhizobiaceae;g__Bradyrhizobium
k__Bacteria;p__Proteobacteria;c__Alphaproteobacteria;o__Rhizobiales;f__Brucellaceae;g__Pseudochrobactrum
k__Bacteria;p__Proteobacteria;c__Alphaproteobacteria;o__Rhizobiales;f__Hyphomicrobiaceae;g__Rhodoplanes
k__Bacteria;p__Proteobacteria;c__Alphaproteobacteria;o__Rhizobiales;f__Methylobacteriaceae;g__Methylobacterium
k__Bacteria;p__Proteobacteria;c__Alphaproteobacteria;o__Rhizobiales;f__Phyllobacteriaceae;g__
k__Bacteria;p__Proteobacteria;c__Alphaproteobacteria;o__Rhizobiales;f__Phyllobacteriaceae;g__Phyllobacterium
k__Bacteria;p__Proteobacteria;c__Alphaproteobacteria;o__Rhizobiales;f__Rhizobiaceae;g__
k__Bacteria;p__Proteobacteria;c__Alphaproteobacteria;o__Rhizobiales;f__Rhizobiaceae;g__Agrobacterium
k__Bacteria;p__Proteobacteria;c__Alphaproteobacteria;o__Rhizobiales;f__Rhizobiaceae;g__Mycoplana
k__Bacteria;p__Proteobacteria;c__Alphaproteobacteria;o__Rhizobiales;f__Rhizobiaceae;g__Rhizobium
k__Bacteria;p__Proteobacteria;c__Alphaproteobacteria;o__Rhizobiales;f__Xanthobacteraceae;g__Ancylobacter
k__Bacteria;p__Proteobacteria;c__Alphaproteobacteria;o__Rhodobacterales;f__Rhodobacteraceae;g__
k__Bacteria;p__Proteobacteria;c__Alphaproteobacteria;o__Rhodobacterales;f__Rhodobacteraceae;g__Paracoccus
k__Bacteria;p__Proteobacteria;c__Alphaproteobacteria;o__Rhodobacterales;f__Rhodobacteraceae;g__Rubellimicrobium
k__Bacteria;p__Proteobacteria;c__Alphaproteobacteria;o__Rhodobacterales;f__Rhodobacteraceae;g__Thioclava
k__Bacteria;p__Proteobacteria;c__Alphaproteobacteria;o__Rhodospirillales;f__Acetobacteraceae;g__
k__Bacteria;p__Proteobacteria;c__Alphaproteobacteria;o__Rhodospirillales;f__Acetobacteraceae;g__Asaia
k__Bacteria;p__Proteobacteria;c__Alphaproteobacteria;o__Rhodospirillales;f__Acetobacteraceae;g__Gluconobacter
k__Bacteria;p__Proteobacteria;c__Alphaproteobacteria;o__Rhodospirillales;f__Acetobacteraceae;g__Roseomonas
k__Bacteria;p__Proteobacteria;c__Alphaproteobacteria;o__Rhodospirillales;f__Rhodospirillaceae;g__Azospirillum
k__Bacteria;p__Proteobacteria;c__Alphaproteobacteria;o__Rhodospirillales;f__Rhodospirillaceae;g__Skermanella
k__Bacteria;p__Proteobacteria;c__Alphaproteobacteria;o__Sphingomonadales;f__;g__
k__Bacteria;p__Proteobacteria;c__Alphaproteobacteria;o__Sphingomonadales;f__Erythrobacteraceae;g__Erythromicrobium
k__Bacteria;p__Proteobacteria;c__Alphaproteobacteria;o__Sphingomonadales;f__Sphingomonadaceae;g__
k__Bacteria;p__Proteobacteria;c__Alphaproteobacteria;o__Sphingomonadales;f__Sphingomonadaceae;g__Blastomonas
k__Bacteria;p__Proteobacteria;c__Alphaproteobacteria;o__Sphingomonadales;f__Sphingomonadaceae;g__Kaistobacter
k__Bacteria;p__Proteobacteria;c__Alphaproteobacteria;o__Sphingomonadales;f__Sphingomonadaceae;g__Novosphingobium
k__Bacteria;p__Proteobacteria;c__Alphaproteobacteria;o__Sphingomonadales;f__Sphingomonadaceae;g__Sphingobium
k__Bacteria;p__Proteobacteria;c__Alphaproteobacteria;o__Sphingomonadales;f__Sphingomonadaceae;g__Sphingomonas
k__Bacteria;p__Proteobacteria;c__Betaproteobacteria;o__;f__;g__
k__Bacteria;p__Proteobacteria;c__Betaproteobacteria;o__Burkholderiales;f__;g__
k__Bacteria;p__Proteobacteria;c__Betaproteobacteria;o__Burkholderiales;f__;g__Aquabacterium
k__Bacteria;p__Proteobacteria;c__Betaproteobacteria;o__Burkholderiales;f__;g__Ideonella
k__Bacteria;p__Proteobacteria;c__Betaproteobacteria;o__Burkholderiales;f__;g__Methylibium
k__Bacteria;p__Proteobacteria;c__Betaproteobacteria;o__Burkholderiales;f__;g__Paucibacter
k__Bacteria;p__Proteobacteria;c__Betaproteobacteria;o__Burkholderiales;f__;g__Rubrivivax
k__Bacteria;p__Proteobacteria;c__Betaproteobacteria;o__Burkholderiales;f__;g__Tepidimonas
k__Bacteria;p__Proteobacteria;c__Betaproteobacteria;o__Burkholderiales;f__Alcaligenaceae;g__
k__Bacteria;p__Proteobacteria;c__Betaproteobacteria;o__Burkholderiales;f__Alcaligenaceae;g__Achromobacter
k__Bacteria;p__Proteobacteria;c__Betaproteobacteria;o__Burkholderiales;f__Alcaligenaceae;g__Alcaligenes
k__Bacteria;p__Proteobacteria;c__Betaproteobacteria;o__Burkholderiales;f__Alcaligenaceae;g__Azohydromonas
k__Bacteria;p__Proteobacteria;c__Betaproteobacteria;o__Burkholderiales;f__Burkholderiaceae;g__
k__Bacteria;p__Proteobacteria;c__Betaproteobacteria;o__Burkholderiales;f__Burkholderiaceae;g__Burkholderia
k__Bacteria;p__Proteobacteria;c__Betaproteobacteria;o__Burkholderiales;f__Burkholderiaceae;g__Cupriavidus
k__Bacteria;p__Proteobacteria;c__Betaproteobacteria;o__Burkholderiales;f__Burkholderiaceae;g__Lautropia
k__Bacteria;p__Proteobacteria;c__Betaproteobacteria;o__Burkholderiales;f__Burkholderiaceae;g__Pandoraea
k__Bacteria;p__Proteobacteria;c__Betaproteobacteria;o__Burkholderiales;f__Burkholderiaceae;g__Ralstonia
k__Bacteria;p__Proteobacteria;c__Betaproteobacteria;o__Burkholderiales;f__Comamonadaceae;g__
k__Bacteria;p__Proteobacteria;c__Betaproteobacteria;o__Burkholderiales;f__Comamonadaceae;g__Acidovorax
k__Bacteria;p__Proteobacteria;c__Betaproteobacteria;o__Burkholderiales;f__Comamonadaceae;g__Aquamonas
k__Bacteria;p__Proteobacteria;c__Betaproteobacteria;o__Burkholderiales;f__Comamonadaceae;g__Brachymonas
k__Bacteria;p__Proteobacteria;c__Betaproteobacteria;o__Burkholderiales;f__Comamonadaceae;g__Comamonas
k__Bacteria;p__Proteobacteria;c__Betaproteobacteria;o__Burkholderiales;f__Comamonadaceae;g__Delftia
k__Bacteria;p__Proteobacteria;c__Betaproteobacteria;o__Burkholderiales;f__Comamonadaceae;g__Hylemonella
k__Bacteria;p__Proteobacteria;c__Betaproteobacteria;o__Burkholderiales;f__Comamonadaceae;g__Limnohabitans
k__Bacteria;p__Proteobacteria;c__Betaproteobacteria;o__Burkholderiales;f__Comamonadaceae;g__Polaromonas
k__Bacteria;p__Proteobacteria;c__Betaproteobacteria;o__Burkholderiales;f__Comamonadaceae;g__Ramlibacter
k__Bacteria;p__Proteobacteria;c__Betaproteobacteria;o__Burkholderiales;f__Comamonadaceae;g__Schlegelella
k__Bacteria;p__Proteobacteria;c__Betaproteobacteria;o__Burkholderiales;f__Oxalobacteraceae;g__
k__Bacteria;p__Proteobacteria;c__Betaproteobacteria;o__Burkholderiales;f__Oxalobacteraceae;g__Herbaspirillum
k__Bacteria;p__Proteobacteria;c__Betaproteobacteria;o__Burkholderiales;f__Oxalobacteraceae;g__Janthinobacterium
k__Bacteria;p__Proteobacteria;c__Betaproteobacteria;o__Burkholderiales;f__Oxalobacteraceae;g__Massilia
k__Bacteria;p__Proteobacteria;c__Betaproteobacteria;o__Hydrogenophilales;f__Hydrogenophilaceae;g__
k__Bacteria;p__Proteobacteria;c__Betaproteobacteria;o__Hydrogenophilales;f__Hydrogenophilaceae;g__Hydrogenophilus
k__Bacteria;p__Proteobacteria;c__Betaproteobacteria;o__Methylophilales;f__Methylophilaceae;g__
k__Bacteria;p__Proteobacteria;c__Betaproteobacteria;o__Methylophilales;f__Methylophilaceae;g__Methylophilus
k__Bacteria;p__Proteobacteria;c__Betaproteobacteria;o__Methylophilales;f__Methylophilaceae;g__Methylotenera
k__Bacteria;p__Proteobacteria;c__Betaproteobacteria;o__Neisseriales;f__Neisseriaceae;g__
k__Bacteria;p__Proteobacteria;c__Betaproteobacteria;o__Neisseriales;f__Neisseriaceae;g__Bergeriella
k__Bacteria;p__Proteobacteria;c__Betaproteobacteria;o__Neisseriales;f__Neisseriaceae;g__Conchiformibius
k__Bacteria;p__Proteobacteria;c__Betaproteobacteria;o__Neisseriales;f__Neisseriaceae;g__Eikenella
k__Bacteria;p__Proteobacteria;c__Betaproteobacteria;o__Neisseriales;f__Neisseriaceae;g__Kingella
k__Bacteria;p__Proteobacteria;c__Betaproteobacteria;o__Neisseriales;f__Neisseriaceae;g__Neisseria
k__Bacteria;p__Proteobacteria;c__Betaproteobacteria;o__Neisseriales;f__Neisseriaceae;g__Stenoxybacter
k__Bacteria;p__Proteobacteria;c__Betaproteobacteria;o__Neisseriales;f__Neisseriaceae;g__Vogesella
k__Bacteria;p__Proteobacteria;c__Betaproteobacteria;o__Rhodocyclales;f__;g__
k__Bacteria;p__Proteobacteria;c__Betaproteobacteria;o__Rhodocyclales;f__Rhodocyclaceae;g__
k__Bacteria;p__Proteobacteria;c__Betaproteobacteria;o__Rhodocyclales;f__Rhodocyclaceae;g__Methyloversatilis
k__Bacteria;p__Proteobacteria;c__Betaproteobacteria;o__Rhodocyclales;f__Rhodocyclaceae;g__Rhodocyclus
k__Bacteria;p__Proteobacteria;c__Betaproteobacteria;o__Rhodocyclales;f__Rhodocyclaceae;g__Zoogloea
k__Bacteria;p__Proteobacteria;c__Deltaproteobacteria;o__CTD005-82B-02;f__;g__
k__Bacteria;p__Proteobacteria;c__Deltaproteobacteria;o__Desulfobacterales;f__Desulfobulbaceae;g__Desulfobulbus
k__Bacteria;p__Proteobacteria;c__Deltaproteobacteria;o__Desulfovibrionales;f__Desulfovibrionaceae;g__Bilophila
k__Bacteria;p__Proteobacteria;c__Deltaproteobacteria;o__MIZ46;f__;g__
k__Bacteria;p__Proteobacteria;c__Deltaproteobacteria;o__Myxococcales;f__;g__
k__Bacteria;p__Proteobacteria;c__Deltaproteobacteria;o__Myxococcales;f__Cystobacteraceae;g__Stigmatella
k__Bacteria;p__Proteobacteria;c__Deltaproteobacteria;o__Myxococcales;f__Polyangiaceae;g__
k__Bacteria;p__Proteobacteria;c__Deltaproteobacteria;o__Syntrophobacterales;f__Syntrophaceae;g__
k__Bacteria;p__Proteobacteria;c__Epsilonproteobacteria;o__Campylobacterales;f__Campylobacteraceae;g__Campylobacter
k__Bacteria;p__Proteobacteria;c__Gammaproteobacteria;o__;f__;g__
k__Bacteria;p__Proteobacteria;c__Gammaproteobacteria;o__Aeromonadales;f__Aeromonadaceae;g__Aeromonas
k__Bacteria;p__Proteobacteria;c__Gammaproteobacteria;o__Alteromonadales;f__Chromatiaceae;g__Rheinheimera
k__Bacteria;p__Proteobacteria;c__Gammaproteobacteria;o__Alteromonadales;f__Moritellaceae;g__Moritella
k__Bacteria;p__Proteobacteria;c__Gammaproteobacteria;o__Alteromonadales;f__Psychromonadaceae;g__Psychromonas
k__Bacteria;p__Proteobacteria;c__Gammaproteobacteria;o__Cardiobacteriales;f__Cardiobacteriaceae;g__Cardiobacterium
k__Bacteria;p__Proteobacteria;c__Gammaproteobacteria;o__Chromatiales;f__;g__
k__Bacteria;p__Proteobacteria;c__Gammaproteobacteria;o__Enterobacteriales;f__Enterobacteriaceae;g__
k__Bacteria;p__Proteobacteria;c__Gammaproteobacteria;o__Enterobacteriales;f__Enterobacteriaceae;g__Citrobacter
k__Bacteria;p__Proteobacteria;c__Gammaproteobacteria;o__Enterobacteriales;f__Enterobacteriaceae;g__Enterobacter
k__Bacteria;p__Proteobacteria;c__Gammaproteobacteria;o__Enterobacteriales;f__Enterobacteriaceae;g__Erwinia
k__Bacteria;p__Proteobacteria;c__Gammaproteobacteria;o__Enterobacteriales;f__Enterobacteriaceae;g__Escherichia
k__Bacteria;p__Proteobacteria;c__Gammaproteobacteria;o__Enterobacteriales;f__Enterobacteriaceae;g__Klebsiella
k__Bacteria;p__Proteobacteria;c__Gammaproteobacteria;o__Enterobacteriales;f__Enterobacteriaceae;g__Leclercia
k__Bacteria;p__Proteobacteria;c__Gammaproteobacteria;o__Enterobacteriales;f__Enterobacteriaceae;g__Pantoea
k__Bacteria;p__Proteobacteria;c__Gammaproteobacteria;o__Enterobacteriales;f__Enterobacteriaceae;g__Raoultella
k__Bacteria;p__Proteobacteria;c__Gammaproteobacteria;o__Enterobacteriales;f__Enterobacteriaceae;g__Shigella
k__Bacteria;p__Proteobacteria;c__Gammaproteobacteria;o__Enterobacteriales;f__Enterobacteriaceae;g__Sodalis
k__Bacteria;p__Proteobacteria;c__Gammaproteobacteria;o__Enterobacteriales;f__Enterobacteriaceae;g__Tatumella
k__Bacteria;p__Proteobacteria;c__Gammaproteobacteria;o__Enterobacteriales;f__Enterobacteriaceae;g__Trabulsiella
k__Bacteria;p__Proteobacteria;c__Gammaproteobacteria;o__Oceanospirillales;f__;g__
k__Bacteria;p__Proteobacteria;c__Gammaproteobacteria;o__Oceanospirillales;f__;g__Congregibacter
k__Bacteria;p__Proteobacteria;c__Gammaproteobacteria;o__Oceanospirillales;f__Alteromonadaceae;g__Microbulbifer
k__Bacteria;p__Proteobacteria;c__Gammaproteobacteria;o__Oceanospirillales;f__Halomonadaceae;g__Chromohalobacter
k__Bacteria;p__Proteobacteria;c__Gammaproteobacteria;o__Oceanospirillales;f__Halomonadaceae;g__Halomonas
k__Bacteria;p__Proteobacteria;c__Gammaproteobacteria;o__Oceanospirillales;f__Pseudomonadaceae;g__
k__Bacteria;p__Proteobacteria;c__Gammaproteobacteria;o__Oceanospirillales;f__Pseudomonadaceae;g__Pseudomonas
k__Bacteria;p__Proteobacteria;c__Gammaproteobacteria;o__Pasteurellales;f__Pasteurellaceae;g__
k__Bacteria;p__Proteobacteria;c__Gammaproteobacteria;o__Pasteurellales;f__Pasteurellaceae;g__Actinobacillus
k__Bacteria;p__Proteobacteria;c__Gammaproteobacteria;o__Pasteurellales;f__Pasteurellaceae;g__Aggregatibacter
k__Bacteria;p__Proteobacteria;c__Gammaproteobacteria;o__Pasteurellales;f__Pasteurellaceae;g__Avibacterium
k__Bacteria;p__Proteobacteria;c__Gammaproteobacteria;o__Pasteurellales;f__Pasteurellaceae;g__Basfia
k__Bacteria;p__Proteobacteria;c__Gammaproteobacteria;o__Pasteurellales;f__Pasteurellaceae;g__Bibersteinia
k__Bacteria;p__Proteobacteria;c__Gammaproteobacteria;o__Pasteurellales;f__Pasteurellaceae;g__Gallibacterium
k__Bacteria;p__Proteobacteria;c__Gammaproteobacteria;o__Pasteurellales;f__Pasteurellaceae;g__Haemophilus
k__Bacteria;p__Proteobacteria;c__Gammaproteobacteria;o__Pasteurellales;f__Pasteurellaceae;g__Histophilus
k__Bacteria;p__Proteobacteria;c__Gammaproteobacteria;o__Pasteurellales;f__Pasteurellaceae;g__Mannheimia
k__Bacteria;p__Proteobacteria;c__Gammaproteobacteria;o__Pasteurellales;f__Pasteurellaceae;g__Pasteurella
k__Bacteria;p__Proteobacteria;c__Gammaproteobacteria;o__Pasteurellales;f__Pasteurellaceae;g__Volucribacter
k__Bacteria;p__Proteobacteria;c__Gammaproteobacteria;o__Pseudomonadales;f__Moraxellaceae;g__
k__Bacteria;p__Proteobacteria;c__Gammaproteobacteria;o__Pseudomonadales;f__Moraxellaceae;g__Acinetobacter
k__Bacteria;p__Proteobacteria;c__Gammaproteobacteria;o__Pseudomonadales;f__Moraxellaceae;g__Enhydrobacter
k__Bacteria;p__Proteobacteria;c__Gammaproteobacteria;o__Pseudomonadales;f__Moraxellaceae;g__Moraxella
k__Bacteria;p__Proteobacteria;c__Gammaproteobacteria;o__Pseudomonadales;f__Moraxellaceae;g__Psychrobacter
k__Bacteria;p__Proteobacteria;c__Gammaproteobacteria;o__Vibrionales;f__Vibrionaceae;g__Aliivibrio
k__Bacteria;p__Proteobacteria;c__Gammaproteobacteria;o__Vibrionales;f__Vibrionaceae;g__Photobacterium
k__Bacteria;p__Proteobacteria;c__Gammaproteobacteria;o__Vibrionales;f__Vibrionaceae;g__Salinivibrio
k__Bacteria;p__Proteobacteria;c__Gammaproteobacteria;o__Vibrionales;f__Vibrionaceae;g__Vibrio
k__Bacteria;p__Proteobacteria;c__Gammaproteobacteria;o__Xanthomonadales;f__Xanthomonadaceae;g__
k__Bacteria;p__Proteobacteria;c__Gammaproteobacteria;o__Xanthomonadales;f__Xanthomonadaceae;g__Dokdonella
k__Bacteria;p__Proteobacteria;c__Gammaproteobacteria;o__Xanthomonadales;f__Xanthomonadaceae;g__Lysobacter
k__Bacteria;p__Proteobacteria;c__Gammaproteobacteria;o__Xanthomonadales;f__Xanthomonadaceae;g__Pseudoxanthomonas
k__Bacteria;p__Proteobacteria;c__Gammaproteobacteria;o__Xanthomonadales;f__Xanthomonadaceae;g__Stenotrophomonas
k__Bacteria;p__Proteobacteria;c__Gammaproteobacteria;o__Xanthomonadales;f__Xanthomonadaceae;g__Xanthomonas
k__Bacteria;p__SPAM;c__;o__;f__;g__
k__Bacteria;p__SR1;c__;o__;f__;g__
k__Bacteria;p__Spirochaetes;c__Spirochaetes (class);o__Spirochaetales;f__Spirochaetaceae;g__Treponema
k__Bacteria;p__Synergistetes;c__Synergistia;o__Synergistales;f__Dethiosulfovibrionaceae;g__TG5
k__Bacteria;p__TM7;c__TM7-3;o__CW040;f__;g__
k__Bacteria;p__TM7;c__TM7-3;o__EW055;f__;g__
k__Bacteria;p__Tenericutes;c__Erysipelotrichi;o__Erysipelotrichales;f__Erysipelotrichaceae;g__
k__Bacteria;p__Tenericutes;c__Erysipelotrichi;o__Erysipelotrichales;f__Erysipelotrichaceae;g__Bulleidia
k__Bacteria;p__Tenericutes;c__Erysipelotrichi;o__Erysipelotrichales;f__Erysipelotrichaceae;g__Catenibacterium
k__Bacteria;p__Tenericutes;c__Erysipelotrichi;o__Erysipelotrichales;f__Erysipelotrichaceae;g__Clostridium
k__Bacteria;p__Tenericutes;c__Erysipelotrichi;o__Erysipelotrichales;f__Erysipelotrichaceae;g__Sharpea
k__Bacteria;p__Tenericutes;c__Mollicutes;o__Mycoplasmatales;f__Mycoplasmataceae;g__Mycoplasma
k__Bacteria;p__Thermi;c__Deinococci;o__Deinococcales;f__Deinococcaceae;g__Deinococcus
k__Bacteria;p__Thermi;c__Deinococci;o__Thermales;f__Thermaceae;g__Thermus
k__Bacteria;p__Verrucomicrobia;c__Spartobacteria;o__;f__;g__Chthoniobacter
k__Bacteria;p__Verrucomicrobia;c__Verrucomicrobiae;o__Verrucomicrobiales;f__Verrucomicrobiaceae;g__Akkermansia
