# Supplementary material for: Moving pictures of the human microbiome
Source: Genome Biol. 2011 May 30;12(5):R50. doi: 10.1186/gb-2011-12-5-r50 (PMC3271711; doi:10.1186/gb-2011-12-5-r50)
Supplement: Additional file 12 — Temporal variation in phylum, class, order, family, and genus abundances (M3 left palm). The x-axis scale differs between M3 and F4 plots. [file sbW9PnWbpKRmPAiGMByLLn4RljykpD_legend.pdf › AdditionalFile12/charts/sbW9PnWbpKRmPAiGMByLLn4RljykpD_legend.pdf]

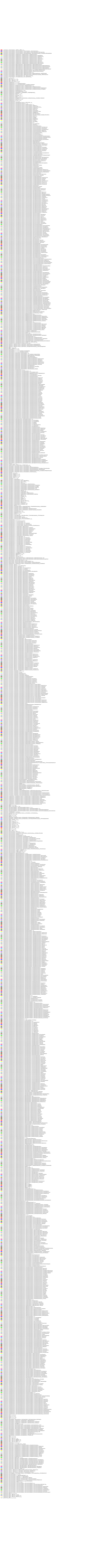

k__Archaea;p__Crenarchaeota;c__Sd-NA;o__NRP-J;f__;g__
k__Archaea;p__Crenarchaeota;c__Thaumarchaeota;o__Cenarchaeales;f__Cenarchaeaceae;g__
k__Archaea;p__Crenarchaeota;c__Thaumarchaeota;o__Cenarchaeales;f__Cenarchaeaceae;g__Nitrosopumilus
k__Archaea;p__Crenarchaeota;c__Thaumarchaeota;o__Nitrososphaerales;f__Nitrososphaeraceae;g__Candidatus Nitrososphaera
k__Archaea;p__Euryarchaeota;c__Halobacteria;o__Halobacteriales;f__Halobacteriaceae;g__
k__Archaea;p__Euryarchaeota;c__Halobacteria;o__Halobacteriales;f__Halobacteriaceae;g__Haladaptatus
k__Archaea;p__Euryarchaeota;c__Halobacteria;o__Halobacteriales;f__Halobacteriaceae;g__Halalkalicoccus
k__Archaea;p__Euryarchaeota;c__Halobacteria;o__Halobacteriales;f__Halobacteriaceae;g__Haloarcula
k__Archaea;p__Euryarchaeota;c__Halobacteria;o__Halobacteriales;f__Halobacteriaceae;g__Halobacterium
k__Archaea;p__Euryarchaeota;c__Halobacteria;o__Halobacteriales;f__Halobacteriaceae;g__Halococcus
k__Archaea;p__Euryarchaeota;c__Halobacteria;o__Halobacteriales;f__Halobacteriaceae;g__Halorubrum
k__Archaea;p__Euryarchaeota;c__Halobacteria;o__Halobacteriales;f__Halobacteriaceae;g__Natronococcus
k__Archaea;p__Euryarchaeota;c__Halobacteria;o__Halobacteriales;f__Halobacteriaceae;g__Natronorubrum
k__Archaea;p__Euryarchaeota;c__Methanobacteria;o__Methanobacteriales;f__Methanobacteriaceae;g__
k__Archaea;p__Euryarchaeota;c__Methanobacteria;o__Methanobacteriales;f__Methanobacteriaceae;g__Methanobacterium
k__Archaea;p__Euryarchaeota;c__Methanobacteria;o__Methanobacteriales;f__Methanobacteriaceae;g__Methanobrevibacter
k__Archaea;p__Euryarchaeota;c__Methanobacteria;o__Methanobacteriales;f__Methanobacteriaceae;g__Methanosphaera
k__Archaea;p__Euryarchaeota;c__Methanobacteria;o__Methanobacteriales;f__Methanobacteriaceae;g__Methanothermobacter
k__Archaea;p__Euryarchaeota;c__Methanomicrobia;o__Methanomicrobiales;f__;g__Candidatus Methanoregula
k__Archaea;p__Euryarchaeota;c__Methanomicrobia;o__Methanomicrobiales;f__Methanocorpusculaceae;g__
k__Archaea;p__Euryarchaeota;c__Methanomicrobia;o__Methanosarcinales;f__;g__
k__Archaea;p__Euryarchaeota;c__Methanomicrobia;o__Methanosarcinales;f__Methanosaetaceae;g__Methanosaeta
k__Archaea;p__Euryarchaeota;c__Methanomicrobia;o__Methanosarcinales;f__Methanosarcinaceae;g__Methanosarcina
k__Archaea;p__Euryarchaeota;c__Thermoplasmata;o__E2;f__Marine group II;g__
k__Archaea;p__Euryarchaeota;c__Thermoplasmata;o__E2;f__WCHD3-02;g__
k__Bacteria;p__;c__;o__;f__;g__
k__Bacteria;p__ABY1_OD1;c__;o__;f__;g__
k__Bacteria;p__AD3;c__ABS-6;o__;f__;g__
k__Bacteria;p__AD3;c__JG37-AG-4;o__;f__;g__
k__Bacteria;p__Acidobacteria;c__;o__;f__;g__
k__Bacteria;p__Acidobacteria;c__;o__;f__Koribacteraceae;g__
k__Bacteria;p__Acidobacteria;c__;o__;f__Koribacteraceae;g__Candidatus Koribacter
k__Bacteria;p__Acidobacteria;c__Acidobacteria (class);o__Acidobacteriales;f__;g__
k__Bacteria;p__Acidobacteria;c__Acidobacteria (class);o__Acidobacteriales;f__Acidobacteriaceae;g__
k__Bacteria;p__Acidobacteria;c__Acidobacteria (class);o__Acidobacteriales;f__Acidobacteriaceae;g__Edaphobacter
k__Bacteria;p__Acidobacteria;c__Acidobacteria (class);o__Acidobacteriales;f__Acidobacteriaceae;g__Terriglobus
k__Bacteria;p__Acidobacteria;c__Acidobacteria-5;o__;f__;g__
k__Bacteria;p__Acidobacteria;c__Chloracidobacteria;o__;f__;g__
k__Bacteria;p__Acidobacteria;c__Holophagae;o__Holophagales;f__Holophagaceae;g__
k__Bacteria;p__Acidobacteria;c__MVS-40;o__;f__;g__
k__Bacteria;p__Acidobacteria;c__OS-K;o__;f__;g__
k__Bacteria;p__Acidobacteria;c__PAUC37f;o__;f__;g__
k__Bacteria;p__Acidobacteria;c__RB25;o__;f__;g__
k__Bacteria;p__Acidobacteria;c__Solibacteres;o__Solibacterales;f__Solibacteraceae;g__Candidatus Solibacter
k__Bacteria;p__Acidobacteria;c__Sva0725;o__;f__;g__
k__Bacteria;p__Acidobacteria;c__iii1-8;o__;f__;g__
k__Bacteria;p__Actinobacteria;c__;o__;f__;g__
k__Bacteria;p__Actinobacteria;c__Actinobacteria (class);o__0319-7L14;f__;g__
k__Bacteria;p__Actinobacteria;c__Actinobacteria (class);o__;f__;g__
k__Bacteria;p__Actinobacteria;c__Actinobacteria (class);o__Acidimicrobiales;f__;g__
k__Bacteria;p__Actinobacteria;c__Actinobacteria (class);o__Acidimicrobiales;f__AKIW874;g__
k__Bacteria;p__Actinobacteria;c__Actinobacteria (class);o__Acidimicrobiales;f__Acidimicrobiaceae;g__
k__Bacteria;p__Actinobacteria;c__Actinobacteria (class);o__Acidimicrobiales;f__CL500-29;g__
k__Bacteria;p__Actinobacteria;c__Actinobacteria (class);o__Acidimicrobiales;f__EB1017;g__
k__Bacteria;p__Actinobacteria;c__Actinobacteria (class);o__Acidimicrobiales;f__Iamiaceae;g__
k__Bacteria;p__Actinobacteria;c__Actinobacteria (class);o__Acidimicrobiales;f__Iamiaceae;g__Iamia
k__Bacteria;p__Actinobacteria;c__Actinobacteria (class);o__Acidimicrobiales;f__JdFBGBact;g__
k__Bacteria;p__Actinobacteria;c__Actinobacteria (class);o__Acidimicrobiales;f__Microthrixaceae;g__
k__Bacteria;p__Actinobacteria;c__Actinobacteria (class);o__Acidimicrobiales;f__Microthrixaceae;g__Candidatus Microthrix
k__Bacteria;p__Actinobacteria;c__Actinobacteria (class);o__Acidimicrobiales;f__SC3-41;g__
k__Bacteria;p__Actinobacteria;c__Actinobacteria (class);o__Acidimicrobiales;f__TK06;g__
k__Bacteria;p__Actinobacteria;c__Actinobacteria (class);o__Acidimicrobiales;f__ZA3409c;g__
k__Bacteria;p__Actinobacteria;c__Actinobacteria (class);o__Acidimicrobiales;f__ZA3612c;g__
k__Bacteria;p__Actinobacteria;c__Actinobacteria (class);o__Acidimicrobiales;f__ntu14;g__
k__Bacteria;p__Actinobacteria;c__Actinobacteria (class);o__Acidimicrobiales;f__wb1_P06;g__
k__Bacteria;p__Actinobacteria;c__Actinobacteria (class);o__Actinomycetales;f__;g__
k__Bacteria;p__Actinobacteria;c__Actinobacteria (class);o__Actinomycetales;f__;g__Tropheryma
k__Bacteria;p__Actinobacteria;c__Actinobacteria (class);o__Actinomycetales;f__ACK-M1;g__
k__Bacteria;p__Actinobacteria;c__Actinobacteria (class);o__Actinomycetales;f__Actinomycetaceae;g__
k__Bacteria;p__Actinobacteria;c__Actinobacteria (class);o__Actinomycetales;f__Actinomycetaceae;g__Actinobaculum
k__Bacteria;p__Actinobacteria;c__Actinobacteria (class);o__Actinomycetales;f__Actinomycetaceae;g__Actinomyces
k__Bacteria;p__Actinobacteria;c__Actinobacteria (class);o__Actinomycetales;f__Actinomycetaceae;g__Arcanobacterium
k__Bacteria;p__Actinobacteria;c__Actinobacteria (class);o__Actinomycetales;f__Actinomycetaceae;g__Mobiluncus
k__Bacteria;p__Actinobacteria;c__Actinobacteria (class);o__Actinomycetales;f__Actinomycetaceae;g__Varibaculum
k__Bacteria;p__Actinobacteria;c__Actinobacteria (class);o__Actinomycetales;f__Actinopolysporaceae;g__
k__Bacteria;p__Actinobacteria;c__Actinobacteria (class);o__Actinomycetales;f__Actinospicaceae;g__
k__Bacteria;p__Actinobacteria;c__Actinobacteria (class);o__Actinomycetales;f__Actinosynnemataceae;g__
k__Bacteria;p__Actinobacteria;c__Actinobacteria (class);o__Actinomycetales;f__Actinosynnemataceae;g__Actinokineospora
k__Bacteria;p__Actinobacteria;c__Actinobacteria (class);o__Actinomycetales;f__Actinosynnemataceae;g__Kibdelosporangium
k__Bacteria;p__Actinobacteria;c__Actinobacteria (class);o__Actinomycetales;f__Actinosynnemataceae;g__Kutzneria
k__Bacteria;p__Actinobacteria;c__Actinobacteria (class);o__Actinomycetales;f__Actinosynnemataceae;g__Saccharothrix
k__Bacteria;p__Actinobacteria;c__Actinobacteria (class);o__Actinomycetales;f__Beutenbergiaceae;g__
k__Bacteria;p__Actinobacteria;c__Actinobacteria (class);o__Actinomycetales;f__Bogoriellaceae;g__
k__Bacteria;p__Actinobacteria;c__Actinobacteria (class);o__Actinomycetales;f__Bogoriellaceae;g__Georgenia
k__Bacteria;p__Actinobacteria;c__Actinobacteria (class);o__Actinomycetales;f__Brevibacteriaceae;g__Brevibacterium
k__Bacteria;p__Actinobacteria;c__Actinobacteria (class);o__Actinomycetales;f__Catenulisporaceae;g__Catenulispora
k__Bacteria;p__Actinobacteria;c__Actinobacteria (class);o__Actinomycetales;f__Cellulomonadaceae;g__
k__Bacteria;p__Actinobacteria;c__Actinobacteria (class);o__Actinomycetales;f__Cellulomonadaceae;g__Actinotalea
k__Bacteria;p__Actinobacteria;c__Actinobacteria (class);o__Actinomycetales;f__Cellulomonadaceae;g__Cellulomonas
k__Bacteria;p__Actinobacteria;c__Actinobacteria (class);o__Actinomycetales;f__Cellulomonadaceae;g__Demequina
