# Supplementary material for: Moving pictures of the human microbiome
Source: Genome Biol. 2011 May 30;12(5):R50. doi: 10.1186/gb-2011-12-5-r50 (PMC3271711; doi:10.1186/gb-2011-12-5-r50)
Supplement: Additional file 12 — Temporal variation in phylum, class, order, family, and genus abundances (M3 left palm). The x-axis scale differs between M3 and F4 plots. [file USXDINjkWUm6UfcbeQiq1G6qELLYjf_legend.pdf › AdditionalFile12/charts/USXDINjkWUm6UfcbeQiq1G6qELLYjf_legend.pdf]

k__Archaea;p__Crenarchaeota;c__Sd-NA;o__NRP-J;f__
k__Archaea;p__Crenarchaeota;c__Thaumarchaeota;o__Cenarchaeales;f__Cenarchaeaceae
k__Archaea;p__Crenarchaeota;c__Thaumarchaeota;o__Nitrososphaerales;f__Nitrososphaeraceae
k__Archaea;p__Euryarchaeota;c__Halobacteria;o__Halobacteriales;f__Halobacteriaceae
k__Archaea;p__Euryarchaeota;c__Methanobacteria;o__Methanobacteriales;f__Methanobacteriaceae
k__Archaea;p__Euryarchaeota;c__Methanomicrobia;o__Methanomicrobiales;f__
k__Archaea;p__Euryarchaeota;c__Methanomicrobia;o__Methanomicrobiales;f__Methanocorpusculaceae
k__Archaea;p__Euryarchaeota;c__Methanomicrobia;o__Methanosarcinales;f__
k__Archaea;p__Euryarchaeota;c__Methanomicrobia;o__Methanosarcinales;f__Methanosaetaceae
k__Archaea;p__Euryarchaeota;c__Methanomicrobia;o__Methanosarcinales;f__Methanosarcinaceae
k__Archaea;p__Euryarchaeota;c__Thermoplasmata;o__E2;f__Marine group II
k__Archaea;p__Euryarchaeota;c__Thermoplasmata;o__E2;f__WCHD3-02
k__Bacteria;p__;c__;o__;f__
k__Bacteria;p__ABY1_OD1;c__;o__;f__
k__Bacteria;p__AD3;c__ABS-6;o__;f__
k__Bacteria;p__AD3;c__JG37-AG-4;o__;f__
k__Bacteria;p__Acidobacteria;c__;o__;f__
k__Bacteria;p__Acidobacteria;c__;o__;f__Koribacteraceae
k__Bacteria;p__Acidobacteria;c__Acidobacteria (class);o__Acidobacteriales;f__
k__Bacteria;p__Acidobacteria;c__Acidobacteria (class);o__Acidobacteriales;f__Acidobacteriaceae
k__Bacteria;p__Acidobacteria;c__Acidobacteria-5;o__;f__
k__Bacteria;p__Acidobacteria;c__Chloracidobacteria;o__;f__
k__Bacteria;p__Acidobacteria;c__Holophagae;o__Holophagales;f__Holophagaceae
k__Bacteria;p__Acidobacteria;c__MVS-40;o__;f__
k__Bacteria;p__Acidobacteria;c__OS-K;o__;f__
k__Bacteria;p__Acidobacteria;c__PAUC37f;o__;f__
k__Bacteria;p__Acidobacteria;c__RB25;o__;f__
k__Bacteria;p__Acidobacteria;c__Solibacteres;o__Solibacterales;f__Solibacteraceae
k__Bacteria;p__Acidobacteria;c__Sva0725;o__;f__
k__Bacteria;p__Acidobacteria;c__iii1-8;o__;f__
k__Bacteria;p__Actinobacteria;c__;o__;f__
k__Bacteria;p__Actinobacteria;c__Actinobacteria (class);o__0319-7L14;f__
k__Bacteria;p__Actinobacteria;c__Actinobacteria (class);o__;f__
k__Bacteria;p__Actinobacteria;c__Actinobacteria (class);o__Acidimicrobiales;f__
k__Bacteria;p__Actinobacteria;c__Actinobacteria (class);o__Acidimicrobiales;f__AKIW874
k__Bacteria;p__Actinobacteria;c__Actinobacteria (class);o__Acidimicrobiales;f__Acidimicrobiaceae
k__Bacteria;p__Actinobacteria;c__Actinobacteria (class);o__Acidimicrobiales;f__CL500-29
k__Bacteria;p__Actinobacteria;c__Actinobacteria (class);o__Acidimicrobiales;f__EB1017
k__Bacteria;p__Actinobacteria;c__Actinobacteria (class);o__Acidimicrobiales;f__Iamiaceae
k__Bacteria;p__Actinobacteria;c__Actinobacteria (class);o__Acidimicrobiales;f__JdFBGBact
k__Bacteria;p__Actinobacteria;c__Actinobacteria (class);o__Acidimicrobiales;f__Microthrixaceae
k__Bacteria;p__Actinobacteria;c__Actinobacteria (class);o__Acidimicrobiales;f__SC3-41
k__Bacteria;p__Actinobacteria;c__Actinobacteria (class);o__Acidimicrobiales;f__TK06
k__Bacteria;p__Actinobacteria;c__Actinobacteria (class);o__Acidimicrobiales;f__ZA3409c
k__Bacteria;p__Actinobacteria;c__Actinobacteria (class);o__Acidimicrobiales;f__ZA3612c
k__Bacteria;p__Actinobacteria;c__Actinobacteria (class);o__Acidimicrobiales;f__ntu14
k__Bacteria;p__Actinobacteria;c__Actinobacteria (class);o__Acidimicrobiales;f__wb1_P06
k__Bacteria;p__Actinobacteria;c__Actinobacteria (class);o__Actinomycetales;f__
k__Bacteria;p__Actinobacteria;c__Actinobacteria (class);o__Actinomycetales;f__ACK-M1
k__Bacteria;p__Actinobacteria;c__Actinobacteria (class);o__Actinomycetales;f__Actinomycetaceae
k__Bacteria;p__Actinobacteria;c__Actinobacteria (class);o__Actinomycetales;f__Actinopolysporaceae
k__Bacteria;p__Actinobacteria;c__Actinobacteria (class);o__Actinomycetales;f__Actinospicaceae
k__Bacteria;p__Actinobacteria;c__Actinobacteria (class);o__Actinomycetales;f__Actinosynnemataceae
k__Bacteria;p__Actinobacteria;c__Actinobacteria (class);o__Actinomycetales;f__Beutenbergiaceae
k__Bacteria;p__Actinobacteria;c__Actinobacteria (class);o__Actinomycetales;f__Bogoriellaceae
k__Bacteria;p__Actinobacteria;c__Actinobacteria (class);o__Actinomycetales;f__Brevibacteriaceae
k__Bacteria;p__Actinobacteria;c__Actinobacteria (class);o__Actinomycetales;f__Catenulisporaceae
k__Bacteria;p__Actinobacteria;c__Actinobacteria (class);o__Actinomycetales;f__Cellulomonadaceae
k__Bacteria;p__Actinobacteria;c__Actinobacteria (class);o__Actinomycetales;f__Corynebacteriaceae
k__Bacteria;p__Actinobacteria;c__Actinobacteria (class);o__Actinomycetales;f__Cryptosporangiaceae
k__Bacteria;p__Actinobacteria;c__Actinobacteria (class);o__Actinomycetales;f__Dermabacteraceae
k__Bacteria;p__Actinobacteria;c__Actinobacteria (class);o__Actinomycetales;f__Dermacoccaceae
k__Bacteria;p__Actinobacteria;c__Actinobacteria (class);o__Actinomycetales;f__Dermatophilaceae
k__Bacteria;p__Actinobacteria;c__Actinobacteria (class);o__Actinomycetales;f__Dietziaceae
k__Bacteria;p__Actinobacteria;c__Actinobacteria (class);o__Actinomycetales;f__Frankiaceae
k__Bacteria;p__Actinobacteria;c__Actinobacteria (class);o__Actinomycetales;f__Geodermatophilaceae
k__Bacteria;p__Actinobacteria;c__Actinobacteria (class);o__Actinomycetales;f__Glycomycetaceae
k__Bacteria;p__Actinobacteria;c__Actinobacteria (class);o__Actinomycetales;f__Gordoniaceae
k__Bacteria;p__Actinobacteria;c__Actinobacteria (class);o__Actinomycetales;f__Intrasporangiaceae
k__Bacteria;p__Actinobacteria;c__Actinobacteria (class);o__Actinomycetales;f__Jonesiaceae
k__Bacteria;p__Actinobacteria;c__Actinobacteria (class);o__Actinomycetales;f__Kineosporiaceae
k__Bacteria;p__Actinobacteria;c__Actinobacteria (class);o__Actinomycetales;f__Microbacteriaceae
k__Bacteria;p__Actinobacteria;c__Actinobacteria (class);o__Actinomycetales;f__Micrococcaceae
k__Bacteria;p__Actinobacteria;c__Actinobacteria (class);o__Actinomycetales;f__Micromonosporaceae
k__Bacteria;p__Actinobacteria;c__Actinobacteria (class);o__Actinomycetales;f__Mycobacteriaceae
k__Bacteria;p__Actinobacteria;c__Actinobacteria (class);o__Actinomycetales;f__Nakamurellaceae
k__Bacteria;p__Actinobacteria;c__Actinobacteria (class);o__Actinomycetales;f__Nocardiaceae
k__Bacteria;p__Actinobacteria;c__Actinobacteria (class);o__Actinomycetales;f__Nocardioidaceae
k__Bacteria;p__Actinobacteria;c__Actinobacteria (class);o__Actinomycetales;f__Nocardiopsaceae
k__Bacteria;p__Actinobacteria;c__Actinobacteria (class);o__Actinomycetales;f__Promicromonosporaceae
k__Bacteria;p__Actinobacteria;c__Actinobacteria (class);o__Actinomycetales;f__Propionibacteriaceae
k__Bacteria;p__Actinobacteria;c__Actinobacteria (class);o__Actinomycetales;f__Pseudonocardiaceae
k__Bacteria;p__Actinobacteria;c__Actinobacteria (class);o__Actinomycetales;f__Rarobacteraceae
k__Bacteria;p__Actinobacteria;c__Actinobacteria (class);o__Actinomycetales;f__Sanguibacteraceae
k__Bacteria;p__Actinobacteria;c__Actinobacteria (class);o__Actinomycetales;f__Segniliparaceae
k__Bacteria;p__Actinobacteria;c__Actinobacteria (class);o__Actinomycetales;f__Sporichthyaceae
k__Bacteria;p__Actinobacteria;c__Actinobacteria (class);o__Actinomycetales;f__Streptomycetaceae
k__Bacteria;p__Actinobacteria;c__Actinobacteria (class);o__Actinomycetales;f__Streptosporangiaceae
k__Bacteria;p__Actinobacteria;c__Actinobacteria (class);o__Actinomycetales;f__Thermomonosporaceae
k__Bacteria;p__Actinobacteria;c__Actinobacteria (class);o__Actinomycetales;f__Tsukamurellaceae
k__Bacteria;p__Actinobacteria;c__Actinobacteria (class);o__Actinomycetales;f__Williamsiaceae
k__Bacteria;p__Actinobacteria;c__Actinobacteria (class);o__Actinomycetales;f__Yaniellaceae
k__Bacteria;p__Actinobacteria;c__Actinobacteria (class);o__Bifidobacteriales;f__
k__Bacteria;p__Actinobacteria;c__Actinobacteria (class);o__Bifidobacteriales;f__Bifidobacteriaceae
k__Bacteria;p__Actinobacteria;c__Actinobacteria (class);o__Coriobacteriales;f__
k__Bacteria;p__Actinobacteria;c__Actinobacteria (class);o__Coriobacteriales;f__Coriobacteriaceae
k__Bacteria;p__Actinobacteria;c__Actinobacteria (class);o__Euzebiales;f__Euzebiaceae
k__Bacteria;p__Actinobacteria;c__Actinobacteria (class);o__MC47;f__
k__Bacteria;p__Actinobacteria;c__Actinobacteria (class);o__Rubrobacterales;f__Rubrobacteraceae
k__Bacteria;p__Actinobacteria;c__Actinobacteria (class);o__Solirubrobacterales;f__
k__Bacteria;p__Actinobacteria;c__Actinobacteria (class);o__Solirubrobacterales;f__Patulibacteraceae
k__Bacteria;p__Actinobacteria;c__Actinobacteria (class);o__Solirubrobacterales;f__Solirubrobacteraceae
k__Bacteria;p__Actinobacteria;c__Actinobacteria (class);o__WCHB1-81;f__At425_EubF1
k__Bacteria;p__Actinobacteria;c__Actinobacteria (class);o__koll13;f__
k__Bacteria;p__Aquificae;c__Aquificae (class);o__Aquificales;f__Aquificaceae
k__Bacteria;p__Aquificae;c__Aquificae (class);o__Aquificales;f__Hydrogenothermaceae
k__Bacteria;p__BRC1;c__;o__;f__
k__Bacteria;p__BRC1;c__PRR-11;o__;f__
k__Bacteria;p__Bacteroidetes;c__;o__;f__
k__Bacteria;p__Bacteroidetes;c__Bacteroidia;o__Bacteroidales;f__
k__Bacteria;p__Bacteroidetes;c__Bacteroidia;o__Bacteroidales;f__Bacteroidaceae
k__Bacteria;p__Bacteroidetes;c__Bacteroidia;o__Bacteroidales;f__Marinilabiaceae
k__Bacteria;p__Bacteroidetes;c__Bacteroidia;o__Bacteroidales;f__Porphyromonadaceae
k__Bacteria;p__Bacteroidetes;c__Bacteroidia;o__Bacteroidales;f__Prevotellaceae
k__Bacteria;p__Bacteroidetes;c__Bacteroidia;o__Bacteroidales;f__Rikenellaceae
k__Bacteria;p__Bacteroidetes;c__Flavobacteria;o__;f__
k__Bacteria;p__Bacteroidetes;c__Flavobacteria;o__Flavobacteriales;f__
k__Bacteria;p__Bacteroidetes;c__Flavobacteria;o__Flavobacteriales;f__Blattabacteriaceae
k__Bacteria;p__Bacteroidetes;c__Flavobacteria;o__Flavobacteriales;f__Cryomorphaceae
k__Bacteria;p__Bacteroidetes;c__Flavobacteria;o__Flavobacteriales;f__Flavobacteriaceae
k__Bacteria;p__Bacteroidetes;c__Sphingobacteria;o__Sphingobacteriales;f__
k__Bacteria;p__Bacteroidetes;c__Sphingobacteria;o__Sphingobacteriales;f__Cyclobacteriaceae
k__Bacteria;p__Bacteroidetes;c__Sphingobacteria;o__Sphingobacteriales;f__Flammeovirgaceae
k__Bacteria;p__Bacteroidetes;c__Sphingobacteria;o__Sphingobacteriales;f__Flexibacteraceae
k__Bacteria;p__Bacteroidetes;c__Sphingobacteria;o__Sphingobacteriales;f__Rhodothermaceae
k__Bacteria;p__Bacteroidetes;c__Sphingobacteria;o__Sphingobacteriales;f__Saprospiraceae
k__Bacteria;p__Bacteroidetes;c__Sphingobacteria;o__Sphingobacteriales;f__Sphingobacteriaceae
k__Bacteria;p__CCM11b;c__;o__;f__
k__Bacteria;p__Caldithrix_KSB1;c__Caldithrixae;o__Caldithrixales;f__
k__Bacteria;p__Chlamydiae;c__Chlamydiae (class);o__Chlamydiales;f__
k__Bacteria;p__Chlamydiae;c__Chlamydiae (class);o__Chlamydiales;f__Parachlamydiaceae
k__Bacteria;p__Chlamydiae;c__Chlamydiae (class);o__Chlamydiales;f__Rhabdochlamydiaceae
k__Bacteria;p__Chlamydiae;c__Chlamydiae (class);o__Chlamydiales;f__Simkaniaceae
k__Bacteria;p__Chlorobi;c__;o__;f__
k__Bacteria;p__Chlorobi;c__BSV19;o__;f__
k__Bacteria;p__Chlorobi;c__OPB56;o__;f__
k__Bacteria;p__Chlorobi;c__SJA-28;o__;f__
k__Bacteria;p__Chlorobi;c__ZB1;o__;f__
k__Bacteria;p__Chloroflexi;c__;o__;f__
k__Bacteria;p__Chloroflexi;c__Anaerolineae;o__;f__
k__Bacteria;p__Chloroflexi;c__Anaerolineae;o__A31;f__KNA6-EB22
k__Bacteria;p__Chloroflexi;c__Anaerolineae;o__A31;f__S47
k__Bacteria;p__Chloroflexi;c__Anaerolineae;o__A4b;f__
k__Bacteria;p__Chloroflexi;c__Anaerolineae;o__Anaerolineales;f__Anaerolinaceae
k__Bacteria;p__Chloroflexi;c__Anaerolineae;o__CFB-26;f__
k__Bacteria;p__Chloroflexi;c__Anaerolineae;o__Caldilineales;f__
k__Bacteria;p__Chloroflexi;c__Anaerolineae;o__Caldilineales;f__Caldilineaceae
k__Bacteria;p__Chloroflexi;c__Anaerolineae;o__DRC31;f__
k__Bacteria;p__Chloroflexi;c__Anaerolineae;o__GCA004;f__
k__Bacteria;p__Chloroflexi;c__Anaerolineae;o__H39;f__
k__Bacteria;p__Chloroflexi;c__Anaerolineae;o__S0208;f__
k__Bacteria;p__Chloroflexi;c__Anaerolineae;o__SB-34;f__
k__Bacteria;p__Chloroflexi;c__Anaerolineae;o__SJA-101;f__SHA-31
k__Bacteria;p__Chloroflexi;c__Anaerolineae;o__SJA-15;f__
k__Bacteria;p__Chloroflexi;c__Anaerolineae;o__WCHB1-50;f__
k__Bacteria;p__Chloroflexi;c__Anaerolineae;o__envOPS12;f__
k__Bacteria;p__Chloroflexi;c__Bljii12;o__;f__
k__Bacteria;p__Chloroflexi;c__Chloroflexi (class);o__Herpetosiphonales;f__Herpetosiphonaceae
k__Bacteria;p__Chloroflexi;c__Chloroflexi (class);o__Roseiflexales;f__
k__Bacteria;p__Chloroflexi;c__Chloroflexi (class);o__Roseiflexales;f__Kouleothrixaceae
k__Bacteria;p__Chloroflexi;c__Chloroflexi-4;o__;f__
k__Bacteria;p__Chloroflexi;c__Ktedonobacteria;o__;f__
k__Bacteria;p__Chloroflexi;c__SOGA31;o__;f__
k__Bacteria;p__Chloroflexi;c__TK17;o__;f__
k__Bacteria;p__Chloroflexi;c__Thermobacula;o__Thermobaculales;f__Thermobaculaceae
k__Bacteria;p__Chloroflexi;c__Thermomicrobia;o__;f__
k__Bacteria;p__Chloroflexi;c__Thermomicrobia;o__HN1-15;f__
k__Bacteria;p__Chloroflexi;c__Thermomicrobia;o__Thermomicrobiales;f__
k__Bacteria;p__Cyanobacteria;c__;o__;f__
k__Bacteria;p__Cyanobacteria;c__;o__Chroococcales;f__
k__Bacteria;p__Cyanobacteria;c__;o__Chroococcales;f__Prochlorococcaceae
k__Bacteria;p__Cyanobacteria;c__;o__Nostocales;f__Nostocaceae
k__Bacteria;p__Cyanobacteria;c__;o__Nostocales;f__Rivulariaceae
k__Bacteria;p__Cyanobacteria;c__;o__Oscillatoriales;f__
k__Bacteria;p__Cyanobacteria;c__;o__Pleurocapsales;f__
k__Bacteria;p__Cyanobacteria;c__;o__Stigonematales;f__
k__Bacteria;p__Cyanobacteria;c__S15B-MN24;o__;f__
k__Bacteria;p__Cyanobacteria;c__SM1D11;o__;f__
k__Bacteria;p__Cyanobacteria;c__YS2;o__;f__
k__Bacteria;p__Cyanobacteria;c__mle1-12;o__;f__
k__Bacteria;p__Deferribacteres;c__Deferribacteres (class);o__Deferribacterales;f__Deferribacteraceae
k__Bacteria;p__Elusimicrobia;c__Elusimicrobia (class);o__;f__
k__Bacteria;p__Elusimicrobia;c__Elusimicrobia (class);o__Elusimicrobiales;f__
k__Bacteria;p__Elusimicrobia;c__Elusimicrobia (class);o__Elusimicrobiales;f__Elusimicrobiaceae
k__Bacteria;p__Fibrobacteres;c__Fibrobacteres (class);o__Fibrobacterales;f__Fibrobacteraceae
k__Bacteria;p__Firmicutes;c__Bacilli;o__;f__
k__Bacteria;p__Firmicutes;c__Bacilli;o__Bacillales;f__
k__Bacteria;p__Firmicutes;c__Bacilli;o__Bacillales;f__Alicyclobacillaceae
k__Bacteria;p__Firmicutes;c__Bacilli;o__Bacillales;f__Bacillaceae
k__Bacteria;p__Firmicutes;c__Bacilli;o__Bacillales;f__Listeriaceae
k__Bacteria;p__Firmicutes;c__Bacilli;o__Bacillales;f__Paenibacillaceae
k__Bacteria;p__Firmicutes;c__Bacilli;o__Bacillales;f__Pasteuriaceae
k__Bacteria;p__Firmicutes;c__Bacilli;o__Bacillales;f__Planococcaceae
k__Bacteria;p__Firmicutes;c__Bacilli;o__Bacillales;f__Sporolactobacillaceae
k__Bacteria;p__Firmicutes;c__Bacilli;o__Bacillales;f__Staphylococcaceae
k__Bacteria;p__Firmicutes;c__Bacilli;o__Bacillales;f__Thermoactinomycetaceae
k__Bacteria;p__Firmicutes;c__Bacilli;o__Erysipelotrichales;f__Erysipelotrichaceae
k__Bacteria;p__Firmicutes;c__Bacilli;o__Lactobacillales;f__
k__Bacteria;p__Firmicutes;c__Bacilli;o__Lactobacillales;f__Aerococcaceae
k__Bacteria;p__Firmicutes;c__Bacilli;o__Lactobacillales;f__Carnobacteriaceae
k__Bacteria;p__Firmicutes;c__Bacilli;o__Lactobacillales;f__Enterococcaceae
k__Bacteria;p__Firmicutes;c__Bacilli;o__Lactobacillales;f__Lactobacillaceae
k__Bacteria;p__Firmicutes;c__Bacilli;o__Lactobacillales;f__Leuconostocaceae
k__Bacteria;p__Firmicutes;c__Bacilli;o__Lactobacillales;f__Streptococcaceae
k__Bacteria;p__Firmicutes;c__Clostridia;o__1B07;f__
k__Bacteria;p__Firmicutes;c__Clostridia;o__;f__
k__Bacteria;p__Firmicutes;c__Clostridia;o__BSA2B-08;f__
k__Bacteria;p__Firmicutes;c__Clostridia;o__Clostridiales;f__
k__Bacteria;p__Firmicutes;c__Clostridia;o__Clostridiales;f__BSV43
k__Bacteria;p__Firmicutes;c__Clostridia;o__Clostridiales;f__Catabacteriaceae
k__Bacteria;p__Firmicutes;c__Clostridia;o__Clostridiales;f__Clostridiaceae
k__Bacteria;p__Firmicutes;c__Clostridia;o__Clostridiales;f__Clostridiales Family XI. Incertae Sedis
k__Bacteria;p__Firmicutes;c__Clostridia;o__Clostridiales;f__Clostridiales Family XII. Incertae Sedis
k__Bacteria;p__Firmicutes;c__Clostridia;o__Clostridiales;f__Clostridiales Family XIII. Incertae Sedis
k__Bacteria;p__Firmicutes;c__Clostridia;o__Clostridiales;f__D2
k__Bacteria;p__Firmicutes;c__Clostridia;o__Clostridiales;f__Dehalobacteriaceae
k__Bacteria;p__Firmicutes;c__Clostridia;o__Clostridiales;f__Eubacteriaceae
k__Bacteria;p__Firmicutes;c__Clostridia;o__Clostridiales;f__Lachnospiraceae
k__Bacteria;p__Firmicutes;c__Clostridia;o__Clostridiales;f__Peptococcaceae
k__Bacteria;p__Firmicutes;c__Clostridia;o__Clostridiales;f__Peptostreptococcaceae
k__Bacteria;p__Firmicutes;c__Clostridia;o__Clostridiales;f__Ruminococcaceae
k__Bacteria;p__Firmicutes;c__Clostridia;o__Clostridiales;f__Sulfobacillaceae
k__Bacteria;p__Firmicutes;c__Clostridia;o__Clostridiales;f__Symbiobacteriaceae
k__Bacteria;p__Firmicutes;c__Clostridia;o__Clostridiales;f__Syntrophomonadaceae
k__Bacteria;p__Firmicutes;c__Clostridia;o__Clostridiales;f__Thermoanaerobacterales Family III. Incertae Sedis
k__Bacteria;p__Firmicutes;c__Clostridia;o__Clostridiales;f__Veillonellaceae
k__Bacteria;p__Firmicutes;c__Clostridia;o__Desulfitobacterales;f__Desulfitobacteraceae
k__Bacteria;p__Firmicutes;c__Clostridia;o__Halanaerobiales;f__Halanaerobiaceae
k__Bacteria;p__Firmicutes;c__Clostridia;o__Halanaerobiales;f__Halobacteroidaceae
k__Bacteria;p__Firmicutes;c__Clostridia;o__MBA08;f__
k__Bacteria;p__Firmicutes;c__Clostridia;o__Natranaerobiales;f__Anaerobrancaceae
k__Bacteria;p__Firmicutes;c__Clostridia;o__Natranaerobiales;f__ML1228J-1
k__Bacteria;p__Firmicutes;c__Clostridia;o__Natranaerobiales;f__YAB3B13
k__Bacteria;p__Firmicutes;c__Clostridia;o__OPB54;f__
k__Bacteria;p__Firmicutes;c__Clostridia;o__Thermoanaerobacterales;f__Caldicellulosiruptoraceae
k__Bacteria;p__Firmicutes;c__Clostridia;o__Thermoanaerobacterales;f__Thermoanaerobacteraceae
k__Bacteria;p__Fusobacteria;c__Fusobacteria (class);o__Fusobacteriales;f__Fusobacteriaceae
k__Bacteria;p__GN02;c__;o__;f__
k__Bacteria;p__GN02;c__VC12-cl04;o__;f__
k__Bacteria;p__Gemmatimonadetes;c__Gemmatimonadetes (class);o__;f__
k__Bacteria;p__Gemmatimonadetes;c__Gemmatimonadetes (class);o__Gemmatimonadales;f__
k__Bacteria;p__Gemmatimonadetes;c__Gemmatimonadetes (class);o__Gemmatimonadales;f__Gemmatimonadaceae
k__Bacteria;p__Lentisphaerae;c__;o__;f__
k__Bacteria;p__Lentisphaerae;c__Lentisphaerae (class);o__Victivallales;f__Victivallaceae
k__Bacteria;p__MVP-15;c__;o__;f__
k__Bacteria;p__NC10;c__;o__;f__
k__Bacteria;p__NKB19;c__;o__;f__
k__Bacteria;p__Nitrospirae;c__Nitrospira (class);o__Nitrospirales;f__Nitrospiraceae
k__Bacteria;p__Nitrospirae;c__Nitrospira (class);o__Nitrospirales;f__Thermodesulfovibrionaceae
k__Bacteria;p__OP10;c__5B-18;o__;f__
k__Bacteria;p__OP10;c__;o__;f__
k__Bacteria;p__OP10;c__CH21;o__;f__
k__Bacteria;p__OP10;c__CL500-48;o__;f__
k__Bacteria;p__OP10;c__OS-L;o__;f__
k__Bacteria;p__OP10;c__S1a-1H;o__;f__
k__Bacteria;p__OP10;c__SJA-176;o__;f__
k__Bacteria;p__OP10;c__SJA-22;o__;f__
k__Bacteria;p__OP11;c__;o__;f__
k__Bacteria;p__OP3;c__;o__;f__
k__Bacteria;p__OP8;c__OP8;o__;f__
k__Bacteria;p__Planctomycetes;c__FFCH393;o__;f__
k__Bacteria;p__Planctomycetes;c__Kueneniae;o__Kueneniales;f__Kueneniaceae
k__Bacteria;p__Planctomycetes;c__PW285;o__;f__
k__Bacteria;p__Planctomycetes;c__Phycisphaerae;o__;f__
k__Bacteria;p__Planctomycetes;c__Phycisphaerae;o__Phycisphaerales;f__
k__Bacteria;p__Planctomycetes;c__Planctomycea;o__Gemmatales;f__Gemmataceae
k__Bacteria;p__Planctomycetes;c__Planctomycea;o__Gemmatales;f__Isosphaeraceae
k__Bacteria;p__Planctomycetes;c__Planctomycea;o__Pirellulales;f__
k__Bacteria;p__Planctomycetes;c__Planctomycea;o__Pirellulales;f__Pirellulaceae
k__Bacteria;p__Planctomycetes;c__Planctomycea;o__Planctomycetales;f__Planctomycetaceae
k__Bacteria;p__Planctomycetes;c__agg27;o__CL500-15;f__
k__Bacteria;p__Planctomycetes;c__agg27;o__OM190;f__
k__Bacteria;p__Planctomycetes;c__vadinHA49;o__;f__
k__Bacteria;p__Proteobacteria;c__Alphaproteobacteria;o__;f__
k__Bacteria;p__Proteobacteria;c__Alphaproteobacteria;o__Caulobacterales;f__Caulobacteraceae
k__Bacteria;p__Proteobacteria;c__Alphaproteobacteria;o__Rhizobiales;f__
k__Bacteria;p__Proteobacteria;c__Alphaproteobacteria;o__Rhizobiales;f__Aurantimonadaceae
k__Bacteria;p__Proteobacteria;c__Alphaproteobacteria;o__Rhizobiales;f__Bartonellaceae
k__Bacteria;p__Proteobacteria;c__Alphaproteobacteria;o__Rhizobiales;f__Beijerinckiaceae
k__Bacteria;p__Proteobacteria;c__Alphaproteobacteria;o__Rhizobiales;f__Bradyrhizobiaceae
k__Bacteria;p__Proteobacteria;c__Alphaproteobacteria;o__Rhizobiales;f__Brucellaceae
k__Bacteria;p__Proteobacteria;c__Alphaproteobacteria;o__Rhizobiales;f__Cohaesibacteraceae
k__Bacteria;p__Proteobacteria;c__Alphaproteobacteria;o__Rhizobiales;f__Hyphomicrobiaceae
k__Bacteria;p__Proteobacteria;c__Alphaproteobacteria;o__Rhizobiales;f__Methylobacteriaceae
k__Bacteria;p__Proteobacteria;c__Alphaproteobacteria;o__Rhizobiales;f__Methylocystaceae
k__Bacteria;p__Proteobacteria;c__Alphaproteobacteria;o__Rhizobiales;f__Phyllobacteriaceae
k__Bacteria;p__Proteobacteria;c__Alphaproteobacteria;o__Rhizobiales;f__Rhizobiaceae
k__Bacteria;p__Proteobacteria;c__Alphaproteobacteria;o__Rhizobiales;f__Rhodobacteraceae
k__Bacteria;p__Proteobacteria;c__Alphaproteobacteria;o__Rhizobiales;f__Rhodobiaceae
k__Bacteria;p__Proteobacteria;c__Alphaproteobacteria;o__Rhizobiales;f__Xanthobacteraceae
k__Bacteria;p__Proteobacteria;c__Alphaproteobacteria;o__Rhodobacterales;f__
k__Bacteria;p__Proteobacteria;c__Alphaproteobacteria;o__Rhodobacterales;f__Hyphomonadaceae
k__Bacteria;p__Proteobacteria;c__Alphaproteobacteria;o__Rhodobacterales;f__Rhodobacteraceae
k__Bacteria;p__Proteobacteria;c__Alphaproteobacteria;o__Rhodospirillales;f__Acetobacteraceae
k__Bacteria;p__Proteobacteria;c__Alphaproteobacteria;o__Rhodospirillales;f__Rhodospirillaceae
k__Bacteria;p__Proteobacteria;c__Alphaproteobacteria;o__Rickettsiales;f__
k__Bacteria;p__Proteobacteria;c__Alphaproteobacteria;o__Rickettsiales;f__Rickettsiaceae
k__Bacteria;p__Proteobacteria;c__Alphaproteobacteria;o__Sphingomonadales;f__
k__Bacteria;p__Proteobacteria;c__Alphaproteobacteria;o__Sphingomonadales;f__Erythrobacteraceae
k__Bacteria;p__Proteobacteria;c__Alphaproteobacteria;o__Sphingomonadales;f__Sphingomonadaceae
k__Bacteria;p__Proteobacteria;c__Betaproteobacteria;o__;f__
k__Bacteria;p__Proteobacteria;c__Betaproteobacteria;o__Burkholderiales;f__
k__Bacteria;p__Proteobacteria;c__Betaproteobacteria;o__Burkholderiales;f__Alcaligenaceae
k__Bacteria;p__Proteobacteria;c__Betaproteobacteria;o__Burkholderiales;f__Burkholderiaceae
k__Bacteria;p__Proteobacteria;c__Betaproteobacteria;o__Burkholderiales;f__Comamonadaceae
k__Bacteria;p__Proteobacteria;c__Betaproteobacteria;o__Burkholderiales;f__Oxalobacteraceae
k__Bacteria;p__Proteobacteria;c__Betaproteobacteria;o__Gallionellales;f__Gallionellaceae
k__Bacteria;p__Proteobacteria;c__Betaproteobacteria;o__Hydrogenophilales;f__Hydrogenophilaceae
k__Bacteria;p__Proteobacteria;c__Betaproteobacteria;o__Methylophilales;f__
k__Bacteria;p__Proteobacteria;c__Betaproteobacteria;o__Methylophilales;f__Methylophilaceae
k__Bacteria;p__Proteobacteria;c__Betaproteobacteria;o__Neisseriales;f__Neisseriaceae
k__Bacteria;p__Proteobacteria;c__Betaproteobacteria;o__Nitrosomonadales;f__Nitrosomonadaceae
k__Bacteria;p__Proteobacteria;c__Betaproteobacteria;o__Procabacteriales;f__Procabacteriaceae
k__Bacteria;p__Proteobacteria;c__Betaproteobacteria;o__Rhodocyclales;f__
k__Bacteria;p__Proteobacteria;c__Betaproteobacteria;o__Rhodocyclales;f__Rhodocyclaceae
k__Bacteria;p__Proteobacteria;c__Deltaproteobacteria;o__;f__
k__Bacteria;p__Proteobacteria;c__Deltaproteobacteria;o__;f__Syntrophorhabdaceae
k__Bacteria;p__Proteobacteria;c__Deltaproteobacteria;o__Bdellovibrionales;f__Bacteriovoracaceae
k__Bacteria;p__Proteobacteria;c__Deltaproteobacteria;o__Bdellovibrionales;f__Bdellovibrionaceae
k__Bacteria;p__Proteobacteria;c__Deltaproteobacteria;o__CTD005-82B-02;f__
k__Bacteria;p__Proteobacteria;c__Deltaproteobacteria;o__Desulfobacterales;f__
k__Bacteria;p__Proteobacteria;c__Deltaproteobacteria;o__Desulfobacterales;f__Desulfobulbaceae
k__Bacteria;p__Proteobacteria;c__Deltaproteobacteria;o__Desulfovibrionales;f__Desulfomicrobiaceae
k__Bacteria;p__Proteobacteria;c__Deltaproteobacteria;o__Desulfovibrionales;f__Desulfonatronumaceae
k__Bacteria;p__Proteobacteria;c__Deltaproteobacteria;o__Desulfovibrionales;f__Desulfovibrionaceae
k__Bacteria;p__Proteobacteria;c__Deltaproteobacteria;o__Desulfuromonadales;f__
k__Bacteria;p__Proteobacteria;c__Deltaproteobacteria;o__Desulfuromonadales;f__Desulfuromonadaceae
k__Bacteria;p__Proteobacteria;c__Deltaproteobacteria;o__Desulfuromonadales;f__Geobacteraceae
k__Bacteria;p__Proteobacteria;c__Deltaproteobacteria;o__Desulfuromonadales;f__Pelobacteraceae
k__Bacteria;p__Proteobacteria;c__Deltaproteobacteria;o__Entotheonellales;f__Entotheonellaceae
k__Bacteria;p__Proteobacteria;c__Deltaproteobacteria;o__MIZ46;f__
k__Bacteria;p__Proteobacteria;c__Deltaproteobacteria;o__Myxococcales;f__
k__Bacteria;p__Proteobacteria;c__Deltaproteobacteria;o__Myxococcales;f__Cystobacteraceae
k__Bacteria;p__Proteobacteria;c__Deltaproteobacteria;o__Myxococcales;f__Haliangiaceae
k__Bacteria;p__Proteobacteria;c__Deltaproteobacteria;o__Myxococcales;f__Myxococcaceae
k__Bacteria;p__Proteobacteria;c__Deltaproteobacteria;o__Myxococcales;f__Nannocystaceae
k__Bacteria;p__Proteobacteria;c__Deltaproteobacteria;o__Myxococcales;f__Polyangiaceae
k__Bacteria;p__Proteobacteria;c__Deltaproteobacteria;o__NB1-j;f__
k__Bacteria;p__Proteobacteria;c__Deltaproteobacteria;o__NB1-j;f__JTB38
k__Bacteria;p__Proteobacteria;c__Deltaproteobacteria;o__NB1-j;f__MND4
k__Bacteria;p__Proteobacteria;c__Deltaproteobacteria;o__NB1-j;f__NB1-i
k__Bacteria;p__Proteobacteria;c__Deltaproteobacteria;o__NKB15;f__
k__Bacteria;p__Proteobacteria;c__Deltaproteobacteria;o__Syntrophobacterales;f__
k__Bacteria;p__Proteobacteria;c__Deltaproteobacteria;o__Syntrophobacterales;f__Desulfobacteraceae
k__Bacteria;p__Proteobacteria;c__Deltaproteobacteria;o__Syntrophobacterales;f__Syntrophaceae
k__Bacteria;p__Proteobacteria;c__Deltaproteobacteria;o__Syntrophobacterales;f__Syntrophobacteraceae
k__Bacteria;p__Proteobacteria;c__Epsilonproteobacteria;o__;f__
k__Bacteria;p__Proteobacteria;c__Epsilonproteobacteria;o__Campylobacterales;f__
k__Bacteria;p__Proteobacteria;c__Epsilonproteobacteria;o__Campylobacterales;f__Campylobacteraceae
k__Bacteria;p__Proteobacteria;c__Epsilonproteobacteria;o__Campylobacterales;f__Helicobacteraceae
k__Bacteria;p__Proteobacteria;c__Gammaproteobacteria;o__;f__
k__Bacteria;p__Proteobacteria;c__Gammaproteobacteria;o__Aeromonadales;f__
k__Bacteria;p__Proteobacteria;c__Gammaproteobacteria;o__Aeromonadales;f__Aeromonadaceae
k__Bacteria;p__Proteobacteria;c__Gammaproteobacteria;o__Aeromonadales;f__Succinivibrionaceae
k__Bacteria;p__Proteobacteria;c__Gammaproteobacteria;o__Alteromonadales;f__
k__Bacteria;p__Proteobacteria;c__Gammaproteobacteria;o__Alteromonadales;f__Alteromonadaceae
k__Bacteria;p__Proteobacteria;c__Gammaproteobacteria;o__Alteromonadales;f__Chromatiaceae
k__Bacteria;p__Proteobacteria;c__Gammaproteobacteria;o__Alteromonadales;f__Colwelliaceae
k__Bacteria;p__Proteobacteria;c__Gammaproteobacteria;o__Alteromonadales;f__Ferrimonadaceae
k__Bacteria;p__Proteobacteria;c__Gammaproteobacteria;o__Alteromonadales;f__Idiomarinaceae
k__Bacteria;p__Proteobacteria;c__Gammaproteobacteria;o__Alteromonadales;f__Moritellaceae
k__Bacteria;p__Proteobacteria;c__Gammaproteobacteria;o__Alteromonadales;f__Pseudoalteromonadaceae
k__Bacteria;p__Proteobacteria;c__Gammaproteobacteria;o__Alteromonadales;f__Psychromonadaceae
k__Bacteria;p__Proteobacteria;c__Gammaproteobacteria;o__Alteromonadales;f__Shewanellaceae
k__Bacteria;p__Proteobacteria;c__Gammaproteobacteria;o__Cardiobacteriales;f__Cardiobacteriaceae
k__Bacteria;p__Proteobacteria;c__Gammaproteobacteria;o__Chromatiales;f__
k__Bacteria;p__Proteobacteria;c__Gammaproteobacteria;o__Chromatiales;f__Chromatiaceae
k__Bacteria;p__Proteobacteria;c__Gammaproteobacteria;o__Chromatiales;f__Ectothiorhodospiraceae
k__Bacteria;p__Proteobacteria;c__Gammaproteobacteria;o__Chromatiales;f__Sinobacteraceae
k__Bacteria;p__Proteobacteria;c__Gammaproteobacteria;o__Enterobacteriales;f__Enterobacteriaceae
k__Bacteria;p__Proteobacteria;c__Gammaproteobacteria;o__Legionellales;f__
k__Bacteria;p__Proteobacteria;c__Gammaproteobacteria;o__Legionellales;f__Coxiellaceae
k__Bacteria;p__Proteobacteria;c__Gammaproteobacteria;o__Legionellales;f__Legionellaceae
k__Bacteria;p__Proteobacteria;c__Gammaproteobacteria;o__Methylococcales;f__
k__Bacteria;p__Proteobacteria;c__Gammaproteobacteria;o__Methylococcales;f__Crenotrichaceae
k__Bacteria;p__Proteobacteria;c__Gammaproteobacteria;o__Methylococcales;f__Methylococcaceae
k__Bacteria;p__Proteobacteria;c__Gammaproteobacteria;o__Oceanospirillales;f__
k__Bacteria;p__Proteobacteria;c__Gammaproteobacteria;o__Oceanospirillales;f__Alcanivoracaceae
k__Bacteria;p__Proteobacteria;c__Gammaproteobacteria;o__Oceanospirillales;f__Alteromonadaceae
k__Bacteria;p__Proteobacteria;c__Gammaproteobacteria;o__Oceanospirillales;f__Hahellaceae
k__Bacteria;p__Proteobacteria;c__Gammaproteobacteria;o__Oceanospirillales;f__Halomonadaceae
k__Bacteria;p__Proteobacteria;c__Gammaproteobacteria;o__Oceanospirillales;f__Oceanospirillaceae
k__Bacteria;p__Proteobacteria;c__Gammaproteobacteria;o__Oceanospirillales;f__Pseudomonadaceae
k__Bacteria;p__Proteobacteria;c__Gammaproteobacteria;o__Pasteurellales;f__Pasteurellaceae
k__Bacteria;p__Proteobacteria;c__Gammaproteobacteria;o__Pseudomonadales;f__
k__Bacteria;p__Proteobacteria;c__Gammaproteobacteria;o__Pseudomonadales;f__Moraxellaceae
k__Bacteria;p__Proteobacteria;c__Gammaproteobacteria;o__Salinisphaerales;f__Salinisphaeraceae
k__Bacteria;p__Proteobacteria;c__Gammaproteobacteria;o__Thiotrichales;f__
k__Bacteria;p__Proteobacteria;c__Gammaproteobacteria;o__Thiotrichales;f__Francisellaceae
k__Bacteria;p__Proteobacteria;c__Gammaproteobacteria;o__Thiotrichales;f__Piscirickettsiaceae
k__Bacteria;p__Proteobacteria;c__Gammaproteobacteria;o__Thiotrichales;f__Thiotrichaceae
k__Bacteria;p__Proteobacteria;c__Gammaproteobacteria;o__Vibrionales;f__Vibrionaceae
k__Bacteria;p__Proteobacteria;c__Gammaproteobacteria;o__Xanthomonadales;f__
k__Bacteria;p__Proteobacteria;c__Gammaproteobacteria;o__Xanthomonadales;f__Sinobacteraceae
k__Bacteria;p__Proteobacteria;c__Gammaproteobacteria;o__Xanthomonadales;f__Xanthomonadaceae
k__Bacteria;p__SC3;c__;o__;f__
k__Bacteria;p__SC4;c__;o__;f__
k__Bacteria;p__SPAM;c__;o__;f__
k__Bacteria;p__SR1;c__;o__;f__
k__Bacteria;p__Spirochaetes;c__Brachyspirae;o__Brachyspirales;f__Brachyspiraceae
k__Bacteria;p__Spirochaetes;c__Leptospirae;o__Leptospirales;f__Leptospiraceae
k__Bacteria;p__Spirochaetes;c__SP_WWE1;o__;f__
k__Bacteria;p__Spirochaetes;c__Spirochaetes (class);o__Spirochaetales;f__Spirochaetaceae
k__Bacteria;p__Synergistetes;c__Synergistia;o__Synergistales;f__Dethiosulfovibrionaceae
k__Bacteria;p__Synergistetes;c__Synergistia;o__Synergistales;f__Synergistaceae
k__Bacteria;p__Synergistetes;c__Synergistia;o__Synergistales;f__TTA_B6
k__Bacteria;p__TM6;c__;o__;f__
k__Bacteria;p__TM7;c__;o__;f__
k__Bacteria;p__TM7;c__TM7-1;o__;f__
k__Bacteria;p__TM7;c__TM7-3;o__CW040;f__
k__Bacteria;p__TM7;c__TM7-3;o__EW055;f__
k__Bacteria;p__TM7;c__TM7-3;o__I025;f__
k__Bacteria;p__Tenericutes;c__;o__;f__
k__Bacteria;p__Tenericutes;c__;o__;f__vadinHA31
k__Bacteria;p__Tenericutes;c__Erysipelotrichi;o__Erysipelotrichales;f__Erysipelotrichaceae
k__Bacteria;p__Tenericutes;c__ML615J-28;o__;f__
k__Bacteria;p__Tenericutes;c__Mollicutes;o__Acholeplasmatales;f__Acholeplasmataceae
k__Bacteria;p__Tenericutes;c__Mollicutes;o__Anaeroplasmatales;f__Anaeroplasmataceae
k__Bacteria;p__Tenericutes;c__Mollicutes;o__Entomoplasmatales;f__Entomoplasmataceae
k__Bacteria;p__Tenericutes;c__Mollicutes;o__Entomoplasmatales;f__Spiroplasmataceae
k__Bacteria;p__Tenericutes;c__Mollicutes;o__Mycoplasmatales;f__Mycoplasmataceae
k__Bacteria;p__Tenericutes;c__Mollicutes;o__RF39;f__
k__Bacteria;p__Thermi;c__Deinococci;o__Deinococcales;f__Deinococcaceae
k__Bacteria;p__Thermi;c__Deinococci;o__Thermales;f__Thermaceae
k__Bacteria;p__Thermotogae;c__Thermotogae (class);o__Thermotogales;f__Thermotogaceae
k__Bacteria;p__Verrucomicrobia;c__;o__;f__
k__Bacteria;p__Verrucomicrobia;c__;o__Methylacidiphilales;f__
k__Bacteria;p__Verrucomicrobia;c__Opitutae;o__;f__Opitutaceae
k__Bacteria;p__Verrucomicrobia;c__Opitutae;o__Puniceicoccales;f__Puniceicoccaceae
k__Bacteria;p__Verrucomicrobia;c__Spartobacteria;o__;f__
k__Bacteria;p__Verrucomicrobia;c__TP21;o__;f__
k__Bacteria;p__Verrucomicrobia;c__Verrucomicrobiae;o__Verrucomicrobiales;f__
k__Bacteria;p__Verrucomicrobia;c__Verrucomicrobiae;o__Verrucomicrobiales;f__Verrucomicrobia subdivision 3
k__Bacteria;p__Verrucomicrobia;c__Verrucomicrobiae;o__Verrucomicrobiales;f__Verrucomicrobiaceae
k__Bacteria;p__WPS-2;c__;o__;f__
k__Bacteria;p__WS3;c__PRR-12;o__;f__
k__Bacteria;p__WS3;c__PRR-12;o__LCP-67;f__
k__Bacteria;p__WS3;c__PRR-12;o__PRR-10;f__
k__Bacteria;p__ZB2;c__;o__;f__
