# Supplementary material for: Moving pictures of the human microbiome
Source: Genome Biol. 2011 May 30;12(5):R50. doi: 10.1186/gb-2011-12-5-r50 (PMC3271711; doi:10.1186/gb-2011-12-5-r50)

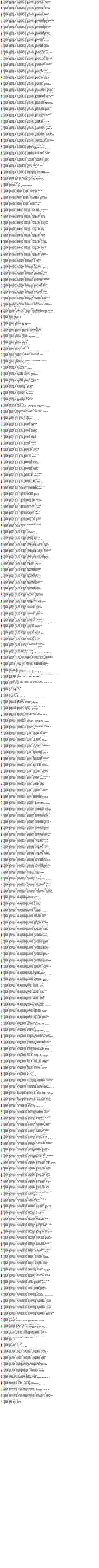

Supplement: Additional file 13 — Temporal variation in phylum, class, order, family, and genus abundances (F4 left palm). The x-axis scale differs between M3 and F4 plots. [file YnanGE3InOEfx0E0OYH0N058BDS9ZS_legend.pdf › AdditionalFile13/charts/YnanGE3InOEfx0E0OYH0N058BDS9ZS_legend.pdf]
