# Supplementary material for: Moving pictures of the human microbiome
Source: Genome Biol. 2011 May 30;12(5):R50. doi: 10.1186/gb-2011-12-5-r50 (PMC3271711; doi:10.1186/gb-2011-12-5-r50)

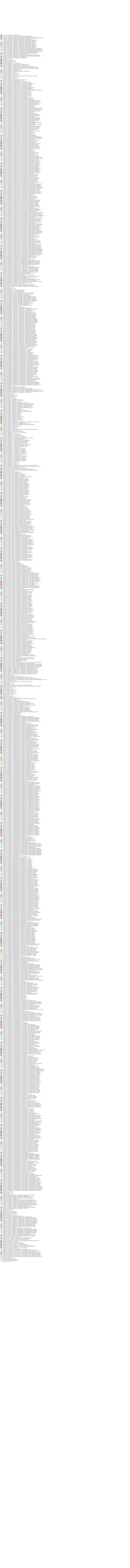

Supplement: Additional file 14 — Temporal variation in phylum, class, order, family, and genus abundances (M3 right palm). The x-axis scale differs between M3 and F4 plots. [file gb-2011-12-5-r50-S14.ZIP › AdditionalFile14/all.tar.bz2!/charts/QF9bzEn47fPeDFrazdy8ifI1S4Jexq_legend.pdf]
