# Supplementary material for: Moving pictures of the human microbiome
Source: Genome Biol. 2011 May 30;12(5):R50. doi: 10.1186/gb-2011-12-5-r50 (PMC3271711; doi:10.1186/gb-2011-12-5-r50)
Supplement: Additional file 14 — Temporal variation in phylum, class, order, family, and genus abundances (M3 right palm). The x-axis scale differs between M3 and F4 plots. [file gb-2011-12-5-r50-S14.ZIP › AdditionalFile14/all.tar.bz2!/charts/W02BnHrAf2rMJeOzuR1fmpYZgdGWox_legend.pdf]

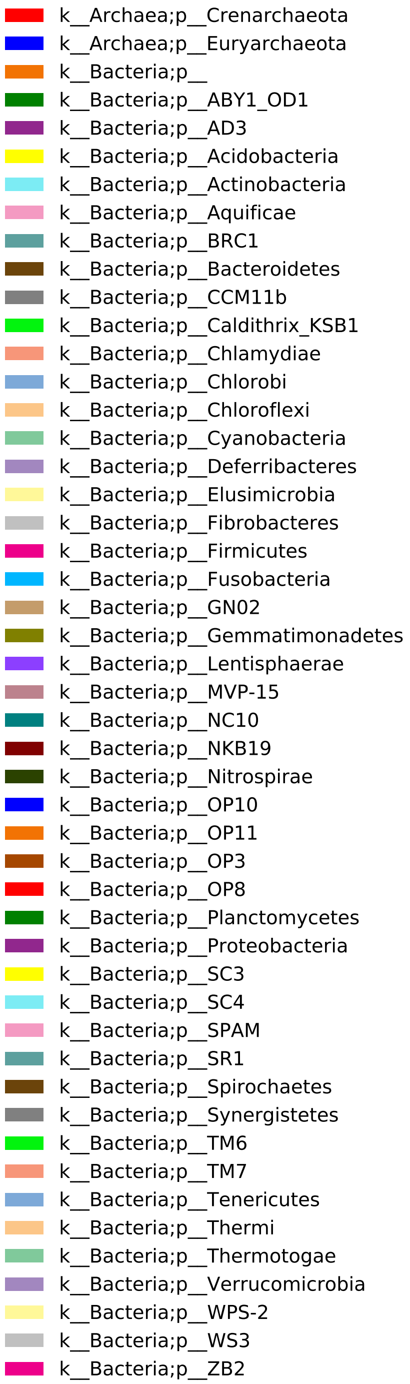
k__Archaea;p__Crenarchaeota
k__Archaea;p__Euryarchaeota
k__Bacteria;p__
k__Bacteria;p__ABY1_OD1
k__Bacteria;p__AD3
k__Bacteria;p__Acidobacteria
k__Bacteria;p__Actinobacteria
k__Bacteria;p__Aquificae
k__Bacteria;p__BRC1
k__Bacteria;p__Bacteroidetes
k__Bacteria;p__CCM11b
k__Bacteria;p__Caldithrix_KSB1
k__Bacteria;p__Chlamydiae
k__Bacteria;p__Chlorobi
k__Bacteria;p__Chloroflexi
k__Bacteria;p__Cyanobacteria
k__Bacteria;p__Deferribacteres
k__Bacteria;p__Elusimicrobia
k__Bacteria;p__Fibrobacteres
k__Bacteria;p__Firmicutes
k__Bacteria;p__Fusobacteria
k__Bacteria;p__GN02
k__Bacteria;p__Gemmatimonadetes
k__Bacteria;p__Lentisphaerae
k__Bacteria;p__MVP-15
k__Bacteria;p__NC10
k__Bacteria;p__NKB19
k__Bacteria;p__Nitrospirae
k__Bacteria;p__OP10
k__Bacteria;p__OP11
k__Bacteria;p__OP3
k__Bacteria;p__OP8
k__Bacteria;p__Planctomycetes
k__Bacteria;p__Proteobacteria
k__Bacteria;p__SC3
k__Bacteria;p__SC4
k__Bacteria;p__SPAM
k__Bacteria;p__SR1
k__Bacteria;p__Spirochaetes
k__Bacteria;p__Synergistetes
k__Bacteria;p__TM6
k__Bacteria;p__TM7
k__Bacteria;p__Tenericutes
k__Bacteria;p__Thermi
k__Bacteria;p__Thermotogae
k__Bacteria;p__Verrucomicrobia
k__Bacteria;p__WPS-2
k__Bacteria;p__WS3
k__Bacteria;p__ZB2
