# Supplementary material for: Moving pictures of the human microbiome
Source: Genome Biol. 2011 May 30;12(5):R50. doi: 10.1186/gb-2011-12-5-r50 (PMC3271711; doi:10.1186/gb-2011-12-5-r50)
Supplement: Additional file 14 — Temporal variation in phylum, class, order, family, and genus abundances (M3 right palm). The x-axis scale differs between M3 and F4 plots. [file gb-2011-12-5-r50-S14.ZIP › AdditionalFile14/all.tar.bz2!/index.html]

 
 
 
 

 
 Taxa Summaries 
 
 
  
 
     
  Taxonomy Summary. Current Level: Phylum  
     View Figure (.pdf)    View Legend (.pdf)   
   
 
     
 
 

 
 
 
 
 
 
 
 
 
 
 
 
 
 
 
 
 
 
 
 
 
 
 
 
 
 
 
 
 
 
 
 
 
 
 
 
 
 
 
 
 
 
 
 
 
 
 
 
 
 
 
 
 
 
 
 
 
 
 
 
 
 
 
 
 
 
 
 
 
 
 
 
 
 
 
 
 
 
 
 
 
 
 
 
 
 
 
 
 
 
 
 
 
 
 
 
 
 
 
 
 
 
 
 
 
 
 
 
 
 
 
 
 
 
 
 
 
 
 
 
 
 
 
 
 
 
 
 
 
 
 
 
 
 
 
 
 
 
 
 
 
 
 
 
 
 
 
 
 
 
 
 
 
 
 
 
 
 
 
 
 
 
 
 
 
 
 
 
 
 
 
 
 
 
 
 
 
 
 
 
 
 
 
 
 
 
 
 
 
 
 
 
 
 
 
 
 
 
 
 
 
 
 
 
 
 
 
 
 
 
 
 
 
 
 
 
 
 
 
 
 
 
 
 
 
 
 
 
 
 
 
 
 
 
 
 
 
 
 
 
 
 
 
 
 
 
 
 
 
 
 
 
 
 
 
 
 
 
 
 
 
 
 
 
 
 
 
 
 
 
 
 
 
 
 
 
 
 
 
 
 
 
 
 
 
 
 
 
 
 
 
 
 
 
 
 
 
 
 
 
 
 
 
 
 
 
 
 
 
 
 
 
 
 
 
 
 
 
 
 
 
 
 
 
 
 
 
 
 
 
 
 
 
 
 
 
 
 
 
 
 
 
 
 
 
 
 
 
 
 
 
 
 
 
 
 
 
 
 
 
 
 
 
 
 
 
 
 
 
 
 
 
 
 
 
 
 
 
 
 
 
 
 
 
 
 
 
 
 
 
 
 
 
 
 
 
 
 
 
 
 
 
 
 
 
 
 
 
 
 
 
 
 
 
 
 
 
 
 
 
 
 
 
 
 
 
 
 
 
 
 
 
 
 
 
 
 
 
 
 
 
 
 
 
 
 
 
 
 
 
 
 
 
 
 
 
 
 
 
 
 
 
 
 
 
 
 
 
 
 
 
 
 
 
 
 
 
 
 
 
 
 
 
 
 
 
 
 
 
 
 
 
 
 
 
 
 
 
 
 
 
 
 
 
 
 
 
 
 
 
 
 
 
 
 
 
 
 
 
 
 
 
 
 
 
 
 
 
 
 
 
 
 
 
 
 
 
 
 
 
 
 
 
 
 
 
 
 
 
 
 
 
 
 
 
 
 
 
 
 
 
 
 
 
 
 
 
 
 
 
 
 
 
 
 
 
 
 
 
 
 
 
 
 
 
 
 
 
 
 
 
 
 
 
 
 
 
 
 
 
 
 
 
 
 
 
 
 
 
 
 
 
 
 
 
 
 
 
 
 
 
 
 
 
 
 
 
 
 
 
 
 
 
 
 
 
 
 
 
 
 
 
 
 
 
 
 
 
 
 
 
 
 
 
 
 
 
 
 
 
 
 
 
 
 
 
 
 
 
 
 
 
 
 
 
 
 
 
 
 
 
 
 
 
 
 
 
 
 
 
 
 
 
 
 
 
 
 
 
 
 
 
 
 
 
 
 
 
 
 
 
 
 
 
 
 
 
 
 
 
 
 
 
 
 
 
 
 
 
 
 
 
 
 
 
 
 
 
 
 
 
 
 
 
 
 
 
 
 
 
 
 
 
 
 
 
 
 
 
 
 
 
 
 
 
 
 
 
 
 
 
 
 
 
 
 
 
 
 
 
 
 
 
 
 
 
 
 
 
 
 
 
 
 
 
 
 
 
 
 
 
 
 
 
 
 
 
 
 
 
 
 
 
 
 
 
 
 
 
 
 
 
 
 
 
 
 
 
 
 
 
 
 
 
 
 
 
 
 
 
 
 
 
 
 
 
 
 
 
 
 
 
 
 
 
 
 
 
 
 
 
 
 
 
 
 
 
 
 
 
 
 
 
 
 
 
 
 
 
 
 
 
 
 
 
 
 
 
 
 
 
 
 
 
 
 
 
 
 
 
 
 
 
 
 
 
 
 
 
 
 
 
 
 
 
 
 
 
 
 
 
 
 
 
 
 
 
 
 
 
 
 
 
 
 
 
 
 
 
 
 
 
 
 
 
 
 
 
 
 
 
 
 
 
 
 
 
 
 
 
 
 
 
 
 
 
 
 
 
 
 
 
 
 
 
 
 
 
 
 
 
 
 
 
 
 
 
 
 
 
 
 
 
 
 
 
 
 
 
 
 
 
 
 
 
 
 
 
 
 
 
 
 
 
 
 
 
 
 
 
 
 
 
 
 
 
 
 
 
 
 
 
 
 
 
 
 
 
 
 
 
 
 
 
 
 
 
 
 
 
 
 
 
 
 
 
 
 
 
 
 
 
 
 
 
 
 
 
 
 
 
 
 
 
 
 
 
 
 
 
 
 
 
 
 
 
 
 
 
 
 
 
 
 
 
 
 
 
 
 
 
 
 
 
 
 
 
 
 
 
 
 
 
 
 
 
 
 
 
 
 
 
 
 
 
 
 
 
 
 
 
 
 
 
 
 
 
 
 
 
 
 
 
 
 
 
 
 
 
 
 
 
 
 
 
 
 
 
 
 
 
 
 
 
 
 
 
 
 
 
 
 
 
 
 
 
 
 
 
 
 
 
 
 
 
 
 
 
 
 
 
 
 
 
 
 
 
 
 
 
 
 
 
 
 
 
 
 
 
 
 
 
 
 
 
 
 
 
 
 
 
 
 
 
 
 
 
 
 
 
 
 
 
 
 
 
 
 
 
 
 
 
 
 
 
 
 
 
 
 
 
 
 
 
 
 
 
 
 
 
 
 
 
 
 
 
 
 
 
 
 
 
 
 
 
 
 
 
 
 
 
 
 
 
 
 
 
 
 
 
 
 
 
 
 
 
 
 
 
 
 
 
 
 
 
 
 
 
 
 
 
 
 
 
 
 
 
 
 
 
 
 
 
 
 
 
 
 
 
 
 
 
 
 
 
 
 
 
 
 
 
 
 
 
 
 
 
 
 
 
 
 
 
 
 
 
 
 
 
 
 
 
 
 
 
 
 
 
 
 
 
 
 
 
 
 
 
 
 
 
 
 
 
 
 
 
 
 
 
 
 
 
 
 
 
 
 
 
 
 
 
 
 
 
 
 
 
 
 
 
 
 
 
 
 
 
 
 
 
 
 
 
 
 
 
 
 
 
 
 
 
 
 
 
 
 
 
 
 
 
 
 
 
 
 
 
 
 
 
 
 
 
 
 
 
 
 
 
 
 
 
 
 
 
 
 
 
 
 
 
 
 
 
 
 
 
 
 
 
 
 
 
 
 
 
 
 
 
 
 
 
 
 
 
 
 
 
 
 
 
 
 
 
 
 
 
 
 
 
 
 
 
 
 
 
 
 
 
 
 
 
 
 
 
 
 
 
 
 
 
 
 
 
 
 
 
 
 
 
 
 
 
 
 
 
 
 
 
 
 
 
 
 
 
 
 
 
 
 
 
 
 
 
 
 
 
 
 
 
 
 
 
 
 
 
 
 
 
 
 
 
 
 
 
 
 
 
 
 
 
 
 
 
 
 
 
 
 
 
 
 
 
 
 
 
 
 
 
 
 
 
 
 
 
 
 
 
 
 
 
 
 
 
 
 
 
 
 
 
 
 
 
 
 
 
 
 
 
 
 
 
 
 
 
 
 
 
 
 
 
 
 
 
 
 
 
 
 
 
 
 
 
 
 
 
 
 
 
 
 
 
 
 
 
 
 
 
 
 
 
 
 
 
 
 
 
 
 
 
 
 
 
 
 
 
 
 
 
 
 
 
 
 
 
 
 
 
 
 
 
 
 
 
 
 
 
 
 
 
 
 
 
 
 
 
 
 
 
 
 
 
 
 
 
 
 
 
 
 
 
 
 
 
 
 
 
 
 
 
 
 
 
 
 
 
 
 
 
 
 
 
 
 
 
 
 
 
 
 
 
 
 
 
 
 
 
 
 
 
 
 
 
 
 
 
 
 
 
 
 
 
 
 
 
 
 
 
 
 
 
 
 
 
 
 
 
 
 
 
 
 
 
 
 
 
 
 
 
 
 
 
 
 
 
 
 
 
 
 
 
 
 
 
 
 
 
 
 
 
 
 
 
 
 
 
 
 
 
 
 
 
 
 
 
 
 
 
 
 
 
 
 
 
 
 
 
 
 
 
 
 
 
 
 
 
 
 
 
 
 
 
 
 
 
 
 
 
 
 
 
 
 
 
 
 
 
 
 
 
 
 
 
 
 
 
 
 
 
 
 
 
 
 
 
 
 
 
 
 
 
 
 
 
 
 
 
 
 
 
 
 
 
 
 
 
 
 
 
 
 
 
 
 
 
 
 
 
 
 
 
 
 
 
 
 
 
 
 
 
 
 
 
 
 
 
 
 
 
 
 
 
 
 
 
 
 
 
 
 
 
 
 
 
 
 
 
 
 
 
 
 
 
 
 
 
 
 
 
 
 
 
 
 
 
 
 
 
 
 
 
 
 
 
 
 
 
 
 
 
 
 
 
 
 
 
 
 
 
 
 
 
 
 
 
 
 
 
 
 
 
 
 
 
 
 
 
 
 
 
 
 
 
 
 
 
 
 
 
 
 
 
 
 
 
 
 
 
 
 
 
 
 
 
 
 
 
 
 
 
 
 
 
 
 
 
 
 
 
 
 
 
 
 
 
 
 
 
 
 
 
 
 
 
 
 
 
 
 
 
 
 
 
 
 
 
 
 
 
 
 
 
 
 
 
 
 
 
 
 
 
 
 
 
 
 
 
 
 
 
 
 
 
 
 
 
 
 
 
 
 
 
 
 
 
 
 
 
 
 
 
 
 
 
 
 
 
 
 
 
 
 
 
 
 
 
 
 
 
 
 
 
 
 
 
 
 
 
 
 
 
 
 
 
 
 
 
 
 
 
 
 
 
 
 
 
 
 
 
 
 
 
 
 
 
 
 
 
 
 
 
 
 
 
 
 
 
 
 
 
 
 
 
 
 
 
 
 
 
 
 
 
 
 
 
 
 
 
 
 
 
 
 
 
 
 
 
 
 
 
 
 
 
 
 
 
 
 
 
 
 
 
 
 
 
 
 
 
 
 
 
 
 
 
 
 
 
 
 
 
 
 
 
 
 
 
 
 
 
 
 
 
 
 
 
 
 
 
 
 
 
 
 
 
 
 
 
 
 
 
 
 
 
 
 
 
 
 
 
 
 
 
 
 
 
 
 
 
 
 
 
 
 
 
 
 
 
 
 
 
 
 
 
 
 
 
 
 
 
 
 
 
 
 
 
 
 
 
 
 
 
 
 
 
 
 
 
 
 
 
 
 
 
 
 
 
 
 
 
 
 
 
 
 
 
 
 
 
 
 
 
 
 
 
 
 
 
 
 
 
 
 
 
 
 
 
 
 
 
 
 
 
 
 
 
 
 
 
 
 
 
 
 
 
 
 
 
 
 
 
 
 
 
 
 
 
 
 
 
 
 
 
 
 
 
 
 
 
 
 
 
 
 
 
 
 
 
 
 
 
 
 
 
 
 
 
 
 
 
 
 
 
 
 
 
 
 
 
 
 
 
 
 
 
 
 
 
 
 
 
 
 
 
 
 
 
 
 
 
 
 
 
 
 
 
 
 
 
 
 
 
 
 
 
 
 
 
 
 
 
 
 
 
 
 
 
 
 
 
 
 
 
 
 
 
 
 
 
 
 
 
 
 
 
 
 
 
 
 
 
 
 
 
 
 
 
 
 
 
 
 
 
 
 
 
 
 
 
 
 
 
 
 
 
 
 
 
 
 
 
 
 
 
 
 
 
 
 
 
 
 
 
 
 
 
 
 
 
 
 
 
 
 
 
 
 
 
 
 
 
 
 
 
 
 
 
 
 
 
 
 
 
 
 
 
 
 
 
 
 
 
 
 
 
 
 
 
 
 
 
 
 
 
 
 
 
 
 
 
 
 
 
 
 
 
 
 
 
 
 
 
 
 
 
 
 
 
 
 
 
 
 
 
 
 
 
 
 
 
 
 
 
 
 
 
 
 
 
 
 
 
 
 
 
 
 
 
 
 
 
 
 
 
 
 
 
 
 
 
 
 
 
 
 
 
 
 
 
 
 
 
 
 
 
 
 
 
 
 
 
 
 
 
 
 
 
 
 
 
 
 
 
 
 
 
 
 
 
 
 
 
 
 
 
 
 
 
 
 
 
 
 
 
 
 
 
 
 
 
 
 
 
 
 
 
 
 
 
 
 
 
 
 
 
 
 
 
 
 
 
 
 
 
 
 
 
 
 
 
 
 
 
 
 
 
 
 
 
 
 
 
 
 
 
 
 
 
 
 
 
 
 
 
 
 
 
 
 
 
 
 
 
 
 
 
 
 
 
 
 
 
 
 
 
 
 
 
 
 
 
 
 
 
 
 
 
 
 
 
 
 
 
 
 
 
 
 
 
 
 
 
 
 
 
 
 
 
 
 
 
 
 
 
 
 
 
 
 
 
 
 
 
 
 
 
 
 
 
 
 
 
 
 
 
 
 
 
 
 
 
 
 
 
 
 
 
 
 
 
 
 
 
 
 
 
 
 
 
 
 
 
 
 
 
 
 
 
 
 
 
 
 
 
 
 
 
 
 
 
 
 
 
 
 
 
 
 
 
 
 
 
 
 
 
 
 
 
 
 
 
 
 
 
 
 
 
 
 
 
 
 
 
 
 
 
 
 
 
 
 
 
 
 
 
 
 
 
 
 
 
 
 
 
 
 
 
 
 
 
 
 
 
 
 
 
 
 
 
 
 
 
 
 
 
 
 
 
 
 
 
 
 
 
 
 
 
 
 
 
 
 
 
 
 
 
 
 
 
 
 
 
 
 
 
 
 
 
 
 
 
 
 
 
 
 
 
 
 
 
 
 
 
 
 
 
 
 
 
 
 
 
 
 
 
 
 
 
 
 
 
 
 
 
 
 
 
 
 
 
 
 
 
 
 
 
 
 
 
 
 
 
 
 
 
 
 
 
 
 
 
 
 
 
 
 
 
 
 
 
 
 
 
 
 
 
 
 
 
 
 
 
 
 
 
 
 
 
 
 
 
 
 
 
 
 
 
 
 
 
 
 
 
 
 
 
 
 
 
 
 
 
 
 
 
 
 
 
 
 
 
 
 
 
 
 
 
 
 
 
 
 
 
 
 
 
 
 
 
 
 
 
 
 
 
 
 
 
 
 
 
 
 
 
 
 
 
 
 
 
 
 
 
 
 
 
 
 
 
 
 
 
 
 
 
 
 
 
 
 
 
 
 
 
 
 
 
 
 
 
 
 
 
 
 
 
 
 
 
 
 
 
 
 
 
 
 
 
 
 
 
 
 
 
 
 
 
 
 
 
 
 
 
 
 
 
 
 
 
 
 
 
 
 
 
 
 
 
 
 
 
 
 
 
 
 
 
 
 
 
 
 
 
 
 
 
 
 
 
 
 
 
 
 
 
 
 
 
 
 
 
 
 
 
 
 
 
 
 
 
 
 
 
 
 
 
 
 
 
 
 
 
 
 
 
 
 
 
 
 
 
 
 
 
 
 
 
 
 
 
 
 
 
 
 
 
 
 
 
 
 
 
 
 
 
 
 
 
 
 
 
 
 
 
 
 
 
 
 
 
 
 
 
 
 
 
 
 
 
 
 
 
 
 
 
 
 
 
 
 
 
 
 
 
 
 
 
 
 
 
 
 
 
 
 
 
 
 
 
 
 
 
 
 
 
 
 
 
 
 
 
 
 
 
 
 
 
 
 
 
 
 
 
 
 
 
 
 
 
 
 
 
 
 
 
 
 
 
 
 
 
 
 
 
 
 
 
 
 
 
 
 
 
 
 
 
 
 
 
 
 
 
 
 
 
 
 
 
 
 
 
 
 
 
 
 
 
 
 
 
 
 
 
 
 
 
 
 
 
 
 
 
 
 
 
 
 
 
 
 
 
 
 
 
 
 
 
 
 
 
 
 
 
 
 
 
 
 
 
 
 
 
 
 
 
 
 
 
 
 
 
 
 
 
 
 
 
 
 
 
 
 
 
 
 
 
 
 
 
 
 
 
 
 
 
 
 
 
 
 
 
 
 
 
 
 
 
 
 
 
 
 
 
 
 
 
 
 
 
 
 
 
 
 
 
 
 
 
 
 
 
 
 
 
 
 
 
 
 
 
 
 
 
 
 
 
 
 
 
 
 
 
 
 
 
 
 
 
 
 
 
 
 
 
 
 
 
 
 
 
 
 
 
 
 
 
 
 
 
 
 
 
 
 
 
 
 
 
 
 
 
 
 
 
 
 
 
 
 
 
 
 
 
 
 
 
 
 
 
 
 
 
 
 
 
 
 
 
 
 
 
 
 
 
 
 
 
 
 
 
 
 
 
 
 
 
 
 
 
 
 
 
 
 
 
 
 
 
 
 
 
 
 
 
 
 
 
 
 
 
 
 
 
 
 
 
 
 
 
 
 
 
 
 
 
 
 
 
 
 
 
 
 
 
 
 
 
 
 
 
 
 
 
 
 
 
 
 
 
 
 
 
 
 
 
 
 
 
 
 
 
 
 
 
 
 
 
 
 
 
 
 
 
 
 
 
 
 
 
 
 
 
 
 
 
 
 
 
 
 
 
 
 
 
 
 
 
 
 
 
 
 
 
 
 
 
 
 
 
 
 
 
 
 
 
 
 
 
 
 
 
 
 
 
 
 
 
 
 
 
 
 
 
 
 
 
 
 
 
 
 
 
 
 
 
 
 
 
 
 
 
 
 
 
 
 
 
 
 
 
 
 
 
 
 
 
 
 
 
 
 
 
 
 
 
 
 
 
 
 
 
 
 
 
 
 
 
 
 
 
 
 
 
 
 
 
 
 
 
 
 
 
 
 
 
 
 
 
 
 
 
 
 
 
 
 
 
 
 
 
 
 
 
 
 
 
 
 
 
 
 
 
 
 
 
 
 
 
 
 
 
 
 
 
 
 
 
 
 
 
 
 
 
 
 
 
 
 
 
 
 
 
 
 
 
 
 
 
 
 
 
 
 
 
 
 
 
 
 
 
 
 
 
 
 
 
 
 
 
 
 
 
 
 
 
 
 
 
 
 
 
 
 
 
 
 
 
 
 
 
 
 
 
 
 
 
 
 
 
 
 
 
 
 
 
 
 
 
 
 
 
 
 
 
 
 
 
 
 
 
 
 
 
 
 
 
 
 
 
 
 
 
 
 
 
 
 
 
 
 
 
 
 
 
 
 
 
 
 
 
 
 
 
 
 
 
 
 
 
 
 
 
 
 
 
 
 
 
 
 
 
 
 
 
 
 
 
 
 
 
 
 
 
 
 
 
 
 
 
 
 
 
 
 
 
 
 
 
 
 
 
 
 
 
 
 
 
 
 
 
 
 
 
 
 
 
 
 
 
 
 
 
 
 
 
 
 
 
 
 
 
 
 
 
 
 
 
 
 
 
 
 
 
 
 
 
 
 
 
 
 
 
 
 
 
 
 
 
 
 
 
 
 
 
 
 
 
 
 
 
 
 
 
 
 
 
 
 
 
 
 
 
 
 
 
 
 
 
 
 
 
 
 
 
 
 
 
 
 
 
 
 
 
 
 
 
 
 
 
 
 
 
 
 
 
 
 
 
 
 
 
 
 
 
 
 
 
 
 
 
 
 
 
 
 
 
 
 
 
 
 
 
 
 
 
 
 
 
 
 
 
 
 
 
 
 
 
 
 
 
 
 
 
 
 
 
 
 
 
 
 
 
 
 
 
 
 
 
 
 
 
 
 
 
 
 
 
 
 
 
 
 
 
 
 
 
 
 
 
 
 
 
 
 
 
 
 
 
 
 
 
 
 
 
 
 
 
 
 
 
 
 
 
 
 
 
 
 
 
 
 
 
 
 
 
 
 
 
 
 
 
 
 
 
 
 
 
 
 
 
 
 
 
 
 
 
 
 
 
 
 
 
 
 
 
 
 
 
 
 
 
 
 
 
 
 
 
 
 
 
 
 
 
 
 
 
 
 
 
 
 
 
 
 
 
 
 
 
 
 
 
 
 
 
 
 
 
 
 
 
 
 
 
 
 
 
 
 
 
 
 
 
 
 
 
 
 
 
 
 
 
 
 
 
 
 
 
 
 
 
 
 
 
 
 
 
 
 
 
 
 
 
 
 
 
 
 
 
 
 
 
 
 
 
 
 
 
 
 
 
 
 
 
 
 
 
 
 
 
 
 
 
 
 
 
 
 
 
 
 
 
 
 
 
 
 
 
 
 
 
 
 
 
 
 
 
 
 
 
 
 
 
 
 
 
 
 
 
 
 
 
 
 
 
 
 
 
 
 
 
 
 
 
 
 
 
 
 
 
 
 
 
 
 
 
 
 
 
 
 
 
 
 
 
 
 
 
 
 
 
 
 
 
 
 
 
 
 
 
 
 
 
 
 
 
 
 
 
 
 
 
 
 
 
 
 
 
 
 
 
 
 
 
 
 
 
 
 
 
 
 
 
 
 
 
 
 
 
 
 
 
 
 
 
 
 
 
 
 
 
 
 
 
 
 
 
 
 
 
 
 
 
 
 
 
 
 
 
 
 
 
 
 
 
 
 
 
 
 
 
 
 
 
 
 
 
 
 
 
 
 
 
 
 
 
 
 
 
 
 
 
 
 
 
 
 
 
 
 
 
 
 
 
 
 
 
 
 
 
 
 
 
 
 
 
 
 
 
 
 
 
 
 
 
 
 
 
 
 
 
 
 
 
 
 
 
 
 
 
 
 
 
 
 
 
 
 
 
 
 
 
 
 
 
 
 
 
 
 
 
 
 
 
 
 
 
 
 
 
 
 
 
 
 
 
 
 
 
 
 
 
 
 
 
 
 
 
 
 
 
 
 
 
 
 
 
 
 
 
 
 
 
 
 
 
 
 
 
 
 
 
 
 
 
 
 
 
 
 
 
 
 
 
 
 
 
 
 
 
 
 
 
 
 
 
 
 
 
 
 
 
 
 
 
 
 
 
 
 
 
 
 
 
 
 
 
 
 
 
 
 
 
 
 
 
 
 
 
 
 
 
 
 
 
 
 
 
 
 
 
 
 
 
 
 
 
 
 
 
 
 
 
 
 
 
 
 
 
 
 
 
 
 
 
 
 
 
 
 
 
 
 
 
 
 
 
 
 
 
 
 
 
 
 
 
 
 
 
 
 
 
 
 
 
 
 
 
 
 
 
 
 
 
 
 
 
 
 
 
 
 
 
 
 
 
 
 
 
 
 
 
 
 
 
 
 
 
 
 
 
 
 
 
 
 
 
 
 
 
 
 
 
 
 
 
 
 
 
 
 
 
 
 
 
 
 
 
 
 
 
 
 
 
 
 
 
 
 
 
 
 
 
 
 
 
 
 
 
 
 
 
 
 
 
 
 
 
 
 
 
 
 
 
 
 
 
 
 
 
 
 
 
 
 
 
 
 
 
 
 
 
 
 
 
 
 
 
 
 
 
 
 
 
 
 
 
 
 
 
 
 
 
 
 
 
 
 
 
 
 
 
 
 
 
 
 
 
 
 
 
 
 
 
 
 
 
 
 
 
 
 
 
 
 
 
 
 
 
 
 
 
 
 
 
 
 
 
 
 
 
 
 
 
 
 
 
 
 
 
 
 
 
 
 
 
 
 
 
 
 
 
 
 
 
 
 
 
 
 
 
 
 
 
 
 
 
 
 
 
 
 
 
 
 
 
 
 
 
 
 
 
 
 
 
 
 
 
 
 
 
 
 
 
 
 
 
 
 
 
 
 
 
 
 
 
 
 
 
 
 
 
 
 
 
 
 
 
 
 
 
 
 
 
 
 
 
 
 
 
 
 
 
 
 
 
 
 
 
 
 
 
 
 
 
 
 
 
 
 
 
 
 
 
 
 
 
 
 
 
 
 
 
 
 
 
 
 
 
 
 
 
 
 
 
 
 
 
 
 
 
 
 
 
 
 
 
 
 
 
 
 
 
 
 
 
 
 
 
 
 
 
 
 
 
 
 
 
 
 
 
 
 
 
 
 
 
 
 
 
 
 
 
 
 
 
 
 
 
 
 
 
 
 
 
 
 
 
 
 
 
 
 
 
 
 
 
 
 
 
 
 
 
 
 
 
 
 
 
 
 
 
 
 
 
 
 
 
 
 
 
 
 
 
 
 
 
 
 
 
 
 
 
 
 
 
 
 
 
 
 
 
 
 
 
 
 
 
 
 
 
 
 
 
 
 
 
 
 
 
 
 
 
 
 
 
 
 
 
 
 
 
 
 
 
 
 
 
 
 
 
 
 
 
 
 
 
 
 
 
 
 
 
 
 
 
 
 
 
 
 
 
 
 
 
 
 
 
 
 
 
 
 
 
 
 
 
 
 
 
 
 
 
 
 
 
 
 
 
 
 
 
 
 
 
 
 
 
 
 
 
 
 
 
 
 
 
 
 
 
 
 
 
 
 
 
 
 
 
 
 
 
 
 
 
 
 
 
 
 
 
 
 
 
 
 
 
 
 
 
 
 
 
 
 
 
 
 
 
 
 
 
 
 
 
 
 
 
 
 
 
 
 
 
 
 
 
 
 
 
 
 
 
 
 
 
 
 
 
 
 
 
 
 
 
 
 
 
 
 
 
 
 
 
 
 
 
 
 
 
 
 
 
 
 
 
 
 
 
 
 
 
 
 
 
 
 
 
 
 
 
 
 
 
 
 
 
 
 
 
 
 
 
 
 
 
 
 
 
 
 
 
 
 
 
 
 
 
 
 
 
 
 
 
 
 
 
 
 
 
 
 
 
 
 
 
 
 
 
 
 
 
 
 
 
 
 
 
 
 
 
 
 
 
 
 
 
 
 
 
 
 
 
 
 
 
 
 
 
 
 
 
 
 
 
 
 
 
 
 
 
 
 
 
 
 
 
 
 
 
 
 
 
 
 
 
 
 
 
 
 
 
 
 
 
 
 
 
 
 
 
 
 
 
 
 
 
 
 
 
 
 
 
 
 
 
 
 
 
 
 
 
 
 
 
 
 
 
 
 
 
 
 
 
 
 
 
 
 
 
 
 
 
 
 
 
 
 
 
 
 
 
 
 
 
 
 
 
 
 
 
 
 
 
 
 
 
 
 
 
 
 
 
 
 
 
 
 
 
 
 
 
 
 
 
 
 
 
 
 
 
 
 
 
 
 
 
 
 
 
 
 
 
 
 
 
 
 
 
 
 
 
 
 
 
 
 
 
 
 
 
 
 
 
 
 
 
 
 
 
 
 
 
 
 
 
 
 
 
 
 
 
 
 
 
 
 
 
 
 
 
 
 
 
 
 
 
 
 
 
 
 
 
 
 
 
 
 
 
 
 
 
 
 
 
 
 
 
 
 
 
 
 
 
 
 
 
 
 
 
 
 
 
 
 
 
 
 
 
 
 
 
 
 
 
 
 
 
 
 
 
 
 
 
 
 
 
 
 
 
 
 
 
 
 
 
 
 
 
 
 
 
 
 
 
 
 
 
 
 
 
 
 
 
 
 
 
 
 
 
 
 
 
 
 
 
 
 
 
 
 
 
 
 
 
 
 
 
 
 
 
 
 
 
 
 
 
 
 
 
 
 
 
 
 
 
 
 
 
 
 
 
 
 
 
 
 
 
 
 
 
 
 
 
 
 
 
 
 
 
 
 
 
 
 
 
 
 
 
 
 
 
 
 
 
 
 
 
 
 
 
 
 
 
 
 
 
 
 
 
 
 
 
 
 
 
 
 
 
 
 
 
 
 
 
 
 
 
 
 
 
 
 
 
 
 
 
 
 
 
 
 
 
 
 
 
 
 
 
 
 
 
 
 
 
 
 
 
 
 
 
 
 
 
 
 
 
 
 
 
 
 
 
 
 
 
 
 
 
 
 
 
 
 
 
 
 
 
 
 
 
 
 
 
 
 
 
 
 
 
 
 
 
 
 
 
 
 
 
 
 
 
 
 
 
 
 
 
 
 
 
 
 
 
 
 
 
 
 
 
 
 
 
 
 
 
 
 
 
 
 
 
 
 
 
 
 
 
 
 
 
 
 
 
 
 
 
 
 
 
 
 
 
 
 
 
 
 
 
 
 
 
 
 
 

 

    View Table (.txt)         Total  0  1  2  3  4  5  6  8  9  10  11  13  14  15  16  17  18  19  20  21  22  23  24  25  26  27  28  29  30  31  32  41  42  43  44  45  46  47  48  49  50  51  52  53  54  56  57  58  59  60  61  62  64  65  66  67  68  69  70  71  72  73  79  80  81  82  83  84  86  87  88  89  90  91  92  93  94  95  96  97  98  99  100  101  102  103  104  105  112  113  114  115  116  117  118  120  121  122  123  124  125  126  127  128  129  130  131  132  134  135  137  138  139  140  141  142  143  144  145  146  147  148  149  150  151  152  153  154  155  157  158  159  160  161  162  163  164  166  167  168  169  170  172  173  174  175  176  177  178  179  180  181  182  183  184  185  186  187  188  189  190  191  192  193  194  195  196  197  198  199  200  201  202  203  204  205  206  208  209  210  211  213  214  215  216  217  218  219  220  221  222  223  225  226  227  228  229  230  231  234  235  236  237  238  240  241  243  244  245  246  247  249  250  251  252  253  254  255  257  258  259  260  261  262  263  264  266  267  268  269  270  271  272  273  274  275  278  279  280  281  283  284  285  286  287  288  289  290  292  293  294  295  296  297  298  299  300  301  302  303  304  305  306  307  308  309  312  314  317  318  319  321  322  323  326  327  330  331  332  333  334  335  336  337  338  339  340  341  343  344  345  348  349  350  351  352  353  354  355  356  357  358  359  360  361  362  363  365  366  368  369  371  372  373  374  375  376  377  378  379  380  381  382  383  384  385  386  387  389  390  391  392  393  394  395  396  397  398  399  400  401  402  403  405  406  410  411  412  413  415  416  417  418  420  438  439  440    Legend  Taxonomy  count  %  %  %  %  %  %  %  %  %  %  %  %  %  %  %  %  %  %  %  %  %  %  %  %  %  %  %  %  %  %  %  %  %  %  %  %  %  %  %  %  %  %  %  %  %  %  %  %  %  %  %  %  %  %  %  %  %  %  %  %  %  %  %  %  %  %  %  %  %  %  %  %  %  %  %  %  %  %  %  %  %  %  %  %  %  %  %  %  %  %  %  %  %  %  %  %  %  %  %  %  %  %  %  %  %  %  %  %  %  %  %  %  %  %  %  %  %  %  %  %  %  %  %  %  %  %  %  %  %  %  %  %  %  %  %  %  %  %  %  %  %  %  %  %  %  %  %  %  %  %  %  %  %  %  %  %  %  %  %  %  %  %  %  %  %  %  %  %  %  %  %  %  %  %  %  %  %  %  %  %  %  %  %  %  %  %  %  %  %  %  %  %  %  %  %  %  %  %  %  %  %  %  %  %  %  %  %  %  %  %  %  %  %  %  %  %  %  %  %  %  %  %  %  %  %  %  %  %  %  %  %  %  %  %  %  %  %  %  %  %  %  %  %  %  %  %  %  %  %  %  %  %  %  %  %  %  %  %  %  %  %  %  %  %  %  %  %  %  %  %  %  %  %  %  %  %  %  %  %  %  %  %  %  %  %  %  %  %  %  %  %  %  %  %  %  %  %  %  %  %  %  %  %  %  %  %  %  %  %  %  %  %  %  %  %  %  %  %  %  %  %  %  %  %  %  %  %  %  %  %  %  %  %  %  %  %  %  %  %  %  %  %  %  %  %  %  %  %  %  %  %  %  %  %  %  %  %  %        k__Archaea; p__Crenarchaeota    3918    0.0%    0.0%    0.0%    0.0%    0.0%    0.0%    0.0%    0.0%    0.0%    0.0%    0.0%    0.0%    0.0%    0.0%    0.0%    0.0%    0.0%    0.0%    0.0%    0.0%    0.0%    0.0%    0.0%    0.0%    0.0%    0.0%    0.0%    0.0%    0.0%    0.0%    0.0%    0.0%    0.0%    0.0%    0.0%    0.0%    0.0%    0.0%    0.0%    0.1%    0.0%    0.0%    0.0%    0.0%    0.0%    0.0%    0.0%    0.0%    0.0%    0.0%    0.0%    0.0%    0.0%    0.0%    0.1%    0.0%    0.0%    0.0%    0.0%    0.1%    0.0%    0.0%    0.0%    0.1%    0.0%    0.1%    0.0%    0.1%    0.0%    0.1%    0.0%    0.0%    0.0%    0.0%    0.0%    0.0%    0.0%    0.0%    0.0%    0.0%    0.0%    0.0%    0.0%    0.0%    0.0%    0.0%    0.0%    0.0%    0.0%    0.0%    0.0%    0.0%    0.0%    0.0%    0.0%    0.0%    0.1%    0.0%    0.0%    0.0%    0.1%    0.0%    0.0%    0.0%    0.0%    0.0%    0.1%    0.0%    0.0%    0.0%    0.0%    0.1%    0.0%    0.0%    0.0%    0.0%    0.0%    0.0%    0.0%    0.2%    0.0%    0.0%    0.1%    0.1%    0.0%    0.1%    0.1%    0.0%    0.1%    0.2%    0.1%    0.0%    0.0%    0.0%    0.2%    0.0%    0.0%    0.0%    0.0%    0.0%    0.0%    0.0%    0.0%    0.0%    0.1%    0.0%    0.0%    0.0%    0.0%    0.0%    0.0%    0.0%    0.1%    0.0%    0.0%    0.0%    0.1%    0.0%    0.0%    0.1%    0.3%    0.1%    0.1%    0.0%    0.0%    0.0%    0.1%    0.0%    0.0%    0.0%    0.0%    0.1%    0.0%    0.0%    0.0%    0.0%    0.0%    0.0%    0.0%    0.0%    0.0%    0.0%    0.0%    0.0%    0.0%    0.0%    0.0%    0.0%    0.0%    0.0%    0.0%    0.0%    0.0%    0.0%    0.0%    0.0%    0.0%    0.0%    0.0%    0.0%    0.0%    0.0%    0.0%    0.1%    0.1%    0.0%    0.0%    0.0%    0.1%    0.1%    0.0%    0.0%    0.0%    0.0%    0.0%    0.0%    0.0%    0.0%    0.0%    0.0%    0.0%    0.0%    0.0%    0.0%    0.0%    0.0%    0.0%    0.0%    0.0%    0.0%    0.0%    0.0%    0.1%    0.0%    0.1%    0.0%    0.1%    0.0%    0.0%    0.0%    0.0%    0.0%    0.0%    0.0%    0.0%    0.0%    0.0%    0.0%    0.0%    0.0%    0.1%    0.1%    0.0%    0.0%    0.0%    0.0%    0.0%    0.0%    0.0%    0.0%    0.1%    0.0%    0.0%    0.0%    0.0%    0.0%    0.0%    0.0%    0.0%    0.0%    0.0%    0.0%    0.0%    0.0%    0.0%    0.0%    0.0%    0.0%    0.0%    0.0%    0.0%    0.0%    0.0%    0.0%    0.0%    0.0%    0.0%    0.1%    0.0%    0.0%    0.0%    0.0%    0.0%    0.1%    0.1%    0.0%    0.0%    0.0%    0.0%    0.0%    0.0%    0.0%    0.0%    0.0%    0.0%    0.0%    0.0%    0.0%    0.0%    0.0%    0.0%    0.0%    0.0%    0.0%    0.0%    0.0%    0.0%    0.0%    0.0%    0.0%    0.0%    0.0%    0.0%    0.0%    0.0%    0.0%    0.0%    0.0%    0.0%    0.0%    0.0%    0.0%    0.0%    0.0%    0.0%    0.0%    0.0%    0.0%    0.0%    0.0%    0.0%    0.0%    0.0%    0.0%    0.0%    0.0%    0.0%    0.0%    0.0%    0.0%    0.0%    0.0%    0.0%    0.0%    0.0%    0.0%    0.0%    0.0%        k__Archaea; p__Euryarchaeota     204    0.0%    0.0%    0.0%    0.0%    0.0%    0.0%    0.0%    0.0%    0.0%    0.0%    0.0%    0.0%    0.0%    0.0%    0.0%    0.0%    0.0%    0.0%    0.0%    0.0%    0.0%    0.0%    0.0%    0.0%    0.0%    0.0%    0.0%    0.0%    0.0%    0.0%    0.0%    0.0%    0.0%    0.0%    0.0%    0.0%    0.0%    0.0%    0.0%    0.0%    0.0%    0.0%    0.0%    0.0%    0.0%    0.0%    0.0%    0.0%    0.0%    0.0%    0.0%    0.0%    0.0%    0.0%    0.0%    0.0%    0.0%    0.0%    0.0%    0.0%    0.0%    0.0%    0.0%    0.0%    0.0%    0.0%    0.0%    0.0%    0.0%    0.0%    0.0%    0.0%    0.0%    0.0%    0.0%    0.0%    0.0%    0.0%    0.0%    0.0%    0.0%    0.0%    0.0%    0.0%    0.1%    0.0%    0.0%    0.0%    0.0%    0.0%    0.0%    0.0%    0.0%    0.0%    0.0%    0.0%    0.0%    0.0%    0.0%    0.0%    0.0%    0.0%    0.0%    0.0%    0.0%    0.0%    0.0%    0.0%    0.0%    0.0%    0.0%    0.0%    0.0%    0.0%    0.0%    0.0%    0.0%    0.0%    0.0%    0.0%    0.0%    0.0%    0.0%    0.0%    0.0%    0.0%    0.0%    0.0%    0.0%    0.0%    0.0%    0.0%    0.0%    0.0%    0.0%    0.0%    0.0%    0.0%    0.0%    0.0%    0.0%    0.0%    0.0%    0.0%    0.0%    0.0%    0.0%    0.0%    0.0%    0.0%    0.0%    0.0%    0.0%    0.0%    0.0%    0.0%    0.0%    0.0%    0.0%    0.0%    0.0%    0.0%    0.0%    0.0%    0.0%    0.0%    0.0%    0.0%    0.0%    0.0%    0.0%    0.0%    0.0%    0.0%    0.0%    0.0%    0.0%    0.0%    0.0%    0.0%    0.0%    0.0%    0.0%    0.0%    0.0%    0.0%    0.0%    0.0%    0.0%    0.0%    0.0%    0.0%    0.0%    0.0%    0.0%    0.0%    0.0%    0.0%    0.0%    0.0%    0.0%    0.0%    0.0%    0.0%    0.0%    0.0%    0.0%    0.0%    0.0%    0.0%    0.0%    0.0%    0.0%    0.0%    0.0%    0.0%    0.0%    0.0%    0.0%    0.0%    0.0%    0.0%    0.0%    0.0%    0.0%    0.0%    0.0%    0.0%    0.0%    0.0%    0.0%    0.0%    0.0%    0.0%    0.0%    0.0%    0.0%    0.0%    0.0%    0.0%    0.0%    0.0%    0.0%    0.0%    0.0%    0.0%    0.0%    0.0%    0.0%    0.0%    0.0%    0.0%    0.0%    0.0%    0.0%    0.0%    0.0%    0.0%    0.0%    0.0%    0.0%    0.0%    0.0%    0.0%    0.0%    0.0%    0.0%    0.0%    0.0%    0.0%    0.0%    0.0%    0.0%    0.0%    0.0%    0.0%    0.0%    0.0%    0.0%    0.0%    0.0%    0.0%    0.0%    0.0%    0.0%    0.0%    0.0%    0.0%    0.0%    0.0%    0.0%    0.0%    0.0%    0.0%    0.0%    0.0%    0.0%    0.0%    0.0%    0.0%    0.0%    0.0%    0.0%    0.0%    0.0%    0.0%    0.0%    0.0%    0.0%    0.0%    0.0%    0.2%    0.0%    0.0%    0.0%    0.0%    0.0%    0.0%    0.0%    0.0%    0.0%    0.0%    0.0%    0.0%    0.0%    0.0%    0.0%    0.0%    0.0%    0.0%    0.0%    0.0%    0.0%    0.0%    0.0%    0.0%    0.0%    0.0%    0.0%    0.0%    0.0%    0.0%    0.0%    0.0%    0.0%    0.0%    0.0%    0.0%    0.0%    0.0%    0.0%    0.0%    0.0%    0.0%    0.0%    0.0%    0.0%    0.0%        k__Bacteria; p__       6    0.0%    0.0%    0.0%    0.0%    0.0%    0.0%    0.0%    0.0%    0.0%    0.0%    0.0%    0.0%    0.0%    0.0%    0.0%    0.0%    0.0%    0.0%    0.0%    0.0%    0.0%    0.0%    0.0%    0.0%    0.0%    0.0%    0.0%    0.0%    0.0%    0.0%    0.0%    0.0%    0.0%    0.0%    0.0%    0.0%    0.0%    0.0%    0.0%    0.0%    0.0%    0.0%    0.0%    0.0%    0.0%    0.0%    0.0%    0.0%    0.0%    0.0%    0.0%    0.0%    0.0%    0.0%    0.0%    0.0%    0.0%    0.0%    0.0%    0.0%    0.0%    0.0%    0.0%    0.0%    0.0%    0.0%    0.0%    0.0%    0.0%    0.0%    0.0%    0.0%    0.0%    0.0%    0.0%    0.0%    0.0%    0.0%    0.0%    0.0%    0.0%    0.0%    0.0%    0.0%    0.0%    0.0%    0.0%    0.0%    0.0%    0.0%    0.0%    0.0%    0.0%    0.0%    0.0%    0.0%    0.0%    0.0%    0.0%    0.0%    0.0%    0.0%    0.0%    0.0%    0.0%    0.0%    0.0%    0.0%    0.0%    0.0%    0.0%    0.0%    0.0%    0.0%    0.0%    0.0%    0.0%    0.0%    0.0%    0.0%    0.0%    0.0%    0.0%    0.0%    0.0%    0.0%    0.0%    0.0%    0.0%    0.0%    0.0%    0.0%    0.0%    0.0%    0.0%    0.0%    0.0%    0.0%    0.0%    0.0%    0.0%    0.0%    0.0%    0.0%    0.0%    0.0%    0.0%    0.0%    0.0%    0.0%    0.0%    0.0%    0.0%    0.0%    0.0%    0.0%    0.0%    0.0%    0.0%    0.0%    0.0%    0.0%    0.0%    0.0%    0.0%    0.0%    0.0%    0.0%    0.0%    0.0%    0.0%    0.0%    0.0%    0.0%    0.0%    0.0%    0.0%    0.0%    0.0%    0.0%    0.0%    0.0%    0.0%    0.0%    0.0%    0.0%    0.0%    0.0%    0.0%    0.0%    0.0%    0.0%    0.0%    0.0%    0.0%    0.0%    0.0%    0.0%    0.0%    0.0%    0.0%    0.0%    0.0%    0.0%    0.0%    0.0%    0.0%    0.0%    0.0%    0.0%    0.0%    0.0%    0.0%    0.0%    0.0%    0.0%    0.0%    0.0%    0.0%    0.0%    0.0%    0.0%    0.0%    0.0%    0.0%    0.0%    0.0%    0.0%    0.0%    0.0%    0.0%    0.0%    0.0%    0.0%    0.0%    0.0%    0.0%    0.0%    0.0%    0.0%    0.0%    0.0%    0.0%    0.0%    0.0%    0.0%    0.0%    0.0%    0.0%    0.0%    0.0%    0.0%    0.0%    0.0%    0.0%    0.0%    0.0%    0.0%    0.0%    0.0%    0.0%    0.0%    0.0%    0.0%    0.0%    0.0%    0.0%    0.0%    0.0%    0.0%    0.0%    0.0%    0.0%    0.0%    0.0%    0.0%    0.0%    0.0%    0.0%    0.0%    0.0%    0.0%    0.0%    0.0%    0.0%    0.0%    0.0%    0.0%    0.0%    0.0%    0.0%    0.0%    0.0%    0.0%    0.0%    0.0%    0.0%    0.0%    0.0%    0.0%    0.0%    0.0%    0.0%    0.0%    0.0%    0.0%    0.0%    0.0%    0.0%    0.0%    0.0%    0.0%    0.0%    0.0%    0.0%    0.0%    0.0%    0.0%    0.0%    0.0%    0.0%    0.0%    0.0%    0.0%    0.0%    0.0%    0.0%    0.0%    0.0%    0.0%    0.0%    0.0%    0.0%    0.0%    0.0%    0.0%    0.0%    0.0%    0.0%    0.0%    0.0%    0.0%    0.0%    0.0%    0.0%    0.0%    0.0%    0.0%    0.0%    0.0%    0.0%    0.0%    0.0%    0.0%    0.0%    0.0%    0.0%    0.0%        k__Bacteria; p__ABY1_OD1       0    0.0%    0.0%    0.0%    0.0%    0.0%    0.0%    0.0%    0.0%    0.0%    0.0%    0.0%    0.0%    0.0%    0.0%    0.0%    0.0%    0.0%    0.0%    0.0%    0.0%    0.0%    0.0%    0.0%    0.0%    0.0%    0.0%    0.0%    0.0%    0.0%    0.0%    0.0%    0.0%    0.0%    0.0%    0.0%    0.0%    0.0%    0.0%    0.0%    0.0%    0.0%    0.0%    0.0%    0.0%    0.0%    0.0%    0.0%    0.0%    0.0%    0.0%    0.0%    0.0%    0.0%    0.0%    0.0%    0.0%    0.0%    0.0%    0.0%    0.0%    0.0%    0.0%    0.0%    0.0%    0.0%    0.0%    0.0%    0.0%    0.0%    0.0%    0.0%    0.0%    0.0%    0.0%    0.0%    0.0%    0.0%    0.0%    0.0%    0.0%    0.0%    0.0%    0.0%    0.0%    0.0%    0.0%    0.0%    0.0%    0.0%    0.0%    0.0%    0.0%    0.0%    0.0%    0.0%    0.0%    0.0%    0.0%    0.0%    0.0%    0.0%    0.0%    0.0%    0.0%    0.0%    0.0%    0.0%    0.0%    0.0%    0.0%    0.0%    0.0%    0.0%    0.0%    0.0%    0.0%    0.0%    0.0%    0.0%    0.0%    0.0%    0.0%    0.0%    0.0%    0.0%    0.0%    0.0%    0.0%    0.0%    0.0%    0.0%    0.0%    0.0%    0.0%    0.0%    0.0%    0.0%    0.0%    0.0%    0.0%    0.0%    0.0%    0.0%    0.0%    0.0%    0.0%    0.0%    0.0%    0.0%    0.0%    0.0%    0.0%    0.0%    0.0%    0.0%    0.0%    0.0%    0.0%    0.0%    0.0%    0.0%    0.0%    0.0%    0.0%    0.0%    0.0%    0.0%    0.0%    0.0%    0.0%    0.0%    0.0%    0.0%    0.0%    0.0%    0.0%    0.0%    0.0%    0.0%    0.0%    0.0%    0.0%    0.0%    0.0%    0.0%    0.0%    0.0%    0.0%    0.0%    0.0%    0.0%    0.0%    0.0%    0.0%    0.0%    0.0%    0.0%    0.0%    0.0%    0.0%    0.0%    0.0%    0.0%    0.0%    0.0%    0.0%    0.0%    0.0%    0.0%    0.0%    0.0%    0.0%    0.0%    0.0%    0.0%    0.0%    0.0%    0.0%    0.0%    0.0%    0.0%    0.0%    0.0%    0.0%    0.0%    0.0%    0.0%    0.0%    0.0%    0.0%    0.0%    0.0%    0.0%    0.0%    0.0%    0.0%    0.0%    0.0%    0.0%    0.0%    0.0%    0.0%    0.0%    0.0%    0.0%    0.0%    0.0%    0.0%    0.0%    0.0%    0.0%    0.0%    0.0%    0.0%    0.0%    0.0%    0.0%    0.0%    0.0%    0.0%    0.0%    0.0%    0.0%    0.0%    0.0%    0.0%    0.0%    0.0%    0.0%    0.0%    0.0%    0.0%    0.0%    0.0%    0.0%    0.0%    0.0%    0.0%    0.0%    0.0%    0.0%    0.0%    0.0%    0.0%    0.0%    0.0%    0.0%    0.0%    0.0%    0.0%    0.0%    0.0%    0.0%    0.0%    0.0%    0.0%    0.0%    0.0%    0.0%    0.0%    0.0%    0.0%    0.0%    0.0%    0.0%    0.0%    0.0%    0.0%    0.0%    0.0%    0.0%    0.0%    0.0%    0.0%    0.0%    0.0%    0.0%    0.0%    0.0%    0.0%    0.0%    0.0%    0.0%    0.0%    0.0%    0.0%    0.0%    0.0%    0.0%    0.0%    0.0%    0.0%    0.0%    0.0%    0.0%    0.0%    0.0%    0.0%    0.0%    0.0%    0.0%    0.0%    0.0%    0.0%    0.0%    0.0%    0.0%    0.0%    0.0%    0.0%    0.0%    0.0%    0.0%    0.0%    0.0%    0.0%    0.0%    0.0%        k__Bacteria; p__AD3      27    0.0%    0.0%    0.0%    0.0%    0.0%    0.0%    0.0%    0.0%    0.0%    0.0%    0.0%    0.0%    0.0%    0.0%    0.0%    0.0%    0.0%    0.0%    0.0%    0.0%    0.0%    0.0%    0.0%    0.0%    0.0%    0.0%    0.0%    0.0%    0.0%    0.0%    0.0%    0.0%    0.0%    0.0%    0.0%    0.0%    0.0%    0.0%    0.0%    0.0%    0.0%    0.0%    0.0%    0.0%    0.0%    0.0%    0.0%    0.0%    0.0%    0.0%    0.0%    0.0%    0.0%    0.0%    0.0%    0.0%    0.0%    0.0%    0.0%    0.0%    0.0%    0.0%    0.0%    0.0%    0.0%    0.0%    0.0%    0.0%    0.0%    0.0%    0.0%    0.0%    0.0%    0.0%    0.0%    0.0%    0.0%    0.0%    0.0%    0.0%    0.0%    0.0%    0.0%    0.0%    0.0%    0.0%    0.0%    0.0%    0.0%    0.0%    0.0%    0.0%    0.0%    0.0%    0.0%    0.0%    0.0%    0.0%    0.0%    0.0%    0.0%    0.0%    0.0%    0.0%    0.0%    0.0%    0.0%    0.0%    0.0%    0.0%    0.0%    0.0%    0.0%    0.0%    0.0%    0.0%    0.0%    0.0%    0.0%    0.0%    0.0%    0.0%    0.0%    0.0%    0.0%    0.0%    0.0%    0.0%    0.0%    0.0%    0.0%    0.0%    0.0%    0.0%    0.0%    0.0%    0.0%    0.0%    0.0%    0.0%    0.0%    0.0%    0.0%    0.0%    0.0%    0.0%    0.0%    0.0%    0.0%    0.0%    0.0%    0.0%    0.0%    0.0%    0.0%    0.0%    0.0%    0.0%    0.0%    0.0%    0.0%    0.0%    0.0%    0.0%    0.0%    0.0%    0.0%    0.0%    0.0%    0.0%    0.0%    0.0%    0.0%    0.0%    0.0%    0.0%    0.0%    0.0%    0.0%    0.0%    0.0%    0.0%    0.0%    0.0%    0.0%    0.0%    0.0%    0.0%    0.0%    0.0%    0.0%    0.0%    0.0%    0.0%    0.0%    0.0%    0.0%    0.0%    0.0%    0.0%    0.0%    0.0%    0.0%    0.0%    0.0%    0.0%    0.0%    0.0%    0.0%    0.0%    0.0%    0.0%    0.0%    0.0%    0.0%    0.0%    0.0%    0.0%    0.0%    0.0%    0.0%    0.0%    0.0%    0.0%    0.0%    0.0%    0.0%    0.0%    0.0%    0.0%    0.0%    0.0%    0.0%    0.0%    0.0%    0.0%    0.0%    0.0%    0.0%    0.0%    0.0%    0.0%    0.0%    0.0%    0.0%    0.0%    0.0%    0.0%    0.0%    0.0%    0.0%    0.0%    0.0%    0.0%    0.0%    0.0%    0.0%    0.0%    0.0%    0.0%    0.0%    0.0%    0.0%    0.0%    0.0%    0.0%    0.0%    0.0%    0.0%    0.0%    0.0%    0.0%    0.0%    0.0%    0.0%    0.0%    0.0%    0.0%    0.0%    0.0%    0.0%    0.0%    0.0%    0.0%    0.0%    0.0%    0.0%    0.0%    0.0%    0.0%    0.0%    0.0%    0.0%    0.0%    0.0%    0.0%    0.0%    0.0%    0.0%    0.0%    0.0%    0.0%    0.0%    0.0%    0.0%    0.0%    0.0%    0.0%    0.0%    0.0%    0.0%    0.0%    0.0%    0.0%    0.0%    0.0%    0.0%    0.0%    0.0%    0.0%    0.0%    0.0%    0.0%    0.0%    0.0%    0.0%    0.0%    0.0%    0.0%    0.0%    0.0%    0.0%    0.0%    0.0%    0.0%    0.0%    0.0%    0.0%    0.0%    0.0%    0.0%    0.0%    0.0%    0.0%    0.0%    0.0%    0.0%    0.0%    0.0%    0.0%    0.0%    0.0%    0.0%    0.0%    0.0%    0.0%    0.0%    0.0%        k__Bacteria; p__Acidobacteria   28483    0.2%    0.0%    0.1%    0.0%    0.1%    0.0%    0.0%    0.0%    0.0%    0.0%    0.0%    0.0%    0.1%    0.1%    0.0%    0.1%    0.1%    0.2%    0.1%    0.1%    0.2%    0.1%    0.0%    0.0%    0.0%    0.0%    0.1%    0.1%    0.2%    0.0%    0.0%    0.2%    0.2%    0.0%    0.2%    0.0%    0.2%    0.0%    0.1%    0.3%    0.1%    0.2%    0.0%    0.0%    0.2%    0.0%    0.0%    0.0%    0.0%    0.0%    0.1%    0.1%    0.1%    0.0%    0.3%    0.1%    0.1%    0.0%    0.2%    0.5%    0.2%    0.2%    0.1%    0.6%    0.5%    0.2%    0.0%    0.6%    0.2%    0.3%    0.1%    0.1%    0.1%    0.2%    0.4%    0.0%    0.1%    0.2%    0.1%    0.2%    0.1%    0.1%    0.1%    0.1%    0.0%    0.0%    0.4%    0.0%    0.0%    0.4%    0.0%    0.2%    0.2%    0.1%    0.1%    0.2%    0.5%    0.0%    0.2%    0.2%    0.4%    0.2%    0.0%    0.1%    0.0%    0.0%    0.7%    0.0%    0.0%    0.3%    0.1%    0.4%    0.0%    0.0%    0.2%    0.1%    0.1%    0.1%    0.1%    1.1%    0.0%    0.1%    0.6%    0.2%    0.0%    0.5%    0.6%    0.1%    0.4%    1.0%    0.4%    0.2%    0.3%    0.1%    0.9%    0.1%    0.1%    0.2%    0.0%    0.1%    0.0%    0.1%    0.2%    0.1%    0.4%    0.2%    0.0%    0.0%    0.1%    0.0%    0.1%    0.1%    0.3%    0.1%    0.0%    0.0%    0.3%    0.1%    0.1%    0.5%    1.1%    0.5%    0.5%    0.2%    0.1%    0.1%    0.4%    0.1%    0.1%    0.1%    0.4%    0.3%    0.3%    0.2%    0.0%    0.0%    0.0%    0.0%    0.1%    0.1%    0.0%    0.0%    0.1%    0.1%    0.1%    0.1%    0.1%    0.0%    0.0%    0.1%    0.0%    0.1%    0.1%    0.1%    0.1%    0.1%    0.3%    0.0%    0.1%    0.0%    0.1%    0.1%    0.1%    0.5%    0.3%    0.1%    0.1%    0.2%    0.7%    0.3%    0.3%    0.0%    0.8%    0.1%    0.1%    0.3%    0.0%    0.0%    0.3%    0.1%    0.1%    1.5%    0.0%    0.3%    0.0%    0.2%    0.0%    0.1%    0.3%    0.2%    0.0%    0.0%    0.5%    0.1%    0.5%    0.0%    0.6%    0.1%    0.0%    0.5%    0.1%    0.4%    0.0%    0.0%    0.1%    0.1%    0.2%    0.1%    0.2%    0.1%    0.5%    0.2%    0.1%    0.0%    0.0%    0.0%    0.1%    0.0%    0.1%    0.1%    0.4%    0.3%    0.2%    0.2%    0.2%    0.1%    0.2%    0.2%    0.1%    0.1%    0.1%    0.3%    0.9%    0.3%    0.1%    0.2%    0.0%    0.1%    0.0%    0.0%    0.1%    0.0%    0.1%    0.0%    0.1%    0.0%    0.1%    1.5%    0.0%    1.2%    0.1%    0.0%    0.3%    0.2%    0.3%    0.0%    0.2%    0.3%    0.2%    0.3%    0.1%    0.1%    0.0%    0.3%    0.1%    0.0%    0.1%    0.1%    0.1%    0.1%    0.0%    0.1%    0.1%    0.0%    0.2%    0.3%    0.0%    0.0%    0.0%    0.0%    0.0%    0.1%    0.0%    0.1%    0.1%    0.2%    0.1%    0.0%    0.1%    0.2%    0.0%    0.0%    0.0%    0.2%    0.0%    0.0%    0.0%    0.1%    0.1%    0.1%    0.0%    0.0%    0.0%    0.2%    0.3%    0.0%    0.0%    0.1%    0.0%    0.0%    0.1%    0.1%    0.1%    0.4%    0.1%    0.0%    0.0%    0.0%        k__Bacteria; p__Actinobacteria   3078987   18.1%   16.6%   21.6%   25.0%   28.3%    4.5%   28.4%   12.6%   21.3%   18.0%    9.7%    9.9%   16.4%   19.0%   18.7%   16.3%   25.7%   11.9%   12.9%   25.4%   13.8%   19.7%   15.1%   47.5%   40.4%   20.9%   11.7%   17.8%   20.5%   55.9%   44.1%   17.5%    7.7%   17.3%   16.3%   23.7%   27.9%   20.2%   10.9%   11.1%   12.9%   15.6%   19.3%   23.4%   28.1%   17.4%   15.1%   48.0%   19.8%   45.6%   14.5%    8.4%   11.8%   25.0%   11.3%   51.2%   21.2%    4.6%   12.8%   16.7%   12.3%   12.6%   48.1%   13.2%    8.5%   21.9%    2.9%   20.0%    6.6%   16.7%    7.5%   48.5%    4.2%   21.0%   21.3%    2.2%    7.3%   15.0%   25.9%   23.7%   24.2%    9.4%    9.8%   12.8%   47.0%   35.5%   21.5%    4.2%   12.4%   24.0%    1.6%   11.8%   16.2%    5.6%   64.9%   27.8%   10.0%    1.4%   18.9%   22.9%   27.7%   11.5%   15.3%   33.5%   40.0%   19.2%   16.0%    4.7%    1.4%   28.7%   22.1%   16.1%   22.0%    3.1%   14.9%   19.6%   15.9%   24.6%    9.4%   25.4%    0.6%   16.6%   21.2%   11.5%    2.6%   15.5%   22.7%    4.2%   12.8%   19.2%   19.2%    7.4%    7.9%   14.7%   19.5%    4.9%    9.2%   23.2%    1.6%    6.7%    0.6%    9.1%    8.2%    3.9%   10.1%   31.0%   16.8%   13.2%   13.8%    2.1%    5.3%    2.8%   11.7%   10.2%    6.8%    3.0%   15.2%    6.6%    3.2%   22.6%   16.5%   28.4%   14.0%    3.8%   17.8%   10.1%   27.3%   27.0%   23.9%   21.4%   17.0%   22.3%    7.9%   12.7%   12.6%   25.7%   20.1%    3.4%    4.8%   11.0%   27.3%   23.4%    4.9%    6.9%   50.7%   26.0%   32.9%   21.3%   29.5%   26.6%   37.5%   33.4%   35.2%   23.3%   11.8%   12.5%   20.4%   23.5%   23.7%   25.9%   19.1%   10.5%   14.3%   14.8%   26.1%    8.1%    5.7%   10.3%   18.5%   26.7%   17.3%   13.4%    5.6%   17.3%    5.5%   23.0%   10.2%   10.9%   31.1%   36.4%   22.8%   10.9%    0.7%    7.4%   12.2%   19.4%   19.9%   14.6%   15.1%   27.3%   10.3%   14.5%   11.6%   19.0%   15.3%    9.4%   16.8%   14.3%    4.3%   28.2%   18.3%   17.9%   25.8%   37.7%   11.7%   14.5%   15.4%   25.3%   23.4%   33.6%   21.6%   14.9%    8.0%   11.4%    5.0%   14.3%   14.0%    8.2%   15.8%   12.2%   20.4%   12.4%   17.0%   10.9%   21.1%    4.3%   22.4%   16.5%    7.7%   16.4%   37.0%   29.7%   14.5%   24.1%   13.2%   25.1%   19.6%   23.9%   23.7%   18.5%    9.0%    2.1%   12.5%   30.8%   19.2%   16.4%   25.1%   26.0%   25.0%   13.7%   29.1%   51.9%   35.7%   23.7%   15.7%   12.8%   23.6%   33.7%   28.9%   21.4%   39.6%   16.5%    9.1%   28.6%   24.6%   22.5%   23.7%   33.6%   14.0%   13.7%   40.5%   27.6%   40.0%   15.6%   21.8%   25.8%    7.5%   31.1%   24.3%   59.0%   28.3%   21.7%   20.0%   10.8%   22.2%   35.3%   32.8%   23.4%   20.9%   22.0%   15.6%   25.6%   17.8%    8.2%   18.1%   33.7%    9.4%   20.3%   17.2%   13.3%   26.5%   12.9%   25.9%   32.4%   20.8%   24.4%   34.9%   25.4%   24.9%   51.9%   17.8%   19.8%   21.8%   24.7%   26.7%    0.3%    8.6%    9.4%        k__Bacteria; p__Aquificae      31    0.0%    0.0%    0.0%    0.0%    0.0%    0.0%    0.0%    0.0%    0.0%    0.0%    0.0%    0.0%    0.0%    0.0%    0.0%    0.0%    0.0%    0.0%    0.0%    0.0%    0.0%    0.0%    0.0%    0.0%    0.0%    0.0%    0.0%    0.0%    0.0%    0.0%    0.0%    0.0%    0.0%    0.0%    0.0%    0.0%    0.0%    0.0%    0.0%    0.0%    0.0%    0.0%    0.0%    0.0%    0.0%    0.0%    0.0%    0.0%    0.0%    0.0%    0.0%    0.0%    0.0%    0.0%    0.0%    0.0%    0.0%    0.0%    0.0%    0.0%    0.0%    0.0%    0.0%    0.0%    0.0%    0.0%    0.0%    0.0%    0.0%    0.0%    0.0%    0.0%    0.0%    0.0%    0.0%    0.0%    0.0%    0.0%    0.0%    0.0%    0.0%    0.0%    0.0%    0.0%    0.0%    0.0%    0.0%    0.0%    0.0%    0.0%    0.0%    0.0%    0.0%    0.0%    0.0%    0.0%    0.0%    0.0%    0.0%    0.0%    0.0%    0.0%    0.0%    0.0%    0.0%    0.0%    0.0%    0.0%    0.0%    0.0%    0.0%    0.0%    0.0%    0.0%    0.0%    0.0%    0.0%    0.0%    0.0%    0.0%    0.0%    0.0%    0.0%    0.0%    0.0%    0.0%    0.0%    0.0%    0.0%    0.0%    0.0%    0.0%    0.0%    0.0%    0.0%    0.0%    0.0%    0.0%    0.0%    0.0%    0.0%    0.0%    0.0%    0.0%    0.0%    0.0%    0.0%    0.0%    0.0%    0.0%    0.0%    0.0%    0.0%    0.0%    0.0%    0.0%    0.0%    0.0%    0.0%    0.0%    0.0%    0.0%    0.0%    0.0%    0.0%    0.0%    0.0%    0.0%    0.0%    0.0%    0.0%    0.0%    0.0%    0.0%    0.0%    0.0%    0.0%    0.0%    0.0%    0.0%    0.0%    0.0%    0.0%    0.0%    0.0%    0.0%    0.0%    0.0%    0.0%    0.0%    0.0%    0.0%    0.0%    0.0%    0.0%    0.0%    0.0%    0.0%    0.0%    0.0%    0.0%    0.0%    0.0%    0.0%    0.0%    0.0%    0.0%    0.0%    0.0%    0.0%    0.0%    0.0%    0.0%    0.0%    0.0%    0.0%    0.0%    0.0%    0.0%    0.0%    0.0%    0.0%    0.0%    0.0%    0.0%    0.0%    0.0%    0.0%    0.0%    0.0%    0.0%    0.0%    0.0%    0.0%    0.0%    0.0%    0.0%    0.0%    0.0%    0.0%    0.0%    0.0%    0.0%    0.0%    0.0%    0.0%    0.0%    0.0%    0.0%    0.0%    0.0%    0.0%    0.0%    0.0%    0.0%    0.0%    0.0%    0.0%    0.0%    0.0%    0.0%    0.0%    0.0%    0.0%    0.0%    0.0%    0.0%    0.0%    0.0%    0.0%    0.0%    0.0%    0.0%    0.0%    0.0%    0.0%    0.0%    0.0%    0.0%    0.0%    0.0%    0.0%    0.0%    0.0%    0.0%    0.0%    0.0%    0.0%    0.0%    0.0%    0.0%    0.0%    0.0%    0.0%    0.0%    0.0%    0.0%    0.0%    0.0%    0.0%    0.0%    0.0%    0.0%    0.0%    0.0%    0.0%    0.0%    0.0%    0.0%    0.0%    0.0%    0.0%    0.0%    0.0%    0.0%    0.0%    0.0%    0.0%    0.0%    0.0%    0.0%    0.0%    0.0%    0.0%    0.0%    0.0%    0.0%    0.0%    0.0%    0.0%    0.0%    0.0%    0.0%    0.0%    0.0%    0.0%    0.0%    0.0%    0.0%    0.0%    0.0%    0.0%    0.0%    0.0%    0.0%    0.0%    0.0%    0.0%    0.0%    0.0%    0.0%    0.0%    0.0%    0.0%    0.0%    0.0%    0.0%    0.0%        k__Bacteria; p__BRC1     265    0.0%    0.0%    0.0%    0.0%    0.0%    0.0%    0.0%    0.0%    0.0%    0.0%    0.0%    0.0%    0.0%    0.0%    0.0%    0.0%    0.0%    0.0%    0.0%    0.0%    0.0%    0.0%    0.0%    0.0%    0.0%    0.0%    0.0%    0.0%    0.0%    0.0%    0.0%    0.0%    0.0%    0.0%    0.0%    0.0%    0.0%    0.0%    0.0%    0.0%    0.0%    0.0%    0.0%    0.0%    0.0%    0.0%    0.0%    0.0%    0.0%    0.0%    0.0%    0.0%    0.0%    0.0%    0.0%    0.0%    0.0%    0.0%    0.0%    0.0%    0.0%    0.0%    0.0%    0.0%    0.0%    0.0%    0.0%    0.0%    0.0%    0.0%    0.0%    0.0%    0.0%    0.0%    0.0%    0.0%    0.0%    0.0%    0.0%    0.0%    0.0%    0.0%    0.0%    0.0%    0.0%    0.0%    0.0%    0.0%    0.0%    0.0%    0.0%    0.0%    0.0%    0.0%    0.0%    0.0%    0.0%    0.0%    0.0%    0.0%    0.0%    0.0%    0.0%    0.0%    0.0%    0.0%    0.0%    0.0%    0.0%    0.0%    0.0%    0.0%    0.0%    0.0%    0.0%    0.0%    0.0%    0.0%    0.0%    0.0%    0.0%    0.0%    0.0%    0.0%    0.0%    0.0%    0.0%    0.0%    0.0%    0.0%    0.0%    0.0%    0.0%    0.0%    0.0%    0.0%    0.0%    0.0%    0.0%    0.0%    0.0%    0.0%    0.0%    0.0%    0.0%    0.0%    0.0%    0.0%    0.0%    0.0%    0.0%    0.0%    0.0%    0.0%    0.0%    0.0%    0.0%    0.0%    0.0%    0.0%    0.0%    0.0%    0.0%    0.0%    0.0%    0.0%    0.0%    0.0%    0.0%    0.0%    0.0%    0.0%    0.0%    0.0%    0.0%    0.0%    0.0%    0.0%    0.0%    0.0%    0.0%    0.0%    0.0%    0.0%    0.0%    0.0%    0.0%    0.0%    0.0%    0.0%    0.0%    0.0%    0.0%    0.0%    0.0%    0.0%    0.0%    0.0%    0.0%    0.0%    0.0%    0.0%    0.0%    0.0%    0.0%    0.0%    0.0%    0.0%    0.0%    0.0%    0.0%    0.0%    0.0%    0.0%    0.0%    0.0%    0.0%    0.0%    0.0%    0.0%    0.0%    0.0%    0.0%    0.0%    0.0%    0.0%    0.0%    0.0%    0.0%    0.0%    0.0%    0.0%    0.0%    0.0%    0.0%    0.0%    0.0%    0.0%    0.0%    0.0%    0.0%    0.0%    0.0%    0.0%    0.0%    0.0%    0.0%    0.0%    0.0%    0.0%    0.0%    0.0%    0.0%    0.0%    0.0%    0.0%    0.0%    0.0%    0.0%    0.0%    0.0%    0.0%    0.0%    0.0%    0.0%    0.0%    0.0%    0.0%    0.0%    0.0%    0.0%    0.0%    0.0%    0.0%    0.0%    0.0%    0.0%    0.0%    0.0%    0.0%    0.0%    0.0%    0.0%    0.0%    0.0%    0.0%    0.0%    0.0%    0.0%    0.0%    0.0%    0.0%    0.0%    0.0%    0.0%    0.0%    0.0%    0.0%    0.0%    0.0%    0.0%    0.0%    0.0%    0.0%    0.0%    0.0%    0.0%    0.0%    0.0%    0.0%    0.0%    0.0%    0.0%    0.0%    0.0%    0.0%    0.0%    0.0%    0.0%    0.0%    0.0%    0.0%    0.0%    0.0%    0.0%    0.0%    0.0%    0.0%    0.0%    0.0%    0.0%    0.0%    0.0%    0.0%    0.0%    0.0%    0.0%    0.0%    0.0%    0.0%    0.0%    0.0%    0.0%    0.0%    0.0%    0.0%    0.0%    0.0%    0.0%    0.0%    0.0%    0.0%    0.0%    0.0%    0.0%    0.0%    0.0%    0.0%        k__Bacteria; p__Bacteroidetes   1019534    6.0%    7.7%    6.0%    8.0%    4.7%    3.2%    5.2%    1.3%    4.9%    8.2%    4.3%    1.8%    2.8%    8.4%    5.9%    3.4%    7.3%    3.8%    1.5%    3.2%    4.9%    8.3%    2.2%    9.5%    4.9%    9.3%    1.8%    4.4%    8.3%    3.4%    4.5%    7.5%    2.2%    8.0%    8.7%   14.9%    6.1%   13.9%    4.4%    3.5%    2.7%    6.1%    6.0%    5.9%    4.6%    3.2%    2.4%    1.2%    3.9%    2.5%    3.2%    3.8%    6.2%    3.1%    2.7%    3.2%    2.9%    0.8%    4.0%    3.5%    2.5%    2.3%    2.1%    4.7%   15.0%    1.7%    3.5%    6.1%    2.8%    2.8%    9.8%    3.3%    0.8%    6.1%    4.8%    0.4%    2.0%    6.0%    4.7%    7.4%   13.3%    3.4%    3.8%    5.7%    2.8%    3.6%    6.1%    2.0%    3.8%    4.1%    0.8%    3.7%    5.3%    2.0%    3.2%    2.4%    5.2%    1.8%   11.2%    8.6%    4.7%   12.2%   10.7%    2.7%    2.1%    4.1%   15.6%    3.3%    5.6%    4.3%    3.9%   15.9%    3.6%    0.7%    7.4%    3.9%    8.1%    2.3%    5.2%   15.7%    9.8%    4.1%    5.2%    7.1%    0.7%   11.1%    6.2%    1.2%    3.9%    6.7%    5.0%    6.8%    4.6%    4.5%   10.0%   10.8%    3.2%    6.2%    2.8%    8.1%    2.3%    9.7%    8.9%    8.8%    5.0%    8.6%   12.0%   12.0%    4.2%    3.7%    8.6%   11.4%    7.1%   15.9%    4.3%   11.2%    8.5%    3.0%    1.5%    6.3%    3.4%    5.2%   10.2%    2.1%   13.7%    2.0%    4.9%    7.9%    4.4%    5.6%   12.6%    6.9%    1.8%    8.6%    8.3%    4.1%    3.0%    2.2%    2.5%    2.4%    2.3%    3.8%   10.1%    2.8%    3.4%    3.5%    1.8%    2.3%    2.6%    3.4%    4.2%    3.7%    4.3%   11.6%    3.3%   11.7%    8.1%    5.9%    2.3%    2.3%   10.5%   16.6%    2.6%    6.0%    7.2%    8.8%    2.1%    9.6%    8.0%    6.4%    5.2%   15.0%   13.0%   10.9%    5.4%    4.9%   11.3%    4.6%    6.7%    5.2%    4.2%   11.9%    3.9%   12.2%   12.4%    5.6%    4.4%   11.7%    4.5%    7.5%   12.8%    8.7%    7.0%    8.8%    6.6%    2.1%    5.3%    9.1%    4.7%    1.8%    2.6%    1.5%    3.8%    2.7%    7.2%    2.7%    1.4%    4.5%    2.5%    7.4%   13.6%    6.4%    6.2%    6.2%    4.6%    8.6%    6.5%   19.3%    6.6%   10.5%    9.3%    9.6%    8.2%   10.4%   12.3%    2.2%    5.3%   10.7%    7.4%   16.4%    7.7%    6.1%   10.7%    8.0%   18.3%    9.4%    4.0%    4.2%    4.9%   12.6%    1.8%    2.4%    8.9%    2.2%   11.6%    9.2%    5.5%    8.9%    6.2%    3.5%    4.6%    1.8%    6.0%    6.8%    3.5%    1.9%    8.6%    5.9%    4.5%    5.8%    7.5%    9.9%   13.9%    6.1%    4.3%    8.7%    8.9%    3.5%    1.8%    6.6%    0.6%    2.0%    0.6%    7.2%    6.5%    5.5%    0.6%    2.7%    2.1%    3.3%   11.3%    6.1%   26.1%    1.5%    5.4%    6.9%    5.4%    6.5%    4.7%    6.4%    4.3%    2.7%    4.8%    2.4%    2.5%    1.2%    5.9%    8.9%    6.2%   15.8%    3.1%   11.1%    2.8%    2.5%    4.4%    3.9%    3.6%    7.3%    3.8%    1.6%    2.8%    5.9%    2.9%    3.5%    1.1%    1.9%   26.5%    1.6%        k__Bacteria; p__CCM11b      40    0.0%    0.0%    0.0%    0.0%    0.0%    0.0%    0.0%    0.0%    0.0%    0.0%    0.0%    0.0%    0.0%    0.0%    0.0%    0.0%    0.0%    0.0%    0.0%    0.0%    0.0%    0.0%    0.0%    0.0%    0.0%    0.0%    0.0%    0.0%    0.0%    0.0%    0.0%    0.0%    0.0%    0.0%    0.0%    0.0%    0.0%    0.0%    0.0%    0.0%    0.0%    0.0%    0.0%    0.0%    0.0%    0.0%    0.0%    0.0%    0.0%    0.0%    0.0%    0.0%    0.0%    0.0%    0.0%    0.0%    0.0%    0.0%    0.0%    0.0%    0.0%    0.0%    0.0%    0.0%    0.0%    0.0%    0.0%    0.0%    0.0%    0.0%    0.0%    0.0%    0.0%    0.0%    0.0%    0.0%    0.0%    0.0%    0.0%    0.0%    0.0%    0.0%    0.0%    0.0%    0.0%    0.0%    0.0%    0.0%    0.0%    0.0%    0.0%    0.0%    0.0%    0.0%    0.0%    0.0%    0.0%    0.0%    0.0%    0.0%    0.0%    0.0%    0.0%    0.0%    0.0%    0.0%    0.0%    0.0%    0.0%    0.0%    0.0%    0.0%    0.0%    0.0%    0.0%    0.0%    0.0%    0.0%    0.0%    0.0%    0.0%    0.0%    0.0%    0.0%    0.0%    0.0%    0.0%    0.0%    0.0%    0.0%    0.0%    0.0%    0.0%    0.0%    0.0%    0.0%    0.0%    0.0%    0.0%    0.0%    0.0%    0.0%    0.0%    0.0%    0.0%    0.0%    0.0%    0.0%    0.0%    0.0%    0.0%    0.0%    0.0%    0.0%    0.0%    0.0%    0.0%    0.0%    0.0%    0.0%    0.0%    0.0%    0.0%    0.0%    0.0%    0.0%    0.0%    0.0%    0.0%    0.0%    0.0%    0.0%    0.0%    0.0%    0.0%    0.0%    0.0%    0.0%    0.0%    0.0%    0.0%    0.0%    0.0%    0.0%    0.0%    0.0%    0.0%    0.0%    0.0%    0.0%    0.0%    0.0%    0.0%    0.0%    0.0%    0.0%    0.0%    0.0%    0.0%    0.0%    0.0%    0.0%    0.0%    0.0%    0.0%    0.0%    0.0%    0.0%    0.0%    0.0%    0.0%    0.0%    0.0%    0.0%    0.0%    0.0%    0.0%    0.0%    0.0%    0.0%    0.0%    0.0%    0.0%    0.0%    0.0%    0.0%    0.0%    0.0%    0.0%    0.0%    0.0%    0.0%    0.0%    0.0%    0.0%    0.0%    0.0%    0.0%    0.0%    0.0%    0.0%    0.0%    0.0%    0.0%    0.0%    0.0%    0.0%    0.0%    0.0%    0.0%    0.0%    0.0%    0.0%    0.0%    0.0%    0.0%    0.0%    0.0%    0.0%    0.0%    0.0%    0.0%    0.0%    0.0%    0.0%    0.0%    0.0%    0.0%    0.0%    0.0%    0.0%    0.0%    0.0%    0.0%    0.0%    0.0%    0.0%    0.0%    0.0%    0.0%    0.0%    0.0%    0.0%    0.0%    0.0%    0.0%    0.0%    0.0%    0.0%    0.0%    0.0%    0.0%    0.0%    0.0%    0.0%    0.0%    0.0%    0.0%    0.0%    0.0%    0.0%    0.0%    0.0%    0.0%    0.0%    0.0%    0.0%    0.0%    0.0%    0.0%    0.0%    0.0%    0.0%    0.0%    0.0%    0.0%    0.0%    0.0%    0.0%    0.0%    0.0%    0.0%    0.0%    0.0%    0.0%    0.0%    0.0%    0.0%    0.0%    0.0%    0.0%    0.0%    0.0%    0.0%    0.0%    0.0%    0.0%    0.0%    0.0%    0.0%    0.0%    0.0%    0.0%    0.0%    0.0%    0.0%    0.0%    0.0%    0.0%    0.0%    0.0%    0.0%    0.0%    0.0%    0.0%    0.0%    0.0%    0.0%        k__Bacteria; p__Caldithrix_KSB1       1    0.0%    0.0%    0.0%    0.0%    0.0%    0.0%    0.0%    0.0%    0.0%    0.0%    0.0%    0.0%    0.0%    0.0%    0.0%    0.0%    0.0%    0.0%    0.0%    0.0%    0.0%    0.0%    0.0%    0.0%    0.0%    0.0%    0.0%    0.0%    0.0%    0.0%    0.0%    0.0%    0.0%    0.0%    0.0%    0.0%    0.0%    0.0%    0.0%    0.0%    0.0%    0.0%    0.0%    0.0%    0.0%    0.0%    0.0%    0.0%    0.0%    0.0%    0.0%    0.0%    0.0%    0.0%    0.0%    0.0%    0.0%    0.0%    0.0%    0.0%    0.0%    0.0%    0.0%    0.0%    0.0%    0.0%    0.0%    0.0%    0.0%    0.0%    0.0%    0.0%    0.0%    0.0%    0.0%    0.0%    0.0%    0.0%    0.0%    0.0%    0.0%    0.0%    0.0%    0.0%    0.0%    0.0%    0.0%    0.0%    0.0%    0.0%    0.0%    0.0%    0.0%    0.0%    0.0%    0.0%    0.0%    0.0%    0.0%    0.0%    0.0%    0.0%    0.0%    0.0%    0.0%    0.0%    0.0%    0.0%    0.0%    0.0%    0.0%    0.0%    0.0%    0.0%    0.0%    0.0%    0.0%    0.0%    0.0%    0.0%    0.0%    0.0%    0.0%    0.0%    0.0%    0.0%    0.0%    0.0%    0.0%    0.0%    0.0%    0.0%    0.0%    0.0%    0.0%    0.0%    0.0%    0.0%    0.0%    0.0%    0.0%    0.0%    0.0%    0.0%    0.0%    0.0%    0.0%    0.0%    0.0%    0.0%    0.0%    0.0%    0.0%    0.0%    0.0%    0.0%    0.0%    0.0%    0.0%    0.0%    0.0%    0.0%    0.0%    0.0%    0.0%    0.0%    0.0%    0.0%    0.0%    0.0%    0.0%    0.0%    0.0%    0.0%    0.0%    0.0%    0.0%    0.0%    0.0%    0.0%    0.0%    0.0%    0.0%    0.0%    0.0%    0.0%    0.0%    0.0%    0.0%    0.0%    0.0%    0.0%    0.0%    0.0%    0.0%    0.0%    0.0%    0.0%    0.0%    0.0%    0.0%    0.0%    0.0%    0.0%    0.0%    0.0%    0.0%    0.0%    0.0%    0.0%    0.0%    0.0%    0.0%    0.0%    0.0%    0.0%    0.0%    0.0%    0.0%    0.0%    0.0%    0.0%    0.0%    0.0%    0.0%    0.0%    0.0%    0.0%    0.0%    0.0%    0.0%    0.0%    0.0%    0.0%    0.0%    0.0%    0.0%    0.0%    0.0%    0.0%    0.0%    0.0%    0.0%    0.0%    0.0%    0.0%    0.0%    0.0%    0.0%    0.0%    0.0%    0.0%    0.0%    0.0%    0.0%    0.0%    0.0%    0.0%    0.0%    0.0%    0.0%    0.0%    0.0%    0.0%    0.0%    0.0%    0.0%    0.0%    0.0%    0.0%    0.0%    0.0%    0.0%    0.0%    0.0%    0.0%    0.0%    0.0%    0.0%    0.0%    0.0%    0.0%    0.0%    0.0%    0.0%    0.0%    0.0%    0.0%    0.0%    0.0%    0.0%    0.0%    0.0%    0.0%    0.0%    0.0%    0.0%    0.0%    0.0%    0.0%    0.0%    0.0%    0.0%    0.0%    0.0%    0.0%    0.0%    0.0%    0.0%    0.0%    0.0%    0.0%    0.0%    0.0%    0.0%    0.0%    0.0%    0.0%    0.0%    0.0%    0.0%    0.0%    0.0%    0.0%    0.0%    0.0%    0.0%    0.0%    0.0%    0.0%    0.0%    0.0%    0.0%    0.0%    0.0%    0.0%    0.0%    0.0%    0.0%    0.0%    0.0%    0.0%    0.0%    0.0%    0.0%    0.0%    0.0%    0.0%    0.0%    0.0%    0.0%    0.0%    0.0%    0.0%    0.0%    0.0%    0.0%    0.0%        k__Bacteria; p__Chlamydiae      25    0.0%    0.0%    0.0%    0.0%    0.0%    0.0%    0.0%    0.0%    0.0%    0.0%    0.0%    0.0%    0.0%    0.0%    0.0%    0.0%    0.0%    0.0%    0.0%    0.0%    0.0%    0.0%    0.0%    0.0%    0.0%    0.0%    0.0%    0.0%    0.0%    0.0%    0.0%    0.0%    0.0%    0.0%    0.0%    0.0%    0.0%    0.0%    0.0%    0.0%    0.0%    0.0%    0.0%    0.0%    0.0%    0.0%    0.0%    0.0%    0.0%    0.0%    0.0%    0.0%    0.0%    0.0%    0.0%    0.0%    0.0%    0.0%    0.0%    0.0%    0.0%    0.0%    0.0%    0.0%    0.0%    0.0%    0.0%    0.0%    0.0%    0.0%    0.0%    0.0%    0.0%    0.0%    0.0%    0.0%    0.0%    0.0%    0.0%    0.0%    0.0%    0.0%    0.0%    0.0%    0.0%    0.0%    0.0%    0.0%    0.0%    0.0%    0.0%    0.0%    0.0%    0.0%    0.0%    0.0%    0.0%    0.0%    0.0%    0.0%    0.0%    0.0%    0.0%    0.0%    0.0%    0.0%    0.0%    0.0%    0.0%    0.0%    0.0%    0.0%    0.0%    0.0%    0.0%    0.0%    0.0%    0.0%    0.0%    0.0%    0.0%    0.0%    0.0%    0.0%    0.0%    0.0%    0.0%    0.0%    0.0%    0.0%    0.0%    0.0%    0.0%    0.0%    0.0%    0.0%    0.0%    0.0%    0.0%    0.0%    0.0%    0.0%    0.0%    0.0%    0.0%    0.0%    0.0%    0.0%    0.0%    0.0%    0.0%    0.0%    0.0%    0.0%    0.0%    0.0%    0.0%    0.0%    0.0%    0.0%    0.0%    0.0%    0.0%    0.0%    0.0%    0.0%    0.0%    0.0%    0.0%    0.0%    0.0%    0.0%    0.0%    0.0%    0.0%    0.0%    0.0%    0.0%    0.0%    0.0%    0.0%    0.0%    0.0%    0.0%    0.0%    0.0%    0.0%    0.0%    0.0%    0.0%    0.0%    0.0%    0.0%    0.0%    0.0%    0.0%    0.0%    0.0%    0.0%    0.0%    0.0%    0.0%    0.0%    0.0%    0.0%    0.0%    0.0%    0.0%    0.0%    0.0%    0.0%    0.0%    0.0%    0.0%    0.0%    0.0%    0.0%    0.0%    0.0%    0.0%    0.0%    0.0%    0.0%    0.0%    0.0%    0.0%    0.0%    0.0%    0.0%    0.0%    0.0%    0.0%    0.0%    0.0%    0.0%    0.0%    0.0%    0.0%    0.0%    0.0%    0.0%    0.0%    0.0%    0.0%    0.0%    0.0%    0.0%    0.0%    0.0%    0.0%    0.0%    0.0%    0.0%    0.0%    0.0%    0.0%    0.0%    0.0%    0.0%    0.0%    0.0%    0.0%    0.0%    0.0%    0.0%    0.0%    0.0%    0.0%    0.0%    0.0%    0.0%    0.0%    0.0%    0.0%    0.0%    0.0%    0.0%    0.0%    0.0%    0.0%    0.0%    0.0%    0.0%    0.0%    0.0%    0.0%    0.0%    0.0%    0.0%    0.0%    0.0%    0.0%    0.0%    0.0%    0.0%    0.0%    0.0%    0.0%    0.0%    0.0%    0.0%    0.0%    0.0%    0.0%    0.0%    0.0%    0.0%    0.0%    0.0%    0.0%    0.0%    0.0%    0.0%    0.0%    0.0%    0.0%    0.0%    0.0%    0.0%    0.0%    0.0%    0.0%    0.0%    0.0%    0.0%    0.0%    0.0%    0.0%    0.0%    0.0%    0.0%    0.0%    0.0%    0.0%    0.0%    0.0%    0.0%    0.0%    0.0%    0.0%    0.0%    0.0%    0.0%    0.0%    0.0%    0.0%    0.0%    0.0%    0.0%    0.0%    0.0%    0.0%    0.0%    0.0%    0.0%    0.0%    0.0%    0.0%        k__Bacteria; p__Chlorobi      36    0.0%    0.0%    0.0%    0.0%    0.0%    0.0%    0.0%    0.0%    0.0%    0.0%    0.0%    0.0%    0.0%    0.0%    0.0%    0.0%    0.0%    0.0%    0.0%    0.0%    0.0%    0.0%    0.0%    0.0%    0.0%    0.0%    0.0%    0.0%    0.0%    0.0%    0.0%    0.0%    0.0%    0.0%    0.0%    0.0%    0.0%    0.0%    0.0%    0.0%    0.0%    0.0%    0.0%    0.0%    0.0%    0.0%    0.0%    0.0%    0.0%    0.0%    0.0%    0.0%    0.0%    0.0%    0.0%    0.0%    0.0%    0.0%    0.0%    0.0%    0.0%    0.0%    0.0%    0.0%    0.0%    0.0%    0.0%    0.0%    0.0%    0.0%    0.0%    0.0%    0.0%    0.0%    0.0%    0.0%    0.0%    0.0%    0.0%    0.0%    0.0%    0.0%    0.0%    0.0%    0.0%    0.0%    0.0%    0.0%    0.0%    0.0%    0.0%    0.0%    0.0%    0.0%    0.0%    0.0%    0.0%    0.0%    0.0%    0.0%    0.0%    0.0%    0.0%    0.0%    0.0%    0.0%    0.0%    0.0%    0.0%    0.0%    0.0%    0.0%    0.0%    0.0%    0.0%    0.0%    0.0%    0.0%    0.0%    0.0%    0.0%    0.0%    0.0%    0.0%    0.0%    0.0%    0.0%    0.0%    0.0%    0.0%    0.0%    0.0%    0.0%    0.0%    0.0%    0.0%    0.0%    0.0%    0.0%    0.0%    0.0%    0.0%    0.0%    0.0%    0.0%    0.0%    0.0%    0.0%    0.0%    0.0%    0.0%    0.0%    0.0%    0.0%    0.0%    0.0%    0.0%    0.0%    0.0%    0.0%    0.0%    0.0%    0.0%    0.0%    0.0%    0.0%    0.0%    0.0%    0.0%    0.0%    0.0%    0.0%    0.0%    0.0%    0.0%    0.0%    0.0%    0.0%    0.0%    0.0%    0.0%    0.0%    0.0%    0.0%    0.0%    0.0%    0.0%    0.0%    0.0%    0.0%    0.0%    0.0%    0.0%    0.0%    0.0%    0.0%    0.0%    0.0%    0.0%    0.0%    0.0%    0.0%    0.0%    0.0%    0.0%    0.0%    0.0%    0.0%    0.0%    0.0%    0.0%    0.0%    0.0%    0.0%    0.0%    0.0%    0.0%    0.0%    0.0%    0.0%    0.0%    0.0%    0.0%    0.0%    0.0%    0.0%    0.0%    0.0%    0.0%    0.0%    0.0%    0.0%    0.0%    0.0%    0.0%    0.0%    0.0%    0.0%    0.0%    0.0%    0.0%    0.0%    0.0%    0.0%    0.0%    0.0%    0.0%    0.0%    0.0%    0.0%    0.0%    0.0%    0.0%    0.0%    0.0%    0.0%    0.0%    0.0%    0.0%    0.0%    0.0%    0.0%    0.0%    0.0%    0.0%    0.0%    0.0%    0.0%    0.0%    0.0%    0.0%    0.0%    0.0%    0.0%    0.0%    0.0%    0.0%    0.0%    0.0%    0.0%    0.0%    0.0%    0.0%    0.0%    0.0%    0.0%    0.0%    0.0%    0.0%    0.0%    0.0%    0.0%    0.0%    0.0%    0.0%    0.0%    0.0%    0.0%    0.0%    0.0%    0.0%    0.0%    0.0%    0.0%    0.0%    0.0%    0.0%    0.0%    0.0%    0.0%    0.0%    0.0%    0.0%    0.0%    0.0%    0.0%    0.0%    0.0%    0.0%    0.0%    0.0%    0.0%    0.0%    0.0%    0.0%    0.0%    0.0%    0.0%    0.0%    0.0%    0.0%    0.0%    0.0%    0.0%    0.0%    0.0%    0.0%    0.0%    0.0%    0.0%    0.0%    0.0%    0.0%    0.0%    0.0%    0.0%    0.0%    0.0%    0.0%    0.0%    0.0%    0.0%    0.0%    0.0%    0.0%    0.0%    0.0%    0.0%        k__Bacteria; p__Chloroflexi   19429    0.1%    0.0%    0.2%    0.0%    0.1%    0.0%    0.0%    0.0%    0.0%    0.0%    0.0%    0.0%    0.1%    0.1%    0.0%    0.0%    0.1%    0.2%    0.0%    0.1%    0.1%    0.1%    0.1%    0.0%    0.0%    0.0%    0.1%    0.0%    0.1%    0.0%    0.0%    0.1%    0.0%    0.0%    0.2%    0.0%    0.1%    0.0%    0.1%    0.3%    0.1%    0.2%    0.0%    0.0%    0.1%    0.0%    0.0%    0.0%    0.0%    0.0%    0.1%    0.0%    0.0%    0.0%    0.2%    0.1%    0.1%    0.0%    0.1%    0.2%    0.3%    0.1%    0.0%    0.3%    0.2%    0.1%    0.0%    0.4%    0.1%    0.2%    0.1%    0.0%    0.1%    0.1%    0.4%    0.0%    0.1%    0.2%    0.0%    0.1%    0.1%    0.2%    0.0%    0.1%    0.1%    0.0%    0.3%    0.0%    0.0%    0.3%    0.0%    0.1%    0.2%    0.1%    0.1%    0.2%    0.3%    0.0%    0.1%    0.1%    0.2%    0.1%    0.0%    0.1%    0.0%    0.0%    0.4%    0.0%    0.0%    0.2%    0.2%    0.3%    0.0%    0.0%    0.2%    0.1%    0.0%    0.1%    0.1%    0.9%    0.0%    0.2%    0.5%    0.2%    0.0%    0.5%    0.2%    0.0%    0.3%    0.8%    0.3%    0.1%    0.2%    0.1%    0.8%    0.1%    0.2%    0.2%    0.0%    0.1%    0.0%    0.2%    0.2%    0.1%    0.4%    0.1%    0.0%    0.0%    0.1%    0.0%    0.1%    0.1%    0.4%    0.1%    0.0%    0.0%    0.3%    0.1%    0.1%    0.4%    0.7%    0.4%    0.3%    0.1%    0.1%    0.1%    0.2%    0.1%    0.1%    0.1%    0.4%    0.3%    0.2%    0.1%    0.0%    0.0%    0.1%    0.0%    0.1%    0.1%    0.0%    0.0%    0.0%    0.1%    0.1%    0.1%    0.1%    0.0%    0.0%    0.0%    0.0%    0.0%    0.0%    0.0%    0.1%    0.1%    0.3%    0.0%    0.0%    0.0%    0.0%    0.1%    0.1%    0.3%    0.3%    0.1%    0.1%    0.1%    0.5%    0.2%    0.1%    0.0%    0.2%    0.1%    0.1%    0.1%    0.0%    0.1%    0.0%    0.0%    0.2%    0.2%    0.0%    0.2%    0.0%    0.1%    0.0%    0.1%    0.2%    0.1%    0.0%    0.0%    0.3%    0.0%    0.4%    0.0%    0.4%    0.0%    0.0%    0.0%    0.0%    0.1%    0.0%    0.0%    0.0%    0.0%    0.0%    0.0%    0.0%    0.1%    0.4%    0.1%    0.1%    0.0%    0.0%    0.0%    0.0%    0.1%    0.1%    0.0%    0.2%    0.2%    0.1%    0.1%    0.1%    0.0%    0.1%    0.1%    0.1%    0.1%    0.1%    0.2%    0.3%    0.2%    0.1%    0.1%    0.0%    0.1%    0.0%    0.0%    0.0%    0.0%    0.0%    0.0%    0.1%    0.0%    0.1%    1.5%    0.1%    0.0%    0.1%    0.0%    0.2%    0.1%    0.1%    0.0%    0.1%    0.1%    0.1%    0.3%    0.1%    0.0%    0.1%    0.1%    0.1%    0.0%    0.1%    0.0%    0.1%    0.0%    0.0%    0.0%    0.0%    0.0%    0.1%    0.3%    0.0%    0.0%    0.0%    0.1%    0.1%    0.1%    0.0%    0.0%    0.0%    0.0%    0.1%    0.1%    0.0%    0.1%    0.1%    0.0%    0.0%    0.1%    0.0%    0.0%    0.0%    0.0%    0.0%    0.0%    0.0%    0.0%    0.0%    0.0%    0.4%    0.0%    0.0%    0.0%    0.0%    0.0%    0.0%    0.1%    0.0%    0.2%    0.0%    0.0%    0.5%    0.0%        k__Bacteria; p__Cyanobacteria   2262576   13.3%    0.1%    9.0%    0.9%    1.6%   44.5%    0.3%    0.1%    0.1%    0.0%    0.1%    0.1%   10.1%    6.0%    0.5%    2.1%    0.5%   25.9%   51.0%   19.8%   16.4%    8.1%    3.8%    0.2%    0.5%    1.3%   46.2%    1.7%    1.1%    0.4%    0.1%    8.9%   65.8%   25.7%    3.0%    0.9%    1.1%    3.6%    9.7%   32.8%   26.2%    7.5%    0.3%    0.4%    0.3%    0.6%   17.9%    6.6%    1.3%    0.7%    8.7%   26.9%    5.2%    8.4%   40.6%    1.5%   33.2%    7.3%    3.1%   15.9%   48.3%   15.9%    3.0%    7.7%   17.7%   16.5%   41.6%    3.0%   43.3%   24.8%   13.2%    3.2%   79.3%   21.6%    7.4%   87.5%   46.6%   15.9%    0.6%    1.4%    0.8%    4.5%    9.2%    1.9%    3.3%    0.1%    3.6%   58.6%   20.7%    1.9%   37.6%    6.7%    2.9%   45.5%    1.3%   19.5%   45.4%   65.1%    4.5%    1.8%   20.9%    2.4%    2.3%    3.9%    0.7%    0.9%    1.4%   52.8%   38.7%    1.8%    0.8%    2.3%   14.5%   80.9%    6.4%    2.3%    1.0%    1.1%    1.1%    5.1%    5.9%    2.7%    3.4%   13.4%    1.8%    2.7%    6.6%   44.5%   16.0%    5.8%   13.2%    7.7%   48.7%    4.0%    2.5%   19.9%    0.6%    1.3%    0.4%   36.3%    0.6%    3.1%   26.6%   17.2%   40.5%    3.1%    0.7%    0.4%   19.1%   44.1%   44.7%   50.5%   14.2%    5.6%   13.8%   36.3%    8.4%   51.8%   10.1%    4.5%   41.3%    4.6%    4.2%   44.5%    2.2%   38.9%    5.5%    5.8%    6.4%   12.2%    4.3%    5.8%   55.9%    7.2%    4.6%    0.9%    7.6%   71.3%   55.8%    4.2%    3.4%    6.0%    4.6%   64.9%    6.8%    8.4%    0.9%    2.8%    1.3%    2.0%    0.4%    4.0%    2.4%    2.3%   10.9%    9.7%    7.5%    6.0%   12.0%   20.8%    4.1%    3.7%   43.5%   10.3%    4.9%   18.5%    1.6%   21.8%   14.4%    4.5%   15.4%    1.8%   17.2%   11.6%   40.0%    4.6%    7.7%   44.2%    2.4%    3.6%    2.7%    2.7%   18.9%   14.9%   11.4%   10.0%    1.2%    1.1%   32.1%    4.0%    1.4%   31.3%    7.5%   18.1%   20.9%   39.5%   18.7%    2.0%    0.3%    1.7%    1.0%    0.9%    0.1%    6.8%    0.5%    0.7%   29.7%    0.7%    0.4%    2.1%    2.6%    8.3%   18.0%   41.5%   57.1%   36.8%   18.1%    1.6%   32.9%    1.2%    2.5%    1.6%   13.6%    5.1%    0.9%   56.6%   14.5%    0.4%    3.0%    0.6%    0.9%    9.3%    6.3%    4.3%    1.0%    4.0%   24.1%    1.2%    1.5%    0.5%   36.2%    3.2%   12.8%    3.7%    2.5%    0.1%    0.2%    1.2%    6.0%    2.1%    1.3%    1.2%    0.4%    0.6%    2.2%   10.2%    7.2%    1.1%    2.6%    2.0%    0.3%   10.4%    0.1%    2.2%    0.8%    4.1%    0.9%    4.2%   39.6%    0.3%    0.1%    0.8%    0.1%    0.8%    1.8%    0.7%   78.4%    2.0%    0.0%    0.2%    0.3%    0.2%    0.3%   60.3%   11.7%    1.5%    4.8%    9.7%    1.5%    2.7%    1.2%    0.2%    0.8%    6.6%    0.4%    0.8%    0.1%    1.2%    0.9%    1.0%    2.6%    1.2%    1.4%    2.0%    1.6%    0.1%    1.8%    0.3%    6.4%    8.8%   25.5%    1.2%    9.8%    3.7%   20.0%    8.6%    3.2%    0.4%        k__Bacteria; p__Deferribacteres     468    0.0%    0.0%    0.0%    0.0%    0.0%    0.0%    0.0%    0.0%    0.0%    0.0%    0.0%    0.0%    0.0%    0.0%    0.0%    0.0%    0.0%    0.0%    0.0%    0.0%    0.0%    0.0%    0.0%    0.0%    0.0%    0.0%    0.0%    0.0%    0.0%    0.0%    0.0%    0.0%    0.0%    0.0%    0.3%    0.0%    0.0%    0.0%    0.0%    0.0%    0.0%    0.0%    0.0%    0.0%    0.0%    0.0%    0.0%    0.0%    0.0%    0.0%    0.0%    0.0%    0.0%    0.0%    0.1%    0.0%    0.0%    0.0%    0.0%    0.0%    0.0%    0.0%    0.0%    0.0%    0.0%    0.0%    0.0%    0.0%    0.0%    0.0%    0.0%    0.0%    0.0%    0.0%    0.0%    0.0%    0.0%    0.0%    0.0%    0.0%    0.0%    0.0%    0.0%    0.0%    0.0%    0.0%    0.0%    0.0%    0.0%    0.0%    0.0%    0.0%    0.0%    0.0%    0.0%    0.0%    0.0%    0.0%    0.0%    0.0%    0.0%    0.0%    0.0%    0.0%    0.0%    0.0%    0.0%    0.0%    0.0%    0.0%    0.0%    0.0%    0.0%    0.0%    0.0%    0.0%    0.0%    0.0%    0.0%    0.0%    0.0%    0.0%    0.0%    0.0%    0.0%    0.0%    0.0%    0.0%    0.0%    0.0%    0.0%    0.0%    0.0%    0.0%    0.0%    0.0%    0.0%    0.0%    0.0%    0.0%    0.0%    0.0%    0.0%    0.0%    0.0%    0.0%    0.0%    0.0%    0.0%    0.0%    0.0%    0.0%    0.0%    0.0%    0.0%    0.0%    0.0%    0.0%    0.0%    0.0%    0.0%    0.0%    0.0%    0.0%    0.0%    0.0%    0.0%    0.0%    0.0%    0.0%    0.0%    0.0%    0.0%    0.0%    0.0%    0.0%    0.0%    0.0%    0.0%    0.0%    0.0%    0.0%    0.0%    0.0%    0.0%    0.0%    0.0%    0.0%    0.0%    0.0%    0.0%    0.0%    0.0%    0.0%    0.0%    0.0%    0.0%    0.0%    0.0%    0.0%    0.0%    0.1%    0.0%    0.0%    0.0%    0.0%    0.0%    0.0%    0.0%    0.0%    0.0%    0.0%    0.0%    0.0%    0.0%    0.0%    0.0%    0.0%    0.0%    0.0%    0.0%    0.0%    0.0%    0.0%    0.0%    0.0%    0.0%    0.0%    0.0%    0.0%    0.0%    0.0%    0.0%    0.0%    0.0%    0.0%    0.0%    0.0%    0.0%    0.0%    0.0%    0.0%    0.0%    0.0%    0.0%    0.0%    0.0%    0.0%    0.0%    0.0%    0.0%    0.0%    0.0%    0.0%    0.0%    0.0%    0.0%    0.0%    0.0%    0.0%    0.0%    0.0%    0.0%    0.0%    0.0%    0.0%    0.0%    0.0%    0.0%    0.0%    0.0%    0.0%    0.0%    0.0%    0.0%    0.0%    0.0%    0.0%    0.0%    0.0%    0.0%    0.0%    0.0%    0.0%    0.0%    0.0%    0.0%    0.0%    0.0%    0.0%    0.0%    0.0%    0.0%    0.0%    0.0%    0.0%    0.0%    0.0%    0.0%    0.0%    0.0%    0.0%    0.0%    0.0%    0.0%    0.0%    0.0%    0.0%    0.0%    0.0%    0.0%    0.0%    0.0%    0.0%    0.0%    0.0%    0.0%    0.0%    0.0%    0.0%    0.0%    0.0%    0.0%    0.0%    0.0%    0.0%    0.0%    0.0%    0.0%    0.0%    0.0%    0.0%    0.0%    0.0%    0.0%    0.0%    0.0%    0.0%    0.0%    0.0%    0.0%    0.0%    0.0%    0.0%    0.0%    0.0%    0.0%    0.0%    0.0%    0.0%    0.0%    0.0%    0.0%    0.0%    0.0%    0.0%    0.0%    0.0%        k__Bacteria; p__Elusimicrobia      37    0.0%    0.0%    0.0%    0.0%    0.0%    0.0%    0.0%    0.0%    0.0%    0.0%    0.0%    0.0%    0.0%    0.0%    0.0%    0.0%    0.0%    0.0%    0.0%    0.0%    0.0%    0.0%    0.0%    0.0%    0.0%    0.0%    0.0%    0.0%    0.0%    0.0%    0.0%    0.0%    0.0%    0.0%    0.0%    0.0%    0.0%    0.0%    0.0%    0.0%    0.0%    0.0%    0.0%    0.0%    0.0%    0.0%    0.0%    0.0%    0.0%    0.0%    0.0%    0.0%    0.0%    0.0%    0.0%    0.0%    0.0%    0.0%    0.0%    0.0%    0.0%    0.0%    0.0%    0.0%    0.0%    0.0%    0.0%    0.0%    0.0%    0.0%    0.0%    0.0%    0.0%    0.0%    0.0%    0.0%    0.0%    0.0%    0.0%    0.0%    0.0%    0.0%    0.0%    0.0%    0.0%    0.0%    0.0%    0.0%    0.0%    0.0%    0.0%    0.0%    0.0%    0.0%    0.0%    0.0%    0.0%    0.0%    0.0%    0.0%    0.0%    0.0%    0.0%    0.0%    0.0%    0.0%    0.0%    0.0%    0.0%    0.0%    0.0%    0.0%    0.0%    0.0%    0.0%    0.0%    0.0%    0.0%    0.0%    0.0%    0.0%    0.0%    0.0%    0.0%    0.0%    0.0%    0.0%    0.0%    0.0%    0.0%    0.0%    0.0%    0.0%    0.0%    0.0%    0.0%    0.0%    0.0%    0.0%    0.0%    0.0%    0.0%    0.0%    0.0%    0.0%    0.0%    0.0%    0.0%    0.0%    0.0%    0.0%    0.0%    0.0%    0.0%    0.0%    0.0%    0.0%    0.0%    0.0%    0.0%    0.0%    0.0%    0.0%    0.0%    0.0%    0.0%    0.0%    0.0%    0.0%    0.0%    0.0%    0.0%    0.0%    0.0%    0.0%    0.0%    0.0%    0.0%    0.0%    0.0%    0.0%    0.0%    0.0%    0.0%    0.0%    0.0%    0.0%    0.0%    0.0%    0.0%    0.0%    0.0%    0.0%    0.0%    0.0%    0.0%    0.0%    0.0%    0.0%    0.0%    0.0%    0.0%    0.0%    0.0%    0.0%    0.0%    0.0%    0.0%    0.0%    0.0%    0.0%    0.0%    0.0%    0.0%    0.0%    0.0%    0.0%    0.0%    0.0%    0.0%    0.0%    0.0%    0.0%    0.0%    0.0%    0.0%    0.0%    0.0%    0.0%    0.0%    0.0%    0.0%    0.0%    0.0%    0.0%    0.0%    0.0%    0.0%    0.0%    0.0%    0.0%    0.0%    0.0%    0.0%    0.0%    0.0%    0.0%    0.0%    0.0%    0.0%    0.0%    0.0%    0.0%    0.0%    0.0%    0.0%    0.0%    0.0%    0.0%    0.0%    0.0%    0.0%    0.0%    0.0%    0.0%    0.0%    0.0%    0.0%    0.0%    0.0%    0.0%    0.0%    0.0%    0.0%    0.0%    0.0%    0.0%    0.0%    0.0%    0.0%    0.0%    0.0%    0.0%    0.0%    0.0%    0.0%    0.0%    0.0%    0.0%    0.0%    0.0%    0.0%    0.0%    0.0%    0.0%    0.0%    0.0%    0.0%    0.0%    0.0%    0.0%    0.0%    0.0%    0.0%    0.0%    0.0%    0.0%    0.0%    0.0%    0.0%    0.0%    0.0%    0.0%    0.0%    0.0%    0.0%    0.0%    0.0%    0.0%    0.0%    0.0%    0.0%    0.0%    0.0%    0.0%    0.0%    0.0%    0.0%    0.0%    0.0%    0.0%    0.0%    0.0%    0.0%    0.0%    0.0%    0.0%    0.0%    0.0%    0.0%    0.0%    0.0%    0.0%    0.0%    0.0%    0.0%    0.0%    0.0%    0.0%    0.0%    0.0%    0.0%    0.0%    0.0%    0.0%    0.0%    0.0%    0.0%        k__Bacteria; p__Fibrobacteres       1    0.0%    0.0%    0.0%    0.0%    0.0%    0.0%    0.0%    0.0%    0.0%    0.0%    0.0%    0.0%    0.0%    0.0%    0.0%    0.0%    0.0%    0.0%    0.0%    0.0%    0.0%    0.0%    0.0%    0.0%    0.0%    0.0%    0.0%    0.0%    0.0%    0.0%    0.0%    0.0%    0.0%    0.0%    0.0%    0.0%    0.0%    0.0%    0.0%    0.0%    0.0%    0.0%    0.0%    0.0%    0.0%    0.0%    0.0%    0.0%    0.0%    0.0%    0.0%    0.0%    0.0%    0.0%    0.0%    0.0%    0.0%    0.0%    0.0%    0.0%    0.0%    0.0%    0.0%    0.0%    0.0%    0.0%    0.0%    0.0%    0.0%    0.0%    0.0%    0.0%    0.0%    0.0%    0.0%    0.0%    0.0%    0.0%    0.0%    0.0%    0.0%    0.0%    0.0%    0.0%    0.0%    0.0%    0.0%    0.0%    0.0%    0.0%    0.0%    0.0%    0.0%    0.0%    0.0%    0.0%    0.0%    0.0%    0.0%    0.0%    0.0%    0.0%    0.0%    0.0%    0.0%    0.0%    0.0%    0.0%    0.0%    0.0%    0.0%    0.0%    0.0%    0.0%    0.0%    0.0%    0.0%    0.0%    0.0%    0.0%    0.0%    0.0%    0.0%    0.0%    0.0%    0.0%    0.0%    0.0%    0.0%    0.0%    0.0%    0.0%    0.0%    0.0%    0.0%    0.0%    0.0%    0.0%    0.0%    0.0%    0.0%    0.0%    0.0%    0.0%    0.0%    0.0%    0.0%    0.0%    0.0%    0.0%    0.0%    0.0%    0.0%    0.0%    0.0%    0.0%    0.0%    0.0%    0.0%    0.0%    0.0%    0.0%    0.0%    0.0%    0.0%    0.0%    0.0%    0.0%    0.0%    0.0%    0.0%    0.0%    0.0%    0.0%    0.0%    0.0%    0.0%    0.0%    0.0%    0.0%    0.0%    0.0%    0.0%    0.0%    0.0%    0.0%    0.0%    0.0%    0.0%    0.0%    0.0%    0.0%    0.0%    0.0%    0.0%    0.0%    0.0%    0.0%    0.0%    0.0%    0.0%    0.0%    0.0%    0.0%    0.0%    0.0%    0.0%    0.0%    0.0%    0.0%    0.0%    0.0%    0.0%    0.0%    0.0%    0.0%    0.0%    0.0%    0.0%    0.0%    0.0%    0.0%    0.0%    0.0%    0.0%    0.0%    0.0%    0.0%    0.0%    0.0%    0.0%    0.0%    0.0%    0.0%    0.0%    0.0%    0.0%    0.0%    0.0%    0.0%    0.0%    0.0%    0.0%    0.0%    0.0%    0.0%    0.0%    0.0%    0.0%    0.0%    0.0%    0.0%    0.0%    0.0%    0.0%    0.0%    0.0%    0.0%    0.0%    0.0%    0.0%    0.0%    0.0%    0.0%    0.0%    0.0%    0.0%    0.0%    0.0%    0.0%    0.0%    0.0%    0.0%    0.0%    0.0%    0.0%    0.0%    0.0%    0.0%    0.0%    0.0%    0.0%    0.0%    0.0%    0.0%    0.0%    0.0%    0.0%    0.0%    0.0%    0.0%    0.0%    0.0%    0.0%    0.0%    0.0%    0.0%    0.0%    0.0%    0.0%    0.0%    0.0%    0.0%    0.0%    0.0%    0.0%    0.0%    0.0%    0.0%    0.0%    0.0%    0.0%    0.0%    0.0%    0.0%    0.0%    0.0%    0.0%    0.0%    0.0%    0.0%    0.0%    0.0%    0.0%    0.0%    0.0%    0.0%    0.0%    0.0%    0.0%    0.0%    0.0%    0.0%    0.0%    0.0%    0.0%    0.0%    0.0%    0.0%    0.0%    0.0%    0.0%    0.0%    0.0%    0.0%    0.0%    0.0%    0.0%    0.0%    0.0%    0.0%    0.0%    0.0%    0.0%    0.0%    0.0%    0.0%    0.0%        k__Bacteria; p__Firmicutes   5684461   33.4%   41.5%   35.4%   41.6%   26.4%    3.5%   47.5%   12.9%   59.2%   46.2%   53.3%   16.5%   24.7%   34.9%   46.2%   49.4%   44.8%   15.6%   23.9%   36.0%   31.3%   30.4%   59.2%   40.0%   39.5%   57.8%   18.0%   49.7%   38.4%   33.8%   46.3%   44.6%   17.6%   25.7%   36.5%   38.4%   34.3%   55.2%   31.2%   18.9%   23.6%   34.2%   40.5%   33.5%   38.2%   41.4%   36.3%   34.4%   40.4%   35.2%   41.9%   17.8%   26.7%   41.0%   25.6%   36.3%   26.9%   10.3%   43.4%   25.4%   25.5%   55.8%   33.8%   44.2%   29.9%   48.3%    6.9%   32.2%   12.3%   36.4%    6.1%   34.9%    7.0%   30.1%   46.0%    7.1%   20.2%   40.3%   61.4%   48.9%   49.1%   24.5%   52.6%   60.6%   39.1%   50.9%   42.4%    4.9%   33.2%   48.2%    4.5%   48.8%   52.8%   13.6%   24.7%   38.7%   13.6%    5.7%   45.6%   48.7%   18.3%   36.7%   39.5%   47.9%   51.1%   52.0%   27.3%    5.1%    2.1%   49.2%   59.7%   37.8%   27.0%   10.9%   44.9%   54.5%   34.6%   58.5%   36.2%   13.1%    1.9%   55.2%   44.9%   36.1%    7.3%   21.2%   30.2%    9.2%   19.1%   27.5%   41.4%   17.6%    4.0%   62.7%   27.1%   11.3%   21.5%   21.2%   13.9%   12.7%   18.9%   27.1%   13.9%    6.8%    9.0%   31.0%   38.5%   44.2%   43.1%    3.6%    7.3%    4.1%   25.2%   21.9%   59.8%    9.3%   26.7%   16.1%   23.7%   39.4%   14.2%   25.3%   11.3%    4.4%   21.5%   12.4%   33.7%   44.1%   34.4%   41.3%   25.0%   34.7%   13.6%   40.7%   45.8%   50.3%   52.4%   10.1%    6.9%   36.0%   56.0%   53.7%   45.5%   11.4%   32.3%   51.6%   55.7%   60.7%   51.8%   50.6%   50.7%   45.1%   45.4%   44.2%   49.6%   30.2%   43.1%   51.3%   53.5%   42.2%   34.0%   51.5%   21.4%   35.3%   33.3%   36.5%    9.8%   16.2%   24.7%   40.3%   41.8%   28.4%   15.6%   24.6%   24.8%   48.4%   17.9%   27.8%   35.1%   40.1%   58.3%   20.6%    1.3%   11.1%   17.1%   42.2%   55.5%   45.6%   26.8%   36.5%   45.9%   13.4%   11.4%   27.0%   25.2%   12.9%   28.1%   24.2%   71.1%   53.8%   55.7%   59.3%   50.9%   35.8%   48.3%   75.9%   37.9%   48.7%   33.6%   40.5%   18.6%   18.3%    6.4%   20.0%    4.5%   14.6%   21.6%    9.5%   18.7%   44.1%   24.4%   24.0%   31.3%    8.9%   26.4%    3.2%   26.8%   47.0%   44.3%   27.5%   31.6%   30.1%   12.3%   37.8%   23.1%   22.9%   34.4%   51.3%   35.1%   63.1%   32.9%   80.5%   33.8%   49.4%   25.6%   58.7%   22.8%   26.8%   50.4%   42.1%   45.9%   41.1%   34.9%   35.1%   62.2%   68.9%   42.2%   46.1%   47.4%   54.1%   43.9%   50.0%   62.5%   46.0%   52.5%   40.8%   55.0%   52.8%   20.9%   44.3%   46.3%   53.7%   22.9%   48.8%   53.3%   43.5%   10.0%   50.4%   60.0%   23.7%   36.7%   44.1%   48.2%   17.7%   34.5%   37.3%   40.9%   34.3%   50.5%   49.0%   63.4%   59.6%   66.5%   77.1%   75.5%   55.2%   48.9%   57.6%   54.8%    9.2%   50.2%   21.7%   56.3%   51.5%   47.1%   65.0%   51.2%   56.3%    8.0%   33.3%   30.8%   46.0%   57.9%   51.5%   46.7%    0.9%   37.8%   84.4%        k__Bacteria; p__Fusobacteria   292142    1.7%    9.1%   10.0%    8.4%    3.2%    0.1%    5.0%    1.7%    1.1%    3.9%    4.6%    0.2%    0.3%    7.3%    5.1%    1.3%    2.5%    0.9%    1.5%    0.5%    2.2%    1.4%    0.6%    0.1%    0.9%    1.1%    0.2%    2.7%    2.6%    0.7%    0.3%    4.0%    0.3%    8.1%    2.9%    7.5%    3.6%    1.0%    3.8%    1.4%    8.2%    5.2%    4.6%    4.3%   10.1%   19.1%    2.6%    0.9%    7.7%    4.2%    2.9%    0.8%    0.3%    1.8%    2.2%    2.0%    1.6%    0.7%    5.5%    1.2%    1.2%    1.7%    3.5%    6.5%    8.5%    2.2%    0.3%    4.3%    0.9%    3.6%    1.7%    1.1%    0.8%    2.7%    2.5%    0.2%    0.6%    3.4%    2.0%    4.5%    3.3%    0.5%    2.2%    2.9%    0.3%    0.7%    2.6%    0.1%    2.0%    1.7%    0.0%    0.6%    2.0%    0.6%    1.0%    0.7%    1.0%    0.1%    2.1%    2.3%    0.7%    0.4%    0.5%    0.8%    0.8%    3.8%    7.3%    0.1%    0.0%    1.2%    0.7%    4.0%    1.4%    0.1%    2.7%    0.8%    0.5%    0.5%    1.0%    1.0%    0.0%    0.7%    1.5%    1.5%    0.1%    1.0%    1.9%    0.1%    0.9%    0.7%    1.1%    0.3%    0.1%    0.8%    4.0%    0.2%    0.7%    0.5%    0.1%    0.7%    0.0%    1.7%    1.0%    0.2%    0.4%    0.5%    4.4%    1.1%    0.4%    0.1%    0.2%    0.4%    1.6%    0.4%    1.2%    0.3%    2.7%    0.2%    0.4%    1.9%    3.5%    2.0%    3.7%    0.8%    0.5%    0.9%    3.8%    0.8%    1.1%    1.7%    1.6%    2.9%    0.3%    1.4%    1.8%    0.6%    0.9%    0.8%    0.3%    1.0%    0.9%    0.9%    0.7%    0.3%    0.6%    0.8%    0.3%    1.2%    0.9%    1.1%    0.5%    1.5%    0.7%    0.4%    0.4%    0.5%    1.4%    1.7%    0.2%    0.4%    2.3%    6.6%    0.4%    1.4%    1.6%    1.3%    0.2%   14.4%    1.5%    1.0%    1.0%    0.6%    0.5%    9.6%    0.4%    0.5%    0.6%    1.5%    1.5%    0.6%    1.1%    0.5%    0.0%    0.4%    0.3%    0.8%    0.7%    2.8%    0.5%    2.8%    0.9%    0.2%    0.1%    0.8%    0.8%    0.1%    0.4%    0.4%    0.1%    0.2%    0.3%    0.4%    1.9%    1.2%    0.5%    0.1%    0.9%    0.0%    1.1%    2.2%    1.2%    2.9%    0.4%    0.5%    0.3%    0.2%    2.2%    0.4%    0.5%    2.8%    0.5%    0.6%    0.8%    0.2%    3.6%    0.0%    0.5%    1.4%    0.6%    0.2%    0.9%    1.1%    0.7%    1.5%    0.3%    0.5%    0.4%    0.9%    1.5%    0.2%    0.2%    0.1%    0.4%    0.1%    0.8%    1.8%    0.8%    1.1%    0.3%    0.0%    0.4%    0.3%    1.9%    1.8%    1.2%    0.2%    1.8%    1.1%    2.1%    0.8%    0.8%    0.8%    1.3%    0.9%    2.5%    1.0%    1.2%    0.5%    0.4%    5.3%    0.0%    0.3%    0.2%    3.6%    2.8%    0.6%    0.3%    2.0%    2.4%    0.6%    3.5%    4.2%    0.3%    0.3%    1.1%    1.3%    1.5%    0.9%    2.1%    1.5%    0.7%    1.5%    1.5%    0.3%    0.2%    0.8%    4.8%    1.5%    3.7%    0.1%    0.6%    0.1%    1.7%    0.5%    0.3%    0.7%    0.1%    1.7%    0.1%    0.1%    0.5%    8.7%    0.6%    0.4%    0.7%    0.1%    0.0%    1.7%        k__Bacteria; p__GN02       1    0.0%    0.0%    0.0%    0.0%    0.0%    0.0%    0.0%    0.0%    0.0%    0.0%    0.0%    0.0%    0.0%    0.0%    0.0%    0.0%    0.0%    0.0%    0.0%    0.0%    0.0%    0.0%    0.0%    0.0%    0.0%    0.0%    0.0%    0.0%    0.0%    0.0%    0.0%    0.0%    0.0%    0.0%    0.0%    0.0%    0.0%    0.0%    0.0%    0.0%    0.0%    0.0%    0.0%    0.0%    0.0%    0.0%    0.0%    0.0%    0.0%    0.0%    0.0%    0.0%    0.0%    0.0%    0.0%    0.0%    0.0%    0.0%    0.0%    0.0%    0.0%    0.0%    0.0%    0.0%    0.0%    0.0%    0.0%    0.0%    0.0%    0.0%    0.0%    0.0%    0.0%    0.0%    0.0%    0.0%    0.0%    0.0%    0.0%    0.0%    0.0%    0.0%    0.0%    0.0%    0.0%    0.0%    0.0%    0.0%    0.0%    0.0%    0.0%    0.0%    0.0%    0.0%    0.0%    0.0%    0.0%    0.0%    0.0%    0.0%    0.0%    0.0%    0.0%    0.0%    0.0%    0.0%    0.0%    0.0%    0.0%    0.0%    0.0%    0.0%    0.0%    0.0%    0.0%    0.0%    0.0%    0.0%    0.0%    0.0%    0.0%    0.0%    0.0%    0.0%    0.0%    0.0%    0.0%    0.0%    0.0%    0.0%    0.0%    0.0%    0.0%    0.0%    0.0%    0.0%    0.0%    0.0%    0.0%    0.0%    0.0%    0.0%    0.0%    0.0%    0.0%    0.0%    0.0%    0.0%    0.0%    0.0%    0.0%    0.0%    0.0%    0.0%    0.0%    0.0%    0.0%    0.0%    0.0%    0.0%    0.0%    0.0%    0.0%    0.0%    0.0%    0.0%    0.0%    0.0%    0.0%    0.0%    0.0%    0.0%    0.0%    0.0%    0.0%    0.0%    0.0%    0.0%    0.0%    0.0%    0.0%    0.0%    0.0%    0.0%    0.0%    0.0%    0.0%    0.0%    0.0%    0.0%    0.0%    0.0%    0.0%    0.0%    0.0%    0.0%    0.0%    0.0%    0.0%    0.0%    0.0%    0.0%    0.0%    0.0%    0.0%    0.0%    0.0%    0.0%    0.0%    0.0%    0.0%    0.0%    0.0%    0.0%    0.0%    0.0%    0.0%    0.0%    0.0%    0.0%    0.0%    0.0%    0.0%    0.0%    0.0%    0.0%    0.0%    0.0%    0.0%    0.0%    0.0%    0.0%    0.0%    0.0%    0.0%    0.0%    0.0%    0.0%    0.0%    0.0%    0.0%    0.0%    0.0%    0.0%    0.0%    0.0%    0.0%    0.0%    0.0%    0.0%    0.0%    0.0%    0.0%    0.0%    0.0%    0.0%    0.0%    0.0%    0.0%    0.0%    0.0%    0.0%    0.0%    0.0%    0.0%    0.0%    0.0%    0.0%    0.0%    0.0%    0.0%    0.0%    0.0%    0.0%    0.0%    0.0%    0.0%    0.0%    0.0%    0.0%    0.0%    0.0%    0.0%    0.0%    0.0%    0.0%    0.0%    0.0%    0.0%    0.0%    0.0%    0.0%    0.0%    0.0%    0.0%    0.0%    0.0%    0.0%    0.0%    0.0%    0.0%    0.0%    0.0%    0.0%    0.0%    0.0%    0.0%    0.0%    0.0%    0.0%    0.0%    0.0%    0.0%    0.0%    0.0%    0.0%    0.0%    0.0%    0.0%    0.0%    0.0%    0.0%    0.0%    0.0%    0.0%    0.0%    0.0%    0.0%    0.0%    0.0%    0.0%    0.0%    0.0%    0.0%    0.0%    0.0%    0.0%    0.0%    0.0%    0.0%    0.0%    0.0%    0.0%    0.0%    0.0%    0.0%    0.0%    0.0%    0.0%    0.0%    0.0%    0.0%    0.0%    0.0%    0.0%    0.0%    0.0%    0.0%        k__Bacteria; p__Gemmatimonadetes    4768    0.0%    0.0%    0.0%    0.0%    0.0%    0.0%    0.0%    0.0%    0.0%    0.0%    0.0%    0.0%    0.0%    0.0%    0.0%    0.0%    0.0%    0.0%    0.0%    0.0%    0.0%    0.0%    0.0%    0.0%    0.0%    0.0%    0.0%    0.0%    0.0%    0.0%    0.0%    0.0%    0.0%    0.0%    0.0%    0.0%    0.0%    0.0%    0.0%    0.1%    0.0%    0.0%    0.0%    0.0%    0.0%    0.0%    0.0%    0.0%    0.0%    0.0%    0.0%    0.0%    0.0%    0.0%    0.0%    0.0%    0.0%    0.0%    0.0%    0.0%    0.0%    0.0%    0.0%    0.0%    0.1%    0.0%    0.0%    0.1%    0.0%    0.1%    0.0%    0.0%    0.0%    0.1%    0.1%    0.0%    0.0%    0.0%    0.0%    0.0%    0.0%    0.0%    0.0%    0.0%    0.0%    0.0%    0.1%    0.0%    0.0%    0.1%    0.0%    0.0%    0.0%    0.0%    0.0%    0.0%    0.1%    0.0%    0.0%    0.1%    0.1%    0.0%    0.0%    0.0%    0.0%    0.0%    0.1%    0.0%    0.0%    0.1%    0.0%    0.1%    0.0%    0.0%    0.0%    0.0%    0.0%    0.0%    0.0%    0.2%    0.0%    0.0%    0.1%    0.1%    0.0%    0.3%    0.2%    0.0%    0.1%    0.1%    0.1%    0.0%    0.1%    0.0%    0.2%    0.0%    0.0%    0.0%    0.0%    0.0%    0.0%    0.0%    0.1%    0.0%    0.1%    0.0%    0.0%    0.0%    0.0%    0.0%    0.0%    0.0%    0.1%    0.0%    0.0%    0.0%    0.1%    0.0%    0.0%    0.1%    0.1%    0.1%    0.1%    0.0%    0.0%    0.0%    0.1%    0.0%    0.0%    0.0%    0.0%    0.1%    0.0%    0.0%    0.0%    0.0%    0.0%    0.0%    0.0%    0.0%    0.0%    0.0%    0.0%    0.0%    0.0%    0.0%    0.0%    0.0%    0.0%    0.0%    0.0%    0.0%    0.0%    0.0%    0.0%    0.0%    0.1%    0.0%    0.0%    0.0%    0.0%    0.0%    0.0%    0.1%    0.0%    0.0%    0.0%    0.0%    0.1%    0.1%    0.1%    0.0%    0.2%    0.0%    0.0%    0.0%    0.0%    0.0%    0.0%    0.1%    0.0%    0.0%    0.0%    0.0%    0.0%    0.0%    0.0%    0.0%    0.0%    0.0%    0.0%    0.0%    0.2%    0.0%    0.1%    0.0%    0.1%    0.0%    0.0%    0.0%    0.0%    0.0%    0.0%    0.0%    0.0%    0.0%    0.0%    0.0%    0.0%    0.0%    0.1%    0.0%    0.0%    0.0%    0.0%    0.0%    0.0%    0.0%    0.0%    0.0%    0.1%    0.1%    0.0%    0.0%    0.1%    0.0%    0.0%    0.0%    0.0%    0.0%    0.0%    0.0%    0.1%    0.1%    0.0%    0.0%    0.0%    0.0%    0.0%    0.0%    0.0%    0.0%    0.0%    0.0%    0.0%    0.0%    0.0%    0.2%    0.0%    0.0%    0.0%    0.0%    0.0%    0.0%    0.1%    0.0%    0.0%    0.0%    0.0%    0.0%    0.0%    0.0%    0.0%    0.1%    0.0%    0.0%    0.0%    0.0%    0.0%    0.0%    0.0%    0.0%    0.0%    0.0%    0.0%    0.0%    0.0%    0.0%    0.0%    0.0%    0.0%    0.0%    0.0%    0.0%    0.0%    0.0%    0.0%    0.0%    0.0%    0.0%    0.0%    0.0%    0.0%    0.0%    0.0%    0.0%    0.0%    0.0%    0.0%    0.0%    0.0%    0.0%    0.0%    0.0%    0.0%    0.0%    0.0%    0.0%    0.0%    0.0%    0.0%    0.0%    0.0%    0.0%    0.0%    0.0%    0.0%    0.0%        k__Bacteria; p__Lentisphaerae       7    0.0%    0.0%    0.0%    0.0%    0.0%    0.0%    0.0%    0.0%    0.0%    0.0%    0.0%    0.0%    0.0%    0.0%    0.0%    0.0%    0.0%    0.0%    0.0%    0.0%    0.0%    0.0%    0.0%    0.0%    0.0%    0.0%    0.0%    0.0%    0.0%    0.0%    0.0%    0.0%    0.0%    0.0%    0.0%    0.0%    0.0%    0.0%    0.0%    0.0%    0.0%    0.0%    0.0%    0.0%    0.0%    0.0%    0.0%    0.0%    0.0%    0.0%    0.0%    0.0%    0.0%    0.0%    0.0%    0.0%    0.0%    0.0%    0.0%    0.0%    0.0%    0.0%    0.0%    0.0%    0.0%    0.0%    0.0%    0.0%    0.0%    0.0%    0.0%    0.0%    0.0%    0.0%    0.0%    0.0%    0.0%    0.0%    0.0%    0.0%    0.0%    0.0%    0.0%    0.0%    0.0%    0.0%    0.0%    0.0%    0.0%    0.0%    0.0%    0.0%    0.0%    0.0%    0.0%    0.0%    0.0%    0.0%    0.0%    0.0%    0.0%    0.0%    0.0%    0.0%    0.0%    0.0%    0.0%    0.0%    0.0%    0.0%    0.0%    0.0%    0.0%    0.0%    0.0%    0.0%    0.0%    0.0%    0.0%    0.0%    0.0%    0.0%    0.0%    0.0%    0.0%    0.0%    0.0%    0.0%    0.0%    0.0%    0.0%    0.0%    0.0%    0.0%    0.0%    0.0%    0.0%    0.0%    0.0%    0.0%    0.0%    0.0%    0.0%    0.0%    0.0%    0.0%    0.0%    0.0%    0.0%    0.0%    0.0%    0.0%    0.0%    0.0%    0.0%    0.0%    0.0%    0.0%    0.0%    0.0%    0.0%    0.0%    0.0%    0.0%    0.0%    0.0%    0.0%    0.0%    0.0%    0.0%    0.0%    0.0%    0.0%    0.0%    0.0%    0.0%    0.0%    0.0%    0.0%    0.0%    0.0%    0.0%    0.0%    0.0%    0.0%    0.0%    0.0%    0.0%    0.0%    0.0%    0.0%    0.0%    0.0%    0.0%    0.0%    0.0%    0.0%    0.0%    0.0%    0.0%    0.0%    0.0%    0.0%    0.0%    0.0%    0.0%    0.0%    0.0%    0.0%    0.0%    0.0%    0.0%    0.0%    0.0%    0.0%    0.0%    0.0%    0.0%    0.0%    0.0%    0.0%    0.0%    0.0%    0.0%    0.0%    0.0%    0.0%    0.0%    0.0%    0.0%    0.0%    0.0%    0.0%    0.0%    0.0%    0.0%    0.0%    0.0%    0.0%    0.0%    0.0%    0.0%    0.0%    0.0%    0.0%    0.0%    0.0%    0.0%    0.0%    0.0%    0.0%    0.0%    0.0%    0.0%    0.0%    0.0%    0.0%    0.0%    0.0%    0.0%    0.0%    0.0%    0.0%    0.0%    0.0%    0.0%    0.0%    0.0%    0.0%    0.0%    0.0%    0.0%    0.0%    0.0%    0.0%    0.0%    0.0%    0.0%    0.0%    0.0%    0.0%    0.0%    0.0%    0.0%    0.0%    0.0%    0.0%    0.0%    0.0%    0.0%    0.0%    0.0%    0.0%    0.0%    0.0%    0.0%    0.0%    0.0%    0.0%    0.0%    0.0%    0.0%    0.0%    0.0%    0.0%    0.0%    0.0%    0.0%    0.0%    0.0%    0.0%    0.0%    0.0%    0.0%    0.0%    0.0%    0.0%    0.0%    0.0%    0.0%    0.0%    0.0%    0.0%    0.0%    0.0%    0.0%    0.0%    0.0%    0.0%    0.0%    0.0%    0.0%    0.0%    0.0%    0.0%    0.0%    0.0%    0.0%    0.0%    0.0%    0.0%    0.0%    0.0%    0.0%    0.0%    0.0%    0.0%    0.0%    0.0%    0.0%    0.0%    0.0%    0.0%    0.0%    0.0%    0.0%    0.0%    0.0%        k__Bacteria; p__MVP-15       1    0.0%    0.0%    0.0%    0.0%    0.0%    0.0%    0.0%    0.0%    0.0%    0.0%    0.0%    0.0%    0.0%    0.0%    0.0%    0.0%    0.0%    0.0%    0.0%    0.0%    0.0%    0.0%    0.0%    0.0%    0.0%    0.0%    0.0%    0.0%    0.0%    0.0%    0.0%    0.0%    0.0%    0.0%    0.0%    0.0%    0.0%    0.0%    0.0%    0.0%    0.0%    0.0%    0.0%    0.0%    0.0%    0.0%    0.0%    0.0%    0.0%    0.0%    0.0%    0.0%    0.0%    0.0%    0.0%    0.0%    0.0%    0.0%    0.0%    0.0%    0.0%    0.0%    0.0%    0.0%    0.0%    0.0%    0.0%    0.0%    0.0%    0.0%    0.0%    0.0%    0.0%    0.0%    0.0%    0.0%    0.0%    0.0%    0.0%    0.0%    0.0%    0.0%    0.0%    0.0%    0.0%    0.0%    0.0%    0.0%    0.0%    0.0%    0.0%    0.0%    0.0%    0.0%    0.0%    0.0%    0.0%    0.0%    0.0%    0.0%    0.0%    0.0%    0.0%    0.0%    0.0%    0.0%    0.0%    0.0%    0.0%    0.0%    0.0%    0.0%    0.0%    0.0%    0.0%    0.0%    0.0%    0.0%    0.0%    0.0%    0.0%    0.0%    0.0%    0.0%    0.0%    0.0%    0.0%    0.0%    0.0%    0.0%    0.0%    0.0%    0.0%    0.0%    0.0%    0.0%    0.0%    0.0%    0.0%    0.0%    0.0%    0.0%    0.0%    0.0%    0.0%    0.0%    0.0%    0.0%    0.0%    0.0%    0.0%    0.0%    0.0%    0.0%    0.0%    0.0%    0.0%    0.0%    0.0%    0.0%    0.0%    0.0%    0.0%    0.0%    0.0%    0.0%    0.0%    0.0%    0.0%    0.0%    0.0%    0.0%    0.0%    0.0%    0.0%    0.0%    0.0%    0.0%    0.0%    0.0%    0.0%    0.0%    0.0%    0.0%    0.0%    0.0%    0.0%    0.0%    0.0%    0.0%    0.0%    0.0%    0.0%    0.0%    0.0%    0.0%    0.0%    0.0%    0.0%    0.0%    0.0%    0.0%    0.0%    0.0%    0.0%    0.0%    0.0%    0.0%    0.0%    0.0%    0.0%    0.0%    0.0%    0.0%    0.0%    0.0%    0.0%    0.0%    0.0%    0.0%    0.0%    0.0%    0.0%    0.0%    0.0%    0.0%    0.0%    0.0%    0.0%    0.0%    0.0%    0.0%    0.0%    0.0%    0.0%    0.0%    0.0%    0.0%    0.0%    0.0%    0.0%    0.0%    0.0%    0.0%    0.0%    0.0%    0.0%    0.0%    0.0%    0.0%    0.0%    0.0%    0.0%    0.0%    0.0%    0.0%    0.0%    0.0%    0.0%    0.0%    0.0%    0.0%    0.0%    0.0%    0.0%    0.0%    0.0%    0.0%    0.0%    0.0%    0.0%    0.0%    0.0%    0.0%    0.0%    0.0%    0.0%    0.0%    0.0%    0.0%    0.0%    0.0%    0.0%    0.0%    0.0%    0.0%    0.0%    0.0%    0.0%    0.0%    0.0%    0.0%    0.0%    0.0%    0.0%    0.0%    0.0%    0.0%    0.0%    0.0%    0.0%    0.0%    0.0%    0.0%    0.0%    0.0%    0.0%    0.0%    0.0%    0.0%    0.0%    0.0%    0.0%    0.0%    0.0%    0.0%    0.0%    0.0%    0.0%    0.0%    0.0%    0.0%    0.0%    0.0%    0.0%    0.0%    0.0%    0.0%    0.0%    0.0%    0.0%    0.0%    0.0%    0.0%    0.0%    0.0%    0.0%    0.0%    0.0%    0.0%    0.0%    0.0%    0.0%    0.0%    0.0%    0.0%    0.0%    0.0%    0.0%    0.0%    0.0%    0.0%    0.0%    0.0%    0.0%    0.0%    0.0%    0.0%        k__Bacteria; p__NC10       6    0.0%    0.0%    0.0%    0.0%    0.0%    0.0%    0.0%    0.0%    0.0%    0.0%    0.0%    0.0%    0.0%    0.0%    0.0%    0.0%    0.0%    0.0%    0.0%    0.0%    0.0%    0.0%    0.0%    0.0%    0.0%    0.0%    0.0%    0.0%    0.0%    0.0%    0.0%    0.0%    0.0%    0.0%    0.0%    0.0%    0.0%    0.0%    0.0%    0.0%    0.0%    0.0%    0.0%    0.0%    0.0%    0.0%    0.0%    0.0%    0.0%    0.0%    0.0%    0.0%    0.0%    0.0%    0.0%    0.0%    0.0%    0.0%    0.0%    0.0%    0.0%    0.0%    0.0%    0.0%    0.0%    0.0%    0.0%    0.0%    0.0%    0.0%    0.0%    0.0%    0.0%    0.0%    0.0%    0.0%    0.0%    0.0%    0.0%    0.0%    0.0%    0.0%    0.0%    0.0%    0.0%    0.0%    0.0%    0.0%    0.0%    0.0%    0.0%    0.0%    0.0%    0.0%    0.0%    0.0%    0.0%    0.0%    0.0%    0.0%    0.0%    0.0%    0.0%    0.0%    0.0%    0.0%    0.0%    0.0%    0.0%    0.0%    0.0%    0.0%    0.0%    0.0%    0.0%    0.0%    0.0%    0.0%    0.0%    0.0%    0.0%    0.0%    0.0%    0.0%    0.0%    0.0%    0.0%    0.0%    0.0%    0.0%    0.0%    0.0%    0.0%    0.0%    0.0%    0.0%    0.0%    0.0%    0.0%    0.0%    0.0%    0.0%    0.0%    0.0%    0.0%    0.0%    0.0%    0.0%    0.0%    0.0%    0.0%    0.0%    0.0%    0.0%    0.0%    0.0%    0.0%    0.0%    0.0%    0.0%    0.0%    0.0%    0.0%    0.0%    0.0%    0.0%    0.0%    0.0%    0.0%    0.0%    0.0%    0.0%    0.0%    0.0%    0.0%    0.0%    0.0%    0.0%    0.0%    0.0%    0.0%    0.0%    0.0%    0.0%    0.0%    0.0%    0.0%    0.0%    0.0%    0.0%    0.0%    0.0%    0.0%    0.0%    0.0%    0.0%    0.0%    0.0%    0.0%    0.0%    0.0%    0.0%    0.0%    0.0%    0.0%    0.0%    0.0%    0.0%    0.0%    0.0%    0.0%    0.0%    0.0%    0.0%    0.0%    0.0%    0.0%    0.0%    0.0%    0.0%    0.0%    0.0%    0.0%    0.0%    0.0%    0.0%    0.0%    0.0%    0.0%    0.0%    0.0%    0.0%    0.0%    0.0%    0.0%    0.0%    0.0%    0.0%    0.0%    0.0%    0.0%    0.0%    0.0%    0.0%    0.0%    0.0%    0.0%    0.0%    0.0%    0.0%    0.0%    0.0%    0.0%    0.0%    0.0%    0.0%    0.0%    0.0%    0.0%    0.0%    0.0%    0.0%    0.0%    0.0%    0.0%    0.0%    0.0%    0.0%    0.0%    0.0%    0.0%    0.0%    0.0%    0.0%    0.0%    0.0%    0.0%    0.0%    0.0%    0.0%    0.0%    0.0%    0.0%    0.0%    0.0%    0.0%    0.0%    0.0%    0.0%    0.0%    0.0%    0.0%    0.0%    0.0%    0.0%    0.0%    0.0%    0.0%    0.0%    0.0%    0.0%    0.0%    0.0%    0.0%    0.0%    0.0%    0.0%    0.0%    0.0%    0.0%    0.0%    0.0%    0.0%    0.0%    0.0%    0.0%    0.0%    0.0%    0.0%    0.0%    0.0%    0.0%    0.0%    0.0%    0.0%    0.0%    0.0%    0.0%    0.0%    0.0%    0.0%    0.0%    0.0%    0.0%    0.0%    0.0%    0.0%    0.0%    0.0%    0.0%    0.0%    0.0%    0.0%    0.0%    0.0%    0.0%    0.0%    0.0%    0.0%    0.0%    0.0%    0.0%    0.0%    0.0%    0.0%    0.0%    0.0%    0.0%        k__Bacteria; p__NKB19      24    0.0%    0.0%    0.0%    0.0%    0.0%    0.0%    0.0%    0.0%    0.0%    0.0%    0.0%    0.0%    0.0%    0.0%    0.0%    0.0%    0.0%    0.0%    0.0%    0.0%    0.0%    0.0%    0.0%    0.0%    0.0%    0.0%    0.0%    0.0%    0.0%    0.0%    0.0%    0.0%    0.0%    0.0%    0.0%    0.0%    0.0%    0.0%    0.0%    0.0%    0.0%    0.0%    0.0%    0.0%    0.0%    0.0%    0.0%    0.0%    0.0%    0.0%    0.0%    0.0%    0.0%    0.0%    0.0%    0.0%    0.0%    0.0%    0.0%    0.0%    0.0%    0.0%    0.0%    0.0%    0.0%    0.0%    0.0%    0.0%    0.0%    0.0%    0.0%    0.0%    0.0%    0.0%    0.0%    0.0%    0.0%    0.0%    0.0%    0.0%    0.0%    0.0%    0.0%    0.0%    0.0%    0.0%    0.0%    0.0%    0.0%    0.0%    0.0%    0.0%    0.0%    0.0%    0.0%    0.0%    0.0%    0.0%    0.0%    0.0%    0.0%    0.0%    0.0%    0.0%    0.0%    0.0%    0.0%    0.0%    0.0%    0.0%    0.0%    0.0%    0.0%    0.0%    0.0%    0.0%    0.0%    0.0%    0.0%    0.0%    0.0%    0.0%    0.0%    0.0%    0.0%    0.0%    0.0%    0.0%    0.0%    0.0%    0.0%    0.0%    0.0%    0.0%    0.0%    0.0%    0.0%    0.0%    0.0%    0.0%    0.0%    0.0%    0.0%    0.0%    0.0%    0.0%    0.0%    0.0%    0.0%    0.0%    0.0%    0.0%    0.0%    0.0%    0.0%    0.0%    0.0%    0.0%    0.0%    0.0%    0.0%    0.0%    0.0%    0.0%    0.0%    0.0%    0.0%    0.0%    0.0%    0.0%    0.0%    0.0%    0.0%    0.0%    0.0%    0.0%    0.0%    0.0%    0.0%    0.0%    0.0%    0.0%    0.0%    0.0%    0.0%    0.0%    0.0%    0.0%    0.0%    0.0%    0.0%    0.0%    0.0%    0.0%    0.0%    0.0%    0.0%    0.0%    0.0%    0.0%    0.0%    0.0%    0.0%    0.0%    0.0%    0.0%    0.0%    0.0%    0.0%    0.0%    0.0%    0.0%    0.0%    0.0%    0.0%    0.0%    0.0%    0.0%    0.0%    0.0%    0.0%    0.0%    0.0%    0.0%    0.0%    0.0%    0.0%    0.0%    0.0%    0.0%    0.0%    0.0%    0.0%    0.0%    0.0%    0.0%    0.0%    0.0%    0.0%    0.0%    0.0%    0.0%    0.0%    0.0%    0.0%    0.0%    0.0%    0.0%    0.0%    0.0%    0.0%    0.0%    0.0%    0.0%    0.0%    0.0%    0.0%    0.0%    0.0%    0.0%    0.0%    0.0%    0.0%    0.0%    0.0%    0.0%    0.0%    0.0%    0.0%    0.0%    0.0%    0.0%    0.0%    0.0%    0.0%    0.0%    0.0%    0.0%    0.0%    0.0%    0.0%    0.0%    0.0%    0.0%    0.0%    0.0%    0.0%    0.0%    0.0%    0.0%    0.0%    0.0%    0.0%    0.0%    0.0%    0.0%    0.0%    0.0%    0.0%    0.0%    0.0%    0.0%    0.0%    0.0%    0.0%    0.0%    0.0%    0.0%    0.0%    0.0%    0.0%    0.0%    0.0%    0.0%    0.0%    0.0%    0.0%    0.0%    0.0%    0.0%    0.0%    0.0%    0.0%    0.0%    0.0%    0.0%    0.0%    0.0%    0.0%    0.0%    0.0%    0.0%    0.0%    0.0%    0.0%    0.0%    0.0%    0.0%    0.0%    0.0%    0.0%    0.0%    0.0%    0.0%    0.0%    0.0%    0.0%    0.0%    0.0%    0.0%    0.0%    0.0%    0.0%    0.0%    0.0%    0.0%    0.0%    0.0%        k__Bacteria; p__Nitrospirae     714    0.0%    0.0%    0.0%    0.0%    0.0%    0.0%    0.0%    0.0%    0.0%    0.0%    0.0%    0.0%    0.0%    0.0%    0.0%    0.0%    0.0%    0.0%    0.0%    0.0%    0.0%    0.0%    0.0%    0.0%    0.0%    0.0%    0.0%    0.0%    0.0%    0.0%    0.0%    0.0%    0.0%    0.0%    0.0%    0.0%    0.0%    0.0%    0.0%    0.0%    0.0%    0.0%    0.0%    0.0%    0.0%    0.0%    0.0%    0.0%    0.0%    0.0%    0.0%    0.0%    0.0%    0.0%    0.0%    0.0%    0.0%    0.0%    0.0%    0.0%    0.0%    0.0%    0.0%    0.0%    0.0%    0.0%    0.0%    0.0%    0.0%    0.0%    0.0%    0.0%    0.0%    0.0%    0.0%    0.0%    0.0%    0.0%    0.0%    0.0%    0.0%    0.0%    0.0%    0.0%    0.0%    0.0%    0.0%    0.0%    0.0%    0.0%    0.0%    0.0%    0.0%    0.0%    0.0%    0.0%    0.0%    0.0%    0.0%    0.0%    0.0%    0.0%    0.0%    0.0%    0.0%    0.0%    0.0%    0.0%    0.0%    0.0%    0.0%    0.0%    0.0%    0.0%    0.0%    0.0%    0.0%    0.0%    0.0%    0.0%    0.0%    0.0%    0.0%    0.0%    0.0%    0.0%    0.0%    0.0%    0.0%    0.0%    0.0%    0.0%    0.0%    0.0%    0.0%    0.0%    0.0%    0.0%    0.0%    0.0%    0.0%    0.0%    0.0%    0.0%    0.0%    0.0%    0.0%    0.0%    0.0%    0.0%    0.0%    0.0%    0.0%    0.0%    0.0%    0.0%    0.0%    0.0%    0.0%    0.0%    0.0%    0.0%    0.0%    0.0%    0.0%    0.0%    0.0%    0.0%    0.0%    0.0%    0.0%    0.0%    0.0%    0.0%    0.0%    0.0%    0.0%    0.0%    0.0%    0.0%    0.0%    0.0%    0.0%    0.0%    0.0%    0.0%    0.0%    0.0%    0.0%    0.0%    0.0%    0.0%    0.0%    0.0%    0.0%    0.0%    0.0%    0.0%    0.0%    0.0%    0.0%    0.0%    0.0%    0.0%    0.0%    0.0%    0.0%    0.0%    0.0%    0.0%    0.0%    0.0%    0.0%    0.0%    0.0%    0.0%    0.0%    0.0%    0.0%    0.0%    0.0%    0.0%    0.0%    0.0%    0.0%    0.0%    0.0%    0.0%    0.0%    0.0%    0.0%    0.0%    0.0%    0.0%    0.0%    0.0%    0.0%    0.0%    0.0%    0.0%    0.0%    0.0%    0.0%    0.0%    0.0%    0.0%    0.0%    0.0%    0.0%    0.0%    0.0%    0.0%    0.0%    0.0%    0.0%    0.0%    0.0%    0.0%    0.0%    0.0%    0.0%    0.0%    0.0%    0.0%    0.0%    0.0%    0.0%    0.0%    0.0%    0.0%    0.0%    0.0%    0.1%    0.0%    0.0%    0.0%    0.0%    0.0%    0.0%    0.0%    0.0%    0.0%    0.0%    0.0%    0.0%    0.0%    0.0%    0.1%    0.0%    0.0%    0.0%    0.0%    0.0%    0.0%    0.0%    0.0%    0.0%    0.0%    0.0%    0.0%    0.0%    0.0%    0.0%    0.0%    0.0%    0.0%    0.0%    0.0%    0.0%    0.0%    0.0%    0.0%    0.0%    0.0%    0.0%    0.0%    0.0%    0.0%    0.0%    0.0%    0.0%    0.0%    0.0%    0.0%    0.0%    0.0%    0.0%    0.0%    0.0%    0.0%    0.0%    0.0%    0.0%    0.0%    0.0%    0.0%    0.0%    0.0%    0.0%    0.0%    0.0%    0.0%    0.0%    0.0%    0.0%    0.0%    0.0%    0.0%    0.0%    0.0%    0.0%    0.0%    0.0%    0.0%    0.0%    0.0%    0.0%    0.0%        k__Bacteria; p__OP10     484    0.0%    0.0%    0.0%    0.0%    0.0%    0.0%    0.0%    0.0%    0.0%    0.0%    0.0%    0.0%    0.0%    0.0%    0.0%    0.0%    0.0%    0.0%    0.0%    0.0%    0.0%    0.0%    0.0%    0.0%    0.0%    0.0%    0.0%    0.0%    0.0%    0.0%    0.0%    0.0%    0.0%    0.0%    0.0%    0.0%    0.0%    0.0%    0.0%    0.0%    0.0%    0.0%    0.0%    0.0%    0.0%    0.0%    0.0%    0.0%    0.0%    0.0%    0.0%    0.0%    0.0%    0.0%    0.0%    0.0%    0.0%    0.0%    0.0%    0.0%    0.0%    0.0%    0.0%    0.0%    0.0%    0.0%    0.0%    0.0%    0.0%    0.0%    0.0%    0.0%    0.0%    0.0%    0.0%    0.0%    0.0%    0.0%    0.0%    0.0%    0.0%    0.0%    0.0%    0.0%    0.0%    0.0%    0.0%    0.0%    0.0%    0.0%    0.0%    0.0%    0.0%    0.0%    0.0%    0.0%    0.0%    0.0%    0.0%    0.0%    0.0%    0.0%    0.0%    0.0%    0.0%    0.0%    0.0%    0.0%    0.0%    0.0%    0.0%    0.0%    0.0%    0.0%    0.0%    0.0%    0.0%    0.0%    0.0%    0.0%    0.0%    0.0%    0.0%    0.0%    0.0%    0.0%    0.0%    0.0%    0.0%    0.0%    0.0%    0.0%    0.0%    0.0%    0.0%    0.0%    0.0%    0.0%    0.0%    0.0%    0.0%    0.0%    0.0%    0.0%    0.0%    0.0%    0.0%    0.0%    0.0%    0.0%    0.0%    0.0%    0.0%    0.0%    0.0%    0.0%    0.0%    0.0%    0.0%    0.0%    0.0%    0.0%    0.0%    0.0%    0.0%    0.0%    0.0%    0.0%    0.0%    0.0%    0.0%    0.0%    0.0%    0.0%    0.0%    0.0%    0.0%    0.0%    0.0%    0.0%    0.0%    0.0%    0.0%    0.0%    0.0%    0.0%    0.0%    0.0%    0.0%    0.0%    0.0%    0.0%    0.0%    0.0%    0.0%    0.0%    0.0%    0.0%    0.0%    0.0%    0.0%    0.0%    0.0%    0.0%    0.0%    0.0%    0.0%    0.0%    0.0%    0.0%    0.0%    0.0%    0.0%    0.0%    0.0%    0.0%    0.0%    0.0%    0.0%    0.0%    0.0%    0.0%    0.0%    0.0%    0.0%    0.0%    0.0%    0.0%    0.0%    0.0%    0.0%    0.0%    0.0%    0.0%    0.0%    0.0%    0.0%    0.0%    0.0%    0.0%    0.0%    0.0%    0.0%    0.0%    0.0%    0.0%    0.0%    0.0%    0.0%    0.0%    0.0%    0.0%    0.0%    0.0%    0.0%    0.0%    0.0%    0.0%    0.0%    0.0%    0.0%    0.0%    0.0%    0.0%    0.0%    0.0%    0.0%    0.0%    0.0%    0.0%    0.0%    0.0%    0.0%    0.0%    0.0%    0.0%    0.0%    0.0%    0.0%    0.0%    0.0%    0.0%    0.0%    0.0%    0.0%    0.0%    0.0%    0.0%    0.0%    0.0%    0.0%    0.0%    0.0%    0.0%    0.0%    0.0%    0.0%    0.0%    0.0%    0.0%    0.0%    0.0%    0.0%    0.0%    0.0%    0.0%    0.0%    0.0%    0.0%    0.0%    0.0%    0.0%    0.0%    0.0%    0.0%    0.0%    0.0%    0.0%    0.0%    0.0%    0.0%    0.0%    0.0%    0.0%    0.0%    0.0%    0.0%    0.0%    0.0%    0.0%    0.0%    0.0%    0.0%    0.0%    0.0%    0.0%    0.0%    0.0%    0.0%    0.0%    0.0%    0.0%    0.0%    0.0%    0.0%    0.0%    0.0%    0.0%    0.0%    0.0%    0.0%    0.0%    0.0%    0.0%    0.0%    0.0%    0.0%    0.0%        k__Bacteria; p__OP11       2    0.0%    0.0%    0.0%    0.0%    0.0%    0.0%    0.0%    0.0%    0.0%    0.0%    0.0%    0.0%    0.0%    0.0%    0.0%    0.0%    0.0%    0.0%    0.0%    0.0%    0.0%    0.0%    0.0%    0.0%    0.0%    0.0%    0.0%    0.0%    0.0%    0.0%    0.0%    0.0%    0.0%    0.0%    0.0%    0.0%    0.0%    0.0%    0.0%    0.0%    0.0%    0.0%    0.0%    0.0%    0.0%    0.0%    0.0%    0.0%    0.0%    0.0%    0.0%    0.0%    0.0%    0.0%    0.0%    0.0%    0.0%    0.0%    0.0%    0.0%    0.0%    0.0%    0.0%    0.0%    0.0%    0.0%    0.0%    0.0%    0.0%    0.0%    0.0%    0.0%    0.0%    0.0%    0.0%    0.0%    0.0%    0.0%    0.0%    0.0%    0.0%    0.0%    0.0%    0.0%    0.0%    0.0%    0.0%    0.0%    0.0%    0.0%    0.0%    0.0%    0.0%    0.0%    0.0%    0.0%    0.0%    0.0%    0.0%    0.0%    0.0%    0.0%    0.0%    0.0%    0.0%    0.0%    0.0%    0.0%    0.0%    0.0%    0.0%    0.0%    0.0%    0.0%    0.0%    0.0%    0.0%    0.0%    0.0%    0.0%    0.0%    0.0%    0.0%    0.0%    0.0%    0.0%    0.0%    0.0%    0.0%    0.0%    0.0%    0.0%    0.0%    0.0%    0.0%    0.0%    0.0%    0.0%    0.0%    0.0%    0.0%    0.0%    0.0%    0.0%    0.0%    0.0%    0.0%    0.0%    0.0%    0.0%    0.0%    0.0%    0.0%    0.0%    0.0%    0.0%    0.0%    0.0%    0.0%    0.0%    0.0%    0.0%    0.0%    0.0%    0.0%    0.0%    0.0%    0.0%    0.0%    0.0%    0.0%    0.0%    0.0%    0.0%    0.0%    0.0%    0.0%    0.0%    0.0%    0.0%    0.0%    0.0%    0.0%    0.0%    0.0%    0.0%    0.0%    0.0%    0.0%    0.0%    0.0%    0.0%    0.0%    0.0%    0.0%    0.0%    0.0%    0.0%    0.0%    0.0%    0.0%    0.0%    0.0%    0.0%    0.0%    0.0%    0.0%    0.0%    0.0%    0.0%    0.0%    0.0%    0.0%    0.0%    0.0%    0.0%    0.0%    0.0%    0.0%    0.0%    0.0%    0.0%    0.0%    0.0%    0.0%    0.0%    0.0%    0.0%    0.0%    0.0%    0.0%    0.0%    0.0%    0.0%    0.0%    0.0%    0.0%    0.0%    0.0%    0.0%    0.0%    0.0%    0.0%    0.0%    0.0%    0.0%    0.0%    0.0%    0.0%    0.0%    0.0%    0.0%    0.0%    0.0%    0.0%    0.0%    0.0%    0.0%    0.0%    0.0%    0.0%    0.0%    0.0%    0.0%    0.0%    0.0%    0.0%    0.0%    0.0%    0.0%    0.0%    0.0%    0.0%    0.0%    0.0%    0.0%    0.0%    0.0%    0.0%    0.0%    0.0%    0.0%    0.0%    0.0%    0.0%    0.0%    0.0%    0.0%    0.0%    0.0%    0.0%    0.0%    0.0%    0.0%    0.0%    0.0%    0.0%    0.0%    0.0%    0.0%    0.0%    0.0%    0.0%    0.0%    0.0%    0.0%    0.0%    0.0%    0.0%    0.0%    0.0%    0.0%    0.0%    0.0%    0.0%    0.0%    0.0%    0.0%    0.0%    0.0%    0.0%    0.0%    0.0%    0.0%    0.0%    0.0%    0.0%    0.0%    0.0%    0.0%    0.0%    0.0%    0.0%    0.0%    0.0%    0.0%    0.0%    0.0%    0.0%    0.0%    0.0%    0.0%    0.0%    0.0%    0.0%    0.0%    0.0%    0.0%    0.0%    0.0%    0.0%    0.0%    0.0%    0.0%    0.0%    0.0%    0.0%    0.0%        k__Bacteria; p__OP3       2    0.0%    0.0%    0.0%    0.0%    0.0%    0.0%    0.0%    0.0%    0.0%    0.0%    0.0%    0.0%    0.0%    0.0%    0.0%    0.0%    0.0%    0.0%    0.0%    0.0%    0.0%    0.0%    0.0%    0.0%    0.0%    0.0%    0.0%    0.0%    0.0%    0.0%    0.0%    0.0%    0.0%    0.0%    0.0%    0.0%    0.0%    0.0%    0.0%    0.0%    0.0%    0.0%    0.0%    0.0%    0.0%    0.0%    0.0%    0.0%    0.0%    0.0%    0.0%    0.0%    0.0%    0.0%    0.0%    0.0%    0.0%    0.0%    0.0%    0.0%    0.0%    0.0%    0.0%    0.0%    0.0%    0.0%    0.0%    0.0%    0.0%    0.0%    0.0%    0.0%    0.0%    0.0%    0.0%    0.0%    0.0%    0.0%    0.0%    0.0%    0.0%    0.0%    0.0%    0.0%    0.0%    0.0%    0.0%    0.0%    0.0%    0.0%    0.0%    0.0%    0.0%    0.0%    0.0%    0.0%    0.0%    0.0%    0.0%    0.0%    0.0%    0.0%    0.0%    0.0%    0.0%    0.0%    0.0%    0.0%    0.0%    0.0%    0.0%    0.0%    0.0%    0.0%    0.0%    0.0%    0.0%    0.0%    0.0%    0.0%    0.0%    0.0%    0.0%    0.0%    0.0%    0.0%    0.0%    0.0%    0.0%    0.0%    0.0%    0.0%    0.0%    0.0%    0.0%    0.0%    0.0%    0.0%    0.0%    0.0%    0.0%    0.0%    0.0%    0.0%    0.0%    0.0%    0.0%    0.0%    0.0%    0.0%    0.0%    0.0%    0.0%    0.0%    0.0%    0.0%    0.0%    0.0%    0.0%    0.0%    0.0%    0.0%    0.0%    0.0%    0.0%    0.0%    0.0%    0.0%    0.0%    0.0%    0.0%    0.0%    0.0%    0.0%    0.0%    0.0%    0.0%    0.0%    0.0%    0.0%    0.0%    0.0%    0.0%    0.0%    0.0%    0.0%    0.0%    0.0%    0.0%    0.0%    0.0%    0.0%    0.0%    0.0%    0.0%    0.0%    0.0%    0.0%    0.0%    0.0%    0.0%    0.0%    0.0%    0.0%    0.0%    0.0%    0.0%    0.0%    0.0%    0.0%    0.0%    0.0%    0.0%    0.0%    0.0%    0.0%    0.0%    0.0%    0.0%    0.0%    0.0%    0.0%    0.0%    0.0%    0.0%    0.0%    0.0%    0.0%    0.0%    0.0%    0.0%    0.0%    0.0%    0.0%    0.0%    0.0%    0.0%    0.0%    0.0%    0.0%    0.0%    0.0%    0.0%    0.0%    0.0%    0.0%    0.0%    0.0%    0.0%    0.0%    0.0%    0.0%    0.0%    0.0%    0.0%    0.0%    0.0%    0.0%    0.0%    0.0%    0.0%    0.0%    0.0%    0.0%    0.0%    0.0%    0.0%    0.0%    0.0%    0.0%    0.0%    0.0%    0.0%    0.0%    0.0%    0.0%    0.0%    0.0%    0.0%    0.0%    0.0%    0.0%    0.0%    0.0%    0.0%    0.0%    0.0%    0.0%    0.0%    0.0%    0.0%    0.0%    0.0%    0.0%    0.0%    0.0%    0.0%    0.0%    0.0%    0.0%    0.0%    0.0%    0.0%    0.0%    0.0%    0.0%    0.0%    0.0%    0.0%    0.0%    0.0%    0.0%    0.0%    0.0%    0.0%    0.0%    0.0%    0.0%    0.0%    0.0%    0.0%    0.0%    0.0%    0.0%    0.0%    0.0%    0.0%    0.0%    0.0%    0.0%    0.0%    0.0%    0.0%    0.0%    0.0%    0.0%    0.0%    0.0%    0.0%    0.0%    0.0%    0.0%    0.0%    0.0%    0.0%    0.0%    0.0%    0.0%    0.0%    0.0%    0.0%    0.0%    0.0%    0.0%    0.0%    0.0%    0.0%    0.0%        k__Bacteria; p__OP8       1    0.0%    0.0%    0.0%    0.0%    0.0%    0.0%    0.0%    0.0%    0.0%    0.0%    0.0%    0.0%    0.0%    0.0%    0.0%    0.0%    0.0%    0.0%    0.0%    0.0%    0.0%    0.0%    0.0%    0.0%    0.0%    0.0%    0.0%    0.0%    0.0%    0.0%    0.0%    0.0%    0.0%    0.0%    0.0%    0.0%    0.0%    0.0%    0.0%    0.0%    0.0%    0.0%    0.0%    0.0%    0.0%    0.0%    0.0%    0.0%    0.0%    0.0%    0.0%    0.0%    0.0%    0.0%    0.0%    0.0%    0.0%    0.0%    0.0%    0.0%    0.0%    0.0%    0.0%    0.0%    0.0%    0.0%    0.0%    0.0%    0.0%    0.0%    0.0%    0.0%    0.0%    0.0%    0.0%    0.0%    0.0%    0.0%    0.0%    0.0%    0.0%    0.0%    0.0%    0.0%    0.0%    0.0%    0.0%    0.0%    0.0%    0.0%    0.0%    0.0%    0.0%    0.0%    0.0%    0.0%    0.0%    0.0%    0.0%    0.0%    0.0%    0.0%    0.0%    0.0%    0.0%    0.0%    0.0%    0.0%    0.0%    0.0%    0.0%    0.0%    0.0%    0.0%    0.0%    0.0%    0.0%    0.0%    0.0%    0.0%    0.0%    0.0%    0.0%    0.0%    0.0%    0.0%    0.0%    0.0%    0.0%    0.0%    0.0%    0.0%    0.0%    0.0%    0.0%    0.0%    0.0%    0.0%    0.0%    0.0%    0.0%    0.0%    0.0%    0.0%    0.0%    0.0%    0.0%    0.0%    0.0%    0.0%    0.0%    0.0%    0.0%    0.0%    0.0%    0.0%    0.0%    0.0%    0.0%    0.0%    0.0%    0.0%    0.0%    0.0%    0.0%    0.0%    0.0%    0.0%    0.0%    0.0%    0.0%    0.0%    0.0%    0.0%    0.0%    0.0%    0.0%    0.0%    0.0%    0.0%    0.0%    0.0%    0.0%    0.0%    0.0%    0.0%    0.0%    0.0%    0.0%    0.0%    0.0%    0.0%    0.0%    0.0%    0.0%    0.0%    0.0%    0.0%    0.0%    0.0%    0.0%    0.0%    0.0%    0.0%    0.0%    0.0%    0.0%    0.0%    0.0%    0.0%    0.0%    0.0%    0.0%    0.0%    0.0%    0.0%    0.0%    0.0%    0.0%    0.0%    0.0%    0.0%    0.0%    0.0%    0.0%    0.0%    0.0%    0.0%    0.0%    0.0%    0.0%    0.0%    0.0%    0.0%    0.0%    0.0%    0.0%    0.0%    0.0%    0.0%    0.0%    0.0%    0.0%    0.0%    0.0%    0.0%    0.0%    0.0%    0.0%    0.0%    0.0%    0.0%    0.0%    0.0%    0.0%    0.0%    0.0%    0.0%    0.0%    0.0%    0.0%    0.0%    0.0%    0.0%    0.0%    0.0%    0.0%    0.0%    0.0%    0.0%    0.0%    0.0%    0.0%    0.0%    0.0%    0.0%    0.0%    0.0%    0.0%    0.0%    0.0%    0.0%    0.0%    0.0%    0.0%    0.0%    0.0%    0.0%    0.0%    0.0%    0.0%    0.0%    0.0%    0.0%    0.0%    0.0%    0.0%    0.0%    0.0%    0.0%    0.0%    0.0%    0.0%    0.0%    0.0%    0.0%    0.0%    0.0%    0.0%    0.0%    0.0%    0.0%    0.0%    0.0%    0.0%    0.0%    0.0%    0.0%    0.0%    0.0%    0.0%    0.0%    0.0%    0.0%    0.0%    0.0%    0.0%    0.0%    0.0%    0.0%    0.0%    0.0%    0.0%    0.0%    0.0%    0.0%    0.0%    0.0%    0.0%    0.0%    0.0%    0.0%    0.0%    0.0%    0.0%    0.0%    0.0%    0.0%    0.0%    0.0%    0.0%    0.0%    0.0%    0.0%    0.0%    0.0%    0.0%    0.0%        k__Bacteria; p__Planctomycetes    5856    0.0%    0.0%    0.0%    0.0%    0.0%    0.0%    0.0%    0.0%    0.0%    0.0%    0.0%    0.0%    0.0%    0.0%    0.0%    0.0%    0.0%    0.0%    0.0%    0.0%    0.1%    0.0%    0.0%    0.0%    0.0%    0.0%    0.0%    0.0%    0.1%    0.0%    0.0%    0.1%    0.0%    0.0%    0.0%    0.0%    0.0%    0.0%    0.0%    0.1%    0.0%    0.1%    0.0%    0.0%    0.0%    0.0%    0.0%    0.0%    0.0%    0.0%    0.0%    0.0%    0.0%    0.0%    0.1%    0.1%    0.0%    0.0%    0.0%    0.1%    0.1%    0.0%    0.0%    0.1%    0.1%    0.1%    0.0%    0.1%    0.0%    0.0%    0.0%    0.0%    0.0%    0.1%    0.0%    0.0%    0.0%    0.1%    0.0%    0.0%    0.0%    0.0%    0.0%    0.0%    0.0%    0.0%    0.0%    0.0%    0.0%    0.1%    0.0%    0.1%    0.1%    0.0%    0.0%    0.0%    0.1%    0.0%    0.0%    0.1%    0.1%    0.0%    0.0%    0.0%    0.0%    0.0%    0.1%    0.0%    0.0%    0.1%    0.0%    0.1%    0.0%    0.0%    0.1%    0.0%    0.0%    0.0%    0.0%    0.2%    0.0%    0.0%    0.1%    0.0%    0.0%    0.2%    0.1%    0.0%    0.1%    0.3%    0.1%    0.0%    0.1%    0.0%    0.2%    0.0%    0.0%    0.0%    0.0%    0.0%    0.0%    0.0%    0.0%    0.0%    0.1%    0.0%    0.0%    0.0%    0.0%    0.0%    0.0%    0.0%    0.1%    0.0%    0.0%    0.0%    0.0%    0.0%    0.0%    0.1%    0.2%    0.1%    0.1%    0.0%    0.0%    0.0%    0.1%    0.0%    0.0%    0.0%    0.1%    0.0%    0.0%    0.0%    0.0%    0.0%    0.0%    0.0%    0.0%    0.0%    0.0%    0.0%    0.0%    0.0%    0.0%    0.0%    0.0%    0.0%    0.0%    0.0%    0.0%    0.0%    0.0%    0.0%    0.0%    0.0%    0.1%    0.0%    0.0%    0.0%    0.1%    0.0%    0.0%    0.1%    0.1%    0.0%    0.0%    0.1%    0.2%    0.1%    0.1%    0.0%    0.2%    0.1%    0.0%    0.1%    0.0%    0.1%    0.0%    0.1%    0.0%    0.1%    0.0%    0.0%    0.0%    0.1%    0.0%    0.0%    0.1%    0.0%    0.0%    0.0%    0.1%    0.0%    0.1%    0.0%    0.1%    0.0%    0.0%    0.0%    0.0%    0.0%    0.0%    0.0%    0.0%    0.0%    0.0%    0.0%    0.0%    0.0%    0.1%    0.0%    0.0%    0.0%    0.0%    0.0%    0.0%    0.0%    0.0%    0.0%    0.1%    0.0%    0.1%    0.0%    0.0%    0.0%    0.0%    0.0%    0.0%    0.0%    0.0%    0.1%    0.1%    0.0%    0.0%    0.0%    0.0%    0.0%    0.0%    0.0%    0.0%    0.0%    0.0%    0.0%    0.0%    0.0%    0.0%    0.2%    0.0%    0.0%    0.0%    0.0%    0.0%    0.0%    0.0%    0.0%    0.0%    0.2%    0.0%    0.1%    0.0%    0.0%    0.0%    0.1%    0.0%    0.0%    0.0%    0.0%    0.0%    0.0%    0.0%    0.0%    0.0%    0.0%    0.0%    0.0%    0.0%    0.0%    0.0%    0.0%    0.0%    0.0%    0.0%    0.0%    0.0%    0.0%    0.0%    0.0%    0.0%    0.0%    0.0%    0.0%    0.0%    0.0%    0.0%    0.0%    0.0%    0.0%    0.0%    0.0%    0.0%    0.0%    0.0%    0.0%    0.0%    0.0%    0.0%    0.0%    0.0%    0.0%    0.0%    0.0%    0.0%    0.1%    0.0%    0.0%    0.0%    0.1%        k__Bacteria; p__Proteobacteria   4545818   26.7%   24.6%   17.4%   14.8%   35.1%   44.2%   13.5%   70.7%   13.4%   23.7%   28.0%   70.9%   44.3%   23.8%   23.4%   27.2%   18.3%   40.4%    8.9%   14.8%   30.2%   30.2%   18.9%    2.3%   13.8%    9.1%   21.6%   22.6%   27.0%    5.7%    4.4%   16.5%    6.1%   14.5%   29.8%   13.5%   25.4%    6.0%   39.2%   30.8%   22.9%   29.1%   29.0%   32.2%   17.2%   17.0%   25.5%    8.8%   26.7%   11.5%   28.1%   42.0%   49.6%   20.5%   16.0%    5.1%   13.6%   76.1%   30.1%   36.0%    9.2%   10.8%    9.0%   21.5%   16.8%    8.7%   44.3%   31.9%   33.5%   14.2%   60.2%    8.7%    7.5%   17.6%   16.6%    2.5%   22.9%   18.2%    5.0%   12.2%    8.6%   57.1%   21.6%   15.3%    7.2%    9.0%   22.0%   30.1%   27.7%   18.4%   55.5%   27.6%   20.0%   32.3%    4.4%   10.2%   23.3%   25.8%   14.4%   12.6%   25.9%   36.1%   31.5%   10.2%    5.2%   19.8%   29.0%   33.8%   52.3%   13.3%   12.2%   20.7%   31.0%    4.3%   22.6%   18.4%   39.7%   12.7%   46.5%   35.5%   81.8%   19.8%   21.5%   29.5%   87.3%   45.3%   30.7%   40.7%   45.6%   35.9%   18.4%   59.5%   33.8%   12.7%   32.9%   52.6%   64.1%   47.0%   81.0%   35.1%   77.6%   48.3%   39.9%   63.0%   33.7%   25.2%   26.9%   28.9%   18.9%   46.3%   33.6%   30.4%   38.3%   45.4%   13.8%   39.7%   36.2%   22.1%   60.8%   22.8%   16.9%   32.1%   53.9%   43.8%   43.6%   35.2%   23.0%   13.8%   28.9%   17.5%   37.2%   25.2%   19.4%   26.3%   26.6%   18.3%   15.7%   12.0%   29.5%   45.1%    9.6%   11.9%   33.5%   13.5%    5.9%    9.5%    7.9%   11.6%   13.6%   16.0%    6.5%   11.8%   11.8%   17.9%   23.6%   34.9%   18.0%   11.2%    8.0%    8.1%   28.2%    8.7%   17.3%   30.4%   25.2%   26.0%   80.1%   26.5%   30.0%   19.5%   17.7%   40.4%   46.3%   25.1%   23.5%   17.5%   51.9%   10.7%   22.8%   13.4%   10.1%   51.4%   75.0%   52.9%   46.4%   21.1%   18.0%   23.7%   19.9%   21.3%   28.5%   31.8%   60.9%   26.0%   29.4%   35.8%   29.0%   49.8%   19.4%   13.8%   21.7%   19.5%   17.3%   15.5%   31.6%    6.1%   14.2%   20.5%   38.8%   13.4%   40.5%   48.3%   60.5%   20.2%   28.4%   25.2%   37.1%   60.7%   23.5%   28.6%   41.1%   50.6%   27.5%   63.7%   34.9%   33.6%   29.6%   23.0%   36.4%   38.6%   21.4%   22.8%   53.3%   23.4%   43.8%   37.8%   17.3%   18.3%   32.9%    5.0%   19.7%   11.5%   31.4%   13.7%   39.8%   13.7%   45.1%   31.3%   11.7%   37.3%   18.6%    3.4%   20.1%   31.2%   14.3%    5.8%   15.9%   11.1%   14.1%   14.9%    7.3%   12.2%   12.6%   14.8%   14.5%   22.9%    9.9%    5.0%   23.0%   29.5%   12.5%   15.2%   36.1%   23.9%   13.3%   22.5%    3.2%   11.7%   10.9%   12.8%   19.6%   23.3%    5.1%    9.3%   24.7%   17.2%   14.1%   24.9%   19.9%   17.5%   14.5%   10.2%    8.5%    5.0%    3.3%    8.2%   30.8%   10.1%   17.1%   60.5%   16.6%   52.9%   11.7%   10.7%   24.8%    5.8%    8.2%    8.9%   56.7%    4.2%   22.2%   18.1%    6.7%   15.1%    4.7%   88.0%   22.7%    2.6%        k__Bacteria; p__SC3       6    0.0%    0.0%    0.0%    0.0%    0.0%    0.0%    0.0%    0.0%    0.0%    0.0%    0.0%    0.0%    0.0%    0.0%    0.0%    0.0%    0.0%    0.0%    0.0%    0.0%    0.0%    0.0%    0.0%    0.0%    0.0%    0.0%    0.0%    0.0%    0.0%    0.0%    0.0%    0.0%    0.0%    0.0%    0.0%    0.0%    0.0%    0.0%    0.0%    0.0%    0.0%    0.0%    0.0%    0.0%    0.0%    0.0%    0.0%    0.0%    0.0%    0.0%    0.0%    0.0%    0.0%    0.0%    0.0%    0.0%    0.0%    0.0%    0.0%    0.0%    0.0%    0.0%    0.0%    0.0%    0.0%    0.0%    0.0%    0.0%    0.0%    0.0%    0.0%    0.0%    0.0%    0.0%    0.0%    0.0%    0.0%    0.0%    0.0%    0.0%    0.0%    0.0%    0.0%    0.0%    0.0%    0.0%    0.0%    0.0%    0.0%    0.0%    0.0%    0.0%    0.0%    0.0%    0.0%    0.0%    0.0%    0.0%    0.0%    0.0%    0.0%    0.0%    0.0%    0.0%    0.0%    0.0%    0.0%    0.0%    0.0%    0.0%    0.0%    0.0%    0.0%    0.0%    0.0%    0.0%    0.0%    0.0%    0.0%    0.0%    0.0%    0.0%    0.0%    0.0%    0.0%    0.0%    0.0%    0.0%    0.0%    0.0%    0.0%    0.0%    0.0%    0.0%    0.0%    0.0%    0.0%    0.0%    0.0%    0.0%    0.0%    0.0%    0.0%    0.0%    0.0%    0.0%    0.0%    0.0%    0.0%    0.0%    0.0%    0.0%    0.0%    0.0%    0.0%    0.0%    0.0%    0.0%    0.0%    0.0%    0.0%    0.0%    0.0%    0.0%    0.0%    0.0%    0.0%    0.0%    0.0%    0.0%    0.0%    0.0%    0.0%    0.0%    0.0%    0.0%    0.0%    0.0%    0.0%    0.0%    0.0%    0.0%    0.0%    0.0%    0.0%    0.0%    0.0%    0.0%    0.0%    0.0%    0.0%    0.0%    0.0%    0.0%    0.0%    0.0%    0.0%    0.0%    0.0%    0.0%    0.0%    0.0%    0.0%    0.0%    0.0%    0.0%    0.0%    0.0%    0.0%    0.0%    0.0%    0.0%    0.0%    0.0%    0.0%    0.0%    0.0%    0.0%    0.0%    0.0%    0.0%    0.0%    0.0%    0.0%    0.0%    0.0%    0.0%    0.0%    0.0%    0.0%    0.0%    0.0%    0.0%    0.0%    0.0%    0.0%    0.0%    0.0%    0.0%    0.0%    0.0%    0.0%    0.0%    0.0%    0.0%    0.0%    0.0%    0.0%    0.0%    0.0%    0.0%    0.0%    0.0%    0.0%    0.0%    0.0%    0.0%    0.0%    0.0%    0.0%    0.0%    0.0%    0.0%    0.0%    0.0%    0.0%    0.0%    0.0%    0.0%    0.0%    0.0%    0.0%    0.0%    0.0%    0.0%    0.0%    0.0%    0.0%    0.0%    0.0%    0.0%    0.0%    0.0%    0.0%    0.0%    0.0%    0.0%    0.0%    0.0%    0.0%    0.0%    0.0%    0.0%    0.0%    0.0%    0.0%    0.0%    0.0%    0.0%    0.0%    0.0%    0.0%    0.0%    0.0%    0.0%    0.0%    0.0%    0.0%    0.0%    0.0%    0.0%    0.0%    0.0%    0.0%    0.0%    0.0%    0.0%    0.0%    0.0%    0.0%    0.0%    0.0%    0.0%    0.0%    0.0%    0.0%    0.0%    0.0%    0.0%    0.0%    0.0%    0.0%    0.0%    0.0%    0.0%    0.0%    0.0%    0.0%    0.0%    0.0%    0.0%    0.0%    0.0%    0.0%    0.0%    0.0%    0.0%    0.0%    0.0%    0.0%    0.0%    0.0%    0.0%    0.0%    0.0%    0.0%    0.0%    0.0%        k__Bacteria; p__SC4    1057    0.0%    0.0%    0.0%    0.0%    0.0%    0.0%    0.0%    0.0%    0.0%    0.0%    0.0%    0.0%    0.0%    0.0%    0.0%    0.0%    0.0%    0.0%    0.0%    0.0%    0.0%    0.0%    0.0%    0.0%    0.0%    0.0%    0.0%    0.0%    0.0%    0.0%    0.0%    0.0%    0.0%    0.0%    0.0%    0.0%    0.0%    0.0%    0.0%    0.0%    0.0%    0.0%    0.0%    0.0%    0.0%    0.0%    0.0%    0.0%    0.0%    0.0%    0.0%    0.0%    0.0%    0.0%    0.0%    0.0%    0.0%    0.0%    0.0%    0.0%    0.0%    0.0%    0.0%    0.1%    0.0%    0.0%    0.0%    0.0%    0.0%    0.0%    0.0%    0.0%    0.0%    0.0%    0.0%    0.0%    0.0%    0.0%    0.0%    0.0%    0.0%    0.0%    0.0%    0.0%    0.0%    0.0%    0.0%    0.0%    0.0%    0.0%    0.0%    0.0%    0.0%    0.0%    0.0%    0.0%    0.0%    0.0%    0.0%    0.0%    0.0%    0.0%    0.0%    0.0%    0.0%    0.0%    0.0%    0.0%    0.0%    0.0%    0.0%    0.0%    0.0%    0.0%    0.0%    0.0%    0.0%    0.0%    0.0%    0.0%    0.0%    0.0%    0.0%    0.0%    0.0%    0.0%    0.0%    0.0%    0.0%    0.0%    0.0%    0.0%    0.0%    0.0%    0.0%    0.0%    0.0%    0.0%    0.0%    0.0%    0.0%    0.0%    0.0%    0.0%    0.0%    0.0%    0.0%    0.0%    0.0%    0.0%    0.0%    0.0%    0.0%    0.0%    0.0%    0.0%    0.0%    0.0%    0.0%    0.0%    0.0%    0.0%    0.0%    0.0%    0.0%    0.0%    0.0%    0.0%    0.0%    0.0%    0.0%    0.0%    0.0%    0.0%    0.0%    0.0%    0.0%    0.0%    0.0%    0.0%    0.0%    0.0%    0.0%    0.0%    0.0%    0.0%    0.0%    0.0%    0.0%    0.0%    0.0%    0.0%    0.0%    0.0%    0.0%    0.0%    0.0%    0.0%    0.0%    0.0%    0.0%    0.0%    0.0%    0.0%    0.0%    0.0%    0.0%    0.0%    0.0%    0.0%    0.0%    0.0%    0.0%    0.0%    0.0%    0.0%    0.0%    0.0%    0.0%    0.0%    0.0%    0.0%    0.0%    0.0%    0.0%    0.0%    0.0%    0.0%    0.0%    0.0%    0.0%    0.0%    0.0%    0.0%    0.0%    0.0%    0.0%    0.0%    0.0%    0.0%    0.0%    0.0%    0.0%    0.0%    0.0%    0.0%    0.0%    0.0%    0.0%    0.0%    0.0%    0.0%    0.0%    0.0%    0.0%    0.0%    0.0%    0.0%    0.0%    0.0%    0.0%    0.0%    0.0%    0.0%    0.0%    0.0%    0.0%    0.0%    0.0%    0.0%    0.0%    0.0%    0.1%    0.0%    0.0%    0.0%    0.0%    0.0%    0.0%    0.0%    0.0%    0.0%    0.0%    0.0%    0.0%    0.0%    0.0%    0.1%    0.0%    0.0%    0.0%    0.0%    0.0%    0.0%    0.0%    0.0%    0.0%    0.0%    0.0%    0.1%    0.0%    0.0%    0.0%    0.1%    0.0%    0.0%    0.0%    0.0%    0.0%    0.0%    0.0%    0.0%    0.0%    0.0%    0.0%    0.0%    0.0%    0.0%    0.0%    0.0%    0.0%    0.0%    0.0%    0.0%    0.0%    0.0%    0.0%    0.0%    0.0%    0.0%    0.0%    0.0%    0.0%    0.0%    0.0%    0.0%    0.0%    0.0%    0.0%    0.0%    0.0%    0.0%    0.0%    0.0%    0.0%    0.0%    0.0%    0.0%    0.0%    0.0%    0.0%    0.0%    0.0%    0.0%    0.0%    0.0%    0.0%    0.0%        k__Bacteria; p__SPAM     334    0.0%    0.0%    0.0%    0.0%    0.0%    0.0%    0.0%    0.0%    0.0%    0.0%    0.0%    0.0%    0.0%    0.0%    0.0%    0.0%    0.0%    0.0%    0.0%    0.0%    0.0%    0.0%    0.0%    0.0%    0.0%    0.0%    0.0%    0.0%    0.0%    0.0%    0.0%    0.0%    0.0%    0.0%    0.0%    0.0%    0.0%    0.0%    0.0%    0.0%    0.0%    0.0%    0.0%    0.0%    0.0%    0.0%    0.0%    0.0%    0.0%    0.0%    0.0%    0.0%    0.0%    0.0%    0.0%    0.0%    0.0%    0.0%    0.0%    0.0%    0.0%    0.0%    0.0%    0.0%    0.0%    0.0%    0.0%    0.0%    0.0%    0.0%    0.0%    0.0%    0.0%    0.0%    0.0%    0.0%    0.0%    0.0%    0.0%    0.0%    0.0%    0.0%    0.0%    0.0%    0.0%    0.0%    0.0%    0.0%    0.0%    0.0%    0.0%    0.0%    0.0%    0.0%    0.0%    0.0%    0.0%    0.0%    0.0%    0.0%    0.0%    0.0%    0.0%    0.0%    0.0%    0.0%    0.0%    0.0%    0.0%    0.0%    0.0%    0.0%    0.0%    0.0%    0.0%    0.0%    0.0%    0.0%    0.0%    0.0%    0.0%    0.0%    0.0%    0.0%    0.0%    0.0%    0.0%    0.0%    0.0%    0.0%    0.0%    0.0%    0.0%    0.0%    0.0%    0.0%    0.0%    0.0%    0.0%    0.0%    0.0%    0.0%    0.0%    0.0%    0.0%    0.0%    0.0%    0.0%    0.0%    0.0%    0.0%    0.0%    0.0%    0.0%    0.0%    0.0%    0.0%    0.0%    0.0%    0.0%    0.0%    0.0%    0.0%    0.0%    0.0%    0.0%    0.0%    0.0%    0.0%    0.0%    0.0%    0.0%    0.0%    0.0%    0.0%    0.0%    0.0%    0.0%    0.0%    0.0%    0.0%    0.0%    0.0%    0.0%    0.0%    0.0%    0.0%    0.0%    0.0%    0.0%    0.0%    0.0%    0.0%    0.0%    0.0%    0.0%    0.0%    0.0%    0.0%    0.0%    0.0%    0.0%    0.0%    0.0%    0.0%    0.0%    0.0%    0.0%    0.0%    0.0%    0.0%    0.0%    0.0%    0.0%    0.0%    0.0%    0.0%    0.0%    0.0%    0.0%    0.0%    0.0%    0.0%    0.0%    0.0%    0.0%    0.0%    0.0%    0.0%    0.0%    0.0%    0.0%    0.0%    0.0%    0.0%    0.0%    0.0%    0.0%    0.0%    0.0%    0.0%    0.0%    0.0%    0.0%    0.0%    0.0%    0.0%    0.0%    0.0%    0.0%    0.0%    0.0%    0.0%    0.0%    0.0%    0.0%    0.0%    0.0%    0.0%    0.0%    0.0%    0.0%    0.0%    0.0%    0.0%    0.0%    0.0%    0.0%    0.0%    0.0%    0.0%    0.0%    0.0%    0.0%    0.0%    0.0%    0.0%    0.0%    0.0%    0.0%    0.0%    0.0%    0.0%    0.0%    0.0%    0.0%    0.0%    0.0%    0.0%    0.0%    0.0%    0.0%    0.0%    0.0%    0.0%    0.0%    0.0%    0.0%    0.0%    0.0%    0.0%    0.0%    0.0%    0.0%    0.0%    0.0%    0.0%    0.0%    0.0%    0.0%    0.0%    0.0%    0.0%    0.0%    0.0%    0.0%    0.0%    0.0%    0.0%    0.0%    0.0%    0.0%    0.0%    0.0%    0.0%    0.0%    0.0%    0.0%    0.0%    0.0%    0.0%    0.0%    0.0%    0.0%    0.0%    0.0%    0.0%    0.0%    0.0%    0.0%    0.0%    0.0%    0.0%    0.0%    0.0%    0.0%    0.0%    0.0%    0.0%    0.0%    0.0%    0.0%    0.0%    0.0%    0.0%    0.0%    0.0%    0.0%        k__Bacteria; p__SR1    1149    0.0%    0.0%    0.0%    0.0%    0.0%    0.0%    0.0%    0.0%    0.0%    0.0%    0.0%    0.0%    0.0%    0.0%    0.0%    0.0%    0.0%    0.0%    0.0%    0.0%    0.0%    0.0%    0.0%    0.0%    0.0%    0.0%    0.0%    0.0%    0.0%    0.0%    0.0%    0.0%    0.0%    0.0%    0.0%    0.0%    0.0%    0.0%    0.0%    0.0%    0.0%    0.0%    0.0%    0.0%    0.0%    0.0%    0.0%    0.0%    0.0%    0.0%    0.0%    0.0%    0.0%    0.0%    0.0%    0.0%    0.0%    0.0%    0.0%    0.0%    0.0%    0.0%    0.0%    0.0%    0.0%    0.0%    0.0%    0.0%    0.0%    0.0%    0.0%    0.0%    0.0%    0.0%    0.0%    0.0%    0.0%    0.0%    0.0%    0.0%    0.0%    0.0%    0.0%    0.0%    0.0%    0.0%    0.0%    0.0%    0.0%    0.0%    0.0%    0.0%    0.0%    0.0%    0.0%    0.0%    0.0%    0.0%    0.0%    0.0%    0.0%    0.0%    0.0%    0.0%    0.0%    0.0%    0.1%    0.0%    0.0%    0.0%    0.0%    0.0%    0.0%    0.0%    0.0%    0.0%    0.0%    0.0%    0.0%    0.0%    0.0%    0.0%    0.0%    0.0%    0.0%    0.0%    0.0%    0.0%    0.0%    0.0%    0.0%    0.0%    0.0%    0.0%    0.0%    0.0%    0.0%    0.0%    0.0%    0.0%    0.0%    0.0%    0.0%    0.0%    0.0%    0.0%    0.0%    0.0%    0.0%    0.0%    0.0%    0.0%    0.0%    0.0%    0.0%    0.0%    0.0%    0.0%    0.0%    0.0%    0.0%    0.0%    0.1%    0.0%    0.0%    0.0%    0.0%    0.0%    0.0%    0.0%    0.0%    0.0%    0.0%    0.0%    0.0%    0.0%    0.0%    0.0%    0.0%    0.0%    0.0%    0.0%    0.0%    0.0%    0.0%    0.0%    0.0%    0.0%    0.0%    0.0%    0.0%    0.0%    0.0%    0.0%    0.0%    0.0%    0.0%    0.0%    0.0%    0.0%    0.0%    0.0%    0.0%    0.0%    0.0%    0.0%    0.0%    0.0%    0.0%    0.0%    0.0%    0.0%    0.0%    0.0%    0.0%    0.0%    0.0%    0.0%    0.0%    0.0%    0.0%    0.0%    0.0%    0.0%    0.0%    0.0%    0.0%    0.0%    0.0%    0.0%    0.0%    0.0%    0.0%    0.0%    0.0%    0.0%    0.0%    0.0%    0.0%    0.0%    0.0%    0.0%    0.0%    0.0%    0.0%    0.0%    0.0%    0.0%    0.0%    0.0%    0.0%    0.0%    0.0%    0.0%    0.0%    0.0%    0.0%    0.0%    0.0%    0.0%    0.0%    0.0%    0.0%    0.0%    0.0%    0.0%    0.0%    0.0%    0.0%    0.0%    0.0%    0.0%    0.0%    0.0%    0.0%    0.0%    0.0%    0.0%    0.0%    0.0%    0.0%    0.0%    0.0%    0.0%    0.0%    0.0%    0.0%    0.0%    0.0%    0.0%    0.0%    0.0%    0.0%    0.0%    0.0%    0.0%    0.0%    0.1%    0.0%    0.0%    0.0%    0.0%    0.0%    0.0%    0.0%    0.0%    0.0%    0.0%    0.0%    0.0%    0.0%    0.0%    0.0%    0.0%    0.0%    0.0%    0.0%    0.0%    0.0%    0.0%    0.0%    0.0%    0.0%    0.0%    0.0%    0.0%    0.0%    0.0%    0.0%    0.0%    0.0%    0.0%    0.0%    0.0%    0.0%    0.0%    0.0%    0.0%    0.0%    0.0%    0.0%    0.0%    0.0%    0.0%    0.0%    0.0%    0.0%    0.0%    0.0%    0.0%    0.0%    0.0%    0.0%    0.0%    0.0%    0.0%    0.0%    0.0%        k__Bacteria; p__Spirochaetes    5992    0.0%    0.1%    0.0%    0.1%    0.1%    0.0%    0.0%    0.0%    0.0%    0.0%    0.0%    0.0%    0.0%    0.1%    0.1%    0.0%    0.1%    0.0%    0.1%    0.0%    0.0%    0.1%    0.0%    0.0%    0.0%    0.0%    0.0%    0.0%    0.1%    0.0%    0.0%    0.1%    0.0%    0.4%    0.2%    0.6%    0.1%    0.0%    0.0%    0.0%    0.0%    0.2%    0.0%    0.1%    0.0%    0.0%    0.0%    0.0%    0.0%    0.0%    0.0%    0.0%    0.0%    0.0%    0.1%    0.0%    0.1%    0.0%    0.0%    0.0%    0.0%    0.0%    0.1%    0.0%    0.0%    0.0%    0.0%    0.1%    0.0%    0.0%    0.9%    0.1%    0.0%    0.0%    0.0%    0.0%    0.0%    0.0%    0.0%    0.0%    0.0%    0.0%    0.0%    0.0%    0.0%    0.0%    0.2%    0.0%    0.0%    0.1%    0.0%    0.0%    0.1%    0.0%    0.0%    0.0%    0.0%    0.0%    0.0%    0.1%    0.0%    0.1%    0.0%    0.0%    0.0%    0.0%    0.1%    0.0%    0.0%    0.0%    0.0%    0.1%    0.1%    0.0%    0.0%    0.0%    0.0%    0.0%    0.0%    0.6%    0.0%    0.0%    0.0%    0.0%    0.0%    0.0%    0.0%    0.0%    0.0%    0.0%    0.0%    0.0%    0.0%    0.0%    0.0%    0.0%    0.0%    0.0%    0.0%    0.0%    0.0%    0.2%    0.0%    0.0%    0.0%    0.0%    0.3%    0.1%    0.1%    0.0%    0.0%    0.0%    0.0%    0.0%    0.0%    0.0%    0.0%    0.0%    0.0%    0.1%    0.0%    0.0%    0.0%    0.0%    0.0%    0.0%    0.0%    0.0%    0.0%    0.0%    0.0%    0.1%    0.0%    0.0%    0.0%    0.0%    0.0%    0.0%    0.0%    0.0%    0.0%    0.0%    0.1%    0.0%    0.0%    0.0%    0.0%    0.0%    0.0%    0.0%    0.0%    0.0%    0.0%    0.0%    0.0%    0.0%    0.0%    0.0%    0.0%    0.0%    0.1%    0.0%    0.0%    0.0%    0.0%    0.0%    0.0%    0.2%    0.0%    0.0%    0.0%    0.0%    0.0%    0.1%    0.0%    0.0%    0.0%    0.0%    0.0%    0.1%    0.0%    0.0%    0.0%    0.0%    0.0%    0.0%    0.0%    0.1%    0.0%    0.0%    0.0%    0.0%    0.0%    0.0%    0.0%    0.0%    0.0%    0.0%    0.0%    0.0%    0.0%    0.0%    0.0%    0.0%    0.0%    0.0%    0.0%    0.0%    0.0%    0.0%    0.0%    0.0%    0.0%    0.0%    0.0%    0.0%    0.0%    0.0%    0.0%    0.0%    0.0%    0.0%    0.0%    0.0%    0.0%    0.0%    0.0%    0.0%    0.0%    0.0%    0.0%    0.0%    0.0%    0.0%    0.0%    0.0%    0.0%    0.0%    0.0%    0.0%    0.0%    0.0%    0.0%    0.0%    0.0%    0.1%    0.0%    0.0%    0.0%    0.0%    0.0%    0.0%    0.1%    0.0%    0.0%    0.0%    0.0%    0.0%    0.0%    0.0%    0.0%    0.0%    0.0%    0.0%    0.0%    0.0%    0.0%    0.0%    0.0%    0.0%    0.0%    0.0%    0.0%    0.0%    0.0%    0.1%    0.0%    0.0%    0.0%    0.0%    0.0%    0.0%    0.0%    0.0%    0.0%    0.0%    0.0%    0.0%    0.0%    0.4%    0.0%    0.1%    0.0%    0.0%    0.0%    0.0%    0.0%    0.0%    0.0%    0.0%    0.0%    0.0%    0.0%    0.1%    0.0%    0.0%    0.0%    0.0%    0.0%    0.0%    0.0%    0.0%    0.0%    0.1%    0.0%    0.0%    0.0%    0.0%        k__Bacteria; p__Synergistetes    1745    0.0%    0.0%    0.0%    0.0%    0.0%    0.0%    0.0%    0.0%    0.0%    0.0%    0.0%    0.0%    0.0%    0.0%    0.0%    0.0%    0.0%    0.0%    0.0%    0.0%    0.0%    0.0%    0.0%    0.0%    0.0%    0.0%    0.0%    0.0%    0.0%    0.0%    0.0%    0.0%    0.0%    0.1%    0.0%    0.2%    0.0%    0.0%    0.0%    0.0%    0.0%    0.0%    0.0%    0.0%    0.0%    0.0%    0.0%    0.0%    0.0%    0.0%    0.0%    0.0%    0.0%    0.0%    0.0%    0.0%    0.0%    0.0%    0.0%    0.0%    0.0%    0.0%    0.0%    0.0%    0.0%    0.0%    0.0%    0.0%    0.0%    0.0%    0.1%    0.0%    0.0%    0.0%    0.0%    0.0%    0.0%    0.0%    0.0%    0.0%    0.0%    0.0%    0.0%    0.0%    0.0%    0.0%    0.0%    0.0%    0.0%    0.0%    0.0%    0.0%    0.0%    0.0%    0.0%    0.0%    0.0%    0.0%    0.0%    0.0%    0.0%    0.0%    0.0%    0.0%    0.0%    0.0%    0.0%    0.0%    0.0%    0.0%    0.0%    0.0%    0.0%    0.0%    0.0%    0.0%    0.0%    0.0%    0.0%    0.1%    0.0%    0.0%    0.0%    0.0%    0.0%    0.0%    0.0%    0.0%    0.0%    0.0%    0.0%    0.0%    0.0%    0.0%    0.0%    0.0%    0.0%    0.0%    0.0%    0.0%    0.0%    0.1%    0.0%    0.0%    0.0%    0.0%    0.3%    0.1%    0.0%    0.0%    0.0%    0.0%    0.0%    0.0%    0.0%    0.0%    0.0%    0.0%    0.0%    0.0%    0.0%    0.0%    0.0%    0.0%    0.0%    0.0%    0.0%    0.0%    0.0%    0.0%    0.0%    0.0%    0.0%    0.0%    0.0%    0.0%    0.0%    0.0%    0.0%    0.0%    0.0%    0.0%    0.0%    0.0%    0.0%    0.0%    0.0%    0.1%    0.0%    0.0%    0.0%    0.1%    0.0%    0.0%    0.0%    0.0%    0.0%    0.0%    0.0%    0.0%    0.0%    0.0%    0.0%    0.0%    0.0%    0.0%    0.0%    0.1%    0.0%    0.0%    0.0%    0.0%    0.0%    0.1%    0.0%    0.0%    0.0%    0.0%    0.0%    0.0%    0.0%    0.0%    0.0%    0.0%    0.0%    0.0%    0.0%    0.0%    0.0%    0.0%    0.0%    0.0%    0.0%    0.0%    0.0%    0.0%    0.0%    0.0%    0.0%    0.0%    0.0%    0.0%    0.0%    0.0%    0.0%    0.0%    0.0%    0.0%    0.0%    0.0%    0.0%    0.0%    0.0%    0.0%    0.0%    0.0%    0.0%    0.0%    0.0%    0.0%    0.0%    0.0%    0.0%    0.0%    0.1%    0.0%    0.0%    0.0%    0.0%    0.0%    0.0%    0.0%    0.0%    0.0%    0.0%    0.0%    0.0%    0.0%    0.0%    0.0%    0.0%    0.0%    0.0%    0.0%    0.0%    0.0%    0.0%    0.0%    0.0%    0.0%    0.0%    0.0%    0.0%    0.0%    0.0%    0.0%    0.0%    0.0%    0.1%    0.0%    0.0%    0.0%    0.0%    0.0%    0.0%    0.0%    0.0%    0.0%    0.0%    0.0%    0.0%    0.0%    0.0%    0.0%    0.0%    0.0%    0.0%    0.0%    0.0%    0.0%    0.0%    0.0%    0.0%    0.0%    0.0%    0.0%    0.0%    0.0%    0.0%    0.1%    0.0%    0.0%    0.0%    0.0%    0.0%    0.0%    0.0%    0.2%    0.0%    0.0%    0.0%    0.0%    0.0%    0.0%    0.0%    0.0%    0.0%    0.0%    0.0%    0.0%    0.0%    0.0%    0.0%    0.0%    0.0%    0.0%    0.0%    0.0%        k__Bacteria; p__TM6       4    0.0%    0.0%    0.0%    0.0%    0.0%    0.0%    0.0%    0.0%    0.0%    0.0%    0.0%    0.0%    0.0%    0.0%    0.0%    0.0%    0.0%    0.0%    0.0%    0.0%    0.0%    0.0%    0.0%    0.0%    0.0%    0.0%    0.0%    0.0%    0.0%    0.0%    0.0%    0.0%    0.0%    0.0%    0.0%    0.0%    0.0%    0.0%    0.0%    0.0%    0.0%    0.0%    0.0%    0.0%    0.0%    0.0%    0.0%    0.0%    0.0%    0.0%    0.0%    0.0%    0.0%    0.0%    0.0%    0.0%    0.0%    0.0%    0.0%    0.0%    0.0%    0.0%    0.0%    0.0%    0.0%    0.0%    0.0%    0.0%    0.0%    0.0%    0.0%    0.0%    0.0%    0.0%    0.0%    0.0%    0.0%    0.0%    0.0%    0.0%    0.0%    0.0%    0.0%    0.0%    0.0%    0.0%    0.0%    0.0%    0.0%    0.0%    0.0%    0.0%    0.0%    0.0%    0.0%    0.0%    0.0%    0.0%    0.0%    0.0%    0.0%    0.0%    0.0%    0.0%    0.0%    0.0%    0.0%    0.0%    0.0%    0.0%    0.0%    0.0%    0.0%    0.0%    0.0%    0.0%    0.0%    0.0%    0.0%    0.0%    0.0%    0.0%    0.0%    0.0%    0.0%    0.0%    0.0%    0.0%    0.0%    0.0%    0.0%    0.0%    0.0%    0.0%    0.0%    0.0%    0.0%    0.0%    0.0%    0.0%    0.0%    0.0%    0.0%    0.0%    0.0%    0.0%    0.0%    0.0%    0.0%    0.0%    0.0%    0.0%    0.0%    0.0%    0.0%    0.0%    0.0%    0.0%    0.0%    0.0%    0.0%    0.0%    0.0%    0.0%    0.0%    0.0%    0.0%    0.0%    0.0%    0.0%    0.0%    0.0%    0.0%    0.0%    0.0%    0.0%    0.0%    0.0%    0.0%    0.0%    0.0%    0.0%    0.0%    0.0%    0.0%    0.0%    0.0%    0.0%    0.0%    0.0%    0.0%    0.0%    0.0%    0.0%    0.0%    0.0%    0.0%    0.0%    0.0%    0.0%    0.0%    0.0%    0.0%    0.0%    0.0%    0.0%    0.0%    0.0%    0.0%    0.0%    0.0%    0.0%    0.0%    0.0%    0.0%    0.0%    0.0%    0.0%    0.0%    0.0%    0.0%    0.0%    0.0%    0.0%    0.0%    0.0%    0.0%    0.0%    0.0%    0.0%    0.0%    0.0%    0.0%    0.0%    0.0%    0.0%    0.0%    0.0%    0.0%    0.0%    0.0%    0.0%    0.0%    0.0%    0.0%    0.0%    0.0%    0.0%    0.0%    0.0%    0.0%    0.0%    0.0%    0.0%    0.0%    0.0%    0.0%    0.0%    0.0%    0.0%    0.0%    0.0%    0.0%    0.0%    0.0%    0.0%    0.0%    0.0%    0.0%    0.0%    0.0%    0.0%    0.0%    0.0%    0.0%    0.0%    0.0%    0.0%    0.0%    0.0%    0.0%    0.0%    0.0%    0.0%    0.0%    0.0%    0.0%    0.0%    0.0%    0.0%    0.0%    0.0%    0.0%    0.0%    0.0%    0.0%    0.0%    0.0%    0.0%    0.0%    0.0%    0.0%    0.0%    0.0%    0.0%    0.0%    0.0%    0.0%    0.0%    0.0%    0.0%    0.0%    0.0%    0.0%    0.0%    0.0%    0.0%    0.0%    0.0%    0.0%    0.0%    0.0%    0.0%    0.0%    0.0%    0.0%    0.0%    0.0%    0.0%    0.0%    0.0%    0.0%    0.0%    0.0%    0.0%    0.0%    0.0%    0.0%    0.0%    0.0%    0.0%    0.0%    0.0%    0.0%    0.0%    0.0%    0.0%    0.0%    0.0%    0.0%    0.0%    0.0%    0.0%    0.0%    0.0%    0.0%    0.0%    0.0%        k__Bacteria; p__TM7     363    0.0%    0.0%    0.0%    0.0%    0.0%    0.0%    0.0%    0.0%    0.0%    0.0%    0.0%    0.0%    0.0%    0.0%    0.0%    0.0%    0.0%    0.0%    0.0%    0.0%    0.0%    0.0%    0.0%    0.0%    0.0%    0.0%    0.0%    0.0%    0.0%    0.0%    0.0%    0.0%    0.0%    0.0%    0.0%    0.0%    0.0%    0.0%    0.0%    0.0%    0.0%    0.0%    0.0%    0.0%    0.0%    0.0%    0.0%    0.0%    0.0%    0.0%    0.0%    0.0%    0.0%    0.0%    0.0%    0.0%    0.0%    0.0%    0.0%    0.0%    0.0%    0.0%    0.0%    0.0%    0.0%    0.0%    0.0%    0.0%    0.0%    0.0%    0.0%    0.0%    0.0%    0.0%    0.0%    0.0%    0.0%    0.0%    0.0%    0.0%    0.0%    0.0%    0.0%    0.0%    0.0%    0.0%    0.0%    0.0%    0.0%    0.0%    0.0%    0.0%    0.0%    0.0%    0.0%    0.0%    0.0%    0.0%    0.0%    0.0%    0.0%    0.0%    0.0%    0.0%    0.0%    0.0%    0.0%    0.0%    0.0%    0.0%    0.0%    0.0%    0.0%    0.0%    0.0%    0.0%    0.0%    0.0%    0.0%    0.0%    0.0%    0.0%    0.0%    0.0%    0.0%    0.0%    0.0%    0.0%    0.0%    0.0%    0.0%    0.0%    0.0%    0.0%    0.0%    0.0%    0.0%    0.0%    0.0%    0.0%    0.0%    0.0%    0.0%    0.0%    0.0%    0.0%    0.0%    0.0%    0.0%    0.0%    0.0%    0.0%    0.0%    0.0%    0.0%    0.0%    0.0%    0.0%    0.0%    0.0%    0.0%    0.0%    0.0%    0.0%    0.0%    0.0%    0.0%    0.0%    0.0%    0.0%    0.0%    0.0%    0.0%    0.0%    0.0%    0.0%    0.0%    0.0%    0.0%    0.0%    0.0%    0.0%    0.0%    0.0%    0.0%    0.0%    0.0%    0.0%    0.0%    0.0%    0.0%    0.0%    0.0%    0.0%    0.0%    0.0%    0.0%    0.0%    0.0%    0.0%    0.0%    0.0%    0.0%    0.0%    0.0%    0.0%    0.0%    0.0%    0.0%    0.0%    0.0%    0.0%    0.0%    0.0%    0.0%    0.0%    0.0%    0.0%    0.0%    0.0%    0.0%    0.0%    0.0%    0.0%    0.0%    0.0%    0.0%    0.0%    0.0%    0.0%    0.0%    0.0%    0.0%    0.0%    0.0%    0.0%    0.0%    0.0%    0.0%    0.0%    0.0%    0.0%    0.0%    0.0%    0.0%    0.0%    0.0%    0.0%    0.0%    0.0%    0.0%    0.0%    0.0%    0.0%    0.0%    0.0%    0.0%    0.0%    0.0%    0.0%    0.0%    0.0%    0.0%    0.0%    0.0%    0.0%    0.0%    0.0%    0.0%    0.0%    0.0%    0.0%    0.0%    0.0%    0.0%    0.0%    0.0%    0.0%    0.0%    0.0%    0.0%    0.0%    0.0%    0.0%    0.0%    0.0%    0.0%    0.0%    0.0%    0.0%    0.0%    0.0%    0.0%    0.0%    0.0%    0.0%    0.0%    0.0%    0.0%    0.0%    0.0%    0.0%    0.0%    0.0%    0.0%    0.0%    0.0%    0.0%    0.0%    0.0%    0.0%    0.0%    0.0%    0.0%    0.0%    0.0%    0.0%    0.0%    0.0%    0.0%    0.0%    0.0%    0.0%    0.0%    0.0%    0.0%    0.0%    0.0%    0.0%    0.0%    0.0%    0.0%    0.0%    0.0%    0.0%    0.0%    0.0%    0.0%    0.0%    0.0%    0.0%    0.0%    0.0%    0.0%    0.0%    0.0%    0.0%    0.0%    0.0%    0.0%    0.0%    0.0%    0.0%    0.0%    0.0%    0.0%    0.0%    0.0%        k__Bacteria; p__Tenericutes   56361    0.3%    0.2%    0.3%    1.2%    0.2%    0.0%    0.0%    0.0%    0.0%    0.0%    0.0%    0.0%    0.9%    0.2%    0.0%    0.0%    0.4%    0.7%    0.1%    0.1%    0.7%    1.1%    0.0%    0.0%    0.0%    0.2%    0.2%    0.8%    1.5%    0.1%    0.1%    0.3%    0.0%    0.2%    1.7%    0.1%    0.9%    0.1%    0.4%    0.6%    3.2%    1.4%    0.1%    0.2%    1.0%    1.1%    0.1%    0.0%    0.2%    0.2%    0.3%    0.1%    0.0%    0.1%    0.6%    0.3%    0.2%    0.1%    0.7%    0.2%    0.2%    0.3%    0.2%    0.8%    2.6%    0.2%    0.1%    0.8%    0.2%    0.6%    0.1%    0.1%    0.1%    0.3%    0.4%    0.0%    0.1%    0.5%    0.2%    1.4%    0.2%    0.1%    0.6%    0.5%    0.1%    0.0%    0.6%    0.0%    0.2%    0.6%    0.0%    0.1%    0.2%    0.2%    0.2%    0.1%    0.2%    0.0%    2.7%    2.4%    0.5%    0.2%    0.1%    0.1%    0.0%    0.0%    1.4%    0.0%    0.0%    0.3%    0.1%    1.8%    0.1%    0.0%    0.5%    0.2%    0.1%    0.1%    0.3%    0.1%    0.0%    0.3%    0.6%    0.1%    0.0%    0.4%    0.2%    0.0%    0.4%    1.3%    0.5%    0.1%    0.0%    0.1%    1.4%    0.1%    0.2%    0.1%    0.0%    0.1%    0.0%    0.3%    0.7%    0.1%    0.1%    0.2%    0.1%    0.0%    0.1%    0.0%    0.1%    0.2%    0.7%    0.3%    0.1%    0.0%    1.1%    0.1%    0.1%    1.0%    1.5%    1.0%    1.1%    0.2%    0.3%    0.2%    0.8%    0.1%    0.2%    0.1%    0.8%    1.1%    0.2%    0.6%    0.0%    0.0%    0.1%    0.2%    0.1%    0.0%    0.0%    0.0%    0.4%    0.1%    0.1%    0.1%    0.0%    0.0%    0.0%    0.1%    0.0%    0.1%    0.0%    0.1%    0.1%    0.2%    0.4%    0.1%    0.0%    0.0%    1.2%    2.2%    0.2%    0.6%    0.6%    0.4%    0.2%    0.3%    1.0%    0.7%    0.7%    0.1%    0.0%    0.2%    0.1%    0.1%    0.2%    0.2%    0.0%    0.1%    0.3%    0.2%    0.0%    0.3%    0.0%    0.3%    0.1%    0.1%    0.3%    0.2%    0.1%    0.0%    0.1%    0.1%    0.4%    0.0%    0.2%    0.0%    0.0%    0.0%    0.0%    0.0%    0.1%    0.1%    0.0%    0.0%    0.0%    0.0%    0.0%    0.5%    0.5%    0.2%    0.2%    0.1%    0.1%    0.0%    0.2%    0.1%    1.6%    0.3%    0.8%    0.4%    0.9%    0.3%    0.2%    0.0%    0.2%    0.3%    0.2%    0.0%    0.2%    0.1%    0.2%    0.2%    0.1%    0.1%    0.0%    0.1%    0.1%    0.0%    0.0%    0.0%    0.0%    0.0%    0.1%    0.0%    0.0%    0.3%    0.0%    0.0%    0.0%    0.0%    0.1%    0.2%    0.0%    0.0%    0.3%    0.2%    0.1%    0.1%    0.3%    0.1%    0.3%    0.4%    0.4%    0.1%    0.2%    0.1%    0.1%    0.1%    0.0%    0.0%    0.0%    0.0%    0.0%    0.4%    0.0%    0.0%    0.0%    0.1%    0.2%    0.1%    0.0%    0.0%    0.1%    0.0%    0.1%    0.0%    0.1%    0.0%    0.0%    0.0%    0.1%    0.0%    0.0%    0.0%    0.0%    0.1%    0.1%    0.0%    0.0%    0.0%    0.0%    0.1%    0.0%    0.0%    0.0%    0.1%    0.0%    0.1%    0.0%    0.1%    0.0%    0.2%    0.0%    0.0%    0.0%    0.0%        k__Bacteria; p__Thermi    8498    0.0%    0.0%    0.0%    0.0%    0.0%    0.0%    0.0%    0.6%    0.0%    0.0%    0.0%    0.5%    0.0%    0.0%    0.0%    0.0%    0.0%    0.1%    0.0%    0.0%    0.0%    0.1%    0.0%    0.0%    0.0%    0.0%    0.0%    0.0%    0.0%    0.0%    0.0%    0.0%    0.0%    0.0%    0.0%    0.0%    0.0%    0.0%    0.0%    0.0%    0.0%    0.0%    0.0%    0.0%    0.0%    0.0%    0.0%    0.0%    0.0%    0.0%    0.0%    0.0%    0.0%    0.0%    0.0%    0.0%    0.0%    0.0%    0.0%    0.0%    0.0%    0.1%    0.0%    0.0%    0.0%    0.1%    0.0%    0.1%    0.0%    0.0%    0.0%    0.0%    0.0%    0.0%    0.1%    0.0%    0.0%    0.0%    0.0%    0.0%    0.0%    0.1%    0.0%    0.0%    0.0%    0.0%    0.0%    0.0%    0.0%    0.0%    0.0%    0.0%    0.0%    0.0%    0.0%    0.1%    0.0%    0.0%    0.0%    0.0%    0.0%    0.0%    0.0%    0.4%    0.1%    0.1%    0.1%    0.0%    0.0%    0.4%    0.1%    0.2%    0.0%    0.0%    0.1%    0.0%    0.0%    0.0%    0.1%    0.1%    0.0%    0.1%    0.1%    0.2%    0.0%    0.1%    0.1%    0.0%    0.1%    0.1%    0.1%    0.0%    0.0%    0.1%    0.2%    0.0%    0.0%    0.0%    0.0%    0.0%    0.0%    0.0%    0.0%    0.0%    0.0%    0.0%    0.0%    0.0%    0.0%    0.0%    0.0%    0.0%    0.1%    0.0%    0.1%    0.0%    0.2%    0.0%    0.0%    0.1%    0.1%    0.2%    0.1%    0.0%    0.0%    0.0%    0.1%    0.2%    0.3%    0.0%    0.4%    0.2%    0.1%    2.1%    0.1%    0.0%    0.0%    0.0%    0.1%    0.1%    0.3%    0.2%    0.0%    0.0%    0.0%    0.1%    0.2%    0.0%    0.0%    0.1%    0.0%    0.1%    0.0%    0.0%    0.0%    0.0%    0.1%    0.0%    0.1%    0.1%    0.1%    0.0%    0.0%    0.1%    0.1%    0.0%    0.0%    0.0%    0.1%    0.0%    0.0%    0.1%    0.0%    0.1%    0.0%    0.1%    0.0%    0.0%    0.0%    0.2%    0.0%    0.0%    0.0%    0.0%    0.0%    0.0%    0.0%    0.0%    0.0%    0.0%    0.0%    0.0%    0.0%    0.0%    0.1%    0.0%    0.0%    0.1%    0.0%    0.0%    0.0%    0.0%    0.0%    0.0%    0.0%    0.0%    0.0%    0.0%    0.0%    0.0%    0.1%    0.0%    0.0%    0.0%    0.0%    0.0%    0.0%    0.0%    0.0%    0.0%    0.0%    0.0%    0.1%    0.0%    0.0%    0.0%    0.0%    0.0%    0.0%    0.0%    0.0%    0.0%    0.2%    0.1%    0.0%    0.0%    0.0%    0.0%    0.0%    0.0%    0.0%    0.0%    0.0%    0.0%    0.1%    0.0%    0.0%    0.3%    0.1%    0.0%    0.0%    0.0%    0.2%    0.1%    0.1%    0.0%    0.1%    0.0%    0.0%    0.1%    0.0%    0.0%    0.0%    0.0%    0.0%    0.0%    0.1%    0.0%    0.0%    0.0%    0.0%    0.0%    0.0%    0.0%    0.0%    0.1%    0.1%    0.1%    0.0%    0.0%    0.0%    0.1%    0.0%    0.0%    0.1%    0.0%    0.0%    0.0%    0.1%    0.1%    0.0%    0.0%    0.0%    0.1%    0.0%    0.0%    0.0%    0.0%    0.0%    0.0%    0.1%    0.0%    0.0%    0.0%    0.1%    0.0%    0.0%    0.0%    0.0%    0.0%    0.1%    0.0%    0.0%    0.2%    0.0%    0.0%    0.0%    0.0%        k__Bacteria; p__Thermotogae     106    0.0%    0.0%    0.0%    0.0%    0.0%    0.0%    0.0%    0.0%    0.0%    0.0%    0.0%    0.0%    0.0%    0.0%    0.0%    0.0%    0.0%    0.0%    0.0%    0.0%    0.0%    0.0%    0.0%    0.0%    0.0%    0.0%    0.0%    0.0%    0.0%    0.0%    0.0%    0.0%    0.0%    0.0%    0.0%    0.0%    0.0%    0.0%    0.0%    0.0%    0.0%    0.0%    0.0%    0.0%    0.0%    0.0%    0.0%    0.0%    0.0%    0.0%    0.0%    0.1%    0.0%    0.0%    0.0%    0.0%    0.0%    0.0%    0.0%    0.0%    0.0%    0.0%    0.0%    0.0%    0.0%    0.0%    0.0%    0.0%    0.0%    0.0%    0.0%    0.0%    0.0%    0.0%    0.0%    0.0%    0.0%    0.0%    0.0%    0.0%    0.0%    0.0%    0.0%    0.0%    0.0%    0.0%    0.0%    0.0%    0.0%    0.0%    0.0%    0.0%    0.0%    0.0%    0.0%    0.0%    0.0%    0.0%    0.0%    0.0%    0.0%    0.0%    0.0%    0.0%    0.0%    0.0%    0.0%    0.0%    0.0%    0.0%    0.0%    0.0%    0.0%    0.0%    0.0%    0.0%    0.0%    0.0%    0.0%    0.0%    0.0%    0.0%    0.0%    0.0%    0.0%    0.0%    0.0%    0.0%    0.0%    0.0%    0.0%    0.0%    0.0%    0.0%    0.0%    0.0%    0.0%    0.0%    0.0%    0.0%    0.0%    0.0%    0.0%    0.0%    0.0%    0.0%    0.0%    0.0%    0.0%    0.0%    0.0%    0.0%    0.0%    0.0%    0.0%    0.0%    0.0%    0.0%    0.0%    0.0%    0.0%    0.0%    0.0%    0.0%    0.0%    0.0%    0.0%    0.0%    0.0%    0.0%    0.0%    0.0%    0.0%    0.0%    0.0%    0.0%    0.0%    0.0%    0.0%    0.0%    0.0%    0.0%    0.0%    0.0%    0.0%    0.0%    0.0%    0.0%    0.0%    0.0%    0.0%    0.0%    0.0%    0.0%    0.0%    0.0%    0.0%    0.0%    0.0%    0.0%    0.0%    0.0%    0.0%    0.0%    0.0%    0.0%    0.0%    0.0%    0.0%    0.0%    0.0%    0.0%    0.0%    0.0%    0.0%    0.0%    0.0%    0.0%    0.0%    0.0%    0.0%    0.0%    0.0%    0.0%    0.0%    0.0%    0.0%    0.0%    0.0%    0.0%    0.0%    0.0%    0.0%    0.0%    0.0%    0.0%    0.0%    0.0%    0.0%    0.0%    0.0%    0.0%    0.0%    0.0%    0.0%    0.0%    0.0%    0.0%    0.0%    0.0%    0.0%    0.0%    0.0%    0.0%    0.0%    0.0%    0.0%    0.0%    0.0%    0.0%    0.0%    0.0%    0.0%    0.0%    0.0%    0.0%    0.0%    0.0%    0.0%    0.0%    0.0%    0.0%    0.0%    0.0%    0.0%    0.0%    0.0%    0.0%    0.0%    0.0%    0.0%    0.0%    0.0%    0.0%    0.0%    0.0%    0.0%    0.0%    0.0%    0.0%    0.0%    0.0%    0.0%    0.0%    0.0%    0.0%    0.0%    0.0%    0.0%    0.0%    0.0%    0.0%    0.0%    0.0%    0.0%    0.0%    0.0%    0.0%    0.0%    0.0%    0.0%    0.0%    0.0%    0.0%    0.0%    0.0%    0.0%    0.0%    0.0%    0.0%    0.0%    0.0%    0.0%    0.0%    0.0%    0.0%    0.0%    0.0%    0.0%    0.0%    0.0%    0.0%    0.0%    0.0%    0.0%    0.0%    0.0%    0.0%    0.0%    0.0%    0.0%    0.0%    0.0%    0.0%    0.0%    0.0%    0.0%    0.0%    0.0%    0.0%    0.0%    0.0%    0.0%    0.0%    0.0%    0.0%    0.0%    0.0%        k__Bacteria; p__Verrucomicrobia    8014    0.0%    0.0%    0.1%    0.0%    0.0%    0.0%    0.0%    0.0%    0.0%    0.0%    0.0%    0.1%    0.0%    0.0%    0.0%    0.0%    0.0%    0.0%    0.0%    0.0%    0.1%    0.0%    0.0%    0.4%    0.0%    0.0%    0.0%    0.0%    0.1%    0.0%    0.0%    0.0%    0.0%    0.0%    0.1%    0.0%    0.2%    0.0%    0.0%    0.0%    0.0%    0.1%    0.0%    0.0%    0.0%    0.0%    0.0%    0.0%    0.0%    0.0%    0.0%    0.0%    0.0%    0.0%    0.1%    0.1%    0.1%    0.0%    0.0%    0.1%    0.0%    0.0%    0.0%    0.1%    0.1%    0.1%    0.1%    0.1%    0.0%    0.0%    0.0%    0.0%    0.0%    0.0%    0.1%    0.0%    0.0%    0.1%    0.0%    0.0%    0.0%    0.0%    0.0%    0.0%    0.0%    0.0%    0.0%    0.0%    0.0%    0.1%    0.0%    0.0%    0.0%    0.0%    0.0%    0.0%    0.1%    0.0%    0.1%    0.1%    0.1%    0.1%    0.0%    0.0%    0.0%    0.0%    0.2%    0.0%    0.0%    0.0%    0.0%    0.1%    0.0%    0.0%    0.0%    0.0%    0.0%    0.0%    0.0%    0.7%    0.0%    0.0%    0.1%    0.0%    0.0%    0.8%    0.2%    0.0%    0.2%    0.3%    0.1%    0.0%    0.1%    0.0%    0.2%    0.0%    0.0%    0.0%    0.0%    0.0%    0.0%    0.0%    0.1%    0.0%    0.1%    0.0%    0.0%    0.0%    0.0%    0.0%    0.0%    0.0%    0.1%    0.0%    0.0%    0.0%    0.1%    0.0%    0.0%    0.1%    0.1%    0.1%    0.1%    0.0%    0.1%    0.0%    0.1%    0.0%    0.0%    0.0%    0.2%    0.0%    0.1%    0.0%    0.0%    0.0%    0.0%    0.0%    0.0%    0.0%    0.0%    0.0%    0.0%    0.0%    0.0%    0.0%    0.0%    0.0%    0.0%    0.0%    0.0%    0.0%    0.0%    0.0%    0.0%    0.1%    0.1%    0.0%    0.0%    0.1%    0.1%    0.0%    0.0%    0.1%    0.1%    0.0%    0.1%    0.1%    0.3%    0.1%    0.1%    0.0%    0.3%    0.0%    0.0%    0.1%    0.0%    0.0%    0.0%    0.0%    0.0%    0.0%    0.0%    0.1%    0.0%    0.1%    0.0%    0.0%    0.1%    0.0%    0.0%    0.0%    0.1%    0.0%    0.1%    0.0%    0.1%    0.0%    0.0%    0.0%    0.1%    0.0%    0.0%    0.0%    0.0%    0.0%    0.1%    0.0%    0.0%    0.0%    0.1%    0.1%    0.1%    0.0%    0.0%    0.0%    0.0%    0.0%    0.0%    0.0%    0.1%    0.0%    0.1%    0.0%    0.0%    0.0%    0.0%    0.0%    0.0%    0.0%    0.1%    0.1%    0.1%    0.1%    0.0%    0.0%    0.0%    0.0%    0.0%    0.0%    0.0%    0.0%    0.0%    0.0%    0.0%    0.0%    0.0%    0.4%    0.0%    0.2%    0.0%    0.0%    0.0%    0.1%    0.0%    0.0%    0.1%    0.1%    0.0%    0.1%    0.0%    0.0%    0.0%    0.0%    0.0%    0.0%    0.0%    0.0%    0.0%    0.0%    0.0%    0.1%    0.0%    0.0%    0.0%    0.1%    0.0%    0.0%    0.0%    0.1%    0.0%    0.0%    0.0%    0.0%    0.0%    0.0%    0.1%    0.0%    0.0%    0.0%    0.1%    0.0%    0.0%    0.0%    0.1%    0.0%    0.0%    0.0%    0.1%    0.0%    0.0%    0.0%    0.0%    0.0%    0.0%    0.0%    0.0%    0.0%    0.0%    0.0%    0.0%    0.0%    0.0%    0.0%    0.0%    0.2%    0.5%    0.0%        k__Bacteria; p__WPS-2     101    0.0%    0.0%    0.0%    0.0%    0.0%    0.0%    0.0%    0.0%    0.0%    0.0%    0.0%    0.0%    0.0%    0.0%    0.0%    0.0%    0.0%    0.0%    0.0%    0.0%    0.0%    0.0%    0.0%    0.0%    0.0%    0.0%    0.0%    0.0%    0.0%    0.0%    0.0%    0.0%    0.0%    0.0%    0.0%    0.0%    0.0%    0.0%    0.0%    0.0%    0.0%    0.0%    0.0%    0.0%    0.0%    0.0%    0.0%    0.0%    0.0%    0.0%    0.0%    0.0%    0.0%    0.0%    0.0%    0.0%    0.0%    0.0%    0.0%    0.0%    0.0%    0.0%    0.0%    0.0%    0.0%    0.0%    0.0%    0.0%    0.0%    0.0%    0.0%    0.0%    0.0%    0.0%    0.0%    0.0%    0.0%    0.0%    0.0%    0.0%    0.0%    0.0%    0.0%    0.0%    0.0%    0.0%    0.0%    0.0%    0.0%    0.0%    0.0%    0.0%    0.0%    0.0%    0.0%    0.0%    0.0%    0.0%    0.0%    0.0%    0.0%    0.0%    0.0%    0.0%    0.0%    0.0%    0.0%    0.0%    0.0%    0.0%    0.0%    0.0%    0.0%    0.0%    0.0%    0.0%    0.0%    0.0%    0.0%    0.0%    0.0%    0.0%    0.0%    0.0%    0.0%    0.0%    0.0%    0.0%    0.0%    0.0%    0.0%    0.0%    0.0%    0.0%    0.0%    0.0%    0.0%    0.0%    0.0%    0.0%    0.0%    0.0%    0.0%    0.0%    0.0%    0.0%    0.0%    0.0%    0.0%    0.0%    0.0%    0.0%    0.0%    0.0%    0.0%    0.0%    0.0%    0.0%    0.0%    0.0%    0.0%    0.0%    0.0%    0.0%    0.0%    0.0%    0.0%    0.0%    0.0%    0.0%    0.0%    0.0%    0.0%    0.0%    0.0%    0.0%    0.0%    0.0%    0.0%    0.0%    0.0%    0.0%    0.0%    0.0%    0.0%    0.0%    0.0%    0.0%    0.0%    0.0%    0.0%    0.0%    0.0%    0.0%    0.0%    0.0%    0.0%    0.0%    0.0%    0.0%    0.0%    0.0%    0.0%    0.0%    0.0%    0.0%    0.0%    0.0%    0.0%    0.0%    0.0%    0.0%    0.0%    0.0%    0.0%    0.0%    0.0%    0.0%    0.0%    0.0%    0.0%    0.0%    0.0%    0.0%    0.0%    0.0%    0.0%    0.0%    0.0%    0.0%    0.0%    0.0%    0.0%    0.0%    0.0%    0.0%    0.0%    0.0%    0.0%    0.0%    0.0%    0.0%    0.0%    0.0%    0.0%    0.0%    0.0%    0.0%    0.0%    0.0%    0.0%    0.0%    0.0%    0.0%    0.0%    0.0%    0.0%    0.0%    0.0%    0.0%    0.0%    0.0%    0.0%    0.0%    0.0%    0.0%    0.0%    0.0%    0.0%    0.0%    0.0%    0.0%    0.0%    0.0%    0.0%    0.0%    0.0%    0.0%    0.0%    0.0%    0.0%    0.0%    0.0%    0.0%    0.0%    0.0%    0.0%    0.0%    0.0%    0.0%    0.0%    0.0%    0.0%    0.0%    0.0%    0.0%    0.0%    0.0%    0.0%    0.0%    0.0%    0.0%    0.0%    0.0%    0.0%    0.0%    0.0%    0.0%    0.0%    0.0%    0.0%    0.0%    0.0%    0.0%    0.0%    0.0%    0.0%    0.0%    0.0%    0.0%    0.0%    0.0%    0.0%    0.0%    0.0%    0.0%    0.0%    0.0%    0.0%    0.0%    0.0%    0.0%    0.0%    0.0%    0.0%    0.0%    0.0%    0.0%    0.0%    0.0%    0.0%    0.0%    0.0%    0.0%    0.0%    0.0%    0.0%    0.0%    0.0%    0.0%    0.0%    0.0%    0.0%    0.0%    0.0%    0.0%    0.0%    0.0%        k__Bacteria; p__WS3      42    0.0%    0.0%    0.0%    0.0%    0.0%    0.0%    0.0%    0.0%    0.0%    0.0%    0.0%    0.0%    0.0%    0.0%    0.0%    0.0%    0.0%    0.0%    0.0%    0.0%    0.0%    0.0%    0.0%    0.0%    0.0%    0.0%    0.0%    0.0%    0.0%    0.0%    0.0%    0.0%    0.0%    0.0%    0.0%    0.0%    0.0%    0.0%    0.0%    0.0%    0.0%    0.0%    0.0%    0.0%    0.0%    0.0%    0.0%    0.0%    0.0%    0.0%    0.0%    0.0%    0.0%    0.0%    0.0%    0.0%    0.0%    0.0%    0.0%    0.0%    0.0%    0.0%    0.0%    0.0%    0.0%    0.0%    0.0%    0.0%    0.0%    0.0%    0.0%    0.0%    0.0%    0.0%    0.0%    0.0%    0.0%    0.0%    0.0%    0.0%    0.0%    0.0%    0.0%    0.0%    0.0%    0.0%    0.0%    0.0%    0.0%    0.0%    0.0%    0.0%    0.0%    0.0%    0.0%    0.0%    0.0%    0.0%    0.0%    0.0%    0.0%    0.0%    0.0%    0.0%    0.0%    0.0%    0.0%    0.0%    0.0%    0.0%    0.0%    0.0%    0.0%    0.0%    0.0%    0.0%    0.0%    0.0%    0.0%    0.0%    0.0%    0.0%    0.0%    0.0%    0.0%    0.0%    0.0%    0.0%    0.0%    0.0%    0.0%    0.0%    0.0%    0.0%    0.0%    0.0%    0.0%    0.0%    0.0%    0.0%    0.0%    0.0%    0.0%    0.0%    0.0%    0.0%    0.0%    0.0%    0.0%    0.0%    0.0%    0.0%    0.0%    0.0%    0.0%    0.0%    0.0%    0.0%    0.0%    0.0%    0.0%    0.0%    0.0%    0.0%    0.0%    0.0%    0.0%    0.0%    0.0%    0.0%    0.0%    0.0%    0.0%    0.0%    0.0%    0.0%    0.0%    0.0%    0.0%    0.0%    0.0%    0.0%    0.0%    0.0%    0.0%    0.0%    0.0%    0.0%    0.0%    0.0%    0.0%    0.0%    0.0%    0.0%    0.0%    0.0%    0.0%    0.0%    0.0%    0.0%    0.0%    0.0%    0.0%    0.0%    0.0%    0.0%    0.0%    0.0%    0.0%    0.0%    0.0%    0.0%    0.0%    0.0%    0.0%    0.0%    0.0%    0.0%    0.0%    0.0%    0.0%    0.0%    0.0%    0.0%    0.0%    0.0%    0.0%    0.0%    0.0%    0.0%    0.0%    0.0%    0.0%    0.0%    0.0%    0.0%    0.0%    0.0%    0.0%    0.0%    0.0%    0.0%    0.0%    0.0%    0.0%    0.0%    0.0%    0.0%    0.0%    0.0%    0.0%    0.0%    0.0%    0.0%    0.0%    0.0%    0.0%    0.0%    0.0%    0.0%    0.0%    0.0%    0.0%    0.0%    0.0%    0.0%    0.0%    0.0%    0.0%    0.0%    0.0%    0.0%    0.0%    0.0%    0.0%    0.0%    0.0%    0.0%    0.0%    0.0%    0.0%    0.0%    0.0%    0.0%    0.0%    0.0%    0.0%    0.0%    0.0%    0.0%    0.0%    0.0%    0.0%    0.0%    0.0%    0.0%    0.0%    0.0%    0.0%    0.0%    0.0%    0.0%    0.0%    0.0%    0.0%    0.0%    0.0%    0.0%    0.0%    0.0%    0.0%    0.0%    0.0%    0.0%    0.0%    0.0%    0.0%    0.0%    0.0%    0.0%    0.0%    0.0%    0.0%    0.0%    0.0%    0.0%    0.0%    0.0%    0.0%    0.0%    0.0%    0.0%    0.0%    0.0%    0.0%    0.0%    0.0%    0.0%    0.0%    0.0%    0.0%    0.0%    0.0%    0.0%    0.0%    0.0%    0.0%    0.0%    0.0%    0.0%    0.0%    0.0%    0.0%    0.0%    0.0%    0.0%    0.0%    0.0%        k__Bacteria; p__ZB2       1    0.0%    0.0%    0.0%    0.0%    0.0%    0.0%    0.0%    0.0%    0.0%    0.0%    0.0%    0.0%    0.0%    0.0%    0.0%    0.0%    0.0%    0.0%    0.0%    0.0%    0.0%    0.0%    0.0%    0.0%    0.0%    0.0%    0.0%    0.0%    0.0%    0.0%    0.0%    0.0%    0.0%    0.0%    0.0%    0.0%    0.0%    0.0%    0.0%    0.0%    0.0%    0.0%    0.0%    0.0%    0.0%    0.0%    0.0%    0.0%    0.0%    0.0%    0.0%    0.0%    0.0%    0.0%    0.0%    0.0%    0.0%    0.0%    0.0%    0.0%    0.0%    0.0%    0.0%    0.0%    0.0%    0.0%    0.0%    0.0%    0.0%    0.0%    0.0%    0.0%    0.0%    0.0%    0.0%    0.0%    0.0%    0.0%    0.0%    0.0%    0.0%    0.0%    0.0%    0.0%    0.0%    0.0%    0.0%    0.0%    0.0%    0.0%    0.0%    0.0%    0.0%    0.0%    0.0%    0.0%    0.0%    0.0%    0.0%    0.0%    0.0%    0.0%    0.0%    0.0%    0.0%    0.0%    0.0%    0.0%    0.0%    0.0%    0.0%    0.0%    0.0%    0.0%    0.0%    0.0%    0.0%    0.0%    0.0%    0.0%    0.0%    0.0%    0.0%    0.0%    0.0%    0.0%    0.0%    0.0%    0.0%    0.0%    0.0%    0.0%    0.0%    0.0%    0.0%    0.0%    0.0%    0.0%    0.0%    0.0%    0.0%    0.0%    0.0%    0.0%    0.0%    0.0%    0.0%    0.0%    0.0%    0.0%    0.0%    0.0%    0.0%    0.0%    0.0%    0.0%    0.0%    0.0%    0.0%    0.0%    0.0%    0.0%    0.0%    0.0%    0.0%    0.0%    0.0%    0.0%    0.0%    0.0%    0.0%    0.0%    0.0%    0.0%    0.0%    0.0%    0.0%    0.0%    0.0%    0.0%    0.0%    0.0%    0.0%    0.0%    0.0%    0.0%    0.0%    0.0%    0.0%    0.0%    0.0%    0.0%    0.0%    0.0%    0.0%    0.0%    0.0%    0.0%    0.0%    0.0%    0.0%    0.0%    0.0%    0.0%    0.0%    0.0%    0.0%    0.0%    0.0%    0.0%    0.0%    0.0%    0.0%    0.0%    0.0%    0.0%    0.0%    0.0%    0.0%    0.0%    0.0%    0.0%    0.0%    0.0%    0.0%    0.0%    0.0%    0.0%    0.0%    0.0%    0.0%    0.0%    0.0%    0.0%    0.0%    0.0%    0.0%    0.0%    0.0%    0.0%    0.0%    0.0%    0.0%    0.0%    0.0%    0.0%    0.0%    0.0%    0.0%    0.0%    0.0%    0.0%    0.0%    0.0%    0.0%    0.0%    0.0%    0.0%    0.0%    0.0%    0.0%    0.0%    0.0%    0.0%    0.0%    0.0%    0.0%    0.0%    0.0%    0.0%    0.0%    0.0%    0.0%    0.0%    0.0%    0.0%    0.0%    0.0%    0.0%    0.0%    0.0%    0.0%    0.0%    0.0%    0.0%    0.0%    0.0%    0.0%    0.0%    0.0%    0.0%    0.0%    0.0%    0.0%    0.0%    0.0%    0.0%    0.0%    0.0%    0.0%    0.0%    0.0%    0.0%    0.0%    0.0%    0.0%    0.0%    0.0%    0.0%    0.0%    0.0%    0.0%    0.0%    0.0%    0.0%    0.0%    0.0%    0.0%    0.0%    0.0%    0.0%    0.0%    0.0%    0.0%    0.0%    0.0%    0.0%    0.0%    0.0%    0.0%    0.0%    0.0%    0.0%    0.0%    0.0%    0.0%    0.0%    0.0%    0.0%    0.0%    0.0%    0.0%    0.0%    0.0%    0.0%    0.0%    0.0%    0.0%    0.0%    0.0%    0.0%    0.0%    0.0%    0.0%    0.0%    0.0%    0.0%    0.0%    
     
  Taxonomy Summary. Current Level: Class  
     View Figure (.pdf)    View Legend (.pdf)   
   
 
     
 
 

 
 
 
 
 
 
 
 
 
 
 
 
 
 
 
 
 
 
 
 
 
 
 
 
 
 
 
 
 
 
 
 
 
 
 
 
 
 
 
 
 
 
 
 
 
 
 
 
 
 
 
 
 
 
 
 
 
 
 
 
 
 
 
 
 
 
 
 
 
 
 
 
 
 
 
 
 
 
 
 
 
 
 
 
 
 
 
 
 
 
 
 
 
 
 
 
 
 
 
 
 
 
 
 
 
 
 
 
 
 
 
 
 
 
 
 
 
 
 
 
 
 
 
 
 
 
 
 
 
 
 
 
 
 
 
 
 
 
 
 
 
 
 
 
 
 
 
 
 
 
 
 
 
 
 
 
 
 
 
 
 
 
 
 
 
 
 
 
 
 
 
 
 
 
 
 
 
 
 
 
 
 
 
 
 
 
 
 
 
 
 
 
 
 
 
 
 
 
 
 
 
 
 
 
 
 
 
 
 
 
 
 
 
 
 
 
 
 
 
 
 
 
 
 
 
 
 
 
 
 
 
 
 
 
 
 
 
 
 
 
 
 
 
 
 
 
 
 
 
 
 
 
 
 
 
 
 
 
 
 
 
 
 
 
 
 
 
 
 
 
 
 
 
 
 
 
 
 
 
 
 
 
 
 
 
 
 
 
 
 
 
 
 
 
 
 
 
 
 
 
 
 
 
 
 
 
 
 
 
 
 
 
 
 
 
 
 
 
 
 
 
 
 
 
 
 
 
 
 
 
 
 
 
 
 
 
 
 
 
 
 
 
 
 
 
 
 
 
 
 
 
 
 
 
 
 
 
 
 
 
 
 
 
 
 
 
 
 
 
 
 
 
 
 
 
 
 
 
 
 
 
 
 
 
 
 
 
 
 
 
 
 
 
 
 
 
 
 
 
 
 
 
 
 
 
 
 
 
 
 
 
 
 
 
 
 
 
 
 
 
 
 
 
 
 
 
 
 
 
 
 
 
 
 
 
 
 
 
 
 
 
 
 
 
 
 
 
 
 
 
 
 
 
 
 
 
 
 
 
 
 
 
 
 
 
 
 
 
 
 
 
 
 
 
 
 
 
 
 
 
 
 
 
 
 
 
 
 
 
 
 
 
 
 
 
 
 
 
 
 
 
 
 
 
 
 
 
 
 
 
 
 
 
 
 
 
 
 
 
 
 
 
 
 
 
 
 
 
 
 
 
 
 
 
 
 
 
 
 
 
 
 
 
 
 
 
 
 
 
 
 
 
 
 
 
 
 
 
 
 
 
 
 
 
 
 
 
 
 
 
 
 
 
 
 
 
 
 
 
 
 
 
 
 
 
 
 
 
 
 
 
 
 
 
 
 
 
 
 
 
 
 
 
 
 
 
 
 
 
 
 
 
 
 
 
 
 
 
 
 
 
 
 
 
 
 
 
 
 
 
 
 
 
 
 
 
 
 
 
 
 
 
 
 
 
 
 
 
 
 
 
 
 
 
 
 
 
 
 
 
 
 
 
 
 
 
 
 
 
 
 
 
 
 
 
 
 
 
 
 
 
 
 
 
 
 
 
 
 
 
 
 
 
 
 
 
 
 
 
 
 
 
 
 
 
 
 
 
 
 
 
 
 
 
 
 
 
 
 
 
 
 
 
 
 
 
 
 
 
 
 
 
 
 
 
 
 
 
 
 
 
 
 
 
 
 
 
 
 
 
 
 
 
 
 
 
 
 
 
 
 
 
 
 
 
 
 
 
 
 
 
 
 
 
 
 
 
 
 
 
 
 
 
 
 
 
 
 
 
 
 
 
 
 
 
 
 
 
 
 
 
 
 
 
 
 
 
 
 
 
 
 
 
 
 
 
 
 
 
 
 
 
 
 
 
 
 
 
 
 
 
 
 
 
 
 
 
 
 
 
 
 
 
 
 
 
 
 
 
 
 
 
 
 
 
 
 
 
 
 
 
 
 
 
 
 
 
 
 
 
 
 
 
 
 
 
 
 
 
 
 
 
 
 
 
 
 
 
 
 
 
 
 
 
 
 
 
 
 
 
 
 
 
 
 
 
 
 
 
 
 
 
 
 
 
 
 
 
 
 
 
 
 
 
 
 
 
 
 
 
 
 
 
 
 
 
 
 
 
 
 
 
 
 
 
 
 
 
 
 
 
 
 
 
 
 
 
 
 
 
 
 
 
 
 
 
 
 
 
 
 
 
 
 
 
 
 
 
 
 
 
 
 
 
 
 
 
 
 
 
 
 
 
 
 
 
 
 
 
 
 
 
 
 
 
 
 
 
 
 
 
 
 
 
 
 
 
 
 
 
 
 
 
 
 
 
 
 
 
 
 
 
 
 
 
 
 
 
 
 
 
 
 
 
 
 
 
 
 
 
 
 
 
 
 
 
 
 
 
 
 
 
 
 
 
 
 
 
 
 
 
 
 
 
 
 
 
 
 
 
 
 
 
 
 
 
 
 
 
 
 
 
 
 
 
 
 
 
 
 
 
 
 
 
 
 
 
 
 
 
 
 
 
 
 
 
 
 
 
 
 
 
 
 
 
 
 
 
 
 
 
 
 
 
 
 
 
 
 
 
 
 
 
 
 
 
 
 
 
 
 
 
 
 
 
 
 
 
 
 
 
 
 
 
 
 
 
 
 
 
 
 
 
 
 
 
 
 
 
 
 
 
 
 
 
 
 
 
 
 
 
 
 
 
 
 
 
 
 
 
 
 
 
 
 
 
 
 
 
 
 
 
 
 
 
 
 
 
 
 
 
 
 
 
 
 
 
 
 
 
 
 
 
 
 
 
 
 
 
 
 
 
 
 
 
 
 
 
 
 
 
 
 
 
 
 
 
 
 
 
 
 
 
 
 
 
 
 
 
 
 
 
 
 
 
 
 
 
 
 
 
 
 
 
 
 
 
 
 
 
 
 
 
 
 
 
 
 
 
 
 
 
 
 
 
 
 
 
 
 
 
 
 
 
 
 
 
 
 
 
 
 
 
 
 
 
 
 
 
 
 
 
 
 
 
 
 
 
 
 
 
 
 
 
 
 
 
 
 
 
 
 
 
 
 
 
 
 
 
 
 
 
 
 
 
 
 
 
 
 
 
 
 
 
 
 
 
 
 
 
 
 
 
 
 
 
 
 
 
 
 
 
 
 
 
 
 
 
 
 
 
 
 
 
 
 
 
 
 
 
 
 
 
 
 
 
 
 
 
 
 
 
 
 
 
 
 
 
 
 
 
 
 
 
 
 
 
 
 
 
 
 
 
 
 
 
 
 
 
 
 
 
 
 
 
 
 
 
 
 
 
 
 
 
 
 
 
 
 
 
 
 
 
 
 
 
 
 
 
 
 
 
 
 
 
 
 
 
 
 
 
 
 
 
 
 
 
 
 
 
 
 
 
 
 
 
 
 
 
 
 
 
 
 
 
 
 
 
 
 
 
 
 
 
 
 
 
 
 
 
 
 
 
 
 
 
 
 
 
 
 
 
 
 
 
 
 
 
 
 
 
 
 
 
 
 
 
 
 
 
 
 
 
 
 
 
 
 
 
 
 
 
 
 
 
 
 
 
 
 
 
 
 
 
 
 
 
 
 
 
 
 
 
 
 
 
 
 
 
 
 
 
 
 
 
 
 
 
 
 
 
 
 
 
 
 
 
 
 
 
 
 
 
 
 
 
 
 
 
 
 
 
 
 
 
 
 
 
 
 
 
 
 
 
 
 
 
 
 
 
 
 
 
 
 
 
 
 
 
 
 
 
 
 
 
 
 
 
 
 
 
 
 
 
 
 
 
 
 
 
 
 
 
 
 
 
 
 
 
 
 
 
 
 
 
 
 
 
 
 
 
 
 
 
 
 
 
 
 
 
 
 
 
 
 
 
 
 
 
 
 
 
 
 
 
 
 
 
 
 
 
 
 
 
 
 
 
 
 
 
 
 
 
 
 
 
 
 
 
 
 
 
 
 
 
 
 
 
 
 
 
 
 
 
 
 
 
 
 
 
 
 
 
 
 
 
 
 
 
 
 
 
 
 
 
 
 
 
 
 
 
 
 
 
 
 
 
 
 
 
 
 
 
 
 
 
 
 
 
 
 
 
 
 
 
 
 
 
 
 
 
 
 
 
 
 
 
 
 
 
 
 
 
 
 
 
 
 
 
 
 
 
 
 
 
 
 
 
 
 
 
 
 
 
 
 
 
 
 
 
 
 
 
 
 
 
 
 
 
 
 
 
 
 
 
 
 
 
 
 
 
 
 
 
 
 
 
 
 
 
 
 
 
 
 
 
 
 
 
 
 
 
 
 
 
 
 
 
 
 
 
 
 
 
 
 
 
 
 
 
 
 
 
 
 
 
 
 
 
 
 
 
 
 
 
 
 
 
 
 
 
 
 
 
 
 
 
 
 
 
 
 
 
 
 
 
 
 
 
 
 
 
 
 
 
 
 
 
 
 
 
 
 
 
 
 
 
 
 
 
 
 
 
 
 
 
 
 
 
 
 
 
 
 
 
 
 
 
 
 
 
 
 
 
 
 
 
 
 
 
 
 
 
 
 
 
 
 
 
 
 
 
 
 
 
 
 
 
 
 
 
 
 
 
 
 
 
 
 
 
 
 
 
 
 
 
 
 
 
 
 
 
 
 
 
 
 
 
 
 
 
 
 
 
 
 
 
 
 
 
 
 
 
 
 
 
 
 
 
 
 
 
 
 
 
 
 
 
 
 
 
 
 
 
 
 
 
 
 
 
 
 
 
 
 
 
 
 
 
 
 
 
 
 
 
 
 
 
 
 
 
 
 
 
 
 
 
 
 
 
 
 
 
 
 
 
 
 
 
 
 
 
 
 
 
 
 
 
 
 
 
 
 
 
 
 
 
 
 
 
 
 
 
 
 
 
 
 
 
 
 
 
 
 
 
 
 
 
 
 
 
 
 
 
 
 
 
 
 
 
 
 
 
 
 
 
 
 
 
 
 
 
 
 
 
 
 
 
 
 
 
 
 
 
 
 
 
 
 
 
 
 
 
 
 
 
 
 
 
 
 
 
 
 
 
 
 
 
 
 
 
 
 
 
 
 
 
 
 
 
 
 
 
 
 
 
 
 
 
 
 
 
 
 
 
 
 
 
 
 
 
 
 
 
 
 
 
 
 
 
 
 
 
 
 
 
 
 
 
 
 
 
 
 
 
 
 
 
 
 
 
 
 
 
 
 
 
 
 
 
 
 
 
 
 
 
 
 
 
 
 
 
 
 
 
 
 
 
 
 
 
 
 
 
 
 
 
 
 
 
 
 
 
 
 
 
 
 
 
 
 
 
 
 
 
 
 
 
 
 
 
 
 
 
 
 
 
 
 
 
 
 
 
 
 
 
 
 
 
 
 
 
 
 
 
 
 
 
 
 
 
 
 
 
 
 
 
 
 
 
 
 
 
 
 
 
 
 
 
 
 
 
 
 
 
 
 
 
 
 
 
 
 
 
 
 
 
 
 
 
 
 
 
 
 
 
 
 
 
 
 
 
 
 
 
 
 
 
 
 
 
 
 
 
 
 
 
 
 
 
 
 
 
 
 
 
 
 
 
 
 
 
 
 
 
 
 
 
 
 
 
 
 
 
 
 
 
 
 
 
 
 
 
 
 
 
 
 
 
 
 
 
 
 
 
 
 
 
 
 
 
 
 
 
 
 
 
 
 
 
 
 
 
 
 
 
 
 
 
 
 
 
 
 
 
 
 
 
 
 
 
 
 
 
 
 
 
 
 
 
 
 
 
 
 
 
 
 
 
 
 
 
 
 
 
 
 
 
 
 
 
 
 
 
 
 
 
 
 
 
 
 
 
 
 
 
 
 
 
 
 
 
 
 
 
 
 
 
 
 
 
 
 
 
 
 
 
 
 
 
 
 
 
 
 
 
 
 
 
 
 
 
 
 
 
 
 
 
 
 
 
 
 
 
 
 
 
 
 
 
 
 
 
 
 
 
 
 
 
 
 
 
 
 
 
 
 
 
 
 
 
 
 
 
 
 
 
 
 
 
 
 
 
 
 
 
 
 
 
 
 
 
 
 
 
 
 
 
 
 
 
 
 
 
 
 
 
 
 
 
 
 
 
 
 
 
 
 
 
 
 
 
 
 
 
 
 
 
 
 
 
 
 
 
 
 
 
 
 
 
 
 
 
 
 
 
 
 
 
 
 
 
 
 
 
 
 
 
 
 
 
 
 
 
 
 
 
 
 
 
 
 
 
 
 
 
 
 
 
 
 
 
 
 
 
 
 
 
 
 
 
 
 
 
 
 
 
 
 
 
 
 
 
 
 
 
 
 
 
 
 
 
 
 
 
 
 
 
 
 
 
 
 
 
 
 
 
 
 
 
 
 
 
 
 
 
 
 
 
 
 
 
 
 
 
 
 
 
 
 
 
 
 
 
 
 
 
 
 
 
 
 
 
 
 
 
 
 
 
 
 
 
 
 
 
 
 
 
 
 
 
 
 
 
 
 
 
 
 
 
 
 
 
 
 
 
 
 
 
 
 
 
 
 
 
 
 
 
 
 
 
 
 
 
 
 
 
 
 
 
 
 
 
 
 
 
 
 
 
 
 
 
 
 
 
 
 
 
 
 
 
 
 
 
 
 
 
 
 
 
 
 
 
 
 
 
 
 
 
 
 
 
 
 
 
 
 
 
 
 
 
 
 
 
 
 
 
 
 
 
 
 
 
 
 
 
 
 
 
 
 
 
 
 
 
 
 
 
 
 
 
 
 
 
 
 
 
 
 
 
 
 
 
 
 
 
 
 
 
 
 
 
 
 
 
 
 
 
 
 
 
 
 
 
 
 
 
 
 
 
 
 
 
 
 
 
 
 
 
 
 
 
 
 
 
 
 
 
 
 
 
 
 
 
 
 
 
 
 
 
 
 
 
 
 
 
 
 
 
 
 
 
 
 
 
 
 
 
 
 
 
 
 
 
 
 
 
 
 
 
 
 
 
 
 
 
 
 
 
 
 
 
 
 
 
 
 
 
 
 
 
 
 
 
 
 
 
 
 
 
 
 
 
 
 
 
 
 
 
 
 
 
 
 
 
 
 
 
 
 
 
 
 
 
 
 
 
 
 
 
 
 
 
 
 
 
 
 
 
 
 
 
 
 
 
 
 
 
 
 
 
 
 
 
 
 
 
 
 
 
 
 
 
 
 
 
 
 
 
 
 
 
 
 
 
 
 
 
 
 
 
 
 
 
 
 
 
 
 
 
 
 
 
 
 
 
 
 
 
 
 
 
 
 
 
 
 
 
 
 
 
 
 
 
 
 
 
 
 
 
 
 
 
 
 
 
 
 
 
 
 
 
 
 
 
 
 
 
 
 
 
 
 
 
 
 
 
 
 
 
 
 
 
 
 
 
 
 
 
 
 
 
 
 
 
 
 
 
 
 
 
 
 
 
 
 
 
 
 
 
 
 
 
 
 
 
 
 
 
 
 
 
 
 
 
 
 
 
 
 
 
 
 
 
 
 
 
 
 
 
 
 
 
 
 
 
 
 
 
 
 
 
 
 
 
 
 
 
 
 
 
 
 
 
 
 
 
 
 
 
 
 
 
 
 
 
 
 
 
 
 
 
 
 
 
 
 
 
 
 
 
 
 
 
 
 
 
 
 
 
 
 
 
 
 
 
 
 
 
 
 
 
 
 
 
 
 
 
 
 
 
 
 
 
 
 
 
 
 
 
 
 
 
 
 
 
 
 
 
 
 
 
 
 
 
 
 
 
 
 
 
 
 
 
 
 
 
 
 
 
 
 
 
 
 
 
 
 
 
 
 
 
 
 
 
 
 
 
 
 
 
 
 
 
 
 
 
 
 
 
 
 
 
 
 
 
 
 
 
 
 
 
 
 
 
 
 
 
 
 
 
 
 
 
 
 
 
 
 
 
 
 
 
 
 
 
 
 
 
 
 
 
 
 
 
 
 
 
 
 
 
 
 
 
 
 
 
 
 
 
 
 
 
 
 
 
 
 
 
 
 
 
 
 
 
 
 
 
 
 
 
 
 
 
 
 
 
 
 
 
 
 
 
 
 
 
 
 
 
 
 
 
 
 
 
 
 
 
 
 
 
 
 
 
 
 
 
 
 
 
 
 
 
 
 
 
 
 
 
 
 
 
 
 
 
 
 
 
 
 
 
 
 
 
 
 
 
 
 
 
 
 
 
 
 
 
 
 
 
 
 
 
 
 
 
 
 
 
 
 
 
 
 
 
 
 
 
 
 
 
 
 
 
 
 
 
 
 
 
 
 
 
 
 
 
 
 
 
 
 
 
 
 
 
 
 
 
 
 
 
 
 
 
 
 
 
 
 
 
 
 
 
 
 
 
 
 
 
 
 
 
 
 
 
 
 
 
 
 
 
 
 
 
 
 
 
 
 
 
 
 
 
 
 
 
 
 
 
 
 
 
 
 
 
 
 
 
 
 
 
 
 
 
 
 
 
 
 
 
 
 
 
 
 
 
 
 
 
 
 
 
 
 
 
 
 
 
 
 
 
 
 
 
 
 
 
 
 
 
 
 
 
 
 
 
 
 
 
 
 
 
 
 
 
 
 
 
 
 
 
 
 
 
 
 
 
 
 
 
 
 
 
 
 
 
 
 
 
 
 
 
 
 
 
 
 
 
 
 
 
 
 
 
 
 
 
 
 
 
 
 
 
 
 
 
 
 
 
 
 
 
 
 
 
 
 
 
 
 
 
 
 
 
 
 
 
 
 
 
 
 
 
 
 
 
 
 
 
 
 
 
 
 
 
 
 
 
 
 
 
 
 
 
 
 
 
 
 
 
 
 
 
 
 
 
 
 
 
 
 
 
 
 
 
 
 
 
 
 
 
 
 
 
 
 
 
 
 
 
 
 
 
 
 
 
 
 
 
 
 
 
 
 
 
 
 
 
 
 
 
 
 
 
 
 
 
 
 
 
 
 
 
 
 
 
 
 
 
 
 
 
 
 
 
 
 
 
 
 
 
 
 
 
 
 
 
 
 
 
 
 
 
 
 
 
 
 
 
 
 
 
 
 
 
 
 
 
 
 
 
 
 
 
 
 
 
 
 
 
 
 
 
 
 
 
 
 
 
 
 
 
 
 
 
 
 
 
 
 
 
 
 
 
 
 
 
 
 
 
 
 
 
 
 
 
 
 
 
 
 
 
 
 
 
 
 
 
 
 
 
 
 
 
 
 
 
 
 
 
 
 
 
 
 
 
 
 
 
 
 
 
 
 
 
 
 
 
 
 
 
 
 
 
 
 
 
 
 
 
 
 
 
 
 
 
 
 
 
 
 
 
 
 
 
 
 
 
 
 
 
 
 
 
 
 
 
 
 
 
 
 
 
 
 
 
 
 
 
 
 
 
 
 
 
 
 
 
 
 
 
 
 
 
 
 
 
 
 
 
 
 
 
 
 
 
 
 
 
 
 
 
 
 
 
 
 
 
 
 
 
 
 
 
 
 
 
 
 
 
 
 
 
 
 
 
 
 
 
 
 
 
 
 
 
 
 
 
 
 
 
 
 
 
 
 
 
 
 
 
 
 
 
 
 
 
 
 
 
 
 
 
 
 
 
 
 
 
 
 
 
 
 
 
 
 
 
 
 
 
 
 
 
 
 
 
 
 
 
 
 
 
 
 
 
 
 
 
 
 
 
 
 
 
 
 
 
 
 
 
 
 
 
 
 
 
 
 
 
 
 
 
 
 
 
 
 
 
 
 
 
 
 
 
 
 
 
 
 
 
 
 
 
 
 
 
 
 
 
 
 
 
 
 
 
 
 
 
 
 
 
 
 
 
 
 
 
 
 
 
 
 
 
 
 
 
 
 
 
 
 
 
 
 
 
 
 
 
 
 
 
 
 
 
 
 
 
 
 
 
 
 
 
 
 
 
 
 
 
 
 
 
 
 
 
 
 
 
 
 
 
 
 
 
 
 
 
 
 
 
 
 
 
 
 
 
 
 
 
 
 
 
 
 
 
 
 
 
 
 
 
 
 
 
 
 
 
 
 
 
 
 
 
 
 
 
 
 
 
 
 
 
 
 
 
 
 
 
 
 
 
 
 
 
 
 
 
 
 
 
 
 
 
 
 
 
 
 
 
 
 
 
 
 
 
 
 
 
 
 
 
 
 
 
 
 
 
 
 
 
 
 
 
 
 
 
 
 
 
 
 
 
 
 
 
 
 
 
 
 
 
 
 
 
 
 
 
 
 
 
 
 
 
 
 
 
 
 
 
 
 
 
 
 
 
 
 
 
 
 
 
 
 
 
 
 
 
 
 
 
 
 
 
 
 
 
 
 
 
 
 
 
 
 
 
 
 
 
 
 
 
 
 
 
 
 
 
 
 
 
 
 
 
 
 
 
 
 
 
 
 
 
 
 
 
 
 
 
 
 
 
 
 
 
 
 
 
 
 
 
 
 
 
 
 
 
 
 
 
 
 
 
 
 
 
 
 
 
 
 
 
 
 
 
 
 
 
 
 
 
 
 
 
 
 
 
 
 
 
 
 
 
 
 
 
 
 
 
 
 
 
 
 
 
 
 
 
 
 
 
 
 
 
 
 
 
 
 
 
 
 
 
 
 
 
 
 
 
 
 
 
 
 
 
 
 
 
 
 
 
 
 
 
 
 
 
 
 
 
 
 
 
 
 
 
 
 
 
 
 
 
 
 
 
 
 
 
 
 
 
 
 
 
 
 
 
 
 
 
 
 
 
 
 
 
 
 
 
 
 
 
 
 
 
 
 
 
 
 
 
 
 
 
 
 
 
 
 
 
 
 
 
 
 
 
 
 
 
 
 
 
 
 
 
 
 
 
 
 
 
 
 
 
 
 
 
 
 
 
 
 
 
 
 
 
 
 
 
 
 
 
 
 
 
 
 
 
 
 
 
 
 
 
 
 
 
 
 
 
 
 
 
 
 
 
 
 
 
 
 
 
 
 
 
 
 
 
 
 
 
 
 
 
 
 
 
 
 
 
 
 
 
 
 
 
 
 
 
 
 
 
 
 
 
 
 
 
 
 
 
 
 
 
 
 
 
 
 
 
 
 
 
 
 
 
 
 
 
 
 
 
 
 
 
 
 
 
 
 
 
 
 
 
 
 
 
 
 
 
 
 
 
 
 
 
 
 
 
 
 
 
 
 
 
 
 
 
 
 
 
 
 
 
 
 
 
 
 
 
 
 
 
 
 
 
 
 
 
 
 
 
 
 
 
 
 
 
 
 
 
 
 
 
 
 
 
 
 
 
 
 
 
 
 
 
 
 
 
 
 
 
 
 
 
 
 
 
 
 
 
 
 
 
 
 
 
 
 
 
 
 
 
 
 
 
 
 
 
 
 
 
 
 
 
 
 
 
 
 
 
 
 
 
 
 
 
 
 
 
 
 
 
 
 
 
 
 
 
 
 
 
 
 
 
 
 
 
 
 
 
 
 
 
 
 
 
 
 
 
 
 
 
 
 
 
 
 
 
 
 
 
 
 
 
 
 
 
 
 
 
 
 
 
 
 
 
 
 
 
 
 
 
 
 
 
 
 
 
 
 
 
 
 
 
 
 
 
 
 
 
 
 
 
 
 
 
 
 
 
 
 
 
 
 
 
 
 
 
 
 
 
 
 
 
 
 
 
 
 
 
 
 
 
 
 
 
 
 
 
 
 
 
 
 
 
 
 
 
 
 
 
 
 
 
 
 
 
 
 
 
 
 
 
 
 
 
 
 
 
 
 
 
 
 
 
 
 
 
 
 
 
 
 
 
 
 
 
 
 
 
 
 
 
 
 
 
 
 
 
 
 
 
 
 
 
 
 
 
 
 
 
 
 
 
 
 
 
 
 
 
 
 
 
 
 
 
 
 
 
 
 
 
 
 
 
 
 
 
 
 
 
 
 
 
 
 
 
 
 
 
 
 
 
 
 
 
 
 
 
 
 
 
 
 
 
 
 
 
 
 
 
 
 
 
 
 
 
 
 
 
 
 
 
 
 
 
 
 
 
 
 
 
 
 
 
 
 
 
 
 
 
 
 
 
 
 
 
 
 
 
 
 
 
 
 
 
 
 
 
 
 
 
 
 
 
 
 
 
 
 
 
 
 
 
 
 
 
 
 
 
 
 
 
 
 
 
 
 
 
 
 
 
 
 
 
 
 
 
 
 
 
 
 
 
 
 
 
 
 
 
 
 
 
 
 
 
 
 
 
 
 
 
 
 
 
 
 
 
 
 
 
 
 
 
 
 
 
 
 
 
 
 
 
 
 
 
 
 
 
 
 
 
 
 
 
 
 
 
 
 
 
 
 
 
 
 
 
 
 
 
 
 
 
 
 
 
 
 
 
 
 
 
 
 
 
 
 
 
 
 
 
 
 
 
 
 
 
 
 
 
 
 
 
 
 
 
 
 
 
 
 
 
 
 
 
 
 
 
 
 
 
 
 
 
 
 
 
 
 
 
 
 
 
 
 
 
 
 
 
 
 
 
 
 
 
 
 
 
 
 
 
 
 
 
 
 
 
 
 
 
 
 
 
 
 
 
 
 
 
 
 
 
 
 
 
 
 
 
 
 
 
 
 
 
 
 
 
 
 
 
 
 
 
 
 
 
 
 
 
 
 
 
 
 
 
 
 
 
 
 
 
 
 
 
 
 
 
 
 
 
 
 
 
 
 
 
 
 
 
 
 
 
 
 
 
 
 
 
 
 
 
 
 
 
 
 
 
 
 
 
 
 
 
 
 
 
 
 
 
 
 
 
 
 
 
 
 
 
 
 
 
 
 
 
 
 
 
 
 
 
 
 
 
 
 
 
 
 
 
 
 
 
 
 
 
 
 
 
 
 
 
 
 
 
 
 
 
 
 
 
 
 
 
 
 
 
 
 
 
 
 
 
 
 
 
 
 
 
 
 
 
 
 
 
 
 
 
 
 
 
 
 
 
 
 
 
 
 
 
 
 
 
 
 
 
 
 
 
 
 
 
 
 
 
 
 
 
 
 
 
 
 
 
 
 
 
 
 
 
 
 
 
 
 
 
 
 
 
 
 
 
 
 
 
 
 
 
 
 
 
 
 
 
 
 
 
 
 
 
 
 
 
 
 
 
 
 
 
 
 
 
 
 
 
 
 
 
 
 
 
 
 
 
 
 
 
 
 
 
 
 
 
 
 
 
 
 
 
 
 
 
 
 
 
 
 
 
 
 
 
 
 
 
 
 
 
 
 
 
 
 
 
 
 
 
 
 
 
 
 
 
 
 
 
 
 
 
 
 
 
 
 
 
 
 
 
 
 
 
 
 
 
 
 
 
 
 
 
 
 
 
 
 
 
 
 
 
 
 
 
 
 
 
 
 
 
 
 
 
 
 
 
 
 
 
 
 
 
 
 
 
 
 
 
 
 
 
 
 
 
 
 
 
 
 
 
 
 
 
 
 
 
 
 
 
 
 
 
 
 
 
 
 
 
 
 
 
 
 
 
 
 
 
 
 
 
 
 
 
 
 
 
 
 
 
 
 
 
 
 
 
 
 
 
 
 
 
 
 
 
 
 
 
 
 
 
 
 
 
 
 
 
 
 
 
 
 
 
 
 
 
 
 
 
 
 
 
 
 
 
 
 
 
 
 
 
 
 
 
 
 
 
 
 
 
 
 
 
 
 
 
 
 
 
 
 
 
 
 
 
 
 
 
 
 
 
 
 
 
 
 
 
 
 
 
 
 
 
 
 
 
 
 
 
 
 
 
 
 
 
 
 
 
 
 
 
 
 
 
 
 
 
 
 
 
 
 
 
 
 
 
 
 
 
 
 
 
 
 
 
 
 
 
 
 
 
 
 
 
 
 
 
 
 
 
 
 
 
 
 
 
 
 
 
 
 
 
 
 
 
 
 
 
 
 
 
 
 
 
 
 
 
 
 
 
 
 
 
 
 
 
 
 
 
 
 
 
 
 
 
 
 
 
 
 
 
 
 
 
 
 
 
 
 
 
 
 
 
 
 
 
 
 
 
 
 
 
 
 
 
 
 
 
 
 
 
 
 
 
 
 
 
 
 
 
 
 
 
 
 
 
 
 
 
 
 
 
 
 
 
 
 
 
 
 
 
 
 
 
 
 
 
 
 
 
 
 
 
 
 
 
 
 
 
 
 
 
 
 
 
 
 
 
 
 
 
 
 
 
 
 
 
 
 
 
 
 
 
 
 
 
 
 
 
 
 
 
 
 
 
 
 
 
 
 
 
 
 
 
 
 
 
 
 
 
 
 
 
 
 
 
 
 
 
 
 
 
 
 
 
 
 
 
 
 
 
 
 
 
 
 
 
 
 
 
 
 
 
 
 
 
 
 
 
 
 
 
 
 
 
 
 
 
 
 
 
 
 
 
 
 
 
 
 
 
 
 
 
 
 
 
 
 
 
 
 
 
 
 
 
 
 
 
 
 
 
 
 
 
 
 
 
 
 
 
 
 
 
 
 
 
 
 
 
 
 
 
 
 
 
 
 
 
 
 
 
 
 
 
 
 
 
 
 
 
 
 
 
 
 
 
 
 
 
 
 
 
 
 
 
 
 
 
 
 
 
 
 
 
 
 
 
 
 
 
 
 
 
 
 
 
 
 
 
 
 
 
 
 
 
 
 
 
 
 
 
 
 
 
 
 
 
 
 
 
 
 
 
 
 
 
 
 
 
 
 
 
 
 
 
 
 
 
 
 
 
 
 
 
 
 
 
 
 
 
 
 
 
 
 
 
 
 
 
 
 
 
 
 
 
 
 
 
 
 
 
 
 
 
 
 
 
 
 
 
 
 
 
 
 
 
 
 
 
 
 
 
 
 
 
 
 
 
 
 
 
 
 
 
 
 
 
 
 
 
 
 
 
 
 
 
 
 
 
 
 
 
 
 
 
 
 
 
 
 
 
 
 
 
 
 
 
 
 
 
 
 
 
 
 
 
 
 
 
 
 
 
 
 
 
 
 
 
 
 
 
 
 
 
 
 
 
 
 
 
 
 
 
 
 
 
 
 
 
 
 
 
 
 
 
 
 
 
 
 
 
 
 
 
 
 
 
 
 
 
 
 
 
 
 
 
 
 
 
 
 
 
 
 
 
 
 
 
 
 
 
 
 
 
 
 
 
 
 
 
 
 
 
 
 
 
 
 
 
 
 
 
 
 
 
 
 
 
 
 
 
 
 
 
 
 
 
 
 
 
 
 
 
 
 
 
 
 
 
 
 
 
 
 
 
 
 
 
 
 
 
 
 
 
 
 
 
 
 
 
 
 
 
 
 
 
 
 
 
 
 
 
 
 
 
 
 
 
 
 
 
 
 
 
 
 
 
 
 
 
 
 
 
 
 
 
 
 
 
 
 
 
 
 
 
 
 
 
 
 
 
 
 
 
 
 
 
 
 
 
 
 
 
 
 
 
 
 
 
 
 
 
 
 
 
 
 
 
 
 
 
 
 
 
 
 
 
 
 
 
 
 
 
 
 
 
 
 
 
 
 
 
 
 
 
 
 
 
 
 
 
 
 
 
 
 
 
 
 
 
 
 
 
 
 
 
 
 
 
 
 
 
 
 
 
 
 
 
 
 
 
 
 
 
 
 
 
 
 
 
 
 
 
 
 
 
 
 
 
 
 
 
 
 
 
 
 
 
 
 
 
 
 
 
 
 
 
 
 
 
 
 
 
 
 
 
 
 
 
 
 
 
 
 
 
 
 
 
 
 
 
 
 
 
 
 
 
 
 
 
 
 
 
 
 
 
 
 
 
 
 
 
 
 
 
 
 
 
 
 
 
 
 
 
 
 
 
 
 
 
 
 
 
 
 
 
 
 
 
 
 
 
 
 
 
 
 
 
 
 
 
 
 
 
 
 
 
 
 
 
 
 
 
 
 
 
 
 
 
 
 
 
 
 
 
 
 
 
 
 
 
 
 
 
 
 
 
 
 
 
 
 
 
 
 
 
 
 
 
 
 
 
 
 
 
 
 
 
 
 
 
 
 
 
 
 
 
 
 
 
 
 
 
 
 
 
 
 
 
 
 
 
 
 
 
 
 
 
 
 
 
 
 
 
 
 
 
 
 
 
 
 
 
 
 
 
 
 
 
 
 
 
 
 
 
 
 
 
 
 
 
 
 
 
 
 
 
 
 
 
 
 
 
 
 
 
 
 
 
 
 
 
 
 
 
 
 
 
 
 
 
 
 
 
 
 
 
 
 
 
 
 
 
 
 
 
 
 
 
 
 
 
 
 
 
 
 
 
 
 
 
 
 
 
 
 
 
 
 
 
 
 
 
 
 
 
 
 
 
 
 
 
 
 
 
 
 
 
 
 
 
 
 
 
 
 
 
 
 
 
 
 
 
 
 
 
 
 
 
 
 
 
 
 
 
 
 
 
 
 
 
 
 
 
 
 
 
 
 
 
 
 
 
 
 
 
 
 
 
 
 
 
 
 
 
 
 
 
 
 
 
 
 
 
 
 
 
 
 
 
 
 
 
 
 
 
 
 
 
 
 
 
 
 
 
 
 
 
 
 
 
 
 
 
 
 
 
 
 
 
 
 
 
 
 
 
 
 
 
 
 
 
 
 
 
 
 
 
 
 
 
 
 
 
 
 
 
 
 
 
 
 
 
 
 
 
 
 
 
 
 
 
 
 
 
 
 
 
 
 
 
 
 
 
 
 
 
 
 
 
 
 
 
 
 
 
 
 
 
 
 
 
 
 
 
 
 
 
 
 
 
 
 
 
 
 
 
 
 
 
 
 
 
 
 
 
 
 
 
 
 
 
 
 
 
 
 
 
 
 
 
 
 
 
 
 
 
 
 
 
 
 
 
 
 
 
 
 
 
 
 
 
 
 
 
 
 
 
 
 
 
 
 
 
 
 
 
 
 
 
 
 
 
 
 
 
 
 
 
 
 
 
 
 
 
 
 
 
 
 
 
 
 
 
 
 
 
 
 
 
 
 
 
 
 
 
 
 
 
 
 
 
 
 
 
 
 
 
 
 
 
 
 
 
 
 
 
 
 
 
 
 
 
 
 
 
 
 
 
 
 
 
 
 
 
 
 
 
 
 
 
 
 
 
 
 
 
 
 
 
 
 
 
 
 
 
 
 
 
 
 
 
 
 
 
 
 
 
 
 
 
 
 
 
 
 
 
 
 
 
 
 
 
 
 
 
 
 
 
 
 
 
 
 
 
 
 
 
 
 
 
 
 
 
 
 
 
 
 
 
 
 
 
 
 
 
 
 
 
 
 
 
 
 
 
 
 
 
 
 
 
 
 
 
 
 
 
 
 
 
 
 
 
 
 
 
 
 
 
 
 
 
 
 
 
 
 
 
 
 
 
 
 
 
 
 
 
 
 
 
 
 
 
 
 
 
 
 
 
 
 
 
 
 
 
 
 
 
 
 
 
 
 
 
 
 
 
 
 
 
 
 
 
 
 
 
 
 
 
 
 
 
 
 
 
 
 
 
 
 
 
 
 
 
 
 
 
 
 
 
 
 
 
 
 
 
 
 
 
 
 
 
 
 
 
 
 
 
 
 
 
 
 
 
 
 
 
 
 
 
 
 
 
 
 
 
 
 
 
 
 
 
 
 
 
 
 
 
 
 
 
 
 
 
 
 
 
 
 
 
 
 
 
 
 
 
 
 
 
 
 
 
 
 
 
 
 
 
 
 
 
 
 
 
 
 
 
 
 
 
 
 
 
 
 
 
 
 
 
 
 
 
 
 
 
 
 
 
 
 
 
 
 
 
 
 
 
 
 
 
 
 
 
 
 
 
 
 
 
 
 
 
 
 
 
 
 
 
 
 
 
 
 
 
 
 
 
 
 
 
 
 
 
 
 
 
 
 
 
 
 
 
 
 
 
 
 
 
 
 
 
 
 
 
 
 
 
 
 
 
 
 
 
 
 
 
 
 
 
 
 
 
 
 
 
 
 
 
 
 
 
 
 
 
 
 
 
 
 
 
 
 
 
 
 
 
 
 
 
 
 
 
 
 
 
 
 
 
 
 
 
 
 
 
 
 
 
 
 
 
 
 
 
 
 
 
 
 
 
 
 
 
 
 
 
 
 
 
 
 
 
 
 
 
 
 
 
 
 
 
 
 
 
 
 
 
 
 
 
 
 
 
 
 
 
 
 
 
 
 
 
 
 
 
 
 
 
 
 
 
 
 
 
 
 
 
 
 
 
 
 
 
 
 
 
 
 
 
 
 
 
 
 
 
 
 
 
 
 
 
 
 
 
 
 
 
 
 
 
 
 
 
 
 
 
 
 
 
 
 
 
 
 
 
 
 
 
 
 
 
 
 
 
 
 
 
 
 
 
 
 
 
 
 
 
 
 
 
 
 
 
 
 
 
 
 
 
 
 
 
 
 
 
 
 
 
 
 
 
 
 
 
 
 
 
 
 
 
 
 
 
 
 
 
 
 
 
 
 
 
 
 
 
 
 
 
 
 
 
 
 
 
 
 
 
 
 
 
 
 
 
 
 
 
 
 
 
 
 
 
 
 
 
 
 
 
 
 
 
 
 
 
 
 
 
 
 
 
 
 
 
 
 
 
 
 
 
 
 
 
 
 
 
 
 
 
 
 
 
 
 
 
 
 
 
 
 
 
 
 
 
 
 
 
 
 
 
 
 
 
 
 
 
 
 
 
 
 
 
 
 
 
 
 
 
 
 
 
 
 
 
 
 
 
 
 
 
 
 
 
 
 
 
 
 
 
 
 
 
 
 
 
 
 
 
 
 
 
 
 
 
 
 
 
 
 
 
 
 
 
 
 
 
 
 
 
 
 
 
 
 
 
 
 
 
 
 
 
 
 
 
 
 
 
 
 
 
 
 
 
 
 
 
 
 
 
 
 
 
 
 
 
 
 
 
 
 
 
 
 
 
 
 
 
 
 
 
 
 
 
 
 
 
 
 
 
 
 
 
 
 
 
 
 
 
 
 
 
 
 
 
 
 
 
 
 
 
 
 
 
 
 
 
 
 
 
 
 
 
 
 
 
 
 
 
 
 
 
 
 
 
 
 
 
 
 
 
 
 
 
 
 
 
 
 
 
 
 
 
 
 
 
 
 
 
 
 
 
 
 
 
 
 
 
 
 
 
 
 
 
 
 
 
 
 
 
 
 
 
 
 
 
 
 
 
 
 
 
 
 
 
 
 
 
 
 
 
 
 
 
 
 
 
 
 
 
 
 
 
 
 
 
 
 
 
 
 
 
 
 
 
 
 
 
 
 
 
 
 
 
 
 
 
 
 
 
 
 
 
 
 
 
 
 
 
 
 
 
 
 
 
 
 
 
 
 
 
 
 
 
 
 
 
 
 
 
 
 
 
 
 
 
 
 
 
 
 
 
 
 
 
 
 
 
 
 
 
 
 
 
 
 
 
 
 
 
 
 
 
 
 
 
 
 
 
 
 
 
 
 
 
 
 
 
 
 
 
 
 
 
 
 
 
 
 
 
 
 
 
 
 
 
 
 
 
 
 
 
 
 
 
 
 
 
 
 
 
 
 
 
 
 
 
 
 
 
 
 
 
 
 
 
 
 
 
 
 
 
 
 
 
 
 
 
 
 
 
 
 
 
 
 
 
 
 
 
 
 
 
 
 
 
 
 
 
 
 
 
 
 
 
 
 
 
 
 
 
 
 
 
 
 
 
 
 
 
 
 
 
 
 
 
 
 
 
 
 
 
 
 
 
 
 
 
 
 
 
 
 
 
 
 
 
 
 
 
 
 
 
 
 
 
 
 
 
 
 
 
 
 
 
 
 
 
 
 
 
 
 
 
 
 
 
 
 
 
 
 
 
 
 
 
 
 
 
 
 
 
 
 
 
 
 
 
 
 
 
 
 
 
 
 
 
 
 
 
 
 
 
 
 
 
 
 
 
 
 
 
 
 
 
 
 
 
 
 
 
 
 
 
 
 
 
 
 
 
 
 
 
 
 
 
 
 
 
 
 
 
 
 
 
 
 
 
 
 
 
 
 
 
 
 
 
 
 
 
 
 
 
 
 
 
 
 
 
 
 
 
 
 
 
 
 
 
 
 
 
 
 
 
 
 
 
 
 
 
 
 
 
 
 
 
 
 
 
 
 
 
 
 
 
 
 
 
 
 
 
 
 
 
 
 
 
 
 
 
 
 
 
 
 
 
 
 
 
 
 
 
 
 
 
 
 
 
 
 
 
 
 
 
 
 
 
 
 
 
 
 
 
 
 
 
 
 
 
 
 
 
 
 
 
 
 
 
 
 
 
 
 
 
 
 
 
 
 
 
 
 
 
 
 
 
 
 
 
 
 
 
 
 
 
 
 
 
 
 
 
 
 
 
 
 
 
 
 
 
 
 
 
 
 
 
 
 
 
 
 
 
 
 
 
 
 
 
 
 
 
 
 
 
 
 
 
 
 
 
 
 
 
 
 
 
 
 
 
 
 
 
 
 
 
 
 
 
 
 
 
 
 
 
 
 
 
 
 
 
 
 
 
 
 
 
 
 
 
 
 
 
 
 
 
 
 
 
 
 
 
 
 
 
 
 
 
 
 
 
 
 
 
 
 
 
 
 
 
 
 
 
 
 
 
 
 
 
 
 
 
 
 
 
 
 
 
 
 
 
 
 
 
 
 
 
 
 
 
 
 
 
 
 
 
 
 
 
 
 
 
 
 
 
 
 
 
 
 
 
 
 
 
 
 
 
 
 
 
 
 
 
 
 
 
 
 
 
 
 
 
 
 
 
 
 
 
 
 
 
 
 
 
 
 
 
 
 
 
 
 
 
 
 
 
 
 
 
 
 
 
 
 
 
 
 
 
 
 
 
 
 
 
 
 
 
 
 
 
 
 
 
 
 
 
 
 
 
 
 
 
 
 
 
 
 
 
 
 
 
 
 
 
 
 
 
 
 
 
 
 
 
 
 
 
 
 
 
 
 
 
 
 
 
 
 
 
 
 
 
 
 
 
 
 
 
 
 
 
 
 
 
 
 
 
 
 
 
 
 
 
 
 
 
 
 
 
 
 
 
 
 
 
 
 
 
 
 
 
 
 
 
 
 
 
 
 
 
 
 
 
 
 
 
 
 
 
 
 
 
 
 
 
 
 
 
 
 
 
 
 
 
 
 
 
 
 
 
 
 
 
 
 
 
 
 
 
 
 
 
 
 
 
 
 
 
 
 
 
 
 
 
 
 
 
 
 
 
 
 
 
 
 
 
 
 
 
 
 
 
 
 
 
 
 
 
 
 
 
 
 
 
 
 
 
 
 
 
 
 
 
 
 
 
 
 
 
 
 
 
 
 
 
 
 
 
 
 
 
 
 
 
 
 
 
 
 
 
 
 
 
 
 
 
 
 
 
 
 
 
 
 
 
 
 
 
 
 
 
 
 
 
 
 
 
 
 
 
 
 
 
 
 
 
 
 
 
 
 
 
 
 
 
 
 
 
 
 
 
 
 
 
 
 
 
 
 
 
 
 
 
 
 
 
 
 
 
 
 
 
 
 
 
 
 
 
 
 
 
 
 
 
 
 
 
 
 
 
 
 
 
 
 
 
 
 
 
 
 
 
 
 
 
 
 
 
 
 
 
 
 
 
 
 
 
 
 
 
 
 
 
 
 
 
 
 
 
 
 
 
 
 
 
 
 
 
 
 
 
 
 
 
 
 
 
 
 
 
 
 
 
 
 
 
 
 
 
 
 
 
 
 
 
 
 
 
 
 
 
 
 
 
 
 
 
 
 
 
 
 
 
 
 
 
 
 
 
 
 
 
 
 
 
 
 
 
 
 
 
 
 
 
 
 
 
 
 
 
 
 
 
 
 
 
 
 
 
 
 
 
 
 
 
 
 
 
 
 
 
 
 
 
 
 
 
 
 
 
 
 
 
 
 
 
 
 
 
 
 
 
 
 
 
 
 
 
 
 
 
 
 
 
 
 
 
 
 
 
 
 
 
 
 
 
 
 
 
 
 
 
 
 
 
 
 
 
 
 
 
 
 
 
 
 
 
 
 
 
 
 
 
 
 
 
 
 
 
 
 
 
 
 
 
 
 
 
 
 
 
 
 
 
 
 
 
 
 
 
 
 
 
 
 
 
 
 
 
 
 
 
 
 
 
 
 
 
 
 
 
 
 
 
 
 
 
 
 
 
 
 
 
 
 
 
 
 
 
 
 
 
 
 
 
 
 
 
 
 
 
 
 
 
 
 
 
 
 
 
 
 
 
 
 
 
 
 
 
 
 
 
 
 
 
 
 
 
 
 
 
 
 
 
 
 
 
 
 
 
 
 
 
 
 
 
 
 
 
 
 
 
 
 
 
 
 
 
 
 
 
 
 
 
 
 
 
 
 
 
 
 
 
 
 
 
 
 
 
 
 
 
 
 
 
 
 
 
 
 
 
 
 
 
 
 
 
 
 
 
 
 
 
 
 
 
 
 
 
 
 
 
 
 
 
 
 
 
 
 
 
 
 
 
 
 
 
 
 
 
 
 
 
 
 
 
 
 
 
 
 
 
 
 
 
 
 
 
 
 
 
 
 
 
 
 
 
 
 
 
 
 
 
 
 
 
 
 
 
 
 
 
 
 
 
 
 
 
 
 
 
 
 
 
 
 
 
 
 
 
 
 
 
 
 
 
 
 
 
 
 
 
 
 
 
 
 
 
 
 
 
 
 
 
 
 
 
 
 
 
 
 
 
 
 
 
 
 
 
 
 
 
 
 
 
 
 
 
 
 
 
 
 
 
 
 
 
 
 
 
 
 
 
 
 
 
 
 
 
 
 
 
 
 
 
 
 
 
 
 
 
 
 
 
 
 
 
 
 
 
 
 
 
 
 
 
 
 
 
 
 
 
 
 
 
 
 
 
 
 
 
 
 
 
 
 
 
 
 
 
 
 
 
 
 
 
 
 
 
 
 
 
 
 
 
 
 
 
 
 
 
 
 
 
 
 
 
 
 
 
 
 
 
 
 
 
 
 
 
 
 
 
 
 
 
 
 
 
 
 
 
 
 
 
 
 
 
 
 
 
 
 
 
 
 
 
 
 
 
 
 
 
 
 
 
 
 
 
 
 
 
 
 
 
 
 
 
 
 
 
 
 
 
 
 
 
 
 
 
 
 
 
 
 
 
 
 
 
 
 
 
 
 
 
 
 
 
 
 
 
 
 
 
 
 
 
 
 
 
 
 
 
 
 
 
 
 
 
 
 
 
 
 
 
 
 
 
 
 
 
 
 
 
 
 
 
 
 
 
 
 
 
 
 
 
 
 
 
 
 
 
 
 
 
 
 
 
 
 
 
 
 
 
 
 
 
 
 
 
 
 
 
 
 
 
 
 
 
 
 
 
 
 
 
 
 
 
 
 
 
 
 
 
 
 
 
 
 
 
 
 
 
 
 
 
 
 
 
 
 
 
 
 
 
 
 
 
 
 
 
 
 
 
 
 
 
 
 
 
 
 
 
 
 
 
 
 
 
 
 
 
 
 
 
 
 
 
 
 
 
 
 
 
 
 
 
 
 
 
 
 
 
 
 
 
 
 
 
 
 
 
 
 
 
 
 
 
 
 
 
 
 
 
 
 
 
 
 
 
 
 
 
 
 
 
 
 
 
 
 
 
 
 
 
 
 
 
 
 
 
 
 
 
 
 
 
 
 
 
 
 
 
 
 
 
 
 
 
 
 
 
 
 
 
 
 
 
 
 
 
 
 
 
 
 
 
 
 
 
 
 
 
 
 
 
 
 
 
 
 
 
 
 
 
 
 
 
 
 
 
 
 
 
 
 
 
 
 
 
 
 
 
 
 
 
 
 
 
 
 
 
 
 
 
 
 
 
 
 
 
 
 
 
 
 
 
 
 
 
 
 
 
 
 
 
 
 
 
 
 
 
 
 
 
 
 
 
 
 
 
 
 
 
 
 
 
 
 
 
 
 
 
 
 
 
 
 
 
 
 
 
 
 
 
 
 
 
 
 
 
 
 
 
 
 
 
 
 
 
 
 
 
 
 
 
 
 
 
 
 
 
 
 
 
 
 
 
 
 
 
 
 
 
 
 
 
 
 
 
 
 
 
 
 
 
 
 
 
 
 
 
 
 
 
 
 
 
 
 
 
 
 
 
 
 
 
 
 
 
 
 
 
 
 
 
 
 
 
 
 
 
 
 
 
 
 
 
 
 
 
 
 
 
 
 
 
 
 
 
 
 
 
 
 
 
 
 
 
 
 
 
 
 
 
 
 
 
 
 
 
 
 
 
 
 
 
 
 
 
 
 
 
 
 
 
 
 
 
 
 
 
 
 
 
 
 
 
 
 
 
 
 
 
 
 
 
 
 
 
 
 
 
 
 
 
 
 
 
 
 
 
 
 
 
 
 
 
 
 
 
 
 
 
 
 
 
 
 
 
 
 
 
 
 
 
 
 
 
 
 
 
 
 
 
 
 
 
 
 
 
 
 
 
 
 
 
 
 
 
 
 
 
 
 
 
 
 
 
 
 
 
 
 
 
 
 
 
 
 
 
 
 
 
 
 
 
 
 
 
 
 
 
 
 
 
 
 
 
 
 
 
 
 
 
 
 
 
 
 
 
 
 
 
 
 
 
 
 
 
 
 
 
 
 
 
 
 
 
 
 
 
 
 
 
 
 
 
 
 
 
 
 
 
 
 
 
 
 
 
 
 
 
 
 
 
 
 
 
 
 
 
 
 
 
 
 
 
 
 
 
 
 
 
 
 
 
 
 
 
 
 
 
 
 
 
 
 
 
 
 
 
 
 
 
 
 
 
 
 
 
 
 
 
 
 
 
 
 
 
 
 
 
 
 
 
 
 
 
 
 
 
 
 
 
 
 
 
 
 
 
 
 
 
 
 
 
 
 
 
 
 
 
 
 
 
 
 
 
 
 
 
 
 
 
 
 
 
 
 
 
 
 
 
 
 
 
 
 
 
 
 
 
 
 
 
 
 
 
 
 
 
 
 
 
 
 
 
 
 
 
 
 
 
 
 
 
 
 
 
 
 
 
 
 
 
 
 
 
 
 
 
 
 
 
 
 
 
 
 
 
 
 
 
 
 
 
 
 
 
 
 
 
 
 
 
 
 
 
 
 
 
 
 
 
 
 
 
 
 
 
 
 
 
 
 
 
 
 
 
 
 
 
 
 
 
 
 
 
 
 
 
 
 
 
 
 
 
 
 
 
 
 
 
 
 
 
 
 
 
 
 
 
 
 
 
 
 
 
 
 
 
 
 
 
 
 
 
 
 
 
 
 
 
 
 
 
 
 
 
 
 
 
 
 
 
 
 
 
 
 
 
 
 
 
 
 
 
 
 
 
 
 
 
 
 
 
 
 
 
 
 
 
 
 
 
 
 
 
 
 
 
 
 
 
 
 
 
 
 
 
 
 
 
 
 
 
 
 
 
 
 
 
 
 
 
 
 
 
 
 
 
 
 
 
 
 
 
 
 
 
 
 
 
 
 
 
 
 
 
 
 
 
 
 
 
 
 
 
 
 
 
 
 
 
 
 
 
 
 
 
 
 
 
 
 
 
 
 
 
 
 
 
 
 
 
 
 
 
 
 
 
 
 
 
 
 
 
 
 
 
 
 
 
 
 
 
 
 
 
 
 
 
 
 
 
 
 
 
 
 
 
 
 
 
 
 
 
 
 
 
 
 
 
 
 
 
 
 
 
 
 
 
 
 
 
 
 
 
 
 
 
 
 
 
 
 
 
 
 
 
 
 
 
 
 
 
 
 
 
 
 
 
 
 
 
 
 
 
 
 
 
 
 
 
 
 
 
 
 
 
 
 
 
 
 
 
 
 
 
 
 
 
 
 
 
 
 
 
 
 
 
 
 
 
 
 
 
 
 
 
 
 
 
 
 
 
 
 
 
 
 
 
 
 
 
 
 
 
 
 
 
 
 
 
 
 
 
 
 
 
 
 
 
 
 
 
 
 
 
 
 
 
 
 
 
 
 
 
 
 
 
 
 
 
 
 
 
 
 
 
 
 
 
 
 
 
 
 
 
 
 
 
 
 
 
 
 
 
 
 
 
 
 
 
 
 
 
 
 
 
 
 
 
 
 
 
 
 
 
 
 
 
 
 
 
 
 
 
 
 
 
 
 
 
 
 
 
 
 
 
 
 
 
 
 
 
 
 
 
 
 
 
 
 
 
 
 
 
 
 
 
 
 
 
 
 
 
 
 
 
 
 
 
 
 
 
 
 
 
 
 
 
 
 
 
 
 
 
 
 
 
 
 
 
 
 
 
 
 
 
 
 
 
 
 
 
 
 
 
 
 
 
 
 
 
 
 
 
 
 
 
 
 
 
 
 
 
 
 
 
 
 
 
 
 
 
 
 
 
 
 
 
 
 
 
 
 
 
 
 
 
 
 
 
 
 
 
 
 
 
 
 
 
 
 
 
 
 
 
 
 
 
 
 
 
 
 
 
 
 
 
 
 
 
 
 
 
 
 
 
 
 
 
 
 
 
 
 
 
 
 
 
 
 
 
 
 
 
 
 
 
 
 
 
 
 
 
 
 
 
 
 
 
 
 
 
 
 
 
 
 
 
 
 
 
 
 
 
 
 
 
 
 
 
 
 
 
 
 
 
 
 
 
 
 
 
 
 
 
 
 
 
 
 
 
 
 
 
 
 
 
 
 
 
 
 
 
 
 
 
 
 

 

    View Table (.txt)         Total  0  1  2  3  4  5  6  8  9  10  11  13  14  15  16  17  18  19  20  21  22  23  24  25  26  27  28  29  30  31  32  41  42  43  44  45  46  47  48  49  50  51  52  53  54  56  57  58  59  60  61  62  64  65  66  67  68  69  70  71  72  73  79  80  81  82  83  84  86  87  88  89  90  91  92  93  94  95  96  97  98  99  100  101  102  103  104  105  112  113  114  115  116  117  118  120  121  122  123  124  125  126  127  128  129  130  131  132  134  135  137  138  139  140  141  142  143  144  145  146  147  148  149  150  151  152  153  154  155  157  158  159  160  161  162  163  164  166  167  168  169  170  172  173  174  175  176  177  178  179  180  181  182  183  184  185  186  187  188  189  190  191  192  193  194  195  196  197  198  199  200  201  202  203  204  205  206  208  209  210  211  213  214  215  216  217  218  219  220  221  222  223  225  226  227  228  229  230  231  234  235  236  237  238  240  241  243  244  245  246  247  249  250  251  252  253  254  255  257  258  259  260  261  262  263  264  266  267  268  269  270  271  272  273  274  275  278  279  280  281  283  284  285  286  287  288  289  290  292  293  294  295  296  297  298  299  300  301  302  303  304  305  306  307  308  309  312  314  317  318  319  321  322  323  326  327  330  331  332  333  334  335  336  337  338  339  340  341  343  344  345  348  349  350  351  352  353  354  355  356  357  358  359  360  361  362  363  365  366  368  369  371  372  373  374  375  376  377  378  379  380  381  382  383  384  385  386  387  389  390  391  392  393  394  395  396  397  398  399  400  401  402  403  405  406  410  411  412  413  415  416  417  418  420  438  439  440    Legend  Taxonomy  count  %  %  %  %  %  %  %  %  %  %  %  %  %  %  %  %  %  %  %  %  %  %  %  %  %  %  %  %  %  %  %  %  %  %  %  %  %  %  %  %  %  %  %  %  %  %  %  %  %  %  %  %  %  %  %  %  %  %  %  %  %  %  %  %  %  %  %  %  %  %  %  %  %  %  %  %  %  %  %  %  %  %  %  %  %  %  %  %  %  %  %  %  %  %  %  %  %  %  %  %  %  %  %  %  %  %  %  %  %  %  %  %  %  %  %  %  %  %  %  %  %  %  %  %  %  %  %  %  %  %  %  %  %  %  %  %  %  %  %  %  %  %  %  %  %  %  %  %  %  %  %  %  %  %  %  %  %  %  %  %  %  %  %  %  %  %  %  %  %  %  %  %  %  %  %  %  %  %  %  %  %  %  %  %  %  %  %  %  %  %  %  %  %  %  %  %  %  %  %  %  %  %  %  %  %  %  %  %  %  %  %  %  %  %  %  %  %  %  %  %  %  %  %  %  %  %  %  %  %  %  %  %  %  %  %  %  %  %  %  %  %  %  %  %  %  %  %  %  %  %  %  %  %  %  %  %  %  %  %  %  %  %  %  %  %  %  %  %  %  %  %  %  %  %  %  %  %  %  %  %  %  %  %  %  %  %  %  %  %  %  %  %  %  %  %  %  %  %  %  %  %  %  %  %  %  %  %  %  %  %  %  %  %  %  %  %  %  %  %  %  %  %  %  %  %  %  %  %  %  %  %  %  %  %  %  %  %  %  %  %  %  %  %  %  %  %  %  %  %  %  %  %  %  %  %  %  %  %        k__Archaea;p__Crenarchaeota; c__Sd-NA       3    0.0%    0.0%    0.0%    0.0%    0.0%    0.0%    0.0%    0.0%    0.0%    0.0%    0.0%    0.0%    0.0%    0.0%    0.0%    0.0%    0.0%    0.0%    0.0%    0.0%    0.0%    0.0%    0.0%    0.0%    0.0%    0.0%    0.0%    0.0%    0.0%    0.0%    0.0%    0.0%    0.0%    0.0%    0.0%    0.0%    0.0%    0.0%    0.0%    0.0%    0.0%    0.0%    0.0%    0.0%    0.0%    0.0%    0.0%    0.0%    0.0%    0.0%    0.0%    0.0%    0.0%    0.0%    0.0%    0.0%    0.0%    0.0%    0.0%    0.0%    0.0%    0.0%    0.0%    0.0%    0.0%    0.0%    0.0%    0.0%    0.0%    0.0%    0.0%    0.0%    0.0%    0.0%    0.0%    0.0%    0.0%    0.0%    0.0%    0.0%    0.0%    0.0%    0.0%    0.0%    0.0%    0.0%    0.0%    0.0%    0.0%    0.0%    0.0%    0.0%    0.0%    0.0%    0.0%    0.0%    0.0%    0.0%    0.0%    0.0%    0.0%    0.0%    0.0%    0.0%    0.0%    0.0%    0.0%    0.0%    0.0%    0.0%    0.0%    0.0%    0.0%    0.0%    0.0%    0.0%    0.0%    0.0%    0.0%    0.0%    0.0%    0.0%    0.0%    0.0%    0.0%    0.0%    0.0%    0.0%    0.0%    0.0%    0.0%    0.0%    0.0%    0.0%    0.0%    0.0%    0.0%    0.0%    0.0%    0.0%    0.0%    0.0%    0.0%    0.0%    0.0%    0.0%    0.0%    0.0%    0.0%    0.0%    0.0%    0.0%    0.0%    0.0%    0.0%    0.0%    0.0%    0.0%    0.0%    0.0%    0.0%    0.0%    0.0%    0.0%    0.0%    0.0%    0.0%    0.0%    0.0%    0.0%    0.0%    0.0%    0.0%    0.0%    0.0%    0.0%    0.0%    0.0%    0.0%    0.0%    0.0%    0.0%    0.0%    0.0%    0.0%    0.0%    0.0%    0.0%    0.0%    0.0%    0.0%    0.0%    0.0%    0.0%    0.0%    0.0%    0.0%    0.0%    0.0%    0.0%    0.0%    0.0%    0.0%    0.0%    0.0%    0.0%    0.0%    0.0%    0.0%    0.0%    0.0%    0.0%    0.0%    0.0%    0.0%    0.0%    0.0%    0.0%    0.0%    0.0%    0.0%    0.0%    0.0%    0.0%    0.0%    0.0%    0.0%    0.0%    0.0%    0.0%    0.0%    0.0%    0.0%    0.0%    0.0%    0.0%    0.0%    0.0%    0.0%    0.0%    0.0%    0.0%    0.0%    0.0%    0.0%    0.0%    0.0%    0.0%    0.0%    0.0%    0.0%    0.0%    0.0%    0.0%    0.0%    0.0%    0.0%    0.0%    0.0%    0.0%    0.0%    0.0%    0.0%    0.0%    0.0%    0.0%    0.0%    0.0%    0.0%    0.0%    0.0%    0.0%    0.0%    0.0%    0.0%    0.0%    0.0%    0.0%    0.0%    0.0%    0.0%    0.0%    0.0%    0.0%    0.0%    0.0%    0.0%    0.0%    0.0%    0.0%    0.0%    0.0%    0.0%    0.0%    0.0%    0.0%    0.0%    0.0%    0.0%    0.0%    0.0%    0.0%    0.0%    0.0%    0.0%    0.0%    0.0%    0.0%    0.0%    0.0%    0.0%    0.0%    0.0%    0.0%    0.0%    0.0%    0.0%    0.0%    0.0%    0.0%    0.0%    0.0%    0.0%    0.0%    0.0%    0.0%    0.0%    0.0%    0.0%    0.0%    0.0%    0.0%    0.0%    0.0%    0.0%    0.0%    0.0%    0.0%    0.0%    0.0%    0.0%    0.0%    0.0%    0.0%    0.0%    0.0%    0.0%    0.0%    0.0%    0.0%    0.0%    0.0%    0.0%    0.0%    0.0%    0.0%    0.0%    0.0%        k__Archaea;p__Crenarchaeota; c__Thaumarchaeota    3915    0.0%    0.0%    0.0%    0.0%    0.0%    0.0%    0.0%    0.0%    0.0%    0.0%    0.0%    0.0%    0.0%    0.0%    0.0%    0.0%    0.0%    0.0%    0.0%    0.0%    0.0%    0.0%    0.0%    0.0%    0.0%    0.0%    0.0%    0.0%    0.0%    0.0%    0.0%    0.0%    0.0%    0.0%    0.0%    0.0%    0.0%    0.0%    0.0%    0.1%    0.0%    0.0%    0.0%    0.0%    0.0%    0.0%    0.0%    0.0%    0.0%    0.0%    0.0%    0.0%    0.0%    0.0%    0.1%    0.0%    0.0%    0.0%    0.0%    0.1%    0.0%    0.0%    0.0%    0.1%    0.0%    0.1%    0.0%    0.1%    0.0%    0.1%    0.0%    0.0%    0.0%    0.0%    0.0%    0.0%    0.0%    0.0%    0.0%    0.0%    0.0%    0.0%    0.0%    0.0%    0.0%    0.0%    0.0%    0.0%    0.0%    0.0%    0.0%    0.0%    0.0%    0.0%    0.0%    0.0%    0.1%    0.0%    0.0%    0.0%    0.1%    0.0%    0.0%    0.0%    0.0%    0.0%    0.1%    0.0%    0.0%    0.0%    0.0%    0.1%    0.0%    0.0%    0.0%    0.0%    0.0%    0.0%    0.0%    0.2%    0.0%    0.0%    0.1%    0.1%    0.0%    0.1%    0.1%    0.0%    0.1%    0.2%    0.1%    0.0%    0.0%    0.0%    0.2%    0.0%    0.0%    0.0%    0.0%    0.0%    0.0%    0.0%    0.0%    0.0%    0.1%    0.0%    0.0%    0.0%    0.0%    0.0%    0.0%    0.0%    0.1%    0.0%    0.0%    0.0%    0.1%    0.0%    0.0%    0.1%    0.3%    0.1%    0.1%    0.0%    0.0%    0.0%    0.1%    0.0%    0.0%    0.0%    0.0%    0.1%    0.0%    0.0%    0.0%    0.0%    0.0%    0.0%    0.0%    0.0%    0.0%    0.0%    0.0%    0.0%    0.0%    0.0%    0.0%    0.0%    0.0%    0.0%    0.0%    0.0%    0.0%    0.0%    0.0%    0.0%    0.0%    0.0%    0.0%    0.0%    0.0%    0.0%    0.0%    0.1%    0.1%    0.0%    0.0%    0.0%    0.1%    0.1%    0.0%    0.0%    0.0%    0.0%    0.0%    0.0%    0.0%    0.0%    0.0%    0.0%    0.0%    0.0%    0.0%    0.0%    0.0%    0.0%    0.0%    0.0%    0.0%    0.0%    0.0%    0.0%    0.1%    0.0%    0.1%    0.0%    0.1%    0.0%    0.0%    0.0%    0.0%    0.0%    0.0%    0.0%    0.0%    0.0%    0.0%    0.0%    0.0%    0.0%    0.1%    0.1%    0.0%    0.0%    0.0%    0.0%    0.0%    0.0%    0.0%    0.0%    0.1%    0.0%    0.0%    0.0%    0.0%    0.0%    0.0%    0.0%    0.0%    0.0%    0.0%    0.0%    0.0%    0.0%    0.0%    0.0%    0.0%    0.0%    0.0%    0.0%    0.0%    0.0%    0.0%    0.0%    0.0%    0.0%    0.0%    0.1%    0.0%    0.0%    0.0%    0.0%    0.0%    0.1%    0.1%    0.0%    0.0%    0.0%    0.0%    0.0%    0.0%    0.0%    0.0%    0.0%    0.0%    0.0%    0.0%    0.0%    0.0%    0.0%    0.0%    0.0%    0.0%    0.0%    0.0%    0.0%    0.0%    0.0%    0.0%    0.0%    0.0%    0.0%    0.0%    0.0%    0.0%    0.0%    0.0%    0.0%    0.0%    0.0%    0.0%    0.0%    0.0%    0.0%    0.0%    0.0%    0.0%    0.0%    0.0%    0.0%    0.0%    0.0%    0.0%    0.0%    0.0%    0.0%    0.0%    0.0%    0.0%    0.0%    0.0%    0.0%    0.0%    0.0%    0.0%    0.0%    0.0%    0.0%        k__Archaea;p__Euryarchaeota; c__Halobacteria      48    0.0%    0.0%    0.0%    0.0%    0.0%    0.0%    0.0%    0.0%    0.0%    0.0%    0.0%    0.0%    0.0%    0.0%    0.0%    0.0%    0.0%    0.0%    0.0%    0.0%    0.0%    0.0%    0.0%    0.0%    0.0%    0.0%    0.0%    0.0%    0.0%    0.0%    0.0%    0.0%    0.0%    0.0%    0.0%    0.0%    0.0%    0.0%    0.0%    0.0%    0.0%    0.0%    0.0%    0.0%    0.0%    0.0%    0.0%    0.0%    0.0%    0.0%    0.0%    0.0%    0.0%    0.0%    0.0%    0.0%    0.0%    0.0%    0.0%    0.0%    0.0%    0.0%    0.0%    0.0%    0.0%    0.0%    0.0%    0.0%    0.0%    0.0%    0.0%    0.0%    0.0%    0.0%    0.0%    0.0%    0.0%    0.0%    0.0%    0.0%    0.0%    0.0%    0.0%    0.0%    0.0%    0.0%    0.0%    0.0%    0.0%    0.0%    0.0%    0.0%    0.0%    0.0%    0.0%    0.0%    0.0%    0.0%    0.0%    0.0%    0.0%    0.0%    0.0%    0.0%    0.0%    0.0%    0.0%    0.0%    0.0%    0.0%    0.0%    0.0%    0.0%    0.0%    0.0%    0.0%    0.0%    0.0%    0.0%    0.0%    0.0%    0.0%    0.0%    0.0%    0.0%    0.0%    0.0%    0.0%    0.0%    0.0%    0.0%    0.0%    0.0%    0.0%    0.0%    0.0%    0.0%    0.0%    0.0%    0.0%    0.0%    0.0%    0.0%    0.0%    0.0%    0.0%    0.0%    0.0%    0.0%    0.0%    0.0%    0.0%    0.0%    0.0%    0.0%    0.0%    0.0%    0.0%    0.0%    0.0%    0.0%    0.0%    0.0%    0.0%    0.0%    0.0%    0.0%    0.0%    0.0%    0.0%    0.0%    0.0%    0.0%    0.0%    0.0%    0.0%    0.0%    0.0%    0.0%    0.0%    0.0%    0.0%    0.0%    0.0%    0.0%    0.0%    0.0%    0.0%    0.0%    0.0%    0.0%    0.0%    0.0%    0.0%    0.0%    0.0%    0.0%    0.0%    0.0%    0.0%    0.0%    0.0%    0.0%    0.0%    0.0%    0.0%    0.0%    0.0%    0.0%    0.0%    0.0%    0.0%    0.0%    0.0%    0.0%    0.0%    0.0%    0.0%    0.0%    0.0%    0.0%    0.0%    0.0%    0.0%    0.0%    0.0%    0.0%    0.0%    0.0%    0.0%    0.0%    0.0%    0.0%    0.0%    0.0%    0.0%    0.0%    0.0%    0.0%    0.0%    0.0%    0.0%    0.0%    0.0%    0.0%    0.0%    0.0%    0.0%    0.0%    0.0%    0.0%    0.0%    0.0%    0.0%    0.0%    0.0%    0.0%    0.0%    0.0%    0.0%    0.0%    0.0%    0.0%    0.0%    0.0%    0.0%    0.0%    0.0%    0.0%    0.0%    0.0%    0.0%    0.0%    0.0%    0.0%    0.0%    0.0%    0.0%    0.0%    0.0%    0.0%    0.0%    0.0%    0.0%    0.0%    0.0%    0.0%    0.0%    0.0%    0.0%    0.0%    0.0%    0.0%    0.0%    0.0%    0.0%    0.0%    0.0%    0.0%    0.0%    0.0%    0.0%    0.0%    0.0%    0.0%    0.0%    0.0%    0.0%    0.0%    0.0%    0.0%    0.0%    0.0%    0.0%    0.0%    0.0%    0.0%    0.0%    0.0%    0.0%    0.0%    0.0%    0.0%    0.0%    0.0%    0.0%    0.0%    0.0%    0.0%    0.0%    0.0%    0.0%    0.0%    0.0%    0.0%    0.0%    0.0%    0.0%    0.0%    0.0%    0.0%    0.0%    0.0%    0.0%    0.0%    0.0%    0.0%    0.0%    0.0%    0.0%    0.0%    0.0%    0.0%    0.0%    0.0%    0.0%    0.0%    0.0%        k__Archaea;p__Euryarchaeota; c__Methanobacteria     137    0.0%    0.0%    0.0%    0.0%    0.0%    0.0%    0.0%    0.0%    0.0%    0.0%    0.0%    0.0%    0.0%    0.0%    0.0%    0.0%    0.0%    0.0%    0.0%    0.0%    0.0%    0.0%    0.0%    0.0%    0.0%    0.0%    0.0%    0.0%    0.0%    0.0%    0.0%    0.0%    0.0%    0.0%    0.0%    0.0%    0.0%    0.0%    0.0%    0.0%    0.0%    0.0%    0.0%    0.0%    0.0%    0.0%    0.0%    0.0%    0.0%    0.0%    0.0%    0.0%    0.0%    0.0%    0.0%    0.0%    0.0%    0.0%    0.0%    0.0%    0.0%    0.0%    0.0%    0.0%    0.0%    0.0%    0.0%    0.0%    0.0%    0.0%    0.0%    0.0%    0.0%    0.0%    0.0%    0.0%    0.0%    0.0%    0.0%    0.0%    0.0%    0.0%    0.0%    0.0%    0.1%    0.0%    0.0%    0.0%    0.0%    0.0%    0.0%    0.0%    0.0%    0.0%    0.0%    0.0%    0.0%    0.0%    0.0%    0.0%    0.0%    0.0%    0.0%    0.0%    0.0%    0.0%    0.0%    0.0%    0.0%    0.0%    0.0%    0.0%    0.0%    0.0%    0.0%    0.0%    0.0%    0.0%    0.0%    0.0%    0.0%    0.0%    0.0%    0.0%    0.0%    0.0%    0.0%    0.0%    0.0%    0.0%    0.0%    0.0%    0.0%    0.0%    0.0%    0.0%    0.0%    0.0%    0.0%    0.0%    0.0%    0.0%    0.0%    0.0%    0.0%    0.0%    0.0%    0.0%    0.0%    0.0%    0.0%    0.0%    0.0%    0.0%    0.0%    0.0%    0.0%    0.0%    0.0%    0.0%    0.0%    0.0%    0.0%    0.0%    0.0%    0.0%    0.0%    0.0%    0.0%    0.0%    0.0%    0.0%    0.0%    0.0%    0.0%    0.0%    0.0%    0.0%    0.0%    0.0%    0.0%    0.0%    0.0%    0.0%    0.0%    0.0%    0.0%    0.0%    0.0%    0.0%    0.0%    0.0%    0.0%    0.0%    0.0%    0.0%    0.0%    0.0%    0.0%    0.0%    0.0%    0.0%    0.0%    0.0%    0.0%    0.0%    0.0%    0.0%    0.0%    0.0%    0.0%    0.0%    0.0%    0.0%    0.0%    0.0%    0.0%    0.0%    0.0%    0.0%    0.0%    0.0%    0.0%    0.0%    0.0%    0.0%    0.0%    0.0%    0.0%    0.0%    0.0%    0.0%    0.0%    0.0%    0.0%    0.0%    0.0%    0.0%    0.0%    0.0%    0.0%    0.0%    0.0%    0.0%    0.0%    0.0%    0.0%    0.0%    0.0%    0.0%    0.0%    0.0%    0.0%    0.0%    0.0%    0.0%    0.0%    0.0%    0.0%    0.0%    0.0%    0.0%    0.0%    0.0%    0.0%    0.0%    0.0%    0.0%    0.0%    0.0%    0.0%    0.0%    0.0%    0.0%    0.0%    0.0%    0.0%    0.0%    0.0%    0.0%    0.0%    0.0%    0.0%    0.0%    0.0%    0.0%    0.0%    0.0%    0.0%    0.0%    0.0%    0.0%    0.0%    0.0%    0.0%    0.0%    0.0%    0.0%    0.0%    0.0%    0.0%    0.0%    0.0%    0.0%    0.0%    0.0%    0.0%    0.0%    0.0%    0.0%    0.0%    0.0%    0.0%    0.0%    0.0%    0.0%    0.0%    0.0%    0.0%    0.0%    0.0%    0.0%    0.0%    0.0%    0.0%    0.0%    0.0%    0.0%    0.0%    0.0%    0.0%    0.0%    0.0%    0.0%    0.0%    0.0%    0.0%    0.0%    0.0%    0.0%    0.0%    0.0%    0.0%    0.0%    0.0%    0.0%    0.0%    0.0%    0.0%    0.0%    0.0%    0.0%    0.0%    0.0%    0.0%    0.0%    0.0%    0.0%        k__Archaea;p__Euryarchaeota; c__Methanomicrobia      13    0.0%    0.0%    0.0%    0.0%    0.0%    0.0%    0.0%    0.0%    0.0%    0.0%    0.0%    0.0%    0.0%    0.0%    0.0%    0.0%    0.0%    0.0%    0.0%    0.0%    0.0%    0.0%    0.0%    0.0%    0.0%    0.0%    0.0%    0.0%    0.0%    0.0%    0.0%    0.0%    0.0%    0.0%    0.0%    0.0%    0.0%    0.0%    0.0%    0.0%    0.0%    0.0%    0.0%    0.0%    0.0%    0.0%    0.0%    0.0%    0.0%    0.0%    0.0%    0.0%    0.0%    0.0%    0.0%    0.0%    0.0%    0.0%    0.0%    0.0%    0.0%    0.0%    0.0%    0.0%    0.0%    0.0%    0.0%    0.0%    0.0%    0.0%    0.0%    0.0%    0.0%    0.0%    0.0%    0.0%    0.0%    0.0%    0.0%    0.0%    0.0%    0.0%    0.0%    0.0%    0.0%    0.0%    0.0%    0.0%    0.0%    0.0%    0.0%    0.0%    0.0%    0.0%    0.0%    0.0%    0.0%    0.0%    0.0%    0.0%    0.0%    0.0%    0.0%    0.0%    0.0%    0.0%    0.0%    0.0%    0.0%    0.0%    0.0%    0.0%    0.0%    0.0%    0.0%    0.0%    0.0%    0.0%    0.0%    0.0%    0.0%    0.0%    0.0%    0.0%    0.0%    0.0%    0.0%    0.0%    0.0%    0.0%    0.0%    0.0%    0.0%    0.0%    0.0%    0.0%    0.0%    0.0%    0.0%    0.0%    0.0%    0.0%    0.0%    0.0%    0.0%    0.0%    0.0%    0.0%    0.0%    0.0%    0.0%    0.0%    0.0%    0.0%    0.0%    0.0%    0.0%    0.0%    0.0%    0.0%    0.0%    0.0%    0.0%    0.0%    0.0%    0.0%    0.0%    0.0%    0.0%    0.0%    0.0%    0.0%    0.0%    0.0%    0.0%    0.0%    0.0%    0.0%    0.0%    0.0%    0.0%    0.0%    0.0%    0.0%    0.0%    0.0%    0.0%    0.0%    0.0%    0.0%    0.0%    0.0%    0.0%    0.0%    0.0%    0.0%    0.0%    0.0%    0.0%    0.0%    0.0%    0.0%    0.0%    0.0%    0.0%    0.0%    0.0%    0.0%    0.0%    0.0%    0.0%    0.0%    0.0%    0.0%    0.0%    0.0%    0.0%    0.0%    0.0%    0.0%    0.0%    0.0%    0.0%    0.0%    0.0%    0.0%    0.0%    0.0%    0.0%    0.0%    0.0%    0.0%    0.0%    0.0%    0.0%    0.0%    0.0%    0.0%    0.0%    0.0%    0.0%    0.0%    0.0%    0.0%    0.0%    0.0%    0.0%    0.0%    0.0%    0.0%    0.0%    0.0%    0.0%    0.0%    0.0%    0.0%    0.0%    0.0%    0.0%    0.0%    0.0%    0.0%    0.0%    0.0%    0.0%    0.0%    0.0%    0.0%    0.0%    0.0%    0.0%    0.0%    0.0%    0.0%    0.0%    0.0%    0.0%    0.0%    0.0%    0.0%    0.0%    0.0%    0.0%    0.0%    0.0%    0.0%    0.0%    0.0%    0.0%    0.0%    0.0%    0.0%    0.0%    0.0%    0.0%    0.0%    0.0%    0.0%    0.0%    0.0%    0.0%    0.0%    0.0%    0.0%    0.0%    0.0%    0.0%    0.0%    0.0%    0.0%    0.0%    0.0%    0.0%    0.0%    0.0%    0.0%    0.0%    0.0%    0.0%    0.0%    0.0%    0.0%    0.0%    0.0%    0.0%    0.0%    0.0%    0.0%    0.0%    0.0%    0.0%    0.0%    0.0%    0.0%    0.0%    0.0%    0.0%    0.0%    0.0%    0.0%    0.0%    0.0%    0.0%    0.0%    0.0%    0.0%    0.0%    0.0%    0.0%    0.0%    0.0%    0.0%    0.0%    0.0%    0.0%    0.0%    0.0%    0.0%        k__Archaea;p__Euryarchaeota; c__Thermoplasmata       6    0.0%    0.0%    0.0%    0.0%    0.0%    0.0%    0.0%    0.0%    0.0%    0.0%    0.0%    0.0%    0.0%    0.0%    0.0%    0.0%    0.0%    0.0%    0.0%    0.0%    0.0%    0.0%    0.0%    0.0%    0.0%    0.0%    0.0%    0.0%    0.0%    0.0%    0.0%    0.0%    0.0%    0.0%    0.0%    0.0%    0.0%    0.0%    0.0%    0.0%    0.0%    0.0%    0.0%    0.0%    0.0%    0.0%    0.0%    0.0%    0.0%    0.0%    0.0%    0.0%    0.0%    0.0%    0.0%    0.0%    0.0%    0.0%    0.0%    0.0%    0.0%    0.0%    0.0%    0.0%    0.0%    0.0%    0.0%    0.0%    0.0%    0.0%    0.0%    0.0%    0.0%    0.0%    0.0%    0.0%    0.0%    0.0%    0.0%    0.0%    0.0%    0.0%    0.0%    0.0%    0.0%    0.0%    0.0%    0.0%    0.0%    0.0%    0.0%    0.0%    0.0%    0.0%    0.0%    0.0%    0.0%    0.0%    0.0%    0.0%    0.0%    0.0%    0.0%    0.0%    0.0%    0.0%    0.0%    0.0%    0.0%    0.0%    0.0%    0.0%    0.0%    0.0%    0.0%    0.0%    0.0%    0.0%    0.0%    0.0%    0.0%    0.0%    0.0%    0.0%    0.0%    0.0%    0.0%    0.0%    0.0%    0.0%    0.0%    0.0%    0.0%    0.0%    0.0%    0.0%    0.0%    0.0%    0.0%    0.0%    0.0%    0.0%    0.0%    0.0%    0.0%    0.0%    0.0%    0.0%    0.0%    0.0%    0.0%    0.0%    0.0%    0.0%    0.0%    0.0%    0.0%    0.0%    0.0%    0.0%    0.0%    0.0%    0.0%    0.0%    0.0%    0.0%    0.0%    0.0%    0.0%    0.0%    0.0%    0.0%    0.0%    0.0%    0.0%    0.0%    0.0%    0.0%    0.0%    0.0%    0.0%    0.0%    0.0%    0.0%    0.0%    0.0%    0.0%    0.0%    0.0%    0.0%    0.0%    0.0%    0.0%    0.0%    0.0%    0.0%    0.0%    0.0%    0.0%    0.0%    0.0%    0.0%    0.0%    0.0%    0.0%    0.0%    0.0%    0.0%    0.0%    0.0%    0.0%    0.0%    0.0%    0.0%    0.0%    0.0%    0.0%    0.0%    0.0%    0.0%    0.0%    0.0%    0.0%    0.0%    0.0%    0.0%    0.0%    0.0%    0.0%    0.0%    0.0%    0.0%    0.0%    0.0%    0.0%    0.0%    0.0%    0.0%    0.0%    0.0%    0.0%    0.0%    0.0%    0.0%    0.0%    0.0%    0.0%    0.0%    0.0%    0.0%    0.0%    0.0%    0.0%    0.0%    0.0%    0.0%    0.0%    0.0%    0.0%    0.0%    0.0%    0.0%    0.0%    0.0%    0.0%    0.0%    0.0%    0.0%    0.0%    0.0%    0.0%    0.0%    0.0%    0.0%    0.0%    0.0%    0.0%    0.0%    0.0%    0.0%    0.0%    0.0%    0.0%    0.0%    0.0%    0.0%    0.0%    0.0%    0.0%    0.0%    0.0%    0.0%    0.0%    0.0%    0.0%    0.0%    0.0%    0.0%    0.0%    0.0%    0.0%    0.0%    0.0%    0.0%    0.0%    0.0%    0.0%    0.0%    0.0%    0.0%    0.0%    0.2%    0.0%    0.0%    0.0%    0.0%    0.0%    0.0%    0.0%    0.0%    0.0%    0.0%    0.0%    0.0%    0.0%    0.0%    0.0%    0.0%    0.0%    0.0%    0.0%    0.0%    0.0%    0.0%    0.0%    0.0%    0.0%    0.0%    0.0%    0.0%    0.0%    0.0%    0.0%    0.0%    0.0%    0.0%    0.0%    0.0%    0.0%    0.0%    0.0%    0.0%    0.0%    0.0%    0.0%    0.0%    0.0%    0.0%        k__Bacteria;p__; c__       6    0.0%    0.0%    0.0%    0.0%    0.0%    0.0%    0.0%    0.0%    0.0%    0.0%    0.0%    0.0%    0.0%    0.0%    0.0%    0.0%    0.0%    0.0%    0.0%    0.0%    0.0%    0.0%    0.0%    0.0%    0.0%    0.0%    0.0%    0.0%    0.0%    0.0%    0.0%    0.0%    0.0%    0.0%    0.0%    0.0%    0.0%    0.0%    0.0%    0.0%    0.0%    0.0%    0.0%    0.0%    0.0%    0.0%    0.0%    0.0%    0.0%    0.0%    0.0%    0.0%    0.0%    0.0%    0.0%    0.0%    0.0%    0.0%    0.0%    0.0%    0.0%    0.0%    0.0%    0.0%    0.0%    0.0%    0.0%    0.0%    0.0%    0.0%    0.0%    0.0%    0.0%    0.0%    0.0%    0.0%    0.0%    0.0%    0.0%    0.0%    0.0%    0.0%    0.0%    0.0%    0.0%    0.0%    0.0%    0.0%    0.0%    0.0%    0.0%    0.0%    0.0%    0.0%    0.0%    0.0%    0.0%    0.0%    0.0%    0.0%    0.0%    0.0%    0.0%    0.0%    0.0%    0.0%    0.0%    0.0%    0.0%    0.0%    0.0%    0.0%    0.0%    0.0%    0.0%    0.0%    0.0%    0.0%    0.0%    0.0%    0.0%    0.0%    0.0%    0.0%    0.0%    0.0%    0.0%    0.0%    0.0%    0.0%    0.0%    0.0%    0.0%    0.0%    0.0%    0.0%    0.0%    0.0%    0.0%    0.0%    0.0%    0.0%    0.0%    0.0%    0.0%    0.0%    0.0%    0.0%    0.0%    0.0%    0.0%    0.0%    0.0%    0.0%    0.0%    0.0%    0.0%    0.0%    0.0%    0.0%    0.0%    0.0%    0.0%    0.0%    0.0%    0.0%    0.0%    0.0%    0.0%    0.0%    0.0%    0.0%    0.0%    0.0%    0.0%    0.0%    0.0%    0.0%    0.0%    0.0%    0.0%    0.0%    0.0%    0.0%    0.0%    0.0%    0.0%    0.0%    0.0%    0.0%    0.0%    0.0%    0.0%    0.0%    0.0%    0.0%    0.0%    0.0%    0.0%    0.0%    0.0%    0.0%    0.0%    0.0%    0.0%    0.0%    0.0%    0.0%    0.0%    0.0%    0.0%    0.0%    0.0%    0.0%    0.0%    0.0%    0.0%    0.0%    0.0%    0.0%    0.0%    0.0%    0.0%    0.0%    0.0%    0.0%    0.0%    0.0%    0.0%    0.0%    0.0%    0.0%    0.0%    0.0%    0.0%    0.0%    0.0%    0.0%    0.0%    0.0%    0.0%    0.0%    0.0%    0.0%    0.0%    0.0%    0.0%    0.0%    0.0%    0.0%    0.0%    0.0%    0.0%    0.0%    0.0%    0.0%    0.0%    0.0%    0.0%    0.0%    0.0%    0.0%    0.0%    0.0%    0.0%    0.0%    0.0%    0.0%    0.0%    0.0%    0.0%    0.0%    0.0%    0.0%    0.0%    0.0%    0.0%    0.0%    0.0%    0.0%    0.0%    0.0%    0.0%    0.0%    0.0%    0.0%    0.0%    0.0%    0.0%    0.0%    0.0%    0.0%    0.0%    0.0%    0.0%    0.0%    0.0%    0.0%    0.0%    0.0%    0.0%    0.0%    0.0%    0.0%    0.0%    0.0%    0.0%    0.0%    0.0%    0.0%    0.0%    0.0%    0.0%    0.0%    0.0%    0.0%    0.0%    0.0%    0.0%    0.0%    0.0%    0.0%    0.0%    0.0%    0.0%    0.0%    0.0%    0.0%    0.0%    0.0%    0.0%    0.0%    0.0%    0.0%    0.0%    0.0%    0.0%    0.0%    0.0%    0.0%    0.0%    0.0%    0.0%    0.0%    0.0%    0.0%    0.0%    0.0%    0.0%    0.0%    0.0%    0.0%    0.0%    0.0%    0.0%    0.0%    0.0%    0.0%        k__Bacteria;p__ABY1_OD1; c__       0    0.0%    0.0%    0.0%    0.0%    0.0%    0.0%    0.0%    0.0%    0.0%    0.0%    0.0%    0.0%    0.0%    0.0%    0.0%    0.0%    0.0%    0.0%    0.0%    0.0%    0.0%    0.0%    0.0%    0.0%    0.0%    0.0%    0.0%    0.0%    0.0%    0.0%    0.0%    0.0%    0.0%    0.0%    0.0%    0.0%    0.0%    0.0%    0.0%    0.0%    0.0%    0.0%    0.0%    0.0%    0.0%    0.0%    0.0%    0.0%    0.0%    0.0%    0.0%    0.0%    0.0%    0.0%    0.0%    0.0%    0.0%    0.0%    0.0%    0.0%    0.0%    0.0%    0.0%    0.0%    0.0%    0.0%    0.0%    0.0%    0.0%    0.0%    0.0%    0.0%    0.0%    0.0%    0.0%    0.0%    0.0%    0.0%    0.0%    0.0%    0.0%    0.0%    0.0%    0.0%    0.0%    0.0%    0.0%    0.0%    0.0%    0.0%    0.0%    0.0%    0.0%    0.0%    0.0%    0.0%    0.0%    0.0%    0.0%    0.0%    0.0%    0.0%    0.0%    0.0%    0.0%    0.0%    0.0%    0.0%    0.0%    0.0%    0.0%    0.0%    0.0%    0.0%    0.0%    0.0%    0.0%    0.0%    0.0%    0.0%    0.0%    0.0%    0.0%    0.0%    0.0%    0.0%    0.0%    0.0%    0.0%    0.0%    0.0%    0.0%    0.0%    0.0%    0.0%    0.0%    0.0%    0.0%    0.0%    0.0%    0.0%    0.0%    0.0%    0.0%    0.0%    0.0%    0.0%    0.0%    0.0%    0.0%    0.0%    0.0%    0.0%    0.0%    0.0%    0.0%    0.0%    0.0%    0.0%    0.0%    0.0%    0.0%    0.0%    0.0%    0.0%    0.0%    0.0%    0.0%    0.0%    0.0%    0.0%    0.0%    0.0%    0.0%    0.0%    0.0%    0.0%    0.0%    0.0%    0.0%    0.0%    0.0%    0.0%    0.0%    0.0%    0.0%    0.0%    0.0%    0.0%    0.0%    0.0%    0.0%    0.0%    0.0%    0.0%    0.0%    0.0%    0.0%    0.0%    0.0%    0.0%    0.0%    0.0%    0.0%    0.0%    0.0%    0.0%    0.0%    0.0%    0.0%    0.0%    0.0%    0.0%    0.0%    0.0%    0.0%    0.0%    0.0%    0.0%    0.0%    0.0%    0.0%    0.0%    0.0%    0.0%    0.0%    0.0%    0.0%    0.0%    0.0%    0.0%    0.0%    0.0%    0.0%    0.0%    0.0%    0.0%    0.0%    0.0%    0.0%    0.0%    0.0%    0.0%    0.0%    0.0%    0.0%    0.0%    0.0%    0.0%    0.0%    0.0%    0.0%    0.0%    0.0%    0.0%    0.0%    0.0%    0.0%    0.0%    0.0%    0.0%    0.0%    0.0%    0.0%    0.0%    0.0%    0.0%    0.0%    0.0%    0.0%    0.0%    0.0%    0.0%    0.0%    0.0%    0.0%    0.0%    0.0%    0.0%    0.0%    0.0%    0.0%    0.0%    0.0%    0.0%    0.0%    0.0%    0.0%    0.0%    0.0%    0.0%    0.0%    0.0%    0.0%    0.0%    0.0%    0.0%    0.0%    0.0%    0.0%    0.0%    0.0%    0.0%    0.0%    0.0%    0.0%    0.0%    0.0%    0.0%    0.0%    0.0%    0.0%    0.0%    0.0%    0.0%    0.0%    0.0%    0.0%    0.0%    0.0%    0.0%    0.0%    0.0%    0.0%    0.0%    0.0%    0.0%    0.0%    0.0%    0.0%    0.0%    0.0%    0.0%    0.0%    0.0%    0.0%    0.0%    0.0%    0.0%    0.0%    0.0%    0.0%    0.0%    0.0%    0.0%    0.0%    0.0%    0.0%    0.0%    0.0%    0.0%    0.0%    0.0%    0.0%    0.0%    0.0%    0.0%    0.0%        k__Bacteria;p__AD3; c__ABS-6      19    0.0%    0.0%    0.0%    0.0%    0.0%    0.0%    0.0%    0.0%    0.0%    0.0%    0.0%    0.0%    0.0%    0.0%    0.0%    0.0%    0.0%    0.0%    0.0%    0.0%    0.0%    0.0%    0.0%    0.0%    0.0%    0.0%    0.0%    0.0%    0.0%    0.0%    0.0%    0.0%    0.0%    0.0%    0.0%    0.0%    0.0%    0.0%    0.0%    0.0%    0.0%    0.0%    0.0%    0.0%    0.0%    0.0%    0.0%    0.0%    0.0%    0.0%    0.0%    0.0%    0.0%    0.0%    0.0%    0.0%    0.0%    0.0%    0.0%    0.0%    0.0%    0.0%    0.0%    0.0%    0.0%    0.0%    0.0%    0.0%    0.0%    0.0%    0.0%    0.0%    0.0%    0.0%    0.0%    0.0%    0.0%    0.0%    0.0%    0.0%    0.0%    0.0%    0.0%    0.0%    0.0%    0.0%    0.0%    0.0%    0.0%    0.0%    0.0%    0.0%    0.0%    0.0%    0.0%    0.0%    0.0%    0.0%    0.0%    0.0%    0.0%    0.0%    0.0%    0.0%    0.0%    0.0%    0.0%    0.0%    0.0%    0.0%    0.0%    0.0%    0.0%    0.0%    0.0%    0.0%    0.0%    0.0%    0.0%    0.0%    0.0%    0.0%    0.0%    0.0%    0.0%    0.0%    0.0%    0.0%    0.0%    0.0%    0.0%    0.0%    0.0%    0.0%    0.0%    0.0%    0.0%    0.0%    0.0%    0.0%    0.0%    0.0%    0.0%    0.0%    0.0%    0.0%    0.0%    0.0%    0.0%    0.0%    0.0%    0.0%    0.0%    0.0%    0.0%    0.0%    0.0%    0.0%    0.0%    0.0%    0.0%    0.0%    0.0%    0.0%    0.0%    0.0%    0.0%    0.0%    0.0%    0.0%    0.0%    0.0%    0.0%    0.0%    0.0%    0.0%    0.0%    0.0%    0.0%    0.0%    0.0%    0.0%    0.0%    0.0%    0.0%    0.0%    0.0%    0.0%    0.0%    0.0%    0.0%    0.0%    0.0%    0.0%    0.0%    0.0%    0.0%    0.0%    0.0%    0.0%    0.0%    0.0%    0.0%    0.0%    0.0%    0.0%    0.0%    0.0%    0.0%    0.0%    0.0%    0.0%    0.0%    0.0%    0.0%    0.0%    0.0%    0.0%    0.0%    0.0%    0.0%    0.0%    0.0%    0.0%    0.0%    0.0%    0.0%    0.0%    0.0%    0.0%    0.0%    0.0%    0.0%    0.0%    0.0%    0.0%    0.0%    0.0%    0.0%    0.0%    0.0%    0.0%    0.0%    0.0%    0.0%    0.0%    0.0%    0.0%    0.0%    0.0%    0.0%    0.0%    0.0%    0.0%    0.0%    0.0%    0.0%    0.0%    0.0%    0.0%    0.0%    0.0%    0.0%    0.0%    0.0%    0.0%    0.0%    0.0%    0.0%    0.0%    0.0%    0.0%    0.0%    0.0%    0.0%    0.0%    0.0%    0.0%    0.0%    0.0%    0.0%    0.0%    0.0%    0.0%    0.0%    0.0%    0.0%    0.0%    0.0%    0.0%    0.0%    0.0%    0.0%    0.0%    0.0%    0.0%    0.0%    0.0%    0.0%    0.0%    0.0%    0.0%    0.0%    0.0%    0.0%    0.0%    0.0%    0.0%    0.0%    0.0%    0.0%    0.0%    0.0%    0.0%    0.0%    0.0%    0.0%    0.0%    0.0%    0.0%    0.0%    0.0%    0.0%    0.0%    0.0%    0.0%    0.0%    0.0%    0.0%    0.0%    0.0%    0.0%    0.0%    0.0%    0.0%    0.0%    0.0%    0.0%    0.0%    0.0%    0.0%    0.0%    0.0%    0.0%    0.0%    0.0%    0.0%    0.0%    0.0%    0.0%    0.0%    0.0%    0.0%    0.0%    0.0%    0.0%    0.0%    0.0%        k__Bacteria;p__AD3; c__JG37-AG-4       8    0.0%    0.0%    0.0%    0.0%    0.0%    0.0%    0.0%    0.0%    0.0%    0.0%    0.0%    0.0%    0.0%    0.0%    0.0%    0.0%    0.0%    0.0%    0.0%    0.0%    0.0%    0.0%    0.0%    0.0%    0.0%    0.0%    0.0%    0.0%    0.0%    0.0%    0.0%    0.0%    0.0%    0.0%    0.0%    0.0%    0.0%    0.0%    0.0%    0.0%    0.0%    0.0%    0.0%    0.0%    0.0%    0.0%    0.0%    0.0%    0.0%    0.0%    0.0%    0.0%    0.0%    0.0%    0.0%    0.0%    0.0%    0.0%    0.0%    0.0%    0.0%    0.0%    0.0%    0.0%    0.0%    0.0%    0.0%    0.0%    0.0%    0.0%    0.0%    0.0%    0.0%    0.0%    0.0%    0.0%    0.0%    0.0%    0.0%    0.0%    0.0%    0.0%    0.0%    0.0%    0.0%    0.0%    0.0%    0.0%    0.0%    0.0%    0.0%    0.0%    0.0%    0.0%    0.0%    0.0%    0.0%    0.0%    0.0%    0.0%    0.0%    0.0%    0.0%    0.0%    0.0%    0.0%    0.0%    0.0%    0.0%    0.0%    0.0%    0.0%    0.0%    0.0%    0.0%    0.0%    0.0%    0.0%    0.0%    0.0%    0.0%    0.0%    0.0%    0.0%    0.0%    0.0%    0.0%    0.0%    0.0%    0.0%    0.0%    0.0%    0.0%    0.0%    0.0%    0.0%    0.0%    0.0%    0.0%    0.0%    0.0%    0.0%    0.0%    0.0%    0.0%    0.0%    0.0%    0.0%    0.0%    0.0%    0.0%    0.0%    0.0%    0.0%    0.0%    0.0%    0.0%    0.0%    0.0%    0.0%    0.0%    0.0%    0.0%    0.0%    0.0%    0.0%    0.0%    0.0%    0.0%    0.0%    0.0%    0.0%    0.0%    0.0%    0.0%    0.0%    0.0%    0.0%    0.0%    0.0%    0.0%    0.0%    0.0%    0.0%    0.0%    0.0%    0.0%    0.0%    0.0%    0.0%    0.0%    0.0%    0.0%    0.0%    0.0%    0.0%    0.0%    0.0%    0.0%    0.0%    0.0%    0.0%    0.0%    0.0%    0.0%    0.0%    0.0%    0.0%    0.0%    0.0%    0.0%    0.0%    0.0%    0.0%    0.0%    0.0%    0.0%    0.0%    0.0%    0.0%    0.0%    0.0%    0.0%    0.0%    0.0%    0.0%    0.0%    0.0%    0.0%    0.0%    0.0%    0.0%    0.0%    0.0%    0.0%    0.0%    0.0%    0.0%    0.0%    0.0%    0.0%    0.0%    0.0%    0.0%    0.0%    0.0%    0.0%    0.0%    0.0%    0.0%    0.0%    0.0%    0.0%    0.0%    0.0%    0.0%    0.0%    0.0%    0.0%    0.0%    0.0%    0.0%    0.0%    0.0%    0.0%    0.0%    0.0%    0.0%    0.0%    0.0%    0.0%    0.0%    0.0%    0.0%    0.0%    0.0%    0.0%    0.0%    0.0%    0.0%    0.0%    0.0%    0.0%    0.0%    0.0%    0.0%    0.0%    0.0%    0.0%    0.0%    0.0%    0.0%    0.0%    0.0%    0.0%    0.0%    0.0%    0.0%    0.0%    0.0%    0.0%    0.0%    0.0%    0.0%    0.0%    0.0%    0.0%    0.0%    0.0%    0.0%    0.0%    0.0%    0.0%    0.0%    0.0%    0.0%    0.0%    0.0%    0.0%    0.0%    0.0%    0.0%    0.0%    0.0%    0.0%    0.0%    0.0%    0.0%    0.0%    0.0%    0.0%    0.0%    0.0%    0.0%    0.0%    0.0%    0.0%    0.0%    0.0%    0.0%    0.0%    0.0%    0.0%    0.0%    0.0%    0.0%    0.0%    0.0%    0.0%    0.0%    0.0%    0.0%    0.0%    0.0%    0.0%    0.0%    0.0%    0.0%        k__Bacteria;p__Acidobacteria; c__     430    0.0%    0.0%    0.0%    0.0%    0.0%    0.0%    0.0%    0.0%    0.0%    0.0%    0.0%    0.0%    0.0%    0.0%    0.0%    0.0%    0.0%    0.0%    0.0%    0.0%    0.0%    0.0%    0.0%    0.0%    0.0%    0.0%    0.0%    0.0%    0.0%    0.0%    0.0%    0.0%    0.0%    0.0%    0.0%    0.0%    0.0%    0.0%    0.0%    0.0%    0.0%    0.0%    0.0%    0.0%    0.0%    0.0%    0.0%    0.0%    0.0%    0.0%    0.0%    0.0%    0.0%    0.0%    0.0%    0.0%    0.0%    0.0%    0.0%    0.0%    0.0%    0.0%    0.0%    0.0%    0.0%    0.0%    0.0%    0.0%    0.0%    0.0%    0.0%    0.0%    0.0%    0.0%    0.0%    0.0%    0.0%    0.0%    0.0%    0.0%    0.0%    0.0%    0.0%    0.0%    0.0%    0.0%    0.0%    0.0%    0.0%    0.0%    0.0%    0.0%    0.0%    0.0%    0.0%    0.0%    0.0%    0.0%    0.0%    0.0%    0.0%    0.0%    0.0%    0.0%    0.0%    0.0%    0.0%    0.0%    0.0%    0.0%    0.0%    0.0%    0.0%    0.0%    0.0%    0.0%    0.0%    0.0%    0.0%    0.0%    0.0%    0.0%    0.0%    0.0%    0.0%    0.0%    0.0%    0.0%    0.0%    0.0%    0.0%    0.0%    0.0%    0.0%    0.0%    0.0%    0.0%    0.0%    0.0%    0.0%    0.0%    0.0%    0.0%    0.0%    0.0%    0.0%    0.0%    0.0%    0.0%    0.0%    0.0%    0.0%    0.0%    0.0%    0.0%    0.0%    0.0%    0.0%    0.0%    0.0%    0.0%    0.0%    0.0%    0.0%    0.0%    0.0%    0.0%    0.0%    0.0%    0.0%    0.0%    0.0%    0.0%    0.0%    0.0%    0.0%    0.0%    0.0%    0.0%    0.0%    0.0%    0.0%    0.0%    0.0%    0.0%    0.0%    0.0%    0.0%    0.0%    0.0%    0.0%    0.0%    0.0%    0.0%    0.0%    0.0%    0.0%    0.0%    0.0%    0.0%    0.0%    0.0%    0.0%    0.0%    0.0%    0.0%    0.0%    0.0%    0.0%    0.0%    0.0%    0.0%    0.0%    0.0%    0.0%    0.0%    0.0%    0.0%    0.0%    0.0%    0.0%    0.0%    0.0%    0.0%    0.0%    0.0%    0.0%    0.0%    0.0%    0.0%    0.0%    0.0%    0.0%    0.0%    0.0%    0.0%    0.0%    0.0%    0.0%    0.0%    0.0%    0.0%    0.0%    0.0%    0.0%    0.0%    0.0%    0.0%    0.0%    0.0%    0.0%    0.0%    0.0%    0.0%    0.0%    0.0%    0.0%    0.0%    0.0%    0.0%    0.0%    0.0%    0.0%    0.0%    0.0%    0.0%    0.0%    0.0%    0.0%    0.0%    0.0%    0.0%    0.0%    0.0%    0.0%    0.0%    0.0%    0.0%    0.0%    0.0%    0.0%    0.0%    0.0%    0.0%    0.0%    0.0%    0.0%    0.0%    0.0%    0.0%    0.0%    0.0%    0.0%    0.0%    0.0%    0.0%    0.0%    0.0%    0.0%    0.0%    0.0%    0.0%    0.0%    0.0%    0.0%    0.0%    0.0%    0.0%    0.0%    0.0%    0.0%    0.0%    0.0%    0.0%    0.0%    0.0%    0.0%    0.0%    0.0%    0.0%    0.0%    0.0%    0.0%    0.0%    0.0%    0.0%    0.0%    0.0%    0.0%    0.0%    0.0%    0.0%    0.0%    0.0%    0.0%    0.0%    0.0%    0.0%    0.0%    0.0%    0.0%    0.0%    0.0%    0.0%    0.0%    0.0%    0.0%    0.0%    0.0%    0.0%    0.0%    0.0%    0.0%    0.0%    0.0%    0.0%    0.0%    0.0%        k__Bacteria;p__Acidobacteria; c__Acidobacteria (class)   17419    0.1%    0.0%    0.1%    0.0%    0.0%    0.0%    0.0%    0.0%    0.0%    0.0%    0.0%    0.0%    0.1%    0.0%    0.0%    0.0%    0.1%    0.1%    0.0%    0.1%    0.1%    0.1%    0.0%    0.0%    0.0%    0.0%    0.1%    0.1%    0.1%    0.0%    0.0%    0.1%    0.0%    0.0%    0.2%    0.0%    0.1%    0.0%    0.1%    0.2%    0.1%    0.2%    0.0%    0.0%    0.1%    0.0%    0.0%    0.0%    0.0%    0.0%    0.1%    0.1%    0.0%    0.0%    0.1%    0.1%    0.1%    0.0%    0.1%    0.3%    0.2%    0.1%    0.1%    0.4%    0.2%    0.2%    0.0%    0.4%    0.1%    0.2%    0.1%    0.0%    0.1%    0.1%    0.2%    0.0%    0.1%    0.1%    0.0%    0.1%    0.0%    0.0%    0.0%    0.1%    0.0%    0.0%    0.2%    0.0%    0.0%    0.2%    0.0%    0.1%    0.2%    0.0%    0.1%    0.1%    0.3%    0.0%    0.1%    0.2%    0.3%    0.1%    0.0%    0.1%    0.0%    0.0%    0.5%    0.0%    0.0%    0.2%    0.1%    0.3%    0.0%    0.0%    0.1%    0.0%    0.0%    0.1%    0.0%    0.7%    0.0%    0.1%    0.4%    0.1%    0.0%    0.3%    0.3%    0.0%    0.2%    0.7%    0.2%    0.1%    0.2%    0.1%    0.6%    0.1%    0.1%    0.1%    0.0%    0.1%    0.0%    0.1%    0.1%    0.1%    0.3%    0.1%    0.0%    0.0%    0.1%    0.0%    0.0%    0.1%    0.2%    0.1%    0.0%    0.0%    0.3%    0.0%    0.0%    0.3%    0.8%    0.2%    0.3%    0.1%    0.1%    0.1%    0.3%    0.1%    0.1%    0.1%    0.3%    0.2%    0.2%    0.1%    0.0%    0.0%    0.0%    0.0%    0.1%    0.0%    0.0%    0.0%    0.1%    0.1%    0.1%    0.0%    0.0%    0.0%    0.0%    0.1%    0.0%    0.0%    0.1%    0.1%    0.1%    0.1%    0.2%    0.0%    0.0%    0.0%    0.1%    0.0%    0.1%    0.3%    0.2%    0.1%    0.0%    0.2%    0.4%    0.2%    0.1%    0.0%    0.3%    0.1%    0.0%    0.2%    0.0%    0.0%    0.1%    0.0%    0.1%    0.2%    0.0%    0.2%    0.0%    0.1%    0.0%    0.0%    0.2%    0.1%    0.0%    0.0%    0.2%    0.0%    0.4%    0.0%    0.4%    0.1%    0.0%    0.1%    0.0%    0.3%    0.0%    0.0%    0.0%    0.0%    0.1%    0.1%    0.1%    0.0%    0.3%    0.2%    0.1%    0.0%    0.0%    0.0%    0.1%    0.0%    0.1%    0.1%    0.3%    0.2%    0.2%    0.1%    0.1%    0.0%    0.1%    0.2%    0.0%    0.1%    0.1%    0.2%    0.7%    0.1%    0.0%    0.1%    0.0%    0.1%    0.0%    0.0%    0.0%    0.0%    0.1%    0.0%    0.1%    0.0%    0.1%    1.1%    0.0%    0.0%    0.1%    0.0%    0.2%    0.1%    0.2%    0.0%    0.1%    0.1%    0.1%    0.2%    0.0%    0.1%    0.0%    0.3%    0.1%    0.0%    0.1%    0.1%    0.0%    0.0%    0.0%    0.0%    0.0%    0.0%    0.1%    0.1%    0.0%    0.0%    0.0%    0.0%    0.0%    0.0%    0.0%    0.1%    0.1%    0.1%    0.1%    0.0%    0.0%    0.1%    0.0%    0.0%    0.0%    0.2%    0.0%    0.0%    0.0%    0.1%    0.0%    0.0%    0.0%    0.0%    0.0%    0.1%    0.2%    0.0%    0.0%    0.1%    0.0%    0.0%    0.1%    0.1%    0.0%    0.2%    0.1%    0.0%    0.0%    0.0%        k__Bacteria;p__Acidobacteria; c__Acidobacteria-5     248    0.0%    0.0%    0.0%    0.0%    0.0%    0.0%    0.0%    0.0%    0.0%    0.0%    0.0%    0.0%    0.0%    0.0%    0.0%    0.0%    0.0%    0.0%    0.0%    0.0%    0.0%    0.0%    0.0%    0.0%    0.0%    0.0%    0.0%    0.0%    0.0%    0.0%    0.0%    0.0%    0.0%    0.0%    0.0%    0.0%    0.0%    0.0%    0.0%    0.0%    0.0%    0.0%    0.0%    0.0%    0.0%    0.0%    0.0%    0.0%    0.0%    0.0%    0.0%    0.0%    0.0%    0.0%    0.0%    0.0%    0.0%    0.0%    0.0%    0.0%    0.0%    0.0%    0.0%    0.0%    0.0%    0.0%    0.0%    0.0%    0.0%    0.0%    0.0%    0.0%    0.0%    0.0%    0.0%    0.0%    0.0%    0.0%    0.0%    0.0%    0.0%    0.0%    0.0%    0.0%    0.0%    0.0%    0.0%    0.0%    0.0%    0.0%    0.0%    0.0%    0.0%    0.0%    0.0%    0.0%    0.0%    0.0%    0.0%    0.0%    0.0%    0.0%    0.0%    0.0%    0.0%    0.0%    0.0%    0.0%    0.0%    0.0%    0.0%    0.0%    0.0%    0.0%    0.0%    0.0%    0.0%    0.0%    0.0%    0.0%    0.0%    0.0%    0.0%    0.0%    0.0%    0.0%    0.0%    0.0%    0.0%    0.0%    0.0%    0.0%    0.0%    0.0%    0.0%    0.0%    0.0%    0.0%    0.0%    0.0%    0.0%    0.0%    0.0%    0.0%    0.0%    0.0%    0.0%    0.0%    0.0%    0.0%    0.0%    0.0%    0.0%    0.0%    0.0%    0.0%    0.0%    0.0%    0.0%    0.0%    0.0%    0.0%    0.0%    0.0%    0.0%    0.0%    0.0%    0.0%    0.0%    0.0%    0.0%    0.0%    0.0%    0.0%    0.0%    0.0%    0.0%    0.0%    0.0%    0.0%    0.0%    0.0%    0.0%    0.0%    0.0%    0.0%    0.0%    0.0%    0.0%    0.0%    0.0%    0.0%    0.0%    0.0%    0.0%    0.0%    0.0%    0.0%    0.0%    0.0%    0.0%    0.0%    0.0%    0.0%    0.0%    0.0%    0.0%    0.0%    0.0%    0.0%    0.0%    0.0%    0.0%    0.0%    0.0%    0.0%    0.0%    0.0%    0.0%    0.0%    0.0%    0.0%    0.0%    0.0%    0.0%    0.0%    0.0%    0.0%    0.0%    0.0%    0.0%    0.0%    0.0%    0.0%    0.0%    0.0%    0.0%    0.0%    0.0%    0.0%    0.0%    0.0%    0.0%    0.0%    0.0%    0.0%    0.0%    0.0%    0.0%    0.0%    0.0%    0.0%    0.0%    0.0%    0.0%    0.0%    0.0%    0.0%    0.0%    0.0%    0.0%    0.0%    0.0%    0.0%    0.0%    0.0%    0.0%    0.0%    0.0%    0.0%    0.0%    0.0%    0.0%    0.0%    0.0%    0.0%    0.0%    0.0%    0.0%    0.0%    0.0%    0.0%    0.0%    0.0%    0.0%    0.0%    0.0%    0.0%    0.0%    0.0%    0.0%    0.0%    0.0%    0.0%    0.0%    0.0%    0.0%    0.0%    0.0%    0.0%    0.0%    0.0%    0.0%    0.0%    0.0%    0.0%    0.0%    0.0%    0.0%    0.0%    0.0%    0.0%    0.0%    0.0%    0.0%    0.0%    0.0%    0.0%    0.0%    0.0%    0.0%    0.0%    0.0%    0.0%    0.0%    0.0%    0.0%    0.0%    0.0%    0.0%    0.0%    0.0%    0.0%    0.0%    0.0%    0.0%    0.0%    0.0%    0.0%    0.0%    0.0%    0.0%    0.0%    0.0%    0.0%    0.0%    0.0%    0.0%    0.0%    0.0%    0.0%    0.0%    0.0%    0.0%    0.0%    0.0%    0.0%    0.0%        k__Bacteria;p__Acidobacteria; c__Chloracidobacteria    6682    0.0%    0.0%    0.0%    0.0%    0.0%    0.0%    0.0%    0.0%    0.0%    0.0%    0.0%    0.0%    0.0%    0.0%    0.0%    0.0%    0.0%    0.1%    0.0%    0.0%    0.0%    0.0%    0.0%    0.0%    0.0%    0.0%    0.0%    0.0%    0.0%    0.0%    0.0%    0.0%    0.0%    0.0%    0.0%    0.0%    0.1%    0.0%    0.0%    0.1%    0.0%    0.0%    0.0%    0.0%    0.0%    0.0%    0.0%    0.0%    0.0%    0.0%    0.0%    0.0%    0.1%    0.0%    0.0%    0.0%    0.0%    0.0%    0.0%    0.1%    0.0%    0.1%    0.0%    0.1%    0.3%    0.0%    0.0%    0.1%    0.0%    0.0%    0.0%    0.0%    0.0%    0.0%    0.1%    0.0%    0.0%    0.0%    0.0%    0.0%    0.0%    0.1%    0.0%    0.0%    0.0%    0.0%    0.1%    0.0%    0.0%    0.1%    0.0%    0.1%    0.0%    0.0%    0.0%    0.0%    0.1%    0.0%    0.1%    0.1%    0.1%    0.0%    0.0%    0.0%    0.0%    0.0%    0.1%    0.0%    0.0%    0.1%    0.1%    0.1%    0.0%    0.0%    0.1%    0.0%    0.0%    0.0%    0.0%    0.2%    0.0%    0.0%    0.1%    0.1%    0.0%    0.1%    0.1%    0.0%    0.1%    0.1%    0.1%    0.0%    0.1%    0.0%    0.2%    0.0%    0.0%    0.0%    0.0%    0.0%    0.0%    0.0%    0.0%    0.0%    0.1%    0.0%    0.0%    0.0%    0.0%    0.0%    0.0%    0.0%    0.1%    0.0%    0.0%    0.0%    0.0%    0.0%    0.0%    0.1%    0.1%    0.2%    0.1%    0.0%    0.0%    0.0%    0.0%    0.0%    0.0%    0.0%    0.1%    0.1%    0.1%    0.0%    0.0%    0.0%    0.0%    0.0%    0.0%    0.0%    0.0%    0.0%    0.0%    0.0%    0.0%    0.0%    0.0%    0.0%    0.0%    0.0%    0.0%    0.0%    0.0%    0.0%    0.0%    0.0%    0.1%    0.0%    0.0%    0.0%    0.0%    0.0%    0.0%    0.1%    0.1%    0.0%    0.0%    0.1%    0.2%    0.1%    0.1%    0.0%    0.3%    0.1%    0.0%    0.1%    0.0%    0.0%    0.2%    0.1%    0.0%    1.3%    0.0%    0.0%    0.0%    0.1%    0.0%    0.0%    0.1%    0.1%    0.0%    0.0%    0.3%    0.0%    0.1%    0.0%    0.1%    0.0%    0.0%    0.3%    0.1%    0.1%    0.0%    0.0%    0.0%    0.0%    0.0%    0.0%    0.0%    0.0%    0.1%    0.0%    0.0%    0.0%    0.0%    0.0%    0.0%    0.0%    0.0%    0.0%    0.1%    0.1%    0.1%    0.0%    0.0%    0.0%    0.1%    0.1%    0.0%    0.0%    0.0%    0.1%    0.1%    0.1%    0.0%    0.0%    0.0%    0.0%    0.0%    0.0%    0.0%    0.0%    0.0%    0.0%    0.0%    0.0%    0.0%    0.4%    0.0%    1.2%    0.0%    0.0%    0.1%    0.1%    0.1%    0.0%    0.0%    0.2%    0.0%    0.0%    0.0%    0.0%    0.0%    0.0%    0.0%    0.0%    0.0%    0.0%    0.1%    0.0%    0.0%    0.1%    0.0%    0.0%    0.0%    0.1%    0.0%    0.0%    0.0%    0.0%    0.0%    0.0%    0.0%    0.0%    0.0%    0.0%    0.0%    0.0%    0.0%    0.1%    0.0%    0.0%    0.0%    0.0%    0.0%    0.0%    0.0%    0.0%    0.0%    0.1%    0.0%    0.0%    0.0%    0.1%    0.0%    0.0%    0.0%    0.0%    0.0%    0.0%    0.0%    0.0%    0.0%    0.1%    0.0%    0.0%    0.0%    0.0%        k__Bacteria;p__Acidobacteria; c__Holophagae      19    0.0%    0.0%    0.0%    0.0%    0.0%    0.0%    0.0%    0.0%    0.0%    0.0%    0.0%    0.0%    0.0%    0.0%    0.0%    0.0%    0.0%    0.0%    0.0%    0.0%    0.0%    0.0%    0.0%    0.0%    0.0%    0.0%    0.0%    0.0%    0.0%    0.0%    0.0%    0.0%    0.0%    0.0%    0.0%    0.0%    0.0%    0.0%    0.0%    0.0%    0.0%    0.0%    0.0%    0.0%    0.0%    0.0%    0.0%    0.0%    0.0%    0.0%    0.0%    0.0%    0.0%    0.0%    0.0%    0.0%    0.0%    0.0%    0.0%    0.0%    0.0%    0.0%    0.0%    0.0%    0.0%    0.0%    0.0%    0.0%    0.0%    0.0%    0.0%    0.0%    0.0%    0.0%    0.0%    0.0%    0.0%    0.0%    0.0%    0.0%    0.0%    0.0%    0.0%    0.0%    0.0%    0.0%    0.0%    0.0%    0.0%    0.0%    0.0%    0.0%    0.0%    0.0%    0.0%    0.0%    0.0%    0.0%    0.0%    0.0%    0.0%    0.0%    0.0%    0.0%    0.0%    0.0%    0.0%    0.0%    0.0%    0.0%    0.0%    0.0%    0.0%    0.0%    0.0%    0.0%    0.0%    0.0%    0.0%    0.0%    0.0%    0.0%    0.0%    0.0%    0.0%    0.0%    0.0%    0.0%    0.0%    0.0%    0.0%    0.0%    0.0%    0.0%    0.0%    0.0%    0.0%    0.0%    0.0%    0.0%    0.0%    0.0%    0.0%    0.0%    0.0%    0.0%    0.0%    0.0%    0.0%    0.0%    0.0%    0.0%    0.0%    0.0%    0.0%    0.0%    0.0%    0.0%    0.0%    0.0%    0.0%    0.0%    0.0%    0.0%    0.0%    0.0%    0.0%    0.0%    0.0%    0.0%    0.0%    0.0%    0.0%    0.0%    0.0%    0.0%    0.0%    0.0%    0.0%    0.0%    0.0%    0.0%    0.0%    0.0%    0.0%    0.0%    0.0%    0.0%    0.0%    0.0%    0.0%    0.0%    0.0%    0.0%    0.0%    0.0%    0.0%    0.0%    0.0%    0.0%    0.0%    0.0%    0.0%    0.0%    0.0%    0.0%    0.0%    0.0%    0.0%    0.0%    0.0%    0.0%    0.0%    0.0%    0.0%    0.0%    0.0%    0.0%    0.0%    0.0%    0.0%    0.0%    0.0%    0.0%    0.0%    0.0%    0.0%    0.0%    0.0%    0.0%    0.0%    0.0%    0.0%    0.0%    0.0%    0.0%    0.0%    0.0%    0.0%    0.0%    0.0%    0.0%    0.0%    0.0%    0.0%    0.0%    0.0%    0.0%    0.0%    0.0%    0.0%    0.0%    0.0%    0.0%    0.0%    0.0%    0.0%    0.0%    0.0%    0.0%    0.0%    0.0%    0.0%    0.0%    0.0%    0.0%    0.0%    0.0%    0.0%    0.0%    0.0%    0.0%    0.0%    0.0%    0.0%    0.0%    0.0%    0.0%    0.0%    0.0%    0.0%    0.0%    0.0%    0.0%    0.0%    0.0%    0.0%    0.0%    0.0%    0.0%    0.0%    0.0%    0.0%    0.0%    0.0%    0.0%    0.0%    0.0%    0.0%    0.0%    0.0%    0.0%    0.0%    0.0%    0.0%    0.0%    0.0%    0.0%    0.0%    0.0%    0.0%    0.0%    0.0%    0.0%    0.0%    0.0%    0.0%    0.0%    0.0%    0.0%    0.0%    0.0%    0.0%    0.0%    0.0%    0.0%    0.0%    0.0%    0.0%    0.0%    0.0%    0.0%    0.0%    0.0%    0.0%    0.0%    0.0%    0.0%    0.0%    0.0%    0.0%    0.0%    0.0%    0.0%    0.0%    0.0%    0.0%    0.0%    0.0%    0.0%    0.0%    0.0%    0.0%    0.0%    0.0%    0.0%    0.0%    0.0%        k__Bacteria;p__Acidobacteria; c__MVS-40      16    0.0%    0.0%    0.0%    0.0%    0.0%    0.0%    0.0%    0.0%    0.0%    0.0%    0.0%    0.0%    0.0%    0.0%    0.0%    0.0%    0.0%    0.0%    0.0%    0.0%    0.0%    0.0%    0.0%    0.0%    0.0%    0.0%    0.0%    0.0%    0.0%    0.0%    0.0%    0.0%    0.0%    0.0%    0.0%    0.0%    0.0%    0.0%    0.0%    0.0%    0.0%    0.0%    0.0%    0.0%    0.0%    0.0%    0.0%    0.0%    0.0%    0.0%    0.0%    0.0%    0.0%    0.0%    0.0%    0.0%    0.0%    0.0%    0.0%    0.0%    0.0%    0.0%    0.0%    0.0%    0.0%    0.0%    0.0%    0.0%    0.0%    0.0%    0.0%    0.0%    0.0%    0.0%    0.0%    0.0%    0.0%    0.0%    0.0%    0.0%    0.0%    0.0%    0.0%    0.0%    0.0%    0.0%    0.0%    0.0%    0.0%    0.0%    0.0%    0.0%    0.0%    0.0%    0.0%    0.0%    0.0%    0.0%    0.0%    0.0%    0.0%    0.0%    0.0%    0.0%    0.0%    0.0%    0.0%    0.0%    0.0%    0.0%    0.0%    0.0%    0.0%    0.0%    0.0%    0.0%    0.0%    0.0%    0.0%    0.0%    0.0%    0.0%    0.0%    0.0%    0.0%    0.0%    0.0%    0.0%    0.0%    0.0%    0.0%    0.0%    0.0%    0.0%    0.0%    0.0%    0.0%    0.0%    0.0%    0.0%    0.0%    0.0%    0.0%    0.0%    0.0%    0.0%    0.0%    0.0%    0.0%    0.0%    0.0%    0.0%    0.0%    0.0%    0.0%    0.0%    0.0%    0.0%    0.0%    0.0%    0.0%    0.0%    0.0%    0.0%    0.0%    0.0%    0.0%    0.0%    0.0%    0.0%    0.0%    0.0%    0.0%    0.0%    0.0%    0.0%    0.0%    0.0%    0.0%    0.0%    0.0%    0.0%    0.0%    0.0%    0.0%    0.0%    0.0%    0.0%    0.0%    0.0%    0.0%    0.0%    0.0%    0.0%    0.0%    0.0%    0.0%    0.0%    0.0%    0.0%    0.0%    0.0%    0.0%    0.0%    0.0%    0.0%    0.0%    0.0%    0.0%    0.0%    0.0%    0.0%    0.0%    0.0%    0.0%    0.0%    0.0%    0.0%    0.0%    0.0%    0.0%    0.0%    0.0%    0.0%    0.0%    0.0%    0.0%    0.0%    0.0%    0.0%    0.0%    0.0%    0.0%    0.0%    0.0%    0.0%    0.0%    0.0%    0.0%    0.0%    0.0%    0.0%    0.0%    0.0%    0.0%    0.0%    0.0%    0.0%    0.0%    0.0%    0.0%    0.0%    0.0%    0.0%    0.0%    0.0%    0.0%    0.0%    0.0%    0.0%    0.0%    0.0%    0.0%    0.0%    0.0%    0.0%    0.0%    0.0%    0.0%    0.0%    0.0%    0.0%    0.0%    0.0%    0.0%    0.0%    0.0%    0.0%    0.0%    0.0%    0.0%    0.0%    0.0%    0.0%    0.0%    0.0%    0.0%    0.0%    0.0%    0.0%    0.0%    0.0%    0.0%    0.0%    0.0%    0.0%    0.0%    0.0%    0.0%    0.0%    0.0%    0.0%    0.0%    0.0%    0.0%    0.0%    0.0%    0.0%    0.0%    0.0%    0.0%    0.0%    0.0%    0.0%    0.0%    0.0%    0.0%    0.0%    0.0%    0.0%    0.0%    0.0%    0.0%    0.0%    0.0%    0.0%    0.0%    0.0%    0.0%    0.0%    0.0%    0.0%    0.0%    0.0%    0.0%    0.0%    0.0%    0.0%    0.0%    0.0%    0.0%    0.0%    0.0%    0.0%    0.0%    0.0%    0.0%    0.0%    0.0%    0.0%    0.0%    0.0%    0.0%    0.0%    0.0%    0.0%    0.0%    0.0%        k__Bacteria;p__Acidobacteria; c__OS-K       5    0.0%    0.0%    0.0%    0.0%    0.0%    0.0%    0.0%    0.0%    0.0%    0.0%    0.0%    0.0%    0.0%    0.0%    0.0%    0.0%    0.0%    0.0%    0.0%    0.0%    0.0%    0.0%    0.0%    0.0%    0.0%    0.0%    0.0%    0.0%    0.0%    0.0%    0.0%    0.0%    0.0%    0.0%    0.0%    0.0%    0.0%    0.0%    0.0%    0.0%    0.0%    0.0%    0.0%    0.0%    0.0%    0.0%    0.0%    0.0%    0.0%    0.0%    0.0%    0.0%    0.0%    0.0%    0.0%    0.0%    0.0%    0.0%    0.0%    0.0%    0.0%    0.0%    0.0%    0.0%    0.0%    0.0%    0.0%    0.0%    0.0%    0.0%    0.0%    0.0%    0.0%    0.0%    0.0%    0.0%    0.0%    0.0%    0.0%    0.0%    0.0%    0.0%    0.0%    0.0%    0.0%    0.0%    0.0%    0.0%    0.0%    0.0%    0.0%    0.0%    0.0%    0.0%    0.0%    0.0%    0.0%    0.0%    0.0%    0.0%    0.0%    0.0%    0.0%    0.0%    0.0%    0.0%    0.0%    0.0%    0.0%    0.0%    0.0%    0.0%    0.0%    0.0%    0.0%    0.0%    0.0%    0.0%    0.0%    0.0%    0.0%    0.0%    0.0%    0.0%    0.0%    0.0%    0.0%    0.0%    0.0%    0.0%    0.0%    0.0%    0.0%    0.0%    0.0%    0.0%    0.0%    0.0%    0.0%    0.0%    0.0%    0.0%    0.0%    0.0%    0.0%    0.0%    0.0%    0.0%    0.0%    0.0%    0.0%    0.0%    0.0%    0.0%    0.0%    0.0%    0.0%    0.0%    0.0%    0.0%    0.0%    0.0%    0.0%    0.0%    0.0%    0.0%    0.0%    0.0%    0.0%    0.0%    0.0%    0.0%    0.0%    0.0%    0.0%    0.0%    0.0%    0.0%    0.0%    0.0%    0.0%    0.0%    0.0%    0.0%    0.0%    0.0%    0.0%    0.0%    0.0%    0.0%    0.0%    0.0%    0.0%    0.0%    0.0%    0.0%    0.0%    0.0%    0.0%    0.0%    0.0%    0.0%    0.0%    0.0%    0.0%    0.0%    0.0%    0.0%    0.0%    0.0%    0.0%    0.0%    0.0%    0.0%    0.0%    0.0%    0.0%    0.0%    0.0%    0.0%    0.0%    0.0%    0.0%    0.0%    0.0%    0.0%    0.0%    0.0%    0.0%    0.0%    0.0%    0.0%    0.0%    0.0%    0.0%    0.0%    0.0%    0.0%    0.0%    0.0%    0.0%    0.0%    0.0%    0.0%    0.0%    0.0%    0.0%    0.0%    0.0%    0.0%    0.0%    0.0%    0.0%    0.0%    0.0%    0.0%    0.0%    0.0%    0.0%    0.0%    0.0%    0.0%    0.0%    0.0%    0.0%    0.0%    0.0%    0.0%    0.0%    0.0%    0.0%    0.0%    0.0%    0.0%    0.0%    0.0%    0.0%    0.0%    0.0%    0.0%    0.0%    0.0%    0.0%    0.0%    0.0%    0.0%    0.0%    0.0%    0.0%    0.0%    0.0%    0.0%    0.0%    0.0%    0.0%    0.0%    0.0%    0.0%    0.0%    0.0%    0.0%    0.0%    0.0%    0.0%    0.0%    0.0%    0.0%    0.0%    0.0%    0.0%    0.0%    0.0%    0.0%    0.0%    0.0%    0.0%    0.0%    0.0%    0.0%    0.0%    0.0%    0.0%    0.0%    0.0%    0.0%    0.0%    0.0%    0.0%    0.0%    0.0%    0.0%    0.0%    0.0%    0.0%    0.0%    0.0%    0.0%    0.0%    0.0%    0.0%    0.0%    0.0%    0.0%    0.0%    0.0%    0.0%    0.0%    0.0%    0.0%    0.0%    0.0%    0.0%    0.0%    0.0%    0.0%    0.0%    0.0%    0.0%        k__Bacteria;p__Acidobacteria; c__PAUC37f      31    0.0%    0.0%    0.0%    0.0%    0.0%    0.0%    0.0%    0.0%    0.0%    0.0%    0.0%    0.0%    0.0%    0.0%    0.0%    0.0%    0.0%    0.0%    0.0%    0.0%    0.0%    0.0%    0.0%    0.0%    0.0%    0.0%    0.0%    0.0%    0.0%    0.0%    0.0%    0.0%    0.0%    0.0%    0.0%    0.0%    0.0%    0.0%    0.0%    0.0%    0.0%    0.0%    0.0%    0.0%    0.0%    0.0%    0.0%    0.0%    0.0%    0.0%    0.0%    0.0%    0.0%    0.0%    0.0%    0.0%    0.0%    0.0%    0.0%    0.0%    0.0%    0.0%    0.0%    0.0%    0.0%    0.0%    0.0%    0.0%    0.0%    0.0%    0.0%    0.0%    0.0%    0.0%    0.0%    0.0%    0.0%    0.0%    0.0%    0.0%    0.0%    0.0%    0.0%    0.0%    0.0%    0.0%    0.0%    0.0%    0.0%    0.0%    0.0%    0.0%    0.0%    0.0%    0.0%    0.0%    0.0%    0.0%    0.0%    0.0%    0.0%    0.0%    0.0%    0.0%    0.0%    0.0%    0.0%    0.0%    0.0%    0.0%    0.0%    0.0%    0.0%    0.0%    0.0%    0.0%    0.0%    0.0%    0.0%    0.0%    0.0%    0.0%    0.0%    0.0%    0.0%    0.0%    0.0%    0.0%    0.0%    0.0%    0.0%    0.0%    0.0%    0.0%    0.0%    0.0%    0.0%    0.0%    0.0%    0.0%    0.0%    0.0%    0.0%    0.0%    0.0%    0.0%    0.0%    0.0%    0.0%    0.0%    0.0%    0.0%    0.0%    0.0%    0.0%    0.0%    0.0%    0.0%    0.0%    0.0%    0.0%    0.0%    0.0%    0.0%    0.0%    0.0%    0.0%    0.0%    0.0%    0.0%    0.0%    0.0%    0.0%    0.0%    0.0%    0.0%    0.0%    0.0%    0.0%    0.0%    0.0%    0.0%    0.0%    0.0%    0.0%    0.0%    0.0%    0.0%    0.0%    0.0%    0.0%    0.0%    0.0%    0.0%    0.0%    0.0%    0.0%    0.0%    0.0%    0.0%    0.0%    0.0%    0.0%    0.0%    0.0%    0.0%    0.0%    0.0%    0.0%    0.0%    0.0%    0.0%    0.0%    0.0%    0.0%    0.0%    0.0%    0.0%    0.0%    0.0%    0.0%    0.0%    0.0%    0.0%    0.0%    0.0%    0.0%    0.0%    0.0%    0.0%    0.0%    0.0%    0.0%    0.0%    0.0%    0.0%    0.0%    0.0%    0.0%    0.0%    0.0%    0.0%    0.0%    0.0%    0.0%    0.0%    0.0%    0.0%    0.0%    0.0%    0.0%    0.0%    0.0%    0.0%    0.0%    0.0%    0.0%    0.0%    0.0%    0.0%    0.0%    0.0%    0.0%    0.0%    0.0%    0.0%    0.0%    0.0%    0.0%    0.0%    0.0%    0.0%    0.0%    0.0%    0.0%    0.0%    0.0%    0.0%    0.0%    0.0%    0.0%    0.0%    0.0%    0.0%    0.0%    0.0%    0.0%    0.0%    0.0%    0.0%    0.0%    0.0%    0.0%    0.0%    0.0%    0.0%    0.0%    0.0%    0.0%    0.0%    0.0%    0.0%    0.0%    0.0%    0.0%    0.0%    0.0%    0.0%    0.0%    0.0%    0.0%    0.0%    0.0%    0.0%    0.0%    0.0%    0.0%    0.0%    0.0%    0.0%    0.0%    0.0%    0.0%    0.0%    0.0%    0.0%    0.0%    0.0%    0.0%    0.0%    0.0%    0.0%    0.0%    0.0%    0.0%    0.0%    0.0%    0.0%    0.0%    0.0%    0.0%    0.0%    0.0%    0.0%    0.0%    0.0%    0.0%    0.0%    0.0%    0.0%    0.0%    0.0%    0.0%    0.0%    0.0%    0.0%    0.0%    0.0%        k__Bacteria;p__Acidobacteria; c__RB25      17    0.0%    0.0%    0.0%    0.0%    0.0%    0.0%    0.0%    0.0%    0.0%    0.0%    0.0%    0.0%    0.0%    0.0%    0.0%    0.0%    0.0%    0.0%    0.0%    0.0%    0.0%    0.0%    0.0%    0.0%    0.0%    0.0%    0.0%    0.0%    0.0%    0.0%    0.0%    0.0%    0.0%    0.0%    0.0%    0.0%    0.0%    0.0%    0.0%    0.0%    0.0%    0.0%    0.0%    0.0%    0.0%    0.0%    0.0%    0.0%    0.0%    0.0%    0.0%    0.0%    0.0%    0.0%    0.0%    0.0%    0.0%    0.0%    0.0%    0.0%    0.0%    0.0%    0.0%    0.0%    0.0%    0.0%    0.0%    0.0%    0.0%    0.0%    0.0%    0.0%    0.0%    0.0%    0.0%    0.0%    0.0%    0.0%    0.0%    0.0%    0.0%    0.0%    0.0%    0.0%    0.0%    0.0%    0.0%    0.0%    0.0%    0.0%    0.0%    0.0%    0.0%    0.0%    0.0%    0.0%    0.0%    0.0%    0.0%    0.0%    0.0%    0.0%    0.0%    0.0%    0.0%    0.0%    0.0%    0.0%    0.0%    0.0%    0.0%    0.0%    0.0%    0.0%    0.0%    0.0%    0.0%    0.0%    0.0%    0.0%    0.0%    0.0%    0.0%    0.0%    0.0%    0.0%    0.0%    0.0%    0.0%    0.0%    0.0%    0.0%    0.0%    0.0%    0.0%    0.0%    0.0%    0.0%    0.0%    0.0%    0.0%    0.0%    0.0%    0.0%    0.0%    0.0%    0.0%    0.0%    0.0%    0.0%    0.0%    0.0%    0.0%    0.0%    0.0%    0.0%    0.0%    0.0%    0.0%    0.0%    0.0%    0.0%    0.0%    0.0%    0.0%    0.0%    0.0%    0.0%    0.0%    0.0%    0.0%    0.0%    0.0%    0.0%    0.0%    0.0%    0.0%    0.0%    0.0%    0.0%    0.0%    0.0%    0.0%    0.0%    0.0%    0.0%    0.0%    0.0%    0.0%    0.0%    0.0%    0.0%    0.0%    0.0%    0.0%    0.0%    0.0%    0.0%    0.0%    0.0%    0.0%    0.0%    0.0%    0.0%    0.0%    0.0%    0.0%    0.0%    0.0%    0.0%    0.0%    0.0%    0.0%    0.0%    0.0%    0.0%    0.0%    0.0%    0.0%    0.0%    0.0%    0.0%    0.0%    0.0%    0.0%    0.0%    0.0%    0.0%    0.0%    0.0%    0.0%    0.0%    0.0%    0.0%    0.0%    0.0%    0.0%    0.0%    0.0%    0.0%    0.0%    0.0%    0.0%    0.0%    0.0%    0.0%    0.0%    0.0%    0.0%    0.0%    0.0%    0.0%    0.0%    0.0%    0.0%    0.0%    0.0%    0.0%    0.0%    0.0%    0.0%    0.0%    0.0%    0.0%    0.0%    0.0%    0.0%    0.0%    0.0%    0.0%    0.0%    0.0%    0.0%    0.0%    0.0%    0.0%    0.0%    0.0%    0.0%    0.0%    0.0%    0.0%    0.0%    0.0%    0.0%    0.0%    0.0%    0.0%    0.0%    0.0%    0.0%    0.0%    0.0%    0.0%    0.0%    0.0%    0.0%    0.0%    0.0%    0.0%    0.0%    0.0%    0.0%    0.0%    0.0%    0.0%    0.0%    0.0%    0.0%    0.0%    0.0%    0.0%    0.0%    0.0%    0.0%    0.0%    0.0%    0.0%    0.0%    0.0%    0.0%    0.0%    0.0%    0.0%    0.0%    0.0%    0.0%    0.0%    0.0%    0.0%    0.0%    0.0%    0.0%    0.0%    0.0%    0.0%    0.0%    0.0%    0.0%    0.0%    0.0%    0.0%    0.0%    0.0%    0.0%    0.0%    0.0%    0.0%    0.0%    0.0%    0.0%    0.0%    0.0%    0.0%    0.0%    0.0%    0.0%    0.0%        k__Bacteria;p__Acidobacteria; c__Solibacteres    2539    0.0%    0.0%    0.0%    0.0%    0.0%    0.0%    0.0%    0.0%    0.0%    0.0%    0.0%    0.0%    0.0%    0.0%    0.0%    0.0%    0.0%    0.0%    0.0%    0.0%    0.0%    0.0%    0.0%    0.0%    0.0%    0.0%    0.0%    0.0%    0.0%    0.0%    0.0%    0.0%    0.1%    0.0%    0.0%    0.0%    0.0%    0.0%    0.0%    0.0%    0.0%    0.0%    0.0%    0.0%    0.0%    0.0%    0.0%    0.0%    0.0%    0.0%    0.0%    0.0%    0.0%    0.0%    0.1%    0.0%    0.0%    0.0%    0.0%    0.0%    0.0%    0.0%    0.0%    0.1%    0.0%    0.0%    0.0%    0.0%    0.0%    0.0%    0.0%    0.0%    0.0%    0.0%    0.0%    0.0%    0.0%    0.0%    0.0%    0.0%    0.0%    0.0%    0.0%    0.0%    0.0%    0.0%    0.0%    0.0%    0.0%    0.0%    0.0%    0.0%    0.0%    0.0%    0.0%    0.0%    0.0%    0.0%    0.0%    0.0%    0.0%    0.0%    0.0%    0.0%    0.0%    0.0%    0.1%    0.0%    0.0%    0.0%    0.0%    0.0%    0.0%    0.0%    0.0%    0.0%    0.0%    0.0%    0.0%    0.1%    0.0%    0.0%    0.0%    0.0%    0.0%    0.0%    0.2%    0.0%    0.0%    0.1%    0.1%    0.0%    0.1%    0.0%    0.1%    0.0%    0.0%    0.0%    0.0%    0.0%    0.0%    0.0%    0.0%    0.0%    0.0%    0.0%    0.0%    0.0%    0.0%    0.0%    0.0%    0.0%    0.0%    0.0%    0.0%    0.0%    0.0%    0.0%    0.0%    0.1%    0.1%    0.0%    0.0%    0.0%    0.0%    0.0%    0.0%    0.0%    0.0%    0.0%    0.0%    0.0%    0.0%    0.0%    0.0%    0.0%    0.0%    0.0%    0.0%    0.0%    0.0%    0.0%    0.0%    0.0%    0.0%    0.0%    0.0%    0.0%    0.0%    0.0%    0.0%    0.0%    0.0%    0.0%    0.0%    0.0%    0.0%    0.0%    0.0%    0.0%    0.0%    0.0%    0.0%    0.1%    0.0%    0.0%    0.0%    0.0%    0.1%    0.0%    0.0%    0.0%    0.1%    0.0%    0.0%    0.1%    0.0%    0.0%    0.0%    0.0%    0.0%    0.0%    0.0%    0.0%    0.0%    0.0%    0.0%    0.0%    0.0%    0.0%    0.0%    0.0%    0.0%    0.0%    0.0%    0.0%    0.1%    0.0%    0.0%    0.1%    0.0%    0.0%    0.0%    0.0%    0.0%    0.0%    0.0%    0.0%    0.0%    0.0%    0.0%    0.0%    0.0%    0.0%    0.0%    0.0%    0.0%    0.0%    0.0%    0.0%    0.0%    0.0%    0.0%    0.0%    0.0%    0.0%    0.0%    0.0%    0.0%    0.0%    0.0%    0.0%    0.0%    0.0%    0.0%    0.0%    0.0%    0.0%    0.0%    0.0%    0.0%    0.0%    0.0%    0.0%    0.0%    0.0%    0.0%    0.0%    0.0%    0.0%    0.0%    0.0%    0.0%    0.0%    0.0%    0.0%    0.0%    0.0%    0.0%    0.0%    0.0%    0.0%    0.0%    0.0%    0.0%    0.0%    0.0%    0.0%    0.0%    0.0%    0.0%    0.0%    0.0%    0.0%    0.0%    0.0%    0.0%    0.0%    0.0%    0.0%    0.0%    0.0%    0.0%    0.0%    0.0%    0.0%    0.0%    0.0%    0.0%    0.0%    0.0%    0.0%    0.0%    0.0%    0.0%    0.0%    0.0%    0.0%    0.0%    0.0%    0.0%    0.0%    0.0%    0.0%    0.0%    0.0%    0.0%    0.0%    0.0%    0.0%    0.0%    0.0%    0.0%    0.1%    0.0%    0.0%    0.0%    0.0%        k__Bacteria;p__Acidobacteria; c__Sva0725     563    0.0%    0.0%    0.0%    0.0%    0.0%    0.0%    0.0%    0.0%    0.0%    0.0%    0.0%    0.0%    0.0%    0.0%    0.0%    0.0%    0.0%    0.0%    0.0%    0.0%    0.0%    0.0%    0.0%    0.0%    0.0%    0.0%    0.0%    0.0%    0.0%    0.0%    0.0%    0.0%    0.0%    0.0%    0.0%    0.0%    0.0%    0.0%    0.0%    0.0%    0.0%    0.0%    0.0%    0.0%    0.0%    0.0%    0.0%    0.0%    0.0%    0.0%    0.0%    0.0%    0.0%    0.0%    0.0%    0.0%    0.0%    0.0%    0.0%    0.0%    0.0%    0.0%    0.0%    0.0%    0.0%    0.0%    0.0%    0.0%    0.0%    0.1%    0.0%    0.0%    0.0%    0.0%    0.0%    0.0%    0.0%    0.0%    0.0%    0.0%    0.0%    0.0%    0.0%    0.0%    0.0%    0.0%    0.0%    0.0%    0.0%    0.0%    0.0%    0.0%    0.0%    0.0%    0.0%    0.0%    0.0%    0.0%    0.0%    0.0%    0.0%    0.0%    0.0%    0.0%    0.0%    0.0%    0.0%    0.0%    0.0%    0.0%    0.0%    0.0%    0.0%    0.0%    0.0%    0.0%    0.0%    0.0%    0.0%    0.0%    0.0%    0.0%    0.0%    0.0%    0.0%    0.0%    0.0%    0.0%    0.0%    0.0%    0.0%    0.0%    0.0%    0.0%    0.0%    0.0%    0.0%    0.0%    0.0%    0.0%    0.0%    0.0%    0.0%    0.0%    0.0%    0.0%    0.0%    0.0%    0.0%    0.0%    0.0%    0.0%    0.0%    0.0%    0.0%    0.0%    0.0%    0.0%    0.0%    0.0%    0.0%    0.0%    0.0%    0.0%    0.0%    0.0%    0.0%    0.0%    0.0%    0.0%    0.0%    0.0%    0.0%    0.0%    0.0%    0.0%    0.0%    0.0%    0.0%    0.0%    0.0%    0.0%    0.0%    0.0%    0.0%    0.0%    0.0%    0.0%    0.0%    0.0%    0.0%    0.0%    0.0%    0.0%    0.0%    0.0%    0.0%    0.0%    0.0%    0.0%    0.0%    0.0%    0.0%    0.0%    0.0%    0.0%    0.0%    0.0%    0.0%    0.0%    0.0%    0.0%    0.0%    0.0%    0.0%    0.0%    0.0%    0.0%    0.0%    0.0%    0.0%    0.0%    0.0%    0.0%    0.0%    0.0%    0.0%    0.0%    0.0%    0.0%    0.0%    0.0%    0.0%    0.0%    0.0%    0.0%    0.0%    0.0%    0.0%    0.0%    0.0%    0.0%    0.0%    0.0%    0.0%    0.0%    0.0%    0.0%    0.0%    0.0%    0.0%    0.0%    0.0%    0.0%    0.0%    0.0%    0.0%    0.0%    0.0%    0.0%    0.0%    0.0%    0.0%    0.0%    0.0%    0.0%    0.0%    0.0%    0.0%    0.0%    0.0%    0.0%    0.0%    0.0%    0.0%    0.0%    0.0%    0.0%    0.0%    0.0%    0.0%    0.0%    0.0%    0.0%    0.0%    0.0%    0.0%    0.0%    0.0%    0.0%    0.0%    0.0%    0.0%    0.0%    0.0%    0.0%    0.0%    0.0%    0.0%    0.0%    0.0%    0.0%    0.0%    0.0%    0.0%    0.0%    0.0%    0.0%    0.0%    0.0%    0.0%    0.0%    0.0%    0.0%    0.1%    0.0%    0.0%    0.0%    0.0%    0.0%    0.0%    0.0%    0.0%    0.0%    0.0%    0.0%    0.0%    0.0%    0.0%    0.0%    0.0%    0.0%    0.0%    0.0%    0.0%    0.0%    0.0%    0.0%    0.0%    0.0%    0.0%    0.0%    0.0%    0.0%    0.0%    0.0%    0.0%    0.0%    0.0%    0.0%    0.0%    0.0%    0.0%    0.0%    0.0%    0.0%    0.0%    0.0%        k__Bacteria;p__Acidobacteria; c__iii1-8     514    0.0%    0.0%    0.0%    0.0%    0.0%    0.0%    0.0%    0.0%    0.0%    0.0%    0.0%    0.0%    0.0%    0.0%    0.0%    0.0%    0.0%    0.0%    0.0%    0.0%    0.0%    0.0%    0.0%    0.0%    0.0%    0.0%    0.0%    0.0%    0.0%    0.0%    0.0%    0.0%    0.0%    0.0%    0.0%    0.0%    0.0%    0.0%    0.0%    0.0%    0.0%    0.0%    0.0%    0.0%    0.0%    0.0%    0.0%    0.0%    0.0%    0.0%    0.0%    0.0%    0.0%    0.0%    0.0%    0.0%    0.0%    0.0%    0.0%    0.0%    0.0%    0.0%    0.0%    0.0%    0.0%    0.0%    0.0%    0.0%    0.0%    0.0%    0.0%    0.0%    0.0%    0.0%    0.0%    0.0%    0.0%    0.0%    0.0%    0.0%    0.0%    0.0%    0.0%    0.0%    0.0%    0.0%    0.0%    0.0%    0.0%    0.0%    0.0%    0.0%    0.0%    0.0%    0.0%    0.0%    0.0%    0.0%    0.0%    0.0%    0.0%    0.0%    0.0%    0.0%    0.0%    0.0%    0.0%    0.0%    0.0%    0.0%    0.0%    0.0%    0.0%    0.0%    0.0%    0.0%    0.0%    0.0%    0.0%    0.0%    0.0%    0.0%    0.0%    0.0%    0.0%    0.0%    0.0%    0.0%    0.0%    0.0%    0.0%    0.0%    0.0%    0.0%    0.0%    0.0%    0.0%    0.0%    0.0%    0.0%    0.0%    0.0%    0.0%    0.0%    0.0%    0.0%    0.0%    0.0%    0.0%    0.0%    0.0%    0.0%    0.0%    0.0%    0.0%    0.0%    0.0%    0.0%    0.0%    0.0%    0.0%    0.0%    0.0%    0.0%    0.0%    0.0%    0.0%    0.0%    0.0%    0.0%    0.0%    0.0%    0.0%    0.0%    0.0%    0.0%    0.0%    0.0%    0.0%    0.0%    0.0%    0.0%    0.0%    0.0%    0.0%    0.0%    0.0%    0.0%    0.0%    0.0%    0.0%    0.0%    0.0%    0.0%    0.0%    0.0%    0.0%    0.0%    0.0%    0.0%    0.0%    0.0%    0.0%    0.0%    0.0%    0.0%    0.0%    0.0%    0.0%    0.0%    0.0%    0.0%    0.0%    0.0%    0.0%    0.0%    0.0%    0.0%    0.0%    0.0%    0.0%    0.0%    0.0%    0.0%    0.0%    0.0%    0.0%    0.0%    0.0%    0.0%    0.0%    0.0%    0.0%    0.0%    0.0%    0.0%    0.0%    0.0%    0.0%    0.0%    0.0%    0.0%    0.0%    0.0%    0.0%    0.0%    0.0%    0.0%    0.0%    0.0%    0.0%    0.0%    0.0%    0.0%    0.0%    0.0%    0.0%    0.0%    0.0%    0.0%    0.0%    0.0%    0.0%    0.0%    0.0%    0.0%    0.0%    0.0%    0.0%    0.0%    0.0%    0.0%    0.0%    0.0%    0.0%    0.0%    0.0%    0.0%    0.0%    0.0%    0.0%    0.0%    0.0%    0.0%    0.0%    0.0%    0.0%    0.0%    0.0%    0.0%    0.0%    0.0%    0.0%    0.0%    0.0%    0.0%    0.0%    0.0%    0.0%    0.0%    0.0%    0.0%    0.0%    0.0%    0.0%    0.0%    0.0%    0.0%    0.0%    0.0%    0.0%    0.0%    0.0%    0.0%    0.0%    0.0%    0.0%    0.0%    0.0%    0.0%    0.0%    0.0%    0.0%    0.0%    0.0%    0.0%    0.0%    0.0%    0.0%    0.0%    0.0%    0.0%    0.0%    0.0%    0.0%    0.0%    0.0%    0.0%    0.0%    0.0%    0.0%    0.0%    0.0%    0.0%    0.0%    0.0%    0.0%    0.0%    0.0%    0.0%    0.0%    0.0%    0.0%    0.0%    0.0%    0.0%    0.0%    0.0%        k__Bacteria;p__Actinobacteria; c__    1871    0.0%    0.0%    0.0%    0.0%    0.0%    0.0%    0.0%    0.0%    0.0%    0.0%    0.0%    0.0%    0.0%    0.0%    0.0%    0.0%    0.0%    0.0%    0.0%    0.0%    0.0%    0.0%    0.0%    0.0%    0.0%    0.0%    0.0%    0.0%    0.0%    0.0%    0.0%    0.0%    0.0%    0.0%    0.0%    0.0%    0.0%    0.0%    0.0%    0.0%    0.0%    0.0%    0.0%    0.0%    0.0%    0.0%    0.0%    0.0%    0.0%    0.0%    0.0%    0.0%    0.0%    0.0%    0.0%    0.0%    0.0%    0.0%    0.0%    0.0%    0.0%    0.0%    0.0%    0.0%    0.0%    0.0%    0.0%    0.0%    0.0%    0.0%    0.0%    0.0%    0.0%    0.0%    0.0%    0.0%    0.0%    0.0%    0.0%    0.0%    0.0%    0.0%    0.0%    0.0%    0.0%    0.0%    0.0%    0.0%    0.0%    0.0%    0.0%    0.0%    0.0%    0.0%    0.0%    0.0%    0.0%    0.0%    0.1%    0.0%    0.0%    0.0%    0.0%    0.0%    0.0%    0.0%    0.0%    0.0%    0.0%    0.0%    0.0%    0.0%    0.0%    0.0%    0.0%    0.0%    0.0%    0.0%    0.0%    0.0%    0.0%    0.0%    0.0%    0.0%    0.0%    0.0%    0.0%    0.0%    0.0%    0.0%    0.0%    0.0%    0.0%    0.0%    0.0%    0.0%    0.0%    0.0%    0.0%    0.0%    0.0%    0.0%    0.0%    0.0%    0.0%    0.0%    0.0%    0.0%    0.1%    0.0%    0.0%    0.0%    0.0%    0.0%    0.0%    0.6%    0.1%    0.0%    0.0%    0.0%    0.0%    0.1%    0.0%    0.0%    0.0%    0.0%    0.0%    0.0%    0.0%    0.0%    0.0%    0.0%    0.0%    0.0%    0.0%    0.0%    0.0%    0.0%    0.0%    0.0%    0.0%    0.0%    0.0%    0.0%    0.0%    0.0%    0.0%    0.0%    0.1%    0.0%    0.0%    0.0%    0.0%    0.0%    0.0%    0.0%    0.0%    0.0%    0.0%    0.0%    0.0%    0.0%    0.0%    0.0%    0.1%    0.0%    0.0%    0.0%    0.0%    0.0%    0.0%    0.0%    0.0%    0.0%    0.0%    0.0%    0.1%    0.0%    0.1%    0.0%    0.0%    0.0%    0.0%    0.0%    0.0%    0.0%    0.0%    0.0%    0.0%    0.0%    0.0%    0.0%    0.1%    0.0%    0.0%    0.0%    0.0%    0.0%    0.0%    0.0%    0.0%    0.0%    0.0%    0.0%    0.0%    0.0%    0.0%    0.0%    0.0%    0.0%    0.0%    0.0%    0.0%    0.0%    0.0%    0.0%    0.0%    0.0%    0.0%    0.0%    0.0%    0.0%    0.0%    0.0%    0.0%    0.0%    0.0%    0.0%    0.0%    0.0%    0.0%    0.0%    0.0%    0.0%    0.0%    0.0%    0.0%    0.0%    0.0%    0.0%    0.0%    0.0%    0.0%    0.0%    0.0%    0.0%    0.0%    0.0%    0.0%    0.0%    0.0%    0.0%    0.0%    0.0%    0.0%    0.0%    0.0%    0.0%    0.0%    0.0%    0.0%    0.0%    0.0%    0.0%    0.0%    0.0%    0.0%    0.0%    0.0%    0.0%    0.0%    0.1%    0.0%    0.0%    0.0%    0.0%    0.0%    0.0%    0.0%    0.0%    0.0%    0.0%    0.0%    0.0%    0.0%    0.0%    0.0%    0.0%    0.0%    0.0%    0.0%    0.0%    0.0%    0.0%    0.0%    0.0%    0.0%    0.0%    0.0%    0.0%    0.0%    0.0%    0.0%    0.0%    0.0%    0.0%    0.0%    0.0%    0.0%    0.0%    0.0%    0.0%    0.2%    0.0%    0.0%    0.0%    0.0%    0.0%        k__Bacteria;p__Actinobacteria; c__Actinobacteria (class)   3077116   18.1%   16.6%   21.6%   25.0%   28.3%    4.5%   28.4%   12.6%   21.3%   18.0%    9.7%    9.9%   16.4%   19.0%   18.7%   16.3%   25.7%   11.9%   12.8%   25.4%   13.8%   19.7%   15.1%   47.5%   40.4%   20.9%   11.7%   17.8%   20.5%   55.9%   44.1%   17.5%    7.7%   17.3%   16.2%   23.7%   27.9%   20.2%   10.9%   11.1%   12.9%   15.5%   19.3%   23.4%   28.1%   17.4%   15.1%   48.0%   19.8%   45.6%   14.5%    8.4%   11.8%   25.0%   11.2%   51.2%   21.2%    4.5%   12.8%   16.7%   12.3%   12.6%   48.1%   13.2%    8.5%   21.9%    2.9%   20.0%    6.6%   16.7%    7.5%   48.5%    4.2%   20.9%   21.3%    2.2%    7.3%   15.0%   25.9%   23.7%   24.2%    9.3%    9.8%   12.8%   47.0%   35.5%   21.5%    4.2%   12.4%   24.0%    1.6%   11.8%   16.2%    5.6%   64.9%   27.8%   10.0%    1.4%   18.9%   22.9%   27.7%   11.5%   15.3%   33.5%   40.0%   19.2%   16.0%    4.7%    1.4%   28.7%   22.1%   16.0%   22.0%    3.1%   14.9%   19.6%   15.9%   24.6%    9.4%   25.4%    0.6%   16.6%   21.2%   11.4%    2.6%   15.5%   22.7%    4.2%   12.7%   19.2%   19.2%    7.4%    7.9%   14.7%   19.5%    4.9%    9.2%   23.2%    1.6%    6.7%    0.6%    9.1%    8.2%    3.9%   10.1%   31.0%   16.8%   13.2%   13.7%    2.1%    5.3%    2.8%   11.7%   10.2%    6.8%    2.4%   15.1%    6.5%    3.2%   22.5%   16.4%   28.4%   13.9%    3.8%   17.7%   10.1%   27.3%   27.0%   23.9%   21.4%   17.0%   22.2%    7.9%   12.7%   12.6%   25.7%   20.1%    3.4%    4.8%   11.0%   27.3%   23.4%    4.9%    6.9%   50.7%   26.0%   32.9%   21.2%   29.5%   26.6%   37.5%   33.4%   35.2%   23.3%   11.7%   12.5%   20.4%   23.5%   23.7%   25.9%   19.1%   10.5%   14.2%   14.8%   26.0%    8.1%    5.7%   10.3%   18.5%   26.7%   17.3%   13.4%    5.6%   17.3%    5.5%   23.0%   10.1%   10.9%   31.0%   36.4%   22.8%   10.9%    0.7%    7.4%   12.2%   19.4%   19.9%   14.6%   15.1%   27.2%   10.3%   14.5%   11.5%   19.0%   15.3%    9.4%   16.8%   14.3%    4.3%   28.2%   18.3%   17.9%   25.8%   37.7%   11.7%   14.5%   15.4%   25.3%   23.4%   33.6%   21.6%   14.9%    8.0%   11.4%    5.0%   14.3%   13.9%    8.2%   15.8%   12.1%   20.4%   12.4%   17.0%   10.9%   21.1%    4.3%   22.4%   16.5%    7.7%   16.4%   36.9%   29.7%   14.5%   24.1%   13.2%   25.1%   19.6%   23.9%   23.7%   18.5%    9.0%    2.1%   12.5%   30.8%   19.2%   16.4%   25.1%   26.0%   25.0%   13.7%   29.1%   51.9%   35.7%   23.6%   15.7%   12.8%   23.6%   33.7%   28.9%   21.4%   39.6%   16.5%    9.1%   28.6%   24.6%   22.5%   23.7%   33.6%   14.0%   13.7%   40.5%   27.6%   40.0%   15.6%   21.8%   25.7%    7.5%   31.1%   24.3%   59.0%   28.3%   21.7%   20.0%   10.8%   22.2%   35.3%   32.8%   23.4%   20.9%   22.0%   15.6%   25.6%   17.8%    8.2%   18.1%   33.7%    9.4%   20.3%   17.2%   13.3%   26.5%   12.9%   25.9%   32.4%   20.8%   24.4%   34.9%   25.4%   24.9%   51.9%   17.8%   19.8%   21.6%   24.6%   26.7%    0.3%    8.6%    9.4%        k__Bacteria;p__Aquificae; c__Aquificae (class)      31    0.0%    0.0%    0.0%    0.0%    0.0%    0.0%    0.0%    0.0%    0.0%    0.0%    0.0%    0.0%    0.0%    0.0%    0.0%    0.0%    0.0%    0.0%    0.0%    0.0%    0.0%    0.0%    0.0%    0.0%    0.0%    0.0%    0.0%    0.0%    0.0%    0.0%    0.0%    0.0%    0.0%    0.0%    0.0%    0.0%    0.0%    0.0%    0.0%    0.0%    0.0%    0.0%    0.0%    0.0%    0.0%    0.0%    0.0%    0.0%    0.0%    0.0%    0.0%    0.0%    0.0%    0.0%    0.0%    0.0%    0.0%    0.0%    0.0%    0.0%    0.0%    0.0%    0.0%    0.0%    0.0%    0.0%    0.0%    0.0%    0.0%    0.0%    0.0%    0.0%    0.0%    0.0%    0.0%    0.0%    0.0%    0.0%    0.0%    0.0%    0.0%    0.0%    0.0%    0.0%    0.0%    0.0%    0.0%    0.0%    0.0%    0.0%    0.0%    0.0%    0.0%    0.0%    0.0%    0.0%    0.0%    0.0%    0.0%    0.0%    0.0%    0.0%    0.0%    0.0%    0.0%    0.0%    0.0%    0.0%    0.0%    0.0%    0.0%    0.0%    0.0%    0.0%    0.0%    0.0%    0.0%    0.0%    0.0%    0.0%    0.0%    0.0%    0.0%    0.0%    0.0%    0.0%    0.0%    0.0%    0.0%    0.0%    0.0%    0.0%    0.0%    0.0%    0.0%    0.0%    0.0%    0.0%    0.0%    0.0%    0.0%    0.0%    0.0%    0.0%    0.0%    0.0%    0.0%    0.0%    0.0%    0.0%    0.0%    0.0%    0.0%    0.0%    0.0%    0.0%    0.0%    0.0%    0.0%    0.0%    0.0%    0.0%    0.0%    0.0%    0.0%    0.0%    0.0%    0.0%    0.0%    0.0%    0.0%    0.0%    0.0%    0.0%    0.0%    0.0%    0.0%    0.0%    0.0%    0.0%    0.0%    0.0%    0.0%    0.0%    0.0%    0.0%    0.0%    0.0%    0.0%    0.0%    0.0%    0.0%    0.0%    0.0%    0.0%    0.0%    0.0%    0.0%    0.0%    0.0%    0.0%    0.0%    0.0%    0.0%    0.0%    0.0%    0.0%    0.0%    0.0%    0.0%    0.0%    0.0%    0.0%    0.0%    0.0%    0.0%    0.0%    0.0%    0.0%    0.0%    0.0%    0.0%    0.0%    0.0%    0.0%    0.0%    0.0%    0.0%    0.0%    0.0%    0.0%    0.0%    0.0%    0.0%    0.0%    0.0%    0.0%    0.0%    0.0%    0.0%    0.0%    0.0%    0.0%    0.0%    0.0%    0.0%    0.0%    0.0%    0.0%    0.0%    0.0%    0.0%    0.0%    0.0%    0.0%    0.0%    0.0%    0.0%    0.0%    0.0%    0.0%    0.0%    0.0%    0.0%    0.0%    0.0%    0.0%    0.0%    0.0%    0.0%    0.0%    0.0%    0.0%    0.0%    0.0%    0.0%    0.0%    0.0%    0.0%    0.0%    0.0%    0.0%    0.0%    0.0%    0.0%    0.0%    0.0%    0.0%    0.0%    0.0%    0.0%    0.0%    0.0%    0.0%    0.0%    0.0%    0.0%    0.0%    0.0%    0.0%    0.0%    0.0%    0.0%    0.0%    0.0%    0.0%    0.0%    0.0%    0.0%    0.0%    0.0%    0.0%    0.0%    0.0%    0.0%    0.0%    0.0%    0.0%    0.0%    0.0%    0.0%    0.0%    0.0%    0.0%    0.0%    0.0%    0.0%    0.0%    0.0%    0.0%    0.0%    0.0%    0.0%    0.0%    0.0%    0.0%    0.0%    0.0%    0.0%    0.0%    0.0%    0.0%    0.0%    0.0%    0.0%    0.0%    0.0%    0.0%    0.0%    0.0%    0.0%    0.0%    0.0%    0.0%    0.0%    0.0%    0.0%    0.0%        k__Bacteria;p__BRC1; c__       0    0.0%    0.0%    0.0%    0.0%    0.0%    0.0%    0.0%    0.0%    0.0%    0.0%    0.0%    0.0%    0.0%    0.0%    0.0%    0.0%    0.0%    0.0%    0.0%    0.0%    0.0%    0.0%    0.0%    0.0%    0.0%    0.0%    0.0%    0.0%    0.0%    0.0%    0.0%    0.0%    0.0%    0.0%    0.0%    0.0%    0.0%    0.0%    0.0%    0.0%    0.0%    0.0%    0.0%    0.0%    0.0%    0.0%    0.0%    0.0%    0.0%    0.0%    0.0%    0.0%    0.0%    0.0%    0.0%    0.0%    0.0%    0.0%    0.0%    0.0%    0.0%    0.0%    0.0%    0.0%    0.0%    0.0%    0.0%    0.0%    0.0%    0.0%    0.0%    0.0%    0.0%    0.0%    0.0%    0.0%    0.0%    0.0%    0.0%    0.0%    0.0%    0.0%    0.0%    0.0%    0.0%    0.0%    0.0%    0.0%    0.0%    0.0%    0.0%    0.0%    0.0%    0.0%    0.0%    0.0%    0.0%    0.0%    0.0%    0.0%    0.0%    0.0%    0.0%    0.0%    0.0%    0.0%    0.0%    0.0%    0.0%    0.0%    0.0%    0.0%    0.0%    0.0%    0.0%    0.0%    0.0%    0.0%    0.0%    0.0%    0.0%    0.0%    0.0%    0.0%    0.0%    0.0%    0.0%    0.0%    0.0%    0.0%    0.0%    0.0%    0.0%    0.0%    0.0%    0.0%    0.0%    0.0%    0.0%    0.0%    0.0%    0.0%    0.0%    0.0%    0.0%    0.0%    0.0%    0.0%    0.0%    0.0%    0.0%    0.0%    0.0%    0.0%    0.0%    0.0%    0.0%    0.0%    0.0%    0.0%    0.0%    0.0%    0.0%    0.0%    0.0%    0.0%    0.0%    0.0%    0.0%    0.0%    0.0%    0.0%    0.0%    0.0%    0.0%    0.0%    0.0%    0.0%    0.0%    0.0%    0.0%    0.0%    0.0%    0.0%    0.0%    0.0%    0.0%    0.0%    0.0%    0.0%    0.0%    0.0%    0.0%    0.0%    0.0%    0.0%    0.0%    0.0%    0.0%    0.0%    0.0%    0.0%    0.0%    0.0%    0.0%    0.0%    0.0%    0.0%    0.0%    0.0%    0.0%    0.0%    0.0%    0.0%    0.0%    0.0%    0.0%    0.0%    0.0%    0.0%    0.0%    0.0%    0.0%    0.0%    0.0%    0.0%    0.0%    0.0%    0.0%    0.0%    0.0%    0.0%    0.0%    0.0%    0.0%    0.0%    0.0%    0.0%    0.0%    0.0%    0.0%    0.0%    0.0%    0.0%    0.0%    0.0%    0.0%    0.0%    0.0%    0.0%    0.0%    0.0%    0.0%    0.0%    0.0%    0.0%    0.0%    0.0%    0.0%    0.0%    0.0%    0.0%    0.0%    0.0%    0.0%    0.0%    0.0%    0.0%    0.0%    0.0%    0.0%    0.0%    0.0%    0.0%    0.0%    0.0%    0.0%    0.0%    0.0%    0.0%    0.0%    0.0%    0.0%    0.0%    0.0%    0.0%    0.0%    0.0%    0.0%    0.0%    0.0%    0.0%    0.0%    0.0%    0.0%    0.0%    0.0%    0.0%    0.0%    0.0%    0.0%    0.0%    0.0%    0.0%    0.0%    0.0%    0.0%    0.0%    0.0%    0.0%    0.0%    0.0%    0.0%    0.0%    0.0%    0.0%    0.0%    0.0%    0.0%    0.0%    0.0%    0.0%    0.0%    0.0%    0.0%    0.0%    0.0%    0.0%    0.0%    0.0%    0.0%    0.0%    0.0%    0.0%    0.0%    0.0%    0.0%    0.0%    0.0%    0.0%    0.0%    0.0%    0.0%    0.0%    0.0%    0.0%    0.0%    0.0%    0.0%    0.0%    0.0%    0.0%    0.0%    0.0%    0.0%    0.0%    0.0%    0.0%        k__Bacteria;p__BRC1; c__PRR-11     265    0.0%    0.0%    0.0%    0.0%    0.0%    0.0%    0.0%    0.0%    0.0%    0.0%    0.0%    0.0%    0.0%    0.0%    0.0%    0.0%    0.0%    0.0%    0.0%    0.0%    0.0%    0.0%    0.0%    0.0%    0.0%    0.0%    0.0%    0.0%    0.0%    0.0%    0.0%    0.0%    0.0%    0.0%    0.0%    0.0%    0.0%    0.0%    0.0%    0.0%    0.0%    0.0%    0.0%    0.0%    0.0%    0.0%    0.0%    0.0%    0.0%    0.0%    0.0%    0.0%    0.0%    0.0%    0.0%    0.0%    0.0%    0.0%    0.0%    0.0%    0.0%    0.0%    0.0%    0.0%    0.0%    0.0%    0.0%    0.0%    0.0%    0.0%    0.0%    0.0%    0.0%    0.0%    0.0%    0.0%    0.0%    0.0%    0.0%    0.0%    0.0%    0.0%    0.0%    0.0%    0.0%    0.0%    0.0%    0.0%    0.0%    0.0%    0.0%    0.0%    0.0%    0.0%    0.0%    0.0%    0.0%    0.0%    0.0%    0.0%    0.0%    0.0%    0.0%    0.0%    0.0%    0.0%    0.0%    0.0%    0.0%    0.0%    0.0%    0.0%    0.0%    0.0%    0.0%    0.0%    0.0%    0.0%    0.0%    0.0%    0.0%    0.0%    0.0%    0.0%    0.0%    0.0%    0.0%    0.0%    0.0%    0.0%    0.0%    0.0%    0.0%    0.0%    0.0%    0.0%    0.0%    0.0%    0.0%    0.0%    0.0%    0.0%    0.0%    0.0%    0.0%    0.0%    0.0%    0.0%    0.0%    0.0%    0.0%    0.0%    0.0%    0.0%    0.0%    0.0%    0.0%    0.0%    0.0%    0.0%    0.0%    0.0%    0.0%    0.0%    0.0%    0.0%    0.0%    0.0%    0.0%    0.0%    0.0%    0.0%    0.0%    0.0%    0.0%    0.0%    0.0%    0.0%    0.0%    0.0%    0.0%    0.0%    0.0%    0.0%    0.0%    0.0%    0.0%    0.0%    0.0%    0.0%    0.0%    0.0%    0.0%    0.0%    0.0%    0.0%    0.0%    0.0%    0.0%    0.0%    0.0%    0.0%    0.0%    0.0%    0.0%    0.0%    0.0%    0.0%    0.0%    0.0%    0.0%    0.0%    0.0%    0.0%    0.0%    0.0%    0.0%    0.0%    0.0%    0.0%    0.0%    0.0%    0.0%    0.0%    0.0%    0.0%    0.0%    0.0%    0.0%    0.0%    0.0%    0.0%    0.0%    0.0%    0.0%    0.0%    0.0%    0.0%    0.0%    0.0%    0.0%    0.0%    0.0%    0.0%    0.0%    0.0%    0.0%    0.0%    0.0%    0.0%    0.0%    0.0%    0.0%    0.0%    0.0%    0.0%    0.0%    0.0%    0.0%    0.0%    0.0%    0.0%    0.0%    0.0%    0.0%    0.0%    0.0%    0.0%    0.0%    0.0%    0.0%    0.0%    0.0%    0.0%    0.0%    0.0%    0.0%    0.0%    0.0%    0.0%    0.0%    0.0%    0.0%    0.0%    0.0%    0.0%    0.0%    0.0%    0.0%    0.0%    0.0%    0.0%    0.0%    0.0%    0.0%    0.0%    0.0%    0.0%    0.0%    0.0%    0.0%    0.0%    0.0%    0.0%    0.0%    0.0%    0.0%    0.0%    0.0%    0.0%    0.0%    0.0%    0.0%    0.0%    0.0%    0.0%    0.0%    0.0%    0.0%    0.0%    0.0%    0.0%    0.0%    0.0%    0.0%    0.0%    0.0%    0.0%    0.0%    0.0%    0.0%    0.0%    0.0%    0.0%    0.0%    0.0%    0.0%    0.0%    0.0%    0.0%    0.0%    0.0%    0.0%    0.0%    0.0%    0.0%    0.0%    0.0%    0.0%    0.0%    0.0%    0.0%    0.0%    0.0%    0.0%    0.0%    0.0%    0.0%        k__Bacteria;p__Bacteroidetes; c__      67    0.0%    0.0%    0.0%    0.0%    0.0%    0.0%    0.0%    0.0%    0.0%    0.0%    0.0%    0.0%    0.0%    0.0%    0.0%    0.0%    0.0%    0.0%    0.0%    0.0%    0.0%    0.0%    0.0%    0.0%    0.0%    0.0%    0.0%    0.0%    0.0%    0.0%    0.0%    0.0%    0.0%    0.0%    0.0%    0.0%    0.0%    0.0%    0.0%    0.0%    0.0%    0.0%    0.0%    0.0%    0.0%    0.0%    0.0%    0.0%    0.0%    0.0%    0.0%    0.0%    0.0%    0.0%    0.0%    0.0%    0.0%    0.0%    0.0%    0.0%    0.0%    0.0%    0.0%    0.0%    0.0%    0.0%    0.0%    0.0%    0.0%    0.0%    0.0%    0.0%    0.0%    0.0%    0.0%    0.0%    0.0%    0.0%    0.0%    0.0%    0.0%    0.0%    0.0%    0.0%    0.0%    0.0%    0.0%    0.0%    0.0%    0.0%    0.0%    0.0%    0.0%    0.0%    0.0%    0.0%    0.0%    0.0%    0.0%    0.0%    0.0%    0.0%    0.0%    0.0%    0.0%    0.0%    0.0%    0.0%    0.0%    0.0%    0.0%    0.0%    0.0%    0.0%    0.0%    0.0%    0.0%    0.0%    0.0%    0.0%    0.0%    0.0%    0.0%    0.0%    0.0%    0.0%    0.0%    0.0%    0.0%    0.0%    0.0%    0.0%    0.0%    0.0%    0.0%    0.0%    0.0%    0.0%    0.0%    0.0%    0.0%    0.0%    0.0%    0.0%    0.0%    0.0%    0.0%    0.0%    0.0%    0.0%    0.0%    0.0%    0.0%    0.0%    0.0%    0.0%    0.0%    0.0%    0.0%    0.0%    0.0%    0.0%    0.0%    0.0%    0.0%    0.0%    0.0%    0.0%    0.0%    0.0%    0.0%    0.0%    0.0%    0.0%    0.0%    0.0%    0.0%    0.0%    0.0%    0.0%    0.0%    0.0%    0.0%    0.0%    0.0%    0.0%    0.0%    0.0%    0.0%    0.0%    0.0%    0.0%    0.0%    0.0%    0.0%    0.0%    0.0%    0.0%    0.0%    0.0%    0.0%    0.0%    0.0%    0.0%    0.0%    0.0%    0.0%    0.0%    0.0%    0.0%    0.0%    0.0%    0.0%    0.0%    0.0%    0.0%    0.0%    0.0%    0.0%    0.0%    0.0%    0.0%    0.0%    0.0%    0.0%    0.0%    0.0%    0.0%    0.0%    0.0%    0.0%    0.0%    0.0%    0.0%    0.0%    0.0%    0.0%    0.0%    0.0%    0.0%    0.0%    0.0%    0.0%    0.0%    0.0%    0.0%    0.0%    0.0%    0.0%    0.0%    0.0%    0.0%    0.0%    0.0%    0.0%    0.0%    0.0%    0.0%    0.0%    0.0%    0.0%    0.0%    0.0%    0.0%    0.0%    0.0%    0.0%    0.0%    0.0%    0.0%    0.0%    0.0%    0.0%    0.0%    0.0%    0.0%    0.0%    0.0%    0.0%    0.0%    0.0%    0.0%    0.0%    0.0%    0.0%    0.0%    0.0%    0.0%    0.0%    0.0%    0.0%    0.0%    0.0%    0.0%    0.0%    0.0%    0.0%    0.0%    0.0%    0.0%    0.0%    0.0%    0.0%    0.0%    0.0%    0.0%    0.0%    0.0%    0.0%    0.0%    0.0%    0.0%    0.0%    0.0%    0.0%    0.0%    0.0%    0.0%    0.0%    0.0%    0.0%    0.0%    0.0%    0.0%    0.0%    0.0%    0.0%    0.0%    0.0%    0.0%    0.0%    0.0%    0.0%    0.0%    0.0%    0.0%    0.0%    0.0%    0.0%    0.0%    0.0%    0.0%    0.0%    0.0%    0.0%    0.0%    0.0%    0.0%    0.0%    0.0%    0.0%    0.0%    0.0%    0.0%    0.0%    0.0%    0.0%    0.0%        k__Bacteria;p__Bacteroidetes; c__Bacteroidia   499072    2.9%    5.7%    3.2%    5.3%    3.3%    0.2%    4.0%    0.9%    4.7%    7.7%    3.3%    1.5%    1.2%    6.4%    4.5%    2.3%    5.8%    1.9%    0.9%    2.1%    3.0%    4.5%    1.3%    9.5%    4.5%    8.8%    0.7%    3.4%    4.9%    3.0%    4.3%    5.9%    1.6%    6.8%    6.3%   12.9%    3.6%   13.7%    3.3%    1.1%    1.5%    4.1%    3.2%    4.1%    2.6%    2.4%    1.3%    0.9%    1.4%    1.7%    1.2%    0.5%    2.1%    0.9%    1.8%    2.7%    1.6%    0.4%    1.8%    1.0%    1.2%    1.3%    0.9%    1.9%   13.5%    0.9%    0.1%    3.3%    0.5%    1.5%    6.6%    2.6%    0.4%    3.9%    2.6%    0.3%    0.7%    4.6%    4.4%    6.3%   12.9%    0.6%    2.9%    4.9%    2.5%    3.2%    3.7%    0.1%    1.9%    2.3%    0.1%    2.0%    4.0%    0.9%    2.8%    1.2%    2.6%    0.2%    8.6%    7.5%    2.1%    1.2%    1.1%    1.8%    1.8%    2.6%   11.0%    0.3%    0.0%    3.2%    3.2%   13.8%    2.9%    0.5%    6.3%    3.4%    1.8%    1.6%    3.0%    7.2%    0.1%    2.6%    3.4%    4.3%    0.3%    3.3%    3.9%    0.3%    1.5%    2.7%    3.4%    0.7%    0.3%    3.7%    5.3%    0.5%    1.0%    2.0%    0.1%    1.0%    0.1%    3.3%    1.7%    0.3%    0.5%    1.7%    6.9%    3.9%    3.0%    0.2%    0.5%    0.3%    2.4%    1.0%    3.7%    0.4%    1.4%    0.9%    0.5%    2.6%    0.5%    1.9%    1.6%    0.5%    1.2%    0.9%    2.4%    7.1%    2.9%    4.3%    2.3%    3.7%    0.3%    6.0%    1.2%    2.3%    2.2%    1.4%    0.4%    1.4%    1.9%    3.3%    1.6%    0.3%    2.5%    3.2%    1.4%    1.8%    2.1%    2.6%    3.7%    2.8%    3.4%    8.1%    1.3%    1.5%    5.4%    5.2%    1.8%    2.0%    6.1%   15.8%    1.3%    2.9%    2.6%    3.4%    0.7%    6.5%    2.3%    3.3%    2.8%    1.3%    0.6%    5.6%    1.1%    3.7%    2.8%    3.0%    4.6%    2.5%    2.3%    1.5%    0.0%    1.0%    0.6%    3.0%    1.5%    7.6%    2.1%    3.5%    6.5%    1.0%    0.5%    2.2%    2.0%    1.4%    2.0%    1.2%    0.4%    0.6%    1.4%    0.5%    2.7%    2.1%    5.5%    2.5%    1.0%    0.3%    0.7%    4.8%    1.6%    2.8%    0.6%    2.3%    0.3%    1.6%    2.1%    0.7%    1.4%    6.2%    1.3%    2.7%    4.0%    0.6%    3.3%    0.1%    2.2%    5.1%    1.5%    1.8%    2.8%    3.5%    1.7%    5.3%    1.0%    1.5%    1.3%    2.8%    3.7%   11.9%    0.9%    0.3%    1.3%    1.0%    1.3%    8.3%    2.6%    2.6%    5.3%    2.9%    2.5%    1.6%    3.3%    3.7%    1.6%    1.5%    6.1%    4.5%    2.9%    3.7%    6.8%    9.1%   12.9%    4.2%    2.7%    7.7%    7.8%    2.7%    0.6%    4.3%    0.3%    1.8%    0.3%    5.2%    5.5%    2.4%    0.5%    1.7%    1.6%    1.3%    9.5%    3.7%   25.8%    0.9%    3.1%    5.1%    3.4%    5.3%    2.3%    4.9%    3.8%    2.0%    4.2%    2.0%    2.4%    0.8%    3.2%    7.8%    4.4%    0.2%    1.1%    0.6%    1.6%    1.4%    1.0%    3.5%    0.5%    6.2%    0.2%    1.2%    1.6%    4.4%    2.4%    1.4%    0.8%    0.4%   26.5%    1.3%        k__Bacteria;p__Bacteroidetes; c__Flavobacteria   168640    1.0%    1.9%    2.2%    2.7%    1.0%    1.8%    1.2%    0.4%    0.1%    0.5%    1.0%    0.2%    0.9%    1.5%    1.4%    0.7%    1.1%    0.9%    0.4%    0.4%    1.2%    2.7%    0.4%    0.0%    0.3%    0.3%    0.5%    0.5%    2.6%    0.2%    0.1%    0.8%    0.3%    1.1%    1.6%    1.9%    1.8%    0.1%    0.8%    0.8%    0.9%    1.4%    2.7%    1.7%    1.4%    0.7%    1.0%    0.2%    1.9%    0.8%    1.2%    2.9%    3.8%    1.3%    0.3%    0.3%    0.5%    0.2%    1.8%    0.8%    0.3%    0.5%    0.8%    1.0%    0.2%    0.3%    1.1%    1.4%    1.0%    0.5%    2.2%    0.3%    0.1%    1.2%    0.8%    0.1%    0.6%    0.5%    0.1%    0.5%    0.1%    1.7%    0.6%    0.6%    0.2%    0.4%    1.2%    1.1%    1.3%    0.9%    0.1%    0.3%    0.5%    0.9%    0.2%    0.2%    1.3%    0.2%    0.4%    0.6%    1.1%    0.2%    0.1%    0.2%    0.2%    1.5%    3.1%    1.5%    2.9%    0.4%    0.2%    1.1%    0.5%    0.1%    0.4%    0.3%    0.4%    0.3%    1.8%    2.5%    3.6%    0.7%    0.5%    0.8%    0.1%    2.2%    0.5%    0.3%    1.5%    2.2%    0.7%    0.2%    0.3%    0.5%    2.9%    0.3%    0.6%    1.0%    1.1%    0.7%    1.9%    2.7%    1.6%    6.8%    0.8%    0.3%    0.9%    0.2%    0.6%    1.7%    0.7%    0.3%    3.0%    0.7%    0.4%    0.6%    4.8%    1.0%    0.6%    1.2%    0.6%    0.9%    7.4%    0.8%    0.9%    0.4%    1.2%    0.6%    0.5%    0.9%    6.8%    1.8%    0.3%    1.4%    0.4%    0.4%    0.3%    0.7%    0.7%    0.6%    0.2%    0.3%    4.7%    0.3%    0.2%    0.2%    0.2%    0.4%    0.4%    0.5%    0.2%    0.4%    0.8%    0.6%    0.6%    0.9%    1.1%    0.4%    0.2%    0.2%    3.2%    0.3%    0.4%    1.1%    1.9%    0.8%    0.7%    1.7%    2.1%    1.2%    1.0%    0.3%    0.4%    4.4%    0.5%    0.6%    0.7%    0.1%    0.5%    0.4%    0.8%    2.5%    3.1%    1.6%    0.3%    0.8%    0.3%    0.8%    0.7%    2.1%    0.2%    0.0%    2.8%    0.4%    1.1%    0.6%    1.0%    0.1%    0.1%    0.1%    0.4%    0.1%    0.3%    0.5%    0.8%    0.0%    0.2%    0.1%    0.1%    0.4%    0.9%    1.7%    2.2%    0.2%    0.7%    0.2%    0.8%    0.2%    1.2%    1.2%    0.8%    2.0%    1.6%    4.8%    1.1%    0.5%    0.7%    2.1%    0.8%    0.7%    0.8%    1.0%    2.1%    1.2%    0.2%    0.7%    1.0%    0.5%    0.9%    0.3%    0.2%    0.2%    0.2%    0.2%    0.4%    0.9%    2.2%    1.3%    0.4%    0.2%    0.7%    0.1%    1.5%    1.5%    1.0%    0.2%    1.9%    0.9%    0.9%    0.7%    0.3%    0.4%    0.9%    0.3%    1.0%    0.7%    0.4%    0.3%    0.4%    2.2%    0.3%    0.2%    0.2%    1.9%    0.7%    1.1%    0.1%    0.9%    0.6%    1.7%    1.4%    1.7%    0.2%    0.2%    1.0%    1.0%    1.0%    0.6%    1.5%    0.6%    0.5%    0.7%    0.5%    0.4%    0.0%    0.4%    2.8%    0.9%    1.5%    4.0%    0.9%    2.6%    0.7%    0.7%    1.0%    0.3%    0.7%    0.9%    0.1%    0.2%    0.7%    1.5%    0.2%    0.6%    0.2%    0.3%    0.0%    0.0%        k__Bacteria;p__Bacteroidetes; c__Sphingobacteria   351755    2.1%    0.1%    0.6%    0.1%    0.4%    1.2%    0.0%    0.0%    0.0%    0.0%    0.0%    0.1%    0.7%    0.4%    0.1%    0.3%    0.4%    1.0%    0.2%    0.7%    0.7%    1.2%    0.5%    0.0%    0.0%    0.2%    0.7%    0.5%    0.8%    0.1%    0.1%    0.8%    0.3%    0.1%    0.9%    0.1%    0.7%    0.1%    0.4%    1.6%    0.3%    0.6%    0.0%    0.0%    0.6%    0.2%    0.1%    0.1%    0.6%    0.0%    0.8%    0.4%    0.3%    0.9%    0.7%    0.3%    0.8%    0.2%    0.5%    1.7%    1.0%    0.4%    0.4%    1.8%    1.2%    0.6%    2.3%    1.4%    1.3%    0.8%    1.0%    0.4%    0.3%    1.1%    1.3%    0.1%    0.7%    0.9%    0.2%    0.6%    0.3%    1.1%    0.2%    0.2%    0.1%    0.0%    1.2%    0.8%    0.6%    0.8%    0.6%    1.4%    0.7%    0.2%    0.2%    1.0%    1.3%    1.4%    2.3%    0.5%    1.5%   10.8%    9.5%    0.7%    0.1%    0.1%    1.4%    1.5%    2.6%    0.7%    0.5%    0.9%    0.2%    0.1%    0.8%    0.2%    5.9%    0.4%    0.4%    6.0%    6.2%    0.7%    1.4%    2.1%    0.2%    5.6%    1.8%    0.6%    0.9%    1.7%    0.9%    6.0%    4.0%    0.3%    1.8%   10.0%    1.6%    3.2%    1.5%    6.3%    0.4%    3.6%    5.6%    1.7%    3.7%    6.6%    4.2%    7.9%    0.6%    1.8%    7.5%   10.8%    1.7%   14.2%    0.2%   10.2%    2.4%    1.0%    0.4%    2.6%    2.3%    2.4%    1.2%    0.8%   11.6%    0.7%    1.3%    0.3%    1.0%    0.4%    3.5%    1.4%    1.2%    1.1%    6.8%    1.4%    0.5%    0.1%    1.4%    0.5%    0.3%    0.1%    3.8%    2.1%    0.8%    0.2%    0.3%    0.1%    0.1%    0.3%    0.3%    0.4%    0.2%    2.9%    1.4%    9.3%    1.6%    0.3%    0.3%    0.1%    1.3%    0.5%    0.8%    2.0%    2.8%    4.7%    0.7%    1.4%    3.6%    1.9%    1.4%   13.4%   12.0%    0.9%    3.7%    0.7%    7.7%    1.5%    1.6%    2.3%    1.1%    7.9%    0.8%    9.6%   11.4%    1.8%    2.6%    3.3%    1.6%    1.9%    6.2%    7.7%    3.7%    6.2%    3.5%    0.2%    2.4%    7.7%    4.3%    1.1%    0.8%    1.0%    0.9%    0.2%    0.8%    0.2%    0.2%    4.1%    1.6%    2.3%   11.1%    1.9%    3.3%    3.6%    3.5%    6.8%    3.5%   18.4%    4.0%    3.1%    7.2%    4.8%    2.7%    4.9%    7.9%    1.5%    2.5%    3.5%    5.0%   13.8%    4.1%    1.5%    6.8%    1.4%   17.1%    7.2%    1.8%    0.9%    0.3%    0.4%    0.6%    2.0%    7.4%    1.0%    9.8%    0.1%    0.6%    4.9%    0.6%    0.4%    1.3%    0.1%    1.2%    1.6%    1.0%    0.3%    0.7%    0.6%    0.6%    1.3%    0.4%    0.4%    0.1%    1.6%    0.6%    0.3%    0.6%    0.5%    0.8%    0.2%    0.0%    0.1%    0.1%    0.1%    0.3%    1.9%    0.0%    0.2%    0.0%    0.3%    0.3%    0.7%    0.1%    0.4%    1.2%    0.8%    1.0%    0.6%    0.8%    0.8%    0.0%    0.0%    0.1%    0.1%    0.0%    0.0%    0.0%    0.2%    0.4%   11.6%    1.0%    8.0%    0.6%    0.5%    2.4%    0.1%    2.4%    0.2%    3.5%    0.2%    0.5%    0.1%    0.4%    1.5%    0.2%    1.3%    0.0%    0.3%        k__Bacteria;p__CCM11b; c__      40    0.0%    0.0%    0.0%    0.0%    0.0%    0.0%    0.0%    0.0%    0.0%    0.0%    0.0%    0.0%    0.0%    0.0%    0.0%    0.0%    0.0%    0.0%    0.0%    0.0%    0.0%    0.0%    0.0%    0.0%    0.0%    0.0%    0.0%    0.0%    0.0%    0.0%    0.0%    0.0%    0.0%    0.0%    0.0%    0.0%    0.0%    0.0%    0.0%    0.0%    0.0%    0.0%    0.0%    0.0%    0.0%    0.0%    0.0%    0.0%    0.0%    0.0%    0.0%    0.0%    0.0%    0.0%    0.0%    0.0%    0.0%    0.0%    0.0%    0.0%    0.0%    0.0%    0.0%    0.0%    0.0%    0.0%    0.0%    0.0%    0.0%    0.0%    0.0%    0.0%    0.0%    0.0%    0.0%    0.0%    0.0%    0.0%    0.0%    0.0%    0.0%    0.0%    0.0%    0.0%    0.0%    0.0%    0.0%    0.0%    0.0%    0.0%    0.0%    0.0%    0.0%    0.0%    0.0%    0.0%    0.0%    0.0%    0.0%    0.0%    0.0%    0.0%    0.0%    0.0%    0.0%    0.0%    0.0%    0.0%    0.0%    0.0%    0.0%    0.0%    0.0%    0.0%    0.0%    0.0%    0.0%    0.0%    0.0%    0.0%    0.0%    0.0%    0.0%    0.0%    0.0%    0.0%    0.0%    0.0%    0.0%    0.0%    0.0%    0.0%    0.0%    0.0%    0.0%    0.0%    0.0%    0.0%    0.0%    0.0%    0.0%    0.0%    0.0%    0.0%    0.0%    0.0%    0.0%    0.0%    0.0%    0.0%    0.0%    0.0%    0.0%    0.0%    0.0%    0.0%    0.0%    0.0%    0.0%    0.0%    0.0%    0.0%    0.0%    0.0%    0.0%    0.0%    0.0%    0.0%    0.0%    0.0%    0.0%    0.0%    0.0%    0.0%    0.0%    0.0%    0.0%    0.0%    0.0%    0.0%    0.0%    0.0%    0.0%    0.0%    0.0%    0.0%    0.0%    0.0%    0.0%    0.0%    0.0%    0.0%    0.0%    0.0%    0.0%    0.0%    0.0%    0.0%    0.0%    0.0%    0.0%    0.0%    0.0%    0.0%    0.0%    0.0%    0.0%    0.0%    0.0%    0.0%    0.0%    0.0%    0.0%    0.0%    0.0%    0.0%    0.0%    0.0%    0.0%    0.0%    0.0%    0.0%    0.0%    0.0%    0.0%    0.0%    0.0%    0.0%    0.0%    0.0%    0.0%    0.0%    0.0%    0.0%    0.0%    0.0%    0.0%    0.0%    0.0%    0.0%    0.0%    0.0%    0.0%    0.0%    0.0%    0.0%    0.0%    0.0%    0.0%    0.0%    0.0%    0.0%    0.0%    0.0%    0.0%    0.0%    0.0%    0.0%    0.0%    0.0%    0.0%    0.0%    0.0%    0.0%    0.0%    0.0%    0.0%    0.0%    0.0%    0.0%    0.0%    0.0%    0.0%    0.0%    0.0%    0.0%    0.0%    0.0%    0.0%    0.0%    0.0%    0.0%    0.0%    0.0%    0.0%    0.0%    0.0%    0.0%    0.0%    0.0%    0.0%    0.0%    0.0%    0.0%    0.0%    0.0%    0.0%    0.0%    0.0%    0.0%    0.0%    0.0%    0.0%    0.0%    0.0%    0.0%    0.0%    0.0%    0.0%    0.0%    0.0%    0.0%    0.0%    0.0%    0.0%    0.0%    0.0%    0.0%    0.0%    0.0%    0.0%    0.0%    0.0%    0.0%    0.0%    0.0%    0.0%    0.0%    0.0%    0.0%    0.0%    0.0%    0.0%    0.0%    0.0%    0.0%    0.0%    0.0%    0.0%    0.0%    0.0%    0.0%    0.0%    0.0%    0.0%    0.0%    0.0%    0.0%    0.0%    0.0%    0.0%    0.0%    0.0%    0.0%    0.0%    0.0%    0.0%    0.0%        k__Bacteria;p__Caldithrix_KSB1; c__Caldithrixae       1    0.0%    0.0%    0.0%    0.0%    0.0%    0.0%    0.0%    0.0%    0.0%    0.0%    0.0%    0.0%    0.0%    0.0%    0.0%    0.0%    0.0%    0.0%    0.0%    0.0%    0.0%    0.0%    0.0%    0.0%    0.0%    0.0%    0.0%    0.0%    0.0%    0.0%    0.0%    0.0%    0.0%    0.0%    0.0%    0.0%    0.0%    0.0%    0.0%    0.0%    0.0%    0.0%    0.0%    0.0%    0.0%    0.0%    0.0%    0.0%    0.0%    0.0%    0.0%    0.0%    0.0%    0.0%    0.0%    0.0%    0.0%    0.0%    0.0%    0.0%    0.0%    0.0%    0.0%    0.0%    0.0%    0.0%    0.0%    0.0%    0.0%    0.0%    0.0%    0.0%    0.0%    0.0%    0.0%    0.0%    0.0%    0.0%    0.0%    0.0%    0.0%    0.0%    0.0%    0.0%    0.0%    0.0%    0.0%    0.0%    0.0%    0.0%    0.0%    0.0%    0.0%    0.0%    0.0%    0.0%    0.0%    0.0%    0.0%    0.0%    0.0%    0.0%    0.0%    0.0%    0.0%    0.0%    0.0%    0.0%    0.0%    0.0%    0.0%    0.0%    0.0%    0.0%    0.0%    0.0%    0.0%    0.0%    0.0%    0.0%    0.0%    0.0%    0.0%    0.0%    0.0%    0.0%    0.0%    0.0%    0.0%    0.0%    0.0%    0.0%    0.0%    0.0%    0.0%    0.0%    0.0%    0.0%    0.0%    0.0%    0.0%    0.0%    0.0%    0.0%    0.0%    0.0%    0.0%    0.0%    0.0%    0.0%    0.0%    0.0%    0.0%    0.0%    0.0%    0.0%    0.0%    0.0%    0.0%    0.0%    0.0%    0.0%    0.0%    0.0%    0.0%    0.0%    0.0%    0.0%    0.0%    0.0%    0.0%    0.0%    0.0%    0.0%    0.0%    0.0%    0.0%    0.0%    0.0%    0.0%    0.0%    0.0%    0.0%    0.0%    0.0%    0.0%    0.0%    0.0%    0.0%    0.0%    0.0%    0.0%    0.0%    0.0%    0.0%    0.0%    0.0%    0.0%    0.0%    0.0%    0.0%    0.0%    0.0%    0.0%    0.0%    0.0%    0.0%    0.0%    0.0%    0.0%    0.0%    0.0%    0.0%    0.0%    0.0%    0.0%    0.0%    0.0%    0.0%    0.0%    0.0%    0.0%    0.0%    0.0%    0.0%    0.0%    0.0%    0.0%    0.0%    0.0%    0.0%    0.0%    0.0%    0.0%    0.0%    0.0%    0.0%    0.0%    0.0%    0.0%    0.0%    0.0%    0.0%    0.0%    0.0%    0.0%    0.0%    0.0%    0.0%    0.0%    0.0%    0.0%    0.0%    0.0%    0.0%    0.0%    0.0%    0.0%    0.0%    0.0%    0.0%    0.0%    0.0%    0.0%    0.0%    0.0%    0.0%    0.0%    0.0%    0.0%    0.0%    0.0%    0.0%    0.0%    0.0%    0.0%    0.0%    0.0%    0.0%    0.0%    0.0%    0.0%    0.0%    0.0%    0.0%    0.0%    0.0%    0.0%    0.0%    0.0%    0.0%    0.0%    0.0%    0.0%    0.0%    0.0%    0.0%    0.0%    0.0%    0.0%    0.0%    0.0%    0.0%    0.0%    0.0%    0.0%    0.0%    0.0%    0.0%    0.0%    0.0%    0.0%    0.0%    0.0%    0.0%    0.0%    0.0%    0.0%    0.0%    0.0%    0.0%    0.0%    0.0%    0.0%    0.0%    0.0%    0.0%    0.0%    0.0%    0.0%    0.0%    0.0%    0.0%    0.0%    0.0%    0.0%    0.0%    0.0%    0.0%    0.0%    0.0%    0.0%    0.0%    0.0%    0.0%    0.0%    0.0%    0.0%    0.0%    0.0%    0.0%    0.0%    0.0%    0.0%    0.0%    0.0%    0.0%    0.0%        k__Bacteria;p__Chlamydiae; c__Chlamydiae (class)      25    0.0%    0.0%    0.0%    0.0%    0.0%    0.0%    0.0%    0.0%    0.0%    0.0%    0.0%    0.0%    0.0%    0.0%    0.0%    0.0%    0.0%    0.0%    0.0%    0.0%    0.0%    0.0%    0.0%    0.0%    0.0%    0.0%    0.0%    0.0%    0.0%    0.0%    0.0%    0.0%    0.0%    0.0%    0.0%    0.0%    0.0%    0.0%    0.0%    0.0%    0.0%    0.0%    0.0%    0.0%    0.0%    0.0%    0.0%    0.0%    0.0%    0.0%    0.0%    0.0%    0.0%    0.0%    0.0%    0.0%    0.0%    0.0%    0.0%    0.0%    0.0%    0.0%    0.0%    0.0%    0.0%    0.0%    0.0%    0.0%    0.0%    0.0%    0.0%    0.0%    0.0%    0.0%    0.0%    0.0%    0.0%    0.0%    0.0%    0.0%    0.0%    0.0%    0.0%    0.0%    0.0%    0.0%    0.0%    0.0%    0.0%    0.0%    0.0%    0.0%    0.0%    0.0%    0.0%    0.0%    0.0%    0.0%    0.0%    0.0%    0.0%    0.0%    0.0%    0.0%    0.0%    0.0%    0.0%    0.0%    0.0%    0.0%    0.0%    0.0%    0.0%    0.0%    0.0%    0.0%    0.0%    0.0%    0.0%    0.0%    0.0%    0.0%    0.0%    0.0%    0.0%    0.0%    0.0%    0.0%    0.0%    0.0%    0.0%    0.0%    0.0%    0.0%    0.0%    0.0%    0.0%    0.0%    0.0%    0.0%    0.0%    0.0%    0.0%    0.0%    0.0%    0.0%    0.0%    0.0%    0.0%    0.0%    0.0%    0.0%    0.0%    0.0%    0.0%    0.0%    0.0%    0.0%    0.0%    0.0%    0.0%    0.0%    0.0%    0.0%    0.0%    0.0%    0.0%    0.0%    0.0%    0.0%    0.0%    0.0%    0.0%    0.0%    0.0%    0.0%    0.0%    0.0%    0.0%    0.0%    0.0%    0.0%    0.0%    0.0%    0.0%    0.0%    0.0%    0.0%    0.0%    0.0%    0.0%    0.0%    0.0%    0.0%    0.0%    0.0%    0.0%    0.0%    0.0%    0.0%    0.0%    0.0%    0.0%    0.0%    0.0%    0.0%    0.0%    0.0%    0.0%    0.0%    0.0%    0.0%    0.0%    0.0%    0.0%    0.0%    0.0%    0.0%    0.0%    0.0%    0.0%    0.0%    0.0%    0.0%    0.0%    0.0%    0.0%    0.0%    0.0%    0.0%    0.0%    0.0%    0.0%    0.0%    0.0%    0.0%    0.0%    0.0%    0.0%    0.0%    0.0%    0.0%    0.0%    0.0%    0.0%    0.0%    0.0%    0.0%    0.0%    0.0%    0.0%    0.0%    0.0%    0.0%    0.0%    0.0%    0.0%    0.0%    0.0%    0.0%    0.0%    0.0%    0.0%    0.0%    0.0%    0.0%    0.0%    0.0%    0.0%    0.0%    0.0%    0.0%    0.0%    0.0%    0.0%    0.0%    0.0%    0.0%    0.0%    0.0%    0.0%    0.0%    0.0%    0.0%    0.0%    0.0%    0.0%    0.0%    0.0%    0.0%    0.0%    0.0%    0.0%    0.0%    0.0%    0.0%    0.0%    0.0%    0.0%    0.0%    0.0%    0.0%    0.0%    0.0%    0.0%    0.0%    0.0%    0.0%    0.0%    0.0%    0.0%    0.0%    0.0%    0.0%    0.0%    0.0%    0.0%    0.0%    0.0%    0.0%    0.0%    0.0%    0.0%    0.0%    0.0%    0.0%    0.0%    0.0%    0.0%    0.0%    0.0%    0.0%    0.0%    0.0%    0.0%    0.0%    0.0%    0.0%    0.0%    0.0%    0.0%    0.0%    0.0%    0.0%    0.0%    0.0%    0.0%    0.0%    0.0%    0.0%    0.0%    0.0%    0.0%    0.0%    0.0%    0.0%    0.0%    0.0%        k__Bacteria;p__Chlorobi; c__       1    0.0%    0.0%    0.0%    0.0%    0.0%    0.0%    0.0%    0.0%    0.0%    0.0%    0.0%    0.0%    0.0%    0.0%    0.0%    0.0%    0.0%    0.0%    0.0%    0.0%    0.0%    0.0%    0.0%    0.0%    0.0%    0.0%    0.0%    0.0%    0.0%    0.0%    0.0%    0.0%    0.0%    0.0%    0.0%    0.0%    0.0%    0.0%    0.0%    0.0%    0.0%    0.0%    0.0%    0.0%    0.0%    0.0%    0.0%    0.0%    0.0%    0.0%    0.0%    0.0%    0.0%    0.0%    0.0%    0.0%    0.0%    0.0%    0.0%    0.0%    0.0%    0.0%    0.0%    0.0%    0.0%    0.0%    0.0%    0.0%    0.0%    0.0%    0.0%    0.0%    0.0%    0.0%    0.0%    0.0%    0.0%    0.0%    0.0%    0.0%    0.0%    0.0%    0.0%    0.0%    0.0%    0.0%    0.0%    0.0%    0.0%    0.0%    0.0%    0.0%    0.0%    0.0%    0.0%    0.0%    0.0%    0.0%    0.0%    0.0%    0.0%    0.0%    0.0%    0.0%    0.0%    0.0%    0.0%    0.0%    0.0%    0.0%    0.0%    0.0%    0.0%    0.0%    0.0%    0.0%    0.0%    0.0%    0.0%    0.0%    0.0%    0.0%    0.0%    0.0%    0.0%    0.0%    0.0%    0.0%    0.0%    0.0%    0.0%    0.0%    0.0%    0.0%    0.0%    0.0%    0.0%    0.0%    0.0%    0.0%    0.0%    0.0%    0.0%    0.0%    0.0%    0.0%    0.0%    0.0%    0.0%    0.0%    0.0%    0.0%    0.0%    0.0%    0.0%    0.0%    0.0%    0.0%    0.0%    0.0%    0.0%    0.0%    0.0%    0.0%    0.0%    0.0%    0.0%    0.0%    0.0%    0.0%    0.0%    0.0%    0.0%    0.0%    0.0%    0.0%    0.0%    0.0%    0.0%    0.0%    0.0%    0.0%    0.0%    0.0%    0.0%    0.0%    0.0%    0.0%    0.0%    0.0%    0.0%    0.0%    0.0%    0.0%    0.0%    0.0%    0.0%    0.0%    0.0%    0.0%    0.0%    0.0%    0.0%    0.0%    0.0%    0.0%    0.0%    0.0%    0.0%    0.0%    0.0%    0.0%    0.0%    0.0%    0.0%    0.0%    0.0%    0.0%    0.0%    0.0%    0.0%    0.0%    0.0%    0.0%    0.0%    0.0%    0.0%    0.0%    0.0%    0.0%    0.0%    0.0%    0.0%    0.0%    0.0%    0.0%    0.0%    0.0%    0.0%    0.0%    0.0%    0.0%    0.0%    0.0%    0.0%    0.0%    0.0%    0.0%    0.0%    0.0%    0.0%    0.0%    0.0%    0.0%    0.0%    0.0%    0.0%    0.0%    0.0%    0.0%    0.0%    0.0%    0.0%    0.0%    0.0%    0.0%    0.0%    0.0%    0.0%    0.0%    0.0%    0.0%    0.0%    0.0%    0.0%    0.0%    0.0%    0.0%    0.0%    0.0%    0.0%    0.0%    0.0%    0.0%    0.0%    0.0%    0.0%    0.0%    0.0%    0.0%    0.0%    0.0%    0.0%    0.0%    0.0%    0.0%    0.0%    0.0%    0.0%    0.0%    0.0%    0.0%    0.0%    0.0%    0.0%    0.0%    0.0%    0.0%    0.0%    0.0%    0.0%    0.0%    0.0%    0.0%    0.0%    0.0%    0.0%    0.0%    0.0%    0.0%    0.0%    0.0%    0.0%    0.0%    0.0%    0.0%    0.0%    0.0%    0.0%    0.0%    0.0%    0.0%    0.0%    0.0%    0.0%    0.0%    0.0%    0.0%    0.0%    0.0%    0.0%    0.0%    0.0%    0.0%    0.0%    0.0%    0.0%    0.0%    0.0%    0.0%    0.0%    0.0%    0.0%    0.0%    0.0%    0.0%    0.0%    0.0%        k__Bacteria;p__Chlorobi; c__BSV19       0    0.0%    0.0%    0.0%    0.0%    0.0%    0.0%    0.0%    0.0%    0.0%    0.0%    0.0%    0.0%    0.0%    0.0%    0.0%    0.0%    0.0%    0.0%    0.0%    0.0%    0.0%    0.0%    0.0%    0.0%    0.0%    0.0%    0.0%    0.0%    0.0%    0.0%    0.0%    0.0%    0.0%    0.0%    0.0%    0.0%    0.0%    0.0%    0.0%    0.0%    0.0%    0.0%    0.0%    0.0%    0.0%    0.0%    0.0%    0.0%    0.0%    0.0%    0.0%    0.0%    0.0%    0.0%    0.0%    0.0%    0.0%    0.0%    0.0%    0.0%    0.0%    0.0%    0.0%    0.0%    0.0%    0.0%    0.0%    0.0%    0.0%    0.0%    0.0%    0.0%    0.0%    0.0%    0.0%    0.0%    0.0%    0.0%    0.0%    0.0%    0.0%    0.0%    0.0%    0.0%    0.0%    0.0%    0.0%    0.0%    0.0%    0.0%    0.0%    0.0%    0.0%    0.0%    0.0%    0.0%    0.0%    0.0%    0.0%    0.0%    0.0%    0.0%    0.0%    0.0%    0.0%    0.0%    0.0%    0.0%    0.0%    0.0%    0.0%    0.0%    0.0%    0.0%    0.0%    0.0%    0.0%    0.0%    0.0%    0.0%    0.0%    0.0%    0.0%    0.0%    0.0%    0.0%    0.0%    0.0%    0.0%    0.0%    0.0%    0.0%    0.0%    0.0%    0.0%    0.0%    0.0%    0.0%    0.0%    0.0%    0.0%    0.0%    0.0%    0.0%    0.0%    0.0%    0.0%    0.0%    0.0%    0.0%    0.0%    0.0%    0.0%    0.0%    0.0%    0.0%    0.0%    0.0%    0.0%    0.0%    0.0%    0.0%    0.0%    0.0%    0.0%    0.0%    0.0%    0.0%    0.0%    0.0%    0.0%    0.0%    0.0%    0.0%    0.0%    0.0%    0.0%    0.0%    0.0%    0.0%    0.0%    0.0%    0.0%    0.0%    0.0%    0.0%    0.0%    0.0%    0.0%    0.0%    0.0%    0.0%    0.0%    0.0%    0.0%    0.0%    0.0%    0.0%    0.0%    0.0%    0.0%    0.0%    0.0%    0.0%    0.0%    0.0%    0.0%    0.0%    0.0%    0.0%    0.0%    0.0%    0.0%    0.0%    0.0%    0.0%    0.0%    0.0%    0.0%    0.0%    0.0%    0.0%    0.0%    0.0%    0.0%    0.0%    0.0%    0.0%    0.0%    0.0%    0.0%    0.0%    0.0%    0.0%    0.0%    0.0%    0.0%    0.0%    0.0%    0.0%    0.0%    0.0%    0.0%    0.0%    0.0%    0.0%    0.0%    0.0%    0.0%    0.0%    0.0%    0.0%    0.0%    0.0%    0.0%    0.0%    0.0%    0.0%    0.0%    0.0%    0.0%    0.0%    0.0%    0.0%    0.0%    0.0%    0.0%    0.0%    0.0%    0.0%    0.0%    0.0%    0.0%    0.0%    0.0%    0.0%    0.0%    0.0%    0.0%    0.0%    0.0%    0.0%    0.0%    0.0%    0.0%    0.0%    0.0%    0.0%    0.0%    0.0%    0.0%    0.0%    0.0%    0.0%    0.0%    0.0%    0.0%    0.0%    0.0%    0.0%    0.0%    0.0%    0.0%    0.0%    0.0%    0.0%    0.0%    0.0%    0.0%    0.0%    0.0%    0.0%    0.0%    0.0%    0.0%    0.0%    0.0%    0.0%    0.0%    0.0%    0.0%    0.0%    0.0%    0.0%    0.0%    0.0%    0.0%    0.0%    0.0%    0.0%    0.0%    0.0%    0.0%    0.0%    0.0%    0.0%    0.0%    0.0%    0.0%    0.0%    0.0%    0.0%    0.0%    0.0%    0.0%    0.0%    0.0%    0.0%    0.0%    0.0%    0.0%    0.0%    0.0%    0.0%    0.0%    0.0%    0.0%    0.0%        k__Bacteria;p__Chlorobi; c__OPB56      21    0.0%    0.0%    0.0%    0.0%    0.0%    0.0%    0.0%    0.0%    0.0%    0.0%    0.0%    0.0%    0.0%    0.0%    0.0%    0.0%    0.0%    0.0%    0.0%    0.0%    0.0%    0.0%    0.0%    0.0%    0.0%    0.0%    0.0%    0.0%    0.0%    0.0%    0.0%    0.0%    0.0%    0.0%    0.0%    0.0%    0.0%    0.0%    0.0%    0.0%    0.0%    0.0%    0.0%    0.0%    0.0%    0.0%    0.0%    0.0%    0.0%    0.0%    0.0%    0.0%    0.0%    0.0%    0.0%    0.0%    0.0%    0.0%    0.0%    0.0%    0.0%    0.0%    0.0%    0.0%    0.0%    0.0%    0.0%    0.0%    0.0%    0.0%    0.0%    0.0%    0.0%    0.0%    0.0%    0.0%    0.0%    0.0%    0.0%    0.0%    0.0%    0.0%    0.0%    0.0%    0.0%    0.0%    0.0%    0.0%    0.0%    0.0%    0.0%    0.0%    0.0%    0.0%    0.0%    0.0%    0.0%    0.0%    0.0%    0.0%    0.0%    0.0%    0.0%    0.0%    0.0%    0.0%    0.0%    0.0%    0.0%    0.0%    0.0%    0.0%    0.0%    0.0%    0.0%    0.0%    0.0%    0.0%    0.0%    0.0%    0.0%    0.0%    0.0%    0.0%    0.0%    0.0%    0.0%    0.0%    0.0%    0.0%    0.0%    0.0%    0.0%    0.0%    0.0%    0.0%    0.0%    0.0%    0.0%    0.0%    0.0%    0.0%    0.0%    0.0%    0.0%    0.0%    0.0%    0.0%    0.0%    0.0%    0.0%    0.0%    0.0%    0.0%    0.0%    0.0%    0.0%    0.0%    0.0%    0.0%    0.0%    0.0%    0.0%    0.0%    0.0%    0.0%    0.0%    0.0%    0.0%    0.0%    0.0%    0.0%    0.0%    0.0%    0.0%    0.0%    0.0%    0.0%    0.0%    0.0%    0.0%    0.0%    0.0%    0.0%    0.0%    0.0%    0.0%    0.0%    0.0%    0.0%    0.0%    0.0%    0.0%    0.0%    0.0%    0.0%    0.0%    0.0%    0.0%    0.0%    0.0%    0.0%    0.0%    0.0%    0.0%    0.0%    0.0%    0.0%    0.0%    0.0%    0.0%    0.0%    0.0%    0.0%    0.0%    0.0%    0.0%    0.0%    0.0%    0.0%    0.0%    0.0%    0.0%    0.0%    0.0%    0.0%    0.0%    0.0%    0.0%    0.0%    0.0%    0.0%    0.0%    0.0%    0.0%    0.0%    0.0%    0.0%    0.0%    0.0%    0.0%    0.0%    0.0%    0.0%    0.0%    0.0%    0.0%    0.0%    0.0%    0.0%    0.0%    0.0%    0.0%    0.0%    0.0%    0.0%    0.0%    0.0%    0.0%    0.0%    0.0%    0.0%    0.0%    0.0%    0.0%    0.0%    0.0%    0.0%    0.0%    0.0%    0.0%    0.0%    0.0%    0.0%    0.0%    0.0%    0.0%    0.0%    0.0%    0.0%    0.0%    0.0%    0.0%    0.0%    0.0%    0.0%    0.0%    0.0%    0.0%    0.0%    0.0%    0.0%    0.0%    0.0%    0.0%    0.0%    0.0%    0.0%    0.0%    0.0%    0.0%    0.0%    0.0%    0.0%    0.0%    0.0%    0.0%    0.0%    0.0%    0.0%    0.0%    0.0%    0.0%    0.0%    0.0%    0.0%    0.0%    0.0%    0.0%    0.0%    0.0%    0.0%    0.0%    0.0%    0.0%    0.0%    0.0%    0.0%    0.0%    0.0%    0.0%    0.0%    0.0%    0.0%    0.0%    0.0%    0.0%    0.0%    0.0%    0.0%    0.0%    0.0%    0.0%    0.0%    0.0%    0.0%    0.0%    0.0%    0.0%    0.0%    0.0%    0.0%    0.0%    0.0%    0.0%    0.0%    0.0%    0.0%        k__Bacteria;p__Chlorobi; c__SJA-28      12    0.0%    0.0%    0.0%    0.0%    0.0%    0.0%    0.0%    0.0%    0.0%    0.0%    0.0%    0.0%    0.0%    0.0%    0.0%    0.0%    0.0%    0.0%    0.0%    0.0%    0.0%    0.0%    0.0%    0.0%    0.0%    0.0%    0.0%    0.0%    0.0%    0.0%    0.0%    0.0%    0.0%    0.0%    0.0%    0.0%    0.0%    0.0%    0.0%    0.0%    0.0%    0.0%    0.0%    0.0%    0.0%    0.0%    0.0%    0.0%    0.0%    0.0%    0.0%    0.0%    0.0%    0.0%    0.0%    0.0%    0.0%    0.0%    0.0%    0.0%    0.0%    0.0%    0.0%    0.0%    0.0%    0.0%    0.0%    0.0%    0.0%    0.0%    0.0%    0.0%    0.0%    0.0%    0.0%    0.0%    0.0%    0.0%    0.0%    0.0%    0.0%    0.0%    0.0%    0.0%    0.0%    0.0%    0.0%    0.0%    0.0%    0.0%    0.0%    0.0%    0.0%    0.0%    0.0%    0.0%    0.0%    0.0%    0.0%    0.0%    0.0%    0.0%    0.0%    0.0%    0.0%    0.0%    0.0%    0.0%    0.0%    0.0%    0.0%    0.0%    0.0%    0.0%    0.0%    0.0%    0.0%    0.0%    0.0%    0.0%    0.0%    0.0%    0.0%    0.0%    0.0%    0.0%    0.0%    0.0%    0.0%    0.0%    0.0%    0.0%    0.0%    0.0%    0.0%    0.0%    0.0%    0.0%    0.0%    0.0%    0.0%    0.0%    0.0%    0.0%    0.0%    0.0%    0.0%    0.0%    0.0%    0.0%    0.0%    0.0%    0.0%    0.0%    0.0%    0.0%    0.0%    0.0%    0.0%    0.0%    0.0%    0.0%    0.0%    0.0%    0.0%    0.0%    0.0%    0.0%    0.0%    0.0%    0.0%    0.0%    0.0%    0.0%    0.0%    0.0%    0.0%    0.0%    0.0%    0.0%    0.0%    0.0%    0.0%    0.0%    0.0%    0.0%    0.0%    0.0%    0.0%    0.0%    0.0%    0.0%    0.0%    0.0%    0.0%    0.0%    0.0%    0.0%    0.0%    0.0%    0.0%    0.0%    0.0%    0.0%    0.0%    0.0%    0.0%    0.0%    0.0%    0.0%    0.0%    0.0%    0.0%    0.0%    0.0%    0.0%    0.0%    0.0%    0.0%    0.0%    0.0%    0.0%    0.0%    0.0%    0.0%    0.0%    0.0%    0.0%    0.0%    0.0%    0.0%    0.0%    0.0%    0.0%    0.0%    0.0%    0.0%    0.0%    0.0%    0.0%    0.0%    0.0%    0.0%    0.0%    0.0%    0.0%    0.0%    0.0%    0.0%    0.0%    0.0%    0.0%    0.0%    0.0%    0.0%    0.0%    0.0%    0.0%    0.0%    0.0%    0.0%    0.0%    0.0%    0.0%    0.0%    0.0%    0.0%    0.0%    0.0%    0.0%    0.0%    0.0%    0.0%    0.0%    0.0%    0.0%    0.0%    0.0%    0.0%    0.0%    0.0%    0.0%    0.0%    0.0%    0.0%    0.0%    0.0%    0.0%    0.0%    0.0%    0.0%    0.0%    0.0%    0.0%    0.0%    0.0%    0.0%    0.0%    0.0%    0.0%    0.0%    0.0%    0.0%    0.0%    0.0%    0.0%    0.0%    0.0%    0.0%    0.0%    0.0%    0.0%    0.0%    0.0%    0.0%    0.0%    0.0%    0.0%    0.0%    0.0%    0.0%    0.0%    0.0%    0.0%    0.0%    0.0%    0.0%    0.0%    0.0%    0.0%    0.0%    0.0%    0.0%    0.0%    0.0%    0.0%    0.0%    0.0%    0.0%    0.0%    0.0%    0.0%    0.0%    0.0%    0.0%    0.0%    0.0%    0.0%    0.0%    0.0%    0.0%    0.0%    0.0%    0.0%    0.0%    0.0%    0.0%    0.0%        k__Bacteria;p__Chlorobi; c__ZB1       2    0.0%    0.0%    0.0%    0.0%    0.0%    0.0%    0.0%    0.0%    0.0%    0.0%    0.0%    0.0%    0.0%    0.0%    0.0%    0.0%    0.0%    0.0%    0.0%    0.0%    0.0%    0.0%    0.0%    0.0%    0.0%    0.0%    0.0%    0.0%    0.0%    0.0%    0.0%    0.0%    0.0%    0.0%    0.0%    0.0%    0.0%    0.0%    0.0%    0.0%    0.0%    0.0%    0.0%    0.0%    0.0%    0.0%    0.0%    0.0%    0.0%    0.0%    0.0%    0.0%    0.0%    0.0%    0.0%    0.0%    0.0%    0.0%    0.0%    0.0%    0.0%    0.0%    0.0%    0.0%    0.0%    0.0%    0.0%    0.0%    0.0%    0.0%    0.0%    0.0%    0.0%    0.0%    0.0%    0.0%    0.0%    0.0%    0.0%    0.0%    0.0%    0.0%    0.0%    0.0%    0.0%    0.0%    0.0%    0.0%    0.0%    0.0%    0.0%    0.0%    0.0%    0.0%    0.0%    0.0%    0.0%    0.0%    0.0%    0.0%    0.0%    0.0%    0.0%    0.0%    0.0%    0.0%    0.0%    0.0%    0.0%    0.0%    0.0%    0.0%    0.0%    0.0%    0.0%    0.0%    0.0%    0.0%    0.0%    0.0%    0.0%    0.0%    0.0%    0.0%    0.0%    0.0%    0.0%    0.0%    0.0%    0.0%    0.0%    0.0%    0.0%    0.0%    0.0%    0.0%    0.0%    0.0%    0.0%    0.0%    0.0%    0.0%    0.0%    0.0%    0.0%    0.0%    0.0%    0.0%    0.0%    0.0%    0.0%    0.0%    0.0%    0.0%    0.0%    0.0%    0.0%    0.0%    0.0%    0.0%    0.0%    0.0%    0.0%    0.0%    0.0%    0.0%    0.0%    0.0%    0.0%    0.0%    0.0%    0.0%    0.0%    0.0%    0.0%    0.0%    0.0%    0.0%    0.0%    0.0%    0.0%    0.0%    0.0%    0.0%    0.0%    0.0%    0.0%    0.0%    0.0%    0.0%    0.0%    0.0%    0.0%    0.0%    0.0%    0.0%    0.0%    0.0%    0.0%    0.0%    0.0%    0.0%    0.0%    0.0%    0.0%    0.0%    0.0%    0.0%    0.0%    0.0%    0.0%    0.0%    0.0%    0.0%    0.0%    0.0%    0.0%    0.0%    0.0%    0.0%    0.0%    0.0%    0.0%    0.0%    0.0%    0.0%    0.0%    0.0%    0.0%    0.0%    0.0%    0.0%    0.0%    0.0%    0.0%    0.0%    0.0%    0.0%    0.0%    0.0%    0.0%    0.0%    0.0%    0.0%    0.0%    0.0%    0.0%    0.0%    0.0%    0.0%    0.0%    0.0%    0.0%    0.0%    0.0%    0.0%    0.0%    0.0%    0.0%    0.0%    0.0%    0.0%    0.0%    0.0%    0.0%    0.0%    0.0%    0.0%    0.0%    0.0%    0.0%    0.0%    0.0%    0.0%    0.0%    0.0%    0.0%    0.0%    0.0%    0.0%    0.0%    0.0%    0.0%    0.0%    0.0%    0.0%    0.0%    0.0%    0.0%    0.0%    0.0%    0.0%    0.0%    0.0%    0.0%    0.0%    0.0%    0.0%    0.0%    0.0%    0.0%    0.0%    0.0%    0.0%    0.0%    0.0%    0.0%    0.0%    0.0%    0.0%    0.0%    0.0%    0.0%    0.0%    0.0%    0.0%    0.0%    0.0%    0.0%    0.0%    0.0%    0.0%    0.0%    0.0%    0.0%    0.0%    0.0%    0.0%    0.0%    0.0%    0.0%    0.0%    0.0%    0.0%    0.0%    0.0%    0.0%    0.0%    0.0%    0.0%    0.0%    0.0%    0.0%    0.0%    0.0%    0.0%    0.0%    0.0%    0.0%    0.0%    0.0%    0.0%    0.0%    0.0%    0.0%    0.0%    0.0%    0.0%        k__Bacteria;p__Chloroflexi; c__      12    0.0%    0.0%    0.0%    0.0%    0.0%    0.0%    0.0%    0.0%    0.0%    0.0%    0.0%    0.0%    0.0%    0.0%    0.0%    0.0%    0.0%    0.0%    0.0%    0.0%    0.0%    0.0%    0.0%    0.0%    0.0%    0.0%    0.0%    0.0%    0.0%    0.0%    0.0%    0.0%    0.0%    0.0%    0.0%    0.0%    0.0%    0.0%    0.0%    0.0%    0.0%    0.0%    0.0%    0.0%    0.0%    0.0%    0.0%    0.0%    0.0%    0.0%    0.0%    0.0%    0.0%    0.0%    0.0%    0.0%    0.0%    0.0%    0.0%    0.0%    0.0%    0.0%    0.0%    0.0%    0.0%    0.0%    0.0%    0.0%    0.0%    0.0%    0.0%    0.0%    0.0%    0.0%    0.0%    0.0%    0.0%    0.0%    0.0%    0.0%    0.0%    0.0%    0.0%    0.0%    0.0%    0.0%    0.0%    0.0%    0.0%    0.0%    0.0%    0.0%    0.0%    0.0%    0.0%    0.0%    0.0%    0.0%    0.0%    0.0%    0.0%    0.0%    0.0%    0.0%    0.0%    0.0%    0.0%    0.0%    0.0%    0.0%    0.0%    0.0%    0.0%    0.0%    0.0%    0.0%    0.0%    0.0%    0.0%    0.0%    0.0%    0.0%    0.0%    0.0%    0.0%    0.0%    0.0%    0.0%    0.0%    0.0%    0.0%    0.0%    0.0%    0.0%    0.0%    0.0%    0.0%    0.0%    0.0%    0.0%    0.0%    0.0%    0.0%    0.0%    0.0%    0.0%    0.0%    0.0%    0.0%    0.0%    0.0%    0.0%    0.0%    0.0%    0.0%    0.0%    0.0%    0.0%    0.0%    0.0%    0.0%    0.0%    0.0%    0.0%    0.0%    0.0%    0.0%    0.0%    0.0%    0.0%    0.0%    0.0%    0.0%    0.0%    0.0%    0.0%    0.0%    0.0%    0.0%    0.0%    0.0%    0.0%    0.0%    0.0%    0.0%    0.0%    0.0%    0.0%    0.0%    0.0%    0.0%    0.0%    0.0%    0.0%    0.0%    0.0%    0.0%    0.0%    0.0%    0.0%    0.0%    0.0%    0.0%    0.0%    0.0%    0.0%    0.0%    0.0%    0.0%    0.0%    0.0%    0.0%    0.0%    0.0%    0.0%    0.0%    0.0%    0.0%    0.0%    0.0%    0.0%    0.0%    0.0%    0.0%    0.0%    0.0%    0.0%    0.0%    0.0%    0.0%    0.0%    0.0%    0.0%    0.0%    0.0%    0.0%    0.0%    0.0%    0.0%    0.0%    0.0%    0.0%    0.0%    0.0%    0.0%    0.0%    0.0%    0.0%    0.0%    0.0%    0.0%    0.0%    0.0%    0.0%    0.0%    0.0%    0.0%    0.0%    0.0%    0.0%    0.0%    0.0%    0.0%    0.0%    0.0%    0.0%    0.0%    0.0%    0.0%    0.0%    0.0%    0.0%    0.0%    0.0%    0.0%    0.0%    0.0%    0.0%    0.0%    0.0%    0.0%    0.0%    0.0%    0.0%    0.0%    0.0%    0.0%    0.0%    0.0%    0.0%    0.0%    0.0%    0.0%    0.0%    0.0%    0.0%    0.0%    0.0%    0.0%    0.0%    0.0%    0.0%    0.0%    0.0%    0.0%    0.0%    0.0%    0.0%    0.0%    0.0%    0.0%    0.0%    0.0%    0.0%    0.0%    0.0%    0.0%    0.0%    0.0%    0.0%    0.0%    0.0%    0.0%    0.0%    0.0%    0.0%    0.0%    0.0%    0.0%    0.0%    0.0%    0.0%    0.0%    0.0%    0.0%    0.0%    0.0%    0.0%    0.0%    0.0%    0.0%    0.0%    0.0%    0.0%    0.0%    0.0%    0.0%    0.0%    0.0%    0.0%    0.0%    0.0%    0.0%    0.0%    0.0%    0.0%    0.0%    0.0%        k__Bacteria;p__Chloroflexi; c__Anaerolineae     611    0.0%    0.0%    0.0%    0.0%    0.0%    0.0%    0.0%    0.0%    0.0%    0.0%    0.0%    0.0%    0.0%    0.0%    0.0%    0.0%    0.0%    0.0%    0.0%    0.0%    0.0%    0.0%    0.0%    0.0%    0.0%    0.0%    0.0%    0.0%    0.0%    0.0%    0.0%    0.0%    0.0%    0.0%    0.0%    0.0%    0.0%    0.0%    0.0%    0.0%    0.0%    0.0%    0.0%    0.0%    0.0%    0.0%    0.0%    0.0%    0.0%    0.0%    0.0%    0.0%    0.0%    0.0%    0.0%    0.0%    0.0%    0.0%    0.0%    0.0%    0.1%    0.0%    0.0%    0.0%    0.0%    0.0%    0.0%    0.0%    0.0%    0.0%    0.0%    0.0%    0.0%    0.0%    0.0%    0.0%    0.0%    0.0%    0.0%    0.0%    0.0%    0.0%    0.0%    0.0%    0.0%    0.0%    0.0%    0.0%    0.0%    0.0%    0.0%    0.0%    0.0%    0.0%    0.0%    0.0%    0.0%    0.0%    0.0%    0.0%    0.0%    0.0%    0.0%    0.0%    0.0%    0.0%    0.0%    0.0%    0.0%    0.0%    0.0%    0.0%    0.0%    0.0%    0.0%    0.0%    0.0%    0.0%    0.0%    0.0%    0.0%    0.0%    0.0%    0.0%    0.0%    0.0%    0.0%    0.0%    0.0%    0.0%    0.0%    0.0%    0.0%    0.0%    0.0%    0.0%    0.0%    0.0%    0.0%    0.0%    0.0%    0.0%    0.0%    0.0%    0.0%    0.0%    0.0%    0.0%    0.0%    0.0%    0.0%    0.0%    0.0%    0.0%    0.0%    0.0%    0.0%    0.0%    0.0%    0.0%    0.0%    0.0%    0.0%    0.0%    0.0%    0.0%    0.0%    0.0%    0.0%    0.0%    0.0%    0.0%    0.0%    0.0%    0.0%    0.0%    0.0%    0.0%    0.0%    0.0%    0.0%    0.0%    0.0%    0.0%    0.0%    0.0%    0.0%    0.0%    0.0%    0.0%    0.0%    0.0%    0.0%    0.0%    0.0%    0.0%    0.0%    0.0%    0.0%    0.0%    0.0%    0.0%    0.0%    0.0%    0.0%    0.0%    0.0%    0.0%    0.0%    0.0%    0.0%    0.0%    0.0%    0.0%    0.0%    0.0%    0.0%    0.0%    0.0%    0.0%    0.0%    0.0%    0.0%    0.0%    0.0%    0.0%    0.0%    0.0%    0.0%    0.0%    0.0%    0.0%    0.0%    0.0%    0.0%    0.0%    0.0%    0.0%    0.0%    0.0%    0.0%    0.0%    0.0%    0.0%    0.0%    0.0%    0.0%    0.0%    0.0%    0.0%    0.0%    0.0%    0.0%    0.0%    0.0%    0.0%    0.0%    0.0%    0.0%    0.0%    0.0%    0.0%    0.0%    0.0%    0.0%    0.0%    0.0%    0.0%    0.0%    0.0%    0.0%    0.0%    0.0%    0.0%    0.0%    0.0%    0.0%    0.0%    0.0%    0.0%    0.0%    0.0%    0.0%    0.0%    0.0%    0.0%    0.0%    0.0%    0.0%    0.0%    0.0%    0.0%    0.0%    0.0%    0.0%    0.0%    0.0%    0.0%    0.0%    0.0%    0.0%    0.0%    0.0%    0.0%    0.0%    0.0%    0.0%    0.0%    0.0%    0.0%    0.0%    0.0%    0.0%    0.0%    0.0%    0.0%    0.0%    0.0%    0.0%    0.0%    0.0%    0.0%    0.0%    0.0%    0.0%    0.0%    0.0%    0.0%    0.0%    0.0%    0.0%    0.0%    0.0%    0.0%    0.0%    0.0%    0.0%    0.0%    0.0%    0.0%    0.0%    0.0%    0.0%    0.0%    0.0%    0.0%    0.0%    0.0%    0.0%    0.0%    0.0%    0.0%    0.0%    0.0%    0.0%    0.0%    0.0%    0.0%        k__Bacteria;p__Chloroflexi; c__Bljii12     781    0.0%    0.0%    0.0%    0.0%    0.0%    0.0%    0.0%    0.0%    0.0%    0.0%    0.0%    0.0%    0.0%    0.0%    0.0%    0.0%    0.0%    0.0%    0.0%    0.0%    0.0%    0.0%    0.0%    0.0%    0.0%    0.0%    0.0%    0.0%    0.0%    0.0%    0.0%    0.0%    0.0%    0.0%    0.0%    0.0%    0.0%    0.0%    0.0%    0.0%    0.0%    0.0%    0.0%    0.0%    0.0%    0.0%    0.0%    0.0%    0.0%    0.0%    0.0%    0.0%    0.0%    0.0%    0.0%    0.0%    0.0%    0.0%    0.0%    0.0%    0.0%    0.0%    0.0%    0.0%    0.0%    0.0%    0.0%    0.0%    0.0%    0.0%    0.0%    0.0%    0.0%    0.0%    0.0%    0.0%    0.0%    0.0%    0.0%    0.0%    0.0%    0.0%    0.0%    0.0%    0.0%    0.0%    0.0%    0.0%    0.0%    0.0%    0.0%    0.0%    0.0%    0.0%    0.0%    0.0%    0.0%    0.0%    0.0%    0.0%    0.0%    0.0%    0.0%    0.0%    0.0%    0.0%    0.0%    0.0%    0.0%    0.0%    0.0%    0.0%    0.0%    0.0%    0.0%    0.0%    0.0%    0.0%    0.0%    0.0%    0.0%    0.0%    0.0%    0.0%    0.0%    0.0%    0.0%    0.0%    0.0%    0.0%    0.0%    0.0%    0.0%    0.0%    0.0%    0.0%    0.0%    0.0%    0.0%    0.0%    0.0%    0.0%    0.0%    0.0%    0.0%    0.0%    0.0%    0.0%    0.0%    0.0%    0.0%    0.0%    0.0%    0.0%    0.0%    0.0%    0.0%    0.0%    0.0%    0.0%    0.0%    0.0%    0.0%    0.0%    0.0%    0.0%    0.0%    0.0%    0.0%    0.0%    0.0%    0.0%    0.0%    0.0%    0.0%    0.0%    0.0%    0.0%    0.0%    0.0%    0.0%    0.0%    0.0%    0.0%    0.0%    0.0%    0.0%    0.0%    0.0%    0.0%    0.0%    0.0%    0.0%    0.0%    0.0%    0.0%    0.0%    0.0%    0.0%    0.0%    0.0%    0.0%    0.0%    0.0%    0.0%    0.0%    0.0%    0.0%    0.0%    0.0%    0.0%    0.0%    0.0%    0.0%    0.0%    0.0%    0.0%    0.0%    0.0%    0.0%    0.0%    0.0%    0.0%    0.0%    0.0%    0.0%    0.0%    0.0%    0.0%    0.0%    0.0%    0.0%    0.0%    0.0%    0.0%    0.0%    0.0%    0.0%    0.0%    0.0%    0.0%    0.0%    0.0%    0.0%    0.0%    0.0%    0.0%    0.0%    0.0%    0.0%    0.0%    0.0%    0.0%    0.0%    0.0%    0.0%    0.0%    0.0%    0.0%    0.0%    0.0%    0.0%    0.0%    0.0%    0.0%    0.0%    0.0%    0.0%    0.0%    0.0%    0.0%    0.0%    0.0%    0.0%    0.0%    0.0%    0.0%    0.0%    0.0%    0.0%    0.0%    0.0%    0.0%    0.0%    0.0%    0.0%    0.0%    0.0%    0.0%    0.0%    0.0%    0.0%    0.0%    0.0%    0.0%    0.0%    0.0%    0.0%    0.0%    0.0%    0.0%    0.0%    0.0%    0.0%    0.0%    0.0%    0.0%    0.0%    0.0%    0.0%    0.0%    0.0%    0.0%    0.0%    0.0%    0.0%    0.0%    0.0%    0.0%    0.0%    0.0%    0.0%    0.0%    0.0%    0.0%    0.0%    0.0%    0.0%    0.0%    0.0%    0.0%    0.0%    0.0%    0.0%    0.0%    0.0%    0.0%    0.0%    0.0%    0.0%    0.0%    0.0%    0.0%    0.0%    0.0%    0.0%    0.0%    0.0%    0.0%    0.0%    0.0%    0.0%    0.0%    0.0%    0.0%    0.0%    0.0%    0.0%        k__Bacteria;p__Chloroflexi; c__Chloroflexi (class)      67    0.0%    0.0%    0.0%    0.0%    0.0%    0.0%    0.0%    0.0%    0.0%    0.0%    0.0%    0.0%    0.0%    0.0%    0.0%    0.0%    0.0%    0.0%    0.0%    0.0%    0.0%    0.0%    0.0%    0.0%    0.0%    0.0%    0.0%    0.0%    0.0%    0.0%    0.0%    0.0%    0.0%    0.0%    0.0%    0.0%    0.0%    0.0%    0.0%    0.0%    0.0%    0.0%    0.0%    0.0%    0.0%    0.0%    0.0%    0.0%    0.0%    0.0%    0.0%    0.0%    0.0%    0.0%    0.0%    0.0%    0.0%    0.0%    0.0%    0.0%    0.0%    0.0%    0.0%    0.0%    0.0%    0.0%    0.0%    0.0%    0.0%    0.0%    0.0%    0.0%    0.0%    0.0%    0.0%    0.0%    0.0%    0.0%    0.0%    0.0%    0.0%    0.0%    0.0%    0.0%    0.0%    0.0%    0.0%    0.0%    0.0%    0.0%    0.0%    0.0%    0.0%    0.0%    0.0%    0.0%    0.0%    0.0%    0.0%    0.0%    0.0%    0.0%    0.0%    0.0%    0.0%    0.0%    0.0%    0.0%    0.0%    0.0%    0.0%    0.0%    0.0%    0.0%    0.0%    0.0%    0.0%    0.0%    0.0%    0.0%    0.0%    0.0%    0.0%    0.0%    0.0%    0.0%    0.0%    0.0%    0.0%    0.0%    0.0%    0.0%    0.0%    0.0%    0.0%    0.0%    0.0%    0.0%    0.0%    0.0%    0.0%    0.0%    0.0%    0.0%    0.0%    0.0%    0.0%    0.0%    0.0%    0.0%    0.0%    0.0%    0.0%    0.0%    0.0%    0.0%    0.0%    0.0%    0.0%    0.0%    0.0%    0.0%    0.0%    0.0%    0.0%    0.0%    0.0%    0.0%    0.0%    0.0%    0.0%    0.0%    0.0%    0.0%    0.0%    0.0%    0.0%    0.0%    0.0%    0.0%    0.0%    0.0%    0.0%    0.0%    0.0%    0.0%    0.0%    0.0%    0.0%    0.0%    0.0%    0.0%    0.0%    0.0%    0.0%    0.0%    0.0%    0.0%    0.0%    0.0%    0.0%    0.0%    0.0%    0.0%    0.0%    0.0%    0.0%    0.0%    0.0%    0.0%    0.0%    0.0%    0.0%    0.0%    0.0%    0.0%    0.0%    0.0%    0.0%    0.0%    0.0%    0.0%    0.0%    0.0%    0.0%    0.0%    0.0%    0.0%    0.0%    0.0%    0.0%    0.0%    0.0%    0.0%    0.0%    0.0%    0.0%    0.0%    0.0%    0.0%    0.0%    0.0%    0.0%    0.0%    0.0%    0.0%    0.0%    0.0%    0.0%    0.0%    0.0%    0.0%    0.0%    0.0%    0.0%    0.0%    0.0%    0.0%    0.0%    0.0%    0.0%    0.0%    0.0%    0.0%    0.0%    0.0%    0.0%    0.0%    0.0%    0.0%    0.0%    0.0%    0.0%    0.0%    0.0%    0.0%    0.0%    0.0%    0.0%    0.0%    0.0%    0.0%    0.0%    0.0%    0.0%    0.0%    0.0%    0.0%    0.0%    0.0%    0.0%    0.0%    0.0%    0.0%    0.0%    0.0%    0.0%    0.0%    0.0%    0.0%    0.0%    0.0%    0.0%    0.0%    0.0%    0.0%    0.0%    0.0%    0.0%    0.0%    0.0%    0.0%    0.0%    0.0%    0.0%    0.0%    0.0%    0.0%    0.0%    0.0%    0.0%    0.0%    0.0%    0.0%    0.0%    0.0%    0.0%    0.0%    0.0%    0.0%    0.0%    0.0%    0.0%    0.0%    0.0%    0.0%    0.0%    0.0%    0.0%    0.0%    0.0%    0.0%    0.0%    0.0%    0.0%    0.0%    0.0%    0.0%    0.0%    0.0%    0.0%    0.0%    0.0%    0.0%    0.0%    0.0%    0.0%    0.0%        k__Bacteria;p__Chloroflexi; c__Chloroflexi-4       1    0.0%    0.0%    0.0%    0.0%    0.0%    0.0%    0.0%    0.0%    0.0%    0.0%    0.0%    0.0%    0.0%    0.0%    0.0%    0.0%    0.0%    0.0%    0.0%    0.0%    0.0%    0.0%    0.0%    0.0%    0.0%    0.0%    0.0%    0.0%    0.0%    0.0%    0.0%    0.0%    0.0%    0.0%    0.0%    0.0%    0.0%    0.0%    0.0%    0.0%    0.0%    0.0%    0.0%    0.0%    0.0%    0.0%    0.0%    0.0%    0.0%    0.0%    0.0%    0.0%    0.0%    0.0%    0.0%    0.0%    0.0%    0.0%    0.0%    0.0%    0.0%    0.0%    0.0%    0.0%    0.0%    0.0%    0.0%    0.0%    0.0%    0.0%    0.0%    0.0%    0.0%    0.0%    0.0%    0.0%    0.0%    0.0%    0.0%    0.0%    0.0%    0.0%    0.0%    0.0%    0.0%    0.0%    0.0%    0.0%    0.0%    0.0%    0.0%    0.0%    0.0%    0.0%    0.0%    0.0%    0.0%    0.0%    0.0%    0.0%    0.0%    0.0%    0.0%    0.0%    0.0%    0.0%    0.0%    0.0%    0.0%    0.0%    0.0%    0.0%    0.0%    0.0%    0.0%    0.0%    0.0%    0.0%    0.0%    0.0%    0.0%    0.0%    0.0%    0.0%    0.0%    0.0%    0.0%    0.0%    0.0%    0.0%    0.0%    0.0%    0.0%    0.0%    0.0%    0.0%    0.0%    0.0%    0.0%    0.0%    0.0%    0.0%    0.0%    0.0%    0.0%    0.0%    0.0%    0.0%    0.0%    0.0%    0.0%    0.0%    0.0%    0.0%    0.0%    0.0%    0.0%    0.0%    0.0%    0.0%    0.0%    0.0%    0.0%    0.0%    0.0%    0.0%    0.0%    0.0%    0.0%    0.0%    0.0%    0.0%    0.0%    0.0%    0.0%    0.0%    0.0%    0.0%    0.0%    0.0%    0.0%    0.0%    0.0%    0.0%    0.0%    0.0%    0.0%    0.0%    0.0%    0.0%    0.0%    0.0%    0.0%    0.0%    0.0%    0.0%    0.0%    0.0%    0.0%    0.0%    0.0%    0.0%    0.0%    0.0%    0.0%    0.0%    0.0%    0.0%    0.0%    0.0%    0.0%    0.0%    0.0%    0.0%    0.0%    0.0%    0.0%    0.0%    0.0%    0.0%    0.0%    0.0%    0.0%    0.0%    0.0%    0.0%    0.0%    0.0%    0.0%    0.0%    0.0%    0.0%    0.0%    0.0%    0.0%    0.0%    0.0%    0.0%    0.0%    0.0%    0.0%    0.0%    0.0%    0.0%    0.0%    0.0%    0.0%    0.0%    0.0%    0.0%    0.0%    0.0%    0.0%    0.0%    0.0%    0.0%    0.0%    0.0%    0.0%    0.0%    0.0%    0.0%    0.0%    0.0%    0.0%    0.0%    0.0%    0.0%    0.0%    0.0%    0.0%    0.0%    0.0%    0.0%    0.0%    0.0%    0.0%    0.0%    0.0%    0.0%    0.0%    0.0%    0.0%    0.0%    0.0%    0.0%    0.0%    0.0%    0.0%    0.0%    0.0%    0.0%    0.0%    0.0%    0.0%    0.0%    0.0%    0.0%    0.0%    0.0%    0.0%    0.0%    0.0%    0.0%    0.0%    0.0%    0.0%    0.0%    0.0%    0.0%    0.0%    0.0%    0.0%    0.0%    0.0%    0.0%    0.0%    0.0%    0.0%    0.0%    0.0%    0.0%    0.0%    0.0%    0.0%    0.0%    0.0%    0.0%    0.0%    0.0%    0.0%    0.0%    0.0%    0.0%    0.0%    0.0%    0.0%    0.0%    0.0%    0.0%    0.0%    0.0%    0.0%    0.0%    0.0%    0.0%    0.0%    0.0%    0.0%    0.0%    0.0%    0.0%    0.0%    0.0%    0.0%    0.0%    0.0%    0.0%        k__Bacteria;p__Chloroflexi; c__Ktedonobacteria       8    0.0%    0.0%    0.0%    0.0%    0.0%    0.0%    0.0%    0.0%    0.0%    0.0%    0.0%    0.0%    0.0%    0.0%    0.0%    0.0%    0.0%    0.0%    0.0%    0.0%    0.0%    0.0%    0.0%    0.0%    0.0%    0.0%    0.0%    0.0%    0.0%    0.0%    0.0%    0.0%    0.0%    0.0%    0.0%    0.0%    0.0%    0.0%    0.0%    0.0%    0.0%    0.0%    0.0%    0.0%    0.0%    0.0%    0.0%    0.0%    0.0%    0.0%    0.0%    0.0%    0.0%    0.0%    0.0%    0.0%    0.0%    0.0%    0.0%    0.0%    0.0%    0.0%    0.0%    0.0%    0.0%    0.0%    0.0%    0.0%    0.0%    0.0%    0.0%    0.0%    0.0%    0.0%    0.0%    0.0%    0.0%    0.0%    0.0%    0.0%    0.0%    0.0%    0.0%    0.0%    0.0%    0.0%    0.0%    0.0%    0.0%    0.0%    0.0%    0.0%    0.0%    0.0%    0.0%    0.0%    0.0%    0.0%    0.0%    0.0%    0.0%    0.0%    0.0%    0.0%    0.0%    0.0%    0.0%    0.0%    0.0%    0.0%    0.0%    0.0%    0.0%    0.0%    0.0%    0.0%    0.0%    0.0%    0.0%    0.0%    0.0%    0.0%    0.0%    0.0%    0.0%    0.0%    0.0%    0.0%    0.0%    0.0%    0.0%    0.0%    0.0%    0.0%    0.0%    0.0%    0.0%    0.0%    0.0%    0.0%    0.0%    0.0%    0.0%    0.0%    0.0%    0.0%    0.0%    0.0%    0.0%    0.0%    0.0%    0.0%    0.0%    0.0%    0.0%    0.0%    0.0%    0.0%    0.0%    0.0%    0.0%    0.0%    0.0%    0.0%    0.0%    0.0%    0.0%    0.0%    0.0%    0.0%    0.0%    0.0%    0.0%    0.0%    0.0%    0.0%    0.0%    0.0%    0.0%    0.0%    0.0%    0.0%    0.0%    0.0%    0.0%    0.0%    0.0%    0.0%    0.0%    0.0%    0.0%    0.0%    0.0%    0.0%    0.0%    0.0%    0.0%    0.0%    0.0%    0.0%    0.0%    0.0%    0.0%    0.0%    0.0%    0.0%    0.0%    0.0%    0.0%    0.0%    0.0%    0.0%    0.0%    0.0%    0.0%    0.0%    0.0%    0.0%    0.0%    0.0%    0.0%    0.0%    0.0%    0.0%    0.0%    0.0%    0.0%    0.0%    0.0%    0.0%    0.0%    0.0%    0.0%    0.0%    0.0%    0.0%    0.0%    0.0%    0.0%    0.0%    0.0%    0.0%    0.0%    0.0%    0.0%    0.0%    0.0%    0.0%    0.0%    0.0%    0.0%    0.0%    0.0%    0.0%    0.0%    0.0%    0.0%    0.0%    0.0%    0.0%    0.0%    0.0%    0.0%    0.0%    0.0%    0.0%    0.0%    0.0%    0.0%    0.0%    0.0%    0.0%    0.0%    0.0%    0.0%    0.0%    0.0%    0.0%    0.0%    0.0%    0.0%    0.0%    0.0%    0.0%    0.0%    0.0%    0.0%    0.0%    0.0%    0.0%    0.0%    0.0%    0.0%    0.0%    0.0%    0.0%    0.0%    0.0%    0.0%    0.0%    0.0%    0.0%    0.0%    0.0%    0.0%    0.0%    0.0%    0.0%    0.0%    0.0%    0.0%    0.0%    0.0%    0.0%    0.0%    0.0%    0.0%    0.0%    0.0%    0.0%    0.0%    0.0%    0.0%    0.0%    0.0%    0.0%    0.0%    0.0%    0.0%    0.0%    0.0%    0.0%    0.0%    0.0%    0.0%    0.0%    0.0%    0.0%    0.0%    0.0%    0.0%    0.0%    0.0%    0.0%    0.0%    0.0%    0.0%    0.0%    0.0%    0.0%    0.0%    0.0%    0.0%    0.0%    0.0%    0.0%    0.0%    0.0%        k__Bacteria;p__Chloroflexi; c__SOGA31    9477    0.1%    0.0%    0.1%    0.0%    0.1%    0.0%    0.0%    0.0%    0.0%    0.0%    0.0%    0.0%    0.0%    0.0%    0.0%    0.0%    0.0%    0.1%    0.0%    0.0%    0.0%    0.0%    0.0%    0.0%    0.0%    0.0%    0.0%    0.0%    0.1%    0.0%    0.0%    0.0%    0.0%    0.0%    0.1%    0.0%    0.0%    0.0%    0.0%    0.1%    0.0%    0.1%    0.0%    0.0%    0.0%    0.0%    0.0%    0.0%    0.0%    0.0%    0.0%    0.0%    0.0%    0.0%    0.1%    0.0%    0.1%    0.0%    0.0%    0.1%    0.1%    0.1%    0.0%    0.2%    0.1%    0.0%    0.0%    0.2%    0.1%    0.1%    0.0%    0.0%    0.1%    0.1%    0.1%    0.0%    0.0%    0.1%    0.0%    0.1%    0.0%    0.1%    0.0%    0.0%    0.1%    0.0%    0.2%    0.0%    0.0%    0.1%    0.0%    0.1%    0.1%    0.0%    0.0%    0.1%    0.2%    0.0%    0.1%    0.1%    0.1%    0.1%    0.0%    0.1%    0.0%    0.0%    0.2%    0.0%    0.0%    0.1%    0.1%    0.2%    0.0%    0.0%    0.1%    0.0%    0.0%    0.0%    0.0%    0.5%    0.0%    0.1%    0.2%    0.1%    0.0%    0.3%    0.1%    0.0%    0.1%    0.4%    0.1%    0.1%    0.1%    0.0%    0.3%    0.0%    0.1%    0.1%    0.0%    0.0%    0.0%    0.1%    0.1%    0.0%    0.2%    0.1%    0.0%    0.0%    0.1%    0.0%    0.0%    0.0%    0.2%    0.0%    0.0%    0.0%    0.2%    0.0%    0.0%    0.2%    0.4%    0.2%    0.2%    0.1%    0.0%    0.0%    0.1%    0.0%    0.0%    0.0%    0.1%    0.2%    0.1%    0.0%    0.0%    0.0%    0.0%    0.0%    0.0%    0.0%    0.0%    0.0%    0.0%    0.1%    0.1%    0.0%    0.0%    0.0%    0.0%    0.0%    0.0%    0.0%    0.0%    0.0%    0.0%    0.0%    0.2%    0.0%    0.0%    0.0%    0.0%    0.1%    0.0%    0.2%    0.1%    0.0%    0.1%    0.1%    0.3%    0.1%    0.1%    0.0%    0.1%    0.0%    0.0%    0.1%    0.0%    0.1%    0.0%    0.0%    0.1%    0.1%    0.0%    0.1%    0.0%    0.0%    0.0%    0.0%    0.1%    0.0%    0.0%    0.0%    0.2%    0.0%    0.3%    0.0%    0.2%    0.0%    0.0%    0.0%    0.0%    0.0%    0.0%    0.0%    0.0%    0.0%    0.0%    0.0%    0.0%    0.0%    0.2%    0.1%    0.0%    0.0%    0.0%    0.0%    0.0%    0.0%    0.0%    0.0%    0.1%    0.1%    0.1%    0.1%    0.0%    0.0%    0.1%    0.1%    0.0%    0.1%    0.0%    0.1%    0.2%    0.1%    0.0%    0.0%    0.0%    0.0%    0.0%    0.0%    0.0%    0.0%    0.0%    0.0%    0.0%    0.0%    0.0%    0.7%    0.0%    0.0%    0.1%    0.0%    0.1%    0.1%    0.1%    0.0%    0.0%    0.1%    0.0%    0.1%    0.0%    0.0%    0.0%    0.0%    0.0%    0.0%    0.0%    0.0%    0.0%    0.0%    0.0%    0.0%    0.0%    0.0%    0.1%    0.2%    0.0%    0.0%    0.0%    0.0%    0.0%    0.0%    0.0%    0.0%    0.0%    0.0%    0.0%    0.0%    0.0%    0.0%    0.1%    0.0%    0.0%    0.0%    0.0%    0.0%    0.0%    0.0%    0.0%    0.0%    0.0%    0.0%    0.0%    0.0%    0.2%    0.0%    0.0%    0.0%    0.0%    0.0%    0.0%    0.1%    0.0%    0.1%    0.0%    0.0%    0.5%    0.0%        k__Bacteria;p__Chloroflexi; c__TK17     369    0.0%    0.0%    0.0%    0.0%    0.0%    0.0%    0.0%    0.0%    0.0%    0.0%    0.0%    0.0%    0.0%    0.0%    0.0%    0.0%    0.0%    0.0%    0.0%    0.0%    0.0%    0.0%    0.0%    0.0%    0.0%    0.0%    0.0%    0.0%    0.0%    0.0%    0.0%    0.0%    0.0%    0.0%    0.0%    0.0%    0.0%    0.0%    0.0%    0.0%    0.0%    0.0%    0.0%    0.0%    0.0%    0.0%    0.0%    0.0%    0.0%    0.0%    0.0%    0.0%    0.0%    0.0%    0.0%    0.0%    0.0%    0.0%    0.0%    0.0%    0.0%    0.0%    0.0%    0.0%    0.0%    0.0%    0.0%    0.0%    0.0%    0.0%    0.0%    0.0%    0.0%    0.0%    0.0%    0.0%    0.0%    0.0%    0.0%    0.0%    0.0%    0.0%    0.0%    0.0%    0.0%    0.0%    0.0%    0.0%    0.0%    0.0%    0.0%    0.0%    0.0%    0.0%    0.0%    0.0%    0.0%    0.0%    0.0%    0.0%    0.0%    0.0%    0.0%    0.0%    0.0%    0.0%    0.0%    0.0%    0.0%    0.0%    0.0%    0.0%    0.0%    0.0%    0.0%    0.0%    0.0%    0.0%    0.0%    0.0%    0.0%    0.0%    0.0%    0.0%    0.0%    0.0%    0.0%    0.0%    0.0%    0.0%    0.0%    0.0%    0.0%    0.0%    0.0%    0.0%    0.0%    0.0%    0.0%    0.0%    0.0%    0.0%    0.0%    0.0%    0.0%    0.0%    0.0%    0.0%    0.0%    0.0%    0.0%    0.0%    0.0%    0.0%    0.0%    0.0%    0.0%    0.0%    0.0%    0.0%    0.0%    0.0%    0.0%    0.0%    0.0%    0.0%    0.0%    0.0%    0.0%    0.0%    0.0%    0.0%    0.0%    0.0%    0.0%    0.0%    0.0%    0.0%    0.0%    0.0%    0.0%    0.0%    0.0%    0.0%    0.0%    0.0%    0.0%    0.0%    0.0%    0.0%    0.0%    0.0%    0.0%    0.0%    0.0%    0.0%    0.0%    0.0%    0.0%    0.0%    0.0%    0.0%    0.0%    0.0%    0.0%    0.0%    0.0%    0.0%    0.0%    0.0%    0.0%    0.0%    0.0%    0.0%    0.0%    0.0%    0.0%    0.0%    0.0%    0.0%    0.0%    0.0%    0.0%    0.0%    0.0%    0.0%    0.0%    0.0%    0.0%    0.0%    0.0%    0.0%    0.0%    0.0%    0.0%    0.0%    0.0%    0.0%    0.0%    0.0%    0.0%    0.0%    0.0%    0.0%    0.0%    0.0%    0.0%    0.0%    0.0%    0.0%    0.0%    0.0%    0.0%    0.0%    0.0%    0.0%    0.0%    0.0%    0.0%    0.0%    0.0%    0.0%    0.0%    0.0%    0.0%    0.0%    0.0%    0.0%    0.0%    0.0%    0.0%    0.0%    0.0%    0.0%    0.0%    0.0%    0.0%    0.0%    0.0%    0.0%    0.0%    0.0%    0.0%    0.0%    0.0%    0.0%    0.0%    0.0%    0.0%    0.0%    0.0%    0.0%    0.0%    0.0%    0.0%    0.0%    0.0%    0.0%    0.0%    0.0%    0.0%    0.0%    0.0%    0.0%    0.0%    0.0%    0.0%    0.0%    0.0%    0.0%    0.0%    0.0%    0.0%    0.0%    0.0%    0.0%    0.0%    0.0%    0.0%    0.0%    0.0%    0.0%    0.0%    0.0%    0.0%    0.0%    0.0%    0.0%    0.0%    0.0%    0.0%    0.0%    0.0%    0.0%    0.0%    0.0%    0.0%    0.0%    0.0%    0.0%    0.0%    0.0%    0.0%    0.0%    0.0%    0.0%    0.0%    0.0%    0.0%    0.0%    0.0%    0.0%    0.0%    0.0%    0.0%    0.0%    0.0%    0.0%        k__Bacteria;p__Chloroflexi; c__Thermobacula       0    0.0%    0.0%    0.0%    0.0%    0.0%    0.0%    0.0%    0.0%    0.0%    0.0%    0.0%    0.0%    0.0%    0.0%    0.0%    0.0%    0.0%    0.0%    0.0%    0.0%    0.0%    0.0%    0.0%    0.0%    0.0%    0.0%    0.0%    0.0%    0.0%    0.0%    0.0%    0.0%    0.0%    0.0%    0.0%    0.0%    0.0%    0.0%    0.0%    0.0%    0.0%    0.0%    0.0%    0.0%    0.0%    0.0%    0.0%    0.0%    0.0%    0.0%    0.0%    0.0%    0.0%    0.0%    0.0%    0.0%    0.0%    0.0%    0.0%    0.0%    0.0%    0.0%    0.0%    0.0%    0.0%    0.0%    0.0%    0.0%    0.0%    0.0%    0.0%    0.0%    0.0%    0.0%    0.0%    0.0%    0.0%    0.0%    0.0%    0.0%    0.0%    0.0%    0.0%    0.0%    0.0%    0.0%    0.0%    0.0%    0.0%    0.0%    0.0%    0.0%    0.0%    0.0%    0.0%    0.0%    0.0%    0.0%    0.0%    0.0%    0.0%    0.0%    0.0%    0.0%    0.0%    0.0%    0.0%    0.0%    0.0%    0.0%    0.0%    0.0%    0.0%    0.0%    0.0%    0.0%    0.0%    0.0%    0.0%    0.0%    0.0%    0.0%    0.0%    0.0%    0.0%    0.0%    0.0%    0.0%    0.0%    0.0%    0.0%    0.0%    0.0%    0.0%    0.0%    0.0%    0.0%    0.0%    0.0%    0.0%    0.0%    0.0%    0.0%    0.0%    0.0%    0.0%    0.0%    0.0%    0.0%    0.0%    0.0%    0.0%    0.0%    0.0%    0.0%    0.0%    0.0%    0.0%    0.0%    0.0%    0.0%    0.0%    0.0%    0.0%    0.0%    0.0%    0.0%    0.0%    0.0%    0.0%    0.0%    0.0%    0.0%    0.0%    0.0%    0.0%    0.0%    0.0%    0.0%    0.0%    0.0%    0.0%    0.0%    0.0%    0.0%    0.0%    0.0%    0.0%    0.0%    0.0%    0.0%    0.0%    0.0%    0.0%    0.0%    0.0%    0.0%    0.0%    0.0%    0.0%    0.0%    0.0%    0.0%    0.0%    0.0%    0.0%    0.0%    0.0%    0.0%    0.0%    0.0%    0.0%    0.0%    0.0%    0.0%    0.0%    0.0%    0.0%    0.0%    0.0%    0.0%    0.0%    0.0%    0.0%    0.0%    0.0%    0.0%    0.0%    0.0%    0.0%    0.0%    0.0%    0.0%    0.0%    0.0%    0.0%    0.0%    0.0%    0.0%    0.0%    0.0%    0.0%    0.0%    0.0%    0.0%    0.0%    0.0%    0.0%    0.0%    0.0%    0.0%    0.0%    0.0%    0.0%    0.0%    0.0%    0.0%    0.0%    0.0%    0.0%    0.0%    0.0%    0.0%    0.0%    0.0%    0.0%    0.0%    0.0%    0.0%    0.0%    0.0%    0.0%    0.0%    0.0%    0.0%    0.0%    0.0%    0.0%    0.0%    0.0%    0.0%    0.0%    0.0%    0.0%    0.0%    0.0%    0.0%    0.0%    0.0%    0.0%    0.0%    0.0%    0.0%    0.0%    0.0%    0.0%    0.0%    0.0%    0.0%    0.0%    0.0%    0.0%    0.0%    0.0%    0.0%    0.0%    0.0%    0.0%    0.0%    0.0%    0.0%    0.0%    0.0%    0.0%    0.0%    0.0%    0.0%    0.0%    0.0%    0.0%    0.0%    0.0%    0.0%    0.0%    0.0%    0.0%    0.0%    0.0%    0.0%    0.0%    0.0%    0.0%    0.0%    0.0%    0.0%    0.0%    0.0%    0.0%    0.0%    0.0%    0.0%    0.0%    0.0%    0.0%    0.0%    0.0%    0.0%    0.0%    0.0%    0.0%    0.0%    0.0%    0.0%    0.0%    0.0%    0.0%    0.0%    0.0%        k__Bacteria;p__Chloroflexi; c__Thermomicrobia    8103    0.0%    0.0%    0.1%    0.0%    0.0%    0.0%    0.0%    0.0%    0.0%    0.0%    0.0%    0.0%    0.0%    0.0%    0.0%    0.0%    0.0%    0.1%    0.0%    0.0%    0.1%    0.1%    0.0%    0.0%    0.0%    0.0%    0.0%    0.0%    0.1%    0.0%    0.0%    0.1%    0.0%    0.0%    0.1%    0.0%    0.0%    0.0%    0.1%    0.2%    0.0%    0.1%    0.0%    0.0%    0.1%    0.0%    0.0%    0.0%    0.0%    0.0%    0.0%    0.0%    0.0%    0.0%    0.1%    0.0%    0.0%    0.0%    0.0%    0.1%    0.1%    0.0%    0.0%    0.1%    0.1%    0.1%    0.0%    0.2%    0.1%    0.1%    0.0%    0.0%    0.0%    0.0%    0.2%    0.0%    0.0%    0.1%    0.0%    0.0%    0.0%    0.1%    0.0%    0.0%    0.0%    0.0%    0.1%    0.0%    0.0%    0.1%    0.0%    0.1%    0.1%    0.0%    0.0%    0.2%    0.1%    0.0%    0.0%    0.0%    0.1%    0.0%    0.0%    0.1%    0.0%    0.0%    0.1%    0.0%    0.0%    0.1%    0.1%    0.2%    0.0%    0.0%    0.1%    0.0%    0.0%    0.0%    0.1%    0.3%    0.0%    0.1%    0.2%    0.1%    0.0%    0.1%    0.0%    0.0%    0.1%    0.2%    0.1%    0.1%    0.1%    0.1%    0.4%    0.0%    0.1%    0.1%    0.0%    0.0%    0.0%    0.1%    0.1%    0.0%    0.2%    0.0%    0.0%    0.0%    0.0%    0.0%    0.0%    0.0%    0.2%    0.1%    0.0%    0.0%    0.1%    0.0%    0.0%    0.1%    0.2%    0.2%    0.2%    0.0%    0.0%    0.0%    0.1%    0.1%    0.1%    0.1%    0.2%    0.1%    0.1%    0.0%    0.0%    0.0%    0.0%    0.0%    0.0%    0.1%    0.0%    0.0%    0.0%    0.0%    0.0%    0.0%    0.0%    0.0%    0.0%    0.0%    0.0%    0.0%    0.0%    0.0%    0.0%    0.1%    0.1%    0.0%    0.0%    0.0%    0.0%    0.0%    0.0%    0.1%    0.2%    0.0%    0.0%    0.0%    0.2%    0.1%    0.1%    0.0%    0.0%    0.0%    0.0%    0.0%    0.0%    0.0%    0.0%    0.0%    0.1%    0.1%    0.0%    0.1%    0.0%    0.0%    0.0%    0.0%    0.1%    0.1%    0.0%    0.0%    0.1%    0.0%    0.1%    0.0%    0.1%    0.0%    0.0%    0.0%    0.0%    0.0%    0.0%    0.0%    0.0%    0.0%    0.0%    0.0%    0.0%    0.0%    0.2%    0.0%    0.0%    0.0%    0.0%    0.0%    0.0%    0.0%    0.0%    0.0%    0.1%    0.1%    0.1%    0.0%    0.0%    0.0%    0.1%    0.0%    0.0%    0.0%    0.0%    0.1%    0.1%    0.1%    0.0%    0.0%    0.0%    0.0%    0.0%    0.0%    0.0%    0.0%    0.0%    0.0%    0.1%    0.0%    0.1%    0.8%    0.1%    0.0%    0.0%    0.0%    0.1%    0.0%    0.0%    0.0%    0.1%    0.1%    0.1%    0.2%    0.1%    0.0%    0.0%    0.1%    0.1%    0.0%    0.0%    0.0%    0.1%    0.0%    0.0%    0.0%    0.0%    0.0%    0.0%    0.1%    0.0%    0.0%    0.0%    0.1%    0.0%    0.0%    0.0%    0.0%    0.0%    0.0%    0.0%    0.0%    0.0%    0.1%    0.0%    0.0%    0.0%    0.1%    0.0%    0.0%    0.0%    0.0%    0.0%    0.0%    0.0%    0.0%    0.0%    0.0%    0.2%    0.0%    0.0%    0.0%    0.0%    0.0%    0.0%    0.0%    0.0%    0.1%    0.0%    0.0%    0.0%    0.0%        k__Bacteria;p__Cyanobacteria; c__   2262225   13.3%    0.1%    9.0%    0.9%    1.6%   44.5%    0.3%    0.1%    0.1%    0.0%    0.1%    0.1%   10.1%    6.0%    0.5%    2.1%    0.5%   25.9%   51.0%   19.8%   16.4%    8.1%    3.8%    0.2%    0.5%    1.3%   46.2%    1.7%    1.1%    0.4%    0.1%    8.9%   65.8%   25.7%    3.0%    0.9%    1.1%    3.6%    9.7%   32.8%   26.2%    7.5%    0.3%    0.4%    0.3%    0.6%   17.9%    6.6%    1.3%    0.7%    8.7%   26.9%    5.2%    8.4%   40.6%    1.5%   33.2%    7.3%    3.1%   15.9%   48.3%   15.9%    3.0%    7.7%   17.7%   16.5%   41.6%    3.0%   43.3%   24.8%   13.2%    3.2%   79.3%   21.6%    7.4%   87.5%   46.6%   15.9%    0.6%    1.4%    0.8%    4.3%    9.2%    1.9%    3.3%    0.1%    3.6%   58.6%   20.7%    1.9%   37.6%    6.7%    2.9%   45.5%    1.3%   19.5%   45.4%   65.1%    4.5%    1.8%   20.9%    2.4%    2.3%    3.9%    0.7%    0.9%    1.4%   52.8%   38.7%    1.8%    0.8%    2.3%   14.5%   80.9%    6.4%    2.3%    1.0%    1.1%    1.1%    5.1%    5.9%    2.7%    3.4%   13.4%    1.8%    2.7%    6.6%   44.5%   16.0%    5.8%   13.2%    7.7%   48.7%    4.0%    2.5%   19.9%    0.6%    1.3%    0.4%   36.3%    0.6%    3.1%   26.6%   17.2%   40.5%    3.1%    0.7%    0.4%   19.1%   44.1%   44.7%   50.5%   14.2%    5.6%   13.8%   36.3%    8.4%   51.8%   10.1%    4.5%   41.3%    4.6%    4.2%   44.5%    2.2%   38.9%    5.5%    5.8%    6.4%   12.2%    4.3%    5.8%   55.9%    7.2%    4.6%    0.9%    7.6%   71.3%   55.8%    4.2%    3.4%    6.0%    4.6%   64.9%    6.8%    8.4%    0.9%    2.8%    1.3%    2.0%    0.4%    4.0%    2.4%    2.3%   10.9%    9.7%    7.5%    6.0%   12.0%   20.8%    4.1%    3.7%   43.5%   10.3%    4.9%   18.5%    1.6%   21.8%   14.4%    4.5%   15.4%    1.8%   17.2%   11.6%   40.0%    4.6%    7.7%   44.2%    2.4%    3.6%    2.7%    2.7%   18.9%   14.9%   11.4%   10.0%    1.2%    1.1%   32.1%    4.0%    1.4%   31.3%    7.5%   18.1%   20.9%   39.5%   18.7%    2.0%    0.3%    1.7%    1.0%    0.9%    0.1%    6.8%    0.5%    0.7%   29.7%    0.7%    0.4%    2.1%    2.6%    8.3%   18.0%   41.5%   57.1%   36.8%   18.1%    1.6%   32.9%    1.2%    2.5%    1.6%   13.6%    5.1%    0.9%   56.6%   14.5%    0.4%    3.0%    0.6%    0.9%    9.3%    6.3%    4.3%    1.0%    4.0%   24.1%    1.2%    1.5%    0.5%   36.2%    3.2%   12.8%    3.7%    2.5%    0.1%    0.2%    1.2%    6.0%    2.1%    1.3%    1.2%    0.4%    0.6%    2.2%   10.2%    7.2%    1.1%    2.6%    2.0%    0.3%   10.4%    0.1%    2.2%    0.8%    4.1%    0.9%    4.2%   39.6%    0.3%    0.1%    0.8%    0.1%    0.8%    1.8%    0.7%   78.4%    2.0%    0.0%    0.2%    0.3%    0.2%    0.3%   60.3%   11.7%    1.5%    4.8%    9.7%    1.5%    2.7%    1.2%    0.2%    0.8%    6.6%    0.4%    0.8%    0.1%    1.2%    0.9%    1.0%    2.6%    1.2%    1.4%    2.0%    1.6%    0.1%    1.8%    0.3%    6.4%    8.8%   25.5%    1.2%    9.8%    3.7%   20.0%    8.6%    3.2%    0.4%        k__Bacteria;p__Cyanobacteria; c__S15B-MN24     250    0.0%    0.0%    0.0%    0.0%    0.0%    0.0%    0.0%    0.0%    0.0%    0.0%    0.0%    0.0%    0.0%    0.0%    0.0%    0.0%    0.0%    0.0%    0.0%    0.0%    0.0%    0.0%    0.0%    0.0%    0.0%    0.0%    0.0%    0.0%    0.0%    0.0%    0.0%    0.0%    0.0%    0.0%    0.0%    0.0%    0.0%    0.0%    0.0%    0.0%    0.0%    0.0%    0.0%    0.0%    0.0%    0.0%    0.0%    0.0%    0.0%    0.0%    0.0%    0.0%    0.0%    0.0%    0.0%    0.0%    0.0%    0.0%    0.0%    0.0%    0.0%    0.0%    0.0%    0.0%    0.0%    0.0%    0.0%    0.0%    0.0%    0.0%    0.0%    0.0%    0.0%    0.0%    0.0%    0.0%    0.0%    0.0%    0.0%    0.0%    0.0%    0.1%    0.0%    0.0%    0.0%    0.0%    0.0%    0.0%    0.0%    0.0%    0.0%    0.0%    0.0%    0.0%    0.0%    0.0%    0.0%    0.0%    0.0%    0.0%    0.0%    0.0%    0.0%    0.0%    0.0%    0.0%    0.0%    0.0%    0.0%    0.0%    0.0%    0.0%    0.0%    0.0%    0.0%    0.0%    0.0%    0.0%    0.0%    0.0%    0.0%    0.0%    0.0%    0.0%    0.0%    0.0%    0.0%    0.0%    0.0%    0.0%    0.0%    0.0%    0.0%    0.0%    0.0%    0.0%    0.0%    0.0%    0.0%    0.0%    0.0%    0.0%    0.0%    0.0%    0.0%    0.0%    0.0%    0.0%    0.0%    0.0%    0.0%    0.0%    0.0%    0.0%    0.0%    0.0%    0.0%    0.0%    0.0%    0.0%    0.0%    0.0%    0.0%    0.0%    0.0%    0.0%    0.0%    0.0%    0.0%    0.0%    0.0%    0.0%    0.0%    0.0%    0.0%    0.0%    0.0%    0.0%    0.0%    0.0%    0.0%    0.0%    0.0%    0.0%    0.0%    0.0%    0.0%    0.0%    0.0%    0.0%    0.0%    0.0%    0.0%    0.0%    0.0%    0.0%    0.0%    0.0%    0.0%    0.0%    0.0%    0.0%    0.0%    0.0%    0.0%    0.0%    0.0%    0.0%    0.0%    0.0%    0.0%    0.0%    0.0%    0.0%    0.0%    0.0%    0.0%    0.0%    0.0%    0.0%    0.0%    0.0%    0.0%    0.0%    0.0%    0.0%    0.0%    0.0%    0.0%    0.0%    0.0%    0.0%    0.0%    0.0%    0.0%    0.0%    0.0%    0.0%    0.0%    0.0%    0.0%    0.0%    0.0%    0.0%    0.0%    0.0%    0.0%    0.0%    0.0%    0.0%    0.0%    0.0%    0.0%    0.0%    0.0%    0.0%    0.0%    0.0%    0.0%    0.0%    0.0%    0.0%    0.0%    0.0%    0.0%    0.0%    0.0%    0.0%    0.0%    0.0%    0.0%    0.0%    0.0%    0.0%    0.0%    0.0%    0.0%    0.0%    0.0%    0.0%    0.0%    0.0%    0.0%    0.0%    0.0%    0.0%    0.0%    0.0%    0.0%    0.0%    0.0%    0.0%    0.0%    0.0%    0.0%    0.0%    0.0%    0.0%    0.0%    0.0%    0.0%    0.0%    0.0%    0.0%    0.0%    0.0%    0.0%    0.0%    0.0%    0.0%    0.0%    0.0%    0.0%    0.0%    0.0%    0.0%    0.0%    0.0%    0.0%    0.0%    0.0%    0.0%    0.0%    0.0%    0.0%    0.0%    0.0%    0.0%    0.0%    0.0%    0.0%    0.0%    0.0%    0.0%    0.0%    0.0%    0.0%    0.0%    0.0%    0.0%    0.0%    0.0%    0.0%    0.0%    0.0%    0.0%    0.0%    0.0%    0.0%    0.0%    0.0%    0.0%    0.0%    0.0%    0.0%    0.0%    0.0%    0.0%        k__Bacteria;p__Cyanobacteria; c__SM1D11       6    0.0%    0.0%    0.0%    0.0%    0.0%    0.0%    0.0%    0.0%    0.0%    0.0%    0.0%    0.0%    0.0%    0.0%    0.0%    0.0%    0.0%    0.0%    0.0%    0.0%    0.0%    0.0%    0.0%    0.0%    0.0%    0.0%    0.0%    0.0%    0.0%    0.0%    0.0%    0.0%    0.0%    0.0%    0.0%    0.0%    0.0%    0.0%    0.0%    0.0%    0.0%    0.0%    0.0%    0.0%    0.0%    0.0%    0.0%    0.0%    0.0%    0.0%    0.0%    0.0%    0.0%    0.0%    0.0%    0.0%    0.0%    0.0%    0.0%    0.0%    0.0%    0.0%    0.0%    0.0%    0.0%    0.0%    0.0%    0.0%    0.0%    0.0%    0.0%    0.0%    0.0%    0.0%    0.0%    0.0%    0.0%    0.0%    0.0%    0.0%    0.0%    0.0%    0.0%    0.0%    0.0%    0.0%    0.0%    0.0%    0.0%    0.0%    0.0%    0.0%    0.0%    0.0%    0.0%    0.0%    0.0%    0.0%    0.0%    0.0%    0.0%    0.0%    0.0%    0.0%    0.0%    0.0%    0.0%    0.0%    0.0%    0.0%    0.0%    0.0%    0.0%    0.0%    0.0%    0.0%    0.0%    0.0%    0.0%    0.0%    0.0%    0.0%    0.0%    0.0%    0.0%    0.0%    0.0%    0.0%    0.0%    0.0%    0.0%    0.0%    0.0%    0.0%    0.0%    0.0%    0.0%    0.0%    0.0%    0.0%    0.0%    0.0%    0.0%    0.0%    0.0%    0.0%    0.0%    0.0%    0.0%    0.0%    0.0%    0.0%    0.0%    0.0%    0.0%    0.0%    0.0%    0.0%    0.0%    0.0%    0.0%    0.0%    0.0%    0.0%    0.0%    0.0%    0.0%    0.0%    0.0%    0.0%    0.0%    0.0%    0.0%    0.0%    0.0%    0.0%    0.0%    0.0%    0.0%    0.0%    0.0%    0.0%    0.0%    0.0%    0.0%    0.0%    0.0%    0.0%    0.0%    0.0%    0.0%    0.0%    0.0%    0.0%    0.0%    0.0%    0.0%    0.0%    0.0%    0.0%    0.0%    0.0%    0.0%    0.0%    0.0%    0.0%    0.0%    0.0%    0.0%    0.0%    0.0%    0.0%    0.0%    0.0%    0.0%    0.0%    0.0%    0.0%    0.0%    0.0%    0.0%    0.0%    0.0%    0.0%    0.0%    0.0%    0.0%    0.0%    0.0%    0.0%    0.0%    0.0%    0.0%    0.0%    0.0%    0.0%    0.0%    0.0%    0.0%    0.0%    0.0%    0.0%    0.0%    0.0%    0.0%    0.0%    0.0%    0.0%    0.0%    0.0%    0.0%    0.0%    0.0%    0.0%    0.0%    0.0%    0.0%    0.0%    0.0%    0.0%    0.0%    0.0%    0.0%    0.0%    0.0%    0.0%    0.0%    0.0%    0.0%    0.0%    0.0%    0.0%    0.0%    0.0%    0.0%    0.0%    0.0%    0.0%    0.0%    0.0%    0.0%    0.0%    0.0%    0.0%    0.0%    0.0%    0.0%    0.0%    0.0%    0.0%    0.0%    0.0%    0.0%    0.0%    0.0%    0.0%    0.0%    0.0%    0.0%    0.0%    0.0%    0.0%    0.0%    0.0%    0.0%    0.0%    0.0%    0.0%    0.0%    0.0%    0.0%    0.0%    0.0%    0.0%    0.0%    0.0%    0.0%    0.0%    0.0%    0.0%    0.0%    0.0%    0.0%    0.0%    0.0%    0.0%    0.0%    0.0%    0.0%    0.0%    0.0%    0.0%    0.0%    0.0%    0.0%    0.0%    0.0%    0.0%    0.0%    0.0%    0.0%    0.0%    0.0%    0.0%    0.0%    0.0%    0.0%    0.0%    0.0%    0.0%    0.0%    0.0%    0.0%    0.0%    0.0%    0.0%    0.0%    0.0%        k__Bacteria;p__Cyanobacteria; c__YS2      68    0.0%    0.0%    0.0%    0.0%    0.0%    0.0%    0.0%    0.0%    0.0%    0.0%    0.0%    0.0%    0.0%    0.0%    0.0%    0.0%    0.0%    0.0%    0.0%    0.0%    0.0%    0.0%    0.0%    0.0%    0.0%    0.0%    0.0%    0.0%    0.0%    0.0%    0.0%    0.0%    0.0%    0.0%    0.0%    0.0%    0.0%    0.0%    0.0%    0.0%    0.0%    0.0%    0.0%    0.0%    0.0%    0.0%    0.0%    0.0%    0.0%    0.0%    0.0%    0.0%    0.0%    0.0%    0.0%    0.0%    0.0%    0.0%    0.0%    0.0%    0.0%    0.0%    0.0%    0.0%    0.0%    0.0%    0.0%    0.0%    0.0%    0.0%    0.0%    0.0%    0.0%    0.0%    0.0%    0.0%    0.0%    0.0%    0.0%    0.0%    0.0%    0.0%    0.0%    0.0%    0.0%    0.0%    0.0%    0.0%    0.0%    0.0%    0.0%    0.0%    0.0%    0.0%    0.0%    0.0%    0.0%    0.0%    0.0%    0.0%    0.0%    0.0%    0.0%    0.0%    0.0%    0.0%    0.0%    0.0%    0.0%    0.0%    0.0%    0.0%    0.0%    0.0%    0.0%    0.0%    0.0%    0.0%    0.0%    0.0%    0.0%    0.0%    0.0%    0.0%    0.0%    0.0%    0.0%    0.0%    0.0%    0.0%    0.0%    0.0%    0.0%    0.0%    0.0%    0.0%    0.0%    0.0%    0.0%    0.0%    0.0%    0.0%    0.0%    0.0%    0.0%    0.0%    0.0%    0.0%    0.0%    0.0%    0.0%    0.0%    0.0%    0.0%    0.0%    0.0%    0.0%    0.0%    0.0%    0.0%    0.0%    0.0%    0.0%    0.0%    0.0%    0.0%    0.0%    0.0%    0.0%    0.0%    0.0%    0.0%    0.0%    0.0%    0.0%    0.0%    0.0%    0.0%    0.0%    0.0%    0.0%    0.0%    0.0%    0.0%    0.0%    0.0%    0.0%    0.0%    0.0%    0.0%    0.0%    0.0%    0.0%    0.0%    0.0%    0.0%    0.0%    0.0%    0.0%    0.0%    0.0%    0.0%    0.0%    0.0%    0.0%    0.0%    0.0%    0.0%    0.0%    0.0%    0.0%    0.0%    0.0%    0.0%    0.0%    0.0%    0.0%    0.0%    0.0%    0.0%    0.0%    0.0%    0.0%    0.0%    0.0%    0.0%    0.0%    0.0%    0.0%    0.0%    0.0%    0.0%    0.0%    0.0%    0.0%    0.0%    0.0%    0.0%    0.0%    0.0%    0.0%    0.0%    0.0%    0.0%    0.0%    0.0%    0.0%    0.0%    0.0%    0.0%    0.0%    0.0%    0.0%    0.0%    0.0%    0.0%    0.0%    0.0%    0.0%    0.0%    0.0%    0.0%    0.0%    0.0%    0.0%    0.0%    0.0%    0.0%    0.0%    0.0%    0.0%    0.0%    0.0%    0.0%    0.0%    0.0%    0.0%    0.0%    0.0%    0.0%    0.0%    0.0%    0.0%    0.0%    0.0%    0.0%    0.0%    0.0%    0.0%    0.0%    0.0%    0.0%    0.0%    0.0%    0.0%    0.0%    0.0%    0.0%    0.0%    0.0%    0.0%    0.0%    0.0%    0.0%    0.0%    0.0%    0.0%    0.0%    0.0%    0.0%    0.0%    0.0%    0.0%    0.0%    0.0%    0.0%    0.0%    0.0%    0.0%    0.0%    0.0%    0.0%    0.0%    0.0%    0.0%    0.0%    0.0%    0.0%    0.0%    0.0%    0.0%    0.0%    0.0%    0.0%    0.0%    0.0%    0.0%    0.0%    0.0%    0.0%    0.0%    0.0%    0.0%    0.0%    0.0%    0.0%    0.0%    0.0%    0.0%    0.0%    0.0%    0.0%    0.0%    0.0%    0.0%    0.0%    0.0%    0.0%        k__Bacteria;p__Cyanobacteria; c__mle1-12      27    0.0%    0.0%    0.0%    0.0%    0.0%    0.0%    0.0%    0.0%    0.0%    0.0%    0.0%    0.0%    0.0%    0.0%    0.0%    0.0%    0.0%    0.0%    0.0%    0.0%    0.0%    0.0%    0.0%    0.0%    0.0%    0.0%    0.0%    0.0%    0.0%    0.0%    0.0%    0.0%    0.0%    0.0%    0.0%    0.0%    0.0%    0.0%    0.0%    0.0%    0.0%    0.0%    0.0%    0.0%    0.0%    0.0%    0.0%    0.0%    0.0%    0.0%    0.0%    0.0%    0.0%    0.0%    0.0%    0.0%    0.0%    0.0%    0.0%    0.0%    0.0%    0.0%    0.0%    0.0%    0.0%    0.0%    0.0%    0.0%    0.0%    0.0%    0.0%    0.0%    0.0%    0.0%    0.0%    0.0%    0.0%    0.0%    0.0%    0.0%    0.0%    0.0%    0.0%    0.0%    0.0%    0.0%    0.0%    0.0%    0.0%    0.0%    0.0%    0.0%    0.0%    0.0%    0.0%    0.0%    0.0%    0.0%    0.0%    0.0%    0.0%    0.0%    0.0%    0.0%    0.0%    0.0%    0.0%    0.0%    0.0%    0.0%    0.0%    0.0%    0.0%    0.0%    0.0%    0.0%    0.0%    0.0%    0.0%    0.0%    0.0%    0.0%    0.0%    0.0%    0.0%    0.0%    0.0%    0.0%    0.0%    0.0%    0.0%    0.0%    0.0%    0.0%    0.0%    0.0%    0.0%    0.0%    0.0%    0.0%    0.0%    0.0%    0.0%    0.0%    0.0%    0.0%    0.0%    0.0%    0.0%    0.0%    0.0%    0.0%    0.0%    0.0%    0.0%    0.0%    0.0%    0.0%    0.0%    0.0%    0.0%    0.0%    0.0%    0.0%    0.0%    0.0%    0.0%    0.0%    0.0%    0.0%    0.0%    0.0%    0.0%    0.0%    0.0%    0.0%    0.0%    0.0%    0.0%    0.0%    0.0%    0.0%    0.0%    0.0%    0.0%    0.0%    0.0%    0.0%    0.0%    0.0%    0.0%    0.0%    0.0%    0.0%    0.0%    0.0%    0.0%    0.0%    0.0%    0.0%    0.0%    0.0%    0.0%    0.0%    0.0%    0.0%    0.0%    0.0%    0.0%    0.0%    0.0%    0.0%    0.0%    0.0%    0.0%    0.0%    0.0%    0.0%    0.0%    0.0%    0.0%    0.0%    0.0%    0.0%    0.0%    0.0%    0.0%    0.0%    0.0%    0.0%    0.0%    0.0%    0.0%    0.0%    0.0%    0.0%    0.0%    0.0%    0.0%    0.0%    0.0%    0.0%    0.0%    0.0%    0.0%    0.0%    0.0%    0.0%    0.0%    0.0%    0.0%    0.0%    0.0%    0.0%    0.0%    0.0%    0.0%    0.0%    0.0%    0.0%    0.0%    0.0%    0.0%    0.0%    0.0%    0.0%    0.0%    0.0%    0.0%    0.0%    0.0%    0.0%    0.0%    0.0%    0.0%    0.0%    0.0%    0.0%    0.0%    0.0%    0.0%    0.0%    0.0%    0.0%    0.0%    0.0%    0.0%    0.0%    0.0%    0.0%    0.0%    0.0%    0.0%    0.0%    0.0%    0.0%    0.0%    0.0%    0.0%    0.0%    0.0%    0.0%    0.0%    0.0%    0.0%    0.0%    0.0%    0.0%    0.0%    0.0%    0.0%    0.0%    0.0%    0.0%    0.0%    0.0%    0.0%    0.0%    0.0%    0.0%    0.0%    0.0%    0.0%    0.0%    0.0%    0.0%    0.0%    0.0%    0.0%    0.0%    0.0%    0.0%    0.0%    0.0%    0.0%    0.0%    0.0%    0.0%    0.0%    0.0%    0.0%    0.0%    0.0%    0.0%    0.0%    0.0%    0.0%    0.0%    0.0%    0.0%    0.0%    0.0%    0.0%    0.0%    0.0%    0.0%    0.0%    0.0%        k__Bacteria;p__Deferribacteres; c__Deferribacteres (class)     468    0.0%    0.0%    0.0%    0.0%    0.0%    0.0%    0.0%    0.0%    0.0%    0.0%    0.0%    0.0%    0.0%    0.0%    0.0%    0.0%    0.0%    0.0%    0.0%    0.0%    0.0%    0.0%    0.0%    0.0%    0.0%    0.0%    0.0%    0.0%    0.0%    0.0%    0.0%    0.0%    0.0%    0.0%    0.3%    0.0%    0.0%    0.0%    0.0%    0.0%    0.0%    0.0%    0.0%    0.0%    0.0%    0.0%    0.0%    0.0%    0.0%    0.0%    0.0%    0.0%    0.0%    0.0%    0.1%    0.0%    0.0%    0.0%    0.0%    0.0%    0.0%    0.0%    0.0%    0.0%    0.0%    0.0%    0.0%    0.0%    0.0%    0.0%    0.0%    0.0%    0.0%    0.0%    0.0%    0.0%    0.0%    0.0%    0.0%    0.0%    0.0%    0.0%    0.0%    0.0%    0.0%    0.0%    0.0%    0.0%    0.0%    0.0%    0.0%    0.0%    0.0%    0.0%    0.0%    0.0%    0.0%    0.0%    0.0%    0.0%    0.0%    0.0%    0.0%    0.0%    0.0%    0.0%    0.0%    0.0%    0.0%    0.0%    0.0%    0.0%    0.0%    0.0%    0.0%    0.0%    0.0%    0.0%    0.0%    0.0%    0.0%    0.0%    0.0%    0.0%    0.0%    0.0%    0.0%    0.0%    0.0%    0.0%    0.0%    0.0%    0.0%    0.0%    0.0%    0.0%    0.0%    0.0%    0.0%    0.0%    0.0%    0.0%    0.0%    0.0%    0.0%    0.0%    0.0%    0.0%    0.0%    0.0%    0.0%    0.0%    0.0%    0.0%    0.0%    0.0%    0.0%    0.0%    0.0%    0.0%    0.0%    0.0%    0.0%    0.0%    0.0%    0.0%    0.0%    0.0%    0.0%    0.0%    0.0%    0.0%    0.0%    0.0%    0.0%    0.0%    0.0%    0.0%    0.0%    0.0%    0.0%    0.0%    0.0%    0.0%    0.0%    0.0%    0.0%    0.0%    0.0%    0.0%    0.0%    0.0%    0.0%    0.0%    0.0%    0.0%    0.0%    0.0%    0.0%    0.0%    0.0%    0.1%    0.0%    0.0%    0.0%    0.0%    0.0%    0.0%    0.0%    0.0%    0.0%    0.0%    0.0%    0.0%    0.0%    0.0%    0.0%    0.0%    0.0%    0.0%    0.0%    0.0%    0.0%    0.0%    0.0%    0.0%    0.0%    0.0%    0.0%    0.0%    0.0%    0.0%    0.0%    0.0%    0.0%    0.0%    0.0%    0.0%    0.0%    0.0%    0.0%    0.0%    0.0%    0.0%    0.0%    0.0%    0.0%    0.0%    0.0%    0.0%    0.0%    0.0%    0.0%    0.0%    0.0%    0.0%    0.0%    0.0%    0.0%    0.0%    0.0%    0.0%    0.0%    0.0%    0.0%    0.0%    0.0%    0.0%    0.0%    0.0%    0.0%    0.0%    0.0%    0.0%    0.0%    0.0%    0.0%    0.0%    0.0%    0.0%    0.0%    0.0%    0.0%    0.0%    0.0%    0.0%    0.0%    0.0%    0.0%    0.0%    0.0%    0.0%    0.0%    0.0%    0.0%    0.0%    0.0%    0.0%    0.0%    0.0%    0.0%    0.0%    0.0%    0.0%    0.0%    0.0%    0.0%    0.0%    0.0%    0.0%    0.0%    0.0%    0.0%    0.0%    0.0%    0.0%    0.0%    0.0%    0.0%    0.0%    0.0%    0.0%    0.0%    0.0%    0.0%    0.0%    0.0%    0.0%    0.0%    0.0%    0.0%    0.0%    0.0%    0.0%    0.0%    0.0%    0.0%    0.0%    0.0%    0.0%    0.0%    0.0%    0.0%    0.0%    0.0%    0.0%    0.0%    0.0%    0.0%    0.0%    0.0%    0.0%    0.0%    0.0%    0.0%    0.0%    0.0%    0.0%        k__Bacteria;p__Elusimicrobia; c__Elusimicrobia (class)      37    0.0%    0.0%    0.0%    0.0%    0.0%    0.0%    0.0%    0.0%    0.0%    0.0%    0.0%    0.0%    0.0%    0.0%    0.0%    0.0%    0.0%    0.0%    0.0%    0.0%    0.0%    0.0%    0.0%    0.0%    0.0%    0.0%    0.0%    0.0%    0.0%    0.0%    0.0%    0.0%    0.0%    0.0%    0.0%    0.0%    0.0%    0.0%    0.0%    0.0%    0.0%    0.0%    0.0%    0.0%    0.0%    0.0%    0.0%    0.0%    0.0%    0.0%    0.0%    0.0%    0.0%    0.0%    0.0%    0.0%    0.0%    0.0%    0.0%    0.0%    0.0%    0.0%    0.0%    0.0%    0.0%    0.0%    0.0%    0.0%    0.0%    0.0%    0.0%    0.0%    0.0%    0.0%    0.0%    0.0%    0.0%    0.0%    0.0%    0.0%    0.0%    0.0%    0.0%    0.0%    0.0%    0.0%    0.0%    0.0%    0.0%    0.0%    0.0%    0.0%    0.0%    0.0%    0.0%    0.0%    0.0%    0.0%    0.0%    0.0%    0.0%    0.0%    0.0%    0.0%    0.0%    0.0%    0.0%    0.0%    0.0%    0.0%    0.0%    0.0%    0.0%    0.0%    0.0%    0.0%    0.0%    0.0%    0.0%    0.0%    0.0%    0.0%    0.0%    0.0%    0.0%    0.0%    0.0%    0.0%    0.0%    0.0%    0.0%    0.0%    0.0%    0.0%    0.0%    0.0%    0.0%    0.0%    0.0%    0.0%    0.0%    0.0%    0.0%    0.0%    0.0%    0.0%    0.0%    0.0%    0.0%    0.0%    0.0%    0.0%    0.0%    0.0%    0.0%    0.0%    0.0%    0.0%    0.0%    0.0%    0.0%    0.0%    0.0%    0.0%    0.0%    0.0%    0.0%    0.0%    0.0%    0.0%    0.0%    0.0%    0.0%    0.0%    0.0%    0.0%    0.0%    0.0%    0.0%    0.0%    0.0%    0.0%    0.0%    0.0%    0.0%    0.0%    0.0%    0.0%    0.0%    0.0%    0.0%    0.0%    0.0%    0.0%    0.0%    0.0%    0.0%    0.0%    0.0%    0.0%    0.0%    0.0%    0.0%    0.0%    0.0%    0.0%    0.0%    0.0%    0.0%    0.0%    0.0%    0.0%    0.0%    0.0%    0.0%    0.0%    0.0%    0.0%    0.0%    0.0%    0.0%    0.0%    0.0%    0.0%    0.0%    0.0%    0.0%    0.0%    0.0%    0.0%    0.0%    0.0%    0.0%    0.0%    0.0%    0.0%    0.0%    0.0%    0.0%    0.0%    0.0%    0.0%    0.0%    0.0%    0.0%    0.0%    0.0%    0.0%    0.0%    0.0%    0.0%    0.0%    0.0%    0.0%    0.0%    0.0%    0.0%    0.0%    0.0%    0.0%    0.0%    0.0%    0.0%    0.0%    0.0%    0.0%    0.0%    0.0%    0.0%    0.0%    0.0%    0.0%    0.0%    0.0%    0.0%    0.0%    0.0%    0.0%    0.0%    0.0%    0.0%    0.0%    0.0%    0.0%    0.0%    0.0%    0.0%    0.0%    0.0%    0.0%    0.0%    0.0%    0.0%    0.0%    0.0%    0.0%    0.0%    0.0%    0.0%    0.0%    0.0%    0.0%    0.0%    0.0%    0.0%    0.0%    0.0%    0.0%    0.0%    0.0%    0.0%    0.0%    0.0%    0.0%    0.0%    0.0%    0.0%    0.0%    0.0%    0.0%    0.0%    0.0%    0.0%    0.0%    0.0%    0.0%    0.0%    0.0%    0.0%    0.0%    0.0%    0.0%    0.0%    0.0%    0.0%    0.0%    0.0%    0.0%    0.0%    0.0%    0.0%    0.0%    0.0%    0.0%    0.0%    0.0%    0.0%    0.0%    0.0%    0.0%    0.0%    0.0%    0.0%    0.0%    0.0%    0.0%    0.0%    0.0%        k__Bacteria;p__Fibrobacteres; c__Fibrobacteres (class)       1    0.0%    0.0%    0.0%    0.0%    0.0%    0.0%    0.0%    0.0%    0.0%    0.0%    0.0%    0.0%    0.0%    0.0%    0.0%    0.0%    0.0%    0.0%    0.0%    0.0%    0.0%    0.0%    0.0%    0.0%    0.0%    0.0%    0.0%    0.0%    0.0%    0.0%    0.0%    0.0%    0.0%    0.0%    0.0%    0.0%    0.0%    0.0%    0.0%    0.0%    0.0%    0.0%    0.0%    0.0%    0.0%    0.0%    0.0%    0.0%    0.0%    0.0%    0.0%    0.0%    0.0%    0.0%    0.0%    0.0%    0.0%    0.0%    0.0%    0.0%    0.0%    0.0%    0.0%    0.0%    0.0%    0.0%    0.0%    0.0%    0.0%    0.0%    0.0%    0.0%    0.0%    0.0%    0.0%    0.0%    0.0%    0.0%    0.0%    0.0%    0.0%    0.0%    0.0%    0.0%    0.0%    0.0%    0.0%    0.0%    0.0%    0.0%    0.0%    0.0%    0.0%    0.0%    0.0%    0.0%    0.0%    0.0%    0.0%    0.0%    0.0%    0.0%    0.0%    0.0%    0.0%    0.0%    0.0%    0.0%    0.0%    0.0%    0.0%    0.0%    0.0%    0.0%    0.0%    0.0%    0.0%    0.0%    0.0%    0.0%    0.0%    0.0%    0.0%    0.0%    0.0%    0.0%    0.0%    0.0%    0.0%    0.0%    0.0%    0.0%    0.0%    0.0%    0.0%    0.0%    0.0%    0.0%    0.0%    0.0%    0.0%    0.0%    0.0%    0.0%    0.0%    0.0%    0.0%    0.0%    0.0%    0.0%    0.0%    0.0%    0.0%    0.0%    0.0%    0.0%    0.0%    0.0%    0.0%    0.0%    0.0%    0.0%    0.0%    0.0%    0.0%    0.0%    0.0%    0.0%    0.0%    0.0%    0.0%    0.0%    0.0%    0.0%    0.0%    0.0%    0.0%    0.0%    0.0%    0.0%    0.0%    0.0%    0.0%    0.0%    0.0%    0.0%    0.0%    0.0%    0.0%    0.0%    0.0%    0.0%    0.0%    0.0%    0.0%    0.0%    0.0%    0.0%    0.0%    0.0%    0.0%    0.0%    0.0%    0.0%    0.0%    0.0%    0.0%    0.0%    0.0%    0.0%    0.0%    0.0%    0.0%    0.0%    0.0%    0.0%    0.0%    0.0%    0.0%    0.0%    0.0%    0.0%    0.0%    0.0%    0.0%    0.0%    0.0%    0.0%    0.0%    0.0%    0.0%    0.0%    0.0%    0.0%    0.0%    0.0%    0.0%    0.0%    0.0%    0.0%    0.0%    0.0%    0.0%    0.0%    0.0%    0.0%    0.0%    0.0%    0.0%    0.0%    0.0%    0.0%    0.0%    0.0%    0.0%    0.0%    0.0%    0.0%    0.0%    0.0%    0.0%    0.0%    0.0%    0.0%    0.0%    0.0%    0.0%    0.0%    0.0%    0.0%    0.0%    0.0%    0.0%    0.0%    0.0%    0.0%    0.0%    0.0%    0.0%    0.0%    0.0%    0.0%    0.0%    0.0%    0.0%    0.0%    0.0%    0.0%    0.0%    0.0%    0.0%    0.0%    0.0%    0.0%    0.0%    0.0%    0.0%    0.0%    0.0%    0.0%    0.0%    0.0%    0.0%    0.0%    0.0%    0.0%    0.0%    0.0%    0.0%    0.0%    0.0%    0.0%    0.0%    0.0%    0.0%    0.0%    0.0%    0.0%    0.0%    0.0%    0.0%    0.0%    0.0%    0.0%    0.0%    0.0%    0.0%    0.0%    0.0%    0.0%    0.0%    0.0%    0.0%    0.0%    0.0%    0.0%    0.0%    0.0%    0.0%    0.0%    0.0%    0.0%    0.0%    0.0%    0.0%    0.0%    0.0%    0.0%    0.0%    0.0%    0.0%    0.0%    0.0%    0.0%    0.0%    0.0%    0.0%    0.0%        k__Bacteria;p__Firmicutes; c__Bacilli   4376665   25.7%   35.2%   30.2%   34.7%   21.3%    3.3%   38.3%    9.7%   26.9%   41.8%   48.2%   11.4%   21.7%   24.7%   37.4%   45.1%   28.2%   12.1%   20.5%   17.6%   24.0%   22.4%   48.2%   11.3%   24.3%   41.8%   11.1%   42.3%   31.4%   16.6%   12.2%   36.9%   14.5%   17.3%   22.3%   18.5%   26.1%   11.0%   24.5%   16.2%   17.5%   28.3%   33.7%   27.1%   29.9%   31.6%   30.1%   22.8%   32.9%   26.1%   37.0%   15.9%   21.3%   37.1%   19.7%   17.7%   20.2%    8.8%   35.3%   20.5%   21.6%   48.9%   27.5%   39.3%   14.1%   41.2%    6.3%   25.0%   10.3%   31.2%    3.3%   29.2%    5.7%   19.3%   31.9%    6.5%   17.9%   34.8%   40.0%   40.4%   39.9%   20.1%   45.2%   54.0%   24.7%   34.9%   33.8%    4.7%   29.9%   39.6%    4.0%   44.6%   46.1%   12.0%   14.5%   27.5%   10.8%    5.4%   35.4%   39.6%   14.8%   32.1%   33.9%   35.2%   34.9%   47.3%   16.0%    4.4%    1.9%   39.7%   52.6%   19.7%   23.3%    9.3%   33.0%   48.8%   25.5%   49.3%   31.8%    8.9%    1.8%   47.2%   35.5%   31.5%    6.4%   15.6%   22.2%    8.0%   15.5%   20.9%   32.7%   15.6%    3.3%   57.4%   18.3%    9.7%   19.7%   16.1%   13.5%   11.4%   18.8%   20.6%   10.8%    5.9%    7.5%   21.7%   30.0%   37.8%   26.5%    3.1%    5.2%    3.3%   21.0%   16.6%   55.9%    8.4%   21.4%   12.9%   22.8%   32.6%   12.2%   20.1%    9.3%    3.9%   14.9%    9.6%   23.7%   25.5%   24.0%   33.5%   19.2%   24.1%   12.6%   34.0%   39.9%   38.1%   44.7%    8.7%    5.3%   30.9%   42.6%   40.8%   42.0%   10.2%   23.1%   37.7%   37.5%   50.8%   39.3%   45.4%   36.6%   31.2%   34.0%   27.4%   46.1%   25.5%   27.3%   36.6%   45.6%   33.9%   24.6%   19.3%   13.6%   25.5%   25.5%   31.6%    7.9%   10.6%   19.1%   22.1%   34.0%   21.5%   14.4%   18.2%   22.4%   29.0%    8.7%   21.8%   17.4%   30.8%   45.5%   16.0%    1.2%    8.7%   11.4%   32.0%   49.5%   39.5%   22.3%   26.6%   37.4%    9.9%    9.5%   21.1%   20.5%    6.9%   22.1%   21.1%   70.0%   47.5%   47.0%   54.4%   42.1%   29.3%   42.6%   64.1%   33.9%   44.2%   28.4%   14.1%   14.3%   13.5%    4.8%   14.9%    3.6%    8.3%   16.5%    6.9%   14.9%   38.0%   19.2%   19.7%   27.7%    6.5%   15.4%    2.7%   21.9%   41.1%   42.4%   18.9%   17.6%   22.2%    9.3%   27.0%   19.1%   16.4%   22.8%   44.0%   28.2%    7.3%   30.6%   79.9%   27.1%   38.9%   17.7%   41.1%   16.1%   21.0%   30.1%   39.4%   38.8%   28.9%   24.6%   28.4%   58.5%   63.8%   34.1%   38.5%   39.8%   48.3%   22.2%   43.7%   53.5%   39.7%   46.9%   35.3%   46.7%   46.4%   17.6%   34.4%   40.4%   45.4%   19.9%   40.1%   47.7%   34.4%    8.4%   41.2%   49.4%   18.1%   27.5%   38.1%    7.4%   15.8%   28.9%   28.8%   30.9%   21.7%   43.7%   42.5%   57.7%   54.2%   58.3%   74.4%   71.6%   48.9%   41.6%   41.6%   46.7%    8.9%   46.7%   20.2%   49.0%   42.8%   42.5%   47.0%   47.1%   36.2%    6.6%   21.6%   26.6%   38.0%   50.5%   43.1%   41.5%    0.6%   30.3%   76.9%        k__Bacteria;p__Firmicutes; c__Clostridia   1307796    7.7%    6.4%    5.2%    6.9%    5.2%    0.2%    9.3%    3.2%   32.2%    4.5%    5.1%    5.0%    3.1%   10.2%    8.9%    4.3%   16.6%    3.5%    3.4%   18.3%    7.3%    8.0%   10.9%   28.7%   15.2%   16.0%    6.9%    7.4%    7.0%   17.2%   34.2%    7.7%    3.1%    8.3%   14.2%   19.9%    8.1%   44.2%    6.7%    2.7%    6.1%    5.9%    6.8%    6.4%    8.3%    9.8%    6.2%   11.6%    7.4%    9.0%    4.9%    1.8%    5.3%    3.9%    5.8%   18.6%    6.7%    1.5%    8.1%    5.0%    4.0%    6.9%    6.3%    4.9%   15.8%    7.1%    0.7%    7.3%    2.0%    5.2%    2.8%    5.6%    1.3%   10.8%   14.0%    0.6%    2.3%    5.5%   21.4%    8.4%    9.3%    4.4%    7.4%    6.7%   14.4%   16.1%    8.6%    0.3%    3.3%    8.6%    0.4%    4.2%    6.7%    1.6%   10.2%   11.2%    2.8%    0.3%   10.2%    9.1%    3.5%    4.7%    5.6%   12.7%   16.2%    4.6%   11.4%    0.8%    0.1%    9.5%    7.1%   18.2%    3.7%    1.6%   11.9%    5.7%    9.1%    9.2%    4.4%    4.2%    0.1%    7.9%    9.4%    4.6%    0.9%    5.7%    8.0%    1.1%    3.5%    6.6%    8.7%    2.1%    0.7%    5.3%    8.8%    1.6%    1.8%    5.1%    0.5%    1.3%    0.1%    6.5%    3.1%    0.8%    1.5%    9.3%    8.4%    6.5%   16.7%    0.5%    2.1%    0.7%    4.2%    5.3%    3.9%    0.9%    5.4%    3.2%    1.0%    6.8%    2.0%    5.2%    1.9%    0.5%    6.6%    2.8%   10.0%   18.5%   10.3%    7.8%    5.7%   10.6%    1.0%    6.7%    5.9%   12.2%    7.7%    1.3%    1.6%    5.1%   13.4%   12.9%    3.5%    1.2%    9.2%   13.9%   18.2%    9.9%   12.6%    5.1%   14.1%   13.9%   11.3%   16.8%    3.5%    4.7%   15.8%   14.7%    7.8%    8.3%    9.4%   32.2%    7.9%    9.8%    7.8%    4.9%    1.9%    5.6%    5.7%   18.2%    7.8%    7.0%    1.3%    6.4%    2.4%   19.4%    9.1%    5.9%   17.8%    9.3%   12.9%    4.6%    0.1%    2.4%    5.6%   10.2%    6.1%    6.1%    4.5%    9.9%    8.5%    3.6%    1.9%    5.9%    4.7%    6.0%    6.0%    3.0%    1.1%    6.3%    8.6%    4.9%    8.8%    6.5%    5.6%   11.8%    4.1%    4.5%    5.2%   26.4%    4.4%    4.9%    1.6%    5.1%    0.9%    6.3%    5.1%    2.6%    3.8%    6.2%    5.2%    4.3%    3.6%    2.4%   11.0%    0.5%    5.0%    5.9%    1.9%    8.6%   14.0%    7.9%    3.1%   10.8%    3.9%    6.5%   11.5%    7.3%    6.9%   55.8%    2.4%    0.6%    6.8%   10.6%    7.9%   17.5%    6.8%    5.8%   20.3%    2.7%    7.1%   12.2%   10.3%    6.7%    3.7%    5.1%    8.0%    7.6%    7.6%    5.8%   21.7%    6.2%    9.0%    6.3%    5.6%    5.5%    8.3%    6.4%    3.3%    9.9%    6.0%    8.3%    3.0%    8.7%    5.7%    9.2%    1.6%    9.1%   10.7%    5.7%    9.1%    6.0%   40.8%    1.9%    5.5%    8.6%   10.1%   12.6%    6.8%    6.6%    5.7%    5.4%    8.2%    2.7%    3.9%    6.3%    7.3%   16.0%    8.0%    0.3%    3.6%    1.4%    7.3%    8.7%    4.6%   18.0%    4.1%   20.1%    1.3%   11.7%    4.2%    8.0%    7.4%    8.4%    5.2%    0.3%    7.6%    7.4%        k__Bacteria;p__Fusobacteria; c__Fusobacteria (class)   292142    1.7%    9.1%   10.0%    8.4%    3.2%    0.1%    5.0%    1.7%    1.1%    3.9%    4.6%    0.2%    0.3%    7.3%    5.1%    1.3%    2.5%    0.9%    1.5%    0.5%    2.2%    1.4%    0.6%    0.1%    0.9%    1.1%    0.2%    2.7%    2.6%    0.7%    0.3%    4.0%    0.3%    8.1%    2.9%    7.5%    3.6%    1.0%    3.8%    1.4%    8.2%    5.2%    4.6%    4.3%   10.1%   19.1%    2.6%    0.9%    7.7%    4.2%    2.9%    0.8%    0.3%    1.8%    2.2%    2.0%    1.6%    0.7%    5.5%    1.2%    1.2%    1.7%    3.5%    6.5%    8.5%    2.2%    0.3%    4.3%    0.9%    3.6%    1.7%    1.1%    0.8%    2.7%    2.5%    0.2%    0.6%    3.4%    2.0%    4.5%    3.3%    0.5%    2.2%    2.9%    0.3%    0.7%    2.6%    0.1%    2.0%    1.7%    0.0%    0.6%    2.0%    0.6%    1.0%    0.7%    1.0%    0.1%    2.1%    2.3%    0.7%    0.4%    0.5%    0.8%    0.8%    3.8%    7.3%    0.1%    0.0%    1.2%    0.7%    4.0%    1.4%    0.1%    2.7%    0.8%    0.5%    0.5%    1.0%    1.0%    0.0%    0.7%    1.5%    1.5%    0.1%    1.0%    1.9%    0.1%    0.9%    0.7%    1.1%    0.3%    0.1%    0.8%    4.0%    0.2%    0.7%    0.5%    0.1%    0.7%    0.0%    1.7%    1.0%    0.2%    0.4%    0.5%    4.4%    1.1%    0.4%    0.1%    0.2%    0.4%    1.6%    0.4%    1.2%    0.3%    2.7%    0.2%    0.4%    1.9%    3.5%    2.0%    3.7%    0.8%    0.5%    0.9%    3.8%    0.8%    1.1%    1.7%    1.6%    2.9%    0.3%    1.4%    1.8%    0.6%    0.9%    0.8%    0.3%    1.0%    0.9%    0.9%    0.7%    0.3%    0.6%    0.8%    0.3%    1.2%    0.9%    1.1%    0.5%    1.5%    0.7%    0.4%    0.4%    0.5%    1.4%    1.7%    0.2%    0.4%    2.3%    6.6%    0.4%    1.4%    1.6%    1.3%    0.2%   14.4%    1.5%    1.0%    1.0%    0.6%    0.5%    9.6%    0.4%    0.5%    0.6%    1.5%    1.5%    0.6%    1.1%    0.5%    0.0%    0.4%    0.3%    0.8%    0.7%    2.8%    0.5%    2.8%    0.9%    0.2%    0.1%    0.8%    0.8%    0.1%    0.4%    0.4%    0.1%    0.2%    0.3%    0.4%    1.9%    1.2%    0.5%    0.1%    0.9%    0.0%    1.1%    2.2%    1.2%    2.9%    0.4%    0.5%    0.3%    0.2%    2.2%    0.4%    0.5%    2.8%    0.5%    0.6%    0.8%    0.2%    3.6%    0.0%    0.5%    1.4%    0.6%    0.2%    0.9%    1.1%    0.7%    1.5%    0.3%    0.5%    0.4%    0.9%    1.5%    0.2%    0.2%    0.1%    0.4%    0.1%    0.8%    1.8%    0.8%    1.1%    0.3%    0.0%    0.4%    0.3%    1.9%    1.8%    1.2%    0.2%    1.8%    1.1%    2.1%    0.8%    0.8%    0.8%    1.3%    0.9%    2.5%    1.0%    1.2%    0.5%    0.4%    5.3%    0.0%    0.3%    0.2%    3.6%    2.8%    0.6%    0.3%    2.0%    2.4%    0.6%    3.5%    4.2%    0.3%    0.3%    1.1%    1.3%    1.5%    0.9%    2.1%    1.5%    0.7%    1.5%    1.5%    0.3%    0.2%    0.8%    4.8%    1.5%    3.7%    0.1%    0.6%    0.1%    1.7%    0.5%    0.3%    0.7%    0.1%    1.7%    0.1%    0.1%    0.5%    8.7%    0.6%    0.4%    0.7%    0.1%    0.0%    1.7%        k__Bacteria;p__GN02; c__       0    0.0%    0.0%    0.0%    0.0%    0.0%    0.0%    0.0%    0.0%    0.0%    0.0%    0.0%    0.0%    0.0%    0.0%    0.0%    0.0%    0.0%    0.0%    0.0%    0.0%    0.0%    0.0%    0.0%    0.0%    0.0%    0.0%    0.0%    0.0%    0.0%    0.0%    0.0%    0.0%    0.0%    0.0%    0.0%    0.0%    0.0%    0.0%    0.0%    0.0%    0.0%    0.0%    0.0%    0.0%    0.0%    0.0%    0.0%    0.0%    0.0%    0.0%    0.0%    0.0%    0.0%    0.0%    0.0%    0.0%    0.0%    0.0%    0.0%    0.0%    0.0%    0.0%    0.0%    0.0%    0.0%    0.0%    0.0%    0.0%    0.0%    0.0%    0.0%    0.0%    0.0%    0.0%    0.0%    0.0%    0.0%    0.0%    0.0%    0.0%    0.0%    0.0%    0.0%    0.0%    0.0%    0.0%    0.0%    0.0%    0.0%    0.0%    0.0%    0.0%    0.0%    0.0%    0.0%    0.0%    0.0%    0.0%    0.0%    0.0%    0.0%    0.0%    0.0%    0.0%    0.0%    0.0%    0.0%    0.0%    0.0%    0.0%    0.0%    0.0%    0.0%    0.0%    0.0%    0.0%    0.0%    0.0%    0.0%    0.0%    0.0%    0.0%    0.0%    0.0%    0.0%    0.0%    0.0%    0.0%    0.0%    0.0%    0.0%    0.0%    0.0%    0.0%    0.0%    0.0%    0.0%    0.0%    0.0%    0.0%    0.0%    0.0%    0.0%    0.0%    0.0%    0.0%    0.0%    0.0%    0.0%    0.0%    0.0%    0.0%    0.0%    0.0%    0.0%    0.0%    0.0%    0.0%    0.0%    0.0%    0.0%    0.0%    0.0%    0.0%    0.0%    0.0%    0.0%    0.0%    0.0%    0.0%    0.0%    0.0%    0.0%    0.0%    0.0%    0.0%    0.0%    0.0%    0.0%    0.0%    0.0%    0.0%    0.0%    0.0%    0.0%    0.0%    0.0%    0.0%    0.0%    0.0%    0.0%    0.0%    0.0%    0.0%    0.0%    0.0%    0.0%    0.0%    0.0%    0.0%    0.0%    0.0%    0.0%    0.0%    0.0%    0.0%    0.0%    0.0%    0.0%    0.0%    0.0%    0.0%    0.0%    0.0%    0.0%    0.0%    0.0%    0.0%    0.0%    0.0%    0.0%    0.0%    0.0%    0.0%    0.0%    0.0%    0.0%    0.0%    0.0%    0.0%    0.0%    0.0%    0.0%    0.0%    0.0%    0.0%    0.0%    0.0%    0.0%    0.0%    0.0%    0.0%    0.0%    0.0%    0.0%    0.0%    0.0%    0.0%    0.0%    0.0%    0.0%    0.0%    0.0%    0.0%    0.0%    0.0%    0.0%    0.0%    0.0%    0.0%    0.0%    0.0%    0.0%    0.0%    0.0%    0.0%    0.0%    0.0%    0.0%    0.0%    0.0%    0.0%    0.0%    0.0%    0.0%    0.0%    0.0%    0.0%    0.0%    0.0%    0.0%    0.0%    0.0%    0.0%    0.0%    0.0%    0.0%    0.0%    0.0%    0.0%    0.0%    0.0%    0.0%    0.0%    0.0%    0.0%    0.0%    0.0%    0.0%    0.0%    0.0%    0.0%    0.0%    0.0%    0.0%    0.0%    0.0%    0.0%    0.0%    0.0%    0.0%    0.0%    0.0%    0.0%    0.0%    0.0%    0.0%    0.0%    0.0%    0.0%    0.0%    0.0%    0.0%    0.0%    0.0%    0.0%    0.0%    0.0%    0.0%    0.0%    0.0%    0.0%    0.0%    0.0%    0.0%    0.0%    0.0%    0.0%    0.0%    0.0%    0.0%    0.0%    0.0%    0.0%    0.0%    0.0%    0.0%    0.0%    0.0%    0.0%    0.0%    0.0%    0.0%    0.0%    0.0%    0.0%    0.0%    0.0%        k__Bacteria;p__GN02; c__VC12-cl04       1    0.0%    0.0%    0.0%    0.0%    0.0%    0.0%    0.0%    0.0%    0.0%    0.0%    0.0%    0.0%    0.0%    0.0%    0.0%    0.0%    0.0%    0.0%    0.0%    0.0%    0.0%    0.0%    0.0%    0.0%    0.0%    0.0%    0.0%    0.0%    0.0%    0.0%    0.0%    0.0%    0.0%    0.0%    0.0%    0.0%    0.0%    0.0%    0.0%    0.0%    0.0%    0.0%    0.0%    0.0%    0.0%    0.0%    0.0%    0.0%    0.0%    0.0%    0.0%    0.0%    0.0%    0.0%    0.0%    0.0%    0.0%    0.0%    0.0%    0.0%    0.0%    0.0%    0.0%    0.0%    0.0%    0.0%    0.0%    0.0%    0.0%    0.0%    0.0%    0.0%    0.0%    0.0%    0.0%    0.0%    0.0%    0.0%    0.0%    0.0%    0.0%    0.0%    0.0%    0.0%    0.0%    0.0%    0.0%    0.0%    0.0%    0.0%    0.0%    0.0%    0.0%    0.0%    0.0%    0.0%    0.0%    0.0%    0.0%    0.0%    0.0%    0.0%    0.0%    0.0%    0.0%    0.0%    0.0%    0.0%    0.0%    0.0%    0.0%    0.0%    0.0%    0.0%    0.0%    0.0%    0.0%    0.0%    0.0%    0.0%    0.0%    0.0%    0.0%    0.0%    0.0%    0.0%    0.0%    0.0%    0.0%    0.0%    0.0%    0.0%    0.0%    0.0%    0.0%    0.0%    0.0%    0.0%    0.0%    0.0%    0.0%    0.0%    0.0%    0.0%    0.0%    0.0%    0.0%    0.0%    0.0%    0.0%    0.0%    0.0%    0.0%    0.0%    0.0%    0.0%    0.0%    0.0%    0.0%    0.0%    0.0%    0.0%    0.0%    0.0%    0.0%    0.0%    0.0%    0.0%    0.0%    0.0%    0.0%    0.0%    0.0%    0.0%    0.0%    0.0%    0.0%    0.0%    0.0%    0.0%    0.0%    0.0%    0.0%    0.0%    0.0%    0.0%    0.0%    0.0%    0.0%    0.0%    0.0%    0.0%    0.0%    0.0%    0.0%    0.0%    0.0%    0.0%    0.0%    0.0%    0.0%    0.0%    0.0%    0.0%    0.0%    0.0%    0.0%    0.0%    0.0%    0.0%    0.0%    0.0%    0.0%    0.0%    0.0%    0.0%    0.0%    0.0%    0.0%    0.0%    0.0%    0.0%    0.0%    0.0%    0.0%    0.0%    0.0%    0.0%    0.0%    0.0%    0.0%    0.0%    0.0%    0.0%    0.0%    0.0%    0.0%    0.0%    0.0%    0.0%    0.0%    0.0%    0.0%    0.0%    0.0%    0.0%    0.0%    0.0%    0.0%    0.0%    0.0%    0.0%    0.0%    0.0%    0.0%    0.0%    0.0%    0.0%    0.0%    0.0%    0.0%    0.0%    0.0%    0.0%    0.0%    0.0%    0.0%    0.0%    0.0%    0.0%    0.0%    0.0%    0.0%    0.0%    0.0%    0.0%    0.0%    0.0%    0.0%    0.0%    0.0%    0.0%    0.0%    0.0%    0.0%    0.0%    0.0%    0.0%    0.0%    0.0%    0.0%    0.0%    0.0%    0.0%    0.0%    0.0%    0.0%    0.0%    0.0%    0.0%    0.0%    0.0%    0.0%    0.0%    0.0%    0.0%    0.0%    0.0%    0.0%    0.0%    0.0%    0.0%    0.0%    0.0%    0.0%    0.0%    0.0%    0.0%    0.0%    0.0%    0.0%    0.0%    0.0%    0.0%    0.0%    0.0%    0.0%    0.0%    0.0%    0.0%    0.0%    0.0%    0.0%    0.0%    0.0%    0.0%    0.0%    0.0%    0.0%    0.0%    0.0%    0.0%    0.0%    0.0%    0.0%    0.0%    0.0%    0.0%    0.0%    0.0%    0.0%    0.0%    0.0%    0.0%    0.0%    0.0%    0.0%    0.0%        k__Bacteria;p__Gemmatimonadetes; c__Gemmatimonadetes (class)    4768    0.0%    0.0%    0.0%    0.0%    0.0%    0.0%    0.0%    0.0%    0.0%    0.0%    0.0%    0.0%    0.0%    0.0%    0.0%    0.0%    0.0%    0.0%    0.0%    0.0%    0.0%    0.0%    0.0%    0.0%    0.0%    0.0%    0.0%    0.0%    0.0%    0.0%    0.0%    0.0%    0.0%    0.0%    0.0%    0.0%    0.0%    0.0%    0.0%    0.1%    0.0%    0.0%    0.0%    0.0%    0.0%    0.0%    0.0%    0.0%    0.0%    0.0%    0.0%    0.0%    0.0%    0.0%    0.0%    0.0%    0.0%    0.0%    0.0%    0.0%    0.0%    0.0%    0.0%    0.0%    0.1%    0.0%    0.0%    0.1%    0.0%    0.1%    0.0%    0.0%    0.0%    0.1%    0.1%    0.0%    0.0%    0.0%    0.0%    0.0%    0.0%    0.0%    0.0%    0.0%    0.0%    0.0%    0.1%    0.0%    0.0%    0.1%    0.0%    0.0%    0.0%    0.0%    0.0%    0.0%    0.1%    0.0%    0.0%    0.1%    0.1%    0.0%    0.0%    0.0%    0.0%    0.0%    0.1%    0.0%    0.0%    0.1%    0.0%    0.1%    0.0%    0.0%    0.0%    0.0%    0.0%    0.0%    0.0%    0.2%    0.0%    0.0%    0.1%    0.1%    0.0%    0.3%    0.2%    0.0%    0.1%    0.1%    0.1%    0.0%    0.1%    0.0%    0.2%    0.0%    0.0%    0.0%    0.0%    0.0%    0.0%    0.0%    0.1%    0.0%    0.1%    0.0%    0.0%    0.0%    0.0%    0.0%    0.0%    0.0%    0.1%    0.0%    0.0%    0.0%    0.1%    0.0%    0.0%    0.1%    0.1%    0.1%    0.1%    0.0%    0.0%    0.0%    0.1%    0.0%    0.0%    0.0%    0.0%    0.1%    0.0%    0.0%    0.0%    0.0%    0.0%    0.0%    0.0%    0.0%    0.0%    0.0%    0.0%    0.0%    0.0%    0.0%    0.0%    0.0%    0.0%    0.0%    0.0%    0.0%    0.0%    0.0%    0.0%    0.0%    0.1%    0.0%    0.0%    0.0%    0.0%    0.0%    0.0%    0.1%    0.0%    0.0%    0.0%    0.0%    0.1%    0.1%    0.1%    0.0%    0.2%    0.0%    0.0%    0.0%    0.0%    0.0%    0.0%    0.1%    0.0%    0.0%    0.0%    0.0%    0.0%    0.0%    0.0%    0.0%    0.0%    0.0%    0.0%    0.0%    0.2%    0.0%    0.1%    0.0%    0.1%    0.0%    0.0%    0.0%    0.0%    0.0%    0.0%    0.0%    0.0%    0.0%    0.0%    0.0%    0.0%    0.0%    0.1%    0.0%    0.0%    0.0%    0.0%    0.0%    0.0%    0.0%    0.0%    0.0%    0.1%    0.1%    0.0%    0.0%    0.1%    0.0%    0.0%    0.0%    0.0%    0.0%    0.0%    0.0%    0.1%    0.1%    0.0%    0.0%    0.0%    0.0%    0.0%    0.0%    0.0%    0.0%    0.0%    0.0%    0.0%    0.0%    0.0%    0.2%    0.0%    0.0%    0.0%    0.0%    0.0%    0.0%    0.1%    0.0%    0.0%    0.0%    0.0%    0.0%    0.0%    0.0%    0.0%    0.1%    0.0%    0.0%    0.0%    0.0%    0.0%    0.0%    0.0%    0.0%    0.0%    0.0%    0.0%    0.0%    0.0%    0.0%    0.0%    0.0%    0.0%    0.0%    0.0%    0.0%    0.0%    0.0%    0.0%    0.0%    0.0%    0.0%    0.0%    0.0%    0.0%    0.0%    0.0%    0.0%    0.0%    0.0%    0.0%    0.0%    0.0%    0.0%    0.0%    0.0%    0.0%    0.0%    0.0%    0.0%    0.0%    0.0%    0.0%    0.0%    0.0%    0.0%    0.0%    0.0%    0.0%    0.0%        k__Bacteria;p__Lentisphaerae; c__       0    0.0%    0.0%    0.0%    0.0%    0.0%    0.0%    0.0%    0.0%    0.0%    0.0%    0.0%    0.0%    0.0%    0.0%    0.0%    0.0%    0.0%    0.0%    0.0%    0.0%    0.0%    0.0%    0.0%    0.0%    0.0%    0.0%    0.0%    0.0%    0.0%    0.0%    0.0%    0.0%    0.0%    0.0%    0.0%    0.0%    0.0%    0.0%    0.0%    0.0%    0.0%    0.0%    0.0%    0.0%    0.0%    0.0%    0.0%    0.0%    0.0%    0.0%    0.0%    0.0%    0.0%    0.0%    0.0%    0.0%    0.0%    0.0%    0.0%    0.0%    0.0%    0.0%    0.0%    0.0%    0.0%    0.0%    0.0%    0.0%    0.0%    0.0%    0.0%    0.0%    0.0%    0.0%    0.0%    0.0%    0.0%    0.0%    0.0%    0.0%    0.0%    0.0%    0.0%    0.0%    0.0%    0.0%    0.0%    0.0%    0.0%    0.0%    0.0%    0.0%    0.0%    0.0%    0.0%    0.0%    0.0%    0.0%    0.0%    0.0%    0.0%    0.0%    0.0%    0.0%    0.0%    0.0%    0.0%    0.0%    0.0%    0.0%    0.0%    0.0%    0.0%    0.0%    0.0%    0.0%    0.0%    0.0%    0.0%    0.0%    0.0%    0.0%    0.0%    0.0%    0.0%    0.0%    0.0%    0.0%    0.0%    0.0%    0.0%    0.0%    0.0%    0.0%    0.0%    0.0%    0.0%    0.0%    0.0%    0.0%    0.0%    0.0%    0.0%    0.0%    0.0%    0.0%    0.0%    0.0%    0.0%    0.0%    0.0%    0.0%    0.0%    0.0%    0.0%    0.0%    0.0%    0.0%    0.0%    0.0%    0.0%    0.0%    0.0%    0.0%    0.0%    0.0%    0.0%    0.0%    0.0%    0.0%    0.0%    0.0%    0.0%    0.0%    0.0%    0.0%    0.0%    0.0%    0.0%    0.0%    0.0%    0.0%    0.0%    0.0%    0.0%    0.0%    0.0%    0.0%    0.0%    0.0%    0.0%    0.0%    0.0%    0.0%    0.0%    0.0%    0.0%    0.0%    0.0%    0.0%    0.0%    0.0%    0.0%    0.0%    0.0%    0.0%    0.0%    0.0%    0.0%    0.0%    0.0%    0.0%    0.0%    0.0%    0.0%    0.0%    0.0%    0.0%    0.0%    0.0%    0.0%    0.0%    0.0%    0.0%    0.0%    0.0%    0.0%    0.0%    0.0%    0.0%    0.0%    0.0%    0.0%    0.0%    0.0%    0.0%    0.0%    0.0%    0.0%    0.0%    0.0%    0.0%    0.0%    0.0%    0.0%    0.0%    0.0%    0.0%    0.0%    0.0%    0.0%    0.0%    0.0%    0.0%    0.0%    0.0%    0.0%    0.0%    0.0%    0.0%    0.0%    0.0%    0.0%    0.0%    0.0%    0.0%    0.0%    0.0%    0.0%    0.0%    0.0%    0.0%    0.0%    0.0%    0.0%    0.0%    0.0%    0.0%    0.0%    0.0%    0.0%    0.0%    0.0%    0.0%    0.0%    0.0%    0.0%    0.0%    0.0%    0.0%    0.0%    0.0%    0.0%    0.0%    0.0%    0.0%    0.0%    0.0%    0.0%    0.0%    0.0%    0.0%    0.0%    0.0%    0.0%    0.0%    0.0%    0.0%    0.0%    0.0%    0.0%    0.0%    0.0%    0.0%    0.0%    0.0%    0.0%    0.0%    0.0%    0.0%    0.0%    0.0%    0.0%    0.0%    0.0%    0.0%    0.0%    0.0%    0.0%    0.0%    0.0%    0.0%    0.0%    0.0%    0.0%    0.0%    0.0%    0.0%    0.0%    0.0%    0.0%    0.0%    0.0%    0.0%    0.0%    0.0%    0.0%    0.0%    0.0%    0.0%    0.0%    0.0%    0.0%    0.0%    0.0%    0.0%    0.0%    0.0%        k__Bacteria;p__Lentisphaerae; c__Lentisphaerae (class)       7    0.0%    0.0%    0.0%    0.0%    0.0%    0.0%    0.0%    0.0%    0.0%    0.0%    0.0%    0.0%    0.0%    0.0%    0.0%    0.0%    0.0%    0.0%    0.0%    0.0%    0.0%    0.0%    0.0%    0.0%    0.0%    0.0%    0.0%    0.0%    0.0%    0.0%    0.0%    0.0%    0.0%    0.0%    0.0%    0.0%    0.0%    0.0%    0.0%    0.0%    0.0%    0.0%    0.0%    0.0%    0.0%    0.0%    0.0%    0.0%    0.0%    0.0%    0.0%    0.0%    0.0%    0.0%    0.0%    0.0%    0.0%    0.0%    0.0%    0.0%    0.0%    0.0%    0.0%    0.0%    0.0%    0.0%    0.0%    0.0%    0.0%    0.0%    0.0%    0.0%    0.0%    0.0%    0.0%    0.0%    0.0%    0.0%    0.0%    0.0%    0.0%    0.0%    0.0%    0.0%    0.0%    0.0%    0.0%    0.0%    0.0%    0.0%    0.0%    0.0%    0.0%    0.0%    0.0%    0.0%    0.0%    0.0%    0.0%    0.0%    0.0%    0.0%    0.0%    0.0%    0.0%    0.0%    0.0%    0.0%    0.0%    0.0%    0.0%    0.0%    0.0%    0.0%    0.0%    0.0%    0.0%    0.0%    0.0%    0.0%    0.0%    0.0%    0.0%    0.0%    0.0%    0.0%    0.0%    0.0%    0.0%    0.0%    0.0%    0.0%    0.0%    0.0%    0.0%    0.0%    0.0%    0.0%    0.0%    0.0%    0.0%    0.0%    0.0%    0.0%    0.0%    0.0%    0.0%    0.0%    0.0%    0.0%    0.0%    0.0%    0.0%    0.0%    0.0%    0.0%    0.0%    0.0%    0.0%    0.0%    0.0%    0.0%    0.0%    0.0%    0.0%    0.0%    0.0%    0.0%    0.0%    0.0%    0.0%    0.0%    0.0%    0.0%    0.0%    0.0%    0.0%    0.0%    0.0%    0.0%    0.0%    0.0%    0.0%    0.0%    0.0%    0.0%    0.0%    0.0%    0.0%    0.0%    0.0%    0.0%    0.0%    0.0%    0.0%    0.0%    0.0%    0.0%    0.0%    0.0%    0.0%    0.0%    0.0%    0.0%    0.0%    0.0%    0.0%    0.0%    0.0%    0.0%    0.0%    0.0%    0.0%    0.0%    0.0%    0.0%    0.0%    0.0%    0.0%    0.0%    0.0%    0.0%    0.0%    0.0%    0.0%    0.0%    0.0%    0.0%    0.0%    0.0%    0.0%    0.0%    0.0%    0.0%    0.0%    0.0%    0.0%    0.0%    0.0%    0.0%    0.0%    0.0%    0.0%    0.0%    0.0%    0.0%    0.0%    0.0%    0.0%    0.0%    0.0%    0.0%    0.0%    0.0%    0.0%    0.0%    0.0%    0.0%    0.0%    0.0%    0.0%    0.0%    0.0%    0.0%    0.0%    0.0%    0.0%    0.0%    0.0%    0.0%    0.0%    0.0%    0.0%    0.0%    0.0%    0.0%    0.0%    0.0%    0.0%    0.0%    0.0%    0.0%    0.0%    0.0%    0.0%    0.0%    0.0%    0.0%    0.0%    0.0%    0.0%    0.0%    0.0%    0.0%    0.0%    0.0%    0.0%    0.0%    0.0%    0.0%    0.0%    0.0%    0.0%    0.0%    0.0%    0.0%    0.0%    0.0%    0.0%    0.0%    0.0%    0.0%    0.0%    0.0%    0.0%    0.0%    0.0%    0.0%    0.0%    0.0%    0.0%    0.0%    0.0%    0.0%    0.0%    0.0%    0.0%    0.0%    0.0%    0.0%    0.0%    0.0%    0.0%    0.0%    0.0%    0.0%    0.0%    0.0%    0.0%    0.0%    0.0%    0.0%    0.0%    0.0%    0.0%    0.0%    0.0%    0.0%    0.0%    0.0%    0.0%    0.0%    0.0%    0.0%    0.0%    0.0%    0.0%    0.0%        k__Bacteria;p__MVP-15; c__       1    0.0%    0.0%    0.0%    0.0%    0.0%    0.0%    0.0%    0.0%    0.0%    0.0%    0.0%    0.0%    0.0%    0.0%    0.0%    0.0%    0.0%    0.0%    0.0%    0.0%    0.0%    0.0%    0.0%    0.0%    0.0%    0.0%    0.0%    0.0%    0.0%    0.0%    0.0%    0.0%    0.0%    0.0%    0.0%    0.0%    0.0%    0.0%    0.0%    0.0%    0.0%    0.0%    0.0%    0.0%    0.0%    0.0%    0.0%    0.0%    0.0%    0.0%    0.0%    0.0%    0.0%    0.0%    0.0%    0.0%    0.0%    0.0%    0.0%    0.0%    0.0%    0.0%    0.0%    0.0%    0.0%    0.0%    0.0%    0.0%    0.0%    0.0%    0.0%    0.0%    0.0%    0.0%    0.0%    0.0%    0.0%    0.0%    0.0%    0.0%    0.0%    0.0%    0.0%    0.0%    0.0%    0.0%    0.0%    0.0%    0.0%    0.0%    0.0%    0.0%    0.0%    0.0%    0.0%    0.0%    0.0%    0.0%    0.0%    0.0%    0.0%    0.0%    0.0%    0.0%    0.0%    0.0%    0.0%    0.0%    0.0%    0.0%    0.0%    0.0%    0.0%    0.0%    0.0%    0.0%    0.0%    0.0%    0.0%    0.0%    0.0%    0.0%    0.0%    0.0%    0.0%    0.0%    0.0%    0.0%    0.0%    0.0%    0.0%    0.0%    0.0%    0.0%    0.0%    0.0%    0.0%    0.0%    0.0%    0.0%    0.0%    0.0%    0.0%    0.0%    0.0%    0.0%    0.0%    0.0%    0.0%    0.0%    0.0%    0.0%    0.0%    0.0%    0.0%    0.0%    0.0%    0.0%    0.0%    0.0%    0.0%    0.0%    0.0%    0.0%    0.0%    0.0%    0.0%    0.0%    0.0%    0.0%    0.0%    0.0%    0.0%    0.0%    0.0%    0.0%    0.0%    0.0%    0.0%    0.0%    0.0%    0.0%    0.0%    0.0%    0.0%    0.0%    0.0%    0.0%    0.0%    0.0%    0.0%    0.0%    0.0%    0.0%    0.0%    0.0%    0.0%    0.0%    0.0%    0.0%    0.0%    0.0%    0.0%    0.0%    0.0%    0.0%    0.0%    0.0%    0.0%    0.0%    0.0%    0.0%    0.0%    0.0%    0.0%    0.0%    0.0%    0.0%    0.0%    0.0%    0.0%    0.0%    0.0%    0.0%    0.0%    0.0%    0.0%    0.0%    0.0%    0.0%    0.0%    0.0%    0.0%    0.0%    0.0%    0.0%    0.0%    0.0%    0.0%    0.0%    0.0%    0.0%    0.0%    0.0%    0.0%    0.0%    0.0%    0.0%    0.0%    0.0%    0.0%    0.0%    0.0%    0.0%    0.0%    0.0%    0.0%    0.0%    0.0%    0.0%    0.0%    0.0%    0.0%    0.0%    0.0%    0.0%    0.0%    0.0%    0.0%    0.0%    0.0%    0.0%    0.0%    0.0%    0.0%    0.0%    0.0%    0.0%    0.0%    0.0%    0.0%    0.0%    0.0%    0.0%    0.0%    0.0%    0.0%    0.0%    0.0%    0.0%    0.0%    0.0%    0.0%    0.0%    0.0%    0.0%    0.0%    0.0%    0.0%    0.0%    0.0%    0.0%    0.0%    0.0%    0.0%    0.0%    0.0%    0.0%    0.0%    0.0%    0.0%    0.0%    0.0%    0.0%    0.0%    0.0%    0.0%    0.0%    0.0%    0.0%    0.0%    0.0%    0.0%    0.0%    0.0%    0.0%    0.0%    0.0%    0.0%    0.0%    0.0%    0.0%    0.0%    0.0%    0.0%    0.0%    0.0%    0.0%    0.0%    0.0%    0.0%    0.0%    0.0%    0.0%    0.0%    0.0%    0.0%    0.0%    0.0%    0.0%    0.0%    0.0%    0.0%    0.0%    0.0%    0.0%    0.0%    0.0%        k__Bacteria;p__NC10; c__       6    0.0%    0.0%    0.0%    0.0%    0.0%    0.0%    0.0%    0.0%    0.0%    0.0%    0.0%    0.0%    0.0%    0.0%    0.0%    0.0%    0.0%    0.0%    0.0%    0.0%    0.0%    0.0%    0.0%    0.0%    0.0%    0.0%    0.0%    0.0%    0.0%    0.0%    0.0%    0.0%    0.0%    0.0%    0.0%    0.0%    0.0%    0.0%    0.0%    0.0%    0.0%    0.0%    0.0%    0.0%    0.0%    0.0%    0.0%    0.0%    0.0%    0.0%    0.0%    0.0%    0.0%    0.0%    0.0%    0.0%    0.0%    0.0%    0.0%    0.0%    0.0%    0.0%    0.0%    0.0%    0.0%    0.0%    0.0%    0.0%    0.0%    0.0%    0.0%    0.0%    0.0%    0.0%    0.0%    0.0%    0.0%    0.0%    0.0%    0.0%    0.0%    0.0%    0.0%    0.0%    0.0%    0.0%    0.0%    0.0%    0.0%    0.0%    0.0%    0.0%    0.0%    0.0%    0.0%    0.0%    0.0%    0.0%    0.0%    0.0%    0.0%    0.0%    0.0%    0.0%    0.0%    0.0%    0.0%    0.0%    0.0%    0.0%    0.0%    0.0%    0.0%    0.0%    0.0%    0.0%    0.0%    0.0%    0.0%    0.0%    0.0%    0.0%    0.0%    0.0%    0.0%    0.0%    0.0%    0.0%    0.0%    0.0%    0.0%    0.0%    0.0%    0.0%    0.0%    0.0%    0.0%    0.0%    0.0%    0.0%    0.0%    0.0%    0.0%    0.0%    0.0%    0.0%    0.0%    0.0%    0.0%    0.0%    0.0%    0.0%    0.0%    0.0%    0.0%    0.0%    0.0%    0.0%    0.0%    0.0%    0.0%    0.0%    0.0%    0.0%    0.0%    0.0%    0.0%    0.0%    0.0%    0.0%    0.0%    0.0%    0.0%    0.0%    0.0%    0.0%    0.0%    0.0%    0.0%    0.0%    0.0%    0.0%    0.0%    0.0%    0.0%    0.0%    0.0%    0.0%    0.0%    0.0%    0.0%    0.0%    0.0%    0.0%    0.0%    0.0%    0.0%    0.0%    0.0%    0.0%    0.0%    0.0%    0.0%    0.0%    0.0%    0.0%    0.0%    0.0%    0.0%    0.0%    0.0%    0.0%    0.0%    0.0%    0.0%    0.0%    0.0%    0.0%    0.0%    0.0%    0.0%    0.0%    0.0%    0.0%    0.0%    0.0%    0.0%    0.0%    0.0%    0.0%    0.0%    0.0%    0.0%    0.0%    0.0%    0.0%    0.0%    0.0%    0.0%    0.0%    0.0%    0.0%    0.0%    0.0%    0.0%    0.0%    0.0%    0.0%    0.0%    0.0%    0.0%    0.0%    0.0%    0.0%    0.0%    0.0%    0.0%    0.0%    0.0%    0.0%    0.0%    0.0%    0.0%    0.0%    0.0%    0.0%    0.0%    0.0%    0.0%    0.0%    0.0%    0.0%    0.0%    0.0%    0.0%    0.0%    0.0%    0.0%    0.0%    0.0%    0.0%    0.0%    0.0%    0.0%    0.0%    0.0%    0.0%    0.0%    0.0%    0.0%    0.0%    0.0%    0.0%    0.0%    0.0%    0.0%    0.0%    0.0%    0.0%    0.0%    0.0%    0.0%    0.0%    0.0%    0.0%    0.0%    0.0%    0.0%    0.0%    0.0%    0.0%    0.0%    0.0%    0.0%    0.0%    0.0%    0.0%    0.0%    0.0%    0.0%    0.0%    0.0%    0.0%    0.0%    0.0%    0.0%    0.0%    0.0%    0.0%    0.0%    0.0%    0.0%    0.0%    0.0%    0.0%    0.0%    0.0%    0.0%    0.0%    0.0%    0.0%    0.0%    0.0%    0.0%    0.0%    0.0%    0.0%    0.0%    0.0%    0.0%    0.0%    0.0%    0.0%    0.0%    0.0%    0.0%    0.0%    0.0%        k__Bacteria;p__NKB19; c__      24    0.0%    0.0%    0.0%    0.0%    0.0%    0.0%    0.0%    0.0%    0.0%    0.0%    0.0%    0.0%    0.0%    0.0%    0.0%    0.0%    0.0%    0.0%    0.0%    0.0%    0.0%    0.0%    0.0%    0.0%    0.0%    0.0%    0.0%    0.0%    0.0%    0.0%    0.0%    0.0%    0.0%    0.0%    0.0%    0.0%    0.0%    0.0%    0.0%    0.0%    0.0%    0.0%    0.0%    0.0%    0.0%    0.0%    0.0%    0.0%    0.0%    0.0%    0.0%    0.0%    0.0%    0.0%    0.0%    0.0%    0.0%    0.0%    0.0%    0.0%    0.0%    0.0%    0.0%    0.0%    0.0%    0.0%    0.0%    0.0%    0.0%    0.0%    0.0%    0.0%    0.0%    0.0%    0.0%    0.0%    0.0%    0.0%    0.0%    0.0%    0.0%    0.0%    0.0%    0.0%    0.0%    0.0%    0.0%    0.0%    0.0%    0.0%    0.0%    0.0%    0.0%    0.0%    0.0%    0.0%    0.0%    0.0%    0.0%    0.0%    0.0%    0.0%    0.0%    0.0%    0.0%    0.0%    0.0%    0.0%    0.0%    0.0%    0.0%    0.0%    0.0%    0.0%    0.0%    0.0%    0.0%    0.0%    0.0%    0.0%    0.0%    0.0%    0.0%    0.0%    0.0%    0.0%    0.0%    0.0%    0.0%    0.0%    0.0%    0.0%    0.0%    0.0%    0.0%    0.0%    0.0%    0.0%    0.0%    0.0%    0.0%    0.0%    0.0%    0.0%    0.0%    0.0%    0.0%    0.0%    0.0%    0.0%    0.0%    0.0%    0.0%    0.0%    0.0%    0.0%    0.0%    0.0%    0.0%    0.0%    0.0%    0.0%    0.0%    0.0%    0.0%    0.0%    0.0%    0.0%    0.0%    0.0%    0.0%    0.0%    0.0%    0.0%    0.0%    0.0%    0.0%    0.0%    0.0%    0.0%    0.0%    0.0%    0.0%    0.0%    0.0%    0.0%    0.0%    0.0%    0.0%    0.0%    0.0%    0.0%    0.0%    0.0%    0.0%    0.0%    0.0%    0.0%    0.0%    0.0%    0.0%    0.0%    0.0%    0.0%    0.0%    0.0%    0.0%    0.0%    0.0%    0.0%    0.0%    0.0%    0.0%    0.0%    0.0%    0.0%    0.0%    0.0%    0.0%    0.0%    0.0%    0.0%    0.0%    0.0%    0.0%    0.0%    0.0%    0.0%    0.0%    0.0%    0.0%    0.0%    0.0%    0.0%    0.0%    0.0%    0.0%    0.0%    0.0%    0.0%    0.0%    0.0%    0.0%    0.0%    0.0%    0.0%    0.0%    0.0%    0.0%    0.0%    0.0%    0.0%    0.0%    0.0%    0.0%    0.0%    0.0%    0.0%    0.0%    0.0%    0.0%    0.0%    0.0%    0.0%    0.0%    0.0%    0.0%    0.0%    0.0%    0.0%    0.0%    0.0%    0.0%    0.0%    0.0%    0.0%    0.0%    0.0%    0.0%    0.0%    0.0%    0.0%    0.0%    0.0%    0.0%    0.0%    0.0%    0.0%    0.0%    0.0%    0.0%    0.0%    0.0%    0.0%    0.0%    0.0%    0.0%    0.0%    0.0%    0.0%    0.0%    0.0%    0.0%    0.0%    0.0%    0.0%    0.0%    0.0%    0.0%    0.0%    0.0%    0.0%    0.0%    0.0%    0.0%    0.0%    0.0%    0.0%    0.0%    0.0%    0.0%    0.0%    0.0%    0.0%    0.0%    0.0%    0.0%    0.0%    0.0%    0.0%    0.0%    0.0%    0.0%    0.0%    0.0%    0.0%    0.0%    0.0%    0.0%    0.0%    0.0%    0.0%    0.0%    0.0%    0.0%    0.0%    0.0%    0.0%    0.0%    0.0%    0.0%    0.0%    0.0%    0.0%    0.0%    0.0%    0.0%    0.0%        k__Bacteria;p__Nitrospirae; c__Nitrospira (class)     714    0.0%    0.0%    0.0%    0.0%    0.0%    0.0%    0.0%    0.0%    0.0%    0.0%    0.0%    0.0%    0.0%    0.0%    0.0%    0.0%    0.0%    0.0%    0.0%    0.0%    0.0%    0.0%    0.0%    0.0%    0.0%    0.0%    0.0%    0.0%    0.0%    0.0%    0.0%    0.0%    0.0%    0.0%    0.0%    0.0%    0.0%    0.0%    0.0%    0.0%    0.0%    0.0%    0.0%    0.0%    0.0%    0.0%    0.0%    0.0%    0.0%    0.0%    0.0%    0.0%    0.0%    0.0%    0.0%    0.0%    0.0%    0.0%    0.0%    0.0%    0.0%    0.0%    0.0%    0.0%    0.0%    0.0%    0.0%    0.0%    0.0%    0.0%    0.0%    0.0%    0.0%    0.0%    0.0%    0.0%    0.0%    0.0%    0.0%    0.0%    0.0%    0.0%    0.0%    0.0%    0.0%    0.0%    0.0%    0.0%    0.0%    0.0%    0.0%    0.0%    0.0%    0.0%    0.0%    0.0%    0.0%    0.0%    0.0%    0.0%    0.0%    0.0%    0.0%    0.0%    0.0%    0.0%    0.0%    0.0%    0.0%    0.0%    0.0%    0.0%    0.0%    0.0%    0.0%    0.0%    0.0%    0.0%    0.0%    0.0%    0.0%    0.0%    0.0%    0.0%    0.0%    0.0%    0.0%    0.0%    0.0%    0.0%    0.0%    0.0%    0.0%    0.0%    0.0%    0.0%    0.0%    0.0%    0.0%    0.0%    0.0%    0.0%    0.0%    0.0%    0.0%    0.0%    0.0%    0.0%    0.0%    0.0%    0.0%    0.0%    0.0%    0.0%    0.0%    0.0%    0.0%    0.0%    0.0%    0.0%    0.0%    0.0%    0.0%    0.0%    0.0%    0.0%    0.0%    0.0%    0.0%    0.0%    0.0%    0.0%    0.0%    0.0%    0.0%    0.0%    0.0%    0.0%    0.0%    0.0%    0.0%    0.0%    0.0%    0.0%    0.0%    0.0%    0.0%    0.0%    0.0%    0.0%    0.0%    0.0%    0.0%    0.0%    0.0%    0.0%    0.0%    0.0%    0.0%    0.0%    0.0%    0.0%    0.0%    0.0%    0.0%    0.0%    0.0%    0.0%    0.0%    0.0%    0.0%    0.0%    0.0%    0.0%    0.0%    0.0%    0.0%    0.0%    0.0%    0.0%    0.0%    0.0%    0.0%    0.0%    0.0%    0.0%    0.0%    0.0%    0.0%    0.0%    0.0%    0.0%    0.0%    0.0%    0.0%    0.0%    0.0%    0.0%    0.0%    0.0%    0.0%    0.0%    0.0%    0.0%    0.0%    0.0%    0.0%    0.0%    0.0%    0.0%    0.0%    0.0%    0.0%    0.0%    0.0%    0.0%    0.0%    0.0%    0.0%    0.0%    0.0%    0.0%    0.0%    0.0%    0.0%    0.0%    0.0%    0.0%    0.0%    0.0%    0.0%    0.0%    0.1%    0.0%    0.0%    0.0%    0.0%    0.0%    0.0%    0.0%    0.0%    0.0%    0.0%    0.0%    0.0%    0.0%    0.0%    0.1%    0.0%    0.0%    0.0%    0.0%    0.0%    0.0%    0.0%    0.0%    0.0%    0.0%    0.0%    0.0%    0.0%    0.0%    0.0%    0.0%    0.0%    0.0%    0.0%    0.0%    0.0%    0.0%    0.0%    0.0%    0.0%    0.0%    0.0%    0.0%    0.0%    0.0%    0.0%    0.0%    0.0%    0.0%    0.0%    0.0%    0.0%    0.0%    0.0%    0.0%    0.0%    0.0%    0.0%    0.0%    0.0%    0.0%    0.0%    0.0%    0.0%    0.0%    0.0%    0.0%    0.0%    0.0%    0.0%    0.0%    0.0%    0.0%    0.0%    0.0%    0.0%    0.0%    0.0%    0.0%    0.0%    0.0%    0.0%    0.0%    0.0%    0.0%        k__Bacteria;p__OP10; c__       1    0.0%    0.0%    0.0%    0.0%    0.0%    0.0%    0.0%    0.0%    0.0%    0.0%    0.0%    0.0%    0.0%    0.0%    0.0%    0.0%    0.0%    0.0%    0.0%    0.0%    0.0%    0.0%    0.0%    0.0%    0.0%    0.0%    0.0%    0.0%    0.0%    0.0%    0.0%    0.0%    0.0%    0.0%    0.0%    0.0%    0.0%    0.0%    0.0%    0.0%    0.0%    0.0%    0.0%    0.0%    0.0%    0.0%    0.0%    0.0%    0.0%    0.0%    0.0%    0.0%    0.0%    0.0%    0.0%    0.0%    0.0%    0.0%    0.0%    0.0%    0.0%    0.0%    0.0%    0.0%    0.0%    0.0%    0.0%    0.0%    0.0%    0.0%    0.0%    0.0%    0.0%    0.0%    0.0%    0.0%    0.0%    0.0%    0.0%    0.0%    0.0%    0.0%    0.0%    0.0%    0.0%    0.0%    0.0%    0.0%    0.0%    0.0%    0.0%    0.0%    0.0%    0.0%    0.0%    0.0%    0.0%    0.0%    0.0%    0.0%    0.0%    0.0%    0.0%    0.0%    0.0%    0.0%    0.0%    0.0%    0.0%    0.0%    0.0%    0.0%    0.0%    0.0%    0.0%    0.0%    0.0%    0.0%    0.0%    0.0%    0.0%    0.0%    0.0%    0.0%    0.0%    0.0%    0.0%    0.0%    0.0%    0.0%    0.0%    0.0%    0.0%    0.0%    0.0%    0.0%    0.0%    0.0%    0.0%    0.0%    0.0%    0.0%    0.0%    0.0%    0.0%    0.0%    0.0%    0.0%    0.0%    0.0%    0.0%    0.0%    0.0%    0.0%    0.0%    0.0%    0.0%    0.0%    0.0%    0.0%    0.0%    0.0%    0.0%    0.0%    0.0%    0.0%    0.0%    0.0%    0.0%    0.0%    0.0%    0.0%    0.0%    0.0%    0.0%    0.0%    0.0%    0.0%    0.0%    0.0%    0.0%    0.0%    0.0%    0.0%    0.0%    0.0%    0.0%    0.0%    0.0%    0.0%    0.0%    0.0%    0.0%    0.0%    0.0%    0.0%    0.0%    0.0%    0.0%    0.0%    0.0%    0.0%    0.0%    0.0%    0.0%    0.0%    0.0%    0.0%    0.0%    0.0%    0.0%    0.0%    0.0%    0.0%    0.0%    0.0%    0.0%    0.0%    0.0%    0.0%    0.0%    0.0%    0.0%    0.0%    0.0%    0.0%    0.0%    0.0%    0.0%    0.0%    0.0%    0.0%    0.0%    0.0%    0.0%    0.0%    0.0%    0.0%    0.0%    0.0%    0.0%    0.0%    0.0%    0.0%    0.0%    0.0%    0.0%    0.0%    0.0%    0.0%    0.0%    0.0%    0.0%    0.0%    0.0%    0.0%    0.0%    0.0%    0.0%    0.0%    0.0%    0.0%    0.0%    0.0%    0.0%    0.0%    0.0%    0.0%    0.0%    0.0%    0.0%    0.0%    0.0%    0.0%    0.0%    0.0%    0.0%    0.0%    0.0%    0.0%    0.0%    0.0%    0.0%    0.0%    0.0%    0.0%    0.0%    0.0%    0.0%    0.0%    0.0%    0.0%    0.0%    0.0%    0.0%    0.0%    0.0%    0.0%    0.0%    0.0%    0.0%    0.0%    0.0%    0.0%    0.0%    0.0%    0.0%    0.0%    0.0%    0.0%    0.0%    0.0%    0.0%    0.0%    0.0%    0.0%    0.0%    0.0%    0.0%    0.0%    0.0%    0.0%    0.0%    0.0%    0.0%    0.0%    0.0%    0.0%    0.0%    0.0%    0.0%    0.0%    0.0%    0.0%    0.0%    0.0%    0.0%    0.0%    0.0%    0.0%    0.0%    0.0%    0.0%    0.0%    0.0%    0.0%    0.0%    0.0%    0.0%    0.0%    0.0%    0.0%    0.0%    0.0%    0.0%    0.0%    0.0%    0.0%        k__Bacteria;p__OP10; c__5B-18      41    0.0%    0.0%    0.0%    0.0%    0.0%    0.0%    0.0%    0.0%    0.0%    0.0%    0.0%    0.0%    0.0%    0.0%    0.0%    0.0%    0.0%    0.0%    0.0%    0.0%    0.0%    0.0%    0.0%    0.0%    0.0%    0.0%    0.0%    0.0%    0.0%    0.0%    0.0%    0.0%    0.0%    0.0%    0.0%    0.0%    0.0%    0.0%    0.0%    0.0%    0.0%    0.0%    0.0%    0.0%    0.0%    0.0%    0.0%    0.0%    0.0%    0.0%    0.0%    0.0%    0.0%    0.0%    0.0%    0.0%    0.0%    0.0%    0.0%    0.0%    0.0%    0.0%    0.0%    0.0%    0.0%    0.0%    0.0%    0.0%    0.0%    0.0%    0.0%    0.0%    0.0%    0.0%    0.0%    0.0%    0.0%    0.0%    0.0%    0.0%    0.0%    0.0%    0.0%    0.0%    0.0%    0.0%    0.0%    0.0%    0.0%    0.0%    0.0%    0.0%    0.0%    0.0%    0.0%    0.0%    0.0%    0.0%    0.0%    0.0%    0.0%    0.0%    0.0%    0.0%    0.0%    0.0%    0.0%    0.0%    0.0%    0.0%    0.0%    0.0%    0.0%    0.0%    0.0%    0.0%    0.0%    0.0%    0.0%    0.0%    0.0%    0.0%    0.0%    0.0%    0.0%    0.0%    0.0%    0.0%    0.0%    0.0%    0.0%    0.0%    0.0%    0.0%    0.0%    0.0%    0.0%    0.0%    0.0%    0.0%    0.0%    0.0%    0.0%    0.0%    0.0%    0.0%    0.0%    0.0%    0.0%    0.0%    0.0%    0.0%    0.0%    0.0%    0.0%    0.0%    0.0%    0.0%    0.0%    0.0%    0.0%    0.0%    0.0%    0.0%    0.0%    0.0%    0.0%    0.0%    0.0%    0.0%    0.0%    0.0%    0.0%    0.0%    0.0%    0.0%    0.0%    0.0%    0.0%    0.0%    0.0%    0.0%    0.0%    0.0%    0.0%    0.0%    0.0%    0.0%    0.0%    0.0%    0.0%    0.0%    0.0%    0.0%    0.0%    0.0%    0.0%    0.0%    0.0%    0.0%    0.0%    0.0%    0.0%    0.0%    0.0%    0.0%    0.0%    0.0%    0.0%    0.0%    0.0%    0.0%    0.0%    0.0%    0.0%    0.0%    0.0%    0.0%    0.0%    0.0%    0.0%    0.0%    0.0%    0.0%    0.0%    0.0%    0.0%    0.0%    0.0%    0.0%    0.0%    0.0%    0.0%    0.0%    0.0%    0.0%    0.0%    0.0%    0.0%    0.0%    0.0%    0.0%    0.0%    0.0%    0.0%    0.0%    0.0%    0.0%    0.0%    0.0%    0.0%    0.0%    0.0%    0.0%    0.0%    0.0%    0.0%    0.0%    0.0%    0.0%    0.0%    0.0%    0.0%    0.0%    0.0%    0.0%    0.0%    0.0%    0.0%    0.0%    0.0%    0.0%    0.0%    0.0%    0.0%    0.0%    0.0%    0.0%    0.0%    0.0%    0.0%    0.0%    0.0%    0.0%    0.0%    0.0%    0.0%    0.0%    0.0%    0.0%    0.0%    0.0%    0.0%    0.0%    0.0%    0.0%    0.0%    0.0%    0.0%    0.0%    0.0%    0.0%    0.0%    0.0%    0.0%    0.0%    0.0%    0.0%    0.0%    0.0%    0.0%    0.0%    0.0%    0.0%    0.0%    0.0%    0.0%    0.0%    0.0%    0.0%    0.0%    0.0%    0.0%    0.0%    0.0%    0.0%    0.0%    0.0%    0.0%    0.0%    0.0%    0.0%    0.0%    0.0%    0.0%    0.0%    0.0%    0.0%    0.0%    0.0%    0.0%    0.0%    0.0%    0.0%    0.0%    0.0%    0.0%    0.0%    0.0%    0.0%    0.0%    0.0%    0.0%    0.0%    0.0%    0.0%    0.0%    0.0%        k__Bacteria;p__OP10; c__CH21      27    0.0%    0.0%    0.0%    0.0%    0.0%    0.0%    0.0%    0.0%    0.0%    0.0%    0.0%    0.0%    0.0%    0.0%    0.0%    0.0%    0.0%    0.0%    0.0%    0.0%    0.0%    0.0%    0.0%    0.0%    0.0%    0.0%    0.0%    0.0%    0.0%    0.0%    0.0%    0.0%    0.0%    0.0%    0.0%    0.0%    0.0%    0.0%    0.0%    0.0%    0.0%    0.0%    0.0%    0.0%    0.0%    0.0%    0.0%    0.0%    0.0%    0.0%    0.0%    0.0%    0.0%    0.0%    0.0%    0.0%    0.0%    0.0%    0.0%    0.0%    0.0%    0.0%    0.0%    0.0%    0.0%    0.0%    0.0%    0.0%    0.0%    0.0%    0.0%    0.0%    0.0%    0.0%    0.0%    0.0%    0.0%    0.0%    0.0%    0.0%    0.0%    0.0%    0.0%    0.0%    0.0%    0.0%    0.0%    0.0%    0.0%    0.0%    0.0%    0.0%    0.0%    0.0%    0.0%    0.0%    0.0%    0.0%    0.0%    0.0%    0.0%    0.0%    0.0%    0.0%    0.0%    0.0%    0.0%    0.0%    0.0%    0.0%    0.0%    0.0%    0.0%    0.0%    0.0%    0.0%    0.0%    0.0%    0.0%    0.0%    0.0%    0.0%    0.0%    0.0%    0.0%    0.0%    0.0%    0.0%    0.0%    0.0%    0.0%    0.0%    0.0%    0.0%    0.0%    0.0%    0.0%    0.0%    0.0%    0.0%    0.0%    0.0%    0.0%    0.0%    0.0%    0.0%    0.0%    0.0%    0.0%    0.0%    0.0%    0.0%    0.0%    0.0%    0.0%    0.0%    0.0%    0.0%    0.0%    0.0%    0.0%    0.0%    0.0%    0.0%    0.0%    0.0%    0.0%    0.0%    0.0%    0.0%    0.0%    0.0%    0.0%    0.0%    0.0%    0.0%    0.0%    0.0%    0.0%    0.0%    0.0%    0.0%    0.0%    0.0%    0.0%    0.0%    0.0%    0.0%    0.0%    0.0%    0.0%    0.0%    0.0%    0.0%    0.0%    0.0%    0.0%    0.0%    0.0%    0.0%    0.0%    0.0%    0.0%    0.0%    0.0%    0.0%    0.0%    0.0%    0.0%    0.0%    0.0%    0.0%    0.0%    0.0%    0.0%    0.0%    0.0%    0.0%    0.0%    0.0%    0.0%    0.0%    0.0%    0.0%    0.0%    0.0%    0.0%    0.0%    0.0%    0.0%    0.0%    0.0%    0.0%    0.0%    0.0%    0.0%    0.0%    0.0%    0.0%    0.0%    0.0%    0.0%    0.0%    0.0%    0.0%    0.0%    0.0%    0.0%    0.0%    0.0%    0.0%    0.0%    0.0%    0.0%    0.0%    0.0%    0.0%    0.0%    0.0%    0.0%    0.0%    0.0%    0.0%    0.0%    0.0%    0.0%    0.0%    0.0%    0.0%    0.0%    0.0%    0.0%    0.0%    0.0%    0.0%    0.0%    0.0%    0.0%    0.0%    0.0%    0.0%    0.0%    0.0%    0.0%    0.0%    0.0%    0.0%    0.0%    0.0%    0.0%    0.0%    0.0%    0.0%    0.0%    0.0%    0.0%    0.0%    0.0%    0.0%    0.0%    0.0%    0.0%    0.0%    0.0%    0.0%    0.0%    0.0%    0.0%    0.0%    0.0%    0.0%    0.0%    0.0%    0.0%    0.0%    0.0%    0.0%    0.0%    0.0%    0.0%    0.0%    0.0%    0.0%    0.0%    0.0%    0.0%    0.0%    0.0%    0.0%    0.0%    0.0%    0.0%    0.0%    0.0%    0.0%    0.0%    0.0%    0.0%    0.0%    0.0%    0.0%    0.0%    0.0%    0.0%    0.0%    0.0%    0.0%    0.0%    0.0%    0.0%    0.0%    0.0%    0.0%    0.0%    0.0%    0.0%    0.0%    0.0%        k__Bacteria;p__OP10; c__CL500-48     299    0.0%    0.0%    0.0%    0.0%    0.0%    0.0%    0.0%    0.0%    0.0%    0.0%    0.0%    0.0%    0.0%    0.0%    0.0%    0.0%    0.0%    0.0%    0.0%    0.0%    0.0%    0.0%    0.0%    0.0%    0.0%    0.0%    0.0%    0.0%    0.0%    0.0%    0.0%    0.0%    0.0%    0.0%    0.0%    0.0%    0.0%    0.0%    0.0%    0.0%    0.0%    0.0%    0.0%    0.0%    0.0%    0.0%    0.0%    0.0%    0.0%    0.0%    0.0%    0.0%    0.0%    0.0%    0.0%    0.0%    0.0%    0.0%    0.0%    0.0%    0.0%    0.0%    0.0%    0.0%    0.0%    0.0%    0.0%    0.0%    0.0%    0.0%    0.0%    0.0%    0.0%    0.0%    0.0%    0.0%    0.0%    0.0%    0.0%    0.0%    0.0%    0.0%    0.0%    0.0%    0.0%    0.0%    0.0%    0.0%    0.0%    0.0%    0.0%    0.0%    0.0%    0.0%    0.0%    0.0%    0.0%    0.0%    0.0%    0.0%    0.0%    0.0%    0.0%    0.0%    0.0%    0.0%    0.0%    0.0%    0.0%    0.0%    0.0%    0.0%    0.0%    0.0%    0.0%    0.0%    0.0%    0.0%    0.0%    0.0%    0.0%    0.0%    0.0%    0.0%    0.0%    0.0%    0.0%    0.0%    0.0%    0.0%    0.0%    0.0%    0.0%    0.0%    0.0%    0.0%    0.0%    0.0%    0.0%    0.0%    0.0%    0.0%    0.0%    0.0%    0.0%    0.0%    0.0%    0.0%    0.0%    0.0%    0.0%    0.0%    0.0%    0.0%    0.0%    0.0%    0.0%    0.0%    0.0%    0.0%    0.0%    0.0%    0.0%    0.0%    0.0%    0.0%    0.0%    0.0%    0.0%    0.0%    0.0%    0.0%    0.0%    0.0%    0.0%    0.0%    0.0%    0.0%    0.0%    0.0%    0.0%    0.0%    0.0%    0.0%    0.0%    0.0%    0.0%    0.0%    0.0%    0.0%    0.0%    0.0%    0.0%    0.0%    0.0%    0.0%    0.0%    0.0%    0.0%    0.0%    0.0%    0.0%    0.0%    0.0%    0.0%    0.0%    0.0%    0.0%    0.0%    0.0%    0.0%    0.0%    0.0%    0.0%    0.0%    0.0%    0.0%    0.0%    0.0%    0.0%    0.0%    0.0%    0.0%    0.0%    0.0%    0.0%    0.0%    0.0%    0.0%    0.0%    0.0%    0.0%    0.0%    0.0%    0.0%    0.0%    0.0%    0.0%    0.0%    0.0%    0.0%    0.0%    0.0%    0.0%    0.0%    0.0%    0.0%    0.0%    0.0%    0.0%    0.0%    0.0%    0.0%    0.0%    0.0%    0.0%    0.0%    0.0%    0.0%    0.0%    0.0%    0.0%    0.0%    0.0%    0.0%    0.0%    0.0%    0.0%    0.0%    0.0%    0.0%    0.0%    0.0%    0.0%    0.0%    0.0%    0.0%    0.0%    0.0%    0.0%    0.0%    0.0%    0.0%    0.0%    0.0%    0.0%    0.0%    0.0%    0.0%    0.0%    0.0%    0.0%    0.0%    0.0%    0.0%    0.0%    0.0%    0.0%    0.0%    0.0%    0.0%    0.0%    0.0%    0.0%    0.0%    0.0%    0.0%    0.0%    0.0%    0.0%    0.0%    0.0%    0.0%    0.0%    0.0%    0.0%    0.0%    0.0%    0.0%    0.0%    0.0%    0.0%    0.0%    0.0%    0.0%    0.0%    0.0%    0.0%    0.0%    0.0%    0.0%    0.0%    0.0%    0.0%    0.0%    0.0%    0.0%    0.0%    0.0%    0.0%    0.0%    0.0%    0.0%    0.0%    0.0%    0.0%    0.0%    0.0%    0.0%    0.0%    0.0%    0.0%    0.0%    0.0%    0.0%    0.0%    0.0%    0.0%        k__Bacteria;p__OP10; c__OS-L      89    0.0%    0.0%    0.0%    0.0%    0.0%    0.0%    0.0%    0.0%    0.0%    0.0%    0.0%    0.0%    0.0%    0.0%    0.0%    0.0%    0.0%    0.0%    0.0%    0.0%    0.0%    0.0%    0.0%    0.0%    0.0%    0.0%    0.0%    0.0%    0.0%    0.0%    0.0%    0.0%    0.0%    0.0%    0.0%    0.0%    0.0%    0.0%    0.0%    0.0%    0.0%    0.0%    0.0%    0.0%    0.0%    0.0%    0.0%    0.0%    0.0%    0.0%    0.0%    0.0%    0.0%    0.0%    0.0%    0.0%    0.0%    0.0%    0.0%    0.0%    0.0%    0.0%    0.0%    0.0%    0.0%    0.0%    0.0%    0.0%    0.0%    0.0%    0.0%    0.0%    0.0%    0.0%    0.0%    0.0%    0.0%    0.0%    0.0%    0.0%    0.0%    0.0%    0.0%    0.0%    0.0%    0.0%    0.0%    0.0%    0.0%    0.0%    0.0%    0.0%    0.0%    0.0%    0.0%    0.0%    0.0%    0.0%    0.0%    0.0%    0.0%    0.0%    0.0%    0.0%    0.0%    0.0%    0.0%    0.0%    0.0%    0.0%    0.0%    0.0%    0.0%    0.0%    0.0%    0.0%    0.0%    0.0%    0.0%    0.0%    0.0%    0.0%    0.0%    0.0%    0.0%    0.0%    0.0%    0.0%    0.0%    0.0%    0.0%    0.0%    0.0%    0.0%    0.0%    0.0%    0.0%    0.0%    0.0%    0.0%    0.0%    0.0%    0.0%    0.0%    0.0%    0.0%    0.0%    0.0%    0.0%    0.0%    0.0%    0.0%    0.0%    0.0%    0.0%    0.0%    0.0%    0.0%    0.0%    0.0%    0.0%    0.0%    0.0%    0.0%    0.0%    0.0%    0.0%    0.0%    0.0%    0.0%    0.0%    0.0%    0.0%    0.0%    0.0%    0.0%    0.0%    0.0%    0.0%    0.0%    0.0%    0.0%    0.0%    0.0%    0.0%    0.0%    0.0%    0.0%    0.0%    0.0%    0.0%    0.0%    0.0%    0.0%    0.0%    0.0%    0.0%    0.0%    0.0%    0.0%    0.0%    0.0%    0.0%    0.0%    0.0%    0.0%    0.0%    0.0%    0.0%    0.0%    0.0%    0.0%    0.0%    0.0%    0.0%    0.0%    0.0%    0.0%    0.0%    0.0%    0.0%    0.0%    0.0%    0.0%    0.0%    0.0%    0.0%    0.0%    0.0%    0.0%    0.0%    0.0%    0.0%    0.0%    0.0%    0.0%    0.0%    0.0%    0.0%    0.0%    0.0%    0.0%    0.0%    0.0%    0.0%    0.0%    0.0%    0.0%    0.0%    0.0%    0.0%    0.0%    0.0%    0.0%    0.0%    0.0%    0.0%    0.0%    0.0%    0.0%    0.0%    0.0%    0.0%    0.0%    0.0%    0.0%    0.0%    0.0%    0.0%    0.0%    0.0%    0.0%    0.0%    0.0%    0.0%    0.0%    0.0%    0.0%    0.0%    0.0%    0.0%    0.0%    0.0%    0.0%    0.0%    0.0%    0.0%    0.0%    0.0%    0.0%    0.0%    0.0%    0.0%    0.0%    0.0%    0.0%    0.0%    0.0%    0.0%    0.0%    0.0%    0.0%    0.0%    0.0%    0.0%    0.0%    0.0%    0.0%    0.0%    0.0%    0.0%    0.0%    0.0%    0.0%    0.0%    0.0%    0.0%    0.0%    0.0%    0.0%    0.0%    0.0%    0.0%    0.0%    0.0%    0.0%    0.0%    0.0%    0.0%    0.0%    0.0%    0.0%    0.0%    0.0%    0.0%    0.0%    0.0%    0.0%    0.0%    0.0%    0.0%    0.0%    0.0%    0.0%    0.0%    0.0%    0.0%    0.0%    0.0%    0.0%    0.0%    0.0%    0.0%    0.0%    0.0%    0.0%    0.0%    0.0%        k__Bacteria;p__OP10; c__S1a-1H      23    0.0%    0.0%    0.0%    0.0%    0.0%    0.0%    0.0%    0.0%    0.0%    0.0%    0.0%    0.0%    0.0%    0.0%    0.0%    0.0%    0.0%    0.0%    0.0%    0.0%    0.0%    0.0%    0.0%    0.0%    0.0%    0.0%    0.0%    0.0%    0.0%    0.0%    0.0%    0.0%    0.0%    0.0%    0.0%    0.0%    0.0%    0.0%    0.0%    0.0%    0.0%    0.0%    0.0%    0.0%    0.0%    0.0%    0.0%    0.0%    0.0%    0.0%    0.0%    0.0%    0.0%    0.0%    0.0%    0.0%    0.0%    0.0%    0.0%    0.0%    0.0%    0.0%    0.0%    0.0%    0.0%    0.0%    0.0%    0.0%    0.0%    0.0%    0.0%    0.0%    0.0%    0.0%    0.0%    0.0%    0.0%    0.0%    0.0%    0.0%    0.0%    0.0%    0.0%    0.0%    0.0%    0.0%    0.0%    0.0%    0.0%    0.0%    0.0%    0.0%    0.0%    0.0%    0.0%    0.0%    0.0%    0.0%    0.0%    0.0%    0.0%    0.0%    0.0%    0.0%    0.0%    0.0%    0.0%    0.0%    0.0%    0.0%    0.0%    0.0%    0.0%    0.0%    0.0%    0.0%    0.0%    0.0%    0.0%    0.0%    0.0%    0.0%    0.0%    0.0%    0.0%    0.0%    0.0%    0.0%    0.0%    0.0%    0.0%    0.0%    0.0%    0.0%    0.0%    0.0%    0.0%    0.0%    0.0%    0.0%    0.0%    0.0%    0.0%    0.0%    0.0%    0.0%    0.0%    0.0%    0.0%    0.0%    0.0%    0.0%    0.0%    0.0%    0.0%    0.0%    0.0%    0.0%    0.0%    0.0%    0.0%    0.0%    0.0%    0.0%    0.0%    0.0%    0.0%    0.0%    0.0%    0.0%    0.0%    0.0%    0.0%    0.0%    0.0%    0.0%    0.0%    0.0%    0.0%    0.0%    0.0%    0.0%    0.0%    0.0%    0.0%    0.0%    0.0%    0.0%    0.0%    0.0%    0.0%    0.0%    0.0%    0.0%    0.0%    0.0%    0.0%    0.0%    0.0%    0.0%    0.0%    0.0%    0.0%    0.0%    0.0%    0.0%    0.0%    0.0%    0.0%    0.0%    0.0%    0.0%    0.0%    0.0%    0.0%    0.0%    0.0%    0.0%    0.0%    0.0%    0.0%    0.0%    0.0%    0.0%    0.0%    0.0%    0.0%    0.0%    0.0%    0.0%    0.0%    0.0%    0.0%    0.0%    0.0%    0.0%    0.0%    0.0%    0.0%    0.0%    0.0%    0.0%    0.0%    0.0%    0.0%    0.0%    0.0%    0.0%    0.0%    0.0%    0.0%    0.0%    0.0%    0.0%    0.0%    0.0%    0.0%    0.0%    0.0%    0.0%    0.0%    0.0%    0.0%    0.0%    0.0%    0.0%    0.0%    0.0%    0.0%    0.0%    0.0%    0.0%    0.0%    0.0%    0.0%    0.0%    0.0%    0.0%    0.0%    0.0%    0.0%    0.0%    0.0%    0.0%    0.0%    0.0%    0.0%    0.0%    0.0%    0.0%    0.0%    0.0%    0.0%    0.0%    0.0%    0.0%    0.0%    0.0%    0.0%    0.0%    0.0%    0.0%    0.0%    0.0%    0.0%    0.0%    0.0%    0.0%    0.0%    0.0%    0.0%    0.0%    0.0%    0.0%    0.0%    0.0%    0.0%    0.0%    0.0%    0.0%    0.0%    0.0%    0.0%    0.0%    0.0%    0.0%    0.0%    0.0%    0.0%    0.0%    0.0%    0.0%    0.0%    0.0%    0.0%    0.0%    0.0%    0.0%    0.0%    0.0%    0.0%    0.0%    0.0%    0.0%    0.0%    0.0%    0.0%    0.0%    0.0%    0.0%    0.0%    0.0%    0.0%    0.0%    0.0%    0.0%    0.0%    0.0%        k__Bacteria;p__OP10; c__SJA-176       1    0.0%    0.0%    0.0%    0.0%    0.0%    0.0%    0.0%    0.0%    0.0%    0.0%    0.0%    0.0%    0.0%    0.0%    0.0%    0.0%    0.0%    0.0%    0.0%    0.0%    0.0%    0.0%    0.0%    0.0%    0.0%    0.0%    0.0%    0.0%    0.0%    0.0%    0.0%    0.0%    0.0%    0.0%    0.0%    0.0%    0.0%    0.0%    0.0%    0.0%    0.0%    0.0%    0.0%    0.0%    0.0%    0.0%    0.0%    0.0%    0.0%    0.0%    0.0%    0.0%    0.0%    0.0%    0.0%    0.0%    0.0%    0.0%    0.0%    0.0%    0.0%    0.0%    0.0%    0.0%    0.0%    0.0%    0.0%    0.0%    0.0%    0.0%    0.0%    0.0%    0.0%    0.0%    0.0%    0.0%    0.0%    0.0%    0.0%    0.0%    0.0%    0.0%    0.0%    0.0%    0.0%    0.0%    0.0%    0.0%    0.0%    0.0%    0.0%    0.0%    0.0%    0.0%    0.0%    0.0%    0.0%    0.0%    0.0%    0.0%    0.0%    0.0%    0.0%    0.0%    0.0%    0.0%    0.0%    0.0%    0.0%    0.0%    0.0%    0.0%    0.0%    0.0%    0.0%    0.0%    0.0%    0.0%    0.0%    0.0%    0.0%    0.0%    0.0%    0.0%    0.0%    0.0%    0.0%    0.0%    0.0%    0.0%    0.0%    0.0%    0.0%    0.0%    0.0%    0.0%    0.0%    0.0%    0.0%    0.0%    0.0%    0.0%    0.0%    0.0%    0.0%    0.0%    0.0%    0.0%    0.0%    0.0%    0.0%    0.0%    0.0%    0.0%    0.0%    0.0%    0.0%    0.0%    0.0%    0.0%    0.0%    0.0%    0.0%    0.0%    0.0%    0.0%    0.0%    0.0%    0.0%    0.0%    0.0%    0.0%    0.0%    0.0%    0.0%    0.0%    0.0%    0.0%    0.0%    0.0%    0.0%    0.0%    0.0%    0.0%    0.0%    0.0%    0.0%    0.0%    0.0%    0.0%    0.0%    0.0%    0.0%    0.0%    0.0%    0.0%    0.0%    0.0%    0.0%    0.0%    0.0%    0.0%    0.0%    0.0%    0.0%    0.0%    0.0%    0.0%    0.0%    0.0%    0.0%    0.0%    0.0%    0.0%    0.0%    0.0%    0.0%    0.0%    0.0%    0.0%    0.0%    0.0%    0.0%    0.0%    0.0%    0.0%    0.0%    0.0%    0.0%    0.0%    0.0%    0.0%    0.0%    0.0%    0.0%    0.0%    0.0%    0.0%    0.0%    0.0%    0.0%    0.0%    0.0%    0.0%    0.0%    0.0%    0.0%    0.0%    0.0%    0.0%    0.0%    0.0%    0.0%    0.0%    0.0%    0.0%    0.0%    0.0%    0.0%    0.0%    0.0%    0.0%    0.0%    0.0%    0.0%    0.0%    0.0%    0.0%    0.0%    0.0%    0.0%    0.0%    0.0%    0.0%    0.0%    0.0%    0.0%    0.0%    0.0%    0.0%    0.0%    0.0%    0.0%    0.0%    0.0%    0.0%    0.0%    0.0%    0.0%    0.0%    0.0%    0.0%    0.0%    0.0%    0.0%    0.0%    0.0%    0.0%    0.0%    0.0%    0.0%    0.0%    0.0%    0.0%    0.0%    0.0%    0.0%    0.0%    0.0%    0.0%    0.0%    0.0%    0.0%    0.0%    0.0%    0.0%    0.0%    0.0%    0.0%    0.0%    0.0%    0.0%    0.0%    0.0%    0.0%    0.0%    0.0%    0.0%    0.0%    0.0%    0.0%    0.0%    0.0%    0.0%    0.0%    0.0%    0.0%    0.0%    0.0%    0.0%    0.0%    0.0%    0.0%    0.0%    0.0%    0.0%    0.0%    0.0%    0.0%    0.0%    0.0%    0.0%    0.0%    0.0%    0.0%    0.0%    0.0%    0.0%        k__Bacteria;p__OP10; c__SJA-22       3    0.0%    0.0%    0.0%    0.0%    0.0%    0.0%    0.0%    0.0%    0.0%    0.0%    0.0%    0.0%    0.0%    0.0%    0.0%    0.0%    0.0%    0.0%    0.0%    0.0%    0.0%    0.0%    0.0%    0.0%    0.0%    0.0%    0.0%    0.0%    0.0%    0.0%    0.0%    0.0%    0.0%    0.0%    0.0%    0.0%    0.0%    0.0%    0.0%    0.0%    0.0%    0.0%    0.0%    0.0%    0.0%    0.0%    0.0%    0.0%    0.0%    0.0%    0.0%    0.0%    0.0%    0.0%    0.0%    0.0%    0.0%    0.0%    0.0%    0.0%    0.0%    0.0%    0.0%    0.0%    0.0%    0.0%    0.0%    0.0%    0.0%    0.0%    0.0%    0.0%    0.0%    0.0%    0.0%    0.0%    0.0%    0.0%    0.0%    0.0%    0.0%    0.0%    0.0%    0.0%    0.0%    0.0%    0.0%    0.0%    0.0%    0.0%    0.0%    0.0%    0.0%    0.0%    0.0%    0.0%    0.0%    0.0%    0.0%    0.0%    0.0%    0.0%    0.0%    0.0%    0.0%    0.0%    0.0%    0.0%    0.0%    0.0%    0.0%    0.0%    0.0%    0.0%    0.0%    0.0%    0.0%    0.0%    0.0%    0.0%    0.0%    0.0%    0.0%    0.0%    0.0%    0.0%    0.0%    0.0%    0.0%    0.0%    0.0%    0.0%    0.0%    0.0%    0.0%    0.0%    0.0%    0.0%    0.0%    0.0%    0.0%    0.0%    0.0%    0.0%    0.0%    0.0%    0.0%    0.0%    0.0%    0.0%    0.0%    0.0%    0.0%    0.0%    0.0%    0.0%    0.0%    0.0%    0.0%    0.0%    0.0%    0.0%    0.0%    0.0%    0.0%    0.0%    0.0%    0.0%    0.0%    0.0%    0.0%    0.0%    0.0%    0.0%    0.0%    0.0%    0.0%    0.0%    0.0%    0.0%    0.0%    0.0%    0.0%    0.0%    0.0%    0.0%    0.0%    0.0%    0.0%    0.0%    0.0%    0.0%    0.0%    0.0%    0.0%    0.0%    0.0%    0.0%    0.0%    0.0%    0.0%    0.0%    0.0%    0.0%    0.0%    0.0%    0.0%    0.0%    0.0%    0.0%    0.0%    0.0%    0.0%    0.0%    0.0%    0.0%    0.0%    0.0%    0.0%    0.0%    0.0%    0.0%    0.0%    0.0%    0.0%    0.0%    0.0%    0.0%    0.0%    0.0%    0.0%    0.0%    0.0%    0.0%    0.0%    0.0%    0.0%    0.0%    0.0%    0.0%    0.0%    0.0%    0.0%    0.0%    0.0%    0.0%    0.0%    0.0%    0.0%    0.0%    0.0%    0.0%    0.0%    0.0%    0.0%    0.0%    0.0%    0.0%    0.0%    0.0%    0.0%    0.0%    0.0%    0.0%    0.0%    0.0%    0.0%    0.0%    0.0%    0.0%    0.0%    0.0%    0.0%    0.0%    0.0%    0.0%    0.0%    0.0%    0.0%    0.0%    0.0%    0.0%    0.0%    0.0%    0.0%    0.0%    0.0%    0.0%    0.0%    0.0%    0.0%    0.0%    0.0%    0.0%    0.0%    0.0%    0.0%    0.0%    0.0%    0.0%    0.0%    0.0%    0.0%    0.0%    0.0%    0.0%    0.0%    0.0%    0.0%    0.0%    0.0%    0.0%    0.0%    0.0%    0.0%    0.0%    0.0%    0.0%    0.0%    0.0%    0.0%    0.0%    0.0%    0.0%    0.0%    0.0%    0.0%    0.0%    0.0%    0.0%    0.0%    0.0%    0.0%    0.0%    0.0%    0.0%    0.0%    0.0%    0.0%    0.0%    0.0%    0.0%    0.0%    0.0%    0.0%    0.0%    0.0%    0.0%    0.0%    0.0%    0.0%    0.0%    0.0%    0.0%    0.0%    0.0%    0.0%    0.0%        k__Bacteria;p__OP11; c__       2    0.0%    0.0%    0.0%    0.0%    0.0%    0.0%    0.0%    0.0%    0.0%    0.0%    0.0%    0.0%    0.0%    0.0%    0.0%    0.0%    0.0%    0.0%    0.0%    0.0%    0.0%    0.0%    0.0%    0.0%    0.0%    0.0%    0.0%    0.0%    0.0%    0.0%    0.0%    0.0%    0.0%    0.0%    0.0%    0.0%    0.0%    0.0%    0.0%    0.0%    0.0%    0.0%    0.0%    0.0%    0.0%    0.0%    0.0%    0.0%    0.0%    0.0%    0.0%    0.0%    0.0%    0.0%    0.0%    0.0%    0.0%    0.0%    0.0%    0.0%    0.0%    0.0%    0.0%    0.0%    0.0%    0.0%    0.0%    0.0%    0.0%    0.0%    0.0%    0.0%    0.0%    0.0%    0.0%    0.0%    0.0%    0.0%    0.0%    0.0%    0.0%    0.0%    0.0%    0.0%    0.0%    0.0%    0.0%    0.0%    0.0%    0.0%    0.0%    0.0%    0.0%    0.0%    0.0%    0.0%    0.0%    0.0%    0.0%    0.0%    0.0%    0.0%    0.0%    0.0%    0.0%    0.0%    0.0%    0.0%    0.0%    0.0%    0.0%    0.0%    0.0%    0.0%    0.0%    0.0%    0.0%    0.0%    0.0%    0.0%    0.0%    0.0%    0.0%    0.0%    0.0%    0.0%    0.0%    0.0%    0.0%    0.0%    0.0%    0.0%    0.0%    0.0%    0.0%    0.0%    0.0%    0.0%    0.0%    0.0%    0.0%    0.0%    0.0%    0.0%    0.0%    0.0%    0.0%    0.0%    0.0%    0.0%    0.0%    0.0%    0.0%    0.0%    0.0%    0.0%    0.0%    0.0%    0.0%    0.0%    0.0%    0.0%    0.0%    0.0%    0.0%    0.0%    0.0%    0.0%    0.0%    0.0%    0.0%    0.0%    0.0%    0.0%    0.0%    0.0%    0.0%    0.0%    0.0%    0.0%    0.0%    0.0%    0.0%    0.0%    0.0%    0.0%    0.0%    0.0%    0.0%    0.0%    0.0%    0.0%    0.0%    0.0%    0.0%    0.0%    0.0%    0.0%    0.0%    0.0%    0.0%    0.0%    0.0%    0.0%    0.0%    0.0%    0.0%    0.0%    0.0%    0.0%    0.0%    0.0%    0.0%    0.0%    0.0%    0.0%    0.0%    0.0%    0.0%    0.0%    0.0%    0.0%    0.0%    0.0%    0.0%    0.0%    0.0%    0.0%    0.0%    0.0%    0.0%    0.0%    0.0%    0.0%    0.0%    0.0%    0.0%    0.0%    0.0%    0.0%    0.0%    0.0%    0.0%    0.0%    0.0%    0.0%    0.0%    0.0%    0.0%    0.0%    0.0%    0.0%    0.0%    0.0%    0.0%    0.0%    0.0%    0.0%    0.0%    0.0%    0.0%    0.0%    0.0%    0.0%    0.0%    0.0%    0.0%    0.0%    0.0%    0.0%    0.0%    0.0%    0.0%    0.0%    0.0%    0.0%    0.0%    0.0%    0.0%    0.0%    0.0%    0.0%    0.0%    0.0%    0.0%    0.0%    0.0%    0.0%    0.0%    0.0%    0.0%    0.0%    0.0%    0.0%    0.0%    0.0%    0.0%    0.0%    0.0%    0.0%    0.0%    0.0%    0.0%    0.0%    0.0%    0.0%    0.0%    0.0%    0.0%    0.0%    0.0%    0.0%    0.0%    0.0%    0.0%    0.0%    0.0%    0.0%    0.0%    0.0%    0.0%    0.0%    0.0%    0.0%    0.0%    0.0%    0.0%    0.0%    0.0%    0.0%    0.0%    0.0%    0.0%    0.0%    0.0%    0.0%    0.0%    0.0%    0.0%    0.0%    0.0%    0.0%    0.0%    0.0%    0.0%    0.0%    0.0%    0.0%    0.0%    0.0%    0.0%    0.0%    0.0%    0.0%    0.0%    0.0%    0.0%    0.0%        k__Bacteria;p__OP3; c__       2    0.0%    0.0%    0.0%    0.0%    0.0%    0.0%    0.0%    0.0%    0.0%    0.0%    0.0%    0.0%    0.0%    0.0%    0.0%    0.0%    0.0%    0.0%    0.0%    0.0%    0.0%    0.0%    0.0%    0.0%    0.0%    0.0%    0.0%    0.0%    0.0%    0.0%    0.0%    0.0%    0.0%    0.0%    0.0%    0.0%    0.0%    0.0%    0.0%    0.0%    0.0%    0.0%    0.0%    0.0%    0.0%    0.0%    0.0%    0.0%    0.0%    0.0%    0.0%    0.0%    0.0%    0.0%    0.0%    0.0%    0.0%    0.0%    0.0%    0.0%    0.0%    0.0%    0.0%    0.0%    0.0%    0.0%    0.0%    0.0%    0.0%    0.0%    0.0%    0.0%    0.0%    0.0%    0.0%    0.0%    0.0%    0.0%    0.0%    0.0%    0.0%    0.0%    0.0%    0.0%    0.0%    0.0%    0.0%    0.0%    0.0%    0.0%    0.0%    0.0%    0.0%    0.0%    0.0%    0.0%    0.0%    0.0%    0.0%    0.0%    0.0%    0.0%    0.0%    0.0%    0.0%    0.0%    0.0%    0.0%    0.0%    0.0%    0.0%    0.0%    0.0%    0.0%    0.0%    0.0%    0.0%    0.0%    0.0%    0.0%    0.0%    0.0%    0.0%    0.0%    0.0%    0.0%    0.0%    0.0%    0.0%    0.0%    0.0%    0.0%    0.0%    0.0%    0.0%    0.0%    0.0%    0.0%    0.0%    0.0%    0.0%    0.0%    0.0%    0.0%    0.0%    0.0%    0.0%    0.0%    0.0%    0.0%    0.0%    0.0%    0.0%    0.0%    0.0%    0.0%    0.0%    0.0%    0.0%    0.0%    0.0%    0.0%    0.0%    0.0%    0.0%    0.0%    0.0%    0.0%    0.0%    0.0%    0.0%    0.0%    0.0%    0.0%    0.0%    0.0%    0.0%    0.0%    0.0%    0.0%    0.0%    0.0%    0.0%    0.0%    0.0%    0.0%    0.0%    0.0%    0.0%    0.0%    0.0%    0.0%    0.0%    0.0%    0.0%    0.0%    0.0%    0.0%    0.0%    0.0%    0.0%    0.0%    0.0%    0.0%    0.0%    0.0%    0.0%    0.0%    0.0%    0.0%    0.0%    0.0%    0.0%    0.0%    0.0%    0.0%    0.0%    0.0%    0.0%    0.0%    0.0%    0.0%    0.0%    0.0%    0.0%    0.0%    0.0%    0.0%    0.0%    0.0%    0.0%    0.0%    0.0%    0.0%    0.0%    0.0%    0.0%    0.0%    0.0%    0.0%    0.0%    0.0%    0.0%    0.0%    0.0%    0.0%    0.0%    0.0%    0.0%    0.0%    0.0%    0.0%    0.0%    0.0%    0.0%    0.0%    0.0%    0.0%    0.0%    0.0%    0.0%    0.0%    0.0%    0.0%    0.0%    0.0%    0.0%    0.0%    0.0%    0.0%    0.0%    0.0%    0.0%    0.0%    0.0%    0.0%    0.0%    0.0%    0.0%    0.0%    0.0%    0.0%    0.0%    0.0%    0.0%    0.0%    0.0%    0.0%    0.0%    0.0%    0.0%    0.0%    0.0%    0.0%    0.0%    0.0%    0.0%    0.0%    0.0%    0.0%    0.0%    0.0%    0.0%    0.0%    0.0%    0.0%    0.0%    0.0%    0.0%    0.0%    0.0%    0.0%    0.0%    0.0%    0.0%    0.0%    0.0%    0.0%    0.0%    0.0%    0.0%    0.0%    0.0%    0.0%    0.0%    0.0%    0.0%    0.0%    0.0%    0.0%    0.0%    0.0%    0.0%    0.0%    0.0%    0.0%    0.0%    0.0%    0.0%    0.0%    0.0%    0.0%    0.0%    0.0%    0.0%    0.0%    0.0%    0.0%    0.0%    0.0%    0.0%    0.0%    0.0%    0.0%    0.0%    0.0%    0.0%    0.0%        k__Bacteria;p__OP8; c__OP8       1    0.0%    0.0%    0.0%    0.0%    0.0%    0.0%    0.0%    0.0%    0.0%    0.0%    0.0%    0.0%    0.0%    0.0%    0.0%    0.0%    0.0%    0.0%    0.0%    0.0%    0.0%    0.0%    0.0%    0.0%    0.0%    0.0%    0.0%    0.0%    0.0%    0.0%    0.0%    0.0%    0.0%    0.0%    0.0%    0.0%    0.0%    0.0%    0.0%    0.0%    0.0%    0.0%    0.0%    0.0%    0.0%    0.0%    0.0%    0.0%    0.0%    0.0%    0.0%    0.0%    0.0%    0.0%    0.0%    0.0%    0.0%    0.0%    0.0%    0.0%    0.0%    0.0%    0.0%    0.0%    0.0%    0.0%    0.0%    0.0%    0.0%    0.0%    0.0%    0.0%    0.0%    0.0%    0.0%    0.0%    0.0%    0.0%    0.0%    0.0%    0.0%    0.0%    0.0%    0.0%    0.0%    0.0%    0.0%    0.0%    0.0%    0.0%    0.0%    0.0%    0.0%    0.0%    0.0%    0.0%    0.0%    0.0%    0.0%    0.0%    0.0%    0.0%    0.0%    0.0%    0.0%    0.0%    0.0%    0.0%    0.0%    0.0%    0.0%    0.0%    0.0%    0.0%    0.0%    0.0%    0.0%    0.0%    0.0%    0.0%    0.0%    0.0%    0.0%    0.0%    0.0%    0.0%    0.0%    0.0%    0.0%    0.0%    0.0%    0.0%    0.0%    0.0%    0.0%    0.0%    0.0%    0.0%    0.0%    0.0%    0.0%    0.0%    0.0%    0.0%    0.0%    0.0%    0.0%    0.0%    0.0%    0.0%    0.0%    0.0%    0.0%    0.0%    0.0%    0.0%    0.0%    0.0%    0.0%    0.0%    0.0%    0.0%    0.0%    0.0%    0.0%    0.0%    0.0%    0.0%    0.0%    0.0%    0.0%    0.0%    0.0%    0.0%    0.0%    0.0%    0.0%    0.0%    0.0%    0.0%    0.0%    0.0%    0.0%    0.0%    0.0%    0.0%    0.0%    0.0%    0.0%    0.0%    0.0%    0.0%    0.0%    0.0%    0.0%    0.0%    0.0%    0.0%    0.0%    0.0%    0.0%    0.0%    0.0%    0.0%    0.0%    0.0%    0.0%    0.0%    0.0%    0.0%    0.0%    0.0%    0.0%    0.0%    0.0%    0.0%    0.0%    0.0%    0.0%    0.0%    0.0%    0.0%    0.0%    0.0%    0.0%    0.0%    0.0%    0.0%    0.0%    0.0%    0.0%    0.0%    0.0%    0.0%    0.0%    0.0%    0.0%    0.0%    0.0%    0.0%    0.0%    0.0%    0.0%    0.0%    0.0%    0.0%    0.0%    0.0%    0.0%    0.0%    0.0%    0.0%    0.0%    0.0%    0.0%    0.0%    0.0%    0.0%    0.0%    0.0%    0.0%    0.0%    0.0%    0.0%    0.0%    0.0%    0.0%    0.0%    0.0%    0.0%    0.0%    0.0%    0.0%    0.0%    0.0%    0.0%    0.0%    0.0%    0.0%    0.0%    0.0%    0.0%    0.0%    0.0%    0.0%    0.0%    0.0%    0.0%    0.0%    0.0%    0.0%    0.0%    0.0%    0.0%    0.0%    0.0%    0.0%    0.0%    0.0%    0.0%    0.0%    0.0%    0.0%    0.0%    0.0%    0.0%    0.0%    0.0%    0.0%    0.0%    0.0%    0.0%    0.0%    0.0%    0.0%    0.0%    0.0%    0.0%    0.0%    0.0%    0.0%    0.0%    0.0%    0.0%    0.0%    0.0%    0.0%    0.0%    0.0%    0.0%    0.0%    0.0%    0.0%    0.0%    0.0%    0.0%    0.0%    0.0%    0.0%    0.0%    0.0%    0.0%    0.0%    0.0%    0.0%    0.0%    0.0%    0.0%    0.0%    0.0%    0.0%    0.0%    0.0%    0.0%    0.0%    0.0%    0.0%    0.0%        k__Bacteria;p__Planctomycetes; c__FFCH393     153    0.0%    0.0%    0.0%    0.0%    0.0%    0.0%    0.0%    0.0%    0.0%    0.0%    0.0%    0.0%    0.0%    0.0%    0.0%    0.0%    0.0%    0.0%    0.0%    0.0%    0.0%    0.0%    0.0%    0.0%    0.0%    0.0%    0.0%    0.0%    0.0%    0.0%    0.0%    0.0%    0.0%    0.0%    0.0%    0.0%    0.0%    0.0%    0.0%    0.0%    0.0%    0.0%    0.0%    0.0%    0.0%    0.0%    0.0%    0.0%    0.0%    0.0%    0.0%    0.0%    0.0%    0.0%    0.0%    0.0%    0.0%    0.0%    0.0%    0.0%    0.0%    0.0%    0.0%    0.0%    0.0%    0.0%    0.0%    0.0%    0.0%    0.0%    0.0%    0.0%    0.0%    0.0%    0.0%    0.0%    0.0%    0.0%    0.0%    0.0%    0.0%    0.0%    0.0%    0.0%    0.0%    0.0%    0.0%    0.0%    0.0%    0.0%    0.0%    0.0%    0.0%    0.0%    0.0%    0.0%    0.0%    0.0%    0.0%    0.0%    0.0%    0.0%    0.0%    0.0%    0.0%    0.0%    0.0%    0.0%    0.0%    0.0%    0.0%    0.0%    0.0%    0.0%    0.0%    0.0%    0.0%    0.0%    0.0%    0.0%    0.0%    0.0%    0.0%    0.0%    0.0%    0.0%    0.0%    0.0%    0.0%    0.0%    0.0%    0.0%    0.0%    0.0%    0.0%    0.0%    0.0%    0.0%    0.0%    0.0%    0.0%    0.0%    0.0%    0.0%    0.0%    0.0%    0.0%    0.0%    0.0%    0.0%    0.0%    0.0%    0.0%    0.0%    0.0%    0.0%    0.0%    0.0%    0.0%    0.0%    0.0%    0.0%    0.0%    0.0%    0.0%    0.0%    0.0%    0.0%    0.0%    0.0%    0.0%    0.0%    0.0%    0.0%    0.0%    0.0%    0.0%    0.0%    0.0%    0.0%    0.0%    0.0%    0.0%    0.0%    0.0%    0.0%    0.0%    0.0%    0.0%    0.0%    0.0%    0.0%    0.0%    0.0%    0.0%    0.0%    0.0%    0.0%    0.0%    0.0%    0.0%    0.0%    0.0%    0.0%    0.0%    0.0%    0.0%    0.0%    0.0%    0.0%    0.0%    0.0%    0.0%    0.0%    0.0%    0.0%    0.0%    0.0%    0.0%    0.0%    0.0%    0.0%    0.0%    0.0%    0.0%    0.0%    0.0%    0.0%    0.0%    0.0%    0.0%    0.0%    0.0%    0.0%    0.0%    0.0%    0.0%    0.0%    0.0%    0.0%    0.0%    0.0%    0.0%    0.0%    0.0%    0.0%    0.0%    0.0%    0.0%    0.0%    0.0%    0.0%    0.0%    0.0%    0.0%    0.0%    0.0%    0.0%    0.0%    0.0%    0.0%    0.0%    0.0%    0.0%    0.0%    0.0%    0.0%    0.0%    0.0%    0.0%    0.0%    0.0%    0.0%    0.0%    0.0%    0.0%    0.0%    0.0%    0.0%    0.0%    0.0%    0.0%    0.0%    0.0%    0.0%    0.0%    0.0%    0.0%    0.0%    0.0%    0.0%    0.0%    0.0%    0.0%    0.0%    0.0%    0.0%    0.0%    0.0%    0.0%    0.0%    0.0%    0.0%    0.0%    0.0%    0.0%    0.0%    0.0%    0.0%    0.0%    0.0%    0.0%    0.0%    0.0%    0.0%    0.0%    0.0%    0.0%    0.0%    0.0%    0.0%    0.0%    0.0%    0.0%    0.0%    0.0%    0.0%    0.0%    0.0%    0.0%    0.0%    0.0%    0.0%    0.0%    0.0%    0.0%    0.0%    0.0%    0.0%    0.0%    0.0%    0.0%    0.0%    0.0%    0.0%    0.0%    0.0%    0.0%    0.0%    0.0%    0.0%    0.0%    0.0%    0.0%    0.0%    0.0%    0.0%    0.0%        k__Bacteria;p__Planctomycetes; c__Kueneniae       1    0.0%    0.0%    0.0%    0.0%    0.0%    0.0%    0.0%    0.0%    0.0%    0.0%    0.0%    0.0%    0.0%    0.0%    0.0%    0.0%    0.0%    0.0%    0.0%    0.0%    0.0%    0.0%    0.0%    0.0%    0.0%    0.0%    0.0%    0.0%    0.0%    0.0%    0.0%    0.0%    0.0%    0.0%    0.0%    0.0%    0.0%    0.0%    0.0%    0.0%    0.0%    0.0%    0.0%    0.0%    0.0%    0.0%    0.0%    0.0%    0.0%    0.0%    0.0%    0.0%    0.0%    0.0%    0.0%    0.0%    0.0%    0.0%    0.0%    0.0%    0.0%    0.0%    0.0%    0.0%    0.0%    0.0%    0.0%    0.0%    0.0%    0.0%    0.0%    0.0%    0.0%    0.0%    0.0%    0.0%    0.0%    0.0%    0.0%    0.0%    0.0%    0.0%    0.0%    0.0%    0.0%    0.0%    0.0%    0.0%    0.0%    0.0%    0.0%    0.0%    0.0%    0.0%    0.0%    0.0%    0.0%    0.0%    0.0%    0.0%    0.0%    0.0%    0.0%    0.0%    0.0%    0.0%    0.0%    0.0%    0.0%    0.0%    0.0%    0.0%    0.0%    0.0%    0.0%    0.0%    0.0%    0.0%    0.0%    0.0%    0.0%    0.0%    0.0%    0.0%    0.0%    0.0%    0.0%    0.0%    0.0%    0.0%    0.0%    0.0%    0.0%    0.0%    0.0%    0.0%    0.0%    0.0%    0.0%    0.0%    0.0%    0.0%    0.0%    0.0%    0.0%    0.0%    0.0%    0.0%    0.0%    0.0%    0.0%    0.0%    0.0%    0.0%    0.0%    0.0%    0.0%    0.0%    0.0%    0.0%    0.0%    0.0%    0.0%    0.0%    0.0%    0.0%    0.0%    0.0%    0.0%    0.0%    0.0%    0.0%    0.0%    0.0%    0.0%    0.0%    0.0%    0.0%    0.0%    0.0%    0.0%    0.0%    0.0%    0.0%    0.0%    0.0%    0.0%    0.0%    0.0%    0.0%    0.0%    0.0%    0.0%    0.0%    0.0%    0.0%    0.0%    0.0%    0.0%    0.0%    0.0%    0.0%    0.0%    0.0%    0.0%    0.0%    0.0%    0.0%    0.0%    0.0%    0.0%    0.0%    0.0%    0.0%    0.0%    0.0%    0.0%    0.0%    0.0%    0.0%    0.0%    0.0%    0.0%    0.0%    0.0%    0.0%    0.0%    0.0%    0.0%    0.0%    0.0%    0.0%    0.0%    0.0%    0.0%    0.0%    0.0%    0.0%    0.0%    0.0%    0.0%    0.0%    0.0%    0.0%    0.0%    0.0%    0.0%    0.0%    0.0%    0.0%    0.0%    0.0%    0.0%    0.0%    0.0%    0.0%    0.0%    0.0%    0.0%    0.0%    0.0%    0.0%    0.0%    0.0%    0.0%    0.0%    0.0%    0.0%    0.0%    0.0%    0.0%    0.0%    0.0%    0.0%    0.0%    0.0%    0.0%    0.0%    0.0%    0.0%    0.0%    0.0%    0.0%    0.0%    0.0%    0.0%    0.0%    0.0%    0.0%    0.0%    0.0%    0.0%    0.0%    0.0%    0.0%    0.0%    0.0%    0.0%    0.0%    0.0%    0.0%    0.0%    0.0%    0.0%    0.0%    0.0%    0.0%    0.0%    0.0%    0.0%    0.0%    0.0%    0.0%    0.0%    0.0%    0.0%    0.0%    0.0%    0.0%    0.0%    0.0%    0.0%    0.0%    0.0%    0.0%    0.0%    0.0%    0.0%    0.0%    0.0%    0.0%    0.0%    0.0%    0.0%    0.0%    0.0%    0.0%    0.0%    0.0%    0.0%    0.0%    0.0%    0.0%    0.0%    0.0%    0.0%    0.0%    0.0%    0.0%    0.0%    0.0%    0.0%    0.0%    0.0%    0.0%    0.0%    0.0%    0.0%        k__Bacteria;p__Planctomycetes; c__PW285       0    0.0%    0.0%    0.0%    0.0%    0.0%    0.0%    0.0%    0.0%    0.0%    0.0%    0.0%    0.0%    0.0%    0.0%    0.0%    0.0%    0.0%    0.0%    0.0%    0.0%    0.0%    0.0%    0.0%    0.0%    0.0%    0.0%    0.0%    0.0%    0.0%    0.0%    0.0%    0.0%    0.0%    0.0%    0.0%    0.0%    0.0%    0.0%    0.0%    0.0%    0.0%    0.0%    0.0%    0.0%    0.0%    0.0%    0.0%    0.0%    0.0%    0.0%    0.0%    0.0%    0.0%    0.0%    0.0%    0.0%    0.0%    0.0%    0.0%    0.0%    0.0%    0.0%    0.0%    0.0%    0.0%    0.0%    0.0%    0.0%    0.0%    0.0%    0.0%    0.0%    0.0%    0.0%    0.0%    0.0%    0.0%    0.0%    0.0%    0.0%    0.0%    0.0%    0.0%    0.0%    0.0%    0.0%    0.0%    0.0%    0.0%    0.0%    0.0%    0.0%    0.0%    0.0%    0.0%    0.0%    0.0%    0.0%    0.0%    0.0%    0.0%    0.0%    0.0%    0.0%    0.0%    0.0%    0.0%    0.0%    0.0%    0.0%    0.0%    0.0%    0.0%    0.0%    0.0%    0.0%    0.0%    0.0%    0.0%    0.0%    0.0%    0.0%    0.0%    0.0%    0.0%    0.0%    0.0%    0.0%    0.0%    0.0%    0.0%    0.0%    0.0%    0.0%    0.0%    0.0%    0.0%    0.0%    0.0%    0.0%    0.0%    0.0%    0.0%    0.0%    0.0%    0.0%    0.0%    0.0%    0.0%    0.0%    0.0%    0.0%    0.0%    0.0%    0.0%    0.0%    0.0%    0.0%    0.0%    0.0%    0.0%    0.0%    0.0%    0.0%    0.0%    0.0%    0.0%    0.0%    0.0%    0.0%    0.0%    0.0%    0.0%    0.0%    0.0%    0.0%    0.0%    0.0%    0.0%    0.0%    0.0%    0.0%    0.0%    0.0%    0.0%    0.0%    0.0%    0.0%    0.0%    0.0%    0.0%    0.0%    0.0%    0.0%    0.0%    0.0%    0.0%    0.0%    0.0%    0.0%    0.0%    0.0%    0.0%    0.0%    0.0%    0.0%    0.0%    0.0%    0.0%    0.0%    0.0%    0.0%    0.0%    0.0%    0.0%    0.0%    0.0%    0.0%    0.0%    0.0%    0.0%    0.0%    0.0%    0.0%    0.0%    0.0%    0.0%    0.0%    0.0%    0.0%    0.0%    0.0%    0.0%    0.0%    0.0%    0.0%    0.0%    0.0%    0.0%    0.0%    0.0%    0.0%    0.0%    0.0%    0.0%    0.0%    0.0%    0.0%    0.0%    0.0%    0.0%    0.0%    0.0%    0.0%    0.0%    0.0%    0.0%    0.0%    0.0%    0.0%    0.0%    0.0%    0.0%    0.0%    0.0%    0.0%    0.0%    0.0%    0.0%    0.0%    0.0%    0.0%    0.0%    0.0%    0.0%    0.0%    0.0%    0.0%    0.0%    0.0%    0.0%    0.0%    0.0%    0.0%    0.0%    0.0%    0.0%    0.0%    0.0%    0.0%    0.0%    0.0%    0.0%    0.0%    0.0%    0.0%    0.0%    0.0%    0.0%    0.0%    0.0%    0.0%    0.0%    0.0%    0.0%    0.0%    0.0%    0.0%    0.0%    0.0%    0.0%    0.0%    0.0%    0.0%    0.0%    0.0%    0.0%    0.0%    0.0%    0.0%    0.0%    0.0%    0.0%    0.0%    0.0%    0.0%    0.0%    0.0%    0.0%    0.0%    0.0%    0.0%    0.0%    0.0%    0.0%    0.0%    0.0%    0.0%    0.0%    0.0%    0.0%    0.0%    0.0%    0.0%    0.0%    0.0%    0.0%    0.0%    0.0%    0.0%    0.0%    0.0%    0.0%    0.0%    0.0%    0.0%    0.0%    0.0%        k__Bacteria;p__Planctomycetes; c__Phycisphaerae    2005    0.0%    0.0%    0.0%    0.0%    0.0%    0.0%    0.0%    0.0%    0.0%    0.0%    0.0%    0.0%    0.0%    0.0%    0.0%    0.0%    0.0%    0.0%    0.0%    0.0%    0.0%    0.0%    0.0%    0.0%    0.0%    0.0%    0.0%    0.0%    0.0%    0.0%    0.0%    0.0%    0.0%    0.0%    0.0%    0.0%    0.0%    0.0%    0.0%    0.0%    0.0%    0.0%    0.0%    0.0%    0.0%    0.0%    0.0%    0.0%    0.0%    0.0%    0.0%    0.0%    0.0%    0.0%    0.0%    0.0%    0.0%    0.0%    0.0%    0.0%    0.1%    0.0%    0.0%    0.0%    0.0%    0.0%    0.0%    0.0%    0.0%    0.0%    0.0%    0.0%    0.0%    0.0%    0.0%    0.0%    0.0%    0.0%    0.0%    0.0%    0.0%    0.0%    0.0%    0.0%    0.0%    0.0%    0.0%    0.0%    0.0%    0.0%    0.0%    0.0%    0.0%    0.0%    0.0%    0.0%    0.0%    0.0%    0.0%    0.0%    0.0%    0.0%    0.0%    0.0%    0.0%    0.0%    0.0%    0.0%    0.0%    0.0%    0.0%    0.0%    0.0%    0.0%    0.0%    0.0%    0.0%    0.0%    0.0%    0.1%    0.0%    0.0%    0.0%    0.0%    0.0%    0.0%    0.0%    0.0%    0.1%    0.1%    0.0%    0.0%    0.0%    0.0%    0.1%    0.0%    0.0%    0.0%    0.0%    0.0%    0.0%    0.0%    0.0%    0.0%    0.0%    0.0%    0.0%    0.0%    0.0%    0.0%    0.0%    0.0%    0.0%    0.0%    0.0%    0.0%    0.0%    0.0%    0.0%    0.0%    0.1%    0.0%    0.0%    0.0%    0.0%    0.0%    0.0%    0.0%    0.0%    0.0%    0.0%    0.0%    0.0%    0.0%    0.0%    0.0%    0.0%    0.0%    0.0%    0.0%    0.0%    0.0%    0.0%    0.0%    0.0%    0.0%    0.0%    0.0%    0.0%    0.0%    0.0%    0.0%    0.0%    0.0%    0.0%    0.0%    0.0%    0.0%    0.0%    0.0%    0.0%    0.0%    0.0%    0.1%    0.0%    0.0%    0.0%    0.0%    0.1%    0.0%    0.0%    0.0%    0.0%    0.0%    0.0%    0.1%    0.0%    0.1%    0.0%    0.1%    0.0%    0.0%    0.0%    0.0%    0.0%    0.0%    0.0%    0.0%    0.0%    0.0%    0.0%    0.0%    0.0%    0.0%    0.0%    0.0%    0.0%    0.0%    0.0%    0.0%    0.0%    0.0%    0.0%    0.0%    0.0%    0.0%    0.0%    0.0%    0.0%    0.0%    0.0%    0.0%    0.0%    0.0%    0.0%    0.0%    0.0%    0.0%    0.0%    0.0%    0.0%    0.0%    0.0%    0.0%    0.0%    0.0%    0.0%    0.0%    0.0%    0.0%    0.0%    0.0%    0.0%    0.0%    0.0%    0.0%    0.0%    0.0%    0.0%    0.0%    0.0%    0.0%    0.0%    0.0%    0.0%    0.0%    0.0%    0.1%    0.0%    0.0%    0.0%    0.0%    0.0%    0.0%    0.0%    0.0%    0.0%    0.1%    0.0%    0.0%    0.0%    0.0%    0.0%    0.0%    0.0%    0.0%    0.0%    0.0%    0.0%    0.0%    0.0%    0.0%    0.0%    0.0%    0.0%    0.0%    0.0%    0.0%    0.0%    0.0%    0.0%    0.0%    0.0%    0.0%    0.0%    0.0%    0.0%    0.0%    0.0%    0.0%    0.0%    0.0%    0.0%    0.0%    0.0%    0.0%    0.0%    0.0%    0.0%    0.0%    0.0%    0.0%    0.0%    0.0%    0.0%    0.0%    0.0%    0.0%    0.0%    0.0%    0.0%    0.0%    0.0%    0.0%    0.0%    0.0%    0.0%    0.0%        k__Bacteria;p__Planctomycetes; c__Planctomycea    3631    0.0%    0.0%    0.0%    0.0%    0.0%    0.0%    0.0%    0.0%    0.0%    0.0%    0.0%    0.0%    0.0%    0.0%    0.0%    0.0%    0.0%    0.0%    0.0%    0.0%    0.0%    0.0%    0.0%    0.0%    0.0%    0.0%    0.0%    0.0%    0.0%    0.0%    0.0%    0.0%    0.0%    0.0%    0.0%    0.0%    0.0%    0.0%    0.0%    0.0%    0.0%    0.0%    0.0%    0.0%    0.0%    0.0%    0.0%    0.0%    0.0%    0.0%    0.0%    0.0%    0.0%    0.0%    0.1%    0.0%    0.0%    0.0%    0.0%    0.1%    0.0%    0.0%    0.0%    0.1%    0.1%    0.1%    0.0%    0.1%    0.0%    0.0%    0.0%    0.0%    0.0%    0.1%    0.0%    0.0%    0.0%    0.0%    0.0%    0.0%    0.0%    0.0%    0.0%    0.0%    0.0%    0.0%    0.0%    0.0%    0.0%    0.1%    0.0%    0.0%    0.0%    0.0%    0.0%    0.0%    0.1%    0.0%    0.0%    0.0%    0.1%    0.0%    0.0%    0.0%    0.0%    0.0%    0.1%    0.0%    0.0%    0.0%    0.0%    0.1%    0.0%    0.0%    0.0%    0.0%    0.0%    0.0%    0.0%    0.1%    0.0%    0.0%    0.1%    0.0%    0.0%    0.2%    0.1%    0.0%    0.1%    0.2%    0.1%    0.0%    0.0%    0.0%    0.1%    0.0%    0.0%    0.0%    0.0%    0.0%    0.0%    0.0%    0.0%    0.0%    0.0%    0.0%    0.0%    0.0%    0.0%    0.0%    0.0%    0.0%    0.0%    0.0%    0.0%    0.0%    0.0%    0.0%    0.0%    0.1%    0.2%    0.1%    0.1%    0.0%    0.0%    0.0%    0.1%    0.0%    0.0%    0.0%    0.1%    0.0%    0.0%    0.0%    0.0%    0.0%    0.0%    0.0%    0.0%    0.0%    0.0%    0.0%    0.0%    0.0%    0.0%    0.0%    0.0%    0.0%    0.0%    0.0%    0.0%    0.0%    0.0%    0.0%    0.0%    0.0%    0.0%    0.0%    0.0%    0.0%    0.1%    0.0%    0.0%    0.0%    0.0%    0.0%    0.0%    0.1%    0.1%    0.0%    0.0%    0.0%    0.2%    0.1%    0.0%    0.1%    0.0%    0.0%    0.0%    0.0%    0.0%    0.0%    0.0%    0.0%    0.0%    0.0%    0.0%    0.0%    0.0%    0.0%    0.0%    0.0%    0.1%    0.0%    0.0%    0.0%    0.1%    0.0%    0.0%    0.0%    0.0%    0.0%    0.0%    0.0%    0.0%    0.0%    0.0%    0.0%    0.0%    0.0%    0.1%    0.0%    0.0%    0.0%    0.0%    0.0%    0.0%    0.0%    0.0%    0.0%    0.1%    0.0%    0.0%    0.0%    0.0%    0.0%    0.0%    0.0%    0.0%    0.0%    0.0%    0.0%    0.1%    0.0%    0.0%    0.0%    0.0%    0.0%    0.0%    0.0%    0.0%    0.0%    0.0%    0.0%    0.0%    0.0%    0.0%    0.1%    0.0%    0.0%    0.0%    0.0%    0.0%    0.0%    0.0%    0.0%    0.0%    0.1%    0.0%    0.0%    0.0%    0.0%    0.0%    0.1%    0.0%    0.0%    0.0%    0.0%    0.0%    0.0%    0.0%    0.0%    0.0%    0.0%    0.0%    0.0%    0.0%    0.0%    0.0%    0.0%    0.0%    0.0%    0.0%    0.0%    0.0%    0.0%    0.0%    0.0%    0.0%    0.0%    0.0%    0.0%    0.0%    0.0%    0.0%    0.0%    0.0%    0.0%    0.0%    0.0%    0.0%    0.0%    0.0%    0.0%    0.0%    0.0%    0.0%    0.0%    0.0%    0.0%    0.0%    0.0%    0.0%    0.1%    0.0%    0.0%    0.0%    0.1%        k__Bacteria;p__Planctomycetes; c__agg27      42    0.0%    0.0%    0.0%    0.0%    0.0%    0.0%    0.0%    0.0%    0.0%    0.0%    0.0%    0.0%    0.0%    0.0%    0.0%    0.0%    0.0%    0.0%    0.0%    0.0%    0.0%    0.0%    0.0%    0.0%    0.0%    0.0%    0.0%    0.0%    0.0%    0.0%    0.0%    0.0%    0.0%    0.0%    0.0%    0.0%    0.0%    0.0%    0.0%    0.0%    0.0%    0.0%    0.0%    0.0%    0.0%    0.0%    0.0%    0.0%    0.0%    0.0%    0.0%    0.0%    0.0%    0.0%    0.0%    0.0%    0.0%    0.0%    0.0%    0.0%    0.0%    0.0%    0.0%    0.0%    0.0%    0.0%    0.0%    0.0%    0.0%    0.0%    0.0%    0.0%    0.0%    0.0%    0.0%    0.0%    0.0%    0.0%    0.0%    0.0%    0.0%    0.0%    0.0%    0.0%    0.0%    0.0%    0.0%    0.0%    0.0%    0.0%    0.0%    0.0%    0.0%    0.0%    0.0%    0.0%    0.0%    0.0%    0.0%    0.0%    0.0%    0.0%    0.0%    0.0%    0.0%    0.0%    0.0%    0.0%    0.0%    0.0%    0.0%    0.0%    0.0%    0.0%    0.0%    0.0%    0.0%    0.0%    0.0%    0.0%    0.0%    0.0%    0.0%    0.0%    0.0%    0.0%    0.0%    0.0%    0.0%    0.0%    0.0%    0.0%    0.0%    0.0%    0.0%    0.0%    0.0%    0.0%    0.0%    0.0%    0.0%    0.0%    0.0%    0.0%    0.0%    0.0%    0.0%    0.0%    0.0%    0.0%    0.0%    0.0%    0.0%    0.0%    0.0%    0.0%    0.0%    0.0%    0.0%    0.0%    0.0%    0.0%    0.0%    0.0%    0.0%    0.0%    0.0%    0.0%    0.0%    0.0%    0.0%    0.0%    0.0%    0.0%    0.0%    0.0%    0.0%    0.0%    0.0%    0.0%    0.0%    0.0%    0.0%    0.0%    0.0%    0.0%    0.0%    0.0%    0.0%    0.0%    0.0%    0.0%    0.0%    0.0%    0.0%    0.0%    0.0%    0.0%    0.0%    0.0%    0.0%    0.0%    0.0%    0.0%    0.0%    0.0%    0.0%    0.0%    0.0%    0.0%    0.0%    0.0%    0.0%    0.0%    0.0%    0.0%    0.0%    0.0%    0.0%    0.0%    0.0%    0.0%    0.0%    0.0%    0.0%    0.0%    0.0%    0.0%    0.0%    0.0%    0.0%    0.0%    0.0%    0.0%    0.0%    0.0%    0.0%    0.0%    0.0%    0.0%    0.0%    0.0%    0.0%    0.0%    0.0%    0.0%    0.0%    0.0%    0.0%    0.0%    0.0%    0.0%    0.0%    0.0%    0.0%    0.0%    0.0%    0.0%    0.0%    0.0%    0.0%    0.0%    0.0%    0.0%    0.0%    0.0%    0.0%    0.0%    0.0%    0.0%    0.0%    0.0%    0.0%    0.0%    0.0%    0.0%    0.0%    0.0%    0.0%    0.0%    0.0%    0.0%    0.0%    0.0%    0.0%    0.0%    0.0%    0.0%    0.0%    0.0%    0.0%    0.0%    0.0%    0.0%    0.0%    0.0%    0.0%    0.0%    0.0%    0.0%    0.0%    0.0%    0.0%    0.0%    0.0%    0.0%    0.0%    0.0%    0.0%    0.0%    0.0%    0.0%    0.0%    0.0%    0.0%    0.0%    0.0%    0.0%    0.0%    0.0%    0.0%    0.0%    0.0%    0.0%    0.0%    0.0%    0.0%    0.0%    0.0%    0.0%    0.0%    0.0%    0.0%    0.0%    0.0%    0.0%    0.0%    0.0%    0.0%    0.0%    0.0%    0.0%    0.0%    0.0%    0.0%    0.0%    0.0%    0.0%    0.0%    0.0%    0.0%    0.0%    0.0%    0.0%    0.0%    0.0%    0.0%    0.0%        k__Bacteria;p__Planctomycetes; c__vadinHA49      24    0.0%    0.0%    0.0%    0.0%    0.0%    0.0%    0.0%    0.0%    0.0%    0.0%    0.0%    0.0%    0.0%    0.0%    0.0%    0.0%    0.0%    0.0%    0.0%    0.0%    0.0%    0.0%    0.0%    0.0%    0.0%    0.0%    0.0%    0.0%    0.0%    0.0%    0.0%    0.0%    0.0%    0.0%    0.0%    0.0%    0.0%    0.0%    0.0%    0.0%    0.0%    0.0%    0.0%    0.0%    0.0%    0.0%    0.0%    0.0%    0.0%    0.0%    0.0%    0.0%    0.0%    0.0%    0.0%    0.0%    0.0%    0.0%    0.0%    0.0%    0.0%    0.0%    0.0%    0.0%    0.0%    0.0%    0.0%    0.0%    0.0%    0.0%    0.0%    0.0%    0.0%    0.0%    0.0%    0.0%    0.0%    0.0%    0.0%    0.0%    0.0%    0.0%    0.0%    0.0%    0.0%    0.0%    0.0%    0.0%    0.0%    0.0%    0.0%    0.0%    0.0%    0.0%    0.0%    0.0%    0.0%    0.0%    0.0%    0.0%    0.0%    0.0%    0.0%    0.0%    0.0%    0.0%    0.0%    0.0%    0.0%    0.0%    0.0%    0.0%    0.0%    0.0%    0.0%    0.0%    0.0%    0.0%    0.0%    0.0%    0.0%    0.0%    0.0%    0.0%    0.0%    0.0%    0.0%    0.0%    0.0%    0.0%    0.0%    0.0%    0.0%    0.0%    0.0%    0.0%    0.0%    0.0%    0.0%    0.0%    0.0%    0.0%    0.0%    0.0%    0.0%    0.0%    0.0%    0.0%    0.0%    0.0%    0.0%    0.0%    0.0%    0.0%    0.0%    0.0%    0.0%    0.0%    0.0%    0.0%    0.0%    0.0%    0.0%    0.0%    0.0%    0.0%    0.0%    0.0%    0.0%    0.0%    0.0%    0.0%    0.0%    0.0%    0.0%    0.0%    0.0%    0.0%    0.0%    0.0%    0.0%    0.0%    0.0%    0.0%    0.0%    0.0%    0.0%    0.0%    0.0%    0.0%    0.0%    0.0%    0.0%    0.0%    0.0%    0.0%    0.0%    0.0%    0.0%    0.0%    0.0%    0.0%    0.0%    0.0%    0.0%    0.0%    0.0%    0.0%    0.0%    0.0%    0.0%    0.0%    0.0%    0.0%    0.0%    0.0%    0.0%    0.0%    0.0%    0.0%    0.0%    0.0%    0.0%    0.0%    0.0%    0.0%    0.0%    0.0%    0.0%    0.0%    0.0%    0.0%    0.0%    0.0%    0.0%    0.0%    0.0%    0.0%    0.0%    0.0%    0.0%    0.0%    0.0%    0.0%    0.0%    0.0%    0.0%    0.0%    0.0%    0.0%    0.0%    0.0%    0.0%    0.0%    0.0%    0.0%    0.0%    0.0%    0.0%    0.0%    0.0%    0.0%    0.0%    0.0%    0.0%    0.0%    0.0%    0.0%    0.0%    0.0%    0.0%    0.0%    0.0%    0.0%    0.0%    0.0%    0.0%    0.0%    0.0%    0.0%    0.0%    0.0%    0.0%    0.0%    0.0%    0.0%    0.0%    0.0%    0.0%    0.0%    0.0%    0.0%    0.0%    0.0%    0.0%    0.0%    0.0%    0.0%    0.0%    0.0%    0.0%    0.0%    0.0%    0.0%    0.0%    0.0%    0.0%    0.0%    0.0%    0.0%    0.0%    0.0%    0.0%    0.0%    0.0%    0.0%    0.0%    0.0%    0.0%    0.0%    0.0%    0.0%    0.0%    0.0%    0.0%    0.0%    0.0%    0.0%    0.0%    0.0%    0.0%    0.0%    0.0%    0.0%    0.0%    0.0%    0.0%    0.0%    0.0%    0.0%    0.0%    0.0%    0.0%    0.0%    0.0%    0.0%    0.0%    0.0%    0.0%    0.0%    0.0%    0.0%    0.0%    0.0%    0.0%    0.0%    0.0%    0.0%        k__Bacteria;p__Proteobacteria; c__Alphaproteobacteria   558886    3.3%    0.5%    1.9%    0.7%    1.8%    2.2%    0.3%    0.4%    0.1%    0.0%    0.3%    0.6%    1.6%    0.7%    0.2%    1.4%    1.4%    3.4%    0.8%    1.8%    2.5%    3.7%    2.6%    0.1%    0.4%    0.6%    2.1%    1.4%    2.6%    0.4%    0.3%    2.7%    1.2%    0.3%    3.7%    0.4%    2.5%    0.1%    1.3%    4.2%    1.0%    2.5%    0.3%    0.5%    2.2%    0.9%    1.1%    0.3%    1.1%    0.3%    2.7%    5.0%    5.1%    1.7%    2.5%    1.3%    2.9%    0.8%    2.1%    2.9%    1.9%    2.1%    0.9%    5.8%    4.2%    2.1%    2.8%    5.5%    1.9%    2.8%    2.9%    1.0%    1.3%    2.1%    6.1%    0.4%    1.1%    2.7%    0.9%    1.8%    0.8%   13.1%    1.2%    0.9%    0.6%    0.3%    3.7%    0.9%    0.8%    3.7%    0.2%    3.0%    2.2%    0.7%    0.7%    3.3%    4.4%    0.7%    2.7%    1.9%    6.6%    5.8%    5.5%    1.9%    0.3%    0.3%    4.5%    1.8%    0.8%    2.6%    1.6%    3.4%    0.5%    0.2%    1.6%    0.9%    3.7%    2.4%    1.2%   13.8%    0.5%    1.8%    4.2%    2.4%   21.5%    8.6%    4.0%    3.2%    3.8%    5.7%    3.0%   18.1%    3.8%    1.3%    7.0%   13.3%   23.8%    5.4%    5.2%    4.1%    2.2%    3.2%    4.6%    1.9%    5.0%    3.6%    1.6%    4.2%    1.7%    2.4%    5.0%    2.0%    7.5%    7.6%    0.6%    0.7%    5.3%    1.1%   16.4%    5.8%    6.8%    8.1%    3.0%    9.6%    6.9%    7.7%    4.0%    1.7%    4.0%    1.3%    8.2%    3.8%    3.2%    2.8%    4.1%    1.1%    1.3%    0.5%    1.6%   15.2%    0.8%    0.6%    2.5%    1.4%    0.8%    0.9%    0.9%    0.5%    0.6%    1.1%    0.9%    1.2%    0.6%    1.9%    1.5%    6.0%    3.9%    0.8%    1.3%    0.5%    3.3%    1.5%    2.0%    6.5%    8.6%    4.4%   12.3%    3.9%   10.5%    5.3%    4.3%    6.5%    7.2%    2.3%    2.4%    3.6%    6.6%    1.8%    6.4%    4.2%    3.4%   10.1%    0.3%    6.7%    8.7%    4.4%    1.7%    2.2%    4.4%    3.3%    5.0%    6.3%    9.5%    3.1%    7.1%    2.4%    6.0%    9.2%    2.9%    4.5%    3.3%    4.0%    1.0%    0.5%    0.8%    0.7%    1.0%    4.1%    1.9%    2.4%    9.7%    3.2%    2.8%    2.4%    2.0%    3.3%    3.2%    9.6%    3.5%    2.1%   11.6%    5.0%    4.1%   19.8%    5.5%    1.8%    5.1%    3.4%    3.7%    6.6%    3.8%    4.4%   10.2%    4.7%    6.8%    3.8%    1.9%    1.8%    2.0%    1.0%    1.2%    2.1%    3.9%    0.7%    6.4%    0.5%    1.5%   14.0%    3.0%   12.1%    2.3%    0.5%    3.7%    3.6%    2.4%    0.7%    2.3%    2.8%    2.2%    4.1%    1.9%    2.7%    0.8%    4.7%    2.0%    0.3%    1.4%    1.0%    2.0%    0.3%    1.5%    3.9%    1.3%    1.9%    1.1%    7.5%    0.9%    0.6%    0.2%    1.8%    1.2%    2.1%    0.5%    2.4%    3.4%    3.6%    2.5%    1.2%    2.8%    2.3%    1.1%    0.3%    1.1%    0.9%    0.6%    0.2%    0.0%    0.5%    1.3%    0.7%    1.9%    0.7%    0.5%    3.8%    3.8%    0.6%    0.7%    0.4%    0.4%    0.6%    2.2%    0.5%    1.5%    5.1%    0.8%    0.3%    4.3%    1.7%        k__Bacteria;p__Proteobacteria; c__Betaproteobacteria   1310157    7.7%    7.2%    6.6%    4.2%    5.0%    1.4%    7.0%   66.4%    3.7%   10.1%   18.2%   60.1%    2.2%    6.7%   10.3%    5.1%    5.9%    3.5%    1.8%    2.2%    3.7%    5.2%    4.9%    0.3%    1.6%    2.5%    1.8%    5.3%    6.1%    0.9%    0.6%    3.3%    1.8%    5.7%    5.2%    5.8%    5.2%    1.3%    5.6%    5.1%    5.7%    7.8%   14.0%   16.3%    4.9%    4.2%    3.2%    1.1%    6.4%    4.4%    8.5%   15.9%   19.2%    2.4%    3.2%    1.5%    2.9%    0.7%    9.3%    4.0%    1.8%    3.7%    2.1%    4.3%    3.7%    1.2%    0.7%   10.5%    0.9%    3.1%    9.2%    2.6%    1.2%    6.5%    3.9%    1.0%    2.7%    3.7%    1.7%    3.5%    3.3%    4.0%    3.7%    4.1%    2.9%    4.2%    7.1%    1.2%    5.2%    5.9%    0.7%    3.0%    3.4%   12.7%    1.4%    2.1%    4.6%    3.1%    4.1%    3.7%    3.1%   24.2%   23.5%    3.1%    2.1%    9.6%    6.9%    2.9%    1.5%    4.7%    4.9%    6.2%    4.3%    0.8%    5.3%    9.5%   28.4%    4.9%   24.9%    9.5%    0.6%    4.5%    4.4%    5.6%    1.0%   22.5%   16.1%    4.7%   25.5%   12.9%    5.0%   16.4%   10.4%    3.9%    7.7%   26.9%    5.9%   11.5%   22.7%   20.3%   18.7%   15.1%   19.3%   17.6%   12.9%   18.2%   16.6%   20.1%    4.4%    1.2%   20.1%    4.2%    6.5%   32.9%    5.6%    1.5%    9.7%    1.9%    8.8%    5.3%    5.3%    6.6%   12.7%    2.8%   29.7%    2.5%    8.4%    4.1%    5.2%    7.7%    9.4%    8.3%    2.4%    7.8%   18.3%    7.9%    3.4%    2.5%    2.1%    4.8%    3.2%    3.1%   14.7%    5.4%    2.9%    2.4%    1.2%    3.8%    3.5%    8.0%    2.6%    3.4%    4.9%    9.0%    2.6%   20.5%    7.0%    4.0%    2.2%    2.8%    7.5%    2.1%    1.8%    4.8%    5.1%   11.2%    1.5%    3.8%    5.9%    3.7%    3.8%   28.3%   23.3%   10.6%    8.9%    4.2%   25.4%    5.9%    6.0%    5.2%    3.4%   20.7%    1.3%   14.1%   25.1%    4.6%    9.7%    9.9%    5.8%    6.5%   16.8%   23.5%    9.6%   14.4%    5.2%    2.0%    5.0%   36.3%   12.9%    3.7%    2.9%    8.0%    9.3%    4.0%   11.1%    1.5%    5.1%   10.4%   15.7%    5.4%   22.1%    9.7%    5.0%   12.5%    5.4%   17.4%   13.9%   45.0%    9.5%   15.5%   15.3%   29.9%    6.4%    9.1%   20.9%    2.6%    6.4%    8.0%   10.9%   25.7%    9.8%    5.3%   23.4%    5.4%   31.3%   15.7%    7.4%    4.1%    4.8%    0.8%    1.2%    3.8%   17.2%    2.8%   24.1%    5.7%   22.0%    5.3%    1.4%   12.9%    6.7%    1.3%    6.1%    6.3%    4.4%    0.8%    4.7%    2.7%    3.6%    3.3%    1.7%    2.6%    4.8%    3.3%    6.9%    3.4%    2.5%    1.2%    1.3%   13.9%    4.9%    4.8%    0.6%    6.8%    4.8%    5.7%    0.5%    6.4%    4.3%    4.1%    8.9%    9.1%    1.8%    1.5%    4.4%    4.5%    4.4%    4.3%    7.2%    5.1%    8.1%    6.6%    3.8%    1.6%    1.8%    6.2%   17.9%    4.2%    9.5%    0.2%    2.7%    0.7%    3.9%    2.4%    4.5%    2.0%    0.6%    3.5%    0.9%    1.0%    2.5%    9.3%    1.8%    3.3%    1.6%    0.4%    4.3%    0.0%        k__Bacteria;p__Proteobacteria; c__Deltaproteobacteria   13081    0.1%    0.0%    0.2%    0.0%    0.1%    0.0%    0.0%    0.0%    0.0%    0.0%    0.0%    0.0%    0.0%    0.0%    0.0%    0.0%    0.1%    0.1%    0.0%    0.0%    0.0%    0.1%    0.0%    0.0%    0.1%    0.0%    0.0%    0.1%    0.1%    0.0%    0.0%    0.1%    0.0%    0.0%    0.2%    0.0%    0.1%    0.0%    0.1%    0.1%    0.0%    0.1%    0.0%    0.1%    0.1%    0.0%    0.0%    0.0%    0.0%    0.0%    0.1%    0.1%    0.0%    0.0%    0.1%    0.1%    0.0%    0.0%    0.1%    0.1%    0.0%    0.1%    0.0%    0.2%    0.1%    0.1%    0.0%    0.2%    0.0%    0.1%    0.2%    0.1%    0.0%    0.0%    0.1%    0.0%    0.0%    0.1%    0.0%    0.1%    0.0%    0.2%    0.0%    0.0%    0.0%    0.0%    0.2%    0.0%    0.0%    0.1%    0.0%    0.1%    0.1%    0.0%    0.1%    0.1%    0.2%    0.0%    0.1%    0.1%    0.1%    0.0%    0.1%    0.0%    0.0%    0.0%    0.2%    0.0%    0.0%    0.1%    0.1%    0.2%    0.1%    0.0%    0.1%    0.0%    0.0%    0.0%    0.1%    0.6%    0.0%    0.1%    0.2%    0.1%    0.0%    0.7%    0.2%    0.0%    0.1%    0.4%    0.1%    0.0%    0.1%    0.0%    0.3%    0.0%    0.1%    0.1%    0.0%    0.0%    0.0%    0.1%    0.1%    0.0%    0.1%    0.1%    0.0%    0.0%    0.0%    0.0%    0.0%    0.0%    0.2%    0.1%    0.0%    0.0%    0.3%    0.0%    0.0%    0.3%    0.4%    0.2%    0.2%    0.1%    0.1%    0.1%    0.2%    0.0%    0.1%    0.1%    0.3%    0.2%    0.1%    0.1%    0.0%    0.0%    0.0%    0.0%    0.0%    0.0%    0.0%    0.0%    0.0%    0.0%    0.0%    0.0%    0.0%    0.0%    0.0%    0.0%    0.0%    0.1%    0.0%    0.1%    0.0%    0.1%    0.2%    0.0%    0.0%    0.0%    0.1%    0.0%    0.1%    0.2%    0.2%    0.1%    0.1%    0.1%    0.3%    0.3%    0.1%    0.0%    0.3%    0.1%    0.1%    0.1%    0.1%    0.0%    0.1%    0.1%    0.2%    0.1%    0.0%    0.2%    0.0%    0.2%    0.0%    0.0%    0.1%    0.1%    0.0%    0.0%    0.3%    0.1%    0.2%    0.0%    0.3%    0.0%    0.0%    0.0%    0.0%    0.1%    0.0%    0.0%    0.0%    0.0%    0.1%    0.1%    0.1%    0.0%    0.3%    0.1%    0.1%    0.0%    0.0%    0.1%    0.0%    0.0%    0.1%    0.1%    0.2%    0.3%    0.1%    0.1%    0.1%    0.0%    0.1%    0.1%    0.1%    0.0%    0.1%    0.3%    0.3%    0.1%    0.1%    0.0%    0.0%    0.0%    0.0%    0.1%    0.1%    0.0%    0.0%    0.0%    0.0%    0.0%    0.0%    0.6%    0.0%    0.0%    0.0%    0.0%    0.2%    0.2%    0.2%    0.1%    0.0%    0.1%    0.0%    0.2%    0.0%    0.1%    0.0%    0.2%    0.1%    0.0%    0.1%    0.0%    0.1%    0.0%    0.0%    0.0%    0.0%    0.0%    0.1%    0.2%    0.1%    0.0%    0.0%    0.0%    0.0%    0.0%    0.0%    0.1%    0.1%    0.0%    0.1%    0.0%    0.0%    0.1%    0.0%    0.0%    0.0%    0.1%    0.1%    0.0%    0.0%    0.0%    0.0%    0.0%    0.0%    0.0%    0.0%    0.0%    0.1%    0.0%    0.0%    0.0%    0.0%    0.0%    0.0%    0.0%    0.0%    0.2%    0.0%    0.0%    0.0%    0.0%        k__Bacteria;p__Proteobacteria; c__Epsilonproteobacteria   25726    0.2%    0.6%    0.5%    1.4%    0.4%    0.0%    0.3%    0.3%    0.1%    0.1%    0.3%    0.1%    0.0%    0.3%    0.5%    0.1%    0.2%    0.1%    0.1%    0.1%    0.2%    0.3%    0.0%    0.2%    0.0%    0.1%    0.0%    0.1%    0.2%    0.1%    0.1%    0.3%    0.2%    0.4%    1.0%    1.2%    0.4%    0.3%    0.3%    0.1%    0.2%    0.3%    0.4%    0.3%    0.1%    0.2%    0.0%    0.1%    0.2%    0.2%    0.3%    0.0%    0.1%    0.2%    0.1%    0.2%    0.1%    0.0%    0.1%    0.1%    0.0%    0.1%    0.1%    0.1%    3.3%    0.1%    0.0%    0.2%    0.0%    0.1%    0.4%    0.1%    0.0%    0.1%    0.2%    0.0%    0.1%    0.1%    0.2%    0.1%    0.1%    0.1%    0.3%    0.1%    0.4%    0.3%    0.2%    0.0%    0.2%    0.1%    0.0%    0.0%    0.1%    0.0%    0.0%    0.0%    0.1%    0.0%    0.1%    0.1%    0.1%    0.1%    0.1%    0.0%    0.2%    0.1%    0.2%    0.0%    0.0%    0.2%    0.1%    0.5%    0.1%    0.1%    0.1%    0.1%    0.0%    0.2%    0.1%    0.3%    0.0%    0.1%    0.1%    0.1%    0.0%    0.6%    0.3%    0.0%    0.1%    0.1%    0.2%    0.0%    0.0%    0.1%    0.1%    0.0%    0.0%    0.1%    0.0%    0.1%    0.0%    0.2%    0.1%    0.0%    0.0%    0.1%    0.3%    0.1%    0.2%    0.0%    0.0%    0.0%    0.1%    0.1%    0.2%    0.0%    0.1%    0.0%    0.0%    0.2%    0.0%    0.2%    0.1%    0.0%    0.0%    0.0%    0.1%    0.4%    0.1%    0.2%    0.1%    0.2%    0.0%    0.2%    0.2%    0.1%    0.1%    0.1%    0.0%    0.1%    0.2%    0.1%    0.1%    0.0%    0.2%    0.1%    0.3%    0.1%    0.1%    0.2%    0.2%    0.6%    0.1%    0.2%    0.1%    0.1%    0.2%    0.3%    0.1%    0.1%    0.2%    0.2%    0.1%    0.1%    0.2%    0.1%    0.0%    0.1%    0.1%    0.1%    0.2%    0.0%    0.0%    0.8%    0.0%    0.1%    0.0%    0.0%    0.4%    0.1%    0.0%    0.1%    0.0%    0.1%    0.1%    0.1%    0.2%    0.2%    0.1%    0.2%    0.1%    0.0%    0.0%    0.1%    0.1%    0.1%    0.1%    0.1%    0.0%    0.0%    0.2%    0.0%    0.0%    0.1%    0.0%    0.0%    0.0%    0.0%    0.0%    0.5%    0.1%    0.3%    0.1%    0.2%    0.0%    0.0%    0.1%    0.0%    0.2%    0.2%    0.2%    0.0%    0.1%    0.0%    0.2%    0.0%    0.1%    0.1%    0.1%    0.0%    0.1%    0.1%    0.1%    0.2%    0.1%    0.2%    0.2%    0.1%    0.1%    0.8%    0.0%    0.0%    0.0%    0.3%    0.1%    0.2%    0.1%    0.0%    0.1%    0.0%    0.0%    0.2%    0.1%    0.2%    0.0%    0.1%    0.1%    0.1%    0.1%    0.2%    0.3%    0.1%    0.2%    0.1%    0.2%    0.1%    0.1%    0.0%    0.0%    0.4%    0.0%    0.0%    0.0%    0.3%    0.1%    0.1%    0.0%    0.1%    0.3%    0.0%    0.4%    0.3%    1.2%    0.0%    0.2%    0.1%    0.1%    0.1%    0.4%    0.2%    0.0%    0.0%    0.0%    0.1%    0.0%    0.0%    0.1%    0.7%    0.3%    0.0%    0.0%    0.0%    0.1%    0.1%    0.1%    0.3%    0.0%    0.3%    0.0%    0.1%    0.0%    0.3%    0.2%    0.1%    0.0%    0.0%    0.0%    0.0%        k__Bacteria;p__Proteobacteria; c__Gammaproteobacteria   2637968   15.5%   16.3%    8.3%    8.5%   27.8%   40.5%    5.8%    3.7%    9.6%   13.4%    9.2%   10.1%   40.5%   16.1%   12.4%   20.5%   10.6%   33.4%    6.2%   10.7%   23.8%   20.9%   11.4%    1.7%   11.6%    5.9%   17.5%   15.8%   18.0%    4.3%    3.4%   10.2%    3.0%    8.0%   19.8%    6.1%   17.2%    4.4%   31.8%   21.4%   16.0%   18.5%   14.3%   15.0%    9.8%   11.7%   21.2%    7.4%   19.0%    6.6%   16.5%   20.9%   25.1%   16.2%   10.0%    2.1%    7.7%   74.6%   18.5%   29.0%    5.4%    4.9%    5.9%   11.0%    5.5%    5.1%   40.8%   15.6%   30.5%    8.0%   47.4%    4.9%    4.9%    8.8%    6.4%    1.1%   18.9%   11.6%    2.2%    6.8%    4.4%   39.8%   16.3%   10.1%    3.4%    4.3%   10.8%   28.1%   21.5%    8.7%   54.7%   21.5%   14.1%   18.7%    2.1%    4.7%   14.1%   22.0%    7.5%    6.7%   16.0%    6.0%    2.4%    5.2%    2.6%    9.7%   17.1%   29.1%   50.0%    5.8%    5.6%   10.5%   26.1%    3.3%   15.6%    7.9%    7.7%    5.1%   20.4%   11.4%   80.7%   13.4%   12.5%   21.3%   64.7%   12.9%   10.1%   32.8%   16.1%   16.8%   10.2%   24.9%   19.5%    7.4%   17.8%   12.3%   34.3%   29.9%   53.1%   10.7%   56.6%   29.7%   15.8%   43.4%   15.6%    3.3%    8.3%    4.4%   12.5%   42.8%    8.5%   24.2%   24.1%    4.7%    7.3%   37.5%   20.9%   19.0%   35.6%   11.2%    4.4%   17.1%   38.0%   31.4%    6.9%   25.0%   10.3%    7.5%   19.5%    8.3%   19.2%   12.7%   13.7%   15.4%    4.0%    9.2%   10.9%    8.8%   25.7%   25.0%    5.4%    8.1%   16.1%    6.6%    2.1%    6.1%    5.5%    7.1%    9.4%    6.7%    2.7%    6.5%    6.1%    6.8%   19.4%    8.2%    6.8%    6.1%    4.4%    4.6%   17.2%    5.0%   13.4%   18.8%   11.1%   10.2%   66.2%   18.6%   13.2%   10.1%    9.2%    5.7%   15.4%   11.3%   12.2%    9.5%   19.7%    3.1%    9.8%    3.9%    3.1%   20.5%   73.5%   31.9%   12.5%   11.9%    6.5%   11.5%    9.6%   11.2%    6.7%    1.9%   41.5%    8.3%   16.7%   31.2%   17.5%    4.2%    3.5%    5.6%   15.3%    7.4%    7.1%   10.9%   19.6%    3.9%    8.0%    5.9%   21.1%    5.1%    8.3%   35.0%   52.6%    5.1%   20.9%    4.3%   19.8%    6.1%   10.2%   10.6%   13.8%   15.5%   16.8%   34.7%    8.2%   29.1%   18.0%   11.4%   21.6%    6.2%    7.6%   12.7%   19.4%   13.0%    5.5%   18.1%    7.8%   12.3%   26.0%    2.4%   17.2%    5.5%   10.2%    9.8%    9.2%    7.2%   21.6%   11.4%    7.3%   12.3%    9.6%    1.4%   10.0%   21.0%    7.3%    4.1%    8.8%    5.3%    8.1%    7.0%    3.3%    6.8%    6.7%    6.5%    5.3%   19.1%    5.8%    2.7%   19.6%   14.9%    6.2%    6.5%   34.1%   14.8%    7.1%    9.0%    1.7%    4.5%    6.1%    6.9%    9.1%   11.7%    1.6%    5.2%   16.7%    9.0%    7.1%   19.3%    9.5%    9.8%    5.2%    3.3%    3.6%    2.4%    0.8%    1.9%   12.7%    4.7%    6.0%   59.6%   12.0%   51.5%    7.2%    4.3%   16.3%    2.9%    6.9%    4.7%   55.4%    2.5%   17.4%    8.1%    3.2%    6.4%    2.2%   87.3%   14.1%    0.8%        k__Bacteria;p__SC3; c__       6    0.0%    0.0%    0.0%    0.0%    0.0%    0.0%    0.0%    0.0%    0.0%    0.0%    0.0%    0.0%    0.0%    0.0%    0.0%    0.0%    0.0%    0.0%    0.0%    0.0%    0.0%    0.0%    0.0%    0.0%    0.0%    0.0%    0.0%    0.0%    0.0%    0.0%    0.0%    0.0%    0.0%    0.0%    0.0%    0.0%    0.0%    0.0%    0.0%    0.0%    0.0%    0.0%    0.0%    0.0%    0.0%    0.0%    0.0%    0.0%    0.0%    0.0%    0.0%    0.0%    0.0%    0.0%    0.0%    0.0%    0.0%    0.0%    0.0%    0.0%    0.0%    0.0%    0.0%    0.0%    0.0%    0.0%    0.0%    0.0%    0.0%    0.0%    0.0%    0.0%    0.0%    0.0%    0.0%    0.0%    0.0%    0.0%    0.0%    0.0%    0.0%    0.0%    0.0%    0.0%    0.0%    0.0%    0.0%    0.0%    0.0%    0.0%    0.0%    0.0%    0.0%    0.0%    0.0%    0.0%    0.0%    0.0%    0.0%    0.0%    0.0%    0.0%    0.0%    0.0%    0.0%    0.0%    0.0%    0.0%    0.0%    0.0%    0.0%    0.0%    0.0%    0.0%    0.0%    0.0%    0.0%    0.0%    0.0%    0.0%    0.0%    0.0%    0.0%    0.0%    0.0%    0.0%    0.0%    0.0%    0.0%    0.0%    0.0%    0.0%    0.0%    0.0%    0.0%    0.0%    0.0%    0.0%    0.0%    0.0%    0.0%    0.0%    0.0%    0.0%    0.0%    0.0%    0.0%    0.0%    0.0%    0.0%    0.0%    0.0%    0.0%    0.0%    0.0%    0.0%    0.0%    0.0%    0.0%    0.0%    0.0%    0.0%    0.0%    0.0%    0.0%    0.0%    0.0%    0.0%    0.0%    0.0%    0.0%    0.0%    0.0%    0.0%    0.0%    0.0%    0.0%    0.0%    0.0%    0.0%    0.0%    0.0%    0.0%    0.0%    0.0%    0.0%    0.0%    0.0%    0.0%    0.0%    0.0%    0.0%    0.0%    0.0%    0.0%    0.0%    0.0%    0.0%    0.0%    0.0%    0.0%    0.0%    0.0%    0.0%    0.0%    0.0%    0.0%    0.0%    0.0%    0.0%    0.0%    0.0%    0.0%    0.0%    0.0%    0.0%    0.0%    0.0%    0.0%    0.0%    0.0%    0.0%    0.0%    0.0%    0.0%    0.0%    0.0%    0.0%    0.0%    0.0%    0.0%    0.0%    0.0%    0.0%    0.0%    0.0%    0.0%    0.0%    0.0%    0.0%    0.0%    0.0%    0.0%    0.0%    0.0%    0.0%    0.0%    0.0%    0.0%    0.0%    0.0%    0.0%    0.0%    0.0%    0.0%    0.0%    0.0%    0.0%    0.0%    0.0%    0.0%    0.0%    0.0%    0.0%    0.0%    0.0%    0.0%    0.0%    0.0%    0.0%    0.0%    0.0%    0.0%    0.0%    0.0%    0.0%    0.0%    0.0%    0.0%    0.0%    0.0%    0.0%    0.0%    0.0%    0.0%    0.0%    0.0%    0.0%    0.0%    0.0%    0.0%    0.0%    0.0%    0.0%    0.0%    0.0%    0.0%    0.0%    0.0%    0.0%    0.0%    0.0%    0.0%    0.0%    0.0%    0.0%    0.0%    0.0%    0.0%    0.0%    0.0%    0.0%    0.0%    0.0%    0.0%    0.0%    0.0%    0.0%    0.0%    0.0%    0.0%    0.0%    0.0%    0.0%    0.0%    0.0%    0.0%    0.0%    0.0%    0.0%    0.0%    0.0%    0.0%    0.0%    0.0%    0.0%    0.0%    0.0%    0.0%    0.0%    0.0%    0.0%    0.0%    0.0%    0.0%    0.0%    0.0%    0.0%    0.0%    0.0%    0.0%    0.0%    0.0%    0.0%    0.0%    0.0%    0.0%    0.0%        k__Bacteria;p__SC4; c__    1057    0.0%    0.0%    0.0%    0.0%    0.0%    0.0%    0.0%    0.0%    0.0%    0.0%    0.0%    0.0%    0.0%    0.0%    0.0%    0.0%    0.0%    0.0%    0.0%    0.0%    0.0%    0.0%    0.0%    0.0%    0.0%    0.0%    0.0%    0.0%    0.0%    0.0%    0.0%    0.0%    0.0%    0.0%    0.0%    0.0%    0.0%    0.0%    0.0%    0.0%    0.0%    0.0%    0.0%    0.0%    0.0%    0.0%    0.0%    0.0%    0.0%    0.0%    0.0%    0.0%    0.0%    0.0%    0.0%    0.0%    0.0%    0.0%    0.0%    0.0%    0.0%    0.0%    0.0%    0.1%    0.0%    0.0%    0.0%    0.0%    0.0%    0.0%    0.0%    0.0%    0.0%    0.0%    0.0%    0.0%    0.0%    0.0%    0.0%    0.0%    0.0%    0.0%    0.0%    0.0%    0.0%    0.0%    0.0%    0.0%    0.0%    0.0%    0.0%    0.0%    0.0%    0.0%    0.0%    0.0%    0.0%    0.0%    0.0%    0.0%    0.0%    0.0%    0.0%    0.0%    0.0%    0.0%    0.0%    0.0%    0.0%    0.0%    0.0%    0.0%    0.0%    0.0%    0.0%    0.0%    0.0%    0.0%    0.0%    0.0%    0.0%    0.0%    0.0%    0.0%    0.0%    0.0%    0.0%    0.0%    0.0%    0.0%    0.0%    0.0%    0.0%    0.0%    0.0%    0.0%    0.0%    0.0%    0.0%    0.0%    0.0%    0.0%    0.0%    0.0%    0.0%    0.0%    0.0%    0.0%    0.0%    0.0%    0.0%    0.0%    0.0%    0.0%    0.0%    0.0%    0.0%    0.0%    0.0%    0.0%    0.0%    0.0%    0.0%    0.0%    0.0%    0.0%    0.0%    0.0%    0.0%    0.0%    0.0%    0.0%    0.0%    0.0%    0.0%    0.0%    0.0%    0.0%    0.0%    0.0%    0.0%    0.0%    0.0%    0.0%    0.0%    0.0%    0.0%    0.0%    0.0%    0.0%    0.0%    0.0%    0.0%    0.0%    0.0%    0.0%    0.0%    0.0%    0.0%    0.0%    0.0%    0.0%    0.0%    0.0%    0.0%    0.0%    0.0%    0.0%    0.0%    0.0%    0.0%    0.0%    0.0%    0.0%    0.0%    0.0%    0.0%    0.0%    0.0%    0.0%    0.0%    0.0%    0.0%    0.0%    0.0%    0.0%    0.0%    0.0%    0.0%    0.0%    0.0%    0.0%    0.0%    0.0%    0.0%    0.0%    0.0%    0.0%    0.0%    0.0%    0.0%    0.0%    0.0%    0.0%    0.0%    0.0%    0.0%    0.0%    0.0%    0.0%    0.0%    0.0%    0.0%    0.0%    0.0%    0.0%    0.0%    0.0%    0.0%    0.0%    0.0%    0.0%    0.0%    0.0%    0.0%    0.0%    0.0%    0.0%    0.0%    0.0%    0.0%    0.0%    0.1%    0.0%    0.0%    0.0%    0.0%    0.0%    0.0%    0.0%    0.0%    0.0%    0.0%    0.0%    0.0%    0.0%    0.0%    0.1%    0.0%    0.0%    0.0%    0.0%    0.0%    0.0%    0.0%    0.0%    0.0%    0.0%    0.0%    0.1%    0.0%    0.0%    0.0%    0.1%    0.0%    0.0%    0.0%    0.0%    0.0%    0.0%    0.0%    0.0%    0.0%    0.0%    0.0%    0.0%    0.0%    0.0%    0.0%    0.0%    0.0%    0.0%    0.0%    0.0%    0.0%    0.0%    0.0%    0.0%    0.0%    0.0%    0.0%    0.0%    0.0%    0.0%    0.0%    0.0%    0.0%    0.0%    0.0%    0.0%    0.0%    0.0%    0.0%    0.0%    0.0%    0.0%    0.0%    0.0%    0.0%    0.0%    0.0%    0.0%    0.0%    0.0%    0.0%    0.0%    0.0%    0.0%        k__Bacteria;p__SPAM; c__     334    0.0%    0.0%    0.0%    0.0%    0.0%    0.0%    0.0%    0.0%    0.0%    0.0%    0.0%    0.0%    0.0%    0.0%    0.0%    0.0%    0.0%    0.0%    0.0%    0.0%    0.0%    0.0%    0.0%    0.0%    0.0%    0.0%    0.0%    0.0%    0.0%    0.0%    0.0%    0.0%    0.0%    0.0%    0.0%    0.0%    0.0%    0.0%    0.0%    0.0%    0.0%    0.0%    0.0%    0.0%    0.0%    0.0%    0.0%    0.0%    0.0%    0.0%    0.0%    0.0%    0.0%    0.0%    0.0%    0.0%    0.0%    0.0%    0.0%    0.0%    0.0%    0.0%    0.0%    0.0%    0.0%    0.0%    0.0%    0.0%    0.0%    0.0%    0.0%    0.0%    0.0%    0.0%    0.0%    0.0%    0.0%    0.0%    0.0%    0.0%    0.0%    0.0%    0.0%    0.0%    0.0%    0.0%    0.0%    0.0%    0.0%    0.0%    0.0%    0.0%    0.0%    0.0%    0.0%    0.0%    0.0%    0.0%    0.0%    0.0%    0.0%    0.0%    0.0%    0.0%    0.0%    0.0%    0.0%    0.0%    0.0%    0.0%    0.0%    0.0%    0.0%    0.0%    0.0%    0.0%    0.0%    0.0%    0.0%    0.0%    0.0%    0.0%    0.0%    0.0%    0.0%    0.0%    0.0%    0.0%    0.0%    0.0%    0.0%    0.0%    0.0%    0.0%    0.0%    0.0%    0.0%    0.0%    0.0%    0.0%    0.0%    0.0%    0.0%    0.0%    0.0%    0.0%    0.0%    0.0%    0.0%    0.0%    0.0%    0.0%    0.0%    0.0%    0.0%    0.0%    0.0%    0.0%    0.0%    0.0%    0.0%    0.0%    0.0%    0.0%    0.0%    0.0%    0.0%    0.0%    0.0%    0.0%    0.0%    0.0%    0.0%    0.0%    0.0%    0.0%    0.0%    0.0%    0.0%    0.0%    0.0%    0.0%    0.0%    0.0%    0.0%    0.0%    0.0%    0.0%    0.0%    0.0%    0.0%    0.0%    0.0%    0.0%    0.0%    0.0%    0.0%    0.0%    0.0%    0.0%    0.0%    0.0%    0.0%    0.0%    0.0%    0.0%    0.0%    0.0%    0.0%    0.0%    0.0%    0.0%    0.0%    0.0%    0.0%    0.0%    0.0%    0.0%    0.0%    0.0%    0.0%    0.0%    0.0%    0.0%    0.0%    0.0%    0.0%    0.0%    0.0%    0.0%    0.0%    0.0%    0.0%    0.0%    0.0%    0.0%    0.0%    0.0%    0.0%    0.0%    0.0%    0.0%    0.0%    0.0%    0.0%    0.0%    0.0%    0.0%    0.0%    0.0%    0.0%    0.0%    0.0%    0.0%    0.0%    0.0%    0.0%    0.0%    0.0%    0.0%    0.0%    0.0%    0.0%    0.0%    0.0%    0.0%    0.0%    0.0%    0.0%    0.0%    0.0%    0.0%    0.0%    0.0%    0.0%    0.0%    0.0%    0.0%    0.0%    0.0%    0.0%    0.0%    0.0%    0.0%    0.0%    0.0%    0.0%    0.0%    0.0%    0.0%    0.0%    0.0%    0.0%    0.0%    0.0%    0.0%    0.0%    0.0%    0.0%    0.0%    0.0%    0.0%    0.0%    0.0%    0.0%    0.0%    0.0%    0.0%    0.0%    0.0%    0.0%    0.0%    0.0%    0.0%    0.0%    0.0%    0.0%    0.0%    0.0%    0.0%    0.0%    0.0%    0.0%    0.0%    0.0%    0.0%    0.0%    0.0%    0.0%    0.0%    0.0%    0.0%    0.0%    0.0%    0.0%    0.0%    0.0%    0.0%    0.0%    0.0%    0.0%    0.0%    0.0%    0.0%    0.0%    0.0%    0.0%    0.0%    0.0%    0.0%    0.0%    0.0%    0.0%    0.0%    0.0%    0.0%    0.0%    0.0%        k__Bacteria;p__SR1; c__    1149    0.0%    0.0%    0.0%    0.0%    0.0%    0.0%    0.0%    0.0%    0.0%    0.0%    0.0%    0.0%    0.0%    0.0%    0.0%    0.0%    0.0%    0.0%    0.0%    0.0%    0.0%    0.0%    0.0%    0.0%    0.0%    0.0%    0.0%    0.0%    0.0%    0.0%    0.0%    0.0%    0.0%    0.0%    0.0%    0.0%    0.0%    0.0%    0.0%    0.0%    0.0%    0.0%    0.0%    0.0%    0.0%    0.0%    0.0%    0.0%    0.0%    0.0%    0.0%    0.0%    0.0%    0.0%    0.0%    0.0%    0.0%    0.0%    0.0%    0.0%    0.0%    0.0%    0.0%    0.0%    0.0%    0.0%    0.0%    0.0%    0.0%    0.0%    0.0%    0.0%    0.0%    0.0%    0.0%    0.0%    0.0%    0.0%    0.0%    0.0%    0.0%    0.0%    0.0%    0.0%    0.0%    0.0%    0.0%    0.0%    0.0%    0.0%    0.0%    0.0%    0.0%    0.0%    0.0%    0.0%    0.0%    0.0%    0.0%    0.0%    0.0%    0.0%    0.0%    0.0%    0.0%    0.0%    0.1%    0.0%    0.0%    0.0%    0.0%    0.0%    0.0%    0.0%    0.0%    0.0%    0.0%    0.0%    0.0%    0.0%    0.0%    0.0%    0.0%    0.0%    0.0%    0.0%    0.0%    0.0%    0.0%    0.0%    0.0%    0.0%    0.0%    0.0%    0.0%    0.0%    0.0%    0.0%    0.0%    0.0%    0.0%    0.0%    0.0%    0.0%    0.0%    0.0%    0.0%    0.0%    0.0%    0.0%    0.0%    0.0%    0.0%    0.0%    0.0%    0.0%    0.0%    0.0%    0.0%    0.0%    0.0%    0.0%    0.1%    0.0%    0.0%    0.0%    0.0%    0.0%    0.0%    0.0%    0.0%    0.0%    0.0%    0.0%    0.0%    0.0%    0.0%    0.0%    0.0%    0.0%    0.0%    0.0%    0.0%    0.0%    0.0%    0.0%    0.0%    0.0%    0.0%    0.0%    0.0%    0.0%    0.0%    0.0%    0.0%    0.0%    0.0%    0.0%    0.0%    0.0%    0.0%    0.0%    0.0%    0.0%    0.0%    0.0%    0.0%    0.0%    0.0%    0.0%    0.0%    0.0%    0.0%    0.0%    0.0%    0.0%    0.0%    0.0%    0.0%    0.0%    0.0%    0.0%    0.0%    0.0%    0.0%    0.0%    0.0%    0.0%    0.0%    0.0%    0.0%    0.0%    0.0%    0.0%    0.0%    0.0%    0.0%    0.0%    0.0%    0.0%    0.0%    0.0%    0.0%    0.0%    0.0%    0.0%    0.0%    0.0%    0.0%    0.0%    0.0%    0.0%    0.0%    0.0%    0.0%    0.0%    0.0%    0.0%    0.0%    0.0%    0.0%    0.0%    0.0%    0.0%    0.0%    0.0%    0.0%    0.0%    0.0%    0.0%    0.0%    0.0%    0.0%    0.0%    0.0%    0.0%    0.0%    0.0%    0.0%    0.0%    0.0%    0.0%    0.0%    0.0%    0.0%    0.0%    0.0%    0.0%    0.0%    0.0%    0.0%    0.0%    0.0%    0.0%    0.0%    0.0%    0.0%    0.1%    0.0%    0.0%    0.0%    0.0%    0.0%    0.0%    0.0%    0.0%    0.0%    0.0%    0.0%    0.0%    0.0%    0.0%    0.0%    0.0%    0.0%    0.0%    0.0%    0.0%    0.0%    0.0%    0.0%    0.0%    0.0%    0.0%    0.0%    0.0%    0.0%    0.0%    0.0%    0.0%    0.0%    0.0%    0.0%    0.0%    0.0%    0.0%    0.0%    0.0%    0.0%    0.0%    0.0%    0.0%    0.0%    0.0%    0.0%    0.0%    0.0%    0.0%    0.0%    0.0%    0.0%    0.0%    0.0%    0.0%    0.0%    0.0%    0.0%    0.0%        k__Bacteria;p__Spirochaetes; c__Brachyspirae       3    0.0%    0.0%    0.0%    0.0%    0.0%    0.0%    0.0%    0.0%    0.0%    0.0%    0.0%    0.0%    0.0%    0.0%    0.0%    0.0%    0.0%    0.0%    0.0%    0.0%    0.0%    0.0%    0.0%    0.0%    0.0%    0.0%    0.0%    0.0%    0.0%    0.0%    0.0%    0.0%    0.0%    0.0%    0.0%    0.0%    0.0%    0.0%    0.0%    0.0%    0.0%    0.0%    0.0%    0.0%    0.0%    0.0%    0.0%    0.0%    0.0%    0.0%    0.0%    0.0%    0.0%    0.0%    0.0%    0.0%    0.0%    0.0%    0.0%    0.0%    0.0%    0.0%    0.0%    0.0%    0.0%    0.0%    0.0%    0.0%    0.0%    0.0%    0.0%    0.0%    0.0%    0.0%    0.0%    0.0%    0.0%    0.0%    0.0%    0.0%    0.0%    0.0%    0.0%    0.0%    0.0%    0.0%    0.0%    0.0%    0.0%    0.0%    0.0%    0.0%    0.0%    0.0%    0.0%    0.0%    0.0%    0.0%    0.0%    0.0%    0.0%    0.0%    0.0%    0.0%    0.0%    0.0%    0.0%    0.0%    0.0%    0.0%    0.0%    0.0%    0.0%    0.0%    0.0%    0.0%    0.0%    0.0%    0.0%    0.0%    0.0%    0.0%    0.0%    0.0%    0.0%    0.0%    0.0%    0.0%    0.0%    0.0%    0.0%    0.0%    0.0%    0.0%    0.0%    0.0%    0.0%    0.0%    0.0%    0.0%    0.0%    0.0%    0.0%    0.0%    0.0%    0.0%    0.0%    0.0%    0.0%    0.0%    0.0%    0.0%    0.0%    0.0%    0.0%    0.0%    0.0%    0.0%    0.0%    0.0%    0.0%    0.0%    0.0%    0.0%    0.0%    0.0%    0.0%    0.0%    0.0%    0.0%    0.0%    0.0%    0.0%    0.0%    0.0%    0.0%    0.0%    0.0%    0.0%    0.0%    0.0%    0.0%    0.0%    0.0%    0.0%    0.0%    0.0%    0.0%    0.0%    0.0%    0.0%    0.0%    0.0%    0.0%    0.0%    0.0%    0.0%    0.0%    0.0%    0.0%    0.0%    0.0%    0.0%    0.0%    0.0%    0.0%    0.0%    0.0%    0.0%    0.0%    0.0%    0.0%    0.0%    0.0%    0.0%    0.0%    0.0%    0.0%    0.0%    0.0%    0.0%    0.0%    0.0%    0.0%    0.0%    0.0%    0.0%    0.0%    0.0%    0.0%    0.0%    0.0%    0.0%    0.0%    0.0%    0.0%    0.0%    0.0%    0.0%    0.0%    0.0%    0.0%    0.0%    0.0%    0.0%    0.0%    0.0%    0.0%    0.0%    0.0%    0.0%    0.0%    0.0%    0.0%    0.0%    0.0%    0.0%    0.0%    0.0%    0.0%    0.0%    0.0%    0.0%    0.0%    0.0%    0.0%    0.0%    0.0%    0.0%    0.0%    0.0%    0.0%    0.0%    0.0%    0.0%    0.0%    0.0%    0.0%    0.0%    0.0%    0.0%    0.0%    0.0%    0.0%    0.0%    0.0%    0.0%    0.0%    0.0%    0.0%    0.0%    0.0%    0.0%    0.0%    0.0%    0.0%    0.0%    0.0%    0.0%    0.0%    0.0%    0.0%    0.0%    0.0%    0.0%    0.0%    0.0%    0.0%    0.0%    0.0%    0.0%    0.0%    0.0%    0.0%    0.0%    0.0%    0.0%    0.0%    0.0%    0.0%    0.0%    0.0%    0.0%    0.0%    0.0%    0.0%    0.0%    0.0%    0.0%    0.0%    0.0%    0.0%    0.0%    0.0%    0.0%    0.0%    0.0%    0.0%    0.0%    0.0%    0.0%    0.0%    0.0%    0.0%    0.0%    0.0%    0.0%    0.0%    0.0%    0.0%    0.0%    0.0%    0.0%    0.0%    0.0%    0.0%    0.0%    0.0%        k__Bacteria;p__Spirochaetes; c__Leptospirae       1    0.0%    0.0%    0.0%    0.0%    0.0%    0.0%    0.0%    0.0%    0.0%    0.0%    0.0%    0.0%    0.0%    0.0%    0.0%    0.0%    0.0%    0.0%    0.0%    0.0%    0.0%    0.0%    0.0%    0.0%    0.0%    0.0%    0.0%    0.0%    0.0%    0.0%    0.0%    0.0%    0.0%    0.0%    0.0%    0.0%    0.0%    0.0%    0.0%    0.0%    0.0%    0.0%    0.0%    0.0%    0.0%    0.0%    0.0%    0.0%    0.0%    0.0%    0.0%    0.0%    0.0%    0.0%    0.0%    0.0%    0.0%    0.0%    0.0%    0.0%    0.0%    0.0%    0.0%    0.0%    0.0%    0.0%    0.0%    0.0%    0.0%    0.0%    0.0%    0.0%    0.0%    0.0%    0.0%    0.0%    0.0%    0.0%    0.0%    0.0%    0.0%    0.0%    0.0%    0.0%    0.0%    0.0%    0.0%    0.0%    0.0%    0.0%    0.0%    0.0%    0.0%    0.0%    0.0%    0.0%    0.0%    0.0%    0.0%    0.0%    0.0%    0.0%    0.0%    0.0%    0.0%    0.0%    0.0%    0.0%    0.0%    0.0%    0.0%    0.0%    0.0%    0.0%    0.0%    0.0%    0.0%    0.0%    0.0%    0.0%    0.0%    0.0%    0.0%    0.0%    0.0%    0.0%    0.0%    0.0%    0.0%    0.0%    0.0%    0.0%    0.0%    0.0%    0.0%    0.0%    0.0%    0.0%    0.0%    0.0%    0.0%    0.0%    0.0%    0.0%    0.0%    0.0%    0.0%    0.0%    0.0%    0.0%    0.0%    0.0%    0.0%    0.0%    0.0%    0.0%    0.0%    0.0%    0.0%    0.0%    0.0%    0.0%    0.0%    0.0%    0.0%    0.0%    0.0%    0.0%    0.0%    0.0%    0.0%    0.0%    0.0%    0.0%    0.0%    0.0%    0.0%    0.0%    0.0%    0.0%    0.0%    0.0%    0.0%    0.0%    0.0%    0.0%    0.0%    0.0%    0.0%    0.0%    0.0%    0.0%    0.0%    0.0%    0.0%    0.0%    0.0%    0.0%    0.0%    0.0%    0.0%    0.0%    0.0%    0.0%    0.0%    0.0%    0.0%    0.0%    0.0%    0.0%    0.0%    0.0%    0.0%    0.0%    0.0%    0.0%    0.0%    0.0%    0.0%    0.0%    0.0%    0.0%    0.0%    0.0%    0.0%    0.0%    0.0%    0.0%    0.0%    0.0%    0.0%    0.0%    0.0%    0.0%    0.0%    0.0%    0.0%    0.0%    0.0%    0.0%    0.0%    0.0%    0.0%    0.0%    0.0%    0.0%    0.0%    0.0%    0.0%    0.0%    0.0%    0.0%    0.0%    0.0%    0.0%    0.0%    0.0%    0.0%    0.0%    0.0%    0.0%    0.0%    0.0%    0.0%    0.0%    0.0%    0.0%    0.0%    0.0%    0.0%    0.0%    0.0%    0.0%    0.0%    0.0%    0.0%    0.0%    0.0%    0.0%    0.0%    0.0%    0.0%    0.0%    0.0%    0.0%    0.0%    0.0%    0.0%    0.0%    0.0%    0.0%    0.0%    0.0%    0.0%    0.0%    0.0%    0.0%    0.0%    0.0%    0.0%    0.0%    0.0%    0.0%    0.0%    0.0%    0.0%    0.0%    0.0%    0.0%    0.0%    0.0%    0.0%    0.0%    0.0%    0.0%    0.0%    0.0%    0.0%    0.0%    0.0%    0.0%    0.0%    0.0%    0.0%    0.0%    0.0%    0.0%    0.0%    0.0%    0.0%    0.0%    0.0%    0.0%    0.0%    0.0%    0.0%    0.0%    0.0%    0.0%    0.0%    0.0%    0.0%    0.0%    0.0%    0.0%    0.0%    0.0%    0.0%    0.0%    0.0%    0.0%    0.0%    0.0%    0.0%    0.0%    0.0%    0.0%    0.0%        k__Bacteria;p__Spirochaetes; c__SP_WWE1       4    0.0%    0.0%    0.0%    0.0%    0.0%    0.0%    0.0%    0.0%    0.0%    0.0%    0.0%    0.0%    0.0%    0.0%    0.0%    0.0%    0.0%    0.0%    0.0%    0.0%    0.0%    0.0%    0.0%    0.0%    0.0%    0.0%    0.0%    0.0%    0.0%    0.0%    0.0%    0.0%    0.0%    0.0%    0.0%    0.0%    0.0%    0.0%    0.0%    0.0%    0.0%    0.0%    0.0%    0.0%    0.0%    0.0%    0.0%    0.0%    0.0%    0.0%    0.0%    0.0%    0.0%    0.0%    0.0%    0.0%    0.0%    0.0%    0.0%    0.0%    0.0%    0.0%    0.0%    0.0%    0.0%    0.0%    0.0%    0.0%    0.0%    0.0%    0.0%    0.0%    0.0%    0.0%    0.0%    0.0%    0.0%    0.0%    0.0%    0.0%    0.0%    0.0%    0.0%    0.0%    0.0%    0.0%    0.0%    0.0%    0.0%    0.0%    0.0%    0.0%    0.0%    0.0%    0.0%    0.0%    0.0%    0.0%    0.0%    0.0%    0.0%    0.0%    0.0%    0.0%    0.0%    0.0%    0.0%    0.0%    0.0%    0.0%    0.0%    0.0%    0.0%    0.0%    0.0%    0.0%    0.0%    0.0%    0.0%    0.0%    0.0%    0.0%    0.0%    0.0%    0.0%    0.0%    0.0%    0.0%    0.0%    0.0%    0.0%    0.0%    0.0%    0.0%    0.0%    0.0%    0.0%    0.0%    0.0%    0.0%    0.0%    0.0%    0.0%    0.0%    0.0%    0.0%    0.0%    0.0%    0.0%    0.0%    0.0%    0.0%    0.0%    0.0%    0.0%    0.0%    0.0%    0.0%    0.0%    0.0%    0.0%    0.0%    0.0%    0.0%    0.0%    0.0%    0.0%    0.0%    0.0%    0.0%    0.0%    0.0%    0.0%    0.0%    0.0%    0.0%    0.0%    0.0%    0.0%    0.0%    0.0%    0.0%    0.0%    0.0%    0.0%    0.0%    0.0%    0.0%    0.0%    0.0%    0.0%    0.0%    0.0%    0.0%    0.0%    0.0%    0.0%    0.0%    0.0%    0.0%    0.0%    0.0%    0.0%    0.0%    0.0%    0.0%    0.0%    0.0%    0.0%    0.0%    0.0%    0.0%    0.0%    0.0%    0.0%    0.0%    0.0%    0.0%    0.0%    0.0%    0.0%    0.0%    0.0%    0.0%    0.0%    0.0%    0.0%    0.0%    0.0%    0.0%    0.0%    0.0%    0.0%    0.0%    0.0%    0.0%    0.0%    0.0%    0.0%    0.0%    0.0%    0.0%    0.0%    0.0%    0.0%    0.0%    0.0%    0.0%    0.0%    0.0%    0.0%    0.0%    0.0%    0.0%    0.0%    0.0%    0.0%    0.0%    0.0%    0.0%    0.0%    0.0%    0.0%    0.0%    0.0%    0.0%    0.0%    0.0%    0.0%    0.0%    0.0%    0.0%    0.0%    0.0%    0.0%    0.0%    0.0%    0.0%    0.0%    0.0%    0.0%    0.0%    0.0%    0.0%    0.0%    0.0%    0.0%    0.0%    0.0%    0.0%    0.0%    0.0%    0.0%    0.0%    0.0%    0.0%    0.0%    0.0%    0.0%    0.0%    0.0%    0.0%    0.0%    0.0%    0.0%    0.0%    0.0%    0.0%    0.0%    0.0%    0.0%    0.0%    0.0%    0.0%    0.0%    0.0%    0.0%    0.0%    0.0%    0.0%    0.0%    0.0%    0.0%    0.0%    0.0%    0.0%    0.0%    0.0%    0.0%    0.0%    0.0%    0.0%    0.0%    0.0%    0.0%    0.0%    0.0%    0.0%    0.0%    0.0%    0.0%    0.0%    0.0%    0.0%    0.0%    0.0%    0.0%    0.0%    0.0%    0.0%    0.0%    0.0%    0.0%    0.0%    0.0%    0.0%    0.0%    0.0%        k__Bacteria;p__Spirochaetes; c__Spirochaetes (class)    5984    0.0%    0.1%    0.0%    0.1%    0.1%    0.0%    0.0%    0.0%    0.0%    0.0%    0.0%    0.0%    0.0%    0.1%    0.1%    0.0%    0.1%    0.0%    0.1%    0.0%    0.0%    0.1%    0.0%    0.0%    0.0%    0.0%    0.0%    0.0%    0.1%    0.0%    0.0%    0.1%    0.0%    0.4%    0.2%    0.6%    0.1%    0.0%    0.0%    0.0%    0.0%    0.2%    0.0%    0.1%    0.0%    0.0%    0.0%    0.0%    0.0%    0.0%    0.0%    0.0%    0.0%    0.0%    0.1%    0.0%    0.1%    0.0%    0.0%    0.0%    0.0%    0.0%    0.1%    0.0%    0.0%    0.0%    0.0%    0.1%    0.0%    0.0%    0.9%    0.1%    0.0%    0.0%    0.0%    0.0%    0.0%    0.0%    0.0%    0.0%    0.0%    0.0%    0.0%    0.0%    0.0%    0.0%    0.2%    0.0%    0.0%    0.1%    0.0%    0.0%    0.1%    0.0%    0.0%    0.0%    0.0%    0.0%    0.0%    0.1%    0.0%    0.1%    0.0%    0.0%    0.0%    0.0%    0.1%    0.0%    0.0%    0.0%    0.0%    0.1%    0.1%    0.0%    0.0%    0.0%    0.0%    0.0%    0.0%    0.6%    0.0%    0.0%    0.0%    0.0%    0.0%    0.0%    0.0%    0.0%    0.0%    0.0%    0.0%    0.0%    0.0%    0.0%    0.0%    0.0%    0.0%    0.0%    0.0%    0.0%    0.0%    0.2%    0.0%    0.0%    0.0%    0.0%    0.3%    0.1%    0.1%    0.0%    0.0%    0.0%    0.0%    0.0%    0.0%    0.0%    0.0%    0.0%    0.0%    0.1%    0.0%    0.0%    0.0%    0.0%    0.0%    0.0%    0.0%    0.0%    0.0%    0.0%    0.0%    0.1%    0.0%    0.0%    0.0%    0.0%    0.0%    0.0%    0.0%    0.0%    0.0%    0.0%    0.1%    0.0%    0.0%    0.0%    0.0%    0.0%    0.0%    0.0%    0.0%    0.0%    0.0%    0.0%    0.0%    0.0%    0.0%    0.0%    0.0%    0.0%    0.1%    0.0%    0.0%    0.0%    0.0%    0.0%    0.0%    0.2%    0.0%    0.0%    0.0%    0.0%    0.0%    0.1%    0.0%    0.0%    0.0%    0.0%    0.0%    0.1%    0.0%    0.0%    0.0%    0.0%    0.0%    0.0%    0.0%    0.1%    0.0%    0.0%    0.0%    0.0%    0.0%    0.0%    0.0%    0.0%    0.0%    0.0%    0.0%    0.0%    0.0%    0.0%    0.0%    0.0%    0.0%    0.0%    0.0%    0.0%    0.0%    0.0%    0.0%    0.0%    0.0%    0.0%    0.0%    0.0%    0.0%    0.0%    0.0%    0.0%    0.0%    0.0%    0.0%    0.0%    0.0%    0.0%    0.0%    0.0%    0.0%    0.0%    0.0%    0.0%    0.0%    0.0%    0.0%    0.0%    0.0%    0.0%    0.0%    0.0%    0.0%    0.0%    0.0%    0.0%    0.0%    0.1%    0.0%    0.0%    0.0%    0.0%    0.0%    0.0%    0.1%    0.0%    0.0%    0.0%    0.0%    0.0%    0.0%    0.0%    0.0%    0.0%    0.0%    0.0%    0.0%    0.0%    0.0%    0.0%    0.0%    0.0%    0.0%    0.0%    0.0%    0.0%    0.0%    0.1%    0.0%    0.0%    0.0%    0.0%    0.0%    0.0%    0.0%    0.0%    0.0%    0.0%    0.0%    0.0%    0.0%    0.4%    0.0%    0.1%    0.0%    0.0%    0.0%    0.0%    0.0%    0.0%    0.0%    0.0%    0.0%    0.0%    0.0%    0.1%    0.0%    0.0%    0.0%    0.0%    0.0%    0.0%    0.0%    0.0%    0.0%    0.1%    0.0%    0.0%    0.0%    0.0%        k__Bacteria;p__Synergistetes; c__Synergistia    1745    0.0%    0.0%    0.0%    0.0%    0.0%    0.0%    0.0%    0.0%    0.0%    0.0%    0.0%    0.0%    0.0%    0.0%    0.0%    0.0%    0.0%    0.0%    0.0%    0.0%    0.0%    0.0%    0.0%    0.0%    0.0%    0.0%    0.0%    0.0%    0.0%    0.0%    0.0%    0.0%    0.0%    0.1%    0.0%    0.2%    0.0%    0.0%    0.0%    0.0%    0.0%    0.0%    0.0%    0.0%    0.0%    0.0%    0.0%    0.0%    0.0%    0.0%    0.0%    0.0%    0.0%    0.0%    0.0%    0.0%    0.0%    0.0%    0.0%    0.0%    0.0%    0.0%    0.0%    0.0%    0.0%    0.0%    0.0%    0.0%    0.0%    0.0%    0.1%    0.0%    0.0%    0.0%    0.0%    0.0%    0.0%    0.0%    0.0%    0.0%    0.0%    0.0%    0.0%    0.0%    0.0%    0.0%    0.0%    0.0%    0.0%    0.0%    0.0%    0.0%    0.0%    0.0%    0.0%    0.0%    0.0%    0.0%    0.0%    0.0%    0.0%    0.0%    0.0%    0.0%    0.0%    0.0%    0.0%    0.0%    0.0%    0.0%    0.0%    0.0%    0.0%    0.0%    0.0%    0.0%    0.0%    0.0%    0.0%    0.1%    0.0%    0.0%    0.0%    0.0%    0.0%    0.0%    0.0%    0.0%    0.0%    0.0%    0.0%    0.0%    0.0%    0.0%    0.0%    0.0%    0.0%    0.0%    0.0%    0.0%    0.0%    0.1%    0.0%    0.0%    0.0%    0.0%    0.3%    0.1%    0.0%    0.0%    0.0%    0.0%    0.0%    0.0%    0.0%    0.0%    0.0%    0.0%    0.0%    0.0%    0.0%    0.0%    0.0%    0.0%    0.0%    0.0%    0.0%    0.0%    0.0%    0.0%    0.0%    0.0%    0.0%    0.0%    0.0%    0.0%    0.0%    0.0%    0.0%    0.0%    0.0%    0.0%    0.0%    0.0%    0.0%    0.0%    0.0%    0.1%    0.0%    0.0%    0.0%    0.1%    0.0%    0.0%    0.0%    0.0%    0.0%    0.0%    0.0%    0.0%    0.0%    0.0%    0.0%    0.0%    0.0%    0.0%    0.0%    0.1%    0.0%    0.0%    0.0%    0.0%    0.0%    0.1%    0.0%    0.0%    0.0%    0.0%    0.0%    0.0%    0.0%    0.0%    0.0%    0.0%    0.0%    0.0%    0.0%    0.0%    0.0%    0.0%    0.0%    0.0%    0.0%    0.0%    0.0%    0.0%    0.0%    0.0%    0.0%    0.0%    0.0%    0.0%    0.0%    0.0%    0.0%    0.0%    0.0%    0.0%    0.0%    0.0%    0.0%    0.0%    0.0%    0.0%    0.0%    0.0%    0.0%    0.0%    0.0%    0.0%    0.0%    0.0%    0.0%    0.0%    0.1%    0.0%    0.0%    0.0%    0.0%    0.0%    0.0%    0.0%    0.0%    0.0%    0.0%    0.0%    0.0%    0.0%    0.0%    0.0%    0.0%    0.0%    0.0%    0.0%    0.0%    0.0%    0.0%    0.0%    0.0%    0.0%    0.0%    0.0%    0.0%    0.0%    0.0%    0.0%    0.0%    0.0%    0.1%    0.0%    0.0%    0.0%    0.0%    0.0%    0.0%    0.0%    0.0%    0.0%    0.0%    0.0%    0.0%    0.0%    0.0%    0.0%    0.0%    0.0%    0.0%    0.0%    0.0%    0.0%    0.0%    0.0%    0.0%    0.0%    0.0%    0.0%    0.0%    0.0%    0.0%    0.1%    0.0%    0.0%    0.0%    0.0%    0.0%    0.0%    0.0%    0.2%    0.0%    0.0%    0.0%    0.0%    0.0%    0.0%    0.0%    0.0%    0.0%    0.0%    0.0%    0.0%    0.0%    0.0%    0.0%    0.0%    0.0%    0.0%    0.0%    0.0%        k__Bacteria;p__TM6; c__       4    0.0%    0.0%    0.0%    0.0%    0.0%    0.0%    0.0%    0.0%    0.0%    0.0%    0.0%    0.0%    0.0%    0.0%    0.0%    0.0%    0.0%    0.0%    0.0%    0.0%    0.0%    0.0%    0.0%    0.0%    0.0%    0.0%    0.0%    0.0%    0.0%    0.0%    0.0%    0.0%    0.0%    0.0%    0.0%    0.0%    0.0%    0.0%    0.0%    0.0%    0.0%    0.0%    0.0%    0.0%    0.0%    0.0%    0.0%    0.0%    0.0%    0.0%    0.0%    0.0%    0.0%    0.0%    0.0%    0.0%    0.0%    0.0%    0.0%    0.0%    0.0%    0.0%    0.0%    0.0%    0.0%    0.0%    0.0%    0.0%    0.0%    0.0%    0.0%    0.0%    0.0%    0.0%    0.0%    0.0%    0.0%    0.0%    0.0%    0.0%    0.0%    0.0%    0.0%    0.0%    0.0%    0.0%    0.0%    0.0%    0.0%    0.0%    0.0%    0.0%    0.0%    0.0%    0.0%    0.0%    0.0%    0.0%    0.0%    0.0%    0.0%    0.0%    0.0%    0.0%    0.0%    0.0%    0.0%    0.0%    0.0%    0.0%    0.0%    0.0%    0.0%    0.0%    0.0%    0.0%    0.0%    0.0%    0.0%    0.0%    0.0%    0.0%    0.0%    0.0%    0.0%    0.0%    0.0%    0.0%    0.0%    0.0%    0.0%    0.0%    0.0%    0.0%    0.0%    0.0%    0.0%    0.0%    0.0%    0.0%    0.0%    0.0%    0.0%    0.0%    0.0%    0.0%    0.0%    0.0%    0.0%    0.0%    0.0%    0.0%    0.0%    0.0%    0.0%    0.0%    0.0%    0.0%    0.0%    0.0%    0.0%    0.0%    0.0%    0.0%    0.0%    0.0%    0.0%    0.0%    0.0%    0.0%    0.0%    0.0%    0.0%    0.0%    0.0%    0.0%    0.0%    0.0%    0.0%    0.0%    0.0%    0.0%    0.0%    0.0%    0.0%    0.0%    0.0%    0.0%    0.0%    0.0%    0.0%    0.0%    0.0%    0.0%    0.0%    0.0%    0.0%    0.0%    0.0%    0.0%    0.0%    0.0%    0.0%    0.0%    0.0%    0.0%    0.0%    0.0%    0.0%    0.0%    0.0%    0.0%    0.0%    0.0%    0.0%    0.0%    0.0%    0.0%    0.0%    0.0%    0.0%    0.0%    0.0%    0.0%    0.0%    0.0%    0.0%    0.0%    0.0%    0.0%    0.0%    0.0%    0.0%    0.0%    0.0%    0.0%    0.0%    0.0%    0.0%    0.0%    0.0%    0.0%    0.0%    0.0%    0.0%    0.0%    0.0%    0.0%    0.0%    0.0%    0.0%    0.0%    0.0%    0.0%    0.0%    0.0%    0.0%    0.0%    0.0%    0.0%    0.0%    0.0%    0.0%    0.0%    0.0%    0.0%    0.0%    0.0%    0.0%    0.0%    0.0%    0.0%    0.0%    0.0%    0.0%    0.0%    0.0%    0.0%    0.0%    0.0%    0.0%    0.0%    0.0%    0.0%    0.0%    0.0%    0.0%    0.0%    0.0%    0.0%    0.0%    0.0%    0.0%    0.0%    0.0%    0.0%    0.0%    0.0%    0.0%    0.0%    0.0%    0.0%    0.0%    0.0%    0.0%    0.0%    0.0%    0.0%    0.0%    0.0%    0.0%    0.0%    0.0%    0.0%    0.0%    0.0%    0.0%    0.0%    0.0%    0.0%    0.0%    0.0%    0.0%    0.0%    0.0%    0.0%    0.0%    0.0%    0.0%    0.0%    0.0%    0.0%    0.0%    0.0%    0.0%    0.0%    0.0%    0.0%    0.0%    0.0%    0.0%    0.0%    0.0%    0.0%    0.0%    0.0%    0.0%    0.0%    0.0%    0.0%    0.0%    0.0%    0.0%    0.0%    0.0%    0.0%    0.0%    0.0%        k__Bacteria;p__TM7; c__       3    0.0%    0.0%    0.0%    0.0%    0.0%    0.0%    0.0%    0.0%    0.0%    0.0%    0.0%    0.0%    0.0%    0.0%    0.0%    0.0%    0.0%    0.0%    0.0%    0.0%    0.0%    0.0%    0.0%    0.0%    0.0%    0.0%    0.0%    0.0%    0.0%    0.0%    0.0%    0.0%    0.0%    0.0%    0.0%    0.0%    0.0%    0.0%    0.0%    0.0%    0.0%    0.0%    0.0%    0.0%    0.0%    0.0%    0.0%    0.0%    0.0%    0.0%    0.0%    0.0%    0.0%    0.0%    0.0%    0.0%    0.0%    0.0%    0.0%    0.0%    0.0%    0.0%    0.0%    0.0%    0.0%    0.0%    0.0%    0.0%    0.0%    0.0%    0.0%    0.0%    0.0%    0.0%    0.0%    0.0%    0.0%    0.0%    0.0%    0.0%    0.0%    0.0%    0.0%    0.0%    0.0%    0.0%    0.0%    0.0%    0.0%    0.0%    0.0%    0.0%    0.0%    0.0%    0.0%    0.0%    0.0%    0.0%    0.0%    0.0%    0.0%    0.0%    0.0%    0.0%    0.0%    0.0%    0.0%    0.0%    0.0%    0.0%    0.0%    0.0%    0.0%    0.0%    0.0%    0.0%    0.0%    0.0%    0.0%    0.0%    0.0%    0.0%    0.0%    0.0%    0.0%    0.0%    0.0%    0.0%    0.0%    0.0%    0.0%    0.0%    0.0%    0.0%    0.0%    0.0%    0.0%    0.0%    0.0%    0.0%    0.0%    0.0%    0.0%    0.0%    0.0%    0.0%    0.0%    0.0%    0.0%    0.0%    0.0%    0.0%    0.0%    0.0%    0.0%    0.0%    0.0%    0.0%    0.0%    0.0%    0.0%    0.0%    0.0%    0.0%    0.0%    0.0%    0.0%    0.0%    0.0%    0.0%    0.0%    0.0%    0.0%    0.0%    0.0%    0.0%    0.0%    0.0%    0.0%    0.0%    0.0%    0.0%    0.0%    0.0%    0.0%    0.0%    0.0%    0.0%    0.0%    0.0%    0.0%    0.0%    0.0%    0.0%    0.0%    0.0%    0.0%    0.0%    0.0%    0.0%    0.0%    0.0%    0.0%    0.0%    0.0%    0.0%    0.0%    0.0%    0.0%    0.0%    0.0%    0.0%    0.0%    0.0%    0.0%    0.0%    0.0%    0.0%    0.0%    0.0%    0.0%    0.0%    0.0%    0.0%    0.0%    0.0%    0.0%    0.0%    0.0%    0.0%    0.0%    0.0%    0.0%    0.0%    0.0%    0.0%    0.0%    0.0%    0.0%    0.0%    0.0%    0.0%    0.0%    0.0%    0.0%    0.0%    0.0%    0.0%    0.0%    0.0%    0.0%    0.0%    0.0%    0.0%    0.0%    0.0%    0.0%    0.0%    0.0%    0.0%    0.0%    0.0%    0.0%    0.0%    0.0%    0.0%    0.0%    0.0%    0.0%    0.0%    0.0%    0.0%    0.0%    0.0%    0.0%    0.0%    0.0%    0.0%    0.0%    0.0%    0.0%    0.0%    0.0%    0.0%    0.0%    0.0%    0.0%    0.0%    0.0%    0.0%    0.0%    0.0%    0.0%    0.0%    0.0%    0.0%    0.0%    0.0%    0.0%    0.0%    0.0%    0.0%    0.0%    0.0%    0.0%    0.0%    0.0%    0.0%    0.0%    0.0%    0.0%    0.0%    0.0%    0.0%    0.0%    0.0%    0.0%    0.0%    0.0%    0.0%    0.0%    0.0%    0.0%    0.0%    0.0%    0.0%    0.0%    0.0%    0.0%    0.0%    0.0%    0.0%    0.0%    0.0%    0.0%    0.0%    0.0%    0.0%    0.0%    0.0%    0.0%    0.0%    0.0%    0.0%    0.0%    0.0%    0.0%    0.0%    0.0%    0.0%    0.0%    0.0%    0.0%    0.0%    0.0%    0.0%    0.0%    0.0%        k__Bacteria;p__TM7; c__TM7-1       2    0.0%    0.0%    0.0%    0.0%    0.0%    0.0%    0.0%    0.0%    0.0%    0.0%    0.0%    0.0%    0.0%    0.0%    0.0%    0.0%    0.0%    0.0%    0.0%    0.0%    0.0%    0.0%    0.0%    0.0%    0.0%    0.0%    0.0%    0.0%    0.0%    0.0%    0.0%    0.0%    0.0%    0.0%    0.0%    0.0%    0.0%    0.0%    0.0%    0.0%    0.0%    0.0%    0.0%    0.0%    0.0%    0.0%    0.0%    0.0%    0.0%    0.0%    0.0%    0.0%    0.0%    0.0%    0.0%    0.0%    0.0%    0.0%    0.0%    0.0%    0.0%    0.0%    0.0%    0.0%    0.0%    0.0%    0.0%    0.0%    0.0%    0.0%    0.0%    0.0%    0.0%    0.0%    0.0%    0.0%    0.0%    0.0%    0.0%    0.0%    0.0%    0.0%    0.0%    0.0%    0.0%    0.0%    0.0%    0.0%    0.0%    0.0%    0.0%    0.0%    0.0%    0.0%    0.0%    0.0%    0.0%    0.0%    0.0%    0.0%    0.0%    0.0%    0.0%    0.0%    0.0%    0.0%    0.0%    0.0%    0.0%    0.0%    0.0%    0.0%    0.0%    0.0%    0.0%    0.0%    0.0%    0.0%    0.0%    0.0%    0.0%    0.0%    0.0%    0.0%    0.0%    0.0%    0.0%    0.0%    0.0%    0.0%    0.0%    0.0%    0.0%    0.0%    0.0%    0.0%    0.0%    0.0%    0.0%    0.0%    0.0%    0.0%    0.0%    0.0%    0.0%    0.0%    0.0%    0.0%    0.0%    0.0%    0.0%    0.0%    0.0%    0.0%    0.0%    0.0%    0.0%    0.0%    0.0%    0.0%    0.0%    0.0%    0.0%    0.0%    0.0%    0.0%    0.0%    0.0%    0.0%    0.0%    0.0%    0.0%    0.0%    0.0%    0.0%    0.0%    0.0%    0.0%    0.0%    0.0%    0.0%    0.0%    0.0%    0.0%    0.0%    0.0%    0.0%    0.0%    0.0%    0.0%    0.0%    0.0%    0.0%    0.0%    0.0%    0.0%    0.0%    0.0%    0.0%    0.0%    0.0%    0.0%    0.0%    0.0%    0.0%    0.0%    0.0%    0.0%    0.0%    0.0%    0.0%    0.0%    0.0%    0.0%    0.0%    0.0%    0.0%    0.0%    0.0%    0.0%    0.0%    0.0%    0.0%    0.0%    0.0%    0.0%    0.0%    0.0%    0.0%    0.0%    0.0%    0.0%    0.0%    0.0%    0.0%    0.0%    0.0%    0.0%    0.0%    0.0%    0.0%    0.0%    0.0%    0.0%    0.0%    0.0%    0.0%    0.0%    0.0%    0.0%    0.0%    0.0%    0.0%    0.0%    0.0%    0.0%    0.0%    0.0%    0.0%    0.0%    0.0%    0.0%    0.0%    0.0%    0.0%    0.0%    0.0%    0.0%    0.0%    0.0%    0.0%    0.0%    0.0%    0.0%    0.0%    0.0%    0.0%    0.0%    0.0%    0.0%    0.0%    0.0%    0.0%    0.0%    0.0%    0.0%    0.0%    0.0%    0.0%    0.0%    0.0%    0.0%    0.0%    0.0%    0.0%    0.0%    0.0%    0.0%    0.0%    0.0%    0.0%    0.0%    0.0%    0.0%    0.0%    0.0%    0.0%    0.0%    0.0%    0.0%    0.0%    0.0%    0.0%    0.0%    0.0%    0.0%    0.0%    0.0%    0.0%    0.0%    0.0%    0.0%    0.0%    0.0%    0.0%    0.0%    0.0%    0.0%    0.0%    0.0%    0.0%    0.0%    0.0%    0.0%    0.0%    0.0%    0.0%    0.0%    0.0%    0.0%    0.0%    0.0%    0.0%    0.0%    0.0%    0.0%    0.0%    0.0%    0.0%    0.0%    0.0%    0.0%    0.0%    0.0%    0.0%    0.0%    0.0%    0.0%        k__Bacteria;p__TM7; c__TM7-3     358    0.0%    0.0%    0.0%    0.0%    0.0%    0.0%    0.0%    0.0%    0.0%    0.0%    0.0%    0.0%    0.0%    0.0%    0.0%    0.0%    0.0%    0.0%    0.0%    0.0%    0.0%    0.0%    0.0%    0.0%    0.0%    0.0%    0.0%    0.0%    0.0%    0.0%    0.0%    0.0%    0.0%    0.0%    0.0%    0.0%    0.0%    0.0%    0.0%    0.0%    0.0%    0.0%    0.0%    0.0%    0.0%    0.0%    0.0%    0.0%    0.0%    0.0%    0.0%    0.0%    0.0%    0.0%    0.0%    0.0%    0.0%    0.0%    0.0%    0.0%    0.0%    0.0%    0.0%    0.0%    0.0%    0.0%    0.0%    0.0%    0.0%    0.0%    0.0%    0.0%    0.0%    0.0%    0.0%    0.0%    0.0%    0.0%    0.0%    0.0%    0.0%    0.0%    0.0%    0.0%    0.0%    0.0%    0.0%    0.0%    0.0%    0.0%    0.0%    0.0%    0.0%    0.0%    0.0%    0.0%    0.0%    0.0%    0.0%    0.0%    0.0%    0.0%    0.0%    0.0%    0.0%    0.0%    0.0%    0.0%    0.0%    0.0%    0.0%    0.0%    0.0%    0.0%    0.0%    0.0%    0.0%    0.0%    0.0%    0.0%    0.0%    0.0%    0.0%    0.0%    0.0%    0.0%    0.0%    0.0%    0.0%    0.0%    0.0%    0.0%    0.0%    0.0%    0.0%    0.0%    0.0%    0.0%    0.0%    0.0%    0.0%    0.0%    0.0%    0.0%    0.0%    0.0%    0.0%    0.0%    0.0%    0.0%    0.0%    0.0%    0.0%    0.0%    0.0%    0.0%    0.0%    0.0%    0.0%    0.0%    0.0%    0.0%    0.0%    0.0%    0.0%    0.0%    0.0%    0.0%    0.0%    0.0%    0.0%    0.0%    0.0%    0.0%    0.0%    0.0%    0.0%    0.0%    0.0%    0.0%    0.0%    0.0%    0.0%    0.0%    0.0%    0.0%    0.0%    0.0%    0.0%    0.0%    0.0%    0.0%    0.0%    0.0%    0.0%    0.0%    0.0%    0.0%    0.0%    0.0%    0.0%    0.0%    0.0%    0.0%    0.0%    0.0%    0.0%    0.0%    0.0%    0.0%    0.0%    0.0%    0.0%    0.0%    0.0%    0.0%    0.0%    0.0%    0.0%    0.0%    0.0%    0.0%    0.0%    0.0%    0.0%    0.0%    0.0%    0.0%    0.0%    0.0%    0.0%    0.0%    0.0%    0.0%    0.0%    0.0%    0.0%    0.0%    0.0%    0.0%    0.0%    0.0%    0.0%    0.0%    0.0%    0.0%    0.0%    0.0%    0.0%    0.0%    0.0%    0.0%    0.0%    0.0%    0.0%    0.0%    0.0%    0.0%    0.0%    0.0%    0.0%    0.0%    0.0%    0.0%    0.0%    0.0%    0.0%    0.0%    0.0%    0.0%    0.0%    0.0%    0.0%    0.0%    0.0%    0.0%    0.0%    0.0%    0.0%    0.0%    0.0%    0.0%    0.0%    0.0%    0.0%    0.0%    0.0%    0.0%    0.0%    0.0%    0.0%    0.0%    0.0%    0.0%    0.0%    0.0%    0.0%    0.0%    0.0%    0.0%    0.0%    0.0%    0.0%    0.0%    0.0%    0.0%    0.0%    0.0%    0.0%    0.0%    0.0%    0.0%    0.0%    0.0%    0.0%    0.0%    0.0%    0.0%    0.0%    0.0%    0.0%    0.0%    0.0%    0.0%    0.0%    0.0%    0.0%    0.0%    0.0%    0.0%    0.0%    0.0%    0.0%    0.0%    0.0%    0.0%    0.0%    0.0%    0.0%    0.0%    0.0%    0.0%    0.0%    0.0%    0.0%    0.0%    0.0%    0.0%    0.0%    0.0%    0.0%    0.0%    0.0%    0.0%    0.0%    0.0%    0.0%    0.0%        k__Bacteria;p__Tenericutes; c__       2    0.0%    0.0%    0.0%    0.0%    0.0%    0.0%    0.0%    0.0%    0.0%    0.0%    0.0%    0.0%    0.0%    0.0%    0.0%    0.0%    0.0%    0.0%    0.0%    0.0%    0.0%    0.0%    0.0%    0.0%    0.0%    0.0%    0.0%    0.0%    0.0%    0.0%    0.0%    0.0%    0.0%    0.0%    0.0%    0.0%    0.0%    0.0%    0.0%    0.0%    0.0%    0.0%    0.0%    0.0%    0.0%    0.0%    0.0%    0.0%    0.0%    0.0%    0.0%    0.0%    0.0%    0.0%    0.0%    0.0%    0.0%    0.0%    0.0%    0.0%    0.0%    0.0%    0.0%    0.0%    0.0%    0.0%    0.0%    0.0%    0.0%    0.0%    0.0%    0.0%    0.0%    0.0%    0.0%    0.0%    0.0%    0.0%    0.0%    0.0%    0.0%    0.0%    0.0%    0.0%    0.0%    0.0%    0.0%    0.0%    0.0%    0.0%    0.0%    0.0%    0.0%    0.0%    0.0%    0.0%    0.0%    0.0%    0.0%    0.0%    0.0%    0.0%    0.0%    0.0%    0.0%    0.0%    0.0%    0.0%    0.0%    0.0%    0.0%    0.0%    0.0%    0.0%    0.0%    0.0%    0.0%    0.0%    0.0%    0.0%    0.0%    0.0%    0.0%    0.0%    0.0%    0.0%    0.0%    0.0%    0.0%    0.0%    0.0%    0.0%    0.0%    0.0%    0.0%    0.0%    0.0%    0.0%    0.0%    0.0%    0.0%    0.0%    0.0%    0.0%    0.0%    0.0%    0.0%    0.0%    0.0%    0.0%    0.0%    0.0%    0.0%    0.0%    0.0%    0.0%    0.0%    0.0%    0.0%    0.0%    0.0%    0.0%    0.0%    0.0%    0.0%    0.0%    0.0%    0.0%    0.0%    0.0%    0.0%    0.0%    0.0%    0.0%    0.0%    0.0%    0.0%    0.0%    0.0%    0.0%    0.0%    0.0%    0.0%    0.0%    0.0%    0.0%    0.0%    0.0%    0.0%    0.0%    0.0%    0.0%    0.0%    0.0%    0.0%    0.0%    0.0%    0.0%    0.0%    0.0%    0.0%    0.0%    0.0%    0.0%    0.0%    0.0%    0.0%    0.0%    0.0%    0.0%    0.0%    0.0%    0.0%    0.0%    0.0%    0.0%    0.0%    0.0%    0.0%    0.0%    0.0%    0.0%    0.0%    0.0%    0.0%    0.0%    0.0%    0.0%    0.0%    0.0%    0.0%    0.0%    0.0%    0.0%    0.0%    0.0%    0.0%    0.0%    0.0%    0.0%    0.0%    0.0%    0.0%    0.0%    0.0%    0.0%    0.0%    0.0%    0.0%    0.0%    0.0%    0.0%    0.0%    0.0%    0.0%    0.0%    0.0%    0.0%    0.0%    0.0%    0.0%    0.0%    0.0%    0.0%    0.0%    0.0%    0.0%    0.0%    0.0%    0.0%    0.0%    0.0%    0.0%    0.0%    0.0%    0.0%    0.0%    0.0%    0.0%    0.0%    0.0%    0.0%    0.0%    0.0%    0.0%    0.0%    0.0%    0.0%    0.0%    0.0%    0.0%    0.0%    0.0%    0.0%    0.0%    0.0%    0.0%    0.0%    0.0%    0.0%    0.0%    0.0%    0.0%    0.0%    0.0%    0.0%    0.0%    0.0%    0.0%    0.0%    0.0%    0.0%    0.0%    0.0%    0.0%    0.0%    0.0%    0.0%    0.0%    0.0%    0.0%    0.0%    0.0%    0.0%    0.0%    0.0%    0.0%    0.0%    0.0%    0.0%    0.0%    0.0%    0.0%    0.0%    0.0%    0.0%    0.0%    0.0%    0.0%    0.0%    0.0%    0.0%    0.0%    0.0%    0.0%    0.0%    0.0%    0.0%    0.0%    0.0%    0.0%    0.0%    0.0%    0.0%    0.0%    0.0%    0.0%    0.0%        k__Bacteria;p__Tenericutes; c__Erysipelotrichi   24056    0.1%    0.2%    0.1%    1.2%    0.2%    0.0%    0.0%    0.0%    0.0%    0.0%    0.0%    0.0%    0.1%    0.1%    0.0%    0.0%    0.1%    0.3%    0.1%    0.0%    0.4%    0.3%    0.0%    0.0%    0.0%    0.0%    0.1%    0.6%    0.8%    0.1%    0.0%    0.2%    0.0%    0.1%    0.6%    0.1%    0.3%    0.0%    0.1%    0.1%    0.1%    0.3%    0.1%    0.1%    0.2%    0.2%    0.0%    0.0%    0.0%    0.0%    0.1%    0.0%    0.0%    0.0%    0.2%    0.0%    0.0%    0.0%    0.1%    0.0%    0.0%    0.1%    0.0%    0.2%    2.3%    0.0%    0.0%    0.2%    0.1%    0.2%    0.0%    0.0%    0.1%    0.1%    0.1%    0.0%    0.0%    0.1%    0.2%    0.1%    0.1%    0.0%    0.2%    0.1%    0.1%    0.0%    0.1%    0.0%    0.0%    0.1%    0.0%    0.1%    0.1%    0.0%    0.0%    0.0%    0.1%    0.0%    2.6%    1.7%    0.3%    0.1%    0.1%    0.1%    0.0%    0.0%    0.7%    0.0%    0.0%    0.2%    0.1%    1.6%    0.1%    0.0%    0.5%    0.1%    0.0%    0.0%    0.2%    0.1%    0.0%    0.3%    0.5%    0.1%    0.0%    0.3%    0.2%    0.0%    0.2%    0.6%    0.3%    0.1%    0.0%    0.1%    0.9%    0.0%    0.1%    0.0%    0.0%    0.0%    0.0%    0.2%    0.2%    0.0%    0.1%    0.0%    0.1%    0.0%    0.0%    0.0%    0.1%    0.1%    0.3%    0.0%    0.1%    0.0%    0.2%    0.0%    0.0%    0.2%    0.0%    0.1%    0.0%    0.0%    0.1%    0.0%    0.1%    0.0%    0.1%    0.1%    0.4%    0.4%    0.0%    0.1%    0.0%    0.0%    0.0%    0.1%    0.0%    0.0%    0.0%    0.0%    0.3%    0.1%    0.0%    0.0%    0.0%    0.0%    0.0%    0.1%    0.0%    0.1%    0.0%    0.0%    0.0%    0.1%    0.2%    0.1%    0.0%    0.0%    0.7%    2.1%    0.1%    0.4%    0.3%    0.2%    0.1%    0.1%    0.3%    0.2%    0.1%    0.0%    0.0%    0.1%    0.0%    0.1%    0.2%    0.2%    0.0%    0.1%    0.2%    0.1%    0.0%    0.1%    0.0%    0.2%    0.0%    0.1%    0.1%    0.1%    0.0%    0.0%    0.0%    0.0%    0.1%    0.0%    0.1%    0.0%    0.0%    0.0%    0.0%    0.0%    0.1%    0.1%    0.0%    0.0%    0.0%    0.0%    0.0%    0.0%    0.1%    0.1%    0.0%    0.0%    0.0%    0.0%    0.0%    0.0%    0.0%    0.1%    0.1%    0.1%    0.0%    0.0%    0.0%    0.0%    0.0%    0.1%    0.1%    0.0%    0.1%    0.0%    0.1%    0.0%    0.1%    0.1%    0.0%    0.1%    0.1%    0.0%    0.0%    0.0%    0.0%    0.0%    0.0%    0.0%    0.0%    0.2%    0.0%    0.0%    0.0%    0.0%    0.1%    0.1%    0.0%    0.0%    0.3%    0.1%    0.1%    0.0%    0.1%    0.1%    0.1%    0.1%    0.0%    0.1%    0.1%    0.0%    0.0%    0.1%    0.0%    0.0%    0.0%    0.0%    0.0%    0.1%    0.0%    0.0%    0.0%    0.1%    0.2%    0.0%    0.0%    0.0%    0.1%    0.0%    0.0%    0.0%    0.1%    0.0%    0.0%    0.0%    0.0%    0.0%    0.0%    0.0%    0.0%    0.1%    0.1%    0.0%    0.0%    0.0%    0.0%    0.0%    0.0%    0.0%    0.0%    0.0%    0.0%    0.0%    0.0%    0.0%    0.0%    0.1%    0.0%    0.0%    0.0%    0.0%        k__Bacteria;p__Tenericutes; c__ML615J-28       5    0.0%    0.0%    0.0%    0.0%    0.0%    0.0%    0.0%    0.0%    0.0%    0.0%    0.0%    0.0%    0.0%    0.0%    0.0%    0.0%    0.0%    0.0%    0.0%    0.0%    0.0%    0.0%    0.0%    0.0%    0.0%    0.0%    0.0%    0.0%    0.0%    0.0%    0.0%    0.0%    0.0%    0.0%    0.0%    0.0%    0.0%    0.0%    0.0%    0.0%    0.0%    0.0%    0.0%    0.0%    0.0%    0.0%    0.0%    0.0%    0.0%    0.0%    0.0%    0.0%    0.0%    0.0%    0.0%    0.0%    0.0%    0.0%    0.0%    0.0%    0.0%    0.0%    0.0%    0.0%    0.0%    0.0%    0.0%    0.0%    0.0%    0.0%    0.0%    0.0%    0.0%    0.0%    0.0%    0.0%    0.0%    0.0%    0.0%    0.0%    0.0%    0.0%    0.0%    0.0%    0.0%    0.0%    0.0%    0.0%    0.0%    0.0%    0.0%    0.0%    0.0%    0.0%    0.0%    0.0%    0.0%    0.0%    0.0%    0.0%    0.0%    0.0%    0.0%    0.0%    0.0%    0.0%    0.0%    0.0%    0.0%    0.0%    0.0%    0.0%    0.0%    0.0%    0.0%    0.0%    0.0%    0.0%    0.0%    0.0%    0.0%    0.0%    0.0%    0.0%    0.0%    0.0%    0.0%    0.0%    0.0%    0.0%    0.0%    0.0%    0.0%    0.0%    0.0%    0.0%    0.0%    0.0%    0.0%    0.0%    0.0%    0.0%    0.0%    0.0%    0.0%    0.0%    0.0%    0.0%    0.0%    0.0%    0.0%    0.0%    0.0%    0.0%    0.0%    0.0%    0.0%    0.0%    0.0%    0.0%    0.0%    0.0%    0.0%    0.0%    0.0%    0.0%    0.0%    0.0%    0.0%    0.0%    0.0%    0.0%    0.0%    0.0%    0.0%    0.0%    0.0%    0.0%    0.0%    0.0%    0.0%    0.0%    0.0%    0.0%    0.0%    0.0%    0.0%    0.0%    0.0%    0.0%    0.0%    0.0%    0.0%    0.0%    0.0%    0.0%    0.0%    0.0%    0.0%    0.0%    0.0%    0.0%    0.0%    0.0%    0.0%    0.0%    0.0%    0.0%    0.0%    0.0%    0.0%    0.0%    0.0%    0.0%    0.0%    0.0%    0.0%    0.0%    0.0%    0.0%    0.0%    0.0%    0.0%    0.0%    0.0%    0.0%    0.0%    0.0%    0.0%    0.0%    0.0%    0.0%    0.0%    0.0%    0.0%    0.0%    0.0%    0.0%    0.0%    0.0%    0.0%    0.0%    0.0%    0.0%    0.0%    0.0%    0.0%    0.0%    0.0%    0.0%    0.0%    0.0%    0.0%    0.0%    0.0%    0.0%    0.0%    0.0%    0.0%    0.0%    0.0%    0.0%    0.0%    0.0%    0.0%    0.0%    0.0%    0.0%    0.0%    0.0%    0.0%    0.0%    0.0%    0.0%    0.0%    0.0%    0.0%    0.0%    0.0%    0.0%    0.0%    0.0%    0.0%    0.0%    0.0%    0.0%    0.0%    0.0%    0.0%    0.0%    0.0%    0.0%    0.0%    0.0%    0.0%    0.0%    0.0%    0.0%    0.0%    0.0%    0.0%    0.0%    0.0%    0.0%    0.0%    0.0%    0.0%    0.0%    0.0%    0.0%    0.0%    0.0%    0.0%    0.0%    0.0%    0.0%    0.0%    0.0%    0.0%    0.0%    0.0%    0.0%    0.0%    0.0%    0.0%    0.0%    0.0%    0.0%    0.0%    0.0%    0.0%    0.0%    0.0%    0.0%    0.0%    0.0%    0.0%    0.0%    0.0%    0.0%    0.0%    0.0%    0.0%    0.0%    0.0%    0.0%    0.0%    0.0%    0.0%    0.0%    0.0%    0.0%    0.0%    0.0%    0.0%    0.0%    0.0%    0.0%        k__Bacteria;p__Tenericutes; c__Mollicutes   32298    0.2%    0.0%    0.1%    0.0%    0.0%    0.0%    0.0%    0.0%    0.0%    0.0%    0.0%    0.0%    0.9%    0.1%    0.0%    0.0%    0.3%    0.4%    0.1%    0.0%    0.3%    0.8%    0.0%    0.0%    0.0%    0.2%    0.1%    0.2%    0.7%    0.0%    0.0%    0.1%    0.0%    0.0%    1.1%    0.1%    0.6%    0.0%    0.3%    0.4%    3.1%    1.1%    0.1%    0.1%    0.7%    0.9%    0.1%    0.0%    0.1%    0.1%    0.2%    0.0%    0.0%    0.1%    0.4%    0.3%    0.1%    0.1%    0.7%    0.2%    0.2%    0.2%    0.2%    0.5%    0.3%    0.1%    0.1%    0.6%    0.1%    0.4%    0.1%    0.1%    0.1%    0.1%    0.2%    0.0%    0.0%    0.4%    0.1%    1.3%    0.2%    0.1%    0.4%    0.4%    0.0%    0.0%    0.5%    0.0%    0.1%    0.6%    0.0%    0.1%    0.1%    0.2%    0.2%    0.1%    0.1%    0.0%    0.1%    0.7%    0.2%    0.1%    0.0%    0.1%    0.0%    0.0%    0.7%    0.0%    0.0%    0.1%    0.0%    0.2%    0.0%    0.0%    0.0%    0.0%    0.0%    0.0%    0.1%    0.0%    0.0%    0.0%    0.1%    0.0%    0.0%    0.1%    0.0%    0.0%    0.3%    0.7%    0.2%    0.0%    0.0%    0.0%    0.5%    0.0%    0.1%    0.0%    0.0%    0.0%    0.0%    0.1%    0.5%    0.1%    0.1%    0.1%    0.0%    0.0%    0.1%    0.0%    0.0%    0.1%    0.5%    0.2%    0.0%    0.0%    0.9%    0.1%    0.1%    0.8%    1.4%    0.9%    1.1%    0.2%    0.2%    0.2%    0.7%    0.1%    0.2%    0.1%    0.3%    0.8%    0.1%    0.4%    0.0%    0.0%    0.0%    0.1%    0.0%    0.0%    0.0%    0.0%    0.2%    0.0%    0.1%    0.0%    0.0%    0.0%    0.0%    0.0%    0.0%    0.0%    0.0%    0.0%    0.1%    0.1%    0.3%    0.0%    0.0%    0.0%    0.5%    0.1%    0.1%    0.2%    0.3%    0.2%    0.1%    0.2%    0.6%    0.5%    0.5%    0.0%    0.0%    0.0%    0.0%    0.0%    0.0%    0.0%    0.0%    0.0%    0.1%    0.1%    0.0%    0.1%    0.0%    0.1%    0.1%    0.1%    0.2%    0.2%    0.0%    0.0%    0.1%    0.1%    0.2%    0.0%    0.1%    0.0%    0.0%    0.0%    0.0%    0.0%    0.0%    0.0%    0.0%    0.0%    0.0%    0.0%    0.0%    0.5%    0.4%    0.1%    0.1%    0.0%    0.0%    0.0%    0.1%    0.1%    1.5%    0.1%    0.8%    0.3%    0.8%    0.2%    0.2%    0.0%    0.2%    0.3%    0.1%    0.0%    0.1%    0.1%    0.0%    0.1%    0.0%    0.0%    0.0%    0.0%    0.0%    0.0%    0.0%    0.0%    0.0%    0.0%    0.0%    0.0%    0.0%    0.1%    0.0%    0.0%    0.0%    0.0%    0.0%    0.1%    0.0%    0.0%    0.0%    0.1%    0.0%    0.1%    0.2%    0.0%    0.1%    0.4%    0.3%    0.0%    0.1%    0.1%    0.1%    0.1%    0.0%    0.0%    0.0%    0.0%    0.0%    0.3%    0.0%    0.0%    0.0%    0.0%    0.0%    0.1%    0.0%    0.0%    0.0%    0.0%    0.1%    0.0%    0.1%    0.0%    0.0%    0.0%    0.0%    0.0%    0.0%    0.0%    0.0%    0.0%    0.0%    0.0%    0.0%    0.0%    0.0%    0.1%    0.0%    0.0%    0.0%    0.0%    0.0%    0.0%    0.0%    0.0%    0.0%    0.1%    0.0%    0.0%    0.0%    0.0%        k__Bacteria;p__Thermi; c__Deinococci    8498    0.0%    0.0%    0.0%    0.0%    0.0%    0.0%    0.0%    0.6%    0.0%    0.0%    0.0%    0.5%    0.0%    0.0%    0.0%    0.0%    0.0%    0.1%    0.0%    0.0%    0.0%    0.1%    0.0%    0.0%    0.0%    0.0%    0.0%    0.0%    0.0%    0.0%    0.0%    0.0%    0.0%    0.0%    0.0%    0.0%    0.0%    0.0%    0.0%    0.0%    0.0%    0.0%    0.0%    0.0%    0.0%    0.0%    0.0%    0.0%    0.0%    0.0%    0.0%    0.0%    0.0%    0.0%    0.0%    0.0%    0.0%    0.0%    0.0%    0.0%    0.0%    0.1%    0.0%    0.0%    0.0%    0.1%    0.0%    0.1%    0.0%    0.0%    0.0%    0.0%    0.0%    0.0%    0.1%    0.0%    0.0%    0.0%    0.0%    0.0%    0.0%    0.1%    0.0%    0.0%    0.0%    0.0%    0.0%    0.0%    0.0%    0.0%    0.0%    0.0%    0.0%    0.0%    0.0%    0.1%    0.0%    0.0%    0.0%    0.0%    0.0%    0.0%    0.0%    0.4%    0.1%    0.1%    0.1%    0.0%    0.0%    0.4%    0.1%    0.2%    0.0%    0.0%    0.1%    0.0%    0.0%    0.0%    0.1%    0.1%    0.0%    0.1%    0.1%    0.2%    0.0%    0.1%    0.1%    0.0%    0.1%    0.1%    0.1%    0.0%    0.0%    0.1%    0.2%    0.0%    0.0%    0.0%    0.0%    0.0%    0.0%    0.0%    0.0%    0.0%    0.0%    0.0%    0.0%    0.0%    0.0%    0.0%    0.0%    0.0%    0.1%    0.0%    0.1%    0.0%    0.2%    0.0%    0.0%    0.1%    0.1%    0.2%    0.1%    0.0%    0.0%    0.0%    0.1%    0.2%    0.3%    0.0%    0.4%    0.2%    0.1%    2.1%    0.1%    0.0%    0.0%    0.0%    0.1%    0.1%    0.3%    0.2%    0.0%    0.0%    0.0%    0.1%    0.2%    0.0%    0.0%    0.1%    0.0%    0.1%    0.0%    0.0%    0.0%    0.0%    0.1%    0.0%    0.1%    0.1%    0.1%    0.0%    0.0%    0.1%    0.1%    0.0%    0.0%    0.0%    0.1%    0.0%    0.0%    0.1%    0.0%    0.1%    0.0%    0.1%    0.0%    0.0%    0.0%    0.2%    0.0%    0.0%    0.0%    0.0%    0.0%    0.0%    0.0%    0.0%    0.0%    0.0%    0.0%    0.0%    0.0%    0.0%    0.1%    0.0%    0.0%    0.1%    0.0%    0.0%    0.0%    0.0%    0.0%    0.0%    0.0%    0.0%    0.0%    0.0%    0.0%    0.0%    0.1%    0.0%    0.0%    0.0%    0.0%    0.0%    0.0%    0.0%    0.0%    0.0%    0.0%    0.0%    0.1%    0.0%    0.0%    0.0%    0.0%    0.0%    0.0%    0.0%    0.0%    0.0%    0.2%    0.1%    0.0%    0.0%    0.0%    0.0%    0.0%    0.0%    0.0%    0.0%    0.0%    0.0%    0.1%    0.0%    0.0%    0.3%    0.1%    0.0%    0.0%    0.0%    0.2%    0.1%    0.1%    0.0%    0.1%    0.0%    0.0%    0.1%    0.0%    0.0%    0.0%    0.0%    0.0%    0.0%    0.1%    0.0%    0.0%    0.0%    0.0%    0.0%    0.0%    0.0%    0.0%    0.1%    0.1%    0.1%    0.0%    0.0%    0.0%    0.1%    0.0%    0.0%    0.1%    0.0%    0.0%    0.0%    0.1%    0.1%    0.0%    0.0%    0.0%    0.1%    0.0%    0.0%    0.0%    0.0%    0.0%    0.0%    0.1%    0.0%    0.0%    0.0%    0.1%    0.0%    0.0%    0.0%    0.0%    0.0%    0.1%    0.0%    0.0%    0.2%    0.0%    0.0%    0.0%    0.0%        k__Bacteria;p__Thermotogae; c__Thermotogae (class)     106    0.0%    0.0%    0.0%    0.0%    0.0%    0.0%    0.0%    0.0%    0.0%    0.0%    0.0%    0.0%    0.0%    0.0%    0.0%    0.0%    0.0%    0.0%    0.0%    0.0%    0.0%    0.0%    0.0%    0.0%    0.0%    0.0%    0.0%    0.0%    0.0%    0.0%    0.0%    0.0%    0.0%    0.0%    0.0%    0.0%    0.0%    0.0%    0.0%    0.0%    0.0%    0.0%    0.0%    0.0%    0.0%    0.0%    0.0%    0.0%    0.0%    0.0%    0.0%    0.1%    0.0%    0.0%    0.0%    0.0%    0.0%    0.0%    0.0%    0.0%    0.0%    0.0%    0.0%    0.0%    0.0%    0.0%    0.0%    0.0%    0.0%    0.0%    0.0%    0.0%    0.0%    0.0%    0.0%    0.0%    0.0%    0.0%    0.0%    0.0%    0.0%    0.0%    0.0%    0.0%    0.0%    0.0%    0.0%    0.0%    0.0%    0.0%    0.0%    0.0%    0.0%    0.0%    0.0%    0.0%    0.0%    0.0%    0.0%    0.0%    0.0%    0.0%    0.0%    0.0%    0.0%    0.0%    0.0%    0.0%    0.0%    0.0%    0.0%    0.0%    0.0%    0.0%    0.0%    0.0%    0.0%    0.0%    0.0%    0.0%    0.0%    0.0%    0.0%    0.0%    0.0%    0.0%    0.0%    0.0%    0.0%    0.0%    0.0%    0.0%    0.0%    0.0%    0.0%    0.0%    0.0%    0.0%    0.0%    0.0%    0.0%    0.0%    0.0%    0.0%    0.0%    0.0%    0.0%    0.0%    0.0%    0.0%    0.0%    0.0%    0.0%    0.0%    0.0%    0.0%    0.0%    0.0%    0.0%    0.0%    0.0%    0.0%    0.0%    0.0%    0.0%    0.0%    0.0%    0.0%    0.0%    0.0%    0.0%    0.0%    0.0%    0.0%    0.0%    0.0%    0.0%    0.0%    0.0%    0.0%    0.0%    0.0%    0.0%    0.0%    0.0%    0.0%    0.0%    0.0%    0.0%    0.0%    0.0%    0.0%    0.0%    0.0%    0.0%    0.0%    0.0%    0.0%    0.0%    0.0%    0.0%    0.0%    0.0%    0.0%    0.0%    0.0%    0.0%    0.0%    0.0%    0.0%    0.0%    0.0%    0.0%    0.0%    0.0%    0.0%    0.0%    0.0%    0.0%    0.0%    0.0%    0.0%    0.0%    0.0%    0.0%    0.0%    0.0%    0.0%    0.0%    0.0%    0.0%    0.0%    0.0%    0.0%    0.0%    0.0%    0.0%    0.0%    0.0%    0.0%    0.0%    0.0%    0.0%    0.0%    0.0%    0.0%    0.0%    0.0%    0.0%    0.0%    0.0%    0.0%    0.0%    0.0%    0.0%    0.0%    0.0%    0.0%    0.0%    0.0%    0.0%    0.0%    0.0%    0.0%    0.0%    0.0%    0.0%    0.0%    0.0%    0.0%    0.0%    0.0%    0.0%    0.0%    0.0%    0.0%    0.0%    0.0%    0.0%    0.0%    0.0%    0.0%    0.0%    0.0%    0.0%    0.0%    0.0%    0.0%    0.0%    0.0%    0.0%    0.0%    0.0%    0.0%    0.0%    0.0%    0.0%    0.0%    0.0%    0.0%    0.0%    0.0%    0.0%    0.0%    0.0%    0.0%    0.0%    0.0%    0.0%    0.0%    0.0%    0.0%    0.0%    0.0%    0.0%    0.0%    0.0%    0.0%    0.0%    0.0%    0.0%    0.0%    0.0%    0.0%    0.0%    0.0%    0.0%    0.0%    0.0%    0.0%    0.0%    0.0%    0.0%    0.0%    0.0%    0.0%    0.0%    0.0%    0.0%    0.0%    0.0%    0.0%    0.0%    0.0%    0.0%    0.0%    0.0%    0.0%    0.0%    0.0%    0.0%    0.0%    0.0%    0.0%    0.0%    0.0%    0.0%    0.0%        k__Bacteria;p__Verrucomicrobia; c__      38    0.0%    0.0%    0.0%    0.0%    0.0%    0.0%    0.0%    0.0%    0.0%    0.0%    0.0%    0.0%    0.0%    0.0%    0.0%    0.0%    0.0%    0.0%    0.0%    0.0%    0.0%    0.0%    0.0%    0.0%    0.0%    0.0%    0.0%    0.0%    0.0%    0.0%    0.0%    0.0%    0.0%    0.0%    0.0%    0.0%    0.0%    0.0%    0.0%    0.0%    0.0%    0.0%    0.0%    0.0%    0.0%    0.0%    0.0%    0.0%    0.0%    0.0%    0.0%    0.0%    0.0%    0.0%    0.0%    0.0%    0.0%    0.0%    0.0%    0.0%    0.0%    0.0%    0.0%    0.0%    0.0%    0.0%    0.0%    0.0%    0.0%    0.0%    0.0%    0.0%    0.0%    0.0%    0.0%    0.0%    0.0%    0.0%    0.0%    0.0%    0.0%    0.0%    0.0%    0.0%    0.0%    0.0%    0.0%    0.0%    0.0%    0.0%    0.0%    0.0%    0.0%    0.0%    0.0%    0.0%    0.0%    0.0%    0.0%    0.0%    0.0%    0.0%    0.0%    0.0%    0.0%    0.0%    0.0%    0.0%    0.0%    0.0%    0.0%    0.0%    0.0%    0.0%    0.0%    0.0%    0.0%    0.0%    0.0%    0.0%    0.0%    0.0%    0.0%    0.0%    0.0%    0.0%    0.0%    0.0%    0.0%    0.0%    0.0%    0.0%    0.0%    0.0%    0.0%    0.0%    0.0%    0.0%    0.0%    0.0%    0.0%    0.0%    0.0%    0.0%    0.0%    0.0%    0.0%    0.0%    0.0%    0.0%    0.0%    0.0%    0.0%    0.0%    0.0%    0.0%    0.0%    0.0%    0.0%    0.0%    0.0%    0.0%    0.0%    0.0%    0.0%    0.0%    0.0%    0.0%    0.0%    0.0%    0.0%    0.0%    0.0%    0.0%    0.0%    0.0%    0.0%    0.0%    0.0%    0.0%    0.0%    0.0%    0.0%    0.0%    0.0%    0.0%    0.0%    0.0%    0.0%    0.0%    0.0%    0.0%    0.0%    0.0%    0.0%    0.0%    0.0%    0.0%    0.0%    0.0%    0.0%    0.0%    0.0%    0.0%    0.0%    0.0%    0.0%    0.0%    0.0%    0.0%    0.0%    0.0%    0.0%    0.0%    0.0%    0.0%    0.0%    0.0%    0.0%    0.0%    0.0%    0.0%    0.0%    0.0%    0.0%    0.0%    0.0%    0.0%    0.0%    0.0%    0.0%    0.0%    0.0%    0.0%    0.0%    0.0%    0.0%    0.0%    0.0%    0.0%    0.0%    0.0%    0.0%    0.0%    0.0%    0.0%    0.0%    0.0%    0.0%    0.0%    0.0%    0.0%    0.0%    0.0%    0.0%    0.0%    0.0%    0.0%    0.0%    0.0%    0.0%    0.0%    0.0%    0.0%    0.0%    0.0%    0.0%    0.0%    0.0%    0.0%    0.0%    0.0%    0.0%    0.0%    0.0%    0.0%    0.0%    0.0%    0.0%    0.0%    0.0%    0.0%    0.0%    0.0%    0.0%    0.0%    0.0%    0.0%    0.0%    0.0%    0.0%    0.0%    0.0%    0.0%    0.0%    0.0%    0.0%    0.0%    0.0%    0.0%    0.0%    0.0%    0.0%    0.0%    0.0%    0.0%    0.0%    0.0%    0.0%    0.0%    0.0%    0.0%    0.0%    0.0%    0.0%    0.0%    0.0%    0.0%    0.0%    0.0%    0.0%    0.0%    0.0%    0.0%    0.0%    0.0%    0.0%    0.0%    0.0%    0.0%    0.0%    0.0%    0.0%    0.0%    0.0%    0.0%    0.0%    0.0%    0.0%    0.0%    0.0%    0.0%    0.0%    0.0%    0.0%    0.0%    0.0%    0.0%    0.0%    0.0%    0.0%    0.0%    0.0%    0.0%    0.0%    0.0%    0.0%    0.0%        k__Bacteria;p__Verrucomicrobia; c__Opitutae     403    0.0%    0.0%    0.0%    0.0%    0.0%    0.0%    0.0%    0.0%    0.0%    0.0%    0.0%    0.0%    0.0%    0.0%    0.0%    0.0%    0.0%    0.0%    0.0%    0.0%    0.0%    0.0%    0.0%    0.0%    0.0%    0.0%    0.0%    0.0%    0.0%    0.0%    0.0%    0.0%    0.0%    0.0%    0.0%    0.0%    0.0%    0.0%    0.0%    0.0%    0.0%    0.0%    0.0%    0.0%    0.0%    0.0%    0.0%    0.0%    0.0%    0.0%    0.0%    0.0%    0.0%    0.0%    0.0%    0.0%    0.0%    0.0%    0.0%    0.0%    0.0%    0.0%    0.0%    0.0%    0.0%    0.0%    0.0%    0.0%    0.0%    0.0%    0.0%    0.0%    0.0%    0.0%    0.0%    0.0%    0.0%    0.0%    0.0%    0.0%    0.0%    0.0%    0.0%    0.0%    0.0%    0.0%    0.0%    0.0%    0.0%    0.0%    0.0%    0.0%    0.0%    0.0%    0.0%    0.0%    0.0%    0.0%    0.0%    0.0%    0.0%    0.0%    0.0%    0.0%    0.0%    0.0%    0.0%    0.0%    0.0%    0.0%    0.0%    0.0%    0.0%    0.0%    0.0%    0.0%    0.0%    0.0%    0.0%    0.0%    0.0%    0.0%    0.0%    0.0%    0.0%    0.1%    0.0%    0.0%    0.0%    0.0%    0.0%    0.0%    0.0%    0.0%    0.0%    0.0%    0.0%    0.0%    0.0%    0.0%    0.0%    0.0%    0.0%    0.0%    0.0%    0.0%    0.0%    0.0%    0.0%    0.0%    0.0%    0.0%    0.0%    0.0%    0.0%    0.0%    0.0%    0.0%    0.0%    0.0%    0.0%    0.0%    0.0%    0.0%    0.0%    0.0%    0.0%    0.0%    0.0%    0.0%    0.0%    0.0%    0.0%    0.0%    0.0%    0.0%    0.0%    0.0%    0.0%    0.0%    0.0%    0.0%    0.0%    0.0%    0.0%    0.0%    0.0%    0.0%    0.0%    0.0%    0.0%    0.0%    0.0%    0.0%    0.0%    0.0%    0.0%    0.0%    0.0%    0.0%    0.0%    0.0%    0.0%    0.0%    0.0%    0.0%    0.0%    0.0%    0.0%    0.0%    0.0%    0.0%    0.0%    0.0%    0.0%    0.0%    0.0%    0.0%    0.0%    0.0%    0.0%    0.0%    0.0%    0.0%    0.0%    0.0%    0.0%    0.0%    0.0%    0.0%    0.0%    0.0%    0.0%    0.0%    0.0%    0.0%    0.0%    0.0%    0.0%    0.0%    0.0%    0.0%    0.0%    0.0%    0.0%    0.0%    0.0%    0.0%    0.0%    0.0%    0.0%    0.0%    0.0%    0.0%    0.0%    0.0%    0.0%    0.0%    0.0%    0.0%    0.0%    0.0%    0.0%    0.0%    0.0%    0.0%    0.0%    0.0%    0.0%    0.0%    0.0%    0.0%    0.0%    0.0%    0.0%    0.0%    0.0%    0.0%    0.0%    0.0%    0.0%    0.0%    0.0%    0.0%    0.0%    0.0%    0.0%    0.0%    0.0%    0.0%    0.0%    0.0%    0.0%    0.0%    0.0%    0.0%    0.0%    0.0%    0.0%    0.0%    0.0%    0.0%    0.0%    0.0%    0.0%    0.0%    0.0%    0.0%    0.0%    0.0%    0.0%    0.1%    0.0%    0.0%    0.0%    0.0%    0.0%    0.0%    0.0%    0.0%    0.0%    0.0%    0.0%    0.0%    0.0%    0.0%    0.0%    0.0%    0.0%    0.0%    0.0%    0.0%    0.0%    0.0%    0.0%    0.0%    0.0%    0.0%    0.0%    0.0%    0.0%    0.0%    0.0%    0.0%    0.0%    0.0%    0.0%    0.0%    0.0%    0.0%    0.0%    0.0%    0.0%    0.0%    0.0%    0.0%    0.0%    0.0%        k__Bacteria;p__Verrucomicrobia; c__Spartobacteria    2258    0.0%    0.0%    0.0%    0.0%    0.0%    0.0%    0.0%    0.0%    0.0%    0.0%    0.0%    0.0%    0.0%    0.0%    0.0%    0.0%    0.0%    0.0%    0.0%    0.0%    0.0%    0.0%    0.0%    0.0%    0.0%    0.0%    0.0%    0.0%    0.0%    0.0%    0.0%    0.0%    0.0%    0.0%    0.0%    0.0%    0.0%    0.0%    0.0%    0.0%    0.0%    0.0%    0.0%    0.0%    0.0%    0.0%    0.0%    0.0%    0.0%    0.0%    0.0%    0.0%    0.0%    0.0%    0.0%    0.0%    0.0%    0.0%    0.0%    0.0%    0.0%    0.0%    0.0%    0.1%    0.0%    0.0%    0.0%    0.0%    0.0%    0.0%    0.0%    0.0%    0.0%    0.0%    0.0%    0.0%    0.0%    0.0%    0.0%    0.0%    0.0%    0.0%    0.0%    0.0%    0.0%    0.0%    0.0%    0.0%    0.0%    0.0%    0.0%    0.0%    0.0%    0.0%    0.0%    0.0%    0.0%    0.0%    0.1%    0.0%    0.0%    0.0%    0.0%    0.0%    0.0%    0.0%    0.0%    0.0%    0.0%    0.0%    0.0%    0.0%    0.0%    0.0%    0.0%    0.0%    0.0%    0.0%    0.0%    0.1%    0.0%    0.0%    0.0%    0.0%    0.0%    0.1%    0.1%    0.0%    0.0%    0.1%    0.0%    0.0%    0.0%    0.0%    0.1%    0.0%    0.0%    0.0%    0.0%    0.0%    0.0%    0.0%    0.0%    0.0%    0.0%    0.0%    0.0%    0.0%    0.0%    0.0%    0.0%    0.0%    0.0%    0.0%    0.0%    0.0%    0.0%    0.0%    0.0%    0.0%    0.0%    0.0%    0.0%    0.0%    0.0%    0.0%    0.0%    0.0%    0.0%    0.0%    0.1%    0.0%    0.0%    0.0%    0.0%    0.0%    0.0%    0.0%    0.0%    0.0%    0.0%    0.0%    0.0%    0.0%    0.0%    0.0%    0.0%    0.0%    0.0%    0.0%    0.0%    0.0%    0.0%    0.0%    0.0%    0.0%    0.0%    0.0%    0.0%    0.0%    0.0%    0.0%    0.0%    0.0%    0.0%    0.0%    0.0%    0.0%    0.1%    0.0%    0.0%    0.0%    0.1%    0.0%    0.0%    0.1%    0.0%    0.0%    0.0%    0.0%    0.0%    0.0%    0.0%    0.0%    0.0%    0.0%    0.0%    0.0%    0.0%    0.0%    0.0%    0.0%    0.0%    0.0%    0.0%    0.0%    0.0%    0.0%    0.0%    0.0%    0.0%    0.0%    0.0%    0.0%    0.0%    0.0%    0.1%    0.0%    0.0%    0.0%    0.0%    0.0%    0.0%    0.0%    0.0%    0.0%    0.0%    0.0%    0.0%    0.0%    0.0%    0.0%    0.0%    0.0%    0.0%    0.0%    0.0%    0.0%    0.0%    0.0%    0.0%    0.0%    0.0%    0.0%    0.0%    0.0%    0.0%    0.0%    0.0%    0.0%    0.0%    0.0%    0.0%    0.0%    0.0%    0.0%    0.0%    0.1%    0.0%    0.2%    0.0%    0.0%    0.0%    0.0%    0.0%    0.0%    0.0%    0.0%    0.0%    0.1%    0.0%    0.0%    0.0%    0.0%    0.0%    0.0%    0.0%    0.0%    0.0%    0.0%    0.0%    0.0%    0.0%    0.0%    0.0%    0.0%    0.0%    0.0%    0.0%    0.0%    0.0%    0.0%    0.0%    0.0%    0.0%    0.0%    0.0%    0.0%    0.0%    0.0%    0.0%    0.0%    0.0%    0.0%    0.0%    0.0%    0.0%    0.0%    0.0%    0.0%    0.0%    0.0%    0.0%    0.0%    0.0%    0.0%    0.0%    0.0%    0.0%    0.0%    0.0%    0.0%    0.0%    0.0%    0.0%    0.0%    0.0%    0.0%        k__Bacteria;p__Verrucomicrobia; c__TP21       0    0.0%    0.0%    0.0%    0.0%    0.0%    0.0%    0.0%    0.0%    0.0%    0.0%    0.0%    0.0%    0.0%    0.0%    0.0%    0.0%    0.0%    0.0%    0.0%    0.0%    0.0%    0.0%    0.0%    0.0%    0.0%    0.0%    0.0%    0.0%    0.0%    0.0%    0.0%    0.0%    0.0%    0.0%    0.0%    0.0%    0.0%    0.0%    0.0%    0.0%    0.0%    0.0%    0.0%    0.0%    0.0%    0.0%    0.0%    0.0%    0.0%    0.0%    0.0%    0.0%    0.0%    0.0%    0.0%    0.0%    0.0%    0.0%    0.0%    0.0%    0.0%    0.0%    0.0%    0.0%    0.0%    0.0%    0.0%    0.0%    0.0%    0.0%    0.0%    0.0%    0.0%    0.0%    0.0%    0.0%    0.0%    0.0%    0.0%    0.0%    0.0%    0.0%    0.0%    0.0%    0.0%    0.0%    0.0%    0.0%    0.0%    0.0%    0.0%    0.0%    0.0%    0.0%    0.0%    0.0%    0.0%    0.0%    0.0%    0.0%    0.0%    0.0%    0.0%    0.0%    0.0%    0.0%    0.0%    0.0%    0.0%    0.0%    0.0%    0.0%    0.0%    0.0%    0.0%    0.0%    0.0%    0.0%    0.0%    0.0%    0.0%    0.0%    0.0%    0.0%    0.0%    0.0%    0.0%    0.0%    0.0%    0.0%    0.0%    0.0%    0.0%    0.0%    0.0%    0.0%    0.0%    0.0%    0.0%    0.0%    0.0%    0.0%    0.0%    0.0%    0.0%    0.0%    0.0%    0.0%    0.0%    0.0%    0.0%    0.0%    0.0%    0.0%    0.0%    0.0%    0.0%    0.0%    0.0%    0.0%    0.0%    0.0%    0.0%    0.0%    0.0%    0.0%    0.0%    0.0%    0.0%    0.0%    0.0%    0.0%    0.0%    0.0%    0.0%    0.0%    0.0%    0.0%    0.0%    0.0%    0.0%    0.0%    0.0%    0.0%    0.0%    0.0%    0.0%    0.0%    0.0%    0.0%    0.0%    0.0%    0.0%    0.0%    0.0%    0.0%    0.0%    0.0%    0.0%    0.0%    0.0%    0.0%    0.0%    0.0%    0.0%    0.0%    0.0%    0.0%    0.0%    0.0%    0.0%    0.0%    0.0%    0.0%    0.0%    0.0%    0.0%    0.0%    0.0%    0.0%    0.0%    0.0%    0.0%    0.0%    0.0%    0.0%    0.0%    0.0%    0.0%    0.0%    0.0%    0.0%    0.0%    0.0%    0.0%    0.0%    0.0%    0.0%    0.0%    0.0%    0.0%    0.0%    0.0%    0.0%    0.0%    0.0%    0.0%    0.0%    0.0%    0.0%    0.0%    0.0%    0.0%    0.0%    0.0%    0.0%    0.0%    0.0%    0.0%    0.0%    0.0%    0.0%    0.0%    0.0%    0.0%    0.0%    0.0%    0.0%    0.0%    0.0%    0.0%    0.0%    0.0%    0.0%    0.0%    0.0%    0.0%    0.0%    0.0%    0.0%    0.0%    0.0%    0.0%    0.0%    0.0%    0.0%    0.0%    0.0%    0.0%    0.0%    0.0%    0.0%    0.0%    0.0%    0.0%    0.0%    0.0%    0.0%    0.0%    0.0%    0.0%    0.0%    0.0%    0.0%    0.0%    0.0%    0.0%    0.0%    0.0%    0.0%    0.0%    0.0%    0.0%    0.0%    0.0%    0.0%    0.0%    0.0%    0.0%    0.0%    0.0%    0.0%    0.0%    0.0%    0.0%    0.0%    0.0%    0.0%    0.0%    0.0%    0.0%    0.0%    0.0%    0.0%    0.0%    0.0%    0.0%    0.0%    0.0%    0.0%    0.0%    0.0%    0.0%    0.0%    0.0%    0.0%    0.0%    0.0%    0.0%    0.0%    0.0%    0.0%    0.0%    0.0%    0.0%    0.0%    0.0%    0.0%        k__Bacteria;p__Verrucomicrobia; c__Verrucomicrobiae    5315    0.0%    0.0%    0.0%    0.0%    0.0%    0.0%    0.0%    0.0%    0.0%    0.0%    0.0%    0.1%    0.0%    0.0%    0.0%    0.0%    0.0%    0.0%    0.0%    0.0%    0.0%    0.0%    0.0%    0.4%    0.0%    0.0%    0.0%    0.0%    0.0%    0.0%    0.0%    0.0%    0.0%    0.0%    0.0%    0.0%    0.1%    0.0%    0.0%    0.0%    0.0%    0.0%    0.0%    0.0%    0.0%    0.0%    0.0%    0.0%    0.0%    0.0%    0.0%    0.0%    0.0%    0.0%    0.1%    0.1%    0.1%    0.0%    0.0%    0.1%    0.0%    0.0%    0.0%    0.1%    0.0%    0.1%    0.1%    0.1%    0.0%    0.0%    0.0%    0.0%    0.0%    0.0%    0.1%    0.0%    0.0%    0.1%    0.0%    0.0%    0.0%    0.0%    0.0%    0.0%    0.0%    0.0%    0.0%    0.0%    0.0%    0.0%    0.0%    0.0%    0.0%    0.0%    0.0%    0.0%    0.1%    0.0%    0.0%    0.0%    0.1%    0.0%    0.0%    0.0%    0.0%    0.0%    0.1%    0.0%    0.0%    0.0%    0.0%    0.0%    0.0%    0.0%    0.0%    0.0%    0.0%    0.0%    0.0%    0.5%    0.0%    0.0%    0.1%    0.0%    0.0%    0.6%    0.1%    0.0%    0.1%    0.2%    0.1%    0.0%    0.0%    0.0%    0.1%    0.0%    0.0%    0.0%    0.0%    0.0%    0.0%    0.0%    0.1%    0.0%    0.1%    0.0%    0.0%    0.0%    0.0%    0.0%    0.0%    0.0%    0.1%    0.0%    0.0%    0.0%    0.1%    0.0%    0.0%    0.1%    0.1%    0.1%    0.1%    0.0%    0.0%    0.0%    0.0%    0.0%    0.0%    0.0%    0.1%    0.0%    0.0%    0.0%    0.0%    0.0%    0.0%    0.0%    0.0%    0.0%    0.0%    0.0%    0.0%    0.0%    0.0%    0.0%    0.0%    0.0%    0.0%    0.0%    0.0%    0.0%    0.0%    0.0%    0.0%    0.0%    0.0%    0.0%    0.0%    0.1%    0.0%    0.0%    0.0%    0.1%    0.1%    0.0%    0.0%    0.1%    0.2%    0.0%    0.1%    0.0%    0.2%    0.0%    0.0%    0.0%    0.0%    0.0%    0.0%    0.0%    0.0%    0.0%    0.0%    0.0%    0.0%    0.1%    0.0%    0.0%    0.0%    0.0%    0.0%    0.0%    0.1%    0.0%    0.0%    0.0%    0.1%    0.0%    0.0%    0.0%    0.1%    0.0%    0.0%    0.0%    0.0%    0.0%    0.0%    0.0%    0.0%    0.0%    0.1%    0.1%    0.1%    0.0%    0.0%    0.0%    0.0%    0.0%    0.0%    0.0%    0.0%    0.0%    0.0%    0.0%    0.0%    0.0%    0.0%    0.0%    0.0%    0.0%    0.0%    0.1%    0.1%    0.0%    0.0%    0.0%    0.0%    0.0%    0.0%    0.0%    0.0%    0.0%    0.0%    0.0%    0.0%    0.0%    0.0%    0.2%    0.0%    0.0%    0.0%    0.0%    0.0%    0.1%    0.0%    0.0%    0.1%    0.0%    0.0%    0.0%    0.0%    0.0%    0.0%    0.0%    0.0%    0.0%    0.0%    0.0%    0.0%    0.0%    0.0%    0.0%    0.0%    0.0%    0.0%    0.1%    0.0%    0.0%    0.0%    0.1%    0.0%    0.0%    0.0%    0.0%    0.0%    0.0%    0.0%    0.0%    0.0%    0.0%    0.1%    0.0%    0.0%    0.0%    0.1%    0.0%    0.0%    0.0%    0.1%    0.0%    0.0%    0.0%    0.0%    0.0%    0.0%    0.0%    0.0%    0.0%    0.0%    0.0%    0.0%    0.0%    0.0%    0.0%    0.0%    0.2%    0.5%    0.0%        k__Bacteria;p__WPS-2; c__     101    0.0%    0.0%    0.0%    0.0%    0.0%    0.0%    0.0%    0.0%    0.0%    0.0%    0.0%    0.0%    0.0%    0.0%    0.0%    0.0%    0.0%    0.0%    0.0%    0.0%    0.0%    0.0%    0.0%    0.0%    0.0%    0.0%    0.0%    0.0%    0.0%    0.0%    0.0%    0.0%    0.0%    0.0%    0.0%    0.0%    0.0%    0.0%    0.0%    0.0%    0.0%    0.0%    0.0%    0.0%    0.0%    0.0%    0.0%    0.0%    0.0%    0.0%    0.0%    0.0%    0.0%    0.0%    0.0%    0.0%    0.0%    0.0%    0.0%    0.0%    0.0%    0.0%    0.0%    0.0%    0.0%    0.0%    0.0%    0.0%    0.0%    0.0%    0.0%    0.0%    0.0%    0.0%    0.0%    0.0%    0.0%    0.0%    0.0%    0.0%    0.0%    0.0%    0.0%    0.0%    0.0%    0.0%    0.0%    0.0%    0.0%    0.0%    0.0%    0.0%    0.0%    0.0%    0.0%    0.0%    0.0%    0.0%    0.0%    0.0%    0.0%    0.0%    0.0%    0.0%    0.0%    0.0%    0.0%    0.0%    0.0%    0.0%    0.0%    0.0%    0.0%    0.0%    0.0%    0.0%    0.0%    0.0%    0.0%    0.0%    0.0%    0.0%    0.0%    0.0%    0.0%    0.0%    0.0%    0.0%    0.0%    0.0%    0.0%    0.0%    0.0%    0.0%    0.0%    0.0%    0.0%    0.0%    0.0%    0.0%    0.0%    0.0%    0.0%    0.0%    0.0%    0.0%    0.0%    0.0%    0.0%    0.0%    0.0%    0.0%    0.0%    0.0%    0.0%    0.0%    0.0%    0.0%    0.0%    0.0%    0.0%    0.0%    0.0%    0.0%    0.0%    0.0%    0.0%    0.0%    0.0%    0.0%    0.0%    0.0%    0.0%    0.0%    0.0%    0.0%    0.0%    0.0%    0.0%    0.0%    0.0%    0.0%    0.0%    0.0%    0.0%    0.0%    0.0%    0.0%    0.0%    0.0%    0.0%    0.0%    0.0%    0.0%    0.0%    0.0%    0.0%    0.0%    0.0%    0.0%    0.0%    0.0%    0.0%    0.0%    0.0%    0.0%    0.0%    0.0%    0.0%    0.0%    0.0%    0.0%    0.0%    0.0%    0.0%    0.0%    0.0%    0.0%    0.0%    0.0%    0.0%    0.0%    0.0%    0.0%    0.0%    0.0%    0.0%    0.0%    0.0%    0.0%    0.0%    0.0%    0.0%    0.0%    0.0%    0.0%    0.0%    0.0%    0.0%    0.0%    0.0%    0.0%    0.0%    0.0%    0.0%    0.0%    0.0%    0.0%    0.0%    0.0%    0.0%    0.0%    0.0%    0.0%    0.0%    0.0%    0.0%    0.0%    0.0%    0.0%    0.0%    0.0%    0.0%    0.0%    0.0%    0.0%    0.0%    0.0%    0.0%    0.0%    0.0%    0.0%    0.0%    0.0%    0.0%    0.0%    0.0%    0.0%    0.0%    0.0%    0.0%    0.0%    0.0%    0.0%    0.0%    0.0%    0.0%    0.0%    0.0%    0.0%    0.0%    0.0%    0.0%    0.0%    0.0%    0.0%    0.0%    0.0%    0.0%    0.0%    0.0%    0.0%    0.0%    0.0%    0.0%    0.0%    0.0%    0.0%    0.0%    0.0%    0.0%    0.0%    0.0%    0.0%    0.0%    0.0%    0.0%    0.0%    0.0%    0.0%    0.0%    0.0%    0.0%    0.0%    0.0%    0.0%    0.0%    0.0%    0.0%    0.0%    0.0%    0.0%    0.0%    0.0%    0.0%    0.0%    0.0%    0.0%    0.0%    0.0%    0.0%    0.0%    0.0%    0.0%    0.0%    0.0%    0.0%    0.0%    0.0%    0.0%    0.0%    0.0%    0.0%    0.0%    0.0%    0.0%    0.0%    0.0%        k__Bacteria;p__WS3; c__PRR-12      42    0.0%    0.0%    0.0%    0.0%    0.0%    0.0%    0.0%    0.0%    0.0%    0.0%    0.0%    0.0%    0.0%    0.0%    0.0%    0.0%    0.0%    0.0%    0.0%    0.0%    0.0%    0.0%    0.0%    0.0%    0.0%    0.0%    0.0%    0.0%    0.0%    0.0%    0.0%    0.0%    0.0%    0.0%    0.0%    0.0%    0.0%    0.0%    0.0%    0.0%    0.0%    0.0%    0.0%    0.0%    0.0%    0.0%    0.0%    0.0%    0.0%    0.0%    0.0%    0.0%    0.0%    0.0%    0.0%    0.0%    0.0%    0.0%    0.0%    0.0%    0.0%    0.0%    0.0%    0.0%    0.0%    0.0%    0.0%    0.0%    0.0%    0.0%    0.0%    0.0%    0.0%    0.0%    0.0%    0.0%    0.0%    0.0%    0.0%    0.0%    0.0%    0.0%    0.0%    0.0%    0.0%    0.0%    0.0%    0.0%    0.0%    0.0%    0.0%    0.0%    0.0%    0.0%    0.0%    0.0%    0.0%    0.0%    0.0%    0.0%    0.0%    0.0%    0.0%    0.0%    0.0%    0.0%    0.0%    0.0%    0.0%    0.0%    0.0%    0.0%    0.0%    0.0%    0.0%    0.0%    0.0%    0.0%    0.0%    0.0%    0.0%    0.0%    0.0%    0.0%    0.0%    0.0%    0.0%    0.0%    0.0%    0.0%    0.0%    0.0%    0.0%    0.0%    0.0%    0.0%    0.0%    0.0%    0.0%    0.0%    0.0%    0.0%    0.0%    0.0%    0.0%    0.0%    0.0%    0.0%    0.0%    0.0%    0.0%    0.0%    0.0%    0.0%    0.0%    0.0%    0.0%    0.0%    0.0%    0.0%    0.0%    0.0%    0.0%    0.0%    0.0%    0.0%    0.0%    0.0%    0.0%    0.0%    0.0%    0.0%    0.0%    0.0%    0.0%    0.0%    0.0%    0.0%    0.0%    0.0%    0.0%    0.0%    0.0%    0.0%    0.0%    0.0%    0.0%    0.0%    0.0%    0.0%    0.0%    0.0%    0.0%    0.0%    0.0%    0.0%    0.0%    0.0%    0.0%    0.0%    0.0%    0.0%    0.0%    0.0%    0.0%    0.0%    0.0%    0.0%    0.0%    0.0%    0.0%    0.0%    0.0%    0.0%    0.0%    0.0%    0.0%    0.0%    0.0%    0.0%    0.0%    0.0%    0.0%    0.0%    0.0%    0.0%    0.0%    0.0%    0.0%    0.0%    0.0%    0.0%    0.0%    0.0%    0.0%    0.0%    0.0%    0.0%    0.0%    0.0%    0.0%    0.0%    0.0%    0.0%    0.0%    0.0%    0.0%    0.0%    0.0%    0.0%    0.0%    0.0%    0.0%    0.0%    0.0%    0.0%    0.0%    0.0%    0.0%    0.0%    0.0%    0.0%    0.0%    0.0%    0.0%    0.0%    0.0%    0.0%    0.0%    0.0%    0.0%    0.0%    0.0%    0.0%    0.0%    0.0%    0.0%    0.0%    0.0%    0.0%    0.0%    0.0%    0.0%    0.0%    0.0%    0.0%    0.0%    0.0%    0.0%    0.0%    0.0%    0.0%    0.0%    0.0%    0.0%    0.0%    0.0%    0.0%    0.0%    0.0%    0.0%    0.0%    0.0%    0.0%    0.0%    0.0%    0.0%    0.0%    0.0%    0.0%    0.0%    0.0%    0.0%    0.0%    0.0%    0.0%    0.0%    0.0%    0.0%    0.0%    0.0%    0.0%    0.0%    0.0%    0.0%    0.0%    0.0%    0.0%    0.0%    0.0%    0.0%    0.0%    0.0%    0.0%    0.0%    0.0%    0.0%    0.0%    0.0%    0.0%    0.0%    0.0%    0.0%    0.0%    0.0%    0.0%    0.0%    0.0%    0.0%    0.0%    0.0%    0.0%    0.0%    0.0%    0.0%    0.0%    0.0%    0.0%        k__Bacteria;p__ZB2; c__       1    0.0%    0.0%    0.0%    0.0%    0.0%    0.0%    0.0%    0.0%    0.0%    0.0%    0.0%    0.0%    0.0%    0.0%    0.0%    0.0%    0.0%    0.0%    0.0%    0.0%    0.0%    0.0%    0.0%    0.0%    0.0%    0.0%    0.0%    0.0%    0.0%    0.0%    0.0%    0.0%    0.0%    0.0%    0.0%    0.0%    0.0%    0.0%    0.0%    0.0%    0.0%    0.0%    0.0%    0.0%    0.0%    0.0%    0.0%    0.0%    0.0%    0.0%    0.0%    0.0%    0.0%    0.0%    0.0%    0.0%    0.0%    0.0%    0.0%    0.0%    0.0%    0.0%    0.0%    0.0%    0.0%    0.0%    0.0%    0.0%    0.0%    0.0%    0.0%    0.0%    0.0%    0.0%    0.0%    0.0%    0.0%    0.0%    0.0%    0.0%    0.0%    0.0%    0.0%    0.0%    0.0%    0.0%    0.0%    0.0%    0.0%    0.0%    0.0%    0.0%    0.0%    0.0%    0.0%    0.0%    0.0%    0.0%    0.0%    0.0%    0.0%    0.0%    0.0%    0.0%    0.0%    0.0%    0.0%    0.0%    0.0%    0.0%    0.0%    0.0%    0.0%    0.0%    0.0%    0.0%    0.0%    0.0%    0.0%    0.0%    0.0%    0.0%    0.0%    0.0%    0.0%    0.0%    0.0%    0.0%    0.0%    0.0%    0.0%    0.0%    0.0%    0.0%    0.0%    0.0%    0.0%    0.0%    0.0%    0.0%    0.0%    0.0%    0.0%    0.0%    0.0%    0.0%    0.0%    0.0%    0.0%    0.0%    0.0%    0.0%    0.0%    0.0%    0.0%    0.0%    0.0%    0.0%    0.0%    0.0%    0.0%    0.0%    0.0%    0.0%    0.0%    0.0%    0.0%    0.0%    0.0%    0.0%    0.0%    0.0%    0.0%    0.0%    0.0%    0.0%    0.0%    0.0%    0.0%    0.0%    0.0%    0.0%    0.0%    0.0%    0.0%    0.0%    0.0%    0.0%    0.0%    0.0%    0.0%    0.0%    0.0%    0.0%    0.0%    0.0%    0.0%    0.0%    0.0%    0.0%    0.0%    0.0%    0.0%    0.0%    0.0%    0.0%    0.0%    0.0%    0.0%    0.0%    0.0%    0.0%    0.0%    0.0%    0.0%    0.0%    0.0%    0.0%    0.0%    0.0%    0.0%    0.0%    0.0%    0.0%    0.0%    0.0%    0.0%    0.0%    0.0%    0.0%    0.0%    0.0%    0.0%    0.0%    0.0%    0.0%    0.0%    0.0%    0.0%    0.0%    0.0%    0.0%    0.0%    0.0%    0.0%    0.0%    0.0%    0.0%    0.0%    0.0%    0.0%    0.0%    0.0%    0.0%    0.0%    0.0%    0.0%    0.0%    0.0%    0.0%    0.0%    0.0%    0.0%    0.0%    0.0%    0.0%    0.0%    0.0%    0.0%    0.0%    0.0%    0.0%    0.0%    0.0%    0.0%    0.0%    0.0%    0.0%    0.0%    0.0%    0.0%    0.0%    0.0%    0.0%    0.0%    0.0%    0.0%    0.0%    0.0%    0.0%    0.0%    0.0%    0.0%    0.0%    0.0%    0.0%    0.0%    0.0%    0.0%    0.0%    0.0%    0.0%    0.0%    0.0%    0.0%    0.0%    0.0%    0.0%    0.0%    0.0%    0.0%    0.0%    0.0%    0.0%    0.0%    0.0%    0.0%    0.0%    0.0%    0.0%    0.0%    0.0%    0.0%    0.0%    0.0%    0.0%    0.0%    0.0%    0.0%    0.0%    0.0%    0.0%    0.0%    0.0%    0.0%    0.0%    0.0%    0.0%    0.0%    0.0%    0.0%    0.0%    0.0%    0.0%    0.0%    0.0%    0.0%    0.0%    0.0%    0.0%    0.0%    0.0%    0.0%    0.0%    0.0%    0.0%    0.0%    0.0%    
     
  Taxonomy Summary. Current Level: Order  
     View Figure (.pdf)    View Legend (.pdf)   
   
 
     
 
 

 
 
 
 
 
 
 
 
 
 
 
 
 
 
 
 
 
 
 
 
 
 
 
 
 
 
 
 
 
 
 
 
 
 
 
 
 
 
 
 
 
 
 
 
 
 
 
 
 
 
 
 
 
 
 
 
 
 
 
 
 
 
 
 
 
 
 
 
 
 
 
 
 
 
 
 
 
 
 
 
 
 
 
 
 
 
 
 
 
 
 
 
 
 
 
 
 
 
 
 
 
 
 
 
 
 
 
 
 
 
 
 
 
 
 
 
 
 
 
 
 
 
 
 
 
 
 
 
 
 
 
 
 
 
 
 
 
 
 
 
 
 
 
 
 
 
 
 
 
 
 
 
 
 
 
 
 
 
 
 
 
 
 
 
 
 
 
 
 
 
 
 
 
 
 
 
 
 
 
 
 
 
 
 
 
 
 
 
 
 
 
 
 
 
 
 
 
 
 
 
 
 
 
 
 
 
 
 
 
 
 
 
 
 
 
 
 
 
 
 
 
 
 
 
 
 
 
 
 
 
 
 
 
 
 
 
 
 
 
 
 
 
 
 
 
 
 
 
 
 
 
 
 
 
 
 
 
 
 
 
 
 
 
 
 
 
 
 
 
 
 
 
 
 
 
 
 
 
 
 
 
 
 
 
 
 
 
 
 
 
 
 
 
 
 
 
 
 
 
 
 
 
 
 
 
 
 
 
 
 
 
 
 
 
 
 
 
 
 
 
 
 
 
 
 
 
 
 
 
 
 
 
 
 
 
 
 
 
 
 
 
 
 
 
 
 
 
 
 
 
 
 
 
 
 
 
 
 
 
 
 
 
 
 
 
 
 
 
 
 
 
 
 
 
 
 
 
 
 
 
 
 
 
 
 
 
 
 
 
 
 
 
 
 
 
 
 
 
 
 
 
 
 
 
 
 
 
 
 
 
 
 
 
 
 
 
 
 
 
 
 
 
 
 
 
 
 
 
 
 
 
 
 
 
 
 
 
 
 
 
 
 
 
 
 
 
 
 
 
 
 
 
 
 
 
 
 
 
 
 
 
 
 
 
 
 
 
 
 
 
 
 
 
 
 
 
 
 
 
 
 
 
 
 
 
 
 
 
 
 
 
 
 
 
 
 
 
 
 
 
 
 
 
 
 
 
 
 
 
 
 
 
 
 
 
 
 
 
 
 
 
 
 
 
 
 
 
 
 
 
 
 
 
 
 
 
 
 
 
 
 
 
 
 
 
 
 
 
 
 
 
 
 
 
 
 
 
 
 
 
 
 
 
 
 
 
 
 
 
 
 
 
 
 
 
 
 
 
 
 
 
 
 
 
 
 
 
 
 
 
 
 
 
 
 
 
 
 
 
 
 
 
 
 
 
 
 
 
 
 
 
 
 
 
 
 
 
 
 
 
 
 
 
 
 
 
 
 
 
 
 
 
 
 
 
 
 
 
 
 
 
 
 
 
 
 
 
 
 
 
 
 
 
 
 
 
 
 
 
 
 
 
 
 
 
 
 
 
 
 
 
 
 
 
 
 
 
 
 
 
 
 
 
 
 
 
 
 
 
 
 
 
 
 
 
 
 
 
 
 
 
 
 
 
 
 
 
 
 
 
 
 
 
 
 
 
 
 
 
 
 
 
 
 
 
 
 
 
 
 
 
 
 
 
 
 
 
 
 
 
 
 
 
 
 
 
 
 
 
 
 
 
 
 
 
 
 
 
 
 
 
 
 
 
 
 
 
 
 
 
 
 
 
 
 
 
 
 
 
 
 
 
 
 
 
 
 
 
 
 
 
 
 
 
 
 
 
 
 
 
 
 
 
 
 
 
 
 
 
 
 
 
 
 
 
 
 
 
 
 
 
 
 
 
 
 
 
 
 
 
 
 
 
 
 
 
 
 
 
 
 
 
 
 
 
 
 
 
 
 
 
 
 
 
 
 
 
 
 
 
 
 
 
 
 
 
 
 
 
 
 
 
 
 
 
 
 
 
 
 
 
 
 
 
 
 
 
 
 
 
 
 
 
 
 
 
 
 
 
 
 
 
 
 
 
 
 
 
 
 
 
 
 
 
 
 
 
 
 
 
 
 
 
 
 
 
 
 
 
 
 
 
 
 
 
 
 
 
 
 
 
 
 
 
 
 
 
 
 
 
 
 
 
 
 
 
 
 
 
 
 
 
 
 
 
 
 
 
 
 
 
 
 
 
 
 
 
 
 
 
 
 
 
 
 
 
 
 
 
 
 
 
 
 
 
 
 
 
 
 
 
 
 
 
 
 
 
 
 
 
 
 
 
 
 
 
 
 
 
 
 
 
 
 
 
 
 
 
 
 
 
 
 
 
 
 
 
 
 
 
 
 
 
 
 
 
 
 
 
 
 
 
 
 
 
 
 
 
 
 
 
 
 
 
 
 
 
 
 
 
 
 
 
 
 
 
 
 
 
 
 
 
 
 
 
 
 
 
 
 
 
 
 
 
 
 
 
 
 
 
 
 
 
 
 
 
 
 
 
 
 
 
 
 
 
 
 
 
 
 
 
 
 
 
 
 
 
 
 
 
 
 
 
 
 
 
 
 
 
 
 
 
 
 
 
 
 
 
 
 
 
 
 
 
 
 
 
 
 
 
 
 
 
 
 
 
 
 
 
 
 
 
 
 
 
 
 
 
 
 
 
 
 
 
 
 
 
 
 
 
 
 
 
 
 
 
 
 
 
 
 
 
 
 
 
 
 
 
 
 
 
 
 
 
 
 
 
 
 
 
 
 
 
 
 
 
 
 
 
 
 
 
 
 
 
 
 
 
 
 
 
 
 
 
 
 
 
 
 
 
 
 
 
 
 
 
 
 
 
 
 
 
 
 
 
 
 
 
 
 
 
 
 
 
 
 
 
 
 
 
 
 
 
 
 
 
 
 
 
 
 
 
 
 
 
 
 
 
 
 
 
 
 
 
 
 
 
 
 
 
 
 
 
 
 
 
 
 
 
 
 
 
 
 
 
 
 
 
 
 
 
 
 
 
 
 
 
 
 
 
 
 
 
 
 
 
 
 
 
 
 
 
 
 
 
 
 
 
 
 
 
 
 
 
 
 
 
 
 
 
 
 
 
 
 
 
 
 
 
 
 
 
 
 
 
 
 
 
 
 
 
 
 
 
 
 
 
 
 
 
 
 
 
 
 
 
 
 
 
 
 
 
 
 
 
 
 
 
 
 
 
 
 
 
 
 
 
 
 
 
 
 
 
 
 
 
 
 
 
 
 
 
 
 
 
 
 
 
 
 
 
 
 
 
 
 
 
 
 
 
 
 
 
 
 
 
 
 
 
 
 
 
 
 
 
 
 
 
 
 
 
 
 
 
 
 
 
 
 
 
 
 
 
 
 
 
 
 
 
 
 
 
 
 
 
 
 
 
 
 
 
 
 
 
 
 
 
 
 
 
 
 
 
 
 
 
 
 
 
 
 
 
 
 
 
 
 
 
 
 
 
 
 
 
 
 
 
 
 
 
 
 
 
 
 
 
 
 
 
 
 
 
 
 
 
 
 
 
 
 
 
 
 
 
 
 
 
 
 
 
 
 
 
 
 
 
 
 
 
 
 
 
 
 
 
 
 
 
 
 
 
 
 
 
 
 
 
 
 
 
 
 
 
 
 
 
 
 
 
 
 
 
 
 
 
 
 
 
 
 
 
 
 
 
 
 
 
 
 
 
 
 
 
 
 
 
 
 
 
 
 
 
 
 
 
 
 
 
 
 
 
 
 
 
 
 
 
 
 
 
 
 
 
 
 
 
 
 
 
 
 
 
 
 
 
 
 
 
 
 
 
 
 
 
 
 
 
 
 
 
 
 
 
 
 
 
 
 
 
 
 
 
 
 
 
 
 
 
 
 
 
 
 
 
 
 
 
 
 
 
 
 
 
 
 
 
 
 
 
 
 
 
 
 
 
 
 
 
 
 
 
 
 
 
 
 
 
 
 
 
 
 
 
 
 
 
 
 
 
 
 
 
 
 
 
 
 
 
 
 
 
 
 
 
 
 
 
 
 
 
 
 
 
 
 
 
 
 
 
 
 
 
 
 
 
 
 
 
 
 
 
 
 
 
 
 
 
 
 
 
 
 
 
 
 
 
 
 
 
 
 
 
 
 
 
 
 
 
 
 
 
 
 
 
 
 
 
 
 
 
 
 
 
 
 
 
 
 
 
 
 
 
 
 
 
 
 
 
 
 
 
 
 
 
 
 
 
 
 
 
 
 
 
 
 
 
 
 
 
 
 
 
 
 
 
 
 
 
 
 
 
 
 
 
 
 
 
 
 
 
 
 
 
 
 
 
 
 
 
 
 
 
 
 
 
 
 
 
 
 
 
 
 
 
 
 
 
 
 
 
 
 
 
 
 
 
 
 
 
 
 
 
 
 
 
 
 
 
 
 
 
 
 
 
 
 
 
 
 
 
 
 
 
 
 
 
 
 
 
 
 
 
 
 
 
 
 
 
 
 
 
 
 
 
 
 
 
 
 
 
 
 
 
 
 
 
 
 
 
 
 
 
 
 
 
 
 
 
 
 
 
 
 
 
 
 
 
 
 
 
 
 
 
 
 
 
 
 
 
 
 
 
 
 
 
 
 
 
 
 
 
 
 
 
 
 
 
 
 
 
 
 
 
 
 
 
 
 
 
 
 
 
 
 
 
 
 
 
 
 
 
 
 
 
 
 
 
 
 
 
 
 
 
 
 
 
 
 
 
 
 
 
 
 
 
 
 
 
 
 
 
 
 
 
 
 
 
 
 
 
 
 
 
 
 
 
 
 
 
 
 
 
 
 
 
 
 
 
 
 
 
 
 
 
 
 
 
 
 
 
 
 
 
 
 
 
 
 
 
 
 
 
 
 
 
 
 
 
 
 
 
 
 
 
 
 
 
 
 
 
 
 
 
 
 
 
 
 
 
 
 
 
 
 
 
 
 
 
 
 
 
 
 
 
 
 
 
 
 
 
 
 
 
 
 
 
 
 
 
 
 
 
 
 
 
 
 
 
 
 
 
 
 
 
 
 
 
 
 
 
 
 
 
 
 
 
 
 
 
 
 
 
 
 
 
 
 
 
 
 
 
 
 
 
 
 
 
 
 
 
 
 
 
 
 
 
 
 
 
 
 
 
 
 
 
 
 
 
 
 
 
 
 
 
 
 
 
 
 
 
 
 
 
 
 
 
 
 
 
 
 
 
 
 
 
 
 
 
 
 
 
 
 
 
 
 
 
 
 
 
 
 
 
 
 
 
 
 
 
 
 
 
 
 
 
 
 
 
 
 
 
 
 
 
 
 
 
 
 
 
 
 
 
 
 
 
 
 
 
 
 
 
 
 
 
 
 
 
 
 
 
 
 
 
 
 
 
 
 
 
 
 
 
 
 
 
 
 
 
 
 
 
 
 
 
 
 
 
 
 
 
 
 
 
 
 
 
 
 
 
 
 
 
 
 
 
 
 
 
 
 
 
 
 
 
 
 
 
 
 
 
 
 
 
 
 
 
 
 
 
 
 
 
 
 
 
 
 
 
 
 
 
 
 
 
 
 
 
 
 
 
 
 
 
 
 
 
 
 
 
 
 
 
 
 
 
 
 
 
 
 
 
 
 
 
 
 
 
 
 
 
 
 
 
 
 
 
 
 
 
 
 
 
 
 
 
 
 
 
 
 
 
 
 
 
 
 
 
 
 
 
 
 
 
 
 
 
 
 
 
 
 
 
 
 
 
 
 
 
 
 
 
 
 
 
 
 
 
 
 
 
 
 
 
 
 
 
 
 
 
 
 
 
 
 
 
 
 
 
 
 
 
 
 
 
 
 
 
 
 
 
 
 
 
 
 
 
 
 
 
 
 
 
 
 
 
 
 
 
 
 
 
 
 
 
 
 
 
 
 
 
 
 
 
 
 
 
 
 
 
 
 
 
 
 
 
 
 
 
 
 
 
 
 
 
 
 
 
 
 
 
 
 
 
 
 
 
 
 
 
 
 
 
 
 
 
 
 
 
 
 
 
 
 
 
 
 
 
 
 
 
 
 
 
 
 
 
 
 
 
 
 
 
 
 
 
 
 
 
 
 
 
 
 
 
 
 
 
 
 
 
 
 
 
 
 
 
 
 
 
 
 
 
 
 
 
 
 
 
 
 
 
 
 
 
 
 
 
 
 
 
 
 
 
 
 
 
 
 
 
 
 
 
 
 
 
 
 
 
 
 
 
 
 
 
 
 
 
 
 
 
 
 
 
 
 
 
 
 
 
 
 
 
 
 
 
 
 
 
 
 
 
 
 
 
 
 
 
 
 
 
 
 
 
 
 
 
 
 
 
 
 
 
 
 
 
 
 
 
 
 
 
 
 
 
 
 
 
 
 
 
 
 
 
 
 
 
 
 
 
 
 
 
 
 
 
 
 
 
 
 
 
 
 
 
 
 
 
 
 
 
 
 
 
 
 
 
 
 
 
 
 
 
 
 
 
 
 
 
 
 
 
 
 
 
 
 
 
 
 
 
 
 
 
 
 
 
 
 
 
 
 
 
 
 
 
 
 
 
 
 
 
 
 
 
 
 
 
 
 
 
 
 
 
 
 
 
 
 
 
 
 
 
 
 
 
 
 
 
 
 
 
 
 
 
 
 
 
 
 
 
 
 
 
 
 
 
 
 
 
 
 
 
 
 
 
 
 
 
 
 
 
 
 
 
 
 
 
 
 
 
 
 
 
 
 
 
 
 
 
 
 
 
 
 
 
 
 
 
 
 
 
 
 
 
 
 
 
 
 
 
 
 
 
 
 
 
 
 
 
 
 
 
 
 
 
 
 
 
 
 
 
 
 
 
 
 
 
 
 
 
 
 
 
 
 
 
 
 
 
 
 
 
 
 
 
 
 
 
 
 
 
 
 
 
 
 
 
 
 
 
 
 
 
 
 
 
 
 
 
 
 
 
 
 
 
 
 
 
 
 
 
 
 
 
 
 
 
 
 
 
 
 
 
 
 
 
 
 
 
 
 
 
 
 
 
 
 
 
 
 
 
 
 
 
 
 
 
 
 
 
 
 
 
 
 
 
 
 
 
 
 
 
 
 
 
 
 
 
 
 
 
 
 
 
 
 
 
 
 
 
 
 
 
 
 
 
 
 
 
 
 
 
 
 
 
 
 
 
 
 
 
 
 
 
 
 
 
 
 
 
 
 
 
 
 
 
 
 
 
 
 
 
 
 
 
 
 
 
 
 
 
 
 
 
 
 
 
 
 
 
 
 
 
 
 
 
 
 
 
 
 
 
 
 
 
 
 
 
 
 
 
 
 
 
 
 
 
 
 
 
 
 
 
 
 
 
 
 
 
 
 
 
 
 
 
 
 
 
 
 
 
 
 
 
 
 
 
 
 
 
 
 
 
 
 
 
 
 
 
 
 
 
 
 
 
 
 
 
 
 
 
 
 
 
 
 
 
 
 
 
 
 
 
 
 
 
 
 
 
 
 
 
 
 
 
 
 
 
 
 
 
 
 
 
 
 
 
 
 
 
 
 
 
 
 
 
 
 
 
 
 
 
 
 
 
 
 
 
 
 
 
 
 
 
 
 
 
 
 
 
 
 
 
 
 
 
 
 
 
 
 
 
 
 
 
 
 
 
 
 
 
 
 
 
 
 
 
 
 
 
 
 
 
 
 
 
 
 
 
 
 
 
 
 
 
 
 
 
 
 
 
 
 
 
 
 
 
 
 
 
 
 
 
 
 
 
 
 
 
 
 
 
 
 
 
 
 
 
 
 
 
 
 
 
 
 
 
 
 
 
 
 
 
 
 
 
 
 
 
 
 
 
 
 
 
 
 
 
 
 
 
 
 
 
 
 
 
 
 
 
 
 
 
 
 
 
 
 
 
 
 
 
 
 
 
 
 
 
 
 
 
 
 
 
 
 
 
 
 
 
 
 
 
 
 
 
 
 
 
 
 
 
 
 
 
 
 
 
 
 
 
 
 
 
 
 
 
 
 
 
 
 
 
 
 
 
 
 
 
 
 
 
 
 
 
 
 
 
 
 
 
 
 
 
 
 
 
 
 
 
 
 
 
 
 
 
 
 
 
 
 
 
 
 
 
 
 
 
 
 
 
 
 
 
 
 
 
 
 
 
 
 
 
 
 
 
 
 
 
 
 
 
 
 
 
 
 
 
 
 
 
 
 
 
 
 
 
 
 
 
 
 
 
 
 
 
 
 
 
 
 
 
 
 
 
 
 
 
 
 
 
 
 
 
 
 
 
 
 
 
 
 
 
 
 
 
 
 
 
 
 
 
 
 
 
 
 
 
 
 
 
 
 
 
 
 
 
 
 
 
 
 
 
 
 
 
 
 
 
 
 
 
 
 
 
 
 
 
 
 
 
 
 
 
 
 
 
 
 
 
 
 
 
 
 
 
 
 
 
 
 
 
 
 
 
 
 
 
 
 
 
 
 
 
 
 
 
 
 
 
 
 
 
 
 
 
 
 
 
 
 
 
 
 
 
 
 
 
 
 
 
 
 
 
 
 
 
 
 
 
 
 
 
 
 
 
 
 
 
 
 
 
 
 
 
 
 
 
 
 
 
 
 
 
 
 
 
 
 
 
 
 
 
 
 
 
 
 
 
 
 
 
 
 
 
 
 
 
 
 
 
 
 
 
 
 
 
 
 
 
 
 
 
 
 
 
 
 
 
 
 
 
 
 
 
 
 
 
 
 
 
 
 
 
 
 
 
 
 
 
 
 
 
 
 
 
 
 
 
 
 
 
 
 
 
 
 
 
 
 
 
 
 
 
 
 
 
 
 
 
 
 
 
 
 
 
 
 
 
 
 
 
 
 
 
 
 
 
 
 
 
 
 
 
 
 
 
 
 
 
 
 
 
 
 
 
 
 
 
 
 
 
 
 
 
 
 
 
 
 
 
 
 
 
 
 
 
 
 
 
 
 
 
 
 
 
 
 
 
 
 
 
 
 
 
 
 
 
 
 
 
 
 
 
 
 
 
 
 
 
 
 
 
 
 
 
 
 
 
 
 
 
 
 
 
 
 
 
 
 
 
 
 
 
 
 
 
 
 
 
 
 
 
 
 
 
 
 
 
 
 
 
 
 
 
 
 
 
 
 
 
 
 
 
 
 
 
 
 
 
 
 
 
 
 
 
 
 
 
 
 
 
 
 
 
 
 
 
 
 
 
 
 
 
 
 
 
 
 
 
 
 
 
 
 
 
 
 
 
 
 
 
 
 
 
 
 
 
 
 
 
 
 
 
 
 
 
 
 
 
 
 
 
 
 
 
 
 
 
 
 
 
 
 
 
 
 
 
 
 
 
 
 
 
 
 
 
 
 
 
 
 
 
 
 
 
 
 
 
 
 
 
 
 
 
 
 
 
 
 
 
 
 
 
 
 
 
 
 
 
 
 
 
 
 
 
 
 
 
 
 
 
 
 
 
 
 
 
 
 
 
 
 
 
 
 
 
 
 
 
 
 
 
 
 
 
 
 
 
 
 
 
 
 
 
 
 
 
 
 
 
 
 
 
 
 
 
 
 
 
 
 
 
 
 
 
 
 
 
 
 
 
 
 
 
 
 
 
 
 
 
 
 
 
 
 
 
 
 
 
 
 
 
 
 
 
 
 
 
 
 
 
 
 
 
 
 
 
 
 
 
 
 
 
 
 
 
 
 
 
 
 
 
 
 
 
 
 
 
 
 
 
 
 
 
 
 
 
 
 
 
 
 
 
 
 
 
 
 
 
 
 
 
 
 
 
 
 
 
 
 
 
 
 
 
 
 
 
 
 
 
 
 
 
 
 
 
 
 
 
 
 
 
 
 
 
 
 
 
 
 
 
 
 
 
 
 
 
 
 
 
 
 
 
 
 
 
 
 
 
 
 
 
 
 
 
 
 
 
 
 
 
 
 
 
 
 
 
 
 
 
 
 
 
 
 
 
 
 
 
 
 
 
 
 
 
 
 
 
 
 
 
 
 
 
 
 
 
 
 
 
 
 
 
 
 
 
 
 
 
 
 
 
 
 
 
 
 
 
 
 
 
 
 
 
 
 
 
 
 
 
 
 
 
 
 
 
 
 
 
 
 
 
 
 
 
 
 
 
 
 
 
 
 
 
 
 
 
 
 
 
 
 
 
 
 
 
 
 
 
 
 
 
 
 
 
 
 
 
 
 
 
 
 
 
 
 
 
 
 
 
 
 
 
 
 
 
 
 
 
 
 
 
 
 
 
 
 
 
 
 
 
 
 
 
 
 
 
 
 
 
 
 
 
 
 
 
 
 
 
 
 
 
 
 
 
 
 
 
 
 
 
 
 
 
 
 
 
 
 
 
 
 
 
 
 
 
 
 
 
 
 
 
 
 
 
 
 
 
 
 
 
 
 
 
 
 
 
 
 
 
 
 
 
 
 
 
 
 
 
 
 
 
 
 
 
 
 
 
 
 
 
 
 
 
 
 
 
 
 
 
 
 
 
 
 
 
 
 
 
 
 
 
 
 
 
 
 
 
 
 
 
 
 
 
 
 
 
 
 
 
 
 
 
 
 
 
 
 
 
 
 
 
 
 
 
 
 
 
 
 
 
 
 
 
 
 
 
 
 
 
 
 
 
 
 
 
 
 
 
 
 
 
 
 
 
 
 
 
 
 
 
 
 
 
 
 
 
 
 
 
 
 
 
 
 
 
 
 
 
 
 
 
 
 
 
 
 
 
 
 
 
 
 
 
 
 
 
 
 
 
 
 
 
 
 
 
 
 
 
 
 
 
 
 
 
 
 
 
 
 
 
 
 
 
 
 
 
 
 
 
 
 
 
 
 
 
 
 
 
 
 
 
 
 
 
 
 
 
 
 
 
 
 
 
 
 
 
 
 
 
 
 
 
 
 
 
 
 
 
 
 
 
 
 
 
 
 
 
 
 
 
 
 
 
 
 
 
 
 
 
 
 
 
 
 
 
 
 
 
 
 
 
 
 
 
 
 
 
 
 
 
 
 
 
 
 
 
 
 
 
 
 
 
 
 
 
 
 
 
 
 
 
 
 
 
 
 
 
 
 
 
 
 
 
 
 
 
 
 
 
 
 
 
 
 
 
 
 
 
 
 
 
 
 
 
 
 
 
 
 
 
 
 
 
 
 
 
 
 
 
 
 
 
 
 
 
 
 
 
 
 
 
 
 
 
 
 
 
 
 
 
 
 
 
 
 
 
 
 
 
 
 
 
 
 
 
 
 
 
 
 
 
 
 
 
 
 
 
 
 
 
 
 
 
 
 
 
 
 
 
 
 
 
 
 
 
 
 
 
 
 
 
 
 
 
 
 
 
 
 
 
 
 
 
 
 
 
 
 
 
 
 
 
 
 
 
 
 
 
 
 
 
 
 
 
 
 
 
 
 
 
 
 
 
 
 
 
 
 
 
 
 
 
 
 
 
 
 
 
 
 
 
 
 
 
 
 
 
 
 
 
 
 
 
 
 
 
 
 
 
 
 
 
 
 
 
 
 
 
 
 
 
 
 
 
 
 
 
 
 
 
 
 
 
 
 
 
 
 
 
 
 
 
 
 
 
 
 
 
 
 
 
 
 
 
 
 
 
 
 
 
 
 
 
 
 
 
 
 
 
 
 
 
 
 
 
 
 
 
 
 
 
 
 
 
 
 
 
 
 
 
 
 
 
 
 
 
 
 
 
 
 
 
 
 
 
 
 
 
 
 
 
 
 
 
 
 
 
 
 
 
 
 
 
 
 
 
 
 
 
 
 
 
 
 
 
 
 
 
 
 
 
 
 
 
 
 
 
 
 
 
 
 
 
 
 
 
 
 
 
 
 
 
 
 
 
 
 
 
 
 
 
 
 
 
 
 
 
 
 
 
 
 
 
 
 
 
 
 
 
 
 
 
 
 
 
 
 
 
 
 
 
 
 
 
 
 
 
 
 
 
 
 
 
 
 
 
 
 
 
 
 
 
 
 
 
 
 
 
 
 
 
 
 
 
 
 
 
 
 
 
 
 
 
 
 
 
 
 
 
 
 
 
 
 
 
 
 
 
 
 
 
 
 
 
 
 
 
 
 
 
 
 
 
 
 
 
 
 
 
 
 
 
 
 
 
 
 
 
 
 
 
 
 
 
 
 
 
 
 
 
 
 
 
 
 
 
 
 
 
 
 
 
 
 
 
 
 
 
 
 
 
 
 
 
 
 
 
 
 
 
 
 
 
 
 
 
 
 
 
 
 
 
 
 
 
 
 
 
 
 
 
 
 
 
 
 
 
 
 
 
 
 
 
 
 
 
 
 
 
 
 
 
 
 
 
 
 
 
 
 
 
 
 
 
 
 
 
 
 
 
 
 
 
 
 
 
 
 
 
 
 
 
 
 
 
 
 
 
 
 
 
 
 
 
 
 
 
 
 
 
 
 
 
 
 
 
 
 
 
 
 
 
 
 
 
 
 
 
 
 
 
 
 
 
 
 
 
 
 
 
 
 
 
 
 
 
 
 
 
 
 
 
 
 
 
 
 
 
 
 
 
 
 
 
 
 
 
 
 
 
 
 
 
 
 
 
 
 
 
 
 
 
 
 
 
 
 
 
 
 
 
 
 
 
 
 
 
 
 
 
 
 
 
 
 
 
 
 
 
 
 
 
 
 
 
 
 
 
 
 
 
 
 
 
 
 
 
 
 
 
 
 
 
 
 
 
 
 
 
 
 
 
 
 
 
 
 
 
 
 
 
 
 
 
 
 
 
 
 
 
 
 
 
 
 
 
 
 
 
 
 
 
 
 
 
 
 
 
 
 
 
 
 
 
 
 
 
 
 
 
 
 
 
 
 
 
 
 
 
 
 
 
 
 
 
 
 
 
 
 
 
 
 
 
 
 
 
 
 
 
 
 
 
 
 
 
 
 
 
 
 
 
 
 
 
 
 
 
 
 
 
 
 
 
 
 
 
 
 
 
 
 
 
 
 
 
 
 
 
 
 
 
 
 
 
 
 
 
 
 
 
 
 
 
 
 
 
 
 
 
 
 
 
 
 
 
 
 
 
 
 
 
 
 
 
 
 
 
 
 
 
 
 
 
 
 
 
 
 
 
 
 
 
 
 
 
 
 
 
 
 
 
 
 
 
 
 
 
 
 
 
 
 
 
 
 
 
 
 
 
 
 
 
 
 
 
 
 
 
 
 
 
 
 
 
 
 
 
 
 
 
 
 
 
 
 
 
 
 
 
 
 
 
 
 
 
 
 
 
 
 
 
 
 
 
 
 
 
 
 
 
 
 
 
 
 
 
 
 
 
 
 
 
 
 
 
 
 
 
 
 
 
 
 
 
 
 
 
 
 
 
 
 
 
 
 
 
 
 
 
 
 
 
 
 
 
 
 
 
 
 
 
 
 
 
 
 
 
 
 
 
 
 
 
 
 
 
 
 
 
 
 
 
 
 
 
 
 
 
 
 
 
 
 
 
 
 
 
 
 
 
 
 
 
 
 
 
 
 
 
 
 
 
 
 
 
 
 
 
 
 
 
 
 
 
 
 
 
 
 
 
 
 
 
 
 
 
 
 
 
 
 
 
 
 
 
 
 
 
 
 
 
 
 
 
 
 
 
 
 
 
 
 
 
 
 
 
 
 
 
 
 
 
 
 
 
 
 
 
 
 
 
 
 
 
 
 
 
 
 
 
 
 
 
 
 
 
 
 
 
 
 
 
 
 
 
 
 
 
 
 
 
 
 
 
 
 
 
 
 
 
 
 
 
 
 
 
 
 
 
 
 
 
 
 
 
 
 
 
 
 
 
 
 
 
 
 
 
 
 
 
 
 
 
 
 
 
 
 
 
 
 
 
 
 
 
 
 
 
 
 
 
 
 
 
 
 
 
 
 
 
 
 
 
 
 
 
 
 
 
 
 
 
 
 
 
 
 
 
 
 
 
 
 
 
 
 
 
 
 
 
 
 
 
 
 
 
 
 
 
 
 
 
 
 
 
 
 
 
 
 
 
 
 
 
 
 
 
 
 
 
 
 
 
 
 
 
 
 
 
 
 
 
 
 
 
 
 
 
 
 
 
 
 
 
 
 
 
 
 
 
 
 
 
 
 
 
 
 
 
 
 
 
 
 
 
 
 
 
 
 
 
 
 
 
 
 
 
 
 
 
 
 
 
 
 
 
 
 
 
 
 
 
 
 
 
 
 
 
 
 
 
 
 
 
 
 
 
 
 
 
 
 
 
 
 
 
 
 
 
 
 
 
 
 
 
 
 
 
 
 
 
 
 
 
 
 
 
 
 
 
 
 
 
 
 
 
 
 
 
 
 
 
 
 
 
 
 
 
 
 
 
 
 
 
 
 
 
 
 
 
 
 
 
 
 
 
 
 
 
 
 
 
 
 
 
 
 
 
 
 
 
 
 
 
 
 
 
 
 
 
 
 
 
 
 
 
 
 
 
 
 
 
 
 
 
 
 
 
 
 
 
 
 
 
 
 
 
 
 
 
 
 
 
 
 
 
 
 
 
 
 
 
 
 
 
 
 
 
 
 
 
 
 
 
 
 
 
 
 
 
 
 
 
 
 
 
 
 
 
 
 
 
 
 
 
 
 
 
 
 
 
 
 
 
 
 
 
 
 
 
 
 
 
 
 
 
 
 
 
 
 
 
 
 
 
 
 
 
 
 
 
 
 
 
 
 
 
 
 
 
 
 
 
 
 
 
 
 
 
 
 
 
 
 
 
 
 
 
 
 
 
 
 
 
 
 
 
 
 
 
 
 
 
 
 
 
 
 
 
 
 
 
 
 
 
 
 
 
 
 
 
 
 
 
 
 
 
 
 
 
 
 
 
 
 
 
 
 
 
 
 
 
 
 
 
 
 
 
 
 
 
 
 
 
 
 
 
 
 
 
 
 
 
 
 
 
 
 
 
 
 
 
 
 
 
 
 
 
 
 
 
 
 
 
 
 
 
 
 
 
 
 
 
 
 
 
 
 
 
 
 
 
 
 
 
 
 
 
 
 
 
 
 
 
 
 
 
 
 
 
 
 
 
 
 
 
 
 
 
 
 
 
 
 
 
 
 
 
 
 
 
 
 
 
 
 
 
 
 
 
 
 
 
 
 
 
 
 
 
 
 
 
 
 
 
 
 
 
 
 
 
 
 
 
 
 
 
 
 
 
 
 
 
 
 
 
 
 
 
 
 
 
 
 
 
 
 
 
 
 
 
 
 
 
 
 
 
 
 
 
 
 
 
 
 
 
 
 
 
 
 
 
 
 
 
 
 
 
 
 
 
 
 
 
 
 
 
 
 
 
 
 
 
 
 
 
 
 
 
 
 
 
 
 
 
 
 
 
 
 
 
 
 
 
 
 
 
 
 
 
 
 
 
 
 
 
 
 
 
 
 
 
 
 
 
 
 
 
 
 
 
 
 
 
 
 
 
 
 
 
 
 
 
 
 
 
 
 
 
 
 
 
 
 
 
 
 
 
 
 
 
 
 
 
 
 
 
 
 
 
 
 
 
 
 
 
 
 
 
 
 
 
 
 
 
 
 
 
 
 
 
 
 
 
 
 
 
 
 
 
 
 
 
 
 
 
 
 
 
 
 
 
 
 
 
 
 
 
 
 
 
 
 
 
 
 
 
 
 
 
 
 
 
 
 
 
 
 
 
 
 
 
 
 
 
 
 
 
 
 
 
 
 
 
 
 
 
 
 
 
 
 
 
 
 
 
 
 
 
 
 
 
 
 
 
 
 
 
 
 
 
 
 
 
 
 
 
 
 
 
 
 
 
 
 
 
 
 
 
 
 
 
 
 
 
 
 
 
 
 
 
 
 
 
 
 
 
 
 
 
 
 
 
 
 
 
 
 
 
 
 
 
 
 
 
 
 
 
 
 
 
 
 
 
 
 
 
 
 
 
 
 
 
 
 
 
 
 
 
 
 
 
 
 
 
 
 
 
 
 
 
 
 
 
 
 
 
 
 
 
 
 
 
 
 
 
 
 
 
 
 
 
 
 
 
 
 
 
 
 
 
 
 
 
 
 
 
 
 
 
 
 
 
 
 
 
 
 
 
 
 
 
 
 
 
 
 
 
 
 
 
 
 
 
 
 
 
 
 
 
 
 
 
 
 
 
 
 
 
 
 
 
 
 
 
 
 
 
 
 
 
 
 
 
 
 
 
 
 
 
 
 
 
 
 
 
 
 
 
 
 
 
 
 
 
 
 
 
 
 
 
 
 
 
 
 
 
 
 
 
 
 
 
 
 
 
 
 
 
 
 
 
 
 
 
 
 
 
 
 
 
 
 
 
 
 
 
 
 
 
 
 
 
 
 
 
 
 
 
 
 
 
 
 
 
 
 
 
 
 
 
 
 
 
 
 
 
 
 
 
 
 
 
 
 
 
 
 
 
 
 
 
 
 
 
 
 
 
 
 
 
 
 
 
 
 
 
 
 
 
 
 
 
 
 
 
 
 
 
 
 
 
 
 
 
 
 
 
 
 
 
 
 
 
 
 
 
 
 
 
 
 
 
 
 
 
 
 
 
 
 
 
 
 
 
 
 
 
 
 
 
 
 
 
 
 
 
 
 
 
 
 
 
 
 
 
 
 
 
 
 
 
 
 
 
 
 
 
 
 
 
 
 
 
 
 
 
 
 
 
 
 
 
 
 
 
 
 
 
 
 
 
 
 
 
 
 
 
 
 
 
 
 
 
 
 
 
 
 
 
 
 
 
 
 
 
 
 
 
 
 
 
 
 
 
 
 
 
 
 
 
 
 
 
 
 
 
 
 
 
 
 
 
 
 
 
 
 
 
 
 
 
 
 
 
 
 
 
 
 
 
 
 
 
 
 
 
 
 
 
 
 
 
 
 
 
 
 
 
 
 
 
 
 
 
 
 
 
 
 
 
 
 
 
 
 
 
 
 
 
 
 
 
 
 
 
 
 
 
 
 
 
 
 
 
 
 
 
 
 
 
 
 
 
 
 
 
 
 
 
 
 
 
 
 
 
 
 
 
 
 
 
 
 
 
 
 
 
 
 
 
 
 
 
 
 
 
 
 
 
 
 
 
 
 
 
 
 
 
 
 
 
 
 
 
 
 
 
 
 
 
 
 
 
 
 
 
 
 
 
 
 
 
 
 
 
 
 
 
 
 
 
 
 
 
 
 
 
 
 
 
 
 
 
 
 
 
 
 
 
 
 
 
 
 
 
 
 
 
 
 
 
 
 
 
 
 
 
 
 
 
 
 
 
 
 
 
 
 
 
 
 
 
 
 
 
 
 
 
 
 
 
 
 
 
 
 
 
 
 
 
 
 
 
 
 
 
 
 
 
 
 
 
 
 
 
 
 
 
 
 
 
 
 
 
 
 
 
 
 
 
 
 
 
 
 
 
 
 
 
 
 
 
 
 
 
 
 
 
 
 
 
 
 
 
 
 
 
 
 
 
 
 
 
 
 
 
 
 
 
 
 
 
 
 
 
 
 
 
 
 
 
 
 
 
 
 
 
 
 
 
 
 
 
 
 
 
 
 
 
 
 
 
 
 
 
 
 
 
 
 
 
 
 
 
 
 
 
 
 
 
 
 
 
 
 
 
 
 
 
 
 
 
 
 
 
 
 
 
 
 
 
 
 
 
 
 
 
 
 
 
 
 
 
 
 
 
 
 
 
 
 
 
 
 
 
 
 
 
 
 
 
 
 
 
 
 
 
 
 
 
 
 
 
 
 
 
 
 
 
 
 
 
 
 
 
 
 
 
 
 
 
 
 
 
 
 
 
 
 
 
 
 
 
 
 
 
 
 
 
 
 
 
 
 
 
 
 
 
 
 
 
 
 
 
 
 
 
 
 
 
 
 
 
 
 
 
 
 
 
 
 
 
 
 
 
 
 
 
 
 
 
 
 
 
 
 
 
 
 
 
 
 
 
 
 
 
 
 
 
 
 
 
 
 
 
 
 
 
 
 
 
 
 
 
 
 
 
 
 
 
 
 
 
 
 
 
 
 
 
 
 
 
 
 
 
 
 
 
 
 
 
 
 
 
 
 
 
 
 
 
 
 
 
 
 
 
 
 
 
 
 
 
 
 
 
 
 
 
 
 
 
 
 
 
 
 
 
 
 
 
 
 
 
 
 
 
 
 
 
 
 
 
 
 
 
 
 
 
 
 
 
 
 
 
 
 
 
 
 
 
 
 
 
 
 
 
 
 
 
 
 
 
 
 
 
 
 
 
 
 
 
 
 
 
 
 
 
 
 
 
 
 
 
 
 
 
 
 
 
 
 
 
 
 
 
 
 
 
 
 
 
 
 
 
 
 
 
 
 
 
 
 
 
 
 
 
 
 
 
 
 
 
 
 
 
 
 
 
 
 
 
 
 
 
 
 
 
 
 
 
 
 
 
 
 
 
 
 
 
 
 
 
 
 
 
 
 
 
 
 
 
 
 
 
 
 
 
 
 
 
 
 
 
 
 
 
 
 
 
 
 
 
 
 
 
 
 
 
 
 
 
 
 
 
 
 
 
 
 
 
 
 
 
 
 
 
 
 
 
 
 
 
 
 
 
 
 
 
 
 
 
 
 
 
 
 
 
 
 
 
 
 
 
 
 
 
 
 
 
 
 
 
 
 
 
 
 
 
 
 
 
 
 
 
 
 
 
 
 
 
 
 
 
 
 
 
 
 
 
 
 
 
 
 
 
 
 
 
 
 
 
 
 
 
 
 
 
 
 
 
 
 
 
 
 
 
 
 
 
 
 
 
 
 
 
 
 
 
 
 
 
 
 
 
 
 
 
 
 
 
 
 
 
 
 
 
 
 
 
 
 
 
 
 
 
 
 
 
 
 
 
 
 
 
 
 
 
 
 
 
 
 
 
 
 
 
 
 
 
 
 
 
 
 
 
 
 
 
 
 
 
 
 
 
 
 
 
 
 
 
 
 
 
 
 
 
 
 
 
 
 
 
 
 
 
 
 
 
 
 
 
 
 
 
 
 
 
 
 
 
 
 
 
 
 
 
 
 
 
 
 
 
 
 
 
 
 
 
 
 
 
 
 
 
 
 
 
 
 
 
 
 
 
 
 
 
 
 
 
 
 
 
 
 
 
 
 
 
 
 
 
 
 
 
 
 
 
 
 
 
 
 
 
 
 
 
 
 
 
 
 
 
 
 
 
 
 
 
 
 
 
 
 
 
 
 
 
 
 
 
 
 
 
 
 
 
 
 
 
 
 
 
 
 
 
 
 
 
 
 
 
 
 
 
 
 
 
 
 
 
 
 
 
 
 
 
 
 
 
 
 
 
 
 
 
 
 
 
 
 
 
 
 
 
 
 
 
 
 
 
 
 
 
 
 
 
 
 
 
 
 
 
 
 
 
 
 
 
 
 
 
 
 
 
 
 
 
 
 
 
 
 
 
 
 
 
 
 
 
 
 
 
 
 
 
 
 
 
 
 
 
 
 
 
 
 
 
 
 
 
 
 
 
 
 
 
 
 
 
 
 
 
 
 
 
 
 
 
 
 
 
 
 
 
 
 
 
 
 
 
 
 
 
 
 
 
 
 
 
 
 
 
 
 
 
 
 
 
 
 
 
 
 
 
 
 
 
 
 
 
 
 
 
 
 
 
 
 
 
 
 
 
 
 
 
 
 
 
 
 
 
 
 
 
 
 
 
 
 
 
 
 
 
 
 
 
 
 
 
 
 
 
 
 
 
 
 
 
 
 
 
 
 
 
 
 
 
 
 
 
 
 
 
 
 
 
 
 
 
 
 
 
 
 
 
 
 
 
 
 
 
 
 
 
 
 
 
 
 
 
 
 
 
 
 
 
 
 
 
 
 
 
 
 
 
 
 
 
 
 
 
 
 
 
 
 
 
 
 
 
 
 
 
 
 
 
 
 
 
 
 
 
 
 
 
 
 
 
 
 
 
 
 
 
 
 
 
 
 
 
 
 
 
 
 
 
 
 
 
 
 
 
 
 
 
 
 
 
 
 
 
 
 
 
 
 
 
 
 
 
 
 
 
 
 
 
 
 
 
 
 
 
 
 
 
 
 
 
 
 
 
 
 
 
 
 
 
 
 
 
 
 
 
 
 
 
 
 
 
 
 
 
 
 
 
 
 
 
 
 
 
 
 
 
 
 
 
 
 
 
 
 
 
 
 
 
 
 
 
 
 
 
 
 
 
 
 
 
 
 
 
 
 
 
 
 
 
 
 
 
 
 
 
 
 
 
 
 
 
 
 
 
 
 
 
 
 
 
 
 
 
 
 
 
 
 
 
 
 
 
 
 
 
 
 
 
 
 
 
 
 
 
 
 
 
 
 
 
 
 
 
 
 
 
 
 
 
 
 
 
 
 
 
 
 
 
 
 
 
 
 
 
 
 
 
 
 
 
 
 
 
 
 
 
 
 
 
 
 
 
 
 
 
 
 
 
 
 
 
 
 
 
 
 
 
 
 
 
 
 
 
 
 
 
 
 
 
 
 
 
 
 
 
 
 
 
 
 
 
 
 
 
 
 
 
 
 
 
 
 
 
 
 
 
 
 
 
 
 
 
 
 
 
 
 
 
 
 
 
 
 
 
 
 
 
 
 
 
 
 
 
 
 
 
 
 
 
 
 
 
 
 
 
 
 
 
 
 
 
 
 
 
 
 
 
 
 
 
 
 
 
 
 
 
 
 
 
 
 
 
 
 
 
 
 
 
 
 
 
 
 
 
 
 
 
 
 
 
 
 
 
 
 
 
 
 
 
 
 
 
 
 
 
 
 
 
 
 
 
 
 
 
 
 
 
 
 
 
 
 
 
 
 
 
 
 
 
 
 
 
 
 
 
 
 
 
 
 
 
 
 
 
 
 
 
 
 
 
 
 
 
 
 
 
 
 
 
 
 
 
 
 
 
 
 
 
 
 
 
 
 
 
 
 
 
 
 
 
 
 
 
 
 
 
 
 
 
 
 
 
 
 
 
 
 
 
 
 
 
 
 
 
 
 
 
 
 
 
 
 
 
 
 
 
 
 
 
 
 
 
 
 
 
 
 
 
 
 
 
 
 
 
 
 
 
 
 
 
 
 
 
 
 
 
 
 
 
 
 
 
 
 
 
 
 
 
 
 
 
 
 
 
 
 
 
 
 
 
 
 
 
 
 
 
 
 
 
 
 
 
 
 
 
 
 
 
 
 
 
 
 
 
 
 
 
 
 
 
 
 
 
 
 
 
 
 
 
 
 
 
 
 
 
 
 
 
 
 
 
 
 
 
 
 
 
 
 
 
 
 
 
 
 
 
 
 
 
 
 
 
 
 
 
 
 
 
 
 
 
 
 
 
 
 
 
 
 
 
 
 
 
 
 
 
 
 
 
 
 
 
 
 
 
 
 
 
 
 
 
 
 
 
 
 
 
 
 
 
 
 
 
 
 
 
 
 
 
 
 
 
 
 
 
 
 
 
 
 
 
 
 
 
 
 
 
 
 
 
 
 
 
 
 
 
 
 
 
 
 
 
 
 
 
 
 
 
 
 
 
 
 
 
 
 
 
 
 
 
 
 
 
 
 
 
 
 
 
 
 
 
 
 
 
 
 
 
 
 
 
 
 
 
 
 
 
 
 
 
 
 
 
 
 
 
 
 
 
 
 
 
 
 
 
 
 
 
 
 
 
 
 
 
 
 
 
 
 
 
 
 
 
 
 
 
 
 
 
 
 
 
 
 
 
 
 
 
 
 
 
 
 
 
 
 
 
 
 
 
 
 
 
 
 
 
 
 
 
 
 
 
 
 
 
 
 
 
 
 
 
 
 
 
 
 
 
 
 
 
 
 
 
 
 
 
 
 
 
 
 
 
 
 
 
 
 
 
 
 
 
 
 
 
 
 
 
 
 
 
 
 
 
 
 
 
 
 
 
 
 
 
 
 
 
 
 
 
 
 
 
 
 
 
 
 
 
 
 
 
 
 
 
 
 
 
 
 
 
 
 
 
 
 
 
 
 
 
 
 
 
 
 
 
 
 
 
 
 
 
 
 
 
 
 
 
 
 
 
 
 
 
 
 
 
 
 
 
 
 
 
 
 
 
 
 
 
 
 
 
 
 
 
 
 
 
 
 
 
 
 
 
 
 
 
 
 
 
 
 
 
 
 
 
 
 
 
 
 
 
 
 
 
 
 
 
 
 
 
 
 
 
 
 
 
 
 
 
 
 
 
 
 
 
 
 
 
 
 
 
 
 
 
 
 
 
 
 
 
 
 
 
 
 
 
 
 
 
 
 
 
 
 
 
 
 
 
 
 
 
 
 
 
 
 
 
 
 
 
 
 
 
 
 
 
 
 
 
 
 
 
 
 
 
 
 
 
 
 
 
 
 
 
 
 
 
 
 
 
 
 
 
 
 
 
 
 
 
 
 
 
 
 
 
 
 
 
 
 
 
 
 
 
 
 
 
 
 
 
 
 
 
 
 
 
 
 
 
 
 
 
 
 
 
 
 
 
 
 
 
 
 
 
 
 
 
 
 
 
 
 
 
 
 
 
 
 
 
 
 
 
 
 
 
 
 
 
 
 
 
 
 
 
 
 
 
 
 
 
 
 
 
 
 
 
 
 
 
 
 
 
 
 
 
 
 
 
 
 
 
 
 
 
 
 
 
 
 
 
 
 
 
 
 
 
 
 
 
 
 
 
 
 
 
 
 
 
 
 
 
 
 
 
 
 
 
 
 
 
 
 
 
 
 
 
 
 
 
 
 
 
 
 
 
 
 
 
 
 
 
 
 
 
 
 
 
 
 
 
 
 
 
 
 
 
 
 
 
 
 
 
 
 
 
 
 
 
 
 
 
 
 
 
 
 
 
 
 
 
 
 
 
 
 
 
 
 
 
 
 
 
 
 
 
 
 
 
 
 
 
 
 
 
 
 
 
 
 
 
 
 
 
 
 
 
 
 
 
 
 
 
 
 
 
 
 
 
 
 
 
 
 
 
 
 
 
 
 
 
 
 
 
 
 
 
 
 
 
 
 
 
 
 
 
 
 
 
 
 
 
 
 
 
 
 
 
 
 
 
 
 
 
 
 
 
 
 
 
 
 
 
 
 
 
 
 
 
 
 
 
 
 
 
 
 
 
 
 
 
 
 
 
 
 
 
 
 
 
 
 
 
 
 
 
 
 
 
 
 
 
 
 
 
 
 
 
 
 
 
 
 
 
 
 
 
 
 
 
 
 
 
 
 
 
 
 
 
 
 
 
 
 
 
 
 
 
 
 
 
 
 
 
 
 
 
 
 
 
 
 
 
 
 
 
 
 
 
 
 
 
 
 
 
 
 
 
 
 
 
 
 
 
 
 
 
 
 
 
 
 
 
 
 
 
 
 
 
 
 
 
 
 
 
 
 
 
 
 
 
 
 
 
 
 
 
 
 
 
 
 
 
 
 
 
 
 
 
 
 
 
 
 
 
 
 
 
 
 
 
 
 
 
 
 
 
 
 
 
 
 
 
 
 
 
 
 
 
 
 
 
 
 
 
 
 
 
 
 
 
 
 
 
 
 
 
 
 
 
 
 
 
 
 
 
 
 
 
 
 
 
 
 
 
 
 
 
 
 
 
 
 
 
 
 
 
 
 
 
 
 
 
 
 
 
 
 
 
 
 
 
 
 
 
 
 
 
 
 
 
 
 
 
 
 
 
 
 
 
 
 
 
 
 
 
 
 
 
 
 
 
 
 
 
 
 
 
 
 
 
 
 
 
 
 
 
 
 
 
 
 
 
 
 
 
 
 
 
 
 
 
 
 
 
 
 
 
 
 
 
 
 
 
 
 
 
 
 
 
 
 
 
 
 
 
 
 
 
 
 
 
 
 
 
 
 
 
 
 
 
 
 
 
 
 
 
 
 
 
 
 
 
 
 
 
 
 
 
 
 
 
 
 
 
 
 
 
 
 
 
 
 
 
 
 
 
 
 
 
 
 
 
 
 
 
 
 
 
 
 
 
 
 
 
 
 
 
 
 
 
 
 
 
 
 
 
 
 
 
 
 
 
 
 
 
 
 
 
 
 
 
 
 
 
 
 
 
 
 
 
 
 
 
 
 
 
 
 
 
 
 
 
 
 
 
 
 
 
 
 
 
 
 
 
 
 
 
 
 
 
 
 
 
 
 
 
 
 
 
 
 
 
 
 
 
 
 
 
 
 
 
 
 
 
 
 
 
 
 
 
 
 
 
 
 
 
 
 
 
 
 
 
 
 
 
 
 
 
 
 
 
 
 
 
 
 
 
 
 
 
 
 
 
 
 
 
 
 
 
 
 
 
 
 
 
 
 
 
 
 
 
 
 
 
 
 
 
 
 
 
 
 
 
 
 
 
 
 
 
 
 
 
 
 
 
 
 
 
 
 
 
 
 
 
 
 
 
 
 
 
 
 
 
 
 
 
 
 
 
 
 
 
 
 
 
 
 
 
 
 
 
 
 
 
 
 
 
 
 
 
 
 
 
 
 
 
 
 
 
 
 
 
 
 
 
 
 
 
 
 
 
 
 
 
 
 
 
 
 
 
 
 
 
 
 
 
 
 
 
 
 
 
 
 
 
 
 
 
 
 
 
 
 
 
 
 
 
 
 
 
 
 
 
 
 
 
 
 
 
 
 
 
 
 
 
 
 
 
 
 
 
 
 
 
 
 
 
 
 
 
 
 
 
 
 
 
 
 
 
 
 
 
 
 
 
 
 
 
 
 
 
 
 
 
 
 
 
 
 
 
 
 
 
 
 
 
 
 
 
 
 
 
 
 
 
 
 
 
 
 
 
 
 
 
 
 
 
 
 
 
 
 
 
 
 
 
 
 
 
 
 
 
 
 
 
 
 
 
 
 
 
 
 
 
 
 
 
 
 
 
 
 
 
 
 
 
 
 
 
 
 
 
 
 
 
 
 
 
 
 
 
 
 
 
 
 
 
 
 
 
 
 
 
 
 
 
 
 
 
 
 
 
 
 
 
 
 
 
 
 
 
 
 
 
 
 
 
 
 
 
 
 
 
 
 
 
 
 
 
 
 
 
 
 
 
 
 
 
 
 
 
 
 
 
 
 
 
 
 
 
 
 
 
 
 
 
 
 
 
 
 
 
 
 
 
 
 
 
 
 
 
 
 
 
 
 
 
 
 
 
 
 
 
 
 
 
 
 
 
 
 
 
 
 
 
 
 
 
 
 
 
 
 
 
 
 
 
 
 
 
 
 
 
 
 
 
 
 
 
 
 
 
 
 
 
 
 
 
 
 
 
 
 
 
 
 
 
 
 
 
 
 
 
 
 
 
 
 
 
 
 
 
 
 
 
 
 
 
 
 
 
 
 
 
 
 
 
 
 
 
 
 
 
 
 
 
 
 
 
 
 
 
 
 
 
 
 
 
 
 
 
 
 
 
 
 
 
 
 
 
 
 
 
 
 
 
 
 
 
 
 
 
 
 
 
 
 
 
 
 
 
 
 
 
 
 
 
 
 
 
 
 
 
 
 
 
 
 
 
 
 
 
 
 
 
 
 
 
 
 
 
 
 
 
 
 
 
 
 
 
 
 
 
 
 
 
 
 
 
 
 
 
 
 
 
 
 
 
 
 
 
 
 
 
 
 
 
 
 
 
 
 
 
 
 
 
 
 
 
 
 
 
 
 
 
 
 
 
 
 
 
 
 
 
 
 
 
 
 
 
 
 
 
 
 
 
 
 
 
 
 
 
 
 
 
 
 
 
 
 
 
 
 
 
 
 
 
 
 
 
 
 
 
 
 
 
 
 
 
 
 
 
 
 
 
 
 
 
 
 
 
 
 
 
 
 
 
 
 
 
 
 
 
 
 
 
 
 
 
 
 
 
 
 
 
 
 
 
 
 
 
 
 
 
 
 
 
 
 
 
 
 
 
 
 
 
 
 
 
 
 
 
 
 
 
 
 
 
 
 
 
 
 
 
 
 
 
 
 
 
 
 
 
 
 
 
 
 
 
 
 
 
 
 
 
 
 
 
 
 
 
 
 
 
 
 
 
 
 
 
 
 
 
 
 
 
 
 
 
 
 
 
 
 
 
 
 
 
 
 
 
 
 
 
 
 
 
 
 
 
 
 
 
 
 
 
 
 
 
 
 
 
 
 
 
 
 
 
 
 
 
 
 
 
 
 
 
 
 
 
 
 
 
 
 
 
 
 
 
 
 
 
 
 
 
 
 
 
 
 
 
 
 
 
 
 
 
 
 
 
 
 
 
 
 
 
 
 
 
 
 
 
 
 
 
 
 
 
 
 
 
 
 
 
 
 
 
 
 
 
 
 
 
 
 
 
 
 
 
 
 
 
 
 
 
 
 
 
 
 
 
 
 
 
 
 
 
 
 
 
 
 
 
 
 
 
 
 
 
 
 
 
 
 
 
 
 
 
 
 
 
 
 
 
 
 
 
 
 
 
 
 
 
 
 
 
 
 
 
 
 
 
 
 
 
 
 
 
 
 
 
 
 
 
 
 
 
 
 
 
 
 
 
 
 
 
 
 
 
 
 
 
 
 
 
 
 
 
 
 
 
 
 
 
 
 
 
 
 
 
 
 
 
 
 
 
 
 
 
 
 
 
 
 
 
 
 
 
 
 
 
 
 
 
 
 
 
 
 
 
 
 
 
 
 
 
 
 
 
 
 
 
 
 
 
 
 
 
 
 
 
 
 
 
 
 
 
 
 
 
 
 
 
 
 
 
 
 
 
 
 
 
 
 
 
 
 
 
 
 
 
 
 
 
 
 
 
 
 
 
 
 
 
 
 
 
 
 
 
 
 
 
 
 
 
 
 
 
 
 
 
 
 
 
 
 
 
 
 
 
 
 
 
 
 
 
 
 
 
 
 
 
 
 
 
 
 
 
 
 
 
 
 
 
 
 
 
 
 
 
 
 
 
 
 
 
 
 
 
 
 
 
 
 
 
 
 
 
 
 
 
 
 
 
 
 
 
 
 
 
 
 
 
 
 
 
 
 
 
 
 
 
 
 
 
 
 
 
 
 
 
 
 
 
 
 
 
 
 
 
 
 
 
 
 
 
 
 
 
 
 
 
 
 
 
 
 
 
 
 
 
 
 
 
 
 
 
 
 
 
 
 
 
 
 
 
 
 
 
 
 
 
 
 
 
 
 
 
 
 
 
 
 
 
 
 
 
 
 
 
 
 
 
 
 
 
 
 
 
 
 
 
 
 
 
 
 
 
 
 
 
 
 
 
 
 
 
 
 
 
 
 
 
 
 
 
 
 
 
 
 
 
 
 
 
 
 
 
 
 
 
 
 
 
 
 
 
 
 
 
 
 
 
 
 
 
 
 
 
 
 
 
 
 
 
 
 
 
 
 
 
 
 
 
 
 
 
 
 
 
 
 
 
 
 
 
 
 
 
 
 
 
 
 
 
 
 
 
 
 
 
 
 
 
 
 
 
 
 
 
 
 
 
 
 
 
 
 
 
 
 
 
 
 
 
 
 
 
 
 
 
 
 
 
 
 
 
 
 
 
 
 
 
 
 
 
 
 
 
 
 
 
 
 
 
 
 
 
 
 
 
 
 
 
 
 
 
 
 
 
 
 
 
 
 
 
 
 
 
 
 
 
 
 
 
 
 
 
 
 
 
 
 
 
 
 
 
 
 
 
 
 
 
 
 
 
 
 
 
 
 
 
 
 
 
 
 
 
 
 
 
 
 
 
 
 
 
 
 
 
 
 
 
 
 
 
 
 
 
 
 
 
 
 
 
 
 
 
 
 
 
 
 
 
 
 
 
 
 
 
 
 
 
 
 
 
 
 
 
 
 
 
 
 
 
 
 
 
 
 
 
 
 
 
 
 
 
 
 
 
 
 
 
 
 
 
 
 
 
 
 
 
 
 
 
 
 
 
 
 
 
 
 
 
 
 
 
 
 
 
 
 
 
 
 
 
 
 
 
 
 
 
 
 
 
 
 
 
 
 
 
 
 
 
 
 
 
 
 
 
 
 
 
 
 
 
 
 
 
 
 
 
 
 
 
 
 
 
 
 
 
 
 
 
 
 
 
 
 
 
 
 
 
 
 
 
 
 
 
 
 
 
 
 
 
 
 
 
 
 
 
 
 
 
 
 
 
 
 
 
 
 
 
 
 
 
 
 
 
 
 
 
 
 
 
 
 
 
 
 
 
 
 
 
 
 
 
 
 
 
 
 
 
 
 
 
 
 
 
 
 
 
 
 
 
 
 
 
 
 
 
 
 
 
 
 
 
 
 
 
 
 
 
 
 
 
 
 
 
 
 
 
 
 
 
 
 
 
 
 
 
 
 
 
 
 
 
 
 
 
 
 
 
 
 
 
 
 
 
 
 
 
 
 
 
 
 
 
 
 
 
 
 
 
 
 
 
 
 
 
 
 
 
 
 
 
 
 
 
 
 
 
 
 
 
 
 
 
 
 
 
 
 
 
 
 
 
 
 
 
 
 
 
 
 
 
 
 
 
 
 
 
 
 
 
 
 
 
 
 
 
 
 
 
 
 
 
 
 
 
 
 
 
 
 
 
 
 
 
 
 
 
 
 
 
 
 
 
 
 
 
 
 
 
 
 
 
 
 
 
 
 
 
 
 
 
 
 
 
 
 
 
 
 
 
 
 
 
 
 
 
 
 
 
 
 
 
 
 
 
 
 
 
 
 
 
 
 
 
 
 
 
 
 
 
 
 
 
 
 
 
 
 
 
 
 
 
 
 
 
 
 
 
 
 
 
 
 
 
 
 
 
 
 
 
 
 
 
 
 
 
 
 
 
 
 
 
 
 
 
 
 
 
 
 
 
 
 
 
 
 
 
 
 
 
 
 
 
 
 
 
 
 
 
 
 
 
 
 
 
 
 
 
 
 
 
 
 
 
 
 
 
 
 
 
 
 
 
 
 
 
 
 
 
 
 
 
 
 
 
 
 
 
 
 
 
 
 
 
 
 
 
 
 
 
 
 
 
 
 
 
 
 
 
 
 
 
 
 
 
 
 
 
 
 
 
 
 
 
 
 
 
 
 
 
 
 
 
 
 
 
 
 
 
 
 
 
 
 
 
 
 
 
 
 
 
 
 
 
 
 
 
 
 
 
 
 
 
 
 
 
 
 
 
 
 
 
 
 
 
 
 
 
 
 
 
 
 
 
 
 
 
 
 
 
 
 
 
 
 
 
 
 
 
 
 
 
 
 
 
 
 
 
 
 
 
 
 
 
 
 
 
 
 
 
 
 
 
 
 
 
 
 
 
 
 
 
 
 
 
 
 
 
 
 
 
 
 
 
 
 
 
 
 
 
 
 
 
 
 
 
 
 
 
 
 
 
 
 
 
 
 
 
 
 
 
 
 
 
 
 
 
 
 
 
 
 
 
 
 
 
 
 
 
 
 
 
 
 
 
 
 
 
 
 
 
 
 
 
 
 
 
 
 
 
 
 
 
 
 
 
 
 
 
 
 
 
 
 
 
 
 
 
 
 
 
 
 
 
 
 
 
 
 
 
 
 
 
 
 
 
 
 
 
 
 
 
 
 
 
 
 
 
 
 
 
 
 
 
 
 
 
 
 
 
 
 
 
 
 
 
 
 
 
 
 
 
 
 
 
 
 
 
 
 
 
 
 
 
 
 
 
 
 
 
 
 
 
 
 
 
 
 
 
 
 
 
 
 
 
 
 
 
 
 
 
 
 
 
 
 
 
 
 
 
 
 
 
 
 
 
 
 
 
 
 
 
 
 
 
 
 
 
 
 
 
 
 
 
 
 
 
 
 
 
 
 
 
 
 
 
 
 
 
 
 
 
 
 
 
 
 
 
 
 
 
 
 
 
 
 
 
 
 
 
 
 
 
 
 
 
 
 
 
 
 
 
 
 
 
 
 
 
 
 
 
 
 
 
 
 
 
 
 
 
 
 
 
 
 
 
 
 
 
 
 
 
 
 
 
 
 
 
 
 
 
 
 
 
 
 
 
 
 
 
 
 
 
 
 
 
 
 
 
 
 
 
 
 
 
 
 
 
 
 
 
 
 
 
 
 
 
 
 
 
 
 
 
 
 
 
 
 
 
 
 
 
 
 
 
 
 
 
 
 
 
 
 
 
 
 
 
 
 
 
 
 
 
 
 
 
 
 
 
 
 
 
 
 
 
 
 
 
 
 
 
 
 
 
 
 
 
 
 
 
 
 
 
 
 
 
 
 
 
 
 
 
 
 
 
 
 
 
 
 
 
 
 
 
 
 
 
 
 
 
 
 
 
 
 
 
 
 
 
 
 
 
 
 
 
 
 
 
 
 
 
 
 
 
 
 
 
 
 
 
 
 
 
 
 
 
 
 
 
 
 
 
 
 
 
 
 
 
 
 
 
 
 
 
 
 
 
 
 
 
 
 
 
 
 
 
 
 
 
 
 
 
 
 
 
 
 
 
 
 
 
 
 
 
 
 
 
 
 
 
 
 
 
 
 
 
 
 
 
 
 
 
 
 
 
 
 
 
 
 
 
 
 
 
 
 
 
 
 
 
 
 
 
 
 
 
 
 
 
 
 
 
 
 
 
 
 
 
 
 
 
 
 
 
 
 
 
 
 
 
 
 
 
 
 
 
 
 
 
 
 
 
 
 
 
 
 
 
 
 
 
 
 
 
 
 
 
 
 
 
 
 
 
 
 
 
 
 
 
 
 
 
 
 
 
 
 
 
 
 
 
 
 
 
 
 
 
 
 
 
 
 
 
 
 
 
 
 
 
 
 
 
 
 
 
 
 
 
 
 
 
 
 
 
 
 
 
 
 
 
 
 
 
 
 
 
 
 
 
 
 
 
 
 
 
 
 
 
 
 
 
 
 
 
 
 
 
 
 
 
 
 
 
 
 
 
 
 
 
 
 
 
 
 
 
 
 
 
 
 
 
 
 
 
 
 
 
 
 
 
 
 
 
 
 
 
 
 
 
 
 
 
 
 
 
 
 
 
 
 
 
 
 
 
 
 
 
 
 
 
 
 
 
 
 
 
 
 
 
 
 
 
 
 
 
 
 
 
 
 
 
 
 
 
 
 
 
 
 
 
 
 
 
 
 
 
 
 
 
 
 
 
 
 
 
 
 
 
 
 
 
 
 
 
 
 
 
 
 
 
 
 
 
 
 
 
 
 
 
 
 
 
 
 
 
 
 
 
 
 
 
 
 
 
 
 
 
 
 
 
 
 
 
 
 
 
 
 
 
 
 
 
 
 
 
 
 
 
 
 
 
 
 
 
 
 
 
 
 
 
 
 
 
 
 
 
 
 
 
 
 
 
 
 
 
 
 
 
 
 
 
 
 
 
 
 
 
 
 
 
 
 
 
 
 
 
 
 
 
 
 
 
 
 
 
 
 
 
 
 
 
 
 
 
 
 
 
 
 
 
 
 
 
 
 
 
 
 
 
 
 
 
 
 
 
 
 
 
 
 
 
 
 
 
 
 
 
 
 
 
 
 
 
 
 
 
 
 
 
 
 
 
 
 
 
 
 
 
 
 
 
 
 
 
 
 
 
 
 
 
 
 
 
 
 
 
 
 
 
 
 
 
 
 
 
 
 
 
 
 
 
 
 
 
 
 
 
 
 
 
 
 
 
 
 
 
 
 
 
 
 
 
 
 
 
 
 
 
 
 
 
 
 
 
 
 
 
 
 
 
 
 
 
 
 
 
 
 
 
 
 
 
 
 
 
 
 
 
 
 
 
 
 
 
 
 
 
 
 
 
 
 
 
 
 
 
 
 
 
 
 
 
 
 
 
 
 
 
 
 
 
 
 
 
 
 
 
 
 
 
 
 
 
 
 
 
 
 
 
 
 
 
 
 
 
 
 
 
 
 
 
 
 
 
 
 
 
 
 
 
 
 
 
 
 
 
 
 
 
 
 
 
 
 
 
 
 
 
 
 
 
 
 
 
 
 
 
 
 
 
 
 
 
 
 
 
 
 
 
 
 
 
 
 
 
 
 
 
 
 
 
 
 
 
 
 
 
 
 
 
 
 
 
 
 
 
 
 
 
 
 
 
 
 
 
 
 
 
 
 
 
 
 
 
 
 
 
 
 
 
 
 
 
 
 
 
 
 
 
 
 
 
 
 
 
 
 
 
 
 
 
 
 
 
 
 
 
 
 
 
 
 
 
 
 
 
 
 
 
 
 
 
 
 
 
 
 
 
 
 
 
 
 
 
 
 
 
 
 
 
 
 
 
 
 
 
 
 
 
 
 
 
 
 
 
 
 
 
 
 
 
 
 
 
 
 
 
 
 
 
 
 
 
 
 
 
 
 
 
 
 
 
 
 
 
 
 
 
 
 
 
 
 
 
 
 
 
 
 
 
 
 
 
 
 
 
 
 
 
 
 
 
 
 
 
 
 
 
 
 
 
 
 
 
 
 
 
 
 
 
 
 
 
 
 
 
 
 
 
 
 
 
 
 
 
 
 
 
 
 
 
 
 
 
 
 
 
 
 
 
 
 
 
 
 
 
 
 
 
 
 
 
 
 
 
 
 
 
 
 
 
 
 
 
 
 
 
 
 
 
 
 
 
 
 
 
 
 
 
 
 
 
 
 
 
 
 
 
 
 
 
 
 
 
 
 
 
 
 
 
 
 
 
 
 
 
 
 
 
 
 
 
 
 
 
 
 
 
 
 
 
 
 
 
 
 
 
 
 
 
 
 
 
 
 
 
 
 
 
 
 
 
 
 
 
 
 
 
 
 
 
 
 
 
 
 
 
 
 
 
 
 
 
 
 
 
 
 
 
 
 
 
 
 
 
 
 
 
 
 
 
 
 
 
 
 
 
 
 
 
 
 
 
 
 
 
 
 
 
 
 
 
 
 
 
 
 
 
 
 
 
 
 
 
 
 
 
 
 
 
 
 
 
 
 
 
 
 
 
 
 
 
 
 
 
 
 
 
 
 
 
 
 
 
 
 
 
 
 
 
 
 
 
 
 
 
 
 
 
 
 
 
 
 
 
 
 
 
 
 
 
 
 
 
 
 
 
 
 
 
 
 
 
 
 
 
 
 
 
 
 
 
 
 
 
 
 
 
 
 
 
 
 
 
 
 
 
 
 
 
 
 
 
 
 
 
 
 
 
 
 
 
 
 
 
 
 
 
 
 
 
 
 
 
 
 
 
 
 
 
 
 
 
 
 
 
 
 
 
 
 
 
 
 
 
 
 
 
 
 
 
 
 
 
 
 
 
 
 
 
 
 
 
 
 
 
 
 
 
 
 
 
 
 
 
 
 
 
 
 
 
 
 
 
 
 
 
 
 
 
 
 
 
 
 
 
 
 
 
 
 
 
 
 
 
 
 
 
 
 
 
 
 
 
 
 
 
 
 
 
 
 
 
 
 
 
 
 
 
 
 
 
 
 
 
 
 
 
 
 
 
 
 
 
 
 
 
 
 
 
 
 
 
 
 
 
 
 
 
 
 
 
 
 
 
 
 
 
 
 
 
 
 
 
 
 
 
 
 
 
 
 
 
 
 
 
 
 
 
 
 
 
 
 
 
 
 
 
 
 
 
 
 
 
 
 
 
 
 
 
 
 
 
 
 
 
 
 
 
 
 
 
 
 
 
 
 
 
 
 
 
 
 
 
 
 
 
 
 
 
 
 
 
 
 
 
 
 
 
 
 
 
 
 
 
 
 
 
 
 
 
 
 
 
 
 
 
 
 
 
 
 
 
 
 
 
 
 
 
 
 
 
 
 
 
 
 
 
 
 
 
 
 
 
 
 
 
 
 
 
 
 
 
 
 
 
 
 
 
 
 
 
 
 
 
 
 
 
 
 
 
 
 
 
 
 
 
 
 
 
 
 
 
 
 
 
 
 
 
 
 
 
 
 
 
 
 
 
 
 
 
 
 
 
 
 
 
 
 
 
 
 
 
 
 
 
 
 
 
 
 
 
 
 
 
 
 
 
 
 
 
 
 
 
 
 
 
 
 
 
 
 
 
 
 
 
 
 
 
 
 
 
 
 
 
 
 
 
 
 
 
 
 
 
 
 
 
 
 
 
 
 
 
 
 
 
 
 
 
 
 
 
 
 
 
 
 
 
 
 
 
 
 
 
 
 
 
 
 
 
 
 
 
 
 
 
 
 
 
 
 
 
 
 
 
 
 
 
 
 
 
 
 
 
 
 
 
 
 
 
 
 
 
 
 
 
 
 
 
 
 
 
 
 
 
 
 
 
 
 
 
 
 
 
 
 
 
 
 
 
 
 
 
 
 
 
 
 
 
 
 
 
 
 
 
 
 
 
 
 
 
 
 
 
 
 
 
 
 
 
 
 
 
 
 
 
 
 
 
 
 
 
 
 
 
 
 
 
 
 
 
 
 
 
 
 
 
 
 
 
 
 
 
 
 
 
 
 
 
 
 
 
 
 
 
 
 
 
 
 
 
 
 
 
 
 
 
 
 
 
 
 
 
 
 
 
 
 
 
 
 
 
 
 
 
 
 
 
 
 
 
 
 
 
 
 
 
 
 
 
 
 
 
 
 
 
 
 
 
 
 
 
 
 
 
 
 
 
 
 
 
 
 
 
 
 
 
 
 
 
 
 
 
 
 
 
 
 
 
 
 
 
 
 
 
 
 
 
 
 
 
 
 
 
 
 
 
 
 
 
 
 
 
 
 
 
 
 
 
 
 
 
 
 
 
 
 
 
 
 
 
 
 
 
 
 
 
 
 
 
 
 
 
 
 
 
 
 
 
 
 
 
 
 
 
 
 
 
 
 
 
 
 
 
 
 
 
 
 
 
 
 
 
 
 
 
 
 
 
 
 
 
 
 
 
 
 
 
 
 
 
 
 
 
 
 
 
 
 
 
 
 
 
 
 
 
 
 
 
 
 
 
 
 
 
 
 
 
 
 
 
 
 
 
 
 
 
 
 
 
 
 
 
 
 
 
 
 
 
 
 
 
 
 
 
 
 
 
 
 
 
 
 
 
 
 
 
 
 
 
 
 
 
 
 
 
 
 
 
 
 
 
 
 
 
 
 
 
 
 
 
 
 
 
 
 
 
 
 
 
 
 
 
 
 
 
 
 
 
 
 
 
 
 
 
 
 
 
 
 
 
 
 
 
 
 
 
 
 
 
 
 
 
 
 
 
 
 
 
 
 
 
 
 
 
 
 
 
 
 
 
 
 
 
 
 
 
 
 
 
 
 
 
 
 
 
 
 
 
 
 
 
 
 
 
 
 
 
 
 
 
 
 
 
 
 
 
 
 
 
 
 
 
 
 
 
 
 
 
 
 
 
 
 
 
 
 
 
 
 
 
 
 
 
 
 
 
 
 
 
 
 
 
 
 
 
 
 
 
 
 
 
 
 
 
 
 
 
 
 
 
 
 
 
 
 
 
 
 
 
 
 
 
 
 
 
 
 
 
 
 
 
 
 
 
 
 
 
 
 
 
 
 
 
 
 
 
 
 
 
 
 
 
 
 
 
 
 
 
 
 
 
 
 
 
 
 
 
 
 
 
 
 
 
 
 
 
 
 
 
 
 
 
 
 
 
 
 
 
 
 
 
 
 
 
 
 
 
 
 
 
 
 
 
 
 
 
 
 
 
 
 
 
 
 
 
 
 
 
 
 
 
 
 
 
 
 
 
 
 
 
 
 
 
 
 
 
 
 
 
 
 
 
 
 
 
 
 
 
 
 
 
 
 
 
 
 
 
 
 
 
 
 
 
 
 
 
 
 
 
 
 
 
 
 
 
 
 
 
 
 
 
 
 
 
 
 
 
 
 
 
 
 
 
 
 
 
 
 
 
 
 
 
 
 
 
 
 
 
 
 
 
 
 
 
 
 
 
 
 
 
 
 
 
 
 
 
 
 
 
 
 
 
 
 
 
 
 
 
 
 
 
 
 
 
 
 
 
 
 
 
 
 
 
 
 
 
 
 
 
 
 
 
 
 
 
 
 
 
 
 
 
 
 
 
 
 
 
 
 
 
 
 
 
 
 
 
 
 
 
 
 
 
 
 
 
 
 
 
 
 
 
 
 
 
 
 
 
 
 
 
 
 
 
 
 
 
 
 
 
 
 
 
 
 
 
 
 
 
 
 
 
 
 
 
 
 
 
 
 
 
 
 
 
 
 
 
 
 
 
 
 
 
 
 
 
 
 
 
 
 
 
 
 
 
 
 
 
 
 
 
 
 
 
 
 
 
 
 
 
 
 
 
 
 
 
 
 
 
 
 
 
 
 
 
 
 
 
 
 
 
 
 
 
 
 
 
 
 
 
 
 
 
 
 
 
 
 
 
 
 
 
 
 
 
 
 
 
 
 
 
 
 
 
 
 
 
 
 
 
 
 
 
 
 
 
 
 
 
 
 
 
 
 
 
 
 
 
 
 
 
 
 
 
 
 
 
 
 
 
 
 
 
 
 
 
 
 
 
 
 
 
 
 
 
 
 
 
 
 
 
 
 
 
 
 
 
 
 
 
 
 
 
 
 
 
 
 
 
 
 
 
 
 
 
 
 
 
 
 
 
 
 
 
 
 
 
 
 
 
 
 
 
 
 
 
 
 
 
 
 
 
 
 
 
 
 
 
 
 
 
 
 
 
 
 
 
 
 
 
 
 
 
 
 
 
 
 
 
 
 
 
 
 
 
 
 
 
 
 
 
 
 
 
 
 
 
 
 
 
 
 
 
 
 
 
 
 
 
 
 
 
 
 
 
 
 
 
 
 
 
 
 
 
 
 
 
 
 
 
 
 
 
 
 
 
 
 
 
 
 
 
 
 
 
 
 
 
 
 
 
 
 
 
 
 
 
 
 
 
 
 
 
 
 
 
 
 
 
 
 
 
 
 
 
 
 
 
 
 
 
 
 
 
 
 
 
 
 
 
 
 
 
 
 
 
 
 
 
 
 
 
 
 
 
 
 
 
 
 
 
 
 
 
 
 
 
 
 
 
 
 
 
 
 
 
 
 
 
 
 
 
 
 
 
 
 
 
 
 
 
 
 
 
 
 
 
 
 
 
 
 
 
 
 
 
 
 
 
 
 
 
 
 
 
 
 
 
 
 
 
 
 
 
 
 
 
 
 
 
 
 
 
 
 
 
 
 
 
 
 
 
 
 
 
 
 
 
 
 
 
 
 
 
 
 
 
 
 
 
 
 
 
 
 
 
 
 
 
 
 
 
 
 
 
 
 
 
 
 
 
 
 
 
 
 
 
 
 
 
 
 
 
 
 
 
 
 
 
 
 
 
 
 
 
 
 
 
 
 
 
 
 
 
 
 
 
 
 
 
 
 
 
 
 
 
 
 
 
 
 
 
 
 
 
 
 
 
 
 
 
 
 
 
 
 
 
 
 
 
 
 
 
 
 
 
 
 
 
 
 
 
 
 
 
 
 
 
 
 
 
 
 
 
 
 
 
 
 
 
 
 
 
 
 
 
 
 
 
 
 
 
 
 
 
 
 
 
 
 
 
 
 
 
 
 
 
 
 
 
 
 
 
 
 
 
 
 
 
 
 
 
 
 
 
 
 
 
 
 
 
 
 
 
 
 
 
 
 
 
 
 
 
 
 
 
 
 
 
 
 
 
 
 
 
 
 
 
 
 
 
 
 
 
 
 
 
 
 
 
 
 
 
 
 
 
 
 
 
 
 
 
 
 
 
 
 
 
 
 
 
 
 
 
 
 
 
 
 
 
 
 
 
 
 
 
 
 
 
 
 
 
 
 
 
 
 
 
 
 
 
 
 
 
 
 
 
 
 
 
 
 
 
 
 
 
 
 
 
 
 
 
 
 
 
 
 
 
 
 
 
 
 
 
 
 
 
 
 
 
 
 
 
 
 
 
 
 
 
 
 
 
 
 
 
 
 
 
 
 
 
 
 
 
 
 
 
 
 
 
 
 
 
 
 
 
 
 
 
 
 
 
 
 
 
 
 
 
 
 
 
 
 
 
 
 
 
 
 
 
 
 
 
 
 
 
 
 
 
 
 
 
 
 
 
 
 
 
 
 
 
 
 
 
 
 
 
 
 
 
 
 
 
 
 
 
 
 
 
 
 
 
 
 
 
 
 
 
 
 
 
 
 
 
 
 
 
 
 
 
 
 
 
 
 
 
 
 
 
 
 
 
 
 
 
 
 
 
 
 
 
 
 
 
 
 
 
 
 
 
 
 
 
 
 
 
 
 
 
 
 
 
 
 
 
 
 
 
 
 
 
 
 
 
 
 
 
 
 
 
 
 
 
 
 
 
 
 
 
 
 
 
 
 
 
 
 
 
 
 
 
 
 
 
 
 
 
 
 
 
 
 
 
 
 
 
 
 
 
 
 
 
 
 
 
 
 
 
 
 
 
 
 
 
 
 
 
 
 
 
 
 
 
 
 
 
 
 
 
 
 
 
 
 
 
 
 
 
 
 
 
 
 
 
 
 
 
 
 
 
 
 
 
 
 
 
 
 
 
 
 
 
 
 
 
 
 
 
 
 
 
 
 
 
 
 
 
 
 
 
 
 
 
 
 
 
 
 
 
 
 
 
 
 
 
 
 
 
 
 
 
 
 
 
 
 
 
 
 
 
 
 
 
 
 
 
 
 
 
 
 
 
 
 
 
 
 
 
 
 
 
 
 
 
 
 
 
 
 
 
 
 
 
 
 
 
 
 
 
 
 
 
 
 
 
 
 
 
 
 
 
 
 
 
 
 
 
 
 
 
 
 
 
 
 
 
 
 
 
 
 
 
 
 
 
 
 
 
 
 
 
 
 
 
 
 
 
 
 
 
 
 
 
 
 
 
 
 
 
 
 
 
 
 
 
 
 
 
 
 
 
 
 
 
 
 
 
 
 
 
 
 
 
 
 
 
 
 
 
 
 
 
 
 
 
 
 
 
 
 
 
 
 
 
 
 
 
 
 
 
 
 
 
 
 
 
 
 
 
 
 
 
 
 
 
 
 
 
 
 
 
 
 
 
 
 
 
 
 
 
 
 
 
 
 
 
 
 
 
 
 
 
 
 
 
 
 
 
 
 
 
 
 
 
 
 
 
 
 
 
 
 
 
 
 
 
 
 
 
 
 
 
 
 
 
 
 
 
 
 
 
 
 
 
 
 
 
 
 
 
 
 
 
 
 
 
 
 
 
 
 
 
 
 
 
 
 
 
 
 
 
 
 
 
 
 
 
 
 
 
 
 
 
 
 
 
 
 
 
 
 
 
 
 
 
 
 
 
 
 
 
 
 
 
 
 
 
 
 
 
 
 
 
 
 
 
 
 
 
 
 
 
 
 
 
 
 
 
 
 
 
 
 
 
 
 
 
 
 
 
 
 
 
 
 
 
 
 
 
 
 
 
 
 
 
 
 
 
 
 
 
 
 
 
 
 
 
 
 
 
 
 
 
 
 
 
 
 
 
 
 
 
 
 
 
 
 
 
 
 
 
 
 
 
 
 
 
 
 
 
 
 
 
 
 
 
 
 
 
 
 
 
 
 
 
 
 
 
 
 
 
 
 
 
 
 
 
 
 
 
 
 
 
 
 
 
 
 
 
 
 
 
 
 
 
 
 
 
 
 
 
 
 
 
 
 
 
 
 
 
 
 
 
 
 
 
 
 
 
 
 
 
 
 
 
 
 
 
 
 
 
 
 
 
 
 
 
 
 
 
 
 
 
 
 
 
 
 
 
 
 
 
 
 
 
 
 
 
 
 
 
 
 
 
 
 
 
 
 
 
 
 
 
 
 
 
 
 
 
 
 
 
 
 
 
 
 
 
 
 
 
 
 
 
 
 
 
 
 
 
 
 
 
 
 
 
 
 
 
 
 
 
 
 
 
 
 
 
 
 
 
 
 
 
 
 
 
 
 
 
 
 
 
 
 
 
 
 
 
 
 
 
 
 
 
 
 
 
 
 
 
 
 
 
 
 
 
 
 
 
 
 
 
 
 
 
 
 
 
 
 
 
 
 
 
 
 
 
 
 
 
 
 
 
 
 
 
 
 
 
 
 
 
 
 
 
 
 
 
 
 
 
 
 
 
 
 
 
 
 
 
 
 
 
 
 
 
 
 
 
 
 
 
 
 
 
 
 
 
 
 
 
 
 
 
 
 
 
 
 
 
 
 
 
 
 
 
 
 
 
 
 
 
 
 
 
 
 
 
 
 
 
 
 
 
 
 
 
 
 
 
 
 
 
 
 
 
 
 
 
 
 
 
 
 
 
 
 
 
 
 
 
 
 
 
 
 
 
 
 
 
 
 
 
 
 
 
 
 
 
 
 
 
 
 
 
 
 
 
 
 
 
 
 
 
 
 
 
 
 
 
 
 
 
 
 
 
 
 
 
 
 
 
 
 
 
 
 
 
 
 
 
 
 
 
 
 
 
 
 
 
 
 
 
 
 
 
 
 
 
 
 
 
 
 
 
 
 
 
 
 
 
 
 
 
 
 
 
 
 
 
 
 
 
 
 
 
 
 
 
 
 
 
 
 
 
 
 
 
 
 
 
 
 
 
 
 
 
 
 
 
 
 
 
 
 
 
 
 
 
 
 
 
 
 
 
 
 
 
 
 
 
 
 
 
 
 
 
 
 
 
 
 
 
 
 
 
 
 
 
 
 
 
 
 
 
 
 
 
 
 
 
 
 
 
 
 
 
 
 
 
 
 
 
 
 
 
 
 
 
 
 
 
 
 
 
 
 
 
 
 
 
 
 
 
 
 
 
 
 
 
 
 
 
 
 
 
 
 
 
 
 
 
 
 
 
 
 
 
 
 
 
 
 
 
 
 
 
 
 
 
 
 
 
 
 
 
 
 
 
 
 
 
 
 
 
 
 
 
 
 
 
 
 
 
 
 
 
 
 
 
 
 
 
 
 
 
 
 
 
 
 
 
 
 
 
 
 
 
 
 
 
 
 
 
 
 
 
 
 
 
 
 
 
 
 
 
 
 
 
 
 
 
 
 
 
 
 
 
 
 
 
 
 
 
 
 
 
 
 
 
 
 
 
 
 
 
 
 
 
 
 
 
 
 
 
 
 
 
 
 
 
 
 
 
 
 
 
 
 
 
 
 
 
 
 
 
 
 
 
 
 
 
 
 
 
 
 
 
 
 
 
 
 
 
 
 
 
 
 
 
 
 
 
 
 
 
 
 
 
 
 
 
 
 
 
 
 
 
 
 
 
 
 
 
 
 
 
 
 
 
 
 
 
 
 
 
 
 
 
 
 
 
 
 
 
 
 
 
 
 
 
 
 
 
 
 
 
 
 
 
 
 
 
 
 
 
 
 
 
 
 
 
 
 
 
 
 
 
 
 
 
 
 
 
 
 
 
 
 
 
 
 
 
 
 
 
 
 
 
 
 
 
 
 
 
 
 
 
 
 
 
 
 
 
 
 
 
 
 
 
 
 
 
 
 
 
 
 
 
 
 
 
 
 
 
 
 
 
 
 
 
 
 
 
 
 
 
 
 
 
 
 
 
 
 
 
 
 
 
 
 
 
 
 
 
 
 
 
 
 
 
 
 
 
 
 
 
 
 
 
 
 
 
 
 
 
 
 
 
 
 
 
 
 
 
 
 
 
 
 
 
 
 
 
 
 
 
 
 
 
 
 
 
 
 
 
 
 
 
 
 
 
 
 
 
 
 
 
 
 
 
 
 
 
 
 
 
 
 
 
 
 
 
 
 
 
 
 
 
 
 
 
 
 
 
 
 
 
 
 
 
 
 
 
 
 
 
 
 
 
 
 
 
 
 
 
 
 
 
 
 
 
 
 
 
 
 
 
 
 
 
 
 
 
 
 
 
 
 
 
 
 
 
 
 
 
 
 
 
 
 
 
 
 
 
 
 
 
 
 
 
 
 
 
 
 
 
 
 
 
 
 
 
 
 
 
 
 
 
 
 
 
 
 
 
 
 
 
 
 
 
 
 
 
 
 
 
 
 
 
 
 
 
 
 
 
 
 
 
 
 
 
 
 
 
 
 
 
 
 
 
 
 
 
 
 
 
 
 
 
 
 
 
 
 
 
 
 
 
 
 
 
 
 
 
 
 
 
 
 
 
 
 
 
 
 
 
 
 
 
 
 
 
 
 
 
 
 
 
 
 
 
 
 
 
 
 
 
 
 
 
 
 
 
 
 
 
 
 
 
 
 
 
 
 
 
 
 
 
 
 
 
 
 
 
 
 
 
 
 
 
 
 
 
 
 
 
 
 
 
 
 
 
 
 
 
 
 
 
 
 
 
 
 
 
 
 
 
 
 
 
 
 
 
 
 
 
 
 
 
 
 
 
 
 
 
 
 
 
 
 
 
 
 
 
 
 
 
 
 
 
 
 
 
 
 
 
 
 
 
 
 
 
 
 
 
 
 
 
 
 
 
 
 
 
 
 
 
 
 
 
 
 
 
 
 
 
 
 
 
 
 
 
 
 
 
 
 
 
 
 
 
 
 
 
 
 
 
 
 
 
 
 
 
 
 
 
 
 
 
 
 
 
 
 
 
 
 
 
 
 
 
 
 
 
 
 
 
 
 
 
 
 
 
 
 
 
 
 
 
 
 
 
 
 
 
 
 
 
 
 
 
 
 
 
 
 
 
 
 
 
 
 
 
 
 
 
 
 
 
 
 
 
 
 
 
 
 
 
 
 
 
 
 
 
 
 
 
 
 
 
 
 
 
 
 
 
 
 
 
 
 
 
 
 
 
 
 
 
 
 
 
 
 
 
 
 
 
 
 
 
 
 
 
 
 
 
 
 
 
 
 
 
 
 
 
 
 
 
 
 
 
 
 
 
 
 
 
 
 
 
 
 
 
 
 
 
 
 
 
 
 
 
 
 
 
 
 
 
 
 
 
 
 
 
 
 
 
 
 
 
 
 
 
 
 
 
 
 
 
 
 
 
 
 
 
 
 
 
 
 
 
 
 
 
 
 
 
 
 
 
 
 
 
 
 
 
 
 
 
 
 
 
 
 
 
 
 
 
 
 
 
 
 
 
 
 
 
 
 
 
 
 
 
 
 
 
 
 
 
 
 
 
 
 
 
 
 
 
 
 
 
 
 
 
 
 
 
 
 
 
 
 
 
 
 
 
 
 
 
 
 
 
 
 
 
 
 
 
 
 
 
 
 
 
 
 
 
 
 
 
 
 
 
 
 
 
 
 
 
 
 
 
 
 
 
 
 
 
 
 
 
 
 
 
 
 
 
 
 
 
 
 
 
 
 
 
 
 
 
 
 
 
 
 
 
 
 
 
 
 
 
 
 
 
 
 
 
 
 
 
 
 
 
 
 
 
 
 
 
 
 
 
 
 
 
 
 
 
 
 
 
 
 
 
 
 
 
 
 
 
 
 
 
 
 
 
 
 
 
 
 
 
 
 
 
 
 
 
 
 
 
 
 
 
 
 
 
 
 
 
 
 
 
 
 
 
 
 
 
 
 
 
 
 
 
 
 
 
 
 
 
 
 
 
 
 
 
 
 
 
 
 
 
 
 
 
 
 
 
 
 
 
 
 
 
 
 
 
 
 
 
 
 
 
 
 
 
 
 
 
 
 
 
 
 
 
 
 
 
 
 
 
 
 
 
 
 
 
 
 
 
 
 
 
 
 
 
 
 
 
 
 
 
 
 
 
 
 
 
 
 
 
 
 
 
 
 
 
 
 
 
 
 
 
 
 
 
 
 
 
 
 
 
 
 
 
 
 
 
 
 
 
 
 
 
 
 
 
 
 
 
 
 
 
 
 
 
 
 
 
 
 
 
 
 
 
 
 
 
 
 
 
 
 
 
 
 
 
 
 
 
 
 
 
 
 
 
 
 
 
 
 
 
 
 
 
 
 
 
 
 
 
 
 
 
 
 
 
 
 
 
 
 
 
 
 
 
 
 
 
 
 
 
 
 
 
 
 
 
 
 
 
 
 
 
 
 
 
 
 
 
 
 
 
 
 
 
 
 
 
 
 
 
 
 
 
 
 
 
 
 
 
 
 
 
 
 
 
 
 
 
 
 
 
 
 
 
 
 
 
 
 
 
 
 
 
 
 
 
 
 
 
 
 
 
 
 
 
 
 
 
 
 
 
 
 
 
 
 
 
 
 
 
 
 
 
 
 
 
 
 
 
 
 
 
 
 
 
 
 
 
 
 
 
 
 
 
 
 
 
 
 
 
 
 
 
 
 
 
 
 
 
 
 
 
 
 
 
 
 
 
 
 
 
 
 
 
 
 
 
 
 
 
 
 
 
 
 
 
 
 
 
 
 
 
 
 
 
 
 
 
 
 
 
 
 
 
 
 
 
 
 
 
 
 
 
 
 
 
 
 
 
 
 
 
 
 
 
 
 
 
 
 
 
 
 
 
 
 
 
 
 
 
 
 
 
 
 
 
 
 
 
 
 
 
 
 
 
 
 
 
 
 
 
 
 
 
 
 
 
 
 
 
 
 
 
 
 
 
 
 
 
 
 
 
 
 
 
 
 
 
 
 
 
 
 
 
 
 
 
 
 
 
 
 
 
 
 
 
 
 
 
 
 
 
 
 
 
 
 
 
 
 
 
 
 
 
 
 
 
 
 
 
 
 
 
 
 
 
 
 
 
 
 
 
 
 
 
 
 
 
 
 
 
 
 
 
 
 
 
 
 
 
 
 
 
 
 
 
 
 
 
 
 
 
 
 
 
 
 
 
 
 
 
 
 
 
 
 
 
 
 
 
 
 
 
 
 
 
 
 
 
 
 
 
 
 
 
 
 
 
 
 
 
 
 
 
 
 
 
 
 
 
 
 
 
 
 
 
 
 
 
 
 
 
 
 
 
 
 
 
 
 
 
 
 
 
 
 
 
 
 
 
 
 
 
 
 
 
 
 
 
 
 
 
 
 
 
 
 
 
 
 
 
 
 
 
 
 
 
 
 
 
 
 
 
 
 
 
 
 
 
 
 
 
 
 
 
 
 
 
 
 
 
 
 
 
 
 
 
 
 
 
 
 
 
 
 
 
 
 
 
 
 
 
 
 
 
 
 
 
 
 
 
 
 
 
 
 
 
 
 
 
 
 
 
 
 
 
 
 
 
 
 
 
 
 
 
 
 
 
 
 
 
 
 
 
 
 
 
 
 
 
 
 
 
 
 
 
 
 
 
 
 
 
 
 
 
 
 
 
 
 
 
 
 
 
 
 
 
 
 
 
 
 
 
 
 
 
 
 
 
 
 
 
 
 
 
 
 
 
 
 
 
 
 
 
 
 
 
 
 
 
 
 
 
 
 
 
 
 
 
 
 
 
 
 
 
 
 
 
 
 
 
 
 
 
 
 
 
 
 
 
 
 
 
 
 
 
 
 
 
 
 
 
 
 
 
 
 
 
 
 
 
 
 
 
 
 
 
 
 
 
 
 
 
 
 
 
 
 
 
 
 
 
 
 
 
 
 
 
 
 
 
 
 
 
 
 
 
 
 
 
 
 
 
 
 
 
 
 
 
 
 
 
 
 
 
 
 
 
 
 
 
 
 
 
 
 
 
 
 
 
 
 
 
 
 
 
 
 
 
 
 
 
 
 
 
 
 
 
 
 
 
 
 
 
 
 
 
 
 
 
 
 
 
 
 
 
 
 
 
 
 
 
 
 
 
 
 
 
 
 
 
 
 
 
 
 
 
 
 
 
 
 
 
 
 
 
 
 
 
 
 
 
 
 
 
 
 
 
 
 
 
 
 
 
 
 
 
 
 
 
 
 
 
 
 
 
 
 
 
 
 
 
 
 
 
 
 
 
 
 
 
 
 
 
 
 
 
 
 
 
 
 
 
 
 
 
 
 
 
 
 
 
 
 
 
 
 
 
 
 
 
 
 
 
 
 
 
 
 
 
 
 
 
 
 
 
 
 
 
 
 
 
 
 
 
 
 
 
 
 
 
 
 
 
 
 
 
 
 
 
 
 
 
 
 
 
 
 
 
 
 
 
 
 
 
 
 
 
 
 
 
 
 
 
 
 
 
 
 
 
 
 
 
 
 
 
 
 
 
 
 
 
 
 
 
 
 
 
 
 
 
 
 
 
 
 
 
 
 
 
 
 
 
 
 
 
 
 
 
 
 
 
 
 
 
 
 
 
 
 
 
 
 
 
 
 
 
 
 
 
 
 
 
 
 
 
 
 
 
 
 
 
 
 
 
 
 
 
 
 
 
 
 
 
 
 
 
 
 
 
 
 
 
 
 
 
 
 
 
 
 
 
 
 
 
 
 
 
 
 
 
 
 
 
 
 
 
 
 
 
 
 
 
 
 
 
 
 
 
 
 
 
 
 
 
 
 
 
 
 
 
 
 
 
 
 
 
 
 
 
 
 
 
 
 
 
 
 
 
 
 
 
 
 
 
 
 
 
 
 
 
 
 
 
 
 
 
 
 
 
 
 
 
 
 
 
 
 
 
 
 
 
 
 
 
 
 
 
 
 
 
 
 
 
 
 
 
 
 
 
 
 
 
 
 
 
 
 
 
 
 
 
 
 
 
 
 
 
 
 
 
 
 
 
 
 
 
 
 
 
 
 
 
 
 
 
 
 
 
 
 
 
 
 
 
 
 
 
 
 
 
 
 
 
 
 
 
 
 
 
 
 
 
 
 
 
 
 
 
 
 
 
 
 
 
 
 
 
 
 
 
 
 
 
 
 
 
 
 
 
 
 
 
 
 
 
 
 
 
 
 
 
 
 
 
 
 
 
 
 
 
 
 
 
 
 
 
 
 
 
 
 
 
 
 
 
 
 
 
 
 
 
 
 
 
 
 
 
 
 
 
 
 
 
 
 
 
 
 
 
 
 
 
 
 
 
 
 
 
 
 
 
 
 
 
 
 
 
 
 
 
 
 
 
 
 
 
 
 
 
 
 
 
 
 
 
 
 
 
 
 
 
 
 
 
 
 
 
 
 
 
 
 
 
 
 
 
 
 
 
 
 
 
 
 
 
 
 
 
 
 
 
 
 
 
 
 
 
 
 
 
 
 
 
 
 
 
 
 
 
 
 
 
 
 
 
 
 
 
 
 
 
 
 
 
 
 
 
 
 
 
 
 
 
 
 
 
 
 
 
 
 
 
 
 
 
 
 
 
 
 
 
 
 
 
 
 
 
 
 
 
 
 
 
 
 
 
 
 
 
 
 
 
 
 
 
 
 
 
 
 
 
 
 
 
 
 
 
 
 
 
 
 
 
 
 
 
 
 
 
 
 
 
 
 
 
 
 
 
 
 
 
 
 
 
 
 
 
 
 
 
 
 
 
 
 
 
 
 
 
 
 
 
 
 
 
 
 
 
 
 
 
 
 
 
 
 
 
 
 
 
 
 
 
 
 
 
 
 
 
 
 
 
 
 
 
 
 
 
 
 
 
 
 
 
 
 
 
 
 
 
 
 
 
 
 
 
 
 
 
 
 
 
 
 
 
 
 
 
 
 
 
 
 
 
 
 
 
 
 
 
 
 
 
 
 
 
 
 
 
 
 
 
 
 
 
 
 
 
 
 
 
 
 
 
 
 
 
 
 
 
 
 
 
 
 
 
 
 
 
 
 
 
 
 
 
 
 
 
 
 
 
 
 
 
 
 
 
 
 
 
 
 
 
 
 
 
 
 
 
 
 
 
 
 
 
 
 
 
 
 
 
 
 
 
 
 
 
 
 
 
 
 
 
 
 
 
 
 
 
 
 
 
 
 
 
 
 
 
 
 
 
 
 
 
 
 
 
 
 
 
 
 
 
 
 
 
 
 
 
 
 
 
 
 
 
 
 
 
 
 
 
 
 
 
 
 
 
 
 
 
 
 
 
 
 
 
 
 
 
 
 
 
 
 
 
 
 
 
 
 
 
 
 
 
 
 
 
 
 
 
 
 
 
 
 
 
 
 
 
 
 
 
 
 
 
 
 
 
 
 
 
 
 
 
 
 
 
 
 
 
 
 
 
 
 
 
 
 
 
 
 
 
 
 
 
 
 
 
 
 
 
 
 
 
 
 
 
 
 
 
 
 
 
 
 
 
 
 
 
 
 
 
 
 
 
 
 
 
 
 
 
 
 
 
 
 
 
 
 
 
 
 
 
 
 
 
 
 
 
 
 
 
 
 
 
 
 
 
 
 
 
 
 
 
 
 
 
 
 
 
 
 
 
 
 
 
 
 
 
 
 
 
 
 
 
 
 
 
 
 
 
 
 
 
 
 
 
 
 
 
 
 
 
 
 
 
 
 
 
 
 
 
 
 
 
 
 
 
 
 
 
 
 
 
 
 
 
 
 
 
 
 
 
 
 
 
 
 
 
 
 
 
 
 
 
 
 
 
 
 
 
 
 
 
 
 
 
 
 
 
 
 
 
 
 
 
 
 
 
 
 
 
 
 
 
 
 
 
 
 
 
 
 
 
 
 
 
 
 
 
 
 
 
 
 
 
 
 
 
 
 
 
 
 
 
 
 
 
 
 
 
 
 
 
 
 
 
 
 
 
 
 
 
 
 
 
 
 
 
 
 
 
 
 
 
 
 
 
 
 
 
 
 
 
 
 
 
 
 
 
 
 
 
 
 
 
 
 
 
 
 
 
 
 
 
 
 
 
 
 
 
 
 
 
 
 
 
 
 
 
 
 
 
 
 
 
 
 
 
 
 
 
 
 
 
 
 
 
 
 
 
 
 
 
 
 
 
 
 
 
 
 
 
 
 
 
 
 
 
 
 
 
 
 
 
 
 
 
 
 
 
 
 
 
 
 
 
 
 
 
 
 
 
 
 
 
 
 
 
 
 
 
 
 
 
 
 
 
 
 
 
 
 
 
 
 
 
 
 
 
 
 
 
 
 
 
 
 
 
 
 
 
 
 
 
 
 
 
 
 
 
 
 
 
 
 
 
 
 
 
 
 
 
 
 
 
 
 
 
 
 
 
 
 
 
 
 
 
 
 
 
 
 
 
 
 
 
 
 
 
 
 
 
 
 
 
 
 
 
 
 
 
 
 
 
 
 
 
 
 
 
 
 
 
 
 
 
 
 
 
 
 
 
 
 
 
 
 
 
 
 
 
 
 
 
 
 
 
 
 
 
 
 
 
 
 
 
 
 
 
 
 
 
 
 
 
 
 
 
 
 
 
 
 
 
 
 
 
 
 
 
 
 
 
 
 
 
 
 
 
 
 
 
 
 
 
 
 
 
 
 
 
 
 
 
 
 
 
 
 
 
 
 
 
 
 
 
 
 
 
 
 
 
 
 
 
 
 
 
 
 
 
 
 
 
 
 
 
 
 
 
 
 
 
 
 
 
 
 
 
 
 
 
 
 
 
 
 
 
 
 
 
 
 
 
 
 
 
 
 
 
 
 
 
 
 
 
 
 
 
 
 
 
 
 
 
 
 
 
 
 
 
 
 
 
 
 
 
 
 
 
 
 
 
 
 
 
 
 
 
 
 
 
 
 
 
 
 
 
 
 
 
 
 
 
 
 
 
 
 
 
 
 
 
 
 
 
 
 
 
 
 
 
 
 
 
 
 
 
 
 
 
 
 
 
 
 
 
 
 
 
 
 
 
 
 
 
 
 
 
 
 
 
 
 
 
 
 
 
 
 
 
 
 
 
 
 
 
 
 
 
 
 
 
 
 
 
 
 
 
 
 
 
 
 
 
 
 
 
 
 
 
 
 
 
 
 
 
 
 
 
 
 
 
 
 
 
 
 
 
 
 
 
 
 
 
 
 
 
 
 
 
 
 
 
 
 
 
 
 
 
 
 
 
 
 
 
 
 
 
 
 
 
 
 
 
 
 
 
 
 
 
 
 
 
 
 
 
 
 
 
 
 
 
 
 
 
 
 
 
 
 
 
 
 
 
 
 
 
 
 
 
 
 
 
 
 
 
 
 
 
 
 
 
 
 
 
 
 
 
 
 
 
 
 
 
 
 
 
 
 
 
 
 
 
 
 
 
 
 
 
 
 
 
 
 
 
 
 
 
 
 
 
 
 
 
 
 
 
 
 
 
 
 
 
 
 
 
 
 
 
 
 
 
 
 
 
 
 
 
 
 
 
 
 
 
 
 
 
 
 
 
 
 
 
 
 
 
 
 
 
 
 
 
 
 
 
 
 
 
 
 
 
 
 
 
 
 
 
 
 
 
 
 
 
 
 
 
 
 
 
 
 
 
 
 
 
 
 
 
 
 
 
 
 
 
 
 
 
 
 
 
 
 
 
 
 
 
 
 
 
 
 
 
 
 
 
 
 
 
 
 
 
 
 
 
 
 
 
 
 
 
 
 
 
 
 
 
 
 
 
 
 
 
 
 
 
 
 
 
 
 
 
 
 
 
 
 
 
 
 
 
 
 
 
 
 
 
 
 
 
 
 
 
 
 
 
 
 
 
 
 
 
 
 
 
 
 
 
 
 
 
 
 
 
 
 
 
 
 
 
 
 
 
 
 
 
 
 
 
 
 
 
 
 
 
 
 
 
 
 
 
 
 
 
 
 
 
 
 
 
 
 
 
 
 
 
 
 
 
 
 
 
 
 
 
 
 
 
 
 
 
 
 
 
 
 
 
 
 
 
 
 
 
 
 
 
 
 
 
 
 
 
 
 
 
 
 
 
 
 
 
 
 
 
 
 
 
 
 
 
 
 
 
 
 
 
 
 
 
 
 
 
 
 
 
 
 
 
 
 
 
 
 
 
 
 
 
 
 
 
 
 
 
 
 
 
 
 
 
 
 
 
 
 
 
 
 
 
 
 
 
 
 
 
 
 
 
 
 
 
 
 
 
 
 
 
 
 
 
 
 
 
 
 
 
 
 
 
 
 
 
 
 
 
 
 
 
 
 
 
 
 
 
 
 
 
 
 
 
 
 
 
 
 
 
 
 
 
 
 
 
 
 
 
 
 
 
 
 
 
 
 
 
 
 
 
 
 
 
 
 
 
 
 
 
 
 
 
 
 
 
 
 
 
 
 
 
 
 
 
 
 
 
 
 
 
 
 
 
 
 
 
 
 
 
 
 
 
 
 
 
 
 
 
 
 
 
 
 
 
 
 
 
 
 
 
 
 
 
 
 
 
 
 
 
 
 
 
 
 
 
 
 
 
 
 
 
 
 
 
 
 
 
 
 
 
 
 
 
 
 
 
 
 
 
 
 
 
 
 
 
 
 
 
 
 
 
 
 
 
 
 
 
 
 
 
 
 
 
 
 
 
 
 
 
 
 
 
 
 
 
 
 
 
 
 
 
 
 
 
 
 
 
 
 
 
 
 
 
 
 
 
 
 
 
 
 
 
 
 
 
 
 
 
 
 
 
 
 
 
 
 
 
 
 
 
 
 
 
 
 
 
 
 
 
 
 
 
 
 
 
 
 
 
 
 
 
 
 
 
 
 
 
 
 
 
 
 
 
 
 
 
 
 
 
 
 
 
 
 
 
 
 
 
 
 
 
 
 
 
 
 
 
 
 
 
 
 
 
 
 
 
 
 
 
 
 
 
 
 
 
 
 
 
 
 
 
 
 
 
 
 
 
 
 
 
 
 
 
 
 
 
 
 
 
 
 
 
 
 
 
 
 
 
 
 
 
 
 
 
 
 
 
 
 
 
 
 
 
 
 
 
 
 
 
 
 
 
 
 
 
 
 
 
 
 
 
 
 
 
 
 
 
 
 
 
 
 
 
 
 
 
 
 
 
 
 
 
 
 
 
 
 
 
 
 
 
 
 
 
 
 
 
 
 
 
 
 
 
 
 
 
 
 
 
 
 
 
 
 
 
 
 
 
 
 
 
 
 
 
 
 
 
 
 
 
 
 
 
 
 
 
 
 
 
 
 
 
 
 
 
 
 
 
 
 
 
 
 
 
 
 
 
 
 
 
 
 
 
 
 
 
 
 
 
 
 
 
 
 
 
 
 
 
 
 
 
 
 
 
 
 
 
 
 
 
 
 
 
 
 
 
 
 
 
 
 
 
 
 
 
 
 
 
 
 
 
 
 
 
 
 
 
 
 
 
 
 
 
 
 
 
 
 
 
 
 
 
 
 
 
 
 
 
 
 
 
 
 
 
 
 
 
 
 
 
 
 
 
 
 
 
 
 
 
 
 
 
 
 
 
 
 
 
 
 
 
 
 
 
 
 
 
 
 
 
 
 
 
 
 
 
 
 
 
 
 
 
 
 
 
 
 
 
 
 
 
 
 
 
 
 
 
 
 
 
 
 
 
 
 
 
 
 
 
 
 
 
 
 
 
 
 
 
 
 
 
 
 
 
 
 
 
 
 
 
 
 
 
 
 
 
 
 
 
 
 
 
 
 
 
 
 
 
 
 
 
 
 
 
 
 
 
 
 
 
 
 
 
 
 
 
 
 
 
 
 
 
 
 
 
 
 
 
 
 
 
 
 
 
 
 
 
 
 
 
 
 
 
 
 
 
 
 
 
 
 
 
 
 
 
 
 
 
 
 
 
 
 
 
 
 
 
 
 
 
 
 
 
 
 
 
 
 
 
 
 
 
 
 
 
 
 
 
 
 
 
 
 
 
 
 
 
 
 
 
 
 
 
 
 
 
 
 
 
 
 
 
 
 
 
 
 
 
 
 
 
 
 
 
 
 
 
 
 
 
 
 
 
 
 
 
 
 
 
 
 
 
 
 
 
 
 
 
 
 
 
 
 
 
 
 
 
 
 
 
 
 
 
 
 
 
 
 
 
 
 
 
 
 
 
 
 
 
 
 
 
 
 
 
 
 
 
 
 
 
 
 
 
 
 
 
 
 
 
 
 
 
 
 
 
 
 
 
 
 
 
 
 
 
 
 
 
 
 
 
 
 
 
 
 
 
 
 
 
 
 
 
 
 
 
 
 
 
 
 
 
 
 
 
 
 
 
 
 
 
 
 
 
 
 
 
 
 
 
 
 
 
 
 
 
 
 
 
 
 
 
 
 
 
 
 
 
 
 
 
 
 
 
 
 
 
 
 
 
 
 
 
 
 
 
 
 
 
 
 
 
 
 
 
 
 
 
 
 
 
 
 
 
 
 
 
 
 
 
 
 
 
 
 
 
 
 
 
 
 
 
 
 
 
 
 
 
 
 
 
 
 
 
 
 
 
 
 
 
 
 
 
 
 
 
 
 
 
 
 
 
 
 
 
 
 
 
 
 
 
 
 
 
 
 
 
 
 
 
 
 
 
 
 
 
 
 
 
 
 
 
 
 
 
 
 
 
 
 
 
 
 
 
 
 
 
 
 
 
 
 
 
 
 
 
 
 
 
 
 
 
 
 
 
 
 
 
 
 
 
 
 
 
 
 
 
 
 
 
 
 
 
 
 
 
 
 
 
 
 
 
 
 
 
 
 
 
 
 
 
 
 
 
 
 
 
 
 
 
 
 
 
 
 
 
 
 
 
 
 
 
 
 
 
 
 
 
 
 
 
 
 
 
 
 
 
 
 
 
 
 
 
 
 
 
 
 
 
 
 
 
 
 
 
 
 
 
 
 
 
 
 
 
 
 
 
 
 
 
 
 
 
 
 
 
 
 
 
 
 
 
 
 
 
 
 
 
 
 
 
 
 
 
 
 
 
 
 
 
 
 
 
 
 
 
 
 
 
 
 
 
 
 
 
 
 
 
 
 
 
 
 
 
 
 
 
 
 
 
 
 
 
 
 
 
 
 
 
 
 
 
 
 
 
 
 
 
 
 
 
 
 
 
 
 
 
 
 
 
 
 
 
 
 
 
 
 
 
 
 
 
 
 
 
 
 
 
 
 
 
 
 
 
 
 
 
 
 
 
 
 
 
 
 
 
 
 
 
 
 
 
 
 
 
 
 
 
 
 
 
 
 
 
 
 
 
 
 
 
 
 
 
 
 
 
 
 
 
 
 
 
 
 
 
 
 
 
 
 
 
 
 
 
 
 
 
 
 
 
 
 
 
 
 
 
 
 
 
 
 
 
 
 
 
 
 
 
 
 
 
 
 
 
 
 
 
 
 
 
 
 
 
 
 
 
 
 
 
 
 
 
 
 
 
 
 
 
 
 
 
 
 
 
 
 
 
 
 
 
 
 
 
 
 
 
 
 
 
 
 
 
 
 
 
 
 
 
 
 
 
 
 
 
 
 
 
 
 
 
 
 
 
 
 
 
 
 
 
 
 
 
 
 
 
 
 
 
 
 
 
 
 
 
 
 
 
 
 
 
 
 
 
 
 
 
 
 
 
 
 
 
 
 
 
 
 
 
 
 
 
 
 
 
 
 
 
 
 
 
 
 
 
 
 
 
 
 
 
 
 
 
 
 
 
 
 
 
 
 
 
 
 
 
 
 
 
 
 
 
 
 
 
 
 
 
 
 
 
 
 
 
 
 
 
 
 
 
 
 
 
 
 
 
 
 
 
 
 
 
 
 
 
 
 
 
 
 
 
 
 
 
 
 
 
 
 
 
 
 
 
 
 
 
 
 
 
 
 
 
 
 
 
 
 
 
 
 
 
 
 
 
 
 
 
 
 
 
 
 
 
 
 
 
 
 
 
 

 

    View Table (.txt)         Total  0  1  2  3  4  5  6  8  9  10  11  13  14  15  16  17  18  19  20  21  22  23  24  25  26  27  28  29  30  31  32  41  42  43  44  45  46  47  48  49  50  51  52  53  54  56  57  58  59  60  61  62  64  65  66  67  68  69  70  71  72  73  79  80  81  82  83  84  86  87  88  89  90  91  92  93  94  95  96  97  98  99  100  101  102  103  104  105  112  113  114  115  116  117  118  120  121  122  123  124  125  126  127  128  129  130  131  132  134  135  137  138  139  140  141  142  143  144  145  146  147  148  149  150  151  152  153  154  155  157  158  159  160  161  162  163  164  166  167  168  169  170  172  173  174  175  176  177  178  179  180  181  182  183  184  185  186  187  188  189  190  191  192  193  194  195  196  197  198  199  200  201  202  203  204  205  206  208  209  210  211  213  214  215  216  217  218  219  220  221  222  223  225  226  227  228  229  230  231  234  235  236  237  238  240  241  243  244  245  246  247  249  250  251  252  253  254  255  257  258  259  260  261  262  263  264  266  267  268  269  270  271  272  273  274  275  278  279  280  281  283  284  285  286  287  288  289  290  292  293  294  295  296  297  298  299  300  301  302  303  304  305  306  307  308  309  312  314  317  318  319  321  322  323  326  327  330  331  332  333  334  335  336  337  338  339  340  341  343  344  345  348  349  350  351  352  353  354  355  356  357  358  359  360  361  362  363  365  366  368  369  371  372  373  374  375  376  377  378  379  380  381  382  383  384  385  386  387  389  390  391  392  393  394  395  396  397  398  399  400  401  402  403  405  406  410  411  412  413  415  416  417  418  420  438  439  440    Legend  Taxonomy  count  %  %  %  %  %  %  %  %  %  %  %  %  %  %  %  %  %  %  %  %  %  %  %  %  %  %  %  %  %  %  %  %  %  %  %  %  %  %  %  %  %  %  %  %  %  %  %  %  %  %  %  %  %  %  %  %  %  %  %  %  %  %  %  %  %  %  %  %  %  %  %  %  %  %  %  %  %  %  %  %  %  %  %  %  %  %  %  %  %  %  %  %  %  %  %  %  %  %  %  %  %  %  %  %  %  %  %  %  %  %  %  %  %  %  %  %  %  %  %  %  %  %  %  %  %  %  %  %  %  %  %  %  %  %  %  %  %  %  %  %  %  %  %  %  %  %  %  %  %  %  %  %  %  %  %  %  %  %  %  %  %  %  %  %  %  %  %  %  %  %  %  %  %  %  %  %  %  %  %  %  %  %  %  %  %  %  %  %  %  %  %  %  %  %  %  %  %  %  %  %  %  %  %  %  %  %  %  %  %  %  %  %  %  %  %  %  %  %  %  %  %  %  %  %  %  %  %  %  %  %  %  %  %  %  %  %  %  %  %  %  %  %  %  %  %  %  %  %  %  %  %  %  %  %  %  %  %  %  %  %  %  %  %  %  %  %  %  %  %  %  %  %  %  %  %  %  %  %  %  %  %  %  %  %  %  %  %  %  %  %  %  %  %  %  %  %  %  %  %  %  %  %  %  %  %  %  %  %  %  %  %  %  %  %  %  %  %  %  %  %  %  %  %  %  %  %  %  %  %  %  %  %  %  %  %  %  %  %  %  %  %  %  %  %  %  %  %  %  %  %  %  %  %  %  %  %  %  %        k__Archaea;p__Crenarchaeota;c__Sd-NA; o__NRP-J       3    0.0%    0.0%    0.0%    0.0%    0.0%    0.0%    0.0%    0.0%    0.0%    0.0%    0.0%    0.0%    0.0%    0.0%    0.0%    0.0%    0.0%    0.0%    0.0%    0.0%    0.0%    0.0%    0.0%    0.0%    0.0%    0.0%    0.0%    0.0%    0.0%    0.0%    0.0%    0.0%    0.0%    0.0%    0.0%    0.0%    0.0%    0.0%    0.0%    0.0%    0.0%    0.0%    0.0%    0.0%    0.0%    0.0%    0.0%    0.0%    0.0%    0.0%    0.0%    0.0%    0.0%    0.0%    0.0%    0.0%    0.0%    0.0%    0.0%    0.0%    0.0%    0.0%    0.0%    0.0%    0.0%    0.0%    0.0%    0.0%    0.0%    0.0%    0.0%    0.0%    0.0%    0.0%    0.0%    0.0%    0.0%    0.0%    0.0%    0.0%    0.0%    0.0%    0.0%    0.0%    0.0%    0.0%    0.0%    0.0%    0.0%    0.0%    0.0%    0.0%    0.0%    0.0%    0.0%    0.0%    0.0%    0.0%    0.0%    0.0%    0.0%    0.0%    0.0%    0.0%    0.0%    0.0%    0.0%    0.0%    0.0%    0.0%    0.0%    0.0%    0.0%    0.0%    0.0%    0.0%    0.0%    0.0%    0.0%    0.0%    0.0%    0.0%    0.0%    0.0%    0.0%    0.0%    0.0%    0.0%    0.0%    0.0%    0.0%    0.0%    0.0%    0.0%    0.0%    0.0%    0.0%    0.0%    0.0%    0.0%    0.0%    0.0%    0.0%    0.0%    0.0%    0.0%    0.0%    0.0%    0.0%    0.0%    0.0%    0.0%    0.0%    0.0%    0.0%    0.0%    0.0%    0.0%    0.0%    0.0%    0.0%    0.0%    0.0%    0.0%    0.0%    0.0%    0.0%    0.0%    0.0%    0.0%    0.0%    0.0%    0.0%    0.0%    0.0%    0.0%    0.0%    0.0%    0.0%    0.0%    0.0%    0.0%    0.0%    0.0%    0.0%    0.0%    0.0%    0.0%    0.0%    0.0%    0.0%    0.0%    0.0%    0.0%    0.0%    0.0%    0.0%    0.0%    0.0%    0.0%    0.0%    0.0%    0.0%    0.0%    0.0%    0.0%    0.0%    0.0%    0.0%    0.0%    0.0%    0.0%    0.0%    0.0%    0.0%    0.0%    0.0%    0.0%    0.0%    0.0%    0.0%    0.0%    0.0%    0.0%    0.0%    0.0%    0.0%    0.0%    0.0%    0.0%    0.0%    0.0%    0.0%    0.0%    0.0%    0.0%    0.0%    0.0%    0.0%    0.0%    0.0%    0.0%    0.0%    0.0%    0.0%    0.0%    0.0%    0.0%    0.0%    0.0%    0.0%    0.0%    0.0%    0.0%    0.0%    0.0%    0.0%    0.0%    0.0%    0.0%    0.0%    0.0%    0.0%    0.0%    0.0%    0.0%    0.0%    0.0%    0.0%    0.0%    0.0%    0.0%    0.0%    0.0%    0.0%    0.0%    0.0%    0.0%    0.0%    0.0%    0.0%    0.0%    0.0%    0.0%    0.0%    0.0%    0.0%    0.0%    0.0%    0.0%    0.0%    0.0%    0.0%    0.0%    0.0%    0.0%    0.0%    0.0%    0.0%    0.0%    0.0%    0.0%    0.0%    0.0%    0.0%    0.0%    0.0%    0.0%    0.0%    0.0%    0.0%    0.0%    0.0%    0.0%    0.0%    0.0%    0.0%    0.0%    0.0%    0.0%    0.0%    0.0%    0.0%    0.0%    0.0%    0.0%    0.0%    0.0%    0.0%    0.0%    0.0%    0.0%    0.0%    0.0%    0.0%    0.0%    0.0%    0.0%    0.0%    0.0%    0.0%    0.0%    0.0%    0.0%    0.0%    0.0%    0.0%    0.0%    0.0%    0.0%    0.0%    0.0%    0.0%    0.0%    0.0%    0.0%    0.0%    0.0%        k__Archaea;p__Crenarchaeota;c__Thaumarchaeota; o__Cenarchaeales       4    0.0%    0.0%    0.0%    0.0%    0.0%    0.0%    0.0%    0.0%    0.0%    0.0%    0.0%    0.0%    0.0%    0.0%    0.0%    0.0%    0.0%    0.0%    0.0%    0.0%    0.0%    0.0%    0.0%    0.0%    0.0%    0.0%    0.0%    0.0%    0.0%    0.0%    0.0%    0.0%    0.0%    0.0%    0.0%    0.0%    0.0%    0.0%    0.0%    0.0%    0.0%    0.0%    0.0%    0.0%    0.0%    0.0%    0.0%    0.0%    0.0%    0.0%    0.0%    0.0%    0.0%    0.0%    0.0%    0.0%    0.0%    0.0%    0.0%    0.0%    0.0%    0.0%    0.0%    0.0%    0.0%    0.0%    0.0%    0.0%    0.0%    0.0%    0.0%    0.0%    0.0%    0.0%    0.0%    0.0%    0.0%    0.0%    0.0%    0.0%    0.0%    0.0%    0.0%    0.0%    0.0%    0.0%    0.0%    0.0%    0.0%    0.0%    0.0%    0.0%    0.0%    0.0%    0.0%    0.0%    0.0%    0.0%    0.0%    0.0%    0.0%    0.0%    0.0%    0.0%    0.0%    0.0%    0.0%    0.0%    0.0%    0.0%    0.0%    0.0%    0.0%    0.0%    0.0%    0.0%    0.0%    0.0%    0.0%    0.0%    0.0%    0.0%    0.0%    0.0%    0.0%    0.0%    0.0%    0.0%    0.0%    0.0%    0.0%    0.0%    0.0%    0.0%    0.0%    0.0%    0.0%    0.0%    0.0%    0.0%    0.0%    0.0%    0.0%    0.0%    0.0%    0.0%    0.0%    0.0%    0.0%    0.0%    0.0%    0.0%    0.0%    0.0%    0.0%    0.0%    0.0%    0.0%    0.0%    0.0%    0.0%    0.0%    0.0%    0.0%    0.0%    0.0%    0.0%    0.0%    0.0%    0.0%    0.0%    0.0%    0.0%    0.0%    0.0%    0.0%    0.0%    0.0%    0.0%    0.0%    0.0%    0.0%    0.0%    0.0%    0.0%    0.0%    0.0%    0.0%    0.0%    0.0%    0.0%    0.0%    0.0%    0.0%    0.0%    0.0%    0.0%    0.0%    0.0%    0.0%    0.0%    0.0%    0.0%    0.0%    0.0%    0.0%    0.0%    0.0%    0.0%    0.0%    0.0%    0.0%    0.0%    0.0%    0.0%    0.0%    0.0%    0.0%    0.0%    0.0%    0.0%    0.0%    0.0%    0.0%    0.0%    0.0%    0.0%    0.0%    0.0%    0.0%    0.0%    0.0%    0.0%    0.0%    0.0%    0.0%    0.0%    0.0%    0.0%    0.0%    0.0%    0.0%    0.0%    0.0%    0.0%    0.0%    0.0%    0.0%    0.0%    0.0%    0.0%    0.0%    0.0%    0.0%    0.0%    0.0%    0.0%    0.0%    0.0%    0.0%    0.0%    0.0%    0.0%    0.0%    0.0%    0.0%    0.0%    0.0%    0.0%    0.0%    0.0%    0.0%    0.0%    0.0%    0.0%    0.0%    0.0%    0.0%    0.0%    0.0%    0.0%    0.0%    0.0%    0.0%    0.0%    0.0%    0.0%    0.0%    0.0%    0.0%    0.0%    0.0%    0.0%    0.0%    0.0%    0.0%    0.0%    0.0%    0.0%    0.0%    0.0%    0.0%    0.0%    0.0%    0.0%    0.0%    0.0%    0.0%    0.0%    0.0%    0.0%    0.0%    0.0%    0.0%    0.0%    0.0%    0.0%    0.0%    0.0%    0.0%    0.0%    0.0%    0.0%    0.0%    0.0%    0.0%    0.0%    0.0%    0.0%    0.0%    0.0%    0.0%    0.0%    0.0%    0.0%    0.0%    0.0%    0.0%    0.0%    0.0%    0.0%    0.0%    0.0%    0.0%    0.0%    0.0%    0.0%    0.0%    0.0%    0.0%    0.0%    0.0%    0.0%    0.0%    0.0%    0.0%    0.0%    0.0%        k__Archaea;p__Crenarchaeota;c__Thaumarchaeota; o__Nitrososphaerales    3911    0.0%    0.0%    0.0%    0.0%    0.0%    0.0%    0.0%    0.0%    0.0%    0.0%    0.0%    0.0%    0.0%    0.0%    0.0%    0.0%    0.0%    0.0%    0.0%    0.0%    0.0%    0.0%    0.0%    0.0%    0.0%    0.0%    0.0%    0.0%    0.0%    0.0%    0.0%    0.0%    0.0%    0.0%    0.0%    0.0%    0.0%    0.0%    0.0%    0.1%    0.0%    0.0%    0.0%    0.0%    0.0%    0.0%    0.0%    0.0%    0.0%    0.0%    0.0%    0.0%    0.0%    0.0%    0.1%    0.0%    0.0%    0.0%    0.0%    0.1%    0.0%    0.0%    0.0%    0.1%    0.0%    0.1%    0.0%    0.1%    0.0%    0.1%    0.0%    0.0%    0.0%    0.0%    0.0%    0.0%    0.0%    0.0%    0.0%    0.0%    0.0%    0.0%    0.0%    0.0%    0.0%    0.0%    0.0%    0.0%    0.0%    0.0%    0.0%    0.0%    0.0%    0.0%    0.0%    0.0%    0.1%    0.0%    0.0%    0.0%    0.1%    0.0%    0.0%    0.0%    0.0%    0.0%    0.1%    0.0%    0.0%    0.0%    0.0%    0.1%    0.0%    0.0%    0.0%    0.0%    0.0%    0.0%    0.0%    0.2%    0.0%    0.0%    0.1%    0.1%    0.0%    0.1%    0.1%    0.0%    0.1%    0.2%    0.1%    0.0%    0.0%    0.0%    0.2%    0.0%    0.0%    0.0%    0.0%    0.0%    0.0%    0.0%    0.0%    0.0%    0.1%    0.0%    0.0%    0.0%    0.0%    0.0%    0.0%    0.0%    0.1%    0.0%    0.0%    0.0%    0.1%    0.0%    0.0%    0.1%    0.3%    0.1%    0.1%    0.0%    0.0%    0.0%    0.1%    0.0%    0.0%    0.0%    0.0%    0.1%    0.0%    0.0%    0.0%    0.0%    0.0%    0.0%    0.0%    0.0%    0.0%    0.0%    0.0%    0.0%    0.0%    0.0%    0.0%    0.0%    0.0%    0.0%    0.0%    0.0%    0.0%    0.0%    0.0%    0.0%    0.0%    0.0%    0.0%    0.0%    0.0%    0.0%    0.0%    0.1%    0.1%    0.0%    0.0%    0.0%    0.1%    0.1%    0.0%    0.0%    0.0%    0.0%    0.0%    0.0%    0.0%    0.0%    0.0%    0.0%    0.0%    0.0%    0.0%    0.0%    0.0%    0.0%    0.0%    0.0%    0.0%    0.0%    0.0%    0.0%    0.1%    0.0%    0.1%    0.0%    0.1%    0.0%    0.0%    0.0%    0.0%    0.0%    0.0%    0.0%    0.0%    0.0%    0.0%    0.0%    0.0%    0.0%    0.1%    0.1%    0.0%    0.0%    0.0%    0.0%    0.0%    0.0%    0.0%    0.0%    0.1%    0.0%    0.0%    0.0%    0.0%    0.0%    0.0%    0.0%    0.0%    0.0%    0.0%    0.0%    0.0%    0.0%    0.0%    0.0%    0.0%    0.0%    0.0%    0.0%    0.0%    0.0%    0.0%    0.0%    0.0%    0.0%    0.0%    0.1%    0.0%    0.0%    0.0%    0.0%    0.0%    0.1%    0.1%    0.0%    0.0%    0.0%    0.0%    0.0%    0.0%    0.0%    0.0%    0.0%    0.0%    0.0%    0.0%    0.0%    0.0%    0.0%    0.0%    0.0%    0.0%    0.0%    0.0%    0.0%    0.0%    0.0%    0.0%    0.0%    0.0%    0.0%    0.0%    0.0%    0.0%    0.0%    0.0%    0.0%    0.0%    0.0%    0.0%    0.0%    0.0%    0.0%    0.0%    0.0%    0.0%    0.0%    0.0%    0.0%    0.0%    0.0%    0.0%    0.0%    0.0%    0.0%    0.0%    0.0%    0.0%    0.0%    0.0%    0.0%    0.0%    0.0%    0.0%    0.0%    0.0%    0.0%        k__Archaea;p__Euryarchaeota;c__Halobacteria; o__Halobacteriales      48    0.0%    0.0%    0.0%    0.0%    0.0%    0.0%    0.0%    0.0%    0.0%    0.0%    0.0%    0.0%    0.0%    0.0%    0.0%    0.0%    0.0%    0.0%    0.0%    0.0%    0.0%    0.0%    0.0%    0.0%    0.0%    0.0%    0.0%    0.0%    0.0%    0.0%    0.0%    0.0%    0.0%    0.0%    0.0%    0.0%    0.0%    0.0%    0.0%    0.0%    0.0%    0.0%    0.0%    0.0%    0.0%    0.0%    0.0%    0.0%    0.0%    0.0%    0.0%    0.0%    0.0%    0.0%    0.0%    0.0%    0.0%    0.0%    0.0%    0.0%    0.0%    0.0%    0.0%    0.0%    0.0%    0.0%    0.0%    0.0%    0.0%    0.0%    0.0%    0.0%    0.0%    0.0%    0.0%    0.0%    0.0%    0.0%    0.0%    0.0%    0.0%    0.0%    0.0%    0.0%    0.0%    0.0%    0.0%    0.0%    0.0%    0.0%    0.0%    0.0%    0.0%    0.0%    0.0%    0.0%    0.0%    0.0%    0.0%    0.0%    0.0%    0.0%    0.0%    0.0%    0.0%    0.0%    0.0%    0.0%    0.0%    0.0%    0.0%    0.0%    0.0%    0.0%    0.0%    0.0%    0.0%    0.0%    0.0%    0.0%    0.0%    0.0%    0.0%    0.0%    0.0%    0.0%    0.0%    0.0%    0.0%    0.0%    0.0%    0.0%    0.0%    0.0%    0.0%    0.0%    0.0%    0.0%    0.0%    0.0%    0.0%    0.0%    0.0%    0.0%    0.0%    0.0%    0.0%    0.0%    0.0%    0.0%    0.0%    0.0%    0.0%    0.0%    0.0%    0.0%    0.0%    0.0%    0.0%    0.0%    0.0%    0.0%    0.0%    0.0%    0.0%    0.0%    0.0%    0.0%    0.0%    0.0%    0.0%    0.0%    0.0%    0.0%    0.0%    0.0%    0.0%    0.0%    0.0%    0.0%    0.0%    0.0%    0.0%    0.0%    0.0%    0.0%    0.0%    0.0%    0.0%    0.0%    0.0%    0.0%    0.0%    0.0%    0.0%    0.0%    0.0%    0.0%    0.0%    0.0%    0.0%    0.0%    0.0%    0.0%    0.0%    0.0%    0.0%    0.0%    0.0%    0.0%    0.0%    0.0%    0.0%    0.0%    0.0%    0.0%    0.0%    0.0%    0.0%    0.0%    0.0%    0.0%    0.0%    0.0%    0.0%    0.0%    0.0%    0.0%    0.0%    0.0%    0.0%    0.0%    0.0%    0.0%    0.0%    0.0%    0.0%    0.0%    0.0%    0.0%    0.0%    0.0%    0.0%    0.0%    0.0%    0.0%    0.0%    0.0%    0.0%    0.0%    0.0%    0.0%    0.0%    0.0%    0.0%    0.0%    0.0%    0.0%    0.0%    0.0%    0.0%    0.0%    0.0%    0.0%    0.0%    0.0%    0.0%    0.0%    0.0%    0.0%    0.0%    0.0%    0.0%    0.0%    0.0%    0.0%    0.0%    0.0%    0.0%    0.0%    0.0%    0.0%    0.0%    0.0%    0.0%    0.0%    0.0%    0.0%    0.0%    0.0%    0.0%    0.0%    0.0%    0.0%    0.0%    0.0%    0.0%    0.0%    0.0%    0.0%    0.0%    0.0%    0.0%    0.0%    0.0%    0.0%    0.0%    0.0%    0.0%    0.0%    0.0%    0.0%    0.0%    0.0%    0.0%    0.0%    0.0%    0.0%    0.0%    0.0%    0.0%    0.0%    0.0%    0.0%    0.0%    0.0%    0.0%    0.0%    0.0%    0.0%    0.0%    0.0%    0.0%    0.0%    0.0%    0.0%    0.0%    0.0%    0.0%    0.0%    0.0%    0.0%    0.0%    0.0%    0.0%    0.0%    0.0%    0.0%    0.0%    0.0%    0.0%    0.0%    0.0%    0.0%    0.0%    0.0%    0.0%    0.0%        k__Archaea;p__Euryarchaeota;c__Methanobacteria; o__Methanobacteriales     137    0.0%    0.0%    0.0%    0.0%    0.0%    0.0%    0.0%    0.0%    0.0%    0.0%    0.0%    0.0%    0.0%    0.0%    0.0%    0.0%    0.0%    0.0%    0.0%    0.0%    0.0%    0.0%    0.0%    0.0%    0.0%    0.0%    0.0%    0.0%    0.0%    0.0%    0.0%    0.0%    0.0%    0.0%    0.0%    0.0%    0.0%    0.0%    0.0%    0.0%    0.0%    0.0%    0.0%    0.0%    0.0%    0.0%    0.0%    0.0%    0.0%    0.0%    0.0%    0.0%    0.0%    0.0%    0.0%    0.0%    0.0%    0.0%    0.0%    0.0%    0.0%    0.0%    0.0%    0.0%    0.0%    0.0%    0.0%    0.0%    0.0%    0.0%    0.0%    0.0%    0.0%    0.0%    0.0%    0.0%    0.0%    0.0%    0.0%    0.0%    0.0%    0.0%    0.0%    0.0%    0.1%    0.0%    0.0%    0.0%    0.0%    0.0%    0.0%    0.0%    0.0%    0.0%    0.0%    0.0%    0.0%    0.0%    0.0%    0.0%    0.0%    0.0%    0.0%    0.0%    0.0%    0.0%    0.0%    0.0%    0.0%    0.0%    0.0%    0.0%    0.0%    0.0%    0.0%    0.0%    0.0%    0.0%    0.0%    0.0%    0.0%    0.0%    0.0%    0.0%    0.0%    0.0%    0.0%    0.0%    0.0%    0.0%    0.0%    0.0%    0.0%    0.0%    0.0%    0.0%    0.0%    0.0%    0.0%    0.0%    0.0%    0.0%    0.0%    0.0%    0.0%    0.0%    0.0%    0.0%    0.0%    0.0%    0.0%    0.0%    0.0%    0.0%    0.0%    0.0%    0.0%    0.0%    0.0%    0.0%    0.0%    0.0%    0.0%    0.0%    0.0%    0.0%    0.0%    0.0%    0.0%    0.0%    0.0%    0.0%    0.0%    0.0%    0.0%    0.0%    0.0%    0.0%    0.0%    0.0%    0.0%    0.0%    0.0%    0.0%    0.0%    0.0%    0.0%    0.0%    0.0%    0.0%    0.0%    0.0%    0.0%    0.0%    0.0%    0.0%    0.0%    0.0%    0.0%    0.0%    0.0%    0.0%    0.0%    0.0%    0.0%    0.0%    0.0%    0.0%    0.0%    0.0%    0.0%    0.0%    0.0%    0.0%    0.0%    0.0%    0.0%    0.0%    0.0%    0.0%    0.0%    0.0%    0.0%    0.0%    0.0%    0.0%    0.0%    0.0%    0.0%    0.0%    0.0%    0.0%    0.0%    0.0%    0.0%    0.0%    0.0%    0.0%    0.0%    0.0%    0.0%    0.0%    0.0%    0.0%    0.0%    0.0%    0.0%    0.0%    0.0%    0.0%    0.0%    0.0%    0.0%    0.0%    0.0%    0.0%    0.0%    0.0%    0.0%    0.0%    0.0%    0.0%    0.0%    0.0%    0.0%    0.0%    0.0%    0.0%    0.0%    0.0%    0.0%    0.0%    0.0%    0.0%    0.0%    0.0%    0.0%    0.0%    0.0%    0.0%    0.0%    0.0%    0.0%    0.0%    0.0%    0.0%    0.0%    0.0%    0.0%    0.0%    0.0%    0.0%    0.0%    0.0%    0.0%    0.0%    0.0%    0.0%    0.0%    0.0%    0.0%    0.0%    0.0%    0.0%    0.0%    0.0%    0.0%    0.0%    0.0%    0.0%    0.0%    0.0%    0.0%    0.0%    0.0%    0.0%    0.0%    0.0%    0.0%    0.0%    0.0%    0.0%    0.0%    0.0%    0.0%    0.0%    0.0%    0.0%    0.0%    0.0%    0.0%    0.0%    0.0%    0.0%    0.0%    0.0%    0.0%    0.0%    0.0%    0.0%    0.0%    0.0%    0.0%    0.0%    0.0%    0.0%    0.0%    0.0%    0.0%    0.0%    0.0%    0.0%    0.0%    0.0%    0.0%    0.0%    0.0%    0.0%        k__Archaea;p__Euryarchaeota;c__Methanomicrobia; o__Methanomicrobiales       5    0.0%    0.0%    0.0%    0.0%    0.0%    0.0%    0.0%    0.0%    0.0%    0.0%    0.0%    0.0%    0.0%    0.0%    0.0%    0.0%    0.0%    0.0%    0.0%    0.0%    0.0%    0.0%    0.0%    0.0%    0.0%    0.0%    0.0%    0.0%    0.0%    0.0%    0.0%    0.0%    0.0%    0.0%    0.0%    0.0%    0.0%    0.0%    0.0%    0.0%    0.0%    0.0%    0.0%    0.0%    0.0%    0.0%    0.0%    0.0%    0.0%    0.0%    0.0%    0.0%    0.0%    0.0%    0.0%    0.0%    0.0%    0.0%    0.0%    0.0%    0.0%    0.0%    0.0%    0.0%    0.0%    0.0%    0.0%    0.0%    0.0%    0.0%    0.0%    0.0%    0.0%    0.0%    0.0%    0.0%    0.0%    0.0%    0.0%    0.0%    0.0%    0.0%    0.0%    0.0%    0.0%    0.0%    0.0%    0.0%    0.0%    0.0%    0.0%    0.0%    0.0%    0.0%    0.0%    0.0%    0.0%    0.0%    0.0%    0.0%    0.0%    0.0%    0.0%    0.0%    0.0%    0.0%    0.0%    0.0%    0.0%    0.0%    0.0%    0.0%    0.0%    0.0%    0.0%    0.0%    0.0%    0.0%    0.0%    0.0%    0.0%    0.0%    0.0%    0.0%    0.0%    0.0%    0.0%    0.0%    0.0%    0.0%    0.0%    0.0%    0.0%    0.0%    0.0%    0.0%    0.0%    0.0%    0.0%    0.0%    0.0%    0.0%    0.0%    0.0%    0.0%    0.0%    0.0%    0.0%    0.0%    0.0%    0.0%    0.0%    0.0%    0.0%    0.0%    0.0%    0.0%    0.0%    0.0%    0.0%    0.0%    0.0%    0.0%    0.0%    0.0%    0.0%    0.0%    0.0%    0.0%    0.0%    0.0%    0.0%    0.0%    0.0%    0.0%    0.0%    0.0%    0.0%    0.0%    0.0%    0.0%    0.0%    0.0%    0.0%    0.0%    0.0%    0.0%    0.0%    0.0%    0.0%    0.0%    0.0%    0.0%    0.0%    0.0%    0.0%    0.0%    0.0%    0.0%    0.0%    0.0%    0.0%    0.0%    0.0%    0.0%    0.0%    0.0%    0.0%    0.0%    0.0%    0.0%    0.0%    0.0%    0.0%    0.0%    0.0%    0.0%    0.0%    0.0%    0.0%    0.0%    0.0%    0.0%    0.0%    0.0%    0.0%    0.0%    0.0%    0.0%    0.0%    0.0%    0.0%    0.0%    0.0%    0.0%    0.0%    0.0%    0.0%    0.0%    0.0%    0.0%    0.0%    0.0%    0.0%    0.0%    0.0%    0.0%    0.0%    0.0%    0.0%    0.0%    0.0%    0.0%    0.0%    0.0%    0.0%    0.0%    0.0%    0.0%    0.0%    0.0%    0.0%    0.0%    0.0%    0.0%    0.0%    0.0%    0.0%    0.0%    0.0%    0.0%    0.0%    0.0%    0.0%    0.0%    0.0%    0.0%    0.0%    0.0%    0.0%    0.0%    0.0%    0.0%    0.0%    0.0%    0.0%    0.0%    0.0%    0.0%    0.0%    0.0%    0.0%    0.0%    0.0%    0.0%    0.0%    0.0%    0.0%    0.0%    0.0%    0.0%    0.0%    0.0%    0.0%    0.0%    0.0%    0.0%    0.0%    0.0%    0.0%    0.0%    0.0%    0.0%    0.0%    0.0%    0.0%    0.0%    0.0%    0.0%    0.0%    0.0%    0.0%    0.0%    0.0%    0.0%    0.0%    0.0%    0.0%    0.0%    0.0%    0.0%    0.0%    0.0%    0.0%    0.0%    0.0%    0.0%    0.0%    0.0%    0.0%    0.0%    0.0%    0.0%    0.0%    0.0%    0.0%    0.0%    0.0%    0.0%    0.0%    0.0%    0.0%    0.0%    0.0%    0.0%    0.0%    0.0%    0.0%        k__Archaea;p__Euryarchaeota;c__Methanomicrobia; o__Methanosarcinales       8    0.0%    0.0%    0.0%    0.0%    0.0%    0.0%    0.0%    0.0%    0.0%    0.0%    0.0%    0.0%    0.0%    0.0%    0.0%    0.0%    0.0%    0.0%    0.0%    0.0%    0.0%    0.0%    0.0%    0.0%    0.0%    0.0%    0.0%    0.0%    0.0%    0.0%    0.0%    0.0%    0.0%    0.0%    0.0%    0.0%    0.0%    0.0%    0.0%    0.0%    0.0%    0.0%    0.0%    0.0%    0.0%    0.0%    0.0%    0.0%    0.0%    0.0%    0.0%    0.0%    0.0%    0.0%    0.0%    0.0%    0.0%    0.0%    0.0%    0.0%    0.0%    0.0%    0.0%    0.0%    0.0%    0.0%    0.0%    0.0%    0.0%    0.0%    0.0%    0.0%    0.0%    0.0%    0.0%    0.0%    0.0%    0.0%    0.0%    0.0%    0.0%    0.0%    0.0%    0.0%    0.0%    0.0%    0.0%    0.0%    0.0%    0.0%    0.0%    0.0%    0.0%    0.0%    0.0%    0.0%    0.0%    0.0%    0.0%    0.0%    0.0%    0.0%    0.0%    0.0%    0.0%    0.0%    0.0%    0.0%    0.0%    0.0%    0.0%    0.0%    0.0%    0.0%    0.0%    0.0%    0.0%    0.0%    0.0%    0.0%    0.0%    0.0%    0.0%    0.0%    0.0%    0.0%    0.0%    0.0%    0.0%    0.0%    0.0%    0.0%    0.0%    0.0%    0.0%    0.0%    0.0%    0.0%    0.0%    0.0%    0.0%    0.0%    0.0%    0.0%    0.0%    0.0%    0.0%    0.0%    0.0%    0.0%    0.0%    0.0%    0.0%    0.0%    0.0%    0.0%    0.0%    0.0%    0.0%    0.0%    0.0%    0.0%    0.0%    0.0%    0.0%    0.0%    0.0%    0.0%    0.0%    0.0%    0.0%    0.0%    0.0%    0.0%    0.0%    0.0%    0.0%    0.0%    0.0%    0.0%    0.0%    0.0%    0.0%    0.0%    0.0%    0.0%    0.0%    0.0%    0.0%    0.0%    0.0%    0.0%    0.0%    0.0%    0.0%    0.0%    0.0%    0.0%    0.0%    0.0%    0.0%    0.0%    0.0%    0.0%    0.0%    0.0%    0.0%    0.0%    0.0%    0.0%    0.0%    0.0%    0.0%    0.0%    0.0%    0.0%    0.0%    0.0%    0.0%    0.0%    0.0%    0.0%    0.0%    0.0%    0.0%    0.0%    0.0%    0.0%    0.0%    0.0%    0.0%    0.0%    0.0%    0.0%    0.0%    0.0%    0.0%    0.0%    0.0%    0.0%    0.0%    0.0%    0.0%    0.0%    0.0%    0.0%    0.0%    0.0%    0.0%    0.0%    0.0%    0.0%    0.0%    0.0%    0.0%    0.0%    0.0%    0.0%    0.0%    0.0%    0.0%    0.0%    0.0%    0.0%    0.0%    0.0%    0.0%    0.0%    0.0%    0.0%    0.0%    0.0%    0.0%    0.0%    0.0%    0.0%    0.0%    0.0%    0.0%    0.0%    0.0%    0.0%    0.0%    0.0%    0.0%    0.0%    0.0%    0.0%    0.0%    0.0%    0.0%    0.0%    0.0%    0.0%    0.0%    0.0%    0.0%    0.0%    0.0%    0.0%    0.0%    0.0%    0.0%    0.0%    0.0%    0.0%    0.0%    0.0%    0.0%    0.0%    0.0%    0.0%    0.0%    0.0%    0.0%    0.0%    0.0%    0.0%    0.0%    0.0%    0.0%    0.0%    0.0%    0.0%    0.0%    0.0%    0.0%    0.0%    0.0%    0.0%    0.0%    0.0%    0.0%    0.0%    0.0%    0.0%    0.0%    0.0%    0.0%    0.0%    0.0%    0.0%    0.0%    0.0%    0.0%    0.0%    0.0%    0.0%    0.0%    0.0%    0.0%    0.0%    0.0%    0.0%    0.0%    0.0%    0.0%    0.0%        k__Archaea;p__Euryarchaeota;c__Thermoplasmata; o__E2       6    0.0%    0.0%    0.0%    0.0%    0.0%    0.0%    0.0%    0.0%    0.0%    0.0%    0.0%    0.0%    0.0%    0.0%    0.0%    0.0%    0.0%    0.0%    0.0%    0.0%    0.0%    0.0%    0.0%    0.0%    0.0%    0.0%    0.0%    0.0%    0.0%    0.0%    0.0%    0.0%    0.0%    0.0%    0.0%    0.0%    0.0%    0.0%    0.0%    0.0%    0.0%    0.0%    0.0%    0.0%    0.0%    0.0%    0.0%    0.0%    0.0%    0.0%    0.0%    0.0%    0.0%    0.0%    0.0%    0.0%    0.0%    0.0%    0.0%    0.0%    0.0%    0.0%    0.0%    0.0%    0.0%    0.0%    0.0%    0.0%    0.0%    0.0%    0.0%    0.0%    0.0%    0.0%    0.0%    0.0%    0.0%    0.0%    0.0%    0.0%    0.0%    0.0%    0.0%    0.0%    0.0%    0.0%    0.0%    0.0%    0.0%    0.0%    0.0%    0.0%    0.0%    0.0%    0.0%    0.0%    0.0%    0.0%    0.0%    0.0%    0.0%    0.0%    0.0%    0.0%    0.0%    0.0%    0.0%    0.0%    0.0%    0.0%    0.0%    0.0%    0.0%    0.0%    0.0%    0.0%    0.0%    0.0%    0.0%    0.0%    0.0%    0.0%    0.0%    0.0%    0.0%    0.0%    0.0%    0.0%    0.0%    0.0%    0.0%    0.0%    0.0%    0.0%    0.0%    0.0%    0.0%    0.0%    0.0%    0.0%    0.0%    0.0%    0.0%    0.0%    0.0%    0.0%    0.0%    0.0%    0.0%    0.0%    0.0%    0.0%    0.0%    0.0%    0.0%    0.0%    0.0%    0.0%    0.0%    0.0%    0.0%    0.0%    0.0%    0.0%    0.0%    0.0%    0.0%    0.0%    0.0%    0.0%    0.0%    0.0%    0.0%    0.0%    0.0%    0.0%    0.0%    0.0%    0.0%    0.0%    0.0%    0.0%    0.0%    0.0%    0.0%    0.0%    0.0%    0.0%    0.0%    0.0%    0.0%    0.0%    0.0%    0.0%    0.0%    0.0%    0.0%    0.0%    0.0%    0.0%    0.0%    0.0%    0.0%    0.0%    0.0%    0.0%    0.0%    0.0%    0.0%    0.0%    0.0%    0.0%    0.0%    0.0%    0.0%    0.0%    0.0%    0.0%    0.0%    0.0%    0.0%    0.0%    0.0%    0.0%    0.0%    0.0%    0.0%    0.0%    0.0%    0.0%    0.0%    0.0%    0.0%    0.0%    0.0%    0.0%    0.0%    0.0%    0.0%    0.0%    0.0%    0.0%    0.0%    0.0%    0.0%    0.0%    0.0%    0.0%    0.0%    0.0%    0.0%    0.0%    0.0%    0.0%    0.0%    0.0%    0.0%    0.0%    0.0%    0.0%    0.0%    0.0%    0.0%    0.0%    0.0%    0.0%    0.0%    0.0%    0.0%    0.0%    0.0%    0.0%    0.0%    0.0%    0.0%    0.0%    0.0%    0.0%    0.0%    0.0%    0.0%    0.0%    0.0%    0.0%    0.0%    0.0%    0.0%    0.0%    0.0%    0.0%    0.0%    0.0%    0.0%    0.0%    0.0%    0.0%    0.0%    0.0%    0.0%    0.0%    0.0%    0.0%    0.0%    0.0%    0.0%    0.0%    0.0%    0.0%    0.0%    0.0%    0.0%    0.2%    0.0%    0.0%    0.0%    0.0%    0.0%    0.0%    0.0%    0.0%    0.0%    0.0%    0.0%    0.0%    0.0%    0.0%    0.0%    0.0%    0.0%    0.0%    0.0%    0.0%    0.0%    0.0%    0.0%    0.0%    0.0%    0.0%    0.0%    0.0%    0.0%    0.0%    0.0%    0.0%    0.0%    0.0%    0.0%    0.0%    0.0%    0.0%    0.0%    0.0%    0.0%    0.0%    0.0%    0.0%    0.0%    0.0%        k__Bacteria;p__;c__; o__       6    0.0%    0.0%    0.0%    0.0%    0.0%    0.0%    0.0%    0.0%    0.0%    0.0%    0.0%    0.0%    0.0%    0.0%    0.0%    0.0%    0.0%    0.0%    0.0%    0.0%    0.0%    0.0%    0.0%    0.0%    0.0%    0.0%    0.0%    0.0%    0.0%    0.0%    0.0%    0.0%    0.0%    0.0%    0.0%    0.0%    0.0%    0.0%    0.0%    0.0%    0.0%    0.0%    0.0%    0.0%    0.0%    0.0%    0.0%    0.0%    0.0%    0.0%    0.0%    0.0%    0.0%    0.0%    0.0%    0.0%    0.0%    0.0%    0.0%    0.0%    0.0%    0.0%    0.0%    0.0%    0.0%    0.0%    0.0%    0.0%    0.0%    0.0%    0.0%    0.0%    0.0%    0.0%    0.0%    0.0%    0.0%    0.0%    0.0%    0.0%    0.0%    0.0%    0.0%    0.0%    0.0%    0.0%    0.0%    0.0%    0.0%    0.0%    0.0%    0.0%    0.0%    0.0%    0.0%    0.0%    0.0%    0.0%    0.0%    0.0%    0.0%    0.0%    0.0%    0.0%    0.0%    0.0%    0.0%    0.0%    0.0%    0.0%    0.0%    0.0%    0.0%    0.0%    0.0%    0.0%    0.0%    0.0%    0.0%    0.0%    0.0%    0.0%    0.0%    0.0%    0.0%    0.0%    0.0%    0.0%    0.0%    0.0%    0.0%    0.0%    0.0%    0.0%    0.0%    0.0%    0.0%    0.0%    0.0%    0.0%    0.0%    0.0%    0.0%    0.0%    0.0%    0.0%    0.0%    0.0%    0.0%    0.0%    0.0%    0.0%    0.0%    0.0%    0.0%    0.0%    0.0%    0.0%    0.0%    0.0%    0.0%    0.0%    0.0%    0.0%    0.0%    0.0%    0.0%    0.0%    0.0%    0.0%    0.0%    0.0%    0.0%    0.0%    0.0%    0.0%    0.0%    0.0%    0.0%    0.0%    0.0%    0.0%    0.0%    0.0%    0.0%    0.0%    0.0%    0.0%    0.0%    0.0%    0.0%    0.0%    0.0%    0.0%    0.0%    0.0%    0.0%    0.0%    0.0%    0.0%    0.0%    0.0%    0.0%    0.0%    0.0%    0.0%    0.0%    0.0%    0.0%    0.0%    0.0%    0.0%    0.0%    0.0%    0.0%    0.0%    0.0%    0.0%    0.0%    0.0%    0.0%    0.0%    0.0%    0.0%    0.0%    0.0%    0.0%    0.0%    0.0%    0.0%    0.0%    0.0%    0.0%    0.0%    0.0%    0.0%    0.0%    0.0%    0.0%    0.0%    0.0%    0.0%    0.0%    0.0%    0.0%    0.0%    0.0%    0.0%    0.0%    0.0%    0.0%    0.0%    0.0%    0.0%    0.0%    0.0%    0.0%    0.0%    0.0%    0.0%    0.0%    0.0%    0.0%    0.0%    0.0%    0.0%    0.0%    0.0%    0.0%    0.0%    0.0%    0.0%    0.0%    0.0%    0.0%    0.0%    0.0%    0.0%    0.0%    0.0%    0.0%    0.0%    0.0%    0.0%    0.0%    0.0%    0.0%    0.0%    0.0%    0.0%    0.0%    0.0%    0.0%    0.0%    0.0%    0.0%    0.0%    0.0%    0.0%    0.0%    0.0%    0.0%    0.0%    0.0%    0.0%    0.0%    0.0%    0.0%    0.0%    0.0%    0.0%    0.0%    0.0%    0.0%    0.0%    0.0%    0.0%    0.0%    0.0%    0.0%    0.0%    0.0%    0.0%    0.0%    0.0%    0.0%    0.0%    0.0%    0.0%    0.0%    0.0%    0.0%    0.0%    0.0%    0.0%    0.0%    0.0%    0.0%    0.0%    0.0%    0.0%    0.0%    0.0%    0.0%    0.0%    0.0%    0.0%    0.0%    0.0%    0.0%    0.0%    0.0%    0.0%    0.0%    0.0%    0.0%    0.0%    0.0%        k__Bacteria;p__ABY1_OD1;c__; o__       0    0.0%    0.0%    0.0%    0.0%    0.0%    0.0%    0.0%    0.0%    0.0%    0.0%    0.0%    0.0%    0.0%    0.0%    0.0%    0.0%    0.0%    0.0%    0.0%    0.0%    0.0%    0.0%    0.0%    0.0%    0.0%    0.0%    0.0%    0.0%    0.0%    0.0%    0.0%    0.0%    0.0%    0.0%    0.0%    0.0%    0.0%    0.0%    0.0%    0.0%    0.0%    0.0%    0.0%    0.0%    0.0%    0.0%    0.0%    0.0%    0.0%    0.0%    0.0%    0.0%    0.0%    0.0%    0.0%    0.0%    0.0%    0.0%    0.0%    0.0%    0.0%    0.0%    0.0%    0.0%    0.0%    0.0%    0.0%    0.0%    0.0%    0.0%    0.0%    0.0%    0.0%    0.0%    0.0%    0.0%    0.0%    0.0%    0.0%    0.0%    0.0%    0.0%    0.0%    0.0%    0.0%    0.0%    0.0%    0.0%    0.0%    0.0%    0.0%    0.0%    0.0%    0.0%    0.0%    0.0%    0.0%    0.0%    0.0%    0.0%    0.0%    0.0%    0.0%    0.0%    0.0%    0.0%    0.0%    0.0%    0.0%    0.0%    0.0%    0.0%    0.0%    0.0%    0.0%    0.0%    0.0%    0.0%    0.0%    0.0%    0.0%    0.0%    0.0%    0.0%    0.0%    0.0%    0.0%    0.0%    0.0%    0.0%    0.0%    0.0%    0.0%    0.0%    0.0%    0.0%    0.0%    0.0%    0.0%    0.0%    0.0%    0.0%    0.0%    0.0%    0.0%    0.0%    0.0%    0.0%    0.0%    0.0%    0.0%    0.0%    0.0%    0.0%    0.0%    0.0%    0.0%    0.0%    0.0%    0.0%    0.0%    0.0%    0.0%    0.0%    0.0%    0.0%    0.0%    0.0%    0.0%    0.0%    0.0%    0.0%    0.0%    0.0%    0.0%    0.0%    0.0%    0.0%    0.0%    0.0%    0.0%    0.0%    0.0%    0.0%    0.0%    0.0%    0.0%    0.0%    0.0%    0.0%    0.0%    0.0%    0.0%    0.0%    0.0%    0.0%    0.0%    0.0%    0.0%    0.0%    0.0%    0.0%    0.0%    0.0%    0.0%    0.0%    0.0%    0.0%    0.0%    0.0%    0.0%    0.0%    0.0%    0.0%    0.0%    0.0%    0.0%    0.0%    0.0%    0.0%    0.0%    0.0%    0.0%    0.0%    0.0%    0.0%    0.0%    0.0%    0.0%    0.0%    0.0%    0.0%    0.0%    0.0%    0.0%    0.0%    0.0%    0.0%    0.0%    0.0%    0.0%    0.0%    0.0%    0.0%    0.0%    0.0%    0.0%    0.0%    0.0%    0.0%    0.0%    0.0%    0.0%    0.0%    0.0%    0.0%    0.0%    0.0%    0.0%    0.0%    0.0%    0.0%    0.0%    0.0%    0.0%    0.0%    0.0%    0.0%    0.0%    0.0%    0.0%    0.0%    0.0%    0.0%    0.0%    0.0%    0.0%    0.0%    0.0%    0.0%    0.0%    0.0%    0.0%    0.0%    0.0%    0.0%    0.0%    0.0%    0.0%    0.0%    0.0%    0.0%    0.0%    0.0%    0.0%    0.0%    0.0%    0.0%    0.0%    0.0%    0.0%    0.0%    0.0%    0.0%    0.0%    0.0%    0.0%    0.0%    0.0%    0.0%    0.0%    0.0%    0.0%    0.0%    0.0%    0.0%    0.0%    0.0%    0.0%    0.0%    0.0%    0.0%    0.0%    0.0%    0.0%    0.0%    0.0%    0.0%    0.0%    0.0%    0.0%    0.0%    0.0%    0.0%    0.0%    0.0%    0.0%    0.0%    0.0%    0.0%    0.0%    0.0%    0.0%    0.0%    0.0%    0.0%    0.0%    0.0%    0.0%    0.0%    0.0%    0.0%    0.0%    0.0%    0.0%    0.0%    0.0%    0.0%        k__Bacteria;p__AD3;c__ABS-6; o__      19    0.0%    0.0%    0.0%    0.0%    0.0%    0.0%    0.0%    0.0%    0.0%    0.0%    0.0%    0.0%    0.0%    0.0%    0.0%    0.0%    0.0%    0.0%    0.0%    0.0%    0.0%    0.0%    0.0%    0.0%    0.0%    0.0%    0.0%    0.0%    0.0%    0.0%    0.0%    0.0%    0.0%    0.0%    0.0%    0.0%    0.0%    0.0%    0.0%    0.0%    0.0%    0.0%    0.0%    0.0%    0.0%    0.0%    0.0%    0.0%    0.0%    0.0%    0.0%    0.0%    0.0%    0.0%    0.0%    0.0%    0.0%    0.0%    0.0%    0.0%    0.0%    0.0%    0.0%    0.0%    0.0%    0.0%    0.0%    0.0%    0.0%    0.0%    0.0%    0.0%    0.0%    0.0%    0.0%    0.0%    0.0%    0.0%    0.0%    0.0%    0.0%    0.0%    0.0%    0.0%    0.0%    0.0%    0.0%    0.0%    0.0%    0.0%    0.0%    0.0%    0.0%    0.0%    0.0%    0.0%    0.0%    0.0%    0.0%    0.0%    0.0%    0.0%    0.0%    0.0%    0.0%    0.0%    0.0%    0.0%    0.0%    0.0%    0.0%    0.0%    0.0%    0.0%    0.0%    0.0%    0.0%    0.0%    0.0%    0.0%    0.0%    0.0%    0.0%    0.0%    0.0%    0.0%    0.0%    0.0%    0.0%    0.0%    0.0%    0.0%    0.0%    0.0%    0.0%    0.0%    0.0%    0.0%    0.0%    0.0%    0.0%    0.0%    0.0%    0.0%    0.0%    0.0%    0.0%    0.0%    0.0%    0.0%    0.0%    0.0%    0.0%    0.0%    0.0%    0.0%    0.0%    0.0%    0.0%    0.0%    0.0%    0.0%    0.0%    0.0%    0.0%    0.0%    0.0%    0.0%    0.0%    0.0%    0.0%    0.0%    0.0%    0.0%    0.0%    0.0%    0.0%    0.0%    0.0%    0.0%    0.0%    0.0%    0.0%    0.0%    0.0%    0.0%    0.0%    0.0%    0.0%    0.0%    0.0%    0.0%    0.0%    0.0%    0.0%    0.0%    0.0%    0.0%    0.0%    0.0%    0.0%    0.0%    0.0%    0.0%    0.0%    0.0%    0.0%    0.0%    0.0%    0.0%    0.0%    0.0%    0.0%    0.0%    0.0%    0.0%    0.0%    0.0%    0.0%    0.0%    0.0%    0.0%    0.0%    0.0%    0.0%    0.0%    0.0%    0.0%    0.0%    0.0%    0.0%    0.0%    0.0%    0.0%    0.0%    0.0%    0.0%    0.0%    0.0%    0.0%    0.0%    0.0%    0.0%    0.0%    0.0%    0.0%    0.0%    0.0%    0.0%    0.0%    0.0%    0.0%    0.0%    0.0%    0.0%    0.0%    0.0%    0.0%    0.0%    0.0%    0.0%    0.0%    0.0%    0.0%    0.0%    0.0%    0.0%    0.0%    0.0%    0.0%    0.0%    0.0%    0.0%    0.0%    0.0%    0.0%    0.0%    0.0%    0.0%    0.0%    0.0%    0.0%    0.0%    0.0%    0.0%    0.0%    0.0%    0.0%    0.0%    0.0%    0.0%    0.0%    0.0%    0.0%    0.0%    0.0%    0.0%    0.0%    0.0%    0.0%    0.0%    0.0%    0.0%    0.0%    0.0%    0.0%    0.0%    0.0%    0.0%    0.0%    0.0%    0.0%    0.0%    0.0%    0.0%    0.0%    0.0%    0.0%    0.0%    0.0%    0.0%    0.0%    0.0%    0.0%    0.0%    0.0%    0.0%    0.0%    0.0%    0.0%    0.0%    0.0%    0.0%    0.0%    0.0%    0.0%    0.0%    0.0%    0.0%    0.0%    0.0%    0.0%    0.0%    0.0%    0.0%    0.0%    0.0%    0.0%    0.0%    0.0%    0.0%    0.0%    0.0%    0.0%    0.0%    0.0%    0.0%    0.0%        k__Bacteria;p__AD3;c__JG37-AG-4; o__       8    0.0%    0.0%    0.0%    0.0%    0.0%    0.0%    0.0%    0.0%    0.0%    0.0%    0.0%    0.0%    0.0%    0.0%    0.0%    0.0%    0.0%    0.0%    0.0%    0.0%    0.0%    0.0%    0.0%    0.0%    0.0%    0.0%    0.0%    0.0%    0.0%    0.0%    0.0%    0.0%    0.0%    0.0%    0.0%    0.0%    0.0%    0.0%    0.0%    0.0%    0.0%    0.0%    0.0%    0.0%    0.0%    0.0%    0.0%    0.0%    0.0%    0.0%    0.0%    0.0%    0.0%    0.0%    0.0%    0.0%    0.0%    0.0%    0.0%    0.0%    0.0%    0.0%    0.0%    0.0%    0.0%    0.0%    0.0%    0.0%    0.0%    0.0%    0.0%    0.0%    0.0%    0.0%    0.0%    0.0%    0.0%    0.0%    0.0%    0.0%    0.0%    0.0%    0.0%    0.0%    0.0%    0.0%    0.0%    0.0%    0.0%    0.0%    0.0%    0.0%    0.0%    0.0%    0.0%    0.0%    0.0%    0.0%    0.0%    0.0%    0.0%    0.0%    0.0%    0.0%    0.0%    0.0%    0.0%    0.0%    0.0%    0.0%    0.0%    0.0%    0.0%    0.0%    0.0%    0.0%    0.0%    0.0%    0.0%    0.0%    0.0%    0.0%    0.0%    0.0%    0.0%    0.0%    0.0%    0.0%    0.0%    0.0%    0.0%    0.0%    0.0%    0.0%    0.0%    0.0%    0.0%    0.0%    0.0%    0.0%    0.0%    0.0%    0.0%    0.0%    0.0%    0.0%    0.0%    0.0%    0.0%    0.0%    0.0%    0.0%    0.0%    0.0%    0.0%    0.0%    0.0%    0.0%    0.0%    0.0%    0.0%    0.0%    0.0%    0.0%    0.0%    0.0%    0.0%    0.0%    0.0%    0.0%    0.0%    0.0%    0.0%    0.0%    0.0%    0.0%    0.0%    0.0%    0.0%    0.0%    0.0%    0.0%    0.0%    0.0%    0.0%    0.0%    0.0%    0.0%    0.0%    0.0%    0.0%    0.0%    0.0%    0.0%    0.0%    0.0%    0.0%    0.0%    0.0%    0.0%    0.0%    0.0%    0.0%    0.0%    0.0%    0.0%    0.0%    0.0%    0.0%    0.0%    0.0%    0.0%    0.0%    0.0%    0.0%    0.0%    0.0%    0.0%    0.0%    0.0%    0.0%    0.0%    0.0%    0.0%    0.0%    0.0%    0.0%    0.0%    0.0%    0.0%    0.0%    0.0%    0.0%    0.0%    0.0%    0.0%    0.0%    0.0%    0.0%    0.0%    0.0%    0.0%    0.0%    0.0%    0.0%    0.0%    0.0%    0.0%    0.0%    0.0%    0.0%    0.0%    0.0%    0.0%    0.0%    0.0%    0.0%    0.0%    0.0%    0.0%    0.0%    0.0%    0.0%    0.0%    0.0%    0.0%    0.0%    0.0%    0.0%    0.0%    0.0%    0.0%    0.0%    0.0%    0.0%    0.0%    0.0%    0.0%    0.0%    0.0%    0.0%    0.0%    0.0%    0.0%    0.0%    0.0%    0.0%    0.0%    0.0%    0.0%    0.0%    0.0%    0.0%    0.0%    0.0%    0.0%    0.0%    0.0%    0.0%    0.0%    0.0%    0.0%    0.0%    0.0%    0.0%    0.0%    0.0%    0.0%    0.0%    0.0%    0.0%    0.0%    0.0%    0.0%    0.0%    0.0%    0.0%    0.0%    0.0%    0.0%    0.0%    0.0%    0.0%    0.0%    0.0%    0.0%    0.0%    0.0%    0.0%    0.0%    0.0%    0.0%    0.0%    0.0%    0.0%    0.0%    0.0%    0.0%    0.0%    0.0%    0.0%    0.0%    0.0%    0.0%    0.0%    0.0%    0.0%    0.0%    0.0%    0.0%    0.0%    0.0%    0.0%    0.0%    0.0%    0.0%    0.0%    0.0%        k__Bacteria;p__Acidobacteria;c__; o__     430    0.0%    0.0%    0.0%    0.0%    0.0%    0.0%    0.0%    0.0%    0.0%    0.0%    0.0%    0.0%    0.0%    0.0%    0.0%    0.0%    0.0%    0.0%    0.0%    0.0%    0.0%    0.0%    0.0%    0.0%    0.0%    0.0%    0.0%    0.0%    0.0%    0.0%    0.0%    0.0%    0.0%    0.0%    0.0%    0.0%    0.0%    0.0%    0.0%    0.0%    0.0%    0.0%    0.0%    0.0%    0.0%    0.0%    0.0%    0.0%    0.0%    0.0%    0.0%    0.0%    0.0%    0.0%    0.0%    0.0%    0.0%    0.0%    0.0%    0.0%    0.0%    0.0%    0.0%    0.0%    0.0%    0.0%    0.0%    0.0%    0.0%    0.0%    0.0%    0.0%    0.0%    0.0%    0.0%    0.0%    0.0%    0.0%    0.0%    0.0%    0.0%    0.0%    0.0%    0.0%    0.0%    0.0%    0.0%    0.0%    0.0%    0.0%    0.0%    0.0%    0.0%    0.0%    0.0%    0.0%    0.0%    0.0%    0.0%    0.0%    0.0%    0.0%    0.0%    0.0%    0.0%    0.0%    0.0%    0.0%    0.0%    0.0%    0.0%    0.0%    0.0%    0.0%    0.0%    0.0%    0.0%    0.0%    0.0%    0.0%    0.0%    0.0%    0.0%    0.0%    0.0%    0.0%    0.0%    0.0%    0.0%    0.0%    0.0%    0.0%    0.0%    0.0%    0.0%    0.0%    0.0%    0.0%    0.0%    0.0%    0.0%    0.0%    0.0%    0.0%    0.0%    0.0%    0.0%    0.0%    0.0%    0.0%    0.0%    0.0%    0.0%    0.0%    0.0%    0.0%    0.0%    0.0%    0.0%    0.0%    0.0%    0.0%    0.0%    0.0%    0.0%    0.0%    0.0%    0.0%    0.0%    0.0%    0.0%    0.0%    0.0%    0.0%    0.0%    0.0%    0.0%    0.0%    0.0%    0.0%    0.0%    0.0%    0.0%    0.0%    0.0%    0.0%    0.0%    0.0%    0.0%    0.0%    0.0%    0.0%    0.0%    0.0%    0.0%    0.0%    0.0%    0.0%    0.0%    0.0%    0.0%    0.0%    0.0%    0.0%    0.0%    0.0%    0.0%    0.0%    0.0%    0.0%    0.0%    0.0%    0.0%    0.0%    0.0%    0.0%    0.0%    0.0%    0.0%    0.0%    0.0%    0.0%    0.0%    0.0%    0.0%    0.0%    0.0%    0.0%    0.0%    0.0%    0.0%    0.0%    0.0%    0.0%    0.0%    0.0%    0.0%    0.0%    0.0%    0.0%    0.0%    0.0%    0.0%    0.0%    0.0%    0.0%    0.0%    0.0%    0.0%    0.0%    0.0%    0.0%    0.0%    0.0%    0.0%    0.0%    0.0%    0.0%    0.0%    0.0%    0.0%    0.0%    0.0%    0.0%    0.0%    0.0%    0.0%    0.0%    0.0%    0.0%    0.0%    0.0%    0.0%    0.0%    0.0%    0.0%    0.0%    0.0%    0.0%    0.0%    0.0%    0.0%    0.0%    0.0%    0.0%    0.0%    0.0%    0.0%    0.0%    0.0%    0.0%    0.0%    0.0%    0.0%    0.0%    0.0%    0.0%    0.0%    0.0%    0.0%    0.0%    0.0%    0.0%    0.0%    0.0%    0.0%    0.0%    0.0%    0.0%    0.0%    0.0%    0.0%    0.0%    0.0%    0.0%    0.0%    0.0%    0.0%    0.0%    0.0%    0.0%    0.0%    0.0%    0.0%    0.0%    0.0%    0.0%    0.0%    0.0%    0.0%    0.0%    0.0%    0.0%    0.0%    0.0%    0.0%    0.0%    0.0%    0.0%    0.0%    0.0%    0.0%    0.0%    0.0%    0.0%    0.0%    0.0%    0.0%    0.0%    0.0%    0.0%    0.0%    0.0%    0.0%    0.0%    0.0%    0.0%    0.0%        k__Bacteria;p__Acidobacteria;c__Acidobacteria (class); o__Acidobacteriales   17419    0.1%    0.0%    0.1%    0.0%    0.0%    0.0%    0.0%    0.0%    0.0%    0.0%    0.0%    0.0%    0.1%    0.0%    0.0%    0.0%    0.1%    0.1%    0.0%    0.1%    0.1%    0.1%    0.0%    0.0%    0.0%    0.0%    0.1%    0.1%    0.1%    0.0%    0.0%    0.1%    0.0%    0.0%    0.2%    0.0%    0.1%    0.0%    0.1%    0.2%    0.1%    0.2%    0.0%    0.0%    0.1%    0.0%    0.0%    0.0%    0.0%    0.0%    0.1%    0.1%    0.0%    0.0%    0.1%    0.1%    0.1%    0.0%    0.1%    0.3%    0.2%    0.1%    0.1%    0.4%    0.2%    0.2%    0.0%    0.4%    0.1%    0.2%    0.1%    0.0%    0.1%    0.1%    0.2%    0.0%    0.1%    0.1%    0.0%    0.1%    0.0%    0.0%    0.0%    0.1%    0.0%    0.0%    0.2%    0.0%    0.0%    0.2%    0.0%    0.1%    0.2%    0.0%    0.1%    0.1%    0.3%    0.0%    0.1%    0.2%    0.3%    0.1%    0.0%    0.1%    0.0%    0.0%    0.5%    0.0%    0.0%    0.2%    0.1%    0.3%    0.0%    0.0%    0.1%    0.0%    0.0%    0.1%    0.0%    0.7%    0.0%    0.1%    0.4%    0.1%    0.0%    0.3%    0.3%    0.0%    0.2%    0.7%    0.2%    0.1%    0.2%    0.1%    0.6%    0.1%    0.1%    0.1%    0.0%    0.1%    0.0%    0.1%    0.1%    0.1%    0.3%    0.1%    0.0%    0.0%    0.1%    0.0%    0.0%    0.1%    0.2%    0.1%    0.0%    0.0%    0.3%    0.0%    0.0%    0.3%    0.8%    0.2%    0.3%    0.1%    0.1%    0.1%    0.3%    0.1%    0.1%    0.1%    0.3%    0.2%    0.2%    0.1%    0.0%    0.0%    0.0%    0.0%    0.1%    0.0%    0.0%    0.0%    0.1%    0.1%    0.1%    0.0%    0.0%    0.0%    0.0%    0.1%    0.0%    0.0%    0.1%    0.1%    0.1%    0.1%    0.2%    0.0%    0.0%    0.0%    0.1%    0.0%    0.1%    0.3%    0.2%    0.1%    0.0%    0.2%    0.4%    0.2%    0.1%    0.0%    0.3%    0.1%    0.0%    0.2%    0.0%    0.0%    0.1%    0.0%    0.1%    0.2%    0.0%    0.2%    0.0%    0.1%    0.0%    0.0%    0.2%    0.1%    0.0%    0.0%    0.2%    0.0%    0.4%    0.0%    0.4%    0.1%    0.0%    0.1%    0.0%    0.3%    0.0%    0.0%    0.0%    0.0%    0.1%    0.1%    0.1%    0.0%    0.3%    0.2%    0.1%    0.0%    0.0%    0.0%    0.1%    0.0%    0.1%    0.1%    0.3%    0.2%    0.2%    0.1%    0.1%    0.0%    0.1%    0.2%    0.0%    0.1%    0.1%    0.2%    0.7%    0.1%    0.0%    0.1%    0.0%    0.1%    0.0%    0.0%    0.0%    0.0%    0.1%    0.0%    0.1%    0.0%    0.1%    1.1%    0.0%    0.0%    0.1%    0.0%    0.2%    0.1%    0.2%    0.0%    0.1%    0.1%    0.1%    0.2%    0.0%    0.1%    0.0%    0.3%    0.1%    0.0%    0.1%    0.1%    0.0%    0.0%    0.0%    0.0%    0.0%    0.0%    0.1%    0.1%    0.0%    0.0%    0.0%    0.0%    0.0%    0.0%    0.0%    0.1%    0.1%    0.1%    0.1%    0.0%    0.0%    0.1%    0.0%    0.0%    0.0%    0.2%    0.0%    0.0%    0.0%    0.1%    0.0%    0.0%    0.0%    0.0%    0.0%    0.1%    0.2%    0.0%    0.0%    0.1%    0.0%    0.0%    0.1%    0.1%    0.0%    0.2%    0.1%    0.0%    0.0%    0.0%        k__Bacteria;p__Acidobacteria;c__Acidobacteria-5; o__     248    0.0%    0.0%    0.0%    0.0%    0.0%    0.0%    0.0%    0.0%    0.0%    0.0%    0.0%    0.0%    0.0%    0.0%    0.0%    0.0%    0.0%    0.0%    0.0%    0.0%    0.0%    0.0%    0.0%    0.0%    0.0%    0.0%    0.0%    0.0%    0.0%    0.0%    0.0%    0.0%    0.0%    0.0%    0.0%    0.0%    0.0%    0.0%    0.0%    0.0%    0.0%    0.0%    0.0%    0.0%    0.0%    0.0%    0.0%    0.0%    0.0%    0.0%    0.0%    0.0%    0.0%    0.0%    0.0%    0.0%    0.0%    0.0%    0.0%    0.0%    0.0%    0.0%    0.0%    0.0%    0.0%    0.0%    0.0%    0.0%    0.0%    0.0%    0.0%    0.0%    0.0%    0.0%    0.0%    0.0%    0.0%    0.0%    0.0%    0.0%    0.0%    0.0%    0.0%    0.0%    0.0%    0.0%    0.0%    0.0%    0.0%    0.0%    0.0%    0.0%    0.0%    0.0%    0.0%    0.0%    0.0%    0.0%    0.0%    0.0%    0.0%    0.0%    0.0%    0.0%    0.0%    0.0%    0.0%    0.0%    0.0%    0.0%    0.0%    0.0%    0.0%    0.0%    0.0%    0.0%    0.0%    0.0%    0.0%    0.0%    0.0%    0.0%    0.0%    0.0%    0.0%    0.0%    0.0%    0.0%    0.0%    0.0%    0.0%    0.0%    0.0%    0.0%    0.0%    0.0%    0.0%    0.0%    0.0%    0.0%    0.0%    0.0%    0.0%    0.0%    0.0%    0.0%    0.0%    0.0%    0.0%    0.0%    0.0%    0.0%    0.0%    0.0%    0.0%    0.0%    0.0%    0.0%    0.0%    0.0%    0.0%    0.0%    0.0%    0.0%    0.0%    0.0%    0.0%    0.0%    0.0%    0.0%    0.0%    0.0%    0.0%    0.0%    0.0%    0.0%    0.0%    0.0%    0.0%    0.0%    0.0%    0.0%    0.0%    0.0%    0.0%    0.0%    0.0%    0.0%    0.0%    0.0%    0.0%    0.0%    0.0%    0.0%    0.0%    0.0%    0.0%    0.0%    0.0%    0.0%    0.0%    0.0%    0.0%    0.0%    0.0%    0.0%    0.0%    0.0%    0.0%    0.0%    0.0%    0.0%    0.0%    0.0%    0.0%    0.0%    0.0%    0.0%    0.0%    0.0%    0.0%    0.0%    0.0%    0.0%    0.0%    0.0%    0.0%    0.0%    0.0%    0.0%    0.0%    0.0%    0.0%    0.0%    0.0%    0.0%    0.0%    0.0%    0.0%    0.0%    0.0%    0.0%    0.0%    0.0%    0.0%    0.0%    0.0%    0.0%    0.0%    0.0%    0.0%    0.0%    0.0%    0.0%    0.0%    0.0%    0.0%    0.0%    0.0%    0.0%    0.0%    0.0%    0.0%    0.0%    0.0%    0.0%    0.0%    0.0%    0.0%    0.0%    0.0%    0.0%    0.0%    0.0%    0.0%    0.0%    0.0%    0.0%    0.0%    0.0%    0.0%    0.0%    0.0%    0.0%    0.0%    0.0%    0.0%    0.0%    0.0%    0.0%    0.0%    0.0%    0.0%    0.0%    0.0%    0.0%    0.0%    0.0%    0.0%    0.0%    0.0%    0.0%    0.0%    0.0%    0.0%    0.0%    0.0%    0.0%    0.0%    0.0%    0.0%    0.0%    0.0%    0.0%    0.0%    0.0%    0.0%    0.0%    0.0%    0.0%    0.0%    0.0%    0.0%    0.0%    0.0%    0.0%    0.0%    0.0%    0.0%    0.0%    0.0%    0.0%    0.0%    0.0%    0.0%    0.0%    0.0%    0.0%    0.0%    0.0%    0.0%    0.0%    0.0%    0.0%    0.0%    0.0%    0.0%    0.0%    0.0%    0.0%    0.0%    0.0%    0.0%    0.0%    0.0%    0.0%    0.0%    0.0%        k__Bacteria;p__Acidobacteria;c__Chloracidobacteria; o__    6682    0.0%    0.0%    0.0%    0.0%    0.0%    0.0%    0.0%    0.0%    0.0%    0.0%    0.0%    0.0%    0.0%    0.0%    0.0%    0.0%    0.0%    0.1%    0.0%    0.0%    0.0%    0.0%    0.0%    0.0%    0.0%    0.0%    0.0%    0.0%    0.0%    0.0%    0.0%    0.0%    0.0%    0.0%    0.0%    0.0%    0.1%    0.0%    0.0%    0.1%    0.0%    0.0%    0.0%    0.0%    0.0%    0.0%    0.0%    0.0%    0.0%    0.0%    0.0%    0.0%    0.1%    0.0%    0.0%    0.0%    0.0%    0.0%    0.0%    0.1%    0.0%    0.1%    0.0%    0.1%    0.3%    0.0%    0.0%    0.1%    0.0%    0.0%    0.0%    0.0%    0.0%    0.0%    0.1%    0.0%    0.0%    0.0%    0.0%    0.0%    0.0%    0.1%    0.0%    0.0%    0.0%    0.0%    0.1%    0.0%    0.0%    0.1%    0.0%    0.1%    0.0%    0.0%    0.0%    0.0%    0.1%    0.0%    0.1%    0.1%    0.1%    0.0%    0.0%    0.0%    0.0%    0.0%    0.1%    0.0%    0.0%    0.1%    0.1%    0.1%    0.0%    0.0%    0.1%    0.0%    0.0%    0.0%    0.0%    0.2%    0.0%    0.0%    0.1%    0.1%    0.0%    0.1%    0.1%    0.0%    0.1%    0.1%    0.1%    0.0%    0.1%    0.0%    0.2%    0.0%    0.0%    0.0%    0.0%    0.0%    0.0%    0.0%    0.0%    0.0%    0.1%    0.0%    0.0%    0.0%    0.0%    0.0%    0.0%    0.0%    0.1%    0.0%    0.0%    0.0%    0.0%    0.0%    0.0%    0.1%    0.1%    0.2%    0.1%    0.0%    0.0%    0.0%    0.0%    0.0%    0.0%    0.0%    0.1%    0.1%    0.1%    0.0%    0.0%    0.0%    0.0%    0.0%    0.0%    0.0%    0.0%    0.0%    0.0%    0.0%    0.0%    0.0%    0.0%    0.0%    0.0%    0.0%    0.0%    0.0%    0.0%    0.0%    0.0%    0.0%    0.1%    0.0%    0.0%    0.0%    0.0%    0.0%    0.0%    0.1%    0.1%    0.0%    0.0%    0.1%    0.2%    0.1%    0.1%    0.0%    0.3%    0.1%    0.0%    0.1%    0.0%    0.0%    0.2%    0.1%    0.0%    1.3%    0.0%    0.0%    0.0%    0.1%    0.0%    0.0%    0.1%    0.1%    0.0%    0.0%    0.3%    0.0%    0.1%    0.0%    0.1%    0.0%    0.0%    0.3%    0.1%    0.1%    0.0%    0.0%    0.0%    0.0%    0.0%    0.0%    0.0%    0.0%    0.1%    0.0%    0.0%    0.0%    0.0%    0.0%    0.0%    0.0%    0.0%    0.0%    0.1%    0.1%    0.1%    0.0%    0.0%    0.0%    0.1%    0.1%    0.0%    0.0%    0.0%    0.1%    0.1%    0.1%    0.0%    0.0%    0.0%    0.0%    0.0%    0.0%    0.0%    0.0%    0.0%    0.0%    0.0%    0.0%    0.0%    0.4%    0.0%    1.2%    0.0%    0.0%    0.1%    0.1%    0.1%    0.0%    0.0%    0.2%    0.0%    0.0%    0.0%    0.0%    0.0%    0.0%    0.0%    0.0%    0.0%    0.0%    0.1%    0.0%    0.0%    0.1%    0.0%    0.0%    0.0%    0.1%    0.0%    0.0%    0.0%    0.0%    0.0%    0.0%    0.0%    0.0%    0.0%    0.0%    0.0%    0.0%    0.0%    0.1%    0.0%    0.0%    0.0%    0.0%    0.0%    0.0%    0.0%    0.0%    0.0%    0.1%    0.0%    0.0%    0.0%    0.1%    0.0%    0.0%    0.0%    0.0%    0.0%    0.0%    0.0%    0.0%    0.0%    0.1%    0.0%    0.0%    0.0%    0.0%        k__Bacteria;p__Acidobacteria;c__Holophagae; o__Holophagales      19    0.0%    0.0%    0.0%    0.0%    0.0%    0.0%    0.0%    0.0%    0.0%    0.0%    0.0%    0.0%    0.0%    0.0%    0.0%    0.0%    0.0%    0.0%    0.0%    0.0%    0.0%    0.0%    0.0%    0.0%    0.0%    0.0%    0.0%    0.0%    0.0%    0.0%    0.0%    0.0%    0.0%    0.0%    0.0%    0.0%    0.0%    0.0%    0.0%    0.0%    0.0%    0.0%    0.0%    0.0%    0.0%    0.0%    0.0%    0.0%    0.0%    0.0%    0.0%    0.0%    0.0%    0.0%    0.0%    0.0%    0.0%    0.0%    0.0%    0.0%    0.0%    0.0%    0.0%    0.0%    0.0%    0.0%    0.0%    0.0%    0.0%    0.0%    0.0%    0.0%    0.0%    0.0%    0.0%    0.0%    0.0%    0.0%    0.0%    0.0%    0.0%    0.0%    0.0%    0.0%    0.0%    0.0%    0.0%    0.0%    0.0%    0.0%    0.0%    0.0%    0.0%    0.0%    0.0%    0.0%    0.0%    0.0%    0.0%    0.0%    0.0%    0.0%    0.0%    0.0%    0.0%    0.0%    0.0%    0.0%    0.0%    0.0%    0.0%    0.0%    0.0%    0.0%    0.0%    0.0%    0.0%    0.0%    0.0%    0.0%    0.0%    0.0%    0.0%    0.0%    0.0%    0.0%    0.0%    0.0%    0.0%    0.0%    0.0%    0.0%    0.0%    0.0%    0.0%    0.0%    0.0%    0.0%    0.0%    0.0%    0.0%    0.0%    0.0%    0.0%    0.0%    0.0%    0.0%    0.0%    0.0%    0.0%    0.0%    0.0%    0.0%    0.0%    0.0%    0.0%    0.0%    0.0%    0.0%    0.0%    0.0%    0.0%    0.0%    0.0%    0.0%    0.0%    0.0%    0.0%    0.0%    0.0%    0.0%    0.0%    0.0%    0.0%    0.0%    0.0%    0.0%    0.0%    0.0%    0.0%    0.0%    0.0%    0.0%    0.0%    0.0%    0.0%    0.0%    0.0%    0.0%    0.0%    0.0%    0.0%    0.0%    0.0%    0.0%    0.0%    0.0%    0.0%    0.0%    0.0%    0.0%    0.0%    0.0%    0.0%    0.0%    0.0%    0.0%    0.0%    0.0%    0.0%    0.0%    0.0%    0.0%    0.0%    0.0%    0.0%    0.0%    0.0%    0.0%    0.0%    0.0%    0.0%    0.0%    0.0%    0.0%    0.0%    0.0%    0.0%    0.0%    0.0%    0.0%    0.0%    0.0%    0.0%    0.0%    0.0%    0.0%    0.0%    0.0%    0.0%    0.0%    0.0%    0.0%    0.0%    0.0%    0.0%    0.0%    0.0%    0.0%    0.0%    0.0%    0.0%    0.0%    0.0%    0.0%    0.0%    0.0%    0.0%    0.0%    0.0%    0.0%    0.0%    0.0%    0.0%    0.0%    0.0%    0.0%    0.0%    0.0%    0.0%    0.0%    0.0%    0.0%    0.0%    0.0%    0.0%    0.0%    0.0%    0.0%    0.0%    0.0%    0.0%    0.0%    0.0%    0.0%    0.0%    0.0%    0.0%    0.0%    0.0%    0.0%    0.0%    0.0%    0.0%    0.0%    0.0%    0.0%    0.0%    0.0%    0.0%    0.0%    0.0%    0.0%    0.0%    0.0%    0.0%    0.0%    0.0%    0.0%    0.0%    0.0%    0.0%    0.0%    0.0%    0.0%    0.0%    0.0%    0.0%    0.0%    0.0%    0.0%    0.0%    0.0%    0.0%    0.0%    0.0%    0.0%    0.0%    0.0%    0.0%    0.0%    0.0%    0.0%    0.0%    0.0%    0.0%    0.0%    0.0%    0.0%    0.0%    0.0%    0.0%    0.0%    0.0%    0.0%    0.0%    0.0%    0.0%    0.0%    0.0%    0.0%    0.0%    0.0%    0.0%    0.0%    0.0%    0.0%    0.0%        k__Bacteria;p__Acidobacteria;c__MVS-40; o__      16    0.0%    0.0%    0.0%    0.0%    0.0%    0.0%    0.0%    0.0%    0.0%    0.0%    0.0%    0.0%    0.0%    0.0%    0.0%    0.0%    0.0%    0.0%    0.0%    0.0%    0.0%    0.0%    0.0%    0.0%    0.0%    0.0%    0.0%    0.0%    0.0%    0.0%    0.0%    0.0%    0.0%    0.0%    0.0%    0.0%    0.0%    0.0%    0.0%    0.0%    0.0%    0.0%    0.0%    0.0%    0.0%    0.0%    0.0%    0.0%    0.0%    0.0%    0.0%    0.0%    0.0%    0.0%    0.0%    0.0%    0.0%    0.0%    0.0%    0.0%    0.0%    0.0%    0.0%    0.0%    0.0%    0.0%    0.0%    0.0%    0.0%    0.0%    0.0%    0.0%    0.0%    0.0%    0.0%    0.0%    0.0%    0.0%    0.0%    0.0%    0.0%    0.0%    0.0%    0.0%    0.0%    0.0%    0.0%    0.0%    0.0%    0.0%    0.0%    0.0%    0.0%    0.0%    0.0%    0.0%    0.0%    0.0%    0.0%    0.0%    0.0%    0.0%    0.0%    0.0%    0.0%    0.0%    0.0%    0.0%    0.0%    0.0%    0.0%    0.0%    0.0%    0.0%    0.0%    0.0%    0.0%    0.0%    0.0%    0.0%    0.0%    0.0%    0.0%    0.0%    0.0%    0.0%    0.0%    0.0%    0.0%    0.0%    0.0%    0.0%    0.0%    0.0%    0.0%    0.0%    0.0%    0.0%    0.0%    0.0%    0.0%    0.0%    0.0%    0.0%    0.0%    0.0%    0.0%    0.0%    0.0%    0.0%    0.0%    0.0%    0.0%    0.0%    0.0%    0.0%    0.0%    0.0%    0.0%    0.0%    0.0%    0.0%    0.0%    0.0%    0.0%    0.0%    0.0%    0.0%    0.0%    0.0%    0.0%    0.0%    0.0%    0.0%    0.0%    0.0%    0.0%    0.0%    0.0%    0.0%    0.0%    0.0%    0.0%    0.0%    0.0%    0.0%    0.0%    0.0%    0.0%    0.0%    0.0%    0.0%    0.0%    0.0%    0.0%    0.0%    0.0%    0.0%    0.0%    0.0%    0.0%    0.0%    0.0%    0.0%    0.0%    0.0%    0.0%    0.0%    0.0%    0.0%    0.0%    0.0%    0.0%    0.0%    0.0%    0.0%    0.0%    0.0%    0.0%    0.0%    0.0%    0.0%    0.0%    0.0%    0.0%    0.0%    0.0%    0.0%    0.0%    0.0%    0.0%    0.0%    0.0%    0.0%    0.0%    0.0%    0.0%    0.0%    0.0%    0.0%    0.0%    0.0%    0.0%    0.0%    0.0%    0.0%    0.0%    0.0%    0.0%    0.0%    0.0%    0.0%    0.0%    0.0%    0.0%    0.0%    0.0%    0.0%    0.0%    0.0%    0.0%    0.0%    0.0%    0.0%    0.0%    0.0%    0.0%    0.0%    0.0%    0.0%    0.0%    0.0%    0.0%    0.0%    0.0%    0.0%    0.0%    0.0%    0.0%    0.0%    0.0%    0.0%    0.0%    0.0%    0.0%    0.0%    0.0%    0.0%    0.0%    0.0%    0.0%    0.0%    0.0%    0.0%    0.0%    0.0%    0.0%    0.0%    0.0%    0.0%    0.0%    0.0%    0.0%    0.0%    0.0%    0.0%    0.0%    0.0%    0.0%    0.0%    0.0%    0.0%    0.0%    0.0%    0.0%    0.0%    0.0%    0.0%    0.0%    0.0%    0.0%    0.0%    0.0%    0.0%    0.0%    0.0%    0.0%    0.0%    0.0%    0.0%    0.0%    0.0%    0.0%    0.0%    0.0%    0.0%    0.0%    0.0%    0.0%    0.0%    0.0%    0.0%    0.0%    0.0%    0.0%    0.0%    0.0%    0.0%    0.0%    0.0%    0.0%    0.0%    0.0%    0.0%    0.0%    0.0%    0.0%    0.0%        k__Bacteria;p__Acidobacteria;c__OS-K; o__       5    0.0%    0.0%    0.0%    0.0%    0.0%    0.0%    0.0%    0.0%    0.0%    0.0%    0.0%    0.0%    0.0%    0.0%    0.0%    0.0%    0.0%    0.0%    0.0%    0.0%    0.0%    0.0%    0.0%    0.0%    0.0%    0.0%    0.0%    0.0%    0.0%    0.0%    0.0%    0.0%    0.0%    0.0%    0.0%    0.0%    0.0%    0.0%    0.0%    0.0%    0.0%    0.0%    0.0%    0.0%    0.0%    0.0%    0.0%    0.0%    0.0%    0.0%    0.0%    0.0%    0.0%    0.0%    0.0%    0.0%    0.0%    0.0%    0.0%    0.0%    0.0%    0.0%    0.0%    0.0%    0.0%    0.0%    0.0%    0.0%    0.0%    0.0%    0.0%    0.0%    0.0%    0.0%    0.0%    0.0%    0.0%    0.0%    0.0%    0.0%    0.0%    0.0%    0.0%    0.0%    0.0%    0.0%    0.0%    0.0%    0.0%    0.0%    0.0%    0.0%    0.0%    0.0%    0.0%    0.0%    0.0%    0.0%    0.0%    0.0%    0.0%    0.0%    0.0%    0.0%    0.0%    0.0%    0.0%    0.0%    0.0%    0.0%    0.0%    0.0%    0.0%    0.0%    0.0%    0.0%    0.0%    0.0%    0.0%    0.0%    0.0%    0.0%    0.0%    0.0%    0.0%    0.0%    0.0%    0.0%    0.0%    0.0%    0.0%    0.0%    0.0%    0.0%    0.0%    0.0%    0.0%    0.0%    0.0%    0.0%    0.0%    0.0%    0.0%    0.0%    0.0%    0.0%    0.0%    0.0%    0.0%    0.0%    0.0%    0.0%    0.0%    0.0%    0.0%    0.0%    0.0%    0.0%    0.0%    0.0%    0.0%    0.0%    0.0%    0.0%    0.0%    0.0%    0.0%    0.0%    0.0%    0.0%    0.0%    0.0%    0.0%    0.0%    0.0%    0.0%    0.0%    0.0%    0.0%    0.0%    0.0%    0.0%    0.0%    0.0%    0.0%    0.0%    0.0%    0.0%    0.0%    0.0%    0.0%    0.0%    0.0%    0.0%    0.0%    0.0%    0.0%    0.0%    0.0%    0.0%    0.0%    0.0%    0.0%    0.0%    0.0%    0.0%    0.0%    0.0%    0.0%    0.0%    0.0%    0.0%    0.0%    0.0%    0.0%    0.0%    0.0%    0.0%    0.0%    0.0%    0.0%    0.0%    0.0%    0.0%    0.0%    0.0%    0.0%    0.0%    0.0%    0.0%    0.0%    0.0%    0.0%    0.0%    0.0%    0.0%    0.0%    0.0%    0.0%    0.0%    0.0%    0.0%    0.0%    0.0%    0.0%    0.0%    0.0%    0.0%    0.0%    0.0%    0.0%    0.0%    0.0%    0.0%    0.0%    0.0%    0.0%    0.0%    0.0%    0.0%    0.0%    0.0%    0.0%    0.0%    0.0%    0.0%    0.0%    0.0%    0.0%    0.0%    0.0%    0.0%    0.0%    0.0%    0.0%    0.0%    0.0%    0.0%    0.0%    0.0%    0.0%    0.0%    0.0%    0.0%    0.0%    0.0%    0.0%    0.0%    0.0%    0.0%    0.0%    0.0%    0.0%    0.0%    0.0%    0.0%    0.0%    0.0%    0.0%    0.0%    0.0%    0.0%    0.0%    0.0%    0.0%    0.0%    0.0%    0.0%    0.0%    0.0%    0.0%    0.0%    0.0%    0.0%    0.0%    0.0%    0.0%    0.0%    0.0%    0.0%    0.0%    0.0%    0.0%    0.0%    0.0%    0.0%    0.0%    0.0%    0.0%    0.0%    0.0%    0.0%    0.0%    0.0%    0.0%    0.0%    0.0%    0.0%    0.0%    0.0%    0.0%    0.0%    0.0%    0.0%    0.0%    0.0%    0.0%    0.0%    0.0%    0.0%    0.0%    0.0%    0.0%    0.0%    0.0%    0.0%    0.0%    0.0%        k__Bacteria;p__Acidobacteria;c__PAUC37f; o__      31    0.0%    0.0%    0.0%    0.0%    0.0%    0.0%    0.0%    0.0%    0.0%    0.0%    0.0%    0.0%    0.0%    0.0%    0.0%    0.0%    0.0%    0.0%    0.0%    0.0%    0.0%    0.0%    0.0%    0.0%    0.0%    0.0%    0.0%    0.0%    0.0%    0.0%    0.0%    0.0%    0.0%    0.0%    0.0%    0.0%    0.0%    0.0%    0.0%    0.0%    0.0%    0.0%    0.0%    0.0%    0.0%    0.0%    0.0%    0.0%    0.0%    0.0%    0.0%    0.0%    0.0%    0.0%    0.0%    0.0%    0.0%    0.0%    0.0%    0.0%    0.0%    0.0%    0.0%    0.0%    0.0%    0.0%    0.0%    0.0%    0.0%    0.0%    0.0%    0.0%    0.0%    0.0%    0.0%    0.0%    0.0%    0.0%    0.0%    0.0%    0.0%    0.0%    0.0%    0.0%    0.0%    0.0%    0.0%    0.0%    0.0%    0.0%    0.0%    0.0%    0.0%    0.0%    0.0%    0.0%    0.0%    0.0%    0.0%    0.0%    0.0%    0.0%    0.0%    0.0%    0.0%    0.0%    0.0%    0.0%    0.0%    0.0%    0.0%    0.0%    0.0%    0.0%    0.0%    0.0%    0.0%    0.0%    0.0%    0.0%    0.0%    0.0%    0.0%    0.0%    0.0%    0.0%    0.0%    0.0%    0.0%    0.0%    0.0%    0.0%    0.0%    0.0%    0.0%    0.0%    0.0%    0.0%    0.0%    0.0%    0.0%    0.0%    0.0%    0.0%    0.0%    0.0%    0.0%    0.0%    0.0%    0.0%    0.0%    0.0%    0.0%    0.0%    0.0%    0.0%    0.0%    0.0%    0.0%    0.0%    0.0%    0.0%    0.0%    0.0%    0.0%    0.0%    0.0%    0.0%    0.0%    0.0%    0.0%    0.0%    0.0%    0.0%    0.0%    0.0%    0.0%    0.0%    0.0%    0.0%    0.0%    0.0%    0.0%    0.0%    0.0%    0.0%    0.0%    0.0%    0.0%    0.0%    0.0%    0.0%    0.0%    0.0%    0.0%    0.0%    0.0%    0.0%    0.0%    0.0%    0.0%    0.0%    0.0%    0.0%    0.0%    0.0%    0.0%    0.0%    0.0%    0.0%    0.0%    0.0%    0.0%    0.0%    0.0%    0.0%    0.0%    0.0%    0.0%    0.0%    0.0%    0.0%    0.0%    0.0%    0.0%    0.0%    0.0%    0.0%    0.0%    0.0%    0.0%    0.0%    0.0%    0.0%    0.0%    0.0%    0.0%    0.0%    0.0%    0.0%    0.0%    0.0%    0.0%    0.0%    0.0%    0.0%    0.0%    0.0%    0.0%    0.0%    0.0%    0.0%    0.0%    0.0%    0.0%    0.0%    0.0%    0.0%    0.0%    0.0%    0.0%    0.0%    0.0%    0.0%    0.0%    0.0%    0.0%    0.0%    0.0%    0.0%    0.0%    0.0%    0.0%    0.0%    0.0%    0.0%    0.0%    0.0%    0.0%    0.0%    0.0%    0.0%    0.0%    0.0%    0.0%    0.0%    0.0%    0.0%    0.0%    0.0%    0.0%    0.0%    0.0%    0.0%    0.0%    0.0%    0.0%    0.0%    0.0%    0.0%    0.0%    0.0%    0.0%    0.0%    0.0%    0.0%    0.0%    0.0%    0.0%    0.0%    0.0%    0.0%    0.0%    0.0%    0.0%    0.0%    0.0%    0.0%    0.0%    0.0%    0.0%    0.0%    0.0%    0.0%    0.0%    0.0%    0.0%    0.0%    0.0%    0.0%    0.0%    0.0%    0.0%    0.0%    0.0%    0.0%    0.0%    0.0%    0.0%    0.0%    0.0%    0.0%    0.0%    0.0%    0.0%    0.0%    0.0%    0.0%    0.0%    0.0%    0.0%    0.0%    0.0%    0.0%    0.0%    0.0%    0.0%    0.0%        k__Bacteria;p__Acidobacteria;c__RB25; o__      17    0.0%    0.0%    0.0%    0.0%    0.0%    0.0%    0.0%    0.0%    0.0%    0.0%    0.0%    0.0%    0.0%    0.0%    0.0%    0.0%    0.0%    0.0%    0.0%    0.0%    0.0%    0.0%    0.0%    0.0%    0.0%    0.0%    0.0%    0.0%    0.0%    0.0%    0.0%    0.0%    0.0%    0.0%    0.0%    0.0%    0.0%    0.0%    0.0%    0.0%    0.0%    0.0%    0.0%    0.0%    0.0%    0.0%    0.0%    0.0%    0.0%    0.0%    0.0%    0.0%    0.0%    0.0%    0.0%    0.0%    0.0%    0.0%    0.0%    0.0%    0.0%    0.0%    0.0%    0.0%    0.0%    0.0%    0.0%    0.0%    0.0%    0.0%    0.0%    0.0%    0.0%    0.0%    0.0%    0.0%    0.0%    0.0%    0.0%    0.0%    0.0%    0.0%    0.0%    0.0%    0.0%    0.0%    0.0%    0.0%    0.0%    0.0%    0.0%    0.0%    0.0%    0.0%    0.0%    0.0%    0.0%    0.0%    0.0%    0.0%    0.0%    0.0%    0.0%    0.0%    0.0%    0.0%    0.0%    0.0%    0.0%    0.0%    0.0%    0.0%    0.0%    0.0%    0.0%    0.0%    0.0%    0.0%    0.0%    0.0%    0.0%    0.0%    0.0%    0.0%    0.0%    0.0%    0.0%    0.0%    0.0%    0.0%    0.0%    0.0%    0.0%    0.0%    0.0%    0.0%    0.0%    0.0%    0.0%    0.0%    0.0%    0.0%    0.0%    0.0%    0.0%    0.0%    0.0%    0.0%    0.0%    0.0%    0.0%    0.0%    0.0%    0.0%    0.0%    0.0%    0.0%    0.0%    0.0%    0.0%    0.0%    0.0%    0.0%    0.0%    0.0%    0.0%    0.0%    0.0%    0.0%    0.0%    0.0%    0.0%    0.0%    0.0%    0.0%    0.0%    0.0%    0.0%    0.0%    0.0%    0.0%    0.0%    0.0%    0.0%    0.0%    0.0%    0.0%    0.0%    0.0%    0.0%    0.0%    0.0%    0.0%    0.0%    0.0%    0.0%    0.0%    0.0%    0.0%    0.0%    0.0%    0.0%    0.0%    0.0%    0.0%    0.0%    0.0%    0.0%    0.0%    0.0%    0.0%    0.0%    0.0%    0.0%    0.0%    0.0%    0.0%    0.0%    0.0%    0.0%    0.0%    0.0%    0.0%    0.0%    0.0%    0.0%    0.0%    0.0%    0.0%    0.0%    0.0%    0.0%    0.0%    0.0%    0.0%    0.0%    0.0%    0.0%    0.0%    0.0%    0.0%    0.0%    0.0%    0.0%    0.0%    0.0%    0.0%    0.0%    0.0%    0.0%    0.0%    0.0%    0.0%    0.0%    0.0%    0.0%    0.0%    0.0%    0.0%    0.0%    0.0%    0.0%    0.0%    0.0%    0.0%    0.0%    0.0%    0.0%    0.0%    0.0%    0.0%    0.0%    0.0%    0.0%    0.0%    0.0%    0.0%    0.0%    0.0%    0.0%    0.0%    0.0%    0.0%    0.0%    0.0%    0.0%    0.0%    0.0%    0.0%    0.0%    0.0%    0.0%    0.0%    0.0%    0.0%    0.0%    0.0%    0.0%    0.0%    0.0%    0.0%    0.0%    0.0%    0.0%    0.0%    0.0%    0.0%    0.0%    0.0%    0.0%    0.0%    0.0%    0.0%    0.0%    0.0%    0.0%    0.0%    0.0%    0.0%    0.0%    0.0%    0.0%    0.0%    0.0%    0.0%    0.0%    0.0%    0.0%    0.0%    0.0%    0.0%    0.0%    0.0%    0.0%    0.0%    0.0%    0.0%    0.0%    0.0%    0.0%    0.0%    0.0%    0.0%    0.0%    0.0%    0.0%    0.0%    0.0%    0.0%    0.0%    0.0%    0.0%    0.0%    0.0%    0.0%    0.0%    0.0%    0.0%        k__Bacteria;p__Acidobacteria;c__Solibacteres; o__Solibacterales    2539    0.0%    0.0%    0.0%    0.0%    0.0%    0.0%    0.0%    0.0%    0.0%    0.0%    0.0%    0.0%    0.0%    0.0%    0.0%    0.0%    0.0%    0.0%    0.0%    0.0%    0.0%    0.0%    0.0%    0.0%    0.0%    0.0%    0.0%    0.0%    0.0%    0.0%    0.0%    0.0%    0.1%    0.0%    0.0%    0.0%    0.0%    0.0%    0.0%    0.0%    0.0%    0.0%    0.0%    0.0%    0.0%    0.0%    0.0%    0.0%    0.0%    0.0%    0.0%    0.0%    0.0%    0.0%    0.1%    0.0%    0.0%    0.0%    0.0%    0.0%    0.0%    0.0%    0.0%    0.1%    0.0%    0.0%    0.0%    0.0%    0.0%    0.0%    0.0%    0.0%    0.0%    0.0%    0.0%    0.0%    0.0%    0.0%    0.0%    0.0%    0.0%    0.0%    0.0%    0.0%    0.0%    0.0%    0.0%    0.0%    0.0%    0.0%    0.0%    0.0%    0.0%    0.0%    0.0%    0.0%    0.0%    0.0%    0.0%    0.0%    0.0%    0.0%    0.0%    0.0%    0.0%    0.0%    0.1%    0.0%    0.0%    0.0%    0.0%    0.0%    0.0%    0.0%    0.0%    0.0%    0.0%    0.0%    0.0%    0.1%    0.0%    0.0%    0.0%    0.0%    0.0%    0.0%    0.2%    0.0%    0.0%    0.1%    0.1%    0.0%    0.1%    0.0%    0.1%    0.0%    0.0%    0.0%    0.0%    0.0%    0.0%    0.0%    0.0%    0.0%    0.0%    0.0%    0.0%    0.0%    0.0%    0.0%    0.0%    0.0%    0.0%    0.0%    0.0%    0.0%    0.0%    0.0%    0.0%    0.1%    0.1%    0.0%    0.0%    0.0%    0.0%    0.0%    0.0%    0.0%    0.0%    0.0%    0.0%    0.0%    0.0%    0.0%    0.0%    0.0%    0.0%    0.0%    0.0%    0.0%    0.0%    0.0%    0.0%    0.0%    0.0%    0.0%    0.0%    0.0%    0.0%    0.0%    0.0%    0.0%    0.0%    0.0%    0.0%    0.0%    0.0%    0.0%    0.0%    0.0%    0.0%    0.0%    0.0%    0.1%    0.0%    0.0%    0.0%    0.0%    0.1%    0.0%    0.0%    0.0%    0.1%    0.0%    0.0%    0.1%    0.0%    0.0%    0.0%    0.0%    0.0%    0.0%    0.0%    0.0%    0.0%    0.0%    0.0%    0.0%    0.0%    0.0%    0.0%    0.0%    0.0%    0.0%    0.0%    0.0%    0.1%    0.0%    0.0%    0.1%    0.0%    0.0%    0.0%    0.0%    0.0%    0.0%    0.0%    0.0%    0.0%    0.0%    0.0%    0.0%    0.0%    0.0%    0.0%    0.0%    0.0%    0.0%    0.0%    0.0%    0.0%    0.0%    0.0%    0.0%    0.0%    0.0%    0.0%    0.0%    0.0%    0.0%    0.0%    0.0%    0.0%    0.0%    0.0%    0.0%    0.0%    0.0%    0.0%    0.0%    0.0%    0.0%    0.0%    0.0%    0.0%    0.0%    0.0%    0.0%    0.0%    0.0%    0.0%    0.0%    0.0%    0.0%    0.0%    0.0%    0.0%    0.0%    0.0%    0.0%    0.0%    0.0%    0.0%    0.0%    0.0%    0.0%    0.0%    0.0%    0.0%    0.0%    0.0%    0.0%    0.0%    0.0%    0.0%    0.0%    0.0%    0.0%    0.0%    0.0%    0.0%    0.0%    0.0%    0.0%    0.0%    0.0%    0.0%    0.0%    0.0%    0.0%    0.0%    0.0%    0.0%    0.0%    0.0%    0.0%    0.0%    0.0%    0.0%    0.0%    0.0%    0.0%    0.0%    0.0%    0.0%    0.0%    0.0%    0.0%    0.0%    0.0%    0.0%    0.0%    0.0%    0.1%    0.0%    0.0%    0.0%    0.0%        k__Bacteria;p__Acidobacteria;c__Sva0725; o__     563    0.0%    0.0%    0.0%    0.0%    0.0%    0.0%    0.0%    0.0%    0.0%    0.0%    0.0%    0.0%    0.0%    0.0%    0.0%    0.0%    0.0%    0.0%    0.0%    0.0%    0.0%    0.0%    0.0%    0.0%    0.0%    0.0%    0.0%    0.0%    0.0%    0.0%    0.0%    0.0%    0.0%    0.0%    0.0%    0.0%    0.0%    0.0%    0.0%    0.0%    0.0%    0.0%    0.0%    0.0%    0.0%    0.0%    0.0%    0.0%    0.0%    0.0%    0.0%    0.0%    0.0%    0.0%    0.0%    0.0%    0.0%    0.0%    0.0%    0.0%    0.0%    0.0%    0.0%    0.0%    0.0%    0.0%    0.0%    0.0%    0.0%    0.1%    0.0%    0.0%    0.0%    0.0%    0.0%    0.0%    0.0%    0.0%    0.0%    0.0%    0.0%    0.0%    0.0%    0.0%    0.0%    0.0%    0.0%    0.0%    0.0%    0.0%    0.0%    0.0%    0.0%    0.0%    0.0%    0.0%    0.0%    0.0%    0.0%    0.0%    0.0%    0.0%    0.0%    0.0%    0.0%    0.0%    0.0%    0.0%    0.0%    0.0%    0.0%    0.0%    0.0%    0.0%    0.0%    0.0%    0.0%    0.0%    0.0%    0.0%    0.0%    0.0%    0.0%    0.0%    0.0%    0.0%    0.0%    0.0%    0.0%    0.0%    0.0%    0.0%    0.0%    0.0%    0.0%    0.0%    0.0%    0.0%    0.0%    0.0%    0.0%    0.0%    0.0%    0.0%    0.0%    0.0%    0.0%    0.0%    0.0%    0.0%    0.0%    0.0%    0.0%    0.0%    0.0%    0.0%    0.0%    0.0%    0.0%    0.0%    0.0%    0.0%    0.0%    0.0%    0.0%    0.0%    0.0%    0.0%    0.0%    0.0%    0.0%    0.0%    0.0%    0.0%    0.0%    0.0%    0.0%    0.0%    0.0%    0.0%    0.0%    0.0%    0.0%    0.0%    0.0%    0.0%    0.0%    0.0%    0.0%    0.0%    0.0%    0.0%    0.0%    0.0%    0.0%    0.0%    0.0%    0.0%    0.0%    0.0%    0.0%    0.0%    0.0%    0.0%    0.0%    0.0%    0.0%    0.0%    0.0%    0.0%    0.0%    0.0%    0.0%    0.0%    0.0%    0.0%    0.0%    0.0%    0.0%    0.0%    0.0%    0.0%    0.0%    0.0%    0.0%    0.0%    0.0%    0.0%    0.0%    0.0%    0.0%    0.0%    0.0%    0.0%    0.0%    0.0%    0.0%    0.0%    0.0%    0.0%    0.0%    0.0%    0.0%    0.0%    0.0%    0.0%    0.0%    0.0%    0.0%    0.0%    0.0%    0.0%    0.0%    0.0%    0.0%    0.0%    0.0%    0.0%    0.0%    0.0%    0.0%    0.0%    0.0%    0.0%    0.0%    0.0%    0.0%    0.0%    0.0%    0.0%    0.0%    0.0%    0.0%    0.0%    0.0%    0.0%    0.0%    0.0%    0.0%    0.0%    0.0%    0.0%    0.0%    0.0%    0.0%    0.0%    0.0%    0.0%    0.0%    0.0%    0.0%    0.0%    0.0%    0.0%    0.0%    0.0%    0.0%    0.0%    0.0%    0.0%    0.0%    0.0%    0.0%    0.0%    0.0%    0.0%    0.0%    0.0%    0.0%    0.0%    0.0%    0.0%    0.0%    0.0%    0.1%    0.0%    0.0%    0.0%    0.0%    0.0%    0.0%    0.0%    0.0%    0.0%    0.0%    0.0%    0.0%    0.0%    0.0%    0.0%    0.0%    0.0%    0.0%    0.0%    0.0%    0.0%    0.0%    0.0%    0.0%    0.0%    0.0%    0.0%    0.0%    0.0%    0.0%    0.0%    0.0%    0.0%    0.0%    0.0%    0.0%    0.0%    0.0%    0.0%    0.0%    0.0%    0.0%    0.0%        k__Bacteria;p__Acidobacteria;c__iii1-8; o__     514    0.0%    0.0%    0.0%    0.0%    0.0%    0.0%    0.0%    0.0%    0.0%    0.0%    0.0%    0.0%    0.0%    0.0%    0.0%    0.0%    0.0%    0.0%    0.0%    0.0%    0.0%    0.0%    0.0%    0.0%    0.0%    0.0%    0.0%    0.0%    0.0%    0.0%    0.0%    0.0%    0.0%    0.0%    0.0%    0.0%    0.0%    0.0%    0.0%    0.0%    0.0%    0.0%    0.0%    0.0%    0.0%    0.0%    0.0%    0.0%    0.0%    0.0%    0.0%    0.0%    0.0%    0.0%    0.0%    0.0%    0.0%    0.0%    0.0%    0.0%    0.0%    0.0%    0.0%    0.0%    0.0%    0.0%    0.0%    0.0%    0.0%    0.0%    0.0%    0.0%    0.0%    0.0%    0.0%    0.0%    0.0%    0.0%    0.0%    0.0%    0.0%    0.0%    0.0%    0.0%    0.0%    0.0%    0.0%    0.0%    0.0%    0.0%    0.0%    0.0%    0.0%    0.0%    0.0%    0.0%    0.0%    0.0%    0.0%    0.0%    0.0%    0.0%    0.0%    0.0%    0.0%    0.0%    0.0%    0.0%    0.0%    0.0%    0.0%    0.0%    0.0%    0.0%    0.0%    0.0%    0.0%    0.0%    0.0%    0.0%    0.0%    0.0%    0.0%    0.0%    0.0%    0.0%    0.0%    0.0%    0.0%    0.0%    0.0%    0.0%    0.0%    0.0%    0.0%    0.0%    0.0%    0.0%    0.0%    0.0%    0.0%    0.0%    0.0%    0.0%    0.0%    0.0%    0.0%    0.0%    0.0%    0.0%    0.0%    0.0%    0.0%    0.0%    0.0%    0.0%    0.0%    0.0%    0.0%    0.0%    0.0%    0.0%    0.0%    0.0%    0.0%    0.0%    0.0%    0.0%    0.0%    0.0%    0.0%    0.0%    0.0%    0.0%    0.0%    0.0%    0.0%    0.0%    0.0%    0.0%    0.0%    0.0%    0.0%    0.0%    0.0%    0.0%    0.0%    0.0%    0.0%    0.0%    0.0%    0.0%    0.0%    0.0%    0.0%    0.0%    0.0%    0.0%    0.0%    0.0%    0.0%    0.0%    0.0%    0.0%    0.0%    0.0%    0.0%    0.0%    0.0%    0.0%    0.0%    0.0%    0.0%    0.0%    0.0%    0.0%    0.0%    0.0%    0.0%    0.0%    0.0%    0.0%    0.0%    0.0%    0.0%    0.0%    0.0%    0.0%    0.0%    0.0%    0.0%    0.0%    0.0%    0.0%    0.0%    0.0%    0.0%    0.0%    0.0%    0.0%    0.0%    0.0%    0.0%    0.0%    0.0%    0.0%    0.0%    0.0%    0.0%    0.0%    0.0%    0.0%    0.0%    0.0%    0.0%    0.0%    0.0%    0.0%    0.0%    0.0%    0.0%    0.0%    0.0%    0.0%    0.0%    0.0%    0.0%    0.0%    0.0%    0.0%    0.0%    0.0%    0.0%    0.0%    0.0%    0.0%    0.0%    0.0%    0.0%    0.0%    0.0%    0.0%    0.0%    0.0%    0.0%    0.0%    0.0%    0.0%    0.0%    0.0%    0.0%    0.0%    0.0%    0.0%    0.0%    0.0%    0.0%    0.0%    0.0%    0.0%    0.0%    0.0%    0.0%    0.0%    0.0%    0.0%    0.0%    0.0%    0.0%    0.0%    0.0%    0.0%    0.0%    0.0%    0.0%    0.0%    0.0%    0.0%    0.0%    0.0%    0.0%    0.0%    0.0%    0.0%    0.0%    0.0%    0.0%    0.0%    0.0%    0.0%    0.0%    0.0%    0.0%    0.0%    0.0%    0.0%    0.0%    0.0%    0.0%    0.0%    0.0%    0.0%    0.0%    0.0%    0.0%    0.0%    0.0%    0.0%    0.0%    0.0%    0.0%    0.0%    0.0%    0.0%    0.0%    0.0%    0.0%    0.0%        k__Bacteria;p__Actinobacteria;c__; o__    1871    0.0%    0.0%    0.0%    0.0%    0.0%    0.0%    0.0%    0.0%    0.0%    0.0%    0.0%    0.0%    0.0%    0.0%    0.0%    0.0%    0.0%    0.0%    0.0%    0.0%    0.0%    0.0%    0.0%    0.0%    0.0%    0.0%    0.0%    0.0%    0.0%    0.0%    0.0%    0.0%    0.0%    0.0%    0.0%    0.0%    0.0%    0.0%    0.0%    0.0%    0.0%    0.0%    0.0%    0.0%    0.0%    0.0%    0.0%    0.0%    0.0%    0.0%    0.0%    0.0%    0.0%    0.0%    0.0%    0.0%    0.0%    0.0%    0.0%    0.0%    0.0%    0.0%    0.0%    0.0%    0.0%    0.0%    0.0%    0.0%    0.0%    0.0%    0.0%    0.0%    0.0%    0.0%    0.0%    0.0%    0.0%    0.0%    0.0%    0.0%    0.0%    0.0%    0.0%    0.0%    0.0%    0.0%    0.0%    0.0%    0.0%    0.0%    0.0%    0.0%    0.0%    0.0%    0.0%    0.0%    0.0%    0.0%    0.1%    0.0%    0.0%    0.0%    0.0%    0.0%    0.0%    0.0%    0.0%    0.0%    0.0%    0.0%    0.0%    0.0%    0.0%    0.0%    0.0%    0.0%    0.0%    0.0%    0.0%    0.0%    0.0%    0.0%    0.0%    0.0%    0.0%    0.0%    0.0%    0.0%    0.0%    0.0%    0.0%    0.0%    0.0%    0.0%    0.0%    0.0%    0.0%    0.0%    0.0%    0.0%    0.0%    0.0%    0.0%    0.0%    0.0%    0.0%    0.0%    0.0%    0.1%    0.0%    0.0%    0.0%    0.0%    0.0%    0.0%    0.6%    0.1%    0.0%    0.0%    0.0%    0.0%    0.1%    0.0%    0.0%    0.0%    0.0%    0.0%    0.0%    0.0%    0.0%    0.0%    0.0%    0.0%    0.0%    0.0%    0.0%    0.0%    0.0%    0.0%    0.0%    0.0%    0.0%    0.0%    0.0%    0.0%    0.0%    0.0%    0.0%    0.1%    0.0%    0.0%    0.0%    0.0%    0.0%    0.0%    0.0%    0.0%    0.0%    0.0%    0.0%    0.0%    0.0%    0.0%    0.0%    0.1%    0.0%    0.0%    0.0%    0.0%    0.0%    0.0%    0.0%    0.0%    0.0%    0.0%    0.0%    0.1%    0.0%    0.1%    0.0%    0.0%    0.0%    0.0%    0.0%    0.0%    0.0%    0.0%    0.0%    0.0%    0.0%    0.0%    0.0%    0.1%    0.0%    0.0%    0.0%    0.0%    0.0%    0.0%    0.0%    0.0%    0.0%    0.0%    0.0%    0.0%    0.0%    0.0%    0.0%    0.0%    0.0%    0.0%    0.0%    0.0%    0.0%    0.0%    0.0%    0.0%    0.0%    0.0%    0.0%    0.0%    0.0%    0.0%    0.0%    0.0%    0.0%    0.0%    0.0%    0.0%    0.0%    0.0%    0.0%    0.0%    0.0%    0.0%    0.0%    0.0%    0.0%    0.0%    0.0%    0.0%    0.0%    0.0%    0.0%    0.0%    0.0%    0.0%    0.0%    0.0%    0.0%    0.0%    0.0%    0.0%    0.0%    0.0%    0.0%    0.0%    0.0%    0.0%    0.0%    0.0%    0.0%    0.0%    0.0%    0.0%    0.0%    0.0%    0.0%    0.0%    0.0%    0.0%    0.1%    0.0%    0.0%    0.0%    0.0%    0.0%    0.0%    0.0%    0.0%    0.0%    0.0%    0.0%    0.0%    0.0%    0.0%    0.0%    0.0%    0.0%    0.0%    0.0%    0.0%    0.0%    0.0%    0.0%    0.0%    0.0%    0.0%    0.0%    0.0%    0.0%    0.0%    0.0%    0.0%    0.0%    0.0%    0.0%    0.0%    0.0%    0.0%    0.0%    0.0%    0.2%    0.0%    0.0%    0.0%    0.0%    0.0%        k__Bacteria;p__Actinobacteria;c__Actinobacteria (class); o__     565    0.0%    0.0%    0.0%    0.0%    0.0%    0.0%    0.0%    0.0%    0.0%    0.0%    0.0%    0.0%    0.0%    0.0%    0.0%    0.0%    0.0%    0.0%    0.0%    0.0%    0.0%    0.0%    0.0%    0.0%    0.0%    0.0%    0.0%    0.0%    0.0%    0.0%    0.0%    0.0%    0.0%    0.0%    0.0%    0.0%    0.0%    0.0%    0.0%    0.0%    0.0%    0.0%    0.0%    0.0%    0.0%    0.0%    0.0%    0.0%    0.0%    0.0%    0.0%    0.0%    0.0%    0.0%    0.0%    0.0%    0.0%    0.0%    0.0%    0.0%    0.0%    0.0%    0.0%    0.0%    0.0%    0.0%    0.0%    0.0%    0.0%    0.0%    0.0%    0.0%    0.0%    0.0%    0.0%    0.0%    0.0%    0.0%    0.0%    0.0%    0.0%    0.0%    0.0%    0.0%    0.0%    0.0%    0.0%    0.0%    0.0%    0.0%    0.0%    0.0%    0.0%    0.0%    0.0%    0.0%    0.0%    0.0%    0.0%    0.0%    0.0%    0.0%    0.0%    0.0%    0.0%    0.0%    0.0%    0.0%    0.0%    0.0%    0.0%    0.0%    0.0%    0.0%    0.0%    0.0%    0.0%    0.0%    0.0%    0.0%    0.0%    0.0%    0.0%    0.0%    0.0%    0.0%    0.0%    0.0%    0.0%    0.0%    0.0%    0.0%    0.0%    0.0%    0.0%    0.0%    0.0%    0.0%    0.0%    0.0%    0.0%    0.0%    0.0%    0.0%    0.0%    0.0%    0.0%    0.0%    0.0%    0.0%    0.0%    0.0%    0.0%    0.0%    0.0%    0.0%    0.0%    0.0%    0.0%    0.0%    0.1%    0.0%    0.0%    0.0%    0.0%    0.0%    0.0%    0.0%    0.0%    0.0%    0.0%    0.0%    0.0%    0.0%    0.0%    0.0%    0.0%    0.0%    0.0%    0.0%    0.0%    0.0%    0.0%    0.0%    0.0%    0.0%    0.0%    0.0%    0.0%    0.0%    0.0%    0.0%    0.0%    0.0%    0.0%    0.0%    0.0%    0.0%    0.0%    0.0%    0.0%    0.0%    0.0%    0.0%    0.0%    0.0%    0.0%    0.0%    0.0%    0.0%    0.0%    0.0%    0.0%    0.0%    0.0%    0.0%    0.0%    0.0%    0.0%    0.0%    0.0%    0.0%    0.0%    0.0%    0.0%    0.0%    0.0%    0.0%    0.0%    0.0%    0.0%    0.0%    0.0%    0.0%    0.0%    0.0%    0.0%    0.0%    0.0%    0.0%    0.0%    0.0%    0.0%    0.0%    0.0%    0.0%    0.0%    0.0%    0.0%    0.0%    0.0%    0.0%    0.0%    0.0%    0.0%    0.0%    0.0%    0.0%    0.0%    0.0%    0.0%    0.0%    0.0%    0.0%    0.0%    0.0%    0.0%    0.0%    0.0%    0.0%    0.0%    0.0%    0.0%    0.0%    0.0%    0.0%    0.0%    0.0%    0.0%    0.0%    0.0%    0.0%    0.0%    0.0%    0.0%    0.0%    0.0%    0.0%    0.0%    0.0%    0.0%    0.0%    0.0%    0.0%    0.0%    0.0%    0.0%    0.0%    0.0%    0.0%    0.0%    0.0%    0.0%    0.0%    0.0%    0.0%    0.0%    0.0%    0.0%    0.0%    0.0%    0.0%    0.0%    0.0%    0.0%    0.0%    0.0%    0.0%    0.0%    0.0%    0.0%    0.0%    0.0%    0.0%    0.0%    0.0%    0.0%    0.0%    0.0%    0.0%    0.0%    0.0%    0.0%    0.0%    0.0%    0.0%    0.0%    0.0%    0.0%    0.0%    0.0%    0.0%    0.0%    0.0%    0.0%    0.0%    0.0%    0.0%    0.0%    0.0%    0.0%    0.0%    0.0%    0.0%    0.0%    0.0%    0.0%    0.0%        k__Bacteria;p__Actinobacteria;c__Actinobacteria (class); o__0319-7L14    1877    0.0%    0.0%    0.0%    0.0%    0.0%    0.0%    0.0%    0.0%    0.0%    0.0%    0.0%    0.0%    0.0%    0.0%    0.0%    0.0%    0.0%    0.0%    0.0%    0.0%    0.0%    0.0%    0.0%    0.0%    0.0%    0.0%    0.0%    0.0%    0.0%    0.0%    0.0%    0.0%    0.0%    0.0%    0.0%    0.0%    0.0%    0.0%    0.0%    0.0%    0.0%    0.0%    0.0%    0.0%    0.0%    0.0%    0.0%    0.0%    0.0%    0.0%    0.0%    0.0%    0.0%    0.0%    0.0%    0.0%    0.0%    0.0%    0.0%    0.0%    0.0%    0.0%    0.0%    0.1%    0.0%    0.0%    0.0%    0.1%    0.0%    0.0%    0.0%    0.0%    0.0%    0.0%    0.0%    0.0%    0.0%    0.0%    0.0%    0.0%    0.0%    0.0%    0.0%    0.0%    0.0%    0.0%    0.0%    0.0%    0.0%    0.0%    0.0%    0.0%    0.0%    0.0%    0.0%    0.0%    0.0%    0.0%    0.0%    0.0%    0.1%    0.0%    0.0%    0.0%    0.0%    0.0%    0.0%    0.0%    0.0%    0.0%    0.0%    0.0%    0.0%    0.0%    0.0%    0.0%    0.0%    0.0%    0.0%    0.0%    0.0%    0.0%    0.0%    0.0%    0.0%    0.0%    0.0%    0.0%    0.0%    0.1%    0.0%    0.0%    0.0%    0.0%    0.1%    0.0%    0.0%    0.0%    0.0%    0.0%    0.0%    0.0%    0.0%    0.0%    0.1%    0.0%    0.0%    0.0%    0.0%    0.0%    0.0%    0.0%    0.0%    0.0%    0.0%    0.0%    0.0%    0.0%    0.0%    0.0%    0.1%    0.0%    0.0%    0.0%    0.0%    0.0%    0.0%    0.0%    0.0%    0.0%    0.0%    0.0%    0.0%    0.0%    0.0%    0.0%    0.0%    0.0%    0.0%    0.0%    0.0%    0.0%    0.0%    0.0%    0.0%    0.0%    0.0%    0.0%    0.0%    0.0%    0.0%    0.0%    0.0%    0.0%    0.0%    0.0%    0.0%    0.0%    0.0%    0.0%    0.0%    0.0%    0.0%    0.0%    0.0%    0.0%    0.0%    0.0%    0.1%    0.0%    0.0%    0.0%    0.0%    0.0%    0.0%    0.0%    0.0%    0.0%    0.0%    0.0%    0.0%    0.0%    0.0%    0.0%    0.0%    0.0%    0.0%    0.0%    0.0%    0.0%    0.0%    0.0%    0.0%    0.0%    0.1%    0.0%    0.0%    0.0%    0.0%    0.0%    0.0%    0.0%    0.0%    0.0%    0.0%    0.0%    0.0%    0.0%    0.0%    0.0%    0.1%    0.0%    0.0%    0.0%    0.0%    0.0%    0.0%    0.0%    0.0%    0.0%    0.0%    0.0%    0.0%    0.0%    0.0%    0.0%    0.0%    0.0%    0.0%    0.0%    0.0%    0.0%    0.0%    0.0%    0.0%    0.0%    0.0%    0.0%    0.0%    0.0%    0.0%    0.0%    0.0%    0.0%    0.0%    0.0%    0.0%    0.0%    0.0%    0.0%    0.0%    0.0%    0.0%    0.0%    0.0%    0.0%    0.0%    0.0%    0.0%    0.0%    0.0%    0.0%    0.0%    0.0%    0.0%    0.0%    0.0%    0.0%    0.0%    0.0%    0.0%    0.0%    0.0%    0.0%    0.0%    0.0%    0.0%    0.0%    0.0%    0.0%    0.0%    0.0%    0.0%    0.0%    0.0%    0.0%    0.0%    0.0%    0.0%    0.0%    0.0%    0.0%    0.0%    0.0%    0.0%    0.0%    0.0%    0.0%    0.0%    0.0%    0.0%    0.0%    0.0%    0.0%    0.0%    0.0%    0.0%    0.0%    0.0%    0.0%    0.0%    0.0%    0.0%    0.0%    0.0%    0.0%    0.0%    0.0%        k__Bacteria;p__Actinobacteria;c__Actinobacteria (class); o__Acidimicrobiales    9844    0.1%    0.0%    0.1%    0.0%    0.1%    0.0%    0.0%    0.0%    0.0%    0.0%    0.0%    0.0%    0.0%    0.0%    0.0%    0.0%    0.0%    0.0%    0.0%    0.0%    0.1%    0.1%    0.0%    0.0%    0.0%    0.0%    0.0%    0.0%    0.0%    0.0%    0.0%    0.1%    0.0%    0.0%    0.1%    0.0%    0.1%    0.0%    0.0%    0.2%    0.0%    0.0%    0.0%    0.0%    0.1%    0.0%    0.0%    0.0%    0.0%    0.0%    0.1%    0.0%    0.0%    0.0%    0.1%    0.1%    0.0%    0.0%    0.1%    0.2%    0.1%    0.0%    0.0%    0.2%    0.1%    0.2%    0.0%    0.2%    0.0%    0.1%    0.0%    0.0%    0.0%    0.1%    0.1%    0.0%    0.0%    0.1%    0.0%    0.1%    0.0%    0.0%    0.0%    0.0%    0.0%    0.0%    0.1%    0.0%    0.0%    0.1%    0.0%    0.1%    0.1%    0.0%    0.0%    0.0%    0.2%    0.0%    0.1%    0.1%    0.1%    0.0%    0.0%    0.0%    0.0%    0.0%    0.2%    0.0%    0.0%    0.0%    0.0%    0.1%    0.0%    0.0%    0.1%    0.0%    0.0%    0.0%    0.0%    0.5%    0.0%    0.1%    0.2%    0.1%    0.0%    0.3%    0.1%    0.0%    0.3%    0.4%    0.1%    0.0%    0.1%    0.0%    0.3%    0.0%    0.1%    0.1%    0.0%    0.0%    0.0%    0.1%    0.1%    0.0%    0.1%    0.1%    0.0%    0.0%    0.0%    0.0%    0.0%    0.0%    0.1%    0.0%    0.0%    0.0%    0.2%    0.0%    0.0%    0.2%    0.5%    0.1%    0.2%    0.1%    0.0%    0.1%    0.2%    0.1%    0.1%    0.1%    0.1%    0.1%    0.1%    0.1%    0.0%    0.0%    0.0%    0.0%    0.0%    0.0%    0.0%    0.0%    0.0%    0.0%    0.0%    0.0%    0.0%    0.0%    0.0%    0.0%    0.0%    0.0%    0.0%    0.0%    0.0%    0.6%    0.2%    0.0%    0.0%    0.0%    0.1%    0.0%    0.0%    0.2%    0.1%    0.0%    0.0%    0.1%    0.2%    0.1%    0.1%    0.0%    0.1%    0.0%    0.0%    0.0%    0.0%    0.0%    0.0%    0.0%    0.1%    0.1%    0.0%    0.1%    0.0%    0.0%    0.0%    0.0%    0.1%    0.1%    0.0%    0.0%    0.2%    0.0%    0.2%    0.0%    0.2%    0.0%    0.0%    0.0%    0.1%    0.0%    0.0%    0.0%    0.0%    0.0%    0.0%    0.1%    0.0%    0.0%    0.1%    0.1%    0.0%    0.0%    0.0%    0.0%    0.0%    0.0%    0.1%    0.0%    0.1%    0.1%    0.1%    0.0%    0.0%    0.0%    0.1%    0.1%    0.0%    0.0%    0.1%    0.1%    0.3%    0.1%    0.0%    0.0%    0.0%    0.1%    0.0%    0.0%    0.0%    0.0%    0.0%    0.0%    0.1%    0.0%    0.1%    0.4%    0.0%    0.0%    0.0%    0.0%    0.2%    0.1%    0.1%    0.0%    0.1%    0.1%    0.0%    0.1%    0.1%    0.0%    0.0%    0.1%    0.1%    0.0%    0.1%    0.0%    0.0%    0.0%    0.2%    0.0%    0.0%    0.0%    0.0%    0.2%    0.0%    0.0%    0.0%    0.0%    0.0%    0.1%    0.0%    0.0%    0.1%    0.1%    0.0%    0.0%    0.1%    0.1%    0.0%    0.0%    0.0%    0.0%    0.0%    0.0%    0.0%    0.0%    0.0%    0.0%    0.0%    0.0%    0.0%    0.0%    0.1%    0.0%    0.0%    0.0%    0.0%    0.0%    0.0%    0.0%    0.0%    0.0%    0.0%    0.0%    0.0%    0.0%        k__Bacteria;p__Actinobacteria;c__Actinobacteria (class); o__Actinomycetales   3006014   17.6%   16.4%   20.6%   24.3%   27.7%    4.4%   27.6%   12.4%   21.2%   17.9%    9.7%    9.9%   16.1%   17.8%   18.4%   16.2%   25.2%   11.1%   12.5%   25.3%   13.4%   19.3%   15.0%   47.4%   40.3%   20.7%   11.4%   17.5%   20.1%   55.8%   44.0%   17.0%    7.6%   16.4%   15.1%   23.0%   27.2%   20.1%   10.5%   10.3%   12.5%   14.8%   18.6%   23.3%   27.5%   17.2%   14.8%   47.9%   19.3%   45.2%   14.2%    8.3%   11.6%   24.9%   10.7%   50.9%   20.8%    4.4%   12.2%   15.8%   11.9%   12.3%   47.9%   12.3%    7.8%   21.5%    2.9%   16.8%    6.3%   15.9%    7.2%   48.3%    3.7%   20.4%   20.4%    2.1%    7.1%   14.2%   25.7%   23.3%   24.0%    9.2%    9.6%   12.6%   46.8%   35.5%   20.8%    4.1%   12.3%   23.3%    1.6%   11.5%   15.6%    5.4%   64.6%   27.3%    9.0%    1.4%   18.3%   22.3%   26.8%   11.3%   15.2%   33.2%   40.0%   19.2%   14.6%    4.7%    1.4%   28.3%   21.8%   14.9%   21.9%    3.1%   14.5%   19.4%   15.8%   24.4%    9.0%   23.2%    0.6%   16.2%   20.0%   10.9%    2.5%   14.4%   21.7%    4.1%   11.6%   16.9%   18.4%    7.0%    7.2%   14.4%   17.7%    4.7%    8.8%   22.8%    1.4%    6.5%    0.5%    8.6%    7.7%    3.7%    9.0%   30.6%   16.0%   13.0%   13.5%    2.0%    5.1%    2.6%   10.8%    9.9%    6.7%    2.4%   14.1%    6.3%    3.1%   21.3%   13.8%   27.4%   13.2%    3.4%   17.4%    9.7%   26.3%   26.6%   23.4%   21.1%   16.1%   21.4%    7.3%   12.2%   12.5%   25.6%   20.0%    3.2%    4.7%   10.8%   27.2%   23.3%    4.7%    6.7%   50.5%   25.8%   32.7%   21.2%   29.3%   26.5%   37.4%   33.2%   35.1%   23.2%   11.5%   11.7%   19.5%   23.4%   23.6%   25.9%   18.8%   10.2%   13.9%   13.3%   25.0%    7.9%    5.3%    9.7%   17.1%   25.7%   16.7%   13.3%    4.8%   16.9%    5.4%   22.7%   10.0%   10.4%   31.0%   36.3%   22.2%   10.5%    0.7%    7.0%   12.1%   19.0%   19.9%   14.4%   14.6%   26.8%   10.2%   14.4%   10.7%   18.8%   14.1%    9.3%   15.9%   13.9%    4.2%   27.3%   18.0%   17.6%   25.6%   37.7%   11.7%   14.4%   15.2%   25.1%   23.2%   33.3%   20.6%   14.6%    7.8%   11.3%    5.0%   14.2%   13.8%    8.1%   15.5%   11.9%   19.6%   11.8%   16.4%   10.6%   20.7%    4.2%   22.1%   13.5%    7.3%   16.1%   36.4%   29.3%   13.6%   23.2%   13.0%   24.8%   19.6%   23.6%   23.5%   18.3%    8.8%    2.0%   12.3%   30.7%   19.0%   16.3%   24.9%   24.2%   24.6%   12.7%   28.6%   51.8%   35.1%   23.2%   15.4%   12.7%   23.1%   33.4%   28.5%   20.9%   39.3%   16.2%    8.8%   28.4%   24.2%   22.4%   23.4%   33.5%   13.9%   13.5%   40.3%   27.6%   39.9%   15.6%   21.5%   25.2%    7.4%   31.0%   24.3%   58.9%   28.2%   21.5%   19.9%   10.7%   21.9%   35.2%   32.7%   23.2%   20.7%   21.8%   15.6%   25.6%   17.8%    8.1%   18.1%   33.7%    9.4%   20.1%   17.1%   13.2%   26.5%   12.9%   25.9%   32.1%   20.4%   24.3%   34.8%   25.3%   24.9%   51.8%   17.5%   19.7%   21.4%   24.2%   26.7%    0.3%    8.1%    9.4%        k__Bacteria;p__Actinobacteria;c__Actinobacteria (class); o__Bifidobacteriales    7525    0.0%    0.1%    0.2%    0.1%    0.3%    0.0%    0.2%    0.1%    0.0%    0.0%    0.0%    0.0%    0.0%    0.3%    0.0%    0.0%    0.1%    0.3%    0.1%    0.0%    0.1%    0.1%    0.0%    0.0%    0.0%    0.0%    0.0%    0.0%    0.1%    0.0%    0.0%    0.1%    0.0%    0.1%    0.1%    0.0%    0.1%    0.0%    0.1%    0.1%    0.1%    0.2%    0.2%    0.1%    0.1%    0.0%    0.0%    0.0%    0.3%    0.1%    0.0%    0.0%    0.1%    0.0%    0.0%    0.0%    0.0%    0.0%    0.1%    0.0%    0.0%    0.0%    0.0%    0.0%    0.0%    0.0%    0.0%    0.5%    0.0%    0.0%    0.0%    0.0%    0.3%    0.1%    0.1%    0.0%    0.0%    0.1%    0.0%    0.0%    0.0%    0.0%    0.0%    0.0%    0.0%    0.0%    0.1%    0.0%    0.0%    0.1%    0.0%    0.1%    0.0%    0.0%    0.0%    0.0%    0.0%    0.0%    0.0%    0.0%    0.0%    0.0%    0.0%    0.1%    0.0%    0.0%    0.1%    0.0%    0.0%    0.0%    0.0%    0.1%    0.0%    0.0%    0.0%    0.0%    0.0%    0.0%    0.0%    0.1%    0.0%    0.0%    0.1%    0.0%    0.0%    0.0%    0.0%    0.0%    0.0%    0.0%    0.0%    0.0%    0.0%    0.0%    0.1%    0.0%    0.0%    0.0%    0.0%    0.0%    0.0%    0.1%    0.0%    0.0%    0.0%    0.0%    0.1%    0.1%    0.0%    0.0%    0.0%    0.0%    0.0%    0.0%    0.0%    0.0%    0.0%    0.0%    0.0%    0.0%    0.0%    0.1%    0.0%    0.0%    0.0%    0.0%    0.0%    0.0%    0.0%    0.0%    0.0%    0.0%    0.0%    0.0%    0.0%    0.0%    0.0%    0.0%    0.0%    0.0%    0.0%    0.0%    0.0%    0.0%    0.0%    0.0%    0.0%    0.0%    0.0%    0.0%    0.0%    0.0%    0.0%    0.0%    0.0%    0.0%    0.0%    0.0%    0.0%    0.0%    0.0%    0.0%    0.0%    0.2%    0.5%    0.1%    0.2%    0.0%    0.1%    0.1%    0.1%    0.0%    0.0%    0.0%    0.0%    0.0%    0.0%    0.0%    0.0%    0.0%    0.1%    0.0%    0.0%    0.0%    0.0%    0.0%    0.0%    0.0%    0.0%    0.0%    0.0%    0.0%    0.0%    0.0%    0.0%    0.0%    0.0%    0.0%    0.0%    0.0%    0.0%    0.0%    0.0%    0.0%    0.0%    0.0%    0.0%    0.0%    0.0%    0.0%    0.0%    0.0%    0.0%    0.0%    0.0%    0.0%    0.0%    0.0%    0.0%    0.0%    0.0%    0.0%    0.0%    0.0%    0.0%    0.0%    0.0%    1.6%    0.1%    0.0%    0.1%    0.1%    0.1%    0.3%    0.0%    0.0%    0.0%    0.0%    0.0%    0.0%    0.0%    0.0%    0.1%    0.1%    0.0%    0.0%    0.0%    0.0%    0.0%    0.0%    0.1%    0.0%    0.0%    0.1%    0.1%    0.0%    0.1%    0.0%    0.0%    0.0%    0.0%    0.0%    0.1%    0.0%    0.1%    0.0%    0.0%    0.0%    0.0%    0.0%    0.0%    0.0%    0.0%    0.0%    0.0%    0.1%    0.0%    0.0%    0.0%    0.0%    0.0%    0.0%    0.0%    0.0%    0.0%    0.0%    0.0%    0.0%    0.0%    0.0%    0.0%    0.0%    0.0%    0.0%    0.0%    0.0%    0.0%    0.0%    0.0%    0.0%    0.0%    0.0%    0.0%    0.1%    0.0%    0.0%    0.0%    0.0%    0.0%    0.0%    0.0%    0.0%    0.0%    0.1%    0.0%    0.0%    0.0%    0.0%        k__Bacteria;p__Actinobacteria;c__Actinobacteria (class); o__Coriobacteriales   12553    0.1%    0.1%    0.3%    0.5%    0.1%    0.0%    0.7%    0.1%    0.1%    0.0%    0.0%    0.0%    0.0%    0.9%    0.2%    0.0%    0.2%    0.2%    0.2%    0.1%    0.1%    0.1%    0.0%    0.1%    0.1%    0.1%    0.0%    0.1%    0.1%    0.0%    0.1%    0.1%    0.0%    0.7%    0.4%    0.6%    0.3%    0.1%    0.2%    0.1%    0.2%    0.2%    0.5%    0.1%    0.1%    0.1%    0.1%    0.1%    0.2%    0.3%    0.1%    0.0%    0.0%    0.0%    0.1%    0.1%    0.1%    0.0%    0.1%    0.0%    0.0%    0.0%    0.1%    0.0%    0.2%    0.0%    0.0%    1.6%    0.0%    0.1%    0.0%    0.0%    0.0%    0.1%    0.1%    0.0%    0.0%    0.0%    0.1%    0.0%    0.1%    0.0%    0.0%    0.1%    0.1%    0.0%    0.0%    0.0%    0.0%    0.0%    0.0%    0.0%    0.0%    0.0%    0.1%    0.0%    0.0%    0.0%    0.1%    0.1%    0.0%    0.0%    0.0%    0.0%    0.0%    0.0%    0.1%    0.0%    0.0%    0.1%    0.0%    0.2%    0.0%    0.0%    0.0%    0.0%    0.0%    0.1%    0.0%    0.0%    0.0%    0.0%    0.1%    0.0%    0.0%    0.0%    0.1%    0.0%    0.0%    0.1%    0.1%    0.0%    0.0%    0.0%    0.1%    0.0%    0.0%    0.1%    0.0%    0.0%    0.0%    0.1%    0.1%    0.0%    0.0%    0.0%    0.6%    0.2%    0.0%    0.0%    0.0%    0.0%    0.0%    0.0%    0.0%    0.0%    0.0%    0.0%    0.0%    0.1%    0.0%    0.0%    0.0%    0.0%    0.0%    0.0%    0.1%    0.0%    0.0%    0.0%    0.1%    0.1%    0.0%    0.0%    0.0%    0.0%    0.0%    0.0%    0.0%    0.0%    0.0%    0.0%    0.0%    0.0%    0.0%    0.0%    0.0%    0.0%    0.0%    0.0%    0.0%    0.1%    0.0%    0.0%    0.0%    0.0%    0.0%    0.1%    0.0%    0.0%    0.0%    0.1%    0.0%    0.1%    0.0%    0.1%    0.0%    0.1%    0.0%    0.0%    0.0%    0.0%    0.0%    0.2%    0.0%    0.0%    0.0%    0.5%    0.0%    0.0%    0.0%    0.0%    0.0%    0.0%    0.0%    0.0%    0.0%    0.0%    0.0%    0.0%    0.0%    0.0%    0.0%    0.0%    0.0%    0.0%    0.0%    0.0%    0.0%    0.0%    0.0%    0.0%    0.0%    0.0%    0.0%    0.0%    0.0%    0.0%    0.0%    0.0%    0.0%    0.0%    0.0%    0.0%    0.0%    0.0%    0.0%    0.0%    0.0%    0.0%    0.0%    0.0%    0.0%    0.0%    0.0%    0.0%    0.0%    1.0%    0.1%    0.0%    0.1%    0.1%    0.0%    0.2%    0.0%    0.0%    0.0%    0.0%    0.0%    0.0%    0.0%    0.0%    0.0%    0.0%    0.0%    0.0%    0.0%    0.1%    0.0%    0.0%    0.1%    0.0%    0.0%    0.0%    0.0%    0.0%    0.2%    0.0%    0.1%    0.0%    0.0%    0.1%    0.2%    0.0%    0.1%    0.1%    0.2%    0.0%    0.0%    0.1%    0.0%    0.0%    0.0%    0.0%    0.1%    0.0%    0.0%    0.0%    0.0%    0.0%    0.0%    0.0%    0.0%    0.0%    0.0%    0.0%    0.0%    0.0%    0.0%    0.0%    0.1%    0.0%    0.0%    0.0%    0.0%    0.0%    0.0%    0.1%    0.1%    0.0%    0.0%    0.0%    0.0%    0.1%    0.0%    0.0%    0.0%    0.0%    0.0%    0.0%    0.0%    0.0%    0.0%    0.1%    0.0%    0.0%    0.0%    0.0%        k__Bacteria;p__Actinobacteria;c__Actinobacteria (class); o__Euzebiales      84    0.0%    0.0%    0.0%    0.0%    0.0%    0.0%    0.0%    0.0%    0.0%    0.0%    0.0%    0.0%    0.0%    0.0%    0.0%    0.0%    0.0%    0.0%    0.0%    0.0%    0.0%    0.0%    0.0%    0.0%    0.0%    0.0%    0.0%    0.0%    0.0%    0.0%    0.0%    0.0%    0.0%    0.0%    0.0%    0.0%    0.0%    0.0%    0.0%    0.0%    0.0%    0.0%    0.0%    0.0%    0.0%    0.0%    0.0%    0.0%    0.0%    0.0%    0.0%    0.0%    0.0%    0.0%    0.0%    0.0%    0.0%    0.0%    0.0%    0.0%    0.0%    0.0%    0.0%    0.0%    0.0%    0.0%    0.0%    0.0%    0.0%    0.0%    0.0%    0.0%    0.0%    0.0%    0.0%    0.0%    0.0%    0.0%    0.0%    0.0%    0.0%    0.0%    0.0%    0.0%    0.0%    0.0%    0.0%    0.0%    0.0%    0.0%    0.0%    0.0%    0.0%    0.0%    0.0%    0.0%    0.0%    0.0%    0.0%    0.0%    0.0%    0.0%    0.0%    0.0%    0.0%    0.0%    0.0%    0.0%    0.0%    0.0%    0.0%    0.0%    0.0%    0.0%    0.0%    0.0%    0.0%    0.0%    0.0%    0.0%    0.0%    0.0%    0.0%    0.0%    0.0%    0.0%    0.0%    0.0%    0.0%    0.0%    0.0%    0.0%    0.0%    0.0%    0.0%    0.0%    0.0%    0.0%    0.0%    0.0%    0.0%    0.0%    0.0%    0.0%    0.0%    0.0%    0.0%    0.0%    0.0%    0.0%    0.0%    0.0%    0.0%    0.0%    0.0%    0.0%    0.0%    0.0%    0.0%    0.0%    0.0%    0.0%    0.0%    0.0%    0.0%    0.0%    0.0%    0.0%    0.0%    0.0%    0.0%    0.0%    0.0%    0.0%    0.0%    0.0%    0.0%    0.0%    0.0%    0.0%    0.0%    0.0%    0.0%    0.0%    0.0%    0.0%    0.0%    0.0%    0.0%    0.0%    0.0%    0.0%    0.0%    0.0%    0.0%    0.0%    0.0%    0.0%    0.0%    0.0%    0.0%    0.0%    0.0%    0.0%    0.0%    0.0%    0.0%    0.0%    0.0%    0.0%    0.0%    0.0%    0.0%    0.0%    0.0%    0.0%    0.0%    0.0%    0.0%    0.0%    0.0%    0.0%    0.0%    0.0%    0.0%    0.0%    0.0%    0.0%    0.0%    0.0%    0.0%    0.0%    0.0%    0.0%    0.0%    0.0%    0.0%    0.0%    0.0%    0.0%    0.0%    0.0%    0.0%    0.0%    0.0%    0.0%    0.0%    0.0%    0.0%    0.0%    0.0%    0.0%    0.0%    0.0%    0.0%    0.0%    0.0%    0.0%    0.0%    0.0%    0.0%    0.0%    0.0%    0.0%    0.0%    0.0%    0.0%    0.0%    0.0%    0.0%    0.0%    0.0%    0.0%    0.0%    0.0%    0.0%    0.0%    0.0%    0.0%    0.0%    0.0%    0.0%    0.0%    0.0%    0.0%    0.0%    0.0%    0.0%    0.0%    0.0%    0.0%    0.0%    0.0%    0.0%    0.0%    0.0%    0.0%    0.0%    0.0%    0.0%    0.0%    0.0%    0.0%    0.0%    0.0%    0.0%    0.0%    0.0%    0.0%    0.0%    0.0%    0.0%    0.0%    0.0%    0.0%    0.0%    0.0%    0.0%    0.0%    0.0%    0.0%    0.0%    0.0%    0.0%    0.0%    0.0%    0.0%    0.0%    0.0%    0.0%    0.0%    0.0%    0.0%    0.0%    0.0%    0.0%    0.0%    0.0%    0.0%    0.0%    0.0%    0.0%    0.0%    0.0%    0.0%    0.0%    0.0%    0.0%    0.0%    0.0%    0.0%    0.0%    0.0%    0.0%    0.0%    0.0%    0.0%    0.0%        k__Bacteria;p__Actinobacteria;c__Actinobacteria (class); o__MC47   10374    0.1%    0.0%    0.1%    0.0%    0.0%    0.0%    0.0%    0.0%    0.0%    0.0%    0.0%    0.0%    0.0%    0.0%    0.0%    0.0%    0.0%    0.0%    0.0%    0.0%    0.0%    0.0%    0.0%    0.0%    0.0%    0.0%    0.0%    0.0%    0.0%    0.0%    0.0%    0.1%    0.0%    0.0%    0.1%    0.0%    0.1%    0.0%    0.0%    0.1%    0.0%    0.1%    0.0%    0.0%    0.1%    0.0%    0.1%    0.0%    0.0%    0.0%    0.0%    0.0%    0.0%    0.0%    0.1%    0.0%    0.0%    0.0%    0.1%    0.2%    0.1%    0.1%    0.0%    0.2%    0.1%    0.0%    0.0%    0.3%    0.1%    0.1%    0.0%    0.1%    0.1%    0.1%    0.1%    0.0%    0.0%    0.1%    0.0%    0.1%    0.0%    0.0%    0.0%    0.0%    0.0%    0.0%    0.1%    0.0%    0.0%    0.1%    0.0%    0.0%    0.1%    0.0%    0.1%    0.1%    0.2%    0.0%    0.1%    0.1%    0.2%    0.0%    0.0%    0.1%    0.0%    0.0%    0.2%    0.0%    0.0%    0.1%    0.1%    0.2%    0.0%    0.0%    0.0%    0.0%    0.0%    0.1%    0.1%    0.3%    0.0%    0.0%    0.2%    0.1%    0.0%    0.2%    0.2%    0.0%    0.2%    0.5%    0.2%    0.1%    0.1%    0.1%    0.3%    0.1%    0.1%    0.1%    0.0%    0.0%    0.0%    0.1%    0.1%    0.0%    0.2%    0.1%    0.0%    0.0%    0.0%    0.0%    0.0%    0.1%    0.2%    0.1%    0.0%    0.0%    0.2%    0.0%    0.0%    0.3%    0.7%    0.2%    0.1%    0.1%    0.1%    0.1%    0.2%    0.0%    0.1%    0.0%    0.2%    0.2%    0.1%    0.1%    0.0%    0.0%    0.0%    0.0%    0.0%    0.0%    0.0%    0.0%    0.0%    0.0%    0.0%    0.0%    0.1%    0.0%    0.0%    0.0%    0.0%    0.0%    0.0%    0.0%    0.0%    0.1%    0.2%    0.0%    0.0%    0.0%    0.1%    0.1%    0.1%    0.3%    0.1%    0.0%    0.0%    0.1%    0.3%    0.2%    0.1%    0.0%    0.1%    0.0%    0.0%    0.0%    0.0%    0.0%    0.0%    0.0%    0.0%    0.1%    0.0%    0.1%    0.0%    0.1%    0.0%    0.0%    0.1%    0.0%    0.0%    0.0%    0.1%    0.0%    0.2%    0.0%    0.2%    0.0%    0.0%    0.2%    0.1%    0.0%    0.0%    0.0%    0.0%    0.0%    0.1%    0.0%    0.0%    0.1%    0.2%    0.1%    0.0%    0.0%    0.0%    0.0%    0.0%    0.0%    0.0%    0.0%    0.1%    0.2%    0.1%    0.1%    0.1%    0.0%    0.1%    0.1%    0.0%    0.0%    0.0%    0.1%    0.1%    0.0%    0.0%    0.1%    0.0%    0.0%    0.0%    0.0%    0.0%    0.0%    0.0%    0.0%    0.0%    0.0%    0.1%    0.4%    0.1%    0.0%    0.0%    0.0%    0.1%    0.1%    0.0%    0.0%    0.0%    0.0%    0.1%    0.0%    0.0%    0.0%    0.0%    0.1%    0.0%    0.0%    0.0%    0.0%    0.0%    0.0%    0.0%    0.0%    0.0%    0.0%    0.0%    0.0%    0.0%    0.0%    0.0%    0.0%    0.0%    0.0%    0.0%    0.0%    0.0%    0.0%    0.0%    0.0%    0.0%    0.0%    0.0%    0.0%    0.0%    0.1%    0.0%    0.0%    0.0%    0.0%    0.0%    0.0%    0.0%    0.0%    0.0%    0.0%    0.0%    0.0%    0.0%    0.0%    0.0%    0.0%    0.0%    0.0%    0.0%    0.0%    0.0%    0.0%    0.0%    0.0%        k__Bacteria;p__Actinobacteria;c__Actinobacteria (class); o__Rubrobacterales    2564    0.0%    0.0%    0.0%    0.0%    0.0%    0.0%    0.0%    0.0%    0.0%    0.0%    0.0%    0.0%    0.0%    0.0%    0.0%    0.0%    0.0%    0.0%    0.0%    0.0%    0.0%    0.0%    0.0%    0.0%    0.0%    0.0%    0.0%    0.0%    0.0%    0.0%    0.0%    0.0%    0.0%    0.0%    0.0%    0.0%    0.0%    0.0%    0.0%    0.0%    0.0%    0.0%    0.0%    0.0%    0.0%    0.0%    0.0%    0.0%    0.0%    0.0%    0.0%    0.0%    0.0%    0.0%    0.0%    0.0%    0.0%    0.0%    0.0%    0.0%    0.0%    0.0%    0.0%    0.0%    0.0%    0.1%    0.0%    0.0%    0.0%    0.0%    0.0%    0.0%    0.0%    0.0%    0.0%    0.0%    0.0%    0.0%    0.0%    0.0%    0.0%    0.0%    0.0%    0.0%    0.0%    0.0%    0.0%    0.0%    0.0%    0.0%    0.0%    0.0%    0.0%    0.0%    0.0%    0.1%    0.0%    0.0%    0.0%    0.0%    0.1%    0.0%    0.0%    0.0%    0.0%    0.0%    0.1%    0.0%    0.0%    0.0%    0.0%    0.1%    0.0%    0.0%    0.0%    0.0%    0.0%    0.0%    0.0%    0.1%    0.0%    0.0%    0.1%    0.0%    0.0%    0.0%    0.0%    0.0%    0.0%    0.1%    0.1%    0.0%    0.0%    0.0%    0.1%    0.0%    0.0%    0.0%    0.0%    0.0%    0.0%    0.0%    0.0%    0.0%    0.1%    0.0%    0.0%    0.0%    0.0%    0.0%    0.0%    0.0%    0.0%    0.0%    0.0%    0.0%    0.0%    0.0%    0.0%    0.1%    0.1%    0.1%    0.0%    0.0%    0.0%    0.0%    0.0%    0.0%    0.0%    0.0%    0.1%    0.0%    0.0%    0.0%    0.0%    0.0%    0.0%    0.0%    0.0%    0.0%    0.0%    0.0%    0.0%    0.0%    0.0%    0.0%    0.0%    0.0%    0.0%    0.0%    0.0%    0.0%    0.0%    0.0%    0.0%    0.0%    0.0%    0.0%    0.0%    0.0%    0.0%    0.0%    0.0%    0.0%    0.0%    0.0%    0.0%    0.0%    0.1%    0.0%    0.0%    0.0%    0.0%    0.0%    0.0%    0.0%    0.0%    0.0%    0.0%    0.0%    0.0%    0.0%    0.0%    0.0%    0.0%    0.0%    0.0%    0.0%    0.0%    0.0%    0.0%    0.0%    0.0%    0.0%    0.0%    0.0%    0.1%    0.1%    0.0%    0.0%    0.0%    0.0%    0.0%    0.0%    0.0%    0.0%    0.0%    0.0%    0.0%    0.0%    0.1%    0.0%    0.0%    0.0%    0.0%    0.0%    0.0%    0.0%    0.0%    0.0%    0.0%    0.0%    0.0%    0.0%    0.0%    0.0%    0.0%    0.0%    0.0%    0.0%    0.0%    0.0%    0.0%    0.0%    0.0%    0.0%    0.0%    0.0%    0.0%    0.0%    0.0%    0.0%    0.0%    0.0%    0.0%    0.0%    0.0%    0.0%    0.0%    0.0%    0.0%    0.0%    0.1%    0.0%    0.0%    0.0%    0.0%    0.0%    0.0%    0.0%    0.0%    0.0%    0.0%    0.1%    0.0%    0.0%    0.0%    0.0%    0.0%    0.0%    0.0%    0.0%    0.0%    0.0%    0.0%    0.0%    0.0%    0.0%    0.0%    0.0%    0.0%    0.0%    0.0%    0.0%    0.0%    0.0%    0.0%    0.0%    0.0%    0.0%    0.0%    0.0%    0.0%    0.0%    0.0%    0.0%    0.0%    0.0%    0.0%    0.0%    0.0%    0.0%    0.0%    0.0%    0.0%    0.0%    0.0%    0.0%    0.0%    0.0%    0.0%    0.0%    0.0%    0.1%    0.0%    0.0%    0.0%    0.0%        k__Bacteria;p__Actinobacteria;c__Actinobacteria (class); o__Solirubrobacterales   23928    0.1%    0.0%    0.2%    0.1%    0.2%    0.0%    0.0%    0.0%    0.0%    0.0%    0.0%    0.0%    0.1%    0.0%    0.0%    0.1%    0.1%    0.2%    0.0%    0.0%    0.1%    0.2%    0.0%    0.0%    0.0%    0.0%    0.1%    0.1%    0.1%    0.0%    0.0%    0.2%    0.0%    0.0%    0.2%    0.0%    0.1%    0.0%    0.1%    0.3%    0.1%    0.1%    0.0%    0.0%    0.2%    0.0%    0.1%    0.0%    0.0%    0.0%    0.1%    0.0%    0.1%    0.0%    0.1%    0.1%    0.2%    0.1%    0.2%    0.3%    0.2%    0.1%    0.0%    0.3%    0.3%    0.1%    0.0%    0.4%    0.1%    0.3%    0.1%    0.1%    0.1%    0.1%    0.3%    0.0%    0.1%    0.3%    0.0%    0.2%    0.1%    0.1%    0.0%    0.1%    0.0%    0.0%    0.2%    0.0%    0.0%    0.3%    0.0%    0.1%    0.2%    0.1%    0.1%    0.1%    0.4%    0.0%    0.2%    0.2%    0.4%    0.1%    0.0%    0.1%    0.0%    0.0%    0.6%    0.0%    0.0%    0.2%    0.2%    0.4%    0.0%    0.0%    0.2%    0.1%    0.0%    0.1%    0.1%    1.1%    0.0%    0.2%    0.5%    0.2%    0.0%    0.5%    0.5%    0.0%    0.5%    1.0%    0.3%    0.2%    0.3%    0.1%    0.7%    0.1%    0.2%    0.1%    0.0%    0.1%    0.0%    0.1%    0.2%    0.1%    0.5%    0.1%    0.0%    0.0%    0.1%    0.0%    0.0%    0.1%    0.4%    0.1%    0.0%    0.0%    0.4%    0.1%    0.0%    0.6%    1.1%    0.4%    0.3%    0.1%    0.2%    0.1%    0.3%    0.1%    0.2%    0.1%    0.4%    0.3%    0.3%    0.2%    0.1%    0.0%    0.1%    0.1%    0.0%    0.0%    0.0%    0.0%    0.1%    0.1%    0.1%    0.0%    0.1%    0.0%    0.1%    0.0%    0.0%    0.1%    0.0%    0.0%    0.1%    0.1%    0.3%    0.0%    0.1%    0.0%    0.1%    0.1%    0.2%    0.6%    0.2%    0.1%    0.1%    0.2%    0.6%    0.4%    0.3%    0.0%    0.5%    0.1%    0.0%    0.1%    0.0%    0.1%    0.0%    0.0%    0.3%    0.2%    0.0%    0.2%    0.0%    0.2%    0.0%    0.1%    0.3%    0.3%    0.0%    0.0%    0.4%    0.1%    0.5%    0.0%    0.4%    0.2%    0.0%    0.5%    0.2%    0.3%    0.0%    0.0%    0.0%    0.0%    0.1%    0.0%    0.1%    0.1%    0.4%    0.2%    0.1%    0.0%    0.0%    0.0%    0.1%    0.0%    0.1%    0.1%    0.3%    0.2%    0.3%    0.2%    0.1%    0.0%    0.1%    0.2%    0.1%    0.2%    0.1%    0.1%    0.4%    0.2%    0.1%    0.1%    0.0%    0.1%    0.1%    0.0%    0.1%    0.0%    0.0%    0.0%    0.1%    0.0%    0.0%    0.8%    0.3%    1.0%    0.2%    0.0%    0.2%    0.2%    0.1%    0.0%    0.1%    0.1%    0.1%    0.2%    0.2%    0.0%    0.0%    0.1%    0.1%    0.0%    0.1%    0.0%    0.0%    0.0%    0.0%    0.0%    0.1%    0.0%    0.3%    0.2%    0.0%    0.0%    0.0%    0.0%    0.0%    0.1%    0.0%    0.0%    0.2%    0.0%    0.0%    0.1%    0.1%    0.1%    0.0%    0.0%    0.0%    0.0%    0.0%    0.0%    0.0%    0.0%    0.0%    0.1%    0.0%    0.0%    0.0%    0.1%    0.3%    0.0%    0.0%    0.0%    0.0%    0.0%    0.1%    0.0%    0.0%    0.1%    0.0%    0.0%    0.5%    0.0%        k__Bacteria;p__Actinobacteria;c__Actinobacteria (class); o__WCHB1-81       2    0.0%    0.0%    0.0%    0.0%    0.0%    0.0%    0.0%    0.0%    0.0%    0.0%    0.0%    0.0%    0.0%    0.0%    0.0%    0.0%    0.0%    0.0%    0.0%    0.0%    0.0%    0.0%    0.0%    0.0%    0.0%    0.0%    0.0%    0.0%    0.0%    0.0%    0.0%    0.0%    0.0%    0.0%    0.0%    0.0%    0.0%    0.0%    0.0%    0.0%    0.0%    0.0%    0.0%    0.0%    0.0%    0.0%    0.0%    0.0%    0.0%    0.0%    0.0%    0.0%    0.0%    0.0%    0.0%    0.0%    0.0%    0.0%    0.0%    0.0%    0.0%    0.0%    0.0%    0.0%    0.0%    0.0%    0.0%    0.0%    0.0%    0.0%    0.0%    0.0%    0.0%    0.0%    0.0%    0.0%    0.0%    0.0%    0.0%    0.0%    0.0%    0.0%    0.0%    0.0%    0.0%    0.0%    0.0%    0.0%    0.0%    0.0%    0.0%    0.0%    0.0%    0.0%    0.0%    0.0%    0.0%    0.0%    0.0%    0.0%    0.0%    0.0%    0.0%    0.0%    0.0%    0.0%    0.0%    0.0%    0.0%    0.0%    0.0%    0.0%    0.0%    0.0%    0.0%    0.0%    0.0%    0.0%    0.0%    0.0%    0.0%    0.0%    0.0%    0.0%    0.0%    0.0%    0.0%    0.0%    0.0%    0.0%    0.0%    0.0%    0.0%    0.0%    0.0%    0.0%    0.0%    0.0%    0.0%    0.0%    0.0%    0.0%    0.0%    0.0%    0.0%    0.0%    0.0%    0.0%    0.0%    0.0%    0.0%    0.0%    0.0%    0.0%    0.0%    0.0%    0.0%    0.0%    0.0%    0.0%    0.0%    0.0%    0.0%    0.0%    0.0%    0.0%    0.0%    0.0%    0.0%    0.0%    0.0%    0.0%    0.0%    0.0%    0.0%    0.0%    0.0%    0.0%    0.0%    0.0%    0.0%    0.0%    0.0%    0.0%    0.0%    0.0%    0.0%    0.0%    0.0%    0.0%    0.0%    0.0%    0.0%    0.0%    0.0%    0.0%    0.0%    0.0%    0.0%    0.0%    0.0%    0.0%    0.0%    0.0%    0.0%    0.0%    0.0%    0.0%    0.0%    0.0%    0.0%    0.0%    0.0%    0.0%    0.0%    0.0%    0.0%    0.0%    0.0%    0.0%    0.0%    0.0%    0.0%    0.0%    0.0%    0.0%    0.0%    0.0%    0.0%    0.0%    0.0%    0.0%    0.0%    0.0%    0.0%    0.0%    0.0%    0.0%    0.0%    0.0%    0.0%    0.0%    0.0%    0.0%    0.0%    0.0%    0.0%    0.0%    0.0%    0.0%    0.0%    0.0%    0.0%    0.0%    0.0%    0.0%    0.0%    0.0%    0.0%    0.0%    0.0%    0.0%    0.0%    0.0%    0.0%    0.0%    0.0%    0.0%    0.0%    0.0%    0.0%    0.0%    0.0%    0.0%    0.0%    0.0%    0.0%    0.0%    0.0%    0.0%    0.0%    0.0%    0.0%    0.0%    0.0%    0.0%    0.0%    0.0%    0.0%    0.0%    0.0%    0.0%    0.0%    0.0%    0.0%    0.0%    0.0%    0.0%    0.0%    0.0%    0.0%    0.0%    0.0%    0.0%    0.0%    0.0%    0.0%    0.0%    0.0%    0.0%    0.0%    0.0%    0.0%    0.0%    0.0%    0.0%    0.0%    0.0%    0.0%    0.0%    0.0%    0.0%    0.0%    0.0%    0.0%    0.0%    0.0%    0.0%    0.0%    0.0%    0.0%    0.0%    0.0%    0.0%    0.0%    0.0%    0.0%    0.0%    0.0%    0.0%    0.0%    0.0%    0.0%    0.0%    0.0%    0.0%    0.0%    0.0%    0.0%    0.0%    0.0%    0.0%    0.0%    0.0%    0.0%    0.0%    0.0%    0.0%        k__Bacteria;p__Actinobacteria;c__Actinobacteria (class); o__koll13    1786    0.0%    0.0%    0.0%    0.0%    0.0%    0.0%    0.0%    0.0%    0.0%    0.0%    0.0%    0.0%    0.0%    0.0%    0.0%    0.0%    0.0%    0.0%    0.0%    0.0%    0.0%    0.0%    0.0%    0.0%    0.0%    0.0%    0.0%    0.0%    0.0%    0.0%    0.0%    0.0%    0.0%    0.0%    0.0%    0.0%    0.0%    0.0%    0.0%    0.0%    0.0%    0.0%    0.0%    0.0%    0.0%    0.0%    0.0%    0.0%    0.0%    0.0%    0.0%    0.0%    0.0%    0.0%    0.0%    0.0%    0.0%    0.0%    0.0%    0.1%    0.0%    0.0%    0.0%    0.1%    0.0%    0.0%    0.0%    0.1%    0.0%    0.0%    0.0%    0.0%    0.0%    0.0%    0.0%    0.0%    0.0%    0.0%    0.0%    0.0%    0.0%    0.0%    0.0%    0.0%    0.0%    0.0%    0.1%    0.0%    0.0%    0.0%    0.0%    0.0%    0.0%    0.0%    0.0%    0.0%    0.1%    0.0%    0.0%    0.0%    0.0%    0.0%    0.0%    0.0%    0.0%    0.0%    0.0%    0.0%    0.0%    0.0%    0.0%    0.0%    0.0%    0.0%    0.0%    0.0%    0.0%    0.0%    0.0%    0.1%    0.0%    0.0%    0.0%    0.0%    0.0%    0.0%    0.0%    0.0%    0.0%    0.1%    0.0%    0.0%    0.0%    0.0%    0.1%    0.0%    0.0%    0.0%    0.0%    0.0%    0.0%    0.0%    0.0%    0.0%    0.0%    0.0%    0.0%    0.0%    0.0%    0.0%    0.0%    0.0%    0.0%    0.0%    0.0%    0.0%    0.0%    0.0%    0.0%    0.0%    0.1%    0.0%    0.0%    0.0%    0.0%    0.0%    0.0%    0.0%    0.0%    0.0%    0.0%    0.0%    0.0%    0.0%    0.0%    0.0%    0.0%    0.0%    0.0%    0.0%    0.0%    0.0%    0.0%    0.0%    0.0%    0.0%    0.0%    0.0%    0.0%    0.0%    0.0%    0.0%    0.0%    0.0%    0.0%    0.0%    0.0%    0.0%    0.0%    0.0%    0.0%    0.0%    0.0%    0.0%    0.0%    0.0%    0.0%    0.0%    0.0%    0.0%    0.0%    0.0%    0.0%    0.0%    0.0%    0.1%    0.0%    0.0%    0.0%    0.0%    0.0%    0.0%    0.0%    0.0%    0.0%    0.0%    0.0%    0.0%    0.0%    0.0%    0.0%    0.0%    0.0%    0.0%    0.1%    0.0%    0.0%    0.0%    0.0%    0.0%    0.0%    0.0%    0.0%    0.0%    0.0%    0.0%    0.0%    0.0%    0.0%    0.0%    0.0%    0.0%    0.0%    0.0%    0.0%    0.0%    0.0%    0.0%    0.0%    0.0%    0.1%    0.0%    0.0%    0.0%    0.0%    0.0%    0.0%    0.0%    0.0%    0.0%    0.0%    0.0%    0.0%    0.0%    0.0%    0.0%    0.0%    0.0%    0.0%    0.0%    0.0%    0.0%    0.0%    0.0%    0.0%    0.0%    0.0%    0.0%    0.0%    0.0%    0.0%    0.0%    0.0%    0.0%    0.0%    0.0%    0.0%    0.0%    0.0%    0.0%    0.0%    0.0%    0.0%    0.0%    0.0%    0.0%    0.0%    0.0%    0.0%    0.0%    0.0%    0.0%    0.0%    0.0%    0.0%    0.0%    0.0%    0.0%    0.0%    0.0%    0.0%    0.0%    0.0%    0.0%    0.0%    0.0%    0.0%    0.0%    0.0%    0.0%    0.0%    0.0%    0.0%    0.0%    0.0%    0.0%    0.0%    0.0%    0.0%    0.0%    0.0%    0.0%    0.0%    0.0%    0.0%    0.0%    0.0%    0.0%    0.0%    0.0%    0.0%    0.0%    0.0%    0.0%    0.0%    0.0%    0.0%    0.0%        k__Bacteria;p__Aquificae;c__Aquificae (class); o__Aquificales      31    0.0%    0.0%    0.0%    0.0%    0.0%    0.0%    0.0%    0.0%    0.0%    0.0%    0.0%    0.0%    0.0%    0.0%    0.0%    0.0%    0.0%    0.0%    0.0%    0.0%    0.0%    0.0%    0.0%    0.0%    0.0%    0.0%    0.0%    0.0%    0.0%    0.0%    0.0%    0.0%    0.0%    0.0%    0.0%    0.0%    0.0%    0.0%    0.0%    0.0%    0.0%    0.0%    0.0%    0.0%    0.0%    0.0%    0.0%    0.0%    0.0%    0.0%    0.0%    0.0%    0.0%    0.0%    0.0%    0.0%    0.0%    0.0%    0.0%    0.0%    0.0%    0.0%    0.0%    0.0%    0.0%    0.0%    0.0%    0.0%    0.0%    0.0%    0.0%    0.0%    0.0%    0.0%    0.0%    0.0%    0.0%    0.0%    0.0%    0.0%    0.0%    0.0%    0.0%    0.0%    0.0%    0.0%    0.0%    0.0%    0.0%    0.0%    0.0%    0.0%    0.0%    0.0%    0.0%    0.0%    0.0%    0.0%    0.0%    0.0%    0.0%    0.0%    0.0%    0.0%    0.0%    0.0%    0.0%    0.0%    0.0%    0.0%    0.0%    0.0%    0.0%    0.0%    0.0%    0.0%    0.0%    0.0%    0.0%    0.0%    0.0%    0.0%    0.0%    0.0%    0.0%    0.0%    0.0%    0.0%    0.0%    0.0%    0.0%    0.0%    0.0%    0.0%    0.0%    0.0%    0.0%    0.0%    0.0%    0.0%    0.0%    0.0%    0.0%    0.0%    0.0%    0.0%    0.0%    0.0%    0.0%    0.0%    0.0%    0.0%    0.0%    0.0%    0.0%    0.0%    0.0%    0.0%    0.0%    0.0%    0.0%    0.0%    0.0%    0.0%    0.0%    0.0%    0.0%    0.0%    0.0%    0.0%    0.0%    0.0%    0.0%    0.0%    0.0%    0.0%    0.0%    0.0%    0.0%    0.0%    0.0%    0.0%    0.0%    0.0%    0.0%    0.0%    0.0%    0.0%    0.0%    0.0%    0.0%    0.0%    0.0%    0.0%    0.0%    0.0%    0.0%    0.0%    0.0%    0.0%    0.0%    0.0%    0.0%    0.0%    0.0%    0.0%    0.0%    0.0%    0.0%    0.0%    0.0%    0.0%    0.0%    0.0%    0.0%    0.0%    0.0%    0.0%    0.0%    0.0%    0.0%    0.0%    0.0%    0.0%    0.0%    0.0%    0.0%    0.0%    0.0%    0.0%    0.0%    0.0%    0.0%    0.0%    0.0%    0.0%    0.0%    0.0%    0.0%    0.0%    0.0%    0.0%    0.0%    0.0%    0.0%    0.0%    0.0%    0.0%    0.0%    0.0%    0.0%    0.0%    0.0%    0.0%    0.0%    0.0%    0.0%    0.0%    0.0%    0.0%    0.0%    0.0%    0.0%    0.0%    0.0%    0.0%    0.0%    0.0%    0.0%    0.0%    0.0%    0.0%    0.0%    0.0%    0.0%    0.0%    0.0%    0.0%    0.0%    0.0%    0.0%    0.0%    0.0%    0.0%    0.0%    0.0%    0.0%    0.0%    0.0%    0.0%    0.0%    0.0%    0.0%    0.0%    0.0%    0.0%    0.0%    0.0%    0.0%    0.0%    0.0%    0.0%    0.0%    0.0%    0.0%    0.0%    0.0%    0.0%    0.0%    0.0%    0.0%    0.0%    0.0%    0.0%    0.0%    0.0%    0.0%    0.0%    0.0%    0.0%    0.0%    0.0%    0.0%    0.0%    0.0%    0.0%    0.0%    0.0%    0.0%    0.0%    0.0%    0.0%    0.0%    0.0%    0.0%    0.0%    0.0%    0.0%    0.0%    0.0%    0.0%    0.0%    0.0%    0.0%    0.0%    0.0%    0.0%    0.0%    0.0%    0.0%    0.0%    0.0%    0.0%    0.0%    0.0%    0.0%    0.0%    0.0%        k__Bacteria;p__BRC1;c__; o__       0    0.0%    0.0%    0.0%    0.0%    0.0%    0.0%    0.0%    0.0%    0.0%    0.0%    0.0%    0.0%    0.0%    0.0%    0.0%    0.0%    0.0%    0.0%    0.0%    0.0%    0.0%    0.0%    0.0%    0.0%    0.0%    0.0%    0.0%    0.0%    0.0%    0.0%    0.0%    0.0%    0.0%    0.0%    0.0%    0.0%    0.0%    0.0%    0.0%    0.0%    0.0%    0.0%    0.0%    0.0%    0.0%    0.0%    0.0%    0.0%    0.0%    0.0%    0.0%    0.0%    0.0%    0.0%    0.0%    0.0%    0.0%    0.0%    0.0%    0.0%    0.0%    0.0%    0.0%    0.0%    0.0%    0.0%    0.0%    0.0%    0.0%    0.0%    0.0%    0.0%    0.0%    0.0%    0.0%    0.0%    0.0%    0.0%    0.0%    0.0%    0.0%    0.0%    0.0%    0.0%    0.0%    0.0%    0.0%    0.0%    0.0%    0.0%    0.0%    0.0%    0.0%    0.0%    0.0%    0.0%    0.0%    0.0%    0.0%    0.0%    0.0%    0.0%    0.0%    0.0%    0.0%    0.0%    0.0%    0.0%    0.0%    0.0%    0.0%    0.0%    0.0%    0.0%    0.0%    0.0%    0.0%    0.0%    0.0%    0.0%    0.0%    0.0%    0.0%    0.0%    0.0%    0.0%    0.0%    0.0%    0.0%    0.0%    0.0%    0.0%    0.0%    0.0%    0.0%    0.0%    0.0%    0.0%    0.0%    0.0%    0.0%    0.0%    0.0%    0.0%    0.0%    0.0%    0.0%    0.0%    0.0%    0.0%    0.0%    0.0%    0.0%    0.0%    0.0%    0.0%    0.0%    0.0%    0.0%    0.0%    0.0%    0.0%    0.0%    0.0%    0.0%    0.0%    0.0%    0.0%    0.0%    0.0%    0.0%    0.0%    0.0%    0.0%    0.0%    0.0%    0.0%    0.0%    0.0%    0.0%    0.0%    0.0%    0.0%    0.0%    0.0%    0.0%    0.0%    0.0%    0.0%    0.0%    0.0%    0.0%    0.0%    0.0%    0.0%    0.0%    0.0%    0.0%    0.0%    0.0%    0.0%    0.0%    0.0%    0.0%    0.0%    0.0%    0.0%    0.0%    0.0%    0.0%    0.0%    0.0%    0.0%    0.0%    0.0%    0.0%    0.0%    0.0%    0.0%    0.0%    0.0%    0.0%    0.0%    0.0%    0.0%    0.0%    0.0%    0.0%    0.0%    0.0%    0.0%    0.0%    0.0%    0.0%    0.0%    0.0%    0.0%    0.0%    0.0%    0.0%    0.0%    0.0%    0.0%    0.0%    0.0%    0.0%    0.0%    0.0%    0.0%    0.0%    0.0%    0.0%    0.0%    0.0%    0.0%    0.0%    0.0%    0.0%    0.0%    0.0%    0.0%    0.0%    0.0%    0.0%    0.0%    0.0%    0.0%    0.0%    0.0%    0.0%    0.0%    0.0%    0.0%    0.0%    0.0%    0.0%    0.0%    0.0%    0.0%    0.0%    0.0%    0.0%    0.0%    0.0%    0.0%    0.0%    0.0%    0.0%    0.0%    0.0%    0.0%    0.0%    0.0%    0.0%    0.0%    0.0%    0.0%    0.0%    0.0%    0.0%    0.0%    0.0%    0.0%    0.0%    0.0%    0.0%    0.0%    0.0%    0.0%    0.0%    0.0%    0.0%    0.0%    0.0%    0.0%    0.0%    0.0%    0.0%    0.0%    0.0%    0.0%    0.0%    0.0%    0.0%    0.0%    0.0%    0.0%    0.0%    0.0%    0.0%    0.0%    0.0%    0.0%    0.0%    0.0%    0.0%    0.0%    0.0%    0.0%    0.0%    0.0%    0.0%    0.0%    0.0%    0.0%    0.0%    0.0%    0.0%    0.0%    0.0%    0.0%    0.0%    0.0%    0.0%    0.0%    0.0%    0.0%    0.0%        k__Bacteria;p__BRC1;c__PRR-11; o__     265    0.0%    0.0%    0.0%    0.0%    0.0%    0.0%    0.0%    0.0%    0.0%    0.0%    0.0%    0.0%    0.0%    0.0%    0.0%    0.0%    0.0%    0.0%    0.0%    0.0%    0.0%    0.0%    0.0%    0.0%    0.0%    0.0%    0.0%    0.0%    0.0%    0.0%    0.0%    0.0%    0.0%    0.0%    0.0%    0.0%    0.0%    0.0%    0.0%    0.0%    0.0%    0.0%    0.0%    0.0%    0.0%    0.0%    0.0%    0.0%    0.0%    0.0%    0.0%    0.0%    0.0%    0.0%    0.0%    0.0%    0.0%    0.0%    0.0%    0.0%    0.0%    0.0%    0.0%    0.0%    0.0%    0.0%    0.0%    0.0%    0.0%    0.0%    0.0%    0.0%    0.0%    0.0%    0.0%    0.0%    0.0%    0.0%    0.0%    0.0%    0.0%    0.0%    0.0%    0.0%    0.0%    0.0%    0.0%    0.0%    0.0%    0.0%    0.0%    0.0%    0.0%    0.0%    0.0%    0.0%    0.0%    0.0%    0.0%    0.0%    0.0%    0.0%    0.0%    0.0%    0.0%    0.0%    0.0%    0.0%    0.0%    0.0%    0.0%    0.0%    0.0%    0.0%    0.0%    0.0%    0.0%    0.0%    0.0%    0.0%    0.0%    0.0%    0.0%    0.0%    0.0%    0.0%    0.0%    0.0%    0.0%    0.0%    0.0%    0.0%    0.0%    0.0%    0.0%    0.0%    0.0%    0.0%    0.0%    0.0%    0.0%    0.0%    0.0%    0.0%    0.0%    0.0%    0.0%    0.0%    0.0%    0.0%    0.0%    0.0%    0.0%    0.0%    0.0%    0.0%    0.0%    0.0%    0.0%    0.0%    0.0%    0.0%    0.0%    0.0%    0.0%    0.0%    0.0%    0.0%    0.0%    0.0%    0.0%    0.0%    0.0%    0.0%    0.0%    0.0%    0.0%    0.0%    0.0%    0.0%    0.0%    0.0%    0.0%    0.0%    0.0%    0.0%    0.0%    0.0%    0.0%    0.0%    0.0%    0.0%    0.0%    0.0%    0.0%    0.0%    0.0%    0.0%    0.0%    0.0%    0.0%    0.0%    0.0%    0.0%    0.0%    0.0%    0.0%    0.0%    0.0%    0.0%    0.0%    0.0%    0.0%    0.0%    0.0%    0.0%    0.0%    0.0%    0.0%    0.0%    0.0%    0.0%    0.0%    0.0%    0.0%    0.0%    0.0%    0.0%    0.0%    0.0%    0.0%    0.0%    0.0%    0.0%    0.0%    0.0%    0.0%    0.0%    0.0%    0.0%    0.0%    0.0%    0.0%    0.0%    0.0%    0.0%    0.0%    0.0%    0.0%    0.0%    0.0%    0.0%    0.0%    0.0%    0.0%    0.0%    0.0%    0.0%    0.0%    0.0%    0.0%    0.0%    0.0%    0.0%    0.0%    0.0%    0.0%    0.0%    0.0%    0.0%    0.0%    0.0%    0.0%    0.0%    0.0%    0.0%    0.0%    0.0%    0.0%    0.0%    0.0%    0.0%    0.0%    0.0%    0.0%    0.0%    0.0%    0.0%    0.0%    0.0%    0.0%    0.0%    0.0%    0.0%    0.0%    0.0%    0.0%    0.0%    0.0%    0.0%    0.0%    0.0%    0.0%    0.0%    0.0%    0.0%    0.0%    0.0%    0.0%    0.0%    0.0%    0.0%    0.0%    0.0%    0.0%    0.0%    0.0%    0.0%    0.0%    0.0%    0.0%    0.0%    0.0%    0.0%    0.0%    0.0%    0.0%    0.0%    0.0%    0.0%    0.0%    0.0%    0.0%    0.0%    0.0%    0.0%    0.0%    0.0%    0.0%    0.0%    0.0%    0.0%    0.0%    0.0%    0.0%    0.0%    0.0%    0.0%    0.0%    0.0%    0.0%    0.0%    0.0%    0.0%    0.0%    0.0%    0.0%    0.0%        k__Bacteria;p__Bacteroidetes;c__; o__      67    0.0%    0.0%    0.0%    0.0%    0.0%    0.0%    0.0%    0.0%    0.0%    0.0%    0.0%    0.0%    0.0%    0.0%    0.0%    0.0%    0.0%    0.0%    0.0%    0.0%    0.0%    0.0%    0.0%    0.0%    0.0%    0.0%    0.0%    0.0%    0.0%    0.0%    0.0%    0.0%    0.0%    0.0%    0.0%    0.0%    0.0%    0.0%    0.0%    0.0%    0.0%    0.0%    0.0%    0.0%    0.0%    0.0%    0.0%    0.0%    0.0%    0.0%    0.0%    0.0%    0.0%    0.0%    0.0%    0.0%    0.0%    0.0%    0.0%    0.0%    0.0%    0.0%    0.0%    0.0%    0.0%    0.0%    0.0%    0.0%    0.0%    0.0%    0.0%    0.0%    0.0%    0.0%    0.0%    0.0%    0.0%    0.0%    0.0%    0.0%    0.0%    0.0%    0.0%    0.0%    0.0%    0.0%    0.0%    0.0%    0.0%    0.0%    0.0%    0.0%    0.0%    0.0%    0.0%    0.0%    0.0%    0.0%    0.0%    0.0%    0.0%    0.0%    0.0%    0.0%    0.0%    0.0%    0.0%    0.0%    0.0%    0.0%    0.0%    0.0%    0.0%    0.0%    0.0%    0.0%    0.0%    0.0%    0.0%    0.0%    0.0%    0.0%    0.0%    0.0%    0.0%    0.0%    0.0%    0.0%    0.0%    0.0%    0.0%    0.0%    0.0%    0.0%    0.0%    0.0%    0.0%    0.0%    0.0%    0.0%    0.0%    0.0%    0.0%    0.0%    0.0%    0.0%    0.0%    0.0%    0.0%    0.0%    0.0%    0.0%    0.0%    0.0%    0.0%    0.0%    0.0%    0.0%    0.0%    0.0%    0.0%    0.0%    0.0%    0.0%    0.0%    0.0%    0.0%    0.0%    0.0%    0.0%    0.0%    0.0%    0.0%    0.0%    0.0%    0.0%    0.0%    0.0%    0.0%    0.0%    0.0%    0.0%    0.0%    0.0%    0.0%    0.0%    0.0%    0.0%    0.0%    0.0%    0.0%    0.0%    0.0%    0.0%    0.0%    0.0%    0.0%    0.0%    0.0%    0.0%    0.0%    0.0%    0.0%    0.0%    0.0%    0.0%    0.0%    0.0%    0.0%    0.0%    0.0%    0.0%    0.0%    0.0%    0.0%    0.0%    0.0%    0.0%    0.0%    0.0%    0.0%    0.0%    0.0%    0.0%    0.0%    0.0%    0.0%    0.0%    0.0%    0.0%    0.0%    0.0%    0.0%    0.0%    0.0%    0.0%    0.0%    0.0%    0.0%    0.0%    0.0%    0.0%    0.0%    0.0%    0.0%    0.0%    0.0%    0.0%    0.0%    0.0%    0.0%    0.0%    0.0%    0.0%    0.0%    0.0%    0.0%    0.0%    0.0%    0.0%    0.0%    0.0%    0.0%    0.0%    0.0%    0.0%    0.0%    0.0%    0.0%    0.0%    0.0%    0.0%    0.0%    0.0%    0.0%    0.0%    0.0%    0.0%    0.0%    0.0%    0.0%    0.0%    0.0%    0.0%    0.0%    0.0%    0.0%    0.0%    0.0%    0.0%    0.0%    0.0%    0.0%    0.0%    0.0%    0.0%    0.0%    0.0%    0.0%    0.0%    0.0%    0.0%    0.0%    0.0%    0.0%    0.0%    0.0%    0.0%    0.0%    0.0%    0.0%    0.0%    0.0%    0.0%    0.0%    0.0%    0.0%    0.0%    0.0%    0.0%    0.0%    0.0%    0.0%    0.0%    0.0%    0.0%    0.0%    0.0%    0.0%    0.0%    0.0%    0.0%    0.0%    0.0%    0.0%    0.0%    0.0%    0.0%    0.0%    0.0%    0.0%    0.0%    0.0%    0.0%    0.0%    0.0%    0.0%    0.0%    0.0%    0.0%    0.0%    0.0%    0.0%    0.0%    0.0%    0.0%    0.0%    0.0%        k__Bacteria;p__Bacteroidetes;c__Bacteroidia; o__Bacteroidales   499072    2.9%    5.7%    3.2%    5.3%    3.3%    0.2%    4.0%    0.9%    4.7%    7.7%    3.3%    1.5%    1.2%    6.4%    4.5%    2.3%    5.8%    1.9%    0.9%    2.1%    3.0%    4.5%    1.3%    9.5%    4.5%    8.8%    0.7%    3.4%    4.9%    3.0%    4.3%    5.9%    1.6%    6.8%    6.3%   12.9%    3.6%   13.7%    3.3%    1.1%    1.5%    4.1%    3.2%    4.1%    2.6%    2.4%    1.3%    0.9%    1.4%    1.7%    1.2%    0.5%    2.1%    0.9%    1.8%    2.7%    1.6%    0.4%    1.8%    1.0%    1.2%    1.3%    0.9%    1.9%   13.5%    0.9%    0.1%    3.3%    0.5%    1.5%    6.6%    2.6%    0.4%    3.9%    2.6%    0.3%    0.7%    4.6%    4.4%    6.3%   12.9%    0.6%    2.9%    4.9%    2.5%    3.2%    3.7%    0.1%    1.9%    2.3%    0.1%    2.0%    4.0%    0.9%    2.8%    1.2%    2.6%    0.2%    8.6%    7.5%    2.1%    1.2%    1.1%    1.8%    1.8%    2.6%   11.0%    0.3%    0.0%    3.2%    3.2%   13.8%    2.9%    0.5%    6.3%    3.4%    1.8%    1.6%    3.0%    7.2%    0.1%    2.6%    3.4%    4.3%    0.3%    3.3%    3.9%    0.3%    1.5%    2.7%    3.4%    0.7%    0.3%    3.7%    5.3%    0.5%    1.0%    2.0%    0.1%    1.0%    0.1%    3.3%    1.7%    0.3%    0.5%    1.7%    6.9%    3.9%    3.0%    0.2%    0.5%    0.3%    2.4%    1.0%    3.7%    0.4%    1.4%    0.9%    0.5%    2.6%    0.5%    1.9%    1.6%    0.5%    1.2%    0.9%    2.4%    7.1%    2.9%    4.3%    2.3%    3.7%    0.3%    6.0%    1.2%    2.3%    2.2%    1.4%    0.4%    1.4%    1.9%    3.3%    1.6%    0.3%    2.5%    3.2%    1.4%    1.8%    2.1%    2.6%    3.7%    2.8%    3.4%    8.1%    1.3%    1.5%    5.4%    5.2%    1.8%    2.0%    6.1%   15.8%    1.3%    2.9%    2.6%    3.4%    0.7%    6.5%    2.3%    3.3%    2.8%    1.3%    0.6%    5.6%    1.1%    3.7%    2.8%    3.0%    4.6%    2.5%    2.3%    1.5%    0.0%    1.0%    0.6%    3.0%    1.5%    7.6%    2.1%    3.5%    6.5%    1.0%    0.5%    2.2%    2.0%    1.4%    2.0%    1.2%    0.4%    0.6%    1.4%    0.5%    2.7%    2.1%    5.5%    2.5%    1.0%    0.3%    0.7%    4.8%    1.6%    2.8%    0.6%    2.3%    0.3%    1.6%    2.1%    0.7%    1.4%    6.2%    1.3%    2.7%    4.0%    0.6%    3.3%    0.1%    2.2%    5.1%    1.5%    1.8%    2.8%    3.5%    1.7%    5.3%    1.0%    1.5%    1.3%    2.8%    3.7%   11.9%    0.9%    0.3%    1.3%    1.0%    1.3%    8.3%    2.6%    2.6%    5.3%    2.9%    2.5%    1.6%    3.3%    3.7%    1.6%    1.5%    6.1%    4.5%    2.9%    3.7%    6.8%    9.1%   12.9%    4.2%    2.7%    7.7%    7.8%    2.7%    0.6%    4.3%    0.3%    1.8%    0.3%    5.2%    5.5%    2.4%    0.5%    1.7%    1.6%    1.3%    9.5%    3.7%   25.8%    0.9%    3.1%    5.1%    3.4%    5.3%    2.3%    4.9%    3.8%    2.0%    4.2%    2.0%    2.4%    0.8%    3.2%    7.8%    4.4%    0.2%    1.1%    0.6%    1.6%    1.4%    1.0%    3.5%    0.5%    6.2%    0.2%    1.2%    1.6%    4.4%    2.4%    1.4%    0.8%    0.4%   26.5%    1.3%        k__Bacteria;p__Bacteroidetes;c__Flavobacteria; o__     180    0.0%    0.0%    0.0%    0.0%    0.0%    0.0%    0.0%    0.0%    0.0%    0.0%    0.0%    0.0%    0.0%    0.0%    0.0%    0.0%    0.0%    0.0%    0.0%    0.0%    0.0%    0.0%    0.0%    0.0%    0.0%    0.0%    0.0%    0.0%    0.0%    0.0%    0.0%    0.0%    0.0%    0.0%    0.0%    0.0%    0.0%    0.0%    0.0%    0.0%    0.0%    0.0%    0.0%    0.0%    0.0%    0.0%    0.0%    0.0%    0.0%    0.0%    0.0%    0.0%    0.0%    0.0%    0.0%    0.0%    0.0%    0.0%    0.0%    0.0%    0.0%    0.0%    0.0%    0.0%    0.0%    0.0%    0.0%    0.0%    0.0%    0.0%    0.0%    0.0%    0.0%    0.0%    0.0%    0.0%    0.0%    0.0%    0.0%    0.0%    0.0%    0.0%    0.0%    0.0%    0.0%    0.0%    0.0%    0.0%    0.0%    0.0%    0.0%    0.0%    0.0%    0.0%    0.0%    0.0%    0.0%    0.0%    0.0%    0.0%    0.0%    0.0%    0.0%    0.0%    0.0%    0.0%    0.0%    0.0%    0.0%    0.0%    0.0%    0.0%    0.0%    0.0%    0.0%    0.0%    0.0%    0.0%    0.0%    0.0%    0.0%    0.0%    0.0%    0.0%    0.0%    0.0%    0.0%    0.0%    0.0%    0.0%    0.0%    0.0%    0.0%    0.0%    0.0%    0.0%    0.0%    0.0%    0.0%    0.0%    0.0%    0.0%    0.0%    0.0%    0.0%    0.0%    0.0%    0.0%    0.0%    0.0%    0.0%    0.0%    0.0%    0.0%    0.0%    0.0%    0.0%    0.0%    0.0%    0.0%    0.0%    0.0%    0.0%    0.0%    0.0%    0.0%    0.0%    0.0%    0.0%    0.0%    0.0%    0.0%    0.0%    0.0%    0.0%    0.0%    0.0%    0.0%    0.0%    0.0%    0.0%    0.0%    0.0%    0.0%    0.0%    0.0%    0.0%    0.0%    0.0%    0.0%    0.0%    0.0%    0.0%    0.0%    0.0%    0.0%    0.0%    0.0%    0.0%    0.0%    0.0%    0.0%    0.0%    0.0%    0.0%    0.0%    0.0%    0.0%    0.0%    0.0%    0.0%    0.0%    0.0%    0.0%    0.0%    0.0%    0.0%    0.0%    0.0%    0.0%    0.0%    0.0%    0.0%    0.0%    0.0%    0.0%    0.0%    0.0%    0.0%    0.0%    0.0%    0.0%    0.0%    0.0%    0.0%    0.0%    0.0%    0.0%    0.0%    0.0%    0.0%    0.0%    0.0%    0.0%    0.0%    0.0%    0.0%    0.0%    0.0%    0.0%    0.0%    0.0%    0.0%    0.0%    0.0%    0.0%    0.0%    0.0%    0.0%    0.0%    0.0%    0.0%    0.0%    0.0%    0.0%    0.0%    0.0%    0.0%    0.0%    0.0%    0.0%    0.0%    0.0%    0.0%    0.0%    0.0%    0.0%    0.0%    0.0%    0.0%    0.0%    0.0%    0.0%    0.0%    0.0%    0.0%    0.0%    0.0%    0.0%    0.0%    0.0%    0.0%    0.0%    0.0%    0.0%    0.0%    0.0%    0.0%    0.0%    0.0%    0.0%    0.0%    0.0%    0.0%    0.0%    0.0%    0.0%    0.0%    0.0%    0.0%    0.0%    0.0%    0.0%    0.0%    0.0%    0.0%    0.0%    0.0%    0.0%    0.0%    0.0%    0.0%    0.0%    0.0%    0.0%    0.0%    0.0%    0.0%    0.0%    0.0%    0.0%    0.0%    0.0%    0.0%    0.0%    0.0%    0.0%    0.0%    0.0%    0.0%    0.0%    0.0%    0.0%    0.0%    0.0%    0.0%    0.0%    0.0%    0.0%    0.0%    0.0%    0.0%    0.0%    0.0%    0.0%    0.0%    0.0%    0.0%        k__Bacteria;p__Bacteroidetes;c__Flavobacteria; o__Flavobacteriales   168460    1.0%    1.9%    2.2%    2.7%    1.0%    1.8%    1.2%    0.4%    0.1%    0.5%    1.0%    0.2%    0.9%    1.5%    1.4%    0.7%    1.1%    0.9%    0.4%    0.4%    1.2%    2.7%    0.4%    0.0%    0.3%    0.3%    0.5%    0.5%    2.5%    0.2%    0.1%    0.8%    0.3%    1.1%    1.6%    1.9%    1.8%    0.1%    0.8%    0.8%    0.9%    1.4%    2.7%    1.7%    1.4%    0.7%    1.0%    0.2%    1.9%    0.8%    1.2%    2.9%    3.8%    1.3%    0.3%    0.3%    0.5%    0.2%    1.8%    0.8%    0.3%    0.5%    0.8%    1.0%    0.2%    0.3%    1.1%    1.4%    1.0%    0.5%    2.2%    0.3%    0.1%    1.2%    0.8%    0.1%    0.6%    0.5%    0.1%    0.5%    0.1%    1.7%    0.6%    0.6%    0.2%    0.4%    1.2%    1.1%    1.3%    0.9%    0.1%    0.3%    0.5%    0.9%    0.2%    0.2%    1.3%    0.2%    0.4%    0.6%    1.1%    0.2%    0.1%    0.2%    0.2%    1.5%    3.1%    1.5%    2.9%    0.4%    0.2%    1.1%    0.5%    0.1%    0.4%    0.3%    0.4%    0.3%    1.8%    2.5%    3.6%    0.7%    0.5%    0.8%    0.1%    2.2%    0.5%    0.3%    1.5%    2.2%    0.7%    0.2%    0.3%    0.5%    2.9%    0.3%    0.6%    1.0%    1.1%    0.7%    1.9%    2.7%    1.6%    6.8%    0.8%    0.3%    0.9%    0.2%    0.6%    1.7%    0.7%    0.3%    3.0%    0.7%    0.4%    0.6%    4.8%    1.0%    0.6%    1.2%    0.6%    0.9%    7.4%    0.8%    0.9%    0.4%    1.2%    0.6%    0.5%    0.9%    6.8%    1.8%    0.3%    1.4%    0.4%    0.4%    0.3%    0.7%    0.7%    0.6%    0.2%    0.3%    4.7%    0.3%    0.2%    0.2%    0.2%    0.4%    0.4%    0.5%    0.2%    0.4%    0.8%    0.6%    0.6%    0.9%    1.1%    0.4%    0.2%    0.2%    3.2%    0.3%    0.4%    1.1%    1.9%    0.8%    0.7%    1.7%    2.1%    1.2%    1.0%    0.3%    0.4%    4.4%    0.5%    0.6%    0.7%    0.1%    0.5%    0.4%    0.8%    2.5%    3.1%    1.6%    0.3%    0.8%    0.3%    0.8%    0.7%    2.1%    0.2%    0.0%    2.8%    0.4%    1.1%    0.6%    1.0%    0.1%    0.1%    0.1%    0.4%    0.1%    0.3%    0.5%    0.8%    0.0%    0.2%    0.1%    0.1%    0.4%    0.9%    1.7%    2.2%    0.2%    0.7%    0.2%    0.8%    0.2%    1.2%    1.2%    0.8%    2.0%    1.5%    4.8%    1.1%    0.5%    0.7%    2.1%    0.8%    0.7%    0.8%    1.0%    2.1%    1.2%    0.2%    0.7%    1.0%    0.5%    0.9%    0.3%    0.2%    0.2%    0.2%    0.2%    0.4%    0.9%    2.2%    1.3%    0.4%    0.2%    0.7%    0.1%    1.5%    1.5%    1.0%    0.2%    1.8%    0.9%    0.9%    0.7%    0.3%    0.4%    0.9%    0.3%    1.0%    0.7%    0.4%    0.3%    0.4%    2.2%    0.3%    0.2%    0.2%    1.9%    0.7%    1.1%    0.1%    0.9%    0.6%    1.7%    1.4%    1.7%    0.2%    0.2%    1.0%    1.0%    1.0%    0.6%    1.5%    0.6%    0.5%    0.7%    0.5%    0.4%    0.0%    0.4%    2.8%    0.9%    1.5%    4.0%    0.9%    2.6%    0.7%    0.7%    1.0%    0.3%    0.7%    0.9%    0.1%    0.2%    0.7%    1.5%    0.2%    0.6%    0.2%    0.3%    0.0%    0.0%        k__Bacteria;p__Bacteroidetes;c__Sphingobacteria; o__Sphingobacteriales   351755    2.1%    0.1%    0.6%    0.1%    0.4%    1.2%    0.0%    0.0%    0.0%    0.0%    0.0%    0.1%    0.7%    0.4%    0.1%    0.3%    0.4%    1.0%    0.2%    0.7%    0.7%    1.2%    0.5%    0.0%    0.0%    0.2%    0.7%    0.5%    0.8%    0.1%    0.1%    0.8%    0.3%    0.1%    0.9%    0.1%    0.7%    0.1%    0.4%    1.6%    0.3%    0.6%    0.0%    0.0%    0.6%    0.2%    0.1%    0.1%    0.6%    0.0%    0.8%    0.4%    0.3%    0.9%    0.7%    0.3%    0.8%    0.2%    0.5%    1.7%    1.0%    0.4%    0.4%    1.8%    1.2%    0.6%    2.3%    1.4%    1.3%    0.8%    1.0%    0.4%    0.3%    1.1%    1.3%    0.1%    0.7%    0.9%    0.2%    0.6%    0.3%    1.1%    0.2%    0.2%    0.1%    0.0%    1.2%    0.8%    0.6%    0.8%    0.6%    1.4%    0.7%    0.2%    0.2%    1.0%    1.3%    1.4%    2.3%    0.5%    1.5%   10.8%    9.5%    0.7%    0.1%    0.1%    1.4%    1.5%    2.6%    0.7%    0.5%    0.9%    0.2%    0.1%    0.8%    0.2%    5.9%    0.4%    0.4%    6.0%    6.2%    0.7%    1.4%    2.1%    0.2%    5.6%    1.8%    0.6%    0.9%    1.7%    0.9%    6.0%    4.0%    0.3%    1.8%   10.0%    1.6%    3.2%    1.5%    6.3%    0.4%    3.6%    5.6%    1.7%    3.7%    6.6%    4.2%    7.9%    0.6%    1.8%    7.5%   10.8%    1.7%   14.2%    0.2%   10.2%    2.4%    1.0%    0.4%    2.6%    2.3%    2.4%    1.2%    0.8%   11.6%    0.7%    1.3%    0.3%    1.0%    0.4%    3.5%    1.4%    1.2%    1.1%    6.8%    1.4%    0.5%    0.1%    1.4%    0.5%    0.3%    0.1%    3.8%    2.1%    0.8%    0.2%    0.3%    0.1%    0.1%    0.3%    0.3%    0.4%    0.2%    2.9%    1.4%    9.3%    1.6%    0.3%    0.3%    0.1%    1.3%    0.5%    0.8%    2.0%    2.8%    4.7%    0.7%    1.4%    3.6%    1.9%    1.4%   13.4%   12.0%    0.9%    3.7%    0.7%    7.7%    1.5%    1.6%    2.3%    1.1%    7.9%    0.8%    9.6%   11.4%    1.8%    2.6%    3.3%    1.6%    1.9%    6.2%    7.7%    3.7%    6.2%    3.5%    0.2%    2.4%    7.7%    4.3%    1.1%    0.8%    1.0%    0.9%    0.2%    0.8%    0.2%    0.2%    4.1%    1.6%    2.3%   11.1%    1.9%    3.3%    3.6%    3.5%    6.8%    3.5%   18.4%    4.0%    3.1%    7.2%    4.8%    2.7%    4.9%    7.9%    1.5%    2.5%    3.5%    5.0%   13.8%    4.1%    1.5%    6.8%    1.4%   17.1%    7.2%    1.8%    0.9%    0.3%    0.4%    0.6%    2.0%    7.4%    1.0%    9.8%    0.1%    0.6%    4.9%    0.6%    0.4%    1.3%    0.1%    1.2%    1.6%    1.0%    0.3%    0.7%    0.6%    0.6%    1.3%    0.4%    0.4%    0.1%    1.6%    0.6%    0.3%    0.6%    0.5%    0.8%    0.2%    0.0%    0.1%    0.1%    0.1%    0.3%    1.9%    0.0%    0.2%    0.0%    0.3%    0.3%    0.7%    0.1%    0.4%    1.2%    0.8%    1.0%    0.6%    0.8%    0.8%    0.0%    0.0%    0.1%    0.1%    0.0%    0.0%    0.0%    0.2%    0.4%   11.6%    1.0%    8.0%    0.6%    0.5%    2.4%    0.1%    2.4%    0.2%    3.5%    0.2%    0.5%    0.1%    0.4%    1.5%    0.2%    1.3%    0.0%    0.3%        k__Bacteria;p__CCM11b;c__; o__      40    0.0%    0.0%    0.0%    0.0%    0.0%    0.0%    0.0%    0.0%    0.0%    0.0%    0.0%    0.0%    0.0%    0.0%    0.0%    0.0%    0.0%    0.0%    0.0%    0.0%    0.0%    0.0%    0.0%    0.0%    0.0%    0.0%    0.0%    0.0%    0.0%    0.0%    0.0%    0.0%    0.0%    0.0%    0.0%    0.0%    0.0%    0.0%    0.0%    0.0%    0.0%    0.0%    0.0%    0.0%    0.0%    0.0%    0.0%    0.0%    0.0%    0.0%    0.0%    0.0%    0.0%    0.0%    0.0%    0.0%    0.0%    0.0%    0.0%    0.0%    0.0%    0.0%    0.0%    0.0%    0.0%    0.0%    0.0%    0.0%    0.0%    0.0%    0.0%    0.0%    0.0%    0.0%    0.0%    0.0%    0.0%    0.0%    0.0%    0.0%    0.0%    0.0%    0.0%    0.0%    0.0%    0.0%    0.0%    0.0%    0.0%    0.0%    0.0%    0.0%    0.0%    0.0%    0.0%    0.0%    0.0%    0.0%    0.0%    0.0%    0.0%    0.0%    0.0%    0.0%    0.0%    0.0%    0.0%    0.0%    0.0%    0.0%    0.0%    0.0%    0.0%    0.0%    0.0%    0.0%    0.0%    0.0%    0.0%    0.0%    0.0%    0.0%    0.0%    0.0%    0.0%    0.0%    0.0%    0.0%    0.0%    0.0%    0.0%    0.0%    0.0%    0.0%    0.0%    0.0%    0.0%    0.0%    0.0%    0.0%    0.0%    0.0%    0.0%    0.0%    0.0%    0.0%    0.0%    0.0%    0.0%    0.0%    0.0%    0.0%    0.0%    0.0%    0.0%    0.0%    0.0%    0.0%    0.0%    0.0%    0.0%    0.0%    0.0%    0.0%    0.0%    0.0%    0.0%    0.0%    0.0%    0.0%    0.0%    0.0%    0.0%    0.0%    0.0%    0.0%    0.0%    0.0%    0.0%    0.0%    0.0%    0.0%    0.0%    0.0%    0.0%    0.0%    0.0%    0.0%    0.0%    0.0%    0.0%    0.0%    0.0%    0.0%    0.0%    0.0%    0.0%    0.0%    0.0%    0.0%    0.0%    0.0%    0.0%    0.0%    0.0%    0.0%    0.0%    0.0%    0.0%    0.0%    0.0%    0.0%    0.0%    0.0%    0.0%    0.0%    0.0%    0.0%    0.0%    0.0%    0.0%    0.0%    0.0%    0.0%    0.0%    0.0%    0.0%    0.0%    0.0%    0.0%    0.0%    0.0%    0.0%    0.0%    0.0%    0.0%    0.0%    0.0%    0.0%    0.0%    0.0%    0.0%    0.0%    0.0%    0.0%    0.0%    0.0%    0.0%    0.0%    0.0%    0.0%    0.0%    0.0%    0.0%    0.0%    0.0%    0.0%    0.0%    0.0%    0.0%    0.0%    0.0%    0.0%    0.0%    0.0%    0.0%    0.0%    0.0%    0.0%    0.0%    0.0%    0.0%    0.0%    0.0%    0.0%    0.0%    0.0%    0.0%    0.0%    0.0%    0.0%    0.0%    0.0%    0.0%    0.0%    0.0%    0.0%    0.0%    0.0%    0.0%    0.0%    0.0%    0.0%    0.0%    0.0%    0.0%    0.0%    0.0%    0.0%    0.0%    0.0%    0.0%    0.0%    0.0%    0.0%    0.0%    0.0%    0.0%    0.0%    0.0%    0.0%    0.0%    0.0%    0.0%    0.0%    0.0%    0.0%    0.0%    0.0%    0.0%    0.0%    0.0%    0.0%    0.0%    0.0%    0.0%    0.0%    0.0%    0.0%    0.0%    0.0%    0.0%    0.0%    0.0%    0.0%    0.0%    0.0%    0.0%    0.0%    0.0%    0.0%    0.0%    0.0%    0.0%    0.0%    0.0%    0.0%    0.0%    0.0%    0.0%    0.0%    0.0%    0.0%    0.0%    0.0%    0.0%    0.0%    0.0%        k__Bacteria;p__Caldithrix_KSB1;c__Caldithrixae; o__Caldithrixales       1    0.0%    0.0%    0.0%    0.0%    0.0%    0.0%    0.0%    0.0%    0.0%    0.0%    0.0%    0.0%    0.0%    0.0%    0.0%    0.0%    0.0%    0.0%    0.0%    0.0%    0.0%    0.0%    0.0%    0.0%    0.0%    0.0%    0.0%    0.0%    0.0%    0.0%    0.0%    0.0%    0.0%    0.0%    0.0%    0.0%    0.0%    0.0%    0.0%    0.0%    0.0%    0.0%    0.0%    0.0%    0.0%    0.0%    0.0%    0.0%    0.0%    0.0%    0.0%    0.0%    0.0%    0.0%    0.0%    0.0%    0.0%    0.0%    0.0%    0.0%    0.0%    0.0%    0.0%    0.0%    0.0%    0.0%    0.0%    0.0%    0.0%    0.0%    0.0%    0.0%    0.0%    0.0%    0.0%    0.0%    0.0%    0.0%    0.0%    0.0%    0.0%    0.0%    0.0%    0.0%    0.0%    0.0%    0.0%    0.0%    0.0%    0.0%    0.0%    0.0%    0.0%    0.0%    0.0%    0.0%    0.0%    0.0%    0.0%    0.0%    0.0%    0.0%    0.0%    0.0%    0.0%    0.0%    0.0%    0.0%    0.0%    0.0%    0.0%    0.0%    0.0%    0.0%    0.0%    0.0%    0.0%    0.0%    0.0%    0.0%    0.0%    0.0%    0.0%    0.0%    0.0%    0.0%    0.0%    0.0%    0.0%    0.0%    0.0%    0.0%    0.0%    0.0%    0.0%    0.0%    0.0%    0.0%    0.0%    0.0%    0.0%    0.0%    0.0%    0.0%    0.0%    0.0%    0.0%    0.0%    0.0%    0.0%    0.0%    0.0%    0.0%    0.0%    0.0%    0.0%    0.0%    0.0%    0.0%    0.0%    0.0%    0.0%    0.0%    0.0%    0.0%    0.0%    0.0%    0.0%    0.0%    0.0%    0.0%    0.0%    0.0%    0.0%    0.0%    0.0%    0.0%    0.0%    0.0%    0.0%    0.0%    0.0%    0.0%    0.0%    0.0%    0.0%    0.0%    0.0%    0.0%    0.0%    0.0%    0.0%    0.0%    0.0%    0.0%    0.0%    0.0%    0.0%    0.0%    0.0%    0.0%    0.0%    0.0%    0.0%    0.0%    0.0%    0.0%    0.0%    0.0%    0.0%    0.0%    0.0%    0.0%    0.0%    0.0%    0.0%    0.0%    0.0%    0.0%    0.0%    0.0%    0.0%    0.0%    0.0%    0.0%    0.0%    0.0%    0.0%    0.0%    0.0%    0.0%    0.0%    0.0%    0.0%    0.0%    0.0%    0.0%    0.0%    0.0%    0.0%    0.0%    0.0%    0.0%    0.0%    0.0%    0.0%    0.0%    0.0%    0.0%    0.0%    0.0%    0.0%    0.0%    0.0%    0.0%    0.0%    0.0%    0.0%    0.0%    0.0%    0.0%    0.0%    0.0%    0.0%    0.0%    0.0%    0.0%    0.0%    0.0%    0.0%    0.0%    0.0%    0.0%    0.0%    0.0%    0.0%    0.0%    0.0%    0.0%    0.0%    0.0%    0.0%    0.0%    0.0%    0.0%    0.0%    0.0%    0.0%    0.0%    0.0%    0.0%    0.0%    0.0%    0.0%    0.0%    0.0%    0.0%    0.0%    0.0%    0.0%    0.0%    0.0%    0.0%    0.0%    0.0%    0.0%    0.0%    0.0%    0.0%    0.0%    0.0%    0.0%    0.0%    0.0%    0.0%    0.0%    0.0%    0.0%    0.0%    0.0%    0.0%    0.0%    0.0%    0.0%    0.0%    0.0%    0.0%    0.0%    0.0%    0.0%    0.0%    0.0%    0.0%    0.0%    0.0%    0.0%    0.0%    0.0%    0.0%    0.0%    0.0%    0.0%    0.0%    0.0%    0.0%    0.0%    0.0%    0.0%    0.0%    0.0%    0.0%    0.0%    0.0%    0.0%    0.0%    0.0%    0.0%    0.0%        k__Bacteria;p__Chlamydiae;c__Chlamydiae (class); o__Chlamydiales      25    0.0%    0.0%    0.0%    0.0%    0.0%    0.0%    0.0%    0.0%    0.0%    0.0%    0.0%    0.0%    0.0%    0.0%    0.0%    0.0%    0.0%    0.0%    0.0%    0.0%    0.0%    0.0%    0.0%    0.0%    0.0%    0.0%    0.0%    0.0%    0.0%    0.0%    0.0%    0.0%    0.0%    0.0%    0.0%    0.0%    0.0%    0.0%    0.0%    0.0%    0.0%    0.0%    0.0%    0.0%    0.0%    0.0%    0.0%    0.0%    0.0%    0.0%    0.0%    0.0%    0.0%    0.0%    0.0%    0.0%    0.0%    0.0%    0.0%    0.0%    0.0%    0.0%    0.0%    0.0%    0.0%    0.0%    0.0%    0.0%    0.0%    0.0%    0.0%    0.0%    0.0%    0.0%    0.0%    0.0%    0.0%    0.0%    0.0%    0.0%    0.0%    0.0%    0.0%    0.0%    0.0%    0.0%    0.0%    0.0%    0.0%    0.0%    0.0%    0.0%    0.0%    0.0%    0.0%    0.0%    0.0%    0.0%    0.0%    0.0%    0.0%    0.0%    0.0%    0.0%    0.0%    0.0%    0.0%    0.0%    0.0%    0.0%    0.0%    0.0%    0.0%    0.0%    0.0%    0.0%    0.0%    0.0%    0.0%    0.0%    0.0%    0.0%    0.0%    0.0%    0.0%    0.0%    0.0%    0.0%    0.0%    0.0%    0.0%    0.0%    0.0%    0.0%    0.0%    0.0%    0.0%    0.0%    0.0%    0.0%    0.0%    0.0%    0.0%    0.0%    0.0%    0.0%    0.0%    0.0%    0.0%    0.0%    0.0%    0.0%    0.0%    0.0%    0.0%    0.0%    0.0%    0.0%    0.0%    0.0%    0.0%    0.0%    0.0%    0.0%    0.0%    0.0%    0.0%    0.0%    0.0%    0.0%    0.0%    0.0%    0.0%    0.0%    0.0%    0.0%    0.0%    0.0%    0.0%    0.0%    0.0%    0.0%    0.0%    0.0%    0.0%    0.0%    0.0%    0.0%    0.0%    0.0%    0.0%    0.0%    0.0%    0.0%    0.0%    0.0%    0.0%    0.0%    0.0%    0.0%    0.0%    0.0%    0.0%    0.0%    0.0%    0.0%    0.0%    0.0%    0.0%    0.0%    0.0%    0.0%    0.0%    0.0%    0.0%    0.0%    0.0%    0.0%    0.0%    0.0%    0.0%    0.0%    0.0%    0.0%    0.0%    0.0%    0.0%    0.0%    0.0%    0.0%    0.0%    0.0%    0.0%    0.0%    0.0%    0.0%    0.0%    0.0%    0.0%    0.0%    0.0%    0.0%    0.0%    0.0%    0.0%    0.0%    0.0%    0.0%    0.0%    0.0%    0.0%    0.0%    0.0%    0.0%    0.0%    0.0%    0.0%    0.0%    0.0%    0.0%    0.0%    0.0%    0.0%    0.0%    0.0%    0.0%    0.0%    0.0%    0.0%    0.0%    0.0%    0.0%    0.0%    0.0%    0.0%    0.0%    0.0%    0.0%    0.0%    0.0%    0.0%    0.0%    0.0%    0.0%    0.0%    0.0%    0.0%    0.0%    0.0%    0.0%    0.0%    0.0%    0.0%    0.0%    0.0%    0.0%    0.0%    0.0%    0.0%    0.0%    0.0%    0.0%    0.0%    0.0%    0.0%    0.0%    0.0%    0.0%    0.0%    0.0%    0.0%    0.0%    0.0%    0.0%    0.0%    0.0%    0.0%    0.0%    0.0%    0.0%    0.0%    0.0%    0.0%    0.0%    0.0%    0.0%    0.0%    0.0%    0.0%    0.0%    0.0%    0.0%    0.0%    0.0%    0.0%    0.0%    0.0%    0.0%    0.0%    0.0%    0.0%    0.0%    0.0%    0.0%    0.0%    0.0%    0.0%    0.0%    0.0%    0.0%    0.0%    0.0%    0.0%    0.0%    0.0%    0.0%    0.0%    0.0%        k__Bacteria;p__Chlorobi;c__; o__       1    0.0%    0.0%    0.0%    0.0%    0.0%    0.0%    0.0%    0.0%    0.0%    0.0%    0.0%    0.0%    0.0%    0.0%    0.0%    0.0%    0.0%    0.0%    0.0%    0.0%    0.0%    0.0%    0.0%    0.0%    0.0%    0.0%    0.0%    0.0%    0.0%    0.0%    0.0%    0.0%    0.0%    0.0%    0.0%    0.0%    0.0%    0.0%    0.0%    0.0%    0.0%    0.0%    0.0%    0.0%    0.0%    0.0%    0.0%    0.0%    0.0%    0.0%    0.0%    0.0%    0.0%    0.0%    0.0%    0.0%    0.0%    0.0%    0.0%    0.0%    0.0%    0.0%    0.0%    0.0%    0.0%    0.0%    0.0%    0.0%    0.0%    0.0%    0.0%    0.0%    0.0%    0.0%    0.0%    0.0%    0.0%    0.0%    0.0%    0.0%    0.0%    0.0%    0.0%    0.0%    0.0%    0.0%    0.0%    0.0%    0.0%    0.0%    0.0%    0.0%    0.0%    0.0%    0.0%    0.0%    0.0%    0.0%    0.0%    0.0%    0.0%    0.0%    0.0%    0.0%    0.0%    0.0%    0.0%    0.0%    0.0%    0.0%    0.0%    0.0%    0.0%    0.0%    0.0%    0.0%    0.0%    0.0%    0.0%    0.0%    0.0%    0.0%    0.0%    0.0%    0.0%    0.0%    0.0%    0.0%    0.0%    0.0%    0.0%    0.0%    0.0%    0.0%    0.0%    0.0%    0.0%    0.0%    0.0%    0.0%    0.0%    0.0%    0.0%    0.0%    0.0%    0.0%    0.0%    0.0%    0.0%    0.0%    0.0%    0.0%    0.0%    0.0%    0.0%    0.0%    0.0%    0.0%    0.0%    0.0%    0.0%    0.0%    0.0%    0.0%    0.0%    0.0%    0.0%    0.0%    0.0%    0.0%    0.0%    0.0%    0.0%    0.0%    0.0%    0.0%    0.0%    0.0%    0.0%    0.0%    0.0%    0.0%    0.0%    0.0%    0.0%    0.0%    0.0%    0.0%    0.0%    0.0%    0.0%    0.0%    0.0%    0.0%    0.0%    0.0%    0.0%    0.0%    0.0%    0.0%    0.0%    0.0%    0.0%    0.0%    0.0%    0.0%    0.0%    0.0%    0.0%    0.0%    0.0%    0.0%    0.0%    0.0%    0.0%    0.0%    0.0%    0.0%    0.0%    0.0%    0.0%    0.0%    0.0%    0.0%    0.0%    0.0%    0.0%    0.0%    0.0%    0.0%    0.0%    0.0%    0.0%    0.0%    0.0%    0.0%    0.0%    0.0%    0.0%    0.0%    0.0%    0.0%    0.0%    0.0%    0.0%    0.0%    0.0%    0.0%    0.0%    0.0%    0.0%    0.0%    0.0%    0.0%    0.0%    0.0%    0.0%    0.0%    0.0%    0.0%    0.0%    0.0%    0.0%    0.0%    0.0%    0.0%    0.0%    0.0%    0.0%    0.0%    0.0%    0.0%    0.0%    0.0%    0.0%    0.0%    0.0%    0.0%    0.0%    0.0%    0.0%    0.0%    0.0%    0.0%    0.0%    0.0%    0.0%    0.0%    0.0%    0.0%    0.0%    0.0%    0.0%    0.0%    0.0%    0.0%    0.0%    0.0%    0.0%    0.0%    0.0%    0.0%    0.0%    0.0%    0.0%    0.0%    0.0%    0.0%    0.0%    0.0%    0.0%    0.0%    0.0%    0.0%    0.0%    0.0%    0.0%    0.0%    0.0%    0.0%    0.0%    0.0%    0.0%    0.0%    0.0%    0.0%    0.0%    0.0%    0.0%    0.0%    0.0%    0.0%    0.0%    0.0%    0.0%    0.0%    0.0%    0.0%    0.0%    0.0%    0.0%    0.0%    0.0%    0.0%    0.0%    0.0%    0.0%    0.0%    0.0%    0.0%    0.0%    0.0%    0.0%    0.0%    0.0%    0.0%    0.0%    0.0%        k__Bacteria;p__Chlorobi;c__BSV19; o__       0    0.0%    0.0%    0.0%    0.0%    0.0%    0.0%    0.0%    0.0%    0.0%    0.0%    0.0%    0.0%    0.0%    0.0%    0.0%    0.0%    0.0%    0.0%    0.0%    0.0%    0.0%    0.0%    0.0%    0.0%    0.0%    0.0%    0.0%    0.0%    0.0%    0.0%    0.0%    0.0%    0.0%    0.0%    0.0%    0.0%    0.0%    0.0%    0.0%    0.0%    0.0%    0.0%    0.0%    0.0%    0.0%    0.0%    0.0%    0.0%    0.0%    0.0%    0.0%    0.0%    0.0%    0.0%    0.0%    0.0%    0.0%    0.0%    0.0%    0.0%    0.0%    0.0%    0.0%    0.0%    0.0%    0.0%    0.0%    0.0%    0.0%    0.0%    0.0%    0.0%    0.0%    0.0%    0.0%    0.0%    0.0%    0.0%    0.0%    0.0%    0.0%    0.0%    0.0%    0.0%    0.0%    0.0%    0.0%    0.0%    0.0%    0.0%    0.0%    0.0%    0.0%    0.0%    0.0%    0.0%    0.0%    0.0%    0.0%    0.0%    0.0%    0.0%    0.0%    0.0%    0.0%    0.0%    0.0%    0.0%    0.0%    0.0%    0.0%    0.0%    0.0%    0.0%    0.0%    0.0%    0.0%    0.0%    0.0%    0.0%    0.0%    0.0%    0.0%    0.0%    0.0%    0.0%    0.0%    0.0%    0.0%    0.0%    0.0%    0.0%    0.0%    0.0%    0.0%    0.0%    0.0%    0.0%    0.0%    0.0%    0.0%    0.0%    0.0%    0.0%    0.0%    0.0%    0.0%    0.0%    0.0%    0.0%    0.0%    0.0%    0.0%    0.0%    0.0%    0.0%    0.0%    0.0%    0.0%    0.0%    0.0%    0.0%    0.0%    0.0%    0.0%    0.0%    0.0%    0.0%    0.0%    0.0%    0.0%    0.0%    0.0%    0.0%    0.0%    0.0%    0.0%    0.0%    0.0%    0.0%    0.0%    0.0%    0.0%    0.0%    0.0%    0.0%    0.0%    0.0%    0.0%    0.0%    0.0%    0.0%    0.0%    0.0%    0.0%    0.0%    0.0%    0.0%    0.0%    0.0%    0.0%    0.0%    0.0%    0.0%    0.0%    0.0%    0.0%    0.0%    0.0%    0.0%    0.0%    0.0%    0.0%    0.0%    0.0%    0.0%    0.0%    0.0%    0.0%    0.0%    0.0%    0.0%    0.0%    0.0%    0.0%    0.0%    0.0%    0.0%    0.0%    0.0%    0.0%    0.0%    0.0%    0.0%    0.0%    0.0%    0.0%    0.0%    0.0%    0.0%    0.0%    0.0%    0.0%    0.0%    0.0%    0.0%    0.0%    0.0%    0.0%    0.0%    0.0%    0.0%    0.0%    0.0%    0.0%    0.0%    0.0%    0.0%    0.0%    0.0%    0.0%    0.0%    0.0%    0.0%    0.0%    0.0%    0.0%    0.0%    0.0%    0.0%    0.0%    0.0%    0.0%    0.0%    0.0%    0.0%    0.0%    0.0%    0.0%    0.0%    0.0%    0.0%    0.0%    0.0%    0.0%    0.0%    0.0%    0.0%    0.0%    0.0%    0.0%    0.0%    0.0%    0.0%    0.0%    0.0%    0.0%    0.0%    0.0%    0.0%    0.0%    0.0%    0.0%    0.0%    0.0%    0.0%    0.0%    0.0%    0.0%    0.0%    0.0%    0.0%    0.0%    0.0%    0.0%    0.0%    0.0%    0.0%    0.0%    0.0%    0.0%    0.0%    0.0%    0.0%    0.0%    0.0%    0.0%    0.0%    0.0%    0.0%    0.0%    0.0%    0.0%    0.0%    0.0%    0.0%    0.0%    0.0%    0.0%    0.0%    0.0%    0.0%    0.0%    0.0%    0.0%    0.0%    0.0%    0.0%    0.0%    0.0%    0.0%    0.0%    0.0%    0.0%    0.0%    0.0%    0.0%    0.0%        k__Bacteria;p__Chlorobi;c__OPB56; o__      21    0.0%    0.0%    0.0%    0.0%    0.0%    0.0%    0.0%    0.0%    0.0%    0.0%    0.0%    0.0%    0.0%    0.0%    0.0%    0.0%    0.0%    0.0%    0.0%    0.0%    0.0%    0.0%    0.0%    0.0%    0.0%    0.0%    0.0%    0.0%    0.0%    0.0%    0.0%    0.0%    0.0%    0.0%    0.0%    0.0%    0.0%    0.0%    0.0%    0.0%    0.0%    0.0%    0.0%    0.0%    0.0%    0.0%    0.0%    0.0%    0.0%    0.0%    0.0%    0.0%    0.0%    0.0%    0.0%    0.0%    0.0%    0.0%    0.0%    0.0%    0.0%    0.0%    0.0%    0.0%    0.0%    0.0%    0.0%    0.0%    0.0%    0.0%    0.0%    0.0%    0.0%    0.0%    0.0%    0.0%    0.0%    0.0%    0.0%    0.0%    0.0%    0.0%    0.0%    0.0%    0.0%    0.0%    0.0%    0.0%    0.0%    0.0%    0.0%    0.0%    0.0%    0.0%    0.0%    0.0%    0.0%    0.0%    0.0%    0.0%    0.0%    0.0%    0.0%    0.0%    0.0%    0.0%    0.0%    0.0%    0.0%    0.0%    0.0%    0.0%    0.0%    0.0%    0.0%    0.0%    0.0%    0.0%    0.0%    0.0%    0.0%    0.0%    0.0%    0.0%    0.0%    0.0%    0.0%    0.0%    0.0%    0.0%    0.0%    0.0%    0.0%    0.0%    0.0%    0.0%    0.0%    0.0%    0.0%    0.0%    0.0%    0.0%    0.0%    0.0%    0.0%    0.0%    0.0%    0.0%    0.0%    0.0%    0.0%    0.0%    0.0%    0.0%    0.0%    0.0%    0.0%    0.0%    0.0%    0.0%    0.0%    0.0%    0.0%    0.0%    0.0%    0.0%    0.0%    0.0%    0.0%    0.0%    0.0%    0.0%    0.0%    0.0%    0.0%    0.0%    0.0%    0.0%    0.0%    0.0%    0.0%    0.0%    0.0%    0.0%    0.0%    0.0%    0.0%    0.0%    0.0%    0.0%    0.0%    0.0%    0.0%    0.0%    0.0%    0.0%    0.0%    0.0%    0.0%    0.0%    0.0%    0.0%    0.0%    0.0%    0.0%    0.0%    0.0%    0.0%    0.0%    0.0%    0.0%    0.0%    0.0%    0.0%    0.0%    0.0%    0.0%    0.0%    0.0%    0.0%    0.0%    0.0%    0.0%    0.0%    0.0%    0.0%    0.0%    0.0%    0.0%    0.0%    0.0%    0.0%    0.0%    0.0%    0.0%    0.0%    0.0%    0.0%    0.0%    0.0%    0.0%    0.0%    0.0%    0.0%    0.0%    0.0%    0.0%    0.0%    0.0%    0.0%    0.0%    0.0%    0.0%    0.0%    0.0%    0.0%    0.0%    0.0%    0.0%    0.0%    0.0%    0.0%    0.0%    0.0%    0.0%    0.0%    0.0%    0.0%    0.0%    0.0%    0.0%    0.0%    0.0%    0.0%    0.0%    0.0%    0.0%    0.0%    0.0%    0.0%    0.0%    0.0%    0.0%    0.0%    0.0%    0.0%    0.0%    0.0%    0.0%    0.0%    0.0%    0.0%    0.0%    0.0%    0.0%    0.0%    0.0%    0.0%    0.0%    0.0%    0.0%    0.0%    0.0%    0.0%    0.0%    0.0%    0.0%    0.0%    0.0%    0.0%    0.0%    0.0%    0.0%    0.0%    0.0%    0.0%    0.0%    0.0%    0.0%    0.0%    0.0%    0.0%    0.0%    0.0%    0.0%    0.0%    0.0%    0.0%    0.0%    0.0%    0.0%    0.0%    0.0%    0.0%    0.0%    0.0%    0.0%    0.0%    0.0%    0.0%    0.0%    0.0%    0.0%    0.0%    0.0%    0.0%    0.0%    0.0%    0.0%    0.0%    0.0%    0.0%    0.0%    0.0%    0.0%    0.0%    0.0%    0.0%        k__Bacteria;p__Chlorobi;c__SJA-28; o__      12    0.0%    0.0%    0.0%    0.0%    0.0%    0.0%    0.0%    0.0%    0.0%    0.0%    0.0%    0.0%    0.0%    0.0%    0.0%    0.0%    0.0%    0.0%    0.0%    0.0%    0.0%    0.0%    0.0%    0.0%    0.0%    0.0%    0.0%    0.0%    0.0%    0.0%    0.0%    0.0%    0.0%    0.0%    0.0%    0.0%    0.0%    0.0%    0.0%    0.0%    0.0%    0.0%    0.0%    0.0%    0.0%    0.0%    0.0%    0.0%    0.0%    0.0%    0.0%    0.0%    0.0%    0.0%    0.0%    0.0%    0.0%    0.0%    0.0%    0.0%    0.0%    0.0%    0.0%    0.0%    0.0%    0.0%    0.0%    0.0%    0.0%    0.0%    0.0%    0.0%    0.0%    0.0%    0.0%    0.0%    0.0%    0.0%    0.0%    0.0%    0.0%    0.0%    0.0%    0.0%    0.0%    0.0%    0.0%    0.0%    0.0%    0.0%    0.0%    0.0%    0.0%    0.0%    0.0%    0.0%    0.0%    0.0%    0.0%    0.0%    0.0%    0.0%    0.0%    0.0%    0.0%    0.0%    0.0%    0.0%    0.0%    0.0%    0.0%    0.0%    0.0%    0.0%    0.0%    0.0%    0.0%    0.0%    0.0%    0.0%    0.0%    0.0%    0.0%    0.0%    0.0%    0.0%    0.0%    0.0%    0.0%    0.0%    0.0%    0.0%    0.0%    0.0%    0.0%    0.0%    0.0%    0.0%    0.0%    0.0%    0.0%    0.0%    0.0%    0.0%    0.0%    0.0%    0.0%    0.0%    0.0%    0.0%    0.0%    0.0%    0.0%    0.0%    0.0%    0.0%    0.0%    0.0%    0.0%    0.0%    0.0%    0.0%    0.0%    0.0%    0.0%    0.0%    0.0%    0.0%    0.0%    0.0%    0.0%    0.0%    0.0%    0.0%    0.0%    0.0%    0.0%    0.0%    0.0%    0.0%    0.0%    0.0%    0.0%    0.0%    0.0%    0.0%    0.0%    0.0%    0.0%    0.0%    0.0%    0.0%    0.0%    0.0%    0.0%    0.0%    0.0%    0.0%    0.0%    0.0%    0.0%    0.0%    0.0%    0.0%    0.0%    0.0%    0.0%    0.0%    0.0%    0.0%    0.0%    0.0%    0.0%    0.0%    0.0%    0.0%    0.0%    0.0%    0.0%    0.0%    0.0%    0.0%    0.0%    0.0%    0.0%    0.0%    0.0%    0.0%    0.0%    0.0%    0.0%    0.0%    0.0%    0.0%    0.0%    0.0%    0.0%    0.0%    0.0%    0.0%    0.0%    0.0%    0.0%    0.0%    0.0%    0.0%    0.0%    0.0%    0.0%    0.0%    0.0%    0.0%    0.0%    0.0%    0.0%    0.0%    0.0%    0.0%    0.0%    0.0%    0.0%    0.0%    0.0%    0.0%    0.0%    0.0%    0.0%    0.0%    0.0%    0.0%    0.0%    0.0%    0.0%    0.0%    0.0%    0.0%    0.0%    0.0%    0.0%    0.0%    0.0%    0.0%    0.0%    0.0%    0.0%    0.0%    0.0%    0.0%    0.0%    0.0%    0.0%    0.0%    0.0%    0.0%    0.0%    0.0%    0.0%    0.0%    0.0%    0.0%    0.0%    0.0%    0.0%    0.0%    0.0%    0.0%    0.0%    0.0%    0.0%    0.0%    0.0%    0.0%    0.0%    0.0%    0.0%    0.0%    0.0%    0.0%    0.0%    0.0%    0.0%    0.0%    0.0%    0.0%    0.0%    0.0%    0.0%    0.0%    0.0%    0.0%    0.0%    0.0%    0.0%    0.0%    0.0%    0.0%    0.0%    0.0%    0.0%    0.0%    0.0%    0.0%    0.0%    0.0%    0.0%    0.0%    0.0%    0.0%    0.0%    0.0%    0.0%    0.0%    0.0%    0.0%    0.0%    0.0%    0.0%    0.0%        k__Bacteria;p__Chlorobi;c__ZB1; o__       2    0.0%    0.0%    0.0%    0.0%    0.0%    0.0%    0.0%    0.0%    0.0%    0.0%    0.0%    0.0%    0.0%    0.0%    0.0%    0.0%    0.0%    0.0%    0.0%    0.0%    0.0%    0.0%    0.0%    0.0%    0.0%    0.0%    0.0%    0.0%    0.0%    0.0%    0.0%    0.0%    0.0%    0.0%    0.0%    0.0%    0.0%    0.0%    0.0%    0.0%    0.0%    0.0%    0.0%    0.0%    0.0%    0.0%    0.0%    0.0%    0.0%    0.0%    0.0%    0.0%    0.0%    0.0%    0.0%    0.0%    0.0%    0.0%    0.0%    0.0%    0.0%    0.0%    0.0%    0.0%    0.0%    0.0%    0.0%    0.0%    0.0%    0.0%    0.0%    0.0%    0.0%    0.0%    0.0%    0.0%    0.0%    0.0%    0.0%    0.0%    0.0%    0.0%    0.0%    0.0%    0.0%    0.0%    0.0%    0.0%    0.0%    0.0%    0.0%    0.0%    0.0%    0.0%    0.0%    0.0%    0.0%    0.0%    0.0%    0.0%    0.0%    0.0%    0.0%    0.0%    0.0%    0.0%    0.0%    0.0%    0.0%    0.0%    0.0%    0.0%    0.0%    0.0%    0.0%    0.0%    0.0%    0.0%    0.0%    0.0%    0.0%    0.0%    0.0%    0.0%    0.0%    0.0%    0.0%    0.0%    0.0%    0.0%    0.0%    0.0%    0.0%    0.0%    0.0%    0.0%    0.0%    0.0%    0.0%    0.0%    0.0%    0.0%    0.0%    0.0%    0.0%    0.0%    0.0%    0.0%    0.0%    0.0%    0.0%    0.0%    0.0%    0.0%    0.0%    0.0%    0.0%    0.0%    0.0%    0.0%    0.0%    0.0%    0.0%    0.0%    0.0%    0.0%    0.0%    0.0%    0.0%    0.0%    0.0%    0.0%    0.0%    0.0%    0.0%    0.0%    0.0%    0.0%    0.0%    0.0%    0.0%    0.0%    0.0%    0.0%    0.0%    0.0%    0.0%    0.0%    0.0%    0.0%    0.0%    0.0%    0.0%    0.0%    0.0%    0.0%    0.0%    0.0%    0.0%    0.0%    0.0%    0.0%    0.0%    0.0%    0.0%    0.0%    0.0%    0.0%    0.0%    0.0%    0.0%    0.0%    0.0%    0.0%    0.0%    0.0%    0.0%    0.0%    0.0%    0.0%    0.0%    0.0%    0.0%    0.0%    0.0%    0.0%    0.0%    0.0%    0.0%    0.0%    0.0%    0.0%    0.0%    0.0%    0.0%    0.0%    0.0%    0.0%    0.0%    0.0%    0.0%    0.0%    0.0%    0.0%    0.0%    0.0%    0.0%    0.0%    0.0%    0.0%    0.0%    0.0%    0.0%    0.0%    0.0%    0.0%    0.0%    0.0%    0.0%    0.0%    0.0%    0.0%    0.0%    0.0%    0.0%    0.0%    0.0%    0.0%    0.0%    0.0%    0.0%    0.0%    0.0%    0.0%    0.0%    0.0%    0.0%    0.0%    0.0%    0.0%    0.0%    0.0%    0.0%    0.0%    0.0%    0.0%    0.0%    0.0%    0.0%    0.0%    0.0%    0.0%    0.0%    0.0%    0.0%    0.0%    0.0%    0.0%    0.0%    0.0%    0.0%    0.0%    0.0%    0.0%    0.0%    0.0%    0.0%    0.0%    0.0%    0.0%    0.0%    0.0%    0.0%    0.0%    0.0%    0.0%    0.0%    0.0%    0.0%    0.0%    0.0%    0.0%    0.0%    0.0%    0.0%    0.0%    0.0%    0.0%    0.0%    0.0%    0.0%    0.0%    0.0%    0.0%    0.0%    0.0%    0.0%    0.0%    0.0%    0.0%    0.0%    0.0%    0.0%    0.0%    0.0%    0.0%    0.0%    0.0%    0.0%    0.0%    0.0%    0.0%    0.0%    0.0%    0.0%    0.0%    0.0%    0.0%        k__Bacteria;p__Chloroflexi;c__; o__      12    0.0%    0.0%    0.0%    0.0%    0.0%    0.0%    0.0%    0.0%    0.0%    0.0%    0.0%    0.0%    0.0%    0.0%    0.0%    0.0%    0.0%    0.0%    0.0%    0.0%    0.0%    0.0%    0.0%    0.0%    0.0%    0.0%    0.0%    0.0%    0.0%    0.0%    0.0%    0.0%    0.0%    0.0%    0.0%    0.0%    0.0%    0.0%    0.0%    0.0%    0.0%    0.0%    0.0%    0.0%    0.0%    0.0%    0.0%    0.0%    0.0%    0.0%    0.0%    0.0%    0.0%    0.0%    0.0%    0.0%    0.0%    0.0%    0.0%    0.0%    0.0%    0.0%    0.0%    0.0%    0.0%    0.0%    0.0%    0.0%    0.0%    0.0%    0.0%    0.0%    0.0%    0.0%    0.0%    0.0%    0.0%    0.0%    0.0%    0.0%    0.0%    0.0%    0.0%    0.0%    0.0%    0.0%    0.0%    0.0%    0.0%    0.0%    0.0%    0.0%    0.0%    0.0%    0.0%    0.0%    0.0%    0.0%    0.0%    0.0%    0.0%    0.0%    0.0%    0.0%    0.0%    0.0%    0.0%    0.0%    0.0%    0.0%    0.0%    0.0%    0.0%    0.0%    0.0%    0.0%    0.0%    0.0%    0.0%    0.0%    0.0%    0.0%    0.0%    0.0%    0.0%    0.0%    0.0%    0.0%    0.0%    0.0%    0.0%    0.0%    0.0%    0.0%    0.0%    0.0%    0.0%    0.0%    0.0%    0.0%    0.0%    0.0%    0.0%    0.0%    0.0%    0.0%    0.0%    0.0%    0.0%    0.0%    0.0%    0.0%    0.0%    0.0%    0.0%    0.0%    0.0%    0.0%    0.0%    0.0%    0.0%    0.0%    0.0%    0.0%    0.0%    0.0%    0.0%    0.0%    0.0%    0.0%    0.0%    0.0%    0.0%    0.0%    0.0%    0.0%    0.0%    0.0%    0.0%    0.0%    0.0%    0.0%    0.0%    0.0%    0.0%    0.0%    0.0%    0.0%    0.0%    0.0%    0.0%    0.0%    0.0%    0.0%    0.0%    0.0%    0.0%    0.0%    0.0%    0.0%    0.0%    0.0%    0.0%    0.0%    0.0%    0.0%    0.0%    0.0%    0.0%    0.0%    0.0%    0.0%    0.0%    0.0%    0.0%    0.0%    0.0%    0.0%    0.0%    0.0%    0.0%    0.0%    0.0%    0.0%    0.0%    0.0%    0.0%    0.0%    0.0%    0.0%    0.0%    0.0%    0.0%    0.0%    0.0%    0.0%    0.0%    0.0%    0.0%    0.0%    0.0%    0.0%    0.0%    0.0%    0.0%    0.0%    0.0%    0.0%    0.0%    0.0%    0.0%    0.0%    0.0%    0.0%    0.0%    0.0%    0.0%    0.0%    0.0%    0.0%    0.0%    0.0%    0.0%    0.0%    0.0%    0.0%    0.0%    0.0%    0.0%    0.0%    0.0%    0.0%    0.0%    0.0%    0.0%    0.0%    0.0%    0.0%    0.0%    0.0%    0.0%    0.0%    0.0%    0.0%    0.0%    0.0%    0.0%    0.0%    0.0%    0.0%    0.0%    0.0%    0.0%    0.0%    0.0%    0.0%    0.0%    0.0%    0.0%    0.0%    0.0%    0.0%    0.0%    0.0%    0.0%    0.0%    0.0%    0.0%    0.0%    0.0%    0.0%    0.0%    0.0%    0.0%    0.0%    0.0%    0.0%    0.0%    0.0%    0.0%    0.0%    0.0%    0.0%    0.0%    0.0%    0.0%    0.0%    0.0%    0.0%    0.0%    0.0%    0.0%    0.0%    0.0%    0.0%    0.0%    0.0%    0.0%    0.0%    0.0%    0.0%    0.0%    0.0%    0.0%    0.0%    0.0%    0.0%    0.0%    0.0%    0.0%    0.0%    0.0%    0.0%    0.0%    0.0%    0.0%    0.0%    0.0%        k__Bacteria;p__Chloroflexi;c__Anaerolineae; o__       4    0.0%    0.0%    0.0%    0.0%    0.0%    0.0%    0.0%    0.0%    0.0%    0.0%    0.0%    0.0%    0.0%    0.0%    0.0%    0.0%    0.0%    0.0%    0.0%    0.0%    0.0%    0.0%    0.0%    0.0%    0.0%    0.0%    0.0%    0.0%    0.0%    0.0%    0.0%    0.0%    0.0%    0.0%    0.0%    0.0%    0.0%    0.0%    0.0%    0.0%    0.0%    0.0%    0.0%    0.0%    0.0%    0.0%    0.0%    0.0%    0.0%    0.0%    0.0%    0.0%    0.0%    0.0%    0.0%    0.0%    0.0%    0.0%    0.0%    0.0%    0.0%    0.0%    0.0%    0.0%    0.0%    0.0%    0.0%    0.0%    0.0%    0.0%    0.0%    0.0%    0.0%    0.0%    0.0%    0.0%    0.0%    0.0%    0.0%    0.0%    0.0%    0.0%    0.0%    0.0%    0.0%    0.0%    0.0%    0.0%    0.0%    0.0%    0.0%    0.0%    0.0%    0.0%    0.0%    0.0%    0.0%    0.0%    0.0%    0.0%    0.0%    0.0%    0.0%    0.0%    0.0%    0.0%    0.0%    0.0%    0.0%    0.0%    0.0%    0.0%    0.0%    0.0%    0.0%    0.0%    0.0%    0.0%    0.0%    0.0%    0.0%    0.0%    0.0%    0.0%    0.0%    0.0%    0.0%    0.0%    0.0%    0.0%    0.0%    0.0%    0.0%    0.0%    0.0%    0.0%    0.0%    0.0%    0.0%    0.0%    0.0%    0.0%    0.0%    0.0%    0.0%    0.0%    0.0%    0.0%    0.0%    0.0%    0.0%    0.0%    0.0%    0.0%    0.0%    0.0%    0.0%    0.0%    0.0%    0.0%    0.0%    0.0%    0.0%    0.0%    0.0%    0.0%    0.0%    0.0%    0.0%    0.0%    0.0%    0.0%    0.0%    0.0%    0.0%    0.0%    0.0%    0.0%    0.0%    0.0%    0.0%    0.0%    0.0%    0.0%    0.0%    0.0%    0.0%    0.0%    0.0%    0.0%    0.0%    0.0%    0.0%    0.0%    0.0%    0.0%    0.0%    0.0%    0.0%    0.0%    0.0%    0.0%    0.0%    0.0%    0.0%    0.0%    0.0%    0.0%    0.0%    0.0%    0.0%    0.0%    0.0%    0.0%    0.0%    0.0%    0.0%    0.0%    0.0%    0.0%    0.0%    0.0%    0.0%    0.0%    0.0%    0.0%    0.0%    0.0%    0.0%    0.0%    0.0%    0.0%    0.0%    0.0%    0.0%    0.0%    0.0%    0.0%    0.0%    0.0%    0.0%    0.0%    0.0%    0.0%    0.0%    0.0%    0.0%    0.0%    0.0%    0.0%    0.0%    0.0%    0.0%    0.0%    0.0%    0.0%    0.0%    0.0%    0.0%    0.0%    0.0%    0.0%    0.0%    0.0%    0.0%    0.0%    0.0%    0.0%    0.0%    0.0%    0.0%    0.0%    0.0%    0.0%    0.0%    0.0%    0.0%    0.0%    0.0%    0.0%    0.0%    0.0%    0.0%    0.0%    0.0%    0.0%    0.0%    0.0%    0.0%    0.0%    0.0%    0.0%    0.0%    0.0%    0.0%    0.0%    0.0%    0.0%    0.0%    0.0%    0.0%    0.0%    0.0%    0.0%    0.0%    0.0%    0.0%    0.0%    0.0%    0.0%    0.0%    0.0%    0.0%    0.0%    0.0%    0.0%    0.0%    0.0%    0.0%    0.0%    0.0%    0.0%    0.0%    0.0%    0.0%    0.0%    0.0%    0.0%    0.0%    0.0%    0.0%    0.0%    0.0%    0.0%    0.0%    0.0%    0.0%    0.0%    0.0%    0.0%    0.0%    0.0%    0.0%    0.0%    0.0%    0.0%    0.0%    0.0%    0.0%    0.0%    0.0%    0.0%    0.0%    0.0%    0.0%    0.0%    0.0%    0.0%        k__Bacteria;p__Chloroflexi;c__Anaerolineae; o__A31      10    0.0%    0.0%    0.0%    0.0%    0.0%    0.0%    0.0%    0.0%    0.0%    0.0%    0.0%    0.0%    0.0%    0.0%    0.0%    0.0%    0.0%    0.0%    0.0%    0.0%    0.0%    0.0%    0.0%    0.0%    0.0%    0.0%    0.0%    0.0%    0.0%    0.0%    0.0%    0.0%    0.0%    0.0%    0.0%    0.0%    0.0%    0.0%    0.0%    0.0%    0.0%    0.0%    0.0%    0.0%    0.0%    0.0%    0.0%    0.0%    0.0%    0.0%    0.0%    0.0%    0.0%    0.0%    0.0%    0.0%    0.0%    0.0%    0.0%    0.0%    0.0%    0.0%    0.0%    0.0%    0.0%    0.0%    0.0%    0.0%    0.0%    0.0%    0.0%    0.0%    0.0%    0.0%    0.0%    0.0%    0.0%    0.0%    0.0%    0.0%    0.0%    0.0%    0.0%    0.0%    0.0%    0.0%    0.0%    0.0%    0.0%    0.0%    0.0%    0.0%    0.0%    0.0%    0.0%    0.0%    0.0%    0.0%    0.0%    0.0%    0.0%    0.0%    0.0%    0.0%    0.0%    0.0%    0.0%    0.0%    0.0%    0.0%    0.0%    0.0%    0.0%    0.0%    0.0%    0.0%    0.0%    0.0%    0.0%    0.0%    0.0%    0.0%    0.0%    0.0%    0.0%    0.0%    0.0%    0.0%    0.0%    0.0%    0.0%    0.0%    0.0%    0.0%    0.0%    0.0%    0.0%    0.0%    0.0%    0.0%    0.0%    0.0%    0.0%    0.0%    0.0%    0.0%    0.0%    0.0%    0.0%    0.0%    0.0%    0.0%    0.0%    0.0%    0.0%    0.0%    0.0%    0.0%    0.0%    0.0%    0.0%    0.0%    0.0%    0.0%    0.0%    0.0%    0.0%    0.0%    0.0%    0.0%    0.0%    0.0%    0.0%    0.0%    0.0%    0.0%    0.0%    0.0%    0.0%    0.0%    0.0%    0.0%    0.0%    0.0%    0.0%    0.0%    0.0%    0.0%    0.0%    0.0%    0.0%    0.0%    0.0%    0.0%    0.0%    0.0%    0.0%    0.0%    0.0%    0.0%    0.0%    0.0%    0.0%    0.0%    0.0%    0.0%    0.0%    0.0%    0.0%    0.0%    0.0%    0.0%    0.0%    0.0%    0.0%    0.0%    0.0%    0.0%    0.0%    0.0%    0.0%    0.0%    0.0%    0.0%    0.0%    0.0%    0.0%    0.0%    0.0%    0.0%    0.0%    0.0%    0.0%    0.0%    0.0%    0.0%    0.0%    0.0%    0.0%    0.0%    0.0%    0.0%    0.0%    0.0%    0.0%    0.0%    0.0%    0.0%    0.0%    0.0%    0.0%    0.0%    0.0%    0.0%    0.0%    0.0%    0.0%    0.0%    0.0%    0.0%    0.0%    0.0%    0.0%    0.0%    0.0%    0.0%    0.0%    0.0%    0.0%    0.0%    0.0%    0.0%    0.0%    0.0%    0.0%    0.0%    0.0%    0.0%    0.0%    0.0%    0.0%    0.0%    0.0%    0.0%    0.0%    0.0%    0.0%    0.0%    0.0%    0.0%    0.0%    0.0%    0.0%    0.0%    0.0%    0.0%    0.0%    0.0%    0.0%    0.0%    0.0%    0.0%    0.0%    0.0%    0.0%    0.0%    0.0%    0.0%    0.0%    0.0%    0.0%    0.0%    0.0%    0.0%    0.0%    0.0%    0.0%    0.0%    0.0%    0.0%    0.0%    0.0%    0.0%    0.0%    0.0%    0.0%    0.0%    0.0%    0.0%    0.0%    0.0%    0.0%    0.0%    0.0%    0.0%    0.0%    0.0%    0.0%    0.0%    0.0%    0.0%    0.0%    0.0%    0.0%    0.0%    0.0%    0.0%    0.0%    0.0%    0.0%    0.0%    0.0%    0.0%    0.0%    0.0%    0.0%    0.0%    0.0%        k__Bacteria;p__Chloroflexi;c__Anaerolineae; o__A4b     159    0.0%    0.0%    0.0%    0.0%    0.0%    0.0%    0.0%    0.0%    0.0%    0.0%    0.0%    0.0%    0.0%    0.0%    0.0%    0.0%    0.0%    0.0%    0.0%    0.0%    0.0%    0.0%    0.0%    0.0%    0.0%    0.0%    0.0%    0.0%    0.0%    0.0%    0.0%    0.0%    0.0%    0.0%    0.0%    0.0%    0.0%    0.0%    0.0%    0.0%    0.0%    0.0%    0.0%    0.0%    0.0%    0.0%    0.0%    0.0%    0.0%    0.0%    0.0%    0.0%    0.0%    0.0%    0.0%    0.0%    0.0%    0.0%    0.0%    0.0%    0.0%    0.0%    0.0%    0.0%    0.0%    0.0%    0.0%    0.0%    0.0%    0.0%    0.0%    0.0%    0.0%    0.0%    0.0%    0.0%    0.0%    0.0%    0.0%    0.0%    0.0%    0.0%    0.0%    0.0%    0.0%    0.0%    0.0%    0.0%    0.0%    0.0%    0.0%    0.0%    0.0%    0.0%    0.0%    0.0%    0.0%    0.0%    0.0%    0.0%    0.0%    0.0%    0.0%    0.0%    0.0%    0.0%    0.0%    0.0%    0.0%    0.0%    0.0%    0.0%    0.0%    0.0%    0.0%    0.0%    0.0%    0.0%    0.0%    0.0%    0.0%    0.0%    0.0%    0.0%    0.0%    0.0%    0.0%    0.0%    0.0%    0.0%    0.0%    0.0%    0.0%    0.0%    0.0%    0.0%    0.0%    0.0%    0.0%    0.0%    0.0%    0.0%    0.0%    0.0%    0.0%    0.0%    0.0%    0.0%    0.0%    0.0%    0.0%    0.0%    0.0%    0.0%    0.0%    0.0%    0.0%    0.0%    0.0%    0.0%    0.0%    0.0%    0.0%    0.0%    0.0%    0.0%    0.0%    0.0%    0.0%    0.0%    0.0%    0.0%    0.0%    0.0%    0.0%    0.0%    0.0%    0.0%    0.0%    0.0%    0.0%    0.0%    0.0%    0.0%    0.0%    0.0%    0.0%    0.0%    0.0%    0.0%    0.0%    0.0%    0.0%    0.0%    0.0%    0.0%    0.0%    0.0%    0.0%    0.0%    0.0%    0.0%    0.0%    0.0%    0.0%    0.0%    0.0%    0.0%    0.0%    0.0%    0.0%    0.0%    0.0%    0.0%    0.0%    0.0%    0.0%    0.0%    0.0%    0.0%    0.0%    0.0%    0.0%    0.0%    0.0%    0.0%    0.0%    0.0%    0.0%    0.0%    0.0%    0.0%    0.0%    0.0%    0.0%    0.0%    0.0%    0.0%    0.0%    0.0%    0.0%    0.0%    0.0%    0.0%    0.0%    0.0%    0.0%    0.0%    0.0%    0.0%    0.0%    0.0%    0.0%    0.0%    0.0%    0.0%    0.0%    0.0%    0.0%    0.0%    0.0%    0.0%    0.0%    0.0%    0.0%    0.0%    0.0%    0.0%    0.0%    0.0%    0.0%    0.0%    0.0%    0.0%    0.0%    0.0%    0.0%    0.0%    0.0%    0.0%    0.0%    0.0%    0.0%    0.0%    0.0%    0.0%    0.0%    0.0%    0.0%    0.0%    0.0%    0.0%    0.0%    0.0%    0.0%    0.0%    0.0%    0.0%    0.0%    0.0%    0.0%    0.0%    0.0%    0.0%    0.0%    0.0%    0.0%    0.0%    0.0%    0.0%    0.0%    0.0%    0.0%    0.0%    0.0%    0.0%    0.0%    0.0%    0.0%    0.0%    0.0%    0.0%    0.0%    0.0%    0.0%    0.0%    0.0%    0.0%    0.0%    0.0%    0.0%    0.0%    0.0%    0.0%    0.0%    0.0%    0.0%    0.0%    0.0%    0.0%    0.0%    0.0%    0.0%    0.0%    0.0%    0.0%    0.0%    0.0%    0.0%    0.0%    0.0%    0.0%    0.0%    0.0%    0.0%    0.0%    0.0%    0.0%        k__Bacteria;p__Chloroflexi;c__Anaerolineae; o__Anaerolineales      27    0.0%    0.0%    0.0%    0.0%    0.0%    0.0%    0.0%    0.0%    0.0%    0.0%    0.0%    0.0%    0.0%    0.0%    0.0%    0.0%    0.0%    0.0%    0.0%    0.0%    0.0%    0.0%    0.0%    0.0%    0.0%    0.0%    0.0%    0.0%    0.0%    0.0%    0.0%    0.0%    0.0%    0.0%    0.0%    0.0%    0.0%    0.0%    0.0%    0.0%    0.0%    0.0%    0.0%    0.0%    0.0%    0.0%    0.0%    0.0%    0.0%    0.0%    0.0%    0.0%    0.0%    0.0%    0.0%    0.0%    0.0%    0.0%    0.0%    0.0%    0.0%    0.0%    0.0%    0.0%    0.0%    0.0%    0.0%    0.0%    0.0%    0.0%    0.0%    0.0%    0.0%    0.0%    0.0%    0.0%    0.0%    0.0%    0.0%    0.0%    0.0%    0.0%    0.0%    0.0%    0.0%    0.0%    0.0%    0.0%    0.0%    0.0%    0.0%    0.0%    0.0%    0.0%    0.0%    0.0%    0.0%    0.0%    0.0%    0.0%    0.0%    0.0%    0.0%    0.0%    0.0%    0.0%    0.0%    0.0%    0.0%    0.0%    0.0%    0.0%    0.0%    0.0%    0.0%    0.0%    0.0%    0.0%    0.0%    0.0%    0.0%    0.0%    0.0%    0.0%    0.0%    0.0%    0.0%    0.0%    0.0%    0.0%    0.0%    0.0%    0.0%    0.0%    0.0%    0.0%    0.0%    0.0%    0.0%    0.0%    0.0%    0.0%    0.0%    0.0%    0.0%    0.0%    0.0%    0.0%    0.0%    0.0%    0.0%    0.0%    0.0%    0.0%    0.0%    0.0%    0.0%    0.0%    0.0%    0.0%    0.0%    0.0%    0.0%    0.0%    0.0%    0.0%    0.0%    0.0%    0.0%    0.0%    0.0%    0.0%    0.0%    0.0%    0.0%    0.0%    0.0%    0.0%    0.0%    0.0%    0.0%    0.0%    0.0%    0.0%    0.0%    0.0%    0.0%    0.0%    0.0%    0.0%    0.0%    0.0%    0.0%    0.0%    0.0%    0.0%    0.0%    0.0%    0.0%    0.0%    0.0%    0.0%    0.0%    0.0%    0.0%    0.0%    0.0%    0.0%    0.0%    0.0%    0.0%    0.0%    0.0%    0.0%    0.0%    0.0%    0.0%    0.0%    0.0%    0.0%    0.0%    0.0%    0.0%    0.0%    0.0%    0.0%    0.0%    0.0%    0.0%    0.0%    0.0%    0.0%    0.0%    0.0%    0.0%    0.0%    0.0%    0.0%    0.0%    0.0%    0.0%    0.0%    0.0%    0.0%    0.0%    0.0%    0.0%    0.0%    0.0%    0.0%    0.0%    0.0%    0.0%    0.0%    0.0%    0.0%    0.0%    0.0%    0.0%    0.0%    0.0%    0.0%    0.0%    0.0%    0.0%    0.0%    0.0%    0.0%    0.0%    0.0%    0.0%    0.0%    0.0%    0.0%    0.0%    0.0%    0.0%    0.0%    0.0%    0.0%    0.0%    0.0%    0.0%    0.0%    0.0%    0.0%    0.0%    0.0%    0.0%    0.0%    0.0%    0.0%    0.0%    0.0%    0.0%    0.0%    0.0%    0.0%    0.0%    0.0%    0.0%    0.0%    0.0%    0.0%    0.0%    0.0%    0.0%    0.0%    0.0%    0.0%    0.0%    0.0%    0.0%    0.0%    0.0%    0.0%    0.0%    0.0%    0.0%    0.0%    0.0%    0.0%    0.0%    0.0%    0.0%    0.0%    0.0%    0.0%    0.0%    0.0%    0.0%    0.0%    0.0%    0.0%    0.0%    0.0%    0.0%    0.0%    0.0%    0.0%    0.0%    0.0%    0.0%    0.0%    0.0%    0.0%    0.0%    0.0%    0.0%    0.0%    0.0%    0.0%    0.0%    0.0%    0.0%    0.0%    0.0%    0.0%        k__Bacteria;p__Chloroflexi;c__Anaerolineae; o__CFB-26      17    0.0%    0.0%    0.0%    0.0%    0.0%    0.0%    0.0%    0.0%    0.0%    0.0%    0.0%    0.0%    0.0%    0.0%    0.0%    0.0%    0.0%    0.0%    0.0%    0.0%    0.0%    0.0%    0.0%    0.0%    0.0%    0.0%    0.0%    0.0%    0.0%    0.0%    0.0%    0.0%    0.0%    0.0%    0.0%    0.0%    0.0%    0.0%    0.0%    0.0%    0.0%    0.0%    0.0%    0.0%    0.0%    0.0%    0.0%    0.0%    0.0%    0.0%    0.0%    0.0%    0.0%    0.0%    0.0%    0.0%    0.0%    0.0%    0.0%    0.0%    0.0%    0.0%    0.0%    0.0%    0.0%    0.0%    0.0%    0.0%    0.0%    0.0%    0.0%    0.0%    0.0%    0.0%    0.0%    0.0%    0.0%    0.0%    0.0%    0.0%    0.0%    0.0%    0.0%    0.0%    0.0%    0.0%    0.0%    0.0%    0.0%    0.0%    0.0%    0.0%    0.0%    0.0%    0.0%    0.0%    0.0%    0.0%    0.0%    0.0%    0.0%    0.0%    0.0%    0.0%    0.0%    0.0%    0.0%    0.0%    0.0%    0.0%    0.0%    0.0%    0.0%    0.0%    0.0%    0.0%    0.0%    0.0%    0.0%    0.0%    0.0%    0.0%    0.0%    0.0%    0.0%    0.0%    0.0%    0.0%    0.0%    0.0%    0.0%    0.0%    0.0%    0.0%    0.0%    0.0%    0.0%    0.0%    0.0%    0.0%    0.0%    0.0%    0.0%    0.0%    0.0%    0.0%    0.0%    0.0%    0.0%    0.0%    0.0%    0.0%    0.0%    0.0%    0.0%    0.0%    0.0%    0.0%    0.0%    0.0%    0.0%    0.0%    0.0%    0.0%    0.0%    0.0%    0.0%    0.0%    0.0%    0.0%    0.0%    0.0%    0.0%    0.0%    0.0%    0.0%    0.0%    0.0%    0.0%    0.0%    0.0%    0.0%    0.0%    0.0%    0.0%    0.0%    0.0%    0.0%    0.0%    0.0%    0.0%    0.0%    0.0%    0.0%    0.0%    0.0%    0.0%    0.0%    0.0%    0.0%    0.0%    0.0%    0.0%    0.0%    0.0%    0.0%    0.0%    0.0%    0.0%    0.0%    0.0%    0.0%    0.0%    0.0%    0.0%    0.0%    0.0%    0.0%    0.0%    0.0%    0.0%    0.0%    0.0%    0.0%    0.0%    0.0%    0.0%    0.0%    0.0%    0.0%    0.0%    0.0%    0.0%    0.0%    0.0%    0.0%    0.0%    0.0%    0.0%    0.0%    0.0%    0.0%    0.0%    0.0%    0.0%    0.0%    0.0%    0.0%    0.0%    0.0%    0.0%    0.0%    0.0%    0.0%    0.0%    0.0%    0.0%    0.0%    0.0%    0.0%    0.0%    0.0%    0.0%    0.0%    0.0%    0.0%    0.0%    0.0%    0.0%    0.0%    0.0%    0.0%    0.0%    0.0%    0.0%    0.0%    0.0%    0.0%    0.0%    0.0%    0.0%    0.0%    0.0%    0.0%    0.0%    0.0%    0.0%    0.0%    0.0%    0.0%    0.0%    0.0%    0.0%    0.0%    0.0%    0.0%    0.0%    0.0%    0.0%    0.0%    0.0%    0.0%    0.0%    0.0%    0.0%    0.0%    0.0%    0.0%    0.0%    0.0%    0.0%    0.0%    0.0%    0.0%    0.0%    0.0%    0.0%    0.0%    0.0%    0.0%    0.0%    0.0%    0.0%    0.0%    0.0%    0.0%    0.0%    0.0%    0.0%    0.0%    0.0%    0.0%    0.0%    0.0%    0.0%    0.0%    0.0%    0.0%    0.0%    0.0%    0.0%    0.0%    0.0%    0.0%    0.0%    0.0%    0.0%    0.0%    0.0%    0.0%    0.0%    0.0%    0.0%    0.0%    0.0%    0.0%    0.0%    0.0%        k__Bacteria;p__Chloroflexi;c__Anaerolineae; o__Caldilineales     344    0.0%    0.0%    0.0%    0.0%    0.0%    0.0%    0.0%    0.0%    0.0%    0.0%    0.0%    0.0%    0.0%    0.0%    0.0%    0.0%    0.0%    0.0%    0.0%    0.0%    0.0%    0.0%    0.0%    0.0%    0.0%    0.0%    0.0%    0.0%    0.0%    0.0%    0.0%    0.0%    0.0%    0.0%    0.0%    0.0%    0.0%    0.0%    0.0%    0.0%    0.0%    0.0%    0.0%    0.0%    0.0%    0.0%    0.0%    0.0%    0.0%    0.0%    0.0%    0.0%    0.0%    0.0%    0.0%    0.0%    0.0%    0.0%    0.0%    0.0%    0.0%    0.0%    0.0%    0.0%    0.0%    0.0%    0.0%    0.0%    0.0%    0.0%    0.0%    0.0%    0.0%    0.0%    0.0%    0.0%    0.0%    0.0%    0.0%    0.0%    0.0%    0.0%    0.0%    0.0%    0.0%    0.0%    0.0%    0.0%    0.0%    0.0%    0.0%    0.0%    0.0%    0.0%    0.0%    0.0%    0.0%    0.0%    0.0%    0.0%    0.0%    0.0%    0.0%    0.0%    0.0%    0.0%    0.0%    0.0%    0.0%    0.0%    0.0%    0.0%    0.0%    0.0%    0.0%    0.0%    0.0%    0.0%    0.0%    0.0%    0.0%    0.0%    0.0%    0.0%    0.0%    0.0%    0.0%    0.0%    0.0%    0.0%    0.0%    0.0%    0.0%    0.0%    0.0%    0.0%    0.0%    0.0%    0.0%    0.0%    0.0%    0.0%    0.0%    0.0%    0.0%    0.0%    0.0%    0.0%    0.0%    0.0%    0.0%    0.0%    0.0%    0.0%    0.0%    0.0%    0.0%    0.0%    0.0%    0.0%    0.0%    0.0%    0.0%    0.0%    0.0%    0.0%    0.0%    0.0%    0.0%    0.0%    0.0%    0.0%    0.0%    0.0%    0.0%    0.0%    0.0%    0.0%    0.0%    0.0%    0.0%    0.0%    0.0%    0.0%    0.0%    0.0%    0.0%    0.0%    0.0%    0.0%    0.0%    0.0%    0.0%    0.0%    0.0%    0.0%    0.0%    0.0%    0.0%    0.0%    0.0%    0.0%    0.0%    0.0%    0.0%    0.0%    0.0%    0.0%    0.0%    0.0%    0.0%    0.0%    0.0%    0.0%    0.0%    0.0%    0.0%    0.0%    0.0%    0.0%    0.0%    0.0%    0.0%    0.0%    0.0%    0.0%    0.0%    0.0%    0.0%    0.0%    0.0%    0.0%    0.0%    0.0%    0.0%    0.0%    0.0%    0.0%    0.0%    0.0%    0.0%    0.0%    0.0%    0.0%    0.0%    0.0%    0.0%    0.0%    0.0%    0.0%    0.0%    0.0%    0.0%    0.0%    0.0%    0.0%    0.0%    0.0%    0.0%    0.0%    0.0%    0.0%    0.0%    0.0%    0.0%    0.0%    0.0%    0.0%    0.0%    0.0%    0.0%    0.0%    0.0%    0.0%    0.0%    0.0%    0.0%    0.0%    0.0%    0.0%    0.0%    0.0%    0.0%    0.0%    0.0%    0.0%    0.0%    0.0%    0.0%    0.0%    0.0%    0.0%    0.0%    0.0%    0.0%    0.0%    0.0%    0.0%    0.0%    0.0%    0.0%    0.0%    0.0%    0.0%    0.0%    0.0%    0.0%    0.0%    0.0%    0.0%    0.0%    0.0%    0.0%    0.0%    0.0%    0.0%    0.0%    0.0%    0.0%    0.0%    0.0%    0.0%    0.0%    0.0%    0.0%    0.0%    0.0%    0.0%    0.0%    0.0%    0.0%    0.0%    0.0%    0.0%    0.0%    0.0%    0.0%    0.0%    0.0%    0.0%    0.0%    0.0%    0.0%    0.0%    0.0%    0.0%    0.0%    0.0%    0.0%    0.0%    0.0%    0.0%    0.0%    0.0%    0.0%    0.0%    0.0%    0.0%        k__Bacteria;p__Chloroflexi;c__Anaerolineae; o__DRC31       9    0.0%    0.0%    0.0%    0.0%    0.0%    0.0%    0.0%    0.0%    0.0%    0.0%    0.0%    0.0%    0.0%    0.0%    0.0%    0.0%    0.0%    0.0%    0.0%    0.0%    0.0%    0.0%    0.0%    0.0%    0.0%    0.0%    0.0%    0.0%    0.0%    0.0%    0.0%    0.0%    0.0%    0.0%    0.0%    0.0%    0.0%    0.0%    0.0%    0.0%    0.0%    0.0%    0.0%    0.0%    0.0%    0.0%    0.0%    0.0%    0.0%    0.0%    0.0%    0.0%    0.0%    0.0%    0.0%    0.0%    0.0%    0.0%    0.0%    0.0%    0.0%    0.0%    0.0%    0.0%    0.0%    0.0%    0.0%    0.0%    0.0%    0.0%    0.0%    0.0%    0.0%    0.0%    0.0%    0.0%    0.0%    0.0%    0.0%    0.0%    0.0%    0.0%    0.0%    0.0%    0.0%    0.0%    0.0%    0.0%    0.0%    0.0%    0.0%    0.0%    0.0%    0.0%    0.0%    0.0%    0.0%    0.0%    0.0%    0.0%    0.0%    0.0%    0.0%    0.0%    0.0%    0.0%    0.0%    0.0%    0.0%    0.0%    0.0%    0.0%    0.0%    0.0%    0.0%    0.0%    0.0%    0.0%    0.0%    0.0%    0.0%    0.0%    0.0%    0.0%    0.0%    0.0%    0.0%    0.0%    0.0%    0.0%    0.0%    0.0%    0.0%    0.0%    0.0%    0.0%    0.0%    0.0%    0.0%    0.0%    0.0%    0.0%    0.0%    0.0%    0.0%    0.0%    0.0%    0.0%    0.0%    0.0%    0.0%    0.0%    0.0%    0.0%    0.0%    0.0%    0.0%    0.0%    0.0%    0.0%    0.0%    0.0%    0.0%    0.0%    0.0%    0.0%    0.0%    0.0%    0.0%    0.0%    0.0%    0.0%    0.0%    0.0%    0.0%    0.0%    0.0%    0.0%    0.0%    0.0%    0.0%    0.0%    0.0%    0.0%    0.0%    0.0%    0.0%    0.0%    0.0%    0.0%    0.0%    0.0%    0.0%    0.0%    0.0%    0.0%    0.0%    0.0%    0.0%    0.0%    0.0%    0.0%    0.0%    0.0%    0.0%    0.0%    0.0%    0.0%    0.0%    0.0%    0.0%    0.0%    0.0%    0.0%    0.0%    0.0%    0.0%    0.0%    0.0%    0.0%    0.0%    0.0%    0.0%    0.0%    0.0%    0.0%    0.0%    0.0%    0.0%    0.0%    0.0%    0.0%    0.0%    0.0%    0.0%    0.0%    0.0%    0.0%    0.0%    0.0%    0.0%    0.0%    0.0%    0.0%    0.0%    0.0%    0.0%    0.0%    0.0%    0.0%    0.0%    0.0%    0.0%    0.0%    0.0%    0.0%    0.0%    0.0%    0.0%    0.0%    0.0%    0.0%    0.0%    0.0%    0.0%    0.0%    0.0%    0.0%    0.0%    0.0%    0.0%    0.0%    0.0%    0.0%    0.0%    0.0%    0.0%    0.0%    0.0%    0.0%    0.0%    0.0%    0.0%    0.0%    0.0%    0.0%    0.0%    0.0%    0.0%    0.0%    0.0%    0.0%    0.0%    0.0%    0.0%    0.0%    0.0%    0.0%    0.0%    0.0%    0.0%    0.0%    0.0%    0.0%    0.0%    0.0%    0.0%    0.0%    0.0%    0.0%    0.0%    0.0%    0.0%    0.0%    0.0%    0.0%    0.0%    0.0%    0.0%    0.0%    0.0%    0.0%    0.0%    0.0%    0.0%    0.0%    0.0%    0.0%    0.0%    0.0%    0.0%    0.0%    0.0%    0.0%    0.0%    0.0%    0.0%    0.0%    0.0%    0.0%    0.0%    0.0%    0.0%    0.0%    0.0%    0.0%    0.0%    0.0%    0.0%    0.0%    0.0%    0.0%    0.0%    0.0%    0.0%    0.0%    0.0%    0.0%        k__Bacteria;p__Chloroflexi;c__Anaerolineae; o__GCA004       0    0.0%    0.0%    0.0%    0.0%    0.0%    0.0%    0.0%    0.0%    0.0%    0.0%    0.0%    0.0%    0.0%    0.0%    0.0%    0.0%    0.0%    0.0%    0.0%    0.0%    0.0%    0.0%    0.0%    0.0%    0.0%    0.0%    0.0%    0.0%    0.0%    0.0%    0.0%    0.0%    0.0%    0.0%    0.0%    0.0%    0.0%    0.0%    0.0%    0.0%    0.0%    0.0%    0.0%    0.0%    0.0%    0.0%    0.0%    0.0%    0.0%    0.0%    0.0%    0.0%    0.0%    0.0%    0.0%    0.0%    0.0%    0.0%    0.0%    0.0%    0.0%    0.0%    0.0%    0.0%    0.0%    0.0%    0.0%    0.0%    0.0%    0.0%    0.0%    0.0%    0.0%    0.0%    0.0%    0.0%    0.0%    0.0%    0.0%    0.0%    0.0%    0.0%    0.0%    0.0%    0.0%    0.0%    0.0%    0.0%    0.0%    0.0%    0.0%    0.0%    0.0%    0.0%    0.0%    0.0%    0.0%    0.0%    0.0%    0.0%    0.0%    0.0%    0.0%    0.0%    0.0%    0.0%    0.0%    0.0%    0.0%    0.0%    0.0%    0.0%    0.0%    0.0%    0.0%    0.0%    0.0%    0.0%    0.0%    0.0%    0.0%    0.0%    0.0%    0.0%    0.0%    0.0%    0.0%    0.0%    0.0%    0.0%    0.0%    0.0%    0.0%    0.0%    0.0%    0.0%    0.0%    0.0%    0.0%    0.0%    0.0%    0.0%    0.0%    0.0%    0.0%    0.0%    0.0%    0.0%    0.0%    0.0%    0.0%    0.0%    0.0%    0.0%    0.0%    0.0%    0.0%    0.0%    0.0%    0.0%    0.0%    0.0%    0.0%    0.0%    0.0%    0.0%    0.0%    0.0%    0.0%    0.0%    0.0%    0.0%    0.0%    0.0%    0.0%    0.0%    0.0%    0.0%    0.0%    0.0%    0.0%    0.0%    0.0%    0.0%    0.0%    0.0%    0.0%    0.0%    0.0%    0.0%    0.0%    0.0%    0.0%    0.0%    0.0%    0.0%    0.0%    0.0%    0.0%    0.0%    0.0%    0.0%    0.0%    0.0%    0.0%    0.0%    0.0%    0.0%    0.0%    0.0%    0.0%    0.0%    0.0%    0.0%    0.0%    0.0%    0.0%    0.0%    0.0%    0.0%    0.0%    0.0%    0.0%    0.0%    0.0%    0.0%    0.0%    0.0%    0.0%    0.0%    0.0%    0.0%    0.0%    0.0%    0.0%    0.0%    0.0%    0.0%    0.0%    0.0%    0.0%    0.0%    0.0%    0.0%    0.0%    0.0%    0.0%    0.0%    0.0%    0.0%    0.0%    0.0%    0.0%    0.0%    0.0%    0.0%    0.0%    0.0%    0.0%    0.0%    0.0%    0.0%    0.0%    0.0%    0.0%    0.0%    0.0%    0.0%    0.0%    0.0%    0.0%    0.0%    0.0%    0.0%    0.0%    0.0%    0.0%    0.0%    0.0%    0.0%    0.0%    0.0%    0.0%    0.0%    0.0%    0.0%    0.0%    0.0%    0.0%    0.0%    0.0%    0.0%    0.0%    0.0%    0.0%    0.0%    0.0%    0.0%    0.0%    0.0%    0.0%    0.0%    0.0%    0.0%    0.0%    0.0%    0.0%    0.0%    0.0%    0.0%    0.0%    0.0%    0.0%    0.0%    0.0%    0.0%    0.0%    0.0%    0.0%    0.0%    0.0%    0.0%    0.0%    0.0%    0.0%    0.0%    0.0%    0.0%    0.0%    0.0%    0.0%    0.0%    0.0%    0.0%    0.0%    0.0%    0.0%    0.0%    0.0%    0.0%    0.0%    0.0%    0.0%    0.0%    0.0%    0.0%    0.0%    0.0%    0.0%    0.0%    0.0%    0.0%    0.0%    0.0%    0.0%    0.0%    0.0%    0.0%        k__Bacteria;p__Chloroflexi;c__Anaerolineae; o__H39       5    0.0%    0.0%    0.0%    0.0%    0.0%    0.0%    0.0%    0.0%    0.0%    0.0%    0.0%    0.0%    0.0%    0.0%    0.0%    0.0%    0.0%    0.0%    0.0%    0.0%    0.0%    0.0%    0.0%    0.0%    0.0%    0.0%    0.0%    0.0%    0.0%    0.0%    0.0%    0.0%    0.0%    0.0%    0.0%    0.0%    0.0%    0.0%    0.0%    0.0%    0.0%    0.0%    0.0%    0.0%    0.0%    0.0%    0.0%    0.0%    0.0%    0.0%    0.0%    0.0%    0.0%    0.0%    0.0%    0.0%    0.0%    0.0%    0.0%    0.0%    0.0%    0.0%    0.0%    0.0%    0.0%    0.0%    0.0%    0.0%    0.0%    0.0%    0.0%    0.0%    0.0%    0.0%    0.0%    0.0%    0.0%    0.0%    0.0%    0.0%    0.0%    0.0%    0.0%    0.0%    0.0%    0.0%    0.0%    0.0%    0.0%    0.0%    0.0%    0.0%    0.0%    0.0%    0.0%    0.0%    0.0%    0.0%    0.0%    0.0%    0.0%    0.0%    0.0%    0.0%    0.0%    0.0%    0.0%    0.0%    0.0%    0.0%    0.0%    0.0%    0.0%    0.0%    0.0%    0.0%    0.0%    0.0%    0.0%    0.0%    0.0%    0.0%    0.0%    0.0%    0.0%    0.0%    0.0%    0.0%    0.0%    0.0%    0.0%    0.0%    0.0%    0.0%    0.0%    0.0%    0.0%    0.0%    0.0%    0.0%    0.0%    0.0%    0.0%    0.0%    0.0%    0.0%    0.0%    0.0%    0.0%    0.0%    0.0%    0.0%    0.0%    0.0%    0.0%    0.0%    0.0%    0.0%    0.0%    0.0%    0.0%    0.0%    0.0%    0.0%    0.0%    0.0%    0.0%    0.0%    0.0%    0.0%    0.0%    0.0%    0.0%    0.0%    0.0%    0.0%    0.0%    0.0%    0.0%    0.0%    0.0%    0.0%    0.0%    0.0%    0.0%    0.0%    0.0%    0.0%    0.0%    0.0%    0.0%    0.0%    0.0%    0.0%    0.0%    0.0%    0.0%    0.0%    0.0%    0.0%    0.0%    0.0%    0.0%    0.0%    0.0%    0.0%    0.0%    0.0%    0.0%    0.0%    0.0%    0.0%    0.0%    0.0%    0.0%    0.0%    0.0%    0.0%    0.0%    0.0%    0.0%    0.0%    0.0%    0.0%    0.0%    0.0%    0.0%    0.0%    0.0%    0.0%    0.0%    0.0%    0.0%    0.0%    0.0%    0.0%    0.0%    0.0%    0.0%    0.0%    0.0%    0.0%    0.0%    0.0%    0.0%    0.0%    0.0%    0.0%    0.0%    0.0%    0.0%    0.0%    0.0%    0.0%    0.0%    0.0%    0.0%    0.0%    0.0%    0.0%    0.0%    0.0%    0.0%    0.0%    0.0%    0.0%    0.0%    0.0%    0.0%    0.0%    0.0%    0.0%    0.0%    0.0%    0.0%    0.0%    0.0%    0.0%    0.0%    0.0%    0.0%    0.0%    0.0%    0.0%    0.0%    0.0%    0.0%    0.0%    0.0%    0.0%    0.0%    0.0%    0.0%    0.0%    0.0%    0.0%    0.0%    0.0%    0.0%    0.0%    0.0%    0.0%    0.0%    0.0%    0.0%    0.0%    0.0%    0.0%    0.0%    0.0%    0.0%    0.0%    0.0%    0.0%    0.0%    0.0%    0.0%    0.0%    0.0%    0.0%    0.0%    0.0%    0.0%    0.0%    0.0%    0.0%    0.0%    0.0%    0.0%    0.0%    0.0%    0.0%    0.0%    0.0%    0.0%    0.0%    0.0%    0.0%    0.0%    0.0%    0.0%    0.0%    0.0%    0.0%    0.0%    0.0%    0.0%    0.0%    0.0%    0.0%    0.0%    0.0%    0.0%    0.0%    0.0%    0.0%    0.0%    0.0%        k__Bacteria;p__Chloroflexi;c__Anaerolineae; o__S0208       4    0.0%    0.0%    0.0%    0.0%    0.0%    0.0%    0.0%    0.0%    0.0%    0.0%    0.0%    0.0%    0.0%    0.0%    0.0%    0.0%    0.0%    0.0%    0.0%    0.0%    0.0%    0.0%    0.0%    0.0%    0.0%    0.0%    0.0%    0.0%    0.0%    0.0%    0.0%    0.0%    0.0%    0.0%    0.0%    0.0%    0.0%    0.0%    0.0%    0.0%    0.0%    0.0%    0.0%    0.0%    0.0%    0.0%    0.0%    0.0%    0.0%    0.0%    0.0%    0.0%    0.0%    0.0%    0.0%    0.0%    0.0%    0.0%    0.0%    0.0%    0.0%    0.0%    0.0%    0.0%    0.0%    0.0%    0.0%    0.0%    0.0%    0.0%    0.0%    0.0%    0.0%    0.0%    0.0%    0.0%    0.0%    0.0%    0.0%    0.0%    0.0%    0.0%    0.0%    0.0%    0.0%    0.0%    0.0%    0.0%    0.0%    0.0%    0.0%    0.0%    0.0%    0.0%    0.0%    0.0%    0.0%    0.0%    0.0%    0.0%    0.0%    0.0%    0.0%    0.0%    0.0%    0.0%    0.0%    0.0%    0.0%    0.0%    0.0%    0.0%    0.0%    0.0%    0.0%    0.0%    0.0%    0.0%    0.0%    0.0%    0.0%    0.0%    0.0%    0.0%    0.0%    0.0%    0.0%    0.0%    0.0%    0.0%    0.0%    0.0%    0.0%    0.0%    0.0%    0.0%    0.0%    0.0%    0.0%    0.0%    0.0%    0.0%    0.0%    0.0%    0.0%    0.0%    0.0%    0.0%    0.0%    0.0%    0.0%    0.0%    0.0%    0.0%    0.0%    0.0%    0.0%    0.0%    0.0%    0.0%    0.0%    0.0%    0.0%    0.0%    0.0%    0.0%    0.0%    0.0%    0.0%    0.0%    0.0%    0.0%    0.0%    0.0%    0.0%    0.0%    0.0%    0.0%    0.0%    0.0%    0.0%    0.0%    0.0%    0.0%    0.0%    0.0%    0.0%    0.0%    0.0%    0.0%    0.0%    0.0%    0.0%    0.0%    0.0%    0.0%    0.0%    0.0%    0.0%    0.0%    0.0%    0.0%    0.0%    0.0%    0.0%    0.0%    0.0%    0.0%    0.0%    0.0%    0.0%    0.0%    0.0%    0.0%    0.0%    0.0%    0.0%    0.0%    0.0%    0.0%    0.0%    0.0%    0.0%    0.0%    0.0%    0.0%    0.0%    0.0%    0.0%    0.0%    0.0%    0.0%    0.0%    0.0%    0.0%    0.0%    0.0%    0.0%    0.0%    0.0%    0.0%    0.0%    0.0%    0.0%    0.0%    0.0%    0.0%    0.0%    0.0%    0.0%    0.0%    0.0%    0.0%    0.0%    0.0%    0.0%    0.0%    0.0%    0.0%    0.0%    0.0%    0.0%    0.0%    0.0%    0.0%    0.0%    0.0%    0.0%    0.0%    0.0%    0.0%    0.0%    0.0%    0.0%    0.0%    0.0%    0.0%    0.0%    0.0%    0.0%    0.0%    0.0%    0.0%    0.0%    0.0%    0.0%    0.0%    0.0%    0.0%    0.0%    0.0%    0.0%    0.0%    0.0%    0.0%    0.0%    0.0%    0.0%    0.0%    0.0%    0.0%    0.0%    0.0%    0.0%    0.0%    0.0%    0.0%    0.0%    0.0%    0.0%    0.0%    0.0%    0.0%    0.0%    0.0%    0.0%    0.0%    0.0%    0.0%    0.0%    0.0%    0.0%    0.0%    0.0%    0.0%    0.0%    0.0%    0.0%    0.0%    0.0%    0.0%    0.0%    0.0%    0.0%    0.0%    0.0%    0.0%    0.0%    0.0%    0.0%    0.0%    0.0%    0.0%    0.0%    0.0%    0.0%    0.0%    0.0%    0.0%    0.0%    0.0%    0.0%    0.0%    0.0%    0.0%    0.0%    0.0%    0.0%        k__Bacteria;p__Chloroflexi;c__Anaerolineae; o__SB-34       0    0.0%    0.0%    0.0%    0.0%    0.0%    0.0%    0.0%    0.0%    0.0%    0.0%    0.0%    0.0%    0.0%    0.0%    0.0%    0.0%    0.0%    0.0%    0.0%    0.0%    0.0%    0.0%    0.0%    0.0%    0.0%    0.0%    0.0%    0.0%    0.0%    0.0%    0.0%    0.0%    0.0%    0.0%    0.0%    0.0%    0.0%    0.0%    0.0%    0.0%    0.0%    0.0%    0.0%    0.0%    0.0%    0.0%    0.0%    0.0%    0.0%    0.0%    0.0%    0.0%    0.0%    0.0%    0.0%    0.0%    0.0%    0.0%    0.0%    0.0%    0.0%    0.0%    0.0%    0.0%    0.0%    0.0%    0.0%    0.0%    0.0%    0.0%    0.0%    0.0%    0.0%    0.0%    0.0%    0.0%    0.0%    0.0%    0.0%    0.0%    0.0%    0.0%    0.0%    0.0%    0.0%    0.0%    0.0%    0.0%    0.0%    0.0%    0.0%    0.0%    0.0%    0.0%    0.0%    0.0%    0.0%    0.0%    0.0%    0.0%    0.0%    0.0%    0.0%    0.0%    0.0%    0.0%    0.0%    0.0%    0.0%    0.0%    0.0%    0.0%    0.0%    0.0%    0.0%    0.0%    0.0%    0.0%    0.0%    0.0%    0.0%    0.0%    0.0%    0.0%    0.0%    0.0%    0.0%    0.0%    0.0%    0.0%    0.0%    0.0%    0.0%    0.0%    0.0%    0.0%    0.0%    0.0%    0.0%    0.0%    0.0%    0.0%    0.0%    0.0%    0.0%    0.0%    0.0%    0.0%    0.0%    0.0%    0.0%    0.0%    0.0%    0.0%    0.0%    0.0%    0.0%    0.0%    0.0%    0.0%    0.0%    0.0%    0.0%    0.0%    0.0%    0.0%    0.0%    0.0%    0.0%    0.0%    0.0%    0.0%    0.0%    0.0%    0.0%    0.0%    0.0%    0.0%    0.0%    0.0%    0.0%    0.0%    0.0%    0.0%    0.0%    0.0%    0.0%    0.0%    0.0%    0.0%    0.0%    0.0%    0.0%    0.0%    0.0%    0.0%    0.0%    0.0%    0.0%    0.0%    0.0%    0.0%    0.0%    0.0%    0.0%    0.0%    0.0%    0.0%    0.0%    0.0%    0.0%    0.0%    0.0%    0.0%    0.0%    0.0%    0.0%    0.0%    0.0%    0.0%    0.0%    0.0%    0.0%    0.0%    0.0%    0.0%    0.0%    0.0%    0.0%    0.0%    0.0%    0.0%    0.0%    0.0%    0.0%    0.0%    0.0%    0.0%    0.0%    0.0%    0.0%    0.0%    0.0%    0.0%    0.0%    0.0%    0.0%    0.0%    0.0%    0.0%    0.0%    0.0%    0.0%    0.0%    0.0%    0.0%    0.0%    0.0%    0.0%    0.0%    0.0%    0.0%    0.0%    0.0%    0.0%    0.0%    0.0%    0.0%    0.0%    0.0%    0.0%    0.0%    0.0%    0.0%    0.0%    0.0%    0.0%    0.0%    0.0%    0.0%    0.0%    0.0%    0.0%    0.0%    0.0%    0.0%    0.0%    0.0%    0.0%    0.0%    0.0%    0.0%    0.0%    0.0%    0.0%    0.0%    0.0%    0.0%    0.0%    0.0%    0.0%    0.0%    0.0%    0.0%    0.0%    0.0%    0.0%    0.0%    0.0%    0.0%    0.0%    0.0%    0.0%    0.0%    0.0%    0.0%    0.0%    0.0%    0.0%    0.0%    0.0%    0.0%    0.0%    0.0%    0.0%    0.0%    0.0%    0.0%    0.0%    0.0%    0.0%    0.0%    0.0%    0.0%    0.0%    0.0%    0.0%    0.0%    0.0%    0.0%    0.0%    0.0%    0.0%    0.0%    0.0%    0.0%    0.0%    0.0%    0.0%    0.0%    0.0%    0.0%    0.0%    0.0%    0.0%    0.0%    0.0%    0.0%        k__Bacteria;p__Chloroflexi;c__Anaerolineae; o__SJA-101       9    0.0%    0.0%    0.0%    0.0%    0.0%    0.0%    0.0%    0.0%    0.0%    0.0%    0.0%    0.0%    0.0%    0.0%    0.0%    0.0%    0.0%    0.0%    0.0%    0.0%    0.0%    0.0%    0.0%    0.0%    0.0%    0.0%    0.0%    0.0%    0.0%    0.0%    0.0%    0.0%    0.0%    0.0%    0.0%    0.0%    0.0%    0.0%    0.0%    0.0%    0.0%    0.0%    0.0%    0.0%    0.0%    0.0%    0.0%    0.0%    0.0%    0.0%    0.0%    0.0%    0.0%    0.0%    0.0%    0.0%    0.0%    0.0%    0.0%    0.0%    0.0%    0.0%    0.0%    0.0%    0.0%    0.0%    0.0%    0.0%    0.0%    0.0%    0.0%    0.0%    0.0%    0.0%    0.0%    0.0%    0.0%    0.0%    0.0%    0.0%    0.0%    0.0%    0.0%    0.0%    0.0%    0.0%    0.0%    0.0%    0.0%    0.0%    0.0%    0.0%    0.0%    0.0%    0.0%    0.0%    0.0%    0.0%    0.0%    0.0%    0.0%    0.0%    0.0%    0.0%    0.0%    0.0%    0.0%    0.0%    0.0%    0.0%    0.0%    0.0%    0.0%    0.0%    0.0%    0.0%    0.0%    0.0%    0.0%    0.0%    0.0%    0.0%    0.0%    0.0%    0.0%    0.0%    0.0%    0.0%    0.0%    0.0%    0.0%    0.0%    0.0%    0.0%    0.0%    0.0%    0.0%    0.0%    0.0%    0.0%    0.0%    0.0%    0.0%    0.0%    0.0%    0.0%    0.0%    0.0%    0.0%    0.0%    0.0%    0.0%    0.0%    0.0%    0.0%    0.0%    0.0%    0.0%    0.0%    0.0%    0.0%    0.0%    0.0%    0.0%    0.0%    0.0%    0.0%    0.0%    0.0%    0.0%    0.0%    0.0%    0.0%    0.0%    0.0%    0.0%    0.0%    0.0%    0.0%    0.0%    0.0%    0.0%    0.0%    0.0%    0.0%    0.0%    0.0%    0.0%    0.0%    0.0%    0.0%    0.0%    0.0%    0.0%    0.0%    0.0%    0.0%    0.0%    0.0%    0.0%    0.0%    0.0%    0.0%    0.0%    0.0%    0.0%    0.0%    0.0%    0.0%    0.0%    0.0%    0.0%    0.0%    0.0%    0.0%    0.0%    0.0%    0.0%    0.0%    0.0%    0.0%    0.0%    0.0%    0.0%    0.0%    0.0%    0.0%    0.0%    0.0%    0.0%    0.0%    0.0%    0.0%    0.0%    0.0%    0.0%    0.0%    0.0%    0.0%    0.0%    0.0%    0.0%    0.0%    0.0%    0.0%    0.0%    0.0%    0.0%    0.0%    0.0%    0.0%    0.0%    0.0%    0.0%    0.0%    0.0%    0.0%    0.0%    0.0%    0.0%    0.0%    0.0%    0.0%    0.0%    0.0%    0.0%    0.0%    0.0%    0.0%    0.0%    0.0%    0.0%    0.0%    0.0%    0.0%    0.0%    0.0%    0.0%    0.0%    0.0%    0.0%    0.0%    0.0%    0.0%    0.0%    0.0%    0.0%    0.0%    0.0%    0.0%    0.0%    0.0%    0.0%    0.0%    0.0%    0.0%    0.0%    0.0%    0.0%    0.0%    0.0%    0.0%    0.0%    0.0%    0.0%    0.0%    0.0%    0.0%    0.0%    0.0%    0.0%    0.0%    0.0%    0.0%    0.0%    0.0%    0.0%    0.0%    0.0%    0.0%    0.0%    0.0%    0.0%    0.0%    0.0%    0.0%    0.0%    0.0%    0.0%    0.0%    0.0%    0.0%    0.0%    0.0%    0.0%    0.0%    0.0%    0.0%    0.0%    0.0%    0.0%    0.0%    0.0%    0.0%    0.0%    0.0%    0.0%    0.0%    0.0%    0.0%    0.0%    0.0%    0.0%    0.0%    0.0%    0.0%    0.0%    0.0%        k__Bacteria;p__Chloroflexi;c__Anaerolineae; o__SJA-15      18    0.0%    0.0%    0.0%    0.0%    0.0%    0.0%    0.0%    0.0%    0.0%    0.0%    0.0%    0.0%    0.0%    0.0%    0.0%    0.0%    0.0%    0.0%    0.0%    0.0%    0.0%    0.0%    0.0%    0.0%    0.0%    0.0%    0.0%    0.0%    0.0%    0.0%    0.0%    0.0%    0.0%    0.0%    0.0%    0.0%    0.0%    0.0%    0.0%    0.0%    0.0%    0.0%    0.0%    0.0%    0.0%    0.0%    0.0%    0.0%    0.0%    0.0%    0.0%    0.0%    0.0%    0.0%    0.0%    0.0%    0.0%    0.0%    0.0%    0.0%    0.0%    0.0%    0.0%    0.0%    0.0%    0.0%    0.0%    0.0%    0.0%    0.0%    0.0%    0.0%    0.0%    0.0%    0.0%    0.0%    0.0%    0.0%    0.0%    0.0%    0.0%    0.0%    0.0%    0.0%    0.0%    0.0%    0.0%    0.0%    0.0%    0.0%    0.0%    0.0%    0.0%    0.0%    0.0%    0.0%    0.0%    0.0%    0.0%    0.0%    0.0%    0.0%    0.0%    0.0%    0.0%    0.0%    0.0%    0.0%    0.0%    0.0%    0.0%    0.0%    0.0%    0.0%    0.0%    0.0%    0.0%    0.0%    0.0%    0.0%    0.0%    0.0%    0.0%    0.0%    0.0%    0.0%    0.0%    0.0%    0.0%    0.0%    0.0%    0.0%    0.0%    0.0%    0.0%    0.0%    0.0%    0.0%    0.0%    0.0%    0.0%    0.0%    0.0%    0.0%    0.0%    0.0%    0.0%    0.0%    0.0%    0.0%    0.0%    0.0%    0.0%    0.0%    0.0%    0.0%    0.0%    0.0%    0.0%    0.0%    0.0%    0.0%    0.0%    0.0%    0.0%    0.0%    0.0%    0.0%    0.0%    0.0%    0.0%    0.0%    0.0%    0.0%    0.0%    0.0%    0.0%    0.0%    0.0%    0.0%    0.0%    0.0%    0.0%    0.0%    0.0%    0.0%    0.0%    0.0%    0.0%    0.0%    0.0%    0.0%    0.0%    0.0%    0.0%    0.0%    0.0%    0.0%    0.0%    0.0%    0.0%    0.0%    0.0%    0.0%    0.0%    0.0%    0.0%    0.0%    0.0%    0.0%    0.0%    0.0%    0.0%    0.0%    0.0%    0.0%    0.0%    0.0%    0.0%    0.0%    0.0%    0.0%    0.0%    0.0%    0.0%    0.0%    0.0%    0.0%    0.0%    0.0%    0.0%    0.0%    0.0%    0.0%    0.0%    0.0%    0.0%    0.0%    0.0%    0.0%    0.0%    0.0%    0.0%    0.0%    0.0%    0.0%    0.0%    0.0%    0.0%    0.0%    0.0%    0.0%    0.0%    0.0%    0.0%    0.0%    0.0%    0.0%    0.0%    0.0%    0.0%    0.0%    0.0%    0.0%    0.0%    0.0%    0.0%    0.0%    0.0%    0.0%    0.0%    0.0%    0.0%    0.0%    0.0%    0.0%    0.0%    0.0%    0.0%    0.0%    0.0%    0.0%    0.0%    0.0%    0.0%    0.0%    0.0%    0.0%    0.0%    0.0%    0.0%    0.0%    0.0%    0.0%    0.0%    0.0%    0.0%    0.0%    0.0%    0.0%    0.0%    0.0%    0.0%    0.0%    0.0%    0.0%    0.0%    0.0%    0.0%    0.0%    0.0%    0.0%    0.0%    0.0%    0.0%    0.0%    0.0%    0.0%    0.0%    0.0%    0.0%    0.0%    0.0%    0.0%    0.0%    0.0%    0.0%    0.0%    0.0%    0.0%    0.0%    0.0%    0.0%    0.0%    0.0%    0.0%    0.0%    0.0%    0.0%    0.0%    0.0%    0.0%    0.0%    0.0%    0.0%    0.0%    0.0%    0.0%    0.0%    0.0%    0.0%    0.0%    0.0%    0.0%    0.0%    0.0%    0.0%    0.0%        k__Bacteria;p__Chloroflexi;c__Anaerolineae; o__WCHB1-50       4    0.0%    0.0%    0.0%    0.0%    0.0%    0.0%    0.0%    0.0%    0.0%    0.0%    0.0%    0.0%    0.0%    0.0%    0.0%    0.0%    0.0%    0.0%    0.0%    0.0%    0.0%    0.0%    0.0%    0.0%    0.0%    0.0%    0.0%    0.0%    0.0%    0.0%    0.0%    0.0%    0.0%    0.0%    0.0%    0.0%    0.0%    0.0%    0.0%    0.0%    0.0%    0.0%    0.0%    0.0%    0.0%    0.0%    0.0%    0.0%    0.0%    0.0%    0.0%    0.0%    0.0%    0.0%    0.0%    0.0%    0.0%    0.0%    0.0%    0.0%    0.0%    0.0%    0.0%    0.0%    0.0%    0.0%    0.0%    0.0%    0.0%    0.0%    0.0%    0.0%    0.0%    0.0%    0.0%    0.0%    0.0%    0.0%    0.0%    0.0%    0.0%    0.0%    0.0%    0.0%    0.0%    0.0%    0.0%    0.0%    0.0%    0.0%    0.0%    0.0%    0.0%    0.0%    0.0%    0.0%    0.0%    0.0%    0.0%    0.0%    0.0%    0.0%    0.0%    0.0%    0.0%    0.0%    0.0%    0.0%    0.0%    0.0%    0.0%    0.0%    0.0%    0.0%    0.0%    0.0%    0.0%    0.0%    0.0%    0.0%    0.0%    0.0%    0.0%    0.0%    0.0%    0.0%    0.0%    0.0%    0.0%    0.0%    0.0%    0.0%    0.0%    0.0%    0.0%    0.0%    0.0%    0.0%    0.0%    0.0%    0.0%    0.0%    0.0%    0.0%    0.0%    0.0%    0.0%    0.0%    0.0%    0.0%    0.0%    0.0%    0.0%    0.0%    0.0%    0.0%    0.0%    0.0%    0.0%    0.0%    0.0%    0.0%    0.0%    0.0%    0.0%    0.0%    0.0%    0.0%    0.0%    0.0%    0.0%    0.0%    0.0%    0.0%    0.0%    0.0%    0.0%    0.0%    0.0%    0.0%    0.0%    0.0%    0.0%    0.0%    0.0%    0.0%    0.0%    0.0%    0.0%    0.0%    0.0%    0.0%    0.0%    0.0%    0.0%    0.0%    0.0%    0.0%    0.0%    0.0%    0.0%    0.0%    0.0%    0.0%    0.0%    0.0%    0.0%    0.0%    0.0%    0.0%    0.0%    0.0%    0.0%    0.0%    0.0%    0.0%    0.0%    0.0%    0.0%    0.0%    0.0%    0.0%    0.0%    0.0%    0.0%    0.0%    0.0%    0.0%    0.0%    0.0%    0.0%    0.0%    0.0%    0.0%    0.0%    0.0%    0.0%    0.0%    0.0%    0.0%    0.0%    0.0%    0.0%    0.0%    0.0%    0.0%    0.0%    0.0%    0.0%    0.0%    0.0%    0.0%    0.0%    0.0%    0.0%    0.0%    0.0%    0.0%    0.0%    0.0%    0.0%    0.0%    0.0%    0.0%    0.0%    0.0%    0.0%    0.0%    0.0%    0.0%    0.0%    0.0%    0.0%    0.0%    0.0%    0.0%    0.0%    0.0%    0.0%    0.0%    0.0%    0.0%    0.0%    0.0%    0.0%    0.0%    0.0%    0.0%    0.0%    0.0%    0.0%    0.0%    0.0%    0.0%    0.0%    0.0%    0.0%    0.0%    0.0%    0.0%    0.0%    0.0%    0.0%    0.0%    0.0%    0.0%    0.0%    0.0%    0.0%    0.0%    0.0%    0.0%    0.0%    0.0%    0.0%    0.0%    0.0%    0.0%    0.0%    0.0%    0.0%    0.0%    0.0%    0.0%    0.0%    0.0%    0.0%    0.0%    0.0%    0.0%    0.0%    0.0%    0.0%    0.0%    0.0%    0.0%    0.0%    0.0%    0.0%    0.0%    0.0%    0.0%    0.0%    0.0%    0.0%    0.0%    0.0%    0.0%    0.0%    0.0%    0.0%    0.0%    0.0%    0.0%    0.0%    0.0%    0.0%    0.0%        k__Bacteria;p__Chloroflexi;c__Anaerolineae; o__envOPS12       1    0.0%    0.0%    0.0%    0.0%    0.0%    0.0%    0.0%    0.0%    0.0%    0.0%    0.0%    0.0%    0.0%    0.0%    0.0%    0.0%    0.0%    0.0%    0.0%    0.0%    0.0%    0.0%    0.0%    0.0%    0.0%    0.0%    0.0%    0.0%    0.0%    0.0%    0.0%    0.0%    0.0%    0.0%    0.0%    0.0%    0.0%    0.0%    0.0%    0.0%    0.0%    0.0%    0.0%    0.0%    0.0%    0.0%    0.0%    0.0%    0.0%    0.0%    0.0%    0.0%    0.0%    0.0%    0.0%    0.0%    0.0%    0.0%    0.0%    0.0%    0.0%    0.0%    0.0%    0.0%    0.0%    0.0%    0.0%    0.0%    0.0%    0.0%    0.0%    0.0%    0.0%    0.0%    0.0%    0.0%    0.0%    0.0%    0.0%    0.0%    0.0%    0.0%    0.0%    0.0%    0.0%    0.0%    0.0%    0.0%    0.0%    0.0%    0.0%    0.0%    0.0%    0.0%    0.0%    0.0%    0.0%    0.0%    0.0%    0.0%    0.0%    0.0%    0.0%    0.0%    0.0%    0.0%    0.0%    0.0%    0.0%    0.0%    0.0%    0.0%    0.0%    0.0%    0.0%    0.0%    0.0%    0.0%    0.0%    0.0%    0.0%    0.0%    0.0%    0.0%    0.0%    0.0%    0.0%    0.0%    0.0%    0.0%    0.0%    0.0%    0.0%    0.0%    0.0%    0.0%    0.0%    0.0%    0.0%    0.0%    0.0%    0.0%    0.0%    0.0%    0.0%    0.0%    0.0%    0.0%    0.0%    0.0%    0.0%    0.0%    0.0%    0.0%    0.0%    0.0%    0.0%    0.0%    0.0%    0.0%    0.0%    0.0%    0.0%    0.0%    0.0%    0.0%    0.0%    0.0%    0.0%    0.0%    0.0%    0.0%    0.0%    0.0%    0.0%    0.0%    0.0%    0.0%    0.0%    0.0%    0.0%    0.0%    0.0%    0.0%    0.0%    0.0%    0.0%    0.0%    0.0%    0.0%    0.0%    0.0%    0.0%    0.0%    0.0%    0.0%    0.0%    0.0%    0.0%    0.0%    0.0%    0.0%    0.0%    0.0%    0.0%    0.0%    0.0%    0.0%    0.0%    0.0%    0.0%    0.0%    0.0%    0.0%    0.0%    0.0%    0.0%    0.0%    0.0%    0.0%    0.0%    0.0%    0.0%    0.0%    0.0%    0.0%    0.0%    0.0%    0.0%    0.0%    0.0%    0.0%    0.0%    0.0%    0.0%    0.0%    0.0%    0.0%    0.0%    0.0%    0.0%    0.0%    0.0%    0.0%    0.0%    0.0%    0.0%    0.0%    0.0%    0.0%    0.0%    0.0%    0.0%    0.0%    0.0%    0.0%    0.0%    0.0%    0.0%    0.0%    0.0%    0.0%    0.0%    0.0%    0.0%    0.0%    0.0%    0.0%    0.0%    0.0%    0.0%    0.0%    0.0%    0.0%    0.0%    0.0%    0.0%    0.0%    0.0%    0.0%    0.0%    0.0%    0.0%    0.0%    0.0%    0.0%    0.0%    0.0%    0.0%    0.0%    0.0%    0.0%    0.0%    0.0%    0.0%    0.0%    0.0%    0.0%    0.0%    0.0%    0.0%    0.0%    0.0%    0.0%    0.0%    0.0%    0.0%    0.0%    0.0%    0.0%    0.0%    0.0%    0.0%    0.0%    0.0%    0.0%    0.0%    0.0%    0.0%    0.0%    0.0%    0.0%    0.0%    0.0%    0.0%    0.0%    0.0%    0.0%    0.0%    0.0%    0.0%    0.0%    0.0%    0.0%    0.0%    0.0%    0.0%    0.0%    0.0%    0.0%    0.0%    0.0%    0.0%    0.0%    0.0%    0.0%    0.0%    0.0%    0.0%    0.0%    0.0%    0.0%    0.0%    0.0%    0.0%    0.0%    0.0%    0.0%        k__Bacteria;p__Chloroflexi;c__Bljii12; o__     781    0.0%    0.0%    0.0%    0.0%    0.0%    0.0%    0.0%    0.0%    0.0%    0.0%    0.0%    0.0%    0.0%    0.0%    0.0%    0.0%    0.0%    0.0%    0.0%    0.0%    0.0%    0.0%    0.0%    0.0%    0.0%    0.0%    0.0%    0.0%    0.0%    0.0%    0.0%    0.0%    0.0%    0.0%    0.0%    0.0%    0.0%    0.0%    0.0%    0.0%    0.0%    0.0%    0.0%    0.0%    0.0%    0.0%    0.0%    0.0%    0.0%    0.0%    0.0%    0.0%    0.0%    0.0%    0.0%    0.0%    0.0%    0.0%    0.0%    0.0%    0.0%    0.0%    0.0%    0.0%    0.0%    0.0%    0.0%    0.0%    0.0%    0.0%    0.0%    0.0%    0.0%    0.0%    0.0%    0.0%    0.0%    0.0%    0.0%    0.0%    0.0%    0.0%    0.0%    0.0%    0.0%    0.0%    0.0%    0.0%    0.0%    0.0%    0.0%    0.0%    0.0%    0.0%    0.0%    0.0%    0.0%    0.0%    0.0%    0.0%    0.0%    0.0%    0.0%    0.0%    0.0%    0.0%    0.0%    0.0%    0.0%    0.0%    0.0%    0.0%    0.0%    0.0%    0.0%    0.0%    0.0%    0.0%    0.0%    0.0%    0.0%    0.0%    0.0%    0.0%    0.0%    0.0%    0.0%    0.0%    0.0%    0.0%    0.0%    0.0%    0.0%    0.0%    0.0%    0.0%    0.0%    0.0%    0.0%    0.0%    0.0%    0.0%    0.0%    0.0%    0.0%    0.0%    0.0%    0.0%    0.0%    0.0%    0.0%    0.0%    0.0%    0.0%    0.0%    0.0%    0.0%    0.0%    0.0%    0.0%    0.0%    0.0%    0.0%    0.0%    0.0%    0.0%    0.0%    0.0%    0.0%    0.0%    0.0%    0.0%    0.0%    0.0%    0.0%    0.0%    0.0%    0.0%    0.0%    0.0%    0.0%    0.0%    0.0%    0.0%    0.0%    0.0%    0.0%    0.0%    0.0%    0.0%    0.0%    0.0%    0.0%    0.0%    0.0%    0.0%    0.0%    0.0%    0.0%    0.0%    0.0%    0.0%    0.0%    0.0%    0.0%    0.0%    0.0%    0.0%    0.0%    0.0%    0.0%    0.0%    0.0%    0.0%    0.0%    0.0%    0.0%    0.0%    0.0%    0.0%    0.0%    0.0%    0.0%    0.0%    0.0%    0.0%    0.0%    0.0%    0.0%    0.0%    0.0%    0.0%    0.0%    0.0%    0.0%    0.0%    0.0%    0.0%    0.0%    0.0%    0.0%    0.0%    0.0%    0.0%    0.0%    0.0%    0.0%    0.0%    0.0%    0.0%    0.0%    0.0%    0.0%    0.0%    0.0%    0.0%    0.0%    0.0%    0.0%    0.0%    0.0%    0.0%    0.0%    0.0%    0.0%    0.0%    0.0%    0.0%    0.0%    0.0%    0.0%    0.0%    0.0%    0.0%    0.0%    0.0%    0.0%    0.0%    0.0%    0.0%    0.0%    0.0%    0.0%    0.0%    0.0%    0.0%    0.0%    0.0%    0.0%    0.0%    0.0%    0.0%    0.0%    0.0%    0.0%    0.0%    0.0%    0.0%    0.0%    0.0%    0.0%    0.0%    0.0%    0.0%    0.0%    0.0%    0.0%    0.0%    0.0%    0.0%    0.0%    0.0%    0.0%    0.0%    0.0%    0.0%    0.0%    0.0%    0.0%    0.0%    0.0%    0.0%    0.0%    0.0%    0.0%    0.0%    0.0%    0.0%    0.0%    0.0%    0.0%    0.0%    0.0%    0.0%    0.0%    0.0%    0.0%    0.0%    0.0%    0.0%    0.0%    0.0%    0.0%    0.0%    0.0%    0.0%    0.0%    0.0%    0.0%    0.0%    0.0%    0.0%    0.0%    0.0%    0.0%    0.0%    0.0%    0.0%        k__Bacteria;p__Chloroflexi;c__Chloroflexi (class); o__Herpetosiphonales       9    0.0%    0.0%    0.0%    0.0%    0.0%    0.0%    0.0%    0.0%    0.0%    0.0%    0.0%    0.0%    0.0%    0.0%    0.0%    0.0%    0.0%    0.0%    0.0%    0.0%    0.0%    0.0%    0.0%    0.0%    0.0%    0.0%    0.0%    0.0%    0.0%    0.0%    0.0%    0.0%    0.0%    0.0%    0.0%    0.0%    0.0%    0.0%    0.0%    0.0%    0.0%    0.0%    0.0%    0.0%    0.0%    0.0%    0.0%    0.0%    0.0%    0.0%    0.0%    0.0%    0.0%    0.0%    0.0%    0.0%    0.0%    0.0%    0.0%    0.0%    0.0%    0.0%    0.0%    0.0%    0.0%    0.0%    0.0%    0.0%    0.0%    0.0%    0.0%    0.0%    0.0%    0.0%    0.0%    0.0%    0.0%    0.0%    0.0%    0.0%    0.0%    0.0%    0.0%    0.0%    0.0%    0.0%    0.0%    0.0%    0.0%    0.0%    0.0%    0.0%    0.0%    0.0%    0.0%    0.0%    0.0%    0.0%    0.0%    0.0%    0.0%    0.0%    0.0%    0.0%    0.0%    0.0%    0.0%    0.0%    0.0%    0.0%    0.0%    0.0%    0.0%    0.0%    0.0%    0.0%    0.0%    0.0%    0.0%    0.0%    0.0%    0.0%    0.0%    0.0%    0.0%    0.0%    0.0%    0.0%    0.0%    0.0%    0.0%    0.0%    0.0%    0.0%    0.0%    0.0%    0.0%    0.0%    0.0%    0.0%    0.0%    0.0%    0.0%    0.0%    0.0%    0.0%    0.0%    0.0%    0.0%    0.0%    0.0%    0.0%    0.0%    0.0%    0.0%    0.0%    0.0%    0.0%    0.0%    0.0%    0.0%    0.0%    0.0%    0.0%    0.0%    0.0%    0.0%    0.0%    0.0%    0.0%    0.0%    0.0%    0.0%    0.0%    0.0%    0.0%    0.0%    0.0%    0.0%    0.0%    0.0%    0.0%    0.0%    0.0%    0.0%    0.0%    0.0%    0.0%    0.0%    0.0%    0.0%    0.0%    0.0%    0.0%    0.0%    0.0%    0.0%    0.0%    0.0%    0.0%    0.0%    0.0%    0.0%    0.0%    0.0%    0.0%    0.0%    0.0%    0.0%    0.0%    0.0%    0.0%    0.0%    0.0%    0.0%    0.0%    0.0%    0.0%    0.0%    0.0%    0.0%    0.0%    0.0%    0.0%    0.0%    0.0%    0.0%    0.0%    0.0%    0.0%    0.0%    0.0%    0.0%    0.0%    0.0%    0.0%    0.0%    0.0%    0.0%    0.0%    0.0%    0.0%    0.0%    0.0%    0.0%    0.0%    0.0%    0.0%    0.0%    0.0%    0.0%    0.0%    0.0%    0.0%    0.0%    0.0%    0.0%    0.0%    0.0%    0.0%    0.0%    0.0%    0.0%    0.0%    0.0%    0.0%    0.0%    0.0%    0.0%    0.0%    0.0%    0.0%    0.0%    0.0%    0.0%    0.0%    0.0%    0.0%    0.0%    0.0%    0.0%    0.0%    0.0%    0.0%    0.0%    0.0%    0.0%    0.0%    0.0%    0.0%    0.0%    0.0%    0.0%    0.0%    0.0%    0.0%    0.0%    0.0%    0.0%    0.0%    0.0%    0.0%    0.0%    0.0%    0.0%    0.0%    0.0%    0.0%    0.0%    0.0%    0.0%    0.0%    0.0%    0.0%    0.0%    0.0%    0.0%    0.0%    0.0%    0.0%    0.0%    0.0%    0.0%    0.0%    0.0%    0.0%    0.0%    0.0%    0.0%    0.0%    0.0%    0.0%    0.0%    0.0%    0.0%    0.0%    0.0%    0.0%    0.0%    0.0%    0.0%    0.0%    0.0%    0.0%    0.0%    0.0%    0.0%    0.0%    0.0%    0.0%    0.0%    0.0%    0.0%    0.0%    0.0%    0.0%    0.0%    0.0%        k__Bacteria;p__Chloroflexi;c__Chloroflexi (class); o__Roseiflexales      58    0.0%    0.0%    0.0%    0.0%    0.0%    0.0%    0.0%    0.0%    0.0%    0.0%    0.0%    0.0%    0.0%    0.0%    0.0%    0.0%    0.0%    0.0%    0.0%    0.0%    0.0%    0.0%    0.0%    0.0%    0.0%    0.0%    0.0%    0.0%    0.0%    0.0%    0.0%    0.0%    0.0%    0.0%    0.0%    0.0%    0.0%    0.0%    0.0%    0.0%    0.0%    0.0%    0.0%    0.0%    0.0%    0.0%    0.0%    0.0%    0.0%    0.0%    0.0%    0.0%    0.0%    0.0%    0.0%    0.0%    0.0%    0.0%    0.0%    0.0%    0.0%    0.0%    0.0%    0.0%    0.0%    0.0%    0.0%    0.0%    0.0%    0.0%    0.0%    0.0%    0.0%    0.0%    0.0%    0.0%    0.0%    0.0%    0.0%    0.0%    0.0%    0.0%    0.0%    0.0%    0.0%    0.0%    0.0%    0.0%    0.0%    0.0%    0.0%    0.0%    0.0%    0.0%    0.0%    0.0%    0.0%    0.0%    0.0%    0.0%    0.0%    0.0%    0.0%    0.0%    0.0%    0.0%    0.0%    0.0%    0.0%    0.0%    0.0%    0.0%    0.0%    0.0%    0.0%    0.0%    0.0%    0.0%    0.0%    0.0%    0.0%    0.0%    0.0%    0.0%    0.0%    0.0%    0.0%    0.0%    0.0%    0.0%    0.0%    0.0%    0.0%    0.0%    0.0%    0.0%    0.0%    0.0%    0.0%    0.0%    0.0%    0.0%    0.0%    0.0%    0.0%    0.0%    0.0%    0.0%    0.0%    0.0%    0.0%    0.0%    0.0%    0.0%    0.0%    0.0%    0.0%    0.0%    0.0%    0.0%    0.0%    0.0%    0.0%    0.0%    0.0%    0.0%    0.0%    0.0%    0.0%    0.0%    0.0%    0.0%    0.0%    0.0%    0.0%    0.0%    0.0%    0.0%    0.0%    0.0%    0.0%    0.0%    0.0%    0.0%    0.0%    0.0%    0.0%    0.0%    0.0%    0.0%    0.0%    0.0%    0.0%    0.0%    0.0%    0.0%    0.0%    0.0%    0.0%    0.0%    0.0%    0.0%    0.0%    0.0%    0.0%    0.0%    0.0%    0.0%    0.0%    0.0%    0.0%    0.0%    0.0%    0.0%    0.0%    0.0%    0.0%    0.0%    0.0%    0.0%    0.0%    0.0%    0.0%    0.0%    0.0%    0.0%    0.0%    0.0%    0.0%    0.0%    0.0%    0.0%    0.0%    0.0%    0.0%    0.0%    0.0%    0.0%    0.0%    0.0%    0.0%    0.0%    0.0%    0.0%    0.0%    0.0%    0.0%    0.0%    0.0%    0.0%    0.0%    0.0%    0.0%    0.0%    0.0%    0.0%    0.0%    0.0%    0.0%    0.0%    0.0%    0.0%    0.0%    0.0%    0.0%    0.0%    0.0%    0.0%    0.0%    0.0%    0.0%    0.0%    0.0%    0.0%    0.0%    0.0%    0.0%    0.0%    0.0%    0.0%    0.0%    0.0%    0.0%    0.0%    0.0%    0.0%    0.0%    0.0%    0.0%    0.0%    0.0%    0.0%    0.0%    0.0%    0.0%    0.0%    0.0%    0.0%    0.0%    0.0%    0.0%    0.0%    0.0%    0.0%    0.0%    0.0%    0.0%    0.0%    0.0%    0.0%    0.0%    0.0%    0.0%    0.0%    0.0%    0.0%    0.0%    0.0%    0.0%    0.0%    0.0%    0.0%    0.0%    0.0%    0.0%    0.0%    0.0%    0.0%    0.0%    0.0%    0.0%    0.0%    0.0%    0.0%    0.0%    0.0%    0.0%    0.0%    0.0%    0.0%    0.0%    0.0%    0.0%    0.0%    0.0%    0.0%    0.0%    0.0%    0.0%    0.0%    0.0%    0.0%    0.0%    0.0%    0.0%    0.0%    0.0%    0.0%        k__Bacteria;p__Chloroflexi;c__Chloroflexi-4; o__       1    0.0%    0.0%    0.0%    0.0%    0.0%    0.0%    0.0%    0.0%    0.0%    0.0%    0.0%    0.0%    0.0%    0.0%    0.0%    0.0%    0.0%    0.0%    0.0%    0.0%    0.0%    0.0%    0.0%    0.0%    0.0%    0.0%    0.0%    0.0%    0.0%    0.0%    0.0%    0.0%    0.0%    0.0%    0.0%    0.0%    0.0%    0.0%    0.0%    0.0%    0.0%    0.0%    0.0%    0.0%    0.0%    0.0%    0.0%    0.0%    0.0%    0.0%    0.0%    0.0%    0.0%    0.0%    0.0%    0.0%    0.0%    0.0%    0.0%    0.0%    0.0%    0.0%    0.0%    0.0%    0.0%    0.0%    0.0%    0.0%    0.0%    0.0%    0.0%    0.0%    0.0%    0.0%    0.0%    0.0%    0.0%    0.0%    0.0%    0.0%    0.0%    0.0%    0.0%    0.0%    0.0%    0.0%    0.0%    0.0%    0.0%    0.0%    0.0%    0.0%    0.0%    0.0%    0.0%    0.0%    0.0%    0.0%    0.0%    0.0%    0.0%    0.0%    0.0%    0.0%    0.0%    0.0%    0.0%    0.0%    0.0%    0.0%    0.0%    0.0%    0.0%    0.0%    0.0%    0.0%    0.0%    0.0%    0.0%    0.0%    0.0%    0.0%    0.0%    0.0%    0.0%    0.0%    0.0%    0.0%    0.0%    0.0%    0.0%    0.0%    0.0%    0.0%    0.0%    0.0%    0.0%    0.0%    0.0%    0.0%    0.0%    0.0%    0.0%    0.0%    0.0%    0.0%    0.0%    0.0%    0.0%    0.0%    0.0%    0.0%    0.0%    0.0%    0.0%    0.0%    0.0%    0.0%    0.0%    0.0%    0.0%    0.0%    0.0%    0.0%    0.0%    0.0%    0.0%    0.0%    0.0%    0.0%    0.0%    0.0%    0.0%    0.0%    0.0%    0.0%    0.0%    0.0%    0.0%    0.0%    0.0%    0.0%    0.0%    0.0%    0.0%    0.0%    0.0%    0.0%    0.0%    0.0%    0.0%    0.0%    0.0%    0.0%    0.0%    0.0%    0.0%    0.0%    0.0%    0.0%    0.0%    0.0%    0.0%    0.0%    0.0%    0.0%    0.0%    0.0%    0.0%    0.0%    0.0%    0.0%    0.0%    0.0%    0.0%    0.0%    0.0%    0.0%    0.0%    0.0%    0.0%    0.0%    0.0%    0.0%    0.0%    0.0%    0.0%    0.0%    0.0%    0.0%    0.0%    0.0%    0.0%    0.0%    0.0%    0.0%    0.0%    0.0%    0.0%    0.0%    0.0%    0.0%    0.0%    0.0%    0.0%    0.0%    0.0%    0.0%    0.0%    0.0%    0.0%    0.0%    0.0%    0.0%    0.0%    0.0%    0.0%    0.0%    0.0%    0.0%    0.0%    0.0%    0.0%    0.0%    0.0%    0.0%    0.0%    0.0%    0.0%    0.0%    0.0%    0.0%    0.0%    0.0%    0.0%    0.0%    0.0%    0.0%    0.0%    0.0%    0.0%    0.0%    0.0%    0.0%    0.0%    0.0%    0.0%    0.0%    0.0%    0.0%    0.0%    0.0%    0.0%    0.0%    0.0%    0.0%    0.0%    0.0%    0.0%    0.0%    0.0%    0.0%    0.0%    0.0%    0.0%    0.0%    0.0%    0.0%    0.0%    0.0%    0.0%    0.0%    0.0%    0.0%    0.0%    0.0%    0.0%    0.0%    0.0%    0.0%    0.0%    0.0%    0.0%    0.0%    0.0%    0.0%    0.0%    0.0%    0.0%    0.0%    0.0%    0.0%    0.0%    0.0%    0.0%    0.0%    0.0%    0.0%    0.0%    0.0%    0.0%    0.0%    0.0%    0.0%    0.0%    0.0%    0.0%    0.0%    0.0%    0.0%    0.0%    0.0%    0.0%    0.0%    0.0%    0.0%    0.0%    0.0%        k__Bacteria;p__Chloroflexi;c__Ktedonobacteria; o__       8    0.0%    0.0%    0.0%    0.0%    0.0%    0.0%    0.0%    0.0%    0.0%    0.0%    0.0%    0.0%    0.0%    0.0%    0.0%    0.0%    0.0%    0.0%    0.0%    0.0%    0.0%    0.0%    0.0%    0.0%    0.0%    0.0%    0.0%    0.0%    0.0%    0.0%    0.0%    0.0%    0.0%    0.0%    0.0%    0.0%    0.0%    0.0%    0.0%    0.0%    0.0%    0.0%    0.0%    0.0%    0.0%    0.0%    0.0%    0.0%    0.0%    0.0%    0.0%    0.0%    0.0%    0.0%    0.0%    0.0%    0.0%    0.0%    0.0%    0.0%    0.0%    0.0%    0.0%    0.0%    0.0%    0.0%    0.0%    0.0%    0.0%    0.0%    0.0%    0.0%    0.0%    0.0%    0.0%    0.0%    0.0%    0.0%    0.0%    0.0%    0.0%    0.0%    0.0%    0.0%    0.0%    0.0%    0.0%    0.0%    0.0%    0.0%    0.0%    0.0%    0.0%    0.0%    0.0%    0.0%    0.0%    0.0%    0.0%    0.0%    0.0%    0.0%    0.0%    0.0%    0.0%    0.0%    0.0%    0.0%    0.0%    0.0%    0.0%    0.0%    0.0%    0.0%    0.0%    0.0%    0.0%    0.0%    0.0%    0.0%    0.0%    0.0%    0.0%    0.0%    0.0%    0.0%    0.0%    0.0%    0.0%    0.0%    0.0%    0.0%    0.0%    0.0%    0.0%    0.0%    0.0%    0.0%    0.0%    0.0%    0.0%    0.0%    0.0%    0.0%    0.0%    0.0%    0.0%    0.0%    0.0%    0.0%    0.0%    0.0%    0.0%    0.0%    0.0%    0.0%    0.0%    0.0%    0.0%    0.0%    0.0%    0.0%    0.0%    0.0%    0.0%    0.0%    0.0%    0.0%    0.0%    0.0%    0.0%    0.0%    0.0%    0.0%    0.0%    0.0%    0.0%    0.0%    0.0%    0.0%    0.0%    0.0%    0.0%    0.0%    0.0%    0.0%    0.0%    0.0%    0.0%    0.0%    0.0%    0.0%    0.0%    0.0%    0.0%    0.0%    0.0%    0.0%    0.0%    0.0%    0.0%    0.0%    0.0%    0.0%    0.0%    0.0%    0.0%    0.0%    0.0%    0.0%    0.0%    0.0%    0.0%    0.0%    0.0%    0.0%    0.0%    0.0%    0.0%    0.0%    0.0%    0.0%    0.0%    0.0%    0.0%    0.0%    0.0%    0.0%    0.0%    0.0%    0.0%    0.0%    0.0%    0.0%    0.0%    0.0%    0.0%    0.0%    0.0%    0.0%    0.0%    0.0%    0.0%    0.0%    0.0%    0.0%    0.0%    0.0%    0.0%    0.0%    0.0%    0.0%    0.0%    0.0%    0.0%    0.0%    0.0%    0.0%    0.0%    0.0%    0.0%    0.0%    0.0%    0.0%    0.0%    0.0%    0.0%    0.0%    0.0%    0.0%    0.0%    0.0%    0.0%    0.0%    0.0%    0.0%    0.0%    0.0%    0.0%    0.0%    0.0%    0.0%    0.0%    0.0%    0.0%    0.0%    0.0%    0.0%    0.0%    0.0%    0.0%    0.0%    0.0%    0.0%    0.0%    0.0%    0.0%    0.0%    0.0%    0.0%    0.0%    0.0%    0.0%    0.0%    0.0%    0.0%    0.0%    0.0%    0.0%    0.0%    0.0%    0.0%    0.0%    0.0%    0.0%    0.0%    0.0%    0.0%    0.0%    0.0%    0.0%    0.0%    0.0%    0.0%    0.0%    0.0%    0.0%    0.0%    0.0%    0.0%    0.0%    0.0%    0.0%    0.0%    0.0%    0.0%    0.0%    0.0%    0.0%    0.0%    0.0%    0.0%    0.0%    0.0%    0.0%    0.0%    0.0%    0.0%    0.0%    0.0%    0.0%    0.0%    0.0%    0.0%    0.0%    0.0%    0.0%    0.0%        k__Bacteria;p__Chloroflexi;c__SOGA31; o__    9477    0.1%    0.0%    0.1%    0.0%    0.1%    0.0%    0.0%    0.0%    0.0%    0.0%    0.0%    0.0%    0.0%    0.0%    0.0%    0.0%    0.0%    0.1%    0.0%    0.0%    0.0%    0.0%    0.0%    0.0%    0.0%    0.0%    0.0%    0.0%    0.1%    0.0%    0.0%    0.0%    0.0%    0.0%    0.1%    0.0%    0.0%    0.0%    0.0%    0.1%    0.0%    0.1%    0.0%    0.0%    0.0%    0.0%    0.0%    0.0%    0.0%    0.0%    0.0%    0.0%    0.0%    0.0%    0.1%    0.0%    0.1%    0.0%    0.0%    0.1%    0.1%    0.1%    0.0%    0.2%    0.1%    0.0%    0.0%    0.2%    0.1%    0.1%    0.0%    0.0%    0.1%    0.1%    0.1%    0.0%    0.0%    0.1%    0.0%    0.1%    0.0%    0.1%    0.0%    0.0%    0.1%    0.0%    0.2%    0.0%    0.0%    0.1%    0.0%    0.1%    0.1%    0.0%    0.0%    0.1%    0.2%    0.0%    0.1%    0.1%    0.1%    0.1%    0.0%    0.1%    0.0%    0.0%    0.2%    0.0%    0.0%    0.1%    0.1%    0.2%    0.0%    0.0%    0.1%    0.0%    0.0%    0.0%    0.0%    0.5%    0.0%    0.1%    0.2%    0.1%    0.0%    0.3%    0.1%    0.0%    0.1%    0.4%    0.1%    0.1%    0.1%    0.0%    0.3%    0.0%    0.1%    0.1%    0.0%    0.0%    0.0%    0.1%    0.1%    0.0%    0.2%    0.1%    0.0%    0.0%    0.1%    0.0%    0.0%    0.0%    0.2%    0.0%    0.0%    0.0%    0.2%    0.0%    0.0%    0.2%    0.4%    0.2%    0.2%    0.1%    0.0%    0.0%    0.1%    0.0%    0.0%    0.0%    0.1%    0.2%    0.1%    0.0%    0.0%    0.0%    0.0%    0.0%    0.0%    0.0%    0.0%    0.0%    0.0%    0.1%    0.1%    0.0%    0.0%    0.0%    0.0%    0.0%    0.0%    0.0%    0.0%    0.0%    0.0%    0.0%    0.2%    0.0%    0.0%    0.0%    0.0%    0.1%    0.0%    0.2%    0.1%    0.0%    0.1%    0.1%    0.3%    0.1%    0.1%    0.0%    0.1%    0.0%    0.0%    0.1%    0.0%    0.1%    0.0%    0.0%    0.1%    0.1%    0.0%    0.1%    0.0%    0.0%    0.0%    0.0%    0.1%    0.0%    0.0%    0.0%    0.2%    0.0%    0.3%    0.0%    0.2%    0.0%    0.0%    0.0%    0.0%    0.0%    0.0%    0.0%    0.0%    0.0%    0.0%    0.0%    0.0%    0.0%    0.2%    0.1%    0.0%    0.0%    0.0%    0.0%    0.0%    0.0%    0.0%    0.0%    0.1%    0.1%    0.1%    0.1%    0.0%    0.0%    0.1%    0.1%    0.0%    0.1%    0.0%    0.1%    0.2%    0.1%    0.0%    0.0%    0.0%    0.0%    0.0%    0.0%    0.0%    0.0%    0.0%    0.0%    0.0%    0.0%    0.0%    0.7%    0.0%    0.0%    0.1%    0.0%    0.1%    0.1%    0.1%    0.0%    0.0%    0.1%    0.0%    0.1%    0.0%    0.0%    0.0%    0.0%    0.0%    0.0%    0.0%    0.0%    0.0%    0.0%    0.0%    0.0%    0.0%    0.0%    0.1%    0.2%    0.0%    0.0%    0.0%    0.0%    0.0%    0.0%    0.0%    0.0%    0.0%    0.0%    0.0%    0.0%    0.0%    0.0%    0.1%    0.0%    0.0%    0.0%    0.0%    0.0%    0.0%    0.0%    0.0%    0.0%    0.0%    0.0%    0.0%    0.0%    0.2%    0.0%    0.0%    0.0%    0.0%    0.0%    0.0%    0.1%    0.0%    0.1%    0.0%    0.0%    0.5%    0.0%        k__Bacteria;p__Chloroflexi;c__TK17; o__     369    0.0%    0.0%    0.0%    0.0%    0.0%    0.0%    0.0%    0.0%    0.0%    0.0%    0.0%    0.0%    0.0%    0.0%    0.0%    0.0%    0.0%    0.0%    0.0%    0.0%    0.0%    0.0%    0.0%    0.0%    0.0%    0.0%    0.0%    0.0%    0.0%    0.0%    0.0%    0.0%    0.0%    0.0%    0.0%    0.0%    0.0%    0.0%    0.0%    0.0%    0.0%    0.0%    0.0%    0.0%    0.0%    0.0%    0.0%    0.0%    0.0%    0.0%    0.0%    0.0%    0.0%    0.0%    0.0%    0.0%    0.0%    0.0%    0.0%    0.0%    0.0%    0.0%    0.0%    0.0%    0.0%    0.0%    0.0%    0.0%    0.0%    0.0%    0.0%    0.0%    0.0%    0.0%    0.0%    0.0%    0.0%    0.0%    0.0%    0.0%    0.0%    0.0%    0.0%    0.0%    0.0%    0.0%    0.0%    0.0%    0.0%    0.0%    0.0%    0.0%    0.0%    0.0%    0.0%    0.0%    0.0%    0.0%    0.0%    0.0%    0.0%    0.0%    0.0%    0.0%    0.0%    0.0%    0.0%    0.0%    0.0%    0.0%    0.0%    0.0%    0.0%    0.0%    0.0%    0.0%    0.0%    0.0%    0.0%    0.0%    0.0%    0.0%    0.0%    0.0%    0.0%    0.0%    0.0%    0.0%    0.0%    0.0%    0.0%    0.0%    0.0%    0.0%    0.0%    0.0%    0.0%    0.0%    0.0%    0.0%    0.0%    0.0%    0.0%    0.0%    0.0%    0.0%    0.0%    0.0%    0.0%    0.0%    0.0%    0.0%    0.0%    0.0%    0.0%    0.0%    0.0%    0.0%    0.0%    0.0%    0.0%    0.0%    0.0%    0.0%    0.0%    0.0%    0.0%    0.0%    0.0%    0.0%    0.0%    0.0%    0.0%    0.0%    0.0%    0.0%    0.0%    0.0%    0.0%    0.0%    0.0%    0.0%    0.0%    0.0%    0.0%    0.0%    0.0%    0.0%    0.0%    0.0%    0.0%    0.0%    0.0%    0.0%    0.0%    0.0%    0.0%    0.0%    0.0%    0.0%    0.0%    0.0%    0.0%    0.0%    0.0%    0.0%    0.0%    0.0%    0.0%    0.0%    0.0%    0.0%    0.0%    0.0%    0.0%    0.0%    0.0%    0.0%    0.0%    0.0%    0.0%    0.0%    0.0%    0.0%    0.0%    0.0%    0.0%    0.0%    0.0%    0.0%    0.0%    0.0%    0.0%    0.0%    0.0%    0.0%    0.0%    0.0%    0.0%    0.0%    0.0%    0.0%    0.0%    0.0%    0.0%    0.0%    0.0%    0.0%    0.0%    0.0%    0.0%    0.0%    0.0%    0.0%    0.0%    0.0%    0.0%    0.0%    0.0%    0.0%    0.0%    0.0%    0.0%    0.0%    0.0%    0.0%    0.0%    0.0%    0.0%    0.0%    0.0%    0.0%    0.0%    0.0%    0.0%    0.0%    0.0%    0.0%    0.0%    0.0%    0.0%    0.0%    0.0%    0.0%    0.0%    0.0%    0.0%    0.0%    0.0%    0.0%    0.0%    0.0%    0.0%    0.0%    0.0%    0.0%    0.0%    0.0%    0.0%    0.0%    0.0%    0.0%    0.0%    0.0%    0.0%    0.0%    0.0%    0.0%    0.0%    0.0%    0.0%    0.0%    0.0%    0.0%    0.0%    0.0%    0.0%    0.0%    0.0%    0.0%    0.0%    0.0%    0.0%    0.0%    0.0%    0.0%    0.0%    0.0%    0.0%    0.0%    0.0%    0.0%    0.0%    0.0%    0.0%    0.0%    0.0%    0.0%    0.0%    0.0%    0.0%    0.0%    0.0%    0.0%    0.0%    0.0%    0.0%    0.0%    0.0%    0.0%    0.0%    0.0%    0.0%    0.0%    0.0%    0.0%    0.0%    0.0%        k__Bacteria;p__Chloroflexi;c__Thermobacula; o__Thermobaculales       0    0.0%    0.0%    0.0%    0.0%    0.0%    0.0%    0.0%    0.0%    0.0%    0.0%    0.0%    0.0%    0.0%    0.0%    0.0%    0.0%    0.0%    0.0%    0.0%    0.0%    0.0%    0.0%    0.0%    0.0%    0.0%    0.0%    0.0%    0.0%    0.0%    0.0%    0.0%    0.0%    0.0%    0.0%    0.0%    0.0%    0.0%    0.0%    0.0%    0.0%    0.0%    0.0%    0.0%    0.0%    0.0%    0.0%    0.0%    0.0%    0.0%    0.0%    0.0%    0.0%    0.0%    0.0%    0.0%    0.0%    0.0%    0.0%    0.0%    0.0%    0.0%    0.0%    0.0%    0.0%    0.0%    0.0%    0.0%    0.0%    0.0%    0.0%    0.0%    0.0%    0.0%    0.0%    0.0%    0.0%    0.0%    0.0%    0.0%    0.0%    0.0%    0.0%    0.0%    0.0%    0.0%    0.0%    0.0%    0.0%    0.0%    0.0%    0.0%    0.0%    0.0%    0.0%    0.0%    0.0%    0.0%    0.0%    0.0%    0.0%    0.0%    0.0%    0.0%    0.0%    0.0%    0.0%    0.0%    0.0%    0.0%    0.0%    0.0%    0.0%    0.0%    0.0%    0.0%    0.0%    0.0%    0.0%    0.0%    0.0%    0.0%    0.0%    0.0%    0.0%    0.0%    0.0%    0.0%    0.0%    0.0%    0.0%    0.0%    0.0%    0.0%    0.0%    0.0%    0.0%    0.0%    0.0%    0.0%    0.0%    0.0%    0.0%    0.0%    0.0%    0.0%    0.0%    0.0%    0.0%    0.0%    0.0%    0.0%    0.0%    0.0%    0.0%    0.0%    0.0%    0.0%    0.0%    0.0%    0.0%    0.0%    0.0%    0.0%    0.0%    0.0%    0.0%    0.0%    0.0%    0.0%    0.0%    0.0%    0.0%    0.0%    0.0%    0.0%    0.0%    0.0%    0.0%    0.0%    0.0%    0.0%    0.0%    0.0%    0.0%    0.0%    0.0%    0.0%    0.0%    0.0%    0.0%    0.0%    0.0%    0.0%    0.0%    0.0%    0.0%    0.0%    0.0%    0.0%    0.0%    0.0%    0.0%    0.0%    0.0%    0.0%    0.0%    0.0%    0.0%    0.0%    0.0%    0.0%    0.0%    0.0%    0.0%    0.0%    0.0%    0.0%    0.0%    0.0%    0.0%    0.0%    0.0%    0.0%    0.0%    0.0%    0.0%    0.0%    0.0%    0.0%    0.0%    0.0%    0.0%    0.0%    0.0%    0.0%    0.0%    0.0%    0.0%    0.0%    0.0%    0.0%    0.0%    0.0%    0.0%    0.0%    0.0%    0.0%    0.0%    0.0%    0.0%    0.0%    0.0%    0.0%    0.0%    0.0%    0.0%    0.0%    0.0%    0.0%    0.0%    0.0%    0.0%    0.0%    0.0%    0.0%    0.0%    0.0%    0.0%    0.0%    0.0%    0.0%    0.0%    0.0%    0.0%    0.0%    0.0%    0.0%    0.0%    0.0%    0.0%    0.0%    0.0%    0.0%    0.0%    0.0%    0.0%    0.0%    0.0%    0.0%    0.0%    0.0%    0.0%    0.0%    0.0%    0.0%    0.0%    0.0%    0.0%    0.0%    0.0%    0.0%    0.0%    0.0%    0.0%    0.0%    0.0%    0.0%    0.0%    0.0%    0.0%    0.0%    0.0%    0.0%    0.0%    0.0%    0.0%    0.0%    0.0%    0.0%    0.0%    0.0%    0.0%    0.0%    0.0%    0.0%    0.0%    0.0%    0.0%    0.0%    0.0%    0.0%    0.0%    0.0%    0.0%    0.0%    0.0%    0.0%    0.0%    0.0%    0.0%    0.0%    0.0%    0.0%    0.0%    0.0%    0.0%    0.0%    0.0%    0.0%    0.0%    0.0%    0.0%    0.0%    0.0%    0.0%    0.0%    0.0%    0.0%        k__Bacteria;p__Chloroflexi;c__Thermomicrobia; o__      31    0.0%    0.0%    0.0%    0.0%    0.0%    0.0%    0.0%    0.0%    0.0%    0.0%    0.0%    0.0%    0.0%    0.0%    0.0%    0.0%    0.0%    0.0%    0.0%    0.0%    0.0%    0.0%    0.0%    0.0%    0.0%    0.0%    0.0%    0.0%    0.0%    0.0%    0.0%    0.0%    0.0%    0.0%    0.0%    0.0%    0.0%    0.0%    0.0%    0.0%    0.0%    0.0%    0.0%    0.0%    0.0%    0.0%    0.0%    0.0%    0.0%    0.0%    0.0%    0.0%    0.0%    0.0%    0.0%    0.0%    0.0%    0.0%    0.0%    0.0%    0.0%    0.0%    0.0%    0.0%    0.0%    0.0%    0.0%    0.0%    0.0%    0.0%    0.0%    0.0%    0.0%    0.0%    0.0%    0.0%    0.0%    0.0%    0.0%    0.0%    0.0%    0.0%    0.0%    0.0%    0.0%    0.0%    0.0%    0.0%    0.0%    0.0%    0.0%    0.0%    0.0%    0.0%    0.0%    0.0%    0.0%    0.0%    0.0%    0.0%    0.0%    0.0%    0.0%    0.0%    0.0%    0.0%    0.0%    0.0%    0.0%    0.0%    0.0%    0.0%    0.0%    0.0%    0.0%    0.0%    0.0%    0.0%    0.0%    0.0%    0.0%    0.0%    0.0%    0.0%    0.0%    0.0%    0.0%    0.0%    0.0%    0.0%    0.0%    0.0%    0.0%    0.0%    0.0%    0.0%    0.0%    0.0%    0.0%    0.0%    0.0%    0.0%    0.0%    0.0%    0.0%    0.0%    0.0%    0.0%    0.0%    0.0%    0.0%    0.0%    0.0%    0.0%    0.0%    0.0%    0.0%    0.0%    0.0%    0.0%    0.0%    0.0%    0.0%    0.0%    0.0%    0.0%    0.0%    0.0%    0.0%    0.0%    0.0%    0.0%    0.0%    0.0%    0.0%    0.0%    0.0%    0.0%    0.0%    0.0%    0.0%    0.0%    0.0%    0.0%    0.0%    0.0%    0.0%    0.0%    0.0%    0.0%    0.0%    0.0%    0.0%    0.0%    0.0%    0.0%    0.0%    0.0%    0.0%    0.0%    0.0%    0.0%    0.0%    0.0%    0.0%    0.0%    0.0%    0.0%    0.0%    0.0%    0.0%    0.0%    0.0%    0.0%    0.0%    0.0%    0.0%    0.0%    0.0%    0.0%    0.0%    0.0%    0.0%    0.0%    0.0%    0.0%    0.0%    0.0%    0.0%    0.0%    0.0%    0.0%    0.0%    0.0%    0.0%    0.0%    0.0%    0.0%    0.0%    0.0%    0.0%    0.0%    0.0%    0.0%    0.0%    0.0%    0.0%    0.0%    0.0%    0.0%    0.0%    0.0%    0.0%    0.0%    0.0%    0.0%    0.0%    0.0%    0.0%    0.0%    0.0%    0.0%    0.0%    0.0%    0.0%    0.0%    0.0%    0.0%    0.0%    0.0%    0.0%    0.0%    0.0%    0.0%    0.0%    0.0%    0.0%    0.0%    0.0%    0.0%    0.0%    0.0%    0.0%    0.0%    0.1%    0.0%    0.0%    0.0%    0.0%    0.0%    0.0%    0.0%    0.0%    0.0%    0.0%    0.0%    0.0%    0.0%    0.0%    0.0%    0.0%    0.0%    0.0%    0.0%    0.0%    0.0%    0.0%    0.0%    0.0%    0.0%    0.0%    0.0%    0.0%    0.0%    0.0%    0.0%    0.0%    0.0%    0.0%    0.0%    0.0%    0.0%    0.0%    0.0%    0.0%    0.0%    0.0%    0.0%    0.0%    0.0%    0.0%    0.0%    0.0%    0.0%    0.0%    0.0%    0.0%    0.0%    0.0%    0.0%    0.0%    0.0%    0.0%    0.0%    0.0%    0.0%    0.0%    0.0%    0.0%    0.0%    0.0%    0.0%    0.0%    0.0%    0.0%    0.0%    0.0%    0.0%        k__Bacteria;p__Chloroflexi;c__Thermomicrobia; o__HN1-15    7920    0.0%    0.0%    0.1%    0.0%    0.0%    0.0%    0.0%    0.0%    0.0%    0.0%    0.0%    0.0%    0.0%    0.0%    0.0%    0.0%    0.0%    0.1%    0.0%    0.0%    0.1%    0.1%    0.0%    0.0%    0.0%    0.0%    0.0%    0.0%    0.1%    0.0%    0.0%    0.1%    0.0%    0.0%    0.1%    0.0%    0.0%    0.0%    0.1%    0.2%    0.0%    0.1%    0.0%    0.0%    0.1%    0.0%    0.0%    0.0%    0.0%    0.0%    0.0%    0.0%    0.0%    0.0%    0.1%    0.0%    0.0%    0.0%    0.0%    0.1%    0.1%    0.0%    0.0%    0.1%    0.1%    0.1%    0.0%    0.2%    0.1%    0.1%    0.0%    0.0%    0.0%    0.0%    0.2%    0.0%    0.0%    0.1%    0.0%    0.0%    0.0%    0.1%    0.0%    0.0%    0.0%    0.0%    0.1%    0.0%    0.0%    0.1%    0.0%    0.1%    0.1%    0.0%    0.0%    0.2%    0.1%    0.0%    0.0%    0.0%    0.1%    0.0%    0.0%    0.1%    0.0%    0.0%    0.1%    0.0%    0.0%    0.1%    0.1%    0.2%    0.0%    0.0%    0.1%    0.0%    0.0%    0.0%    0.1%    0.3%    0.0%    0.1%    0.2%    0.1%    0.0%    0.1%    0.0%    0.0%    0.1%    0.2%    0.1%    0.1%    0.1%    0.1%    0.4%    0.0%    0.1%    0.1%    0.0%    0.0%    0.0%    0.1%    0.1%    0.0%    0.2%    0.0%    0.0%    0.0%    0.0%    0.0%    0.0%    0.0%    0.2%    0.1%    0.0%    0.0%    0.1%    0.0%    0.0%    0.1%    0.2%    0.2%    0.2%    0.0%    0.0%    0.0%    0.1%    0.1%    0.1%    0.1%    0.2%    0.1%    0.1%    0.0%    0.0%    0.0%    0.0%    0.0%    0.0%    0.1%    0.0%    0.0%    0.0%    0.0%    0.0%    0.0%    0.0%    0.0%    0.0%    0.0%    0.0%    0.0%    0.0%    0.0%    0.0%    0.1%    0.1%    0.0%    0.0%    0.0%    0.0%    0.0%    0.0%    0.1%    0.2%    0.0%    0.0%    0.0%    0.2%    0.1%    0.1%    0.0%    0.0%    0.0%    0.0%    0.0%    0.0%    0.0%    0.0%    0.0%    0.1%    0.1%    0.0%    0.1%    0.0%    0.0%    0.0%    0.0%    0.1%    0.1%    0.0%    0.0%    0.1%    0.0%    0.1%    0.0%    0.1%    0.0%    0.0%    0.0%    0.0%    0.0%    0.0%    0.0%    0.0%    0.0%    0.0%    0.0%    0.0%    0.0%    0.2%    0.0%    0.0%    0.0%    0.0%    0.0%    0.0%    0.0%    0.0%    0.0%    0.1%    0.1%    0.1%    0.0%    0.0%    0.0%    0.1%    0.0%    0.0%    0.0%    0.0%    0.1%    0.1%    0.1%    0.0%    0.0%    0.0%    0.0%    0.0%    0.0%    0.0%    0.0%    0.0%    0.0%    0.0%    0.0%    0.1%    0.7%    0.1%    0.0%    0.0%    0.0%    0.1%    0.0%    0.0%    0.0%    0.0%    0.1%    0.0%    0.2%    0.1%    0.0%    0.0%    0.1%    0.1%    0.0%    0.0%    0.0%    0.1%    0.0%    0.0%    0.0%    0.0%    0.0%    0.0%    0.1%    0.0%    0.0%    0.0%    0.1%    0.0%    0.0%    0.0%    0.0%    0.0%    0.0%    0.0%    0.0%    0.0%    0.1%    0.0%    0.0%    0.0%    0.1%    0.0%    0.0%    0.0%    0.0%    0.0%    0.0%    0.0%    0.0%    0.0%    0.0%    0.2%    0.0%    0.0%    0.0%    0.0%    0.0%    0.0%    0.0%    0.0%    0.1%    0.0%    0.0%    0.0%    0.0%        k__Bacteria;p__Chloroflexi;c__Thermomicrobia; o__Thermomicrobiales     152    0.0%    0.0%    0.0%    0.0%    0.0%    0.0%    0.0%    0.0%    0.0%    0.0%    0.0%    0.0%    0.0%    0.0%    0.0%    0.0%    0.0%    0.0%    0.0%    0.0%    0.0%    0.0%    0.0%    0.0%    0.0%    0.0%    0.0%    0.0%    0.0%    0.0%    0.0%    0.0%    0.0%    0.0%    0.0%    0.0%    0.0%    0.0%    0.0%    0.0%    0.0%    0.0%    0.0%    0.0%    0.0%    0.0%    0.0%    0.0%    0.0%    0.0%    0.0%    0.0%    0.0%    0.0%    0.0%    0.0%    0.0%    0.0%    0.0%    0.0%    0.0%    0.0%    0.0%    0.0%    0.0%    0.0%    0.0%    0.0%    0.0%    0.0%    0.0%    0.0%    0.0%    0.0%    0.0%    0.0%    0.0%    0.0%    0.0%    0.0%    0.0%    0.0%    0.0%    0.0%    0.0%    0.0%    0.0%    0.0%    0.0%    0.0%    0.0%    0.0%    0.0%    0.0%    0.0%    0.0%    0.0%    0.0%    0.0%    0.0%    0.0%    0.0%    0.0%    0.0%    0.0%    0.0%    0.0%    0.0%    0.0%    0.0%    0.0%    0.0%    0.0%    0.0%    0.0%    0.0%    0.0%    0.0%    0.0%    0.0%    0.0%    0.0%    0.0%    0.0%    0.0%    0.0%    0.0%    0.0%    0.0%    0.0%    0.0%    0.0%    0.0%    0.0%    0.0%    0.0%    0.0%    0.0%    0.0%    0.0%    0.0%    0.0%    0.0%    0.0%    0.0%    0.0%    0.0%    0.0%    0.0%    0.0%    0.0%    0.0%    0.0%    0.0%    0.0%    0.0%    0.0%    0.0%    0.0%    0.0%    0.0%    0.0%    0.0%    0.0%    0.0%    0.0%    0.0%    0.0%    0.0%    0.0%    0.0%    0.0%    0.0%    0.0%    0.0%    0.0%    0.0%    0.0%    0.0%    0.0%    0.0%    0.0%    0.0%    0.0%    0.0%    0.0%    0.0%    0.0%    0.0%    0.0%    0.0%    0.0%    0.0%    0.0%    0.0%    0.0%    0.0%    0.0%    0.0%    0.0%    0.0%    0.0%    0.0%    0.0%    0.0%    0.0%    0.0%    0.0%    0.0%    0.0%    0.0%    0.0%    0.0%    0.0%    0.0%    0.0%    0.0%    0.0%    0.0%    0.0%    0.0%    0.0%    0.0%    0.0%    0.0%    0.0%    0.0%    0.0%    0.0%    0.0%    0.0%    0.0%    0.0%    0.0%    0.0%    0.0%    0.0%    0.0%    0.0%    0.0%    0.0%    0.0%    0.0%    0.0%    0.0%    0.0%    0.0%    0.0%    0.0%    0.0%    0.0%    0.0%    0.0%    0.0%    0.0%    0.0%    0.0%    0.0%    0.0%    0.0%    0.0%    0.0%    0.0%    0.0%    0.0%    0.0%    0.0%    0.0%    0.0%    0.0%    0.0%    0.0%    0.0%    0.0%    0.0%    0.0%    0.0%    0.0%    0.0%    0.0%    0.0%    0.0%    0.0%    0.0%    0.0%    0.0%    0.0%    0.1%    0.0%    0.0%    0.0%    0.0%    0.0%    0.0%    0.0%    0.0%    0.0%    0.0%    0.0%    0.0%    0.0%    0.0%    0.0%    0.0%    0.0%    0.0%    0.0%    0.0%    0.0%    0.0%    0.0%    0.0%    0.0%    0.0%    0.0%    0.0%    0.0%    0.0%    0.0%    0.0%    0.0%    0.0%    0.0%    0.0%    0.0%    0.0%    0.0%    0.0%    0.0%    0.0%    0.0%    0.0%    0.0%    0.0%    0.0%    0.0%    0.0%    0.0%    0.0%    0.0%    0.0%    0.0%    0.0%    0.0%    0.0%    0.0%    0.0%    0.0%    0.0%    0.0%    0.0%    0.0%    0.0%    0.0%    0.0%    0.0%    0.0%    0.0%        k__Bacteria;p__Cyanobacteria;c__; o__   2261137   13.3%    0.1%    9.0%    0.9%    1.6%   44.5%    0.3%    0.1%    0.1%    0.0%    0.1%    0.1%   10.1%    6.0%    0.5%    2.1%    0.5%   25.9%   51.0%   19.8%   16.4%    8.1%    3.8%    0.2%    0.5%    1.3%   46.2%    1.7%    1.1%    0.4%    0.1%    8.9%   65.8%   25.7%    3.0%    0.9%    1.1%    3.6%    9.7%   32.8%   26.2%    7.5%    0.2%    0.4%    0.3%    0.6%   17.9%    6.6%    1.3%    0.7%    8.7%   26.9%    5.2%    8.4%   40.6%    1.5%   33.2%    7.3%    3.1%   15.9%   48.3%   15.9%    3.0%    7.7%   17.7%   16.5%   41.6%    3.0%   43.3%   24.8%   13.2%    3.2%   79.3%   21.6%    7.4%   87.5%   46.6%   15.9%    0.6%    1.4%    0.8%    4.3%    9.2%    1.9%    3.3%    0.1%    3.6%   58.6%   20.7%    1.9%   37.6%    6.7%    2.9%   45.5%    1.3%   19.5%   45.4%   65.1%    4.5%    1.8%   20.9%    2.4%    2.3%    3.8%    0.7%    0.9%    1.4%   52.8%   38.7%    1.8%    0.8%    2.3%   14.5%   80.9%    6.4%    2.3%    1.0%    1.1%    1.1%    5.1%    5.9%    2.7%    3.4%   13.4%    1.8%    2.7%    6.6%   44.5%   16.0%    5.8%   13.2%    7.7%   48.7%    4.0%    2.5%   19.9%    0.6%    1.3%    0.4%   36.3%    0.6%    3.1%   26.6%   17.1%   40.5%    3.1%    0.7%    0.4%   19.1%   44.1%   44.7%   50.5%   14.2%    5.6%   13.8%   36.3%    8.4%   51.8%   10.1%    4.5%   41.3%    4.6%    4.2%   44.5%    2.2%   38.9%    5.5%    5.8%    6.4%   12.2%    4.3%    5.8%   55.9%    7.2%    4.6%    0.9%    7.6%   71.3%   55.8%    4.2%    3.4%    6.0%    4.6%   64.9%    6.8%    8.3%    0.9%    2.8%    1.3%    2.0%    0.4%    3.9%    2.4%    2.3%   10.9%    9.7%    7.5%    6.0%   12.0%   20.8%    4.1%    3.7%   43.5%   10.3%    4.8%   18.5%    1.6%   21.8%   14.3%    4.5%   15.4%    1.8%   17.2%   11.6%   40.0%    4.6%    7.7%   44.2%    2.4%    3.6%    2.7%    2.6%   18.8%   14.9%   11.4%    9.9%    1.2%    1.1%   32.1%    4.0%    1.4%   31.3%    7.5%   18.1%   20.9%   39.5%   18.6%    2.0%    0.3%    1.7%    1.0%    0.8%    0.1%    6.8%    0.5%    0.6%   29.5%    0.7%    0.4%    2.1%    2.6%    8.2%   18.0%   41.5%   57.1%   36.8%   18.1%    1.6%   32.9%    1.2%    2.5%    1.6%   13.6%    5.1%    0.9%   56.6%   14.5%    0.4%    3.0%    0.6%    0.9%    9.3%    6.2%    4.2%    1.0%    4.0%   24.1%    1.2%    1.5%    0.5%   36.2%    3.2%   12.8%    3.7%    2.5%    0.1%    0.2%    1.1%    5.9%    2.1%    1.3%    1.2%    0.2%    0.6%    2.2%   10.2%    7.2%    1.1%    2.6%    1.9%    0.3%   10.4%    0.1%    2.2%    0.8%    4.1%    0.9%    4.2%   39.6%    0.3%    0.1%    0.8%    0.1%    0.8%    1.8%    0.7%   78.4%    2.0%    0.0%    0.2%    0.3%    0.2%    0.3%   60.3%   11.7%    1.5%    4.8%    9.7%    1.5%    2.7%    1.2%    0.2%    0.8%    6.6%    0.4%    0.8%    0.1%    1.2%    0.8%    1.0%    2.6%    1.2%    1.4%    2.0%    1.6%    0.1%    1.8%    0.3%    6.4%    8.8%   25.5%    1.2%    9.8%    3.7%   20.0%    8.6%    3.2%    0.4%        k__Bacteria;p__Cyanobacteria;c__; o__Chroococcales      16    0.0%    0.0%    0.0%    0.0%    0.0%    0.0%    0.0%    0.0%    0.0%    0.0%    0.0%    0.0%    0.0%    0.0%    0.0%    0.0%    0.0%    0.0%    0.0%    0.0%    0.0%    0.0%    0.0%    0.0%    0.0%    0.0%    0.0%    0.0%    0.0%    0.0%    0.0%    0.0%    0.0%    0.0%    0.0%    0.0%    0.0%    0.0%    0.0%    0.0%    0.0%    0.0%    0.0%    0.0%    0.0%    0.0%    0.0%    0.0%    0.0%    0.0%    0.0%    0.0%    0.0%    0.0%    0.0%    0.0%    0.0%    0.0%    0.0%    0.0%    0.0%    0.0%    0.0%    0.0%    0.0%    0.0%    0.0%    0.0%    0.0%    0.0%    0.0%    0.0%    0.0%    0.0%    0.0%    0.0%    0.0%    0.0%    0.0%    0.0%    0.0%    0.0%    0.0%    0.0%    0.0%    0.0%    0.0%    0.0%    0.0%    0.0%    0.0%    0.0%    0.0%    0.0%    0.0%    0.0%    0.0%    0.0%    0.0%    0.0%    0.0%    0.0%    0.0%    0.0%    0.0%    0.0%    0.0%    0.0%    0.0%    0.0%    0.0%    0.0%    0.0%    0.0%    0.0%    0.0%    0.0%    0.0%    0.0%    0.0%    0.0%    0.0%    0.0%    0.0%    0.0%    0.0%    0.0%    0.0%    0.0%    0.0%    0.0%    0.0%    0.0%    0.0%    0.0%    0.0%    0.0%    0.0%    0.0%    0.0%    0.0%    0.0%    0.0%    0.0%    0.0%    0.0%    0.0%    0.0%    0.0%    0.0%    0.0%    0.0%    0.0%    0.0%    0.0%    0.0%    0.0%    0.0%    0.0%    0.0%    0.0%    0.0%    0.0%    0.0%    0.0%    0.0%    0.0%    0.0%    0.0%    0.0%    0.0%    0.0%    0.0%    0.0%    0.0%    0.0%    0.0%    0.0%    0.0%    0.0%    0.0%    0.0%    0.0%    0.0%    0.0%    0.0%    0.0%    0.0%    0.0%    0.0%    0.0%    0.0%    0.0%    0.0%    0.0%    0.0%    0.0%    0.0%    0.0%    0.0%    0.0%    0.0%    0.0%    0.0%    0.0%    0.0%    0.0%    0.0%    0.0%    0.0%    0.0%    0.0%    0.0%    0.0%    0.0%    0.0%    0.0%    0.0%    0.0%    0.0%    0.0%    0.0%    0.0%    0.0%    0.0%    0.0%    0.0%    0.0%    0.0%    0.0%    0.0%    0.0%    0.0%    0.0%    0.0%    0.0%    0.0%    0.0%    0.0%    0.0%    0.0%    0.0%    0.0%    0.0%    0.0%    0.0%    0.0%    0.0%    0.0%    0.0%    0.0%    0.0%    0.0%    0.0%    0.0%    0.0%    0.0%    0.0%    0.0%    0.0%    0.0%    0.0%    0.0%    0.0%    0.0%    0.0%    0.0%    0.0%    0.0%    0.0%    0.0%    0.0%    0.0%    0.0%    0.0%    0.0%    0.0%    0.0%    0.0%    0.0%    0.0%    0.0%    0.0%    0.0%    0.0%    0.0%    0.0%    0.0%    0.0%    0.0%    0.0%    0.0%    0.0%    0.0%    0.0%    0.0%    0.0%    0.0%    0.0%    0.0%    0.0%    0.0%    0.0%    0.0%    0.0%    0.0%    0.0%    0.0%    0.0%    0.0%    0.0%    0.0%    0.0%    0.0%    0.0%    0.0%    0.0%    0.0%    0.0%    0.0%    0.0%    0.0%    0.0%    0.0%    0.0%    0.0%    0.0%    0.0%    0.0%    0.0%    0.0%    0.0%    0.0%    0.0%    0.0%    0.0%    0.0%    0.0%    0.0%    0.0%    0.0%    0.0%    0.0%    0.0%    0.0%    0.0%    0.0%    0.0%    0.0%    0.0%    0.0%    0.0%    0.0%    0.0%    0.0%    0.0%    0.0%    0.0%        k__Bacteria;p__Cyanobacteria;c__; o__Nostocales      44    0.0%    0.0%    0.0%    0.0%    0.0%    0.0%    0.0%    0.0%    0.0%    0.0%    0.0%    0.0%    0.0%    0.0%    0.0%    0.0%    0.0%    0.0%    0.0%    0.0%    0.0%    0.0%    0.0%    0.0%    0.0%    0.0%    0.0%    0.0%    0.0%    0.0%    0.0%    0.0%    0.0%    0.0%    0.0%    0.0%    0.0%    0.0%    0.0%    0.0%    0.0%    0.0%    0.0%    0.0%    0.0%    0.0%    0.0%    0.0%    0.0%    0.0%    0.0%    0.0%    0.0%    0.0%    0.0%    0.0%    0.0%    0.0%    0.0%    0.0%    0.0%    0.0%    0.0%    0.0%    0.0%    0.0%    0.0%    0.0%    0.0%    0.0%    0.0%    0.0%    0.0%    0.0%    0.0%    0.0%    0.0%    0.0%    0.0%    0.0%    0.0%    0.0%    0.0%    0.0%    0.0%    0.0%    0.0%    0.0%    0.0%    0.0%    0.0%    0.0%    0.0%    0.0%    0.0%    0.0%    0.0%    0.0%    0.0%    0.0%    0.0%    0.0%    0.0%    0.0%    0.0%    0.0%    0.0%    0.0%    0.0%    0.0%    0.0%    0.0%    0.0%    0.0%    0.0%    0.0%    0.0%    0.0%    0.0%    0.0%    0.0%    0.0%    0.0%    0.0%    0.0%    0.0%    0.0%    0.0%    0.0%    0.0%    0.0%    0.0%    0.0%    0.0%    0.0%    0.0%    0.0%    0.0%    0.0%    0.0%    0.0%    0.0%    0.0%    0.0%    0.0%    0.0%    0.0%    0.0%    0.0%    0.0%    0.0%    0.0%    0.0%    0.0%    0.0%    0.0%    0.0%    0.0%    0.0%    0.0%    0.0%    0.0%    0.0%    0.0%    0.0%    0.0%    0.0%    0.0%    0.0%    0.0%    0.0%    0.0%    0.0%    0.0%    0.0%    0.0%    0.0%    0.0%    0.0%    0.0%    0.0%    0.0%    0.0%    0.0%    0.0%    0.0%    0.0%    0.0%    0.0%    0.0%    0.0%    0.0%    0.0%    0.0%    0.0%    0.0%    0.0%    0.0%    0.0%    0.0%    0.0%    0.0%    0.0%    0.0%    0.0%    0.0%    0.0%    0.0%    0.0%    0.0%    0.0%    0.0%    0.0%    0.0%    0.0%    0.0%    0.0%    0.0%    0.0%    0.0%    0.0%    0.0%    0.0%    0.0%    0.0%    0.0%    0.0%    0.0%    0.0%    0.0%    0.0%    0.0%    0.0%    0.0%    0.0%    0.0%    0.0%    0.0%    0.0%    0.0%    0.0%    0.0%    0.0%    0.0%    0.0%    0.0%    0.0%    0.0%    0.0%    0.0%    0.0%    0.0%    0.0%    0.0%    0.0%    0.0%    0.0%    0.0%    0.0%    0.0%    0.0%    0.0%    0.0%    0.0%    0.0%    0.0%    0.0%    0.0%    0.0%    0.0%    0.0%    0.0%    0.0%    0.0%    0.0%    0.0%    0.0%    0.0%    0.0%    0.0%    0.0%    0.0%    0.0%    0.0%    0.0%    0.0%    0.0%    0.0%    0.0%    0.0%    0.0%    0.0%    0.0%    0.0%    0.0%    0.0%    0.0%    0.0%    0.0%    0.0%    0.0%    0.0%    0.0%    0.0%    0.0%    0.0%    0.0%    0.0%    0.0%    0.0%    0.0%    0.0%    0.0%    0.0%    0.0%    0.0%    0.0%    0.0%    0.0%    0.0%    0.0%    0.0%    0.0%    0.0%    0.0%    0.0%    0.0%    0.0%    0.0%    0.0%    0.0%    0.0%    0.0%    0.0%    0.0%    0.0%    0.0%    0.0%    0.0%    0.0%    0.0%    0.0%    0.0%    0.0%    0.0%    0.0%    0.0%    0.0%    0.0%    0.0%    0.0%    0.0%    0.0%    0.0%    0.0%    0.0%    0.0%    0.0%        k__Bacteria;p__Cyanobacteria;c__; o__Oscillatoriales    1005    0.0%    0.0%    0.0%    0.0%    0.0%    0.0%    0.0%    0.0%    0.0%    0.0%    0.0%    0.0%    0.0%    0.0%    0.0%    0.0%    0.0%    0.0%    0.0%    0.0%    0.0%    0.0%    0.0%    0.0%    0.0%    0.0%    0.0%    0.0%    0.0%    0.0%    0.0%    0.0%    0.0%    0.0%    0.0%    0.0%    0.0%    0.0%    0.0%    0.0%    0.0%    0.0%    0.0%    0.0%    0.0%    0.0%    0.0%    0.0%    0.0%    0.0%    0.0%    0.0%    0.0%    0.0%    0.0%    0.0%    0.0%    0.0%    0.0%    0.0%    0.0%    0.0%    0.0%    0.0%    0.0%    0.0%    0.0%    0.0%    0.0%    0.0%    0.0%    0.0%    0.0%    0.0%    0.0%    0.0%    0.0%    0.0%    0.0%    0.0%    0.0%    0.0%    0.0%    0.0%    0.0%    0.0%    0.0%    0.0%    0.0%    0.0%    0.0%    0.0%    0.0%    0.0%    0.0%    0.0%    0.0%    0.0%    0.0%    0.0%    0.0%    0.0%    0.0%    0.0%    0.0%    0.0%    0.0%    0.0%    0.0%    0.0%    0.0%    0.0%    0.0%    0.0%    0.0%    0.0%    0.0%    0.0%    0.0%    0.0%    0.0%    0.0%    0.0%    0.0%    0.0%    0.0%    0.0%    0.0%    0.0%    0.0%    0.0%    0.0%    0.0%    0.0%    0.0%    0.0%    0.0%    0.0%    0.0%    0.0%    0.0%    0.0%    0.0%    0.0%    0.0%    0.0%    0.0%    0.0%    0.0%    0.0%    0.0%    0.0%    0.0%    0.0%    0.0%    0.0%    0.0%    0.0%    0.0%    0.0%    0.0%    0.0%    0.0%    0.0%    0.0%    0.0%    0.0%    0.0%    0.0%    0.0%    0.0%    0.0%    0.0%    0.0%    0.0%    0.0%    0.0%    0.0%    0.0%    0.0%    0.0%    0.0%    0.0%    0.0%    0.0%    0.0%    0.0%    0.0%    0.0%    0.0%    0.0%    0.0%    0.0%    0.0%    0.0%    0.0%    0.0%    0.0%    0.0%    0.0%    0.0%    0.0%    0.0%    0.0%    0.0%    0.0%    0.0%    0.0%    0.0%    0.0%    0.0%    0.0%    0.0%    0.0%    0.0%    0.0%    0.0%    0.0%    0.0%    0.0%    0.0%    0.0%    0.0%    0.0%    0.0%    0.0%    0.0%    0.0%    0.0%    0.0%    0.0%    0.0%    0.0%    0.0%    0.0%    0.0%    0.1%    0.0%    0.0%    0.0%    0.0%    0.0%    0.0%    0.0%    0.0%    0.1%    0.1%    0.0%    0.0%    0.0%    0.0%    0.0%    0.0%    0.0%    0.0%    0.0%    0.0%    0.0%    0.0%    0.0%    0.0%    0.0%    0.0%    0.0%    0.0%    0.0%    0.0%    0.0%    0.0%    0.0%    0.0%    0.0%    0.0%    0.0%    0.0%    0.0%    0.0%    0.0%    0.0%    0.0%    0.0%    0.0%    0.0%    0.0%    0.0%    0.0%    0.0%    0.1%    0.0%    0.0%    0.0%    0.0%    0.1%    0.0%    0.0%    0.0%    0.0%    0.0%    0.0%    0.1%    0.0%    0.0%    0.0%    0.0%    0.0%    0.0%    0.0%    0.0%    0.0%    0.0%    0.0%    0.0%    0.0%    0.0%    0.0%    0.0%    0.0%    0.0%    0.0%    0.0%    0.0%    0.0%    0.0%    0.0%    0.0%    0.0%    0.0%    0.0%    0.0%    0.0%    0.0%    0.0%    0.0%    0.0%    0.0%    0.0%    0.0%    0.0%    0.0%    0.0%    0.0%    0.0%    0.0%    0.0%    0.0%    0.0%    0.0%    0.0%    0.0%    0.0%    0.0%    0.0%    0.0%    0.0%    0.0%    0.0%    0.0%    0.0%        k__Bacteria;p__Cyanobacteria;c__; o__Pleurocapsales      23    0.0%    0.0%    0.0%    0.0%    0.0%    0.0%    0.0%    0.0%    0.0%    0.0%    0.0%    0.0%    0.0%    0.0%    0.0%    0.0%    0.0%    0.0%    0.0%    0.0%    0.0%    0.0%    0.0%    0.0%    0.0%    0.0%    0.0%    0.0%    0.0%    0.0%    0.0%    0.0%    0.0%    0.0%    0.0%    0.0%    0.0%    0.0%    0.0%    0.0%    0.0%    0.0%    0.0%    0.0%    0.0%    0.0%    0.0%    0.0%    0.0%    0.0%    0.0%    0.0%    0.0%    0.0%    0.0%    0.0%    0.0%    0.0%    0.0%    0.0%    0.0%    0.0%    0.0%    0.0%    0.0%    0.0%    0.0%    0.0%    0.0%    0.0%    0.0%    0.0%    0.0%    0.0%    0.0%    0.0%    0.0%    0.0%    0.0%    0.0%    0.0%    0.0%    0.0%    0.0%    0.0%    0.0%    0.0%    0.0%    0.0%    0.0%    0.0%    0.0%    0.0%    0.0%    0.0%    0.0%    0.0%    0.0%    0.0%    0.0%    0.0%    0.0%    0.0%    0.0%    0.0%    0.0%    0.0%    0.0%    0.0%    0.0%    0.0%    0.0%    0.0%    0.0%    0.0%    0.0%    0.0%    0.0%    0.0%    0.0%    0.0%    0.0%    0.0%    0.0%    0.0%    0.0%    0.0%    0.0%    0.0%    0.0%    0.0%    0.0%    0.0%    0.0%    0.0%    0.0%    0.0%    0.0%    0.0%    0.0%    0.0%    0.0%    0.0%    0.0%    0.0%    0.0%    0.0%    0.0%    0.0%    0.0%    0.0%    0.0%    0.0%    0.0%    0.0%    0.0%    0.0%    0.0%    0.0%    0.0%    0.0%    0.0%    0.0%    0.0%    0.0%    0.0%    0.0%    0.0%    0.0%    0.0%    0.0%    0.0%    0.0%    0.0%    0.0%    0.0%    0.0%    0.0%    0.0%    0.0%    0.0%    0.0%    0.0%    0.0%    0.0%    0.0%    0.0%    0.0%    0.0%    0.0%    0.0%    0.0%    0.0%    0.0%    0.0%    0.0%    0.0%    0.0%    0.0%    0.0%    0.0%    0.0%    0.0%    0.0%    0.0%    0.0%    0.0%    0.0%    0.0%    0.0%    0.0%    0.0%    0.0%    0.0%    0.0%    0.0%    0.0%    0.0%    0.0%    0.0%    0.0%    0.0%    0.0%    0.0%    0.0%    0.0%    0.0%    0.0%    0.0%    0.0%    0.0%    0.0%    0.0%    0.0%    0.0%    0.0%    0.0%    0.0%    0.0%    0.0%    0.0%    0.0%    0.0%    0.0%    0.0%    0.0%    0.0%    0.0%    0.0%    0.0%    0.0%    0.0%    0.0%    0.0%    0.0%    0.0%    0.0%    0.0%    0.0%    0.0%    0.0%    0.0%    0.0%    0.0%    0.0%    0.0%    0.0%    0.0%    0.0%    0.0%    0.0%    0.0%    0.0%    0.0%    0.0%    0.0%    0.0%    0.0%    0.0%    0.0%    0.0%    0.0%    0.0%    0.0%    0.0%    0.0%    0.0%    0.0%    0.0%    0.0%    0.0%    0.0%    0.0%    0.0%    0.0%    0.0%    0.0%    0.0%    0.0%    0.0%    0.0%    0.0%    0.0%    0.0%    0.0%    0.0%    0.0%    0.0%    0.0%    0.0%    0.0%    0.0%    0.0%    0.0%    0.0%    0.0%    0.0%    0.0%    0.0%    0.0%    0.0%    0.0%    0.0%    0.0%    0.0%    0.0%    0.0%    0.0%    0.0%    0.0%    0.0%    0.0%    0.0%    0.0%    0.0%    0.0%    0.0%    0.0%    0.0%    0.0%    0.0%    0.0%    0.0%    0.0%    0.0%    0.0%    0.0%    0.0%    0.0%    0.0%    0.0%    0.0%    0.0%    0.0%    0.0%    0.0%    0.0%    0.0%        k__Bacteria;p__Cyanobacteria;c__; o__Stigonematales       0    0.0%    0.0%    0.0%    0.0%    0.0%    0.0%    0.0%    0.0%    0.0%    0.0%    0.0%    0.0%    0.0%    0.0%    0.0%    0.0%    0.0%    0.0%    0.0%    0.0%    0.0%    0.0%    0.0%    0.0%    0.0%    0.0%    0.0%    0.0%    0.0%    0.0%    0.0%    0.0%    0.0%    0.0%    0.0%    0.0%    0.0%    0.0%    0.0%    0.0%    0.0%    0.0%    0.0%    0.0%    0.0%    0.0%    0.0%    0.0%    0.0%    0.0%    0.0%    0.0%    0.0%    0.0%    0.0%    0.0%    0.0%    0.0%    0.0%    0.0%    0.0%    0.0%    0.0%    0.0%    0.0%    0.0%    0.0%    0.0%    0.0%    0.0%    0.0%    0.0%    0.0%    0.0%    0.0%    0.0%    0.0%    0.0%    0.0%    0.0%    0.0%    0.0%    0.0%    0.0%    0.0%    0.0%    0.0%    0.0%    0.0%    0.0%    0.0%    0.0%    0.0%    0.0%    0.0%    0.0%    0.0%    0.0%    0.0%    0.0%    0.0%    0.0%    0.0%    0.0%    0.0%    0.0%    0.0%    0.0%    0.0%    0.0%    0.0%    0.0%    0.0%    0.0%    0.0%    0.0%    0.0%    0.0%    0.0%    0.0%    0.0%    0.0%    0.0%    0.0%    0.0%    0.0%    0.0%    0.0%    0.0%    0.0%    0.0%    0.0%    0.0%    0.0%    0.0%    0.0%    0.0%    0.0%    0.0%    0.0%    0.0%    0.0%    0.0%    0.0%    0.0%    0.0%    0.0%    0.0%    0.0%    0.0%    0.0%    0.0%    0.0%    0.0%    0.0%    0.0%    0.0%    0.0%    0.0%    0.0%    0.0%    0.0%    0.0%    0.0%    0.0%    0.0%    0.0%    0.0%    0.0%    0.0%    0.0%    0.0%    0.0%    0.0%    0.0%    0.0%    0.0%    0.0%    0.0%    0.0%    0.0%    0.0%    0.0%    0.0%    0.0%    0.0%    0.0%    0.0%    0.0%    0.0%    0.0%    0.0%    0.0%    0.0%    0.0%    0.0%    0.0%    0.0%    0.0%    0.0%    0.0%    0.0%    0.0%    0.0%    0.0%    0.0%    0.0%    0.0%    0.0%    0.0%    0.0%    0.0%    0.0%    0.0%    0.0%    0.0%    0.0%    0.0%    0.0%    0.0%    0.0%    0.0%    0.0%    0.0%    0.0%    0.0%    0.0%    0.0%    0.0%    0.0%    0.0%    0.0%    0.0%    0.0%    0.0%    0.0%    0.0%    0.0%    0.0%    0.0%    0.0%    0.0%    0.0%    0.0%    0.0%    0.0%    0.0%    0.0%    0.0%    0.0%    0.0%    0.0%    0.0%    0.0%    0.0%    0.0%    0.0%    0.0%    0.0%    0.0%    0.0%    0.0%    0.0%    0.0%    0.0%    0.0%    0.0%    0.0%    0.0%    0.0%    0.0%    0.0%    0.0%    0.0%    0.0%    0.0%    0.0%    0.0%    0.0%    0.0%    0.0%    0.0%    0.0%    0.0%    0.0%    0.0%    0.0%    0.0%    0.0%    0.0%    0.0%    0.0%    0.0%    0.0%    0.0%    0.0%    0.0%    0.0%    0.0%    0.0%    0.0%    0.0%    0.0%    0.0%    0.0%    0.0%    0.0%    0.0%    0.0%    0.0%    0.0%    0.0%    0.0%    0.0%    0.0%    0.0%    0.0%    0.0%    0.0%    0.0%    0.0%    0.0%    0.0%    0.0%    0.0%    0.0%    0.0%    0.0%    0.0%    0.0%    0.0%    0.0%    0.0%    0.0%    0.0%    0.0%    0.0%    0.0%    0.0%    0.0%    0.0%    0.0%    0.0%    0.0%    0.0%    0.0%    0.0%    0.0%    0.0%    0.0%    0.0%    0.0%    0.0%    0.0%    0.0%    0.0%    0.0%    0.0%        k__Bacteria;p__Cyanobacteria;c__S15B-MN24; o__     250    0.0%    0.0%    0.0%    0.0%    0.0%    0.0%    0.0%    0.0%    0.0%    0.0%    0.0%    0.0%    0.0%    0.0%    0.0%    0.0%    0.0%    0.0%    0.0%    0.0%    0.0%    0.0%    0.0%    0.0%    0.0%    0.0%    0.0%    0.0%    0.0%    0.0%    0.0%    0.0%    0.0%    0.0%    0.0%    0.0%    0.0%    0.0%    0.0%    0.0%    0.0%    0.0%    0.0%    0.0%    0.0%    0.0%    0.0%    0.0%    0.0%    0.0%    0.0%    0.0%    0.0%    0.0%    0.0%    0.0%    0.0%    0.0%    0.0%    0.0%    0.0%    0.0%    0.0%    0.0%    0.0%    0.0%    0.0%    0.0%    0.0%    0.0%    0.0%    0.0%    0.0%    0.0%    0.0%    0.0%    0.0%    0.0%    0.0%    0.0%    0.0%    0.1%    0.0%    0.0%    0.0%    0.0%    0.0%    0.0%    0.0%    0.0%    0.0%    0.0%    0.0%    0.0%    0.0%    0.0%    0.0%    0.0%    0.0%    0.0%    0.0%    0.0%    0.0%    0.0%    0.0%    0.0%    0.0%    0.0%    0.0%    0.0%    0.0%    0.0%    0.0%    0.0%    0.0%    0.0%    0.0%    0.0%    0.0%    0.0%    0.0%    0.0%    0.0%    0.0%    0.0%    0.0%    0.0%    0.0%    0.0%    0.0%    0.0%    0.0%    0.0%    0.0%    0.0%    0.0%    0.0%    0.0%    0.0%    0.0%    0.0%    0.0%    0.0%    0.0%    0.0%    0.0%    0.0%    0.0%    0.0%    0.0%    0.0%    0.0%    0.0%    0.0%    0.0%    0.0%    0.0%    0.0%    0.0%    0.0%    0.0%    0.0%    0.0%    0.0%    0.0%    0.0%    0.0%    0.0%    0.0%    0.0%    0.0%    0.0%    0.0%    0.0%    0.0%    0.0%    0.0%    0.0%    0.0%    0.0%    0.0%    0.0%    0.0%    0.0%    0.0%    0.0%    0.0%    0.0%    0.0%    0.0%    0.0%    0.0%    0.0%    0.0%    0.0%    0.0%    0.0%    0.0%    0.0%    0.0%    0.0%    0.0%    0.0%    0.0%    0.0%    0.0%    0.0%    0.0%    0.0%    0.0%    0.0%    0.0%    0.0%    0.0%    0.0%    0.0%    0.0%    0.0%    0.0%    0.0%    0.0%    0.0%    0.0%    0.0%    0.0%    0.0%    0.0%    0.0%    0.0%    0.0%    0.0%    0.0%    0.0%    0.0%    0.0%    0.0%    0.0%    0.0%    0.0%    0.0%    0.0%    0.0%    0.0%    0.0%    0.0%    0.0%    0.0%    0.0%    0.0%    0.0%    0.0%    0.0%    0.0%    0.0%    0.0%    0.0%    0.0%    0.0%    0.0%    0.0%    0.0%    0.0%    0.0%    0.0%    0.0%    0.0%    0.0%    0.0%    0.0%    0.0%    0.0%    0.0%    0.0%    0.0%    0.0%    0.0%    0.0%    0.0%    0.0%    0.0%    0.0%    0.0%    0.0%    0.0%    0.0%    0.0%    0.0%    0.0%    0.0%    0.0%    0.0%    0.0%    0.0%    0.0%    0.0%    0.0%    0.0%    0.0%    0.0%    0.0%    0.0%    0.0%    0.0%    0.0%    0.0%    0.0%    0.0%    0.0%    0.0%    0.0%    0.0%    0.0%    0.0%    0.0%    0.0%    0.0%    0.0%    0.0%    0.0%    0.0%    0.0%    0.0%    0.0%    0.0%    0.0%    0.0%    0.0%    0.0%    0.0%    0.0%    0.0%    0.0%    0.0%    0.0%    0.0%    0.0%    0.0%    0.0%    0.0%    0.0%    0.0%    0.0%    0.0%    0.0%    0.0%    0.0%    0.0%    0.0%    0.0%    0.0%    0.0%    0.0%    0.0%    0.0%    0.0%    0.0%    0.0%    0.0%        k__Bacteria;p__Cyanobacteria;c__SM1D11; o__       6    0.0%    0.0%    0.0%    0.0%    0.0%    0.0%    0.0%    0.0%    0.0%    0.0%    0.0%    0.0%    0.0%    0.0%    0.0%    0.0%    0.0%    0.0%    0.0%    0.0%    0.0%    0.0%    0.0%    0.0%    0.0%    0.0%    0.0%    0.0%    0.0%    0.0%    0.0%    0.0%    0.0%    0.0%    0.0%    0.0%    0.0%    0.0%    0.0%    0.0%    0.0%    0.0%    0.0%    0.0%    0.0%    0.0%    0.0%    0.0%    0.0%    0.0%    0.0%    0.0%    0.0%    0.0%    0.0%    0.0%    0.0%    0.0%    0.0%    0.0%    0.0%    0.0%    0.0%    0.0%    0.0%    0.0%    0.0%    0.0%    0.0%    0.0%    0.0%    0.0%    0.0%    0.0%    0.0%    0.0%    0.0%    0.0%    0.0%    0.0%    0.0%    0.0%    0.0%    0.0%    0.0%    0.0%    0.0%    0.0%    0.0%    0.0%    0.0%    0.0%    0.0%    0.0%    0.0%    0.0%    0.0%    0.0%    0.0%    0.0%    0.0%    0.0%    0.0%    0.0%    0.0%    0.0%    0.0%    0.0%    0.0%    0.0%    0.0%    0.0%    0.0%    0.0%    0.0%    0.0%    0.0%    0.0%    0.0%    0.0%    0.0%    0.0%    0.0%    0.0%    0.0%    0.0%    0.0%    0.0%    0.0%    0.0%    0.0%    0.0%    0.0%    0.0%    0.0%    0.0%    0.0%    0.0%    0.0%    0.0%    0.0%    0.0%    0.0%    0.0%    0.0%    0.0%    0.0%    0.0%    0.0%    0.0%    0.0%    0.0%    0.0%    0.0%    0.0%    0.0%    0.0%    0.0%    0.0%    0.0%    0.0%    0.0%    0.0%    0.0%    0.0%    0.0%    0.0%    0.0%    0.0%    0.0%    0.0%    0.0%    0.0%    0.0%    0.0%    0.0%    0.0%    0.0%    0.0%    0.0%    0.0%    0.0%    0.0%    0.0%    0.0%    0.0%    0.0%    0.0%    0.0%    0.0%    0.0%    0.0%    0.0%    0.0%    0.0%    0.0%    0.0%    0.0%    0.0%    0.0%    0.0%    0.0%    0.0%    0.0%    0.0%    0.0%    0.0%    0.0%    0.0%    0.0%    0.0%    0.0%    0.0%    0.0%    0.0%    0.0%    0.0%    0.0%    0.0%    0.0%    0.0%    0.0%    0.0%    0.0%    0.0%    0.0%    0.0%    0.0%    0.0%    0.0%    0.0%    0.0%    0.0%    0.0%    0.0%    0.0%    0.0%    0.0%    0.0%    0.0%    0.0%    0.0%    0.0%    0.0%    0.0%    0.0%    0.0%    0.0%    0.0%    0.0%    0.0%    0.0%    0.0%    0.0%    0.0%    0.0%    0.0%    0.0%    0.0%    0.0%    0.0%    0.0%    0.0%    0.0%    0.0%    0.0%    0.0%    0.0%    0.0%    0.0%    0.0%    0.0%    0.0%    0.0%    0.0%    0.0%    0.0%    0.0%    0.0%    0.0%    0.0%    0.0%    0.0%    0.0%    0.0%    0.0%    0.0%    0.0%    0.0%    0.0%    0.0%    0.0%    0.0%    0.0%    0.0%    0.0%    0.0%    0.0%    0.0%    0.0%    0.0%    0.0%    0.0%    0.0%    0.0%    0.0%    0.0%    0.0%    0.0%    0.0%    0.0%    0.0%    0.0%    0.0%    0.0%    0.0%    0.0%    0.0%    0.0%    0.0%    0.0%    0.0%    0.0%    0.0%    0.0%    0.0%    0.0%    0.0%    0.0%    0.0%    0.0%    0.0%    0.0%    0.0%    0.0%    0.0%    0.0%    0.0%    0.0%    0.0%    0.0%    0.0%    0.0%    0.0%    0.0%    0.0%    0.0%    0.0%    0.0%    0.0%    0.0%    0.0%    0.0%    0.0%    0.0%    0.0%    0.0%    0.0%        k__Bacteria;p__Cyanobacteria;c__YS2; o__      68    0.0%    0.0%    0.0%    0.0%    0.0%    0.0%    0.0%    0.0%    0.0%    0.0%    0.0%    0.0%    0.0%    0.0%    0.0%    0.0%    0.0%    0.0%    0.0%    0.0%    0.0%    0.0%    0.0%    0.0%    0.0%    0.0%    0.0%    0.0%    0.0%    0.0%    0.0%    0.0%    0.0%    0.0%    0.0%    0.0%    0.0%    0.0%    0.0%    0.0%    0.0%    0.0%    0.0%    0.0%    0.0%    0.0%    0.0%    0.0%    0.0%    0.0%    0.0%    0.0%    0.0%    0.0%    0.0%    0.0%    0.0%    0.0%    0.0%    0.0%    0.0%    0.0%    0.0%    0.0%    0.0%    0.0%    0.0%    0.0%    0.0%    0.0%    0.0%    0.0%    0.0%    0.0%    0.0%    0.0%    0.0%    0.0%    0.0%    0.0%    0.0%    0.0%    0.0%    0.0%    0.0%    0.0%    0.0%    0.0%    0.0%    0.0%    0.0%    0.0%    0.0%    0.0%    0.0%    0.0%    0.0%    0.0%    0.0%    0.0%    0.0%    0.0%    0.0%    0.0%    0.0%    0.0%    0.0%    0.0%    0.0%    0.0%    0.0%    0.0%    0.0%    0.0%    0.0%    0.0%    0.0%    0.0%    0.0%    0.0%    0.0%    0.0%    0.0%    0.0%    0.0%    0.0%    0.0%    0.0%    0.0%    0.0%    0.0%    0.0%    0.0%    0.0%    0.0%    0.0%    0.0%    0.0%    0.0%    0.0%    0.0%    0.0%    0.0%    0.0%    0.0%    0.0%    0.0%    0.0%    0.0%    0.0%    0.0%    0.0%    0.0%    0.0%    0.0%    0.0%    0.0%    0.0%    0.0%    0.0%    0.0%    0.0%    0.0%    0.0%    0.0%    0.0%    0.0%    0.0%    0.0%    0.0%    0.0%    0.0%    0.0%    0.0%    0.0%    0.0%    0.0%    0.0%    0.0%    0.0%    0.0%    0.0%    0.0%    0.0%    0.0%    0.0%    0.0%    0.0%    0.0%    0.0%    0.0%    0.0%    0.0%    0.0%    0.0%    0.0%    0.0%    0.0%    0.0%    0.0%    0.0%    0.0%    0.0%    0.0%    0.0%    0.0%    0.0%    0.0%    0.0%    0.0%    0.0%    0.0%    0.0%    0.0%    0.0%    0.0%    0.0%    0.0%    0.0%    0.0%    0.0%    0.0%    0.0%    0.0%    0.0%    0.0%    0.0%    0.0%    0.0%    0.0%    0.0%    0.0%    0.0%    0.0%    0.0%    0.0%    0.0%    0.0%    0.0%    0.0%    0.0%    0.0%    0.0%    0.0%    0.0%    0.0%    0.0%    0.0%    0.0%    0.0%    0.0%    0.0%    0.0%    0.0%    0.0%    0.0%    0.0%    0.0%    0.0%    0.0%    0.0%    0.0%    0.0%    0.0%    0.0%    0.0%    0.0%    0.0%    0.0%    0.0%    0.0%    0.0%    0.0%    0.0%    0.0%    0.0%    0.0%    0.0%    0.0%    0.0%    0.0%    0.0%    0.0%    0.0%    0.0%    0.0%    0.0%    0.0%    0.0%    0.0%    0.0%    0.0%    0.0%    0.0%    0.0%    0.0%    0.0%    0.0%    0.0%    0.0%    0.0%    0.0%    0.0%    0.0%    0.0%    0.0%    0.0%    0.0%    0.0%    0.0%    0.0%    0.0%    0.0%    0.0%    0.0%    0.0%    0.0%    0.0%    0.0%    0.0%    0.0%    0.0%    0.0%    0.0%    0.0%    0.0%    0.0%    0.0%    0.0%    0.0%    0.0%    0.0%    0.0%    0.0%    0.0%    0.0%    0.0%    0.0%    0.0%    0.0%    0.0%    0.0%    0.0%    0.0%    0.0%    0.0%    0.0%    0.0%    0.0%    0.0%    0.0%    0.0%    0.0%    0.0%    0.0%    0.0%    0.0%    0.0%        k__Bacteria;p__Cyanobacteria;c__mle1-12; o__      27    0.0%    0.0%    0.0%    0.0%    0.0%    0.0%    0.0%    0.0%    0.0%    0.0%    0.0%    0.0%    0.0%    0.0%    0.0%    0.0%    0.0%    0.0%    0.0%    0.0%    0.0%    0.0%    0.0%    0.0%    0.0%    0.0%    0.0%    0.0%    0.0%    0.0%    0.0%    0.0%    0.0%    0.0%    0.0%    0.0%    0.0%    0.0%    0.0%    0.0%    0.0%    0.0%    0.0%    0.0%    0.0%    0.0%    0.0%    0.0%    0.0%    0.0%    0.0%    0.0%    0.0%    0.0%    0.0%    0.0%    0.0%    0.0%    0.0%    0.0%    0.0%    0.0%    0.0%    0.0%    0.0%    0.0%    0.0%    0.0%    0.0%    0.0%    0.0%    0.0%    0.0%    0.0%    0.0%    0.0%    0.0%    0.0%    0.0%    0.0%    0.0%    0.0%    0.0%    0.0%    0.0%    0.0%    0.0%    0.0%    0.0%    0.0%    0.0%    0.0%    0.0%    0.0%    0.0%    0.0%    0.0%    0.0%    0.0%    0.0%    0.0%    0.0%    0.0%    0.0%    0.0%    0.0%    0.0%    0.0%    0.0%    0.0%    0.0%    0.0%    0.0%    0.0%    0.0%    0.0%    0.0%    0.0%    0.0%    0.0%    0.0%    0.0%    0.0%    0.0%    0.0%    0.0%    0.0%    0.0%    0.0%    0.0%    0.0%    0.0%    0.0%    0.0%    0.0%    0.0%    0.0%    0.0%    0.0%    0.0%    0.0%    0.0%    0.0%    0.0%    0.0%    0.0%    0.0%    0.0%    0.0%    0.0%    0.0%    0.0%    0.0%    0.0%    0.0%    0.0%    0.0%    0.0%    0.0%    0.0%    0.0%    0.0%    0.0%    0.0%    0.0%    0.0%    0.0%    0.0%    0.0%    0.0%    0.0%    0.0%    0.0%    0.0%    0.0%    0.0%    0.0%    0.0%    0.0%    0.0%    0.0%    0.0%    0.0%    0.0%    0.0%    0.0%    0.0%    0.0%    0.0%    0.0%    0.0%    0.0%    0.0%    0.0%    0.0%    0.0%    0.0%    0.0%    0.0%    0.0%    0.0%    0.0%    0.0%    0.0%    0.0%    0.0%    0.0%    0.0%    0.0%    0.0%    0.0%    0.0%    0.0%    0.0%    0.0%    0.0%    0.0%    0.0%    0.0%    0.0%    0.0%    0.0%    0.0%    0.0%    0.0%    0.0%    0.0%    0.0%    0.0%    0.0%    0.0%    0.0%    0.0%    0.0%    0.0%    0.0%    0.0%    0.0%    0.0%    0.0%    0.0%    0.0%    0.0%    0.0%    0.0%    0.0%    0.0%    0.0%    0.0%    0.0%    0.0%    0.0%    0.0%    0.0%    0.0%    0.0%    0.0%    0.0%    0.0%    0.0%    0.0%    0.0%    0.0%    0.0%    0.0%    0.0%    0.0%    0.0%    0.0%    0.0%    0.0%    0.0%    0.0%    0.0%    0.0%    0.0%    0.0%    0.0%    0.0%    0.0%    0.0%    0.0%    0.0%    0.0%    0.0%    0.0%    0.0%    0.0%    0.0%    0.0%    0.0%    0.0%    0.0%    0.0%    0.0%    0.0%    0.0%    0.0%    0.0%    0.0%    0.0%    0.0%    0.0%    0.0%    0.0%    0.0%    0.0%    0.0%    0.0%    0.0%    0.0%    0.0%    0.0%    0.0%    0.0%    0.0%    0.0%    0.0%    0.0%    0.0%    0.0%    0.0%    0.0%    0.0%    0.0%    0.0%    0.0%    0.0%    0.0%    0.0%    0.0%    0.0%    0.0%    0.0%    0.0%    0.0%    0.0%    0.0%    0.0%    0.0%    0.0%    0.0%    0.0%    0.0%    0.0%    0.0%    0.0%    0.0%    0.0%    0.0%    0.0%    0.0%    0.0%    0.0%    0.0%    0.0%    0.0%    0.0%        k__Bacteria;p__Deferribacteres;c__Deferribacteres (class); o__Deferribacterales     468    0.0%    0.0%    0.0%    0.0%    0.0%    0.0%    0.0%    0.0%    0.0%    0.0%    0.0%    0.0%    0.0%    0.0%    0.0%    0.0%    0.0%    0.0%    0.0%    0.0%    0.0%    0.0%    0.0%    0.0%    0.0%    0.0%    0.0%    0.0%    0.0%    0.0%    0.0%    0.0%    0.0%    0.0%    0.3%    0.0%    0.0%    0.0%    0.0%    0.0%    0.0%    0.0%    0.0%    0.0%    0.0%    0.0%    0.0%    0.0%    0.0%    0.0%    0.0%    0.0%    0.0%    0.0%    0.1%    0.0%    0.0%    0.0%    0.0%    0.0%    0.0%    0.0%    0.0%    0.0%    0.0%    0.0%    0.0%    0.0%    0.0%    0.0%    0.0%    0.0%    0.0%    0.0%    0.0%    0.0%    0.0%    0.0%    0.0%    0.0%    0.0%    0.0%    0.0%    0.0%    0.0%    0.0%    0.0%    0.0%    0.0%    0.0%    0.0%    0.0%    0.0%    0.0%    0.0%    0.0%    0.0%    0.0%    0.0%    0.0%    0.0%    0.0%    0.0%    0.0%    0.0%    0.0%    0.0%    0.0%    0.0%    0.0%    0.0%    0.0%    0.0%    0.0%    0.0%    0.0%    0.0%    0.0%    0.0%    0.0%    0.0%    0.0%    0.0%    0.0%    0.0%    0.0%    0.0%    0.0%    0.0%    0.0%    0.0%    0.0%    0.0%    0.0%    0.0%    0.0%    0.0%    0.0%    0.0%    0.0%    0.0%    0.0%    0.0%    0.0%    0.0%    0.0%    0.0%    0.0%    0.0%    0.0%    0.0%    0.0%    0.0%    0.0%    0.0%    0.0%    0.0%    0.0%    0.0%    0.0%    0.0%    0.0%    0.0%    0.0%    0.0%    0.0%    0.0%    0.0%    0.0%    0.0%    0.0%    0.0%    0.0%    0.0%    0.0%    0.0%    0.0%    0.0%    0.0%    0.0%    0.0%    0.0%    0.0%    0.0%    0.0%    0.0%    0.0%    0.0%    0.0%    0.0%    0.0%    0.0%    0.0%    0.0%    0.0%    0.0%    0.0%    0.0%    0.0%    0.0%    0.0%    0.1%    0.0%    0.0%    0.0%    0.0%    0.0%    0.0%    0.0%    0.0%    0.0%    0.0%    0.0%    0.0%    0.0%    0.0%    0.0%    0.0%    0.0%    0.0%    0.0%    0.0%    0.0%    0.0%    0.0%    0.0%    0.0%    0.0%    0.0%    0.0%    0.0%    0.0%    0.0%    0.0%    0.0%    0.0%    0.0%    0.0%    0.0%    0.0%    0.0%    0.0%    0.0%    0.0%    0.0%    0.0%    0.0%    0.0%    0.0%    0.0%    0.0%    0.0%    0.0%    0.0%    0.0%    0.0%    0.0%    0.0%    0.0%    0.0%    0.0%    0.0%    0.0%    0.0%    0.0%    0.0%    0.0%    0.0%    0.0%    0.0%    0.0%    0.0%    0.0%    0.0%    0.0%    0.0%    0.0%    0.0%    0.0%    0.0%    0.0%    0.0%    0.0%    0.0%    0.0%    0.0%    0.0%    0.0%    0.0%    0.0%    0.0%    0.0%    0.0%    0.0%    0.0%    0.0%    0.0%    0.0%    0.0%    0.0%    0.0%    0.0%    0.0%    0.0%    0.0%    0.0%    0.0%    0.0%    0.0%    0.0%    0.0%    0.0%    0.0%    0.0%    0.0%    0.0%    0.0%    0.0%    0.0%    0.0%    0.0%    0.0%    0.0%    0.0%    0.0%    0.0%    0.0%    0.0%    0.0%    0.0%    0.0%    0.0%    0.0%    0.0%    0.0%    0.0%    0.0%    0.0%    0.0%    0.0%    0.0%    0.0%    0.0%    0.0%    0.0%    0.0%    0.0%    0.0%    0.0%    0.0%    0.0%    0.0%    0.0%    0.0%    0.0%    0.0%    0.0%    0.0%        k__Bacteria;p__Elusimicrobia;c__Elusimicrobia (class); o__      31    0.0%    0.0%    0.0%    0.0%    0.0%    0.0%    0.0%    0.0%    0.0%    0.0%    0.0%    0.0%    0.0%    0.0%    0.0%    0.0%    0.0%    0.0%    0.0%    0.0%    0.0%    0.0%    0.0%    0.0%    0.0%    0.0%    0.0%    0.0%    0.0%    0.0%    0.0%    0.0%    0.0%    0.0%    0.0%    0.0%    0.0%    0.0%    0.0%    0.0%    0.0%    0.0%    0.0%    0.0%    0.0%    0.0%    0.0%    0.0%    0.0%    0.0%    0.0%    0.0%    0.0%    0.0%    0.0%    0.0%    0.0%    0.0%    0.0%    0.0%    0.0%    0.0%    0.0%    0.0%    0.0%    0.0%    0.0%    0.0%    0.0%    0.0%    0.0%    0.0%    0.0%    0.0%    0.0%    0.0%    0.0%    0.0%    0.0%    0.0%    0.0%    0.0%    0.0%    0.0%    0.0%    0.0%    0.0%    0.0%    0.0%    0.0%    0.0%    0.0%    0.0%    0.0%    0.0%    0.0%    0.0%    0.0%    0.0%    0.0%    0.0%    0.0%    0.0%    0.0%    0.0%    0.0%    0.0%    0.0%    0.0%    0.0%    0.0%    0.0%    0.0%    0.0%    0.0%    0.0%    0.0%    0.0%    0.0%    0.0%    0.0%    0.0%    0.0%    0.0%    0.0%    0.0%    0.0%    0.0%    0.0%    0.0%    0.0%    0.0%    0.0%    0.0%    0.0%    0.0%    0.0%    0.0%    0.0%    0.0%    0.0%    0.0%    0.0%    0.0%    0.0%    0.0%    0.0%    0.0%    0.0%    0.0%    0.0%    0.0%    0.0%    0.0%    0.0%    0.0%    0.0%    0.0%    0.0%    0.0%    0.0%    0.0%    0.0%    0.0%    0.0%    0.0%    0.0%    0.0%    0.0%    0.0%    0.0%    0.0%    0.0%    0.0%    0.0%    0.0%    0.0%    0.0%    0.0%    0.0%    0.0%    0.0%    0.0%    0.0%    0.0%    0.0%    0.0%    0.0%    0.0%    0.0%    0.0%    0.0%    0.0%    0.0%    0.0%    0.0%    0.0%    0.0%    0.0%    0.0%    0.0%    0.0%    0.0%    0.0%    0.0%    0.0%    0.0%    0.0%    0.0%    0.0%    0.0%    0.0%    0.0%    0.0%    0.0%    0.0%    0.0%    0.0%    0.0%    0.0%    0.0%    0.0%    0.0%    0.0%    0.0%    0.0%    0.0%    0.0%    0.0%    0.0%    0.0%    0.0%    0.0%    0.0%    0.0%    0.0%    0.0%    0.0%    0.0%    0.0%    0.0%    0.0%    0.0%    0.0%    0.0%    0.0%    0.0%    0.0%    0.0%    0.0%    0.0%    0.0%    0.0%    0.0%    0.0%    0.0%    0.0%    0.0%    0.0%    0.0%    0.0%    0.0%    0.0%    0.0%    0.0%    0.0%    0.0%    0.0%    0.0%    0.0%    0.0%    0.0%    0.0%    0.0%    0.0%    0.0%    0.0%    0.0%    0.0%    0.0%    0.0%    0.0%    0.0%    0.0%    0.0%    0.0%    0.0%    0.0%    0.0%    0.0%    0.0%    0.0%    0.0%    0.0%    0.0%    0.0%    0.0%    0.0%    0.0%    0.0%    0.0%    0.0%    0.0%    0.0%    0.0%    0.0%    0.0%    0.0%    0.0%    0.0%    0.0%    0.0%    0.0%    0.0%    0.0%    0.0%    0.0%    0.0%    0.0%    0.0%    0.0%    0.0%    0.0%    0.0%    0.0%    0.0%    0.0%    0.0%    0.0%    0.0%    0.0%    0.0%    0.0%    0.0%    0.0%    0.0%    0.0%    0.0%    0.0%    0.0%    0.0%    0.0%    0.0%    0.0%    0.0%    0.0%    0.0%    0.0%    0.0%    0.0%    0.0%    0.0%    0.0%    0.0%    0.0%    0.0%    0.0%    0.0%        k__Bacteria;p__Elusimicrobia;c__Elusimicrobia (class); o__Elusimicrobiales       6    0.0%    0.0%    0.0%    0.0%    0.0%    0.0%    0.0%    0.0%    0.0%    0.0%    0.0%    0.0%    0.0%    0.0%    0.0%    0.0%    0.0%    0.0%    0.0%    0.0%    0.0%    0.0%    0.0%    0.0%    0.0%    0.0%    0.0%    0.0%    0.0%    0.0%    0.0%    0.0%    0.0%    0.0%    0.0%    0.0%    0.0%    0.0%    0.0%    0.0%    0.0%    0.0%    0.0%    0.0%    0.0%    0.0%    0.0%    0.0%    0.0%    0.0%    0.0%    0.0%    0.0%    0.0%    0.0%    0.0%    0.0%    0.0%    0.0%    0.0%    0.0%    0.0%    0.0%    0.0%    0.0%    0.0%    0.0%    0.0%    0.0%    0.0%    0.0%    0.0%    0.0%    0.0%    0.0%    0.0%    0.0%    0.0%    0.0%    0.0%    0.0%    0.0%    0.0%    0.0%    0.0%    0.0%    0.0%    0.0%    0.0%    0.0%    0.0%    0.0%    0.0%    0.0%    0.0%    0.0%    0.0%    0.0%    0.0%    0.0%    0.0%    0.0%    0.0%    0.0%    0.0%    0.0%    0.0%    0.0%    0.0%    0.0%    0.0%    0.0%    0.0%    0.0%    0.0%    0.0%    0.0%    0.0%    0.0%    0.0%    0.0%    0.0%    0.0%    0.0%    0.0%    0.0%    0.0%    0.0%    0.0%    0.0%    0.0%    0.0%    0.0%    0.0%    0.0%    0.0%    0.0%    0.0%    0.0%    0.0%    0.0%    0.0%    0.0%    0.0%    0.0%    0.0%    0.0%    0.0%    0.0%    0.0%    0.0%    0.0%    0.0%    0.0%    0.0%    0.0%    0.0%    0.0%    0.0%    0.0%    0.0%    0.0%    0.0%    0.0%    0.0%    0.0%    0.0%    0.0%    0.0%    0.0%    0.0%    0.0%    0.0%    0.0%    0.0%    0.0%    0.0%    0.0%    0.0%    0.0%    0.0%    0.0%    0.0%    0.0%    0.0%    0.0%    0.0%    0.0%    0.0%    0.0%    0.0%    0.0%    0.0%    0.0%    0.0%    0.0%    0.0%    0.0%    0.0%    0.0%    0.0%    0.0%    0.0%    0.0%    0.0%    0.0%    0.0%    0.0%    0.0%    0.0%    0.0%    0.0%    0.0%    0.0%    0.0%    0.0%    0.0%    0.0%    0.0%    0.0%    0.0%    0.0%    0.0%    0.0%    0.0%    0.0%    0.0%    0.0%    0.0%    0.0%    0.0%    0.0%    0.0%    0.0%    0.0%    0.0%    0.0%    0.0%    0.0%    0.0%    0.0%    0.0%    0.0%    0.0%    0.0%    0.0%    0.0%    0.0%    0.0%    0.0%    0.0%    0.0%    0.0%    0.0%    0.0%    0.0%    0.0%    0.0%    0.0%    0.0%    0.0%    0.0%    0.0%    0.0%    0.0%    0.0%    0.0%    0.0%    0.0%    0.0%    0.0%    0.0%    0.0%    0.0%    0.0%    0.0%    0.0%    0.0%    0.0%    0.0%    0.0%    0.0%    0.0%    0.0%    0.0%    0.0%    0.0%    0.0%    0.0%    0.0%    0.0%    0.0%    0.0%    0.0%    0.0%    0.0%    0.0%    0.0%    0.0%    0.0%    0.0%    0.0%    0.0%    0.0%    0.0%    0.0%    0.0%    0.0%    0.0%    0.0%    0.0%    0.0%    0.0%    0.0%    0.0%    0.0%    0.0%    0.0%    0.0%    0.0%    0.0%    0.0%    0.0%    0.0%    0.0%    0.0%    0.0%    0.0%    0.0%    0.0%    0.0%    0.0%    0.0%    0.0%    0.0%    0.0%    0.0%    0.0%    0.0%    0.0%    0.0%    0.0%    0.0%    0.0%    0.0%    0.0%    0.0%    0.0%    0.0%    0.0%    0.0%    0.0%    0.0%    0.0%    0.0%    0.0%    0.0%    0.0%        k__Bacteria;p__Fibrobacteres;c__Fibrobacteres (class); o__Fibrobacterales       1    0.0%    0.0%    0.0%    0.0%    0.0%    0.0%    0.0%    0.0%    0.0%    0.0%    0.0%    0.0%    0.0%    0.0%    0.0%    0.0%    0.0%    0.0%    0.0%    0.0%    0.0%    0.0%    0.0%    0.0%    0.0%    0.0%    0.0%    0.0%    0.0%    0.0%    0.0%    0.0%    0.0%    0.0%    0.0%    0.0%    0.0%    0.0%    0.0%    0.0%    0.0%    0.0%    0.0%    0.0%    0.0%    0.0%    0.0%    0.0%    0.0%    0.0%    0.0%    0.0%    0.0%    0.0%    0.0%    0.0%    0.0%    0.0%    0.0%    0.0%    0.0%    0.0%    0.0%    0.0%    0.0%    0.0%    0.0%    0.0%    0.0%    0.0%    0.0%    0.0%    0.0%    0.0%    0.0%    0.0%    0.0%    0.0%    0.0%    0.0%    0.0%    0.0%    0.0%    0.0%    0.0%    0.0%    0.0%    0.0%    0.0%    0.0%    0.0%    0.0%    0.0%    0.0%    0.0%    0.0%    0.0%    0.0%    0.0%    0.0%    0.0%    0.0%    0.0%    0.0%    0.0%    0.0%    0.0%    0.0%    0.0%    0.0%    0.0%    0.0%    0.0%    0.0%    0.0%    0.0%    0.0%    0.0%    0.0%    0.0%    0.0%    0.0%    0.0%    0.0%    0.0%    0.0%    0.0%    0.0%    0.0%    0.0%    0.0%    0.0%    0.0%    0.0%    0.0%    0.0%    0.0%    0.0%    0.0%    0.0%    0.0%    0.0%    0.0%    0.0%    0.0%    0.0%    0.0%    0.0%    0.0%    0.0%    0.0%    0.0%    0.0%    0.0%    0.0%    0.0%    0.0%    0.0%    0.0%    0.0%    0.0%    0.0%    0.0%    0.0%    0.0%    0.0%    0.0%    0.0%    0.0%    0.0%    0.0%    0.0%    0.0%    0.0%    0.0%    0.0%    0.0%    0.0%    0.0%    0.0%    0.0%    0.0%    0.0%    0.0%    0.0%    0.0%    0.0%    0.0%    0.0%    0.0%    0.0%    0.0%    0.0%    0.0%    0.0%    0.0%    0.0%    0.0%    0.0%    0.0%    0.0%    0.0%    0.0%    0.0%    0.0%    0.0%    0.0%    0.0%    0.0%    0.0%    0.0%    0.0%    0.0%    0.0%    0.0%    0.0%    0.0%    0.0%    0.0%    0.0%    0.0%    0.0%    0.0%    0.0%    0.0%    0.0%    0.0%    0.0%    0.0%    0.0%    0.0%    0.0%    0.0%    0.0%    0.0%    0.0%    0.0%    0.0%    0.0%    0.0%    0.0%    0.0%    0.0%    0.0%    0.0%    0.0%    0.0%    0.0%    0.0%    0.0%    0.0%    0.0%    0.0%    0.0%    0.0%    0.0%    0.0%    0.0%    0.0%    0.0%    0.0%    0.0%    0.0%    0.0%    0.0%    0.0%    0.0%    0.0%    0.0%    0.0%    0.0%    0.0%    0.0%    0.0%    0.0%    0.0%    0.0%    0.0%    0.0%    0.0%    0.0%    0.0%    0.0%    0.0%    0.0%    0.0%    0.0%    0.0%    0.0%    0.0%    0.0%    0.0%    0.0%    0.0%    0.0%    0.0%    0.0%    0.0%    0.0%    0.0%    0.0%    0.0%    0.0%    0.0%    0.0%    0.0%    0.0%    0.0%    0.0%    0.0%    0.0%    0.0%    0.0%    0.0%    0.0%    0.0%    0.0%    0.0%    0.0%    0.0%    0.0%    0.0%    0.0%    0.0%    0.0%    0.0%    0.0%    0.0%    0.0%    0.0%    0.0%    0.0%    0.0%    0.0%    0.0%    0.0%    0.0%    0.0%    0.0%    0.0%    0.0%    0.0%    0.0%    0.0%    0.0%    0.0%    0.0%    0.0%    0.0%    0.0%    0.0%    0.0%    0.0%    0.0%    0.0%    0.0%    0.0%    0.0%        k__Bacteria;p__Firmicutes;c__Bacilli; o__    4872    0.0%    0.0%    0.0%    0.0%    0.0%    0.0%    0.0%    0.0%    0.0%    0.0%    0.1%    0.0%    0.0%    0.0%    0.0%    0.0%    0.0%    0.0%    0.0%    0.0%    0.0%    0.0%    0.0%    0.0%    0.0%    0.0%    0.0%    0.0%    0.2%    0.0%    0.0%    0.0%    0.0%    0.0%    0.0%    0.0%    0.0%    0.0%    0.0%    0.0%    0.0%    0.0%    0.1%    0.0%    0.0%    0.0%    0.0%    0.0%    0.0%    0.0%    0.1%    0.0%    0.0%    0.0%    0.0%    0.0%    0.0%    0.0%    0.0%    0.0%    0.0%    0.0%    0.0%    0.0%    0.0%    0.0%    0.1%    0.0%    0.0%    0.0%    0.0%    0.0%    0.0%    0.0%    0.0%    0.0%    0.0%    0.0%    0.0%    0.0%    0.1%    0.0%    0.0%    0.0%    0.0%    0.0%    0.0%    0.0%    0.0%    0.0%    0.0%    0.0%    0.0%    0.0%    0.0%    0.0%    0.0%    0.0%    0.0%    0.0%    0.1%    0.0%    0.0%    0.0%    0.0%    0.0%    0.0%    0.0%    0.0%    0.0%    0.1%    0.0%    0.0%    0.0%    0.1%    0.1%    0.1%    0.0%    0.0%    0.0%    0.0%    0.0%    0.0%    0.0%    0.0%    0.0%    0.1%    0.0%    0.0%    0.0%    0.0%    0.0%    0.0%    0.0%    0.0%    0.0%    0.0%    0.0%    0.1%    0.0%    0.3%    0.0%    0.0%    0.0%    0.0%    0.0%    0.0%    0.0%    0.0%    0.0%    0.0%    0.0%    0.0%    0.0%    0.0%    0.0%    0.1%    0.0%    0.1%    0.0%    0.0%    0.0%    0.0%    0.0%    0.0%    0.0%    0.0%    0.0%    0.0%    0.0%    0.0%    0.0%    0.0%    0.1%    0.0%    0.1%    0.0%    0.0%    0.0%    0.0%    0.0%    0.0%    0.0%    0.0%    0.0%    0.0%    0.0%    0.1%    0.0%    0.4%    0.1%    0.1%    0.0%    0.0%    0.0%    0.0%    0.0%    0.1%    0.0%    0.0%    0.0%    0.0%    0.0%    0.0%    0.1%    0.0%    0.0%    0.0%    0.0%    0.0%    0.0%    0.0%    0.0%    0.0%    0.0%    0.0%    0.0%    0.0%    0.0%    0.0%    0.0%    0.0%    0.0%    0.0%    0.0%    0.0%    0.0%    0.0%    0.0%    0.0%    0.0%    0.0%    0.1%    0.0%    0.0%    0.0%    0.0%    0.0%    0.0%    0.0%    0.0%    0.0%    0.1%    0.0%    0.1%    0.0%    0.1%    0.1%    0.0%    0.0%    0.0%    0.0%    0.0%    0.0%    0.0%    0.0%    0.0%    0.0%    0.0%    0.0%    0.0%    0.0%    0.0%    0.0%    0.0%    0.0%    0.0%    0.0%    0.0%    0.0%    0.0%    0.0%    0.0%    0.0%    0.0%    0.0%    0.0%    0.0%    0.0%    0.0%    0.0%    0.6%    0.0%    0.0%    0.0%    0.0%    0.0%    0.0%    0.0%    0.0%    0.0%    0.0%    0.0%    0.0%    0.0%    0.0%    0.0%    0.0%    0.0%    0.0%    0.0%    0.0%    0.0%    0.1%    0.0%    0.0%    0.0%    0.0%    0.1%    0.0%    0.0%    0.2%    0.0%    0.0%    0.0%    0.1%    0.0%    0.0%    0.0%    0.0%    0.0%    0.0%    0.0%    0.1%    0.1%    0.1%    0.1%    0.0%    0.0%    0.1%    0.0%    0.0%    0.0%    0.0%    0.0%    0.1%    0.1%    0.0%    0.1%    0.0%    0.0%    0.0%    0.1%    0.0%    0.1%    0.0%    0.0%    0.0%    0.0%    0.0%    0.0%    0.1%    0.0%    0.0%    0.0%    0.0%    0.0%    0.2%        k__Bacteria;p__Firmicutes;c__Bacilli; o__Bacillales   2170235   12.7%    6.0%    8.5%    5.2%    7.7%    1.9%   11.0%    4.0%    6.6%    6.5%    8.9%    8.4%    9.6%    7.5%    8.9%   31.9%   10.7%    8.5%    6.0%    7.5%   13.7%   12.0%   27.7%    9.2%    8.6%    9.1%    7.0%   23.9%   18.5%    7.3%    6.5%    9.0%    9.3%    4.0%   13.1%    3.1%   10.6%    3.1%    6.4%    8.3%    6.4%   15.6%    7.2%    6.2%   15.4%   17.0%   20.9%   12.0%   12.1%    8.3%   22.5%   12.2%   14.8%   18.5%    9.0%   10.8%   14.7%    7.2%   11.1%   13.6%   15.4%   11.8%   15.9%   11.1%   11.6%   11.9%    4.1%   13.3%    7.4%   17.7%    1.2%   18.2%    3.7%   12.6%   22.3%    3.7%   12.7%   23.8%    9.2%   17.2%   14.9%   15.0%   17.8%   18.6%   19.4%   24.2%   18.3%    1.8%    7.7%   25.6%    2.8%    9.2%   22.3%    5.1%    9.9%   21.6%    7.4%    2.8%   25.3%   28.5%    8.5%   24.3%   29.3%   26.0%   26.6%   23.3%    8.5%    1.8%    1.4%   27.9%   35.4%    8.9%   11.9%    4.7%   13.1%   26.1%   15.2%   36.3%    4.4%    4.5%    0.6%   25.7%   30.0%   11.3%    1.6%    7.1%    7.3%    5.4%    9.8%   12.9%   19.6%    5.5%    2.2%   23.1%    9.6%    5.7%    3.3%    6.8%   12.6%    3.0%   17.1%    4.4%    6.5%    4.9%    3.7%   14.4%    9.5%   23.3%   14.8%    1.2%    3.2%    2.4%    7.7%    6.5%    4.6%    5.0%   11.7%    3.2%   10.2%   20.3%    8.4%    7.2%    3.2%    0.9%    8.7%    4.7%   12.0%   12.6%   11.9%   11.5%    8.2%    9.3%    3.3%   14.8%   15.9%   15.9%   25.0%    2.3%    3.9%    9.4%   24.3%   21.2%   37.1%    8.5%   15.9%   19.6%   29.0%   34.1%   22.9%   17.5%   27.9%   18.2%   13.1%    6.0%    6.5%   17.3%    8.3%   20.7%   39.2%   22.2%   15.4%    4.7%    8.9%   15.2%   14.9%    9.3%    5.3%    3.5%   12.0%    9.2%   14.2%    7.0%    3.3%    6.6%    4.4%   17.8%    5.8%   13.7%   10.7%   17.6%   38.4%    8.6%    0.4%    5.9%    7.2%   21.2%    4.7%    5.9%   14.0%   13.4%   18.1%    5.3%    6.1%    5.8%    9.1%    2.1%   16.6%   14.7%   66.7%   42.9%   39.4%   50.0%    9.7%   10.0%   12.0%   59.3%   22.2%   41.6%    7.4%    4.2%    8.1%    3.0%    2.0%    3.0%    2.4%    3.2%    5.0%    3.3%    5.6%    4.3%   10.2%    5.5%    7.2%    4.0%    4.0%    1.8%    8.7%    6.4%    3.9%    5.2%    6.9%    7.6%    4.5%    6.9%    7.8%   10.5%   11.7%   23.4%    9.9%    1.6%   22.1%    3.1%    7.0%   24.2%    8.2%    4.9%    4.7%   15.7%   18.2%   21.2%   32.9%   25.1%   13.8%   13.3%   16.9%   21.3%    9.5%   20.7%   14.1%   33.0%    8.5%   18.7%   21.4%   23.5%   12.9%   11.6%   20.6%   19.4%   12.0%    5.5%   37.3%   38.7%   16.6%    8.7%   29.6%   29.0%    6.1%   20.2%   28.1%   14.2%    9.9%   12.0%    2.0%    2.5%   14.6%   14.0%   14.3%   13.4%   20.3%   19.3%   17.9%   14.9%   33.6%   70.3%   67.5%   19.7%   13.5%    7.9%   10.5%    0.7%    7.6%    4.4%   25.4%   32.4%   34.7%   30.2%   44.5%    8.9%    4.0%   17.3%   15.5%   11.1%   30.0%   35.7%   29.6%    0.2%   23.8%   74.7%        k__Bacteria;p__Firmicutes;c__Bacilli; o__Erysipelotrichales    6056    0.0%    0.0%    0.0%    0.0%    0.1%    0.0%    0.0%    0.0%    0.0%    0.0%    0.0%    0.0%    0.0%    0.0%    0.0%    0.0%    0.1%    0.1%    0.0%    0.0%    0.1%    0.1%    0.0%    0.0%    0.0%    0.0%    0.0%    0.0%    0.1%    0.0%    0.0%    0.0%    0.0%    0.0%    1.3%    0.0%    0.1%    0.0%    0.1%    0.0%    0.0%    0.0%    0.0%    0.0%    0.1%    0.2%    0.0%    0.0%    0.0%    0.0%    0.0%    0.0%    0.0%    0.0%    0.1%    0.0%    0.0%    0.0%    0.0%    0.0%    0.0%    0.0%    0.0%    0.2%    0.1%    0.0%    0.0%    0.1%    0.0%    0.2%    0.0%    0.0%    0.0%    0.1%    0.0%    0.0%    0.0%    0.1%    0.0%    0.0%    0.0%    0.0%    0.0%    0.0%    0.0%    0.0%    0.0%    0.0%    0.0%    0.0%    0.0%    0.0%    0.0%    0.0%    0.0%    0.0%    0.0%    0.0%    0.0%    0.1%    0.0%    0.0%    0.0%    0.0%    0.0%    0.0%    0.2%    0.0%    0.0%    0.0%    0.0%    0.4%    0.0%    0.0%    0.1%    0.0%    0.0%    0.0%    0.0%    0.1%    0.0%    0.0%    0.1%    0.1%    0.0%    0.1%    0.0%    0.0%    0.1%    0.1%    0.0%    0.0%    0.0%    0.0%    0.1%    0.0%    0.0%    0.0%    0.0%    0.0%    0.0%    0.0%    0.1%    0.0%    0.0%    0.0%    0.0%    0.0%    0.0%    0.0%    0.0%    0.0%    0.1%    0.0%    0.0%    0.0%    0.1%    0.0%    0.0%    0.0%    0.0%    0.0%    0.1%    0.0%    0.0%    0.0%    0.0%    0.0%    0.0%    0.0%    0.0%    0.1%    0.2%    0.1%    0.0%    0.0%    0.0%    0.0%    0.1%    0.0%    0.0%    0.0%    0.1%    0.0%    0.0%    0.0%    0.0%    0.0%    0.0%    0.0%    0.0%    0.0%    0.0%    0.0%    0.0%    0.0%    0.0%    0.0%    0.0%    0.0%    0.0%    0.1%    0.0%    0.1%    0.1%    0.0%    0.0%    0.0%    0.1%    0.1%    0.0%    0.0%    0.0%    0.0%    0.0%    0.0%    0.0%    0.0%    0.0%    0.0%    0.2%    0.0%    0.0%    0.1%    0.0%    0.1%    0.0%    0.0%    0.1%    0.0%    0.0%    0.0%    0.0%    0.0%    0.1%    0.0%    0.1%    0.0%    0.0%    0.0%    0.0%    0.0%    0.0%    0.0%    0.0%    0.0%    0.0%    0.0%    0.0%    0.0%    0.1%    0.0%    0.0%    0.0%    0.0%    0.0%    0.0%    0.0%    0.0%    0.0%    0.0%    0.0%    0.0%    0.0%    0.0%    0.0%    0.0%    0.0%    0.0%    0.0%    0.0%    0.0%    0.0%    0.0%    0.0%    0.0%    0.0%    0.0%    0.0%    0.0%    0.0%    0.0%    0.0%    0.0%    0.0%    0.0%    0.0%    0.1%    0.0%    0.0%    0.0%    0.0%    0.1%    0.0%    0.0%    0.0%    0.3%    0.0%    0.1%    0.0%    0.0%    0.0%    0.0%    0.1%    0.0%    0.0%    0.0%    0.0%    0.0%    0.1%    0.0%    0.0%    0.0%    0.0%    0.0%    0.5%    0.0%    0.1%    0.0%    0.0%    0.0%    0.0%    0.0%    0.0%    0.0%    0.0%    0.0%    0.0%    0.1%    0.0%    0.0%    0.0%    0.0%    0.0%    0.0%    0.0%    0.0%    0.0%    0.0%    0.0%    0.0%    0.0%    0.0%    0.0%    0.0%    0.0%    0.0%    0.0%    0.0%    0.0%    0.0%    0.0%    0.0%    0.2%    0.0%    0.0%    0.0%    0.0%        k__Bacteria;p__Firmicutes;c__Bacilli; o__Lactobacillales   2195502   12.9%   29.1%   21.7%   29.5%   13.5%    1.4%   27.3%    5.6%   20.3%   35.3%   39.3%    3.0%   12.0%   17.1%   28.4%   13.2%   17.4%    3.5%   14.5%   10.1%   10.1%   10.3%   20.5%    2.0%   15.6%   32.7%    4.1%   18.4%   12.7%    9.3%    5.6%   27.8%    5.2%   13.4%    7.8%   15.4%   15.4%    7.9%   18.0%    7.8%   11.1%   12.6%   26.4%   20.9%   14.4%   14.4%    9.2%   10.8%   20.8%   17.8%   14.4%    3.7%    6.5%   18.6%   10.6%    6.8%    5.5%    1.6%   24.1%    6.8%    6.2%   37.1%   11.6%   28.0%    2.4%   29.3%    2.1%   11.6%    2.9%   13.3%    2.0%   11.1%    2.1%    6.6%    9.6%    2.8%    5.2%   10.9%   30.8%   23.2%   24.9%    5.1%   27.4%   35.3%    5.3%   10.6%   15.4%    2.9%   22.2%   13.9%    1.2%   35.3%   23.7%    6.8%    4.6%    5.8%    3.4%    2.5%   10.0%   11.0%    6.2%    7.8%    4.6%    9.2%    8.2%   24.0%    7.2%    2.5%    0.5%   11.7%   17.1%   10.3%   11.4%    4.6%   19.6%   22.6%   10.2%   12.9%   27.4%    4.3%    1.3%   21.5%    5.4%   20.1%    4.8%    8.4%   14.8%    2.6%    5.7%    7.9%   13.0%   10.0%    1.1%   34.2%    8.5%    4.0%   16.4%    9.3%    0.8%    8.3%    1.3%   16.1%    4.2%    1.0%    3.7%    7.3%   20.5%   14.4%   11.6%    1.9%    1.9%    0.9%   13.1%   10.1%   51.2%    3.4%    9.5%    9.6%   12.4%   12.3%    3.7%   12.9%    6.0%    3.0%    6.1%    4.9%   11.6%   12.9%   12.0%   22.0%   10.9%   14.7%    9.1%   19.1%   24.0%   22.1%   19.7%    6.5%    1.3%   21.5%   18.2%   19.6%    4.7%    1.6%    7.1%   18.0%    8.4%   16.6%   16.3%   27.5%    8.6%   12.9%   20.9%   21.4%   39.6%    8.2%   18.9%   15.8%    6.4%   11.6%    9.1%   14.4%    4.6%   10.2%   10.4%   22.3%    2.6%    7.1%    6.9%   12.8%   19.8%   14.5%   11.1%   11.6%   17.9%   11.1%    2.8%    8.1%    6.6%   13.2%    6.9%    7.3%    0.7%    2.8%    4.3%   10.7%   44.8%   33.5%    8.1%   13.1%   19.3%    4.6%    3.3%   15.3%   11.3%    4.8%    5.4%    6.4%    3.3%    4.6%    7.6%    4.4%   32.3%   19.2%   30.6%    4.7%   11.6%    2.5%   20.9%    9.9%    6.1%   10.4%    2.7%   11.9%    1.2%    5.0%   11.5%    3.6%    9.2%   33.7%    9.0%   14.2%   20.5%    2.5%   11.4%    1.0%   13.1%   34.6%   38.5%   13.7%   10.7%   14.5%    4.7%   20.0%   11.3%    5.9%   11.1%   20.6%   18.3%    5.7%    8.4%   76.2%   20.1%   14.6%    9.5%   36.3%   11.4%    5.2%   11.9%   18.3%    5.9%    3.8%   10.7%   15.0%   41.6%   42.5%   24.3%   17.8%   25.6%   15.2%   13.7%   24.9%   32.1%   16.0%   33.9%   23.7%   26.0%   26.9%    5.5%   28.7%    3.1%    6.5%    3.2%   31.4%   18.1%    4.9%    2.3%   21.0%   21.2%    3.7%   17.6%   26.1%    5.3%   13.3%   14.2%   14.7%   16.5%    8.2%   23.3%   23.1%   39.7%   39.3%   24.7%    4.1%    4.1%   29.2%   28.1%   33.7%   36.1%    8.1%   39.0%   15.8%   23.5%   10.4%    7.7%   16.7%    2.5%   27.4%    2.6%    4.3%   11.1%   26.8%   20.5%    7.2%   11.9%    0.4%    6.5%    2.1%        k__Bacteria;p__Firmicutes;c__Clostridia; o__       1    0.0%    0.0%    0.0%    0.0%    0.0%    0.0%    0.0%    0.0%    0.0%    0.0%    0.0%    0.0%    0.0%    0.0%    0.0%    0.0%    0.0%    0.0%    0.0%    0.0%    0.0%    0.0%    0.0%    0.0%    0.0%    0.0%    0.0%    0.0%    0.0%    0.0%    0.0%    0.0%    0.0%    0.0%    0.0%    0.0%    0.0%    0.0%    0.0%    0.0%    0.0%    0.0%    0.0%    0.0%    0.0%    0.0%    0.0%    0.0%    0.0%    0.0%    0.0%    0.0%    0.0%    0.0%    0.0%    0.0%    0.0%    0.0%    0.0%    0.0%    0.0%    0.0%    0.0%    0.0%    0.0%    0.0%    0.0%    0.0%    0.0%    0.0%    0.0%    0.0%    0.0%    0.0%    0.0%    0.0%    0.0%    0.0%    0.0%    0.0%    0.0%    0.0%    0.0%    0.0%    0.0%    0.0%    0.0%    0.0%    0.0%    0.0%    0.0%    0.0%    0.0%    0.0%    0.0%    0.0%    0.0%    0.0%    0.0%    0.0%    0.0%    0.0%    0.0%    0.0%    0.0%    0.0%    0.0%    0.0%    0.0%    0.0%    0.0%    0.0%    0.0%    0.0%    0.0%    0.0%    0.0%    0.0%    0.0%    0.0%    0.0%    0.0%    0.0%    0.0%    0.0%    0.0%    0.0%    0.0%    0.0%    0.0%    0.0%    0.0%    0.0%    0.0%    0.0%    0.0%    0.0%    0.0%    0.0%    0.0%    0.0%    0.0%    0.0%    0.0%    0.0%    0.0%    0.0%    0.0%    0.0%    0.0%    0.0%    0.0%    0.0%    0.0%    0.0%    0.0%    0.0%    0.0%    0.0%    0.0%    0.0%    0.0%    0.0%    0.0%    0.0%    0.0%    0.0%    0.0%    0.0%    0.0%    0.0%    0.0%    0.0%    0.0%    0.0%    0.0%    0.0%    0.0%    0.0%    0.0%    0.0%    0.0%    0.0%    0.0%    0.0%    0.0%    0.0%    0.0%    0.0%    0.0%    0.0%    0.0%    0.0%    0.0%    0.0%    0.0%    0.0%    0.0%    0.0%    0.0%    0.0%    0.0%    0.0%    0.0%    0.0%    0.0%    0.0%    0.0%    0.0%    0.0%    0.0%    0.0%    0.0%    0.0%    0.0%    0.0%    0.0%    0.0%    0.0%    0.0%    0.0%    0.0%    0.0%    0.0%    0.0%    0.0%    0.0%    0.0%    0.0%    0.0%    0.0%    0.0%    0.0%    0.0%    0.0%    0.0%    0.0%    0.0%    0.0%    0.0%    0.0%    0.0%    0.0%    0.0%    0.0%    0.0%    0.0%    0.0%    0.0%    0.0%    0.0%    0.0%    0.0%    0.0%    0.0%    0.0%    0.0%    0.0%    0.0%    0.0%    0.0%    0.0%    0.0%    0.0%    0.0%    0.0%    0.0%    0.0%    0.0%    0.0%    0.0%    0.0%    0.0%    0.0%    0.0%    0.0%    0.0%    0.0%    0.0%    0.0%    0.0%    0.0%    0.0%    0.0%    0.0%    0.0%    0.0%    0.0%    0.0%    0.0%    0.0%    0.0%    0.0%    0.0%    0.0%    0.0%    0.0%    0.0%    0.0%    0.0%    0.0%    0.0%    0.0%    0.0%    0.0%    0.0%    0.0%    0.0%    0.0%    0.0%    0.0%    0.0%    0.0%    0.0%    0.0%    0.0%    0.0%    0.0%    0.0%    0.0%    0.0%    0.0%    0.0%    0.0%    0.0%    0.0%    0.0%    0.0%    0.0%    0.0%    0.0%    0.0%    0.0%    0.0%    0.0%    0.0%    0.0%    0.0%    0.0%    0.0%    0.0%    0.0%    0.0%    0.0%    0.0%    0.0%    0.0%    0.0%    0.0%    0.0%    0.0%    0.0%    0.0%    0.0%    0.0%    0.0%    0.0%    0.0%        k__Bacteria;p__Firmicutes;c__Clostridia; o__1B07       1    0.0%    0.0%    0.0%    0.0%    0.0%    0.0%    0.0%    0.0%    0.0%    0.0%    0.0%    0.0%    0.0%    0.0%    0.0%    0.0%    0.0%    0.0%    0.0%    0.0%    0.0%    0.0%    0.0%    0.0%    0.0%    0.0%    0.0%    0.0%    0.0%    0.0%    0.0%    0.0%    0.0%    0.0%    0.0%    0.0%    0.0%    0.0%    0.0%    0.0%    0.0%    0.0%    0.0%    0.0%    0.0%    0.0%    0.0%    0.0%    0.0%    0.0%    0.0%    0.0%    0.0%    0.0%    0.0%    0.0%    0.0%    0.0%    0.0%    0.0%    0.0%    0.0%    0.0%    0.0%    0.0%    0.0%    0.0%    0.0%    0.0%    0.0%    0.0%    0.0%    0.0%    0.0%    0.0%    0.0%    0.0%    0.0%    0.0%    0.0%    0.0%    0.0%    0.0%    0.0%    0.0%    0.0%    0.0%    0.0%    0.0%    0.0%    0.0%    0.0%    0.0%    0.0%    0.0%    0.0%    0.0%    0.0%    0.0%    0.0%    0.0%    0.0%    0.0%    0.0%    0.0%    0.0%    0.0%    0.0%    0.0%    0.0%    0.0%    0.0%    0.0%    0.0%    0.0%    0.0%    0.0%    0.0%    0.0%    0.0%    0.0%    0.0%    0.0%    0.0%    0.0%    0.0%    0.0%    0.0%    0.0%    0.0%    0.0%    0.0%    0.0%    0.0%    0.0%    0.0%    0.0%    0.0%    0.0%    0.0%    0.0%    0.0%    0.0%    0.0%    0.0%    0.0%    0.0%    0.0%    0.0%    0.0%    0.0%    0.0%    0.0%    0.0%    0.0%    0.0%    0.0%    0.0%    0.0%    0.0%    0.0%    0.0%    0.0%    0.0%    0.0%    0.0%    0.0%    0.0%    0.0%    0.0%    0.0%    0.0%    0.0%    0.0%    0.0%    0.0%    0.0%    0.0%    0.0%    0.0%    0.0%    0.0%    0.0%    0.0%    0.0%    0.0%    0.0%    0.0%    0.0%    0.0%    0.0%    0.0%    0.0%    0.0%    0.0%    0.0%    0.0%    0.0%    0.0%    0.0%    0.0%    0.0%    0.0%    0.0%    0.0%    0.0%    0.0%    0.0%    0.0%    0.0%    0.0%    0.0%    0.0%    0.0%    0.0%    0.0%    0.0%    0.0%    0.0%    0.0%    0.0%    0.0%    0.0%    0.0%    0.0%    0.0%    0.0%    0.0%    0.0%    0.0%    0.0%    0.0%    0.0%    0.0%    0.0%    0.0%    0.0%    0.0%    0.0%    0.0%    0.0%    0.0%    0.0%    0.0%    0.0%    0.0%    0.0%    0.0%    0.0%    0.0%    0.0%    0.0%    0.0%    0.0%    0.0%    0.0%    0.0%    0.0%    0.0%    0.0%    0.0%    0.0%    0.0%    0.0%    0.0%    0.0%    0.0%    0.0%    0.0%    0.0%    0.0%    0.0%    0.0%    0.0%    0.0%    0.0%    0.0%    0.0%    0.0%    0.0%    0.0%    0.0%    0.0%    0.0%    0.0%    0.0%    0.0%    0.0%    0.0%    0.0%    0.0%    0.0%    0.0%    0.0%    0.0%    0.0%    0.0%    0.0%    0.0%    0.0%    0.0%    0.0%    0.0%    0.0%    0.0%    0.0%    0.0%    0.0%    0.0%    0.0%    0.0%    0.0%    0.0%    0.0%    0.0%    0.0%    0.0%    0.0%    0.0%    0.0%    0.0%    0.0%    0.0%    0.0%    0.0%    0.0%    0.0%    0.0%    0.0%    0.0%    0.0%    0.0%    0.0%    0.0%    0.0%    0.0%    0.0%    0.0%    0.0%    0.0%    0.0%    0.0%    0.0%    0.0%    0.0%    0.0%    0.0%    0.0%    0.0%    0.0%    0.0%    0.0%    0.0%    0.0%    0.0%    0.0%    0.0%    0.0%        k__Bacteria;p__Firmicutes;c__Clostridia; o__BSA2B-08       0    0.0%    0.0%    0.0%    0.0%    0.0%    0.0%    0.0%    0.0%    0.0%    0.0%    0.0%    0.0%    0.0%    0.0%    0.0%    0.0%    0.0%    0.0%    0.0%    0.0%    0.0%    0.0%    0.0%    0.0%    0.0%    0.0%    0.0%    0.0%    0.0%    0.0%    0.0%    0.0%    0.0%    0.0%    0.0%    0.0%    0.0%    0.0%    0.0%    0.0%    0.0%    0.0%    0.0%    0.0%    0.0%    0.0%    0.0%    0.0%    0.0%    0.0%    0.0%    0.0%    0.0%    0.0%    0.0%    0.0%    0.0%    0.0%    0.0%    0.0%    0.0%    0.0%    0.0%    0.0%    0.0%    0.0%    0.0%    0.0%    0.0%    0.0%    0.0%    0.0%    0.0%    0.0%    0.0%    0.0%    0.0%    0.0%    0.0%    0.0%    0.0%    0.0%    0.0%    0.0%    0.0%    0.0%    0.0%    0.0%    0.0%    0.0%    0.0%    0.0%    0.0%    0.0%    0.0%    0.0%    0.0%    0.0%    0.0%    0.0%    0.0%    0.0%    0.0%    0.0%    0.0%    0.0%    0.0%    0.0%    0.0%    0.0%    0.0%    0.0%    0.0%    0.0%    0.0%    0.0%    0.0%    0.0%    0.0%    0.0%    0.0%    0.0%    0.0%    0.0%    0.0%    0.0%    0.0%    0.0%    0.0%    0.0%    0.0%    0.0%    0.0%    0.0%    0.0%    0.0%    0.0%    0.0%    0.0%    0.0%    0.0%    0.0%    0.0%    0.0%    0.0%    0.0%    0.0%    0.0%    0.0%    0.0%    0.0%    0.0%    0.0%    0.0%    0.0%    0.0%    0.0%    0.0%    0.0%    0.0%    0.0%    0.0%    0.0%    0.0%    0.0%    0.0%    0.0%    0.0%    0.0%    0.0%    0.0%    0.0%    0.0%    0.0%    0.0%    0.0%    0.0%    0.0%    0.0%    0.0%    0.0%    0.0%    0.0%    0.0%    0.0%    0.0%    0.0%    0.0%    0.0%    0.0%    0.0%    0.0%    0.0%    0.0%    0.0%    0.0%    0.0%    0.0%    0.0%    0.0%    0.0%    0.0%    0.0%    0.0%    0.0%    0.0%    0.0%    0.0%    0.0%    0.0%    0.0%    0.0%    0.0%    0.0%    0.0%    0.0%    0.0%    0.0%    0.0%    0.0%    0.0%    0.0%    0.0%    0.0%    0.0%    0.0%    0.0%    0.0%    0.0%    0.0%    0.0%    0.0%    0.0%    0.0%    0.0%    0.0%    0.0%    0.0%    0.0%    0.0%    0.0%    0.0%    0.0%    0.0%    0.0%    0.0%    0.0%    0.0%    0.0%    0.0%    0.0%    0.0%    0.0%    0.0%    0.0%    0.0%    0.0%    0.0%    0.0%    0.0%    0.0%    0.0%    0.0%    0.0%    0.0%    0.0%    0.0%    0.0%    0.0%    0.0%    0.0%    0.0%    0.0%    0.0%    0.0%    0.0%    0.0%    0.0%    0.0%    0.0%    0.0%    0.0%    0.0%    0.0%    0.0%    0.0%    0.0%    0.0%    0.0%    0.0%    0.0%    0.0%    0.0%    0.0%    0.0%    0.0%    0.0%    0.0%    0.0%    0.0%    0.0%    0.0%    0.0%    0.0%    0.0%    0.0%    0.0%    0.0%    0.0%    0.0%    0.0%    0.0%    0.0%    0.0%    0.0%    0.0%    0.0%    0.0%    0.0%    0.0%    0.0%    0.0%    0.0%    0.0%    0.0%    0.0%    0.0%    0.0%    0.0%    0.0%    0.0%    0.0%    0.0%    0.0%    0.0%    0.0%    0.0%    0.0%    0.0%    0.0%    0.0%    0.0%    0.0%    0.0%    0.0%    0.0%    0.0%    0.0%    0.0%    0.0%    0.0%    0.0%    0.0%    0.0%    0.0%    0.0%    0.0%    0.0%        k__Bacteria;p__Firmicutes;c__Clostridia; o__Clostridiales   1307443    7.7%    6.4%    5.2%    6.9%    5.2%    0.2%    9.3%    3.2%   32.2%    4.5%    5.1%    5.0%    3.1%   10.2%    8.9%    4.3%   16.6%    3.5%    3.3%   18.3%    7.3%    8.0%   10.9%   28.7%   15.2%   16.0%    6.9%    7.4%    7.0%   17.2%   34.2%    7.7%    3.1%    8.3%   14.2%   19.9%    8.1%   44.2%    6.7%    2.7%    6.1%    5.9%    6.8%    6.4%    8.3%    9.8%    6.2%   11.6%    7.4%    9.0%    4.9%    1.8%    5.3%    3.9%    5.8%   18.6%    6.7%    1.5%    8.1%    5.0%    4.0%    6.9%    6.3%    4.9%   15.8%    7.1%    0.7%    7.3%    2.0%    5.2%    2.8%    5.6%    1.3%   10.8%   14.0%    0.6%    2.3%    5.5%   21.4%    8.4%    9.3%    4.4%    7.4%    6.7%   14.4%   16.1%    8.6%    0.3%    3.3%    8.6%    0.4%    4.2%    6.7%    1.6%   10.2%   11.2%    2.8%    0.3%   10.2%    9.1%    3.5%    4.7%    5.6%   12.7%   16.2%    4.6%   11.4%    0.8%    0.1%    9.5%    7.1%   18.2%    3.7%    1.6%   11.9%    5.7%    9.1%    9.2%    4.4%    4.2%    0.1%    7.9%    9.4%    4.6%    0.9%    5.7%    8.0%    1.1%    3.5%    6.6%    8.7%    2.1%    0.7%    5.3%    8.8%    1.6%    1.8%    5.1%    0.5%    1.3%    0.1%    6.5%    3.1%    0.8%    1.5%    9.3%    8.4%    6.5%   16.7%    0.5%    2.1%    0.7%    4.2%    5.3%    3.9%    0.9%    5.3%    3.2%    1.0%    6.8%    2.0%    5.1%    1.9%    0.5%    6.6%    2.8%   10.0%   18.5%   10.3%    7.8%    5.7%   10.6%    1.0%    6.7%    5.9%   12.2%    7.7%    1.3%    1.6%    5.1%   13.4%   12.9%    3.5%    1.2%    9.2%   13.9%   18.2%    9.9%   12.6%    5.1%   14.1%   13.9%   11.3%   16.8%    3.5%    4.7%   15.8%   14.7%    7.8%    8.3%    9.4%   32.2%    7.9%    9.8%    7.8%    4.9%    1.9%    5.6%    5.7%   18.2%    7.8%    7.0%    1.3%    6.4%    2.4%   19.4%    9.1%    5.9%   17.8%    9.3%   12.9%    4.6%    0.1%    2.4%    5.6%   10.2%    6.1%    6.1%    4.5%    9.9%    8.5%    3.6%    1.9%    5.9%    4.7%    6.0%    6.0%    3.0%    1.1%    6.2%    8.6%    4.9%    8.8%    6.5%    5.6%   11.8%    4.1%    4.5%    5.2%   26.4%    4.4%    4.9%    1.6%    5.1%    0.9%    6.3%    5.1%    2.6%    3.8%    6.2%    5.2%    4.3%    3.6%    2.4%   11.0%    0.5%    5.0%    5.9%    1.9%    8.6%   14.0%    7.9%    3.0%   10.8%    3.9%    6.5%   11.5%    7.3%    6.9%   55.8%    2.4%    0.6%    6.8%   10.6%    7.9%   17.5%    6.8%    5.8%   20.3%    2.7%    7.1%   12.2%   10.3%    6.7%    3.7%    5.1%    8.0%    7.6%    7.5%    5.8%   21.7%    6.2%    9.0%    6.3%    5.6%    5.5%    8.3%    6.4%    3.3%    9.9%    6.0%    8.3%    3.0%    8.7%    5.7%    9.1%    1.6%    9.1%   10.7%    5.7%    9.1%    6.0%   40.8%    1.9%    5.5%    8.6%   10.1%   12.6%    6.8%    6.6%    5.7%    5.4%    8.2%    2.7%    3.9%    6.3%    7.3%   16.0%    8.0%    0.3%    3.6%    1.4%    7.3%    8.6%    4.6%   18.0%    4.1%   20.1%    1.3%   11.7%    4.2%    8.0%    7.4%    8.4%    5.2%    0.3%    7.6%    7.4%        k__Bacteria;p__Firmicutes;c__Clostridia; o__Desulfitobacterales       0    0.0%    0.0%    0.0%    0.0%    0.0%    0.0%    0.0%    0.0%    0.0%    0.0%    0.0%    0.0%    0.0%    0.0%    0.0%    0.0%    0.0%    0.0%    0.0%    0.0%    0.0%    0.0%    0.0%    0.0%    0.0%    0.0%    0.0%    0.0%    0.0%    0.0%    0.0%    0.0%    0.0%    0.0%    0.0%    0.0%    0.0%    0.0%    0.0%    0.0%    0.0%    0.0%    0.0%    0.0%    0.0%    0.0%    0.0%    0.0%    0.0%    0.0%    0.0%    0.0%    0.0%    0.0%    0.0%    0.0%    0.0%    0.0%    0.0%    0.0%    0.0%    0.0%    0.0%    0.0%    0.0%    0.0%    0.0%    0.0%    0.0%    0.0%    0.0%    0.0%    0.0%    0.0%    0.0%    0.0%    0.0%    0.0%    0.0%    0.0%    0.0%    0.0%    0.0%    0.0%    0.0%    0.0%    0.0%    0.0%    0.0%    0.0%    0.0%    0.0%    0.0%    0.0%    0.0%    0.0%    0.0%    0.0%    0.0%    0.0%    0.0%    0.0%    0.0%    0.0%    0.0%    0.0%    0.0%    0.0%    0.0%    0.0%    0.0%    0.0%    0.0%    0.0%    0.0%    0.0%    0.0%    0.0%    0.0%    0.0%    0.0%    0.0%    0.0%    0.0%    0.0%    0.0%    0.0%    0.0%    0.0%    0.0%    0.0%    0.0%    0.0%    0.0%    0.0%    0.0%    0.0%    0.0%    0.0%    0.0%    0.0%    0.0%    0.0%    0.0%    0.0%    0.0%    0.0%    0.0%    0.0%    0.0%    0.0%    0.0%    0.0%    0.0%    0.0%    0.0%    0.0%    0.0%    0.0%    0.0%    0.0%    0.0%    0.0%    0.0%    0.0%    0.0%    0.0%    0.0%    0.0%    0.0%    0.0%    0.0%    0.0%    0.0%    0.0%    0.0%    0.0%    0.0%    0.0%    0.0%    0.0%    0.0%    0.0%    0.0%    0.0%    0.0%    0.0%    0.0%    0.0%    0.0%    0.0%    0.0%    0.0%    0.0%    0.0%    0.0%    0.0%    0.0%    0.0%    0.0%    0.0%    0.0%    0.0%    0.0%    0.0%    0.0%    0.0%    0.0%    0.0%    0.0%    0.0%    0.0%    0.0%    0.0%    0.0%    0.0%    0.0%    0.0%    0.0%    0.0%    0.0%    0.0%    0.0%    0.0%    0.0%    0.0%    0.0%    0.0%    0.0%    0.0%    0.0%    0.0%    0.0%    0.0%    0.0%    0.0%    0.0%    0.0%    0.0%    0.0%    0.0%    0.0%    0.0%    0.0%    0.0%    0.0%    0.0%    0.0%    0.0%    0.0%    0.0%    0.0%    0.0%    0.0%    0.0%    0.0%    0.0%    0.0%    0.0%    0.0%    0.0%    0.0%    0.0%    0.0%    0.0%    0.0%    0.0%    0.0%    0.0%    0.0%    0.0%    0.0%    0.0%    0.0%    0.0%    0.0%    0.0%    0.0%    0.0%    0.0%    0.0%    0.0%    0.0%    0.0%    0.0%    0.0%    0.0%    0.0%    0.0%    0.0%    0.0%    0.0%    0.0%    0.0%    0.0%    0.0%    0.0%    0.0%    0.0%    0.0%    0.0%    0.0%    0.0%    0.0%    0.0%    0.0%    0.0%    0.0%    0.0%    0.0%    0.0%    0.0%    0.0%    0.0%    0.0%    0.0%    0.0%    0.0%    0.0%    0.0%    0.0%    0.0%    0.0%    0.0%    0.0%    0.0%    0.0%    0.0%    0.0%    0.0%    0.0%    0.0%    0.0%    0.0%    0.0%    0.0%    0.0%    0.0%    0.0%    0.0%    0.0%    0.0%    0.0%    0.0%    0.0%    0.0%    0.0%    0.0%    0.0%    0.0%    0.0%    0.0%    0.0%    0.0%    0.0%    0.0%    0.0%    0.0%        k__Bacteria;p__Firmicutes;c__Clostridia; o__Halanaerobiales     184    0.0%    0.0%    0.0%    0.0%    0.0%    0.0%    0.0%    0.0%    0.0%    0.0%    0.0%    0.0%    0.0%    0.0%    0.0%    0.0%    0.0%    0.0%    0.1%    0.0%    0.0%    0.0%    0.0%    0.0%    0.0%    0.0%    0.0%    0.0%    0.0%    0.0%    0.0%    0.0%    0.0%    0.0%    0.0%    0.0%    0.0%    0.0%    0.0%    0.0%    0.0%    0.0%    0.0%    0.0%    0.0%    0.0%    0.0%    0.0%    0.0%    0.0%    0.0%    0.0%    0.0%    0.0%    0.0%    0.0%    0.0%    0.0%    0.0%    0.0%    0.0%    0.0%    0.0%    0.0%    0.0%    0.0%    0.0%    0.0%    0.0%    0.0%    0.0%    0.0%    0.0%    0.0%    0.0%    0.0%    0.0%    0.0%    0.0%    0.0%    0.0%    0.0%    0.0%    0.0%    0.0%    0.0%    0.0%    0.0%    0.0%    0.0%    0.0%    0.0%    0.0%    0.0%    0.0%    0.0%    0.0%    0.0%    0.0%    0.0%    0.0%    0.0%    0.0%    0.0%    0.0%    0.0%    0.0%    0.0%    0.0%    0.0%    0.0%    0.0%    0.0%    0.0%    0.0%    0.0%    0.0%    0.0%    0.0%    0.0%    0.0%    0.0%    0.0%    0.0%    0.0%    0.0%    0.0%    0.0%    0.0%    0.0%    0.0%    0.0%    0.0%    0.0%    0.0%    0.0%    0.0%    0.0%    0.0%    0.0%    0.0%    0.0%    0.0%    0.0%    0.0%    0.0%    0.0%    0.0%    0.0%    0.0%    0.0%    0.0%    0.0%    0.0%    0.0%    0.0%    0.0%    0.0%    0.0%    0.0%    0.0%    0.0%    0.0%    0.0%    0.0%    0.0%    0.0%    0.0%    0.0%    0.0%    0.0%    0.0%    0.0%    0.0%    0.0%    0.0%    0.0%    0.0%    0.0%    0.0%    0.0%    0.0%    0.0%    0.0%    0.0%    0.0%    0.0%    0.0%    0.0%    0.0%    0.0%    0.0%    0.0%    0.0%    0.0%    0.0%    0.0%    0.0%    0.0%    0.0%    0.0%    0.0%    0.0%    0.0%    0.0%    0.0%    0.0%    0.0%    0.0%    0.0%    0.0%    0.0%    0.0%    0.0%    0.0%    0.0%    0.0%    0.0%    0.0%    0.0%    0.0%    0.0%    0.0%    0.0%    0.0%    0.0%    0.0%    0.0%    0.0%    0.0%    0.0%    0.0%    0.0%    0.0%    0.0%    0.0%    0.0%    0.0%    0.0%    0.0%    0.0%    0.0%    0.0%    0.0%    0.0%    0.0%    0.0%    0.0%    0.0%    0.0%    0.0%    0.0%    0.0%    0.0%    0.0%    0.0%    0.0%    0.0%    0.0%    0.0%    0.0%    0.0%    0.0%    0.0%    0.0%    0.0%    0.0%    0.0%    0.0%    0.0%    0.0%    0.0%    0.0%    0.0%    0.0%    0.0%    0.0%    0.0%    0.0%    0.0%    0.0%    0.0%    0.0%    0.0%    0.0%    0.0%    0.0%    0.0%    0.0%    0.0%    0.0%    0.0%    0.0%    0.0%    0.0%    0.0%    0.0%    0.0%    0.0%    0.0%    0.0%    0.0%    0.0%    0.0%    0.0%    0.0%    0.0%    0.0%    0.0%    0.0%    0.0%    0.0%    0.0%    0.0%    0.0%    0.0%    0.0%    0.0%    0.0%    0.0%    0.0%    0.0%    0.0%    0.0%    0.0%    0.0%    0.0%    0.0%    0.0%    0.0%    0.0%    0.0%    0.0%    0.0%    0.0%    0.0%    0.0%    0.0%    0.0%    0.0%    0.0%    0.0%    0.0%    0.0%    0.0%    0.0%    0.0%    0.0%    0.0%    0.0%    0.0%    0.0%    0.0%    0.0%    0.0%    0.0%    0.0%    0.0%        k__Bacteria;p__Firmicutes;c__Clostridia; o__MBA08      39    0.0%    0.0%    0.0%    0.0%    0.0%    0.0%    0.0%    0.0%    0.0%    0.0%    0.0%    0.0%    0.0%    0.0%    0.0%    0.0%    0.0%    0.0%    0.0%    0.0%    0.0%    0.0%    0.0%    0.0%    0.0%    0.0%    0.0%    0.0%    0.0%    0.0%    0.0%    0.0%    0.0%    0.0%    0.0%    0.0%    0.0%    0.0%    0.0%    0.0%    0.0%    0.0%    0.0%    0.0%    0.0%    0.0%    0.0%    0.0%    0.0%    0.0%    0.0%    0.0%    0.0%    0.0%    0.0%    0.0%    0.0%    0.0%    0.0%    0.0%    0.0%    0.0%    0.0%    0.0%    0.0%    0.0%    0.0%    0.0%    0.0%    0.0%    0.0%    0.0%    0.0%    0.0%    0.0%    0.0%    0.0%    0.0%    0.0%    0.0%    0.0%    0.0%    0.0%    0.0%    0.0%    0.0%    0.0%    0.0%    0.0%    0.0%    0.0%    0.0%    0.0%    0.0%    0.0%    0.0%    0.0%    0.0%    0.0%    0.0%    0.0%    0.0%    0.0%    0.0%    0.0%    0.0%    0.0%    0.0%    0.0%    0.0%    0.0%    0.0%    0.0%    0.0%    0.0%    0.0%    0.0%    0.0%    0.0%    0.0%    0.0%    0.0%    0.0%    0.0%    0.0%    0.0%    0.0%    0.0%    0.0%    0.0%    0.0%    0.0%    0.0%    0.0%    0.0%    0.0%    0.0%    0.0%    0.0%    0.0%    0.0%    0.0%    0.0%    0.0%    0.0%    0.0%    0.0%    0.0%    0.0%    0.0%    0.0%    0.0%    0.0%    0.0%    0.0%    0.0%    0.0%    0.0%    0.0%    0.0%    0.0%    0.0%    0.0%    0.0%    0.0%    0.0%    0.0%    0.0%    0.0%    0.0%    0.0%    0.0%    0.0%    0.0%    0.0%    0.0%    0.0%    0.0%    0.0%    0.0%    0.0%    0.0%    0.0%    0.0%    0.0%    0.0%    0.0%    0.0%    0.0%    0.0%    0.0%    0.0%    0.0%    0.0%    0.0%    0.0%    0.0%    0.0%    0.0%    0.0%    0.0%    0.0%    0.0%    0.0%    0.0%    0.0%    0.0%    0.0%    0.0%    0.0%    0.0%    0.0%    0.0%    0.0%    0.0%    0.0%    0.0%    0.0%    0.0%    0.0%    0.0%    0.0%    0.0%    0.0%    0.0%    0.0%    0.0%    0.0%    0.0%    0.0%    0.0%    0.0%    0.0%    0.0%    0.0%    0.0%    0.0%    0.0%    0.0%    0.0%    0.0%    0.0%    0.0%    0.0%    0.0%    0.0%    0.0%    0.0%    0.0%    0.0%    0.0%    0.0%    0.0%    0.0%    0.0%    0.0%    0.0%    0.0%    0.0%    0.0%    0.0%    0.0%    0.0%    0.0%    0.0%    0.0%    0.0%    0.0%    0.0%    0.0%    0.0%    0.0%    0.0%    0.0%    0.0%    0.0%    0.0%    0.0%    0.0%    0.0%    0.0%    0.0%    0.0%    0.0%    0.0%    0.0%    0.0%    0.0%    0.0%    0.0%    0.0%    0.0%    0.0%    0.0%    0.0%    0.0%    0.0%    0.0%    0.0%    0.0%    0.0%    0.0%    0.0%    0.0%    0.0%    0.0%    0.0%    0.0%    0.0%    0.0%    0.0%    0.0%    0.0%    0.0%    0.0%    0.0%    0.0%    0.0%    0.0%    0.0%    0.0%    0.0%    0.0%    0.0%    0.0%    0.0%    0.0%    0.0%    0.0%    0.0%    0.0%    0.0%    0.0%    0.0%    0.0%    0.0%    0.0%    0.0%    0.0%    0.0%    0.0%    0.0%    0.0%    0.0%    0.0%    0.0%    0.0%    0.0%    0.0%    0.0%    0.0%    0.0%    0.0%    0.0%    0.0%    0.0%    0.0%    0.0%        k__Bacteria;p__Firmicutes;c__Clostridia; o__Natranaerobiales      10    0.0%    0.0%    0.0%    0.0%    0.0%    0.0%    0.0%    0.0%    0.0%    0.0%    0.0%    0.0%    0.0%    0.0%    0.0%    0.0%    0.0%    0.0%    0.0%    0.0%    0.0%    0.0%    0.0%    0.0%    0.0%    0.0%    0.0%    0.0%    0.0%    0.0%    0.0%    0.0%    0.0%    0.0%    0.0%    0.0%    0.0%    0.0%    0.0%    0.0%    0.0%    0.0%    0.0%    0.0%    0.0%    0.0%    0.0%    0.0%    0.0%    0.0%    0.0%    0.0%    0.0%    0.0%    0.0%    0.0%    0.0%    0.0%    0.0%    0.0%    0.0%    0.0%    0.0%    0.0%    0.0%    0.0%    0.0%    0.0%    0.0%    0.0%    0.0%    0.0%    0.0%    0.0%    0.0%    0.0%    0.0%    0.0%    0.0%    0.0%    0.0%    0.0%    0.0%    0.0%    0.0%    0.0%    0.0%    0.0%    0.0%    0.0%    0.0%    0.0%    0.0%    0.0%    0.0%    0.0%    0.0%    0.0%    0.0%    0.0%    0.0%    0.0%    0.0%    0.0%    0.0%    0.0%    0.0%    0.0%    0.0%    0.0%    0.0%    0.0%    0.0%    0.0%    0.0%    0.0%    0.0%    0.0%    0.0%    0.0%    0.0%    0.0%    0.0%    0.0%    0.0%    0.0%    0.0%    0.0%    0.0%    0.0%    0.0%    0.0%    0.0%    0.0%    0.0%    0.0%    0.0%    0.0%    0.0%    0.0%    0.0%    0.0%    0.0%    0.0%    0.0%    0.0%    0.0%    0.0%    0.0%    0.0%    0.0%    0.0%    0.0%    0.0%    0.0%    0.0%    0.0%    0.0%    0.0%    0.0%    0.0%    0.0%    0.0%    0.0%    0.0%    0.0%    0.0%    0.0%    0.0%    0.0%    0.0%    0.0%    0.0%    0.0%    0.0%    0.0%    0.0%    0.0%    0.0%    0.0%    0.0%    0.0%    0.0%    0.0%    0.0%    0.0%    0.0%    0.0%    0.0%    0.0%    0.0%    0.0%    0.0%    0.0%    0.0%    0.0%    0.0%    0.0%    0.0%    0.0%    0.0%    0.0%    0.0%    0.0%    0.0%    0.0%    0.0%    0.0%    0.0%    0.0%    0.0%    0.0%    0.0%    0.0%    0.0%    0.0%    0.0%    0.0%    0.0%    0.0%    0.0%    0.0%    0.0%    0.0%    0.0%    0.0%    0.0%    0.0%    0.0%    0.0%    0.0%    0.0%    0.0%    0.0%    0.0%    0.0%    0.0%    0.0%    0.0%    0.0%    0.0%    0.0%    0.0%    0.0%    0.0%    0.0%    0.0%    0.0%    0.0%    0.0%    0.0%    0.0%    0.0%    0.0%    0.0%    0.0%    0.0%    0.0%    0.0%    0.0%    0.0%    0.0%    0.0%    0.0%    0.0%    0.0%    0.0%    0.0%    0.0%    0.0%    0.0%    0.0%    0.0%    0.0%    0.0%    0.0%    0.0%    0.0%    0.0%    0.0%    0.0%    0.0%    0.0%    0.0%    0.0%    0.0%    0.0%    0.0%    0.0%    0.0%    0.0%    0.0%    0.0%    0.0%    0.0%    0.0%    0.0%    0.0%    0.0%    0.0%    0.0%    0.0%    0.0%    0.0%    0.0%    0.0%    0.0%    0.0%    0.0%    0.0%    0.0%    0.0%    0.0%    0.0%    0.0%    0.0%    0.0%    0.0%    0.0%    0.0%    0.0%    0.0%    0.0%    0.0%    0.0%    0.0%    0.0%    0.0%    0.0%    0.0%    0.0%    0.0%    0.0%    0.0%    0.0%    0.0%    0.0%    0.0%    0.0%    0.0%    0.0%    0.0%    0.0%    0.0%    0.0%    0.0%    0.0%    0.0%    0.0%    0.0%    0.0%    0.0%    0.0%    0.0%    0.0%    0.0%    0.0%    0.0%        k__Bacteria;p__Firmicutes;c__Clostridia; o__OPB54     107    0.0%    0.0%    0.0%    0.0%    0.0%    0.0%    0.0%    0.0%    0.0%    0.0%    0.0%    0.0%    0.0%    0.0%    0.0%    0.0%    0.0%    0.0%    0.0%    0.0%    0.0%    0.0%    0.0%    0.0%    0.0%    0.0%    0.0%    0.0%    0.0%    0.0%    0.0%    0.0%    0.0%    0.0%    0.0%    0.0%    0.0%    0.0%    0.0%    0.0%    0.0%    0.0%    0.0%    0.0%    0.0%    0.0%    0.0%    0.0%    0.0%    0.0%    0.0%    0.0%    0.0%    0.0%    0.0%    0.0%    0.0%    0.0%    0.0%    0.0%    0.0%    0.0%    0.0%    0.0%    0.0%    0.0%    0.0%    0.0%    0.0%    0.0%    0.0%    0.0%    0.0%    0.0%    0.0%    0.0%    0.0%    0.0%    0.0%    0.0%    0.0%    0.0%    0.0%    0.0%    0.0%    0.0%    0.0%    0.0%    0.0%    0.0%    0.0%    0.0%    0.0%    0.0%    0.0%    0.0%    0.0%    0.0%    0.0%    0.0%    0.0%    0.0%    0.0%    0.0%    0.0%    0.0%    0.0%    0.0%    0.0%    0.0%    0.0%    0.0%    0.0%    0.0%    0.0%    0.0%    0.0%    0.0%    0.0%    0.0%    0.0%    0.0%    0.0%    0.0%    0.0%    0.0%    0.0%    0.0%    0.0%    0.0%    0.0%    0.0%    0.0%    0.0%    0.0%    0.0%    0.0%    0.0%    0.0%    0.0%    0.0%    0.0%    0.0%    0.0%    0.0%    0.0%    0.0%    0.0%    0.0%    0.0%    0.0%    0.0%    0.0%    0.0%    0.0%    0.0%    0.0%    0.0%    0.0%    0.0%    0.0%    0.0%    0.0%    0.0%    0.0%    0.0%    0.0%    0.0%    0.0%    0.0%    0.0%    0.0%    0.0%    0.0%    0.0%    0.0%    0.0%    0.0%    0.0%    0.0%    0.0%    0.0%    0.0%    0.0%    0.0%    0.0%    0.0%    0.0%    0.0%    0.0%    0.0%    0.0%    0.0%    0.0%    0.0%    0.0%    0.0%    0.0%    0.0%    0.0%    0.0%    0.0%    0.0%    0.0%    0.0%    0.0%    0.0%    0.0%    0.0%    0.0%    0.0%    0.0%    0.0%    0.0%    0.0%    0.0%    0.0%    0.0%    0.0%    0.0%    0.0%    0.0%    0.0%    0.0%    0.0%    0.0%    0.0%    0.0%    0.0%    0.0%    0.0%    0.0%    0.0%    0.0%    0.0%    0.0%    0.0%    0.0%    0.0%    0.0%    0.0%    0.0%    0.0%    0.0%    0.0%    0.0%    0.0%    0.0%    0.0%    0.0%    0.0%    0.0%    0.0%    0.0%    0.0%    0.0%    0.0%    0.0%    0.0%    0.0%    0.0%    0.0%    0.0%    0.0%    0.0%    0.0%    0.0%    0.0%    0.0%    0.0%    0.0%    0.0%    0.0%    0.0%    0.0%    0.0%    0.0%    0.0%    0.0%    0.0%    0.0%    0.0%    0.0%    0.0%    0.0%    0.0%    0.0%    0.0%    0.0%    0.0%    0.0%    0.0%    0.0%    0.0%    0.0%    0.0%    0.0%    0.0%    0.0%    0.0%    0.0%    0.0%    0.0%    0.0%    0.0%    0.0%    0.0%    0.0%    0.0%    0.0%    0.0%    0.0%    0.0%    0.0%    0.0%    0.0%    0.0%    0.0%    0.0%    0.0%    0.0%    0.0%    0.0%    0.0%    0.0%    0.0%    0.0%    0.0%    0.0%    0.0%    0.0%    0.0%    0.0%    0.0%    0.0%    0.0%    0.0%    0.0%    0.0%    0.0%    0.0%    0.0%    0.0%    0.0%    0.0%    0.0%    0.0%    0.0%    0.0%    0.0%    0.0%    0.0%    0.0%    0.0%    0.0%    0.0%    0.0%    0.0%        k__Bacteria;p__Firmicutes;c__Clostridia; o__Thermoanaerobacterales      11    0.0%    0.0%    0.0%    0.0%    0.0%    0.0%    0.0%    0.0%    0.0%    0.0%    0.0%    0.0%    0.0%    0.0%    0.0%    0.0%    0.0%    0.0%    0.0%    0.0%    0.0%    0.0%    0.0%    0.0%    0.0%    0.0%    0.0%    0.0%    0.0%    0.0%    0.0%    0.0%    0.0%    0.0%    0.0%    0.0%    0.0%    0.0%    0.0%    0.0%    0.0%    0.0%    0.0%    0.0%    0.0%    0.0%    0.0%    0.0%    0.0%    0.0%    0.0%    0.0%    0.0%    0.0%    0.0%    0.0%    0.0%    0.0%    0.0%    0.0%    0.0%    0.0%    0.0%    0.0%    0.0%    0.0%    0.0%    0.0%    0.0%    0.0%    0.0%    0.0%    0.0%    0.0%    0.0%    0.0%    0.0%    0.0%    0.0%    0.0%    0.0%    0.0%    0.0%    0.0%    0.0%    0.0%    0.0%    0.0%    0.0%    0.0%    0.0%    0.0%    0.0%    0.0%    0.0%    0.0%    0.0%    0.0%    0.0%    0.0%    0.0%    0.0%    0.0%    0.0%    0.0%    0.0%    0.0%    0.0%    0.0%    0.0%    0.0%    0.0%    0.0%    0.0%    0.0%    0.0%    0.0%    0.0%    0.0%    0.0%    0.0%    0.0%    0.0%    0.0%    0.0%    0.0%    0.0%    0.0%    0.0%    0.0%    0.0%    0.0%    0.0%    0.0%    0.0%    0.0%    0.0%    0.0%    0.0%    0.0%    0.0%    0.0%    0.0%    0.0%    0.0%    0.0%    0.0%    0.0%    0.0%    0.0%    0.0%    0.0%    0.0%    0.0%    0.0%    0.0%    0.0%    0.0%    0.0%    0.0%    0.0%    0.0%    0.0%    0.0%    0.0%    0.0%    0.0%    0.0%    0.0%    0.0%    0.0%    0.0%    0.0%    0.0%    0.0%    0.0%    0.0%    0.0%    0.0%    0.0%    0.0%    0.0%    0.0%    0.0%    0.0%    0.0%    0.0%    0.0%    0.0%    0.0%    0.0%    0.0%    0.0%    0.0%    0.0%    0.0%    0.0%    0.0%    0.0%    0.0%    0.0%    0.0%    0.0%    0.0%    0.0%    0.0%    0.0%    0.0%    0.0%    0.0%    0.0%    0.0%    0.0%    0.0%    0.0%    0.0%    0.0%    0.0%    0.0%    0.0%    0.0%    0.0%    0.0%    0.0%    0.0%    0.0%    0.0%    0.0%    0.0%    0.0%    0.0%    0.0%    0.0%    0.0%    0.0%    0.0%    0.0%    0.0%    0.0%    0.0%    0.0%    0.0%    0.0%    0.0%    0.0%    0.0%    0.0%    0.0%    0.0%    0.0%    0.0%    0.0%    0.0%    0.0%    0.0%    0.0%    0.0%    0.0%    0.0%    0.0%    0.0%    0.0%    0.0%    0.0%    0.0%    0.0%    0.0%    0.0%    0.0%    0.0%    0.0%    0.0%    0.0%    0.0%    0.0%    0.0%    0.0%    0.0%    0.0%    0.0%    0.0%    0.0%    0.0%    0.0%    0.0%    0.0%    0.0%    0.0%    0.0%    0.0%    0.0%    0.0%    0.0%    0.0%    0.0%    0.0%    0.0%    0.0%    0.0%    0.0%    0.0%    0.0%    0.0%    0.0%    0.0%    0.0%    0.0%    0.0%    0.0%    0.0%    0.0%    0.0%    0.0%    0.0%    0.0%    0.0%    0.0%    0.0%    0.0%    0.0%    0.0%    0.0%    0.0%    0.0%    0.0%    0.0%    0.0%    0.0%    0.0%    0.0%    0.0%    0.0%    0.0%    0.0%    0.0%    0.0%    0.0%    0.0%    0.0%    0.0%    0.0%    0.0%    0.0%    0.0%    0.0%    0.0%    0.0%    0.0%    0.0%    0.0%    0.0%    0.0%    0.0%    0.0%    0.0%    0.0%    0.0%    0.0%        k__Bacteria;p__Fusobacteria;c__Fusobacteria (class); o__Fusobacteriales   292142    1.7%    9.1%   10.0%    8.4%    3.2%    0.1%    5.0%    1.7%    1.1%    3.9%    4.6%    0.2%    0.3%    7.3%    5.1%    1.3%    2.5%    0.9%    1.5%    0.5%    2.2%    1.4%    0.6%    0.1%    0.9%    1.1%    0.2%    2.7%    2.6%    0.7%    0.3%    4.0%    0.3%    8.1%    2.9%    7.5%    3.6%    1.0%    3.8%    1.4%    8.2%    5.2%    4.6%    4.3%   10.1%   19.1%    2.6%    0.9%    7.7%    4.2%    2.9%    0.8%    0.3%    1.8%    2.2%    2.0%    1.6%    0.7%    5.5%    1.2%    1.2%    1.7%    3.5%    6.5%    8.5%    2.2%    0.3%    4.3%    0.9%    3.6%    1.7%    1.1%    0.8%    2.7%    2.5%    0.2%    0.6%    3.4%    2.0%    4.5%    3.3%    0.5%    2.2%    2.9%    0.3%    0.7%    2.6%    0.1%    2.0%    1.7%    0.0%    0.6%    2.0%    0.6%    1.0%    0.7%    1.0%    0.1%    2.1%    2.3%    0.7%    0.4%    0.5%    0.8%    0.8%    3.8%    7.3%    0.1%    0.0%    1.2%    0.7%    4.0%    1.4%    0.1%    2.7%    0.8%    0.5%    0.5%    1.0%    1.0%    0.0%    0.7%    1.5%    1.5%    0.1%    1.0%    1.9%    0.1%    0.9%    0.7%    1.1%    0.3%    0.1%    0.8%    4.0%    0.2%    0.7%    0.5%    0.1%    0.7%    0.0%    1.7%    1.0%    0.2%    0.4%    0.5%    4.4%    1.1%    0.4%    0.1%    0.2%    0.4%    1.6%    0.4%    1.2%    0.3%    2.7%    0.2%    0.4%    1.9%    3.5%    2.0%    3.7%    0.8%    0.5%    0.9%    3.8%    0.8%    1.1%    1.7%    1.6%    2.9%    0.3%    1.4%    1.8%    0.6%    0.9%    0.8%    0.3%    1.0%    0.9%    0.9%    0.7%    0.3%    0.6%    0.8%    0.3%    1.2%    0.9%    1.1%    0.5%    1.5%    0.7%    0.4%    0.4%    0.5%    1.4%    1.7%    0.2%    0.4%    2.3%    6.6%    0.4%    1.4%    1.6%    1.3%    0.2%   14.4%    1.5%    1.0%    1.0%    0.6%    0.5%    9.6%    0.4%    0.5%    0.6%    1.5%    1.5%    0.6%    1.1%    0.5%    0.0%    0.4%    0.3%    0.8%    0.7%    2.8%    0.5%    2.8%    0.9%    0.2%    0.1%    0.8%    0.8%    0.1%    0.4%    0.4%    0.1%    0.2%    0.3%    0.4%    1.9%    1.2%    0.5%    0.1%    0.9%    0.0%    1.1%    2.2%    1.2%    2.9%    0.4%    0.5%    0.3%    0.2%    2.2%    0.4%    0.5%    2.8%    0.5%    0.6%    0.8%    0.2%    3.6%    0.0%    0.5%    1.4%    0.6%    0.2%    0.9%    1.1%    0.7%    1.5%    0.3%    0.5%    0.4%    0.9%    1.5%    0.2%    0.2%    0.1%    0.4%    0.1%    0.8%    1.8%    0.8%    1.1%    0.3%    0.0%    0.4%    0.3%    1.9%    1.8%    1.2%    0.2%    1.8%    1.1%    2.1%    0.8%    0.8%    0.8%    1.3%    0.9%    2.5%    1.0%    1.2%    0.5%    0.4%    5.3%    0.0%    0.3%    0.2%    3.6%    2.8%    0.6%    0.3%    2.0%    2.4%    0.6%    3.5%    4.2%    0.3%    0.3%    1.1%    1.3%    1.5%    0.9%    2.1%    1.5%    0.7%    1.5%    1.5%    0.3%    0.2%    0.8%    4.8%    1.5%    3.7%    0.1%    0.6%    0.1%    1.7%    0.5%    0.3%    0.7%    0.1%    1.7%    0.1%    0.1%    0.5%    8.7%    0.6%    0.4%    0.7%    0.1%    0.0%    1.7%        k__Bacteria;p__GN02;c__; o__       0    0.0%    0.0%    0.0%    0.0%    0.0%    0.0%    0.0%    0.0%    0.0%    0.0%    0.0%    0.0%    0.0%    0.0%    0.0%    0.0%    0.0%    0.0%    0.0%    0.0%    0.0%    0.0%    0.0%    0.0%    0.0%    0.0%    0.0%    0.0%    0.0%    0.0%    0.0%    0.0%    0.0%    0.0%    0.0%    0.0%    0.0%    0.0%    0.0%    0.0%    0.0%    0.0%    0.0%    0.0%    0.0%    0.0%    0.0%    0.0%    0.0%    0.0%    0.0%    0.0%    0.0%    0.0%    0.0%    0.0%    0.0%    0.0%    0.0%    0.0%    0.0%    0.0%    0.0%    0.0%    0.0%    0.0%    0.0%    0.0%    0.0%    0.0%    0.0%    0.0%    0.0%    0.0%    0.0%    0.0%    0.0%    0.0%    0.0%    0.0%    0.0%    0.0%    0.0%    0.0%    0.0%    0.0%    0.0%    0.0%    0.0%    0.0%    0.0%    0.0%    0.0%    0.0%    0.0%    0.0%    0.0%    0.0%    0.0%    0.0%    0.0%    0.0%    0.0%    0.0%    0.0%    0.0%    0.0%    0.0%    0.0%    0.0%    0.0%    0.0%    0.0%    0.0%    0.0%    0.0%    0.0%    0.0%    0.0%    0.0%    0.0%    0.0%    0.0%    0.0%    0.0%    0.0%    0.0%    0.0%    0.0%    0.0%    0.0%    0.0%    0.0%    0.0%    0.0%    0.0%    0.0%    0.0%    0.0%    0.0%    0.0%    0.0%    0.0%    0.0%    0.0%    0.0%    0.0%    0.0%    0.0%    0.0%    0.0%    0.0%    0.0%    0.0%    0.0%    0.0%    0.0%    0.0%    0.0%    0.0%    0.0%    0.0%    0.0%    0.0%    0.0%    0.0%    0.0%    0.0%    0.0%    0.0%    0.0%    0.0%    0.0%    0.0%    0.0%    0.0%    0.0%    0.0%    0.0%    0.0%    0.0%    0.0%    0.0%    0.0%    0.0%    0.0%    0.0%    0.0%    0.0%    0.0%    0.0%    0.0%    0.0%    0.0%    0.0%    0.0%    0.0%    0.0%    0.0%    0.0%    0.0%    0.0%    0.0%    0.0%    0.0%    0.0%    0.0%    0.0%    0.0%    0.0%    0.0%    0.0%    0.0%    0.0%    0.0%    0.0%    0.0%    0.0%    0.0%    0.0%    0.0%    0.0%    0.0%    0.0%    0.0%    0.0%    0.0%    0.0%    0.0%    0.0%    0.0%    0.0%    0.0%    0.0%    0.0%    0.0%    0.0%    0.0%    0.0%    0.0%    0.0%    0.0%    0.0%    0.0%    0.0%    0.0%    0.0%    0.0%    0.0%    0.0%    0.0%    0.0%    0.0%    0.0%    0.0%    0.0%    0.0%    0.0%    0.0%    0.0%    0.0%    0.0%    0.0%    0.0%    0.0%    0.0%    0.0%    0.0%    0.0%    0.0%    0.0%    0.0%    0.0%    0.0%    0.0%    0.0%    0.0%    0.0%    0.0%    0.0%    0.0%    0.0%    0.0%    0.0%    0.0%    0.0%    0.0%    0.0%    0.0%    0.0%    0.0%    0.0%    0.0%    0.0%    0.0%    0.0%    0.0%    0.0%    0.0%    0.0%    0.0%    0.0%    0.0%    0.0%    0.0%    0.0%    0.0%    0.0%    0.0%    0.0%    0.0%    0.0%    0.0%    0.0%    0.0%    0.0%    0.0%    0.0%    0.0%    0.0%    0.0%    0.0%    0.0%    0.0%    0.0%    0.0%    0.0%    0.0%    0.0%    0.0%    0.0%    0.0%    0.0%    0.0%    0.0%    0.0%    0.0%    0.0%    0.0%    0.0%    0.0%    0.0%    0.0%    0.0%    0.0%    0.0%    0.0%    0.0%    0.0%    0.0%    0.0%    0.0%    0.0%    0.0%    0.0%    0.0%    0.0%    0.0%        k__Bacteria;p__GN02;c__VC12-cl04; o__       1    0.0%    0.0%    0.0%    0.0%    0.0%    0.0%    0.0%    0.0%    0.0%    0.0%    0.0%    0.0%    0.0%    0.0%    0.0%    0.0%    0.0%    0.0%    0.0%    0.0%    0.0%    0.0%    0.0%    0.0%    0.0%    0.0%    0.0%    0.0%    0.0%    0.0%    0.0%    0.0%    0.0%    0.0%    0.0%    0.0%    0.0%    0.0%    0.0%    0.0%    0.0%    0.0%    0.0%    0.0%    0.0%    0.0%    0.0%    0.0%    0.0%    0.0%    0.0%    0.0%    0.0%    0.0%    0.0%    0.0%    0.0%    0.0%    0.0%    0.0%    0.0%    0.0%    0.0%    0.0%    0.0%    0.0%    0.0%    0.0%    0.0%    0.0%    0.0%    0.0%    0.0%    0.0%    0.0%    0.0%    0.0%    0.0%    0.0%    0.0%    0.0%    0.0%    0.0%    0.0%    0.0%    0.0%    0.0%    0.0%    0.0%    0.0%    0.0%    0.0%    0.0%    0.0%    0.0%    0.0%    0.0%    0.0%    0.0%    0.0%    0.0%    0.0%    0.0%    0.0%    0.0%    0.0%    0.0%    0.0%    0.0%    0.0%    0.0%    0.0%    0.0%    0.0%    0.0%    0.0%    0.0%    0.0%    0.0%    0.0%    0.0%    0.0%    0.0%    0.0%    0.0%    0.0%    0.0%    0.0%    0.0%    0.0%    0.0%    0.0%    0.0%    0.0%    0.0%    0.0%    0.0%    0.0%    0.0%    0.0%    0.0%    0.0%    0.0%    0.0%    0.0%    0.0%    0.0%    0.0%    0.0%    0.0%    0.0%    0.0%    0.0%    0.0%    0.0%    0.0%    0.0%    0.0%    0.0%    0.0%    0.0%    0.0%    0.0%    0.0%    0.0%    0.0%    0.0%    0.0%    0.0%    0.0%    0.0%    0.0%    0.0%    0.0%    0.0%    0.0%    0.0%    0.0%    0.0%    0.0%    0.0%    0.0%    0.0%    0.0%    0.0%    0.0%    0.0%    0.0%    0.0%    0.0%    0.0%    0.0%    0.0%    0.0%    0.0%    0.0%    0.0%    0.0%    0.0%    0.0%    0.0%    0.0%    0.0%    0.0%    0.0%    0.0%    0.0%    0.0%    0.0%    0.0%    0.0%    0.0%    0.0%    0.0%    0.0%    0.0%    0.0%    0.0%    0.0%    0.0%    0.0%    0.0%    0.0%    0.0%    0.0%    0.0%    0.0%    0.0%    0.0%    0.0%    0.0%    0.0%    0.0%    0.0%    0.0%    0.0%    0.0%    0.0%    0.0%    0.0%    0.0%    0.0%    0.0%    0.0%    0.0%    0.0%    0.0%    0.0%    0.0%    0.0%    0.0%    0.0%    0.0%    0.0%    0.0%    0.0%    0.0%    0.0%    0.0%    0.0%    0.0%    0.0%    0.0%    0.0%    0.0%    0.0%    0.0%    0.0%    0.0%    0.0%    0.0%    0.0%    0.0%    0.0%    0.0%    0.0%    0.0%    0.0%    0.0%    0.0%    0.0%    0.0%    0.0%    0.0%    0.0%    0.0%    0.0%    0.0%    0.0%    0.0%    0.0%    0.0%    0.0%    0.0%    0.0%    0.0%    0.0%    0.0%    0.0%    0.0%    0.0%    0.0%    0.0%    0.0%    0.0%    0.0%    0.0%    0.0%    0.0%    0.0%    0.0%    0.0%    0.0%    0.0%    0.0%    0.0%    0.0%    0.0%    0.0%    0.0%    0.0%    0.0%    0.0%    0.0%    0.0%    0.0%    0.0%    0.0%    0.0%    0.0%    0.0%    0.0%    0.0%    0.0%    0.0%    0.0%    0.0%    0.0%    0.0%    0.0%    0.0%    0.0%    0.0%    0.0%    0.0%    0.0%    0.0%    0.0%    0.0%    0.0%    0.0%    0.0%    0.0%    0.0%    0.0%    0.0%    0.0%    0.0%        k__Bacteria;p__Gemmatimonadetes;c__Gemmatimonadetes (class); o__     670    0.0%    0.0%    0.0%    0.0%    0.0%    0.0%    0.0%    0.0%    0.0%    0.0%    0.0%    0.0%    0.0%    0.0%    0.0%    0.0%    0.0%    0.0%    0.0%    0.0%    0.0%    0.0%    0.0%    0.0%    0.0%    0.0%    0.0%    0.0%    0.0%    0.0%    0.0%    0.0%    0.0%    0.0%    0.0%    0.0%    0.0%    0.0%    0.0%    0.0%    0.0%    0.0%    0.0%    0.0%    0.0%    0.0%    0.0%    0.0%    0.0%    0.0%    0.0%    0.0%    0.0%    0.0%    0.0%    0.0%    0.0%    0.0%    0.0%    0.0%    0.0%    0.0%    0.0%    0.0%    0.0%    0.0%    0.0%    0.0%    0.0%    0.0%    0.0%    0.0%    0.0%    0.0%    0.0%    0.0%    0.0%    0.0%    0.0%    0.0%    0.0%    0.0%    0.0%    0.0%    0.0%    0.0%    0.0%    0.0%    0.0%    0.0%    0.0%    0.0%    0.0%    0.0%    0.0%    0.0%    0.0%    0.0%    0.0%    0.0%    0.0%    0.0%    0.0%    0.0%    0.0%    0.0%    0.0%    0.0%    0.0%    0.0%    0.0%    0.0%    0.0%    0.0%    0.0%    0.0%    0.0%    0.0%    0.0%    0.0%    0.0%    0.0%    0.0%    0.0%    0.0%    0.0%    0.0%    0.0%    0.0%    0.0%    0.0%    0.0%    0.0%    0.0%    0.0%    0.0%    0.0%    0.0%    0.0%    0.0%    0.0%    0.0%    0.0%    0.0%    0.0%    0.0%    0.0%    0.0%    0.0%    0.0%    0.0%    0.0%    0.0%    0.0%    0.0%    0.0%    0.0%    0.0%    0.0%    0.0%    0.0%    0.0%    0.0%    0.0%    0.0%    0.0%    0.0%    0.0%    0.0%    0.0%    0.0%    0.0%    0.0%    0.0%    0.0%    0.0%    0.0%    0.0%    0.0%    0.0%    0.0%    0.0%    0.0%    0.0%    0.0%    0.0%    0.0%    0.0%    0.0%    0.0%    0.0%    0.0%    0.0%    0.0%    0.0%    0.0%    0.0%    0.0%    0.0%    0.0%    0.0%    0.0%    0.0%    0.0%    0.0%    0.0%    0.0%    0.0%    0.0%    0.0%    0.0%    0.0%    0.0%    0.0%    0.0%    0.0%    0.0%    0.0%    0.0%    0.0%    0.0%    0.0%    0.0%    0.0%    0.0%    0.0%    0.0%    0.0%    0.0%    0.0%    0.0%    0.0%    0.0%    0.0%    0.0%    0.0%    0.0%    0.0%    0.0%    0.0%    0.0%    0.0%    0.0%    0.0%    0.0%    0.0%    0.0%    0.0%    0.0%    0.0%    0.0%    0.0%    0.0%    0.0%    0.0%    0.0%    0.0%    0.0%    0.0%    0.0%    0.0%    0.0%    0.0%    0.0%    0.0%    0.0%    0.0%    0.0%    0.0%    0.0%    0.0%    0.0%    0.0%    0.0%    0.0%    0.0%    0.0%    0.0%    0.0%    0.0%    0.0%    0.0%    0.0%    0.0%    0.0%    0.0%    0.0%    0.0%    0.0%    0.0%    0.0%    0.0%    0.0%    0.0%    0.0%    0.0%    0.0%    0.0%    0.0%    0.0%    0.0%    0.0%    0.0%    0.0%    0.0%    0.0%    0.0%    0.0%    0.0%    0.0%    0.0%    0.0%    0.0%    0.0%    0.0%    0.0%    0.0%    0.0%    0.0%    0.0%    0.0%    0.0%    0.0%    0.0%    0.0%    0.0%    0.0%    0.0%    0.0%    0.0%    0.0%    0.0%    0.0%    0.0%    0.0%    0.0%    0.0%    0.0%    0.0%    0.0%    0.0%    0.0%    0.0%    0.0%    0.0%    0.0%    0.0%    0.0%    0.0%    0.0%    0.0%    0.0%    0.0%    0.0%    0.0%    0.0%    0.0%    0.0%        k__Bacteria;p__Gemmatimonadetes;c__Gemmatimonadetes (class); o__Gemmatimonadales    4098    0.0%    0.0%    0.0%    0.0%    0.0%    0.0%    0.0%    0.0%    0.0%    0.0%    0.0%    0.0%    0.0%    0.0%    0.0%    0.0%    0.0%    0.0%    0.0%    0.0%    0.0%    0.0%    0.0%    0.0%    0.0%    0.0%    0.0%    0.0%    0.0%    0.0%    0.0%    0.0%    0.0%    0.0%    0.0%    0.0%    0.0%    0.0%    0.0%    0.1%    0.0%    0.0%    0.0%    0.0%    0.0%    0.0%    0.0%    0.0%    0.0%    0.0%    0.0%    0.0%    0.0%    0.0%    0.0%    0.0%    0.0%    0.0%    0.0%    0.0%    0.0%    0.0%    0.0%    0.0%    0.0%    0.0%    0.0%    0.1%    0.0%    0.1%    0.0%    0.0%    0.0%    0.1%    0.1%    0.0%    0.0%    0.0%    0.0%    0.0%    0.0%    0.0%    0.0%    0.0%    0.0%    0.0%    0.0%    0.0%    0.0%    0.0%    0.0%    0.0%    0.0%    0.0%    0.0%    0.0%    0.1%    0.0%    0.0%    0.1%    0.1%    0.0%    0.0%    0.0%    0.0%    0.0%    0.1%    0.0%    0.0%    0.1%    0.0%    0.1%    0.0%    0.0%    0.0%    0.0%    0.0%    0.0%    0.0%    0.1%    0.0%    0.0%    0.1%    0.0%    0.0%    0.3%    0.1%    0.0%    0.1%    0.1%    0.1%    0.0%    0.1%    0.0%    0.1%    0.0%    0.0%    0.0%    0.0%    0.0%    0.0%    0.0%    0.1%    0.0%    0.0%    0.0%    0.0%    0.0%    0.0%    0.0%    0.0%    0.0%    0.1%    0.0%    0.0%    0.0%    0.1%    0.0%    0.0%    0.1%    0.1%    0.1%    0.1%    0.0%    0.0%    0.0%    0.1%    0.0%    0.0%    0.0%    0.0%    0.1%    0.0%    0.0%    0.0%    0.0%    0.0%    0.0%    0.0%    0.0%    0.0%    0.0%    0.0%    0.0%    0.0%    0.0%    0.0%    0.0%    0.0%    0.0%    0.0%    0.0%    0.0%    0.0%    0.0%    0.0%    0.1%    0.0%    0.0%    0.0%    0.0%    0.0%    0.0%    0.0%    0.0%    0.0%    0.0%    0.0%    0.1%    0.1%    0.0%    0.0%    0.1%    0.0%    0.0%    0.0%    0.0%    0.0%    0.0%    0.1%    0.0%    0.0%    0.0%    0.0%    0.0%    0.0%    0.0%    0.0%    0.0%    0.0%    0.0%    0.0%    0.1%    0.0%    0.1%    0.0%    0.1%    0.0%    0.0%    0.0%    0.0%    0.0%    0.0%    0.0%    0.0%    0.0%    0.0%    0.0%    0.0%    0.0%    0.1%    0.0%    0.0%    0.0%    0.0%    0.0%    0.0%    0.0%    0.0%    0.0%    0.1%    0.1%    0.0%    0.0%    0.1%    0.0%    0.0%    0.0%    0.0%    0.0%    0.0%    0.0%    0.1%    0.0%    0.0%    0.0%    0.0%    0.0%    0.0%    0.0%    0.0%    0.0%    0.0%    0.0%    0.0%    0.0%    0.0%    0.2%    0.0%    0.0%    0.0%    0.0%    0.0%    0.0%    0.1%    0.0%    0.0%    0.0%    0.0%    0.0%    0.0%    0.0%    0.0%    0.1%    0.0%    0.0%    0.0%    0.0%    0.0%    0.0%    0.0%    0.0%    0.0%    0.0%    0.0%    0.0%    0.0%    0.0%    0.0%    0.0%    0.0%    0.0%    0.0%    0.0%    0.0%    0.0%    0.0%    0.0%    0.0%    0.0%    0.0%    0.0%    0.0%    0.0%    0.0%    0.0%    0.0%    0.0%    0.0%    0.0%    0.0%    0.0%    0.0%    0.0%    0.0%    0.0%    0.0%    0.0%    0.0%    0.0%    0.0%    0.0%    0.0%    0.0%    0.0%    0.0%    0.0%    0.0%        k__Bacteria;p__Lentisphaerae;c__; o__       0    0.0%    0.0%    0.0%    0.0%    0.0%    0.0%    0.0%    0.0%    0.0%    0.0%    0.0%    0.0%    0.0%    0.0%    0.0%    0.0%    0.0%    0.0%    0.0%    0.0%    0.0%    0.0%    0.0%    0.0%    0.0%    0.0%    0.0%    0.0%    0.0%    0.0%    0.0%    0.0%    0.0%    0.0%    0.0%    0.0%    0.0%    0.0%    0.0%    0.0%    0.0%    0.0%    0.0%    0.0%    0.0%    0.0%    0.0%    0.0%    0.0%    0.0%    0.0%    0.0%    0.0%    0.0%    0.0%    0.0%    0.0%    0.0%    0.0%    0.0%    0.0%    0.0%    0.0%    0.0%    0.0%    0.0%    0.0%    0.0%    0.0%    0.0%    0.0%    0.0%    0.0%    0.0%    0.0%    0.0%    0.0%    0.0%    0.0%    0.0%    0.0%    0.0%    0.0%    0.0%    0.0%    0.0%    0.0%    0.0%    0.0%    0.0%    0.0%    0.0%    0.0%    0.0%    0.0%    0.0%    0.0%    0.0%    0.0%    0.0%    0.0%    0.0%    0.0%    0.0%    0.0%    0.0%    0.0%    0.0%    0.0%    0.0%    0.0%    0.0%    0.0%    0.0%    0.0%    0.0%    0.0%    0.0%    0.0%    0.0%    0.0%    0.0%    0.0%    0.0%    0.0%    0.0%    0.0%    0.0%    0.0%    0.0%    0.0%    0.0%    0.0%    0.0%    0.0%    0.0%    0.0%    0.0%    0.0%    0.0%    0.0%    0.0%    0.0%    0.0%    0.0%    0.0%    0.0%    0.0%    0.0%    0.0%    0.0%    0.0%    0.0%    0.0%    0.0%    0.0%    0.0%    0.0%    0.0%    0.0%    0.0%    0.0%    0.0%    0.0%    0.0%    0.0%    0.0%    0.0%    0.0%    0.0%    0.0%    0.0%    0.0%    0.0%    0.0%    0.0%    0.0%    0.0%    0.0%    0.0%    0.0%    0.0%    0.0%    0.0%    0.0%    0.0%    0.0%    0.0%    0.0%    0.0%    0.0%    0.0%    0.0%    0.0%    0.0%    0.0%    0.0%    0.0%    0.0%    0.0%    0.0%    0.0%    0.0%    0.0%    0.0%    0.0%    0.0%    0.0%    0.0%    0.0%    0.0%    0.0%    0.0%    0.0%    0.0%    0.0%    0.0%    0.0%    0.0%    0.0%    0.0%    0.0%    0.0%    0.0%    0.0%    0.0%    0.0%    0.0%    0.0%    0.0%    0.0%    0.0%    0.0%    0.0%    0.0%    0.0%    0.0%    0.0%    0.0%    0.0%    0.0%    0.0%    0.0%    0.0%    0.0%    0.0%    0.0%    0.0%    0.0%    0.0%    0.0%    0.0%    0.0%    0.0%    0.0%    0.0%    0.0%    0.0%    0.0%    0.0%    0.0%    0.0%    0.0%    0.0%    0.0%    0.0%    0.0%    0.0%    0.0%    0.0%    0.0%    0.0%    0.0%    0.0%    0.0%    0.0%    0.0%    0.0%    0.0%    0.0%    0.0%    0.0%    0.0%    0.0%    0.0%    0.0%    0.0%    0.0%    0.0%    0.0%    0.0%    0.0%    0.0%    0.0%    0.0%    0.0%    0.0%    0.0%    0.0%    0.0%    0.0%    0.0%    0.0%    0.0%    0.0%    0.0%    0.0%    0.0%    0.0%    0.0%    0.0%    0.0%    0.0%    0.0%    0.0%    0.0%    0.0%    0.0%    0.0%    0.0%    0.0%    0.0%    0.0%    0.0%    0.0%    0.0%    0.0%    0.0%    0.0%    0.0%    0.0%    0.0%    0.0%    0.0%    0.0%    0.0%    0.0%    0.0%    0.0%    0.0%    0.0%    0.0%    0.0%    0.0%    0.0%    0.0%    0.0%    0.0%    0.0%    0.0%    0.0%    0.0%    0.0%    0.0%    0.0%    0.0%    0.0%    0.0%        k__Bacteria;p__Lentisphaerae;c__Lentisphaerae (class); o__Victivallales       7    0.0%    0.0%    0.0%    0.0%    0.0%    0.0%    0.0%    0.0%    0.0%    0.0%    0.0%    0.0%    0.0%    0.0%    0.0%    0.0%    0.0%    0.0%    0.0%    0.0%    0.0%    0.0%    0.0%    0.0%    0.0%    0.0%    0.0%    0.0%    0.0%    0.0%    0.0%    0.0%    0.0%    0.0%    0.0%    0.0%    0.0%    0.0%    0.0%    0.0%    0.0%    0.0%    0.0%    0.0%    0.0%    0.0%    0.0%    0.0%    0.0%    0.0%    0.0%    0.0%    0.0%    0.0%    0.0%    0.0%    0.0%    0.0%    0.0%    0.0%    0.0%    0.0%    0.0%    0.0%    0.0%    0.0%    0.0%    0.0%    0.0%    0.0%    0.0%    0.0%    0.0%    0.0%    0.0%    0.0%    0.0%    0.0%    0.0%    0.0%    0.0%    0.0%    0.0%    0.0%    0.0%    0.0%    0.0%    0.0%    0.0%    0.0%    0.0%    0.0%    0.0%    0.0%    0.0%    0.0%    0.0%    0.0%    0.0%    0.0%    0.0%    0.0%    0.0%    0.0%    0.0%    0.0%    0.0%    0.0%    0.0%    0.0%    0.0%    0.0%    0.0%    0.0%    0.0%    0.0%    0.0%    0.0%    0.0%    0.0%    0.0%    0.0%    0.0%    0.0%    0.0%    0.0%    0.0%    0.0%    0.0%    0.0%    0.0%    0.0%    0.0%    0.0%    0.0%    0.0%    0.0%    0.0%    0.0%    0.0%    0.0%    0.0%    0.0%    0.0%    0.0%    0.0%    0.0%    0.0%    0.0%    0.0%    0.0%    0.0%    0.0%    0.0%    0.0%    0.0%    0.0%    0.0%    0.0%    0.0%    0.0%    0.0%    0.0%    0.0%    0.0%    0.0%    0.0%    0.0%    0.0%    0.0%    0.0%    0.0%    0.0%    0.0%    0.0%    0.0%    0.0%    0.0%    0.0%    0.0%    0.0%    0.0%    0.0%    0.0%    0.0%    0.0%    0.0%    0.0%    0.0%    0.0%    0.0%    0.0%    0.0%    0.0%    0.0%    0.0%    0.0%    0.0%    0.0%    0.0%    0.0%    0.0%    0.0%    0.0%    0.0%    0.0%    0.0%    0.0%    0.0%    0.0%    0.0%    0.0%    0.0%    0.0%    0.0%    0.0%    0.0%    0.0%    0.0%    0.0%    0.0%    0.0%    0.0%    0.0%    0.0%    0.0%    0.0%    0.0%    0.0%    0.0%    0.0%    0.0%    0.0%    0.0%    0.0%    0.0%    0.0%    0.0%    0.0%    0.0%    0.0%    0.0%    0.0%    0.0%    0.0%    0.0%    0.0%    0.0%    0.0%    0.0%    0.0%    0.0%    0.0%    0.0%    0.0%    0.0%    0.0%    0.0%    0.0%    0.0%    0.0%    0.0%    0.0%    0.0%    0.0%    0.0%    0.0%    0.0%    0.0%    0.0%    0.0%    0.0%    0.0%    0.0%    0.0%    0.0%    0.0%    0.0%    0.0%    0.0%    0.0%    0.0%    0.0%    0.0%    0.0%    0.0%    0.0%    0.0%    0.0%    0.0%    0.0%    0.0%    0.0%    0.0%    0.0%    0.0%    0.0%    0.0%    0.0%    0.0%    0.0%    0.0%    0.0%    0.0%    0.0%    0.0%    0.0%    0.0%    0.0%    0.0%    0.0%    0.0%    0.0%    0.0%    0.0%    0.0%    0.0%    0.0%    0.0%    0.0%    0.0%    0.0%    0.0%    0.0%    0.0%    0.0%    0.0%    0.0%    0.0%    0.0%    0.0%    0.0%    0.0%    0.0%    0.0%    0.0%    0.0%    0.0%    0.0%    0.0%    0.0%    0.0%    0.0%    0.0%    0.0%    0.0%    0.0%    0.0%    0.0%    0.0%    0.0%    0.0%    0.0%    0.0%    0.0%    0.0%    0.0%    0.0%        k__Bacteria;p__MVP-15;c__; o__       1    0.0%    0.0%    0.0%    0.0%    0.0%    0.0%    0.0%    0.0%    0.0%    0.0%    0.0%    0.0%    0.0%    0.0%    0.0%    0.0%    0.0%    0.0%    0.0%    0.0%    0.0%    0.0%    0.0%    0.0%    0.0%    0.0%    0.0%    0.0%    0.0%    0.0%    0.0%    0.0%    0.0%    0.0%    0.0%    0.0%    0.0%    0.0%    0.0%    0.0%    0.0%    0.0%    0.0%    0.0%    0.0%    0.0%    0.0%    0.0%    0.0%    0.0%    0.0%    0.0%    0.0%    0.0%    0.0%    0.0%    0.0%    0.0%    0.0%    0.0%    0.0%    0.0%    0.0%    0.0%    0.0%    0.0%    0.0%    0.0%    0.0%    0.0%    0.0%    0.0%    0.0%    0.0%    0.0%    0.0%    0.0%    0.0%    0.0%    0.0%    0.0%    0.0%    0.0%    0.0%    0.0%    0.0%    0.0%    0.0%    0.0%    0.0%    0.0%    0.0%    0.0%    0.0%    0.0%    0.0%    0.0%    0.0%    0.0%    0.0%    0.0%    0.0%    0.0%    0.0%    0.0%    0.0%    0.0%    0.0%    0.0%    0.0%    0.0%    0.0%    0.0%    0.0%    0.0%    0.0%    0.0%    0.0%    0.0%    0.0%    0.0%    0.0%    0.0%    0.0%    0.0%    0.0%    0.0%    0.0%    0.0%    0.0%    0.0%    0.0%    0.0%    0.0%    0.0%    0.0%    0.0%    0.0%    0.0%    0.0%    0.0%    0.0%    0.0%    0.0%    0.0%    0.0%    0.0%    0.0%    0.0%    0.0%    0.0%    0.0%    0.0%    0.0%    0.0%    0.0%    0.0%    0.0%    0.0%    0.0%    0.0%    0.0%    0.0%    0.0%    0.0%    0.0%    0.0%    0.0%    0.0%    0.0%    0.0%    0.0%    0.0%    0.0%    0.0%    0.0%    0.0%    0.0%    0.0%    0.0%    0.0%    0.0%    0.0%    0.0%    0.0%    0.0%    0.0%    0.0%    0.0%    0.0%    0.0%    0.0%    0.0%    0.0%    0.0%    0.0%    0.0%    0.0%    0.0%    0.0%    0.0%    0.0%    0.0%    0.0%    0.0%    0.0%    0.0%    0.0%    0.0%    0.0%    0.0%    0.0%    0.0%    0.0%    0.0%    0.0%    0.0%    0.0%    0.0%    0.0%    0.0%    0.0%    0.0%    0.0%    0.0%    0.0%    0.0%    0.0%    0.0%    0.0%    0.0%    0.0%    0.0%    0.0%    0.0%    0.0%    0.0%    0.0%    0.0%    0.0%    0.0%    0.0%    0.0%    0.0%    0.0%    0.0%    0.0%    0.0%    0.0%    0.0%    0.0%    0.0%    0.0%    0.0%    0.0%    0.0%    0.0%    0.0%    0.0%    0.0%    0.0%    0.0%    0.0%    0.0%    0.0%    0.0%    0.0%    0.0%    0.0%    0.0%    0.0%    0.0%    0.0%    0.0%    0.0%    0.0%    0.0%    0.0%    0.0%    0.0%    0.0%    0.0%    0.0%    0.0%    0.0%    0.0%    0.0%    0.0%    0.0%    0.0%    0.0%    0.0%    0.0%    0.0%    0.0%    0.0%    0.0%    0.0%    0.0%    0.0%    0.0%    0.0%    0.0%    0.0%    0.0%    0.0%    0.0%    0.0%    0.0%    0.0%    0.0%    0.0%    0.0%    0.0%    0.0%    0.0%    0.0%    0.0%    0.0%    0.0%    0.0%    0.0%    0.0%    0.0%    0.0%    0.0%    0.0%    0.0%    0.0%    0.0%    0.0%    0.0%    0.0%    0.0%    0.0%    0.0%    0.0%    0.0%    0.0%    0.0%    0.0%    0.0%    0.0%    0.0%    0.0%    0.0%    0.0%    0.0%    0.0%    0.0%    0.0%    0.0%    0.0%    0.0%    0.0%    0.0%    0.0%    0.0%        k__Bacteria;p__NC10;c__; o__       6    0.0%    0.0%    0.0%    0.0%    0.0%    0.0%    0.0%    0.0%    0.0%    0.0%    0.0%    0.0%    0.0%    0.0%    0.0%    0.0%    0.0%    0.0%    0.0%    0.0%    0.0%    0.0%    0.0%    0.0%    0.0%    0.0%    0.0%    0.0%    0.0%    0.0%    0.0%    0.0%    0.0%    0.0%    0.0%    0.0%    0.0%    0.0%    0.0%    0.0%    0.0%    0.0%    0.0%    0.0%    0.0%    0.0%    0.0%    0.0%    0.0%    0.0%    0.0%    0.0%    0.0%    0.0%    0.0%    0.0%    0.0%    0.0%    0.0%    0.0%    0.0%    0.0%    0.0%    0.0%    0.0%    0.0%    0.0%    0.0%    0.0%    0.0%    0.0%    0.0%    0.0%    0.0%    0.0%    0.0%    0.0%    0.0%    0.0%    0.0%    0.0%    0.0%    0.0%    0.0%    0.0%    0.0%    0.0%    0.0%    0.0%    0.0%    0.0%    0.0%    0.0%    0.0%    0.0%    0.0%    0.0%    0.0%    0.0%    0.0%    0.0%    0.0%    0.0%    0.0%    0.0%    0.0%    0.0%    0.0%    0.0%    0.0%    0.0%    0.0%    0.0%    0.0%    0.0%    0.0%    0.0%    0.0%    0.0%    0.0%    0.0%    0.0%    0.0%    0.0%    0.0%    0.0%    0.0%    0.0%    0.0%    0.0%    0.0%    0.0%    0.0%    0.0%    0.0%    0.0%    0.0%    0.0%    0.0%    0.0%    0.0%    0.0%    0.0%    0.0%    0.0%    0.0%    0.0%    0.0%    0.0%    0.0%    0.0%    0.0%    0.0%    0.0%    0.0%    0.0%    0.0%    0.0%    0.0%    0.0%    0.0%    0.0%    0.0%    0.0%    0.0%    0.0%    0.0%    0.0%    0.0%    0.0%    0.0%    0.0%    0.0%    0.0%    0.0%    0.0%    0.0%    0.0%    0.0%    0.0%    0.0%    0.0%    0.0%    0.0%    0.0%    0.0%    0.0%    0.0%    0.0%    0.0%    0.0%    0.0%    0.0%    0.0%    0.0%    0.0%    0.0%    0.0%    0.0%    0.0%    0.0%    0.0%    0.0%    0.0%    0.0%    0.0%    0.0%    0.0%    0.0%    0.0%    0.0%    0.0%    0.0%    0.0%    0.0%    0.0%    0.0%    0.0%    0.0%    0.0%    0.0%    0.0%    0.0%    0.0%    0.0%    0.0%    0.0%    0.0%    0.0%    0.0%    0.0%    0.0%    0.0%    0.0%    0.0%    0.0%    0.0%    0.0%    0.0%    0.0%    0.0%    0.0%    0.0%    0.0%    0.0%    0.0%    0.0%    0.0%    0.0%    0.0%    0.0%    0.0%    0.0%    0.0%    0.0%    0.0%    0.0%    0.0%    0.0%    0.0%    0.0%    0.0%    0.0%    0.0%    0.0%    0.0%    0.0%    0.0%    0.0%    0.0%    0.0%    0.0%    0.0%    0.0%    0.0%    0.0%    0.0%    0.0%    0.0%    0.0%    0.0%    0.0%    0.0%    0.0%    0.0%    0.0%    0.0%    0.0%    0.0%    0.0%    0.0%    0.0%    0.0%    0.0%    0.0%    0.0%    0.0%    0.0%    0.0%    0.0%    0.0%    0.0%    0.0%    0.0%    0.0%    0.0%    0.0%    0.0%    0.0%    0.0%    0.0%    0.0%    0.0%    0.0%    0.0%    0.0%    0.0%    0.0%    0.0%    0.0%    0.0%    0.0%    0.0%    0.0%    0.0%    0.0%    0.0%    0.0%    0.0%    0.0%    0.0%    0.0%    0.0%    0.0%    0.0%    0.0%    0.0%    0.0%    0.0%    0.0%    0.0%    0.0%    0.0%    0.0%    0.0%    0.0%    0.0%    0.0%    0.0%    0.0%    0.0%    0.0%    0.0%    0.0%    0.0%    0.0%    0.0%    0.0%        k__Bacteria;p__NKB19;c__; o__      24    0.0%    0.0%    0.0%    0.0%    0.0%    0.0%    0.0%    0.0%    0.0%    0.0%    0.0%    0.0%    0.0%    0.0%    0.0%    0.0%    0.0%    0.0%    0.0%    0.0%    0.0%    0.0%    0.0%    0.0%    0.0%    0.0%    0.0%    0.0%    0.0%    0.0%    0.0%    0.0%    0.0%    0.0%    0.0%    0.0%    0.0%    0.0%    0.0%    0.0%    0.0%    0.0%    0.0%    0.0%    0.0%    0.0%    0.0%    0.0%    0.0%    0.0%    0.0%    0.0%    0.0%    0.0%    0.0%    0.0%    0.0%    0.0%    0.0%    0.0%    0.0%    0.0%    0.0%    0.0%    0.0%    0.0%    0.0%    0.0%    0.0%    0.0%    0.0%    0.0%    0.0%    0.0%    0.0%    0.0%    0.0%    0.0%    0.0%    0.0%    0.0%    0.0%    0.0%    0.0%    0.0%    0.0%    0.0%    0.0%    0.0%    0.0%    0.0%    0.0%    0.0%    0.0%    0.0%    0.0%    0.0%    0.0%    0.0%    0.0%    0.0%    0.0%    0.0%    0.0%    0.0%    0.0%    0.0%    0.0%    0.0%    0.0%    0.0%    0.0%    0.0%    0.0%    0.0%    0.0%    0.0%    0.0%    0.0%    0.0%    0.0%    0.0%    0.0%    0.0%    0.0%    0.0%    0.0%    0.0%    0.0%    0.0%    0.0%    0.0%    0.0%    0.0%    0.0%    0.0%    0.0%    0.0%    0.0%    0.0%    0.0%    0.0%    0.0%    0.0%    0.0%    0.0%    0.0%    0.0%    0.0%    0.0%    0.0%    0.0%    0.0%    0.0%    0.0%    0.0%    0.0%    0.0%    0.0%    0.0%    0.0%    0.0%    0.0%    0.0%    0.0%    0.0%    0.0%    0.0%    0.0%    0.0%    0.0%    0.0%    0.0%    0.0%    0.0%    0.0%    0.0%    0.0%    0.0%    0.0%    0.0%    0.0%    0.0%    0.0%    0.0%    0.0%    0.0%    0.0%    0.0%    0.0%    0.0%    0.0%    0.0%    0.0%    0.0%    0.0%    0.0%    0.0%    0.0%    0.0%    0.0%    0.0%    0.0%    0.0%    0.0%    0.0%    0.0%    0.0%    0.0%    0.0%    0.0%    0.0%    0.0%    0.0%    0.0%    0.0%    0.0%    0.0%    0.0%    0.0%    0.0%    0.0%    0.0%    0.0%    0.0%    0.0%    0.0%    0.0%    0.0%    0.0%    0.0%    0.0%    0.0%    0.0%    0.0%    0.0%    0.0%    0.0%    0.0%    0.0%    0.0%    0.0%    0.0%    0.0%    0.0%    0.0%    0.0%    0.0%    0.0%    0.0%    0.0%    0.0%    0.0%    0.0%    0.0%    0.0%    0.0%    0.0%    0.0%    0.0%    0.0%    0.0%    0.0%    0.0%    0.0%    0.0%    0.0%    0.0%    0.0%    0.0%    0.0%    0.0%    0.0%    0.0%    0.0%    0.0%    0.0%    0.0%    0.0%    0.0%    0.0%    0.0%    0.0%    0.0%    0.0%    0.0%    0.0%    0.0%    0.0%    0.0%    0.0%    0.0%    0.0%    0.0%    0.0%    0.0%    0.0%    0.0%    0.0%    0.0%    0.0%    0.0%    0.0%    0.0%    0.0%    0.0%    0.0%    0.0%    0.0%    0.0%    0.0%    0.0%    0.0%    0.0%    0.0%    0.0%    0.0%    0.0%    0.0%    0.0%    0.0%    0.0%    0.0%    0.0%    0.0%    0.0%    0.0%    0.0%    0.0%    0.0%    0.0%    0.0%    0.0%    0.0%    0.0%    0.0%    0.0%    0.0%    0.0%    0.0%    0.0%    0.0%    0.0%    0.0%    0.0%    0.0%    0.0%    0.0%    0.0%    0.0%    0.0%    0.0%    0.0%    0.0%    0.0%    0.0%    0.0%    0.0%        k__Bacteria;p__Nitrospirae;c__Nitrospira (class); o__Nitrospirales     714    0.0%    0.0%    0.0%    0.0%    0.0%    0.0%    0.0%    0.0%    0.0%    0.0%    0.0%    0.0%    0.0%    0.0%    0.0%    0.0%    0.0%    0.0%    0.0%    0.0%    0.0%    0.0%    0.0%    0.0%    0.0%    0.0%    0.0%    0.0%    0.0%    0.0%    0.0%    0.0%    0.0%    0.0%    0.0%    0.0%    0.0%    0.0%    0.0%    0.0%    0.0%    0.0%    0.0%    0.0%    0.0%    0.0%    0.0%    0.0%    0.0%    0.0%    0.0%    0.0%    0.0%    0.0%    0.0%    0.0%    0.0%    0.0%    0.0%    0.0%    0.0%    0.0%    0.0%    0.0%    0.0%    0.0%    0.0%    0.0%    0.0%    0.0%    0.0%    0.0%    0.0%    0.0%    0.0%    0.0%    0.0%    0.0%    0.0%    0.0%    0.0%    0.0%    0.0%    0.0%    0.0%    0.0%    0.0%    0.0%    0.0%    0.0%    0.0%    0.0%    0.0%    0.0%    0.0%    0.0%    0.0%    0.0%    0.0%    0.0%    0.0%    0.0%    0.0%    0.0%    0.0%    0.0%    0.0%    0.0%    0.0%    0.0%    0.0%    0.0%    0.0%    0.0%    0.0%    0.0%    0.0%    0.0%    0.0%    0.0%    0.0%    0.0%    0.0%    0.0%    0.0%    0.0%    0.0%    0.0%    0.0%    0.0%    0.0%    0.0%    0.0%    0.0%    0.0%    0.0%    0.0%    0.0%    0.0%    0.0%    0.0%    0.0%    0.0%    0.0%    0.0%    0.0%    0.0%    0.0%    0.0%    0.0%    0.0%    0.0%    0.0%    0.0%    0.0%    0.0%    0.0%    0.0%    0.0%    0.0%    0.0%    0.0%    0.0%    0.0%    0.0%    0.0%    0.0%    0.0%    0.0%    0.0%    0.0%    0.0%    0.0%    0.0%    0.0%    0.0%    0.0%    0.0%    0.0%    0.0%    0.0%    0.0%    0.0%    0.0%    0.0%    0.0%    0.0%    0.0%    0.0%    0.0%    0.0%    0.0%    0.0%    0.0%    0.0%    0.0%    0.0%    0.0%    0.0%    0.0%    0.0%    0.0%    0.0%    0.0%    0.0%    0.0%    0.0%    0.0%    0.0%    0.0%    0.0%    0.0%    0.0%    0.0%    0.0%    0.0%    0.0%    0.0%    0.0%    0.0%    0.0%    0.0%    0.0%    0.0%    0.0%    0.0%    0.0%    0.0%    0.0%    0.0%    0.0%    0.0%    0.0%    0.0%    0.0%    0.0%    0.0%    0.0%    0.0%    0.0%    0.0%    0.0%    0.0%    0.0%    0.0%    0.0%    0.0%    0.0%    0.0%    0.0%    0.0%    0.0%    0.0%    0.0%    0.0%    0.0%    0.0%    0.0%    0.0%    0.0%    0.0%    0.0%    0.0%    0.0%    0.0%    0.0%    0.0%    0.0%    0.0%    0.0%    0.0%    0.0%    0.1%    0.0%    0.0%    0.0%    0.0%    0.0%    0.0%    0.0%    0.0%    0.0%    0.0%    0.0%    0.0%    0.0%    0.0%    0.1%    0.0%    0.0%    0.0%    0.0%    0.0%    0.0%    0.0%    0.0%    0.0%    0.0%    0.0%    0.0%    0.0%    0.0%    0.0%    0.0%    0.0%    0.0%    0.0%    0.0%    0.0%    0.0%    0.0%    0.0%    0.0%    0.0%    0.0%    0.0%    0.0%    0.0%    0.0%    0.0%    0.0%    0.0%    0.0%    0.0%    0.0%    0.0%    0.0%    0.0%    0.0%    0.0%    0.0%    0.0%    0.0%    0.0%    0.0%    0.0%    0.0%    0.0%    0.0%    0.0%    0.0%    0.0%    0.0%    0.0%    0.0%    0.0%    0.0%    0.0%    0.0%    0.0%    0.0%    0.0%    0.0%    0.0%    0.0%    0.0%    0.0%    0.0%        k__Bacteria;p__OP10;c__5B-18; o__      41    0.0%    0.0%    0.0%    0.0%    0.0%    0.0%    0.0%    0.0%    0.0%    0.0%    0.0%    0.0%    0.0%    0.0%    0.0%    0.0%    0.0%    0.0%    0.0%    0.0%    0.0%    0.0%    0.0%    0.0%    0.0%    0.0%    0.0%    0.0%    0.0%    0.0%    0.0%    0.0%    0.0%    0.0%    0.0%    0.0%    0.0%    0.0%    0.0%    0.0%    0.0%    0.0%    0.0%    0.0%    0.0%    0.0%    0.0%    0.0%    0.0%    0.0%    0.0%    0.0%    0.0%    0.0%    0.0%    0.0%    0.0%    0.0%    0.0%    0.0%    0.0%    0.0%    0.0%    0.0%    0.0%    0.0%    0.0%    0.0%    0.0%    0.0%    0.0%    0.0%    0.0%    0.0%    0.0%    0.0%    0.0%    0.0%    0.0%    0.0%    0.0%    0.0%    0.0%    0.0%    0.0%    0.0%    0.0%    0.0%    0.0%    0.0%    0.0%    0.0%    0.0%    0.0%    0.0%    0.0%    0.0%    0.0%    0.0%    0.0%    0.0%    0.0%    0.0%    0.0%    0.0%    0.0%    0.0%    0.0%    0.0%    0.0%    0.0%    0.0%    0.0%    0.0%    0.0%    0.0%    0.0%    0.0%    0.0%    0.0%    0.0%    0.0%    0.0%    0.0%    0.0%    0.0%    0.0%    0.0%    0.0%    0.0%    0.0%    0.0%    0.0%    0.0%    0.0%    0.0%    0.0%    0.0%    0.0%    0.0%    0.0%    0.0%    0.0%    0.0%    0.0%    0.0%    0.0%    0.0%    0.0%    0.0%    0.0%    0.0%    0.0%    0.0%    0.0%    0.0%    0.0%    0.0%    0.0%    0.0%    0.0%    0.0%    0.0%    0.0%    0.0%    0.0%    0.0%    0.0%    0.0%    0.0%    0.0%    0.0%    0.0%    0.0%    0.0%    0.0%    0.0%    0.0%    0.0%    0.0%    0.0%    0.0%    0.0%    0.0%    0.0%    0.0%    0.0%    0.0%    0.0%    0.0%    0.0%    0.0%    0.0%    0.0%    0.0%    0.0%    0.0%    0.0%    0.0%    0.0%    0.0%    0.0%    0.0%    0.0%    0.0%    0.0%    0.0%    0.0%    0.0%    0.0%    0.0%    0.0%    0.0%    0.0%    0.0%    0.0%    0.0%    0.0%    0.0%    0.0%    0.0%    0.0%    0.0%    0.0%    0.0%    0.0%    0.0%    0.0%    0.0%    0.0%    0.0%    0.0%    0.0%    0.0%    0.0%    0.0%    0.0%    0.0%    0.0%    0.0%    0.0%    0.0%    0.0%    0.0%    0.0%    0.0%    0.0%    0.0%    0.0%    0.0%    0.0%    0.0%    0.0%    0.0%    0.0%    0.0%    0.0%    0.0%    0.0%    0.0%    0.0%    0.0%    0.0%    0.0%    0.0%    0.0%    0.0%    0.0%    0.0%    0.0%    0.0%    0.0%    0.0%    0.0%    0.0%    0.0%    0.0%    0.0%    0.0%    0.0%    0.0%    0.0%    0.0%    0.0%    0.0%    0.0%    0.0%    0.0%    0.0%    0.0%    0.0%    0.0%    0.0%    0.0%    0.0%    0.0%    0.0%    0.0%    0.0%    0.0%    0.0%    0.0%    0.0%    0.0%    0.0%    0.0%    0.0%    0.0%    0.0%    0.0%    0.0%    0.0%    0.0%    0.0%    0.0%    0.0%    0.0%    0.0%    0.0%    0.0%    0.0%    0.0%    0.0%    0.0%    0.0%    0.0%    0.0%    0.0%    0.0%    0.0%    0.0%    0.0%    0.0%    0.0%    0.0%    0.0%    0.0%    0.0%    0.0%    0.0%    0.0%    0.0%    0.0%    0.0%    0.0%    0.0%    0.0%    0.0%    0.0%    0.0%    0.0%    0.0%    0.0%    0.0%    0.0%    0.0%    0.0%    0.0%        k__Bacteria;p__OP10;c__; o__       1    0.0%    0.0%    0.0%    0.0%    0.0%    0.0%    0.0%    0.0%    0.0%    0.0%    0.0%    0.0%    0.0%    0.0%    0.0%    0.0%    0.0%    0.0%    0.0%    0.0%    0.0%    0.0%    0.0%    0.0%    0.0%    0.0%    0.0%    0.0%    0.0%    0.0%    0.0%    0.0%    0.0%    0.0%    0.0%    0.0%    0.0%    0.0%    0.0%    0.0%    0.0%    0.0%    0.0%    0.0%    0.0%    0.0%    0.0%    0.0%    0.0%    0.0%    0.0%    0.0%    0.0%    0.0%    0.0%    0.0%    0.0%    0.0%    0.0%    0.0%    0.0%    0.0%    0.0%    0.0%    0.0%    0.0%    0.0%    0.0%    0.0%    0.0%    0.0%    0.0%    0.0%    0.0%    0.0%    0.0%    0.0%    0.0%    0.0%    0.0%    0.0%    0.0%    0.0%    0.0%    0.0%    0.0%    0.0%    0.0%    0.0%    0.0%    0.0%    0.0%    0.0%    0.0%    0.0%    0.0%    0.0%    0.0%    0.0%    0.0%    0.0%    0.0%    0.0%    0.0%    0.0%    0.0%    0.0%    0.0%    0.0%    0.0%    0.0%    0.0%    0.0%    0.0%    0.0%    0.0%    0.0%    0.0%    0.0%    0.0%    0.0%    0.0%    0.0%    0.0%    0.0%    0.0%    0.0%    0.0%    0.0%    0.0%    0.0%    0.0%    0.0%    0.0%    0.0%    0.0%    0.0%    0.0%    0.0%    0.0%    0.0%    0.0%    0.0%    0.0%    0.0%    0.0%    0.0%    0.0%    0.0%    0.0%    0.0%    0.0%    0.0%    0.0%    0.0%    0.0%    0.0%    0.0%    0.0%    0.0%    0.0%    0.0%    0.0%    0.0%    0.0%    0.0%    0.0%    0.0%    0.0%    0.0%    0.0%    0.0%    0.0%    0.0%    0.0%    0.0%    0.0%    0.0%    0.0%    0.0%    0.0%    0.0%    0.0%    0.0%    0.0%    0.0%    0.0%    0.0%    0.0%    0.0%    0.0%    0.0%    0.0%    0.0%    0.0%    0.0%    0.0%    0.0%    0.0%    0.0%    0.0%    0.0%    0.0%    0.0%    0.0%    0.0%    0.0%    0.0%    0.0%    0.0%    0.0%    0.0%    0.0%    0.0%    0.0%    0.0%    0.0%    0.0%    0.0%    0.0%    0.0%    0.0%    0.0%    0.0%    0.0%    0.0%    0.0%    0.0%    0.0%    0.0%    0.0%    0.0%    0.0%    0.0%    0.0%    0.0%    0.0%    0.0%    0.0%    0.0%    0.0%    0.0%    0.0%    0.0%    0.0%    0.0%    0.0%    0.0%    0.0%    0.0%    0.0%    0.0%    0.0%    0.0%    0.0%    0.0%    0.0%    0.0%    0.0%    0.0%    0.0%    0.0%    0.0%    0.0%    0.0%    0.0%    0.0%    0.0%    0.0%    0.0%    0.0%    0.0%    0.0%    0.0%    0.0%    0.0%    0.0%    0.0%    0.0%    0.0%    0.0%    0.0%    0.0%    0.0%    0.0%    0.0%    0.0%    0.0%    0.0%    0.0%    0.0%    0.0%    0.0%    0.0%    0.0%    0.0%    0.0%    0.0%    0.0%    0.0%    0.0%    0.0%    0.0%    0.0%    0.0%    0.0%    0.0%    0.0%    0.0%    0.0%    0.0%    0.0%    0.0%    0.0%    0.0%    0.0%    0.0%    0.0%    0.0%    0.0%    0.0%    0.0%    0.0%    0.0%    0.0%    0.0%    0.0%    0.0%    0.0%    0.0%    0.0%    0.0%    0.0%    0.0%    0.0%    0.0%    0.0%    0.0%    0.0%    0.0%    0.0%    0.0%    0.0%    0.0%    0.0%    0.0%    0.0%    0.0%    0.0%    0.0%    0.0%    0.0%    0.0%    0.0%    0.0%    0.0%    0.0%    0.0%        k__Bacteria;p__OP10;c__CH21; o__      27    0.0%    0.0%    0.0%    0.0%    0.0%    0.0%    0.0%    0.0%    0.0%    0.0%    0.0%    0.0%    0.0%    0.0%    0.0%    0.0%    0.0%    0.0%    0.0%    0.0%    0.0%    0.0%    0.0%    0.0%    0.0%    0.0%    0.0%    0.0%    0.0%    0.0%    0.0%    0.0%    0.0%    0.0%    0.0%    0.0%    0.0%    0.0%    0.0%    0.0%    0.0%    0.0%    0.0%    0.0%    0.0%    0.0%    0.0%    0.0%    0.0%    0.0%    0.0%    0.0%    0.0%    0.0%    0.0%    0.0%    0.0%    0.0%    0.0%    0.0%    0.0%    0.0%    0.0%    0.0%    0.0%    0.0%    0.0%    0.0%    0.0%    0.0%    0.0%    0.0%    0.0%    0.0%    0.0%    0.0%    0.0%    0.0%    0.0%    0.0%    0.0%    0.0%    0.0%    0.0%    0.0%    0.0%    0.0%    0.0%    0.0%    0.0%    0.0%    0.0%    0.0%    0.0%    0.0%    0.0%    0.0%    0.0%    0.0%    0.0%    0.0%    0.0%    0.0%    0.0%    0.0%    0.0%    0.0%    0.0%    0.0%    0.0%    0.0%    0.0%    0.0%    0.0%    0.0%    0.0%    0.0%    0.0%    0.0%    0.0%    0.0%    0.0%    0.0%    0.0%    0.0%    0.0%    0.0%    0.0%    0.0%    0.0%    0.0%    0.0%    0.0%    0.0%    0.0%    0.0%    0.0%    0.0%    0.0%    0.0%    0.0%    0.0%    0.0%    0.0%    0.0%    0.0%    0.0%    0.0%    0.0%    0.0%    0.0%    0.0%    0.0%    0.0%    0.0%    0.0%    0.0%    0.0%    0.0%    0.0%    0.0%    0.0%    0.0%    0.0%    0.0%    0.0%    0.0%    0.0%    0.0%    0.0%    0.0%    0.0%    0.0%    0.0%    0.0%    0.0%    0.0%    0.0%    0.0%    0.0%    0.0%    0.0%    0.0%    0.0%    0.0%    0.0%    0.0%    0.0%    0.0%    0.0%    0.0%    0.0%    0.0%    0.0%    0.0%    0.0%    0.0%    0.0%    0.0%    0.0%    0.0%    0.0%    0.0%    0.0%    0.0%    0.0%    0.0%    0.0%    0.0%    0.0%    0.0%    0.0%    0.0%    0.0%    0.0%    0.0%    0.0%    0.0%    0.0%    0.0%    0.0%    0.0%    0.0%    0.0%    0.0%    0.0%    0.0%    0.0%    0.0%    0.0%    0.0%    0.0%    0.0%    0.0%    0.0%    0.0%    0.0%    0.0%    0.0%    0.0%    0.0%    0.0%    0.0%    0.0%    0.0%    0.0%    0.0%    0.0%    0.0%    0.0%    0.0%    0.0%    0.0%    0.0%    0.0%    0.0%    0.0%    0.0%    0.0%    0.0%    0.0%    0.0%    0.0%    0.0%    0.0%    0.0%    0.0%    0.0%    0.0%    0.0%    0.0%    0.0%    0.0%    0.0%    0.0%    0.0%    0.0%    0.0%    0.0%    0.0%    0.0%    0.0%    0.0%    0.0%    0.0%    0.0%    0.0%    0.0%    0.0%    0.0%    0.0%    0.0%    0.0%    0.0%    0.0%    0.0%    0.0%    0.0%    0.0%    0.0%    0.0%    0.0%    0.0%    0.0%    0.0%    0.0%    0.0%    0.0%    0.0%    0.0%    0.0%    0.0%    0.0%    0.0%    0.0%    0.0%    0.0%    0.0%    0.0%    0.0%    0.0%    0.0%    0.0%    0.0%    0.0%    0.0%    0.0%    0.0%    0.0%    0.0%    0.0%    0.0%    0.0%    0.0%    0.0%    0.0%    0.0%    0.0%    0.0%    0.0%    0.0%    0.0%    0.0%    0.0%    0.0%    0.0%    0.0%    0.0%    0.0%    0.0%    0.0%    0.0%    0.0%    0.0%    0.0%    0.0%    0.0%    0.0%        k__Bacteria;p__OP10;c__CL500-48; o__     299    0.0%    0.0%    0.0%    0.0%    0.0%    0.0%    0.0%    0.0%    0.0%    0.0%    0.0%    0.0%    0.0%    0.0%    0.0%    0.0%    0.0%    0.0%    0.0%    0.0%    0.0%    0.0%    0.0%    0.0%    0.0%    0.0%    0.0%    0.0%    0.0%    0.0%    0.0%    0.0%    0.0%    0.0%    0.0%    0.0%    0.0%    0.0%    0.0%    0.0%    0.0%    0.0%    0.0%    0.0%    0.0%    0.0%    0.0%    0.0%    0.0%    0.0%    0.0%    0.0%    0.0%    0.0%    0.0%    0.0%    0.0%    0.0%    0.0%    0.0%    0.0%    0.0%    0.0%    0.0%    0.0%    0.0%    0.0%    0.0%    0.0%    0.0%    0.0%    0.0%    0.0%    0.0%    0.0%    0.0%    0.0%    0.0%    0.0%    0.0%    0.0%    0.0%    0.0%    0.0%    0.0%    0.0%    0.0%    0.0%    0.0%    0.0%    0.0%    0.0%    0.0%    0.0%    0.0%    0.0%    0.0%    0.0%    0.0%    0.0%    0.0%    0.0%    0.0%    0.0%    0.0%    0.0%    0.0%    0.0%    0.0%    0.0%    0.0%    0.0%    0.0%    0.0%    0.0%    0.0%    0.0%    0.0%    0.0%    0.0%    0.0%    0.0%    0.0%    0.0%    0.0%    0.0%    0.0%    0.0%    0.0%    0.0%    0.0%    0.0%    0.0%    0.0%    0.0%    0.0%    0.0%    0.0%    0.0%    0.0%    0.0%    0.0%    0.0%    0.0%    0.0%    0.0%    0.0%    0.0%    0.0%    0.0%    0.0%    0.0%    0.0%    0.0%    0.0%    0.0%    0.0%    0.0%    0.0%    0.0%    0.0%    0.0%    0.0%    0.0%    0.0%    0.0%    0.0%    0.0%    0.0%    0.0%    0.0%    0.0%    0.0%    0.0%    0.0%    0.0%    0.0%    0.0%    0.0%    0.0%    0.0%    0.0%    0.0%    0.0%    0.0%    0.0%    0.0%    0.0%    0.0%    0.0%    0.0%    0.0%    0.0%    0.0%    0.0%    0.0%    0.0%    0.0%    0.0%    0.0%    0.0%    0.0%    0.0%    0.0%    0.0%    0.0%    0.0%    0.0%    0.0%    0.0%    0.0%    0.0%    0.0%    0.0%    0.0%    0.0%    0.0%    0.0%    0.0%    0.0%    0.0%    0.0%    0.0%    0.0%    0.0%    0.0%    0.0%    0.0%    0.0%    0.0%    0.0%    0.0%    0.0%    0.0%    0.0%    0.0%    0.0%    0.0%    0.0%    0.0%    0.0%    0.0%    0.0%    0.0%    0.0%    0.0%    0.0%    0.0%    0.0%    0.0%    0.0%    0.0%    0.0%    0.0%    0.0%    0.0%    0.0%    0.0%    0.0%    0.0%    0.0%    0.0%    0.0%    0.0%    0.0%    0.0%    0.0%    0.0%    0.0%    0.0%    0.0%    0.0%    0.0%    0.0%    0.0%    0.0%    0.0%    0.0%    0.0%    0.0%    0.0%    0.0%    0.0%    0.0%    0.0%    0.0%    0.0%    0.0%    0.0%    0.0%    0.0%    0.0%    0.0%    0.0%    0.0%    0.0%    0.0%    0.0%    0.0%    0.0%    0.0%    0.0%    0.0%    0.0%    0.0%    0.0%    0.0%    0.0%    0.0%    0.0%    0.0%    0.0%    0.0%    0.0%    0.0%    0.0%    0.0%    0.0%    0.0%    0.0%    0.0%    0.0%    0.0%    0.0%    0.0%    0.0%    0.0%    0.0%    0.0%    0.0%    0.0%    0.0%    0.0%    0.0%    0.0%    0.0%    0.0%    0.0%    0.0%    0.0%    0.0%    0.0%    0.0%    0.0%    0.0%    0.0%    0.0%    0.0%    0.0%    0.0%    0.0%    0.0%    0.0%    0.0%    0.0%    0.0%    0.0%    0.0%        k__Bacteria;p__OP10;c__OS-L; o__      89    0.0%    0.0%    0.0%    0.0%    0.0%    0.0%    0.0%    0.0%    0.0%    0.0%    0.0%    0.0%    0.0%    0.0%    0.0%    0.0%    0.0%    0.0%    0.0%    0.0%    0.0%    0.0%    0.0%    0.0%    0.0%    0.0%    0.0%    0.0%    0.0%    0.0%    0.0%    0.0%    0.0%    0.0%    0.0%    0.0%    0.0%    0.0%    0.0%    0.0%    0.0%    0.0%    0.0%    0.0%    0.0%    0.0%    0.0%    0.0%    0.0%    0.0%    0.0%    0.0%    0.0%    0.0%    0.0%    0.0%    0.0%    0.0%    0.0%    0.0%    0.0%    0.0%    0.0%    0.0%    0.0%    0.0%    0.0%    0.0%    0.0%    0.0%    0.0%    0.0%    0.0%    0.0%    0.0%    0.0%    0.0%    0.0%    0.0%    0.0%    0.0%    0.0%    0.0%    0.0%    0.0%    0.0%    0.0%    0.0%    0.0%    0.0%    0.0%    0.0%    0.0%    0.0%    0.0%    0.0%    0.0%    0.0%    0.0%    0.0%    0.0%    0.0%    0.0%    0.0%    0.0%    0.0%    0.0%    0.0%    0.0%    0.0%    0.0%    0.0%    0.0%    0.0%    0.0%    0.0%    0.0%    0.0%    0.0%    0.0%    0.0%    0.0%    0.0%    0.0%    0.0%    0.0%    0.0%    0.0%    0.0%    0.0%    0.0%    0.0%    0.0%    0.0%    0.0%    0.0%    0.0%    0.0%    0.0%    0.0%    0.0%    0.0%    0.0%    0.0%    0.0%    0.0%    0.0%    0.0%    0.0%    0.0%    0.0%    0.0%    0.0%    0.0%    0.0%    0.0%    0.0%    0.0%    0.0%    0.0%    0.0%    0.0%    0.0%    0.0%    0.0%    0.0%    0.0%    0.0%    0.0%    0.0%    0.0%    0.0%    0.0%    0.0%    0.0%    0.0%    0.0%    0.0%    0.0%    0.0%    0.0%    0.0%    0.0%    0.0%    0.0%    0.0%    0.0%    0.0%    0.0%    0.0%    0.0%    0.0%    0.0%    0.0%    0.0%    0.0%    0.0%    0.0%    0.0%    0.0%    0.0%    0.0%    0.0%    0.0%    0.0%    0.0%    0.0%    0.0%    0.0%    0.0%    0.0%    0.0%    0.0%    0.0%    0.0%    0.0%    0.0%    0.0%    0.0%    0.0%    0.0%    0.0%    0.0%    0.0%    0.0%    0.0%    0.0%    0.0%    0.0%    0.0%    0.0%    0.0%    0.0%    0.0%    0.0%    0.0%    0.0%    0.0%    0.0%    0.0%    0.0%    0.0%    0.0%    0.0%    0.0%    0.0%    0.0%    0.0%    0.0%    0.0%    0.0%    0.0%    0.0%    0.0%    0.0%    0.0%    0.0%    0.0%    0.0%    0.0%    0.0%    0.0%    0.0%    0.0%    0.0%    0.0%    0.0%    0.0%    0.0%    0.0%    0.0%    0.0%    0.0%    0.0%    0.0%    0.0%    0.0%    0.0%    0.0%    0.0%    0.0%    0.0%    0.0%    0.0%    0.0%    0.0%    0.0%    0.0%    0.0%    0.0%    0.0%    0.0%    0.0%    0.0%    0.0%    0.0%    0.0%    0.0%    0.0%    0.0%    0.0%    0.0%    0.0%    0.0%    0.0%    0.0%    0.0%    0.0%    0.0%    0.0%    0.0%    0.0%    0.0%    0.0%    0.0%    0.0%    0.0%    0.0%    0.0%    0.0%    0.0%    0.0%    0.0%    0.0%    0.0%    0.0%    0.0%    0.0%    0.0%    0.0%    0.0%    0.0%    0.0%    0.0%    0.0%    0.0%    0.0%    0.0%    0.0%    0.0%    0.0%    0.0%    0.0%    0.0%    0.0%    0.0%    0.0%    0.0%    0.0%    0.0%    0.0%    0.0%    0.0%    0.0%    0.0%    0.0%    0.0%    0.0%        k__Bacteria;p__OP10;c__S1a-1H; o__      23    0.0%    0.0%    0.0%    0.0%    0.0%    0.0%    0.0%    0.0%    0.0%    0.0%    0.0%    0.0%    0.0%    0.0%    0.0%    0.0%    0.0%    0.0%    0.0%    0.0%    0.0%    0.0%    0.0%    0.0%    0.0%    0.0%    0.0%    0.0%    0.0%    0.0%    0.0%    0.0%    0.0%    0.0%    0.0%    0.0%    0.0%    0.0%    0.0%    0.0%    0.0%    0.0%    0.0%    0.0%    0.0%    0.0%    0.0%    0.0%    0.0%    0.0%    0.0%    0.0%    0.0%    0.0%    0.0%    0.0%    0.0%    0.0%    0.0%    0.0%    0.0%    0.0%    0.0%    0.0%    0.0%    0.0%    0.0%    0.0%    0.0%    0.0%    0.0%    0.0%    0.0%    0.0%    0.0%    0.0%    0.0%    0.0%    0.0%    0.0%    0.0%    0.0%    0.0%    0.0%    0.0%    0.0%    0.0%    0.0%    0.0%    0.0%    0.0%    0.0%    0.0%    0.0%    0.0%    0.0%    0.0%    0.0%    0.0%    0.0%    0.0%    0.0%    0.0%    0.0%    0.0%    0.0%    0.0%    0.0%    0.0%    0.0%    0.0%    0.0%    0.0%    0.0%    0.0%    0.0%    0.0%    0.0%    0.0%    0.0%    0.0%    0.0%    0.0%    0.0%    0.0%    0.0%    0.0%    0.0%    0.0%    0.0%    0.0%    0.0%    0.0%    0.0%    0.0%    0.0%    0.0%    0.0%    0.0%    0.0%    0.0%    0.0%    0.0%    0.0%    0.0%    0.0%    0.0%    0.0%    0.0%    0.0%    0.0%    0.0%    0.0%    0.0%    0.0%    0.0%    0.0%    0.0%    0.0%    0.0%    0.0%    0.0%    0.0%    0.0%    0.0%    0.0%    0.0%    0.0%    0.0%    0.0%    0.0%    0.0%    0.0%    0.0%    0.0%    0.0%    0.0%    0.0%    0.0%    0.0%    0.0%    0.0%    0.0%    0.0%    0.0%    0.0%    0.0%    0.0%    0.0%    0.0%    0.0%    0.0%    0.0%    0.0%    0.0%    0.0%    0.0%    0.0%    0.0%    0.0%    0.0%    0.0%    0.0%    0.0%    0.0%    0.0%    0.0%    0.0%    0.0%    0.0%    0.0%    0.0%    0.0%    0.0%    0.0%    0.0%    0.0%    0.0%    0.0%    0.0%    0.0%    0.0%    0.0%    0.0%    0.0%    0.0%    0.0%    0.0%    0.0%    0.0%    0.0%    0.0%    0.0%    0.0%    0.0%    0.0%    0.0%    0.0%    0.0%    0.0%    0.0%    0.0%    0.0%    0.0%    0.0%    0.0%    0.0%    0.0%    0.0%    0.0%    0.0%    0.0%    0.0%    0.0%    0.0%    0.0%    0.0%    0.0%    0.0%    0.0%    0.0%    0.0%    0.0%    0.0%    0.0%    0.0%    0.0%    0.0%    0.0%    0.0%    0.0%    0.0%    0.0%    0.0%    0.0%    0.0%    0.0%    0.0%    0.0%    0.0%    0.0%    0.0%    0.0%    0.0%    0.0%    0.0%    0.0%    0.0%    0.0%    0.0%    0.0%    0.0%    0.0%    0.0%    0.0%    0.0%    0.0%    0.0%    0.0%    0.0%    0.0%    0.0%    0.0%    0.0%    0.0%    0.0%    0.0%    0.0%    0.0%    0.0%    0.0%    0.0%    0.0%    0.0%    0.0%    0.0%    0.0%    0.0%    0.0%    0.0%    0.0%    0.0%    0.0%    0.0%    0.0%    0.0%    0.0%    0.0%    0.0%    0.0%    0.0%    0.0%    0.0%    0.0%    0.0%    0.0%    0.0%    0.0%    0.0%    0.0%    0.0%    0.0%    0.0%    0.0%    0.0%    0.0%    0.0%    0.0%    0.0%    0.0%    0.0%    0.0%    0.0%    0.0%    0.0%    0.0%    0.0%    0.0%        k__Bacteria;p__OP10;c__SJA-176; o__       1    0.0%    0.0%    0.0%    0.0%    0.0%    0.0%    0.0%    0.0%    0.0%    0.0%    0.0%    0.0%    0.0%    0.0%    0.0%    0.0%    0.0%    0.0%    0.0%    0.0%    0.0%    0.0%    0.0%    0.0%    0.0%    0.0%    0.0%    0.0%    0.0%    0.0%    0.0%    0.0%    0.0%    0.0%    0.0%    0.0%    0.0%    0.0%    0.0%    0.0%    0.0%    0.0%    0.0%    0.0%    0.0%    0.0%    0.0%    0.0%    0.0%    0.0%    0.0%    0.0%    0.0%    0.0%    0.0%    0.0%    0.0%    0.0%    0.0%    0.0%    0.0%    0.0%    0.0%    0.0%    0.0%    0.0%    0.0%    0.0%    0.0%    0.0%    0.0%    0.0%    0.0%    0.0%    0.0%    0.0%    0.0%    0.0%    0.0%    0.0%    0.0%    0.0%    0.0%    0.0%    0.0%    0.0%    0.0%    0.0%    0.0%    0.0%    0.0%    0.0%    0.0%    0.0%    0.0%    0.0%    0.0%    0.0%    0.0%    0.0%    0.0%    0.0%    0.0%    0.0%    0.0%    0.0%    0.0%    0.0%    0.0%    0.0%    0.0%    0.0%    0.0%    0.0%    0.0%    0.0%    0.0%    0.0%    0.0%    0.0%    0.0%    0.0%    0.0%    0.0%    0.0%    0.0%    0.0%    0.0%    0.0%    0.0%    0.0%    0.0%    0.0%    0.0%    0.0%    0.0%    0.0%    0.0%    0.0%    0.0%    0.0%    0.0%    0.0%    0.0%    0.0%    0.0%    0.0%    0.0%    0.0%    0.0%    0.0%    0.0%    0.0%    0.0%    0.0%    0.0%    0.0%    0.0%    0.0%    0.0%    0.0%    0.0%    0.0%    0.0%    0.0%    0.0%    0.0%    0.0%    0.0%    0.0%    0.0%    0.0%    0.0%    0.0%    0.0%    0.0%    0.0%    0.0%    0.0%    0.0%    0.0%    0.0%    0.0%    0.0%    0.0%    0.0%    0.0%    0.0%    0.0%    0.0%    0.0%    0.0%    0.0%    0.0%    0.0%    0.0%    0.0%    0.0%    0.0%    0.0%    0.0%    0.0%    0.0%    0.0%    0.0%    0.0%    0.0%    0.0%    0.0%    0.0%    0.0%    0.0%    0.0%    0.0%    0.0%    0.0%    0.0%    0.0%    0.0%    0.0%    0.0%    0.0%    0.0%    0.0%    0.0%    0.0%    0.0%    0.0%    0.0%    0.0%    0.0%    0.0%    0.0%    0.0%    0.0%    0.0%    0.0%    0.0%    0.0%    0.0%    0.0%    0.0%    0.0%    0.0%    0.0%    0.0%    0.0%    0.0%    0.0%    0.0%    0.0%    0.0%    0.0%    0.0%    0.0%    0.0%    0.0%    0.0%    0.0%    0.0%    0.0%    0.0%    0.0%    0.0%    0.0%    0.0%    0.0%    0.0%    0.0%    0.0%    0.0%    0.0%    0.0%    0.0%    0.0%    0.0%    0.0%    0.0%    0.0%    0.0%    0.0%    0.0%    0.0%    0.0%    0.0%    0.0%    0.0%    0.0%    0.0%    0.0%    0.0%    0.0%    0.0%    0.0%    0.0%    0.0%    0.0%    0.0%    0.0%    0.0%    0.0%    0.0%    0.0%    0.0%    0.0%    0.0%    0.0%    0.0%    0.0%    0.0%    0.0%    0.0%    0.0%    0.0%    0.0%    0.0%    0.0%    0.0%    0.0%    0.0%    0.0%    0.0%    0.0%    0.0%    0.0%    0.0%    0.0%    0.0%    0.0%    0.0%    0.0%    0.0%    0.0%    0.0%    0.0%    0.0%    0.0%    0.0%    0.0%    0.0%    0.0%    0.0%    0.0%    0.0%    0.0%    0.0%    0.0%    0.0%    0.0%    0.0%    0.0%    0.0%    0.0%    0.0%    0.0%    0.0%    0.0%    0.0%        k__Bacteria;p__OP10;c__SJA-22; o__       3    0.0%    0.0%    0.0%    0.0%    0.0%    0.0%    0.0%    0.0%    0.0%    0.0%    0.0%    0.0%    0.0%    0.0%    0.0%    0.0%    0.0%    0.0%    0.0%    0.0%    0.0%    0.0%    0.0%    0.0%    0.0%    0.0%    0.0%    0.0%    0.0%    0.0%    0.0%    0.0%    0.0%    0.0%    0.0%    0.0%    0.0%    0.0%    0.0%    0.0%    0.0%    0.0%    0.0%    0.0%    0.0%    0.0%    0.0%    0.0%    0.0%    0.0%    0.0%    0.0%    0.0%    0.0%    0.0%    0.0%    0.0%    0.0%    0.0%    0.0%    0.0%    0.0%    0.0%    0.0%    0.0%    0.0%    0.0%    0.0%    0.0%    0.0%    0.0%    0.0%    0.0%    0.0%    0.0%    0.0%    0.0%    0.0%    0.0%    0.0%    0.0%    0.0%    0.0%    0.0%    0.0%    0.0%    0.0%    0.0%    0.0%    0.0%    0.0%    0.0%    0.0%    0.0%    0.0%    0.0%    0.0%    0.0%    0.0%    0.0%    0.0%    0.0%    0.0%    0.0%    0.0%    0.0%    0.0%    0.0%    0.0%    0.0%    0.0%    0.0%    0.0%    0.0%    0.0%    0.0%    0.0%    0.0%    0.0%    0.0%    0.0%    0.0%    0.0%    0.0%    0.0%    0.0%    0.0%    0.0%    0.0%    0.0%    0.0%    0.0%    0.0%    0.0%    0.0%    0.0%    0.0%    0.0%    0.0%    0.0%    0.0%    0.0%    0.0%    0.0%    0.0%    0.0%    0.0%    0.0%    0.0%    0.0%    0.0%    0.0%    0.0%    0.0%    0.0%    0.0%    0.0%    0.0%    0.0%    0.0%    0.0%    0.0%    0.0%    0.0%    0.0%    0.0%    0.0%    0.0%    0.0%    0.0%    0.0%    0.0%    0.0%    0.0%    0.0%    0.0%    0.0%    0.0%    0.0%    0.0%    0.0%    0.0%    0.0%    0.0%    0.0%    0.0%    0.0%    0.0%    0.0%    0.0%    0.0%    0.0%    0.0%    0.0%    0.0%    0.0%    0.0%    0.0%    0.0%    0.0%    0.0%    0.0%    0.0%    0.0%    0.0%    0.0%    0.0%    0.0%    0.0%    0.0%    0.0%    0.0%    0.0%    0.0%    0.0%    0.0%    0.0%    0.0%    0.0%    0.0%    0.0%    0.0%    0.0%    0.0%    0.0%    0.0%    0.0%    0.0%    0.0%    0.0%    0.0%    0.0%    0.0%    0.0%    0.0%    0.0%    0.0%    0.0%    0.0%    0.0%    0.0%    0.0%    0.0%    0.0%    0.0%    0.0%    0.0%    0.0%    0.0%    0.0%    0.0%    0.0%    0.0%    0.0%    0.0%    0.0%    0.0%    0.0%    0.0%    0.0%    0.0%    0.0%    0.0%    0.0%    0.0%    0.0%    0.0%    0.0%    0.0%    0.0%    0.0%    0.0%    0.0%    0.0%    0.0%    0.0%    0.0%    0.0%    0.0%    0.0%    0.0%    0.0%    0.0%    0.0%    0.0%    0.0%    0.0%    0.0%    0.0%    0.0%    0.0%    0.0%    0.0%    0.0%    0.0%    0.0%    0.0%    0.0%    0.0%    0.0%    0.0%    0.0%    0.0%    0.0%    0.0%    0.0%    0.0%    0.0%    0.0%    0.0%    0.0%    0.0%    0.0%    0.0%    0.0%    0.0%    0.0%    0.0%    0.0%    0.0%    0.0%    0.0%    0.0%    0.0%    0.0%    0.0%    0.0%    0.0%    0.0%    0.0%    0.0%    0.0%    0.0%    0.0%    0.0%    0.0%    0.0%    0.0%    0.0%    0.0%    0.0%    0.0%    0.0%    0.0%    0.0%    0.0%    0.0%    0.0%    0.0%    0.0%    0.0%    0.0%    0.0%    0.0%    0.0%    0.0%    0.0%    0.0%        k__Bacteria;p__OP11;c__; o__       2    0.0%    0.0%    0.0%    0.0%    0.0%    0.0%    0.0%    0.0%    0.0%    0.0%    0.0%    0.0%    0.0%    0.0%    0.0%    0.0%    0.0%    0.0%    0.0%    0.0%    0.0%    0.0%    0.0%    0.0%    0.0%    0.0%    0.0%    0.0%    0.0%    0.0%    0.0%    0.0%    0.0%    0.0%    0.0%    0.0%    0.0%    0.0%    0.0%    0.0%    0.0%    0.0%    0.0%    0.0%    0.0%    0.0%    0.0%    0.0%    0.0%    0.0%    0.0%    0.0%    0.0%    0.0%    0.0%    0.0%    0.0%    0.0%    0.0%    0.0%    0.0%    0.0%    0.0%    0.0%    0.0%    0.0%    0.0%    0.0%    0.0%    0.0%    0.0%    0.0%    0.0%    0.0%    0.0%    0.0%    0.0%    0.0%    0.0%    0.0%    0.0%    0.0%    0.0%    0.0%    0.0%    0.0%    0.0%    0.0%    0.0%    0.0%    0.0%    0.0%    0.0%    0.0%    0.0%    0.0%    0.0%    0.0%    0.0%    0.0%    0.0%    0.0%    0.0%    0.0%    0.0%    0.0%    0.0%    0.0%    0.0%    0.0%    0.0%    0.0%    0.0%    0.0%    0.0%    0.0%    0.0%    0.0%    0.0%    0.0%    0.0%    0.0%    0.0%    0.0%    0.0%    0.0%    0.0%    0.0%    0.0%    0.0%    0.0%    0.0%    0.0%    0.0%    0.0%    0.0%    0.0%    0.0%    0.0%    0.0%    0.0%    0.0%    0.0%    0.0%    0.0%    0.0%    0.0%    0.0%    0.0%    0.0%    0.0%    0.0%    0.0%    0.0%    0.0%    0.0%    0.0%    0.0%    0.0%    0.0%    0.0%    0.0%    0.0%    0.0%    0.0%    0.0%    0.0%    0.0%    0.0%    0.0%    0.0%    0.0%    0.0%    0.0%    0.0%    0.0%    0.0%    0.0%    0.0%    0.0%    0.0%    0.0%    0.0%    0.0%    0.0%    0.0%    0.0%    0.0%    0.0%    0.0%    0.0%    0.0%    0.0%    0.0%    0.0%    0.0%    0.0%    0.0%    0.0%    0.0%    0.0%    0.0%    0.0%    0.0%    0.0%    0.0%    0.0%    0.0%    0.0%    0.0%    0.0%    0.0%    0.0%    0.0%    0.0%    0.0%    0.0%    0.0%    0.0%    0.0%    0.0%    0.0%    0.0%    0.0%    0.0%    0.0%    0.0%    0.0%    0.0%    0.0%    0.0%    0.0%    0.0%    0.0%    0.0%    0.0%    0.0%    0.0%    0.0%    0.0%    0.0%    0.0%    0.0%    0.0%    0.0%    0.0%    0.0%    0.0%    0.0%    0.0%    0.0%    0.0%    0.0%    0.0%    0.0%    0.0%    0.0%    0.0%    0.0%    0.0%    0.0%    0.0%    0.0%    0.0%    0.0%    0.0%    0.0%    0.0%    0.0%    0.0%    0.0%    0.0%    0.0%    0.0%    0.0%    0.0%    0.0%    0.0%    0.0%    0.0%    0.0%    0.0%    0.0%    0.0%    0.0%    0.0%    0.0%    0.0%    0.0%    0.0%    0.0%    0.0%    0.0%    0.0%    0.0%    0.0%    0.0%    0.0%    0.0%    0.0%    0.0%    0.0%    0.0%    0.0%    0.0%    0.0%    0.0%    0.0%    0.0%    0.0%    0.0%    0.0%    0.0%    0.0%    0.0%    0.0%    0.0%    0.0%    0.0%    0.0%    0.0%    0.0%    0.0%    0.0%    0.0%    0.0%    0.0%    0.0%    0.0%    0.0%    0.0%    0.0%    0.0%    0.0%    0.0%    0.0%    0.0%    0.0%    0.0%    0.0%    0.0%    0.0%    0.0%    0.0%    0.0%    0.0%    0.0%    0.0%    0.0%    0.0%    0.0%    0.0%    0.0%    0.0%    0.0%    0.0%    0.0%    0.0%        k__Bacteria;p__OP3;c__; o__       2    0.0%    0.0%    0.0%    0.0%    0.0%    0.0%    0.0%    0.0%    0.0%    0.0%    0.0%    0.0%    0.0%    0.0%    0.0%    0.0%    0.0%    0.0%    0.0%    0.0%    0.0%    0.0%    0.0%    0.0%    0.0%    0.0%    0.0%    0.0%    0.0%    0.0%    0.0%    0.0%    0.0%    0.0%    0.0%    0.0%    0.0%    0.0%    0.0%    0.0%    0.0%    0.0%    0.0%    0.0%    0.0%    0.0%    0.0%    0.0%    0.0%    0.0%    0.0%    0.0%    0.0%    0.0%    0.0%    0.0%    0.0%    0.0%    0.0%    0.0%    0.0%    0.0%    0.0%    0.0%    0.0%    0.0%    0.0%    0.0%    0.0%    0.0%    0.0%    0.0%    0.0%    0.0%    0.0%    0.0%    0.0%    0.0%    0.0%    0.0%    0.0%    0.0%    0.0%    0.0%    0.0%    0.0%    0.0%    0.0%    0.0%    0.0%    0.0%    0.0%    0.0%    0.0%    0.0%    0.0%    0.0%    0.0%    0.0%    0.0%    0.0%    0.0%    0.0%    0.0%    0.0%    0.0%    0.0%    0.0%    0.0%    0.0%    0.0%    0.0%    0.0%    0.0%    0.0%    0.0%    0.0%    0.0%    0.0%    0.0%    0.0%    0.0%    0.0%    0.0%    0.0%    0.0%    0.0%    0.0%    0.0%    0.0%    0.0%    0.0%    0.0%    0.0%    0.0%    0.0%    0.0%    0.0%    0.0%    0.0%    0.0%    0.0%    0.0%    0.0%    0.0%    0.0%    0.0%    0.0%    0.0%    0.0%    0.0%    0.0%    0.0%    0.0%    0.0%    0.0%    0.0%    0.0%    0.0%    0.0%    0.0%    0.0%    0.0%    0.0%    0.0%    0.0%    0.0%    0.0%    0.0%    0.0%    0.0%    0.0%    0.0%    0.0%    0.0%    0.0%    0.0%    0.0%    0.0%    0.0%    0.0%    0.0%    0.0%    0.0%    0.0%    0.0%    0.0%    0.0%    0.0%    0.0%    0.0%    0.0%    0.0%    0.0%    0.0%    0.0%    0.0%    0.0%    0.0%    0.0%    0.0%    0.0%    0.0%    0.0%    0.0%    0.0%    0.0%    0.0%    0.0%    0.0%    0.0%    0.0%    0.0%    0.0%    0.0%    0.0%    0.0%    0.0%    0.0%    0.0%    0.0%    0.0%    0.0%    0.0%    0.0%    0.0%    0.0%    0.0%    0.0%    0.0%    0.0%    0.0%    0.0%    0.0%    0.0%    0.0%    0.0%    0.0%    0.0%    0.0%    0.0%    0.0%    0.0%    0.0%    0.0%    0.0%    0.0%    0.0%    0.0%    0.0%    0.0%    0.0%    0.0%    0.0%    0.0%    0.0%    0.0%    0.0%    0.0%    0.0%    0.0%    0.0%    0.0%    0.0%    0.0%    0.0%    0.0%    0.0%    0.0%    0.0%    0.0%    0.0%    0.0%    0.0%    0.0%    0.0%    0.0%    0.0%    0.0%    0.0%    0.0%    0.0%    0.0%    0.0%    0.0%    0.0%    0.0%    0.0%    0.0%    0.0%    0.0%    0.0%    0.0%    0.0%    0.0%    0.0%    0.0%    0.0%    0.0%    0.0%    0.0%    0.0%    0.0%    0.0%    0.0%    0.0%    0.0%    0.0%    0.0%    0.0%    0.0%    0.0%    0.0%    0.0%    0.0%    0.0%    0.0%    0.0%    0.0%    0.0%    0.0%    0.0%    0.0%    0.0%    0.0%    0.0%    0.0%    0.0%    0.0%    0.0%    0.0%    0.0%    0.0%    0.0%    0.0%    0.0%    0.0%    0.0%    0.0%    0.0%    0.0%    0.0%    0.0%    0.0%    0.0%    0.0%    0.0%    0.0%    0.0%    0.0%    0.0%    0.0%    0.0%    0.0%    0.0%    0.0%    0.0%    0.0%        k__Bacteria;p__OP8;c__OP8; o__       1    0.0%    0.0%    0.0%    0.0%    0.0%    0.0%    0.0%    0.0%    0.0%    0.0%    0.0%    0.0%    0.0%    0.0%    0.0%    0.0%    0.0%    0.0%    0.0%    0.0%    0.0%    0.0%    0.0%    0.0%    0.0%    0.0%    0.0%    0.0%    0.0%    0.0%    0.0%    0.0%    0.0%    0.0%    0.0%    0.0%    0.0%    0.0%    0.0%    0.0%    0.0%    0.0%    0.0%    0.0%    0.0%    0.0%    0.0%    0.0%    0.0%    0.0%    0.0%    0.0%    0.0%    0.0%    0.0%    0.0%    0.0%    0.0%    0.0%    0.0%    0.0%    0.0%    0.0%    0.0%    0.0%    0.0%    0.0%    0.0%    0.0%    0.0%    0.0%    0.0%    0.0%    0.0%    0.0%    0.0%    0.0%    0.0%    0.0%    0.0%    0.0%    0.0%    0.0%    0.0%    0.0%    0.0%    0.0%    0.0%    0.0%    0.0%    0.0%    0.0%    0.0%    0.0%    0.0%    0.0%    0.0%    0.0%    0.0%    0.0%    0.0%    0.0%    0.0%    0.0%    0.0%    0.0%    0.0%    0.0%    0.0%    0.0%    0.0%    0.0%    0.0%    0.0%    0.0%    0.0%    0.0%    0.0%    0.0%    0.0%    0.0%    0.0%    0.0%    0.0%    0.0%    0.0%    0.0%    0.0%    0.0%    0.0%    0.0%    0.0%    0.0%    0.0%    0.0%    0.0%    0.0%    0.0%    0.0%    0.0%    0.0%    0.0%    0.0%    0.0%    0.0%    0.0%    0.0%    0.0%    0.0%    0.0%    0.0%    0.0%    0.0%    0.0%    0.0%    0.0%    0.0%    0.0%    0.0%    0.0%    0.0%    0.0%    0.0%    0.0%    0.0%    0.0%    0.0%    0.0%    0.0%    0.0%    0.0%    0.0%    0.0%    0.0%    0.0%    0.0%    0.0%    0.0%    0.0%    0.0%    0.0%    0.0%    0.0%    0.0%    0.0%    0.0%    0.0%    0.0%    0.0%    0.0%    0.0%    0.0%    0.0%    0.0%    0.0%    0.0%    0.0%    0.0%    0.0%    0.0%    0.0%    0.0%    0.0%    0.0%    0.0%    0.0%    0.0%    0.0%    0.0%    0.0%    0.0%    0.0%    0.0%    0.0%    0.0%    0.0%    0.0%    0.0%    0.0%    0.0%    0.0%    0.0%    0.0%    0.0%    0.0%    0.0%    0.0%    0.0%    0.0%    0.0%    0.0%    0.0%    0.0%    0.0%    0.0%    0.0%    0.0%    0.0%    0.0%    0.0%    0.0%    0.0%    0.0%    0.0%    0.0%    0.0%    0.0%    0.0%    0.0%    0.0%    0.0%    0.0%    0.0%    0.0%    0.0%    0.0%    0.0%    0.0%    0.0%    0.0%    0.0%    0.0%    0.0%    0.0%    0.0%    0.0%    0.0%    0.0%    0.0%    0.0%    0.0%    0.0%    0.0%    0.0%    0.0%    0.0%    0.0%    0.0%    0.0%    0.0%    0.0%    0.0%    0.0%    0.0%    0.0%    0.0%    0.0%    0.0%    0.0%    0.0%    0.0%    0.0%    0.0%    0.0%    0.0%    0.0%    0.0%    0.0%    0.0%    0.0%    0.0%    0.0%    0.0%    0.0%    0.0%    0.0%    0.0%    0.0%    0.0%    0.0%    0.0%    0.0%    0.0%    0.0%    0.0%    0.0%    0.0%    0.0%    0.0%    0.0%    0.0%    0.0%    0.0%    0.0%    0.0%    0.0%    0.0%    0.0%    0.0%    0.0%    0.0%    0.0%    0.0%    0.0%    0.0%    0.0%    0.0%    0.0%    0.0%    0.0%    0.0%    0.0%    0.0%    0.0%    0.0%    0.0%    0.0%    0.0%    0.0%    0.0%    0.0%    0.0%    0.0%    0.0%    0.0%    0.0%    0.0%    0.0%        k__Bacteria;p__Planctomycetes;c__FFCH393; o__     153    0.0%    0.0%    0.0%    0.0%    0.0%    0.0%    0.0%    0.0%    0.0%    0.0%    0.0%    0.0%    0.0%    0.0%    0.0%    0.0%    0.0%    0.0%    0.0%    0.0%    0.0%    0.0%    0.0%    0.0%    0.0%    0.0%    0.0%    0.0%    0.0%    0.0%    0.0%    0.0%    0.0%    0.0%    0.0%    0.0%    0.0%    0.0%    0.0%    0.0%    0.0%    0.0%    0.0%    0.0%    0.0%    0.0%    0.0%    0.0%    0.0%    0.0%    0.0%    0.0%    0.0%    0.0%    0.0%    0.0%    0.0%    0.0%    0.0%    0.0%    0.0%    0.0%    0.0%    0.0%    0.0%    0.0%    0.0%    0.0%    0.0%    0.0%    0.0%    0.0%    0.0%    0.0%    0.0%    0.0%    0.0%    0.0%    0.0%    0.0%    0.0%    0.0%    0.0%    0.0%    0.0%    0.0%    0.0%    0.0%    0.0%    0.0%    0.0%    0.0%    0.0%    0.0%    0.0%    0.0%    0.0%    0.0%    0.0%    0.0%    0.0%    0.0%    0.0%    0.0%    0.0%    0.0%    0.0%    0.0%    0.0%    0.0%    0.0%    0.0%    0.0%    0.0%    0.0%    0.0%    0.0%    0.0%    0.0%    0.0%    0.0%    0.0%    0.0%    0.0%    0.0%    0.0%    0.0%    0.0%    0.0%    0.0%    0.0%    0.0%    0.0%    0.0%    0.0%    0.0%    0.0%    0.0%    0.0%    0.0%    0.0%    0.0%    0.0%    0.0%    0.0%    0.0%    0.0%    0.0%    0.0%    0.0%    0.0%    0.0%    0.0%    0.0%    0.0%    0.0%    0.0%    0.0%    0.0%    0.0%    0.0%    0.0%    0.0%    0.0%    0.0%    0.0%    0.0%    0.0%    0.0%    0.0%    0.0%    0.0%    0.0%    0.0%    0.0%    0.0%    0.0%    0.0%    0.0%    0.0%    0.0%    0.0%    0.0%    0.0%    0.0%    0.0%    0.0%    0.0%    0.0%    0.0%    0.0%    0.0%    0.0%    0.0%    0.0%    0.0%    0.0%    0.0%    0.0%    0.0%    0.0%    0.0%    0.0%    0.0%    0.0%    0.0%    0.0%    0.0%    0.0%    0.0%    0.0%    0.0%    0.0%    0.0%    0.0%    0.0%    0.0%    0.0%    0.0%    0.0%    0.0%    0.0%    0.0%    0.0%    0.0%    0.0%    0.0%    0.0%    0.0%    0.0%    0.0%    0.0%    0.0%    0.0%    0.0%    0.0%    0.0%    0.0%    0.0%    0.0%    0.0%    0.0%    0.0%    0.0%    0.0%    0.0%    0.0%    0.0%    0.0%    0.0%    0.0%    0.0%    0.0%    0.0%    0.0%    0.0%    0.0%    0.0%    0.0%    0.0%    0.0%    0.0%    0.0%    0.0%    0.0%    0.0%    0.0%    0.0%    0.0%    0.0%    0.0%    0.0%    0.0%    0.0%    0.0%    0.0%    0.0%    0.0%    0.0%    0.0%    0.0%    0.0%    0.0%    0.0%    0.0%    0.0%    0.0%    0.0%    0.0%    0.0%    0.0%    0.0%    0.0%    0.0%    0.0%    0.0%    0.0%    0.0%    0.0%    0.0%    0.0%    0.0%    0.0%    0.0%    0.0%    0.0%    0.0%    0.0%    0.0%    0.0%    0.0%    0.0%    0.0%    0.0%    0.0%    0.0%    0.0%    0.0%    0.0%    0.0%    0.0%    0.0%    0.0%    0.0%    0.0%    0.0%    0.0%    0.0%    0.0%    0.0%    0.0%    0.0%    0.0%    0.0%    0.0%    0.0%    0.0%    0.0%    0.0%    0.0%    0.0%    0.0%    0.0%    0.0%    0.0%    0.0%    0.0%    0.0%    0.0%    0.0%    0.0%    0.0%    0.0%    0.0%    0.0%    0.0%    0.0%    0.0%        k__Bacteria;p__Planctomycetes;c__Kueneniae; o__Kueneniales       1    0.0%    0.0%    0.0%    0.0%    0.0%    0.0%    0.0%    0.0%    0.0%    0.0%    0.0%    0.0%    0.0%    0.0%    0.0%    0.0%    0.0%    0.0%    0.0%    0.0%    0.0%    0.0%    0.0%    0.0%    0.0%    0.0%    0.0%    0.0%    0.0%    0.0%    0.0%    0.0%    0.0%    0.0%    0.0%    0.0%    0.0%    0.0%    0.0%    0.0%    0.0%    0.0%    0.0%    0.0%    0.0%    0.0%    0.0%    0.0%    0.0%    0.0%    0.0%    0.0%    0.0%    0.0%    0.0%    0.0%    0.0%    0.0%    0.0%    0.0%    0.0%    0.0%    0.0%    0.0%    0.0%    0.0%    0.0%    0.0%    0.0%    0.0%    0.0%    0.0%    0.0%    0.0%    0.0%    0.0%    0.0%    0.0%    0.0%    0.0%    0.0%    0.0%    0.0%    0.0%    0.0%    0.0%    0.0%    0.0%    0.0%    0.0%    0.0%    0.0%    0.0%    0.0%    0.0%    0.0%    0.0%    0.0%    0.0%    0.0%    0.0%    0.0%    0.0%    0.0%    0.0%    0.0%    0.0%    0.0%    0.0%    0.0%    0.0%    0.0%    0.0%    0.0%    0.0%    0.0%    0.0%    0.0%    0.0%    0.0%    0.0%    0.0%    0.0%    0.0%    0.0%    0.0%    0.0%    0.0%    0.0%    0.0%    0.0%    0.0%    0.0%    0.0%    0.0%    0.0%    0.0%    0.0%    0.0%    0.0%    0.0%    0.0%    0.0%    0.0%    0.0%    0.0%    0.0%    0.0%    0.0%    0.0%    0.0%    0.0%    0.0%    0.0%    0.0%    0.0%    0.0%    0.0%    0.0%    0.0%    0.0%    0.0%    0.0%    0.0%    0.0%    0.0%    0.0%    0.0%    0.0%    0.0%    0.0%    0.0%    0.0%    0.0%    0.0%    0.0%    0.0%    0.0%    0.0%    0.0%    0.0%    0.0%    0.0%    0.0%    0.0%    0.0%    0.0%    0.0%    0.0%    0.0%    0.0%    0.0%    0.0%    0.0%    0.0%    0.0%    0.0%    0.0%    0.0%    0.0%    0.0%    0.0%    0.0%    0.0%    0.0%    0.0%    0.0%    0.0%    0.0%    0.0%    0.0%    0.0%    0.0%    0.0%    0.0%    0.0%    0.0%    0.0%    0.0%    0.0%    0.0%    0.0%    0.0%    0.0%    0.0%    0.0%    0.0%    0.0%    0.0%    0.0%    0.0%    0.0%    0.0%    0.0%    0.0%    0.0%    0.0%    0.0%    0.0%    0.0%    0.0%    0.0%    0.0%    0.0%    0.0%    0.0%    0.0%    0.0%    0.0%    0.0%    0.0%    0.0%    0.0%    0.0%    0.0%    0.0%    0.0%    0.0%    0.0%    0.0%    0.0%    0.0%    0.0%    0.0%    0.0%    0.0%    0.0%    0.0%    0.0%    0.0%    0.0%    0.0%    0.0%    0.0%    0.0%    0.0%    0.0%    0.0%    0.0%    0.0%    0.0%    0.0%    0.0%    0.0%    0.0%    0.0%    0.0%    0.0%    0.0%    0.0%    0.0%    0.0%    0.0%    0.0%    0.0%    0.0%    0.0%    0.0%    0.0%    0.0%    0.0%    0.0%    0.0%    0.0%    0.0%    0.0%    0.0%    0.0%    0.0%    0.0%    0.0%    0.0%    0.0%    0.0%    0.0%    0.0%    0.0%    0.0%    0.0%    0.0%    0.0%    0.0%    0.0%    0.0%    0.0%    0.0%    0.0%    0.0%    0.0%    0.0%    0.0%    0.0%    0.0%    0.0%    0.0%    0.0%    0.0%    0.0%    0.0%    0.0%    0.0%    0.0%    0.0%    0.0%    0.0%    0.0%    0.0%    0.0%    0.0%    0.0%    0.0%    0.0%    0.0%    0.0%    0.0%    0.0%    0.0%    0.0%        k__Bacteria;p__Planctomycetes;c__PW285; o__       0    0.0%    0.0%    0.0%    0.0%    0.0%    0.0%    0.0%    0.0%    0.0%    0.0%    0.0%    0.0%    0.0%    0.0%    0.0%    0.0%    0.0%    0.0%    0.0%    0.0%    0.0%    0.0%    0.0%    0.0%    0.0%    0.0%    0.0%    0.0%    0.0%    0.0%    0.0%    0.0%    0.0%    0.0%    0.0%    0.0%    0.0%    0.0%    0.0%    0.0%    0.0%    0.0%    0.0%    0.0%    0.0%    0.0%    0.0%    0.0%    0.0%    0.0%    0.0%    0.0%    0.0%    0.0%    0.0%    0.0%    0.0%    0.0%    0.0%    0.0%    0.0%    0.0%    0.0%    0.0%    0.0%    0.0%    0.0%    0.0%    0.0%    0.0%    0.0%    0.0%    0.0%    0.0%    0.0%    0.0%    0.0%    0.0%    0.0%    0.0%    0.0%    0.0%    0.0%    0.0%    0.0%    0.0%    0.0%    0.0%    0.0%    0.0%    0.0%    0.0%    0.0%    0.0%    0.0%    0.0%    0.0%    0.0%    0.0%    0.0%    0.0%    0.0%    0.0%    0.0%    0.0%    0.0%    0.0%    0.0%    0.0%    0.0%    0.0%    0.0%    0.0%    0.0%    0.0%    0.0%    0.0%    0.0%    0.0%    0.0%    0.0%    0.0%    0.0%    0.0%    0.0%    0.0%    0.0%    0.0%    0.0%    0.0%    0.0%    0.0%    0.0%    0.0%    0.0%    0.0%    0.0%    0.0%    0.0%    0.0%    0.0%    0.0%    0.0%    0.0%    0.0%    0.0%    0.0%    0.0%    0.0%    0.0%    0.0%    0.0%    0.0%    0.0%    0.0%    0.0%    0.0%    0.0%    0.0%    0.0%    0.0%    0.0%    0.0%    0.0%    0.0%    0.0%    0.0%    0.0%    0.0%    0.0%    0.0%    0.0%    0.0%    0.0%    0.0%    0.0%    0.0%    0.0%    0.0%    0.0%    0.0%    0.0%    0.0%    0.0%    0.0%    0.0%    0.0%    0.0%    0.0%    0.0%    0.0%    0.0%    0.0%    0.0%    0.0%    0.0%    0.0%    0.0%    0.0%    0.0%    0.0%    0.0%    0.0%    0.0%    0.0%    0.0%    0.0%    0.0%    0.0%    0.0%    0.0%    0.0%    0.0%    0.0%    0.0%    0.0%    0.0%    0.0%    0.0%    0.0%    0.0%    0.0%    0.0%    0.0%    0.0%    0.0%    0.0%    0.0%    0.0%    0.0%    0.0%    0.0%    0.0%    0.0%    0.0%    0.0%    0.0%    0.0%    0.0%    0.0%    0.0%    0.0%    0.0%    0.0%    0.0%    0.0%    0.0%    0.0%    0.0%    0.0%    0.0%    0.0%    0.0%    0.0%    0.0%    0.0%    0.0%    0.0%    0.0%    0.0%    0.0%    0.0%    0.0%    0.0%    0.0%    0.0%    0.0%    0.0%    0.0%    0.0%    0.0%    0.0%    0.0%    0.0%    0.0%    0.0%    0.0%    0.0%    0.0%    0.0%    0.0%    0.0%    0.0%    0.0%    0.0%    0.0%    0.0%    0.0%    0.0%    0.0%    0.0%    0.0%    0.0%    0.0%    0.0%    0.0%    0.0%    0.0%    0.0%    0.0%    0.0%    0.0%    0.0%    0.0%    0.0%    0.0%    0.0%    0.0%    0.0%    0.0%    0.0%    0.0%    0.0%    0.0%    0.0%    0.0%    0.0%    0.0%    0.0%    0.0%    0.0%    0.0%    0.0%    0.0%    0.0%    0.0%    0.0%    0.0%    0.0%    0.0%    0.0%    0.0%    0.0%    0.0%    0.0%    0.0%    0.0%    0.0%    0.0%    0.0%    0.0%    0.0%    0.0%    0.0%    0.0%    0.0%    0.0%    0.0%    0.0%    0.0%    0.0%    0.0%    0.0%    0.0%    0.0%    0.0%    0.0%    0.0%        k__Bacteria;p__Planctomycetes;c__Phycisphaerae; o__    1987    0.0%    0.0%    0.0%    0.0%    0.0%    0.0%    0.0%    0.0%    0.0%    0.0%    0.0%    0.0%    0.0%    0.0%    0.0%    0.0%    0.0%    0.0%    0.0%    0.0%    0.0%    0.0%    0.0%    0.0%    0.0%    0.0%    0.0%    0.0%    0.0%    0.0%    0.0%    0.0%    0.0%    0.0%    0.0%    0.0%    0.0%    0.0%    0.0%    0.0%    0.0%    0.0%    0.0%    0.0%    0.0%    0.0%    0.0%    0.0%    0.0%    0.0%    0.0%    0.0%    0.0%    0.0%    0.0%    0.0%    0.0%    0.0%    0.0%    0.0%    0.1%    0.0%    0.0%    0.0%    0.0%    0.0%    0.0%    0.0%    0.0%    0.0%    0.0%    0.0%    0.0%    0.0%    0.0%    0.0%    0.0%    0.0%    0.0%    0.0%    0.0%    0.0%    0.0%    0.0%    0.0%    0.0%    0.0%    0.0%    0.0%    0.0%    0.0%    0.0%    0.0%    0.0%    0.0%    0.0%    0.0%    0.0%    0.0%    0.0%    0.0%    0.0%    0.0%    0.0%    0.0%    0.0%    0.0%    0.0%    0.0%    0.0%    0.0%    0.0%    0.0%    0.0%    0.0%    0.0%    0.0%    0.0%    0.0%    0.1%    0.0%    0.0%    0.0%    0.0%    0.0%    0.0%    0.0%    0.0%    0.0%    0.1%    0.0%    0.0%    0.0%    0.0%    0.1%    0.0%    0.0%    0.0%    0.0%    0.0%    0.0%    0.0%    0.0%    0.0%    0.0%    0.0%    0.0%    0.0%    0.0%    0.0%    0.0%    0.0%    0.0%    0.0%    0.0%    0.0%    0.0%    0.0%    0.0%    0.0%    0.1%    0.0%    0.0%    0.0%    0.0%    0.0%    0.0%    0.0%    0.0%    0.0%    0.0%    0.0%    0.0%    0.0%    0.0%    0.0%    0.0%    0.0%    0.0%    0.0%    0.0%    0.0%    0.0%    0.0%    0.0%    0.0%    0.0%    0.0%    0.0%    0.0%    0.0%    0.0%    0.0%    0.0%    0.0%    0.0%    0.0%    0.0%    0.0%    0.0%    0.0%    0.0%    0.0%    0.1%    0.0%    0.0%    0.0%    0.0%    0.1%    0.0%    0.0%    0.0%    0.0%    0.0%    0.0%    0.1%    0.0%    0.1%    0.0%    0.1%    0.0%    0.0%    0.0%    0.0%    0.0%    0.0%    0.0%    0.0%    0.0%    0.0%    0.0%    0.0%    0.0%    0.0%    0.0%    0.0%    0.0%    0.0%    0.0%    0.0%    0.0%    0.0%    0.0%    0.0%    0.0%    0.0%    0.0%    0.0%    0.0%    0.0%    0.0%    0.0%    0.0%    0.0%    0.0%    0.0%    0.0%    0.0%    0.0%    0.0%    0.0%    0.0%    0.0%    0.0%    0.0%    0.0%    0.0%    0.0%    0.0%    0.0%    0.0%    0.0%    0.0%    0.0%    0.0%    0.0%    0.0%    0.0%    0.0%    0.0%    0.0%    0.0%    0.0%    0.0%    0.0%    0.0%    0.0%    0.1%    0.0%    0.0%    0.0%    0.0%    0.0%    0.0%    0.0%    0.0%    0.0%    0.1%    0.0%    0.0%    0.0%    0.0%    0.0%    0.0%    0.0%    0.0%    0.0%    0.0%    0.0%    0.0%    0.0%    0.0%    0.0%    0.0%    0.0%    0.0%    0.0%    0.0%    0.0%    0.0%    0.0%    0.0%    0.0%    0.0%    0.0%    0.0%    0.0%    0.0%    0.0%    0.0%    0.0%    0.0%    0.0%    0.0%    0.0%    0.0%    0.0%    0.0%    0.0%    0.0%    0.0%    0.0%    0.0%    0.0%    0.0%    0.0%    0.0%    0.0%    0.0%    0.0%    0.0%    0.0%    0.0%    0.0%    0.0%    0.0%    0.0%    0.0%        k__Bacteria;p__Planctomycetes;c__Phycisphaerae; o__Phycisphaerales      18    0.0%    0.0%    0.0%    0.0%    0.0%    0.0%    0.0%    0.0%    0.0%    0.0%    0.0%    0.0%    0.0%    0.0%    0.0%    0.0%    0.0%    0.0%    0.0%    0.0%    0.0%    0.0%    0.0%    0.0%    0.0%    0.0%    0.0%    0.0%    0.0%    0.0%    0.0%    0.0%    0.0%    0.0%    0.0%    0.0%    0.0%    0.0%    0.0%    0.0%    0.0%    0.0%    0.0%    0.0%    0.0%    0.0%    0.0%    0.0%    0.0%    0.0%    0.0%    0.0%    0.0%    0.0%    0.0%    0.0%    0.0%    0.0%    0.0%    0.0%    0.0%    0.0%    0.0%    0.0%    0.0%    0.0%    0.0%    0.0%    0.0%    0.0%    0.0%    0.0%    0.0%    0.0%    0.0%    0.0%    0.0%    0.0%    0.0%    0.0%    0.0%    0.0%    0.0%    0.0%    0.0%    0.0%    0.0%    0.0%    0.0%    0.0%    0.0%    0.0%    0.0%    0.0%    0.0%    0.0%    0.0%    0.0%    0.0%    0.0%    0.0%    0.0%    0.0%    0.0%    0.0%    0.0%    0.0%    0.0%    0.0%    0.0%    0.0%    0.0%    0.0%    0.0%    0.0%    0.0%    0.0%    0.0%    0.0%    0.0%    0.0%    0.0%    0.0%    0.0%    0.0%    0.0%    0.0%    0.0%    0.0%    0.0%    0.0%    0.0%    0.0%    0.0%    0.0%    0.0%    0.0%    0.0%    0.0%    0.0%    0.0%    0.0%    0.0%    0.0%    0.0%    0.0%    0.0%    0.0%    0.0%    0.0%    0.0%    0.0%    0.0%    0.0%    0.0%    0.0%    0.0%    0.0%    0.0%    0.0%    0.0%    0.0%    0.0%    0.0%    0.0%    0.0%    0.0%    0.0%    0.0%    0.0%    0.0%    0.0%    0.0%    0.0%    0.0%    0.0%    0.0%    0.0%    0.0%    0.0%    0.0%    0.0%    0.0%    0.0%    0.0%    0.0%    0.0%    0.0%    0.0%    0.0%    0.0%    0.0%    0.0%    0.0%    0.0%    0.0%    0.0%    0.0%    0.0%    0.0%    0.0%    0.0%    0.0%    0.0%    0.0%    0.0%    0.0%    0.0%    0.0%    0.0%    0.0%    0.0%    0.0%    0.0%    0.0%    0.0%    0.0%    0.0%    0.0%    0.0%    0.0%    0.0%    0.0%    0.0%    0.0%    0.0%    0.0%    0.0%    0.0%    0.0%    0.0%    0.0%    0.0%    0.0%    0.0%    0.0%    0.0%    0.0%    0.0%    0.0%    0.0%    0.0%    0.0%    0.0%    0.0%    0.0%    0.0%    0.0%    0.0%    0.0%    0.0%    0.0%    0.0%    0.0%    0.0%    0.0%    0.0%    0.0%    0.0%    0.0%    0.0%    0.0%    0.0%    0.0%    0.0%    0.0%    0.0%    0.0%    0.0%    0.0%    0.0%    0.0%    0.0%    0.0%    0.0%    0.0%    0.0%    0.0%    0.0%    0.0%    0.0%    0.0%    0.0%    0.0%    0.0%    0.0%    0.0%    0.0%    0.0%    0.0%    0.0%    0.0%    0.0%    0.0%    0.0%    0.0%    0.0%    0.0%    0.0%    0.0%    0.0%    0.0%    0.0%    0.0%    0.0%    0.0%    0.0%    0.0%    0.0%    0.0%    0.0%    0.0%    0.0%    0.0%    0.0%    0.0%    0.0%    0.0%    0.0%    0.0%    0.0%    0.0%    0.0%    0.0%    0.0%    0.0%    0.0%    0.0%    0.0%    0.0%    0.0%    0.0%    0.0%    0.0%    0.0%    0.0%    0.0%    0.0%    0.0%    0.0%    0.0%    0.0%    0.0%    0.0%    0.0%    0.0%    0.0%    0.0%    0.0%    0.0%    0.0%    0.0%    0.0%    0.0%    0.0%    0.0%    0.0%    0.0%        k__Bacteria;p__Planctomycetes;c__Planctomycea; o__Gemmatales    1872    0.0%    0.0%    0.0%    0.0%    0.0%    0.0%    0.0%    0.0%    0.0%    0.0%    0.0%    0.0%    0.0%    0.0%    0.0%    0.0%    0.0%    0.0%    0.0%    0.0%    0.0%    0.0%    0.0%    0.0%    0.0%    0.0%    0.0%    0.0%    0.0%    0.0%    0.0%    0.0%    0.0%    0.0%    0.0%    0.0%    0.0%    0.0%    0.0%    0.0%    0.0%    0.0%    0.0%    0.0%    0.0%    0.0%    0.0%    0.0%    0.0%    0.0%    0.0%    0.0%    0.0%    0.0%    0.0%    0.0%    0.0%    0.0%    0.0%    0.0%    0.0%    0.0%    0.0%    0.0%    0.0%    0.1%    0.0%    0.0%    0.0%    0.0%    0.0%    0.0%    0.0%    0.0%    0.0%    0.0%    0.0%    0.0%    0.0%    0.0%    0.0%    0.0%    0.0%    0.0%    0.0%    0.0%    0.0%    0.0%    0.0%    0.0%    0.0%    0.0%    0.0%    0.0%    0.0%    0.0%    0.0%    0.0%    0.0%    0.0%    0.0%    0.0%    0.0%    0.0%    0.0%    0.0%    0.1%    0.0%    0.0%    0.0%    0.0%    0.0%    0.0%    0.0%    0.0%    0.0%    0.0%    0.0%    0.0%    0.0%    0.0%    0.0%    0.0%    0.0%    0.0%    0.1%    0.1%    0.0%    0.0%    0.1%    0.1%    0.0%    0.0%    0.0%    0.1%    0.0%    0.0%    0.0%    0.0%    0.0%    0.0%    0.0%    0.0%    0.0%    0.0%    0.0%    0.0%    0.0%    0.0%    0.0%    0.0%    0.0%    0.0%    0.0%    0.0%    0.0%    0.0%    0.0%    0.0%    0.0%    0.1%    0.0%    0.0%    0.0%    0.0%    0.0%    0.0%    0.0%    0.0%    0.0%    0.0%    0.0%    0.0%    0.0%    0.0%    0.0%    0.0%    0.0%    0.0%    0.0%    0.0%    0.0%    0.0%    0.0%    0.0%    0.0%    0.0%    0.0%    0.0%    0.0%    0.0%    0.0%    0.0%    0.0%    0.0%    0.0%    0.0%    0.0%    0.0%    0.0%    0.0%    0.0%    0.0%    0.0%    0.0%    0.0%    0.0%    0.0%    0.1%    0.0%    0.0%    0.0%    0.0%    0.0%    0.0%    0.0%    0.0%    0.0%    0.0%    0.0%    0.0%    0.0%    0.0%    0.0%    0.0%    0.0%    0.0%    0.0%    0.0%    0.0%    0.0%    0.0%    0.0%    0.0%    0.0%    0.0%    0.0%    0.0%    0.0%    0.0%    0.0%    0.0%    0.0%    0.0%    0.0%    0.0%    0.0%    0.0%    0.0%    0.0%    0.0%    0.0%    0.0%    0.0%    0.0%    0.0%    0.0%    0.0%    0.0%    0.0%    0.0%    0.0%    0.0%    0.0%    0.0%    0.0%    0.0%    0.0%    0.0%    0.0%    0.0%    0.0%    0.0%    0.0%    0.0%    0.0%    0.0%    0.0%    0.0%    0.0%    0.0%    0.0%    0.0%    0.0%    0.0%    0.0%    0.0%    0.0%    0.0%    0.0%    0.0%    0.0%    0.0%    0.0%    0.0%    0.0%    0.0%    0.0%    0.0%    0.0%    0.0%    0.0%    0.0%    0.1%    0.0%    0.0%    0.0%    0.0%    0.0%    0.0%    0.0%    0.0%    0.0%    0.0%    0.0%    0.0%    0.0%    0.0%    0.0%    0.0%    0.0%    0.0%    0.0%    0.0%    0.0%    0.0%    0.0%    0.0%    0.0%    0.0%    0.0%    0.0%    0.0%    0.0%    0.0%    0.0%    0.0%    0.0%    0.0%    0.0%    0.0%    0.0%    0.0%    0.0%    0.0%    0.0%    0.0%    0.0%    0.0%    0.0%    0.0%    0.0%    0.0%    0.1%    0.0%    0.0%    0.0%    0.0%        k__Bacteria;p__Planctomycetes;c__Planctomycea; o__Pirellulales    1200    0.0%    0.0%    0.0%    0.0%    0.0%    0.0%    0.0%    0.0%    0.0%    0.0%    0.0%    0.0%    0.0%    0.0%    0.0%    0.0%    0.0%    0.0%    0.0%    0.0%    0.0%    0.0%    0.0%    0.0%    0.0%    0.0%    0.0%    0.0%    0.0%    0.0%    0.0%    0.0%    0.0%    0.0%    0.0%    0.0%    0.0%    0.0%    0.0%    0.0%    0.0%    0.0%    0.0%    0.0%    0.0%    0.0%    0.0%    0.0%    0.0%    0.0%    0.0%    0.0%    0.0%    0.0%    0.1%    0.0%    0.0%    0.0%    0.0%    0.0%    0.0%    0.0%    0.0%    0.0%    0.0%    0.0%    0.0%    0.0%    0.0%    0.0%    0.0%    0.0%    0.0%    0.0%    0.0%    0.0%    0.0%    0.0%    0.0%    0.0%    0.0%    0.0%    0.0%    0.0%    0.0%    0.0%    0.0%    0.0%    0.0%    0.0%    0.0%    0.0%    0.0%    0.0%    0.0%    0.0%    0.0%    0.0%    0.0%    0.0%    0.0%    0.0%    0.0%    0.0%    0.0%    0.0%    0.0%    0.0%    0.0%    0.0%    0.0%    0.0%    0.0%    0.0%    0.0%    0.0%    0.0%    0.0%    0.0%    0.0%    0.0%    0.0%    0.0%    0.0%    0.0%    0.1%    0.0%    0.0%    0.0%    0.1%    0.0%    0.0%    0.0%    0.0%    0.0%    0.0%    0.0%    0.0%    0.0%    0.0%    0.0%    0.0%    0.0%    0.0%    0.0%    0.0%    0.0%    0.0%    0.0%    0.0%    0.0%    0.0%    0.0%    0.0%    0.0%    0.0%    0.0%    0.0%    0.0%    0.0%    0.0%    0.0%    0.0%    0.0%    0.0%    0.0%    0.0%    0.0%    0.0%    0.0%    0.0%    0.0%    0.0%    0.0%    0.0%    0.0%    0.0%    0.0%    0.0%    0.0%    0.0%    0.0%    0.0%    0.0%    0.0%    0.0%    0.0%    0.0%    0.0%    0.0%    0.0%    0.0%    0.0%    0.0%    0.0%    0.0%    0.0%    0.0%    0.0%    0.0%    0.0%    0.0%    0.0%    0.0%    0.0%    0.0%    0.0%    0.0%    0.0%    0.0%    0.0%    0.0%    0.1%    0.1%    0.0%    0.0%    0.0%    0.0%    0.0%    0.0%    0.0%    0.0%    0.0%    0.0%    0.0%    0.0%    0.0%    0.0%    0.0%    0.0%    0.0%    0.0%    0.0%    0.0%    0.0%    0.0%    0.0%    0.0%    0.0%    0.0%    0.0%    0.0%    0.0%    0.0%    0.0%    0.0%    0.0%    0.0%    0.0%    0.0%    0.0%    0.0%    0.0%    0.0%    0.0%    0.0%    0.0%    0.0%    0.0%    0.0%    0.0%    0.0%    0.0%    0.0%    0.0%    0.0%    0.0%    0.0%    0.0%    0.0%    0.0%    0.0%    0.0%    0.0%    0.0%    0.0%    0.0%    0.0%    0.0%    0.0%    0.0%    0.0%    0.0%    0.0%    0.0%    0.0%    0.0%    0.0%    0.0%    0.0%    0.0%    0.0%    0.0%    0.0%    0.0%    0.0%    0.0%    0.1%    0.0%    0.0%    0.0%    0.0%    0.0%    0.1%    0.0%    0.0%    0.0%    0.0%    0.0%    0.0%    0.0%    0.0%    0.0%    0.0%    0.0%    0.0%    0.0%    0.0%    0.0%    0.0%    0.0%    0.0%    0.0%    0.0%    0.0%    0.0%    0.0%    0.0%    0.0%    0.0%    0.0%    0.0%    0.0%    0.0%    0.0%    0.0%    0.0%    0.0%    0.0%    0.0%    0.0%    0.0%    0.0%    0.0%    0.0%    0.0%    0.0%    0.0%    0.0%    0.0%    0.0%    0.0%    0.0%    0.0%    0.0%    0.0%    0.0%    0.1%        k__Bacteria;p__Planctomycetes;c__Planctomycea; o__Planctomycetales     559    0.0%    0.0%    0.0%    0.0%    0.0%    0.0%    0.0%    0.0%    0.0%    0.0%    0.0%    0.0%    0.0%    0.0%    0.0%    0.0%    0.0%    0.0%    0.0%    0.0%    0.0%    0.0%    0.0%    0.0%    0.0%    0.0%    0.0%    0.0%    0.0%    0.0%    0.0%    0.0%    0.0%    0.0%    0.0%    0.0%    0.0%    0.0%    0.0%    0.0%    0.0%    0.0%    0.0%    0.0%    0.0%    0.0%    0.0%    0.0%    0.0%    0.0%    0.0%    0.0%    0.0%    0.0%    0.0%    0.0%    0.0%    0.0%    0.0%    0.0%    0.0%    0.0%    0.0%    0.0%    0.0%    0.0%    0.0%    0.0%    0.0%    0.0%    0.0%    0.0%    0.0%    0.0%    0.0%    0.0%    0.0%    0.0%    0.0%    0.0%    0.0%    0.0%    0.0%    0.0%    0.0%    0.0%    0.0%    0.0%    0.0%    0.0%    0.0%    0.0%    0.0%    0.0%    0.0%    0.0%    0.0%    0.0%    0.0%    0.0%    0.0%    0.0%    0.0%    0.0%    0.0%    0.0%    0.0%    0.0%    0.0%    0.0%    0.0%    0.0%    0.0%    0.0%    0.0%    0.0%    0.0%    0.0%    0.0%    0.0%    0.0%    0.0%    0.0%    0.0%    0.0%    0.0%    0.0%    0.0%    0.0%    0.0%    0.0%    0.0%    0.0%    0.0%    0.0%    0.0%    0.0%    0.0%    0.0%    0.0%    0.0%    0.0%    0.0%    0.0%    0.0%    0.0%    0.0%    0.0%    0.0%    0.0%    0.0%    0.0%    0.0%    0.0%    0.0%    0.0%    0.0%    0.0%    0.0%    0.0%    0.0%    0.0%    0.0%    0.0%    0.0%    0.0%    0.0%    0.0%    0.0%    0.0%    0.0%    0.0%    0.0%    0.0%    0.0%    0.0%    0.0%    0.0%    0.0%    0.0%    0.0%    0.0%    0.0%    0.0%    0.0%    0.0%    0.0%    0.0%    0.0%    0.0%    0.0%    0.0%    0.0%    0.0%    0.0%    0.0%    0.0%    0.0%    0.0%    0.0%    0.0%    0.0%    0.0%    0.0%    0.0%    0.0%    0.0%    0.0%    0.0%    0.0%    0.0%    0.0%    0.1%    0.0%    0.0%    0.0%    0.0%    0.0%    0.0%    0.0%    0.0%    0.0%    0.0%    0.0%    0.0%    0.0%    0.0%    0.0%    0.0%    0.0%    0.0%    0.0%    0.0%    0.0%    0.0%    0.0%    0.0%    0.0%    0.0%    0.0%    0.0%    0.0%    0.0%    0.0%    0.0%    0.0%    0.0%    0.0%    0.0%    0.0%    0.0%    0.0%    0.0%    0.0%    0.0%    0.0%    0.0%    0.0%    0.0%    0.0%    0.0%    0.0%    0.0%    0.0%    0.0%    0.0%    0.0%    0.0%    0.0%    0.0%    0.0%    0.0%    0.0%    0.0%    0.0%    0.0%    0.0%    0.0%    0.0%    0.0%    0.0%    0.0%    0.0%    0.0%    0.0%    0.0%    0.0%    0.0%    0.0%    0.0%    0.0%    0.0%    0.0%    0.0%    0.0%    0.0%    0.0%    0.0%    0.0%    0.0%    0.0%    0.0%    0.0%    0.0%    0.0%    0.0%    0.0%    0.0%    0.0%    0.0%    0.0%    0.0%    0.0%    0.0%    0.0%    0.0%    0.0%    0.0%    0.0%    0.0%    0.0%    0.0%    0.0%    0.0%    0.0%    0.0%    0.0%    0.0%    0.0%    0.0%    0.0%    0.0%    0.0%    0.0%    0.0%    0.0%    0.0%    0.0%    0.0%    0.0%    0.0%    0.0%    0.0%    0.0%    0.0%    0.0%    0.0%    0.0%    0.0%    0.0%    0.0%    0.0%    0.0%    0.0%    0.0%    0.0%    0.0%    0.0%        k__Bacteria;p__Planctomycetes;c__agg27; o__CL500-15      17    0.0%    0.0%    0.0%    0.0%    0.0%    0.0%    0.0%    0.0%    0.0%    0.0%    0.0%    0.0%    0.0%    0.0%    0.0%    0.0%    0.0%    0.0%    0.0%    0.0%    0.0%    0.0%    0.0%    0.0%    0.0%    0.0%    0.0%    0.0%    0.0%    0.0%    0.0%    0.0%    0.0%    0.0%    0.0%    0.0%    0.0%    0.0%    0.0%    0.0%    0.0%    0.0%    0.0%    0.0%    0.0%    0.0%    0.0%    0.0%    0.0%    0.0%    0.0%    0.0%    0.0%    0.0%    0.0%    0.0%    0.0%    0.0%    0.0%    0.0%    0.0%    0.0%    0.0%    0.0%    0.0%    0.0%    0.0%    0.0%    0.0%    0.0%    0.0%    0.0%    0.0%    0.0%    0.0%    0.0%    0.0%    0.0%    0.0%    0.0%    0.0%    0.0%    0.0%    0.0%    0.0%    0.0%    0.0%    0.0%    0.0%    0.0%    0.0%    0.0%    0.0%    0.0%    0.0%    0.0%    0.0%    0.0%    0.0%    0.0%    0.0%    0.0%    0.0%    0.0%    0.0%    0.0%    0.0%    0.0%    0.0%    0.0%    0.0%    0.0%    0.0%    0.0%    0.0%    0.0%    0.0%    0.0%    0.0%    0.0%    0.0%    0.0%    0.0%    0.0%    0.0%    0.0%    0.0%    0.0%    0.0%    0.0%    0.0%    0.0%    0.0%    0.0%    0.0%    0.0%    0.0%    0.0%    0.0%    0.0%    0.0%    0.0%    0.0%    0.0%    0.0%    0.0%    0.0%    0.0%    0.0%    0.0%    0.0%    0.0%    0.0%    0.0%    0.0%    0.0%    0.0%    0.0%    0.0%    0.0%    0.0%    0.0%    0.0%    0.0%    0.0%    0.0%    0.0%    0.0%    0.0%    0.0%    0.0%    0.0%    0.0%    0.0%    0.0%    0.0%    0.0%    0.0%    0.0%    0.0%    0.0%    0.0%    0.0%    0.0%    0.0%    0.0%    0.0%    0.0%    0.0%    0.0%    0.0%    0.0%    0.0%    0.0%    0.0%    0.0%    0.0%    0.0%    0.0%    0.0%    0.0%    0.0%    0.0%    0.0%    0.0%    0.0%    0.0%    0.0%    0.0%    0.0%    0.0%    0.0%    0.0%    0.0%    0.0%    0.0%    0.0%    0.0%    0.0%    0.0%    0.0%    0.0%    0.0%    0.0%    0.0%    0.0%    0.0%    0.0%    0.0%    0.0%    0.0%    0.0%    0.0%    0.0%    0.0%    0.0%    0.0%    0.0%    0.0%    0.0%    0.0%    0.0%    0.0%    0.0%    0.0%    0.0%    0.0%    0.0%    0.0%    0.0%    0.0%    0.0%    0.0%    0.0%    0.0%    0.0%    0.0%    0.0%    0.0%    0.0%    0.0%    0.0%    0.0%    0.0%    0.0%    0.0%    0.0%    0.0%    0.0%    0.0%    0.0%    0.0%    0.0%    0.0%    0.0%    0.0%    0.0%    0.0%    0.0%    0.0%    0.0%    0.0%    0.0%    0.0%    0.0%    0.0%    0.0%    0.0%    0.0%    0.0%    0.0%    0.0%    0.0%    0.0%    0.0%    0.0%    0.0%    0.0%    0.0%    0.0%    0.0%    0.0%    0.0%    0.0%    0.0%    0.0%    0.0%    0.0%    0.0%    0.0%    0.0%    0.0%    0.0%    0.0%    0.0%    0.0%    0.0%    0.0%    0.0%    0.0%    0.0%    0.0%    0.0%    0.0%    0.0%    0.0%    0.0%    0.0%    0.0%    0.0%    0.0%    0.0%    0.0%    0.0%    0.0%    0.0%    0.0%    0.0%    0.0%    0.0%    0.0%    0.0%    0.0%    0.0%    0.0%    0.0%    0.0%    0.0%    0.0%    0.0%    0.0%    0.0%    0.0%    0.0%    0.0%    0.0%    0.0%    0.0%        k__Bacteria;p__Planctomycetes;c__agg27; o__OM190      25    0.0%    0.0%    0.0%    0.0%    0.0%    0.0%    0.0%    0.0%    0.0%    0.0%    0.0%    0.0%    0.0%    0.0%    0.0%    0.0%    0.0%    0.0%    0.0%    0.0%    0.0%    0.0%    0.0%    0.0%    0.0%    0.0%    0.0%    0.0%    0.0%    0.0%    0.0%    0.0%    0.0%    0.0%    0.0%    0.0%    0.0%    0.0%    0.0%    0.0%    0.0%    0.0%    0.0%    0.0%    0.0%    0.0%    0.0%    0.0%    0.0%    0.0%    0.0%    0.0%    0.0%    0.0%    0.0%    0.0%    0.0%    0.0%    0.0%    0.0%    0.0%    0.0%    0.0%    0.0%    0.0%    0.0%    0.0%    0.0%    0.0%    0.0%    0.0%    0.0%    0.0%    0.0%    0.0%    0.0%    0.0%    0.0%    0.0%    0.0%    0.0%    0.0%    0.0%    0.0%    0.0%    0.0%    0.0%    0.0%    0.0%    0.0%    0.0%    0.0%    0.0%    0.0%    0.0%    0.0%    0.0%    0.0%    0.0%    0.0%    0.0%    0.0%    0.0%    0.0%    0.0%    0.0%    0.0%    0.0%    0.0%    0.0%    0.0%    0.0%    0.0%    0.0%    0.0%    0.0%    0.0%    0.0%    0.0%    0.0%    0.0%    0.0%    0.0%    0.0%    0.0%    0.0%    0.0%    0.0%    0.0%    0.0%    0.0%    0.0%    0.0%    0.0%    0.0%    0.0%    0.0%    0.0%    0.0%    0.0%    0.0%    0.0%    0.0%    0.0%    0.0%    0.0%    0.0%    0.0%    0.0%    0.0%    0.0%    0.0%    0.0%    0.0%    0.0%    0.0%    0.0%    0.0%    0.0%    0.0%    0.0%    0.0%    0.0%    0.0%    0.0%    0.0%    0.0%    0.0%    0.0%    0.0%    0.0%    0.0%    0.0%    0.0%    0.0%    0.0%    0.0%    0.0%    0.0%    0.0%    0.0%    0.0%    0.0%    0.0%    0.0%    0.0%    0.0%    0.0%    0.0%    0.0%    0.0%    0.0%    0.0%    0.0%    0.0%    0.0%    0.0%    0.0%    0.0%    0.0%    0.0%    0.0%    0.0%    0.0%    0.0%    0.0%    0.0%    0.0%    0.0%    0.0%    0.0%    0.0%    0.0%    0.0%    0.0%    0.0%    0.0%    0.0%    0.0%    0.0%    0.0%    0.0%    0.0%    0.0%    0.0%    0.0%    0.0%    0.0%    0.0%    0.0%    0.0%    0.0%    0.0%    0.0%    0.0%    0.0%    0.0%    0.0%    0.0%    0.0%    0.0%    0.0%    0.0%    0.0%    0.0%    0.0%    0.0%    0.0%    0.0%    0.0%    0.0%    0.0%    0.0%    0.0%    0.0%    0.0%    0.0%    0.0%    0.0%    0.0%    0.0%    0.0%    0.0%    0.0%    0.0%    0.0%    0.0%    0.0%    0.0%    0.0%    0.0%    0.0%    0.0%    0.0%    0.0%    0.0%    0.0%    0.0%    0.0%    0.0%    0.0%    0.0%    0.0%    0.0%    0.0%    0.0%    0.0%    0.0%    0.0%    0.0%    0.0%    0.0%    0.0%    0.0%    0.0%    0.0%    0.0%    0.0%    0.0%    0.0%    0.0%    0.0%    0.0%    0.0%    0.0%    0.0%    0.0%    0.0%    0.0%    0.0%    0.0%    0.0%    0.0%    0.0%    0.0%    0.0%    0.0%    0.0%    0.0%    0.0%    0.0%    0.0%    0.0%    0.0%    0.0%    0.0%    0.0%    0.0%    0.0%    0.0%    0.0%    0.0%    0.0%    0.0%    0.0%    0.0%    0.0%    0.0%    0.0%    0.0%    0.0%    0.0%    0.0%    0.0%    0.0%    0.0%    0.0%    0.0%    0.0%    0.0%    0.0%    0.0%    0.0%    0.0%    0.0%    0.0%    0.0%    0.0%        k__Bacteria;p__Planctomycetes;c__vadinHA49; o__      24    0.0%    0.0%    0.0%    0.0%    0.0%    0.0%    0.0%    0.0%    0.0%    0.0%    0.0%    0.0%    0.0%    0.0%    0.0%    0.0%    0.0%    0.0%    0.0%    0.0%    0.0%    0.0%    0.0%    0.0%    0.0%    0.0%    0.0%    0.0%    0.0%    0.0%    0.0%    0.0%    0.0%    0.0%    0.0%    0.0%    0.0%    0.0%    0.0%    0.0%    0.0%    0.0%    0.0%    0.0%    0.0%    0.0%    0.0%    0.0%    0.0%    0.0%    0.0%    0.0%    0.0%    0.0%    0.0%    0.0%    0.0%    0.0%    0.0%    0.0%    0.0%    0.0%    0.0%    0.0%    0.0%    0.0%    0.0%    0.0%    0.0%    0.0%    0.0%    0.0%    0.0%    0.0%    0.0%    0.0%    0.0%    0.0%    0.0%    0.0%    0.0%    0.0%    0.0%    0.0%    0.0%    0.0%    0.0%    0.0%    0.0%    0.0%    0.0%    0.0%    0.0%    0.0%    0.0%    0.0%    0.0%    0.0%    0.0%    0.0%    0.0%    0.0%    0.0%    0.0%    0.0%    0.0%    0.0%    0.0%    0.0%    0.0%    0.0%    0.0%    0.0%    0.0%    0.0%    0.0%    0.0%    0.0%    0.0%    0.0%    0.0%    0.0%    0.0%    0.0%    0.0%    0.0%    0.0%    0.0%    0.0%    0.0%    0.0%    0.0%    0.0%    0.0%    0.0%    0.0%    0.0%    0.0%    0.0%    0.0%    0.0%    0.0%    0.0%    0.0%    0.0%    0.0%    0.0%    0.0%    0.0%    0.0%    0.0%    0.0%    0.0%    0.0%    0.0%    0.0%    0.0%    0.0%    0.0%    0.0%    0.0%    0.0%    0.0%    0.0%    0.0%    0.0%    0.0%    0.0%    0.0%    0.0%    0.0%    0.0%    0.0%    0.0%    0.0%    0.0%    0.0%    0.0%    0.0%    0.0%    0.0%    0.0%    0.0%    0.0%    0.0%    0.0%    0.0%    0.0%    0.0%    0.0%    0.0%    0.0%    0.0%    0.0%    0.0%    0.0%    0.0%    0.0%    0.0%    0.0%    0.0%    0.0%    0.0%    0.0%    0.0%    0.0%    0.0%    0.0%    0.0%    0.0%    0.0%    0.0%    0.0%    0.0%    0.0%    0.0%    0.0%    0.0%    0.0%    0.0%    0.0%    0.0%    0.0%    0.0%    0.0%    0.0%    0.0%    0.0%    0.0%    0.0%    0.0%    0.0%    0.0%    0.0%    0.0%    0.0%    0.0%    0.0%    0.0%    0.0%    0.0%    0.0%    0.0%    0.0%    0.0%    0.0%    0.0%    0.0%    0.0%    0.0%    0.0%    0.0%    0.0%    0.0%    0.0%    0.0%    0.0%    0.0%    0.0%    0.0%    0.0%    0.0%    0.0%    0.0%    0.0%    0.0%    0.0%    0.0%    0.0%    0.0%    0.0%    0.0%    0.0%    0.0%    0.0%    0.0%    0.0%    0.0%    0.0%    0.0%    0.0%    0.0%    0.0%    0.0%    0.0%    0.0%    0.0%    0.0%    0.0%    0.0%    0.0%    0.0%    0.0%    0.0%    0.0%    0.0%    0.0%    0.0%    0.0%    0.0%    0.0%    0.0%    0.0%    0.0%    0.0%    0.0%    0.0%    0.0%    0.0%    0.0%    0.0%    0.0%    0.0%    0.0%    0.0%    0.0%    0.0%    0.0%    0.0%    0.0%    0.0%    0.0%    0.0%    0.0%    0.0%    0.0%    0.0%    0.0%    0.0%    0.0%    0.0%    0.0%    0.0%    0.0%    0.0%    0.0%    0.0%    0.0%    0.0%    0.0%    0.0%    0.0%    0.0%    0.0%    0.0%    0.0%    0.0%    0.0%    0.0%    0.0%    0.0%    0.0%    0.0%    0.0%    0.0%    0.0%    0.0%    0.0%        k__Bacteria;p__Proteobacteria;c__Alphaproteobacteria; o__     491    0.0%    0.0%    0.0%    0.0%    0.0%    0.0%    0.0%    0.0%    0.0%    0.0%    0.0%    0.0%    0.0%    0.0%    0.0%    0.0%    0.0%    0.0%    0.0%    0.0%    0.0%    0.0%    0.0%    0.0%    0.0%    0.0%    0.0%    0.0%    0.0%    0.0%    0.0%    0.0%    0.0%    0.0%    0.0%    0.0%    0.0%    0.0%    0.0%    0.0%    0.0%    0.0%    0.0%    0.0%    0.0%    0.0%    0.0%    0.0%    0.0%    0.0%    0.0%    0.0%    0.0%    0.0%    0.0%    0.0%    0.0%    0.0%    0.0%    0.0%    0.0%    0.0%    0.0%    0.0%    0.0%    0.0%    0.0%    0.0%    0.0%    0.0%    0.0%    0.0%    0.0%    0.0%    0.0%    0.0%    0.0%    0.0%    0.0%    0.0%    0.0%    0.0%    0.0%    0.0%    0.0%    0.0%    0.0%    0.0%    0.0%    0.0%    0.0%    0.0%    0.0%    0.0%    0.0%    0.0%    0.0%    0.0%    0.0%    0.0%    0.0%    0.0%    0.0%    0.0%    0.0%    0.0%    0.0%    0.0%    0.0%    0.0%    0.0%    0.0%    0.0%    0.0%    0.0%    0.0%    0.0%    0.0%    0.0%    0.0%    0.0%    0.0%    0.0%    0.0%    0.0%    0.0%    0.0%    0.0%    0.0%    0.0%    0.0%    0.0%    0.0%    0.0%    0.0%    0.0%    0.0%    0.0%    0.0%    0.0%    0.0%    0.0%    0.0%    0.0%    0.0%    0.0%    0.0%    0.0%    0.0%    0.0%    0.0%    0.0%    0.0%    0.0%    0.0%    0.0%    0.0%    0.0%    0.0%    0.0%    0.0%    0.0%    0.0%    0.0%    0.0%    0.0%    0.0%    0.0%    0.0%    0.0%    0.0%    0.0%    0.0%    0.0%    0.0%    0.0%    0.0%    0.0%    0.0%    0.0%    0.0%    0.0%    0.0%    0.0%    0.0%    0.0%    0.0%    0.0%    0.0%    0.0%    0.0%    0.0%    0.0%    0.0%    0.0%    0.0%    0.0%    0.0%    0.0%    0.0%    0.0%    0.0%    0.0%    0.0%    0.0%    0.0%    0.0%    0.0%    0.0%    0.0%    0.0%    0.0%    0.0%    0.0%    0.0%    0.0%    0.0%    0.0%    0.0%    0.0%    0.0%    0.0%    0.0%    0.0%    0.0%    0.0%    0.0%    0.0%    0.0%    0.0%    0.0%    0.0%    0.0%    0.0%    0.0%    0.0%    0.0%    0.0%    0.0%    0.0%    0.0%    0.0%    0.0%    0.0%    0.0%    0.0%    0.0%    0.0%    0.0%    0.0%    0.0%    0.0%    0.0%    0.0%    0.0%    0.0%    0.0%    0.0%    0.0%    0.0%    0.0%    0.0%    0.0%    0.0%    0.0%    0.0%    0.0%    0.0%    0.0%    0.0%    0.0%    0.0%    0.0%    0.0%    0.0%    0.0%    0.0%    0.0%    0.0%    0.0%    0.0%    0.0%    0.0%    0.0%    0.0%    0.0%    0.0%    0.1%    0.0%    0.0%    0.0%    0.0%    0.0%    0.0%    0.0%    0.0%    0.0%    0.0%    0.0%    0.0%    0.0%    0.0%    0.0%    0.0%    0.0%    0.0%    0.0%    0.0%    0.0%    0.0%    0.0%    0.0%    0.0%    0.0%    0.0%    0.0%    0.0%    0.0%    0.0%    0.0%    0.0%    0.0%    0.0%    0.0%    0.0%    0.0%    0.0%    0.0%    0.0%    0.0%    0.0%    0.0%    0.0%    0.0%    0.0%    0.0%    0.0%    0.0%    0.0%    0.0%    0.0%    0.0%    0.0%    0.0%    0.0%    0.0%    0.0%    0.0%    0.0%    0.0%    0.0%    0.0%    0.0%    0.0%    0.0%    0.0%    0.0%    0.0%        k__Bacteria;p__Proteobacteria;c__Alphaproteobacteria; o__Caulobacterales   34397    0.2%    0.0%    0.1%    0.0%    0.1%    0.1%    0.0%    0.0%    0.0%    0.0%    0.0%    0.1%    0.1%    0.0%    0.0%    0.2%    0.1%    0.1%    0.0%    0.1%    0.1%    0.2%    0.1%    0.0%    0.1%    0.0%    0.1%    0.1%    0.1%    0.0%    0.0%    0.1%    0.0%    0.0%    0.2%    0.0%    0.1%    0.0%    0.1%    0.2%    0.0%    0.1%    0.0%    0.0%    0.1%    0.1%    0.1%    0.0%    0.0%    0.0%    0.1%    0.3%    0.2%    0.1%    0.1%    0.1%    0.1%    0.0%    0.1%    0.2%    0.1%    0.0%    0.0%    0.3%    0.2%    0.0%    0.1%    0.2%    0.1%    0.1%    0.1%    0.0%    0.0%    0.1%    1.8%    0.0%    0.1%    0.2%    0.1%    0.1%    0.1%    1.3%    0.1%    0.0%    0.0%    0.0%    0.2%    0.0%    0.0%    0.1%    0.0%    0.1%    0.1%    0.0%    0.0%    0.1%    0.2%    0.0%    0.2%    0.1%    0.4%    0.1%    0.1%    0.1%    0.0%    0.0%    0.2%    0.0%    0.1%    0.1%    0.1%    0.1%    0.0%    0.0%    0.0%    0.1%    0.1%    0.1%    0.0%    1.5%    0.0%    0.1%    0.2%    0.1%    0.1%    0.5%    0.2%    0.0%    0.2%    0.3%    0.1%    0.0%    0.1%    0.1%    0.3%    1.6%    8.3%    1.2%    1.4%    0.1%    1.8%    0.1%    0.3%    0.0%    0.2%    0.1%    0.0%    0.1%    0.1%    0.1%    0.0%    0.0%    0.3%    0.1%    0.0%    0.0%    0.5%    0.1%    0.8%    0.2%    0.3%    1.1%    0.1%    0.1%    0.1%    0.1%    0.2%    0.1%    0.5%    0.1%    0.7%    0.3%    0.1%    0.2%    0.0%    0.1%    0.0%    0.0%    0.1%    1.7%    0.0%    0.0%    0.1%    0.0%    0.0%    0.0%    0.0%    0.1%    0.1%    0.1%    0.1%    0.1%    0.1%    0.1%    0.1%    0.2%    0.2%    0.0%    0.0%    0.0%    0.3%    0.1%    0.1%    0.3%    0.2%    0.2%    3.3%    0.2%    0.5%    0.2%    0.3%    0.1%    0.2%    0.1%    0.1%    0.3%    0.3%    0.1%    0.1%    0.1%    0.1%    0.3%    0.0%    0.2%    0.1%    0.1%    0.0%    0.0%    0.2%    0.1%    0.1%    0.0%    0.5%    0.1%    0.3%    0.0%    0.3%    0.1%    0.1%    0.1%    0.8%    0.1%    0.0%    0.0%    0.1%    0.0%    0.0%    0.2%    0.0%    0.1%    0.3%    0.1%    0.1%    0.1%    0.2%    0.1%    0.1%    0.2%    0.1%    0.0%    0.7%    0.2%    0.2%    0.1%    0.1%    0.1%    0.3%    0.1%    0.2%    0.1%    0.1%    0.2%    0.4%    0.3%    0.1%    0.1%    0.2%    0.2%    0.2%    0.0%    0.0%    0.1%    0.1%    0.0%    0.1%    0.1%    0.2%    0.5%    0.1%    0.0%    0.3%    0.0%    0.2%    0.2%    0.1%    0.1%    0.2%    0.2%    0.0%    0.2%    0.1%    0.0%    0.1%    0.2%    0.1%    0.0%    0.1%    0.0%    0.1%    0.0%    0.0%    0.3%    0.0%    0.0%    0.1%    0.2%    0.0%    0.0%    0.0%    0.2%    0.1%    0.1%    0.0%    0.1%    0.2%    0.2%    0.2%    0.1%    0.1%    0.1%    0.0%    0.0%    0.0%    0.1%    0.1%    0.0%    0.0%    0.0%    0.1%    0.0%    0.0%    0.0%    0.0%    0.4%    0.2%    0.0%    0.0%    0.0%    0.0%    0.0%    0.2%    0.0%    0.1%    0.2%    0.0%    0.1%    0.0%    0.0%        k__Bacteria;p__Proteobacteria;c__Alphaproteobacteria; o__Rhizobiales   136825    0.8%    0.3%    0.5%    0.1%    0.5%    1.6%    0.1%    0.1%    0.0%    0.0%    0.1%    0.1%    0.6%    0.2%    0.1%    0.4%    0.4%    1.1%    0.2%    0.5%    0.8%    1.2%    0.5%    0.0%    0.1%    0.2%    0.8%    0.4%    0.8%    0.1%    0.1%    0.8%    0.4%    0.1%    1.2%    0.1%    0.8%    0.0%    0.4%    1.2%    0.3%    0.8%    0.1%    0.2%    0.6%    0.3%    0.3%    0.1%    0.6%    0.1%    0.9%    2.1%    2.3%    1.1%    0.5%    0.3%    0.7%    0.2%    0.7%    1.2%    0.6%    0.5%    0.2%    1.7%    1.2%    0.5%    1.7%    1.4%    0.8%    0.8%    0.8%    0.3%    0.3%    0.5%    1.4%    0.1%    0.4%    0.8%    0.2%    0.5%    0.2%    5.2%    0.3%    0.3%    0.1%    0.0%    1.2%    0.5%    0.3%    1.0%    0.0%    0.7%    0.7%    0.2%    0.2%    0.6%    1.6%    0.2%    0.6%    0.6%    2.5%    0.6%    0.4%    0.4%    0.1%    0.1%    1.8%    1.2%    0.5%    0.7%    0.4%    1.0%    0.1%    0.0%    0.5%    0.2%    0.4%    0.6%    0.4%    4.3%    0.4%    0.4%    1.2%    0.7%    0.2%    2.3%    1.1%    0.6%    1.2%    2.0%    1.0%    0.6%    0.9%    0.4%    2.4%    3.0%    5.8%    1.1%    0.2%    0.7%    0.1%    0.7%    1.0%    0.7%    1.3%    0.4%    0.1%    0.2%    0.4%    1.3%    0.7%    0.4%    1.3%    0.9%    0.2%    0.3%    2.0%    0.3%    0.3%    1.6%    2.0%    1.6%    0.9%    0.3%    0.8%    0.4%    0.9%    0.4%    0.8%    0.2%    2.6%    1.0%    1.2%    0.8%    0.5%    0.2%    0.5%    0.2%    0.6%    2.7%    0.3%    0.1%    0.5%    0.3%    0.2%    0.1%    0.2%    0.2%    0.2%    0.2%    0.2%    0.3%    0.1%    0.4%    0.4%    0.8%    1.1%    0.2%    0.3%    0.2%    1.1%    0.4%    0.7%    1.6%    1.5%    0.8%    4.7%    1.2%    2.7%    1.8%    1.2%    0.5%    1.2%    0.4%    0.5%    1.2%    1.4%    0.7%    1.5%    0.8%    0.8%    3.1%    0.1%    1.8%    0.9%    1.1%    0.2%    0.4%    1.3%    1.0%    0.3%    0.6%    3.7%    0.4%    2.3%    0.7%    2.0%    0.9%    0.2%    1.5%    0.7%    0.7%    0.3%    0.1%    0.2%    0.2%    0.2%    0.5%    0.3%    0.6%    2.1%    1.5%    1.3%    0.4%    0.9%    0.5%    0.6%    0.7%    0.8%    0.4%    2.8%    1.1%    1.5%   16.9%    0.9%    0.6%    1.2%    0.8%    0.7%    0.6%    0.8%    1.5%    3.1%    1.6%    0.5%    0.4%    0.3%    0.4%    0.4%    0.4%    0.5%    0.5%    0.4%    0.1%    0.8%    0.2%    0.5%    5.1%    0.7%    1.0%    0.4%    0.2%    1.2%    1.3%    0.6%    0.2%    1.0%    0.8%    0.4%    1.2%    0.5%    0.5%    0.2%    1.7%    0.4%    0.1%    0.5%    0.4%    0.6%    0.1%    0.1%    0.8%    0.4%    0.9%    0.4%    1.8%    0.5%    0.2%    0.1%    0.8%    0.4%    0.7%    0.1%    0.8%    1.0%    0.4%    0.8%    0.3%    0.9%    0.7%    0.3%    0.2%    0.3%    0.2%    0.2%    0.1%    0.0%    0.1%    0.2%    0.5%    0.5%    0.3%    0.1%    0.8%    1.0%    0.1%    0.2%    0.1%    0.0%    0.2%    0.6%    0.1%    0.3%    0.8%    0.1%    0.1%    2.7%    0.2%        k__Bacteria;p__Proteobacteria;c__Alphaproteobacteria; o__Rhodobacterales   69773    0.4%    0.1%    0.4%    0.1%    0.4%    0.1%    0.0%    0.1%    0.0%    0.0%    0.0%    0.1%    0.2%    0.2%    0.0%    0.3%    0.3%    0.7%    0.1%    0.3%    0.5%    0.7%    0.5%    0.0%    0.1%    0.1%    0.3%    0.3%    0.5%    0.1%    0.1%    0.6%    0.2%    0.0%    0.7%    0.1%    0.6%    0.0%    0.2%    1.1%    0.2%    0.7%    0.0%    0.1%    0.5%    0.2%    0.3%    0.1%    0.1%    0.1%    0.5%    0.3%    0.2%    0.1%    0.8%    0.4%    0.8%    0.4%    0.3%    0.3%    0.3%    0.6%    0.3%    0.9%    0.8%    0.4%    0.2%    1.0%    0.4%    0.6%    0.3%    0.1%    0.2%    0.5%    1.3%    0.0%    0.2%    0.5%    0.1%    0.4%    0.1%    0.7%    0.2%    0.1%    0.1%    0.0%    0.6%    0.1%    0.2%    0.6%    0.0%    0.6%    0.5%    0.2%    0.1%    1.5%    0.5%    0.0%    0.5%    0.4%    1.2%    0.2%    0.2%    0.4%    0.0%    0.0%    0.8%    0.2%    0.0%    0.5%    0.5%    0.8%    0.1%    0.0%    0.3%    0.2%    0.1%    0.2%    0.2%    2.6%    0.0%    0.3%    0.9%    0.5%    0.1%    2.2%    0.4%    0.2%    0.7%    0.9%    0.5%    0.4%    0.4%    0.2%    1.8%    0.1%    0.7%    1.0%    0.1%    0.2%    0.0%    0.3%    0.5%    0.2%    0.8%    0.2%    0.0%    0.0%    0.4%    0.1%    0.2%    0.2%    1.1%    0.2%    0.1%    0.1%    0.9%    0.2%    0.2%    0.8%    1.1%    1.7%    0.6%    0.2%    0.3%    0.4%    0.6%    0.3%    0.5%    0.3%    1.9%    0.8%    0.4%    0.5%    0.1%    0.1%    0.3%    0.1%    0.1%    0.1%    0.2%    0.1%    0.2%    0.1%    0.1%    0.2%    0.2%    0.1%    0.1%    0.2%    0.4%    0.3%    0.2%    0.1%    0.2%    1.3%    0.7%    0.2%    0.3%    0.0%    0.6%    0.4%    0.2%    0.9%    3.7%    0.4%    0.5%    0.6%    2.1%    0.7%    0.7%    0.1%    0.6%    1.0%    0.2%    0.6%    0.5%    0.3%    0.9%    1.6%    0.6%    0.8%    0.0%    1.0%    0.3%    0.6%    0.1%    0.2%    0.8%    0.7%    0.1%    0.1%    0.7%    0.2%    1.2%    0.0%    0.8%    1.4%    0.2%    0.6%    0.6%    0.6%    0.0%    0.1%    0.1%    0.1%    0.4%    0.3%    0.1%    0.2%    1.3%    0.3%    0.2%    0.1%    0.1%    0.1%    0.2%    0.2%    0.4%    0.2%    0.5%    0.5%    0.4%    0.4%    0.2%    0.1%    0.4%    0.4%    0.4%    0.2%    0.3%    1.0%    1.3%    0.7%    0.1%    0.1%    0.1%    0.4%    0.4%    0.1%    0.1%    0.2%    0.1%    0.0%    0.4%    0.1%    0.3%    2.5%    0.6%    1.3%    0.3%    0.1%    0.8%    0.8%    0.4%    0.1%    0.4%    0.6%    0.3%    1.1%    0.5%    0.5%    0.2%    0.6%    0.4%    0.1%    0.2%    0.2%    0.2%    0.0%    0.1%    0.3%    0.4%    0.9%    0.3%    1.5%    0.1%    0.2%    0.1%    0.4%    0.2%    0.3%    0.1%    0.2%    0.6%    0.8%    0.5%    0.1%    0.7%    0.4%    0.6%    0.0%    0.8%    0.1%    0.1%    0.0%    0.0%    0.1%    0.2%    0.0%    0.4%    0.0%    0.1%    0.1%    0.7%    0.1%    0.1%    0.0%    0.0%    0.1%    0.4%    0.1%    0.5%    2.0%    0.2%    0.0%    0.5%    0.8%        k__Bacteria;p__Proteobacteria;c__Alphaproteobacteria; o__Rhodospirillales   38040    0.2%    0.0%    0.1%    0.1%    0.2%    0.0%    0.0%    0.0%    0.0%    0.0%    0.0%    0.0%    0.1%    0.1%    0.0%    0.1%    0.1%    0.3%    0.1%    0.2%    0.2%    0.4%    0.1%    0.0%    0.0%    0.0%    0.2%    0.1%    0.1%    0.0%    0.0%    0.2%    0.2%    0.0%    0.2%    0.0%    0.2%    0.0%    0.1%    0.4%    0.1%    0.2%    0.0%    0.0%    0.3%    0.2%    0.1%    0.0%    0.0%    0.0%    0.4%    0.2%    0.1%    0.1%    0.3%    0.2%    0.6%    0.1%    0.2%    0.2%    0.3%    0.2%    0.1%    0.4%    0.8%    0.2%    0.1%    0.7%    0.2%    0.5%    0.5%    0.2%    0.4%    0.2%    0.4%    0.2%    0.1%    0.3%    0.1%    0.2%    0.1%    1.1%    0.2%    0.1%    0.0%    0.1%    0.3%    0.0%    0.0%    0.3%    0.0%    0.7%    0.2%    0.1%    0.1%    0.3%    0.3%    0.0%    0.3%    0.2%    0.4%    0.1%    0.1%    0.1%    0.0%    0.0%    0.4%    0.0%    0.0%    0.3%    0.2%    0.4%    0.0%    0.0%    0.2%    0.1%    0.1%    0.0%    0.1%    0.9%    0.0%    0.2%    0.5%    0.3%    0.1%    0.6%    0.8%    0.1%    0.5%    0.8%    0.3%    0.2%    0.3%    0.1%    0.8%    0.5%    2.1%    0.3%    0.1%    0.1%    0.0%    0.1%    0.2%    0.1%    0.4%    0.1%    0.0%    0.1%    0.2%    0.0%    0.1%    0.1%    0.4%    0.2%    0.1%    0.1%    0.4%    0.1%    0.1%    0.9%    0.9%    0.6%    0.3%    0.1%    0.1%    0.1%    0.3%    0.2%    0.3%    0.2%    0.5%    0.3%    0.3%    0.3%    0.1%    0.0%    0.1%    0.0%    0.1%    0.4%    0.0%    0.1%    0.2%    0.1%    0.1%    0.2%    0.1%    0.0%    0.1%    0.4%    0.1%    0.1%    0.0%    0.1%    0.1%    0.2%    0.4%    0.1%    0.1%    0.0%    0.2%    0.1%    0.2%    0.5%    0.9%    0.2%    0.6%    0.5%    1.3%    0.4%    0.5%    0.1%    0.2%    0.3%    0.2%    0.2%    0.3%    0.1%    0.5%    0.2%    0.6%    0.4%    0.0%    0.5%    0.3%    0.5%    0.0%    0.1%    0.5%    0.3%    0.1%    0.1%    0.5%    0.1%    0.6%    0.1%    0.4%    0.3%    0.1%    0.2%    0.3%    0.9%    0.0%    0.0%    0.0%    0.1%    0.0%    0.1%    0.3%    0.2%    0.9%    0.2%    0.1%    0.4%    0.1%    0.1%    0.1%    0.1%    0.2%    0.1%    2.2%    0.2%    0.2%    0.2%    0.3%    0.2%    0.2%    0.1%    0.2%    0.2%    0.2%    0.3%    0.4%    0.3%    0.2%    0.2%    0.1%    0.2%    0.1%    0.0%    0.1%    0.1%    0.1%    0.1%    0.1%    0.0%    0.1%    1.1%    0.0%    2.1%    0.2%    0.0%    0.1%    0.2%    0.3%    0.0%    0.1%    0.2%    0.3%    0.6%    0.3%    0.4%    0.1%    0.3%    0.3%    0.1%    0.2%    0.1%    0.2%    0.0%    0.0%    0.8%    0.3%    0.0%    0.0%    0.8%    0.3%    0.0%    0.0%    0.1%    0.1%    0.2%    0.0%    0.2%    0.2%    0.6%    0.2%    0.2%    0.3%    0.2%    0.0%    0.1%    0.0%    0.1%    0.0%    0.0%    0.0%    0.1%    0.2%    0.0%    0.2%    0.0%    0.1%    0.2%    0.2%    0.1%    0.0%    0.0%    0.0%    0.1%    0.3%    0.0%    0.2%    0.8%    0.2%    0.0%    0.0%    0.0%        k__Bacteria;p__Proteobacteria;c__Alphaproteobacteria; o__Rickettsiales     327    0.0%    0.0%    0.0%    0.0%    0.0%    0.0%    0.0%    0.0%    0.0%    0.0%    0.0%    0.0%    0.0%    0.0%    0.0%    0.0%    0.0%    0.0%    0.0%    0.0%    0.0%    0.0%    0.0%    0.0%    0.0%    0.0%    0.0%    0.0%    0.0%    0.0%    0.0%    0.0%    0.0%    0.0%    0.0%    0.0%    0.0%    0.0%    0.0%    0.0%    0.0%    0.0%    0.0%    0.0%    0.0%    0.0%    0.0%    0.0%    0.0%    0.0%    0.0%    0.0%    0.0%    0.0%    0.0%    0.0%    0.0%    0.0%    0.0%    0.0%    0.0%    0.0%    0.0%    0.0%    0.0%    0.0%    0.0%    0.0%    0.0%    0.0%    0.0%    0.0%    0.0%    0.0%    0.0%    0.0%    0.0%    0.0%    0.0%    0.0%    0.0%    0.0%    0.0%    0.0%    0.0%    0.0%    0.0%    0.0%    0.0%    0.0%    0.0%    0.0%    0.0%    0.0%    0.0%    0.0%    0.0%    0.0%    0.0%    0.0%    0.0%    0.0%    0.0%    0.0%    0.0%    0.0%    0.0%    0.0%    0.0%    0.0%    0.0%    0.0%    0.0%    0.0%    0.0%    0.0%    0.0%    0.0%    0.0%    0.0%    0.0%    0.0%    0.0%    0.0%    0.0%    0.0%    0.0%    0.0%    0.0%    0.0%    0.0%    0.0%    0.0%    0.0%    0.0%    0.0%    0.0%    0.0%    0.0%    0.0%    0.0%    0.0%    0.0%    0.0%    0.0%    0.0%    0.0%    0.0%    0.0%    0.0%    0.0%    0.0%    0.0%    0.0%    0.0%    0.0%    0.0%    0.0%    0.0%    0.0%    0.0%    0.0%    0.0%    0.0%    0.0%    0.0%    0.0%    0.0%    0.0%    0.0%    0.0%    0.0%    0.0%    0.0%    0.0%    0.0%    0.0%    0.0%    0.0%    0.0%    0.0%    0.0%    0.0%    0.0%    0.0%    0.0%    0.0%    0.0%    0.0%    0.0%    0.0%    0.0%    0.0%    0.0%    0.0%    0.0%    0.0%    0.0%    0.0%    0.0%    0.0%    0.0%    0.0%    0.0%    0.0%    0.0%    0.0%    0.0%    0.0%    0.0%    0.0%    0.0%    0.0%    0.0%    0.0%    0.0%    0.0%    0.0%    0.0%    0.0%    0.0%    0.0%    0.0%    0.0%    0.0%    0.0%    0.0%    0.0%    0.0%    0.0%    0.0%    0.0%    0.0%    0.0%    0.0%    0.0%    0.0%    0.0%    0.0%    0.0%    0.0%    0.0%    0.0%    0.0%    0.0%    0.0%    0.0%    0.0%    0.0%    0.0%    0.0%    0.0%    0.0%    0.0%    0.0%    0.0%    0.0%    0.0%    0.0%    0.0%    0.0%    0.0%    0.0%    0.0%    0.0%    0.0%    0.0%    0.0%    0.0%    0.0%    0.0%    0.0%    0.0%    0.0%    0.0%    0.0%    0.0%    0.0%    0.0%    0.0%    0.0%    0.0%    0.0%    0.0%    0.0%    0.0%    0.0%    0.0%    0.0%    0.0%    0.0%    0.0%    0.0%    0.0%    0.0%    0.0%    0.0%    0.0%    0.0%    0.0%    0.0%    0.0%    0.0%    0.0%    0.0%    0.0%    0.0%    0.0%    0.0%    0.0%    0.0%    0.0%    0.0%    0.0%    0.0%    0.0%    0.0%    0.0%    0.0%    0.0%    0.0%    0.0%    0.0%    0.0%    0.0%    0.0%    0.0%    0.0%    0.0%    0.0%    0.0%    0.0%    0.0%    0.0%    0.0%    0.0%    0.0%    0.0%    0.0%    0.0%    0.0%    0.0%    0.0%    0.0%    0.0%    0.0%    0.0%    0.0%    0.0%    0.0%    0.0%    0.0%    0.0%    0.0%    0.0%    0.0%    0.0%    0.0%        k__Bacteria;p__Proteobacteria;c__Alphaproteobacteria; o__Sphingomonadales   279033    1.6%    0.1%    0.7%    0.3%    0.7%    0.4%    0.1%    0.1%    0.0%    0.0%    0.1%    0.3%    0.6%    0.2%    0.1%    0.5%    0.5%    1.2%    0.3%    0.8%    0.9%    1.1%    1.3%    0.0%    0.2%    0.2%    0.8%    0.5%    1.1%    0.1%    0.1%    0.9%    0.4%    0.1%    1.3%    0.1%    0.8%    0.0%    0.5%    1.3%    0.3%    0.7%    0.1%    0.1%    0.7%    0.2%    0.3%    0.1%    0.4%    0.1%    0.8%    2.0%    2.4%    0.2%    0.8%    0.3%    0.7%    0.2%    0.8%    1.0%    0.6%    0.7%    0.3%    2.4%    1.2%    1.0%    0.6%    2.2%    0.5%    0.8%    1.1%    0.4%    0.4%    0.8%    1.3%    0.1%    0.4%    1.0%    0.3%    0.6%    0.3%    4.8%    0.4%    0.4%    0.3%    0.1%    1.4%    0.2%    0.2%    1.6%    0.1%    0.9%    0.8%    0.2%    0.3%    0.8%    1.7%    0.5%    1.1%    0.7%    2.0%    4.8%    4.7%    0.8%    0.1%    0.1%    1.3%    0.3%    0.2%    0.9%    0.5%    1.0%    0.3%    0.0%    0.5%    0.4%    2.9%    1.4%    0.4%    4.5%    0.0%    0.7%    1.4%    0.8%   21.0%    2.9%    1.4%    2.2%    1.2%    1.8%    1.0%   16.8%    2.0%    0.5%    1.8%    8.0%    6.9%    1.9%    3.4%    3.0%    0.3%    1.8%    2.6%    1.0%    2.3%    2.7%    1.4%    3.8%    0.6%    0.9%    4.0%    1.2%    4.4%    6.3%    0.2%    0.2%    1.5%    0.5%   15.1%    2.2%    2.4%    3.0%    1.1%    8.9%    5.5%    6.6%    1.9%    0.8%    1.9%    0.4%    2.4%    1.4%    1.3%    0.9%    3.4%    0.7%    0.4%    0.1%    0.7%   10.2%    0.3%    0.2%    1.5%    0.9%    0.4%    0.4%    0.3%    0.2%    0.2%    0.3%    0.3%    0.4%    0.2%    1.2%    0.7%    3.6%    1.5%    0.2%    0.6%    0.2%    1.0%    0.6%    0.8%    3.1%    2.4%    2.8%    3.2%    1.4%    3.8%    2.0%    1.6%    5.7%    5.0%    0.5%    1.4%    1.2%    4.0%    0.6%    3.5%    1.4%    1.3%    5.5%    0.2%    3.2%    7.1%    2.0%    1.3%    1.4%    1.7%    1.2%    4.3%    5.5%    4.1%    2.3%    2.6%    1.6%    2.5%    6.6%    2.4%    2.1%    0.9%    1.6%    0.6%    0.2%    0.5%    0.3%    0.4%    3.0%    1.1%    1.3%    5.1%    1.0%    1.1%    1.4%    0.9%    2.5%    2.2%    8.4%    2.0%    1.4%    5.4%    3.0%    1.9%    2.2%    3.9%    0.9%    2.9%    1.9%    2.2%    5.5%    2.4%    1.4%    5.0%    1.8%    5.9%    2.9%    1.2%    0.7%    0.9%    0.3%    0.5%    1.3%    3.2%    0.5%    5.0%    0.2%    0.4%    4.7%    1.5%    7.7%    1.1%    0.2%    1.4%    1.2%    1.0%    0.3%    0.6%    1.0%    1.1%    1.1%    0.5%    1.3%    0.3%    1.8%    0.8%    0.1%    0.5%    0.3%    1.0%    0.1%    1.3%    1.8%    0.2%    0.1%    0.3%    3.2%    0.1%    0.2%    0.0%    0.3%    0.4%    0.8%    0.2%    1.1%    1.3%    1.7%    0.8%    0.5%    0.9%    0.9%    0.2%    0.0%    0.0%    0.4%    0.3%    0.0%    0.0%    0.2%    0.6%    0.1%    0.8%    0.3%    0.3%    2.3%    1.6%    0.3%    0.3%    0.2%    0.3%    0.1%    0.8%    0.1%    0.5%    1.3%    0.3%    0.1%    1.1%    0.7%        k__Bacteria;p__Proteobacteria;c__Betaproteobacteria; o__    2509    0.0%    0.0%    0.0%    0.0%    0.0%    0.0%    0.0%    0.0%    0.0%    0.0%    0.0%    0.0%    0.0%    0.0%    0.0%    0.0%    0.0%    0.0%    0.0%    0.0%    0.0%    0.0%    0.0%    0.0%    0.0%    0.0%    0.0%    0.0%    0.0%    0.0%    0.0%    0.0%    0.0%    0.0%    0.0%    0.0%    0.0%    0.0%    0.0%    0.0%    0.0%    0.0%    0.0%    0.0%    0.0%    0.0%    0.0%    0.0%    0.0%    0.0%    0.0%    0.1%    0.1%    0.0%    0.0%    0.1%    0.0%    0.0%    0.0%    0.0%    0.0%    0.0%    0.0%    0.0%    0.0%    0.0%    0.0%    0.0%    0.0%    0.0%    0.0%    0.0%    0.0%    0.0%    0.0%    0.0%    0.0%    0.0%    0.0%    0.0%    0.0%    0.0%    0.0%    0.0%    0.0%    0.0%    0.0%    0.0%    0.0%    0.1%    0.0%    0.0%    0.0%    0.0%    0.0%    0.0%    0.0%    0.0%    0.0%    0.0%    0.1%    0.0%    0.0%    0.0%    0.0%    0.0%    0.0%    0.0%    0.0%    0.0%    0.0%    0.0%    0.0%    0.0%    0.0%    0.0%    0.0%    0.0%    0.0%    0.0%    0.0%    0.0%    0.0%    0.0%    0.0%    0.3%    0.1%    0.0%    0.1%    0.1%    0.0%    0.0%    0.0%    0.0%    0.1%    0.0%    0.0%    0.0%    0.1%    0.0%    0.1%    0.0%    0.0%    0.1%    0.1%    0.0%    0.0%    0.0%    0.0%    0.0%    0.0%    0.0%    0.0%    0.0%    0.0%    0.0%    0.0%    0.0%    0.0%    0.0%    0.1%    0.0%    0.0%    0.0%    0.0%    0.0%    0.0%    0.0%    0.0%    0.0%    0.0%    0.0%    0.0%    0.0%    0.0%    0.0%    0.0%    0.0%    0.0%    0.0%    0.0%    0.0%    0.0%    0.0%    0.0%    0.0%    0.0%    0.0%    0.0%    0.0%    0.0%    0.0%    0.0%    0.0%    0.0%    0.0%    0.0%    0.0%    0.0%    0.0%    0.0%    0.0%    0.0%    0.0%    0.0%    0.0%    0.0%    0.0%    0.0%    0.0%    0.0%    0.0%    0.1%    0.0%    0.0%    0.0%    0.0%    0.0%    0.0%    0.0%    0.0%    0.0%    0.0%    0.0%    0.0%    0.0%    0.0%    0.0%    0.0%    0.0%    0.0%    0.0%    0.0%    0.0%    0.0%    0.0%    0.0%    0.0%    0.0%    0.0%    0.0%    0.0%    0.0%    0.0%    0.0%    0.0%    0.0%    0.0%    0.0%    0.0%    0.0%    0.0%    0.0%    0.0%    0.0%    0.0%    0.0%    0.0%    0.0%    0.0%    0.0%    0.1%    0.0%    0.0%    0.0%    0.0%    0.0%    0.0%    0.0%    0.0%    0.0%    0.0%    0.1%    0.0%    0.0%    0.0%    0.0%    0.0%    0.0%    0.0%    0.0%    0.0%    0.0%    0.0%    0.0%    0.0%    0.0%    0.0%    0.0%    0.2%    0.0%    0.0%    0.0%    0.1%    0.0%    0.0%    0.0%    0.0%    0.0%    0.0%    0.0%    0.0%    0.0%    0.1%    0.0%    0.0%    0.0%    0.0%    0.0%    0.0%    0.0%    0.0%    0.0%    0.0%    0.0%    0.0%    0.0%    0.0%    0.0%    0.0%    0.0%    0.0%    0.0%    0.0%    0.0%    0.0%    0.0%    0.0%    0.0%    0.0%    0.0%    0.0%    0.0%    0.0%    0.0%    0.0%    0.0%    0.0%    0.0%    0.0%    0.0%    0.0%    0.0%    0.0%    0.0%    0.0%    0.0%    0.0%    0.0%    0.0%    0.0%    0.0%    0.0%    0.0%    0.0%    0.0%    0.0%    0.0%        k__Bacteria;p__Proteobacteria;c__Betaproteobacteria; o__Burkholderiales   823074    4.8%    0.9%    0.8%    0.3%    2.0%    1.2%    0.3%   60.1%    1.2%    1.2%    2.9%   56.6%    1.1%    2.3%    1.7%    2.3%    1.3%    1.6%    1.1%    1.3%    1.6%    2.0%    2.9%    0.2%    0.6%    0.7%    1.3%    2.0%    1.7%    0.3%    0.4%    1.4%    1.1%    1.1%    2.4%    1.2%    2.0%    0.1%    2.2%    3.3%    2.2%    3.1%    3.0%    1.9%    1.4%    1.1%    1.7%    0.4%    1.7%    1.0%    2.5%   13.8%   17.7%    1.0%    1.6%    0.5%    1.5%    0.4%    5.7%    1.2%    1.1%    1.0%    0.9%    1.9%    3.2%    0.7%    0.4%    2.6%    0.5%    1.0%    5.0%    0.9%    0.6%    3.8%    2.0%    0.4%    1.6%    1.5%    0.6%    0.8%    0.4%    3.2%    0.5%    0.6%    1.3%    0.5%    2.9%    0.8%    0.6%    4.2%    0.6%    1.2%    1.4%    0.5%    0.6%    1.3%    1.9%    2.7%    1.9%    1.5%    1.9%   22.6%   22.2%    1.3%    0.6%    1.5%    2.6%    2.6%    1.4%    2.2%    1.5%    2.6%    2.2%    0.2%    1.1%    0.7%   12.0%    2.0%    1.0%    5.3%    0.4%    1.1%    2.2%    1.4%    0.6%   17.5%    2.8%    4.2%    2.3%    4.3%    1.9%   15.1%    9.7%    1.0%    3.5%   23.9%    4.8%   10.1%   22.2%   16.6%   18.5%   10.1%   16.9%   17.1%   12.2%   16.8%   10.9%   18.1%    2.2%    1.0%   19.3%    3.8%    3.9%   31.3%    0.7%    1.0%    7.5%    1.2%    7.9%    3.3%    4.0%    4.2%    6.4%    1.3%   27.1%    1.1%    4.2%    1.6%    2.2%    1.1%    5.5%    4.8%    1.7%    2.4%   16.6%    3.5%    1.2%    0.6%    1.5%    2.4%    0.9%    0.5%   10.8%    5.0%    2.0%    0.7%    0.7%    0.7%    0.5%    0.4%    0.4%    0.8%    0.4%    7.3%    1.7%   18.9%    3.3%    1.1%    0.5%    0.4%    3.3%    1.5%    1.2%    3.3%    2.6%    9.0%    1.0%    2.4%    4.4%    2.4%    1.7%   26.2%   22.3%    4.1%    6.9%    1.4%   23.2%    2.8%    4.1%    4.2%    2.2%   19.5%    1.2%   11.6%   23.7%    2.5%    5.5%    7.1%    2.3%    1.7%   14.8%   22.5%    8.8%   12.6%    4.0%    1.6%    3.5%   33.1%   12.4%    2.9%    1.8%    7.2%    4.7%    1.5%    1.4%    0.5%    1.1%    9.7%    4.2%    4.1%   20.4%    4.1%    4.3%   10.0%    4.9%   16.4%   10.8%   41.4%    7.4%    8.3%   13.9%   11.3%    4.9%    8.5%   18.4%    2.5%    5.5%    6.8%    9.1%   22.5%    8.2%    2.2%   14.2%    2.2%   29.4%   14.0%    4.6%    1.6%    1.3%    0.3%    0.6%    3.6%   15.2%    2.1%   22.4%    0.8%    1.3%    4.0%    0.7%    9.4%    4.7%    0.6%    1.8%    1.5%    2.4%    0.3%    1.3%    1.0%    1.1%    2.0%    0.6%    0.7%    0.4%    1.4%    1.9%    1.1%    1.0%    0.6%    0.7%    4.2%    4.4%    3.7%    0.4%    0.5%    0.8%    3.6%    0.3%    2.3%    0.9%    1.3%    2.4%    2.5%    0.6%    0.5%    2.1%    2.3%    2.0%    2.1%    3.0%    1.9%    1.3%    0.4%    0.2%    0.2%    0.2%    0.3%    1.5%    1.4%    3.8%    0.0%    1.2%    0.3%    1.5%    1.1%    2.3%    1.1%    0.4%    0.5%    0.4%    0.3%    1.5%    3.3%    0.8%    2.2%    0.7%    0.3%    2.2%    0.0%        k__Bacteria;p__Proteobacteria;c__Betaproteobacteria; o__Gallionellales     472    0.0%    0.0%    0.0%    0.0%    0.0%    0.0%    0.0%    0.0%    0.0%    0.0%    0.0%    0.0%    0.0%    0.0%    0.0%    0.0%    0.0%    0.0%    0.0%    0.0%    0.0%    0.0%    0.0%    0.0%    0.0%    0.0%    0.0%    0.0%    0.0%    0.0%    0.0%    0.0%    0.0%    0.0%    0.0%    0.0%    0.0%    0.0%    0.0%    0.0%    0.0%    0.0%    0.0%    0.0%    0.0%    0.0%    0.0%    0.0%    0.0%    0.0%    0.0%    0.0%    0.0%    0.0%    0.0%    0.0%    0.0%    0.0%    0.0%    0.0%    0.0%    0.0%    0.0%    0.0%    0.0%    0.0%    0.0%    0.0%    0.0%    0.0%    0.0%    0.0%    0.0%    0.0%    0.0%    0.0%    0.0%    0.0%    0.0%    0.0%    0.0%    0.0%    0.0%    0.0%    0.0%    0.0%    0.0%    0.0%    0.0%    0.0%    0.0%    0.0%    0.0%    0.0%    0.0%    0.0%    0.0%    0.0%    0.0%    0.0%    0.0%    0.0%    0.0%    0.0%    0.0%    0.0%    0.0%    0.0%    0.0%    0.0%    0.0%    0.0%    0.0%    0.0%    0.0%    0.0%    0.0%    0.0%    0.0%    0.0%    0.0%    0.0%    0.0%    0.0%    0.0%    0.1%    0.0%    0.0%    0.0%    0.0%    0.0%    0.0%    0.0%    0.0%    0.0%    0.0%    0.0%    0.0%    0.0%    0.0%    0.0%    0.0%    0.0%    0.0%    0.0%    0.0%    0.0%    0.0%    0.0%    0.0%    0.0%    0.0%    0.0%    0.0%    0.0%    0.0%    0.0%    0.0%    0.0%    0.0%    0.0%    0.0%    0.0%    0.0%    0.0%    0.0%    0.0%    0.0%    0.0%    0.0%    0.0%    0.0%    0.0%    0.0%    0.0%    0.0%    0.0%    0.0%    0.0%    0.0%    0.0%    0.0%    0.0%    0.0%    0.0%    0.0%    0.0%    0.0%    0.0%    0.0%    0.0%    0.0%    0.0%    0.0%    0.0%    0.0%    0.0%    0.0%    0.0%    0.0%    0.0%    0.0%    0.0%    0.0%    0.0%    0.0%    0.0%    0.0%    0.0%    0.0%    0.0%    0.0%    0.0%    0.0%    0.0%    0.0%    0.0%    0.0%    0.0%    0.0%    0.0%    0.0%    0.0%    0.0%    0.0%    0.0%    0.0%    0.0%    0.0%    0.0%    0.0%    0.0%    0.0%    0.0%    0.0%    0.0%    0.0%    0.0%    0.0%    0.0%    0.0%    0.0%    0.0%    0.0%    0.0%    0.0%    0.0%    0.0%    0.0%    0.0%    0.0%    0.0%    0.0%    0.0%    0.0%    0.0%    0.0%    0.0%    0.0%    0.0%    0.0%    0.0%    0.0%    0.0%    0.0%    0.0%    0.0%    0.0%    0.0%    0.0%    0.0%    0.0%    0.0%    0.0%    0.0%    0.0%    0.0%    0.0%    0.0%    0.0%    0.0%    0.0%    0.0%    0.0%    0.0%    0.0%    0.0%    0.0%    0.0%    0.0%    0.0%    0.0%    0.0%    0.0%    0.0%    0.0%    0.0%    0.0%    0.0%    0.0%    0.0%    0.0%    0.0%    0.0%    0.0%    0.0%    0.0%    0.0%    0.0%    0.0%    0.0%    0.0%    0.0%    0.0%    0.0%    0.0%    0.0%    0.0%    0.0%    0.0%    0.0%    0.0%    0.0%    0.0%    0.0%    0.0%    0.0%    0.0%    0.0%    0.0%    0.0%    0.0%    0.0%    0.0%    0.0%    0.0%    0.0%    0.0%    0.0%    0.0%    0.0%    0.0%    0.0%    0.0%    0.0%    0.0%    0.0%    0.0%    0.0%    0.0%    0.0%    0.0%    0.0%    0.0%    0.0%    0.0%    0.0%    0.0%        k__Bacteria;p__Proteobacteria;c__Betaproteobacteria; o__Hydrogenophilales    1958    0.0%    0.0%    0.0%    0.0%    0.0%    0.0%    0.0%    0.0%    0.0%    0.0%    0.0%    0.0%    0.0%    0.0%    0.0%    0.0%    0.0%    0.0%    0.0%    0.0%    0.0%    0.0%    0.0%    0.0%    0.0%    0.0%    0.0%    0.0%    0.0%    0.0%    0.0%    0.0%    0.0%    0.0%    0.0%    0.0%    0.0%    0.0%    0.0%    0.0%    0.0%    0.0%    0.0%    0.0%    0.0%    0.0%    0.0%    0.0%    0.0%    0.0%    0.0%    0.5%    0.2%    0.0%    0.0%    0.0%    0.0%    0.0%    0.0%    0.0%    0.0%    0.0%    0.0%    0.0%    0.0%    0.0%    0.0%    0.0%    0.0%    0.0%    0.0%    0.0%    0.0%    0.0%    0.0%    0.0%    0.0%    0.0%    0.0%    0.0%    0.0%    0.0%    0.0%    0.0%    0.0%    0.0%    0.0%    0.0%    0.0%    0.0%    0.0%    0.0%    0.0%    0.0%    0.0%    0.0%    0.0%    0.0%    0.0%    0.0%    0.0%    0.0%    0.0%    0.0%    0.0%    0.0%    0.0%    0.0%    0.0%    0.0%    0.0%    0.0%    0.0%    0.0%    0.0%    0.0%    0.0%    0.0%    0.0%    0.0%    0.0%    0.1%    0.0%    0.0%    0.0%    0.0%    0.0%    0.0%    0.0%    0.0%    0.0%    0.0%    0.0%    0.0%    0.0%    0.0%    0.0%    0.0%    0.0%    0.0%    0.0%    0.0%    0.0%    0.0%    0.0%    0.0%    0.0%    0.0%    0.0%    0.0%    0.0%    0.0%    0.0%    0.0%    0.0%    0.0%    0.0%    0.0%    0.0%    0.0%    0.0%    0.0%    0.0%    0.0%    0.0%    0.0%    0.0%    0.0%    0.0%    0.0%    0.0%    0.0%    0.0%    0.2%    0.0%    0.0%    0.0%    0.0%    0.0%    0.0%    0.0%    0.0%    0.0%    0.0%    0.0%    0.0%    0.0%    0.0%    0.0%    0.0%    0.0%    0.0%    0.0%    0.0%    0.0%    0.0%    0.0%    0.0%    0.0%    0.0%    0.0%    0.0%    0.0%    0.0%    0.0%    0.0%    0.0%    0.0%    0.0%    0.0%    0.0%    0.0%    0.0%    0.0%    0.0%    0.7%    0.0%    0.0%    0.0%    0.0%    0.0%    0.0%    0.0%    0.0%    0.0%    0.0%    0.0%    0.0%    0.0%    0.0%    0.0%    0.0%    0.0%    0.0%    0.0%    0.0%    0.0%    0.0%    0.0%    0.0%    0.0%    0.0%    0.0%    0.0%    0.0%    0.0%    0.0%    0.0%    0.0%    0.0%    0.0%    0.0%    0.0%    0.0%    0.0%    0.0%    0.0%    0.0%    0.1%    0.0%    0.0%    0.0%    0.0%    0.0%    0.0%    0.0%    0.0%    0.0%    0.0%    0.0%    0.0%    0.0%    0.0%    0.0%    0.0%    0.0%    0.0%    0.0%    0.0%    0.0%    0.0%    0.0%    0.0%    0.0%    0.0%    0.0%    0.0%    0.0%    0.0%    0.0%    0.2%    0.0%    0.0%    0.0%    0.0%    0.0%    0.0%    0.0%    0.0%    0.0%    0.0%    0.0%    0.0%    0.0%    0.0%    0.0%    0.0%    0.0%    0.0%    0.0%    0.0%    0.0%    0.0%    0.0%    0.0%    0.0%    0.0%    0.0%    0.0%    0.0%    0.0%    0.0%    0.0%    0.0%    0.0%    0.0%    0.0%    0.0%    0.1%    0.0%    0.0%    0.0%    0.0%    0.0%    0.0%    0.0%    0.0%    0.0%    0.0%    0.0%    0.0%    0.0%    0.0%    0.0%    0.0%    0.0%    0.0%    0.0%    0.0%    0.0%    0.0%    0.0%    0.0%    0.0%    0.0%    0.0%    0.0%    0.0%        k__Bacteria;p__Proteobacteria;c__Betaproteobacteria; o__Methylophilales   14629    0.1%    0.0%    0.0%    0.0%    0.0%    0.1%    0.0%    0.0%    0.0%    0.0%    0.0%    0.0%    0.0%    0.0%    0.0%    0.0%    0.0%    0.0%    0.0%    0.0%    0.0%    0.0%    0.0%    0.0%    0.0%    0.0%    0.0%    0.0%    0.0%    0.0%    0.0%    0.0%    0.0%    0.0%    0.0%    0.0%    0.0%    0.0%    0.0%    0.0%    0.0%    0.0%    0.0%    0.0%    0.0%    0.0%    0.0%    0.0%    0.0%    0.0%    0.0%    0.1%    0.0%    0.0%    0.0%    0.0%    0.0%    0.0%    0.0%    0.0%    0.0%    0.0%    0.0%    0.0%    0.0%    0.0%    0.2%    0.0%    0.1%    0.0%    0.0%    0.0%    0.0%    0.0%    0.0%    0.0%    0.0%    0.0%    0.0%    0.0%    0.0%    0.4%    0.0%    0.0%    0.0%    0.0%    0.1%    0.0%    0.0%    0.0%    0.0%    0.0%    0.0%    0.0%    0.0%    0.0%    0.1%    0.1%    0.0%    0.0%    0.1%    0.5%    0.5%    0.0%    0.0%    0.0%    0.1%    0.1%    0.1%    0.0%    0.0%    0.1%    0.0%    0.0%    0.0%    0.0%    0.3%    0.0%    0.0%    0.1%    0.1%    0.0%    0.0%    0.0%    0.0%    0.5%    0.0%    0.0%    0.0%    0.1%    0.0%    0.3%    0.1%    0.0%    0.1%    0.5%    0.1%    0.1%    0.1%    0.3%    0.0%    0.1%    0.3%    0.1%    0.2%    0.3%    0.1%    0.3%    0.0%    0.0%    0.3%    0.1%    0.0%    0.8%    0.0%    0.0%    0.1%    0.0%    0.0%    0.0%    0.0%    0.2%    0.0%    0.1%    0.6%    0.0%    0.0%    0.0%    0.1%    0.0%    0.0%    0.0%    0.0%    0.0%    0.3%    0.0%    0.0%    0.0%    0.1%    0.0%    0.0%    0.0%    0.2%    0.1%    0.0%    0.0%    0.0%    0.0%    0.0%    0.0%    0.0%    0.0%    0.0%    0.1%    0.0%    0.4%    0.0%    0.0%    0.0%    0.0%    0.0%    0.0%    0.0%    0.1%    0.1%    0.1%    0.0%    0.3%    0.1%    0.0%    0.0%    0.7%    0.4%    0.0%    0.2%    0.0%    0.5%    0.2%    0.6%    0.1%    0.1%    0.6%    0.0%    0.3%    0.5%    0.1%    0.1%    0.1%    0.1%    0.0%    0.3%    0.4%    0.2%    0.3%    0.1%    0.0%    0.0%    0.9%    0.2%    0.1%    0.0%    0.0%    0.2%    0.0%    0.0%    0.0%    0.0%    0.1%    0.1%    0.1%    0.5%    0.0%    0.2%    0.2%    0.1%    0.3%    0.2%    1.2%    0.2%    0.1%    0.2%    0.3%    0.1%    0.2%    0.5%    0.1%    0.1%    0.1%    0.2%    0.6%    0.2%    0.0%    0.3%    0.1%    0.6%    0.2%    0.1%    0.0%    0.1%    0.0%    0.0%    0.1%    0.3%    0.0%    0.7%    0.0%    0.0%    0.0%    0.0%    0.8%    0.0%    0.0%    0.0%    0.0%    0.0%    0.0%    0.0%    0.0%    0.0%    0.0%    0.0%    0.0%    0.0%    0.0%    0.0%    0.0%    0.0%    0.0%    0.0%    0.0%    0.0%    0.2%    0.0%    0.0%    0.0%    0.0%    0.0%    0.0%    0.0%    0.0%    0.0%    0.0%    0.0%    0.0%    0.0%    0.1%    0.0%    0.0%    0.0%    0.0%    0.0%    0.0%    0.0%    0.1%    0.0%    0.0%    0.0%    0.0%    0.0%    0.0%    0.0%    0.0%    0.0%    0.0%    0.0%    0.0%    0.0%    0.0%    0.0%    0.0%    0.0%    0.0%    0.0%    0.0%    0.0%    0.0%    1.1%    0.0%        k__Bacteria;p__Proteobacteria;c__Betaproteobacteria; o__Neisseriales   457943    2.7%    6.2%    5.8%    3.9%    3.0%    0.2%    6.7%    6.2%    2.5%    8.8%   15.3%    3.3%    1.1%    4.3%    8.6%    2.6%    4.6%    1.8%    0.7%    0.8%    2.0%    3.2%    1.8%    0.1%    1.0%    1.8%    0.5%    3.3%    4.3%    0.6%    0.2%    1.9%    0.7%    4.6%    2.8%    4.6%    3.2%    1.1%    3.4%    1.8%    3.4%    4.6%   11.0%   14.4%    3.5%    3.1%    1.4%    0.6%    4.7%    3.4%    5.9%    0.5%    0.4%    1.4%    1.5%    0.8%    1.4%    0.2%    3.2%    2.7%    0.6%    2.5%    1.1%    2.2%    0.5%    0.4%    0.1%    7.8%    0.3%    2.0%    4.2%    1.6%    0.6%    2.6%    1.8%    0.5%    1.1%    2.1%    1.2%    2.7%    2.9%    0.3%    3.2%    3.5%    1.5%    3.7%    4.0%    0.3%    4.6%    1.4%    0.0%    1.8%    2.0%   12.2%    0.8%    0.7%    2.5%    0.3%    2.0%    2.1%    1.0%    1.1%    0.7%    1.7%    1.5%    8.1%    4.1%    0.2%    0.1%    2.4%    3.4%    3.4%    2.0%    0.6%    4.1%    8.7%   16.0%    2.8%   23.8%    4.0%    0.1%    3.4%    2.1%    4.1%    0.4%    1.9%   13.1%    0.5%   22.9%    8.3%    2.9%    0.9%    0.4%    2.8%    3.8%    2.4%    1.0%    1.2%    0.3%    3.3%    0.1%    4.7%    2.0%    0.4%    0.4%    1.0%    5.5%    1.6%    2.1%    0.1%    0.4%    0.3%    2.5%    0.7%    4.9%    0.4%    1.8%    0.6%    0.8%    1.9%    1.0%    2.1%    6.2%    1.3%    1.8%    1.3%    4.1%    2.5%    2.8%    6.6%    3.8%    3.4%    0.7%    5.2%    1.3%    4.3%    2.2%    1.9%    0.5%    2.2%    2.3%    2.6%    3.7%    0.4%    0.8%    1.6%    0.5%    3.1%    2.9%    7.5%    2.3%    2.6%    4.5%    1.5%    0.9%    1.1%    3.5%    2.9%    1.7%    2.4%    4.1%    0.5%    0.6%    1.3%    2.3%    2.0%    0.5%    1.1%    1.3%    1.2%    2.0%    1.2%    0.5%    6.5%    1.7%    1.9%    1.6%    2.8%    1.3%    0.8%    1.0%    0.6%    0.0%    2.2%    0.8%    1.9%    4.1%    2.6%    3.4%    4.6%    1.7%    0.5%    0.5%    1.5%    1.1%    0.4%    1.4%    2.2%    0.3%    0.7%    1.0%    0.7%    4.3%    2.4%    9.6%    1.0%    4.0%    0.5%   11.3%    1.2%    1.1%    5.5%    0.5%    2.3%    0.4%    0.7%    2.9%    2.2%    1.8%    7.0%    1.1%   18.2%    1.3%    0.3%    1.9%    0.1%    0.7%    1.0%    1.5%    2.6%    1.5%    3.0%    8.7%    3.0%    1.3%    1.4%    2.6%    2.4%    3.4%    0.5%    0.6%    0.1%    1.6%    0.7%    1.0%    4.9%   20.6%    1.1%    0.6%    1.3%    1.3%    0.6%    4.2%    4.7%    2.0%    0.5%    3.4%    1.7%    2.3%    1.2%    1.1%    1.7%    4.4%    1.8%    4.9%    2.3%    1.5%    0.5%    0.5%    9.6%    0.0%    0.9%    0.2%    6.3%    4.0%    2.1%    0.2%    4.1%    3.4%    2.8%    6.5%    6.5%    1.1%    1.0%    2.2%    2.1%    2.2%    2.1%    4.0%    3.2%    6.9%    6.2%    3.5%    1.3%    1.6%    5.8%   16.5%    2.8%    5.6%    0.1%    1.5%    0.4%    2.4%    1.3%    2.1%    0.9%    0.2%    3.0%    0.5%    0.7%    0.9%    6.0%    1.0%    1.0%    0.8%    0.1%    1.1%    0.0%        k__Bacteria;p__Proteobacteria;c__Betaproteobacteria; o__Nitrosomonadales     492    0.0%    0.0%    0.0%    0.0%    0.0%    0.0%    0.0%    0.0%    0.0%    0.0%    0.0%    0.0%    0.0%    0.0%    0.0%    0.0%    0.0%    0.0%    0.0%    0.0%    0.0%    0.0%    0.0%    0.0%    0.0%    0.0%    0.0%    0.0%    0.0%    0.0%    0.0%    0.0%    0.0%    0.0%    0.0%    0.0%    0.0%    0.0%    0.0%    0.0%    0.0%    0.0%    0.0%    0.0%    0.0%    0.0%    0.0%    0.0%    0.0%    0.0%    0.0%    0.0%    0.0%    0.0%    0.0%    0.0%    0.0%    0.0%    0.0%    0.0%    0.0%    0.0%    0.0%    0.0%    0.0%    0.0%    0.0%    0.0%    0.0%    0.0%    0.0%    0.0%    0.0%    0.0%    0.0%    0.0%    0.0%    0.0%    0.0%    0.0%    0.0%    0.0%    0.0%    0.0%    0.0%    0.0%    0.0%    0.0%    0.0%    0.0%    0.0%    0.0%    0.0%    0.0%    0.0%    0.0%    0.0%    0.0%    0.0%    0.0%    0.0%    0.0%    0.0%    0.0%    0.0%    0.0%    0.0%    0.0%    0.0%    0.0%    0.0%    0.0%    0.0%    0.0%    0.0%    0.0%    0.0%    0.0%    0.0%    0.0%    0.0%    0.0%    0.0%    0.0%    0.0%    0.0%    0.0%    0.0%    0.0%    0.0%    0.0%    0.0%    0.0%    0.0%    0.0%    0.0%    0.0%    0.0%    0.0%    0.0%    0.0%    0.0%    0.0%    0.0%    0.0%    0.0%    0.0%    0.0%    0.0%    0.0%    0.0%    0.0%    0.0%    0.0%    0.0%    0.0%    0.0%    0.0%    0.0%    0.0%    0.0%    0.0%    0.0%    0.0%    0.0%    0.0%    0.0%    0.0%    0.0%    0.0%    0.0%    0.0%    0.0%    0.0%    0.0%    0.0%    0.0%    0.0%    0.0%    0.0%    0.0%    0.0%    0.0%    0.0%    0.0%    0.0%    0.0%    0.0%    0.0%    0.0%    0.0%    0.0%    0.0%    0.0%    0.0%    0.0%    0.0%    0.0%    0.0%    0.0%    0.0%    0.0%    0.0%    0.0%    0.0%    0.0%    0.0%    0.0%    0.0%    0.0%    0.0%    0.0%    0.0%    0.0%    0.0%    0.0%    0.0%    0.0%    0.0%    0.0%    0.0%    0.0%    0.0%    0.0%    0.0%    0.0%    0.0%    0.0%    0.0%    0.0%    0.0%    0.0%    0.0%    0.0%    0.0%    0.0%    0.0%    0.0%    0.0%    0.0%    0.0%    0.0%    0.0%    0.0%    0.0%    0.0%    0.0%    0.0%    0.0%    0.0%    0.0%    0.0%    0.0%    0.0%    0.0%    0.0%    0.0%    0.0%    0.0%    0.0%    0.0%    0.0%    0.0%    0.0%    0.0%    0.0%    0.0%    0.0%    0.0%    0.0%    0.0%    0.0%    0.0%    0.0%    0.0%    0.0%    0.0%    0.0%    0.0%    0.0%    0.0%    0.0%    0.0%    0.0%    0.0%    0.0%    0.0%    0.0%    0.0%    0.0%    0.0%    0.0%    0.0%    0.0%    0.0%    0.0%    0.0%    0.0%    0.0%    0.0%    0.0%    0.0%    0.0%    0.0%    0.0%    0.0%    0.0%    0.1%    0.0%    0.0%    0.0%    0.0%    0.0%    0.0%    0.0%    0.0%    0.0%    0.0%    0.0%    0.0%    0.0%    0.0%    0.0%    0.0%    0.0%    0.0%    0.0%    0.0%    0.0%    0.0%    0.0%    0.0%    0.0%    0.0%    0.0%    0.0%    0.0%    0.0%    0.0%    0.0%    0.0%    0.0%    0.0%    0.0%    0.0%    0.0%    0.0%    0.0%    0.0%    0.0%    0.0%    0.0%    0.0%    0.0%    0.0%    0.0%    0.0%    0.0%        k__Bacteria;p__Proteobacteria;c__Betaproteobacteria; o__Procabacteriales       0    0.0%    0.0%    0.0%    0.0%    0.0%    0.0%    0.0%    0.0%    0.0%    0.0%    0.0%    0.0%    0.0%    0.0%    0.0%    0.0%    0.0%    0.0%    0.0%    0.0%    0.0%    0.0%    0.0%    0.0%    0.0%    0.0%    0.0%    0.0%    0.0%    0.0%    0.0%    0.0%    0.0%    0.0%    0.0%    0.0%    0.0%    0.0%    0.0%    0.0%    0.0%    0.0%    0.0%    0.0%    0.0%    0.0%    0.0%    0.0%    0.0%    0.0%    0.0%    0.0%    0.0%    0.0%    0.0%    0.0%    0.0%    0.0%    0.0%    0.0%    0.0%    0.0%    0.0%    0.0%    0.0%    0.0%    0.0%    0.0%    0.0%    0.0%    0.0%    0.0%    0.0%    0.0%    0.0%    0.0%    0.0%    0.0%    0.0%    0.0%    0.0%    0.0%    0.0%    0.0%    0.0%    0.0%    0.0%    0.0%    0.0%    0.0%    0.0%    0.0%    0.0%    0.0%    0.0%    0.0%    0.0%    0.0%    0.0%    0.0%    0.0%    0.0%    0.0%    0.0%    0.0%    0.0%    0.0%    0.0%    0.0%    0.0%    0.0%    0.0%    0.0%    0.0%    0.0%    0.0%    0.0%    0.0%    0.0%    0.0%    0.0%    0.0%    0.0%    0.0%    0.0%    0.0%    0.0%    0.0%    0.0%    0.0%    0.0%    0.0%    0.0%    0.0%    0.0%    0.0%    0.0%    0.0%    0.0%    0.0%    0.0%    0.0%    0.0%    0.0%    0.0%    0.0%    0.0%    0.0%    0.0%    0.0%    0.0%    0.0%    0.0%    0.0%    0.0%    0.0%    0.0%    0.0%    0.0%    0.0%    0.0%    0.0%    0.0%    0.0%    0.0%    0.0%    0.0%    0.0%    0.0%    0.0%    0.0%    0.0%    0.0%    0.0%    0.0%    0.0%    0.0%    0.0%    0.0%    0.0%    0.0%    0.0%    0.0%    0.0%    0.0%    0.0%    0.0%    0.0%    0.0%    0.0%    0.0%    0.0%    0.0%    0.0%    0.0%    0.0%    0.0%    0.0%    0.0%    0.0%    0.0%    0.0%    0.0%    0.0%    0.0%    0.0%    0.0%    0.0%    0.0%    0.0%    0.0%    0.0%    0.0%    0.0%    0.0%    0.0%    0.0%    0.0%    0.0%    0.0%    0.0%    0.0%    0.0%    0.0%    0.0%    0.0%    0.0%    0.0%    0.0%    0.0%    0.0%    0.0%    0.0%    0.0%    0.0%    0.0%    0.0%    0.0%    0.0%    0.0%    0.0%    0.0%    0.0%    0.0%    0.0%    0.0%    0.0%    0.0%    0.0%    0.0%    0.0%    0.0%    0.0%    0.0%    0.0%    0.0%    0.0%    0.0%    0.0%    0.0%    0.0%    0.0%    0.0%    0.0%    0.0%    0.0%    0.0%    0.0%    0.0%    0.0%    0.0%    0.0%    0.0%    0.0%    0.0%    0.0%    0.0%    0.0%    0.0%    0.0%    0.0%    0.0%    0.0%    0.0%    0.0%    0.0%    0.0%    0.0%    0.0%    0.0%    0.0%    0.0%    0.0%    0.0%    0.0%    0.0%    0.0%    0.0%    0.0%    0.0%    0.0%    0.0%    0.0%    0.0%    0.0%    0.0%    0.0%    0.0%    0.0%    0.0%    0.0%    0.0%    0.0%    0.0%    0.0%    0.0%    0.0%    0.0%    0.0%    0.0%    0.0%    0.0%    0.0%    0.0%    0.0%    0.0%    0.0%    0.0%    0.0%    0.0%    0.0%    0.0%    0.0%    0.0%    0.0%    0.0%    0.0%    0.0%    0.0%    0.0%    0.0%    0.0%    0.0%    0.0%    0.0%    0.0%    0.0%    0.0%    0.0%    0.0%    0.0%    0.0%    0.0%    0.0%    0.0%    0.0%    0.0%    0.0%        k__Bacteria;p__Proteobacteria;c__Betaproteobacteria; o__Rhodocyclales    9080    0.1%    0.0%    0.0%    0.0%    0.0%    0.0%    0.0%    0.0%    0.0%    0.0%    0.0%    0.1%    0.0%    0.0%    0.0%    0.0%    0.0%    0.0%    0.0%    0.0%    0.0%    0.0%    0.1%    0.0%    0.0%    0.0%    0.0%    0.0%    0.0%    0.0%    0.0%    0.0%    0.0%    0.0%    0.0%    0.0%    0.0%    0.0%    0.0%    0.1%    0.0%    0.0%    0.0%    0.0%    0.0%    0.0%    0.0%    0.0%    0.0%    0.0%    0.1%    0.8%    0.8%    0.0%    0.0%    0.1%    0.0%    0.0%    0.2%    0.0%    0.0%    0.0%    0.0%    0.0%    0.0%    0.1%    0.0%    0.0%    0.0%    0.0%    0.0%    0.0%    0.0%    0.0%    0.1%    0.0%    0.0%    0.0%    0.0%    0.0%    0.0%    0.1%    0.0%    0.0%    0.0%    0.0%    0.1%    0.0%    0.0%    0.2%    0.0%    0.0%    0.1%    0.0%    0.0%    0.0%    0.1%    0.0%    0.0%    0.0%    0.1%    0.1%    0.1%    0.0%    0.0%    0.0%    0.1%    0.0%    0.0%    0.0%    0.0%    0.1%    0.0%    0.0%    0.0%    0.0%    0.0%    0.0%    0.0%    0.1%    0.0%    0.0%    0.0%    0.0%    0.0%    2.2%    0.1%    0.0%    0.1%    0.1%    0.1%    0.1%    0.1%    0.0%    0.1%    0.1%    0.0%    0.1%    0.0%    0.1%    0.0%    0.0%    0.1%    0.0%    0.1%    0.1%    0.0%    0.1%    0.1%    0.0%    0.1%    0.0%    0.1%    0.1%    0.0%    0.0%    0.2%    0.0%    0.0%    0.0%    0.1%    0.1%    0.0%    0.0%    0.1%    0.0%    0.0%    0.1%    0.1%    0.0%    0.1%    0.0%    0.0%    0.1%    0.1%    0.0%    0.0%    0.0%    0.0%    0.0%    0.0%    0.0%    0.0%    0.0%    0.0%    0.0%    0.0%    0.0%    0.0%    0.0%    0.0%    0.0%    0.0%    0.0%    0.0%    0.1%    0.0%    0.0%    0.0%    0.0%    0.0%    0.0%    0.0%    0.1%    0.1%    0.0%    0.0%    0.0%    0.1%    0.1%    0.0%    0.2%    0.1%    0.1%    0.0%    0.2%    0.0%    0.1%    0.1%    0.1%    0.0%    0.0%    0.0%    0.0%    0.0%    0.1%    0.0%    0.0%    0.1%    0.0%    0.0%    0.0%    0.1%    0.0%    0.0%    0.0%    0.1%    0.1%    0.0%    0.0%    0.0%    0.1%    0.1%    0.0%    0.0%    0.0%    0.0%    0.1%    0.0%    0.0%    0.1%    0.0%    0.0%    0.0%    0.0%    0.0%    0.0%    0.1%    0.0%    0.0%    0.1%    0.1%    0.0%    0.0%    0.1%    0.0%    0.0%    0.0%    0.0%    0.1%    0.0%    0.0%    0.1%    0.0%    0.1%    0.1%    0.1%    0.0%    0.0%    0.0%    0.0%    0.0%    0.0%    0.0%    0.1%    0.0%    0.0%    0.2%    0.1%    1.2%    0.4%    0.1%    0.1%    0.0%    0.1%    0.0%    0.0%    0.1%    0.2%    0.1%    0.0%    0.1%    0.0%    0.1%    0.0%    0.0%    0.0%    0.0%    0.0%    0.0%    0.5%    0.0%    0.0%    0.0%    0.0%    0.0%    0.0%    0.0%    0.0%    0.0%    0.0%    0.0%    0.0%    0.0%    0.0%    0.0%    0.0%    0.0%    0.0%    0.0%    0.0%    0.0%    0.0%    0.1%    0.0%    0.0%    0.0%    0.0%    0.0%    0.0%    0.0%    0.0%    0.0%    0.0%    0.0%    0.0%    0.0%    0.0%    0.0%    0.0%    0.0%    0.0%    0.0%    0.0%    0.0%    0.0%    0.0%    0.0%        k__Bacteria;p__Proteobacteria;c__Deltaproteobacteria; o__     167    0.0%    0.0%    0.0%    0.0%    0.0%    0.0%    0.0%    0.0%    0.0%    0.0%    0.0%    0.0%    0.0%    0.0%    0.0%    0.0%    0.0%    0.0%    0.0%    0.0%    0.0%    0.0%    0.0%    0.0%    0.0%    0.0%    0.0%    0.0%    0.0%    0.0%    0.0%    0.0%    0.0%    0.0%    0.0%    0.0%    0.0%    0.0%    0.0%    0.0%    0.0%    0.0%    0.0%    0.0%    0.0%    0.0%    0.0%    0.0%    0.0%    0.0%    0.0%    0.0%    0.0%    0.0%    0.0%    0.0%    0.0%    0.0%    0.0%    0.0%    0.0%    0.0%    0.0%    0.0%    0.0%    0.0%    0.0%    0.0%    0.0%    0.0%    0.0%    0.0%    0.0%    0.0%    0.0%    0.0%    0.0%    0.0%    0.0%    0.0%    0.0%    0.0%    0.0%    0.0%    0.0%    0.0%    0.0%    0.0%    0.0%    0.0%    0.0%    0.0%    0.0%    0.0%    0.0%    0.0%    0.0%    0.0%    0.0%    0.0%    0.0%    0.0%    0.0%    0.0%    0.0%    0.0%    0.0%    0.0%    0.0%    0.0%    0.0%    0.0%    0.0%    0.0%    0.0%    0.0%    0.0%    0.0%    0.0%    0.0%    0.0%    0.0%    0.0%    0.0%    0.0%    0.0%    0.0%    0.0%    0.0%    0.0%    0.0%    0.0%    0.0%    0.0%    0.0%    0.0%    0.0%    0.0%    0.0%    0.0%    0.0%    0.0%    0.0%    0.0%    0.0%    0.0%    0.0%    0.0%    0.0%    0.0%    0.0%    0.0%    0.0%    0.0%    0.0%    0.0%    0.0%    0.0%    0.0%    0.0%    0.0%    0.0%    0.0%    0.0%    0.0%    0.0%    0.0%    0.0%    0.0%    0.0%    0.0%    0.0%    0.0%    0.0%    0.0%    0.0%    0.0%    0.0%    0.0%    0.0%    0.0%    0.0%    0.0%    0.0%    0.0%    0.0%    0.0%    0.0%    0.0%    0.0%    0.0%    0.0%    0.0%    0.0%    0.0%    0.0%    0.0%    0.0%    0.0%    0.0%    0.0%    0.0%    0.0%    0.0%    0.0%    0.0%    0.0%    0.0%    0.0%    0.0%    0.0%    0.0%    0.0%    0.0%    0.0%    0.0%    0.0%    0.0%    0.0%    0.0%    0.1%    0.0%    0.0%    0.0%    0.0%    0.0%    0.0%    0.0%    0.0%    0.0%    0.0%    0.0%    0.0%    0.0%    0.0%    0.0%    0.0%    0.0%    0.0%    0.0%    0.0%    0.0%    0.0%    0.0%    0.0%    0.0%    0.0%    0.0%    0.0%    0.0%    0.0%    0.0%    0.0%    0.0%    0.0%    0.0%    0.0%    0.0%    0.0%    0.0%    0.0%    0.0%    0.0%    0.0%    0.0%    0.0%    0.0%    0.0%    0.0%    0.0%    0.0%    0.0%    0.0%    0.0%    0.0%    0.0%    0.0%    0.0%    0.0%    0.0%    0.0%    0.0%    0.0%    0.0%    0.0%    0.0%    0.0%    0.0%    0.0%    0.0%    0.0%    0.0%    0.0%    0.0%    0.0%    0.0%    0.0%    0.0%    0.0%    0.0%    0.0%    0.0%    0.0%    0.0%    0.0%    0.0%    0.0%    0.0%    0.0%    0.0%    0.0%    0.0%    0.0%    0.0%    0.0%    0.0%    0.0%    0.0%    0.0%    0.0%    0.0%    0.0%    0.0%    0.0%    0.0%    0.0%    0.0%    0.0%    0.0%    0.0%    0.0%    0.0%    0.0%    0.0%    0.0%    0.0%    0.0%    0.0%    0.0%    0.0%    0.0%    0.0%    0.0%    0.0%    0.0%    0.0%    0.0%    0.0%    0.0%    0.0%    0.0%    0.0%    0.0%    0.0%    0.0%    0.0%    0.0%    0.0%        k__Bacteria;p__Proteobacteria;c__Deltaproteobacteria; o__Bdellovibrionales    2034    0.0%    0.0%    0.0%    0.0%    0.0%    0.0%    0.0%    0.0%    0.0%    0.0%    0.0%    0.0%    0.0%    0.0%    0.0%    0.0%    0.0%    0.0%    0.0%    0.0%    0.0%    0.0%    0.0%    0.0%    0.0%    0.0%    0.0%    0.0%    0.0%    0.0%    0.0%    0.0%    0.0%    0.0%    0.0%    0.0%    0.0%    0.0%    0.0%    0.0%    0.0%    0.0%    0.0%    0.0%    0.0%    0.0%    0.0%    0.0%    0.0%    0.0%    0.0%    0.0%    0.0%    0.0%    0.0%    0.0%    0.0%    0.0%    0.0%    0.0%    0.0%    0.0%    0.0%    0.0%    0.0%    0.0%    0.0%    0.0%    0.0%    0.0%    0.0%    0.0%    0.0%    0.0%    0.0%    0.0%    0.0%    0.0%    0.0%    0.0%    0.0%    0.1%    0.0%    0.0%    0.0%    0.0%    0.0%    0.0%    0.0%    0.0%    0.0%    0.0%    0.0%    0.0%    0.0%    0.0%    0.0%    0.0%    0.0%    0.0%    0.0%    0.0%    0.0%    0.0%    0.0%    0.0%    0.0%    0.0%    0.0%    0.0%    0.0%    0.0%    0.0%    0.0%    0.0%    0.0%    0.0%    0.0%    0.0%    0.0%    0.0%    0.0%    0.0%    0.0%    0.0%    0.1%    0.0%    0.0%    0.0%    0.1%    0.0%    0.0%    0.0%    0.0%    0.0%    0.0%    0.0%    0.0%    0.0%    0.0%    0.0%    0.0%    0.0%    0.0%    0.0%    0.0%    0.0%    0.0%    0.0%    0.0%    0.0%    0.0%    0.1%    0.0%    0.0%    0.0%    0.1%    0.0%    0.0%    0.1%    0.0%    0.0%    0.0%    0.0%    0.0%    0.0%    0.0%    0.0%    0.0%    0.0%    0.2%    0.0%    0.0%    0.0%    0.0%    0.0%    0.0%    0.0%    0.0%    0.0%    0.0%    0.0%    0.0%    0.0%    0.0%    0.0%    0.0%    0.0%    0.0%    0.0%    0.0%    0.0%    0.0%    0.0%    0.0%    0.0%    0.0%    0.0%    0.0%    0.0%    0.0%    0.0%    0.0%    0.0%    0.1%    0.0%    0.0%    0.0%    0.1%    0.0%    0.0%    0.0%    0.0%    0.0%    0.0%    0.1%    0.0%    0.0%    0.0%    0.0%    0.0%    0.0%    0.0%    0.0%    0.0%    0.0%    0.0%    0.0%    0.0%    0.0%    0.0%    0.0%    0.1%    0.0%    0.0%    0.0%    0.0%    0.0%    0.0%    0.0%    0.0%    0.0%    0.0%    0.0%    0.0%    0.0%    0.0%    0.0%    0.0%    0.0%    0.0%    0.0%    0.0%    0.0%    0.0%    0.1%    0.0%    0.0%    0.0%    0.0%    0.0%    0.0%    0.0%    0.0%    0.0%    0.0%    0.0%    0.0%    0.0%    0.0%    0.0%    0.0%    0.1%    0.0%    0.0%    0.0%    0.0%    0.0%    0.0%    0.0%    0.0%    0.0%    0.0%    0.0%    0.0%    0.0%    0.0%    0.1%    0.0%    0.0%    0.0%    0.0%    0.0%    0.0%    0.0%    0.0%    0.0%    0.0%    0.0%    0.0%    0.0%    0.0%    0.0%    0.0%    0.0%    0.0%    0.0%    0.0%    0.0%    0.0%    0.0%    0.0%    0.0%    0.0%    0.0%    0.0%    0.0%    0.0%    0.0%    0.0%    0.0%    0.0%    0.0%    0.0%    0.0%    0.0%    0.0%    0.0%    0.0%    0.0%    0.0%    0.0%    0.0%    0.0%    0.0%    0.0%    0.0%    0.0%    0.0%    0.0%    0.0%    0.0%    0.0%    0.0%    0.0%    0.0%    0.0%    0.0%    0.0%    0.0%    0.0%    0.0%    0.0%    0.0%    0.0%    0.0%    0.0%    0.0%        k__Bacteria;p__Proteobacteria;c__Deltaproteobacteria; o__CTD005-82B-02     464    0.0%    0.0%    0.0%    0.0%    0.0%    0.0%    0.0%    0.0%    0.0%    0.0%    0.0%    0.0%    0.0%    0.0%    0.0%    0.0%    0.0%    0.0%    0.0%    0.0%    0.0%    0.0%    0.0%    0.0%    0.0%    0.0%    0.0%    0.0%    0.0%    0.0%    0.0%    0.0%    0.0%    0.0%    0.0%    0.0%    0.0%    0.0%    0.0%    0.0%    0.0%    0.0%    0.0%    0.0%    0.0%    0.0%    0.0%    0.0%    0.0%    0.0%    0.0%    0.0%    0.0%    0.0%    0.0%    0.0%    0.0%    0.0%    0.0%    0.0%    0.0%    0.0%    0.0%    0.0%    0.0%    0.0%    0.0%    0.0%    0.0%    0.0%    0.0%    0.0%    0.0%    0.0%    0.0%    0.0%    0.0%    0.0%    0.0%    0.0%    0.0%    0.0%    0.0%    0.0%    0.0%    0.0%    0.0%    0.0%    0.0%    0.0%    0.0%    0.0%    0.0%    0.0%    0.0%    0.0%    0.0%    0.0%    0.0%    0.0%    0.0%    0.0%    0.0%    0.0%    0.0%    0.0%    0.0%    0.0%    0.0%    0.0%    0.0%    0.0%    0.0%    0.0%    0.0%    0.0%    0.0%    0.0%    0.0%    0.0%    0.0%    0.0%    0.0%    0.0%    0.0%    0.0%    0.0%    0.0%    0.0%    0.0%    0.0%    0.0%    0.0%    0.0%    0.0%    0.0%    0.0%    0.0%    0.0%    0.0%    0.0%    0.0%    0.0%    0.0%    0.0%    0.0%    0.0%    0.0%    0.0%    0.0%    0.0%    0.0%    0.0%    0.0%    0.0%    0.0%    0.0%    0.0%    0.0%    0.0%    0.0%    0.0%    0.0%    0.0%    0.0%    0.0%    0.0%    0.0%    0.0%    0.0%    0.0%    0.0%    0.0%    0.0%    0.0%    0.0%    0.0%    0.0%    0.0%    0.0%    0.0%    0.0%    0.0%    0.0%    0.0%    0.0%    0.0%    0.0%    0.0%    0.0%    0.0%    0.0%    0.0%    0.0%    0.0%    0.0%    0.0%    0.0%    0.0%    0.0%    0.0%    0.0%    0.0%    0.0%    0.0%    0.0%    0.0%    0.0%    0.0%    0.0%    0.0%    0.0%    0.0%    0.0%    0.0%    0.0%    0.0%    0.0%    0.0%    0.0%    0.0%    0.0%    0.0%    0.0%    0.0%    0.0%    0.0%    0.0%    0.0%    0.0%    0.0%    0.0%    0.0%    0.0%    0.0%    0.0%    0.0%    0.0%    0.0%    0.0%    0.0%    0.0%    0.0%    0.0%    0.0%    0.0%    0.0%    0.0%    0.0%    0.0%    0.0%    0.0%    0.0%    0.0%    0.0%    0.0%    0.0%    0.0%    0.0%    0.0%    0.0%    0.0%    0.0%    0.0%    0.0%    0.0%    0.0%    0.0%    0.0%    0.0%    0.0%    0.0%    0.0%    0.0%    0.0%    0.0%    0.0%    0.0%    0.0%    0.0%    0.0%    0.0%    0.0%    0.0%    0.0%    0.0%    0.0%    0.0%    0.0%    0.0%    0.0%    0.0%    0.0%    0.0%    0.0%    0.0%    0.0%    0.0%    0.0%    0.0%    0.0%    0.0%    0.0%    0.0%    0.0%    0.0%    0.0%    0.0%    0.0%    0.0%    0.0%    0.0%    0.0%    0.0%    0.0%    0.0%    0.0%    0.0%    0.0%    0.0%    0.0%    0.0%    0.0%    0.0%    0.0%    0.0%    0.0%    0.0%    0.0%    0.0%    0.0%    0.0%    0.0%    0.0%    0.0%    0.0%    0.0%    0.0%    0.0%    0.0%    0.0%    0.0%    0.0%    0.0%    0.0%    0.0%    0.0%    0.0%    0.0%    0.0%    0.0%    0.0%    0.0%    0.0%    0.0%    0.0%    0.0%    0.0%        k__Bacteria;p__Proteobacteria;c__Deltaproteobacteria; o__Desulfobacterales     369    0.0%    0.0%    0.0%    0.0%    0.0%    0.0%    0.0%    0.0%    0.0%    0.0%    0.0%    0.0%    0.0%    0.0%    0.0%    0.0%    0.0%    0.0%    0.0%    0.0%    0.0%    0.0%    0.0%    0.0%    0.0%    0.0%    0.0%    0.0%    0.0%    0.0%    0.0%    0.0%    0.0%    0.0%    0.0%    0.0%    0.0%    0.0%    0.0%    0.0%    0.0%    0.0%    0.0%    0.0%    0.0%    0.0%    0.0%    0.0%    0.0%    0.0%    0.0%    0.0%    0.0%    0.0%    0.0%    0.0%    0.0%    0.0%    0.0%    0.0%    0.0%    0.0%    0.0%    0.0%    0.0%    0.0%    0.0%    0.0%    0.0%    0.0%    0.0%    0.0%    0.0%    0.0%    0.0%    0.0%    0.0%    0.0%    0.0%    0.0%    0.0%    0.0%    0.0%    0.0%    0.0%    0.0%    0.0%    0.0%    0.0%    0.0%    0.0%    0.0%    0.0%    0.0%    0.0%    0.0%    0.0%    0.0%    0.0%    0.0%    0.0%    0.0%    0.0%    0.0%    0.0%    0.0%    0.0%    0.0%    0.0%    0.0%    0.0%    0.0%    0.0%    0.0%    0.0%    0.0%    0.0%    0.0%    0.0%    0.0%    0.0%    0.0%    0.0%    0.0%    0.0%    0.2%    0.0%    0.0%    0.0%    0.0%    0.0%    0.0%    0.0%    0.0%    0.0%    0.0%    0.0%    0.0%    0.0%    0.0%    0.0%    0.0%    0.0%    0.0%    0.0%    0.0%    0.0%    0.0%    0.0%    0.0%    0.0%    0.0%    0.0%    0.0%    0.0%    0.0%    0.0%    0.0%    0.0%    0.0%    0.0%    0.0%    0.0%    0.0%    0.0%    0.0%    0.0%    0.0%    0.0%    0.0%    0.0%    0.0%    0.0%    0.0%    0.0%    0.0%    0.0%    0.0%    0.0%    0.0%    0.0%    0.0%    0.0%    0.0%    0.0%    0.0%    0.0%    0.0%    0.0%    0.0%    0.0%    0.0%    0.0%    0.0%    0.0%    0.0%    0.0%    0.0%    0.0%    0.0%    0.0%    0.0%    0.0%    0.0%    0.0%    0.0%    0.0%    0.0%    0.0%    0.0%    0.0%    0.0%    0.0%    0.0%    0.0%    0.0%    0.0%    0.0%    0.0%    0.0%    0.0%    0.0%    0.0%    0.0%    0.0%    0.0%    0.0%    0.0%    0.0%    0.0%    0.0%    0.0%    0.0%    0.0%    0.0%    0.0%    0.0%    0.0%    0.0%    0.0%    0.0%    0.0%    0.0%    0.0%    0.0%    0.0%    0.0%    0.0%    0.0%    0.0%    0.0%    0.0%    0.0%    0.0%    0.0%    0.0%    0.0%    0.0%    0.0%    0.0%    0.0%    0.0%    0.0%    0.0%    0.0%    0.0%    0.0%    0.0%    0.0%    0.0%    0.0%    0.0%    0.0%    0.0%    0.0%    0.0%    0.0%    0.0%    0.0%    0.0%    0.0%    0.0%    0.0%    0.0%    0.0%    0.0%    0.0%    0.0%    0.0%    0.0%    0.0%    0.0%    0.0%    0.0%    0.0%    0.0%    0.0%    0.0%    0.0%    0.0%    0.0%    0.0%    0.0%    0.0%    0.0%    0.0%    0.0%    0.0%    0.0%    0.0%    0.0%    0.0%    0.0%    0.0%    0.0%    0.0%    0.0%    0.0%    0.0%    0.0%    0.0%    0.0%    0.0%    0.0%    0.0%    0.0%    0.0%    0.0%    0.0%    0.0%    0.0%    0.0%    0.0%    0.0%    0.0%    0.0%    0.0%    0.0%    0.0%    0.0%    0.0%    0.0%    0.0%    0.0%    0.0%    0.0%    0.0%    0.0%    0.0%    0.0%    0.0%    0.0%    0.0%    0.0%    0.0%    0.0%    0.0%    0.0%        k__Bacteria;p__Proteobacteria;c__Deltaproteobacteria; o__Desulfovibrionales     825    0.0%    0.0%    0.0%    0.0%    0.0%    0.0%    0.0%    0.0%    0.0%    0.0%    0.0%    0.0%    0.0%    0.0%    0.0%    0.0%    0.0%    0.0%    0.0%    0.0%    0.0%    0.0%    0.0%    0.0%    0.0%    0.0%    0.0%    0.0%    0.0%    0.0%    0.0%    0.0%    0.0%    0.0%    0.0%    0.0%    0.0%    0.0%    0.0%    0.0%    0.0%    0.0%    0.0%    0.0%    0.0%    0.0%    0.0%    0.0%    0.0%    0.0%    0.0%    0.0%    0.0%    0.0%    0.0%    0.0%    0.0%    0.0%    0.0%    0.0%    0.0%    0.0%    0.0%    0.0%    0.0%    0.0%    0.0%    0.0%    0.0%    0.0%    0.1%    0.0%    0.0%    0.0%    0.0%    0.0%    0.0%    0.0%    0.0%    0.0%    0.0%    0.0%    0.0%    0.0%    0.0%    0.0%    0.0%    0.0%    0.0%    0.0%    0.0%    0.0%    0.0%    0.0%    0.0%    0.0%    0.0%    0.0%    0.0%    0.0%    0.0%    0.0%    0.0%    0.0%    0.0%    0.0%    0.0%    0.0%    0.0%    0.0%    0.0%    0.0%    0.0%    0.0%    0.0%    0.0%    0.0%    0.0%    0.0%    0.1%    0.0%    0.0%    0.0%    0.0%    0.0%    0.0%    0.0%    0.0%    0.0%    0.0%    0.0%    0.0%    0.0%    0.0%    0.0%    0.0%    0.0%    0.0%    0.0%    0.0%    0.0%    0.1%    0.0%    0.0%    0.0%    0.0%    0.0%    0.0%    0.0%    0.0%    0.0%    0.0%    0.0%    0.0%    0.0%    0.0%    0.0%    0.0%    0.0%    0.0%    0.0%    0.0%    0.0%    0.0%    0.0%    0.0%    0.0%    0.0%    0.0%    0.0%    0.0%    0.0%    0.0%    0.0%    0.0%    0.0%    0.0%    0.0%    0.0%    0.0%    0.0%    0.0%    0.0%    0.0%    0.0%    0.0%    0.0%    0.0%    0.0%    0.0%    0.0%    0.0%    0.0%    0.0%    0.0%    0.0%    0.0%    0.0%    0.0%    0.0%    0.0%    0.0%    0.0%    0.0%    0.0%    0.0%    0.0%    0.0%    0.0%    0.0%    0.0%    0.0%    0.0%    0.0%    0.0%    0.0%    0.0%    0.0%    0.1%    0.0%    0.0%    0.0%    0.0%    0.0%    0.0%    0.0%    0.0%    0.0%    0.0%    0.0%    0.0%    0.0%    0.0%    0.0%    0.0%    0.0%    0.0%    0.0%    0.0%    0.0%    0.0%    0.0%    0.0%    0.0%    0.0%    0.0%    0.0%    0.0%    0.0%    0.0%    0.0%    0.0%    0.0%    0.0%    0.0%    0.0%    0.0%    0.0%    0.0%    0.0%    0.0%    0.0%    0.0%    0.0%    0.0%    0.0%    0.0%    0.0%    0.0%    0.0%    0.0%    0.0%    0.0%    0.0%    0.0%    0.0%    0.0%    0.0%    0.0%    0.0%    0.0%    0.0%    0.0%    0.0%    0.0%    0.0%    0.0%    0.0%    0.0%    0.0%    0.0%    0.0%    0.0%    0.1%    0.0%    0.0%    0.0%    0.0%    0.0%    0.0%    0.0%    0.0%    0.0%    0.0%    0.0%    0.0%    0.0%    0.0%    0.0%    0.0%    0.0%    0.0%    0.0%    0.0%    0.0%    0.0%    0.0%    0.0%    0.0%    0.0%    0.0%    0.0%    0.0%    0.0%    0.0%    0.0%    0.0%    0.0%    0.0%    0.0%    0.0%    0.0%    0.0%    0.0%    0.1%    0.0%    0.0%    0.0%    0.0%    0.0%    0.0%    0.0%    0.0%    0.0%    0.0%    0.0%    0.0%    0.0%    0.0%    0.0%    0.0%    0.0%    0.0%    0.0%    0.0%    0.0%    0.0%    0.0%        k__Bacteria;p__Proteobacteria;c__Deltaproteobacteria; o__Desulfuromonadales     145    0.0%    0.0%    0.0%    0.0%    0.0%    0.0%    0.0%    0.0%    0.0%    0.0%    0.0%    0.0%    0.0%    0.0%    0.0%    0.0%    0.0%    0.0%    0.0%    0.0%    0.0%    0.0%    0.0%    0.0%    0.0%    0.0%    0.0%    0.0%    0.0%    0.0%    0.0%    0.0%    0.0%    0.0%    0.0%    0.0%    0.0%    0.0%    0.0%    0.0%    0.0%    0.0%    0.0%    0.0%    0.0%    0.0%    0.0%    0.0%    0.0%    0.0%    0.0%    0.0%    0.0%    0.0%    0.0%    0.0%    0.0%    0.0%    0.0%    0.0%    0.0%    0.0%    0.0%    0.0%    0.0%    0.0%    0.0%    0.0%    0.0%    0.0%    0.0%    0.0%    0.0%    0.0%    0.0%    0.0%    0.0%    0.0%    0.0%    0.0%    0.0%    0.0%    0.0%    0.0%    0.0%    0.0%    0.0%    0.0%    0.0%    0.0%    0.0%    0.0%    0.0%    0.0%    0.0%    0.0%    0.0%    0.0%    0.0%    0.0%    0.0%    0.0%    0.0%    0.0%    0.0%    0.0%    0.0%    0.0%    0.0%    0.0%    0.0%    0.0%    0.0%    0.0%    0.0%    0.0%    0.0%    0.0%    0.0%    0.0%    0.0%    0.0%    0.0%    0.0%    0.0%    0.0%    0.0%    0.0%    0.0%    0.0%    0.0%    0.0%    0.0%    0.0%    0.0%    0.0%    0.0%    0.0%    0.0%    0.0%    0.0%    0.0%    0.0%    0.0%    0.0%    0.0%    0.0%    0.0%    0.0%    0.0%    0.0%    0.0%    0.0%    0.0%    0.0%    0.0%    0.0%    0.0%    0.0%    0.0%    0.0%    0.0%    0.0%    0.0%    0.0%    0.0%    0.0%    0.0%    0.0%    0.0%    0.0%    0.0%    0.0%    0.0%    0.0%    0.0%    0.0%    0.0%    0.0%    0.0%    0.0%    0.0%    0.0%    0.0%    0.0%    0.0%    0.0%    0.0%    0.0%    0.0%    0.0%    0.0%    0.0%    0.0%    0.0%    0.0%    0.0%    0.0%    0.0%    0.0%    0.0%    0.0%    0.0%    0.0%    0.0%    0.0%    0.0%    0.0%    0.0%    0.0%    0.0%    0.0%    0.0%    0.0%    0.0%    0.0%    0.0%    0.0%    0.0%    0.0%    0.0%    0.0%    0.0%    0.0%    0.0%    0.0%    0.0%    0.0%    0.0%    0.0%    0.0%    0.0%    0.0%    0.0%    0.0%    0.0%    0.0%    0.0%    0.0%    0.0%    0.0%    0.0%    0.0%    0.0%    0.0%    0.0%    0.0%    0.0%    0.0%    0.0%    0.0%    0.0%    0.0%    0.0%    0.0%    0.0%    0.0%    0.0%    0.0%    0.0%    0.0%    0.0%    0.0%    0.0%    0.0%    0.0%    0.0%    0.0%    0.0%    0.0%    0.0%    0.0%    0.0%    0.0%    0.0%    0.0%    0.0%    0.0%    0.0%    0.0%    0.0%    0.0%    0.0%    0.0%    0.0%    0.0%    0.0%    0.0%    0.0%    0.0%    0.0%    0.0%    0.0%    0.0%    0.0%    0.0%    0.0%    0.0%    0.0%    0.0%    0.0%    0.0%    0.0%    0.0%    0.0%    0.0%    0.0%    0.0%    0.0%    0.0%    0.0%    0.0%    0.0%    0.0%    0.0%    0.0%    0.0%    0.0%    0.0%    0.0%    0.0%    0.0%    0.0%    0.0%    0.0%    0.0%    0.0%    0.0%    0.0%    0.0%    0.0%    0.0%    0.0%    0.0%    0.0%    0.0%    0.0%    0.0%    0.0%    0.0%    0.0%    0.0%    0.0%    0.0%    0.0%    0.0%    0.0%    0.0%    0.0%    0.0%    0.0%    0.0%    0.0%    0.0%    0.0%    0.0%    0.0%    0.0%        k__Bacteria;p__Proteobacteria;c__Deltaproteobacteria; o__Entotheonellales      70    0.0%    0.0%    0.0%    0.0%    0.0%    0.0%    0.0%    0.0%    0.0%    0.0%    0.0%    0.0%    0.0%    0.0%    0.0%    0.0%    0.0%    0.0%    0.0%    0.0%    0.0%    0.0%    0.0%    0.0%    0.0%    0.0%    0.0%    0.0%    0.0%    0.0%    0.0%    0.0%    0.0%    0.0%    0.0%    0.0%    0.0%    0.0%    0.0%    0.0%    0.0%    0.0%    0.0%    0.0%    0.0%    0.0%    0.0%    0.0%    0.0%    0.0%    0.0%    0.0%    0.0%    0.0%    0.0%    0.0%    0.0%    0.0%    0.0%    0.0%    0.0%    0.0%    0.0%    0.0%    0.0%    0.0%    0.0%    0.0%    0.0%    0.0%    0.0%    0.0%    0.0%    0.0%    0.0%    0.0%    0.0%    0.0%    0.0%    0.0%    0.0%    0.0%    0.0%    0.0%    0.0%    0.0%    0.0%    0.0%    0.0%    0.0%    0.0%    0.0%    0.0%    0.0%    0.0%    0.0%    0.0%    0.0%    0.0%    0.0%    0.0%    0.0%    0.0%    0.0%    0.0%    0.0%    0.0%    0.0%    0.0%    0.0%    0.0%    0.0%    0.0%    0.0%    0.0%    0.0%    0.0%    0.0%    0.0%    0.0%    0.0%    0.0%    0.0%    0.0%    0.0%    0.0%    0.0%    0.0%    0.0%    0.0%    0.0%    0.0%    0.0%    0.0%    0.0%    0.0%    0.0%    0.0%    0.0%    0.0%    0.0%    0.0%    0.0%    0.0%    0.0%    0.0%    0.0%    0.0%    0.0%    0.0%    0.0%    0.0%    0.0%    0.0%    0.0%    0.0%    0.0%    0.0%    0.0%    0.0%    0.0%    0.0%    0.0%    0.0%    0.0%    0.0%    0.0%    0.0%    0.0%    0.0%    0.0%    0.0%    0.0%    0.0%    0.0%    0.0%    0.0%    0.0%    0.0%    0.0%    0.0%    0.0%    0.0%    0.0%    0.0%    0.0%    0.0%    0.0%    0.0%    0.0%    0.0%    0.0%    0.0%    0.0%    0.0%    0.0%    0.0%    0.0%    0.0%    0.0%    0.0%    0.0%    0.0%    0.0%    0.0%    0.0%    0.0%    0.0%    0.0%    0.0%    0.0%    0.0%    0.0%    0.0%    0.0%    0.0%    0.0%    0.0%    0.0%    0.0%    0.0%    0.0%    0.0%    0.0%    0.0%    0.0%    0.0%    0.0%    0.0%    0.0%    0.0%    0.0%    0.0%    0.0%    0.0%    0.0%    0.0%    0.0%    0.0%    0.0%    0.0%    0.0%    0.0%    0.0%    0.0%    0.0%    0.0%    0.0%    0.0%    0.0%    0.0%    0.0%    0.0%    0.0%    0.0%    0.0%    0.0%    0.0%    0.0%    0.0%    0.0%    0.0%    0.0%    0.0%    0.0%    0.0%    0.0%    0.0%    0.0%    0.0%    0.0%    0.0%    0.0%    0.0%    0.0%    0.0%    0.0%    0.0%    0.0%    0.0%    0.0%    0.0%    0.0%    0.0%    0.0%    0.0%    0.0%    0.0%    0.0%    0.0%    0.0%    0.0%    0.0%    0.0%    0.0%    0.0%    0.0%    0.0%    0.0%    0.0%    0.0%    0.0%    0.0%    0.0%    0.0%    0.0%    0.0%    0.0%    0.0%    0.0%    0.0%    0.0%    0.0%    0.0%    0.0%    0.0%    0.0%    0.0%    0.0%    0.0%    0.0%    0.0%    0.0%    0.0%    0.0%    0.0%    0.0%    0.0%    0.0%    0.0%    0.0%    0.0%    0.0%    0.0%    0.0%    0.0%    0.0%    0.0%    0.0%    0.0%    0.0%    0.0%    0.0%    0.0%    0.0%    0.0%    0.0%    0.0%    0.0%    0.0%    0.0%    0.0%    0.0%    0.0%    0.0%    0.0%    0.0%    0.0%        k__Bacteria;p__Proteobacteria;c__Deltaproteobacteria; o__MIZ46     482    0.0%    0.0%    0.0%    0.0%    0.0%    0.0%    0.0%    0.0%    0.0%    0.0%    0.0%    0.0%    0.0%    0.0%    0.0%    0.0%    0.0%    0.0%    0.0%    0.0%    0.0%    0.0%    0.0%    0.0%    0.0%    0.0%    0.0%    0.0%    0.0%    0.0%    0.0%    0.0%    0.0%    0.0%    0.0%    0.0%    0.0%    0.0%    0.0%    0.0%    0.0%    0.0%    0.0%    0.0%    0.0%    0.0%    0.0%    0.0%    0.0%    0.0%    0.0%    0.0%    0.0%    0.0%    0.0%    0.0%    0.0%    0.0%    0.0%    0.0%    0.0%    0.0%    0.0%    0.0%    0.0%    0.0%    0.0%    0.0%    0.0%    0.0%    0.0%    0.0%    0.0%    0.0%    0.0%    0.0%    0.0%    0.0%    0.0%    0.0%    0.0%    0.0%    0.0%    0.0%    0.0%    0.0%    0.0%    0.0%    0.0%    0.0%    0.0%    0.0%    0.0%    0.0%    0.0%    0.0%    0.0%    0.0%    0.0%    0.0%    0.0%    0.0%    0.0%    0.0%    0.0%    0.0%    0.0%    0.0%    0.0%    0.0%    0.0%    0.0%    0.0%    0.0%    0.0%    0.0%    0.0%    0.0%    0.0%    0.0%    0.0%    0.0%    0.0%    0.0%    0.0%    0.0%    0.0%    0.0%    0.0%    0.0%    0.0%    0.0%    0.0%    0.0%    0.0%    0.0%    0.0%    0.0%    0.0%    0.0%    0.0%    0.0%    0.0%    0.0%    0.0%    0.0%    0.0%    0.0%    0.0%    0.0%    0.0%    0.0%    0.0%    0.0%    0.0%    0.0%    0.0%    0.0%    0.0%    0.0%    0.0%    0.0%    0.0%    0.0%    0.0%    0.0%    0.0%    0.0%    0.0%    0.0%    0.0%    0.0%    0.0%    0.0%    0.0%    0.0%    0.0%    0.0%    0.0%    0.0%    0.0%    0.0%    0.0%    0.0%    0.0%    0.0%    0.0%    0.0%    0.0%    0.0%    0.0%    0.0%    0.0%    0.0%    0.0%    0.0%    0.0%    0.0%    0.0%    0.0%    0.0%    0.0%    0.0%    0.0%    0.0%    0.0%    0.0%    0.0%    0.0%    0.0%    0.0%    0.0%    0.0%    0.0%    0.0%    0.0%    0.0%    0.0%    0.0%    0.0%    0.0%    0.0%    0.0%    0.0%    0.0%    0.0%    0.0%    0.0%    0.0%    0.0%    0.0%    0.0%    0.0%    0.0%    0.0%    0.0%    0.0%    0.0%    0.0%    0.0%    0.0%    0.0%    0.0%    0.0%    0.0%    0.0%    0.0%    0.0%    0.0%    0.0%    0.0%    0.0%    0.0%    0.0%    0.0%    0.0%    0.0%    0.0%    0.0%    0.0%    0.0%    0.0%    0.0%    0.0%    0.0%    0.0%    0.0%    0.0%    0.0%    0.0%    0.0%    0.0%    0.0%    0.0%    0.0%    0.0%    0.0%    0.0%    0.0%    0.0%    0.0%    0.0%    0.0%    0.0%    0.0%    0.0%    0.0%    0.0%    0.0%    0.0%    0.0%    0.0%    0.0%    0.0%    0.0%    0.0%    0.0%    0.0%    0.0%    0.0%    0.0%    0.0%    0.0%    0.0%    0.0%    0.0%    0.0%    0.0%    0.0%    0.0%    0.0%    0.0%    0.0%    0.0%    0.0%    0.0%    0.0%    0.0%    0.0%    0.0%    0.0%    0.0%    0.0%    0.0%    0.0%    0.0%    0.0%    0.0%    0.0%    0.0%    0.0%    0.0%    0.0%    0.0%    0.0%    0.0%    0.0%    0.0%    0.0%    0.0%    0.0%    0.0%    0.0%    0.0%    0.0%    0.0%    0.0%    0.0%    0.0%    0.0%    0.0%    0.0%    0.0%    0.0%    0.0%    0.0%    0.0%    0.0%        k__Bacteria;p__Proteobacteria;c__Deltaproteobacteria; o__Myxococcales    7156    0.0%    0.0%    0.1%    0.0%    0.0%    0.0%    0.0%    0.0%    0.0%    0.0%    0.0%    0.0%    0.0%    0.0%    0.0%    0.0%    0.0%    0.0%    0.0%    0.0%    0.0%    0.0%    0.0%    0.0%    0.1%    0.0%    0.0%    0.0%    0.1%    0.0%    0.0%    0.0%    0.0%    0.0%    0.1%    0.0%    0.1%    0.0%    0.1%    0.1%    0.0%    0.0%    0.0%    0.0%    0.0%    0.0%    0.0%    0.0%    0.0%    0.0%    0.1%    0.1%    0.0%    0.0%    0.1%    0.0%    0.0%    0.0%    0.0%    0.0%    0.0%    0.0%    0.0%    0.2%    0.1%    0.0%    0.0%    0.1%    0.0%    0.1%    0.1%    0.0%    0.0%    0.0%    0.1%    0.0%    0.0%    0.1%    0.0%    0.1%    0.0%    0.0%    0.0%    0.0%    0.0%    0.0%    0.1%    0.0%    0.0%    0.0%    0.0%    0.1%    0.1%    0.0%    0.0%    0.0%    0.1%    0.0%    0.1%    0.1%    0.0%    0.0%    0.1%    0.0%    0.0%    0.0%    0.1%    0.0%    0.0%    0.1%    0.0%    0.1%    0.0%    0.0%    0.0%    0.0%    0.0%    0.0%    0.0%    0.3%    0.0%    0.0%    0.1%    0.1%    0.0%    0.1%    0.1%    0.0%    0.1%    0.2%    0.0%    0.0%    0.1%    0.0%    0.2%    0.0%    0.0%    0.0%    0.0%    0.0%    0.0%    0.0%    0.0%    0.0%    0.1%    0.0%    0.0%    0.0%    0.0%    0.0%    0.0%    0.0%    0.1%    0.0%    0.0%    0.0%    0.2%    0.0%    0.0%    0.1%    0.3%    0.1%    0.1%    0.0%    0.0%    0.0%    0.1%    0.0%    0.0%    0.0%    0.1%    0.1%    0.1%    0.0%    0.0%    0.0%    0.0%    0.0%    0.0%    0.0%    0.0%    0.0%    0.0%    0.0%    0.0%    0.0%    0.0%    0.0%    0.0%    0.0%    0.0%    0.0%    0.0%    0.0%    0.0%    0.0%    0.1%    0.0%    0.0%    0.0%    0.0%    0.0%    0.0%    0.2%    0.0%    0.1%    0.0%    0.0%    0.1%    0.1%    0.1%    0.0%    0.1%    0.0%    0.0%    0.0%    0.1%    0.0%    0.0%    0.0%    0.0%    0.0%    0.0%    0.1%    0.0%    0.1%    0.0%    0.0%    0.1%    0.1%    0.0%    0.0%    0.2%    0.0%    0.1%    0.0%    0.3%    0.0%    0.0%    0.0%    0.0%    0.0%    0.0%    0.0%    0.0%    0.0%    0.1%    0.0%    0.0%    0.0%    0.2%    0.1%    0.0%    0.0%    0.0%    0.0%    0.0%    0.0%    0.0%    0.1%    0.1%    0.2%    0.1%    0.0%    0.0%    0.0%    0.0%    0.1%    0.1%    0.0%    0.1%    0.3%    0.1%    0.1%    0.0%    0.0%    0.0%    0.0%    0.0%    0.0%    0.1%    0.0%    0.0%    0.0%    0.0%    0.0%    0.0%    0.4%    0.0%    0.0%    0.0%    0.0%    0.2%    0.1%    0.1%    0.1%    0.0%    0.1%    0.0%    0.2%    0.0%    0.1%    0.0%    0.1%    0.1%    0.0%    0.1%    0.0%    0.0%    0.0%    0.0%    0.0%    0.0%    0.0%    0.1%    0.2%    0.0%    0.0%    0.0%    0.0%    0.0%    0.0%    0.0%    0.0%    0.0%    0.0%    0.0%    0.0%    0.0%    0.0%    0.0%    0.0%    0.0%    0.1%    0.0%    0.0%    0.0%    0.0%    0.0%    0.0%    0.0%    0.0%    0.0%    0.0%    0.0%    0.0%    0.0%    0.0%    0.0%    0.0%    0.0%    0.0%    0.0%    0.1%    0.0%    0.0%    0.0%    0.0%        k__Bacteria;p__Proteobacteria;c__Deltaproteobacteria; o__NB1-j     159    0.0%    0.0%    0.0%    0.0%    0.0%    0.0%    0.0%    0.0%    0.0%    0.0%    0.0%    0.0%    0.0%    0.0%    0.0%    0.0%    0.0%    0.0%    0.0%    0.0%    0.0%    0.0%    0.0%    0.0%    0.0%    0.0%    0.0%    0.0%    0.0%    0.0%    0.0%    0.0%    0.0%    0.0%    0.0%    0.0%    0.0%    0.0%    0.0%    0.0%    0.0%    0.0%    0.0%    0.0%    0.0%    0.0%    0.0%    0.0%    0.0%    0.0%    0.0%    0.0%    0.0%    0.0%    0.0%    0.0%    0.0%    0.0%    0.0%    0.0%    0.0%    0.0%    0.0%    0.0%    0.0%    0.0%    0.0%    0.0%    0.0%    0.0%    0.0%    0.0%    0.0%    0.0%    0.0%    0.0%    0.0%    0.0%    0.0%    0.0%    0.0%    0.0%    0.0%    0.0%    0.0%    0.0%    0.0%    0.0%    0.0%    0.0%    0.0%    0.0%    0.0%    0.0%    0.0%    0.0%    0.0%    0.0%    0.0%    0.0%    0.0%    0.0%    0.0%    0.0%    0.0%    0.0%    0.0%    0.0%    0.0%    0.0%    0.0%    0.0%    0.0%    0.0%    0.0%    0.0%    0.0%    0.0%    0.0%    0.0%    0.0%    0.0%    0.0%    0.0%    0.0%    0.0%    0.0%    0.0%    0.0%    0.0%    0.0%    0.0%    0.0%    0.0%    0.0%    0.0%    0.0%    0.0%    0.0%    0.0%    0.0%    0.0%    0.0%    0.0%    0.0%    0.0%    0.0%    0.0%    0.0%    0.0%    0.0%    0.0%    0.0%    0.0%    0.0%    0.0%    0.0%    0.0%    0.0%    0.0%    0.0%    0.0%    0.0%    0.0%    0.0%    0.0%    0.0%    0.0%    0.0%    0.0%    0.0%    0.0%    0.0%    0.0%    0.0%    0.0%    0.0%    0.0%    0.0%    0.0%    0.0%    0.0%    0.0%    0.0%    0.0%    0.0%    0.0%    0.0%    0.0%    0.0%    0.0%    0.0%    0.0%    0.0%    0.0%    0.0%    0.0%    0.0%    0.0%    0.0%    0.0%    0.0%    0.0%    0.0%    0.0%    0.0%    0.0%    0.0%    0.0%    0.0%    0.0%    0.0%    0.0%    0.0%    0.0%    0.0%    0.0%    0.0%    0.0%    0.0%    0.0%    0.0%    0.0%    0.0%    0.0%    0.0%    0.0%    0.0%    0.0%    0.0%    0.0%    0.0%    0.0%    0.0%    0.0%    0.0%    0.0%    0.0%    0.0%    0.0%    0.0%    0.0%    0.0%    0.0%    0.0%    0.0%    0.0%    0.0%    0.0%    0.0%    0.0%    0.0%    0.0%    0.0%    0.0%    0.0%    0.0%    0.0%    0.0%    0.0%    0.0%    0.0%    0.0%    0.0%    0.0%    0.0%    0.0%    0.0%    0.0%    0.0%    0.0%    0.0%    0.0%    0.0%    0.0%    0.0%    0.0%    0.0%    0.0%    0.0%    0.0%    0.0%    0.0%    0.0%    0.0%    0.0%    0.0%    0.0%    0.0%    0.0%    0.0%    0.0%    0.0%    0.0%    0.0%    0.0%    0.0%    0.0%    0.0%    0.0%    0.0%    0.0%    0.0%    0.0%    0.0%    0.0%    0.0%    0.0%    0.0%    0.0%    0.0%    0.0%    0.0%    0.0%    0.0%    0.0%    0.0%    0.0%    0.0%    0.0%    0.0%    0.0%    0.0%    0.0%    0.0%    0.0%    0.0%    0.0%    0.0%    0.0%    0.0%    0.0%    0.0%    0.0%    0.0%    0.0%    0.0%    0.0%    0.0%    0.0%    0.0%    0.0%    0.0%    0.0%    0.0%    0.0%    0.0%    0.0%    0.0%    0.0%    0.0%    0.0%    0.0%    0.0%    0.0%    0.0%    0.0%    0.0%        k__Bacteria;p__Proteobacteria;c__Deltaproteobacteria; o__NKB15       1    0.0%    0.0%    0.0%    0.0%    0.0%    0.0%    0.0%    0.0%    0.0%    0.0%    0.0%    0.0%    0.0%    0.0%    0.0%    0.0%    0.0%    0.0%    0.0%    0.0%    0.0%    0.0%    0.0%    0.0%    0.0%    0.0%    0.0%    0.0%    0.0%    0.0%    0.0%    0.0%    0.0%    0.0%    0.0%    0.0%    0.0%    0.0%    0.0%    0.0%    0.0%    0.0%    0.0%    0.0%    0.0%    0.0%    0.0%    0.0%    0.0%    0.0%    0.0%    0.0%    0.0%    0.0%    0.0%    0.0%    0.0%    0.0%    0.0%    0.0%    0.0%    0.0%    0.0%    0.0%    0.0%    0.0%    0.0%    0.0%    0.0%    0.0%    0.0%    0.0%    0.0%    0.0%    0.0%    0.0%    0.0%    0.0%    0.0%    0.0%    0.0%    0.0%    0.0%    0.0%    0.0%    0.0%    0.0%    0.0%    0.0%    0.0%    0.0%    0.0%    0.0%    0.0%    0.0%    0.0%    0.0%    0.0%    0.0%    0.0%    0.0%    0.0%    0.0%    0.0%    0.0%    0.0%    0.0%    0.0%    0.0%    0.0%    0.0%    0.0%    0.0%    0.0%    0.0%    0.0%    0.0%    0.0%    0.0%    0.0%    0.0%    0.0%    0.0%    0.0%    0.0%    0.0%    0.0%    0.0%    0.0%    0.0%    0.0%    0.0%    0.0%    0.0%    0.0%    0.0%    0.0%    0.0%    0.0%    0.0%    0.0%    0.0%    0.0%    0.0%    0.0%    0.0%    0.0%    0.0%    0.0%    0.0%    0.0%    0.0%    0.0%    0.0%    0.0%    0.0%    0.0%    0.0%    0.0%    0.0%    0.0%    0.0%    0.0%    0.0%    0.0%    0.0%    0.0%    0.0%    0.0%    0.0%    0.0%    0.0%    0.0%    0.0%    0.0%    0.0%    0.0%    0.0%    0.0%    0.0%    0.0%    0.0%    0.0%    0.0%    0.0%    0.0%    0.0%    0.0%    0.0%    0.0%    0.0%    0.0%    0.0%    0.0%    0.0%    0.0%    0.0%    0.0%    0.0%    0.0%    0.0%    0.0%    0.0%    0.0%    0.0%    0.0%    0.0%    0.0%    0.0%    0.0%    0.0%    0.0%    0.0%    0.0%    0.0%    0.0%    0.0%    0.0%    0.0%    0.0%    0.0%    0.0%    0.0%    0.0%    0.0%    0.0%    0.0%    0.0%    0.0%    0.0%    0.0%    0.0%    0.0%    0.0%    0.0%    0.0%    0.0%    0.0%    0.0%    0.0%    0.0%    0.0%    0.0%    0.0%    0.0%    0.0%    0.0%    0.0%    0.0%    0.0%    0.0%    0.0%    0.0%    0.0%    0.0%    0.0%    0.0%    0.0%    0.0%    0.0%    0.0%    0.0%    0.0%    0.0%    0.0%    0.0%    0.0%    0.0%    0.0%    0.0%    0.0%    0.0%    0.0%    0.0%    0.0%    0.0%    0.0%    0.0%    0.0%    0.0%    0.0%    0.0%    0.0%    0.0%    0.0%    0.0%    0.0%    0.0%    0.0%    0.0%    0.0%    0.0%    0.0%    0.0%    0.0%    0.0%    0.0%    0.0%    0.0%    0.0%    0.0%    0.0%    0.0%    0.0%    0.0%    0.0%    0.0%    0.0%    0.0%    0.0%    0.0%    0.0%    0.0%    0.0%    0.0%    0.0%    0.0%    0.0%    0.0%    0.0%    0.0%    0.0%    0.0%    0.0%    0.0%    0.0%    0.0%    0.0%    0.0%    0.0%    0.0%    0.0%    0.0%    0.0%    0.0%    0.0%    0.0%    0.0%    0.0%    0.0%    0.0%    0.0%    0.0%    0.0%    0.0%    0.0%    0.0%    0.0%    0.0%    0.0%    0.0%    0.0%    0.0%    0.0%    0.0%    0.0%    0.0%    0.0%        k__Bacteria;p__Proteobacteria;c__Deltaproteobacteria; o__Syntrophobacterales    1209    0.0%    0.0%    0.0%    0.0%    0.0%    0.0%    0.0%    0.0%    0.0%    0.0%    0.0%    0.0%    0.0%    0.0%    0.0%    0.0%    0.0%    0.0%    0.0%    0.0%    0.0%    0.0%    0.0%    0.0%    0.0%    0.0%    0.0%    0.0%    0.0%    0.0%    0.0%    0.0%    0.0%    0.0%    0.0%    0.0%    0.0%    0.0%    0.0%    0.0%    0.0%    0.0%    0.0%    0.0%    0.0%    0.0%    0.0%    0.0%    0.0%    0.0%    0.0%    0.0%    0.0%    0.0%    0.0%    0.0%    0.0%    0.0%    0.0%    0.0%    0.0%    0.0%    0.0%    0.0%    0.0%    0.0%    0.0%    0.0%    0.0%    0.0%    0.0%    0.0%    0.0%    0.0%    0.0%    0.0%    0.0%    0.0%    0.0%    0.0%    0.0%    0.0%    0.0%    0.0%    0.0%    0.0%    0.0%    0.0%    0.0%    0.0%    0.0%    0.0%    0.0%    0.0%    0.0%    0.0%    0.0%    0.0%    0.0%    0.0%    0.0%    0.0%    0.0%    0.0%    0.0%    0.0%    0.0%    0.0%    0.0%    0.0%    0.0%    0.0%    0.0%    0.0%    0.0%    0.0%    0.0%    0.0%    0.0%    0.0%    0.0%    0.0%    0.0%    0.0%    0.0%    0.1%    0.0%    0.0%    0.0%    0.0%    0.0%    0.0%    0.0%    0.0%    0.0%    0.0%    0.0%    0.0%    0.0%    0.0%    0.0%    0.0%    0.0%    0.0%    0.0%    0.0%    0.0%    0.0%    0.0%    0.0%    0.0%    0.0%    0.0%    0.0%    0.0%    0.0%    0.0%    0.0%    0.0%    0.0%    0.0%    0.0%    0.0%    0.0%    0.0%    0.0%    0.0%    0.0%    0.0%    0.0%    0.0%    0.0%    0.0%    0.0%    0.0%    0.0%    0.0%    0.0%    0.0%    0.0%    0.0%    0.0%    0.0%    0.0%    0.0%    0.0%    0.0%    0.0%    0.0%    0.0%    0.0%    0.0%    0.0%    0.0%    0.0%    0.0%    0.0%    0.0%    0.0%    0.0%    0.0%    0.0%    0.0%    0.0%    0.0%    0.0%    0.0%    0.0%    0.0%    0.0%    0.0%    0.0%    0.1%    0.0%    0.0%    0.0%    0.0%    0.0%    0.0%    0.0%    0.1%    0.0%    0.0%    0.0%    0.0%    0.0%    0.0%    0.0%    0.0%    0.0%    0.0%    0.0%    0.0%    0.0%    0.0%    0.0%    0.0%    0.0%    0.0%    0.0%    0.0%    0.0%    0.0%    0.0%    0.0%    0.0%    0.0%    0.0%    0.0%    0.0%    0.0%    0.0%    0.0%    0.0%    0.0%    0.0%    0.0%    0.0%    0.0%    0.0%    0.0%    0.0%    0.0%    0.0%    0.0%    0.0%    0.0%    0.0%    0.0%    0.0%    0.0%    0.0%    0.0%    0.0%    0.0%    0.0%    0.0%    0.0%    0.0%    0.0%    0.0%    0.0%    0.0%    0.0%    0.0%    0.0%    0.0%    0.0%    0.0%    0.0%    0.0%    0.0%    0.0%    0.0%    0.0%    0.0%    0.0%    0.0%    0.0%    0.0%    0.0%    0.0%    0.0%    0.1%    0.0%    0.0%    0.0%    0.0%    0.0%    0.0%    0.0%    0.0%    0.0%    0.0%    0.0%    0.0%    0.1%    0.0%    0.0%    0.0%    0.0%    0.0%    0.0%    0.0%    0.0%    0.0%    0.0%    0.0%    0.0%    0.0%    0.0%    0.0%    0.0%    0.0%    0.0%    0.0%    0.0%    0.0%    0.0%    0.0%    0.0%    0.0%    0.0%    0.0%    0.0%    0.0%    0.0%    0.0%    0.0%    0.0%    0.0%    0.0%    0.0%    0.0%    0.0%    0.0%    0.0%    0.0%        k__Bacteria;p__Proteobacteria;c__Epsilonproteobacteria; o__       6    0.0%    0.0%    0.0%    0.0%    0.0%    0.0%    0.0%    0.0%    0.0%    0.0%    0.0%    0.0%    0.0%    0.0%    0.0%    0.0%    0.0%    0.0%    0.0%    0.0%    0.0%    0.0%    0.0%    0.0%    0.0%    0.0%    0.0%    0.0%    0.0%    0.0%    0.0%    0.0%    0.0%    0.0%    0.0%    0.0%    0.0%    0.0%    0.0%    0.0%    0.0%    0.0%    0.0%    0.0%    0.0%    0.0%    0.0%    0.0%    0.0%    0.0%    0.0%    0.0%    0.0%    0.0%    0.0%    0.0%    0.0%    0.0%    0.0%    0.0%    0.0%    0.0%    0.0%    0.0%    0.0%    0.0%    0.0%    0.0%    0.0%    0.0%    0.0%    0.0%    0.0%    0.0%    0.0%    0.0%    0.0%    0.0%    0.0%    0.0%    0.0%    0.0%    0.0%    0.0%    0.0%    0.0%    0.0%    0.0%    0.0%    0.0%    0.0%    0.0%    0.0%    0.0%    0.0%    0.0%    0.0%    0.0%    0.0%    0.0%    0.0%    0.0%    0.0%    0.0%    0.0%    0.0%    0.0%    0.0%    0.0%    0.0%    0.0%    0.0%    0.0%    0.0%    0.0%    0.0%    0.0%    0.0%    0.0%    0.0%    0.0%    0.0%    0.0%    0.0%    0.0%    0.0%    0.0%    0.0%    0.0%    0.0%    0.0%    0.0%    0.0%    0.0%    0.0%    0.0%    0.0%    0.0%    0.0%    0.0%    0.0%    0.0%    0.0%    0.0%    0.0%    0.0%    0.0%    0.0%    0.0%    0.0%    0.0%    0.0%    0.0%    0.0%    0.0%    0.0%    0.0%    0.0%    0.0%    0.0%    0.0%    0.0%    0.0%    0.0%    0.0%    0.0%    0.0%    0.0%    0.0%    0.0%    0.0%    0.0%    0.0%    0.0%    0.0%    0.0%    0.0%    0.0%    0.0%    0.0%    0.0%    0.0%    0.0%    0.0%    0.0%    0.0%    0.0%    0.0%    0.0%    0.0%    0.0%    0.0%    0.0%    0.0%    0.0%    0.0%    0.0%    0.0%    0.0%    0.0%    0.0%    0.0%    0.0%    0.0%    0.0%    0.0%    0.0%    0.0%    0.0%    0.0%    0.0%    0.0%    0.0%    0.0%    0.0%    0.0%    0.0%    0.0%    0.0%    0.0%    0.0%    0.0%    0.0%    0.0%    0.0%    0.0%    0.0%    0.0%    0.0%    0.0%    0.0%    0.0%    0.0%    0.0%    0.0%    0.0%    0.0%    0.0%    0.0%    0.0%    0.0%    0.0%    0.0%    0.0%    0.0%    0.0%    0.0%    0.0%    0.0%    0.0%    0.0%    0.0%    0.0%    0.0%    0.0%    0.0%    0.0%    0.0%    0.0%    0.0%    0.0%    0.0%    0.0%    0.0%    0.0%    0.0%    0.0%    0.0%    0.0%    0.0%    0.0%    0.0%    0.0%    0.0%    0.0%    0.0%    0.0%    0.0%    0.0%    0.0%    0.0%    0.0%    0.0%    0.0%    0.0%    0.0%    0.0%    0.0%    0.0%    0.0%    0.0%    0.0%    0.0%    0.0%    0.0%    0.0%    0.0%    0.0%    0.0%    0.0%    0.0%    0.0%    0.0%    0.0%    0.0%    0.0%    0.0%    0.0%    0.0%    0.0%    0.0%    0.0%    0.0%    0.0%    0.0%    0.0%    0.0%    0.0%    0.0%    0.0%    0.0%    0.0%    0.0%    0.0%    0.0%    0.0%    0.0%    0.0%    0.0%    0.0%    0.0%    0.0%    0.0%    0.0%    0.0%    0.0%    0.0%    0.0%    0.0%    0.0%    0.0%    0.0%    0.0%    0.0%    0.0%    0.0%    0.0%    0.0%    0.0%    0.0%    0.0%    0.0%    0.0%    0.0%    0.0%    0.0%    0.0%    0.0%        k__Bacteria;p__Proteobacteria;c__Epsilonproteobacteria; o__Campylobacterales   25720    0.2%    0.6%    0.5%    1.4%    0.4%    0.0%    0.3%    0.3%    0.1%    0.1%    0.3%    0.1%    0.0%    0.3%    0.5%    0.1%    0.2%    0.1%    0.1%    0.1%    0.2%    0.3%    0.0%    0.2%    0.0%    0.1%    0.0%    0.1%    0.2%    0.1%    0.1%    0.3%    0.2%    0.4%    1.0%    1.2%    0.4%    0.3%    0.3%    0.1%    0.2%    0.3%    0.4%    0.3%    0.1%    0.2%    0.0%    0.1%    0.2%    0.2%    0.3%    0.0%    0.1%    0.2%    0.1%    0.2%    0.1%    0.0%    0.1%    0.1%    0.0%    0.1%    0.1%    0.1%    3.3%    0.1%    0.0%    0.2%    0.0%    0.1%    0.4%    0.1%    0.0%    0.1%    0.2%    0.0%    0.1%    0.1%    0.2%    0.1%    0.1%    0.1%    0.3%    0.1%    0.4%    0.3%    0.2%    0.0%    0.2%    0.1%    0.0%    0.0%    0.1%    0.0%    0.0%    0.0%    0.1%    0.0%    0.1%    0.1%    0.1%    0.1%    0.1%    0.0%    0.2%    0.1%    0.2%    0.0%    0.0%    0.2%    0.1%    0.5%    0.1%    0.1%    0.1%    0.1%    0.0%    0.2%    0.1%    0.3%    0.0%    0.1%    0.1%    0.1%    0.0%    0.6%    0.3%    0.0%    0.1%    0.1%    0.2%    0.0%    0.0%    0.1%    0.1%    0.0%    0.0%    0.1%    0.0%    0.1%    0.0%    0.2%    0.1%    0.0%    0.0%    0.1%    0.3%    0.1%    0.2%    0.0%    0.0%    0.0%    0.1%    0.1%    0.2%    0.0%    0.1%    0.0%    0.0%    0.2%    0.0%    0.2%    0.1%    0.0%    0.0%    0.0%    0.1%    0.4%    0.1%    0.2%    0.1%    0.2%    0.0%    0.2%    0.2%    0.1%    0.1%    0.1%    0.0%    0.1%    0.2%    0.1%    0.1%    0.0%    0.2%    0.1%    0.3%    0.1%    0.1%    0.2%    0.2%    0.6%    0.1%    0.2%    0.1%    0.1%    0.2%    0.3%    0.1%    0.1%    0.2%    0.2%    0.1%    0.1%    0.2%    0.1%    0.0%    0.1%    0.1%    0.1%    0.2%    0.0%    0.0%    0.8%    0.0%    0.1%    0.0%    0.0%    0.4%    0.1%    0.0%    0.1%    0.0%    0.1%    0.1%    0.1%    0.2%    0.2%    0.1%    0.2%    0.1%    0.0%    0.0%    0.1%    0.1%    0.1%    0.1%    0.1%    0.0%    0.0%    0.2%    0.0%    0.0%    0.1%    0.0%    0.0%    0.0%    0.0%    0.0%    0.5%    0.1%    0.3%    0.1%    0.2%    0.0%    0.0%    0.1%    0.0%    0.2%    0.2%    0.2%    0.0%    0.1%    0.0%    0.2%    0.0%    0.1%    0.1%    0.1%    0.0%    0.1%    0.1%    0.1%    0.2%    0.1%    0.2%    0.2%    0.1%    0.1%    0.8%    0.0%    0.0%    0.0%    0.3%    0.1%    0.2%    0.1%    0.0%    0.1%    0.0%    0.0%    0.2%    0.1%    0.2%    0.0%    0.1%    0.1%    0.1%    0.1%    0.2%    0.3%    0.1%    0.2%    0.1%    0.2%    0.1%    0.1%    0.0%    0.0%    0.4%    0.0%    0.0%    0.0%    0.3%    0.1%    0.1%    0.0%    0.1%    0.3%    0.0%    0.4%    0.3%    1.2%    0.0%    0.2%    0.1%    0.1%    0.1%    0.4%    0.2%    0.0%    0.0%    0.0%    0.1%    0.0%    0.0%    0.1%    0.7%    0.3%    0.0%    0.0%    0.0%    0.1%    0.1%    0.1%    0.3%    0.0%    0.3%    0.0%    0.1%    0.0%    0.3%    0.2%    0.1%    0.0%    0.0%    0.0%    0.0%        k__Bacteria;p__Proteobacteria;c__Gammaproteobacteria; o__    2454    0.0%    0.0%    0.0%    0.0%    0.2%    0.1%    0.0%    0.1%    0.0%    0.0%    0.0%    0.3%    0.0%    0.1%    0.1%    0.1%    0.0%    0.0%    0.0%    0.1%    0.0%    0.0%    0.1%    0.0%    0.0%    0.0%    0.0%    0.1%    0.0%    0.0%    0.0%    0.0%    0.0%    0.0%    0.1%    0.0%    0.1%    0.0%    0.0%    0.0%    0.0%    0.0%    0.1%    0.0%    0.0%    0.0%    0.0%    0.0%    0.0%    0.0%    0.0%    0.1%    0.2%    0.0%    0.0%    0.0%    0.1%    0.0%    0.1%    0.0%    0.0%    0.0%    0.0%    0.0%    0.0%    0.0%    0.0%    0.0%    0.0%    0.0%    0.0%    0.0%    0.0%    0.0%    0.0%    0.0%    0.0%    0.0%    0.0%    0.0%    0.0%    0.0%    0.0%    0.0%    0.0%    0.0%    0.0%    0.0%    0.0%    0.0%    0.0%    0.0%    0.0%    0.0%    0.0%    0.0%    0.0%    0.0%    0.0%    0.0%    0.0%    0.0%    0.0%    0.0%    0.0%    0.0%    0.0%    0.0%    0.0%    0.0%    0.0%    0.0%    0.0%    0.0%    0.0%    0.0%    0.0%    0.0%    0.0%    0.0%    0.0%    0.0%    0.0%    0.0%    0.0%    0.0%    0.0%    0.0%    0.0%    0.0%    0.0%    0.0%    0.0%    0.0%    0.0%    0.0%    0.0%    0.0%    0.0%    0.0%    0.0%    0.0%    0.0%    0.0%    0.0%    0.0%    0.0%    0.0%    0.0%    0.0%    0.0%    0.0%    0.0%    0.0%    0.0%    0.0%    0.0%    0.0%    0.0%    0.0%    0.0%    0.0%    0.0%    0.0%    0.0%    0.0%    0.0%    0.0%    0.1%    0.0%    0.0%    0.0%    0.0%    0.0%    0.0%    0.0%    0.0%    0.0%    0.0%    0.0%    0.0%    0.0%    0.0%    0.0%    0.0%    0.0%    0.0%    0.0%    0.0%    0.0%    0.0%    0.0%    0.0%    0.0%    0.0%    0.1%    0.0%    0.0%    0.0%    0.0%    0.0%    0.0%    0.0%    0.0%    0.0%    0.2%    0.0%    0.0%    0.0%    0.0%    0.0%    0.0%    0.0%    0.0%    0.0%    0.0%    0.0%    0.0%    0.0%    0.1%    0.1%    0.0%    0.0%    0.0%    0.0%    0.0%    0.0%    0.0%    0.0%    0.0%    0.0%    0.0%    0.0%    0.0%    0.0%    0.0%    0.0%    0.0%    0.0%    0.0%    0.0%    0.0%    0.0%    0.0%    0.0%    0.0%    0.0%    0.0%    0.0%    0.0%    0.0%    0.0%    0.0%    0.0%    0.0%    0.0%    0.0%    0.0%    0.0%    0.0%    0.0%    0.0%    0.0%    0.0%    0.0%    0.0%    0.0%    0.0%    0.0%    0.0%    0.0%    0.0%    0.0%    0.0%    0.0%    0.0%    0.0%    0.0%    0.0%    0.0%    0.0%    0.0%    0.0%    0.0%    0.0%    0.0%    0.0%    0.0%    0.0%    0.0%    0.0%    0.0%    0.0%    0.0%    0.0%    0.0%    0.0%    0.0%    0.1%    0.0%    0.0%    0.0%    0.0%    0.0%    0.0%    0.0%    0.0%    0.0%    0.0%    0.0%    0.1%    0.0%    0.0%    0.0%    0.0%    0.0%    0.0%    0.0%    0.0%    0.0%    0.0%    0.0%    0.0%    0.0%    0.0%    0.0%    0.0%    0.0%    0.0%    0.0%    0.0%    0.0%    0.0%    0.0%    0.0%    0.0%    0.0%    0.0%    0.0%    0.0%    0.0%    0.0%    0.0%    0.0%    0.0%    0.0%    0.0%    0.0%    0.0%    0.0%    0.0%    0.0%    0.0%    0.0%    0.0%    0.0%    0.0%    0.0%        k__Bacteria;p__Proteobacteria;c__Gammaproteobacteria; o__Aeromonadales   11543    0.1%    0.0%    0.1%    0.1%    0.4%    0.1%    0.0%    0.0%    0.0%    0.0%    0.0%    0.0%    0.1%    0.0%    0.0%    0.0%    0.1%    0.1%    0.0%    0.0%    2.1%    0.6%    0.1%    0.0%    0.0%    0.0%    0.0%    0.1%    0.2%    0.0%    0.0%    0.1%    0.0%    0.0%    1.0%    0.1%    0.3%    0.0%    0.0%    0.2%    0.0%    0.2%    0.0%    0.0%    0.3%    0.2%    0.0%    0.0%    0.0%    0.0%    0.0%    0.0%    0.0%    0.0%    0.2%    0.0%    0.0%    0.0%    0.0%    0.0%    0.0%    0.0%    0.0%    0.1%    0.6%    0.0%    0.0%    0.2%    0.0%    0.1%    0.0%    0.0%    0.0%    0.0%    0.1%    0.0%    0.0%    0.0%    0.0%    0.0%    0.0%    0.0%    0.0%    0.0%    0.1%    0.0%    0.0%    0.0%    0.0%    0.1%    0.0%    0.0%    0.1%    0.0%    0.0%    0.0%    0.0%    0.0%    0.0%    0.1%    0.0%    0.0%    0.0%    0.2%    0.0%    0.0%    0.1%    0.0%    0.0%    0.0%    0.0%    0.1%    0.0%    0.0%    0.0%    0.0%    0.0%    0.0%    0.0%    0.0%    0.0%    0.0%    0.0%    0.0%    0.0%    0.0%    0.0%    0.1%    0.0%    0.0%    0.1%    0.0%    0.0%    0.0%    0.4%    0.0%    0.0%    0.0%    0.0%    0.0%    0.0%    0.3%    0.1%    0.0%    0.0%    0.0%    0.0%    0.0%    0.0%    0.0%    0.0%    0.0%    0.1%    0.0%    0.0%    0.0%    0.3%    0.0%    0.0%    0.1%    0.0%    0.0%    0.1%    0.0%    0.0%    0.0%    0.1%    0.0%    0.8%    0.0%    0.3%    0.0%    0.1%    0.1%    0.0%    0.0%    0.0%    0.0%    0.0%    0.0%    0.0%    0.1%    0.0%    0.0%    0.0%    0.1%    0.1%    0.1%    0.0%    0.0%    0.0%    0.1%    0.0%    0.0%    0.0%    0.0%    0.0%    0.0%    0.0%    0.1%    0.1%    0.7%    0.0%    0.4%    0.0%    0.1%    0.0%    0.1%    0.1%    0.1%    0.0%    0.0%    0.0%    0.0%    0.0%    0.0%    0.0%    0.0%    0.0%    0.2%    0.1%    0.0%    0.1%    0.0%    0.0%    0.1%    0.0%    0.0%    0.0%    0.0%    0.0%    0.0%    0.1%    0.0%    0.0%    0.0%    0.0%    0.0%    0.0%    0.1%    0.1%    0.0%    0.0%    0.0%    0.0%    0.0%    0.0%    0.0%    0.0%    0.0%    0.1%    0.1%    0.1%    0.0%    0.1%    0.0%    0.0%    0.0%    0.0%    0.0%    0.0%    0.0%    0.0%    0.2%    0.0%    0.0%    0.0%    0.0%    0.0%    0.0%    0.0%    0.1%    0.0%    0.0%    0.0%    0.0%    0.0%    0.0%    0.0%    0.2%    0.0%    0.0%    0.0%    0.0%    0.0%    0.0%    0.0%    0.2%    0.0%    0.0%    0.0%    0.0%    0.1%    0.1%    0.0%    0.0%    0.1%    0.0%    0.1%    0.1%    0.0%    0.0%    0.0%    0.0%    0.0%    0.0%    0.0%    0.0%    0.1%    0.0%    0.0%    0.0%    0.0%    0.0%    0.0%    0.1%    0.0%    0.0%    0.0%    0.1%    0.0%    0.1%    0.0%    0.0%    0.1%    0.0%    0.0%    0.0%    0.0%    0.0%    0.0%    0.0%    0.0%    0.0%    0.0%    0.0%    0.0%    0.0%    0.0%    0.0%    0.0%    0.0%    0.0%    0.0%    0.0%    0.0%    0.0%    0.0%    0.0%    0.0%    0.0%    0.0%    0.0%    0.0%    0.0%    0.0%    0.0%    0.0%        k__Bacteria;p__Proteobacteria;c__Gammaproteobacteria; o__Alteromonadales   26525    0.2%    0.1%    0.0%    0.0%    0.1%    0.1%    0.0%    0.0%    0.0%    0.0%    0.0%    0.0%    0.0%    0.1%    0.0%    0.0%    0.1%    0.0%    0.0%    0.0%    0.2%    1.8%    0.0%    0.0%    0.0%    0.0%    0.1%    0.0%    0.0%    0.0%    0.0%    0.0%    0.1%    0.0%    0.6%    0.1%    0.4%    0.0%    0.0%    0.8%    0.1%    0.2%    0.0%    0.0%    0.2%    0.1%    0.1%    0.0%    0.0%    0.0%    0.0%    0.1%    0.0%    0.0%    0.0%    0.0%    0.0%    0.0%    0.0%    0.0%    0.0%    0.0%    0.0%    0.0%    0.0%    0.0%    0.0%    0.0%    0.0%    0.0%    0.0%    0.0%    0.0%    0.0%    0.0%    0.0%    0.0%    0.0%    0.0%    0.0%    0.0%    0.0%    0.3%    0.1%    0.1%    0.0%    0.1%    0.0%    0.0%    0.0%    0.0%    0.0%    0.0%    0.0%    0.0%    0.0%    0.0%    0.0%    0.0%    0.0%    0.1%    0.0%    0.0%    0.0%    0.0%    0.0%    0.1%    0.0%    0.0%    0.0%    0.0%    0.0%    0.2%    0.0%    0.0%    0.0%    0.0%    0.0%    0.0%    0.0%    0.0%    0.0%    0.0%    0.0%    0.0%    0.1%    0.1%    6.5%    0.1%    0.0%    0.2%    0.0%    0.0%    0.1%    0.5%    0.1%    0.1%    0.0%    0.0%    0.1%    0.0%    0.2%    0.1%    0.0%    0.0%    0.0%    0.0%    0.0%    0.0%    0.0%    0.0%    0.0%    0.1%    0.1%    0.6%    0.0%    0.2%    0.5%    0.0%    0.1%    0.0%    0.1%    0.0%    0.4%    0.0%    0.0%    0.0%    0.0%    0.0%    0.0%    1.0%    0.1%    0.2%    0.1%    0.0%    0.1%    0.0%    0.0%    0.0%    0.0%    0.0%    0.0%    0.0%    0.0%    0.0%    0.0%    0.0%    0.1%    0.1%    0.0%    0.0%    0.1%    0.0%    0.1%    0.0%    0.3%    0.0%    0.0%    0.0%    0.0%    0.2%    0.0%    0.0%    0.1%    0.1%    0.0%    0.0%    0.2%    0.1%    0.1%    0.1%    0.0%    0.1%    0.4%    0.0%    0.0%    0.0%    0.0%    0.1%    0.1%    0.0%    0.1%   15.7%    0.1%    0.0%    0.4%    0.0%    0.0%    0.1%    0.0%    0.0%    0.0%    0.1%    0.0%    0.4%    0.0%    0.0%    0.0%    0.0%    0.0%    0.0%    0.0%    0.0%    0.1%    0.0%    0.0%    0.0%    0.0%    0.0%    0.0%    0.1%    0.0%    0.2%    0.0%    0.0%    0.0%    0.0%    0.0%    0.3%    0.0%    0.0%    0.0%    0.1%    0.1%    0.0%    0.0%    0.0%    0.0%    0.2%    0.0%    0.0%    1.4%    0.7%    0.6%    0.0%    0.1%    0.0%    0.1%    0.0%    0.2%    0.3%    0.2%    0.1%    0.0%    0.1%    0.1%    0.3%    0.5%    0.0%    0.0%    0.1%    0.0%    0.2%    0.3%    0.1%    0.0%    0.2%    0.1%    0.1%    0.2%    0.0%    0.7%    0.0%    0.3%    0.1%    0.0%    0.1%    0.0%    0.1%    0.0%    0.0%    0.0%    0.0%    0.1%    0.1%    0.3%    0.0%    0.0%    0.0%    0.2%    0.1%    0.2%    0.0%    0.0%    0.1%    0.2%    0.0%    0.1%    0.0%    0.0%    0.0%    0.0%    0.0%    0.0%    0.0%    0.0%    0.0%    0.0%    0.0%    0.0%    0.1%    0.0%    0.0%    0.0%    0.0%    0.0%    0.0%    0.0%    0.0%    0.0%    0.0%    0.0%    0.0%    0.0%    0.0%    0.0%    0.0%    0.0%        k__Bacteria;p__Proteobacteria;c__Gammaproteobacteria; o__Cardiobacteriales   13582    0.1%    0.0%    0.0%    0.0%    0.0%    0.0%    0.0%    0.0%    0.0%    0.0%    0.0%    0.0%    0.0%    0.1%    0.1%    0.1%    0.1%    0.0%    0.1%    0.0%    0.0%    0.0%    0.1%    0.0%    0.0%    0.1%    0.0%    0.1%    0.0%    0.1%    0.0%    0.3%    0.0%    0.4%    0.0%    0.8%    0.3%    0.0%    0.3%    0.1%    0.2%    0.2%    0.8%    0.5%    0.3%    0.1%    0.2%    0.1%    0.6%    0.3%    0.0%    0.0%    0.0%    0.0%    0.0%    0.0%    0.0%    0.0%    0.1%    0.0%    0.0%    0.1%    0.1%    0.0%    0.0%    0.0%    0.0%    0.0%    0.0%    0.0%    0.1%    0.1%    0.0%    0.1%    0.0%    0.0%    0.0%    0.0%    0.0%    0.0%    0.0%    0.0%    0.1%    0.1%    0.0%    0.1%    0.1%    0.0%    0.2%    0.0%    0.0%    0.0%    0.0%    0.0%    0.0%    0.0%    0.0%    0.0%    0.0%    0.1%    0.0%    0.0%    0.0%    0.1%    0.1%    0.4%    0.0%    0.0%    0.0%    0.0%    0.0%    0.0%    0.1%    0.0%    0.0%    0.1%    0.0%    0.0%    0.0%    0.1%    0.0%    0.1%    0.0%    0.2%    0.0%    0.0%    0.2%    0.0%    0.0%    0.1%    0.0%    0.0%    0.0%    0.1%    0.0%    0.0%    0.0%    0.0%    0.0%    0.1%    0.0%    0.1%    0.0%    0.0%    0.0%    0.1%    0.4%    0.0%    0.0%    0.0%    0.0%    0.0%    0.0%    0.0%    0.0%    0.0%    0.0%    0.0%    0.0%    0.1%    0.0%    0.1%    0.2%    0.1%    0.0%    0.0%    0.1%    0.1%    0.1%    0.2%    0.0%    0.3%    0.0%    0.1%    0.2%    0.0%    0.1%    0.1%    0.0%    0.1%    0.0%    0.1%    0.0%    0.0%    0.0%    0.0%    0.0%    0.1%    0.1%    0.2%    0.0%    0.1%    0.0%    0.0%    0.0%    0.0%    0.1%    0.2%    0.0%    0.1%    0.1%    0.0%    0.0%    0.0%    0.3%    0.1%    0.0%    0.1%    0.0%    0.0%    0.0%    0.0%    0.1%    2.1%    0.0%    0.0%    0.1%    0.0%    0.0%    0.0%    0.0%    0.0%    0.0%    0.0%    0.0%    0.0%    0.0%    0.2%    0.0%    0.8%    0.0%    0.0%    0.0%    0.0%    0.0%    0.0%    0.0%    0.0%    0.0%    0.0%    0.0%    0.0%    0.2%    0.1%    0.0%    0.0%    0.1%    0.0%    0.0%    0.0%    0.0%    0.2%    0.0%    0.0%    0.0%    0.0%    0.2%    0.0%    0.0%    0.2%    0.0%    0.0%    0.0%    0.0%    0.2%    0.0%    0.0%    0.1%    0.0%    0.0%    0.1%    0.1%    0.1%    0.1%    0.0%    0.0%    0.1%    0.1%    0.1%    0.0%    0.0%    0.0%    0.0%    0.0%    0.1%    0.2%    0.1%    0.0%    0.0%    0.0%    0.0%    0.0%    0.1%    0.1%    0.2%    0.0%    0.0%    0.1%    0.1%    0.0%    0.0%    0.0%    0.0%    0.0%    0.1%    0.1%    0.0%    0.0%    0.1%    0.7%    0.2%    0.0%    0.1%    0.4%    0.2%    0.0%    0.0%    0.1%    0.1%    0.0%    0.2%    0.5%    0.0%    0.0%    0.2%    0.2%    0.1%    0.0%    0.2%    0.1%    0.3%    0.1%    0.0%    0.0%    0.0%    0.1%    0.1%    0.1%    0.3%    0.0%    0.0%    0.0%    0.1%    0.0%    0.0%    0.1%    0.0%    0.2%    0.0%    0.0%    0.1%    0.2%    0.0%    0.0%    0.1%    0.0%    0.0%    0.0%        k__Bacteria;p__Proteobacteria;c__Gammaproteobacteria; o__Chromatiales    8149    0.0%    0.0%    0.0%    0.0%    0.0%    0.0%    0.0%    0.0%    0.0%    0.0%    0.0%    0.0%    0.0%    0.0%    0.0%    0.0%    0.0%    0.1%    0.0%    0.0%    0.0%    0.0%    0.0%    0.0%    0.0%    0.0%    0.0%    0.0%    0.0%    0.0%    0.0%    0.0%    0.0%    0.0%    0.0%    0.0%    0.0%    0.0%    0.0%    0.1%    0.0%    0.0%    0.0%    0.0%    0.0%    0.0%    0.0%    0.0%    0.0%    0.0%    0.0%    0.0%    0.0%    0.0%    0.1%    0.1%    0.0%    0.0%    0.0%    0.1%    0.0%    0.1%    0.0%    0.1%    0.1%    0.0%    0.0%    0.1%    0.1%    0.0%    0.0%    0.0%    0.0%    0.1%    0.0%    0.0%    0.0%    0.1%    0.0%    0.1%    0.0%    0.0%    0.0%    0.0%    0.0%    0.0%    0.1%    0.0%    0.0%    0.1%    0.0%    0.0%    0.0%    0.0%    0.0%    0.0%    0.2%    0.0%    0.0%    0.1%    0.1%    0.0%    0.0%    0.1%    0.0%    0.0%    0.2%    0.0%    0.0%    0.0%    0.1%    0.1%    0.0%    0.0%    0.0%    0.0%    0.0%    0.0%    0.0%    0.2%    0.1%    0.1%    0.2%    0.0%    0.0%    0.4%    0.1%    0.1%    0.1%    0.3%    0.1%    0.0%    0.1%    0.0%    0.2%    0.0%    0.0%    0.1%    0.1%    0.0%    0.1%    0.0%    0.1%    0.1%    0.1%    0.0%    0.0%    0.0%    0.0%    0.0%    0.0%    0.0%    0.1%    0.0%    0.0%    0.0%    0.1%    0.0%    0.0%    0.2%    0.3%    0.1%    0.1%    0.1%    0.0%    0.1%    0.1%    0.1%    0.0%    0.0%    0.1%    0.1%    0.1%    0.0%    0.0%    0.0%    0.0%    0.0%    0.0%    0.0%    0.0%    0.0%    0.0%    0.0%    0.0%    0.0%    0.0%    0.0%    0.0%    0.0%    0.0%    0.0%    0.0%    0.0%    0.0%    1.0%    0.1%    0.0%    0.0%    0.0%    0.1%    0.0%    0.0%    0.1%    0.0%    0.0%    0.0%    0.1%    0.1%    0.1%    0.1%    0.0%    0.2%    0.2%    0.0%    0.0%    0.0%    0.0%    0.1%    0.0%    0.0%    0.1%    0.0%    0.1%    0.0%    0.1%    0.0%    0.0%    0.1%    0.0%    0.0%    0.0%    0.1%    0.0%    0.1%    0.0%    0.1%    0.0%    0.0%    0.0%    0.0%    0.0%    0.0%    0.0%    0.0%    0.0%    0.1%    0.0%    0.0%    0.0%    0.1%    0.1%    0.1%    0.0%    0.0%    0.0%    0.1%    0.0%    0.0%    0.0%    0.1%    0.1%    0.1%    0.0%    0.0%    0.0%    0.1%    0.1%    0.0%    0.0%    0.0%    0.1%    0.1%    0.0%    0.0%    0.1%    0.0%    0.0%    0.0%    0.0%    0.0%    0.0%    0.0%    0.0%    0.0%    0.0%    0.0%    0.4%    0.0%    0.0%    0.0%    0.0%    0.0%    0.0%    0.0%    0.0%    0.0%    0.0%    0.0%    0.2%    0.0%    0.0%    0.0%    0.0%    0.0%    0.0%    0.0%    0.0%    0.0%    0.0%    0.0%    0.0%    0.0%    0.0%    0.0%    0.1%    0.0%    0.0%    0.0%    0.0%    0.0%    0.0%    0.0%    0.0%    0.0%    0.0%    0.1%    0.0%    0.1%    0.0%    0.0%    0.0%    0.0%    0.0%    0.0%    0.0%    0.0%    0.0%    0.0%    0.0%    0.0%    0.0%    0.0%    0.0%    0.0%    0.0%    0.0%    0.0%    0.0%    0.0%    0.0%    0.0%    0.0%    0.1%    0.0%    0.0%    0.0%    0.0%        k__Bacteria;p__Proteobacteria;c__Gammaproteobacteria; o__Enterobacteriales   164009    1.0%    0.1%    0.2%    0.3%    0.5%    4.8%    0.1%    0.0%    0.2%    0.1%    0.1%    0.1%    0.6%    0.6%    0.1%    0.3%    0.2%    1.1%    0.2%    0.6%    1.0%    0.3%    0.2%    0.1%    0.1%    0.1%    3.4%    1.8%    0.2%    0.2%    0.0%    0.4%    0.3%    0.5%    0.5%    0.1%    0.3%    0.1%    0.3%    1.7%    0.5%    0.5%    0.0%    0.0%    0.1%    0.1%    0.4%    0.1%    0.3%    0.2%    0.2%    0.3%    0.2%    0.1%    0.7%    0.0%    0.6%    0.2%    0.2%    0.2%    0.7%    0.1%    0.1%    0.4%    0.5%    1.1%    0.2%    0.4%    0.2%    0.3%   13.6%    0.6%    1.2%    0.4%    0.4%    0.1%    0.5%    0.2%    0.0%    0.1%    0.0%    1.5%    0.3%    0.1%    0.2%    0.3%    0.2%    0.8%    0.4%    1.0%    6.7%    2.7%    1.1%    1.3%    0.0%    0.2%    0.7%    2.8%    0.5%    0.1%    2.9%    0.9%    1.0%    0.4%    0.0%    0.1%    1.4%    0.1%    0.1%    0.2%    0.3%    0.2%    0.7%    0.0%    1.0%    0.1%    0.5%    0.1%    0.1%    0.2%    0.4%    0.2%    0.5%    4.2%    0.1%    0.9%    1.5%    0.7%    0.2%    0.2%    0.5%    0.5%    0.7%    0.3%    0.2%    1.1%    1.7%    0.4%    0.4%    0.9%    0.3%    1.1%    0.6%    0.9%    2.2%    0.5%    0.3%    0.7%    0.7%    0.2%    1.1%    1.3%    0.1%    1.4%    0.1%    1.0%    0.5%    2.2%    0.2%    0.9%    0.1%    0.6%    0.2%    0.8%    1.3%    0.6%    0.2%    0.4%    5.5%    0.3%    0.2%    0.5%    0.4%    0.3%    0.8%    0.2%    3.2%    0.1%    2.1%    0.3%    0.5%    0.5%    0.5%    1.0%    0.1%    0.2%    0.5%    0.7%    0.5%    0.5%    0.3%    0.5%    0.2%    0.6%    8.8%    1.8%    1.1%    0.3%    0.2%    0.3%    0.4%    1.8%    0.7%    3.2%    1.3%    0.8%    0.4%    1.1%    2.6%    1.3%    1.0%    0.9%    5.8%    0.5%    3.1%    1.5%    1.7%    0.2%    4.4%    0.7%    0.5%    4.9%    2.2%    0.9%    3.8%    1.2%    0.3%    0.4%    1.7%    2.1%    1.4%    0.7%   11.8%    1.2%    2.6%    5.3%    4.1%    1.3%    0.6%    0.6%    1.5%    0.5%    0.1%    0.2%    0.2%    0.1%    0.0%    0.6%    0.3%    0.5%    1.7%    0.5%    1.7%    0.6%    0.4%    0.6%    1.1%    2.2%    0.9%    0.4%    1.9%    0.6%    1.6%    1.5%    0.9%    0.7%    0.9%    0.8%    5.2%    1.7%    0.7%    1.1%    1.3%    0.9%    1.2%    1.3%    0.6%    4.8%   13.9%    0.3%    0.7%    1.2%    1.2%    1.9%    1.8%    0.0%    0.2%    1.2%    1.0%    0.2%    1.8%    0.0%    0.4%    0.3%    0.1%    0.2%    0.4%    0.1%    0.7%    0.8%    0.1%    0.9%    0.0%    0.4%    0.1%    0.9%    0.1%    0.1%    0.3%    0.0%    0.0%    0.8%   32.5%    0.8%    0.7%    0.5%    0.1%    0.2%    0.2%    0.2%    0.4%    0.3%    0.0%    0.9%    0.5%    0.5%    0.6%    0.2%    0.5%    0.5%    0.0%    0.0%    0.3%    0.1%    0.0%    0.0%    0.0%    0.1%    0.1%    2.6%    0.3%    1.5%    0.2%    0.2%    0.6%    0.1%    0.5%    0.1%    1.4%    0.3%    0.8%    0.1%    0.1%    0.5%    0.1%    0.2%    0.5%    0.0%        k__Bacteria;p__Proteobacteria;c__Gammaproteobacteria; o__Legionellales    1150    0.0%    0.0%    0.0%    0.0%    0.0%    0.0%    0.0%    0.0%    0.0%    0.0%    0.0%    0.0%    0.0%    0.0%    0.0%    0.0%    0.0%    0.0%    0.0%    0.0%    0.0%    0.0%    0.0%    0.0%    0.0%    0.0%    0.0%    0.0%    0.0%    0.0%    0.0%    0.0%    0.0%    0.0%    0.0%    0.0%    0.0%    0.0%    0.0%    0.0%    0.0%    0.0%    0.0%    0.0%    0.0%    0.0%    0.0%    0.0%    0.0%    0.0%    0.0%    0.0%    0.0%    0.0%    0.0%    0.0%    0.0%    0.0%    0.0%    0.0%    0.0%    0.0%    0.0%    0.0%    0.0%    0.0%    0.0%    0.0%    0.0%    0.0%    0.0%    0.0%    0.0%    0.0%    0.0%    0.0%    0.0%    0.0%    0.0%    0.0%    0.0%    0.0%    0.0%    0.0%    0.0%    0.0%    0.0%    0.0%    0.0%    0.0%    0.0%    0.0%    0.0%    0.0%    0.0%    0.0%    0.0%    0.0%    0.0%    0.0%    0.0%    0.0%    0.0%    0.0%    0.0%    0.0%    0.0%    0.0%    0.0%    0.0%    0.0%    0.0%    0.0%    0.0%    0.0%    0.0%    0.0%    0.0%    0.0%    0.0%    0.0%    0.0%    0.0%    0.0%    0.0%    0.0%    0.0%    0.0%    0.0%    0.0%    0.0%    0.0%    0.0%    0.0%    0.0%    0.0%    0.0%    0.0%    0.0%    0.0%    0.0%    0.0%    0.0%    0.0%    0.0%    0.0%    0.0%    0.0%    0.0%    0.0%    0.0%    0.0%    0.0%    0.0%    0.0%    0.0%    0.0%    0.0%    0.0%    0.0%    0.0%    0.0%    0.0%    0.0%    0.0%    0.0%    0.0%    0.0%    0.0%    0.0%    0.1%    0.0%    0.0%    0.0%    0.0%    0.0%    0.0%    0.0%    0.0%    0.0%    0.0%    0.0%    0.0%    0.0%    0.0%    0.0%    0.0%    0.0%    0.0%    0.0%    0.0%    0.0%    0.0%    0.0%    0.0%    0.0%    0.0%    0.0%    0.0%    0.0%    0.1%    0.0%    0.0%    0.0%    0.0%    0.0%    0.0%    0.0%    0.0%    0.0%    0.0%    0.0%    0.0%    0.0%    0.0%    0.0%    0.0%    0.0%    0.0%    0.0%    0.0%    0.0%    0.0%    0.0%    0.0%    0.0%    0.0%    0.0%    0.0%    0.0%    0.0%    0.0%    0.0%    0.0%    0.0%    0.0%    0.0%    0.0%    0.0%    0.0%    0.0%    0.0%    0.0%    0.0%    0.0%    0.0%    0.0%    0.0%    0.0%    0.0%    0.0%    0.0%    0.0%    0.0%    0.0%    0.0%    0.0%    0.0%    0.0%    0.0%    0.0%    0.0%    0.0%    0.0%    0.0%    0.0%    0.0%    0.0%    0.0%    0.0%    0.0%    0.0%    0.0%    0.0%    0.0%    0.0%    0.0%    0.0%    0.0%    0.0%    0.0%    0.0%    0.0%    0.0%    0.0%    0.0%    0.0%    0.2%    0.0%    0.0%    0.0%    0.0%    0.1%    0.0%    0.0%    0.0%    0.0%    0.0%    0.0%    0.0%    0.0%    0.0%    0.0%    0.0%    0.0%    0.0%    0.0%    0.0%    0.0%    0.0%    0.0%    0.0%    0.0%    0.0%    0.0%    0.0%    0.0%    0.0%    0.0%    0.0%    0.0%    0.0%    0.0%    0.0%    0.0%    0.0%    0.0%    0.0%    0.0%    0.0%    0.0%    0.0%    0.0%    0.0%    0.0%    0.0%    0.0%    0.0%    0.0%    0.0%    0.0%    0.0%    0.0%    0.0%    0.0%    0.0%    0.0%    0.0%    0.0%    0.0%    0.0%    0.0%    0.0%    0.0%    0.0%    0.0%    0.0%    0.0%        k__Bacteria;p__Proteobacteria;c__Gammaproteobacteria; o__Methylococcales     655    0.0%    0.0%    0.0%    0.0%    0.0%    0.0%    0.0%    0.0%    0.0%    0.0%    0.0%    0.0%    0.0%    0.0%    0.0%    0.0%    0.0%    0.0%    0.0%    0.0%    0.0%    0.0%    0.0%    0.0%    0.0%    0.0%    0.0%    0.0%    0.0%    0.0%    0.0%    0.0%    0.0%    0.0%    0.0%    0.0%    0.0%    0.0%    0.0%    0.0%    0.0%    0.0%    0.0%    0.0%    0.0%    0.0%    0.0%    0.0%    0.0%    0.0%    0.0%    0.0%    0.0%    0.0%    0.0%    0.0%    0.0%    0.0%    0.0%    0.0%    0.0%    0.0%    0.0%    0.0%    0.0%    0.0%    0.0%    0.0%    0.0%    0.0%    0.0%    0.0%    0.0%    0.0%    0.0%    0.0%    0.0%    0.0%    0.0%    0.0%    0.0%    0.0%    0.0%    0.0%    0.0%    0.0%    0.0%    0.0%    0.0%    0.0%    0.0%    0.0%    0.0%    0.0%    0.0%    0.0%    0.0%    0.0%    0.0%    0.0%    0.0%    0.0%    0.0%    0.0%    0.0%    0.0%    0.0%    0.0%    0.0%    0.0%    0.0%    0.0%    0.0%    0.0%    0.0%    0.0%    0.0%    0.0%    0.0%    0.0%    0.0%    0.0%    0.0%    0.0%    0.0%    0.3%    0.0%    0.0%    0.0%    0.0%    0.0%    0.0%    0.0%    0.0%    0.0%    0.0%    0.0%    0.0%    0.0%    0.0%    0.0%    0.0%    0.0%    0.0%    0.0%    0.0%    0.0%    0.0%    0.0%    0.0%    0.0%    0.0%    0.0%    0.0%    0.0%    0.0%    0.0%    0.0%    0.0%    0.0%    0.0%    0.0%    0.0%    0.0%    0.0%    0.0%    0.0%    0.0%    0.0%    0.0%    0.0%    0.0%    0.0%    0.0%    0.0%    0.0%    0.0%    0.0%    0.0%    0.0%    0.0%    0.0%    0.0%    0.0%    0.0%    0.0%    0.0%    0.0%    0.0%    0.0%    0.0%    0.0%    0.0%    0.0%    0.0%    0.0%    0.0%    0.0%    0.0%    0.0%    0.0%    0.0%    0.0%    0.0%    0.0%    0.0%    0.0%    0.0%    0.0%    0.0%    0.0%    0.0%    0.0%    0.0%    0.0%    0.0%    0.0%    0.0%    0.0%    0.0%    0.0%    0.0%    0.0%    0.0%    0.0%    0.0%    0.0%    0.0%    0.0%    0.0%    0.0%    0.0%    0.0%    0.0%    0.0%    0.0%    0.0%    0.0%    0.0%    0.0%    0.0%    0.0%    0.0%    0.0%    0.0%    0.0%    0.0%    0.0%    0.0%    0.0%    0.0%    0.0%    0.0%    0.0%    0.0%    0.0%    0.0%    0.0%    0.0%    0.0%    0.0%    0.0%    0.0%    0.0%    0.0%    0.0%    0.0%    0.0%    0.0%    0.0%    0.0%    0.0%    0.0%    0.0%    0.0%    0.0%    0.0%    0.0%    0.0%    0.0%    0.0%    0.0%    0.0%    0.0%    0.0%    0.0%    0.0%    0.0%    0.0%    0.0%    0.0%    0.0%    0.0%    0.0%    0.0%    0.0%    0.0%    0.0%    0.0%    0.0%    0.0%    0.0%    0.0%    0.0%    0.0%    0.0%    0.0%    0.0%    0.0%    0.0%    0.0%    0.0%    0.0%    0.0%    0.0%    0.0%    0.0%    0.0%    0.0%    0.0%    0.0%    0.0%    0.0%    0.0%    0.0%    0.0%    0.0%    0.0%    0.0%    0.0%    0.0%    0.0%    0.0%    0.0%    0.0%    0.0%    0.0%    0.0%    0.0%    0.0%    0.0%    0.0%    0.0%    0.0%    0.0%    0.0%    0.0%    0.0%    0.0%    0.0%    0.0%    0.0%    0.0%    0.0%    0.0%    0.0%    0.0%    0.0%        k__Bacteria;p__Proteobacteria;c__Gammaproteobacteria; o__Oceanospirillales   1305591    7.7%    1.9%    1.1%    0.5%    2.8%   26.0%    0.8%    0.1%    0.2%    0.2%    0.5%    0.7%   31.9%    8.3%    1.0%    2.6%    1.3%   21.5%    2.3%    3.5%    5.5%    1.5%    1.2%    0.1%    1.1%    0.5%   10.1%    5.9%    0.6%    0.9%    0.4%    1.1%    0.5%    4.6%    1.7%    0.3%    1.2%    1.1%   21.8%   12.0%    5.0%    3.3%    0.4%    0.4%    0.6%    0.7%    6.1%    4.0%    8.2%    0.9%    9.5%    1.9%    3.1%   14.2%    6.2%    0.2%    3.0%   73.4%    6.5%   24.5%    2.4%    1.9%    4.5%    6.2%    2.7%    1.6%   40.0%    1.4%   29.3%    1.9%   27.9%    2.4%    2.3%    5.6%    1.9%    0.4%   16.8%    5.4%    0.3%    0.8%    0.2%   28.2%    8.1%    1.8%    1.1%    0.4%    2.9%   26.3%   16.1%    2.1%   47.6%   13.7%    6.1%    2.8%    0.1%    1.7%    7.1%   15.3%    1.8%    0.6%    4.3%    1.8%    0.4%    0.7%    0.1%    0.1%    1.0%   28.5%   48.2%    1.7%    0.7%    3.8%   18.0%    2.5%    8.5%    1.5%    0.9%    1.0%    1.0%    3.9%   79.8%    8.1%    4.5%   10.9%   63.4%    6.1%    2.6%   22.8%    2.9%    1.0%    4.4%   21.8%   17.4%    1.5%    1.8%    1.5%    2.4%   19.7%   52.0%    2.9%   55.8%   11.5%    9.2%   39.9%   11.1%    1.1%    0.2%    0.3%    6.8%   41.5%    4.6%   21.5%   16.3%    0.5%    0.8%   32.7%    9.8%   12.7%   33.5%    2.9%    0.8%    8.5%    0.7%   23.7%    0.8%   21.7%    3.4%    2.1%    4.2%    0.9%    3.2%    4.0%    7.8%    1.3%    0.7%    2.3%    3.0%    0.2%   22.7%    1.8%    0.7%    0.4%    0.7%    3.7%    0.2%    1.9%    3.4%    1.3%    1.0%    0.6%    0.4%    0.7%    0.5%    2.8%    6.9%    2.0%    0.8%    0.5%    2.2%    0.5%    0.9%    0.5%   10.0%    9.8%    1.9%    3.5%    2.1%    9.5%    3.3%    1.6%    1.4%    0.3%    6.7%    0.7%    4.7%    0.8%   14.0%    0.3%    0.6%    0.4%    0.3%   11.0%   54.4%   25.3%    6.6%    4.7%    1.9%    0.4%    2.3%    1.6%    1.4%    0.2%   25.7%    2.0%    8.5%   24.8%    9.3%    0.7%    0.7%    1.0%    2.3%    1.0%    0.1%    0.3%    0.7%    0.2%    0.1%    0.3%    0.3%    0.5%    1.3%   28.9%   49.4%    1.4%   19.7%    1.0%   14.7%    2.3%    3.4%    0.3%    1.0%    0.8%    7.3%    2.4%    2.0%   27.5%    6.3%    0.7%    4.8%    0.4%    2.5%    4.3%    4.4%    3.0%    0.6%    8.9%    1.6%    1.3%    1.2%    0.7%   14.6%    2.4%    0.8%    0.9%    1.0%    0.1%    0.8%    3.5%    1.8%    6.7%    4.1%    0.3%    1.2%    1.3%    0.5%    2.5%    1.8%    0.5%    4.5%    0.6%    0.4%    1.0%    0.3%    2.2%    0.6%   13.9%    1.6%    1.1%   17.5%    0.2%    4.6%    2.6%    0.4%    2.5%    0.7%    2.1%    1.0%    0.9%    0.6%    1.1%    1.0%    0.9%    0.2%    2.0%   10.7%    1.5%    1.2%   16.2%    1.4%    2.9%    0.8%    0.1%    0.6%    0.4%    0.1%    0.3%    0.0%    0.4%    0.4%   56.6%    6.1%   48.5%    3.1%    1.6%    9.9%    0.3%    5.2%    0.3%   53.5%    1.2%   10.9%    0.4%    0.6%    2.5%    0.4%   86.8%    8.6%    0.5%        k__Bacteria;p__Proteobacteria;c__Gammaproteobacteria; o__Pasteurellales   539675    3.2%   13.6%    5.9%    5.1%    1.9%    0.3%    2.2%    0.9%    7.8%   11.2%    7.2%    0.2%    1.0%    1.2%    4.9%    2.1%    3.9%    5.5%    0.7%    0.8%    3.4%    8.2%    1.6%    0.1%    2.5%    2.0%    0.6%    1.7%   10.8%    0.9%    0.3%    4.4%    1.0%    1.8%    5.2%    2.8%    5.5%    2.6%    4.1%    2.3%    3.7%    7.6%    9.6%   10.2%    4.5%    6.1%    2.2%    0.5%    4.0%    2.5%    3.6%    0.6%    0.8%    1.5%    1.9%    1.3%    1.8%    0.4%    5.7%    3.2%    1.6%    2.1%    0.9%    2.3%    0.7%    1.3%    0.2%   10.2%    0.6%    4.0%    3.4%    1.5%    0.8%    2.0%    2.2%    0.5%    1.2%    4.8%    1.6%    4.6%    3.7%    0.5%    4.7%    7.1%    1.3%    3.3%    5.6%    0.4%    4.5%    2.6%    0.2%    4.1%    5.5%   13.1%    1.3%    1.1%    4.1%    0.6%    2.3%    4.1%    1.3%    1.6%    0.7%    2.2%    2.2%    8.8%    7.6%    0.2%    0.1%    2.2%    2.7%    2.8%    2.4%    0.5%    4.5%    5.5%    5.5%    3.0%   17.5%    3.0%    0.2%    3.9%    2.6%    4.9%    0.4%    2.9%    4.0%    0.4%    8.9%    7.0%    2.8%    1.1%    0.4%    4.4%    6.6%    2.0%    1.4%    2.1%    0.4%    3.4%    0.1%    6.6%    3.2%    0.5%    0.8%    1.0%    7.3%    3.1%    3.0%    0.5%    1.4%    0.7%    3.8%    1.6%    4.8%    2.8%    3.9%    1.8%    1.4%    3.0%    1.8%    3.6%   24.4%    1.9%    2.9%    1.6%    4.4%    2.6%    3.4%    5.8%    2.9%    4.5%    0.8%    9.2%    1.1%    5.7%    2.6%    1.8%    0.3%    1.7%    2.9%    5.7%   10.7%    0.4%    1.3%    2.3%    0.5%    4.3%    6.4%    5.0%    1.5%    2.7%    4.6%    1.7%    1.6%    1.5%    2.8%    4.4%    0.7%    3.0%    8.9%    0.7%    1.1%    2.1%    3.9%    3.5%    0.7%    1.4%    2.5%    2.8%    3.5%    2.9%    2.0%    5.3%    2.8%    2.3%    1.7%    2.2%    2.5%    0.8%    0.8%    2.3%    0.1%    2.2%    1.0%    2.4%    3.9%    9.9%    3.5%    4.8%    3.3%    0.6%    0.3%    2.9%    3.0%    0.7%    1.9%    0.8%    0.4%    0.4%    1.7%    2.6%    3.9%    2.1%   13.0%    0.5%    6.4%    0.3%    9.9%    1.7%    1.7%    3.9%    0.5%    2.5%    0.3%    1.4%    2.8%    1.1%    2.9%    7.7%    2.0%   11.0%    4.4%    0.8%    3.3%    0.2%    2.1%    6.4%    3.5%    3.4%    2.8%    3.3%    6.2%    5.4%    2.6%    2.3%    2.8%    2.8%    4.6%    0.3%    1.0%    0.2%    2.8%    1.7%    1.7%    5.9%   17.9%    1.7%    1.5%    2.9%    0.7%    0.7%    4.8%   14.1%    5.5%    0.7%    4.6%    3.2%    2.1%    2.8%    2.3%    2.5%    5.9%    2.5%    3.7%    3.9%    3.3%    1.1%    0.8%   13.2%    0.1%    1.4%    0.6%    8.8%    3.0%    2.3%    0.1%    2.6%    4.9%    3.0%    6.2%    7.5%    0.9%    1.1%    3.6%    3.7%    3.8%    1.8%    5.0%    4.6%    3.7%    3.1%    2.2%    1.3%    0.5%    1.4%   12.5%    3.4%    3.9%    0.2%    4.3%    1.3%    3.1%    1.5%    3.1%    2.1%    0.5%    3.7%    0.3%    0.7%    2.1%    6.6%    1.9%    2.0%    0.9%    0.1%    2.2%    0.1%        k__Bacteria;p__Proteobacteria;c__Gammaproteobacteria; o__Pseudomonadales   483074    2.8%    0.4%    0.8%    2.3%   21.3%    5.1%    2.0%    2.3%    1.2%    1.8%    1.1%    8.6%    6.3%    4.8%    5.4%   15.0%    4.6%    4.9%    2.8%    5.5%   11.0%    7.8%    7.7%    1.3%    7.9%    3.1%    3.0%    5.9%    5.9%    2.2%    2.5%    3.7%    1.0%    0.7%   10.3%    2.0%    8.8%    0.6%    5.1%    4.0%    6.3%    5.6%    3.3%    3.9%    3.5%    4.2%   10.9%    2.6%    5.8%    2.6%    2.9%   16.6%   19.4%    0.1%    0.7%    0.4%    2.0%    0.4%    5.3%    0.7%    0.5%    0.6%    0.2%    1.5%    0.8%    1.0%    0.2%    2.7%    0.3%    1.5%    1.9%    0.4%    0.4%    0.6%    1.3%    0.1%    0.2%    0.8%    0.1%    1.1%    0.4%    8.4%    2.6%    0.8%    0.5%    0.1%    1.5%    0.5%    0.3%    2.5%    0.1%    0.8%    1.1%    1.3%    0.5%    1.3%    1.5%    2.6%    1.4%    1.4%    4.7%    1.5%    0.3%    1.3%    0.1%    0.2%    6.0%    0.1%    0.1%    1.1%    1.6%    3.0%    0.6%    0.2%    0.9%    0.5%    0.5%    0.8%    1.4%    3.0%    0.1%    0.9%    4.3%    1.0%    0.5%    1.4%    1.3%    2.2%    3.4%    7.2%    1.9%    1.3%    0.7%    0.9%    6.6%    6.9%   27.7%    6.2%    0.2%    1.1%    0.3%    6.0%    2.3%    1.7%    0.9%    0.5%    0.1%    0.3%    1.5%    0.3%    0.9%    0.6%    3.0%    0.9%    0.4%    0.2%    5.6%    1.0%    0.3%    3.8%    0.9%    3.3%    2.8%    0.3%    1.5%    0.6%    1.5%    1.9%    5.0%    0.9%   10.9%    2.5%    1.6%    3.9%    1.1%    0.7%    0.7%    0.8%    0.3%   20.6%    1.2%    1.2%    3.2%    0.2%    0.3%    1.4%    0.8%    0.4%    1.2%    0.3%    0.4%    2.1%    0.5%    1.3%    1.7%    1.2%    1.6%    0.7%    1.0%    0.6%    5.7%    1.1%    0.6%    2.6%    2.4%    1.8%   60.8%    3.0%    3.2%    2.5%    2.0%    1.3%    0.1%    1.6%    1.0%    4.4%    1.8%    0.3%    1.8%    1.2%    0.8%    1.6%    0.5%    2.7%    0.7%    2.4%    0.3%    0.3%    1.5%    1.5%    0.4%    0.2%    2.1%    0.6%    1.4%    0.2%    1.3%    1.1%    1.2%    2.9%    7.6%    2.4%    2.8%    7.9%    5.6%    2.9%    1.3%    4.4%   10.5%    2.3%    2.6%    0.8%    0.5%    0.5%    0.2%    0.5%    0.5%    0.3%    2.2%    0.4%    8.0%    2.6%    2.8%   29.1%    1.2%    0.6%    8.4%    2.8%    2.4%    0.5%    1.0%    1.3%    5.5%    2.1%    0.9%    5.0%    2.3%    2.5%    5.8%    0.6%    0.5%    1.4%    2.2%    4.7%    4.5%    0.5%    1.8%    2.7%    2.3%    2.3%    2.7%    0.3%    2.7%    3.8%    0.7%    0.3%    1.4%    1.1%    0.6%    2.3%    0.3%    0.2%    0.3%    0.7%    0.7%    0.2%    0.6%    0.3%    0.5%    0.3%    1.0%    1.1%    0.3%    1.8%    2.2%    3.4%    0.4%    0.5%    0.2%    2.1%    1.0%    1.5%    0.3%    0.8%    1.1%    2.7%    0.9%    0.9%    1.9%    1.4%    0.3%    0.0%    0.5%    0.4%    0.1%    0.0%    0.1%    0.4%    0.4%    0.2%    1.1%    0.1%    0.2%    0.5%    2.5%    0.1%    0.5%    0.2%    0.1%    0.1%    3.2%    0.2%    0.4%    1.0%    0.5%    0.1%    2.7%    0.2%        k__Bacteria;p__Proteobacteria;c__Gammaproteobacteria; o__Salinisphaerales      73    0.0%    0.0%    0.0%    0.0%    0.0%    0.0%    0.0%    0.0%    0.0%    0.0%    0.0%    0.0%    0.0%    0.0%    0.0%    0.0%    0.0%    0.0%    0.0%    0.0%    0.0%    0.0%    0.0%    0.0%    0.0%    0.0%    0.0%    0.0%    0.0%    0.0%    0.0%    0.0%    0.0%    0.0%    0.0%    0.0%    0.0%    0.0%    0.0%    0.0%    0.0%    0.0%    0.0%    0.0%    0.0%    0.0%    0.0%    0.0%    0.0%    0.0%    0.0%    0.0%    0.0%    0.0%    0.0%    0.0%    0.0%    0.0%    0.0%    0.0%    0.0%    0.0%    0.0%    0.0%    0.0%    0.0%    0.0%    0.0%    0.0%    0.0%    0.0%    0.0%    0.0%    0.0%    0.0%    0.0%    0.0%    0.0%    0.0%    0.0%    0.0%    0.0%    0.0%    0.0%    0.0%    0.0%    0.0%    0.0%    0.0%    0.0%    0.0%    0.0%    0.0%    0.0%    0.0%    0.0%    0.0%    0.0%    0.0%    0.0%    0.0%    0.0%    0.0%    0.0%    0.0%    0.0%    0.0%    0.0%    0.0%    0.0%    0.0%    0.0%    0.0%    0.0%    0.0%    0.0%    0.0%    0.0%    0.0%    0.0%    0.0%    0.0%    0.0%    0.0%    0.0%    0.0%    0.0%    0.0%    0.0%    0.0%    0.0%    0.0%    0.0%    0.0%    0.0%    0.0%    0.0%    0.0%    0.0%    0.0%    0.0%    0.0%    0.0%    0.0%    0.0%    0.0%    0.0%    0.0%    0.0%    0.0%    0.0%    0.0%    0.0%    0.0%    0.0%    0.0%    0.0%    0.0%    0.0%    0.0%    0.0%    0.0%    0.0%    0.0%    0.0%    0.0%    0.0%    0.0%    0.0%    0.0%    0.0%    0.0%    0.0%    0.0%    0.0%    0.0%    0.0%    0.0%    0.0%    0.0%    0.0%    0.0%    0.0%    0.0%    0.0%    0.0%    0.0%    0.0%    0.0%    0.0%    0.0%    0.0%    0.0%    0.0%    0.0%    0.0%    0.0%    0.0%    0.0%    0.0%    0.0%    0.0%    0.0%    0.0%    0.0%    0.0%    0.0%    0.0%    0.0%    0.0%    0.0%    0.0%    0.1%    0.0%    0.0%    0.0%    0.0%    0.0%    0.0%    0.0%    0.0%    0.0%    0.0%    0.0%    0.0%    0.0%    0.0%    0.0%    0.0%    0.0%    0.0%    0.0%    0.0%    0.0%    0.0%    0.0%    0.0%    0.0%    0.0%    0.0%    0.0%    0.0%    0.0%    0.0%    0.0%    0.0%    0.0%    0.0%    0.0%    0.0%    0.0%    0.0%    0.0%    0.0%    0.0%    0.0%    0.0%    0.0%    0.0%    0.0%    0.0%    0.0%    0.0%    0.0%    0.0%    0.0%    0.0%    0.0%    0.0%    0.0%    0.0%    0.0%    0.0%    0.0%    0.0%    0.0%    0.0%    0.0%    0.0%    0.0%    0.0%    0.0%    0.0%    0.0%    0.0%    0.0%    0.0%    0.0%    0.0%    0.0%    0.0%    0.0%    0.0%    0.0%    0.0%    0.0%    0.0%    0.0%    0.0%    0.0%    0.0%    0.0%    0.0%    0.0%    0.0%    0.0%    0.0%    0.0%    0.0%    0.0%    0.0%    0.0%    0.0%    0.0%    0.0%    0.0%    0.0%    0.0%    0.0%    0.0%    0.0%    0.0%    0.0%    0.0%    0.0%    0.0%    0.0%    0.0%    0.0%    0.0%    0.0%    0.0%    0.0%    0.0%    0.0%    0.0%    0.0%    0.0%    0.0%    0.0%    0.0%    0.0%    0.0%    0.0%    0.0%    0.0%    0.0%    0.0%    0.0%    0.0%    0.0%    0.0%    0.0%    0.0%    0.0%    0.0%    0.0%    0.0%        k__Bacteria;p__Proteobacteria;c__Gammaproteobacteria; o__Thiotrichales     239    0.0%    0.0%    0.0%    0.0%    0.0%    0.0%    0.0%    0.0%    0.0%    0.0%    0.0%    0.0%    0.0%    0.0%    0.0%    0.0%    0.0%    0.0%    0.0%    0.0%    0.0%    0.0%    0.0%    0.0%    0.0%    0.0%    0.0%    0.0%    0.0%    0.0%    0.0%    0.0%    0.0%    0.0%    0.0%    0.0%    0.0%    0.0%    0.0%    0.0%    0.0%    0.0%    0.0%    0.0%    0.0%    0.0%    0.0%    0.0%    0.0%    0.0%    0.0%    0.0%    0.0%    0.0%    0.0%    0.0%    0.0%    0.0%    0.0%    0.0%    0.0%    0.0%    0.0%    0.0%    0.0%    0.0%    0.0%    0.0%    0.0%    0.0%    0.0%    0.0%    0.0%    0.0%    0.0%    0.0%    0.0%    0.0%    0.0%    0.0%    0.0%    0.0%    0.0%    0.0%    0.0%    0.0%    0.0%    0.0%    0.0%    0.0%    0.0%    0.0%    0.0%    0.0%    0.0%    0.0%    0.0%    0.0%    0.0%    0.0%    0.0%    0.0%    0.0%    0.0%    0.0%    0.0%    0.0%    0.0%    0.0%    0.0%    0.0%    0.0%    0.0%    0.0%    0.0%    0.0%    0.0%    0.0%    0.0%    0.0%    0.0%    0.0%    0.0%    0.0%    0.0%    0.1%    0.0%    0.0%    0.0%    0.0%    0.0%    0.0%    0.0%    0.0%    0.0%    0.0%    0.0%    0.0%    0.0%    0.0%    0.0%    0.0%    0.0%    0.0%    0.0%    0.0%    0.0%    0.0%    0.0%    0.0%    0.0%    0.0%    0.0%    0.0%    0.0%    0.0%    0.0%    0.0%    0.0%    0.0%    0.0%    0.0%    0.0%    0.0%    0.0%    0.0%    0.0%    0.0%    0.0%    0.0%    0.0%    0.0%    0.0%    0.0%    0.0%    0.0%    0.0%    0.0%    0.0%    0.0%    0.0%    0.0%    0.0%    0.0%    0.0%    0.0%    0.0%    0.0%    0.0%    0.0%    0.0%    0.0%    0.0%    0.0%    0.0%    0.1%    0.0%    0.0%    0.0%    0.0%    0.0%    0.0%    0.0%    0.0%    0.0%    0.0%    0.0%    0.0%    0.0%    0.0%    0.0%    0.0%    0.0%    0.1%    0.0%    0.0%    0.0%    0.0%    0.0%    0.0%    0.0%    0.0%    0.0%    0.0%    0.0%    0.0%    0.0%    0.0%    0.0%    0.0%    0.0%    0.0%    0.0%    0.0%    0.0%    0.0%    0.0%    0.0%    0.0%    0.0%    0.0%    0.0%    0.0%    0.0%    0.0%    0.0%    0.0%    0.0%    0.0%    0.0%    0.0%    0.0%    0.0%    0.0%    0.0%    0.0%    0.0%    0.0%    0.0%    0.0%    0.0%    0.0%    0.0%    0.0%    0.0%    0.0%    0.0%    0.0%    0.0%    0.0%    0.0%    0.0%    0.0%    0.0%    0.0%    0.0%    0.0%    0.0%    0.0%    0.0%    0.0%    0.0%    0.0%    0.0%    0.0%    0.0%    0.0%    0.0%    0.0%    0.0%    0.0%    0.0%    0.0%    0.0%    0.0%    0.0%    0.0%    0.0%    0.0%    0.0%    0.0%    0.0%    0.0%    0.0%    0.0%    0.0%    0.0%    0.0%    0.0%    0.0%    0.0%    0.0%    0.0%    0.0%    0.0%    0.0%    0.0%    0.0%    0.0%    0.0%    0.0%    0.0%    0.0%    0.0%    0.0%    0.0%    0.0%    0.0%    0.0%    0.0%    0.0%    0.0%    0.0%    0.0%    0.0%    0.0%    0.0%    0.0%    0.0%    0.0%    0.0%    0.0%    0.0%    0.0%    0.0%    0.0%    0.0%    0.0%    0.0%    0.0%    0.0%    0.0%    0.0%    0.0%    0.0%    0.0%    0.0%    0.0%        k__Bacteria;p__Proteobacteria;c__Gammaproteobacteria; o__Vibrionales   26290    0.2%    0.0%    0.0%    0.0%    0.1%    3.8%    0.0%    0.0%    0.0%    0.0%    0.0%    0.0%    0.0%    0.0%    0.0%    0.0%    0.0%    0.0%    0.0%    0.0%    0.0%    0.0%    0.1%    0.0%    0.0%    0.0%    0.0%    0.0%    0.0%    0.0%    0.0%    0.0%    0.0%    0.0%    0.0%    0.0%    0.0%    0.0%    0.0%    0.0%    0.0%    0.4%    0.0%    0.0%    0.0%    0.0%    0.9%    0.0%    0.0%    0.0%    0.0%    0.1%    0.0%    0.0%    0.0%    0.0%    0.0%    0.0%    0.0%    0.0%    0.0%    0.0%    0.0%    0.0%    0.0%    0.0%    0.0%    0.0%    0.0%    0.0%    0.0%    0.0%    0.0%    0.0%    0.0%    0.0%    0.0%    0.0%    0.0%    0.0%    0.0%    0.0%    0.1%    0.0%    0.0%    0.0%    0.0%    0.0%    0.0%    0.0%    0.0%    0.0%    0.0%    0.0%    0.0%    0.0%    0.0%    0.0%    0.0%    0.0%    2.1%    0.1%    0.0%    0.1%    0.0%    0.0%    0.0%    0.0%    1.4%    0.2%    0.0%    0.0%    3.7%    0.0%    0.4%    0.1%    0.1%    0.0%    0.1%    0.0%    0.0%    0.0%    0.0%    0.0%    0.0%    0.0%    0.0%    0.0%    0.0%    0.0%    0.0%    0.0%    0.0%    0.0%    0.9%    0.1%    0.1%    0.1%    0.0%    1.7%    0.0%    3.6%    0.0%    0.0%    0.0%    0.0%    0.0%    0.0%    0.0%    0.0%    0.3%    0.0%    0.0%    0.0%    0.4%    0.0%    0.0%    0.0%    0.0%    0.0%    0.0%    0.0%    0.0%    3.9%    0.0%    0.1%    0.0%    0.2%    0.0%    0.1%    0.0%    0.0%    2.5%    0.1%    0.0%    0.0%    0.0%    0.0%    0.0%    0.0%    0.0%    0.0%    0.0%    1.2%    0.0%    0.0%    0.0%    0.0%    0.0%    0.0%    0.0%    0.0%    0.1%    0.2%    0.0%    0.0%    0.0%    0.0%    0.1%    0.0%    0.1%    0.0%    0.6%    0.1%    0.0%    0.0%    0.0%    2.5%    0.5%    1.1%    0.6%    0.1%    0.1%    0.1%    0.3%    0.1%    0.1%    0.0%    0.1%    0.1%    0.1%    0.1%    0.0%    0.1%    0.0%    0.3%    0.0%    0.0%    0.1%    0.1%    0.1%    0.0%    0.1%    1.3%    0.1%    0.0%    0.0%    0.1%    0.0%    0.1%    0.0%    0.0%    0.0%    0.0%    0.0%    0.0%    0.0%    0.0%    0.0%    0.0%    0.0%    0.0%    0.0%    0.0%    0.0%    0.0%    0.0%    0.0%    0.0%    0.0%    0.0%    0.0%    0.0%    0.2%    0.0%    0.0%    0.0%    0.0%    4.3%    0.0%    0.0%    0.0%    0.2%    0.1%    0.1%    0.3%    0.0%    0.1%    0.0%    0.0%    0.0%    0.0%    2.9%    0.6%    0.0%    0.0%    0.0%    0.0%    0.1%    0.0%    0.0%    0.0%    0.0%    0.0%    0.0%    0.3%    0.0%    0.0%    0.0%    0.0%    0.0%    1.3%    0.0%    0.0%    0.0%    0.0%    0.0%    0.0%    0.0%    0.0%    0.0%    0.0%    0.0%    0.0%    0.0%    0.0%    0.0%    0.0%    0.0%    0.0%    0.0%    0.0%    0.0%    0.0%    0.0%    0.0%    0.0%    0.0%    0.0%    0.0%    0.0%    0.0%    0.0%    0.0%    0.0%    0.0%    0.0%    0.0%    0.0%    0.0%    0.0%    0.0%    0.0%    0.0%    0.0%    0.0%    0.0%    0.0%    0.0%    0.0%    0.0%    0.0%    0.0%    0.0%    0.0%    0.0%    0.0%    0.0%        k__Bacteria;p__Proteobacteria;c__Gammaproteobacteria; o__Xanthomonadales   54959    0.3%    0.1%    0.1%    0.1%    0.5%    0.2%    0.7%    0.2%    0.1%    0.1%    0.3%    0.2%    0.5%    1.0%    0.9%    0.3%    0.3%    0.2%    0.0%    0.1%    0.4%    0.6%    0.2%    0.0%    0.1%    0.1%    0.2%    0.1%    0.3%    0.0%    0.1%    0.2%    0.0%    0.0%    0.5%    0.1%    0.3%    0.0%    0.1%    0.2%    0.1%    0.3%    0.0%    0.0%    0.2%    0.1%    0.2%    0.1%    0.1%    0.1%    0.2%    1.1%    1.3%    0.1%    0.2%    0.1%    0.2%    0.0%    0.5%    0.3%    0.2%    0.2%    0.1%    0.4%    0.2%    0.1%    0.1%    0.4%    0.1%    0.2%    0.3%    0.1%    0.1%    0.1%    0.3%    0.0%    0.1%    0.2%    0.0%    0.1%    0.0%    1.0%    0.1%    0.0%    0.1%    0.1%    0.2%    0.1%    0.1%    0.3%    0.1%    0.1%    0.2%    0.1%    0.0%    0.1%    0.5%    0.6%    1.4%    0.1%    0.5%    0.1%    0.0%    0.1%    0.0%    0.0%    0.8%    0.1%    0.1%    0.2%    0.1%    0.4%    0.3%    0.0%    0.1%    0.1%    0.0%    0.1%    0.1%    0.9%    0.0%    0.1%    0.3%    0.1%    0.0%    0.6%    0.2%    0.1%    0.3%    0.9%    0.2%    0.1%    0.2%    0.1%    0.5%    0.4%    0.9%    1.3%    0.0%    0.4%    0.0%    0.4%    0.2%    0.2%    0.4%    0.2%    0.0%    0.0%    0.4%    0.1%    0.3%    0.1%    0.4%    0.1%    0.1%    0.6%    0.4%    0.7%    0.1%    0.3%    0.4%    0.6%    9.5%    0.2%    0.3%    0.3%    0.4%    0.2%    0.3%    0.0%    0.5%    0.6%    0.2%    0.3%    0.1%    0.1%    1.2%    5.9%    0.1%    0.5%    0.1%    0.0%    0.9%    0.1%    0.1%    0.1%    0.1%    0.1%    0.1%    0.1%    0.1%    0.1%    0.1%    0.1%    0.2%    0.2%    0.3%    0.1%    0.1%    0.0%    0.8%    0.2%    0.2%    0.5%    1.1%    0.2%    2.1%    0.6%    0.7%    0.5%    0.4%    0.1%    0.3%    0.1%    0.1%    0.4%    0.1%    0.1%    0.1%    0.3%    0.3%    0.4%    0.2%    0.4%    0.2%    0.3%    0.0%    0.2%    0.3%    0.2%    0.1%    0.1%    1.1%    0.1%    0.5%    0.2%    0.5%    0.2%    0.5%    0.4%    1.9%    0.9%    0.1%    0.2%    0.1%    0.1%    0.0%    0.3%    0.0%    0.1%    0.8%    0.4%    0.1%    0.1%    0.1%    0.7%    0.3%    0.1%    0.4%    1.5%    0.5%    0.3%    0.5%    0.3%    0.5%    0.1%    0.3%    0.5%    1.0%    0.1%    0.3%    0.9%    0.9%    0.7%    0.1%    0.2%    0.3%    0.5%    0.4%    0.1%    0.0%    0.1%    0.1%    0.0%    0.1%    0.4%    0.6%    0.7%    0.5%    0.2%    0.2%    0.0%    0.3%    0.8%    0.1%    0.0%    0.3%    0.2%    0.1%    0.2%    0.1%    0.2%    0.0%    0.4%    0.1%    0.1%    0.1%    0.1%    0.3%    0.4%    0.2%    0.7%    0.4%    0.5%    0.1%    0.2%    0.0%    0.1%    0.0%    0.3%    0.2%    0.6%    0.1%    0.3%    0.5%    0.2%    0.4%    0.1%    0.4%    0.4%    0.1%    0.0%    0.0%    0.1%    0.0%    0.0%    0.0%    0.3%    0.8%    0.0%    0.2%    0.0%    0.4%    0.5%    0.2%    0.2%    0.0%    0.3%    0.2%    0.1%    0.3%    0.6%    0.2%    0.3%    0.1%    0.0%    0.0%    0.0%        k__Bacteria;p__SC3;c__; o__       6    0.0%    0.0%    0.0%    0.0%    0.0%    0.0%    0.0%    0.0%    0.0%    0.0%    0.0%    0.0%    0.0%    0.0%    0.0%    0.0%    0.0%    0.0%    0.0%    0.0%    0.0%    0.0%    0.0%    0.0%    0.0%    0.0%    0.0%    0.0%    0.0%    0.0%    0.0%    0.0%    0.0%    0.0%    0.0%    0.0%    0.0%    0.0%    0.0%    0.0%    0.0%    0.0%    0.0%    0.0%    0.0%    0.0%    0.0%    0.0%    0.0%    0.0%    0.0%    0.0%    0.0%    0.0%    0.0%    0.0%    0.0%    0.0%    0.0%    0.0%    0.0%    0.0%    0.0%    0.0%    0.0%    0.0%    0.0%    0.0%    0.0%    0.0%    0.0%    0.0%    0.0%    0.0%    0.0%    0.0%    0.0%    0.0%    0.0%    0.0%    0.0%    0.0%    0.0%    0.0%    0.0%    0.0%    0.0%    0.0%    0.0%    0.0%    0.0%    0.0%    0.0%    0.0%    0.0%    0.0%    0.0%    0.0%    0.0%    0.0%    0.0%    0.0%    0.0%    0.0%    0.0%    0.0%    0.0%    0.0%    0.0%    0.0%    0.0%    0.0%    0.0%    0.0%    0.0%    0.0%    0.0%    0.0%    0.0%    0.0%    0.0%    0.0%    0.0%    0.0%    0.0%    0.0%    0.0%    0.0%    0.0%    0.0%    0.0%    0.0%    0.0%    0.0%    0.0%    0.0%    0.0%    0.0%    0.0%    0.0%    0.0%    0.0%    0.0%    0.0%    0.0%    0.0%    0.0%    0.0%    0.0%    0.0%    0.0%    0.0%    0.0%    0.0%    0.0%    0.0%    0.0%    0.0%    0.0%    0.0%    0.0%    0.0%    0.0%    0.0%    0.0%    0.0%    0.0%    0.0%    0.0%    0.0%    0.0%    0.0%    0.0%    0.0%    0.0%    0.0%    0.0%    0.0%    0.0%    0.0%    0.0%    0.0%    0.0%    0.0%    0.0%    0.0%    0.0%    0.0%    0.0%    0.0%    0.0%    0.0%    0.0%    0.0%    0.0%    0.0%    0.0%    0.0%    0.0%    0.0%    0.0%    0.0%    0.0%    0.0%    0.0%    0.0%    0.0%    0.0%    0.0%    0.0%    0.0%    0.0%    0.0%    0.0%    0.0%    0.0%    0.0%    0.0%    0.0%    0.0%    0.0%    0.0%    0.0%    0.0%    0.0%    0.0%    0.0%    0.0%    0.0%    0.0%    0.0%    0.0%    0.0%    0.0%    0.0%    0.0%    0.0%    0.0%    0.0%    0.0%    0.0%    0.0%    0.0%    0.0%    0.0%    0.0%    0.0%    0.0%    0.0%    0.0%    0.0%    0.0%    0.0%    0.0%    0.0%    0.0%    0.0%    0.0%    0.0%    0.0%    0.0%    0.0%    0.0%    0.0%    0.0%    0.0%    0.0%    0.0%    0.0%    0.0%    0.0%    0.0%    0.0%    0.0%    0.0%    0.0%    0.0%    0.0%    0.0%    0.0%    0.0%    0.0%    0.0%    0.0%    0.0%    0.0%    0.0%    0.0%    0.0%    0.0%    0.0%    0.0%    0.0%    0.0%    0.0%    0.0%    0.0%    0.0%    0.0%    0.0%    0.0%    0.0%    0.0%    0.0%    0.0%    0.0%    0.0%    0.0%    0.0%    0.0%    0.0%    0.0%    0.0%    0.0%    0.0%    0.0%    0.0%    0.0%    0.0%    0.0%    0.0%    0.0%    0.0%    0.0%    0.0%    0.0%    0.0%    0.0%    0.0%    0.0%    0.0%    0.0%    0.0%    0.0%    0.0%    0.0%    0.0%    0.0%    0.0%    0.0%    0.0%    0.0%    0.0%    0.0%    0.0%    0.0%    0.0%    0.0%    0.0%    0.0%    0.0%    0.0%    0.0%    0.0%    0.0%    0.0%    0.0%    0.0%        k__Bacteria;p__SC4;c__; o__    1057    0.0%    0.0%    0.0%    0.0%    0.0%    0.0%    0.0%    0.0%    0.0%    0.0%    0.0%    0.0%    0.0%    0.0%    0.0%    0.0%    0.0%    0.0%    0.0%    0.0%    0.0%    0.0%    0.0%    0.0%    0.0%    0.0%    0.0%    0.0%    0.0%    0.0%    0.0%    0.0%    0.0%    0.0%    0.0%    0.0%    0.0%    0.0%    0.0%    0.0%    0.0%    0.0%    0.0%    0.0%    0.0%    0.0%    0.0%    0.0%    0.0%    0.0%    0.0%    0.0%    0.0%    0.0%    0.0%    0.0%    0.0%    0.0%    0.0%    0.0%    0.0%    0.0%    0.0%    0.1%    0.0%    0.0%    0.0%    0.0%    0.0%    0.0%    0.0%    0.0%    0.0%    0.0%    0.0%    0.0%    0.0%    0.0%    0.0%    0.0%    0.0%    0.0%    0.0%    0.0%    0.0%    0.0%    0.0%    0.0%    0.0%    0.0%    0.0%    0.0%    0.0%    0.0%    0.0%    0.0%    0.0%    0.0%    0.0%    0.0%    0.0%    0.0%    0.0%    0.0%    0.0%    0.0%    0.0%    0.0%    0.0%    0.0%    0.0%    0.0%    0.0%    0.0%    0.0%    0.0%    0.0%    0.0%    0.0%    0.0%    0.0%    0.0%    0.0%    0.0%    0.0%    0.0%    0.0%    0.0%    0.0%    0.0%    0.0%    0.0%    0.0%    0.0%    0.0%    0.0%    0.0%    0.0%    0.0%    0.0%    0.0%    0.0%    0.0%    0.0%    0.0%    0.0%    0.0%    0.0%    0.0%    0.0%    0.0%    0.0%    0.0%    0.0%    0.0%    0.0%    0.0%    0.0%    0.0%    0.0%    0.0%    0.0%    0.0%    0.0%    0.0%    0.0%    0.0%    0.0%    0.0%    0.0%    0.0%    0.0%    0.0%    0.0%    0.0%    0.0%    0.0%    0.0%    0.0%    0.0%    0.0%    0.0%    0.0%    0.0%    0.0%    0.0%    0.0%    0.0%    0.0%    0.0%    0.0%    0.0%    0.0%    0.0%    0.0%    0.0%    0.0%    0.0%    0.0%    0.0%    0.0%    0.0%    0.0%    0.0%    0.0%    0.0%    0.0%    0.0%    0.0%    0.0%    0.0%    0.0%    0.0%    0.0%    0.0%    0.0%    0.0%    0.0%    0.0%    0.0%    0.0%    0.0%    0.0%    0.0%    0.0%    0.0%    0.0%    0.0%    0.0%    0.0%    0.0%    0.0%    0.0%    0.0%    0.0%    0.0%    0.0%    0.0%    0.0%    0.0%    0.0%    0.0%    0.0%    0.0%    0.0%    0.0%    0.0%    0.0%    0.0%    0.0%    0.0%    0.0%    0.0%    0.0%    0.0%    0.0%    0.0%    0.0%    0.0%    0.0%    0.0%    0.0%    0.0%    0.0%    0.0%    0.0%    0.0%    0.0%    0.0%    0.0%    0.0%    0.0%    0.1%    0.0%    0.0%    0.0%    0.0%    0.0%    0.0%    0.0%    0.0%    0.0%    0.0%    0.0%    0.0%    0.0%    0.0%    0.1%    0.0%    0.0%    0.0%    0.0%    0.0%    0.0%    0.0%    0.0%    0.0%    0.0%    0.0%    0.1%    0.0%    0.0%    0.0%    0.1%    0.0%    0.0%    0.0%    0.0%    0.0%    0.0%    0.0%    0.0%    0.0%    0.0%    0.0%    0.0%    0.0%    0.0%    0.0%    0.0%    0.0%    0.0%    0.0%    0.0%    0.0%    0.0%    0.0%    0.0%    0.0%    0.0%    0.0%    0.0%    0.0%    0.0%    0.0%    0.0%    0.0%    0.0%    0.0%    0.0%    0.0%    0.0%    0.0%    0.0%    0.0%    0.0%    0.0%    0.0%    0.0%    0.0%    0.0%    0.0%    0.0%    0.0%    0.0%    0.0%    0.0%    0.0%        k__Bacteria;p__SPAM;c__; o__     334    0.0%    0.0%    0.0%    0.0%    0.0%    0.0%    0.0%    0.0%    0.0%    0.0%    0.0%    0.0%    0.0%    0.0%    0.0%    0.0%    0.0%    0.0%    0.0%    0.0%    0.0%    0.0%    0.0%    0.0%    0.0%    0.0%    0.0%    0.0%    0.0%    0.0%    0.0%    0.0%    0.0%    0.0%    0.0%    0.0%    0.0%    0.0%    0.0%    0.0%    0.0%    0.0%    0.0%    0.0%    0.0%    0.0%    0.0%    0.0%    0.0%    0.0%    0.0%    0.0%    0.0%    0.0%    0.0%    0.0%    0.0%    0.0%    0.0%    0.0%    0.0%    0.0%    0.0%    0.0%    0.0%    0.0%    0.0%    0.0%    0.0%    0.0%    0.0%    0.0%    0.0%    0.0%    0.0%    0.0%    0.0%    0.0%    0.0%    0.0%    0.0%    0.0%    0.0%    0.0%    0.0%    0.0%    0.0%    0.0%    0.0%    0.0%    0.0%    0.0%    0.0%    0.0%    0.0%    0.0%    0.0%    0.0%    0.0%    0.0%    0.0%    0.0%    0.0%    0.0%    0.0%    0.0%    0.0%    0.0%    0.0%    0.0%    0.0%    0.0%    0.0%    0.0%    0.0%    0.0%    0.0%    0.0%    0.0%    0.0%    0.0%    0.0%    0.0%    0.0%    0.0%    0.0%    0.0%    0.0%    0.0%    0.0%    0.0%    0.0%    0.0%    0.0%    0.0%    0.0%    0.0%    0.0%    0.0%    0.0%    0.0%    0.0%    0.0%    0.0%    0.0%    0.0%    0.0%    0.0%    0.0%    0.0%    0.0%    0.0%    0.0%    0.0%    0.0%    0.0%    0.0%    0.0%    0.0%    0.0%    0.0%    0.0%    0.0%    0.0%    0.0%    0.0%    0.0%    0.0%    0.0%    0.0%    0.0%    0.0%    0.0%    0.0%    0.0%    0.0%    0.0%    0.0%    0.0%    0.0%    0.0%    0.0%    0.0%    0.0%    0.0%    0.0%    0.0%    0.0%    0.0%    0.0%    0.0%    0.0%    0.0%    0.0%    0.0%    0.0%    0.0%    0.0%    0.0%    0.0%    0.0%    0.0%    0.0%    0.0%    0.0%    0.0%    0.0%    0.0%    0.0%    0.0%    0.0%    0.0%    0.0%    0.0%    0.0%    0.0%    0.0%    0.0%    0.0%    0.0%    0.0%    0.0%    0.0%    0.0%    0.0%    0.0%    0.0%    0.0%    0.0%    0.0%    0.0%    0.0%    0.0%    0.0%    0.0%    0.0%    0.0%    0.0%    0.0%    0.0%    0.0%    0.0%    0.0%    0.0%    0.0%    0.0%    0.0%    0.0%    0.0%    0.0%    0.0%    0.0%    0.0%    0.0%    0.0%    0.0%    0.0%    0.0%    0.0%    0.0%    0.0%    0.0%    0.0%    0.0%    0.0%    0.0%    0.0%    0.0%    0.0%    0.0%    0.0%    0.0%    0.0%    0.0%    0.0%    0.0%    0.0%    0.0%    0.0%    0.0%    0.0%    0.0%    0.0%    0.0%    0.0%    0.0%    0.0%    0.0%    0.0%    0.0%    0.0%    0.0%    0.0%    0.0%    0.0%    0.0%    0.0%    0.0%    0.0%    0.0%    0.0%    0.0%    0.0%    0.0%    0.0%    0.0%    0.0%    0.0%    0.0%    0.0%    0.0%    0.0%    0.0%    0.0%    0.0%    0.0%    0.0%    0.0%    0.0%    0.0%    0.0%    0.0%    0.0%    0.0%    0.0%    0.0%    0.0%    0.0%    0.0%    0.0%    0.0%    0.0%    0.0%    0.0%    0.0%    0.0%    0.0%    0.0%    0.0%    0.0%    0.0%    0.0%    0.0%    0.0%    0.0%    0.0%    0.0%    0.0%    0.0%    0.0%    0.0%    0.0%    0.0%    0.0%    0.0%    0.0%    0.0%    0.0%        k__Bacteria;p__SR1;c__; o__    1149    0.0%    0.0%    0.0%    0.0%    0.0%    0.0%    0.0%    0.0%    0.0%    0.0%    0.0%    0.0%    0.0%    0.0%    0.0%    0.0%    0.0%    0.0%    0.0%    0.0%    0.0%    0.0%    0.0%    0.0%    0.0%    0.0%    0.0%    0.0%    0.0%    0.0%    0.0%    0.0%    0.0%    0.0%    0.0%    0.0%    0.0%    0.0%    0.0%    0.0%    0.0%    0.0%    0.0%    0.0%    0.0%    0.0%    0.0%    0.0%    0.0%    0.0%    0.0%    0.0%    0.0%    0.0%    0.0%    0.0%    0.0%    0.0%    0.0%    0.0%    0.0%    0.0%    0.0%    0.0%    0.0%    0.0%    0.0%    0.0%    0.0%    0.0%    0.0%    0.0%    0.0%    0.0%    0.0%    0.0%    0.0%    0.0%    0.0%    0.0%    0.0%    0.0%    0.0%    0.0%    0.0%    0.0%    0.0%    0.0%    0.0%    0.0%    0.0%    0.0%    0.0%    0.0%    0.0%    0.0%    0.0%    0.0%    0.0%    0.0%    0.0%    0.0%    0.0%    0.0%    0.0%    0.0%    0.1%    0.0%    0.0%    0.0%    0.0%    0.0%    0.0%    0.0%    0.0%    0.0%    0.0%    0.0%    0.0%    0.0%    0.0%    0.0%    0.0%    0.0%    0.0%    0.0%    0.0%    0.0%    0.0%    0.0%    0.0%    0.0%    0.0%    0.0%    0.0%    0.0%    0.0%    0.0%    0.0%    0.0%    0.0%    0.0%    0.0%    0.0%    0.0%    0.0%    0.0%    0.0%    0.0%    0.0%    0.0%    0.0%    0.0%    0.0%    0.0%    0.0%    0.0%    0.0%    0.0%    0.0%    0.0%    0.0%    0.1%    0.0%    0.0%    0.0%    0.0%    0.0%    0.0%    0.0%    0.0%    0.0%    0.0%    0.0%    0.0%    0.0%    0.0%    0.0%    0.0%    0.0%    0.0%    0.0%    0.0%    0.0%    0.0%    0.0%    0.0%    0.0%    0.0%    0.0%    0.0%    0.0%    0.0%    0.0%    0.0%    0.0%    0.0%    0.0%    0.0%    0.0%    0.0%    0.0%    0.0%    0.0%    0.0%    0.0%    0.0%    0.0%    0.0%    0.0%    0.0%    0.0%    0.0%    0.0%    0.0%    0.0%    0.0%    0.0%    0.0%    0.0%    0.0%    0.0%    0.0%    0.0%    0.0%    0.0%    0.0%    0.0%    0.0%    0.0%    0.0%    0.0%    0.0%    0.0%    0.0%    0.0%    0.0%    0.0%    0.0%    0.0%    0.0%    0.0%    0.0%    0.0%    0.0%    0.0%    0.0%    0.0%    0.0%    0.0%    0.0%    0.0%    0.0%    0.0%    0.0%    0.0%    0.0%    0.0%    0.0%    0.0%    0.0%    0.0%    0.0%    0.0%    0.0%    0.0%    0.0%    0.0%    0.0%    0.0%    0.0%    0.0%    0.0%    0.0%    0.0%    0.0%    0.0%    0.0%    0.0%    0.0%    0.0%    0.0%    0.0%    0.0%    0.0%    0.0%    0.0%    0.0%    0.0%    0.0%    0.0%    0.0%    0.0%    0.0%    0.0%    0.0%    0.0%    0.1%    0.0%    0.0%    0.0%    0.0%    0.0%    0.0%    0.0%    0.0%    0.0%    0.0%    0.0%    0.0%    0.0%    0.0%    0.0%    0.0%    0.0%    0.0%    0.0%    0.0%    0.0%    0.0%    0.0%    0.0%    0.0%    0.0%    0.0%    0.0%    0.0%    0.0%    0.0%    0.0%    0.0%    0.0%    0.0%    0.0%    0.0%    0.0%    0.0%    0.0%    0.0%    0.0%    0.0%    0.0%    0.0%    0.0%    0.0%    0.0%    0.0%    0.0%    0.0%    0.0%    0.0%    0.0%    0.0%    0.0%    0.0%    0.0%    0.0%    0.0%        k__Bacteria;p__Spirochaetes;c__Brachyspirae; o__Brachyspirales       3    0.0%    0.0%    0.0%    0.0%    0.0%    0.0%    0.0%    0.0%    0.0%    0.0%    0.0%    0.0%    0.0%    0.0%    0.0%    0.0%    0.0%    0.0%    0.0%    0.0%    0.0%    0.0%    0.0%    0.0%    0.0%    0.0%    0.0%    0.0%    0.0%    0.0%    0.0%    0.0%    0.0%    0.0%    0.0%    0.0%    0.0%    0.0%    0.0%    0.0%    0.0%    0.0%    0.0%    0.0%    0.0%    0.0%    0.0%    0.0%    0.0%    0.0%    0.0%    0.0%    0.0%    0.0%    0.0%    0.0%    0.0%    0.0%    0.0%    0.0%    0.0%    0.0%    0.0%    0.0%    0.0%    0.0%    0.0%    0.0%    0.0%    0.0%    0.0%    0.0%    0.0%    0.0%    0.0%    0.0%    0.0%    0.0%    0.0%    0.0%    0.0%    0.0%    0.0%    0.0%    0.0%    0.0%    0.0%    0.0%    0.0%    0.0%    0.0%    0.0%    0.0%    0.0%    0.0%    0.0%    0.0%    0.0%    0.0%    0.0%    0.0%    0.0%    0.0%    0.0%    0.0%    0.0%    0.0%    0.0%    0.0%    0.0%    0.0%    0.0%    0.0%    0.0%    0.0%    0.0%    0.0%    0.0%    0.0%    0.0%    0.0%    0.0%    0.0%    0.0%    0.0%    0.0%    0.0%    0.0%    0.0%    0.0%    0.0%    0.0%    0.0%    0.0%    0.0%    0.0%    0.0%    0.0%    0.0%    0.0%    0.0%    0.0%    0.0%    0.0%    0.0%    0.0%    0.0%    0.0%    0.0%    0.0%    0.0%    0.0%    0.0%    0.0%    0.0%    0.0%    0.0%    0.0%    0.0%    0.0%    0.0%    0.0%    0.0%    0.0%    0.0%    0.0%    0.0%    0.0%    0.0%    0.0%    0.0%    0.0%    0.0%    0.0%    0.0%    0.0%    0.0%    0.0%    0.0%    0.0%    0.0%    0.0%    0.0%    0.0%    0.0%    0.0%    0.0%    0.0%    0.0%    0.0%    0.0%    0.0%    0.0%    0.0%    0.0%    0.0%    0.0%    0.0%    0.0%    0.0%    0.0%    0.0%    0.0%    0.0%    0.0%    0.0%    0.0%    0.0%    0.0%    0.0%    0.0%    0.0%    0.0%    0.0%    0.0%    0.0%    0.0%    0.0%    0.0%    0.0%    0.0%    0.0%    0.0%    0.0%    0.0%    0.0%    0.0%    0.0%    0.0%    0.0%    0.0%    0.0%    0.0%    0.0%    0.0%    0.0%    0.0%    0.0%    0.0%    0.0%    0.0%    0.0%    0.0%    0.0%    0.0%    0.0%    0.0%    0.0%    0.0%    0.0%    0.0%    0.0%    0.0%    0.0%    0.0%    0.0%    0.0%    0.0%    0.0%    0.0%    0.0%    0.0%    0.0%    0.0%    0.0%    0.0%    0.0%    0.0%    0.0%    0.0%    0.0%    0.0%    0.0%    0.0%    0.0%    0.0%    0.0%    0.0%    0.0%    0.0%    0.0%    0.0%    0.0%    0.0%    0.0%    0.0%    0.0%    0.0%    0.0%    0.0%    0.0%    0.0%    0.0%    0.0%    0.0%    0.0%    0.0%    0.0%    0.0%    0.0%    0.0%    0.0%    0.0%    0.0%    0.0%    0.0%    0.0%    0.0%    0.0%    0.0%    0.0%    0.0%    0.0%    0.0%    0.0%    0.0%    0.0%    0.0%    0.0%    0.0%    0.0%    0.0%    0.0%    0.0%    0.0%    0.0%    0.0%    0.0%    0.0%    0.0%    0.0%    0.0%    0.0%    0.0%    0.0%    0.0%    0.0%    0.0%    0.0%    0.0%    0.0%    0.0%    0.0%    0.0%    0.0%    0.0%    0.0%    0.0%    0.0%    0.0%    0.0%    0.0%    0.0%    0.0%    0.0%    0.0%    0.0%    0.0%        k__Bacteria;p__Spirochaetes;c__Leptospirae; o__Leptospirales       1    0.0%    0.0%    0.0%    0.0%    0.0%    0.0%    0.0%    0.0%    0.0%    0.0%    0.0%    0.0%    0.0%    0.0%    0.0%    0.0%    0.0%    0.0%    0.0%    0.0%    0.0%    0.0%    0.0%    0.0%    0.0%    0.0%    0.0%    0.0%    0.0%    0.0%    0.0%    0.0%    0.0%    0.0%    0.0%    0.0%    0.0%    0.0%    0.0%    0.0%    0.0%    0.0%    0.0%    0.0%    0.0%    0.0%    0.0%    0.0%    0.0%    0.0%    0.0%    0.0%    0.0%    0.0%    0.0%    0.0%    0.0%    0.0%    0.0%    0.0%    0.0%    0.0%    0.0%    0.0%    0.0%    0.0%    0.0%    0.0%    0.0%    0.0%    0.0%    0.0%    0.0%    0.0%    0.0%    0.0%    0.0%    0.0%    0.0%    0.0%    0.0%    0.0%    0.0%    0.0%    0.0%    0.0%    0.0%    0.0%    0.0%    0.0%    0.0%    0.0%    0.0%    0.0%    0.0%    0.0%    0.0%    0.0%    0.0%    0.0%    0.0%    0.0%    0.0%    0.0%    0.0%    0.0%    0.0%    0.0%    0.0%    0.0%    0.0%    0.0%    0.0%    0.0%    0.0%    0.0%    0.0%    0.0%    0.0%    0.0%    0.0%    0.0%    0.0%    0.0%    0.0%    0.0%    0.0%    0.0%    0.0%    0.0%    0.0%    0.0%    0.0%    0.0%    0.0%    0.0%    0.0%    0.0%    0.0%    0.0%    0.0%    0.0%    0.0%    0.0%    0.0%    0.0%    0.0%    0.0%    0.0%    0.0%    0.0%    0.0%    0.0%    0.0%    0.0%    0.0%    0.0%    0.0%    0.0%    0.0%    0.0%    0.0%    0.0%    0.0%    0.0%    0.0%    0.0%    0.0%    0.0%    0.0%    0.0%    0.0%    0.0%    0.0%    0.0%    0.0%    0.0%    0.0%    0.0%    0.0%    0.0%    0.0%    0.0%    0.0%    0.0%    0.0%    0.0%    0.0%    0.0%    0.0%    0.0%    0.0%    0.0%    0.0%    0.0%    0.0%    0.0%    0.0%    0.0%    0.0%    0.0%    0.0%    0.0%    0.0%    0.0%    0.0%    0.0%    0.0%    0.0%    0.0%    0.0%    0.0%    0.0%    0.0%    0.0%    0.0%    0.0%    0.0%    0.0%    0.0%    0.0%    0.0%    0.0%    0.0%    0.0%    0.0%    0.0%    0.0%    0.0%    0.0%    0.0%    0.0%    0.0%    0.0%    0.0%    0.0%    0.0%    0.0%    0.0%    0.0%    0.0%    0.0%    0.0%    0.0%    0.0%    0.0%    0.0%    0.0%    0.0%    0.0%    0.0%    0.0%    0.0%    0.0%    0.0%    0.0%    0.0%    0.0%    0.0%    0.0%    0.0%    0.0%    0.0%    0.0%    0.0%    0.0%    0.0%    0.0%    0.0%    0.0%    0.0%    0.0%    0.0%    0.0%    0.0%    0.0%    0.0%    0.0%    0.0%    0.0%    0.0%    0.0%    0.0%    0.0%    0.0%    0.0%    0.0%    0.0%    0.0%    0.0%    0.0%    0.0%    0.0%    0.0%    0.0%    0.0%    0.0%    0.0%    0.0%    0.0%    0.0%    0.0%    0.0%    0.0%    0.0%    0.0%    0.0%    0.0%    0.0%    0.0%    0.0%    0.0%    0.0%    0.0%    0.0%    0.0%    0.0%    0.0%    0.0%    0.0%    0.0%    0.0%    0.0%    0.0%    0.0%    0.0%    0.0%    0.0%    0.0%    0.0%    0.0%    0.0%    0.0%    0.0%    0.0%    0.0%    0.0%    0.0%    0.0%    0.0%    0.0%    0.0%    0.0%    0.0%    0.0%    0.0%    0.0%    0.0%    0.0%    0.0%    0.0%    0.0%    0.0%    0.0%    0.0%    0.0%    0.0%    0.0%        k__Bacteria;p__Spirochaetes;c__SP_WWE1; o__       4    0.0%    0.0%    0.0%    0.0%    0.0%    0.0%    0.0%    0.0%    0.0%    0.0%    0.0%    0.0%    0.0%    0.0%    0.0%    0.0%    0.0%    0.0%    0.0%    0.0%    0.0%    0.0%    0.0%    0.0%    0.0%    0.0%    0.0%    0.0%    0.0%    0.0%    0.0%    0.0%    0.0%    0.0%    0.0%    0.0%    0.0%    0.0%    0.0%    0.0%    0.0%    0.0%    0.0%    0.0%    0.0%    0.0%    0.0%    0.0%    0.0%    0.0%    0.0%    0.0%    0.0%    0.0%    0.0%    0.0%    0.0%    0.0%    0.0%    0.0%    0.0%    0.0%    0.0%    0.0%    0.0%    0.0%    0.0%    0.0%    0.0%    0.0%    0.0%    0.0%    0.0%    0.0%    0.0%    0.0%    0.0%    0.0%    0.0%    0.0%    0.0%    0.0%    0.0%    0.0%    0.0%    0.0%    0.0%    0.0%    0.0%    0.0%    0.0%    0.0%    0.0%    0.0%    0.0%    0.0%    0.0%    0.0%    0.0%    0.0%    0.0%    0.0%    0.0%    0.0%    0.0%    0.0%    0.0%    0.0%    0.0%    0.0%    0.0%    0.0%    0.0%    0.0%    0.0%    0.0%    0.0%    0.0%    0.0%    0.0%    0.0%    0.0%    0.0%    0.0%    0.0%    0.0%    0.0%    0.0%    0.0%    0.0%    0.0%    0.0%    0.0%    0.0%    0.0%    0.0%    0.0%    0.0%    0.0%    0.0%    0.0%    0.0%    0.0%    0.0%    0.0%    0.0%    0.0%    0.0%    0.0%    0.0%    0.0%    0.0%    0.0%    0.0%    0.0%    0.0%    0.0%    0.0%    0.0%    0.0%    0.0%    0.0%    0.0%    0.0%    0.0%    0.0%    0.0%    0.0%    0.0%    0.0%    0.0%    0.0%    0.0%    0.0%    0.0%    0.0%    0.0%    0.0%    0.0%    0.0%    0.0%    0.0%    0.0%    0.0%    0.0%    0.0%    0.0%    0.0%    0.0%    0.0%    0.0%    0.0%    0.0%    0.0%    0.0%    0.0%    0.0%    0.0%    0.0%    0.0%    0.0%    0.0%    0.0%    0.0%    0.0%    0.0%    0.0%    0.0%    0.0%    0.0%    0.0%    0.0%    0.0%    0.0%    0.0%    0.0%    0.0%    0.0%    0.0%    0.0%    0.0%    0.0%    0.0%    0.0%    0.0%    0.0%    0.0%    0.0%    0.0%    0.0%    0.0%    0.0%    0.0%    0.0%    0.0%    0.0%    0.0%    0.0%    0.0%    0.0%    0.0%    0.0%    0.0%    0.0%    0.0%    0.0%    0.0%    0.0%    0.0%    0.0%    0.0%    0.0%    0.0%    0.0%    0.0%    0.0%    0.0%    0.0%    0.0%    0.0%    0.0%    0.0%    0.0%    0.0%    0.0%    0.0%    0.0%    0.0%    0.0%    0.0%    0.0%    0.0%    0.0%    0.0%    0.0%    0.0%    0.0%    0.0%    0.0%    0.0%    0.0%    0.0%    0.0%    0.0%    0.0%    0.0%    0.0%    0.0%    0.0%    0.0%    0.0%    0.0%    0.0%    0.0%    0.0%    0.0%    0.0%    0.0%    0.0%    0.0%    0.0%    0.0%    0.0%    0.0%    0.0%    0.0%    0.0%    0.0%    0.0%    0.0%    0.0%    0.0%    0.0%    0.0%    0.0%    0.0%    0.0%    0.0%    0.0%    0.0%    0.0%    0.0%    0.0%    0.0%    0.0%    0.0%    0.0%    0.0%    0.0%    0.0%    0.0%    0.0%    0.0%    0.0%    0.0%    0.0%    0.0%    0.0%    0.0%    0.0%    0.0%    0.0%    0.0%    0.0%    0.0%    0.0%    0.0%    0.0%    0.0%    0.0%    0.0%    0.0%    0.0%    0.0%    0.0%    0.0%    0.0%    0.0%        k__Bacteria;p__Spirochaetes;c__Spirochaetes (class); o__Spirochaetales    5984    0.0%    0.1%    0.0%    0.1%    0.1%    0.0%    0.0%    0.0%    0.0%    0.0%    0.0%    0.0%    0.0%    0.1%    0.1%    0.0%    0.1%    0.0%    0.1%    0.0%    0.0%    0.1%    0.0%    0.0%    0.0%    0.0%    0.0%    0.0%    0.1%    0.0%    0.0%    0.1%    0.0%    0.4%    0.2%    0.6%    0.1%    0.0%    0.0%    0.0%    0.0%    0.2%    0.0%    0.1%    0.0%    0.0%    0.0%    0.0%    0.0%    0.0%    0.0%    0.0%    0.0%    0.0%    0.1%    0.0%    0.1%    0.0%    0.0%    0.0%    0.0%    0.0%    0.1%    0.0%    0.0%    0.0%    0.0%    0.1%    0.0%    0.0%    0.9%    0.1%    0.0%    0.0%    0.0%    0.0%    0.0%    0.0%    0.0%    0.0%    0.0%    0.0%    0.0%    0.0%    0.0%    0.0%    0.2%    0.0%    0.0%    0.1%    0.0%    0.0%    0.1%    0.0%    0.0%    0.0%    0.0%    0.0%    0.0%    0.1%    0.0%    0.1%    0.0%    0.0%    0.0%    0.0%    0.1%    0.0%    0.0%    0.0%    0.0%    0.1%    0.1%    0.0%    0.0%    0.0%    0.0%    0.0%    0.0%    0.6%    0.0%    0.0%    0.0%    0.0%    0.0%    0.0%    0.0%    0.0%    0.0%    0.0%    0.0%    0.0%    0.0%    0.0%    0.0%    0.0%    0.0%    0.0%    0.0%    0.0%    0.0%    0.2%    0.0%    0.0%    0.0%    0.0%    0.3%    0.1%    0.1%    0.0%    0.0%    0.0%    0.0%    0.0%    0.0%    0.0%    0.0%    0.0%    0.0%    0.1%    0.0%    0.0%    0.0%    0.0%    0.0%    0.0%    0.0%    0.0%    0.0%    0.0%    0.0%    0.1%    0.0%    0.0%    0.0%    0.0%    0.0%    0.0%    0.0%    0.0%    0.0%    0.0%    0.1%    0.0%    0.0%    0.0%    0.0%    0.0%    0.0%    0.0%    0.0%    0.0%    0.0%    0.0%    0.0%    0.0%    0.0%    0.0%    0.0%    0.0%    0.1%    0.0%    0.0%    0.0%    0.0%    0.0%    0.0%    0.2%    0.0%    0.0%    0.0%    0.0%    0.0%    0.1%    0.0%    0.0%    0.0%    0.0%    0.0%    0.1%    0.0%    0.0%    0.0%    0.0%    0.0%    0.0%    0.0%    0.1%    0.0%    0.0%    0.0%    0.0%    0.0%    0.0%    0.0%    0.0%    0.0%    0.0%    0.0%    0.0%    0.0%    0.0%    0.0%    0.0%    0.0%    0.0%    0.0%    0.0%    0.0%    0.0%    0.0%    0.0%    0.0%    0.0%    0.0%    0.0%    0.0%    0.0%    0.0%    0.0%    0.0%    0.0%    0.0%    0.0%    0.0%    0.0%    0.0%    0.0%    0.0%    0.0%    0.0%    0.0%    0.0%    0.0%    0.0%    0.0%    0.0%    0.0%    0.0%    0.0%    0.0%    0.0%    0.0%    0.0%    0.0%    0.1%    0.0%    0.0%    0.0%    0.0%    0.0%    0.0%    0.1%    0.0%    0.0%    0.0%    0.0%    0.0%    0.0%    0.0%    0.0%    0.0%    0.0%    0.0%    0.0%    0.0%    0.0%    0.0%    0.0%    0.0%    0.0%    0.0%    0.0%    0.0%    0.0%    0.1%    0.0%    0.0%    0.0%    0.0%    0.0%    0.0%    0.0%    0.0%    0.0%    0.0%    0.0%    0.0%    0.0%    0.4%    0.0%    0.1%    0.0%    0.0%    0.0%    0.0%    0.0%    0.0%    0.0%    0.0%    0.0%    0.0%    0.0%    0.1%    0.0%    0.0%    0.0%    0.0%    0.0%    0.0%    0.0%    0.0%    0.0%    0.1%    0.0%    0.0%    0.0%    0.0%        k__Bacteria;p__Synergistetes;c__Synergistia; o__Synergistales    1745    0.0%    0.0%    0.0%    0.0%    0.0%    0.0%    0.0%    0.0%    0.0%    0.0%    0.0%    0.0%    0.0%    0.0%    0.0%    0.0%    0.0%    0.0%    0.0%    0.0%    0.0%    0.0%    0.0%    0.0%    0.0%    0.0%    0.0%    0.0%    0.0%    0.0%    0.0%    0.0%    0.0%    0.1%    0.0%    0.2%    0.0%    0.0%    0.0%    0.0%    0.0%    0.0%    0.0%    0.0%    0.0%    0.0%    0.0%    0.0%    0.0%    0.0%    0.0%    0.0%    0.0%    0.0%    0.0%    0.0%    0.0%    0.0%    0.0%    0.0%    0.0%    0.0%    0.0%    0.0%    0.0%    0.0%    0.0%    0.0%    0.0%    0.0%    0.1%    0.0%    0.0%    0.0%    0.0%    0.0%    0.0%    0.0%    0.0%    0.0%    0.0%    0.0%    0.0%    0.0%    0.0%    0.0%    0.0%    0.0%    0.0%    0.0%    0.0%    0.0%    0.0%    0.0%    0.0%    0.0%    0.0%    0.0%    0.0%    0.0%    0.0%    0.0%    0.0%    0.0%    0.0%    0.0%    0.0%    0.0%    0.0%    0.0%    0.0%    0.0%    0.0%    0.0%    0.0%    0.0%    0.0%    0.0%    0.0%    0.1%    0.0%    0.0%    0.0%    0.0%    0.0%    0.0%    0.0%    0.0%    0.0%    0.0%    0.0%    0.0%    0.0%    0.0%    0.0%    0.0%    0.0%    0.0%    0.0%    0.0%    0.0%    0.1%    0.0%    0.0%    0.0%    0.0%    0.3%    0.1%    0.0%    0.0%    0.0%    0.0%    0.0%    0.0%    0.0%    0.0%    0.0%    0.0%    0.0%    0.0%    0.0%    0.0%    0.0%    0.0%    0.0%    0.0%    0.0%    0.0%    0.0%    0.0%    0.0%    0.0%    0.0%    0.0%    0.0%    0.0%    0.0%    0.0%    0.0%    0.0%    0.0%    0.0%    0.0%    0.0%    0.0%    0.0%    0.0%    0.1%    0.0%    0.0%    0.0%    0.1%    0.0%    0.0%    0.0%    0.0%    0.0%    0.0%    0.0%    0.0%    0.0%    0.0%    0.0%    0.0%    0.0%    0.0%    0.0%    0.1%    0.0%    0.0%    0.0%    0.0%    0.0%    0.1%    0.0%    0.0%    0.0%    0.0%    0.0%    0.0%    0.0%    0.0%    0.0%    0.0%    0.0%    0.0%    0.0%    0.0%    0.0%    0.0%    0.0%    0.0%    0.0%    0.0%    0.0%    0.0%    0.0%    0.0%    0.0%    0.0%    0.0%    0.0%    0.0%    0.0%    0.0%    0.0%    0.0%    0.0%    0.0%    0.0%    0.0%    0.0%    0.0%    0.0%    0.0%    0.0%    0.0%    0.0%    0.0%    0.0%    0.0%    0.0%    0.0%    0.0%    0.1%    0.0%    0.0%    0.0%    0.0%    0.0%    0.0%    0.0%    0.0%    0.0%    0.0%    0.0%    0.0%    0.0%    0.0%    0.0%    0.0%    0.0%    0.0%    0.0%    0.0%    0.0%    0.0%    0.0%    0.0%    0.0%    0.0%    0.0%    0.0%    0.0%    0.0%    0.0%    0.0%    0.0%    0.1%    0.0%    0.0%    0.0%    0.0%    0.0%    0.0%    0.0%    0.0%    0.0%    0.0%    0.0%    0.0%    0.0%    0.0%    0.0%    0.0%    0.0%    0.0%    0.0%    0.0%    0.0%    0.0%    0.0%    0.0%    0.0%    0.0%    0.0%    0.0%    0.0%    0.0%    0.1%    0.0%    0.0%    0.0%    0.0%    0.0%    0.0%    0.0%    0.2%    0.0%    0.0%    0.0%    0.0%    0.0%    0.0%    0.0%    0.0%    0.0%    0.0%    0.0%    0.0%    0.0%    0.0%    0.0%    0.0%    0.0%    0.0%    0.0%    0.0%        k__Bacteria;p__TM6;c__; o__       4    0.0%    0.0%    0.0%    0.0%    0.0%    0.0%    0.0%    0.0%    0.0%    0.0%    0.0%    0.0%    0.0%    0.0%    0.0%    0.0%    0.0%    0.0%    0.0%    0.0%    0.0%    0.0%    0.0%    0.0%    0.0%    0.0%    0.0%    0.0%    0.0%    0.0%    0.0%    0.0%    0.0%    0.0%    0.0%    0.0%    0.0%    0.0%    0.0%    0.0%    0.0%    0.0%    0.0%    0.0%    0.0%    0.0%    0.0%    0.0%    0.0%    0.0%    0.0%    0.0%    0.0%    0.0%    0.0%    0.0%    0.0%    0.0%    0.0%    0.0%    0.0%    0.0%    0.0%    0.0%    0.0%    0.0%    0.0%    0.0%    0.0%    0.0%    0.0%    0.0%    0.0%    0.0%    0.0%    0.0%    0.0%    0.0%    0.0%    0.0%    0.0%    0.0%    0.0%    0.0%    0.0%    0.0%    0.0%    0.0%    0.0%    0.0%    0.0%    0.0%    0.0%    0.0%    0.0%    0.0%    0.0%    0.0%    0.0%    0.0%    0.0%    0.0%    0.0%    0.0%    0.0%    0.0%    0.0%    0.0%    0.0%    0.0%    0.0%    0.0%    0.0%    0.0%    0.0%    0.0%    0.0%    0.0%    0.0%    0.0%    0.0%    0.0%    0.0%    0.0%    0.0%    0.0%    0.0%    0.0%    0.0%    0.0%    0.0%    0.0%    0.0%    0.0%    0.0%    0.0%    0.0%    0.0%    0.0%    0.0%    0.0%    0.0%    0.0%    0.0%    0.0%    0.0%    0.0%    0.0%    0.0%    0.0%    0.0%    0.0%    0.0%    0.0%    0.0%    0.0%    0.0%    0.0%    0.0%    0.0%    0.0%    0.0%    0.0%    0.0%    0.0%    0.0%    0.0%    0.0%    0.0%    0.0%    0.0%    0.0%    0.0%    0.0%    0.0%    0.0%    0.0%    0.0%    0.0%    0.0%    0.0%    0.0%    0.0%    0.0%    0.0%    0.0%    0.0%    0.0%    0.0%    0.0%    0.0%    0.0%    0.0%    0.0%    0.0%    0.0%    0.0%    0.0%    0.0%    0.0%    0.0%    0.0%    0.0%    0.0%    0.0%    0.0%    0.0%    0.0%    0.0%    0.0%    0.0%    0.0%    0.0%    0.0%    0.0%    0.0%    0.0%    0.0%    0.0%    0.0%    0.0%    0.0%    0.0%    0.0%    0.0%    0.0%    0.0%    0.0%    0.0%    0.0%    0.0%    0.0%    0.0%    0.0%    0.0%    0.0%    0.0%    0.0%    0.0%    0.0%    0.0%    0.0%    0.0%    0.0%    0.0%    0.0%    0.0%    0.0%    0.0%    0.0%    0.0%    0.0%    0.0%    0.0%    0.0%    0.0%    0.0%    0.0%    0.0%    0.0%    0.0%    0.0%    0.0%    0.0%    0.0%    0.0%    0.0%    0.0%    0.0%    0.0%    0.0%    0.0%    0.0%    0.0%    0.0%    0.0%    0.0%    0.0%    0.0%    0.0%    0.0%    0.0%    0.0%    0.0%    0.0%    0.0%    0.0%    0.0%    0.0%    0.0%    0.0%    0.0%    0.0%    0.0%    0.0%    0.0%    0.0%    0.0%    0.0%    0.0%    0.0%    0.0%    0.0%    0.0%    0.0%    0.0%    0.0%    0.0%    0.0%    0.0%    0.0%    0.0%    0.0%    0.0%    0.0%    0.0%    0.0%    0.0%    0.0%    0.0%    0.0%    0.0%    0.0%    0.0%    0.0%    0.0%    0.0%    0.0%    0.0%    0.0%    0.0%    0.0%    0.0%    0.0%    0.0%    0.0%    0.0%    0.0%    0.0%    0.0%    0.0%    0.0%    0.0%    0.0%    0.0%    0.0%    0.0%    0.0%    0.0%    0.0%    0.0%    0.0%    0.0%    0.0%    0.0%    0.0%    0.0%    0.0%        k__Bacteria;p__TM7;c__; o__       3    0.0%    0.0%    0.0%    0.0%    0.0%    0.0%    0.0%    0.0%    0.0%    0.0%    0.0%    0.0%    0.0%    0.0%    0.0%    0.0%    0.0%    0.0%    0.0%    0.0%    0.0%    0.0%    0.0%    0.0%    0.0%    0.0%    0.0%    0.0%    0.0%    0.0%    0.0%    0.0%    0.0%    0.0%    0.0%    0.0%    0.0%    0.0%    0.0%    0.0%    0.0%    0.0%    0.0%    0.0%    0.0%    0.0%    0.0%    0.0%    0.0%    0.0%    0.0%    0.0%    0.0%    0.0%    0.0%    0.0%    0.0%    0.0%    0.0%    0.0%    0.0%    0.0%    0.0%    0.0%    0.0%    0.0%    0.0%    0.0%    0.0%    0.0%    0.0%    0.0%    0.0%    0.0%    0.0%    0.0%    0.0%    0.0%    0.0%    0.0%    0.0%    0.0%    0.0%    0.0%    0.0%    0.0%    0.0%    0.0%    0.0%    0.0%    0.0%    0.0%    0.0%    0.0%    0.0%    0.0%    0.0%    0.0%    0.0%    0.0%    0.0%    0.0%    0.0%    0.0%    0.0%    0.0%    0.0%    0.0%    0.0%    0.0%    0.0%    0.0%    0.0%    0.0%    0.0%    0.0%    0.0%    0.0%    0.0%    0.0%    0.0%    0.0%    0.0%    0.0%    0.0%    0.0%    0.0%    0.0%    0.0%    0.0%    0.0%    0.0%    0.0%    0.0%    0.0%    0.0%    0.0%    0.0%    0.0%    0.0%    0.0%    0.0%    0.0%    0.0%    0.0%    0.0%    0.0%    0.0%    0.0%    0.0%    0.0%    0.0%    0.0%    0.0%    0.0%    0.0%    0.0%    0.0%    0.0%    0.0%    0.0%    0.0%    0.0%    0.0%    0.0%    0.0%    0.0%    0.0%    0.0%    0.0%    0.0%    0.0%    0.0%    0.0%    0.0%    0.0%    0.0%    0.0%    0.0%    0.0%    0.0%    0.0%    0.0%    0.0%    0.0%    0.0%    0.0%    0.0%    0.0%    0.0%    0.0%    0.0%    0.0%    0.0%    0.0%    0.0%    0.0%    0.0%    0.0%    0.0%    0.0%    0.0%    0.0%    0.0%    0.0%    0.0%    0.0%    0.0%    0.0%    0.0%    0.0%    0.0%    0.0%    0.0%    0.0%    0.0%    0.0%    0.0%    0.0%    0.0%    0.0%    0.0%    0.0%    0.0%    0.0%    0.0%    0.0%    0.0%    0.0%    0.0%    0.0%    0.0%    0.0%    0.0%    0.0%    0.0%    0.0%    0.0%    0.0%    0.0%    0.0%    0.0%    0.0%    0.0%    0.0%    0.0%    0.0%    0.0%    0.0%    0.0%    0.0%    0.0%    0.0%    0.0%    0.0%    0.0%    0.0%    0.0%    0.0%    0.0%    0.0%    0.0%    0.0%    0.0%    0.0%    0.0%    0.0%    0.0%    0.0%    0.0%    0.0%    0.0%    0.0%    0.0%    0.0%    0.0%    0.0%    0.0%    0.0%    0.0%    0.0%    0.0%    0.0%    0.0%    0.0%    0.0%    0.0%    0.0%    0.0%    0.0%    0.0%    0.0%    0.0%    0.0%    0.0%    0.0%    0.0%    0.0%    0.0%    0.0%    0.0%    0.0%    0.0%    0.0%    0.0%    0.0%    0.0%    0.0%    0.0%    0.0%    0.0%    0.0%    0.0%    0.0%    0.0%    0.0%    0.0%    0.0%    0.0%    0.0%    0.0%    0.0%    0.0%    0.0%    0.0%    0.0%    0.0%    0.0%    0.0%    0.0%    0.0%    0.0%    0.0%    0.0%    0.0%    0.0%    0.0%    0.0%    0.0%    0.0%    0.0%    0.0%    0.0%    0.0%    0.0%    0.0%    0.0%    0.0%    0.0%    0.0%    0.0%    0.0%    0.0%    0.0%    0.0%    0.0%    0.0%    0.0%        k__Bacteria;p__TM7;c__TM7-1; o__       2    0.0%    0.0%    0.0%    0.0%    0.0%    0.0%    0.0%    0.0%    0.0%    0.0%    0.0%    0.0%    0.0%    0.0%    0.0%    0.0%    0.0%    0.0%    0.0%    0.0%    0.0%    0.0%    0.0%    0.0%    0.0%    0.0%    0.0%    0.0%    0.0%    0.0%    0.0%    0.0%    0.0%    0.0%    0.0%    0.0%    0.0%    0.0%    0.0%    0.0%    0.0%    0.0%    0.0%    0.0%    0.0%    0.0%    0.0%    0.0%    0.0%    0.0%    0.0%    0.0%    0.0%    0.0%    0.0%    0.0%    0.0%    0.0%    0.0%    0.0%    0.0%    0.0%    0.0%    0.0%    0.0%    0.0%    0.0%    0.0%    0.0%    0.0%    0.0%    0.0%    0.0%    0.0%    0.0%    0.0%    0.0%    0.0%    0.0%    0.0%    0.0%    0.0%    0.0%    0.0%    0.0%    0.0%    0.0%    0.0%    0.0%    0.0%    0.0%    0.0%    0.0%    0.0%    0.0%    0.0%    0.0%    0.0%    0.0%    0.0%    0.0%    0.0%    0.0%    0.0%    0.0%    0.0%    0.0%    0.0%    0.0%    0.0%    0.0%    0.0%    0.0%    0.0%    0.0%    0.0%    0.0%    0.0%    0.0%    0.0%    0.0%    0.0%    0.0%    0.0%    0.0%    0.0%    0.0%    0.0%    0.0%    0.0%    0.0%    0.0%    0.0%    0.0%    0.0%    0.0%    0.0%    0.0%    0.0%    0.0%    0.0%    0.0%    0.0%    0.0%    0.0%    0.0%    0.0%    0.0%    0.0%    0.0%    0.0%    0.0%    0.0%    0.0%    0.0%    0.0%    0.0%    0.0%    0.0%    0.0%    0.0%    0.0%    0.0%    0.0%    0.0%    0.0%    0.0%    0.0%    0.0%    0.0%    0.0%    0.0%    0.0%    0.0%    0.0%    0.0%    0.0%    0.0%    0.0%    0.0%    0.0%    0.0%    0.0%    0.0%    0.0%    0.0%    0.0%    0.0%    0.0%    0.0%    0.0%    0.0%    0.0%    0.0%    0.0%    0.0%    0.0%    0.0%    0.0%    0.0%    0.0%    0.0%    0.0%    0.0%    0.0%    0.0%    0.0%    0.0%    0.0%    0.0%    0.0%    0.0%    0.0%    0.0%    0.0%    0.0%    0.0%    0.0%    0.0%    0.0%    0.0%    0.0%    0.0%    0.0%    0.0%    0.0%    0.0%    0.0%    0.0%    0.0%    0.0%    0.0%    0.0%    0.0%    0.0%    0.0%    0.0%    0.0%    0.0%    0.0%    0.0%    0.0%    0.0%    0.0%    0.0%    0.0%    0.0%    0.0%    0.0%    0.0%    0.0%    0.0%    0.0%    0.0%    0.0%    0.0%    0.0%    0.0%    0.0%    0.0%    0.0%    0.0%    0.0%    0.0%    0.0%    0.0%    0.0%    0.0%    0.0%    0.0%    0.0%    0.0%    0.0%    0.0%    0.0%    0.0%    0.0%    0.0%    0.0%    0.0%    0.0%    0.0%    0.0%    0.0%    0.0%    0.0%    0.0%    0.0%    0.0%    0.0%    0.0%    0.0%    0.0%    0.0%    0.0%    0.0%    0.0%    0.0%    0.0%    0.0%    0.0%    0.0%    0.0%    0.0%    0.0%    0.0%    0.0%    0.0%    0.0%    0.0%    0.0%    0.0%    0.0%    0.0%    0.0%    0.0%    0.0%    0.0%    0.0%    0.0%    0.0%    0.0%    0.0%    0.0%    0.0%    0.0%    0.0%    0.0%    0.0%    0.0%    0.0%    0.0%    0.0%    0.0%    0.0%    0.0%    0.0%    0.0%    0.0%    0.0%    0.0%    0.0%    0.0%    0.0%    0.0%    0.0%    0.0%    0.0%    0.0%    0.0%    0.0%    0.0%    0.0%    0.0%    0.0%    0.0%    0.0%    0.0%        k__Bacteria;p__TM7;c__TM7-3; o__CW040      31    0.0%    0.0%    0.0%    0.0%    0.0%    0.0%    0.0%    0.0%    0.0%    0.0%    0.0%    0.0%    0.0%    0.0%    0.0%    0.0%    0.0%    0.0%    0.0%    0.0%    0.0%    0.0%    0.0%    0.0%    0.0%    0.0%    0.0%    0.0%    0.0%    0.0%    0.0%    0.0%    0.0%    0.0%    0.0%    0.0%    0.0%    0.0%    0.0%    0.0%    0.0%    0.0%    0.0%    0.0%    0.0%    0.0%    0.0%    0.0%    0.0%    0.0%    0.0%    0.0%    0.0%    0.0%    0.0%    0.0%    0.0%    0.0%    0.0%    0.0%    0.0%    0.0%    0.0%    0.0%    0.0%    0.0%    0.0%    0.0%    0.0%    0.0%    0.0%    0.0%    0.0%    0.0%    0.0%    0.0%    0.0%    0.0%    0.0%    0.0%    0.0%    0.0%    0.0%    0.0%    0.0%    0.0%    0.0%    0.0%    0.0%    0.0%    0.0%    0.0%    0.0%    0.0%    0.0%    0.0%    0.0%    0.0%    0.0%    0.0%    0.0%    0.0%    0.0%    0.0%    0.0%    0.0%    0.0%    0.0%    0.0%    0.0%    0.0%    0.0%    0.0%    0.0%    0.0%    0.0%    0.0%    0.0%    0.0%    0.0%    0.0%    0.0%    0.0%    0.0%    0.0%    0.0%    0.0%    0.0%    0.0%    0.0%    0.0%    0.0%    0.0%    0.0%    0.0%    0.0%    0.0%    0.0%    0.0%    0.0%    0.0%    0.0%    0.0%    0.0%    0.0%    0.0%    0.0%    0.0%    0.0%    0.0%    0.0%    0.0%    0.0%    0.0%    0.0%    0.0%    0.0%    0.0%    0.0%    0.0%    0.0%    0.0%    0.0%    0.0%    0.0%    0.0%    0.0%    0.0%    0.0%    0.0%    0.0%    0.0%    0.0%    0.0%    0.0%    0.0%    0.0%    0.0%    0.0%    0.0%    0.0%    0.0%    0.0%    0.0%    0.0%    0.0%    0.0%    0.0%    0.0%    0.0%    0.0%    0.0%    0.0%    0.0%    0.0%    0.0%    0.0%    0.0%    0.0%    0.0%    0.0%    0.0%    0.0%    0.0%    0.0%    0.0%    0.0%    0.0%    0.0%    0.0%    0.0%    0.0%    0.0%    0.0%    0.0%    0.0%    0.0%    0.0%    0.0%    0.0%    0.0%    0.0%    0.0%    0.0%    0.0%    0.0%    0.0%    0.0%    0.0%    0.0%    0.0%    0.0%    0.0%    0.0%    0.0%    0.0%    0.0%    0.0%    0.0%    0.0%    0.0%    0.0%    0.0%    0.0%    0.0%    0.0%    0.0%    0.0%    0.0%    0.0%    0.0%    0.0%    0.0%    0.0%    0.0%    0.0%    0.0%    0.0%    0.0%    0.0%    0.0%    0.0%    0.0%    0.0%    0.0%    0.0%    0.0%    0.0%    0.0%    0.0%    0.0%    0.0%    0.0%    0.0%    0.0%    0.0%    0.0%    0.0%    0.0%    0.0%    0.0%    0.0%    0.0%    0.0%    0.0%    0.0%    0.0%    0.0%    0.0%    0.0%    0.0%    0.0%    0.0%    0.0%    0.0%    0.0%    0.0%    0.0%    0.0%    0.0%    0.0%    0.0%    0.0%    0.0%    0.0%    0.0%    0.0%    0.0%    0.0%    0.0%    0.0%    0.0%    0.0%    0.0%    0.0%    0.0%    0.0%    0.0%    0.0%    0.0%    0.0%    0.0%    0.0%    0.0%    0.0%    0.0%    0.0%    0.0%    0.0%    0.0%    0.0%    0.0%    0.0%    0.0%    0.0%    0.0%    0.0%    0.0%    0.0%    0.0%    0.0%    0.0%    0.0%    0.0%    0.0%    0.0%    0.0%    0.0%    0.0%    0.0%    0.0%    0.0%    0.0%    0.0%    0.0%    0.0%    0.0%    0.0%        k__Bacteria;p__TM7;c__TM7-3; o__EW055     325    0.0%    0.0%    0.0%    0.0%    0.0%    0.0%    0.0%    0.0%    0.0%    0.0%    0.0%    0.0%    0.0%    0.0%    0.0%    0.0%    0.0%    0.0%    0.0%    0.0%    0.0%    0.0%    0.0%    0.0%    0.0%    0.0%    0.0%    0.0%    0.0%    0.0%    0.0%    0.0%    0.0%    0.0%    0.0%    0.0%    0.0%    0.0%    0.0%    0.0%    0.0%    0.0%    0.0%    0.0%    0.0%    0.0%    0.0%    0.0%    0.0%    0.0%    0.0%    0.0%    0.0%    0.0%    0.0%    0.0%    0.0%    0.0%    0.0%    0.0%    0.0%    0.0%    0.0%    0.0%    0.0%    0.0%    0.0%    0.0%    0.0%    0.0%    0.0%    0.0%    0.0%    0.0%    0.0%    0.0%    0.0%    0.0%    0.0%    0.0%    0.0%    0.0%    0.0%    0.0%    0.0%    0.0%    0.0%    0.0%    0.0%    0.0%    0.0%    0.0%    0.0%    0.0%    0.0%    0.0%    0.0%    0.0%    0.0%    0.0%    0.0%    0.0%    0.0%    0.0%    0.0%    0.0%    0.0%    0.0%    0.0%    0.0%    0.0%    0.0%    0.0%    0.0%    0.0%    0.0%    0.0%    0.0%    0.0%    0.0%    0.0%    0.0%    0.0%    0.0%    0.0%    0.0%    0.0%    0.0%    0.0%    0.0%    0.0%    0.0%    0.0%    0.0%    0.0%    0.0%    0.0%    0.0%    0.0%    0.0%    0.0%    0.0%    0.0%    0.0%    0.0%    0.0%    0.0%    0.0%    0.0%    0.0%    0.0%    0.0%    0.0%    0.0%    0.0%    0.0%    0.0%    0.0%    0.0%    0.0%    0.0%    0.0%    0.0%    0.0%    0.0%    0.0%    0.0%    0.0%    0.0%    0.0%    0.0%    0.0%    0.0%    0.0%    0.0%    0.0%    0.0%    0.0%    0.0%    0.0%    0.0%    0.0%    0.0%    0.0%    0.0%    0.0%    0.0%    0.0%    0.0%    0.0%    0.0%    0.0%    0.0%    0.0%    0.0%    0.0%    0.0%    0.0%    0.0%    0.0%    0.0%    0.0%    0.0%    0.0%    0.0%    0.0%    0.0%    0.0%    0.0%    0.0%    0.0%    0.0%    0.0%    0.0%    0.0%    0.0%    0.0%    0.0%    0.0%    0.0%    0.0%    0.0%    0.0%    0.0%    0.0%    0.0%    0.0%    0.0%    0.0%    0.0%    0.0%    0.0%    0.0%    0.0%    0.0%    0.0%    0.0%    0.0%    0.0%    0.0%    0.0%    0.0%    0.0%    0.0%    0.0%    0.0%    0.0%    0.0%    0.0%    0.0%    0.0%    0.0%    0.0%    0.0%    0.0%    0.0%    0.0%    0.0%    0.0%    0.0%    0.0%    0.0%    0.0%    0.0%    0.0%    0.0%    0.0%    0.0%    0.0%    0.0%    0.0%    0.0%    0.0%    0.0%    0.0%    0.0%    0.0%    0.0%    0.0%    0.0%    0.0%    0.0%    0.0%    0.0%    0.0%    0.0%    0.0%    0.0%    0.0%    0.0%    0.0%    0.0%    0.0%    0.0%    0.0%    0.0%    0.0%    0.0%    0.0%    0.0%    0.0%    0.0%    0.0%    0.0%    0.0%    0.0%    0.0%    0.0%    0.0%    0.0%    0.0%    0.0%    0.0%    0.0%    0.0%    0.0%    0.0%    0.0%    0.0%    0.0%    0.0%    0.0%    0.0%    0.0%    0.0%    0.0%    0.0%    0.0%    0.0%    0.0%    0.0%    0.0%    0.0%    0.0%    0.0%    0.0%    0.0%    0.0%    0.0%    0.0%    0.0%    0.0%    0.0%    0.0%    0.0%    0.0%    0.0%    0.0%    0.0%    0.0%    0.0%    0.0%    0.0%    0.0%    0.0%    0.0%    0.0%    0.0%        k__Bacteria;p__TM7;c__TM7-3; o__I025       2    0.0%    0.0%    0.0%    0.0%    0.0%    0.0%    0.0%    0.0%    0.0%    0.0%    0.0%    0.0%    0.0%    0.0%    0.0%    0.0%    0.0%    0.0%    0.0%    0.0%    0.0%    0.0%    0.0%    0.0%    0.0%    0.0%    0.0%    0.0%    0.0%    0.0%    0.0%    0.0%    0.0%    0.0%    0.0%    0.0%    0.0%    0.0%    0.0%    0.0%    0.0%    0.0%    0.0%    0.0%    0.0%    0.0%    0.0%    0.0%    0.0%    0.0%    0.0%    0.0%    0.0%    0.0%    0.0%    0.0%    0.0%    0.0%    0.0%    0.0%    0.0%    0.0%    0.0%    0.0%    0.0%    0.0%    0.0%    0.0%    0.0%    0.0%    0.0%    0.0%    0.0%    0.0%    0.0%    0.0%    0.0%    0.0%    0.0%    0.0%    0.0%    0.0%    0.0%    0.0%    0.0%    0.0%    0.0%    0.0%    0.0%    0.0%    0.0%    0.0%    0.0%    0.0%    0.0%    0.0%    0.0%    0.0%    0.0%    0.0%    0.0%    0.0%    0.0%    0.0%    0.0%    0.0%    0.0%    0.0%    0.0%    0.0%    0.0%    0.0%    0.0%    0.0%    0.0%    0.0%    0.0%    0.0%    0.0%    0.0%    0.0%    0.0%    0.0%    0.0%    0.0%    0.0%    0.0%    0.0%    0.0%    0.0%    0.0%    0.0%    0.0%    0.0%    0.0%    0.0%    0.0%    0.0%    0.0%    0.0%    0.0%    0.0%    0.0%    0.0%    0.0%    0.0%    0.0%    0.0%    0.0%    0.0%    0.0%    0.0%    0.0%    0.0%    0.0%    0.0%    0.0%    0.0%    0.0%    0.0%    0.0%    0.0%    0.0%    0.0%    0.0%    0.0%    0.0%    0.0%    0.0%    0.0%    0.0%    0.0%    0.0%    0.0%    0.0%    0.0%    0.0%    0.0%    0.0%    0.0%    0.0%    0.0%    0.0%    0.0%    0.0%    0.0%    0.0%    0.0%    0.0%    0.0%    0.0%    0.0%    0.0%    0.0%    0.0%    0.0%    0.0%    0.0%    0.0%    0.0%    0.0%    0.0%    0.0%    0.0%    0.0%    0.0%    0.0%    0.0%    0.0%    0.0%    0.0%    0.0%    0.0%    0.0%    0.0%    0.0%    0.0%    0.0%    0.0%    0.0%    0.0%    0.0%    0.0%    0.0%    0.0%    0.0%    0.0%    0.0%    0.0%    0.0%    0.0%    0.0%    0.0%    0.0%    0.0%    0.0%    0.0%    0.0%    0.0%    0.0%    0.0%    0.0%    0.0%    0.0%    0.0%    0.0%    0.0%    0.0%    0.0%    0.0%    0.0%    0.0%    0.0%    0.0%    0.0%    0.0%    0.0%    0.0%    0.0%    0.0%    0.0%    0.0%    0.0%    0.0%    0.0%    0.0%    0.0%    0.0%    0.0%    0.0%    0.0%    0.0%    0.0%    0.0%    0.0%    0.0%    0.0%    0.0%    0.0%    0.0%    0.0%    0.0%    0.0%    0.0%    0.0%    0.0%    0.0%    0.0%    0.0%    0.0%    0.0%    0.0%    0.0%    0.0%    0.0%    0.0%    0.0%    0.0%    0.0%    0.0%    0.0%    0.0%    0.0%    0.0%    0.0%    0.0%    0.0%    0.0%    0.0%    0.0%    0.0%    0.0%    0.0%    0.0%    0.0%    0.0%    0.0%    0.0%    0.0%    0.0%    0.0%    0.0%    0.0%    0.0%    0.0%    0.0%    0.0%    0.0%    0.0%    0.0%    0.0%    0.0%    0.0%    0.0%    0.0%    0.0%    0.0%    0.0%    0.0%    0.0%    0.0%    0.0%    0.0%    0.0%    0.0%    0.0%    0.0%    0.0%    0.0%    0.0%    0.0%    0.0%    0.0%    0.0%    0.0%    0.0%    0.0%    0.0%        k__Bacteria;p__Tenericutes;c__; o__       2    0.0%    0.0%    0.0%    0.0%    0.0%    0.0%    0.0%    0.0%    0.0%    0.0%    0.0%    0.0%    0.0%    0.0%    0.0%    0.0%    0.0%    0.0%    0.0%    0.0%    0.0%    0.0%    0.0%    0.0%    0.0%    0.0%    0.0%    0.0%    0.0%    0.0%    0.0%    0.0%    0.0%    0.0%    0.0%    0.0%    0.0%    0.0%    0.0%    0.0%    0.0%    0.0%    0.0%    0.0%    0.0%    0.0%    0.0%    0.0%    0.0%    0.0%    0.0%    0.0%    0.0%    0.0%    0.0%    0.0%    0.0%    0.0%    0.0%    0.0%    0.0%    0.0%    0.0%    0.0%    0.0%    0.0%    0.0%    0.0%    0.0%    0.0%    0.0%    0.0%    0.0%    0.0%    0.0%    0.0%    0.0%    0.0%    0.0%    0.0%    0.0%    0.0%    0.0%    0.0%    0.0%    0.0%    0.0%    0.0%    0.0%    0.0%    0.0%    0.0%    0.0%    0.0%    0.0%    0.0%    0.0%    0.0%    0.0%    0.0%    0.0%    0.0%    0.0%    0.0%    0.0%    0.0%    0.0%    0.0%    0.0%    0.0%    0.0%    0.0%    0.0%    0.0%    0.0%    0.0%    0.0%    0.0%    0.0%    0.0%    0.0%    0.0%    0.0%    0.0%    0.0%    0.0%    0.0%    0.0%    0.0%    0.0%    0.0%    0.0%    0.0%    0.0%    0.0%    0.0%    0.0%    0.0%    0.0%    0.0%    0.0%    0.0%    0.0%    0.0%    0.0%    0.0%    0.0%    0.0%    0.0%    0.0%    0.0%    0.0%    0.0%    0.0%    0.0%    0.0%    0.0%    0.0%    0.0%    0.0%    0.0%    0.0%    0.0%    0.0%    0.0%    0.0%    0.0%    0.0%    0.0%    0.0%    0.0%    0.0%    0.0%    0.0%    0.0%    0.0%    0.0%    0.0%    0.0%    0.0%    0.0%    0.0%    0.0%    0.0%    0.0%    0.0%    0.0%    0.0%    0.0%    0.0%    0.0%    0.0%    0.0%    0.0%    0.0%    0.0%    0.0%    0.0%    0.0%    0.0%    0.0%    0.0%    0.0%    0.0%    0.0%    0.0%    0.0%    0.0%    0.0%    0.0%    0.0%    0.0%    0.0%    0.0%    0.0%    0.0%    0.0%    0.0%    0.0%    0.0%    0.0%    0.0%    0.0%    0.0%    0.0%    0.0%    0.0%    0.0%    0.0%    0.0%    0.0%    0.0%    0.0%    0.0%    0.0%    0.0%    0.0%    0.0%    0.0%    0.0%    0.0%    0.0%    0.0%    0.0%    0.0%    0.0%    0.0%    0.0%    0.0%    0.0%    0.0%    0.0%    0.0%    0.0%    0.0%    0.0%    0.0%    0.0%    0.0%    0.0%    0.0%    0.0%    0.0%    0.0%    0.0%    0.0%    0.0%    0.0%    0.0%    0.0%    0.0%    0.0%    0.0%    0.0%    0.0%    0.0%    0.0%    0.0%    0.0%    0.0%    0.0%    0.0%    0.0%    0.0%    0.0%    0.0%    0.0%    0.0%    0.0%    0.0%    0.0%    0.0%    0.0%    0.0%    0.0%    0.0%    0.0%    0.0%    0.0%    0.0%    0.0%    0.0%    0.0%    0.0%    0.0%    0.0%    0.0%    0.0%    0.0%    0.0%    0.0%    0.0%    0.0%    0.0%    0.0%    0.0%    0.0%    0.0%    0.0%    0.0%    0.0%    0.0%    0.0%    0.0%    0.0%    0.0%    0.0%    0.0%    0.0%    0.0%    0.0%    0.0%    0.0%    0.0%    0.0%    0.0%    0.0%    0.0%    0.0%    0.0%    0.0%    0.0%    0.0%    0.0%    0.0%    0.0%    0.0%    0.0%    0.0%    0.0%    0.0%    0.0%    0.0%    0.0%    0.0%    0.0%    0.0%    0.0%        k__Bacteria;p__Tenericutes;c__Erysipelotrichi; o__Erysipelotrichales   24056    0.1%    0.2%    0.1%    1.2%    0.2%    0.0%    0.0%    0.0%    0.0%    0.0%    0.0%    0.0%    0.1%    0.1%    0.0%    0.0%    0.1%    0.3%    0.1%    0.0%    0.4%    0.3%    0.0%    0.0%    0.0%    0.0%    0.1%    0.6%    0.8%    0.1%    0.0%    0.2%    0.0%    0.1%    0.6%    0.1%    0.3%    0.0%    0.1%    0.1%    0.1%    0.3%    0.1%    0.1%    0.2%    0.2%    0.0%    0.0%    0.0%    0.0%    0.1%    0.0%    0.0%    0.0%    0.2%    0.0%    0.0%    0.0%    0.1%    0.0%    0.0%    0.1%    0.0%    0.2%    2.3%    0.0%    0.0%    0.2%    0.1%    0.2%    0.0%    0.0%    0.1%    0.1%    0.1%    0.0%    0.0%    0.1%    0.2%    0.1%    0.1%    0.0%    0.2%    0.1%    0.1%    0.0%    0.1%    0.0%    0.0%    0.1%    0.0%    0.1%    0.1%    0.0%    0.0%    0.0%    0.1%    0.0%    2.6%    1.7%    0.3%    0.1%    0.1%    0.1%    0.0%    0.0%    0.7%    0.0%    0.0%    0.2%    0.1%    1.6%    0.1%    0.0%    0.5%    0.1%    0.0%    0.0%    0.2%    0.1%    0.0%    0.3%    0.5%    0.1%    0.0%    0.3%    0.2%    0.0%    0.2%    0.6%    0.3%    0.1%    0.0%    0.1%    0.9%    0.0%    0.1%    0.0%    0.0%    0.0%    0.0%    0.2%    0.2%    0.0%    0.1%    0.0%    0.1%    0.0%    0.0%    0.0%    0.1%    0.1%    0.3%    0.0%    0.1%    0.0%    0.2%    0.0%    0.0%    0.2%    0.0%    0.1%    0.0%    0.0%    0.1%    0.0%    0.1%    0.0%    0.1%    0.1%    0.4%    0.4%    0.0%    0.1%    0.0%    0.0%    0.0%    0.1%    0.0%    0.0%    0.0%    0.0%    0.3%    0.1%    0.0%    0.0%    0.0%    0.0%    0.0%    0.1%    0.0%    0.1%    0.0%    0.0%    0.0%    0.1%    0.2%    0.1%    0.0%    0.0%    0.7%    2.1%    0.1%    0.4%    0.3%    0.2%    0.1%    0.1%    0.3%    0.2%    0.1%    0.0%    0.0%    0.1%    0.0%    0.1%    0.2%    0.2%    0.0%    0.1%    0.2%    0.1%    0.0%    0.1%    0.0%    0.2%    0.0%    0.1%    0.1%    0.1%    0.0%    0.0%    0.0%    0.0%    0.1%    0.0%    0.1%    0.0%    0.0%    0.0%    0.0%    0.0%    0.1%    0.1%    0.0%    0.0%    0.0%    0.0%    0.0%    0.0%    0.1%    0.1%    0.0%    0.0%    0.0%    0.0%    0.0%    0.0%    0.0%    0.1%    0.1%    0.1%    0.0%    0.0%    0.0%    0.0%    0.0%    0.1%    0.1%    0.0%    0.1%    0.0%    0.1%    0.0%    0.1%    0.1%    0.0%    0.1%    0.1%    0.0%    0.0%    0.0%    0.0%    0.0%    0.0%    0.0%    0.0%    0.2%    0.0%    0.0%    0.0%    0.0%    0.1%    0.1%    0.0%    0.0%    0.3%    0.1%    0.1%    0.0%    0.1%    0.1%    0.1%    0.1%    0.0%    0.1%    0.1%    0.0%    0.0%    0.1%    0.0%    0.0%    0.0%    0.0%    0.0%    0.1%    0.0%    0.0%    0.0%    0.1%    0.2%    0.0%    0.0%    0.0%    0.1%    0.0%    0.0%    0.0%    0.1%    0.0%    0.0%    0.0%    0.0%    0.0%    0.0%    0.0%    0.0%    0.1%    0.1%    0.0%    0.0%    0.0%    0.0%    0.0%    0.0%    0.0%    0.0%    0.0%    0.0%    0.0%    0.0%    0.0%    0.0%    0.1%    0.0%    0.0%    0.0%    0.0%        k__Bacteria;p__Tenericutes;c__ML615J-28; o__       5    0.0%    0.0%    0.0%    0.0%    0.0%    0.0%    0.0%    0.0%    0.0%    0.0%    0.0%    0.0%    0.0%    0.0%    0.0%    0.0%    0.0%    0.0%    0.0%    0.0%    0.0%    0.0%    0.0%    0.0%    0.0%    0.0%    0.0%    0.0%    0.0%    0.0%    0.0%    0.0%    0.0%    0.0%    0.0%    0.0%    0.0%    0.0%    0.0%    0.0%    0.0%    0.0%    0.0%    0.0%    0.0%    0.0%    0.0%    0.0%    0.0%    0.0%    0.0%    0.0%    0.0%    0.0%    0.0%    0.0%    0.0%    0.0%    0.0%    0.0%    0.0%    0.0%    0.0%    0.0%    0.0%    0.0%    0.0%    0.0%    0.0%    0.0%    0.0%    0.0%    0.0%    0.0%    0.0%    0.0%    0.0%    0.0%    0.0%    0.0%    0.0%    0.0%    0.0%    0.0%    0.0%    0.0%    0.0%    0.0%    0.0%    0.0%    0.0%    0.0%    0.0%    0.0%    0.0%    0.0%    0.0%    0.0%    0.0%    0.0%    0.0%    0.0%    0.0%    0.0%    0.0%    0.0%    0.0%    0.0%    0.0%    0.0%    0.0%    0.0%    0.0%    0.0%    0.0%    0.0%    0.0%    0.0%    0.0%    0.0%    0.0%    0.0%    0.0%    0.0%    0.0%    0.0%    0.0%    0.0%    0.0%    0.0%    0.0%    0.0%    0.0%    0.0%    0.0%    0.0%    0.0%    0.0%    0.0%    0.0%    0.0%    0.0%    0.0%    0.0%    0.0%    0.0%    0.0%    0.0%    0.0%    0.0%    0.0%    0.0%    0.0%    0.0%    0.0%    0.0%    0.0%    0.0%    0.0%    0.0%    0.0%    0.0%    0.0%    0.0%    0.0%    0.0%    0.0%    0.0%    0.0%    0.0%    0.0%    0.0%    0.0%    0.0%    0.0%    0.0%    0.0%    0.0%    0.0%    0.0%    0.0%    0.0%    0.0%    0.0%    0.0%    0.0%    0.0%    0.0%    0.0%    0.0%    0.0%    0.0%    0.0%    0.0%    0.0%    0.0%    0.0%    0.0%    0.0%    0.0%    0.0%    0.0%    0.0%    0.0%    0.0%    0.0%    0.0%    0.0%    0.0%    0.0%    0.0%    0.0%    0.0%    0.0%    0.0%    0.0%    0.0%    0.0%    0.0%    0.0%    0.0%    0.0%    0.0%    0.0%    0.0%    0.0%    0.0%    0.0%    0.0%    0.0%    0.0%    0.0%    0.0%    0.0%    0.0%    0.0%    0.0%    0.0%    0.0%    0.0%    0.0%    0.0%    0.0%    0.0%    0.0%    0.0%    0.0%    0.0%    0.0%    0.0%    0.0%    0.0%    0.0%    0.0%    0.0%    0.0%    0.0%    0.0%    0.0%    0.0%    0.0%    0.0%    0.0%    0.0%    0.0%    0.0%    0.0%    0.0%    0.0%    0.0%    0.0%    0.0%    0.0%    0.0%    0.0%    0.0%    0.0%    0.0%    0.0%    0.0%    0.0%    0.0%    0.0%    0.0%    0.0%    0.0%    0.0%    0.0%    0.0%    0.0%    0.0%    0.0%    0.0%    0.0%    0.0%    0.0%    0.0%    0.0%    0.0%    0.0%    0.0%    0.0%    0.0%    0.0%    0.0%    0.0%    0.0%    0.0%    0.0%    0.0%    0.0%    0.0%    0.0%    0.0%    0.0%    0.0%    0.0%    0.0%    0.0%    0.0%    0.0%    0.0%    0.0%    0.0%    0.0%    0.0%    0.0%    0.0%    0.0%    0.0%    0.0%    0.0%    0.0%    0.0%    0.0%    0.0%    0.0%    0.0%    0.0%    0.0%    0.0%    0.0%    0.0%    0.0%    0.0%    0.0%    0.0%    0.0%    0.0%    0.0%    0.0%    0.0%    0.0%    0.0%    0.0%    0.0%    0.0%    0.0%        k__Bacteria;p__Tenericutes;c__Mollicutes; o__Acholeplasmatales     636    0.0%    0.0%    0.0%    0.0%    0.0%    0.0%    0.0%    0.0%    0.0%    0.0%    0.0%    0.0%    0.0%    0.0%    0.0%    0.0%    0.0%    0.0%    0.0%    0.0%    0.0%    0.0%    0.0%    0.0%    0.0%    0.0%    0.0%    0.0%    0.0%    0.0%    0.0%    0.0%    0.0%    0.0%    0.0%    0.0%    0.0%    0.0%    0.0%    0.0%    0.0%    0.0%    0.0%    0.0%    0.2%    0.4%    0.0%    0.0%    0.1%    0.1%    0.0%    0.0%    0.0%    0.0%    0.0%    0.0%    0.0%    0.0%    0.0%    0.0%    0.0%    0.0%    0.0%    0.0%    0.0%    0.0%    0.0%    0.0%    0.0%    0.0%    0.0%    0.0%    0.0%    0.0%    0.0%    0.0%    0.0%    0.0%    0.0%    0.0%    0.0%    0.0%    0.0%    0.0%    0.0%    0.0%    0.0%    0.0%    0.0%    0.0%    0.0%    0.0%    0.0%    0.0%    0.0%    0.0%    0.0%    0.0%    0.0%    0.0%    0.0%    0.0%    0.0%    0.0%    0.0%    0.0%    0.0%    0.0%    0.0%    0.0%    0.0%    0.0%    0.0%    0.0%    0.0%    0.0%    0.0%    0.0%    0.0%    0.0%    0.0%    0.0%    0.0%    0.0%    0.0%    0.0%    0.0%    0.0%    0.0%    0.0%    0.0%    0.0%    0.0%    0.0%    0.0%    0.0%    0.0%    0.0%    0.0%    0.0%    0.0%    0.0%    0.0%    0.0%    0.0%    0.0%    0.0%    0.0%    0.0%    0.0%    0.0%    0.0%    0.0%    0.0%    0.0%    0.0%    0.0%    0.0%    0.0%    0.0%    0.0%    0.0%    0.0%    0.0%    0.0%    0.0%    0.0%    0.0%    0.0%    0.0%    0.0%    0.0%    0.0%    0.0%    0.0%    0.0%    0.0%    0.0%    0.0%    0.0%    0.0%    0.0%    0.0%    0.0%    0.0%    0.0%    0.0%    0.0%    0.0%    0.0%    0.0%    0.0%    0.0%    0.0%    0.0%    0.0%    0.0%    0.0%    0.0%    0.0%    0.0%    0.0%    0.0%    0.0%    0.0%    0.0%    0.0%    0.0%    0.0%    0.0%    0.0%    0.0%    0.0%    0.0%    0.0%    0.0%    0.0%    0.0%    0.0%    0.0%    0.0%    0.0%    0.0%    0.0%    0.0%    0.0%    0.0%    0.0%    0.0%    0.0%    0.0%    0.0%    0.0%    0.0%    0.0%    0.0%    0.0%    0.0%    0.0%    0.0%    0.0%    0.0%    0.0%    0.0%    0.0%    0.0%    0.0%    0.0%    0.0%    0.0%    0.0%    0.0%    0.0%    0.0%    0.0%    0.0%    0.0%    0.0%    0.0%    0.0%    0.0%    0.0%    0.0%    0.0%    0.0%    0.0%    0.0%    0.0%    0.0%    0.0%    0.0%    0.0%    0.0%    0.0%    0.0%    0.0%    0.0%    0.0%    0.0%    0.0%    0.0%    0.0%    0.0%    0.0%    0.0%    0.0%    0.0%    0.0%    0.0%    0.0%    0.0%    0.0%    0.0%    0.0%    0.0%    0.0%    0.0%    0.0%    0.0%    0.0%    0.0%    0.0%    0.0%    0.0%    0.0%    0.0%    0.0%    0.0%    0.0%    0.0%    0.0%    0.0%    0.0%    0.0%    0.0%    0.0%    0.0%    0.0%    0.0%    0.0%    0.0%    0.0%    0.0%    0.0%    0.0%    0.0%    0.0%    0.0%    0.0%    0.0%    0.0%    0.0%    0.0%    0.0%    0.0%    0.0%    0.0%    0.0%    0.0%    0.0%    0.0%    0.0%    0.0%    0.0%    0.0%    0.0%    0.0%    0.0%    0.0%    0.0%    0.0%    0.0%    0.0%    0.0%    0.0%    0.0%    0.0%    0.0%        k__Bacteria;p__Tenericutes;c__Mollicutes; o__Anaeroplasmatales       3    0.0%    0.0%    0.0%    0.0%    0.0%    0.0%    0.0%    0.0%    0.0%    0.0%    0.0%    0.0%    0.0%    0.0%    0.0%    0.0%    0.0%    0.0%    0.0%    0.0%    0.0%    0.0%    0.0%    0.0%    0.0%    0.0%    0.0%    0.0%    0.0%    0.0%    0.0%    0.0%    0.0%    0.0%    0.0%    0.0%    0.0%    0.0%    0.0%    0.0%    0.0%    0.0%    0.0%    0.0%    0.0%    0.0%    0.0%    0.0%    0.0%    0.0%    0.0%    0.0%    0.0%    0.0%    0.0%    0.0%    0.0%    0.0%    0.0%    0.0%    0.0%    0.0%    0.0%    0.0%    0.0%    0.0%    0.0%    0.0%    0.0%    0.0%    0.0%    0.0%    0.0%    0.0%    0.0%    0.0%    0.0%    0.0%    0.0%    0.0%    0.0%    0.0%    0.0%    0.0%    0.0%    0.0%    0.0%    0.0%    0.0%    0.0%    0.0%    0.0%    0.0%    0.0%    0.0%    0.0%    0.0%    0.0%    0.0%    0.0%    0.0%    0.0%    0.0%    0.0%    0.0%    0.0%    0.0%    0.0%    0.0%    0.0%    0.0%    0.0%    0.0%    0.0%    0.0%    0.0%    0.0%    0.0%    0.0%    0.0%    0.0%    0.0%    0.0%    0.0%    0.0%    0.0%    0.0%    0.0%    0.0%    0.0%    0.0%    0.0%    0.0%    0.0%    0.0%    0.0%    0.0%    0.0%    0.0%    0.0%    0.0%    0.0%    0.0%    0.0%    0.0%    0.0%    0.0%    0.0%    0.0%    0.0%    0.0%    0.0%    0.0%    0.0%    0.0%    0.0%    0.0%    0.0%    0.0%    0.0%    0.0%    0.0%    0.0%    0.0%    0.0%    0.0%    0.0%    0.0%    0.0%    0.0%    0.0%    0.0%    0.0%    0.0%    0.0%    0.0%    0.0%    0.0%    0.0%    0.0%    0.0%    0.0%    0.0%    0.0%    0.0%    0.0%    0.0%    0.0%    0.0%    0.0%    0.0%    0.0%    0.0%    0.0%    0.0%    0.0%    0.0%    0.0%    0.0%    0.0%    0.0%    0.0%    0.0%    0.0%    0.0%    0.0%    0.0%    0.0%    0.0%    0.0%    0.0%    0.0%    0.0%    0.0%    0.0%    0.0%    0.0%    0.0%    0.0%    0.0%    0.0%    0.0%    0.0%    0.0%    0.0%    0.0%    0.0%    0.0%    0.0%    0.0%    0.0%    0.0%    0.0%    0.0%    0.0%    0.0%    0.0%    0.0%    0.0%    0.0%    0.0%    0.0%    0.0%    0.0%    0.0%    0.0%    0.0%    0.0%    0.0%    0.0%    0.0%    0.0%    0.0%    0.0%    0.0%    0.0%    0.0%    0.0%    0.0%    0.0%    0.0%    0.0%    0.0%    0.0%    0.0%    0.0%    0.0%    0.0%    0.0%    0.0%    0.0%    0.0%    0.0%    0.0%    0.0%    0.0%    0.0%    0.0%    0.0%    0.0%    0.0%    0.0%    0.0%    0.0%    0.0%    0.0%    0.0%    0.0%    0.0%    0.0%    0.0%    0.0%    0.0%    0.0%    0.0%    0.0%    0.0%    0.0%    0.0%    0.0%    0.0%    0.0%    0.0%    0.0%    0.0%    0.0%    0.0%    0.0%    0.0%    0.0%    0.0%    0.0%    0.0%    0.0%    0.0%    0.0%    0.0%    0.0%    0.0%    0.0%    0.0%    0.0%    0.0%    0.0%    0.0%    0.0%    0.0%    0.0%    0.0%    0.0%    0.0%    0.0%    0.0%    0.0%    0.0%    0.0%    0.0%    0.0%    0.0%    0.0%    0.0%    0.0%    0.0%    0.0%    0.0%    0.0%    0.0%    0.0%    0.0%    0.0%    0.0%    0.0%    0.0%    0.0%    0.0%    0.0%    0.0%    0.0%        k__Bacteria;p__Tenericutes;c__Mollicutes; o__Entomoplasmatales     103    0.0%    0.0%    0.0%    0.0%    0.0%    0.0%    0.0%    0.0%    0.0%    0.0%    0.0%    0.0%    0.0%    0.0%    0.0%    0.0%    0.0%    0.0%    0.0%    0.0%    0.0%    0.0%    0.0%    0.0%    0.0%    0.0%    0.0%    0.0%    0.0%    0.0%    0.0%    0.0%    0.0%    0.0%    0.0%    0.0%    0.0%    0.0%    0.0%    0.0%    0.0%    0.0%    0.0%    0.0%    0.0%    0.0%    0.0%    0.0%    0.0%    0.0%    0.0%    0.0%    0.0%    0.0%    0.0%    0.0%    0.0%    0.0%    0.0%    0.0%    0.0%    0.0%    0.0%    0.0%    0.0%    0.0%    0.0%    0.0%    0.0%    0.0%    0.0%    0.0%    0.0%    0.0%    0.0%    0.0%    0.0%    0.0%    0.0%    0.0%    0.0%    0.0%    0.0%    0.0%    0.0%    0.0%    0.0%    0.0%    0.0%    0.0%    0.0%    0.0%    0.0%    0.0%    0.0%    0.0%    0.0%    0.0%    0.0%    0.0%    0.0%    0.0%    0.0%    0.0%    0.0%    0.0%    0.0%    0.0%    0.0%    0.0%    0.0%    0.0%    0.0%    0.0%    0.0%    0.0%    0.0%    0.0%    0.0%    0.0%    0.0%    0.0%    0.0%    0.0%    0.0%    0.0%    0.0%    0.0%    0.0%    0.0%    0.0%    0.0%    0.0%    0.0%    0.0%    0.0%    0.0%    0.0%    0.0%    0.0%    0.0%    0.0%    0.0%    0.0%    0.0%    0.0%    0.0%    0.0%    0.0%    0.0%    0.0%    0.0%    0.0%    0.0%    0.0%    0.0%    0.0%    0.0%    0.0%    0.2%    0.0%    0.0%    0.0%    0.0%    0.0%    0.0%    0.0%    0.0%    0.0%    0.0%    0.0%    0.0%    0.0%    0.0%    0.0%    0.0%    0.0%    0.0%    0.0%    0.0%    0.0%    0.0%    0.0%    0.0%    0.0%    0.0%    0.0%    0.0%    0.0%    0.0%    0.0%    0.0%    0.0%    0.0%    0.0%    0.0%    0.0%    0.0%    0.0%    0.0%    0.0%    0.0%    0.0%    0.0%    0.0%    0.0%    0.0%    0.0%    0.0%    0.0%    0.0%    0.0%    0.0%    0.0%    0.0%    0.0%    0.0%    0.0%    0.0%    0.0%    0.0%    0.0%    0.0%    0.0%    0.0%    0.0%    0.0%    0.0%    0.0%    0.0%    0.0%    0.0%    0.0%    0.0%    0.0%    0.0%    0.0%    0.0%    0.0%    0.0%    0.0%    0.0%    0.0%    0.0%    0.0%    0.0%    0.0%    0.0%    0.0%    0.0%    0.0%    0.0%    0.0%    0.0%    0.0%    0.0%    0.0%    0.0%    0.0%    0.0%    0.0%    0.0%    0.0%    0.0%    0.0%    0.0%    0.0%    0.0%    0.0%    0.0%    0.0%    0.0%    0.0%    0.0%    0.0%    0.0%    0.0%    0.0%    0.0%    0.0%    0.0%    0.0%    0.0%    0.0%    0.0%    0.0%    0.0%    0.0%    0.0%    0.0%    0.0%    0.0%    0.0%    0.0%    0.0%    0.0%    0.0%    0.0%    0.0%    0.0%    0.0%    0.0%    0.0%    0.0%    0.0%    0.0%    0.0%    0.0%    0.0%    0.0%    0.0%    0.0%    0.0%    0.0%    0.0%    0.0%    0.0%    0.0%    0.0%    0.0%    0.0%    0.0%    0.0%    0.0%    0.0%    0.0%    0.0%    0.0%    0.0%    0.0%    0.0%    0.0%    0.0%    0.0%    0.0%    0.0%    0.0%    0.0%    0.0%    0.0%    0.0%    0.0%    0.0%    0.0%    0.0%    0.0%    0.0%    0.0%    0.0%    0.0%    0.0%    0.0%    0.0%    0.0%    0.0%    0.0%    0.0%    0.0%        k__Bacteria;p__Tenericutes;c__Mollicutes; o__Mycoplasmatales   31501    0.2%    0.0%    0.1%    0.0%    0.0%    0.0%    0.0%    0.0%    0.0%    0.0%    0.0%    0.0%    0.9%    0.1%    0.0%    0.0%    0.3%    0.4%    0.1%    0.0%    0.3%    0.8%    0.0%    0.0%    0.0%    0.2%    0.1%    0.2%    0.7%    0.0%    0.0%    0.1%    0.0%    0.0%    1.1%    0.1%    0.6%    0.0%    0.3%    0.4%    3.1%    1.1%    0.1%    0.1%    0.6%    0.5%    0.0%    0.0%    0.1%    0.0%    0.2%    0.0%    0.0%    0.1%    0.4%    0.3%    0.1%    0.1%    0.7%    0.2%    0.2%    0.2%    0.2%    0.5%    0.3%    0.1%    0.1%    0.6%    0.1%    0.4%    0.1%    0.1%    0.1%    0.1%    0.2%    0.0%    0.0%    0.4%    0.1%    1.3%    0.2%    0.1%    0.4%    0.4%    0.0%    0.0%    0.5%    0.0%    0.1%    0.6%    0.0%    0.1%    0.1%    0.2%    0.2%    0.1%    0.1%    0.0%    0.1%    0.7%    0.2%    0.1%    0.0%    0.1%    0.0%    0.0%    0.7%    0.0%    0.0%    0.1%    0.0%    0.2%    0.0%    0.0%    0.0%    0.0%    0.0%    0.0%    0.1%    0.0%    0.0%    0.0%    0.1%    0.0%    0.0%    0.1%    0.0%    0.0%    0.3%    0.7%    0.2%    0.0%    0.0%    0.0%    0.5%    0.0%    0.1%    0.0%    0.0%    0.0%    0.0%    0.1%    0.5%    0.1%    0.1%    0.1%    0.0%    0.0%    0.1%    0.0%    0.0%    0.1%    0.5%    0.2%    0.0%    0.0%    0.9%    0.1%    0.1%    0.6%    1.4%    0.9%    1.1%    0.2%    0.2%    0.2%    0.7%    0.1%    0.2%    0.1%    0.3%    0.8%    0.1%    0.4%    0.0%    0.0%    0.0%    0.1%    0.0%    0.0%    0.0%    0.0%    0.2%    0.0%    0.1%    0.0%    0.0%    0.0%    0.0%    0.0%    0.0%    0.0%    0.0%    0.0%    0.1%    0.1%    0.3%    0.0%    0.0%    0.0%    0.5%    0.1%    0.1%    0.2%    0.3%    0.2%    0.1%    0.2%    0.6%    0.5%    0.5%    0.0%    0.0%    0.0%    0.0%    0.0%    0.0%    0.0%    0.0%    0.0%    0.1%    0.1%    0.0%    0.1%    0.0%    0.1%    0.1%    0.1%    0.2%    0.2%    0.0%    0.0%    0.0%    0.1%    0.2%    0.0%    0.1%    0.0%    0.0%    0.0%    0.0%    0.0%    0.0%    0.0%    0.0%    0.0%    0.0%    0.0%    0.0%    0.5%    0.4%    0.1%    0.1%    0.0%    0.0%    0.0%    0.1%    0.1%    1.5%    0.1%    0.8%    0.3%    0.8%    0.2%    0.2%    0.0%    0.2%    0.3%    0.1%    0.0%    0.1%    0.1%    0.0%    0.1%    0.0%    0.0%    0.0%    0.0%    0.0%    0.0%    0.0%    0.0%    0.0%    0.0%    0.0%    0.0%    0.0%    0.1%    0.0%    0.0%    0.0%    0.0%    0.0%    0.1%    0.0%    0.0%    0.0%    0.1%    0.0%    0.1%    0.2%    0.0%    0.1%    0.4%    0.3%    0.0%    0.1%    0.1%    0.1%    0.1%    0.0%    0.0%    0.0%    0.0%    0.0%    0.3%    0.0%    0.0%    0.0%    0.0%    0.0%    0.1%    0.0%    0.0%    0.0%    0.0%    0.1%    0.0%    0.1%    0.0%    0.0%    0.0%    0.0%    0.0%    0.0%    0.0%    0.0%    0.0%    0.0%    0.0%    0.0%    0.0%    0.0%    0.1%    0.0%    0.0%    0.0%    0.0%    0.0%    0.0%    0.0%    0.0%    0.0%    0.1%    0.0%    0.0%    0.0%    0.0%        k__Bacteria;p__Tenericutes;c__Mollicutes; o__RF39      55    0.0%    0.0%    0.0%    0.0%    0.0%    0.0%    0.0%    0.0%    0.0%    0.0%    0.0%    0.0%    0.0%    0.0%    0.0%    0.0%    0.0%    0.0%    0.0%    0.0%    0.0%    0.0%    0.0%    0.0%    0.0%    0.0%    0.0%    0.0%    0.0%    0.0%    0.0%    0.0%    0.0%    0.0%    0.0%    0.0%    0.0%    0.0%    0.0%    0.0%    0.0%    0.0%    0.0%    0.0%    0.0%    0.0%    0.0%    0.0%    0.0%    0.0%    0.0%    0.0%    0.0%    0.0%    0.0%    0.0%    0.0%    0.0%    0.0%    0.0%    0.0%    0.0%    0.0%    0.0%    0.0%    0.0%    0.0%    0.0%    0.0%    0.0%    0.0%    0.0%    0.0%    0.0%    0.0%    0.0%    0.0%    0.0%    0.0%    0.0%    0.0%    0.0%    0.0%    0.0%    0.0%    0.0%    0.0%    0.0%    0.0%    0.0%    0.0%    0.0%    0.0%    0.0%    0.0%    0.0%    0.0%    0.0%    0.0%    0.0%    0.0%    0.0%    0.0%    0.0%    0.0%    0.0%    0.0%    0.0%    0.0%    0.0%    0.0%    0.0%    0.0%    0.0%    0.0%    0.0%    0.0%    0.0%    0.0%    0.0%    0.0%    0.0%    0.0%    0.0%    0.0%    0.0%    0.0%    0.0%    0.0%    0.0%    0.0%    0.0%    0.0%    0.0%    0.0%    0.0%    0.0%    0.0%    0.0%    0.0%    0.0%    0.0%    0.0%    0.0%    0.0%    0.0%    0.0%    0.0%    0.0%    0.0%    0.0%    0.0%    0.0%    0.0%    0.0%    0.0%    0.0%    0.0%    0.0%    0.0%    0.0%    0.0%    0.0%    0.0%    0.0%    0.0%    0.0%    0.0%    0.0%    0.0%    0.0%    0.0%    0.0%    0.0%    0.0%    0.0%    0.0%    0.0%    0.0%    0.0%    0.0%    0.0%    0.0%    0.0%    0.0%    0.0%    0.0%    0.0%    0.0%    0.0%    0.0%    0.0%    0.0%    0.0%    0.0%    0.0%    0.0%    0.0%    0.0%    0.0%    0.0%    0.0%    0.0%    0.0%    0.0%    0.0%    0.0%    0.0%    0.0%    0.0%    0.0%    0.0%    0.0%    0.0%    0.0%    0.0%    0.0%    0.0%    0.0%    0.0%    0.0%    0.0%    0.0%    0.0%    0.0%    0.0%    0.0%    0.0%    0.0%    0.0%    0.0%    0.0%    0.0%    0.0%    0.0%    0.0%    0.0%    0.0%    0.0%    0.0%    0.0%    0.0%    0.0%    0.0%    0.0%    0.0%    0.0%    0.0%    0.0%    0.0%    0.0%    0.0%    0.0%    0.0%    0.0%    0.0%    0.0%    0.0%    0.0%    0.0%    0.0%    0.0%    0.0%    0.0%    0.0%    0.0%    0.0%    0.0%    0.0%    0.0%    0.0%    0.0%    0.0%    0.0%    0.0%    0.0%    0.0%    0.0%    0.0%    0.0%    0.0%    0.0%    0.0%    0.0%    0.0%    0.0%    0.0%    0.0%    0.0%    0.0%    0.0%    0.0%    0.0%    0.0%    0.0%    0.0%    0.0%    0.0%    0.0%    0.0%    0.0%    0.0%    0.0%    0.0%    0.0%    0.0%    0.0%    0.0%    0.0%    0.0%    0.0%    0.0%    0.0%    0.0%    0.0%    0.0%    0.0%    0.0%    0.0%    0.0%    0.0%    0.0%    0.0%    0.0%    0.0%    0.0%    0.0%    0.0%    0.0%    0.0%    0.0%    0.0%    0.0%    0.0%    0.0%    0.0%    0.0%    0.0%    0.0%    0.0%    0.0%    0.0%    0.0%    0.0%    0.0%    0.0%    0.0%    0.0%    0.0%    0.0%    0.0%    0.0%    0.0%    0.0%    0.0%    0.0%    0.0%    0.0%        k__Bacteria;p__Thermi;c__Deinococci; o__Deinococcales    4108    0.0%    0.0%    0.0%    0.0%    0.0%    0.0%    0.0%    0.6%    0.0%    0.0%    0.0%    0.5%    0.0%    0.0%    0.0%    0.0%    0.0%    0.1%    0.0%    0.0%    0.0%    0.1%    0.0%    0.0%    0.0%    0.0%    0.0%    0.0%    0.0%    0.0%    0.0%    0.0%    0.0%    0.0%    0.0%    0.0%    0.0%    0.0%    0.0%    0.0%    0.0%    0.0%    0.0%    0.0%    0.0%    0.0%    0.0%    0.0%    0.0%    0.0%    0.0%    0.0%    0.0%    0.0%    0.0%    0.0%    0.0%    0.0%    0.0%    0.0%    0.0%    0.1%    0.0%    0.0%    0.0%    0.0%    0.0%    0.1%    0.0%    0.0%    0.0%    0.0%    0.0%    0.0%    0.1%    0.0%    0.0%    0.0%    0.0%    0.0%    0.0%    0.1%    0.0%    0.0%    0.0%    0.0%    0.0%    0.0%    0.0%    0.0%    0.0%    0.0%    0.0%    0.0%    0.0%    0.1%    0.0%    0.0%    0.0%    0.0%    0.0%    0.0%    0.0%    0.0%    0.0%    0.0%    0.1%    0.0%    0.0%    0.0%    0.0%    0.0%    0.0%    0.0%    0.0%    0.0%    0.0%    0.0%    0.0%    0.0%    0.0%    0.0%    0.0%    0.1%    0.0%    0.0%    0.0%    0.0%    0.0%    0.1%    0.0%    0.0%    0.0%    0.0%    0.0%    0.0%    0.0%    0.0%    0.0%    0.0%    0.0%    0.0%    0.0%    0.0%    0.0%    0.0%    0.0%    0.0%    0.0%    0.0%    0.0%    0.0%    0.0%    0.0%    0.0%    0.0%    0.0%    0.0%    0.0%    0.1%    0.0%    0.1%    0.0%    0.0%    0.0%    0.0%    0.0%    0.0%    0.1%    0.0%    0.0%    0.0%    0.0%    0.0%    0.1%    0.0%    0.0%    0.0%    0.0%    0.0%    0.0%    0.0%    0.0%    0.0%    0.0%    0.0%    0.0%    0.0%    0.0%    0.1%    0.0%    0.1%    0.0%    0.0%    0.0%    0.0%    0.1%    0.0%    0.1%    0.0%    0.1%    0.0%    0.0%    0.0%    0.1%    0.0%    0.0%    0.0%    0.1%    0.0%    0.0%    0.1%    0.0%    0.1%    0.0%    0.1%    0.0%    0.0%    0.0%    0.1%    0.0%    0.0%    0.0%    0.0%    0.0%    0.0%    0.0%    0.0%    0.0%    0.0%    0.0%    0.0%    0.0%    0.0%    0.1%    0.0%    0.0%    0.0%    0.0%    0.0%    0.0%    0.0%    0.0%    0.0%    0.0%    0.0%    0.0%    0.0%    0.0%    0.0%    0.1%    0.0%    0.0%    0.0%    0.0%    0.0%    0.0%    0.0%    0.0%    0.0%    0.0%    0.0%    0.1%    0.0%    0.0%    0.0%    0.0%    0.0%    0.0%    0.0%    0.0%    0.0%    0.2%    0.1%    0.0%    0.0%    0.0%    0.0%    0.0%    0.0%    0.0%    0.0%    0.0%    0.0%    0.1%    0.0%    0.0%    0.3%    0.1%    0.0%    0.0%    0.0%    0.2%    0.1%    0.1%    0.0%    0.1%    0.0%    0.0%    0.1%    0.0%    0.0%    0.0%    0.0%    0.0%    0.0%    0.1%    0.0%    0.0%    0.0%    0.0%    0.0%    0.0%    0.0%    0.0%    0.1%    0.1%    0.1%    0.0%    0.0%    0.0%    0.1%    0.0%    0.0%    0.1%    0.0%    0.0%    0.0%    0.1%    0.1%    0.0%    0.0%    0.0%    0.0%    0.0%    0.0%    0.0%    0.0%    0.0%    0.0%    0.1%    0.0%    0.0%    0.0%    0.0%    0.0%    0.0%    0.0%    0.0%    0.0%    0.1%    0.0%    0.0%    0.2%    0.0%    0.0%    0.0%    0.0%        k__Bacteria;p__Thermi;c__Deinococci; o__Thermales    4390    0.0%    0.0%    0.0%    0.0%    0.0%    0.0%    0.0%    0.0%    0.0%    0.0%    0.0%    0.0%    0.0%    0.0%    0.0%    0.0%    0.0%    0.0%    0.0%    0.0%    0.0%    0.0%    0.0%    0.0%    0.0%    0.0%    0.0%    0.0%    0.0%    0.0%    0.0%    0.0%    0.0%    0.0%    0.0%    0.0%    0.0%    0.0%    0.0%    0.0%    0.0%    0.0%    0.0%    0.0%    0.0%    0.0%    0.0%    0.0%    0.0%    0.0%    0.0%    0.0%    0.0%    0.0%    0.0%    0.0%    0.0%    0.0%    0.0%    0.0%    0.0%    0.0%    0.0%    0.0%    0.0%    0.0%    0.0%    0.0%    0.0%    0.0%    0.0%    0.0%    0.0%    0.0%    0.0%    0.0%    0.0%    0.0%    0.0%    0.0%    0.0%    0.0%    0.0%    0.0%    0.0%    0.0%    0.0%    0.0%    0.0%    0.0%    0.0%    0.0%    0.0%    0.0%    0.0%    0.0%    0.0%    0.0%    0.0%    0.0%    0.0%    0.0%    0.0%    0.3%    0.1%    0.1%    0.0%    0.0%    0.0%    0.4%    0.1%    0.2%    0.0%    0.0%    0.0%    0.0%    0.0%    0.0%    0.0%    0.0%    0.0%    0.1%    0.1%    0.1%    0.0%    0.0%    0.0%    0.0%    0.1%    0.0%    0.1%    0.0%    0.0%    0.1%    0.1%    0.0%    0.0%    0.0%    0.0%    0.0%    0.0%    0.0%    0.0%    0.0%    0.0%    0.0%    0.0%    0.0%    0.0%    0.0%    0.0%    0.0%    0.0%    0.0%    0.0%    0.0%    0.1%    0.0%    0.0%    0.1%    0.1%    0.1%    0.1%    0.0%    0.0%    0.0%    0.1%    0.1%    0.2%    0.0%    0.3%    0.2%    0.1%    2.0%    0.0%    0.0%    0.0%    0.0%    0.1%    0.1%    0.3%    0.1%    0.0%    0.0%    0.0%    0.0%    0.1%    0.0%    0.0%    0.0%    0.0%    0.0%    0.0%    0.0%    0.0%    0.0%    0.0%    0.0%    0.0%    0.0%    0.0%    0.0%    0.0%    0.0%    0.0%    0.0%    0.0%    0.0%    0.0%    0.0%    0.0%    0.0%    0.0%    0.0%    0.0%    0.0%    0.0%    0.0%    0.0%    0.1%    0.0%    0.0%    0.0%    0.0%    0.0%    0.0%    0.0%    0.0%    0.0%    0.0%    0.0%    0.0%    0.0%    0.0%    0.0%    0.0%    0.0%    0.0%    0.0%    0.0%    0.0%    0.0%    0.0%    0.0%    0.0%    0.0%    0.0%    0.0%    0.0%    0.0%    0.0%    0.0%    0.0%    0.0%    0.0%    0.0%    0.0%    0.0%    0.0%    0.0%    0.0%    0.0%    0.0%    0.0%    0.0%    0.0%    0.0%    0.0%    0.0%    0.0%    0.0%    0.0%    0.0%    0.0%    0.0%    0.0%    0.0%    0.0%    0.0%    0.0%    0.0%    0.0%    0.0%    0.0%    0.0%    0.0%    0.0%    0.0%    0.0%    0.0%    0.0%    0.0%    0.0%    0.0%    0.0%    0.0%    0.0%    0.0%    0.0%    0.0%    0.0%    0.0%    0.0%    0.0%    0.0%    0.0%    0.0%    0.0%    0.0%    0.0%    0.0%    0.0%    0.0%    0.0%    0.0%    0.0%    0.0%    0.0%    0.0%    0.0%    0.0%    0.0%    0.0%    0.0%    0.0%    0.0%    0.0%    0.0%    0.0%    0.0%    0.0%    0.0%    0.0%    0.1%    0.0%    0.0%    0.0%    0.0%    0.0%    0.0%    0.0%    0.0%    0.0%    0.0%    0.0%    0.0%    0.0%    0.0%    0.0%    0.0%    0.0%    0.0%    0.0%    0.0%    0.0%    0.0%    0.0%    0.0%        k__Bacteria;p__Thermotogae;c__Thermotogae (class); o__Thermotogales     106    0.0%    0.0%    0.0%    0.0%    0.0%    0.0%    0.0%    0.0%    0.0%    0.0%    0.0%    0.0%    0.0%    0.0%    0.0%    0.0%    0.0%    0.0%    0.0%    0.0%    0.0%    0.0%    0.0%    0.0%    0.0%    0.0%    0.0%    0.0%    0.0%    0.0%    0.0%    0.0%    0.0%    0.0%    0.0%    0.0%    0.0%    0.0%    0.0%    0.0%    0.0%    0.0%    0.0%    0.0%    0.0%    0.0%    0.0%    0.0%    0.0%    0.0%    0.0%    0.1%    0.0%    0.0%    0.0%    0.0%    0.0%    0.0%    0.0%    0.0%    0.0%    0.0%    0.0%    0.0%    0.0%    0.0%    0.0%    0.0%    0.0%    0.0%    0.0%    0.0%    0.0%    0.0%    0.0%    0.0%    0.0%    0.0%    0.0%    0.0%    0.0%    0.0%    0.0%    0.0%    0.0%    0.0%    0.0%    0.0%    0.0%    0.0%    0.0%    0.0%    0.0%    0.0%    0.0%    0.0%    0.0%    0.0%    0.0%    0.0%    0.0%    0.0%    0.0%    0.0%    0.0%    0.0%    0.0%    0.0%    0.0%    0.0%    0.0%    0.0%    0.0%    0.0%    0.0%    0.0%    0.0%    0.0%    0.0%    0.0%    0.0%    0.0%    0.0%    0.0%    0.0%    0.0%    0.0%    0.0%    0.0%    0.0%    0.0%    0.0%    0.0%    0.0%    0.0%    0.0%    0.0%    0.0%    0.0%    0.0%    0.0%    0.0%    0.0%    0.0%    0.0%    0.0%    0.0%    0.0%    0.0%    0.0%    0.0%    0.0%    0.0%    0.0%    0.0%    0.0%    0.0%    0.0%    0.0%    0.0%    0.0%    0.0%    0.0%    0.0%    0.0%    0.0%    0.0%    0.0%    0.0%    0.0%    0.0%    0.0%    0.0%    0.0%    0.0%    0.0%    0.0%    0.0%    0.0%    0.0%    0.0%    0.0%    0.0%    0.0%    0.0%    0.0%    0.0%    0.0%    0.0%    0.0%    0.0%    0.0%    0.0%    0.0%    0.0%    0.0%    0.0%    0.0%    0.0%    0.0%    0.0%    0.0%    0.0%    0.0%    0.0%    0.0%    0.0%    0.0%    0.0%    0.0%    0.0%    0.0%    0.0%    0.0%    0.0%    0.0%    0.0%    0.0%    0.0%    0.0%    0.0%    0.0%    0.0%    0.0%    0.0%    0.0%    0.0%    0.0%    0.0%    0.0%    0.0%    0.0%    0.0%    0.0%    0.0%    0.0%    0.0%    0.0%    0.0%    0.0%    0.0%    0.0%    0.0%    0.0%    0.0%    0.0%    0.0%    0.0%    0.0%    0.0%    0.0%    0.0%    0.0%    0.0%    0.0%    0.0%    0.0%    0.0%    0.0%    0.0%    0.0%    0.0%    0.0%    0.0%    0.0%    0.0%    0.0%    0.0%    0.0%    0.0%    0.0%    0.0%    0.0%    0.0%    0.0%    0.0%    0.0%    0.0%    0.0%    0.0%    0.0%    0.0%    0.0%    0.0%    0.0%    0.0%    0.0%    0.0%    0.0%    0.0%    0.0%    0.0%    0.0%    0.0%    0.0%    0.0%    0.0%    0.0%    0.0%    0.0%    0.0%    0.0%    0.0%    0.0%    0.0%    0.0%    0.0%    0.0%    0.0%    0.0%    0.0%    0.0%    0.0%    0.0%    0.0%    0.0%    0.0%    0.0%    0.0%    0.0%    0.0%    0.0%    0.0%    0.0%    0.0%    0.0%    0.0%    0.0%    0.0%    0.0%    0.0%    0.0%    0.0%    0.0%    0.0%    0.0%    0.0%    0.0%    0.0%    0.0%    0.0%    0.0%    0.0%    0.0%    0.0%    0.0%    0.0%    0.0%    0.0%    0.0%    0.0%    0.0%    0.0%    0.0%    0.0%    0.0%    0.0%    0.0%        k__Bacteria;p__Verrucomicrobia;c__; o__      34    0.0%    0.0%    0.0%    0.0%    0.0%    0.0%    0.0%    0.0%    0.0%    0.0%    0.0%    0.0%    0.0%    0.0%    0.0%    0.0%    0.0%    0.0%    0.0%    0.0%    0.0%    0.0%    0.0%    0.0%    0.0%    0.0%    0.0%    0.0%    0.0%    0.0%    0.0%    0.0%    0.0%    0.0%    0.0%    0.0%    0.0%    0.0%    0.0%    0.0%    0.0%    0.0%    0.0%    0.0%    0.0%    0.0%    0.0%    0.0%    0.0%    0.0%    0.0%    0.0%    0.0%    0.0%    0.0%    0.0%    0.0%    0.0%    0.0%    0.0%    0.0%    0.0%    0.0%    0.0%    0.0%    0.0%    0.0%    0.0%    0.0%    0.0%    0.0%    0.0%    0.0%    0.0%    0.0%    0.0%    0.0%    0.0%    0.0%    0.0%    0.0%    0.0%    0.0%    0.0%    0.0%    0.0%    0.0%    0.0%    0.0%    0.0%    0.0%    0.0%    0.0%    0.0%    0.0%    0.0%    0.0%    0.0%    0.0%    0.0%    0.0%    0.0%    0.0%    0.0%    0.0%    0.0%    0.0%    0.0%    0.0%    0.0%    0.0%    0.0%    0.0%    0.0%    0.0%    0.0%    0.0%    0.0%    0.0%    0.0%    0.0%    0.0%    0.0%    0.0%    0.0%    0.0%    0.0%    0.0%    0.0%    0.0%    0.0%    0.0%    0.0%    0.0%    0.0%    0.0%    0.0%    0.0%    0.0%    0.0%    0.0%    0.0%    0.0%    0.0%    0.0%    0.0%    0.0%    0.0%    0.0%    0.0%    0.0%    0.0%    0.0%    0.0%    0.0%    0.0%    0.0%    0.0%    0.0%    0.0%    0.0%    0.0%    0.0%    0.0%    0.0%    0.0%    0.0%    0.0%    0.0%    0.0%    0.0%    0.0%    0.0%    0.0%    0.0%    0.0%    0.0%    0.0%    0.0%    0.0%    0.0%    0.0%    0.0%    0.0%    0.0%    0.0%    0.0%    0.0%    0.0%    0.0%    0.0%    0.0%    0.0%    0.0%    0.0%    0.0%    0.0%    0.0%    0.0%    0.0%    0.0%    0.0%    0.0%    0.0%    0.0%    0.0%    0.0%    0.0%    0.0%    0.0%    0.0%    0.0%    0.0%    0.0%    0.0%    0.0%    0.0%    0.0%    0.0%    0.0%    0.0%    0.0%    0.0%    0.0%    0.0%    0.0%    0.0%    0.0%    0.0%    0.0%    0.0%    0.0%    0.0%    0.0%    0.0%    0.0%    0.0%    0.0%    0.0%    0.0%    0.0%    0.0%    0.0%    0.0%    0.0%    0.0%    0.0%    0.0%    0.0%    0.0%    0.0%    0.0%    0.0%    0.0%    0.0%    0.0%    0.0%    0.0%    0.0%    0.0%    0.0%    0.0%    0.0%    0.0%    0.0%    0.0%    0.0%    0.0%    0.0%    0.0%    0.0%    0.0%    0.0%    0.0%    0.0%    0.0%    0.0%    0.0%    0.0%    0.0%    0.0%    0.0%    0.0%    0.0%    0.0%    0.0%    0.0%    0.0%    0.0%    0.0%    0.0%    0.0%    0.0%    0.0%    0.0%    0.0%    0.0%    0.0%    0.0%    0.0%    0.0%    0.0%    0.0%    0.0%    0.0%    0.0%    0.0%    0.0%    0.0%    0.0%    0.0%    0.0%    0.0%    0.0%    0.0%    0.0%    0.0%    0.0%    0.0%    0.0%    0.0%    0.0%    0.0%    0.0%    0.0%    0.0%    0.0%    0.0%    0.0%    0.0%    0.0%    0.0%    0.0%    0.0%    0.0%    0.0%    0.0%    0.0%    0.0%    0.0%    0.0%    0.0%    0.0%    0.0%    0.0%    0.0%    0.0%    0.0%    0.0%    0.0%    0.0%    0.0%    0.0%    0.0%    0.0%    0.0%    0.0%    0.0%        k__Bacteria;p__Verrucomicrobia;c__; o__Methylacidiphilales       4    0.0%    0.0%    0.0%    0.0%    0.0%    0.0%    0.0%    0.0%    0.0%    0.0%    0.0%    0.0%    0.0%    0.0%    0.0%    0.0%    0.0%    0.0%    0.0%    0.0%    0.0%    0.0%    0.0%    0.0%    0.0%    0.0%    0.0%    0.0%    0.0%    0.0%    0.0%    0.0%    0.0%    0.0%    0.0%    0.0%    0.0%    0.0%    0.0%    0.0%    0.0%    0.0%    0.0%    0.0%    0.0%    0.0%    0.0%    0.0%    0.0%    0.0%    0.0%    0.0%    0.0%    0.0%    0.0%    0.0%    0.0%    0.0%    0.0%    0.0%    0.0%    0.0%    0.0%    0.0%    0.0%    0.0%    0.0%    0.0%    0.0%    0.0%    0.0%    0.0%    0.0%    0.0%    0.0%    0.0%    0.0%    0.0%    0.0%    0.0%    0.0%    0.0%    0.0%    0.0%    0.0%    0.0%    0.0%    0.0%    0.0%    0.0%    0.0%    0.0%    0.0%    0.0%    0.0%    0.0%    0.0%    0.0%    0.0%    0.0%    0.0%    0.0%    0.0%    0.0%    0.0%    0.0%    0.0%    0.0%    0.0%    0.0%    0.0%    0.0%    0.0%    0.0%    0.0%    0.0%    0.0%    0.0%    0.0%    0.0%    0.0%    0.0%    0.0%    0.0%    0.0%    0.0%    0.0%    0.0%    0.0%    0.0%    0.0%    0.0%    0.0%    0.0%    0.0%    0.0%    0.0%    0.0%    0.0%    0.0%    0.0%    0.0%    0.0%    0.0%    0.0%    0.0%    0.0%    0.0%    0.0%    0.0%    0.0%    0.0%    0.0%    0.0%    0.0%    0.0%    0.0%    0.0%    0.0%    0.0%    0.0%    0.0%    0.0%    0.0%    0.0%    0.0%    0.0%    0.0%    0.0%    0.0%    0.0%    0.0%    0.0%    0.0%    0.0%    0.0%    0.0%    0.0%    0.0%    0.0%    0.0%    0.0%    0.0%    0.0%    0.0%    0.0%    0.0%    0.0%    0.0%    0.0%    0.0%    0.0%    0.0%    0.0%    0.0%    0.0%    0.0%    0.0%    0.0%    0.0%    0.0%    0.0%    0.0%    0.0%    0.0%    0.0%    0.0%    0.0%    0.0%    0.0%    0.0%    0.0%    0.0%    0.0%    0.0%    0.0%    0.0%    0.0%    0.0%    0.0%    0.0%    0.0%    0.0%    0.0%    0.0%    0.0%    0.0%    0.0%    0.0%    0.0%    0.0%    0.0%    0.0%    0.0%    0.0%    0.0%    0.0%    0.0%    0.0%    0.0%    0.0%    0.0%    0.0%    0.0%    0.0%    0.0%    0.0%    0.0%    0.0%    0.0%    0.0%    0.0%    0.0%    0.0%    0.0%    0.0%    0.0%    0.0%    0.0%    0.0%    0.0%    0.0%    0.0%    0.0%    0.0%    0.0%    0.0%    0.0%    0.0%    0.0%    0.0%    0.0%    0.0%    0.0%    0.0%    0.0%    0.0%    0.0%    0.0%    0.0%    0.0%    0.0%    0.0%    0.0%    0.0%    0.0%    0.0%    0.0%    0.0%    0.0%    0.0%    0.0%    0.0%    0.0%    0.0%    0.0%    0.0%    0.0%    0.0%    0.0%    0.0%    0.0%    0.0%    0.0%    0.0%    0.0%    0.0%    0.0%    0.0%    0.0%    0.0%    0.0%    0.0%    0.0%    0.0%    0.0%    0.0%    0.0%    0.0%    0.0%    0.0%    0.0%    0.0%    0.0%    0.0%    0.0%    0.0%    0.0%    0.0%    0.0%    0.0%    0.0%    0.0%    0.0%    0.0%    0.0%    0.0%    0.0%    0.0%    0.0%    0.0%    0.0%    0.0%    0.0%    0.0%    0.0%    0.0%    0.0%    0.0%    0.0%    0.0%    0.0%    0.0%    0.0%    0.0%    0.0%    0.0%    0.0%        k__Bacteria;p__Verrucomicrobia;c__Opitutae; o__     397    0.0%    0.0%    0.0%    0.0%    0.0%    0.0%    0.0%    0.0%    0.0%    0.0%    0.0%    0.0%    0.0%    0.0%    0.0%    0.0%    0.0%    0.0%    0.0%    0.0%    0.0%    0.0%    0.0%    0.0%    0.0%    0.0%    0.0%    0.0%    0.0%    0.0%    0.0%    0.0%    0.0%    0.0%    0.0%    0.0%    0.0%    0.0%    0.0%    0.0%    0.0%    0.0%    0.0%    0.0%    0.0%    0.0%    0.0%    0.0%    0.0%    0.0%    0.0%    0.0%    0.0%    0.0%    0.0%    0.0%    0.0%    0.0%    0.0%    0.0%    0.0%    0.0%    0.0%    0.0%    0.0%    0.0%    0.0%    0.0%    0.0%    0.0%    0.0%    0.0%    0.0%    0.0%    0.0%    0.0%    0.0%    0.0%    0.0%    0.0%    0.0%    0.0%    0.0%    0.0%    0.0%    0.0%    0.0%    0.0%    0.0%    0.0%    0.0%    0.0%    0.0%    0.0%    0.0%    0.0%    0.0%    0.0%    0.0%    0.0%    0.0%    0.0%    0.0%    0.0%    0.0%    0.0%    0.0%    0.0%    0.0%    0.0%    0.0%    0.0%    0.0%    0.0%    0.0%    0.0%    0.0%    0.0%    0.0%    0.0%    0.0%    0.0%    0.0%    0.0%    0.0%    0.1%    0.0%    0.0%    0.0%    0.0%    0.0%    0.0%    0.0%    0.0%    0.0%    0.0%    0.0%    0.0%    0.0%    0.0%    0.0%    0.0%    0.0%    0.0%    0.0%    0.0%    0.0%    0.0%    0.0%    0.0%    0.0%    0.0%    0.0%    0.0%    0.0%    0.0%    0.0%    0.0%    0.0%    0.0%    0.0%    0.0%    0.0%    0.0%    0.0%    0.0%    0.0%    0.0%    0.0%    0.0%    0.0%    0.0%    0.0%    0.0%    0.0%    0.0%    0.0%    0.0%    0.0%    0.0%    0.0%    0.0%    0.0%    0.0%    0.0%    0.0%    0.0%    0.0%    0.0%    0.0%    0.0%    0.0%    0.0%    0.0%    0.0%    0.0%    0.0%    0.0%    0.0%    0.0%    0.0%    0.0%    0.0%    0.0%    0.0%    0.0%    0.0%    0.0%    0.0%    0.0%    0.0%    0.0%    0.0%    0.0%    0.0%    0.0%    0.0%    0.0%    0.0%    0.0%    0.0%    0.0%    0.0%    0.0%    0.0%    0.0%    0.0%    0.0%    0.0%    0.0%    0.0%    0.0%    0.0%    0.0%    0.0%    0.0%    0.0%    0.0%    0.0%    0.0%    0.0%    0.0%    0.0%    0.0%    0.0%    0.0%    0.0%    0.0%    0.0%    0.0%    0.0%    0.0%    0.0%    0.0%    0.0%    0.0%    0.0%    0.0%    0.0%    0.0%    0.0%    0.0%    0.0%    0.0%    0.0%    0.0%    0.0%    0.0%    0.0%    0.0%    0.0%    0.0%    0.0%    0.0%    0.0%    0.0%    0.0%    0.0%    0.0%    0.0%    0.0%    0.0%    0.0%    0.0%    0.0%    0.0%    0.0%    0.0%    0.0%    0.0%    0.0%    0.0%    0.0%    0.0%    0.0%    0.0%    0.0%    0.0%    0.0%    0.0%    0.0%    0.0%    0.0%    0.0%    0.0%    0.0%    0.0%    0.0%    0.0%    0.0%    0.0%    0.0%    0.0%    0.0%    0.0%    0.0%    0.0%    0.0%    0.0%    0.0%    0.0%    0.0%    0.0%    0.0%    0.0%    0.0%    0.0%    0.0%    0.0%    0.0%    0.0%    0.0%    0.0%    0.0%    0.0%    0.0%    0.0%    0.0%    0.0%    0.0%    0.0%    0.0%    0.0%    0.0%    0.0%    0.0%    0.0%    0.0%    0.0%    0.0%    0.0%    0.0%    0.0%    0.0%    0.0%    0.0%    0.0%    0.0%        k__Bacteria;p__Verrucomicrobia;c__Opitutae; o__Puniceicoccales       6    0.0%    0.0%    0.0%    0.0%    0.0%    0.0%    0.0%    0.0%    0.0%    0.0%    0.0%    0.0%    0.0%    0.0%    0.0%    0.0%    0.0%    0.0%    0.0%    0.0%    0.0%    0.0%    0.0%    0.0%    0.0%    0.0%    0.0%    0.0%    0.0%    0.0%    0.0%    0.0%    0.0%    0.0%    0.0%    0.0%    0.0%    0.0%    0.0%    0.0%    0.0%    0.0%    0.0%    0.0%    0.0%    0.0%    0.0%    0.0%    0.0%    0.0%    0.0%    0.0%    0.0%    0.0%    0.0%    0.0%    0.0%    0.0%    0.0%    0.0%    0.0%    0.0%    0.0%    0.0%    0.0%    0.0%    0.0%    0.0%    0.0%    0.0%    0.0%    0.0%    0.0%    0.0%    0.0%    0.0%    0.0%    0.0%    0.0%    0.0%    0.0%    0.0%    0.0%    0.0%    0.0%    0.0%    0.0%    0.0%    0.0%    0.0%    0.0%    0.0%    0.0%    0.0%    0.0%    0.0%    0.0%    0.0%    0.0%    0.0%    0.0%    0.0%    0.0%    0.0%    0.0%    0.0%    0.0%    0.0%    0.0%    0.0%    0.0%    0.0%    0.0%    0.0%    0.0%    0.0%    0.0%    0.0%    0.0%    0.0%    0.0%    0.0%    0.0%    0.0%    0.0%    0.0%    0.0%    0.0%    0.0%    0.0%    0.0%    0.0%    0.0%    0.0%    0.0%    0.0%    0.0%    0.0%    0.0%    0.0%    0.0%    0.0%    0.0%    0.0%    0.0%    0.0%    0.0%    0.0%    0.0%    0.0%    0.0%    0.0%    0.0%    0.0%    0.0%    0.0%    0.0%    0.0%    0.0%    0.0%    0.0%    0.0%    0.0%    0.0%    0.0%    0.0%    0.0%    0.0%    0.0%    0.0%    0.0%    0.0%    0.0%    0.0%    0.0%    0.0%    0.0%    0.0%    0.0%    0.0%    0.0%    0.0%    0.0%    0.0%    0.0%    0.0%    0.0%    0.0%    0.0%    0.0%    0.0%    0.0%    0.0%    0.0%    0.0%    0.0%    0.0%    0.0%    0.0%    0.0%    0.0%    0.0%    0.0%    0.0%    0.0%    0.0%    0.0%    0.0%    0.0%    0.0%    0.0%    0.0%    0.0%    0.0%    0.0%    0.0%    0.0%    0.0%    0.0%    0.0%    0.0%    0.0%    0.0%    0.0%    0.0%    0.0%    0.0%    0.0%    0.0%    0.0%    0.0%    0.0%    0.0%    0.0%    0.0%    0.0%    0.0%    0.0%    0.0%    0.0%    0.0%    0.0%    0.0%    0.0%    0.0%    0.0%    0.0%    0.0%    0.0%    0.0%    0.0%    0.0%    0.0%    0.0%    0.0%    0.0%    0.0%    0.0%    0.0%    0.0%    0.0%    0.0%    0.0%    0.0%    0.0%    0.0%    0.0%    0.0%    0.0%    0.0%    0.0%    0.0%    0.0%    0.0%    0.0%    0.0%    0.0%    0.0%    0.0%    0.0%    0.0%    0.0%    0.0%    0.0%    0.0%    0.0%    0.0%    0.0%    0.0%    0.0%    0.0%    0.0%    0.0%    0.0%    0.0%    0.0%    0.0%    0.0%    0.0%    0.0%    0.0%    0.0%    0.0%    0.0%    0.0%    0.0%    0.0%    0.0%    0.0%    0.0%    0.0%    0.1%    0.0%    0.0%    0.0%    0.0%    0.0%    0.0%    0.0%    0.0%    0.0%    0.0%    0.0%    0.0%    0.0%    0.0%    0.0%    0.0%    0.0%    0.0%    0.0%    0.0%    0.0%    0.0%    0.0%    0.0%    0.0%    0.0%    0.0%    0.0%    0.0%    0.0%    0.0%    0.0%    0.0%    0.0%    0.0%    0.0%    0.0%    0.0%    0.0%    0.0%    0.0%    0.0%    0.0%    0.0%    0.0%    0.0%        k__Bacteria;p__Verrucomicrobia;c__Spartobacteria; o__    2258    0.0%    0.0%    0.0%    0.0%    0.0%    0.0%    0.0%    0.0%    0.0%    0.0%    0.0%    0.0%    0.0%    0.0%    0.0%    0.0%    0.0%    0.0%    0.0%    0.0%    0.0%    0.0%    0.0%    0.0%    0.0%    0.0%    0.0%    0.0%    0.0%    0.0%    0.0%    0.0%    0.0%    0.0%    0.0%    0.0%    0.0%    0.0%    0.0%    0.0%    0.0%    0.0%    0.0%    0.0%    0.0%    0.0%    0.0%    0.0%    0.0%    0.0%    0.0%    0.0%    0.0%    0.0%    0.0%    0.0%    0.0%    0.0%    0.0%    0.0%    0.0%    0.0%    0.0%    0.1%    0.0%    0.0%    0.0%    0.0%    0.0%    0.0%    0.0%    0.0%    0.0%    0.0%    0.0%    0.0%    0.0%    0.0%    0.0%    0.0%    0.0%    0.0%    0.0%    0.0%    0.0%    0.0%    0.0%    0.0%    0.0%    0.0%    0.0%    0.0%    0.0%    0.0%    0.0%    0.0%    0.0%    0.0%    0.1%    0.0%    0.0%    0.0%    0.0%    0.0%    0.0%    0.0%    0.0%    0.0%    0.0%    0.0%    0.0%    0.0%    0.0%    0.0%    0.0%    0.0%    0.0%    0.0%    0.0%    0.1%    0.0%    0.0%    0.0%    0.0%    0.0%    0.1%    0.1%    0.0%    0.0%    0.1%    0.0%    0.0%    0.0%    0.0%    0.1%    0.0%    0.0%    0.0%    0.0%    0.0%    0.0%    0.0%    0.0%    0.0%    0.0%    0.0%    0.0%    0.0%    0.0%    0.0%    0.0%    0.0%    0.0%    0.0%    0.0%    0.0%    0.0%    0.0%    0.0%    0.0%    0.0%    0.0%    0.0%    0.0%    0.0%    0.0%    0.0%    0.0%    0.0%    0.0%    0.1%    0.0%    0.0%    0.0%    0.0%    0.0%    0.0%    0.0%    0.0%    0.0%    0.0%    0.0%    0.0%    0.0%    0.0%    0.0%    0.0%    0.0%    0.0%    0.0%    0.0%    0.0%    0.0%    0.0%    0.0%    0.0%    0.0%    0.0%    0.0%    0.0%    0.0%    0.0%    0.0%    0.0%    0.0%    0.0%    0.0%    0.0%    0.1%    0.0%    0.0%    0.0%    0.1%    0.0%    0.0%    0.1%    0.0%    0.0%    0.0%    0.0%    0.0%    0.0%    0.0%    0.0%    0.0%    0.0%    0.0%    0.0%    0.0%    0.0%    0.0%    0.0%    0.0%    0.0%    0.0%    0.0%    0.0%    0.0%    0.0%    0.0%    0.0%    0.0%    0.0%    0.0%    0.0%    0.0%    0.1%    0.0%    0.0%    0.0%    0.0%    0.0%    0.0%    0.0%    0.0%    0.0%    0.0%    0.0%    0.0%    0.0%    0.0%    0.0%    0.0%    0.0%    0.0%    0.0%    0.0%    0.0%    0.0%    0.0%    0.0%    0.0%    0.0%    0.0%    0.0%    0.0%    0.0%    0.0%    0.0%    0.0%    0.0%    0.0%    0.0%    0.0%    0.0%    0.0%    0.0%    0.1%    0.0%    0.2%    0.0%    0.0%    0.0%    0.0%    0.0%    0.0%    0.0%    0.0%    0.0%    0.1%    0.0%    0.0%    0.0%    0.0%    0.0%    0.0%    0.0%    0.0%    0.0%    0.0%    0.0%    0.0%    0.0%    0.0%    0.0%    0.0%    0.0%    0.0%    0.0%    0.0%    0.0%    0.0%    0.0%    0.0%    0.0%    0.0%    0.0%    0.0%    0.0%    0.0%    0.0%    0.0%    0.0%    0.0%    0.0%    0.0%    0.0%    0.0%    0.0%    0.0%    0.0%    0.0%    0.0%    0.0%    0.0%    0.0%    0.0%    0.0%    0.0%    0.0%    0.0%    0.0%    0.0%    0.0%    0.0%    0.0%    0.0%    0.0%        k__Bacteria;p__Verrucomicrobia;c__TP21; o__       0    0.0%    0.0%    0.0%    0.0%    0.0%    0.0%    0.0%    0.0%    0.0%    0.0%    0.0%    0.0%    0.0%    0.0%    0.0%    0.0%    0.0%    0.0%    0.0%    0.0%    0.0%    0.0%    0.0%    0.0%    0.0%    0.0%    0.0%    0.0%    0.0%    0.0%    0.0%    0.0%    0.0%    0.0%    0.0%    0.0%    0.0%    0.0%    0.0%    0.0%    0.0%    0.0%    0.0%    0.0%    0.0%    0.0%    0.0%    0.0%    0.0%    0.0%    0.0%    0.0%    0.0%    0.0%    0.0%    0.0%    0.0%    0.0%    0.0%    0.0%    0.0%    0.0%    0.0%    0.0%    0.0%    0.0%    0.0%    0.0%    0.0%    0.0%    0.0%    0.0%    0.0%    0.0%    0.0%    0.0%    0.0%    0.0%    0.0%    0.0%    0.0%    0.0%    0.0%    0.0%    0.0%    0.0%    0.0%    0.0%    0.0%    0.0%    0.0%    0.0%    0.0%    0.0%    0.0%    0.0%    0.0%    0.0%    0.0%    0.0%    0.0%    0.0%    0.0%    0.0%    0.0%    0.0%    0.0%    0.0%    0.0%    0.0%    0.0%    0.0%    0.0%    0.0%    0.0%    0.0%    0.0%    0.0%    0.0%    0.0%    0.0%    0.0%    0.0%    0.0%    0.0%    0.0%    0.0%    0.0%    0.0%    0.0%    0.0%    0.0%    0.0%    0.0%    0.0%    0.0%    0.0%    0.0%    0.0%    0.0%    0.0%    0.0%    0.0%    0.0%    0.0%    0.0%    0.0%    0.0%    0.0%    0.0%    0.0%    0.0%    0.0%    0.0%    0.0%    0.0%    0.0%    0.0%    0.0%    0.0%    0.0%    0.0%    0.0%    0.0%    0.0%    0.0%    0.0%    0.0%    0.0%    0.0%    0.0%    0.0%    0.0%    0.0%    0.0%    0.0%    0.0%    0.0%    0.0%    0.0%    0.0%    0.0%    0.0%    0.0%    0.0%    0.0%    0.0%    0.0%    0.0%    0.0%    0.0%    0.0%    0.0%    0.0%    0.0%    0.0%    0.0%    0.0%    0.0%    0.0%    0.0%    0.0%    0.0%    0.0%    0.0%    0.0%    0.0%    0.0%    0.0%    0.0%    0.0%    0.0%    0.0%    0.0%    0.0%    0.0%    0.0%    0.0%    0.0%    0.0%    0.0%    0.0%    0.0%    0.0%    0.0%    0.0%    0.0%    0.0%    0.0%    0.0%    0.0%    0.0%    0.0%    0.0%    0.0%    0.0%    0.0%    0.0%    0.0%    0.0%    0.0%    0.0%    0.0%    0.0%    0.0%    0.0%    0.0%    0.0%    0.0%    0.0%    0.0%    0.0%    0.0%    0.0%    0.0%    0.0%    0.0%    0.0%    0.0%    0.0%    0.0%    0.0%    0.0%    0.0%    0.0%    0.0%    0.0%    0.0%    0.0%    0.0%    0.0%    0.0%    0.0%    0.0%    0.0%    0.0%    0.0%    0.0%    0.0%    0.0%    0.0%    0.0%    0.0%    0.0%    0.0%    0.0%    0.0%    0.0%    0.0%    0.0%    0.0%    0.0%    0.0%    0.0%    0.0%    0.0%    0.0%    0.0%    0.0%    0.0%    0.0%    0.0%    0.0%    0.0%    0.0%    0.0%    0.0%    0.0%    0.0%    0.0%    0.0%    0.0%    0.0%    0.0%    0.0%    0.0%    0.0%    0.0%    0.0%    0.0%    0.0%    0.0%    0.0%    0.0%    0.0%    0.0%    0.0%    0.0%    0.0%    0.0%    0.0%    0.0%    0.0%    0.0%    0.0%    0.0%    0.0%    0.0%    0.0%    0.0%    0.0%    0.0%    0.0%    0.0%    0.0%    0.0%    0.0%    0.0%    0.0%    0.0%    0.0%    0.0%    0.0%    0.0%    0.0%    0.0%    0.0%    0.0%        k__Bacteria;p__Verrucomicrobia;c__Verrucomicrobiae; o__Verrucomicrobiales    5315    0.0%    0.0%    0.0%    0.0%    0.0%    0.0%    0.0%    0.0%    0.0%    0.0%    0.0%    0.1%    0.0%    0.0%    0.0%    0.0%    0.0%    0.0%    0.0%    0.0%    0.0%    0.0%    0.0%    0.4%    0.0%    0.0%    0.0%    0.0%    0.0%    0.0%    0.0%    0.0%    0.0%    0.0%    0.0%    0.0%    0.1%    0.0%    0.0%    0.0%    0.0%    0.0%    0.0%    0.0%    0.0%    0.0%    0.0%    0.0%    0.0%    0.0%    0.0%    0.0%    0.0%    0.0%    0.1%    0.1%    0.1%    0.0%    0.0%    0.1%    0.0%    0.0%    0.0%    0.1%    0.0%    0.1%    0.1%    0.1%    0.0%    0.0%    0.0%    0.0%    0.0%    0.0%    0.1%    0.0%    0.0%    0.1%    0.0%    0.0%    0.0%    0.0%    0.0%    0.0%    0.0%    0.0%    0.0%    0.0%    0.0%    0.0%    0.0%    0.0%    0.0%    0.0%    0.0%    0.0%    0.1%    0.0%    0.0%    0.0%    0.1%    0.0%    0.0%    0.0%    0.0%    0.0%    0.1%    0.0%    0.0%    0.0%    0.0%    0.0%    0.0%    0.0%    0.0%    0.0%    0.0%    0.0%    0.0%    0.5%    0.0%    0.0%    0.1%    0.0%    0.0%    0.6%    0.1%    0.0%    0.1%    0.2%    0.1%    0.0%    0.0%    0.0%    0.1%    0.0%    0.0%    0.0%    0.0%    0.0%    0.0%    0.0%    0.1%    0.0%    0.1%    0.0%    0.0%    0.0%    0.0%    0.0%    0.0%    0.0%    0.1%    0.0%    0.0%    0.0%    0.1%    0.0%    0.0%    0.1%    0.1%    0.1%    0.1%    0.0%    0.0%    0.0%    0.0%    0.0%    0.0%    0.0%    0.1%    0.0%    0.0%    0.0%    0.0%    0.0%    0.0%    0.0%    0.0%    0.0%    0.0%    0.0%    0.0%    0.0%    0.0%    0.0%    0.0%    0.0%    0.0%    0.0%    0.0%    0.0%    0.0%    0.0%    0.0%    0.0%    0.0%    0.0%    0.0%    0.1%    0.0%    0.0%    0.0%    0.1%    0.1%    0.0%    0.0%    0.1%    0.2%    0.0%    0.1%    0.0%    0.2%    0.0%    0.0%    0.0%    0.0%    0.0%    0.0%    0.0%    0.0%    0.0%    0.0%    0.0%    0.0%    0.1%    0.0%    0.0%    0.0%    0.0%    0.0%    0.0%    0.1%    0.0%    0.0%    0.0%    0.1%    0.0%    0.0%    0.0%    0.1%    0.0%    0.0%    0.0%    0.0%    0.0%    0.0%    0.0%    0.0%    0.0%    0.1%    0.1%    0.1%    0.0%    0.0%    0.0%    0.0%    0.0%    0.0%    0.0%    0.0%    0.0%    0.0%    0.0%    0.0%    0.0%    0.0%    0.0%    0.0%    0.0%    0.0%    0.1%    0.1%    0.0%    0.0%    0.0%    0.0%    0.0%    0.0%    0.0%    0.0%    0.0%    0.0%    0.0%    0.0%    0.0%    0.0%    0.2%    0.0%    0.0%    0.0%    0.0%    0.0%    0.1%    0.0%    0.0%    0.1%    0.0%    0.0%    0.0%    0.0%    0.0%    0.0%    0.0%    0.0%    0.0%    0.0%    0.0%    0.0%    0.0%    0.0%    0.0%    0.0%    0.0%    0.0%    0.1%    0.0%    0.0%    0.0%    0.1%    0.0%    0.0%    0.0%    0.0%    0.0%    0.0%    0.0%    0.0%    0.0%    0.0%    0.1%    0.0%    0.0%    0.0%    0.1%    0.0%    0.0%    0.0%    0.1%    0.0%    0.0%    0.0%    0.0%    0.0%    0.0%    0.0%    0.0%    0.0%    0.0%    0.0%    0.0%    0.0%    0.0%    0.0%    0.0%    0.2%    0.5%    0.0%        k__Bacteria;p__WPS-2;c__; o__     101    0.0%    0.0%    0.0%    0.0%    0.0%    0.0%    0.0%    0.0%    0.0%    0.0%    0.0%    0.0%    0.0%    0.0%    0.0%    0.0%    0.0%    0.0%    0.0%    0.0%    0.0%    0.0%    0.0%    0.0%    0.0%    0.0%    0.0%    0.0%    0.0%    0.0%    0.0%    0.0%    0.0%    0.0%    0.0%    0.0%    0.0%    0.0%    0.0%    0.0%    0.0%    0.0%    0.0%    0.0%    0.0%    0.0%    0.0%    0.0%    0.0%    0.0%    0.0%    0.0%    0.0%    0.0%    0.0%    0.0%    0.0%    0.0%    0.0%    0.0%    0.0%    0.0%    0.0%    0.0%    0.0%    0.0%    0.0%    0.0%    0.0%    0.0%    0.0%    0.0%    0.0%    0.0%    0.0%    0.0%    0.0%    0.0%    0.0%    0.0%    0.0%    0.0%    0.0%    0.0%    0.0%    0.0%    0.0%    0.0%    0.0%    0.0%    0.0%    0.0%    0.0%    0.0%    0.0%    0.0%    0.0%    0.0%    0.0%    0.0%    0.0%    0.0%    0.0%    0.0%    0.0%    0.0%    0.0%    0.0%    0.0%    0.0%    0.0%    0.0%    0.0%    0.0%    0.0%    0.0%    0.0%    0.0%    0.0%    0.0%    0.0%    0.0%    0.0%    0.0%    0.0%    0.0%    0.0%    0.0%    0.0%    0.0%    0.0%    0.0%    0.0%    0.0%    0.0%    0.0%    0.0%    0.0%    0.0%    0.0%    0.0%    0.0%    0.0%    0.0%    0.0%    0.0%    0.0%    0.0%    0.0%    0.0%    0.0%    0.0%    0.0%    0.0%    0.0%    0.0%    0.0%    0.0%    0.0%    0.0%    0.0%    0.0%    0.0%    0.0%    0.0%    0.0%    0.0%    0.0%    0.0%    0.0%    0.0%    0.0%    0.0%    0.0%    0.0%    0.0%    0.0%    0.0%    0.0%    0.0%    0.0%    0.0%    0.0%    0.0%    0.0%    0.0%    0.0%    0.0%    0.0%    0.0%    0.0%    0.0%    0.0%    0.0%    0.0%    0.0%    0.0%    0.0%    0.0%    0.0%    0.0%    0.0%    0.0%    0.0%    0.0%    0.0%    0.0%    0.0%    0.0%    0.0%    0.0%    0.0%    0.0%    0.0%    0.0%    0.0%    0.0%    0.0%    0.0%    0.0%    0.0%    0.0%    0.0%    0.0%    0.0%    0.0%    0.0%    0.0%    0.0%    0.0%    0.0%    0.0%    0.0%    0.0%    0.0%    0.0%    0.0%    0.0%    0.0%    0.0%    0.0%    0.0%    0.0%    0.0%    0.0%    0.0%    0.0%    0.0%    0.0%    0.0%    0.0%    0.0%    0.0%    0.0%    0.0%    0.0%    0.0%    0.0%    0.0%    0.0%    0.0%    0.0%    0.0%    0.0%    0.0%    0.0%    0.0%    0.0%    0.0%    0.0%    0.0%    0.0%    0.0%    0.0%    0.0%    0.0%    0.0%    0.0%    0.0%    0.0%    0.0%    0.0%    0.0%    0.0%    0.0%    0.0%    0.0%    0.0%    0.0%    0.0%    0.0%    0.0%    0.0%    0.0%    0.0%    0.0%    0.0%    0.0%    0.0%    0.0%    0.0%    0.0%    0.0%    0.0%    0.0%    0.0%    0.0%    0.0%    0.0%    0.0%    0.0%    0.0%    0.0%    0.0%    0.0%    0.0%    0.0%    0.0%    0.0%    0.0%    0.0%    0.0%    0.0%    0.0%    0.0%    0.0%    0.0%    0.0%    0.0%    0.0%    0.0%    0.0%    0.0%    0.0%    0.0%    0.0%    0.0%    0.0%    0.0%    0.0%    0.0%    0.0%    0.0%    0.0%    0.0%    0.0%    0.0%    0.0%    0.0%    0.0%    0.0%    0.0%    0.0%    0.0%    0.0%    0.0%    0.0%    0.0%        k__Bacteria;p__WS3;c__PRR-12; o__      25    0.0%    0.0%    0.0%    0.0%    0.0%    0.0%    0.0%    0.0%    0.0%    0.0%    0.0%    0.0%    0.0%    0.0%    0.0%    0.0%    0.0%    0.0%    0.0%    0.0%    0.0%    0.0%    0.0%    0.0%    0.0%    0.0%    0.0%    0.0%    0.0%    0.0%    0.0%    0.0%    0.0%    0.0%    0.0%    0.0%    0.0%    0.0%    0.0%    0.0%    0.0%    0.0%    0.0%    0.0%    0.0%    0.0%    0.0%    0.0%    0.0%    0.0%    0.0%    0.0%    0.0%    0.0%    0.0%    0.0%    0.0%    0.0%    0.0%    0.0%    0.0%    0.0%    0.0%    0.0%    0.0%    0.0%    0.0%    0.0%    0.0%    0.0%    0.0%    0.0%    0.0%    0.0%    0.0%    0.0%    0.0%    0.0%    0.0%    0.0%    0.0%    0.0%    0.0%    0.0%    0.0%    0.0%    0.0%    0.0%    0.0%    0.0%    0.0%    0.0%    0.0%    0.0%    0.0%    0.0%    0.0%    0.0%    0.0%    0.0%    0.0%    0.0%    0.0%    0.0%    0.0%    0.0%    0.0%    0.0%    0.0%    0.0%    0.0%    0.0%    0.0%    0.0%    0.0%    0.0%    0.0%    0.0%    0.0%    0.0%    0.0%    0.0%    0.0%    0.0%    0.0%    0.0%    0.0%    0.0%    0.0%    0.0%    0.0%    0.0%    0.0%    0.0%    0.0%    0.0%    0.0%    0.0%    0.0%    0.0%    0.0%    0.0%    0.0%    0.0%    0.0%    0.0%    0.0%    0.0%    0.0%    0.0%    0.0%    0.0%    0.0%    0.0%    0.0%    0.0%    0.0%    0.0%    0.0%    0.0%    0.0%    0.0%    0.0%    0.0%    0.0%    0.0%    0.0%    0.0%    0.0%    0.0%    0.0%    0.0%    0.0%    0.0%    0.0%    0.0%    0.0%    0.0%    0.0%    0.0%    0.0%    0.0%    0.0%    0.0%    0.0%    0.0%    0.0%    0.0%    0.0%    0.0%    0.0%    0.0%    0.0%    0.0%    0.0%    0.0%    0.0%    0.0%    0.0%    0.0%    0.0%    0.0%    0.0%    0.0%    0.0%    0.0%    0.0%    0.0%    0.0%    0.0%    0.0%    0.0%    0.0%    0.0%    0.0%    0.0%    0.0%    0.0%    0.0%    0.0%    0.0%    0.0%    0.0%    0.0%    0.0%    0.0%    0.0%    0.0%    0.0%    0.0%    0.0%    0.0%    0.0%    0.0%    0.0%    0.0%    0.0%    0.0%    0.0%    0.0%    0.0%    0.0%    0.0%    0.0%    0.0%    0.0%    0.0%    0.0%    0.0%    0.0%    0.0%    0.0%    0.0%    0.0%    0.0%    0.0%    0.0%    0.0%    0.0%    0.0%    0.0%    0.0%    0.0%    0.0%    0.0%    0.0%    0.0%    0.0%    0.0%    0.0%    0.0%    0.0%    0.0%    0.0%    0.0%    0.0%    0.0%    0.0%    0.0%    0.0%    0.0%    0.0%    0.0%    0.0%    0.0%    0.0%    0.0%    0.0%    0.0%    0.0%    0.0%    0.0%    0.0%    0.0%    0.0%    0.0%    0.0%    0.0%    0.0%    0.0%    0.0%    0.0%    0.0%    0.0%    0.0%    0.0%    0.0%    0.0%    0.0%    0.0%    0.0%    0.0%    0.0%    0.0%    0.0%    0.0%    0.0%    0.0%    0.0%    0.0%    0.0%    0.0%    0.0%    0.0%    0.0%    0.0%    0.0%    0.0%    0.0%    0.0%    0.0%    0.0%    0.0%    0.0%    0.0%    0.0%    0.0%    0.0%    0.0%    0.0%    0.0%    0.0%    0.0%    0.0%    0.0%    0.0%    0.0%    0.0%    0.0%    0.0%    0.0%    0.0%    0.0%    0.0%    0.0%    0.0%    0.0%    0.0%        k__Bacteria;p__WS3;c__PRR-12; o__LCP-67       1    0.0%    0.0%    0.0%    0.0%    0.0%    0.0%    0.0%    0.0%    0.0%    0.0%    0.0%    0.0%    0.0%    0.0%    0.0%    0.0%    0.0%    0.0%    0.0%    0.0%    0.0%    0.0%    0.0%    0.0%    0.0%    0.0%    0.0%    0.0%    0.0%    0.0%    0.0%    0.0%    0.0%    0.0%    0.0%    0.0%    0.0%    0.0%    0.0%    0.0%    0.0%    0.0%    0.0%    0.0%    0.0%    0.0%    0.0%    0.0%    0.0%    0.0%    0.0%    0.0%    0.0%    0.0%    0.0%    0.0%    0.0%    0.0%    0.0%    0.0%    0.0%    0.0%    0.0%    0.0%    0.0%    0.0%    0.0%    0.0%    0.0%    0.0%    0.0%    0.0%    0.0%    0.0%    0.0%    0.0%    0.0%    0.0%    0.0%    0.0%    0.0%    0.0%    0.0%    0.0%    0.0%    0.0%    0.0%    0.0%    0.0%    0.0%    0.0%    0.0%    0.0%    0.0%    0.0%    0.0%    0.0%    0.0%    0.0%    0.0%    0.0%    0.0%    0.0%    0.0%    0.0%    0.0%    0.0%    0.0%    0.0%    0.0%    0.0%    0.0%    0.0%    0.0%    0.0%    0.0%    0.0%    0.0%    0.0%    0.0%    0.0%    0.0%    0.0%    0.0%    0.0%    0.0%    0.0%    0.0%    0.0%    0.0%    0.0%    0.0%    0.0%    0.0%    0.0%    0.0%    0.0%    0.0%    0.0%    0.0%    0.0%    0.0%    0.0%    0.0%    0.0%    0.0%    0.0%    0.0%    0.0%    0.0%    0.0%    0.0%    0.0%    0.0%    0.0%    0.0%    0.0%    0.0%    0.0%    0.0%    0.0%    0.0%    0.0%    0.0%    0.0%    0.0%    0.0%    0.0%    0.0%    0.0%    0.0%    0.0%    0.0%    0.0%    0.0%    0.0%    0.0%    0.0%    0.0%    0.0%    0.0%    0.0%    0.0%    0.0%    0.0%    0.0%    0.0%    0.0%    0.0%    0.0%    0.0%    0.0%    0.0%    0.0%    0.0%    0.0%    0.0%    0.0%    0.0%    0.0%    0.0%    0.0%    0.0%    0.0%    0.0%    0.0%    0.0%    0.0%    0.0%    0.0%    0.0%    0.0%    0.0%    0.0%    0.0%    0.0%    0.0%    0.0%    0.0%    0.0%    0.0%    0.0%    0.0%    0.0%    0.0%    0.0%    0.0%    0.0%    0.0%    0.0%    0.0%    0.0%    0.0%    0.0%    0.0%    0.0%    0.0%    0.0%    0.0%    0.0%    0.0%    0.0%    0.0%    0.0%    0.0%    0.0%    0.0%    0.0%    0.0%    0.0%    0.0%    0.0%    0.0%    0.0%    0.0%    0.0%    0.0%    0.0%    0.0%    0.0%    0.0%    0.0%    0.0%    0.0%    0.0%    0.0%    0.0%    0.0%    0.0%    0.0%    0.0%    0.0%    0.0%    0.0%    0.0%    0.0%    0.0%    0.0%    0.0%    0.0%    0.0%    0.0%    0.0%    0.0%    0.0%    0.0%    0.0%    0.0%    0.0%    0.0%    0.0%    0.0%    0.0%    0.0%    0.0%    0.0%    0.0%    0.0%    0.0%    0.0%    0.0%    0.0%    0.0%    0.0%    0.0%    0.0%    0.0%    0.0%    0.0%    0.0%    0.0%    0.0%    0.0%    0.0%    0.0%    0.0%    0.0%    0.0%    0.0%    0.0%    0.0%    0.0%    0.0%    0.0%    0.0%    0.0%    0.0%    0.0%    0.0%    0.0%    0.0%    0.0%    0.0%    0.0%    0.0%    0.0%    0.0%    0.0%    0.0%    0.0%    0.0%    0.0%    0.0%    0.0%    0.0%    0.0%    0.0%    0.0%    0.0%    0.0%    0.0%    0.0%    0.0%    0.0%    0.0%    0.0%    0.0%    0.0%        k__Bacteria;p__WS3;c__PRR-12; o__PRR-10      16    0.0%    0.0%    0.0%    0.0%    0.0%    0.0%    0.0%    0.0%    0.0%    0.0%    0.0%    0.0%    0.0%    0.0%    0.0%    0.0%    0.0%    0.0%    0.0%    0.0%    0.0%    0.0%    0.0%    0.0%    0.0%    0.0%    0.0%    0.0%    0.0%    0.0%    0.0%    0.0%    0.0%    0.0%    0.0%    0.0%    0.0%    0.0%    0.0%    0.0%    0.0%    0.0%    0.0%    0.0%    0.0%    0.0%    0.0%    0.0%    0.0%    0.0%    0.0%    0.0%    0.0%    0.0%    0.0%    0.0%    0.0%    0.0%    0.0%    0.0%    0.0%    0.0%    0.0%    0.0%    0.0%    0.0%    0.0%    0.0%    0.0%    0.0%    0.0%    0.0%    0.0%    0.0%    0.0%    0.0%    0.0%    0.0%    0.0%    0.0%    0.0%    0.0%    0.0%    0.0%    0.0%    0.0%    0.0%    0.0%    0.0%    0.0%    0.0%    0.0%    0.0%    0.0%    0.0%    0.0%    0.0%    0.0%    0.0%    0.0%    0.0%    0.0%    0.0%    0.0%    0.0%    0.0%    0.0%    0.0%    0.0%    0.0%    0.0%    0.0%    0.0%    0.0%    0.0%    0.0%    0.0%    0.0%    0.0%    0.0%    0.0%    0.0%    0.0%    0.0%    0.0%    0.0%    0.0%    0.0%    0.0%    0.0%    0.0%    0.0%    0.0%    0.0%    0.0%    0.0%    0.0%    0.0%    0.0%    0.0%    0.0%    0.0%    0.0%    0.0%    0.0%    0.0%    0.0%    0.0%    0.0%    0.0%    0.0%    0.0%    0.0%    0.0%    0.0%    0.0%    0.0%    0.0%    0.0%    0.0%    0.0%    0.0%    0.0%    0.0%    0.0%    0.0%    0.0%    0.0%    0.0%    0.0%    0.0%    0.0%    0.0%    0.0%    0.0%    0.0%    0.0%    0.0%    0.0%    0.0%    0.0%    0.0%    0.0%    0.0%    0.0%    0.0%    0.0%    0.0%    0.0%    0.0%    0.0%    0.0%    0.0%    0.0%    0.0%    0.0%    0.0%    0.0%    0.0%    0.0%    0.0%    0.0%    0.0%    0.0%    0.0%    0.0%    0.0%    0.0%    0.0%    0.0%    0.0%    0.0%    0.0%    0.0%    0.0%    0.0%    0.0%    0.0%    0.0%    0.0%    0.0%    0.0%    0.0%    0.0%    0.0%    0.0%    0.0%    0.0%    0.0%    0.0%    0.0%    0.0%    0.0%    0.0%    0.0%    0.0%    0.0%    0.0%    0.0%    0.0%    0.0%    0.0%    0.0%    0.0%    0.0%    0.0%    0.0%    0.0%    0.0%    0.0%    0.0%    0.0%    0.0%    0.0%    0.0%    0.0%    0.0%    0.0%    0.0%    0.0%    0.0%    0.0%    0.0%    0.0%    0.0%    0.0%    0.0%    0.0%    0.0%    0.0%    0.0%    0.0%    0.0%    0.0%    0.0%    0.0%    0.0%    0.0%    0.0%    0.0%    0.0%    0.0%    0.0%    0.0%    0.0%    0.0%    0.0%    0.0%    0.0%    0.0%    0.0%    0.0%    0.0%    0.0%    0.0%    0.0%    0.0%    0.0%    0.0%    0.0%    0.0%    0.0%    0.0%    0.0%    0.0%    0.0%    0.0%    0.0%    0.0%    0.0%    0.0%    0.0%    0.0%    0.0%    0.0%    0.0%    0.0%    0.0%    0.0%    0.0%    0.0%    0.0%    0.0%    0.0%    0.0%    0.0%    0.0%    0.0%    0.0%    0.0%    0.0%    0.0%    0.0%    0.0%    0.0%    0.0%    0.0%    0.0%    0.0%    0.0%    0.0%    0.0%    0.0%    0.0%    0.0%    0.0%    0.0%    0.0%    0.0%    0.0%    0.0%    0.0%    0.0%    0.0%    0.0%    0.0%    0.0%    0.0%        k__Bacteria;p__ZB2;c__; o__       1    0.0%    0.0%    0.0%    0.0%    0.0%    0.0%    0.0%    0.0%    0.0%    0.0%    0.0%    0.0%    0.0%    0.0%    0.0%    0.0%    0.0%    0.0%    0.0%    0.0%    0.0%    0.0%    0.0%    0.0%    0.0%    0.0%    0.0%    0.0%    0.0%    0.0%    0.0%    0.0%    0.0%    0.0%    0.0%    0.0%    0.0%    0.0%    0.0%    0.0%    0.0%    0.0%    0.0%    0.0%    0.0%    0.0%    0.0%    0.0%    0.0%    0.0%    0.0%    0.0%    0.0%    0.0%    0.0%    0.0%    0.0%    0.0%    0.0%    0.0%    0.0%    0.0%    0.0%    0.0%    0.0%    0.0%    0.0%    0.0%    0.0%    0.0%    0.0%    0.0%    0.0%    0.0%    0.0%    0.0%    0.0%    0.0%    0.0%    0.0%    0.0%    0.0%    0.0%    0.0%    0.0%    0.0%    0.0%    0.0%    0.0%    0.0%    0.0%    0.0%    0.0%    0.0%    0.0%    0.0%    0.0%    0.0%    0.0%    0.0%    0.0%    0.0%    0.0%    0.0%    0.0%    0.0%    0.0%    0.0%    0.0%    0.0%    0.0%    0.0%    0.0%    0.0%    0.0%    0.0%    0.0%    0.0%    0.0%    0.0%    0.0%    0.0%    0.0%    0.0%    0.0%    0.0%    0.0%    0.0%    0.0%    0.0%    0.0%    0.0%    0.0%    0.0%    0.0%    0.0%    0.0%    0.0%    0.0%    0.0%    0.0%    0.0%    0.0%    0.0%    0.0%    0.0%    0.0%    0.0%    0.0%    0.0%    0.0%    0.0%    0.0%    0.0%    0.0%    0.0%    0.0%    0.0%    0.0%    0.0%    0.0%    0.0%    0.0%    0.0%    0.0%    0.0%    0.0%    0.0%    0.0%    0.0%    0.0%    0.0%    0.0%    0.0%    0.0%    0.0%    0.0%    0.0%    0.0%    0.0%    0.0%    0.0%    0.0%    0.0%    0.0%    0.0%    0.0%    0.0%    0.0%    0.0%    0.0%    0.0%    0.0%    0.0%    0.0%    0.0%    0.0%    0.0%    0.0%    0.0%    0.0%    0.0%    0.0%    0.0%    0.0%    0.0%    0.0%    0.0%    0.0%    0.0%    0.0%    0.0%    0.0%    0.0%    0.0%    0.0%    0.0%    0.0%    0.0%    0.0%    0.0%    0.0%    0.0%    0.0%    0.0%    0.0%    0.0%    0.0%    0.0%    0.0%    0.0%    0.0%    0.0%    0.0%    0.0%    0.0%    0.0%    0.0%    0.0%    0.0%    0.0%    0.0%    0.0%    0.0%    0.0%    0.0%    0.0%    0.0%    0.0%    0.0%    0.0%    0.0%    0.0%    0.0%    0.0%    0.0%    0.0%    0.0%    0.0%    0.0%    0.0%    0.0%    0.0%    0.0%    0.0%    0.0%    0.0%    0.0%    0.0%    0.0%    0.0%    0.0%    0.0%    0.0%    0.0%    0.0%    0.0%    0.0%    0.0%    0.0%    0.0%    0.0%    0.0%    0.0%    0.0%    0.0%    0.0%    0.0%    0.0%    0.0%    0.0%    0.0%    0.0%    0.0%    0.0%    0.0%    0.0%    0.0%    0.0%    0.0%    0.0%    0.0%    0.0%    0.0%    0.0%    0.0%    0.0%    0.0%    0.0%    0.0%    0.0%    0.0%    0.0%    0.0%    0.0%    0.0%    0.0%    0.0%    0.0%    0.0%    0.0%    0.0%    0.0%    0.0%    0.0%    0.0%    0.0%    0.0%    0.0%    0.0%    0.0%    0.0%    0.0%    0.0%    0.0%    0.0%    0.0%    0.0%    0.0%    0.0%    0.0%    0.0%    0.0%    0.0%    0.0%    0.0%    0.0%    0.0%    0.0%    0.0%    0.0%    0.0%    0.0%    0.0%    0.0%    0.0%    0.0%    0.0%    
     
  Taxonomy Summary. Current Level: Family  
     View Figure (.pdf)    View Legend (.pdf)   
  
[truncated: 7,906,809 more chars]
